# Time-series analysis of rhenium(I) organometallic covalent binding to a model protein for drug development

**DOI:** 10.1107/S2052252524002598

**Published:** 2024-04-19

**Authors:** Francois J.F. Jacobs, John R. Helliwell, Alice Brink

**Affiliations:** a Department of Chemistry, University of the Free State, Nelson Mandela Drive, Bloemfontein, 9301, South Africa; b Department of Chemistry, University of Manchester, Oxford Road, Manchester M13 9PL, United Kingdom; University of Auckland, New Zealand

**Keywords:** fragment-based covalent drug design, covalent drug discovery, time series, macromolecular crystallography, anomalous difference density, induced fit docking, artificial metalloproteins, infra-red spectroscopy

## Abstract

Metal-based complexes with their unique chemical properties, including multiple oxidation states, radio-nuclear capabilities and various coordination geometries yield value as potential pharmaceuticals. Understanding the interactions between metals and biological systems will prove key for site-specific coordination of new metal-based lead compounds. This study merges the concepts of target coordination with fragment-based drug methodologies, supported by varying the anomalous scattering of rhenium along with infrared spectroscopy, and has identified rhenium metal sites bound covalently with two amino acid types within the model protein. A time-based series of lysozyme-rhenium-imidazole (HEWL-Re-Imi) crystals was analysed systematically over a span of 38 weeks. The main rhenium covalent coordination is observed at His15, Asp101 and Asp119. Weak (*i.e.* noncovalent) interactions are observed at other aspartic, asparagine, proline, tyrosine and tryptophan side chains. Detailed bond distance comparisons, including precision estimates, are reported, utilizing the diffraction precision index supplemented with small-molecule data from the Cambridge Structural Database. Key findings include changes in the protein structure induced at the rhenium metal binding site, not observed in similar metal-free structures. The binding sites are typically found along the solvent-channel-accessible protein surface. The three primary covalent metal binding sites are consistent throughout the time series, whereas binding to neighbouring amino acid residues changes through the time series. Co-crystallization was used, consistently yielding crystals four days after setup. After crystal formation, soaking of the compound into the crystal over 38 weeks is continued and explains these structural adjustments. It is the covalent bond stability at the three sites, their proximity to the solvent channel and the movement of residues to accommodate the metal that are important, and may prove useful for future radiopharmaceutical development including target modification.

## Abstract 2

A detailed analysis of rhenium(I) organometallic covalent binding to a model protein is conducted at seven time points over 38 weeks. Changes in the protein structure induced at the Re binding sites over the time series as well as the relationship between the proximity of the solvent channels to the residues containing the highest-occupied Re are described.

## 1. Introduction

Fragment-based drug design (FBDD) utilizes small fragments of molecules, typically organic, which are incubated with a biological target (Erlanson *et al.*, 2016 ▸). These smaller, less-complex molecules tend to have low molar masses (∼150 Da) and steric sizes, and do not have specific high binding affinities to the target protein but display more ‘atom-efficient’ binding interactions than larger molecules. The protein target is then able to interact with a greater variety of fragments. This method has the advantage over high-throughput screening (HTS) which uses complete drug-like molecules by reducing the failure rate of compounds which experience steric and/or electronic clashes with the target protein, even if a portion of the complete compound would have bound favourably to the target (Bon *et al.*, 2022 ▸). In FBDD, the bound fragments are subsequently linked together, known as fragment growing, until a hit is produced. The hit compound is altered by subsequent optimization into a lead compound suitable for pharmaceutical testing, having improved affinity and better pharmacological properties (Kumar *et al.*, 2012 ▸; Doak *et al.*, 2016 ▸; Murray & Blundell, 2010 ▸).

Covalent drug discovery (CDD) is the rationale of creating a drug with a functional group that covalently binds to a drug target (Boike *et al.*, 2022 ▸). These types of compounds are generally excluded from screening collections due to interference with assays and the potential to bind with undesirable protein targets (Singh *et al.*, 2011 ▸). However, recent advances indicate these compounds can bind with protein targets that were previously considered ‘undruggable’ (Boike *et al.*, 2022 ▸). With diligent planning, compounds can be created to have a reversible or irreversible binding nature (Tuley & Fast, 2018 ▸). Covalently binding drugs can be designed for specific residues within a protein target such as cysteine, histidine or other nucleophilic residues which are fairly reactive and can bind covalently with electrophilic functional groups (De Cesco *et al.*, 2017 ▸).

The presence of metal-based drugs in clinical development is less prominent than for organic compounds (Ferraro *et al.*, 2022 ▸). However, organometallics have the advantages of variety by possessing numerous oxidation states, coordination geometries, interesting electronic or optic properties and indeed metal compounds have demonstrated their effectiveness in the clinical environment (Sodhi & Satya, 2019 ▸). Our interest is the development of radiopharmaceuticals utilizing rhenium and its Group 7 congener technetium-99*m*, in particular, trying to understand the parameters which drive coordination within macromolecular settings, such as covalent metal-to-protein binding versus weaker interactions. ^99*m*^Tc has ideal nuclear properties for medical imaging and decays via γ-emission (140 keV) with a 6 h half-life. Pertechnetate, [^99*m*^TcO_4_]^−^ [*i.e.* Technelite (Dodds & Powell, 1968 ▸)], a thyroid-imaging agent, is produced from a ^99^Mo/^99*m*^Tc generator system (Molinski, 1982 ▸). Once eluted, the [^99*m*^TcO_4_]^−^ can be chemically altered to allow for imaging of different organs (Blower, 2015 ▸). Its application in medical imaging is extensive, with examples such as ^99*m*^Tc-medronate (Ostelite) – a bone imaging agent (Schibli, 2007 ▸); ^99*m*^Tc-HMPAO (Ceretec) – a brain perfusion imaging agent (Jürgens *et al.*, 2014 ▸); or ^99*m*^Tc-sestamini (Cardiolite) used for myocardial imaging (Norenberg *et al.*, 2005 ▸). Technetium has no stable isotopes, making routine fundamental chemical investigations difficult given the radioactive waste or radioprotective equipment required. However, the Group 7 therapeutic consociate is rhenium, which exists in both radioactive and ‘cold’ forms and, to some extent, has similar chemical characteristics (Alberto, 2012 ▸), making it a key alternative for fundamental investigations. ^186^Re and ^188^Re have practical therapeutic applications and have been tested in pre-clinical to phase-two clinical trials. Examples are [^186/188^Re]-perrhenate for thyroid, breast and prostate cancer treatment; [^188^Re]-DMSA for metastatic bone cancer and medullary carcinoma; [^186^Re-MAG_3_] for metastatic bone pain (Ogawa *et al.*, 2005 ▸); and [^186/188^Re]-HEDP for metastatic bone cancer, commercially known as ^186^Re-etidronate (Tripunoski *et al.*, 2022 ▸; Shah *et al.*, 2023 ▸). The potential of merging both Re and Tc into a single entity leads to a ‘theranostic pair’ (Kleynhans *et al.*, 2023 ▸), which is a complex containing both therapeutic and imaging capability (Frei *et al.*, 2018 ▸).

Protein crystallographic studies reporting *fac*-[Re(CO)_3_]^+^ in biological settings continue to be rare and tend to report covalent metal-to-protein binding preference almost solely to the histidine imidazole (Binkley *et al.*, 2011 ▸; Zobi & Spingler, 2012 ▸; Santoro *et al.*, 2012 ▸; Takematsu *et al.*, 2013 ▸), with the exception of our work (Brink & Helliwell, 2019 ▸, 2017 ▸). To explore chemical–structure relationships of rhenium in a biological setting, hen egg-white lysozyme (HEWL) was used as a test protein. HEWL is an ideal model protein as it provides a nearly complete range of possible amino acids. The caveat being that Cys and Met are not available for coordination as they are either disulfide bonded (Cys) or buried (Met) in HEWL. It has a relatively small molecular mass of 14 kDa (Ganz, 2006 ▸), is well defined (Charter & Lagarde, 2014 ▸) and generally crystallizes. Though the protein itself is relatively easy to crystallize, the addition of rhenium, an ‘unnatural’ metal element, in our experience, hinders the crystallization process. To circumvent this, the addition of an excess of imidazole was explored to assist in the crystallization. This arose on reviewing the crystallization conditions of all rhenium–protein structures listed in the Worldwide Protein Data Bank (wwPDB) (Burley *et al.*, 2019 ▸; Brink *et al.*, 2022 ▸). We noted that for many of the structures, where rhenium bound covalently to histidine, imidazole was present in the crystallization media. A second motivation for the addition of imidazole is the significant number of imidazole-based compounds that are used in drug design (Gaba & Mohan, 2016 ▸; Siwach & Verma, 2021 ▸; Agarwal *et al.*, 2022 ▸). Imidazole, known to covalently coordinate and stabilize metals, can potentially behave as a crossover-type ligand featuring characteristics of both FBDD and CDD, while also providing kinetic substitution support to aid covalent coordination.

Our study here investigated a time-based series of lysozyme-rhenium-imidazole (HEWL-Re-Imi) crystals monitored systematically over a 38-week period utilizing macromolecular crystallography, supported by varying the anomalous scattering factor of rhenium and by infrared (IR) spectroscopy. By the addition of imidazole, the crystallization of rhenium within the protein is reproducibly achieved within 4 days. We describe the chemical environment around the rhenium within this biological setting, over an extended time period, and the type of bonding (*i.e.* covalent or weak interactions) versus selective binding to specific residues that could be initiated (*i.e.* target-specific drug development). The context of multinuclear cluster formation over time within the protein has previously been observed (Brink & Helliwell, 2019 ▸) and has marked advantages for theranostic applications. Cluster formation is both time and metal-concentration dependent (Frei *et al.*, 2018 ▸). Therefore, specific interest is focused on any movement (Darmanin, 2022 ▸; Stuchebrukhov, 2010 ▸) of rhenium atoms which may occur over a 38-week period.

## 2. Experimental

### 2.1. Crystallization conditions

HEWL (15.0 mg, 1.048 µmol, 1 eq.) was dissolved in water (0.5 ml). *fac*-[Et_4_N]_2_[Re(CO)_3_(Br)_3_] (11.8 mg, 0.015 mmol, 14.6 eq.) was dissolved in water (0.5 ml). To the metal compound solution, imidazole was added (3.0 mg, 0.0441 mmol, 42.0 eq.) and dissolved. A buffer solution of 50:50 1.0 *M* NaCl and 0.05 *M* sodium acetate buffer at 4.5 pH was prepared. The protein and metal solutions were then combined. The protein–metal mixture was then treated to 1 ml of the buffer solution and gently blended. The protein–metal–buffer solution was transferred to a 96-well sitting-drop plate with buffer in the reservoir and the cells sealed. Crystals containing rhenium complexes were consistently observed 4 days after crystallization was set up. These were left soaking in the mother liquor for the duration of the time series. The first X-ray diffraction data collection was carried out one week (7 days) after the crystallization was set up.

**Figure 1:**
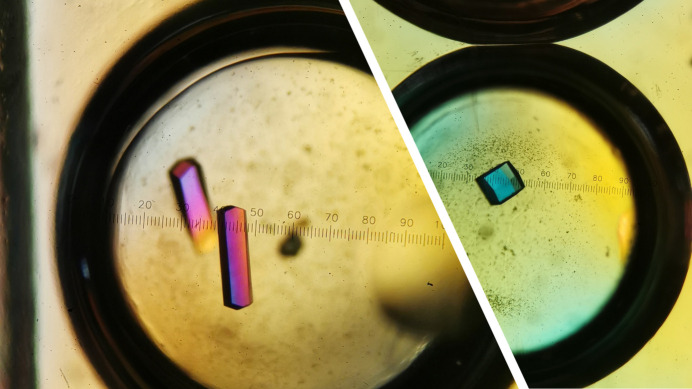
Examples of the HEWL-Re-Imi crystals grown with co-crystallization of HEWL with *fac*-[Et_4_N]_2_[Re(CO)_3_(Br)_3_] and excess imidazole as viewed through a polarizing microscope at 45× magnification in sitting-drop 96-well plates. Both crystal images are from the same crystallization plate, the different external crystal morphologies are typically affected by small changes in the crystallization media and are unlikely to be related to the presence of rhenium (Liu *et al.*, 2010 ▸; Pusey & Nadarajah, 2002 ▸; Judge *et al.*, 1999 ▸).

Subsequent crystallization trays were set up under identical conditions to allow for sequential data collections at both laboratory and tuneable synchrotron X-ray diffraction sources. In total, eight crystal structures were collected at the different time intervals of 1 week, 3 weeks, 9 weeks, 11 weeks, 14 weeks, 18 weeks and 38 weeks from the time of crystallization. All crystallization setups were conducted under laboratory climate control at 17°C and the crystal trays were stored under identical conditions. Selected photographic images of the protein crystals used for the data collection are shown in Fig. 1 ▸. Table 1 ▸ summarizes the diffraction data collection, processing and model-refinement statistics.

### 2.2. Data collection, integration, scaling and refinement

#### 2.2.1. Laboratory X-ray diffraction (Cu *K*α) data

Laboratory datasets were measured at weeks 1, 11 and 14 with a Bruker D8 Venture 4K Kappa Photon III C28 diffractometer utilizing a Cu *K*α X-ray generator with λ = 1.5418 Å. Data collections were conducted at 100 K without cryoprotectant and detector distances of 70 mm. Diffraction data processing was achieved using the Bruker *PROTEUM4* software suite, space group determination with *POINTLESS* (Evans, 2011 ▸) and the scaling of the data with *AIMLESS* (Evans & Murshudov, 2013 ▸). The diffraction resolution cut-off of the data was that suggested by *POINTLESS* and *AIMLESS*. The resolution was confirmed by running the refined crystallographic models through the *PDB_REDO* server (Joosten *et al.*, 2014 ▸) and checked to ensure sufficient completeness in the high-resolution shells. Molecular replacement in *Phaser* (McCoy, 2007 ▸) and the PDB entry 2w1y (Cianci *et al.*, 2008 ▸) were used. Refinement of the molecular models was done using *Phenix* (Liebschner *et al.*, 2019 ▸). Viewing and further optimization was conducted in *Coot* (Emsley *et al.*, 2010 ▸). Alternating between the *Phenix*-refined structure and the *PDB_REDO* model led to a converged final molecular model.

#### 2.2.2. Diamond Light Source (synchrotron) data

Datasets from weeks 3, 18 and 38 were collected on the I04 beamline at Diamond Light Source (DLS). All were collected with an X-ray wavelength of 0.9760 Å to increase the rhenium *f*′′ anomalous signal of the Re *L*_1_ absorption edge to a value of approximately 12.1 electrons versus the 5.9 electrons for Cu *K*α sources, implying a 2.1 increase in an anomalous difference density map peak. Diffraction data collections were performed at 100 K with Paratone-*N* as cryoprotectant. The crystal-to-detector distances for the data collections were set at 168.3 mm. The data reduction, space group determination and scaling were carried out using *fast_dp* (Winter & McAuley, 2011 ▸), *xia23dii* (Winter *et al.*, 2013 ▸) and *xia2dials* (Winter *et al.*, 2018 ▸) on the DLS autoprocessing and downstream-processing service. Refinement of the molecular models against the crystal diffraction datasets was achieved using *Phenix* and *Coot*. The mtz and pdb files for each of the time points are available as supporting information. The raw diffraction images for each time point are archived at DLS and will be publicly released. These total nearly 150 GB and Zenodo allows up to 50 GB. We have nevertheless placed the 38 weeks worth of DLS raw diffraction images totalling 24 GB at Zenodo (Jacobs *et al.*, 2024 ▸) for immediate access alongside the PDB deposition files.

#### 2.2.3. Ligand refinement and atomic coordinate precision calculation estimation

Both the laboratory and the DLS data contained rhenium metal atoms as indicated by the anomalous difference density maps. Metal occupancy values were guided by the anomalous difference density map, the *F*_o_ − *F*_c_ difference map as well as free refinement within *Phenix*. In each case the anomalous difference Fourier peak heights analysis is not as good as the occupancy value estimates in the model refinements. The changes with wavelength of the anomalous difference Fourier peak heights in such a pair of anomalous difference Fourier maps provide firmer evidence than an individual anomalous difference Fourier map single peak height value. Previously, the three model refinement workflows (*SHELXTL*, *CCP*4 and *Phenix*) were compared (Brink & Helliwell, 2017 ▸) and these three workflows, based on the same processed diffraction dataset, do show a variation in the final derived model, especially their estimates of the atomic *B* factors. There is then some sort of systematic error/bias in those procedures. By employing the same workflow to compare each member of the time series, we think it is reasonable to assume that the differences between the model of each time point are indeed real. Furthermore, perhaps more importantly, we have estimated the errors on the coordinates of atoms that have shown any movement in order to estimate their significance.

The addition of metal complexes to the models was achieved using the *eLBOW* (Moriarty *et al.*, 2009 ▸), *REEL* (Moriarty *et al.*, 2017 ▸) and *ReadySet* functionalities available in *Phenix*. The metal complex coordinate files were imported from the Cambridge Structural Database (CSD) (Groom *et al.*, 2016 ▸). The complexes *fac*-[Re(CO)_3_(Imi)_2_ *X*] [where *X* = Asp101, Asp119 (ligand CIF files named VHL)] and *fac*-[Re(CO)_3_(Imi)(H_2_O)*X*] [where *X* = His15, Asp101, Asp119 (ligand CIF files named REI)], were generated from the CSD entry EZASIH (Alberto *et al.*, 1999 ▸). The protein refinement software uses the valence shell electron pair repulsion (VSEPR) theory to define the geometries of ligands in the protein. The VSEPR model does not account for bonds typically found in metal complexes (*i.e.* bonds formed of the metal *d* orbitals). Therefore, in formatting the ligand file the metal carbonyl bonds were defined as a single bond between the metal and the carbon carbonyl and the carbon–oxygen as a triple bond which gave the desired facial geometry expected for *fac*-[Re(CO)_3_ *X*_3_] systems. The molecular model of each time point was refined anisotropically against their respective diffraction dataset. For the rhenium centres these were made isotropic if required for their mobility and/or disorder.

After the molecular model refinements were finalized, each PDB coordinates file (week 1 through to week 38) was submitted to the diffraction precision index server *Online_DPI* (Kumar *et al.*, 2015 ▸) to obtain the standard uncertainties on all atomic positions. Bond distances and their respective errors were calculated for the protein–metal bonds, which had not been restrained. These geometries and their error estimates were compared with the average small-molecule values as found in the CSD. Note that, in general, small-molecule crystallographic structure errors tend to be two orders of magnitude smaller than macromolecular structures since the diffraction resolutions are usually 0.8 Å or better.

### 2.3. Infrared spectroscopic analysis

Infrared spectroscopic measurements were made on individual single crystals obtained from the 96-well plate crystallization trays. A single crystal was sacrificed per spectrum. A Bruker Tensor 27 ATR Standard System infrared spectrophotometer equipped with a 4000–370 cm^−1^ laser range was used. All spectra were collected at room temperature.

## 3. Results

### 3.1. The metal sites

Our interest is the binding of rhenium to a biological model to investigate what changes in the atomic level structural environment occur over time and whether a specific protein residue is consistently selected for the metal coordination. Also, could the addition of free imidazole, which prominently occurs in drug discovery hit compounds, be advantageous? Identification of metal coordination was assisted with the use of tuneable synchrotron radiation to maximize the rhenium anomalous dispersion signal.

**Figure 2:**
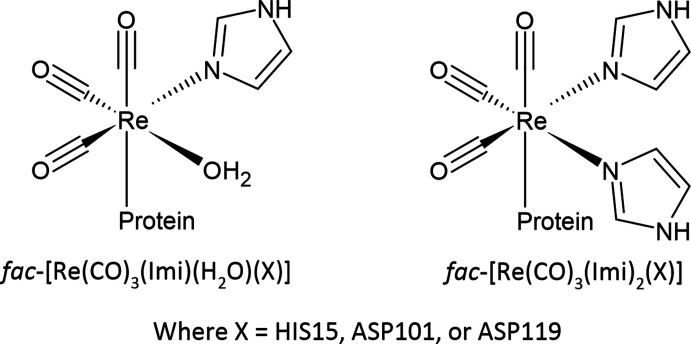
Schematics of the metal complexes found in the lysozyme–rhenium crystal structures.

Throughout the crystal structure series (seven of which are unique crystals and one time point, week 38, involved two datasets being collected at λ = 0.9760 and 1.5400 Å), three covalently bound metal sites are conserved; namely at the side chains of the residues His15, Asp101 and Asp119. Specifically, these sites have sufficient electron density, at the obtained resolutions, to satisfy the placement of the full metal complexes and not just the metal atoms. The complexes are either *fac*-[Re(CO)_3_(Imi)(H_2_O)(*X*)] or *fac*-[Re(CO)_3_(Imi)_2_(*X*)] (where *X* = His15, Asp101 or Asp119). A schematic of the two complexes can be seen in Fig. 2 ▸. Selective rhenium coordination to histidine has been reported (Binkley *et al.*, 2011 ▸; Zobi & Spingler, 2012 ▸; Santoro *et al.*, 2012 ▸; Takematsu *et al.*, 2013 ▸), however rarely for Asp (Brink & Helliwell, 2017 ▸, 2019 ▸). The similarity of coordination in our study is notable when compared with the ruthenium(II) complex of *fac*-[Ru^2+^(CO)_3_(Imi)] complexes in HEWL (Pontillo *et al.*, 2017 ▸) which shows complex bonding to the His15 residue, fragment coordination to Asp119 and a non-binding fragment in the vicinity of Asp101.

### 3.2. Metal complex occupancies and anomalous densities

In the week 1 dataset (laboratory data), three metal complexes are observed that are protein bound, namely *fac*-[Re(CO)_3_(Imi)(H_2_O)(*X*)], where *X* indicates the binding to amino acid residues of His15, Asp101 or Asp119. The His15 bound complex has an occupancy of 56% and anomalous density of 3.7σ. The metal complexes at the Asp101 and Asp119 sites have occupancies of 54 and 44% and anomalous peaks of 5.8 and 5.7σ, respectively. The metal complexes have residual *F*_o_ − *F*_c_ density of 7.4, 8.9 and 4.7σ for the His15, Asp101 and Asp119 metal sites. The residual density is either due to a possible disorder within the metal complex that could not be satisfactorily determined or Fourier series termination ripples around the metal centres. For the higher-resolution crystal structures, anisotropic refinement facilitated the removal of the Fourier ripples.

The week 3 dataset (DLS data) being tuned to optimize the rhenium anomalous dispersion signal therefore has more anomalous density compared with the week 1 laboratory data. The three metal complex sites have been refined to occupancies of 75, 68 and 64% and anomalous difference density values of 29.6, 23.6 and 30.8σ for His15, Asp101 and Asp119, respectively. Like the week 1 data, some residual *F*_o_ − *F*_c_ density of 9.1, 6.5 and 5.5σ at His15, Asp101 and Asp119 is observed in the week 3 data.

The week 9 dataset (DLS data) shows an increase in anomalous density of the three metal complexes with respect to week 3. Namely the His15, Asp101 and Asp119 sites have anomalous densities of 51.8, 50.7 and 58.9σ. The structure has refined metal occupancies of 68, 44 and 48%, respectively. Some residual *F*_o_ − *F*_c_ densities are also present at 4.6, 7.8 and 4.2σ for the His15, Asp101 and Asp119 sites.

Week 11 and week 14 are both laboratory datasets. The refinement of the two shows a direct increase in metal complex occupancies of 75, 55 and 60% to 82, 56 and 65% for the His15, Asp101 and Asp119 metal binding sites of week 11 to week 14. Conversely, the anomalous density for week 14 is 18.1, 15.3 and 22.8σ which is less than those observed in week 11 (20.1, 18.9 and 23.9σ) for the His15, Asp101 and Asp119 sites.

The week 18 (DLS data) structure has refined values of 84, 58 and 58% occupancy for the metal complexes bound to His15, Asp101 and Asp119. The anomalous densities are 53.8, 43.7 and 64.7σ with residual *F*_o_ − *F*_c_ densities of 5.7, 7.1 and 4.1σ.

The week 38 dataset (both DLS data) was collected at both 0.976 and 1.540 Å wavelengths. This was done to compare the anomalous densities and metal occupancies at the different wavelengths at this time point. The metal complex occupancies are the same for the His15 site (84%) and the situation is similar for the Asp119 site at 53% for the 0.976 Å wavelength structure and 57% for the 1.5400 Å wavelength structure. The Asp101 site shows a slight difference in occupancies of the metal complex, the 0.976 Å dataset is at 43% and the 1.54 Å dataset at 56%. The anomalous difference peaks between the two structures are different as expected between the two X-ray wavelengths with 55.5, 39.4 and 54.6σ for the 0.976 Å dataset, and 35.2, 35.2 and 30.9σ for the 1.54 Å dataset for His15, Asp101 and Asp119, respectively. There are residual *F*_o_ − *F*_c_ densities of 5.5, 9.7 and 4.0σ (for the 0.976 Å data) and 12.1, 8.5 and 5.0σ (for the 1.54 Å data) at the His15, Asp101 and Asp119 metal sites.

The final refined structures will show some fluctuations with regards to the metal complex occupancies. Such fluctuations with transition metal bound protein structures have been previously observed as occupancy estimates are affected by different refinement software packages (Brink & Helliwell, 2017 ▸). A detailed discussion of these factors is beyond the scope of the current study as the focus is the chemistry of the metal complexes and proteins as a factor of time. However, we note that previously (Brink & Helliwell, 2017 ▸) we had evaluated model refinements utilizing *SHELX*, *CCP4i* and *Phenix* and found, in general, that the *Phenix* refinement coped best with a high metal electron-density concentration. Therefore, it is useful to note that the overall tendency of the occupancies and anomalous densities of the metal complexes is to increase as time passes (naturally only comparing structures that were collected at the same wavelength). All metal occupancies, residual *F*_o_ − *F*_c_ densities and anomalous difference map peaks can be found in Table 2 ▸. During the refinement of some of the crystal structures, residual *F*_o_ − *F*_c_ densities are found at the Asp101 site. It is suspected that the metal complex is disordered at this site, however attempts to model the three positional disorder led to an unstable refinement.

## 4. Discussion

### 4.1. Overall structure and bond distance analysis

**Figure 3:**
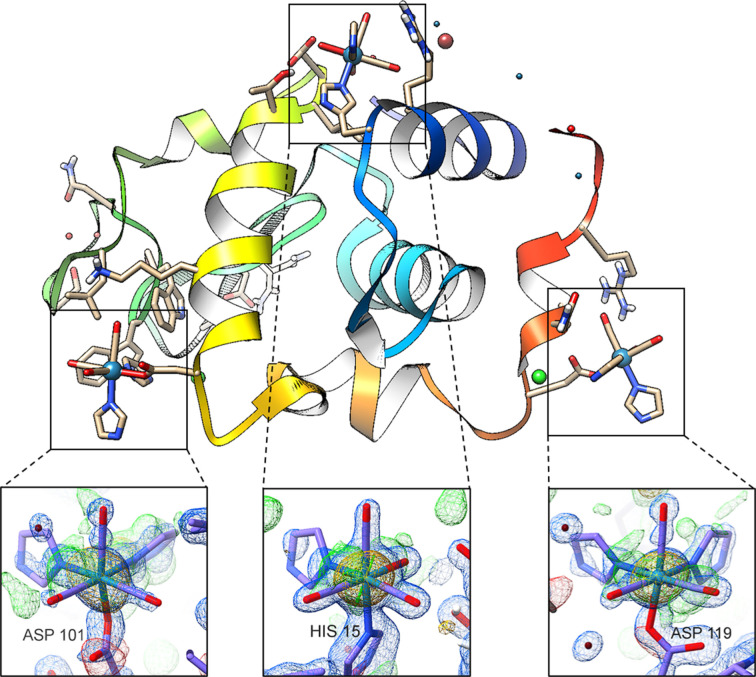
Ribbon diagram of the week 9 (DLS) data depicting the three metal sites consistently conserved between the eight crystal structures. The image was made using the UCFS *Chimera* (Pettersen *et al.*, 2004 ▸). The inset images were drawn using the UCSF *ChimeraX* (Pettersen *et al.*, 2021 ▸) and electron density with the *ISOLDE* plugin (Croll, 2018 ▸).

The coordination of the three main rhenium metal centres relative to the full protein is illustrated in Fig. 3 ▸ for the week 9 structure. Within the image, insets are added to show the metal centres with their respective electron density maps, 2*F*_o_ − *F*_c_ (shown in blue) contoured at 1.5σ, and both the *F*_o_ − *F*_c_ (green for positive density and red for negative) and the anomalous difference density map (orange) contoured at 3.0σ. The electron densities for the ligands (both the carbonyl and the imidazole) bound to the metal atoms are visible. We note that the three major rhenium coordination positions are far from the active site and pocket of lysozyme.

One of our interests is the analysis of the protein–metal bonding distances, in particular whether covalent bonding is present. To formally evaluate covalent bonding, the sum of the covalent radii of the two contributing atoms was evaluated as described by Cordero *et al.* (2008 ▸). These proposed covalent bonding distances were determined by looking at a large subset of structures in the CSD (Groom *et al.*, 2016 ▸) and the proposed covalent radii of the atoms in question were then determined (Cordero *et al.*, 2008 ▸). For aspartic acid binding sites, the metal to protein residue, the proposed covalent distance is 2.17 (7) Å for Re—O. Similarly, for histidine-binding sites the proposed covalent distance is 2.22 (7) Å for Re—N (Cordero *et al.*, 2008 ▸).

**Figure 4:**
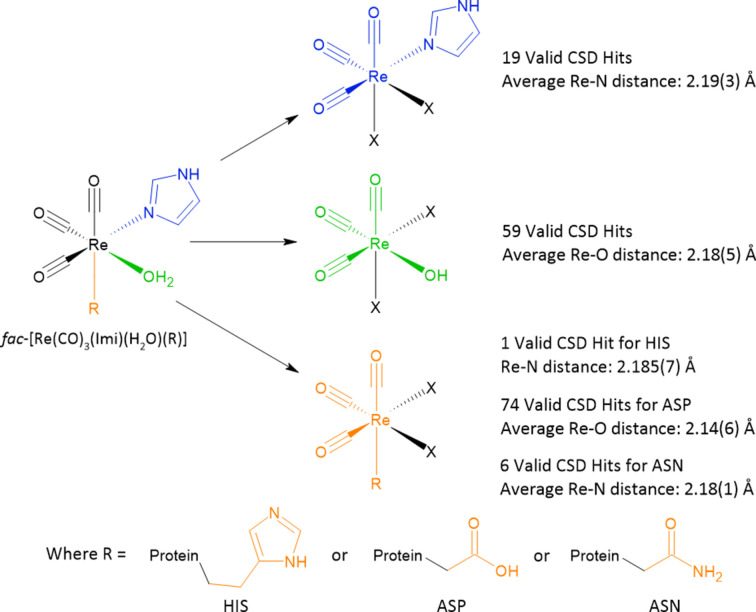
CSD two-dimensional search criteria and a depiction of the general search results. With the scheme are the number of valid hits that have been found with the average metal–N/O bond distance and calculated bond error. *X* in each structure represents the other ligand systems bound to the metal as indicated by the CSD hit result.

We have done a similar study but evaluated specifically the [Re(CO)_3_]^+^ small-molecule bonding distances of rhenium and a donor atom (and not the covalent radii) as well as their respective standard uncertainties from the CSD. Our search criteria were set to obtain a number of hits that are of structural similarity (*i.e.* rhenium tri­carbonyl) to give an experimentally observed bond distance between the atoms of interest. In Fig. 4 ▸, a coloured illustration of the *fac*-[Re(CO)_3_(Imi)(H_2_O)(R)] complex is drawn, indicating the defined search submitted to the CSD to obtain the bond distances that best represent those found in the protein structures of this study. The results of the searches were averaged (including the standard uncertainties) and included in Fig. 4 ▸. It is important to consider both the covalent radii of the atoms and the set specific for [Re(CO)_3_]^+^ to account for any possible effect due to ligand-to-metal electron donation, metal-to-ligand back-bonding, steric effects and other metal–chemical eccentricities such as the *trans* effect that is known to affect metal–ligand bond distances (Coe & Glenwright, 2000 ▸). Every rhenium bond/interaction distance found to be present in this study was determined (distances within 3 Å; Table 3 ▸). The standard uncertainties, both for the protein structures [as calculated by the *Online_DPI* server (Kumar *et al.*, 2015 ▸)] and those calculated from a population of similar small-molecule crystal structures (obtained from their CSD entries) are indicated.

In the week 9 crystal model the rhenium metal centre is 2.28 (4) Å from a His15 side chain imidazole nitro­gen atom which is, within error, equal to the theoretical covalent distance of 2.22 (7) Å and the 2.185 (7) Å of the [Re(CO)_3_]^+^ specific search. The Asp101 and Asp119 binding sites are 2.24 (4) and 2.24 (3) Å in distance between one side of the chain oxygen atoms and the metal centre. These are also, within error, equal to the proposed covalent distance 2.17 (7) Å and the [Re(CO)_3_]^+^ specific search 2.14 (6) Å CSD entry estimate. Therefore, there is covalent bonding for each of the binding sites throughout the time series except for the binding to the Asn46 residue side chain nitro­gen which has a longer bond distance in comparison with a small-molecule estimate. This latter is then a weak interaction.

### 4.2. Changes in protein and metal conformation over time

The amino acid residues situated in the environment of the three main metal sites (*i.e.* His15, Asp101 and Asp119) were investigated throughout the 38 weeks at four time points, namely the metal-free lysozyme crystal structure (PDB entry 2w1y) as the zero-time point, then the week 1 (laboratory data, λ = 1.5418 Å), week 9 (DLS data, λ = 0.9760 Å) and week 38 (DLS data, λ = 0.9760 Å) structures from this study.

**Figure 5:**
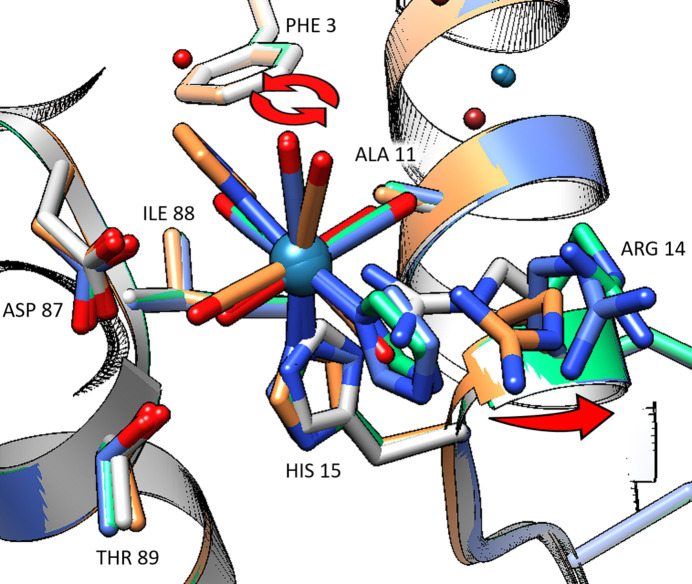
Graphical representation of the conformation changes of the metal complex and protein observed at the His15 site over the 38-week study. The grey model is metal-free (PDB entry 2w1y). The orange model is from the week 1 data. The green model is from the week 9 data and the blue model is from the week 38 data. Red arrows indicate the movement of the complex and residues over time. UCSF *Chimera * version 1.16 was used for figure generation.

The *fac*-[Re(CO)_3_(Imi)(H_2_O)(His15)] complex is surrounded by the Phe3, Ala11, Arg14, Thr89, Asp87 and Ile88 residues. Between the metal-free model (2w1y) and the metal-containing structures, the His15 side chain has a 180° rotation to accommodate the metal binding. Between the week 1 and week 9 structures a conformational change is observed with a 180° rotation of the complex and further outward movement of the Arg14 residue. A graphical representation of these changes can be observed in Fig. 5 ▸. At this site a second imidazole ligand is not observed to be incorporated even at the 38-week time scale, possibly due to the charge of the metal complex. The coordinated water is likely to lose a proton to balance the positive charge on the metal. This now neutral metal complex may be less prone to the coordination of a second neutral imidazole ligand to replace the negatively charged hydroxyl group. Chemical kinetic studies on rhenium–aqua substitution show that the *fac*-[Re(CO)_3_(H_2_O)_2_(OH)] species becomes available at pH values higher than 2.5 (Salignac *et al.*, 2003 ▸), indicating the likelihood that at the crystallization pH of 4.5 a hydroxyl could be present on the metal. Due to the zwitterionic nature of protein structures, this cannot be confirmed unambiguously with the data reported here.

**Figure 6:**
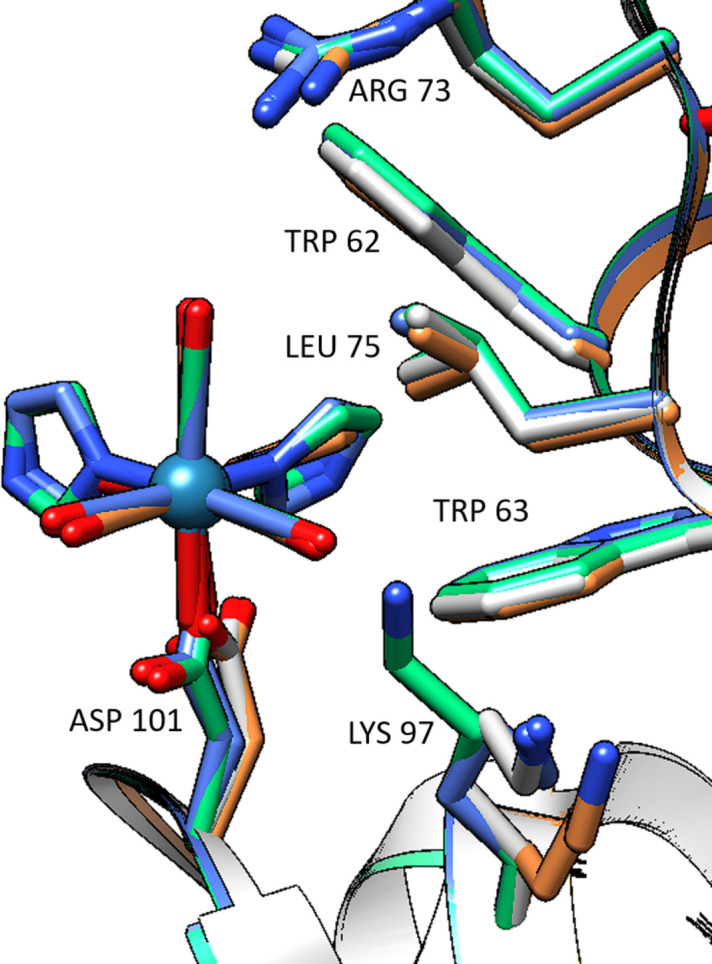
Graphical drawing of the *fac*-[Re(CO)_3_(Imi)_2_(Asp101)] complex and its surrounding residue site over the 38-week study. The grey model is metal-free (PDB entry 2w1y). The orange model is from the week 1 data, the green model is from the week 9 data and the blue model is from the week 38 data. UCSF *Chimera* version 1.16 was used for figure generation.

The *fac*-[Re(CO)_3_(Imi)_2_(Asp101)] complex experiences small conformation changes in both the metal complex and the parent (metal-free) protein. Of note is the flipping of the Asp101 side chain which does not seem to hinder the binding of the metal complex. The complex has interactions with the parent lysozyme residues, namely Trp62, Trp63 and Leu75. The Lys97 side chain shows movement but does not seem to be directly affected by the presence of the metal complex (Fig. 6 ▸).

**Figure 7:**
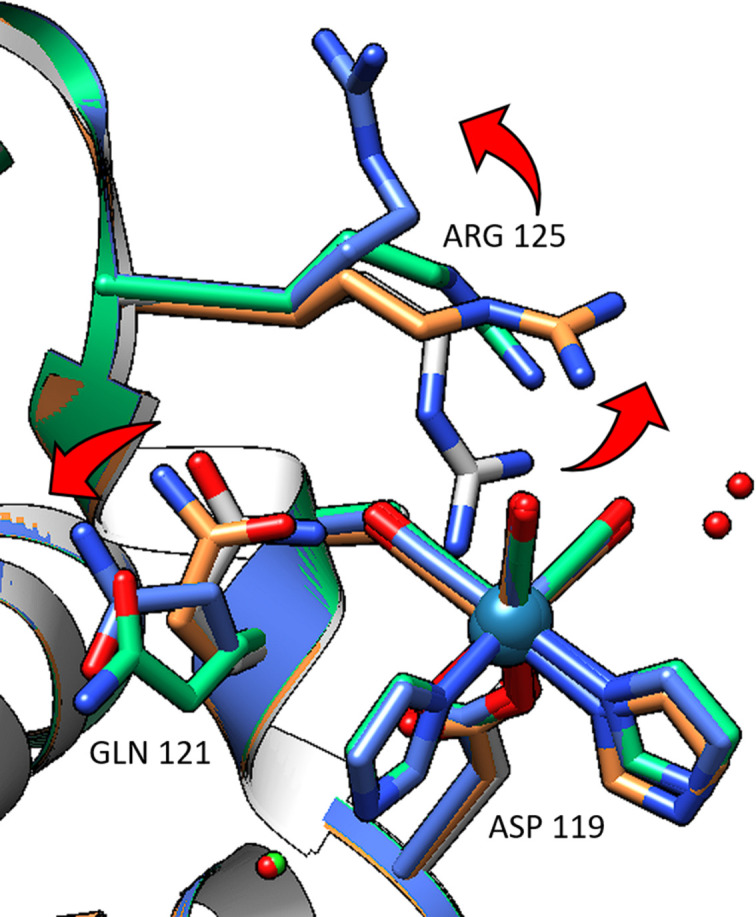
*fac*-[Re(CO)_3_(Imi)_2_(Asp119)] complex and its surrounding side chains over the 38-week study. The grey model is metal-free (PDB entry 2w1y). The orange model is from the week 1 data, the green model is from the week 9 data and the blue model is from the week 38 data. Movement of the side chains is indicated by red arrows. UCSF *Chimera* version 1.16 was used for figure generation.

The *fac*-[Re(CO)_3_(Imi)_2_(Asp119)] complex has fewer interacting residues, but the two residues of note (Arg125 and Gln121) move significantly throughout the time series. The movement of the side chains appears to be a sweep away from the metal location as illustrated in Fig. 7 ▸. The movement is quite marked when comparing non-metal containing structures of HEWL (*i.e.* PDB entries 2w1y, 5uvj or 7ac2) to these metal-based structures (see Fig. S1 of the supporting information). Greater stability to the coordinating Asp119 side chain appears as its position is more conserved than that of Asp101. In both the Asp101 and Asp119 metal complexes, the coordination of a second imidazole ligand to the Re is observed early in the time series, *i.e.* from the week 3 dataset onwards, likely due to charge-balancing. The two neutral imidazole ligands do not balance the positive charge of the *fac*-[Re(CO)_3_]^+^ core, but at the 4.5 pH of the crystallization buffer media, the aspartic acid side chain groups are likely to be deprotonated [Asp-β-COOH has a p*K*_a_ of 3.90 (Voet & Voet, 2004 ▸)] to balance the oxidation state, thereby facilitating the incorporation of the second imidazole ligand in both aspartic acid metal complex sites. It is rather intriguing that of the seven aspartic acid residues of HEWL, only four have anomalous density as evidence to support rhenium metal placement, given that all the aspartic acid residues should be deprotonated at the crystallization pH, and thereby ideal for metal binding. It is likely that there are other factors that play an important role, such as access via solvent channels, space for binding and the positions of free ions near the residues in question.

**Figure 8:**
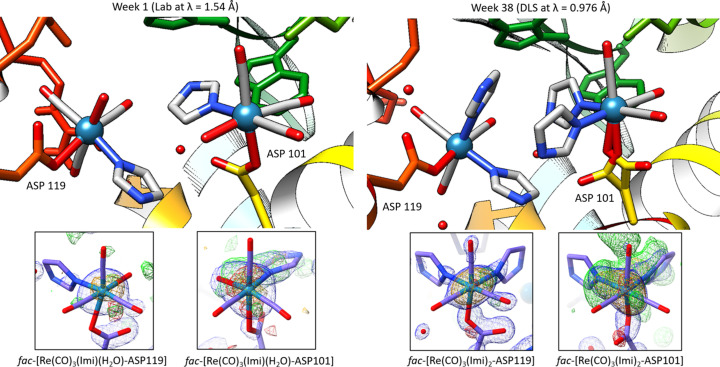
Graphical representation of the formation of the π–π cage structure between the Asp101 and Asp119 metal complex sites. Left: the week 1 data (laboratory at λ = 1.54 Å). Right: the week 38 data (DLS at λ = 0.976 Å). For both images, insets of the metal complex and their electron densities are indicated: 2*F*_o_ – *F*_c_ (blue) contoured at 1.5σ. *F*_o_ − *F*_c_ (green for positive density and red for negative). Anomalous difference density map (orange) contoured at 3.00σ. UCSF *Chimera* version 1.16 was used for figure generation, the insets were drawn using UCSF *ChimeraX* (Pettersen *et al.*, 2021 ▸) version 1.3 and the electron density with the *ISOLDE* plugin (Croll, 2018 ▸).

**Figure 9:**
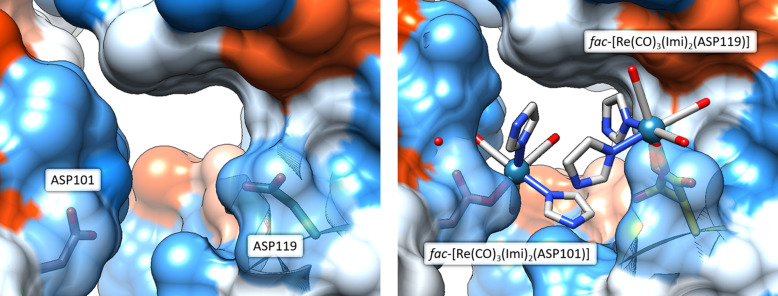
Diagram of the formation of the Asp101/Asp119 crystal contact. Left: the metal-free model (PDB entry 2w1y) at the Asp101/Asp119 pocket. Right: the week 38 (DLS data at λ = 0.976 Å) model of the same pocket also containing the metal complexes *fac*-[Re(CO)_3_(Imi)_2_(Asp101)] and *fac*-[Re(CO)_3_(Imi)_2_(Asp119)]. In both images the surfaces of the proteins have been drawn with the surface of the Asp101 and Asp119 residues at 50% transparency. UCSF *Chimera* version 1.16 was used for figure generation.

A crystal contact is defined as intermolecular contacts that occur solely because of the crystallization of the protein (Dasgupta *et al.*, 1997 ▸). Crystal contacts can reveal insights into protein–protein contact domains and can be biologically relevant (Janin & Rodier, 1995 ▸). Additionally, these types of interactions can be engineered into proteins by selective mutation of residues on the surface of a difficult-to-crystallize protein which resulted in the successful crystallization of proteins that could not possibly be obtained by other means (Yamada *et al.*, 2007 ▸; Lawson *et al.*, 1991 ▸). In the metal-free model of HEWL (PDB entry 2w1y), crystal contacts can be observed between Arg125 to Leu129 and Thr47 to Gly49, between symmetry-related molecules. The residues Asp101 and Asp119 in the 2w1y model do not indicate any inter­actions. However, in the metal-bound model, metal-complex-to-metal-complex interactions are observed at the Asp101 and Asp119 side chain residues. Due to the proximity of the two residues with respect to each other in the crystal structure, a pseudo di-nuclear rhenium species with a four-imidazole tetramer-cage is formed during the time series (Fig. 8 ▸). Note that when the protein surface is generated (Fig. 9 ▸), this pseudo *fac*-[Re(CO)_3_(Imi)_2_(Asp)]_2_ structure fits favourably within the cavity between the two lysozyme structures. To determine whether this structure is just a space filling artefact or possibly induced by π–π interactions between the *fac*-[Re(CO)_3_(Imi)_*n*_] complex, it is useful to look at the interaction distances between the centroids of the imidazole ligands.

**Figure 10:**
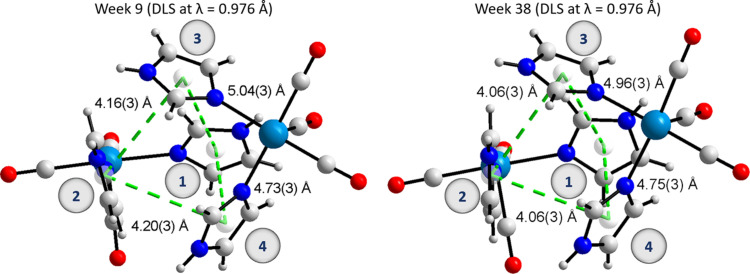
Diagram of the pseudo di-nuclear rhenium species with a four-imidazole tetramer-cage: week 9 (left) and week 38 (right). In both images the bonds between the centroids are indicated as green dashed lines with the corresponding bond lengths with standard uncertainty. The aromatic imidazole rings are labelled identically for both the week 9 and the week 38 models. The protein side chains have been omitted for clarity. These figures are generated using *DIAMOND* (version 4.0; Brandenburg & Putz, 2005 ▸).

In small-molecule crystallography, there are two major categories wherein π–π interactions can be classified (between aromatic structures), namely face-to-face and edge-to-face interactions (Martinez & Iverson, 2012 ▸). For a face-to-face system, the distance between the centroids is less than 4.0 Å and the angle between the planes less than 30°. For an edge-to-face interaction, the distance is less than 5.5 Å and the angle between the planes is between 60 and 120°. Similarly, in macromolecular crystallography these two types of aromatic interactions are also observed with the face-to-face type interactions being defined as having a centroid-to-centroid distance of 3.3 to 3.8 Å. The edge-to-face interaction ranges from 4.96 to 5.025 Å (Zhao *et al.*, 2015 ▸) and the angle between the planes tends to be between 60 and 120° (Chourasia *et al.*, 2011 ▸). The pseudo *fac*-[Re(CO)_3_(Imi)_2_(Asp)]_2_ structure has four edge-to-face aromatic interactions that assist in bridging the protein molecules in the crystal structure. The centroid-to-centroid distances of week 9 and week 38 can be seen in Fig. 10 ▸. The distances for week 9 vary between 4.16 (3) and 5.04 (3) Å and the week 38 distances vary between 4.06 (3) and 4.96 (3) Å. These distances are within range of edge-to-face interactions as described by Zhao *et al.* (2015 ▸) for macromolecules and they fit the small-molecule distance criteria being less than 5.5 Å. The dihedral angle between the planes for week 9 varies between 45.6 and 78.4°, and for week 38 between 44.4 and 86.8°. The lower angles are less than expected for an edge-to-face (between 60 and 120°) type interaction but greater than 30° (which is expected to be less if a face-to-face interaction is proposed). It is therefore likely that these are a set of edge-to-face aromatic interactions.

When comparing the centroid-to-centroid bond distances between the two structures, a contraction over time is observed. The distance between centroid 2 and 3 is 4.16 (3) Å for week 9 and 4.06 (3) Å for week 38, and between centroid 2 and 4 the distance is 4.20 (3) Å for week 9 and 4.06 (3) Å for week 38. The other centroid-to-centroid distances are similar within error. This indicates a strengthening of the contact.

### 4.3. Solvents, solvent channels and their effect on drug development

**Figure 11:**
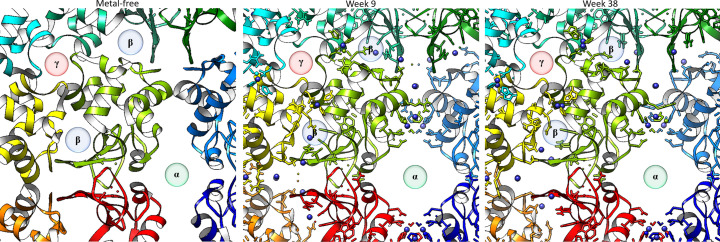
Left: orthographic projection of the solvent channels of the metal-free model (PDB entry 2w1y). Middle: orthographic projection of the solvent channels of the crystal structure obtained after 9 weeks (DLS data). Right: the structure at 38 weeks. The three solvent channels of HEWL are denoted α, β and γ. The rhenium atoms are coloured dark blue. The images were generated perpendicular to the *c* axis with the UCFS *Chimera* 1.16 software.

Solvent channels control access of a compound soaked into a crystal over a period of time from its mother liquor and can be useful to understand interactions between an inhibitor or a substrate with a target protein. This forms an important approach in drug discovery (Sprenger *et al.*, 2021 ▸). The natural (*i.e.* metal-free) tetragonal crystal form of HEWL has three solvent channels, as viewed perpendicular to the *c* axis (Takayama & Nakasako, 2011 ▸) (Fig. 11 ▸). For weeks 9 and 38 the rhenium atoms are included (indicated in dark blue in Fig. 11 ▸) and show that the β solvent channel is populated with rhenium atoms as well as showing the subtle movement of the surrounding residues over time (refer also to Figs. 5 ▸–8 ▸ ▸ ▸). The metal sites are closely located to a solvent channel. The *fac*-[Re(CO)_3_(Imi)(H_2_O)(His15)] complex is near the α channel and both *fac*-[Re(CO)_3_(Imi)_2_(Asp101)] and *fac*-[Re(CO)_3_(Imi)_2_(Asp119)] complexes are between the β channel and the γ channel.

**Figure 12:**
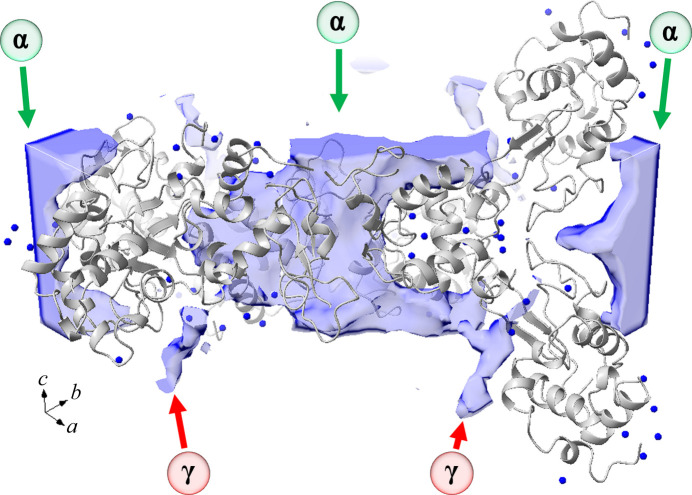
Three-dimensional depiction of the solvent channels in the week 9 (DLS) data. The rhenium metal atoms are coloured blue. The α and λ channels are indicated by green and red arrows, respectively. The solvent channel architectures were calculated by the *MAP_CHANNELS* (Juers & Ruffin, 2014 ▸) program via its *Coot* plugin. This was then loaded into *ChimeraX* version 1.3 with the *ISOLDE* plugin.

Although the two-dimensional top-down view of the solvent channels is informative, a better view can be gained from a general three-dimensional perspective. This ensures that the metal positions are indeed near the solvent channels and not just an artefact of the projection along the *c* axis (Fig. 12 ▸). The rhenium atoms (again dark blue) are indeed in the proximity of the α and γ channels.

### 4.4. Discussion of the infrared spectroscopy results

**Figure 13:**
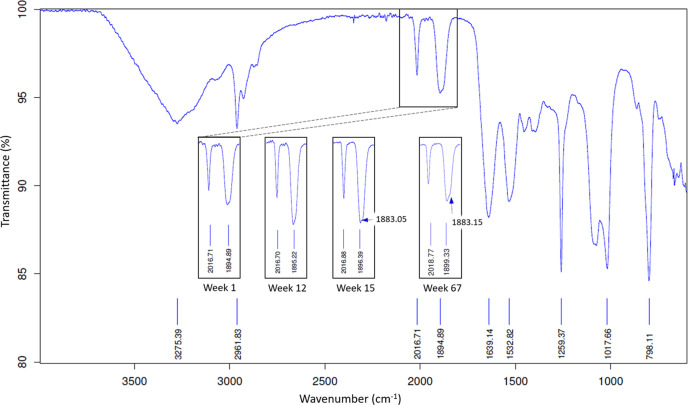
IR spectrum of HEWL co-crystallized with ReAA and imidazole. The carbonyl stretching frequency range has been highlighted and added to the inset 1 week after crystallization was set up. Two additional insets of the same region for a sacrificed crystal at weeks 12, 15 and 67 after crystallization are indicated.

Single crystals of HEWL co-crystallized with the rhenium and imidazole were analysed via IR spectroscopy. The *fac*-[Re(CO)_3_]^+^ core has the advantage of the carbonyl stretching frequencies as spectroscopic probes to determine metal coordination. The *fac*-[Re(CO)_3_ *X*_3_]^+^ core is considered stable due to the low-spin *d*_6_ state of the rhenium(I) metal centre whereas the three-coordinating aqua/halido (*X*) ligands tend to be labile (Alberto *et al.*, 1999 ▸). The carbonyls are sensitive to changes in electron density when the metal coordination takes place as seen in the shift in carbonyl stretching frequencies. This method is routinely used to characterize metal complexes in coordination chemistry. Note IR values are also influenced by packing effects [*i.e.* KBr (compressed pellet in random solid state) versus ATR (ordered crystalline solid state or liquid state)]. The IR spectra of *fac*-[Et_4_N]_2_[Re(CO)_3_(Br)_3_] (ReAA) are typically in the range ν_(CO)_ = 2000, 1870 cm^−1^ (KBr pellet) (Alberto *et al.*, 1996 ▸). IR data of ReAA (loose powder) fall within the range ν_(CO)_ = 1996, 1847 cm^−1^; and ReAA in a buffer solution ν_(CO)_ = 2016, 1870 cm^−1^ (ATR IR) (Brink & Helliwell, 2019 ▸). The IR spectra of the crystals from this study were obtained (Fig. 13 ▸) with ν_(CO)_ = 2017, 1896 cm^−1^ indicating the coordination of the metal complex to the protein from week 1 to week 12. A slight shoulder in the 1896 cm^−1^ peak is observed to start forming during the week 15 IR spectra with a value of 1883 cm^−1^. Extended crystal soaking in the metal solution yielded an increase in the wavenumber ν_(CO)_ = 2019, 1899, 1883 (shoulder) cm^−1^ at 67 weeks indicating a continuation of movement of the rhenium complexes within the protein structure.

### 4.5. Additional rhenium atom positions

Additional, lower-occupancy rhenium sites were identified in the anomalous difference density maps throughout the whole time series. Asp18 has lower occupancies that that of Asp101 and Asp119 sites over the 38-week study despite also being an aspartic acid residue. However, it is located a greater distance from the solvent channels and is in a sterically more constricted local environment, whereas Asp101 and Asp119 are located in close proximity to the γ solvent channel. The rhenium–oxygen covalent bond distance is, within error, the same as its small-molecule crystallography counterpart for the Asp18 metal site (Table 3 ▸). Only a metal atom is modelled, except for week 9, as the observed *F*_o_ − *F*_c_ electron density was insufficient to support the placement of the bound ligands in the metal complex. Additional rhenium sites can be found in the week 3, week 9 and week 18 datasets (DLS data with high resolution) where anomalous difference density is seen and supports the placement of a low-occupancy rhenium metal atom at the Asn46 and Asp52 residues.

Additional rhenium anomalous density is observed at Arg14, Tyr23, Pro70, Trp123 and Asp129. Weak interactions are seen at these sites (*i.e.* covalent binding is uncertain), considering standard uncertainties on distances, and the positions of the rhenium metals were consistent with those seen in previous rhenium-HEWL studies (Brink & Helliwell, 2017 ▸, 2019 ▸). It is curious that 6 of the 12 residues where the metals are located are nearby aspartic acid residues. Similarly, of the three high-occupancy metal sites, two are aspartic acid residues. The occupancy value of the Re bound to the His15 residue however remains the highest and is on this basis preferred to those of the aspartic acid residues. A review of reported rhenium-bound protein structures in the PDB indicated that rhenium, irrespective of oxidation state or chemical formula, has a preference for histidine coordination across a range of different proteins (Brink *et al.*, 2022 ▸). However, note that chemical kinetic studies under non-biological conditions indicate that Re-substitution rates and water-exchange rates are found to be slightly dependant on the nature of the incoming ligand, with the softer S-bonded ligands coordinating faster than N-bonded followed by O-bonded ligands to the rhenium metal centre (Salignac *et al.*, 2003 ▸; Grundler *et al.*, 2006 ▸). These additional binding sites highlight the significance of using crystallographic data obtained at the Re absorption edge for maximal anomalous signal for the metal. The extended soaking times also enable the observation of the weaker binding locations to residues that would otherwise have been overlooked.

A primary concern in drug development is the stability of the compound during the delivery phase. It is imperative that the active form of the compound of choice must be able to reach the target before degradation occurs within the bio­logical system. Degradation can occur via light, temperature, acidity/basicity, oxidation/reduction and other biomolecules (Thorsteinn Loftsson, 2014 ▸; Briscoe & Hage, 2009 ▸). Here we show that from a more chemical perspective the metal complexes at the three main sites (His15, Asp101 and Asp119) have demonstrated their stability as the final products of formation within a biological system over a 38-week period. Additionally, past kinetic evaluations of *fac*-[Re(CO)_3_]^+^ complexes bound to nitro­gen- and oxygen-based ligand systems have a high rate of formation kinetic constants indicating their kinetic stability (Jacobs *et al.*, 2021 ▸).

## 5. Conclusions

In this study, we have reported a time-resolved series of protein crystal structures that were co-crystallized with *fac*-[Et_4_N]_2_[Re(CO)_3_(Br)_3_] (ReAA) and excess imidazole. The crystal structures were collected with both laboratory Cu *K*α X-ray and synchrotron (DLS, I04 Beamline) radiation sources tuned to the rhenium anomalous absorption edge for increased anomalous dispersion signal to highlight low-occupancy metal positions. Three major metal-to-protein binding sites were observed at His15, Asp101 and Asp119 and conformational changes of both the metal complexes and the residues surrounding the metal complexes indicated that the protein accommodates the metal complexes in an induced-fit-type covalent docking mode which varies gradually over the 38-week period. The rhenium metal, particularly with the addition of excess imidazole, tends to bind with donor atoms of residues that are typically found in the active sites of metalloproteins (*i.e.* aspartic acid and histidine) and which are found close to the solvent channels. Importantly, the three main metal-binding sites (one histidine and two aspartic acids) are consistent throughout the 38-week time series while neighbouring amino acid residues adjust to accommodate the metal complex. The formation of multinuclear clusters is not observed over the time period of 9.5 months; however, a pseudo dinuclear *fac*-[Re(CO)_3_(Imi)_2_(Asp)]_2_ species is formed, bridging Asp101 and Asp119. It is the covalent bond stability at the three sites, their proximity to the solvent channel and the movement of residues to accommodate the metal that are important and may prove useful for future radiopharmaceutical development including target modification.

## Supplementary Material

Week 1 HEWL-Re-Im: pdb, mtz, mmcif, mtz with anomalous and PDB validation reports. DOI: 10.1107/S2052252524002598/be5295sup1.zip

Week 3 HEWL-Re-Im: pdb, mtz, mmcif, mtz with anomalous and PDB validation reports. DOI: 10.1107/S2052252524002598/be5295sup2.zip

Week 9 HEWL-Re-Im: pdb, mtz, mmcif, mtz with anomalous and PDB validation reports. DOI: 10.1107/S2052252524002598/be5295sup3.zip

Week 11 HEWL-Re-Im: pdb, mtz, mmcif, mtz with anomalous and PDB validation reports. DOI: 10.1107/S2052252524002598/be5295sup4.zip

Week 14 HEWL-Re-Im: pdb, mtz, mmcif, mtz with anomalous and PDB validation reports. DOI: 10.1107/S2052252524002598/be5295sup5.zip

Week 18 HEWL-Re-Im: pdb, mtz, mmcif, mtz with anomalous and PDB validation reports. DOI: 10.1107/S2052252524002598/be5295sup6.zip

Week 38 HEWL-Re-Im_1.54 A: pdb, mtz, mmcif, mtz with anomalous and PDB validation reports. DOI: 10.1107/S2052252524002598/be5295sup7.zip

Ligand CIFs. DOI: 10.1107/S2052252524002598/be5295sup8.zip

Supporting figures. DOI: 10.1107/S2052252524002598/be5295sup9.pdf

PDB reference: HEWL-[Re(CO)_3_-Im], 8qcu

_3_: https://doi.org/10.5281/zenodo.10972988

## Figures and Tables

**Table 1:** X-ray crystallographic data and model-refinement statistics for the time-resolved HEWL-Re-Imi crystal structures Statistics for the highest-resolution shell are shown in parentheses. SI indicates supporting information.

	Week 1	Week 3	Week 9	Week 11	Week 14	Week 18	Week 38	Week 38
Data reduction								
Space group	*P*4_3_2_1_2	*P*4_3_2_1_2	*P*4_3_2_1_2	*P*4_3_2_1_2	*P*4_3_2_1_2	*P*4_3_2_1_2	*P*4_3_2_1_2	*P*4_3_2_1_2
Unit-cell parameters								
*a* (Å)	78.59 (1)	80.99 (1)	81.08 (1)	80.75 (1)	80.92 (1)	81.23 (1)	81.12 (1)	81.1089 (2)
*b* (Å)	78.59 (1)	80.99 (1)	81.08 (1)	80.75 (1)	80.92 (1)	81.23 (1)	81.12 (1)	81.1089 (2)
*c* (Å)	36.98 (3)	37.14 (3)	37.09 (3)	37.02 (3)	37.03 (3)	37.22 (3)	37.19 (3)	37.1986 (2)
α = β = γ (°)	90	90	90	90	90	90	90	90
Molecules per asymmetric unit	1	1	1	1	1	1	1	1
Detector	Photon III C28	Eiger2 XE 16M	Eiger2 XE 16M	Photon III C28	Photon III C28	Eiger2 XE 16M	Eiger2 XE 16M	Eiger2 XE 16M
Crystal-to-detector distance (mm)	70.0	168.3	168.3	70.0	70.0	168.3	168.3	168.3
X-ray source	Laboratory Cu Kα	DLS	DLS	Laboratory Cu *K*α	Laboratory Cu *K*α	DLS	DLS	DLS
X-ray wavelength (Å)	1.54	0.976	0.976	1.54	1.54	0.976	0.976	1.54
Observed reflections	275950 (23373)	28818 (2634)	897581 (3426)	71132 (6058)	49264 (4607)	76847 (7362)	87971 (7685)	24795 (1841)
Unique reflections	12159 (1185)	14456 (1347)	66836 (5537)	35755 (3215)	24700 (2340)	38460 (3712)	44171 (3979)	12515 (1007)
Resolution (Å)	22.21–1.75 (1.813–1.75)	36.22–1.68 (1.74–1.68)	40.54–1.23 (1.274–1.23)	22.61–1.23 (1.274–1.23)	22.44–1.41 (1.45–1.41)	27.44–1.21 (1.253–1.21)	36.28–1.15 (1.191–1.15)	57.37–1.76 (1.823–1.76)
Completeness (%)	99.61 (100.00)	98.62 (92.23)	97.06 (77.45)	90.94 (86.98)	98.97 (95.88)	99.78 (98.33)	98.87 (89.54)	97.64 (80.69)
*R*_merge_	0.1982 (1.777)	0.081 (2.220)	0.039 (1.911)	0.251 (3.056)	0.161 (1.330)	0.075 (0.424)	0.090 (2.098)	0.082 (0.152)
*R*_p.i.m._	0.04225 (0.4066)	0.033 (1.555)	0.011 (0.982)	0.045 (1.592)	0.029 (0.471)	0.015 (0.162)	0.018 (0.854)	0.017 (0.151)
[*I*/σ(*I*)]	12.97 (1.19)	16.30 (0.74)	23.9 (0.1)	11.60 (0.16)	15.19 (2.57)	48.74 (4.34)	21.34 (0.78)	32.32 (6.07)
Multiplicity	22.7 (19.7)	19.5 (13.0)	22.5 (8.2)	32.4 (3.6)	25.8 (7.8)	22.4 (7.9)	21.0 (6.4)	18.9 (1.1)
Mn(I) half-set correlation CC_1/2_	0.998 (0.723)	0.995 (0.424)	1.000 (0.330)	0.99 (0.445)	0.952 (0.547)	0.993 (0.927)	0.994 (0.448)	0.998 (0.962)
Cruickshank DPI (Å)	0.084	0.072	0.026	0.042	0.040	0.023	0.022	0.077
Average *B* factor (Å^2^)	25	34	21	19	15	20	23	22

Refinement								
*R* factor	0.194 (0.316)	0.196 (0.3303)	0.154 (0.2908)	0.226 (0.6248)	0.160 (0.2961)	0.147 (0.1932)	0.158 (0.3570)	0.180 (0.2809)
*R*_free_	0.250 (0.3681)	0.235 (0.3151)	0.182 (0.3239)	0.257 (0.5988)	0.197 (0.3131)	0.173 (0.2292)	0.177 (0.3969)	0.225 (0.3409)
R.m.s.d. angles (°)	1.46	1.39	1.22	1.36	1.26	1.26	1.15	1.20
Ramachandran plot								
Most favoured (%)	97.64	98.43	97.64	98.43	98.43	98.43	98.43	97.64
Additional allowed (%)	2.36	1.57	2.36	1.57	1.57	1.57	1.57	2.36
Disallowed (%)	0.00	0.00	0.00	0.00	0.00	0.00	0.00	0.00
PDB entry/data access	SI	SI	SI	SI	SI	SI	8qcu	SI

**Table 2:** Rhenium occupancy values, anomalous difference map peaks and residual *F*_o_ − *F*_c_ densities as found in the crystal structures organized by weeks since the crystallization was set up and with the respective wavelengths given in parentheses MSO, metal site occupancy; AD, anomalous density (σ); RD, residual *F*_o_ − *F*_c_ density (σ).

	Week 1 (1.54 Å)			Week 3 (0.976 Å)			Week 9 (0.976 Å)			Week 11 (1.54 Å)			Week 14 (1.54 Å)			Week 18 (0.976 Å)			Week 38 (0.976 Å)			Week 38 (1.54 Å)		
Residue	MSO	AD	RD	MSO	AD	RD	MSO	RD	RD	MSO	AD	RD	MSO	AD	RD	MSO	RD	RD	MSO	AD	RD	MSO	AD	RD
Covalent sites																								
His15	0.56	3.7	7.4	0.75	29.6	9.1	0.68	51.8	4.6	0.75	20.1	4.1	0.65	18.1	4.2	0.84	53.8	5.7	0.84	55.5	5.5	0.84	35.2	12.1
Asp18	–	–	5.9	0.20	9.1	4.4	0.17	10.6	5.3	0.13	5.1	–	0.19	4.4	–	0.21	11.9	5.2	0.17	8.6	–	0.18	5.8	–
Asn46	–	–	–	0.21	6.8	–	0.08	3.9	–	–	–	–	–	–	–	0.2	4.3	–	–	–	–	–	–	–
Asp52	–	–	–	0.21	6.8	–	0.08	3.9	–	–	–	–	–	–	–	0.2	4.3	–	–	–	–	–	–	–
Asp101	0.54	5.8	8.9	0.68	23.6	6.5	0.44	50.7	7.8	0.55	18.9	7.3	0.56	15.3	9.0	0.58	43.7	7.1	0.43	39.4	9.7	0.56	35.2	8.5
Asp119	0.44	5.7	4.7	0.64	30.8	5.5	0.48	58.9	4.2	0.6	23.9	5.3	0.65	22.8	3.5	0.58	64.7	4.1	0.53	54.6	4.0	0.57	30.9	5.0
Other sites																								
Arg14	–	–	–	0.19	8.8	3.8	0.19	4.6	4.4	0.09	9.5	5.3	0.15	6.6	–	0.10	19.2	14.5	0.23	21.5	6.7	0.17	12.5	4.1
Tyr23	–	–	–	–	–	–	–	–	–	0.09	5.7	–	0.26	5.6	–	–	–	–	0.23	3.3	3.6	0.31	6.5	–
Pro70	–	–	–	0.20	7.6	–	0.18	4.3	–	0.08	4.2	–	0.12	3.8	–	0.13	12.3	3.1	0.13	7.8	–	0.14	5.5	–
Asp101	–	–	–	0.20	4.8	–	–	–	–	–	–	–	–	–	–	0.11	5.6	–	–	–	–	–	–	–
Trp123	–	–	–	0.27	3.3	–	–	–	–	–	–	–	–	–	–	–	–	–	0.26	4.2	–	0.28	3.5	–
Asp129	–	–	–	0.29	10.1	3.8	0.21	8.7	3.4	0.15	5.4	–	–	–	–	0.26	11.6	10.1	0.21	9.1	–	0.34	6.1	3.7

**Table 3:** Rhenium–protein bond distances and estimated errors compared with the small-molecule counterparts with averages and errors estimated from equivalent multiple entries in the CSD

			Distances and standard uncertainty value estimates in brackets†								
Residue	Atom 1	Atom 2	Week 1	Week 3	Week 9	Week 11	Week 14	Week 18	Week 38	Week 38	Small-molecule average distance‡
His15	Re1	NE2	2.3 (2)	2.3 (1)	2.28 (4)	2.30 (7)	2.32 (7)	2.14 (6)	2.22 (4)	2.5 (1)	2.185 (7)
Asp18	Re1	OD2	–	2.1 (1)	2.30 (4)	1.99 (7)	2.13 (7)	2.14 (4)	2.09 (4)	2.2 (1)	2.14 (6)
Asn46	Re1	ND2	–	2.6 (1)	2.43 (4)	–	–	2.72 (5)	–	–	2.18 (1)
Asp52	Re1	OD2	–	2.3 (1)	2.40 (4)	–	–	2.45 (5)	–	–	2.14 (6)
Asp101	Re1	OD1/2	2.4 (2)	2.4 (1)	2.24 (4)	2.12 (6)	2.18 (6)	2.14 (4)	2.22 (3)	2.2 (1)	2.14 (6)
Asp119	Re1	OD2	2.5 (2)	2.3 (1)	2.24 (3)	2.17 (6)	2.23 (6)	2.22 (3)	2.26 (3)	2.3 (1)	2.14 (6)
Dataset resolution (Å)			1.75	1.68	1.132	1.23	1.41	1.21	1.15	1.69	–
*a* and *b* axis (Å)			78.59 (1)	80.99 (1)	81.08 (1)	80.75 (1)	80.92 (1)	81.23 (1)	81.12 (1)	81.1089 (2)	–
*c* axis (Å)			36.98 (3)	37.14 (3)	37.09 (3)	37.02 (3)	37.03 (3)	37.22 (3)	37.19 (3)	37.1986(2)	–
X-ray source (wavelength, Å)			Laboratory (1.54)	DLS (0.976)	DLS (0.976)	Laboratory (1.54)	Laboratory (1.54)	DLS (0.976)	DLS (0.976)	DLS (1.54)	–

† These standard uncertainty values were calculated using the *Online_DPI* server.

‡ These standard uncertainty values were calculated from the average distance of all bond distances fitting the description of the search criteria on the CSD.
