# Supplementary material for: Time-series analysis of rhenium(I) organometallic covalent binding to a model protein for drug development
Source: IUCrJ. 2024 Apr 19;11(Pt 3):359–73. doi: 10.1107/S2052252524002598 (PMC11067751; doi:10.1107/S2052252524002598)
Supplement: Supplementary file 1 [file m-11-00359-sup1.zip › Week 1 - V1_22Ffj1/Lab_Week1_refine_51.pdf]

```
REMARK   3
REMARK   3 REFINEMENT.
REMARK   3   PROGRAM     : PHENIX (1.20.1_4487: ???)
REMARK   3   AUTHORS     : Adams,Afonine,Bunkoczi,Burnley,Chen,Dar,Davis,
REMARK   3               : Draizen,Echols,Gildea,Gros,Grosse-Kunstleve,Headd,
REMARK   3               : Hintze,Hung,Ioerger,Liebschner,McCoy,McKee,Moriarty,
REMARK   3               : Oeffner,Poon,Read,Richardson,Richardson,Sacchettini,
REMARK   3               : Sauter,Sobolev,Storoni,Terwilliger,Williams,Zwart
REMARK   3
REMARK   3  X-RAY DATA.
REMARK   3
REMARK   3  REFINEMENT TARGET : ML
REMARK   3
REMARK   3  DATA USED IN REFINEMENT.
REMARK   3   RESOLUTION RANGE HIGH (ANGSTROMS) : 1.75
REMARK   3   RESOLUTION RANGE LOW  (ANGSTROMS) : 24.85
REMARK   3   MIN(FOBS/SIGMA_FOBS)              : 1.33
REMARK   3   COMPLETENESS FOR RANGE        (%) : 99.79
REMARK   3   NUMBER OF REFLECTIONS             : 22275
REMARK   3   NUMBER OF REFLECTIONS (NON-ANOMALOUS) : 12132
REMARK   3
REMARK   3  FIT TO DATA USED IN REFINEMENT.
REMARK   3   R VALUE     (WORKING + TEST SET) : 0.2000
REMARK   3   R VALUE            (WORKING SET) : 0.1945
REMARK   3   FREE R VALUE                     : 0.2503
REMARK   3   FREE R VALUE TEST SET SIZE   (%) : 10.00
REMARK   3   FREE R VALUE TEST SET COUNT      : 2228
REMARK   3
REMARK   3  FIT TO DATA USED IN REFINEMENT (IN BINS).
REMARK   3   BIN  RESOLUTION RANGE  COMPL.    NWORK NFREE   RWORK  RFREE  CCWORK CCFREE
REMARK   3     1   24.85 -    4.40    1.00     1257   145  0.1873 0.2051   0.900  0.851
REMARK   3     2    4.40 -    3.50    1.00     1254   138  0.1459 0.1998   0.937  0.914
REMARK   3     3    3.50 -    3.06    1.00     1252   137  0.1617 0.2531   0.913  0.846
REMARK   3     4    3.06 -    2.78    1.00     1248   140  0.1811 0.2006   0.904  0.863
REMARK   3     5    2.78 -    2.58    1.00     1254   138  0.1924 0.2394   0.889  0.839
REMARK   3     6    2.58 -    2.43    1.00     1252   137  0.1837 0.2686   0.894  0.748
REMARK   3     7    2.43 -    2.31    1.00     1254   136  0.1933 0.2455   0.892  0.852
REMARK   3     8    2.30 -    2.20    1.00     1254   148  0.2020 0.2591   0.879  0.811
REMARK   3     9    2.20 -    2.12    1.00     1239   134  0.2383 0.3048   0.860  0.747
REMARK   3    10    2.12 -    2.05    1.00     1265   141  0.2294 0.3412   0.852  0.672
REMARK   3    11    2.05 -    1.98    1.00     1255   143  0.2280 0.3062   0.859  0.759
REMARK   3    12    1.98 -    1.93    1.00     1223   135  0.2314 0.3284   0.846  0.675
REMARK   3    13    1.93 -    1.88    1.00     1284   139  0.2752 0.3066   0.822  0.735
REMARK   3    14    1.88 -    1.83    1.00     1229   138  0.2695 0.3444   0.790  0.693
REMARK   3    15    1.83 -    1.79    1.00     1296   140  0.3023 0.3488   0.779  0.609
REMARK   3    16    1.79 -    1.75    1.00     1231   139  0.3445 0.4010   0.735  0.560
REMARK   3
REMARK   3  BULK SOLVENT MODELLING.
REMARK   3   METHOD USED        : FLAT BULK SOLVENT MODEL
REMARK   3   SOLVENT RADIUS     : 1.10
REMARK   3   SHRINKAGE RADIUS   : 0.90
REMARK   3   GRID STEP FACTOR   : 4.00
REMARK   3
REMARK   3  ERROR ESTIMATES.
REMARK   3   COORDINATE ERROR (MAXIMUM-LIKELIHOOD BASED)     : 0.28
REMARK   3   PHASE ERROR (DEGREES, MAXIMUM-LIKELIHOOD BASED) : 25.91
REMARK   3
REMARK   3  STRUCTURE FACTORS CALCULATION ALGORITHM : FFT
REMARK   3  B VALUES.
REMARK   3   FROM WILSON PLOT           (A**2) : 22.37
REMARK   3
REMARK   3  GEOMETRY RESTRAINTS LIBRARY: GEOSTD + MONOMER LIBRARY + CDL V1.2
REMARK   3  DEVIATIONS FROM IDEAL VALUES - RMSD. RMSZ FOR BONDS AND ANGLES.
REMARK   3    BOND      :  0.016   0.077   1060  Z= 1.090
REMARK   3    ANGLE     :  1.567  12.165   1441  Z= 0.886
REMARK   3    CHIRALITY :  0.076   0.408    144
REMARK   3    PLANARITY :  0.016   0.092    184
REMARK   3    DIHEDRAL  : 15.912  86.991    374
REMARK   3    MIN NONBONDED DISTANCE : 2.301
REMARK   3
REMARK   3  MOLPROBITY STATISTICS.
REMARK   3    ALL-ATOM CLASHSCORE : 8.97
REMARK   3    RAMACHANDRAN PLOT:
REMARK   3      OUTLIERS :  0.00 %
REMARK   3      ALLOWED  :  2.36 %
REMARK   3      FAVORED  : 97.64 %
REMARK   3    ROTAMER OUTLIERS :  0.00 %
REMARK   3    CBETA DEVIATIONS :  0.00 %
```

```
REMARK   3    PEPTIDE PLANE:
REMARK   3      CIS-PROLINE     : 0.00 %
REMARK   3      CIS-GENERAL     : 0.00 %
REMARK   3      TWISTED PROLINE : 0.00 %
REMARK   3      TWISTED GENERAL : 0.00 %
REMARK   3
REMARK   3  RAMA-Z (RAMACHANDRAN PLOT Z-SCORE):
REMARK   3  INTERPRETATION: BAD |RAMA-Z| > 3; SUSPICIOUS 2 < |RAMA-Z| < 3; GOOD |RAMA-Z| < 2.
REMARK   3  SCORES FOR WHOLE/HELIX/SHEET/LOOP ARE SCALED INDEPENDENTLY;
REMARK   3  THEREFORE, THE VALUES ARE NOT RELATED IN A SIMPLE MANNER.
REMARK   3    WHOLE: -0.30 (0.72), RESIDUES: 127
REMARK   3    HELIX: -1.21 (0.66), RESIDUES: 45
REMARK   3    SHEET: -0.80 (1.50), RESIDUES: 10
REMARK   3    LOOP :  0.82 (0.77), RESIDUES: 72
REMARK   3
REMARK   3                  min    max   mean <Bi,j>   iso aniso
REMARK   3     Overall:   11.06  80.71  25.07   5.01  1136     0
REMARK   3     Protein:   11.06  80.71  24.33   4.54  1001     0
REMARK   3     Water:     14.11  44.74  27.80    N/A    89     0
REMARK   3     Other:     17.25  73.68  35.96    N/A    46     0
REMARK   3     Chain  A:  11.06  80.71  24.85    N/A  1045     0
REMARK   3     Chain  C:  27.69  27.69  27.69    N/A     1     0
REMARK   3     Chain  B:  17.25  17.25  17.25    N/A     1     0
REMARK   3     Chain  S:  14.11  44.74  27.80    N/A    89     0
REMARK   3     Histogram:
REMARK   3         Values      Number of atoms
REMARK   3      11.06 - 18.02       275
REMARK   3      18.02 - 24.99       420
REMARK   3      24.99 - 31.96       243
REMARK   3      31.96 - 38.92        97
REMARK   3      38.92 - 45.89        48
REMARK   3      45.89 - 52.85        24
REMARK   3      52.85 - 59.82        15
REMARK   3      59.82 - 66.78         8
REMARK   3      66.78 - 73.75         5
REMARK   3      73.75 - 80.71         1
REMARK   3
REMARK   3
LINK         NE2 HIS A  15                RE1  RI3 A1139
LINK         OD1 ASP A 101                RE1  RIW A1141
LINK         OD2 ASP A 119                RE1  RI3 A1142
SSBOND   1 CYS A    6    CYS A  127
SSBOND   2 CYS A   30    CYS A  115
SSBOND   3 CYS A   64    CYS A   80
SSBOND   4 CYS A   76    CYS A   94
CRYST1   78.597   78.597   36.976  90.00  90.00  90.00 P 43 21 2
SCALE1      0.012723  0.000000  0.000000        0.00000
SCALE2      0.000000  0.012723  0.000000        0.00000
SCALE3      0.000000  0.000000  0.027045        0.00000
ATOM      1  N   LYS A   1      -3.375 -10.277  -8.575  1.00 22.15           N  0.080
ATOM      2  CA  LYS A   1      -2.431 -10.653  -9.670  1.00 23.18           C  0.081
ATOM      3  C   LYS A   1      -2.435 -12.153  -9.874  1.00 24.46           C  0.084
ATOM      4  O   LYS A   1      -2.361 -12.918  -8.898  1.00 25.85           O  0.086
ATOM      5  CB  LYS A   1      -1.018 -10.168  -9.345  1.00 21.31           C  0.078
ATOM      6  CG  LYS A   1      -0.035 -10.492 -10.435  1.00 29.78           C  0.092
ATOM      7  CD  LYS A   1       1.355 -10.433  -9.882  1.00 28.56           C  0.090
ATOM      8  CE  LYS A   1       2.393 -10.491 -10.963  1.00 29.92           C  0.093
ATOM      9  NZ  LYS A   1       3.686 -10.024 -10.411  1.00 42.16           N  0.110
ATOM     10  H1  LYS A   1      -3.380  -9.393  -8.479  1.00 26.58           H  0.087
ATOM     11  H2  LYS A   1      -4.195 -10.558  -8.781  1.00 26.58           H  0.087
ATOM     12  H3  LYS A   1      -3.114 -10.658  -7.814  1.00 26.58           H  0.087
ATOM     13  HA  LYS A   1      -2.707 -10.229 -10.498  1.00 27.81           H  0.089
ATOM     14  HB2 LYS A   1      -1.033  -9.206  -9.228  1.00 25.57           H  0.086
ATOM     15  HB3 LYS A   1      -0.716 -10.597  -8.529  1.00 25.57           H  0.086
ATOM     16  HG2 LYS A   1      -0.201 -11.386 -10.773  1.00 35.73           H  0.101
ATOM     17  HG3 LYS A   1      -0.115  -9.845 -11.153  1.00 35.73           H  0.101
ATOM     18  HD2 LYS A   1       1.471  -9.601  -9.396  1.00 34.28           H  0.099
ATOM     19  HD3 LYS A   1       1.495 -11.186  -9.287  1.00 34.28           H  0.099
ATOM     20  HE2 LYS A   1       2.494 -11.403 -11.277  1.00 35.90           H  0.101
ATOM     21  HE3 LYS A   1       2.137  -9.913 -11.700  1.00 35.90           H  0.101
ATOM     22  HZ1 LYS A   1       4.311 -10.039 -11.045  1.00 50.59           H  0.120
ATOM     23  HZ2 LYS A   1       3.604  -9.192 -10.106  1.00 50.59           H  0.120
ATOM     24  HZ3 LYS A   1       3.940 -10.553  -9.742  1.00 50.59           H  0.120
ATOM     25  N   VAL A   2      -2.529 -12.594 -11.139  1.00 21.31           N  0.078
ATOM     26  CA  VAL A   2      -2.406 -13.999 -11.477  1.00 22.89           C  0.081
ATOM     27  C   VAL A   2      -0.969 -14.210 -11.961  1.00 29.38           C  0.092
ATOM     28  O   VAL A   2      -0.554 -13.679 -13.004  1.00 23.40           O  0.082
ATOM     29  CB  VAL A   2      -3.468 -14.438 -12.487  1.00 21.93           C  0.079
```

```
ATOM     30  CG1 VAL A   2      -3.308 -15.855 -12.858  1.00 21.55           C  0.079
ATOM     31  CG2 VAL A   2      -4.905 -14.272 -11.900  1.00 21.98           C  0.079
ATOM     32  H   VAL A   2      -2.666 -12.083 -11.817  1.00 25.58           H  0.086
ATOM     33  HA  VAL A   2      -2.534 -14.553 -10.691  1.00 27.46           H  0.089
ATOM     34  HB  VAL A   2      -3.357 -13.876 -13.269  1.00 26.31           H  0.087
ATOM     35 HG11 VAL A   2      -4.073 -16.132 -13.385  1.00 25.86           H  0.086
ATOM     36 HG12 VAL A   2      -2.495 -15.955 -13.377  1.00 25.86           H  0.086
ATOM     37 HG13 VAL A   2      -3.252 -16.389 -12.050  1.00 25.86           H  0.086
ATOM     38 HG21 VAL A   2      -5.549 -14.604 -12.544  1.00 26.37           H  0.087
ATOM     39 HG22 VAL A   2      -4.969 -14.779 -11.075  1.00 26.37           H  0.087
ATOM     40 HG23 VAL A   2      -5.067 -13.332 -11.723  1.00 26.37           H  0.087
ATOM     41  N   PHE A   3      -0.194 -14.942 -11.159  1.00 21.90           N  0.079
ATOM     42  CA  PHE A   3       1.191 -15.232 -11.497  1.00 22.40           C  0.080
ATOM     43  C   PHE A   3       1.268 -16.302 -12.558  1.00 23.32           C  0.082
ATOM     44  O   PHE A   3       0.431 -17.212 -12.633  1.00 17.06           O  0.070
ATOM     45  CB  PHE A   3       1.992 -15.732 -10.286  1.00 21.07           C  0.078
ATOM     46  CG  PHE A   3       2.482 -14.651  -9.420  1.00 26.72           C  0.088
ATOM     47  CD1 PHE A   3       1.610 -13.976  -8.582  1.00 24.46           C  0.084
ATOM     48  CD2 PHE A   3       3.801 -14.324  -9.416  1.00 24.05           C  0.083
ATOM     49  CE1 PHE A   3       2.075 -12.973  -7.762  1.00 27.69           C  0.089
ATOM     50  CE2 PHE A   3       4.276 -13.315  -8.635  1.00 25.21           C  0.085
ATOM     51  CZ  PHE A   3       3.419 -12.629  -7.802  1.00 31.54           C  0.095
ATOM     52  H   PHE A   3      -0.451 -15.281 -10.412  1.00 26.28           H  0.087
ATOM     53  HA  PHE A   3       1.590 -14.408 -11.815  1.00 26.87           H  0.088
ATOM     54  HB2 PHE A   3       1.424 -16.307  -9.751  1.00 25.29           H  0.085
ATOM     55  HB3 PHE A   3       2.762 -16.229 -10.604  1.00 25.29           H  0.085
ATOM     56  HD1 PHE A   3       0.708 -14.201  -8.572  1.00 29.35           H  0.092
ATOM     57  HD2 PHE A   3       4.390 -14.798  -9.957  1.00 28.86           H  0.091
ATOM     58  HE1 PHE A   3       1.494 -12.530  -7.187  1.00 33.23           H  0.098
ATOM     59  HE2 PHE A   3       5.178 -13.090  -8.664  1.00 30.26           H  0.093
ATOM     60  HZ  PHE A   3       3.740 -11.937  -7.268  1.00 37.84           H  0.104
ATOM     61  N   GLY A   4       2.308 -16.221 -13.382  1.00 33.26           N  0.098
ATOM     62  CA  GLY A   4       2.703 -17.374 -14.149  1.00 25.86           C  0.086
ATOM     63  C   GLY A   4       3.437 -18.352 -13.240  1.00 21.47           C  0.078
ATOM     64  O   GLY A   4       3.989 -17.983 -12.202  1.00 23.14           O  0.081
ATOM     65  H   GLY A   4       2.788 -15.518 -13.506  1.00 39.91           H  0.107
ATOM     66  HA2 GLY A   4       1.921 -17.809 -14.523  1.00 31.04           H  0.094
ATOM     67  HA3 GLY A   4       3.291 -17.107 -14.873  1.00 31.04           H  0.094
ATOM     68  N   ARG A   5       3.382 -19.618 -13.635  1.00 21.03           N  0.078
ATOM     69  CA  ARG A   5       4.099 -20.677 -12.950  1.00 21.93           C  0.079
ATOM     70  C   ARG A   5       5.555 -20.311 -12.677  1.00 35.63           C  0.101
ATOM     71  O   ARG A   5       6.001 -20.331 -11.521  1.00 21.80           O  0.079
ATOM     72  CB  ARG A   5       3.975 -21.942 -13.796  1.00 26.69           C  0.087
ATOM     73  CG  ARG A   5       4.601 -23.181 -13.179  1.00 27.86           C  0.089
ATOM     74  CD  ARG A   5       4.397 -24.373 -14.044  1.00 29.31           C  0.092
ATOM     75  NE  ARG A   5       4.977 -24.239 -15.383  1.00 33.99           N  0.099
ATOM     76  CZ  ARG A   5       6.236 -24.538 -15.695  1.00 28.42           C  0.090
ATOM     77  NH1 ARG A   5       7.149 -24.812 -14.758  1.00 27.19           N  0.088
ATOM     78  NH2 ARG A   5       6.597 -24.538 -16.971  1.00 29.10           N  0.091
ATOM     79  H   ARG A   5       2.927 -19.894 -14.311  1.00 25.24           H  0.085
ATOM     80  HA  ARG A   5       3.702 -20.835 -12.079  1.00 26.32           H  0.087
ATOM     81  HB2 ARG A   5       3.033 -22.128 -13.935  1.00 32.03           H  0.096
ATOM     82  HB3 ARG A   5       4.412 -21.787 -14.648  1.00 32.03           H  0.096
ATOM     83  HG2 ARG A   5       5.555 -23.040 -13.071  1.00 33.43           H  0.098
ATOM     84  HG3 ARG A   5       4.192 -23.354 -12.317  1.00 33.43           H  0.098
ATOM     85  HD2 ARG A   5       4.809 -25.141 -13.618  1.00 35.17           H  0.100
ATOM     86  HD3 ARG A   5       3.444 -24.523 -14.148  1.00 35.17           H  0.100
ATOM     87  HE  ARG A   5       4.468 -23.947 -16.012  1.00 40.79           H  0.108
ATOM     88 HH11 ARG A   5       6.930 -24.799 -13.926  1.00 32.63           H  0.097
ATOM     89 HH12 ARG A   5       7.956 -25.002 -14.987  1.00 32.63           H  0.097
ATOM     90 HH21 ARG A   5       6.022 -24.346 -17.581  1.00 34.93           H  0.100
ATOM     91 HH22 ARG A   5       7.407 -24.729 -17.188  1.00 34.93           H  0.100
ATOM     92  N   CYS A   6       6.344 -19.981 -13.722  1.00 23.45           N  0.082
ATOM     93  CA  CYS A   6       7.748 -19.691 -13.440  1.00 28.84           C  0.091
ATOM     94  C   CYS A   6       7.923 -18.350 -12.727  1.00 23.02           C  0.081
ATOM     95  O   CYS A   6       8.838 -18.204 -11.917  1.00 24.98           O  0.085
ATOM     96  CB  CYS A   6       8.575 -19.755 -14.715  1.00 26.77           C  0.088
ATOM     97  SG  CYS A   6       8.645 -21.369 -15.424  1.00 29.74           S  0.092
ATOM     98  H   CYS A   6       6.100 -19.925 -14.544  1.00 28.14           H  0.090
ATOM     99  HA  CYS A   6       8.091 -20.377 -12.846  1.00 34.61           H  0.100
ATOM    100  HB2 CYS A   6       8.185 -19.158 -15.372  1.00 32.13           H  0.096
ATOM    101  HB3 CYS A   6       9.483 -19.479 -14.513  1.00 32.13           H  0.096
ATOM    102  N   GLU A   7       7.069 -17.354 -13.011  1.00 25.31           N  0.085
ATOM    103  CA  GLU A   7       7.127 -16.064 -12.313  1.00 28.81           C  0.091
ATOM    104  C   GLU A   7       6.976 -16.245 -10.808  1.00 23.25           C  0.082
ATOM    105  O   GLU A   7       7.722 -15.671  -9.994  1.00 23.19           O  0.082
ATOM    106  CB  GLU A   7       6.004 -15.177 -12.852  1.00 30.51           C  0.094
ATOM    107  CG  GLU A   7       6.032 -13.742 -12.319  1.00 37.71           C  0.104
```

ATOM	108	CD	GLU	A	7	4.824	-12.939	-12.771	1.00	36.61	C	0.102
ATOM	109	OE1	GLU	A	7	3.788	-13.557	-13.192	1.00	30.30	O	0.093
ATOM	110	OE2	GLU	A	7	4.904	-11.685	-12.693	1.00	30.08	O	0.093
ATOM	111	H	GLU	A	7	6.447	-17.402	-13.603	1.00	30.37	H	0.093
ATOM	112	HA	GLU	A	7	7.985	-15.642	-12.477	1.00	34.57	H	0.100
ATOM	113	HB2	GLU	A	7	6.078	-15.134	-13.818	1.00	36.61	H	0.102
ATOM	114	HB3	GLU	A	7	5.152	-15.567	-12.601	1.00	36.61	H	0.102
ATOM	115	HG2	GLU	A	7	6.037	-13.763	-11.349	1.00	45.25	H	0.114
ATOM	116	HG3	GLU	A	7	6.830	-13.297	-12.645	1.00	45.25	H	0.114
ATOM	117	N	LEU	A	8	5.991	-17.038	-10.421	1.00	21.74	N	0.079
ATOM	118	CA	LEU	A	8	5.766	-17.307	-9.008	1.00	21.15	C	0.078
ATOM	119	C	LEU	A	8	6.905	-18.092	-8.407	1.00	20.31	C	0.076
ATOM	120	O	LEU	A	8	7.367	-17.791	-7.308	1.00	19.69	O	0.075
ATOM	121	CB	LEU	A	8	4.468	-18.072	-8.834	1.00	21.41	C	0.078
ATOM	122	CG	LEU	A	8	4.125	-18.294	-7.380	1.00	18.10	C	0.072
ATOM	123	CD1	LEU	A	8	4.014	-16.992	-6.632	1.00	26.37	C	0.087
ATOM	124	CD2	LEU	A	8	2.851	-19.144	-7.362	1.00	16.24	C	0.068
ATOM	125	H	LEU	A	8	5.440	-17.432	-10.951	1.00	26.09	H	0.086
ATOM	126	HA	LEU	A	8	5.699	-16.460	-8.541	1.00	25.38	H	0.085
ATOM	127	HB2	LEU	A	8	3.747	-17.569	-9.243	1.00	25.70	H	0.086
ATOM	128	HB3	LEU	A	8	4.552	-18.939	-9.261	1.00	25.70	H	0.086
ATOM	129	HG	LEU	A	8	4.823	-18.767	-6.901	1.00	21.72	H	0.079
ATOM	130	HD11	LEU	A	8	3.516	-17.140	-5.813	1.00	31.65	H	0.095
ATOM	131	HD12	LEU	A	8	4.905	-16.670	-6.425	1.00	31.65	H	0.095
ATOM	132	HD13	LEU	A	8	3.550	-16.347	-7.189	1.00	31.65	H	0.095
ATOM	133	HD21	LEU	A	8	2.596	-19.311	-6.441	1.00	19.49	H	0.075
ATOM	134	HD22	LEU	A	8	2.144	-18.662	-7.819	1.00	19.49	H	0.075
ATOM	135	HD23	LEU	A	8	3.025	-19.984	-7.815	1.00	19.49	H	0.075
ATOM	136	N	ALA	A	9	7.337	-19.141	-9.094	1.00	23.52	N	0.082
ATOM	137	CA	ALA	A	9	8.540	-19.855	-8.684	1.00	19.99	C	0.076
ATOM	138	C	ALA	A	9	9.675	-18.896	-8.368	1.00	24.26	C	0.083
ATOM	139	O	ALA	A	9	10.377	-19.025	-7.349	1.00	19.40	O	0.075
ATOM	140	CB	ALA	A	9	8.948	-20.806	-9.806	1.00	21.62	C	0.079
ATOM	141	H	ALA	A	9	6.956	-19.459	-9.797	1.00	28.22	H	0.090
ATOM	142	HA	ALA	A	9	8.354	-20.366	-7.881	1.00	23.99	H	0.083
ATOM	143	HB1	ALA	A	9	9.722	-21.316	-9.521	1.00	25.94	H	0.086
ATOM	144	HB2	ALA	A	9	8.209	-21.404	-9.996	1.00	25.94	H	0.086
ATOM	145	HB3	ALA	A	9	9.166	-20.287	-10.596	1.00	25.94	H	0.086
ATOM	146	N	ALA	A	10	9.905	-17.942	-9.263	1.00	26.36	N	0.087
ATOM	147	CA	ALA	A	10	10.999	-17.009	-9.071	1.00	26.57	C	0.087
ATOM	148	C	ALA	A	10	10.751	-16.137	-7.863	1.00	26.66	C	0.087
ATOM	149	O	ALA	A	10	11.685	-15.834	-7.100	1.00	27.85	O	0.089
ATOM	150	CB	ALA	A	10	11.144	-16.149	-10.328	1.00	32.72	C	0.097
ATOM	151	H	ALA	A	10	9.447	-17.816	-9.980	1.00	31.63	H	0.095
ATOM	152	HA	ALA	A	10	11.826	-17.497	-8.929	1.00	31.88	H	0.096
ATOM	153	HB1	ALA	A	10	11.886	-15.536	-10.207	1.00	39.26	H	0.106
ATOM	154	HB2	ALA	A	10	11.313	-16.727	-11.088	1.00	39.26	H	0.106
ATOM	155	HB3	ALA	A	10	10.323	-15.652	-10.467	1.00	39.26	H	0.106
ATOM	156	N	ALA	A	11	9.521	-15.649	-7.702	1.00	28.61	N	0.091
ATOM	157	CA	ALA	A	11	9.282	-14.729	-6.610	1.00	26.89	C	0.088
ATOM	158	C	ALA	A	11	9.349	-15.481	-5.293	1.00	22.91	C	0.081
ATOM	159	O	ALA	A	11	9.826	-14.957	-4.275	1.00	27.01	O	0.088
ATOM	160	CB	ALA	A	11	7.941	-14.025	-6.763	1.00	30.16	C	0.093
ATOM	161	H	ALA	A	11	8.840	-15.831	-8.195	1.00	34.33	H	0.099
ATOM	162	HA	ALA	A	11	9.959	-14.034	-6.624	1.00	32.27	H	0.096
ATOM	163	HB1	ALA	A	11	7.813	-13.423	-6.013	1.00	36.19	H	0.102
ATOM	164	HB2	ALA	A	11	7.940	-13.523	-7.593	1.00	36.19	H	0.102
ATOM	165	HB3	ALA	A	11	7.235	-14.690	-6.777	1.00	36.19	H	0.102
ATOM	166	N	MET	A	12	8.859	-16.718	-5.301	1.00	21.88	N	0.079
ATOM	167	CA	MET	A	12	8.934	-17.517	-4.091	1.00	21.02	C	0.078
ATOM	168	C	MET	A	12	10.377	-17.733	-3.701	1.00	24.30	C	0.083
ATOM	169	O	MET	A	12	10.745	-17.600	-2.534	1.00	26.61	O	0.087
ATOM	170	CB	MET	A	12	8.239	-18.845	-4.338	1.00	22.89	C	0.081
ATOM	171	CG	MET	A	12	6.765	-18.765	-4.113	1.00	20.82	C	0.077
ATOM	172	SD	MET	A	12	5.941	-20.287	-4.549	1.00	21.50	S	0.078
ATOM	173	CE	MET	A	12	4.367	-19.958	-3.698	1.00	22.26	C	0.080
ATOM	174	H	MET	A	12	8.490	-17.104	-5.976	1.00	26.26	H	0.087
ATOM	175	HA	MET	A	12	8.476	-17.068	-3.363	1.00	25.22	H	0.085
ATOM	176	HB2	MET	A	12	8.390	-19.116	-5.257	1.00	27.47	H	0.089
ATOM	177	HB3	MET	A	12	8.601	-19.510	-3.732	1.00	27.47	H	0.089
ATOM	178	HG2	MET	A	12	6.594	-18.586	-3.176	1.00	24.98	H	0.085
ATOM	179	HG3	MET	A	12	6.397	-18.054	-4.660	1.00	24.98	H	0.085
ATOM	180	HE1	MET	A	12	3.765	-20.704	-3.848	1.00	26.72	H	0.088
ATOM	181	HE2	MET	A	12	4.537	-19.855	-2.749	1.00	26.72	H	0.088
ATOM	182	HE3	MET	A	12	3.979	-19.143	-4.055	1.00	26.72	H	0.088
ATOM	183	N	LYS	A	13	11.217	-18.049	-4.692	1.00	28.28	N	0.090
ATOM	184	CA	LYS	A	13	12.632	-18.303	-4.426	1.00	27.02	C	0.088
ATOM	185	C	LYS	A	13	13.331	-17.051	-3.900	1.00	28.28	C	0.090

ATOM	186	O	LYS	A	13	14.133	-17.114	-2.941	1.00	28.92	O	0.091
ATOM	187	CB	LYS	A	13	13.285	-18.823	-5.708	1.00	26.87	C	0.088
ATOM	188	CG	LYS	A	13	14.812	-18.778	-5.709	1.00	38.71	C	0.105
ATOM	189	CD	LYS	A	13	15.414	-19.494	-6.969	1.00	44.59	C	0.113
ATOM	190	CE	LYS	A	13	16.968	-19.511	-6.980	1.00	52.11	C	0.122
ATOM	191	NZ	LYS	A	13	17.586	-18.352	-6.246	1.00	51.38	N	0.121
ATOM	192	H	LYS	A	13	10.993	-18.121	-5.519	1.00	33.93	H	0.099
ATOM	193	HA	LYS	A	13	12.727	-18.983	-3.741	1.00	32.42	H	0.096
ATOM	194	HB2	LYS	A	13	13.020	-19.747	-5.836	1.00	32.25	H	0.096
ATOM	195	HB3	LYS	A	13	12.976	-18.283	-6.452	1.00	32.25	H	0.096
ATOM	196	HG2	LYS	A	13	15.105	-17.854	-5.716	1.00	46.45	H	0.115
ATOM	197	HG3	LYS	A	13	15.146	-19.226	-4.916	1.00	46.45	H	0.115
ATOM	198	HD2	LYS	A	13	15.106	-20.414	-6.985	1.00	53.51	H	0.124
ATOM	199	HD3	LYS	A	13	15.116	-19.030	-7.767	1.00	53.51	H	0.124
ATOM	200	HE2	LYS	A	13	17.276	-20.328	-6.556	1.00	62.53	H	0.134
ATOM	201	HE3	LYS	A	13	17.276	-19.478	-7.899	1.00	62.53	H	0.134
ATOM	202	HZ1	LYS	A	13	18.473	-18.406	-6.284	1.00	61.65	H	0.133
ATOM	203	HZ2	LYS	A	13	17.326	-17.585	-6.615	1.00	61.65	H	0.133
ATOM	204	HZ3	LYS	A	13	17.332	-18.363	-5.393	1.00	61.65	H	0.133
ATOM	205	N	ARG	A	14	12.962	-15.893	-4.454	1.00	25.10	N	0.085
ATOM	206	CA	ARG	A	14	13.477	-14.605	-4.014	1.00	31.42	C	0.095
ATOM	207	C	ARG	A	14	13.114	-14.333	-2.559	1.00	40.97	C	0.108
ATOM	208	O	ARG	A	14	13.842	-13.625	-1.850	1.00	38.53	O	0.105
ATOM	209	CB	ARG	A	14	12.909	-13.521	-4.951	1.00	32.27	C	0.096
ATOM	210	CG	ARG	A	14	13.036	-12.059	-4.477	1.00	42.24	C	0.110
ATOM	211	CD	ARG	A	14	14.265	-11.355	-5.100	1.00	40.46	C	0.108
ATOM	212	NE	ARG	A	14	14.927	-10.483	-4.140	1.00	42.56	N	0.110
ATOM	213	CZ	ARG	A	14	14.494	-9.274	-3.795	1.00	50.23	C	0.120
ATOM	214	NH1	ARG	A	14	13.337	-8.801	-4.235	1.00	47.97	N	0.117
ATOM	215	NH2	ARG	A	14	15.223	-8.539	-2.951	1.00	43.42	N	0.112
ATOM	216	H	ARG	A	14	12.401	-15.828	-5.102	1.00	30.12	H	0.093
ATOM	217	HA	ARG	A	14	14.445	-14.583	-4.068	1.00	37.71	H	0.104
ATOM	218	HB2	ARG	A	14	13.374	-13.585	-5.800	1.00	38.73	H	0.105
ATOM	219	HB3	ARG	A	14	11.964	-13.698	-5.077	1.00	38.73	H	0.105
ATOM	220	HG2	ARG	A	14	12.241	-11.568	-4.737	1.00	50.69	H	0.121
ATOM	221	HG3	ARG	A	14	13.135	-12.044	-3.512	1.00	50.69	H	0.121
ATOM	222	HD2	ARG	A	14	14.902	-12.024	-5.394	1.00	48.55	H	0.118
ATOM	223	HD3	ARG	A	14	13.977	-10.816	-5.853	1.00	48.55	H	0.118
ATOM	224	HE	ARG	A	14	15.649	-10.770	-3.771	1.00	51.08	H	0.121
ATOM	225	HH11	ARG	A	14	12.848	-9.279	-4.756	1.00	57.57	H	0.128
ATOM	226	HH12	ARG	A	14	13.076	-8.016	-4.000	1.00	57.57	H	0.128
ATOM	227	HH21	ARG	A	14	15.961	-8.850	-2.638	1.00	52.11	H	0.122
ATOM	228	HH22	ARG	A	14	14.954	-7.756	-2.720	1.00	52.11	H	0.122
ATOM	229	N	HIS	A	15	11.948	-14.851	-2.172	1.00	30.75	N	0.094
ATOM	230	CA	HIS	A	15	11.351	-14.619	-0.844	1.00	28.18	C	0.090
ATOM	231	C	HIS	A	15	11.767	-15.703	0.144	1.00	29.22	C	0.092
ATOM	232	O	HIS	A	15	11.240	-15.642	1.227	1.00	34.84	O	0.100
ATOM	233	CB	HIS	A	15	9.843	-14.494	-1.052	1.00	32.15	C	0.096
ATOM	234	CG	HIS	A	15	9.480	-13.159	-1.594	1.00	36.82	C	0.103
ATOM	235	ND1	HIS	A	15	9.541	-12.063	-0.778	1.00	40.74	N	0.108
ATOM	236	CD2	HIS	A	15	9.180	-12.707	-2.843	1.00	35.03	C	0.100
ATOM	237	CE1	HIS	A	15	9.214	-11.017	-1.491	1.00	42.86	C	0.111
ATOM	238	NE2	HIS	A	15	8.956	-11.413	-2.759	1.00	43.31	N	0.111
ATOM	239	H	HIS	A	15	11.358	-15.451	-2.776	1.00	36.90	H	0.103
ATOM	240	HA	HIS	A	15	11.722	-13.665	-0.473	1.00	33.81	H	0.098
ATOM	241	HB2	HIS	A	15	9.507	-15.267	-1.742	1.00	38.57	H	0.105
ATOM	242	HB3	HIS	A	15	9.333	-14.643	-0.102	1.00	38.57	H	0.105
ATOM	243	HD1	HIS	A	15	9.822	-12.062	0.198	1.00	48.89	H	0.118
ATOM	244	HD2	HIS	A	15	8.925	-13.261	-3.712	1.00	42.04	H	0.110
ATOM	245	HE1	HIS	A	15	9.221	-10.003	-1.139	1.00	51.44	H	0.121
ATOM	246	N	GLY	A	16	12.590	-16.680	-0.233	1.00	29.27	N	0.092
ATOM	247	CA	GLY	A	16	13.187	-17.628	0.686	1.00	28.01	C	0.090
ATOM	248	C	GLY	A	16	12.426	-18.908	0.906	1.00	24.10	C	0.083
ATOM	249	O	GLY	A	16	12.697	-19.598	1.889	1.00	26.46	O	0.087
ATOM	250	H	GLY	A	16	12.809	-16.799	-1.056	1.00	35.13	H	0.100
ATOM	251	HA2	GLY	A	16	14.066	-17.866	0.351	1.00	33.61	H	0.098
ATOM	252	HA3	GLY	A	16	13.285	-17.197	1.549	1.00	33.61	H	0.098
ATOM	253	N	LEU	A	17	11.486	-19.260	0.015	1.00	27.65	N	0.089
ATOM	254	CA	LEU	A	17	10.720	-20.476	0.173	1.00	24.74	C	0.084
ATOM	255	C	LEU	A	17	11.430	-21.721	-0.347	1.00	27.47	C	0.089
ATOM	256	O	LEU	A	17	11.104	-22.837	0.094	1.00	30.88	O	0.094
ATOM	257	CB	LEU	A	17	9.365	-20.351	-0.565	1.00	23.01	C	0.081
ATOM	258	CG	LEU	A	17	8.214	-19.749	0.250	1.00	25.65	C	0.086
ATOM	259	CD1	LEU	A	17	6.849	-19.920	-0.502	1.00	19.63	C	0.075
ATOM	260	CD2	LEU	A	17	8.141	-20.351	1.669	1.00	22.99	C	0.081
ATOM	261	H	LEU	A	17	11.281	-18.803	-0.684	1.00	33.18	H	0.098
ATOM	262	HA	LEU	A	17	10.563	-20.596	1.123	1.00	29.69	H	0.092
ATOM	263	HB2	LEU	A	17	9.495	-19.785	-1.342	1.00	27.61	H	0.089

```
ATOM    264  HB3 LEU A  17       9.088 -21.238  -0.843  1.00 27.61           H  0.089
ATOM    265  HG  LEU A  17       8.382 -18.799   0.353  1.00 30.79           H  0.094
ATOM    266 HD11 LEU A  17       6.183 -19.356  -0.080  1.00 23.55           H  0.082
ATOM    267 HD12 LEU A  17       6.965 -19.660  -1.429  1.00 23.55           H  0.082
ATOM    268 HD13 LEU A  17       6.575 -20.850  -0.451  1.00 23.55           H  0.082
ATOM    269 HD21 LEU A  17       7.256 -20.194   2.034  1.00 27.59           H  0.089
ATOM    270 HD22 LEU A  17       8.313 -21.304   1.617  1.00 27.59           H  0.089
ATOM    271 HD23 LEU A  17       8.809 -19.924   2.228  1.00 27.59           H  0.089
ATOM    272  N   ASP A  18      12.378 -21.569  -1.243  1.00 24.67           N  0.084
ATOM    273  CA  ASP A  18      13.051 -22.733  -1.853  1.00 24.98           C  0.085
ATOM    274  C   ASP A  18      13.788 -23.514  -0.760  1.00 26.52           C  0.087
ATOM    275  O   ASP A  18      14.787 -23.004  -0.252  1.00 26.29           O  0.087
ATOM    276  CB  ASP A  18      13.931 -22.330  -3.032  1.00 31.63           C  0.095
ATOM    277  CG  ASP A  18      14.923 -21.209  -2.774  1.00 41.17           C  0.109
ATOM    278  OD1 ASP A  18      14.539 -20.200  -2.148  1.00 41.43           O  0.109
ATOM    279  OD2 ASP A  18      16.066 -21.348  -3.234  1.00 49.81           O  0.119
ATOM    280  H   ASP A  18      12.694 -20.646  -1.591  1.00 29.60           H  0.092
ATOM    281  HA  ASP A  18      12.272 -23.382  -2.250  1.00 29.98           H  0.093
ATOM    282  HB2 ASP A  18      14.488 -23.206  -3.360  1.00 37.96           H  0.104
ATOM    283  HB3 ASP A  18      13.282 -22.019  -3.850  1.00 37.96           H  0.104
ATOM    284  N   ASN A  19      13.294 -24.711  -0.436  1.00 24.90           N  0.084
ATOM    285  CA  ASN A  19      13.881 -25.576   0.583  1.00 20.83           C  0.077
ATOM    286  C   ASN A  19      13.561 -25.025   1.962  1.00 24.49           C  0.084
ATOM    287  O   ASN A  19      14.154 -25.535   2.990  1.00 21.75           O  0.079
ATOM    288  CB  ASN A  19      15.395 -25.738   0.381  1.00 25.16           C  0.085
ATOM    289  CG  ASN A  19      15.884 -27.148   0.647  1.00 37.72           C  0.104
ATOM    290  OD1 ASN A  19      15.087 -28.093   0.792  1.00 46.75           O  0.116
ATOM    291  ND2 ASN A  19      17.213 -27.298   0.786  1.00 39.72           N  0.107
ATOM    292  HA  ASN A  19      13.514 -26.473   0.532  1.00 24.99           H  0.085
ATOM    293  HB2 ASN A  19      15.616 -25.513  -0.537  1.00 30.19           H  0.093
ATOM    294  HB3 ASN A  19      15.859 -25.141   0.989  1.00 30.19           H  0.093
ATOM    295 HD21 ASN A  19      17.544 -28.077   0.939  1.00 47.66           H  0.117
ATOM    296 HD22 ASN A  19      17.732 -26.616   0.723  1.00 47.66           H  0.117
ATOM    297  H  AASN A  19      12.595 -25.057  -0.798  0.57 29.88           H  0.093
ATOM    298  H  BASN A  19      12.579 -25.043  -0.779  0.43 29.88           H  0.093
ATOM    299  N   TYR A  20      12.628 -24.061   2.091  1.00 21.85           N  0.079
ATOM    300  CA  TYR A  20      12.233 -23.615   3.435  1.00 19.11           C  0.074
ATOM    301  C   TYR A  20      11.640 -24.783   4.206  1.00 20.34           C  0.076
ATOM    302  O   TYR A  20      10.748 -25.487   3.708  1.00 19.55           O  0.075
ATOM    303  CB  TYR A  20      11.196 -22.475   3.428  1.00 20.93           C  0.077
ATOM    304  CG  TYR A  20      11.036 -21.880   4.818  1.00 25.13           C  0.085
ATOM    305  CD1 TYR A  20      11.939 -20.958   5.292  1.00 25.90           C  0.086
ATOM    306  CD2 TYR A  20      10.034 -22.299   5.683  1.00 22.69           C  0.081
ATOM    307  CE1 TYR A  20      11.827 -20.456   6.565  1.00 27.39           C  0.089
ATOM    308  CE2 TYR A  20       9.929 -21.801   6.948  1.00 21.81           C  0.079
ATOM    309  CZ  TYR A  20      10.839 -20.883   7.383  1.00 26.73           C  0.088
ATOM    310  OH  TYR A  20      10.749 -20.381   8.651  1.00 29.75           O  0.092
ATOM    311  H   TYR A  20      12.226 -23.667   1.441  1.00 26.22           H  0.087
ATOM    312  HA  TYR A  20      13.034 -23.272   3.862  1.00 22.93           H  0.081
ATOM    313  HB2 TYR A  20      11.490 -21.774   2.825  1.00 25.12           H  0.085
ATOM    314  HB3 TYR A  20      10.337 -22.821   3.140  1.00 25.12           H  0.085
ATOM    315  HD1 TYR A  20      12.633 -20.672   4.744  1.00 31.08           H  0.094
ATOM    316  HD2 TYR A  20       9.421 -22.935   5.393  1.00 27.23           H  0.088
ATOM    317  HE1 TYR A  20      12.434 -19.818   6.865  1.00 32.86           H  0.097
ATOM    318  HE2 TYR A  20       9.242 -22.084   7.508  1.00 26.17           H  0.087
ATOM    319  HH  TYR A  20      11.350 -19.808   8.776  1.00 35.70           H  0.101
ATOM    320  N   ARG A  21      12.145 -25.020   5.412  1.00 23.57           N  0.082
ATOM    321  CA  ARG A  21      11.729 -26.192   6.201  1.00 24.42           C  0.084
ATOM    322  C   ARG A  21      11.828 -27.457   5.356  1.00 20.28           C  0.076
ATOM    323  O   ARG A  21      11.097 -28.452   5.568  1.00 21.64           O  0.079
ATOM    324  CB  ARG A  21      10.320 -26.038   6.776  1.00 24.13           C  0.083
ATOM    325  CG  ARG A  21      10.306 -25.359   8.146  1.00 35.50           C  0.101
ATOM    326  CD  ARG A  21      10.583 -26.360   9.231  1.00 30.33           C  0.093
ATOM    327  NE  ARG A  21      10.458 -25.753  10.542  1.00 54.74           N  0.125
ATOM    328  CZ  ARG A  21      10.386 -26.421  11.689  1.00 37.35           C  0.103
ATOM    329  NH1 ARG A  21      10.291 -27.745  11.723  1.00 44.45           N  0.113
ATOM    330  NH2 ARG A  21      10.398 -25.742  12.827  1.00 44.50           N  0.113
ATOM    331  H   ARG A  21      12.730 -24.523   5.800  1.00 28.28           H  0.090
ATOM    332  HA  ARG A  21      12.330 -26.273   6.958  1.00 29.31           H  0.092
ATOM    333  HB2 ARG A  21       9.789 -25.499   6.169  1.00 28.95           H  0.091
ATOM    334  HB3 ARG A  21       9.922 -26.917   6.874  1.00 28.95           H  0.091
ATOM    335  HG2 ARG A  21      10.991 -24.673   8.176  1.00 42.60           H  0.110
ATOM    336  HG3 ARG A  21       9.434 -24.964   8.304  1.00 42.60           H  0.110
ATOM    337  HD2 ARG A  21       9.946 -27.089   9.168  1.00 36.40           H  0.102
ATOM    338  HD3 ARG A  21      11.486 -26.700   9.134  1.00 36.40           H  0.102
ATOM    339  HE  ARG A  21      10.428 -24.895  10.581  1.00 65.69           H  0.137
ATOM    340 HH11 ARG A  21      10.274 -28.194  10.990  1.00 53.34           H  0.124
ATOM    341 HH12 ARG A  21      10.245 -28.153  12.479  1.00 53.34           H  0.124
```

ATOM	342	HH21	ARG	A	21	10.452	-24.884	12.815	1.00	53.40	H	0.124
ATOM	343	HH22	ARG	A	21	10.352	-26.160	13.578	1.00	53.40	H	0.124
ATOM	344	N	GLY	A	22	12.746	-27.430	4.384	1.00	18.08	N	0.072
ATOM	345	CA	GLY	A	22	13.019	-28.607	3.593	1.00	21.35	C	0.078
ATOM	346	C	GLY	A	22	12.100	-28.852	2.400	1.00	20.40	C	0.076
ATOM	347	O	GLY	A	22	12.249	-29.875	1.745	1.00	18.87	O	0.074
ATOM	348	H	GLY	A	22	13.215	-26.741	4.173	1.00	21.69	H	0.079
ATOM	349	HA2	GLY	A	22	13.925	-28.540	3.251	1.00	25.62	H	0.086
ATOM	350	HA3	GLY	A	22	12.956	-29.383	4.171	1.00	25.62	H	0.086
ATOM	351	N	TYR	A	23	11.166	-27.961	2.104	1.00	18.98	N	0.074
ATOM	352	CA	TYR	A	23	10.234	-28.176	0.998	1.00	19.06	C	0.074
ATOM	353	C	TYR	A	23	10.775	-27.486	-0.238	1.00	19.68	C	0.075
ATOM	354	O	TYR	A	23	10.990	-26.259	-0.238	1.00	19.03	O	0.074
ATOM	355	CB	TYR	A	23	8.856	-27.657	1.358	1.00	20.89	C	0.077
ATOM	356	CG	TYR	A	23	8.221	-28.507	2.425	1.00	15.10	C	0.066
ATOM	357	CD1	TYR	A	23	7.473	-29.623	2.060	1.00	14.02	C	0.063
ATOM	358	CD2	TYR	A	23	8.373	-28.234	3.804	1.00	15.38	C	0.066
ATOM	359	CE1	TYR	A	23	6.869	-30.434	2.996	1.00	17.37	C	0.071
ATOM	360	CE2	TYR	A	23	7.748	-29.060	4.769	1.00	16.12	C	0.068
ATOM	361	CZ	TYR	A	23	7.001	-30.167	4.341	1.00	14.63	C	0.065
ATOM	362	OH	TYR	A	23	6.353	-31.063	5.205	1.00	17.61	O	0.071
ATOM	363	H	TYR	A	23	11.049	-27.221	2.528	1.00	22.78	H	0.081
ATOM	364	HA	TYR	A	23	10.155	-29.124	0.808	1.00	22.87	H	0.081
ATOM	365	HB2	TYR	A	23	8.930	-26.749	1.691	1.00	25.07	H	0.085
ATOM	366	HB3	TYR	A	23	8.289	-27.676	0.571	1.00	25.07	H	0.085
ATOM	367	HD1	TYR	A	23	7.378	-29.827	1.158	1.00	16.82	H	0.069
ATOM	368	HD2	TYR	A	23	8.886	-27.509	4.079	1.00	18.46	H	0.073
ATOM	369	HE1	TYR	A	23	6.368	-31.166	2.718	1.00	20.84	H	0.077
ATOM	370	HE2	TYR	A	23	7.832	-28.871	5.675	1.00	19.34	H	0.074
ATOM	371	HH	TYR	A	23	6.570	-30.903	6.000	1.00	21.13	H	0.078
ATOM	372	N	SER	A	24	11.017	-28.286	-1.275	1.00	20.76	N	0.077
ATOM	373	CA	SER	A	24	11.578	-27.726	-2.481	1.00	18.06	C	0.072
ATOM	374	C	SER	A	24	10.569	-26.776	-3.098	1.00	20.94	C	0.077
ATOM	375	O	SER	A	24	9.346	-26.884	-2.896	1.00	19.04	O	0.074
ATOM	376	CB	SER	A	24	11.955	-28.827	-3.438	1.00	20.72	C	0.077
ATOM	377	OG	SER	A	24	10.793	-29.503	-3.847	1.00	28.35	O	0.090
ATOM	378	H	SER	A	24	10.867	-29.132	-1.297	1.00	24.91	H	0.084
ATOM	379	HA	SER	A	24	12.389	-27.228	-2.292	1.00	21.67	H	0.079
ATOM	380	HB2	SER	A	24	12.393	-28.443	-4.213	1.00	24.87	H	0.084
ATOM	381	HB3	SER	A	24	12.551	-29.450	-2.994	1.00	24.87	H	0.084
ATOM	382	HG	SER	A	24	10.999	-30.173	-4.310	1.00	34.02	H	0.099
ATOM	383	N	LEU	A	25	11.097	-25.825	-3.872	1.00	20.85	N	0.077
ATOM	384	CA	LEU	A	25	10.243	-24.839	-4.513	1.00	17.22	C	0.070
ATOM	385	C	LEU	A	25	9.048	-25.455	-5.232	1.00	23.16	C	0.081
ATOM	386	O	LEU	A	25	7.945	-24.876	-5.256	1.00	17.52	O	0.071
ATOM	387	CB	LEU	A	25	11.078	-24.061	-5.513	1.00	23.21	C	0.082
ATOM	388	CG	LEU	A	25	10.558	-22.754	-5.994	1.00	25.63	C	0.086
ATOM	389	CD1	LEU	A	25	10.221	-21.782	-4.856	1.00	26.24	C	0.087
ATOM	390	CD2	LEU	A	25	11.637	-22.126	-6.940	1.00	28.02	C	0.090
ATOM	391	H	LEU	A	25	11.936	-25.735	-4.037	1.00	25.01	H	0.085
ATOM	392	HA	LEU	A	25	9.890	-24.252	-3.827	1.00	20.66	H	0.077
ATOM	393	HB2	LEU	A	25	11.938	-23.884	-5.101	1.00	27.85	H	0.089
ATOM	394	HB3	LEU	A	25	11.194	-24.620	-6.297	1.00	27.85	H	0.089
ATOM	395	HG	LEU	A	25	9.723	-22.902	-6.465	1.00	30.76	H	0.094
ATOM	396	HD11	LEU	A	25	10.151	-20.885	-5.218	1.00	31.49	H	0.095
ATOM	397	HD12	LEU	A	25	9.378	-22.044	-4.456	1.00	31.49	H	0.095
ATOM	398	HD13	LEU	A	25	10.928	-21.817	-4.192	1.00	31.49	H	0.095
ATOM	399	HD21	LEU	A	25	11.261	-21.344	-7.372	1.00	33.63	H	0.098
ATOM	400	HD22	LEU	A	25	12.411	-21.873	-6.412	1.00	33.63	H	0.098
ATOM	401	HD23	LEU	A	25	11.892	-22.783	-7.607	1.00	33.63	H	0.098
ATOM	402	N	GLY	A	26	9.252	-26.586	-5.876	1.00	18.92	N	0.074
ATOM	403	CA	GLY	A	26	8.154	-27.217	-6.594	1.00	17.27	C	0.070
ATOM	404	C	GLY	A	26	6.962	-27.520	-5.715	1.00	18.90	C	0.074
ATOM	405	O	GLY	A	26	5.809	-27.399	-6.158	1.00	16.48	O	0.069
ATOM	406	H	GLY	A	26	10.001	-27.007	-5.916	1.00	22.70	H	0.081
ATOM	407	HA2	GLY	A	26	7.861	-26.628	-7.307	1.00	20.72	H	0.077
ATOM	408	HA3	GLY	A	26	8.464	-28.051	-6.980	1.00	20.72	H	0.077
ATOM	409	N	ASN	A	27	7.211	-27.844	-4.445	1.00	17.25	N	0.070
ATOM	410	CA	ASN	A	27	6.122	-28.038	-3.499	1.00	16.95	C	0.070
ATOM	411	C	ASN	A	27	5.291	-26.775	-3.291	1.00	14.40	C	0.064
ATOM	412	O	ASN	A	27	4.071	-26.839	-3.208	1.00	15.18	O	0.066
ATOM	413	CB	ASN	A	27	6.714	-28.493	-2.159	1.00	19.31	C	0.074
ATOM	414	CG	ASN	A	27	7.071	-29.969	-2.183	1.00	15.19	C	0.066
ATOM	415	OD1	ASN	A	27	6.203	-30.794	-2.109	1.00	15.27	O	0.066
ATOM	416	ND2	ASN	A	27	8.369	-30.306	-2.275	1.00	18.51	N	0.073
ATOM	417	H	ASN	A	27	7.995	-27.956	-4.112	1.00	20.70	H	0.077
ATOM	418	HA	ASN	A	27	5.527	-28.719	-3.850	1.00	20.34	H	0.076
ATOM	419	HB2	ASN	A	27	7.521	-27.986	-1.975	1.00	23.17	H	0.081

```
ATOM    420  HB3 ASN A  27       6.064 -28.347  -1.454  1.00 23.17           H  0.081
ATOM    421 HD21 ASN A  27       8.599 -31.135  -2.291  1.00 22.22           H  0.080
ATOM    422 HD22 ASN A  27       8.971 -29.692  -2.318  1.00 22.22           H  0.080
ATOM    423  N   TRP A  28       5.972 -25.638  -3.139  1.00 14.88           N  0.065
ATOM    424  CA  TRP A  28       5.321 -24.346  -2.932  1.00 17.30           C  0.070
ATOM    425  C   TRP A  28       4.546 -23.934  -4.148  1.00 15.11           C  0.066
ATOM    426  O   TRP A  28       3.414 -23.456  -4.040  1.00 14.82           O  0.065
ATOM    427  CB  TRP A  28       6.372 -23.331  -2.576  1.00 16.39           C  0.069
ATOM    428  CG  TRP A  28       7.033 -23.671  -1.266  1.00 13.31           C  0.062
ATOM    429  CD1 TRP A  28       8.266 -24.199  -1.081  1.00 16.86           C  0.070
ATOM    430  CD2 TRP A  28       6.438 -23.571   0.029  1.00 20.53           C  0.077
ATOM    431  NE1 TRP A  28       8.510 -24.363   0.289  1.00 17.12           N  0.070
ATOM    432  CE2 TRP A  28       7.386 -24.008   0.972  1.00 16.89           C  0.070
ATOM    433  CE3 TRP A  28       5.184 -23.128   0.493  1.00 18.52           C  0.073
ATOM    434  CZ2 TRP A  28       7.109 -24.003   2.371  1.00 13.92           C  0.063
ATOM    435  CZ3 TRP A  28       4.937 -23.123   1.816  1.00 16.09           C  0.068
ATOM    436  CH2 TRP A  28       5.901 -23.567   2.751  1.00 18.67           C  0.073
ATOM    437  H   TRP A  28       6.831 -25.585  -3.153  1.00 17.86           H  0.072
ATOM    438  HA  TRP A  28       4.690 -24.401  -2.198  1.00 20.76           H  0.077
ATOM    439  HB2 TRP A  28       7.052 -23.314  -3.268  1.00 19.67           H  0.075
ATOM    440  HB3 TRP A  28       5.961 -22.456  -2.495  1.00 19.67           H  0.075
ATOM    441  HD1 TRP A  28       8.861 -24.419  -1.761  1.00 20.23           H  0.076
ATOM    442  HE1 TRP A  28       9.245 -24.641   0.639  1.00 20.54           H  0.077
ATOM    443  HE3 TRP A  28       4.535 -22.842  -0.108  1.00 22.23           H  0.080
ATOM    444  HZ2 TRP A  28       7.739 -24.289   2.992  1.00 16.70           H  0.069
ATOM    445  HZ3 TRP A  28       4.112 -22.821   2.122  1.00 19.31           H  0.074
ATOM    446  HH2 TRP A  28       5.689 -23.556   3.656  1.00 22.41           H  0.080
ATOM    447  N   VAL A  29       5.106 -24.156  -5.327  1.00 17.82           N  0.071
ATOM    448  CA  VAL A  29       4.392 -23.759  -6.555  1.00 14.13           C  0.064
ATOM    449  C   VAL A  29       3.160 -24.646  -6.789  1.00 14.04           C  0.063
ATOM    450  O   VAL A  29       2.090 -24.154  -7.157  1.00 18.85           O  0.073
ATOM    451  CB  VAL A  29       5.356 -23.777  -7.771  1.00 16.23           C  0.068
ATOM    452  CG1 VAL A  29       4.616 -23.538  -9.016  1.00 18.34           C  0.072
ATOM    453  CG2 VAL A  29       6.469 -22.767  -7.531  1.00 21.96           C  0.079
ATOM    454  H   VAL A  29       5.874 -24.523  -5.451  1.00 21.38           H  0.078
ATOM    455  HA  VAL A  29       4.073 -22.851  -6.440  1.00 16.95           H  0.070
ATOM    456  HB  VAL A  29       5.771 -24.648  -7.873  1.00 19.47           H  0.075
ATOM    457 HG11 VAL A  29       5.248 -23.391  -9.736  1.00 22.00           H  0.079
ATOM    458 HG12 VAL A  29       4.067 -24.313  -9.211  1.00 22.00           H  0.079
ATOM    459 HG13 VAL A  29       4.055 -22.754  -8.904  1.00 22.00           H  0.079
ATOM    460 HG21 VAL A  29       7.073 -22.775  -8.290  1.00 26.35           H  0.087
ATOM    461 HG22 VAL A  29       6.079 -21.885  -7.428  1.00 26.35           H  0.087
ATOM    462 HG23 VAL A  29       6.950 -23.013  -6.724  1.00 26.35           H  0.087
ATOM    463  N   CYS A  30       3.309 -25.960  -6.648  1.00 15.44           N  0.067
ATOM    464  CA  CYS A  30       2.190 -26.896  -6.695  1.00 16.92           C  0.070
ATOM    465  C   CYS A  30       1.071 -26.502  -5.713  1.00 13.77           C  0.063
ATOM    466  O   CYS A  30      -0.104 -26.423  -6.073  1.00 14.69           O  0.065
ATOM    467  CB  CYS A  30       2.702 -28.312  -6.365  1.00 14.13           C  0.064
ATOM    468  SG  CYS A  30       1.432 -29.548  -6.522  1.00 16.96           S  0.070
ATOM    469  H   CYS A  30       4.067 -26.346  -6.521  1.00 18.53           H  0.073
ATOM    470  HA  CYS A  30       1.817 -26.889  -7.591  1.00 20.30           H  0.076
ATOM    471  HB2 CYS A  30       3.422 -28.540  -6.974  1.00 16.96           H  0.070
ATOM    472  HB3 CYS A  30       3.026 -28.326  -5.450  1.00 16.96           H  0.070
ATOM    473  N   ALA A  31       1.441 -26.207  -4.472  1.00 15.42           N  0.066
ATOM    474  CA  ALA A  31       0.454 -25.848  -3.456  1.00 14.24           C  0.064
ATOM    475  C   ALA A  31      -0.275 -24.579  -3.863  1.00 14.49           C  0.064
ATOM    476  O   ALA A  31      -1.501 -24.518  -3.853  1.00 16.75           O  0.069
ATOM    477  CB  ALA A  31       1.116 -25.644  -2.120  1.00 14.41           C  0.064
ATOM    478  H   ALA A  31       2.253 -26.206  -4.191  1.00 18.50           H  0.073
ATOM    479  HA  ALA A  31      -0.181 -26.577  -3.368  1.00 17.09           H  0.070
ATOM    480  HB1 ALA A  31       0.438 -25.432  -1.460  1.00 17.30           H  0.070
ATOM    481  HB2 ALA A  31       1.579 -26.459  -1.870  1.00 17.30           H  0.070
ATOM    482  HB3 ALA A  31       1.749 -24.913  -2.190  1.00 17.30           H  0.070
ATOM    483  N   ALA A  32       0.483 -23.572  -4.299  1.00 15.50           N  0.067
ATOM    484  CA  ALA A  32      -0.130 -22.363  -4.834  1.00 17.61           C  0.071
ATOM    485  C   ALA A  32      -1.029 -22.664  -6.039  1.00 14.51           C  0.064
ATOM    486  O   ALA A  32      -2.144 -22.108  -6.147  1.00 15.08           O  0.066
ATOM    487  CB  ALA A  32       0.979 -21.366  -5.178  1.00 17.69           C  0.071
ATOM    488  H   ALA A  32       1.343 -23.566  -4.296  1.00 18.60           H  0.073
ATOM    489  HA  ALA A  32      -0.706 -21.960  -4.166  1.00 21.13           H  0.078
ATOM    490  HB1 ALA A  32       0.580 -20.567  -5.555  1.00 21.23           H  0.078
ATOM    491  HB2 ALA A  32       1.465 -21.143  -4.369  1.00 21.23           H  0.078
ATOM    492  HB3 ALA A  32       1.579 -21.772  -5.823  1.00 21.23           H  0.078
ATOM    493  N   LYS A  33      -0.610 -23.567  -6.927  1.00 15.92           N  0.068
ATOM    494  CA  LYS A  33      -1.445 -23.871  -8.080  1.00 15.64           C  0.067
ATOM    495  C   LYS A  33      -2.817 -24.355  -7.623  1.00 19.61           C  0.075
ATOM    496  O   LYS A  33      -3.874 -23.857  -8.063  1.00 19.02           O  0.074
ATOM    497  CB  LYS A  33      -0.787 -24.929  -8.976  1.00 17.67           C  0.071
```

ATOM	498	CG	LYS	A	33	-1.728	-25.496	-10.012	1.00	17.80	C	0.071
ATOM	499	CD	LYS	A	33	-2.168	-24.473	-10.958	1.00	16.28	C	0.068
ATOM	500	CE	LYS	A	33	-2.983	-25.143	-12.125	1.00	23.41	C	0.082
ATOM	501	NZ	LYS	A	33	-3.229	-24.077	-13.135	1.00	27.24	N	0.088
ATOM	502	H	LYS	A	33	0.130	-24.003	-6.883	1.00	19.10	H	0.074
ATOM	503	HA	LYS	A	33	-1.544	-23.064	-8.608	1.00	18.77	H	0.073
ATOM	504	HB2	LYS	A	33	-0.038	-24.525	-9.442	1.00	21.20	H	0.078
ATOM	505	HB3	LYS	A	33	-0.476	-25.662	-8.422	1.00	21.20	H	0.078
ATOM	506	HG2	LYS	A	33	-1.274	-26.195	-10.509	1.00	21.36	H	0.078
ATOM	507	HG3	LYS	A	33	-2.511	-25.858	-9.569	1.00	21.36	H	0.078
ATOM	508	HD2	LYS	A	33	-2.739	-23.832	-10.505	1.00	19.53	H	0.075
ATOM	509	HD3	LYS	A	33	-1.397	-24.021	-11.335	1.00	19.53	H	0.075
ATOM	510	HE2	LYS	A	33	-2.471	-25.861	-12.528	1.00	28.09	H	0.090
ATOM	511	HE3	LYS	A	33	-3.830	-25.482	-11.797	1.00	28.09	H	0.090
ATOM	512	HZ1	LYS	A	33	-3.667	-24.414	-13.833	1.00	32.69	H	0.097
ATOM	513	HZ2	LYS	A	33	-3.717	-23.424	-12.777	1.00	32.69	H	0.097
ATOM	514	HZ3	LYS	A	33	-2.455	-23.739	-13.416	1.00	32.69	H	0.097
ATOM	515	N	PHE	A	34	-2.824	-25.264	-6.662	1.00	19.03	N	0.074
ATOM	516	CA	PHE	A	34	-4.102	-25.871	-6.329	1.00	19.96	C	0.076
ATOM	517	C	PHE	A	34	-4.852	-25.129	-5.264	1.00	18.26	C	0.072
ATOM	518	O	PHE	A	34	-6.036	-25.390	-5.053	1.00	21.69	O	0.079
ATOM	519	CB	PHE	A	34	-3.866	-27.300	-5.928	1.00	15.92	C	0.068
ATOM	520	CG	PHE	A	34	-3.444	-28.132	-7.082	1.00	16.93	C	0.070
ATOM	521	CD1	PHE	A	34	-4.200	-28.110	-8.267	1.00	17.52	C	0.071
ATOM	522	CD2	PHE	A	34	-2.294	-28.883	-7.050	1.00	17.94	C	0.072
ATOM	523	CE1	PHE	A	34	-3.813	-28.830	-9.315	1.00	14.44	C	0.064
ATOM	524	CE2	PHE	A	34	-1.909	-29.646	-8.186	1.00	20.29	C	0.076
ATOM	525	CZ	PHE	A	34	-2.676	-29.591	-9.312	1.00	20.36	C	0.076
ATOM	526	H	PHE	A	34	-2.141	-25.531	-6.212	1.00	22.84	H	0.081
ATOM	527	HA	PHE	A	34	-4.683	-25.874	-7.106	1.00	23.95	H	0.083
ATOM	528	HB2	PHE	A	34	-3.167	-27.333	-5.257	1.00	19.11	H	0.074
ATOM	529	HB3	PHE	A	34	-4.688	-27.672	-5.570	1.00	19.11	H	0.074
ATOM	530	HD1	PHE	A	34	-4.970	-27.591	-8.318	1.00	21.03	H	0.078
ATOM	531	HD2	PHE	A	34	-1.767	-28.893	-6.284	1.00	21.53	H	0.079
ATOM	532	HE1	PHE	A	34	-4.338	-28.816	-10.082	1.00	17.32	H	0.070
ATOM	533	HE2	PHE	A	34	-1.145	-30.176	-8.161	1.00	24.35	H	0.084
ATOM	534	HZ	PHE	A	34	-2.428	-30.066	-10.072	1.00	24.43	H	0.084
ATOM	535	N	GLU	A	35	-4.171	-24.305	-4.525	1.00	15.45	N	0.067
ATOM	536	CA	GLU	A	35	-4.892	-23.509	-3.547	1.00	14.43	C	0.064
ATOM	537	C	GLU	A	35	-5.569	-22.325	-4.195	1.00	18.52	C	0.073
ATOM	538	O	GLU	A	35	-6.701	-22.012	-3.873	1.00	14.75	O	0.065
ATOM	539	CB	GLU	A	35	-3.951	-23.015	-2.484	1.00	11.67	C	0.058
ATOM	540	CG	GLU	A	35	-3.439	-24.069	-1.530	1.00	18.83	C	0.073
ATOM	541	CD	GLU	A	35	-4.531	-24.652	-0.635	1.00	21.54	C	0.079
ATOM	542	OE1	GLU	A	35	-5.646	-24.046	-0.597	1.00	17.73	O	0.071
ATOM	543	OE2	GLU	A	35	-4.235	-25.660	0.008	1.00	20.61	O	0.077
ATOM	544	H	GLU	A	35	-3.320	-24.182	-4.560	1.00	18.54	H	0.073
ATOM	545	HA	GLU	A	35	-5.566	-24.073	-3.135	1.00	17.32	H	0.070
ATOM	546	HB2	GLU	A	35	-3.179	-22.620	-2.920	1.00	14.00	H	0.063
ATOM	547	HB3	GLU	A	35	-4.412	-22.346	-1.955	1.00	14.00	H	0.063
ATOM	548	HG2	GLU	A	35	-3.053	-24.796	-2.042	1.00	22.59	H	0.080
ATOM	549	HG3	GLU	A	35	-2.764	-23.672	-0.957	1.00	22.59	H	0.080
ATOM	550	N	SER	A	36	-4.860	-21.624	-5.090	1.00	17.65	N	0.071
ATOM	551	CA	SER	A	36	-5.343	-20.350	-5.575	1.00	16.08	C	0.068
ATOM	552	C	SER	A	36	-5.307	-20.215	-7.083	1.00	18.57	C	0.073
ATOM	553	O	SER	A	36	-5.774	-19.183	-7.602	1.00	21.61	O	0.079
ATOM	554	CB	SER	A	36	-4.512	-19.211	-5.034	1.00	18.29	C	0.072
ATOM	555	OG	SER	A	36	-3.303	-19.193	-5.753	1.00	16.62	O	0.069
ATOM	556	H	SER	A	36	-4.106	-21.872	-5.421	1.00	21.19	H	0.078
ATOM	557	HA	SER	A	36	-6.257	-20.264	-5.261	1.00	19.30	H	0.074
ATOM	558	HB2	SER	A	36	-4.982	-18.371	-5.159	1.00	21.95	H	0.079
ATOM	559	HB3	SER	A	36	-4.332	-19.352	-4.091	1.00	21.95	H	0.079
ATOM	560	HG	SER	A	36	-2.955	-19.958	-5.748	1.00	19.95	H	0.076
ATOM	561	N	ASN	A	37	-4.803	-21.227	-7.793	1.00	17.36	N	0.071
ATOM	562	CA	ASN	A	37	-4.575	-21.119	-9.222	1.00	17.36	C	0.071
ATOM	563	C	ASN	A	37	-3.727	-19.893	-9.553	1.00	20.20	C	0.076
ATOM	564	O	ASN	A	37	-3.906	-19.254	-10.585	1.00	19.62	O	0.075
ATOM	565	CB	ASN	A	37	-5.883	-21.098	-9.988	1.00	20.06	C	0.076
ATOM	566	CG	ASN	A	37	-5.662	-21.411	-11.429	1.00	20.85	C	0.077
ATOM	567	OD1	ASN	A	37	-4.854	-22.281	-11.719	1.00	21.59	O	0.079
ATOM	568	ND2	ASN	A	37	-6.390	-20.759	-12.336	1.00	31.23	N	0.095
ATOM	569	H	ASN	A	37	-4.586	-21.990	-7.460	1.00	20.83	H	0.077
ATOM	570	HA	ASN	A	37	-4.079	-21.902	-9.508	1.00	20.83	H	0.077
ATOM	571	HB2	ASN	A	37	-6.484	-21.762	-9.617	1.00	24.07	H	0.083
ATOM	572	HB3	ASN	A	37	-6.281	-20.216	-9.922	1.00	24.07	H	0.083
ATOM	573	HD21	ASN	A	37	-6.281	-20.923	-13.173	1.00	37.48	H	0.104
ATOM	574	HD22	ASN	A	37	-6.969	-20.176	-12.083	1.00	37.48	H	0.104
ATOM	575	N	PHE	A	38	-2.777	-19.589	-8.663	1.00	18.66	N	0.073

```
ATOM    576  CA  PHE A  38      -1.748 -18.560  -8.780  1.00 21.17           C  0.078
ATOM    577  C   PHE A  38      -2.328 -17.139  -8.728  1.00 20.14           C  0.076
ATOM    578  O   PHE A  38      -1.635 -16.169  -9.044  1.00 20.92           O  0.077
ATOM    579  CB  PHE A  38      -0.914 -18.714 -10.056  1.00 15.93           C  0.068
ATOM    580  CG  PHE A  38      -0.230 -20.034 -10.212  1.00 17.29           C  0.070
ATOM    581  CD1 PHE A  38       0.363 -20.662  -9.155  1.00 18.08           C  0.072
ATOM    582  CD2 PHE A  38      -0.172 -20.634 -11.450  1.00 20.10           C  0.076
ATOM    583  CE1 PHE A  38       1.034 -21.856  -9.316  1.00 20.13           C  0.076
ATOM    584  CE2 PHE A  38       0.480 -21.858 -11.614  1.00 22.10           C  0.080
ATOM    585  CZ  PHE A  38       1.093 -22.449 -10.559  1.00 18.79           C  0.073
ATOM    586  H   PHE A  38      -2.705 -20.007  -7.915  1.00 22.40           H  0.080
ATOM    587  HA  PHE A  38      -1.153 -18.670  -8.022  1.00 25.41           H  0.085
ATOM    588  HB2 PHE A  38      -1.500 -18.598 -10.821  1.00 19.12           H  0.074
ATOM    589  HB3 PHE A  38      -0.227 -18.029 -10.058  1.00 19.12           H  0.074
ATOM    590  HD1 PHE A  38       0.314 -20.277  -8.310  1.00 21.70           H  0.079
ATOM    591  HD2 PHE A  38      -0.570 -20.222 -12.182  1.00 24.12           H  0.083
ATOM    592  HE1 PHE A  38       1.447 -22.261  -8.587  1.00 24.15           H  0.083
ATOM    593  HE2 PHE A  38       0.493 -22.269 -12.448  1.00 26.53           H  0.087
ATOM    594  HZ  PHE A  38       1.552 -23.250 -10.673  1.00 22.54           H  0.080
ATOM    595  N   ASN A  39      -3.549 -17.007  -8.245  1.00 17.04           N  0.070
ATOM    596  CA  ASN A  39      -4.257 -15.726  -8.116  1.00 15.93           C  0.068
ATOM    597  C   ASN A  39      -4.100 -15.208  -6.698  1.00 19.12           C  0.074
ATOM    598  O   ASN A  39      -4.682 -15.773  -5.772  1.00 16.11           O  0.068
ATOM    599  CB  ASN A  39      -5.721 -15.947  -8.485  1.00 18.52           C  0.073
ATOM    600  CG  ASN A  39      -6.503 -14.669  -8.454  1.00 12.05           C  0.059
ATOM    601  OD1 ASN A  39      -5.949 -13.615  -8.160  1.00 15.94           O  0.068
ATOM    602  ND2 ASN A  39      -7.737 -14.736  -8.850  1.00 18.80           N  0.073
ATOM    603  H   ASN A  39      -4.020 -17.672  -7.969  1.00 20.44           H  0.077
ATOM    604  HA  ASN A  39      -3.869 -15.057  -8.700  1.00 19.11           H  0.074
ATOM    605  HB2 ASN A  39      -5.773 -16.313  -9.382  1.00 22.22           H  0.080
ATOM    606  HB3 ASN A  39      -6.121 -16.563  -7.851  1.00 22.22           H  0.080
ATOM    607 HD21 ASN A  39      -8.227 -14.029  -8.851  1.00 22.56           H  0.080
ATOM    608 HD22 ASN A  39      -8.064 -15.487  -9.113  1.00 22.56           H  0.080
ATOM    609  N   THR A  40      -3.351 -14.112  -6.537  1.00 16.31           N  0.068
ATOM    610  CA  THR A  40      -3.140 -13.545  -5.208  1.00 18.30           C  0.072
ATOM    611  C   THR A  40      -4.439 -13.103  -4.552  1.00 14.94           C  0.065
ATOM    612  O   THR A  40      -4.510 -13.010  -3.329  1.00 18.39           O  0.073
ATOM    613  CB  THR A  40      -2.236 -12.316  -5.227  1.00 22.64           C  0.081
ATOM    614  OG1 THR A  40      -2.876 -11.299  -6.010  1.00 17.58           O  0.071
ATOM    615  CG2 THR A  40      -0.853 -12.623  -5.730  1.00 21.01           C  0.078
ATOM    616  H   THR A  40      -2.961 -13.685  -7.174  1.00 19.58           H  0.075
ATOM    617  HA  THR A  40      -2.712 -14.252  -4.701  1.00 21.96           H  0.079
ATOM    618  HB  THR A  40      -2.101 -11.991  -4.323  1.00 27.17           H  0.088
ATOM    619  HG1 THR A  40      -3.449 -10.897  -5.545  1.00 21.09           H  0.078
ATOM    620 HG21 THR A  40      -0.307 -11.822  -5.711  1.00 25.21           H  0.085
ATOM    621 HG22 THR A  40      -0.439 -13.300  -5.172  1.00 25.21           H  0.085
ATOM    622 HG23 THR A  40      -0.897 -12.952  -6.642  1.00 25.21           H  0.085
ATOM    623  N   GLN A  41      -5.444 -12.739  -5.347  1.00 15.23           N  0.066
ATOM    624  CA  GLN A  41      -6.720 -12.241  -4.823  1.00 19.02           C  0.074
ATOM    625  C   GLN A  41      -7.717 -13.326  -4.445  1.00 18.16           C  0.072
ATOM    626  O   GLN A  41      -8.822 -12.984  -4.031  1.00 18.91           O  0.074
ATOM    627  CB  GLN A  41      -7.407 -11.307  -5.856  1.00 15.84           C  0.067
ATOM    628  CG  GLN A  41      -6.513 -10.109  -6.124  1.00 21.88           C  0.079
ATOM    629  CD  GLN A  41      -7.221  -8.952  -6.751  1.00 22.92           C  0.081
ATOM    630  OE1 GLN A  41      -7.893  -8.188  -6.070  1.00 19.27           O  0.074
ATOM    631  NE2 GLN A  41      -7.133  -8.851  -8.087  1.00 22.17           N  0.080
ATOM    632  H   GLN A  41      -5.413 -12.771  -6.206  1.00 18.27           H  0.072
ATOM    633  HA  GLN A  41      -6.506 -11.737  -4.022  1.00 22.82           H  0.081
ATOM    634  HB2 GLN A  41      -7.550 -11.785  -6.687  1.00 19.01           H  0.074
ATOM    635  HB3 GLN A  41      -8.254 -10.994  -5.503  1.00 19.01           H  0.074
ATOM    636  HG2 GLN A  41      -6.139  -9.805  -5.282  1.00 26.25           H  0.087
ATOM    637  HG3 GLN A  41      -5.801 -10.380  -6.725  1.00 26.25           H  0.087
ATOM    638 HE21 GLN A  41      -6.687  -9.435  -8.534  1.00 26.61           H  0.087
ATOM    639 HE22 GLN A  41      -7.523  -8.203  -8.496  1.00 26.61           H  0.087
ATOM    640  N   ALA A  42      -7.361 -14.602  -4.529  1.00 17.14           N  0.070
ATOM    641  CA  ALA A  42      -8.334 -15.645  -4.222  1.00 16.14           C  0.068
ATOM    642  C   ALA A  42      -8.759 -15.597  -2.742  1.00 15.49           C  0.067
ATOM    643  O   ALA A  42      -7.931 -15.508  -1.833  1.00 14.59           O  0.065
ATOM    644  CB  ALA A  42      -7.732 -17.011  -4.518  1.00 17.69           C  0.071
ATOM    645  H   ALA A  42      -6.581 -14.885  -4.757  1.00 20.56           H  0.077
ATOM    646  HA  ALA A  42      -9.121 -15.508  -4.773  1.00 19.36           H  0.074
ATOM    647  HB1 ALA A  42      -8.357 -17.699  -4.238  1.00 21.23           H  0.078
ATOM    648  HB2 ALA A  42      -7.565 -17.084  -5.470  1.00 21.23           H  0.078
ATOM    649  HB3 ALA A  42      -6.899 -17.102  -4.028  1.00 21.23           H  0.078
ATOM    650  N   THR A  43     -10.049 -15.798  -2.500  1.00 15.08           N  0.066
ATOM    651  CA  THR A  43     -10.617 -15.933  -1.162  1.00 13.99           C  0.063
ATOM    652  C   THR A  43     -11.649 -17.054  -1.229  1.00 20.04           C  0.076
ATOM    653  O   THR A  43     -12.235 -17.334  -2.283  1.00 23.29           O  0.082
```

ATOM	654	CB	THR	A	43	-11.299	-14.633	-0.672	1.00	16.11	C	0.068
ATOM	655	OG1	THR	A	43	-12.382	-14.313	-1.574	1.00	19.96	O	0.076
ATOM	656	CG2	THR	A	43	-10.295	-13.488	-0.648	1.00	19.97	C	0.076
ATOM	657	H	THR	A	43	-10.642	-15.863	-3.120	1.00	18.10	H	0.072
ATOM	658	HA	THR	A	43	-9.920	-16.149	-0.523	1.00	16.79	H	0.069
ATOM	659	HB	THR	A	43	-11.643	-14.750	0.227	1.00	19.33	H	0.074
ATOM	660	HG1	THR	A	43	-12.099	-14.281	-2.364	1.00	23.95	H	0.083
ATOM	661	HG21	THR	A	43	-10.727	-12.675	-0.343	1.00	23.96	H	0.083
ATOM	662	HG22	THR	A	43	-9.563	-13.700	-0.048	1.00	23.96	H	0.083
ATOM	663	HG23	THR	A	43	-9.940	-13.340	-1.539	1.00	23.96	H	0.083
ATOM	664	N	ASN	A	44	-11.856	-17.714	-0.103	1.00	14.18	N	0.064
ATOM	665	CA	ASN	A	44	-12.805	-18.815	-0.083	1.00	15.55	C	0.067
ATOM	666	C	ASN	A	44	-13.226	-18.961	1.362	1.00	20.95	C	0.077
ATOM	667	O	ASN	A	44	-12.361	-19.114	2.236	1.00	17.56	O	0.071
ATOM	668	CB	ASN	A	44	-12.262	-20.106	-0.604	1.00	22.65	C	0.081
ATOM	669	CG	ASN	A	44	-12.074	-20.055	-2.106	1.00	35.09	C	0.100
ATOM	670	OD1	ASN	A	44	-13.057	-20.148	-2.883	1.00	28.56	O	0.090
ATOM	671	ND2	ASN	A	44	-10.812	-19.899	-2.535	1.00	28.00	N	0.090
ATOM	672	H	ASN	A	44	-11.469	-17.549	0.647	1.00	17.01	H	0.070
ATOM	673	HA	ASN	A	44	-13.554	-18.597	-0.660	1.00	18.66	H	0.073
ATOM	674	HB2	ASN	A	44	-11.401	-20.283	-0.193	1.00	27.18	H	0.088
ATOM	675	HB3	ASN	A	44	-12.882	-20.823	-0.397	1.00	27.18	H	0.088
ATOM	676	HD21	ASN	A	44	-10.645	-19.863	-3.378	1.00	33.60	H	0.098
ATOM	677	HD22	ASN	A	44	-10.169	-19.834	-1.967	1.00	33.60	H	0.098
ATOM	678	N	ARG	A	45	-14.517	-18.860	1.579	1.00	20.11	N	0.076
ATOM	679	CA	ARG	A	45	-15.101	-18.995	2.902	1.00	20.91	C	0.077
ATOM	680	C	ARG	A	45	-15.196	-20.453	3.250	1.00	18.93	C	0.074
ATOM	681	O	ARG	A	45	-15.650	-21.265	2.439	1.00	23.18	O	0.081
ATOM	682	CB	ARG	A	45	-16.502	-18.419	2.915	1.00	21.43	C	0.078
ATOM	683	CG	ARG	A	45	-17.061	-18.145	4.296	1.00	21.91	C	0.079
ATOM	684	CD	ARG	A	45	-16.737	-16.738	4.674	1.00	23.81	C	0.083
ATOM	685	NE	ARG	A	45	-17.118	-16.460	6.053	1.00	47.44	N	0.117
ATOM	686	CZ	ARG	A	45	-17.942	-15.486	6.415	1.00	34.81	C	0.100
ATOM	687	NH1	ARG	A	45	-18.624	-14.786	5.516	1.00	45.93	N	0.115
ATOM	688	NH2	ARG	A	45	-18.041	-15.177	7.694	1.00	24.99	N	0.085
ATOM	689	H	ARG	A	45	-15.098	-18.709	0.963	1.00	24.13	H	0.083
ATOM	690	HA	ARG	A	45	-14.555	-18.526	3.552	1.00	25.09	H	0.085
ATOM	691	HB2	ARG	A	45	-16.494	-17.578	2.432	1.00	25.72	H	0.086
ATOM	692	HB3	ARG	A	45	-17.098	-19.047	2.478	1.00	25.72	H	0.086
ATOM	693	HG2	ARG	A	45	-18.024	-18.258	4.292	1.00	26.29	H	0.087
ATOM	694	HG3	ARG	A	45	-16.659	-18.747	4.941	1.00	26.29	H	0.087
ATOM	695	HD2	ARG	A	45	-15.782	-16.592	4.585	1.00	28.57	H	0.090
ATOM	696	HD3	ARG	A	45	-17.221	-16.129	4.095	1.00	28.57	H	0.090
ATOM	697	HE	ARG	A	45	-16.787	-16.959	6.671	1.00	56.93	H	0.128
ATOM	698	HH11	ARG	A	45	-18.537	-14.960	4.678	1.00	55.12	H	0.126
ATOM	699	HH12	ARG	A	45	-19.153	-14.158	5.772	1.00	55.12	H	0.126
ATOM	700	HH21	ARG	A	45	-17.576	-15.605	8.278	1.00	29.99	H	0.093
ATOM	701	HH22	ARG	A	45	-18.571	-14.548	7.945	1.00	29.99	H	0.093
ATOM	702	N	ASN	A	46	-14.939	-20.757	4.508	1.00	20.73	N	0.077
ATOM	703	CA	ASN	A	46	-15.114	-22.101	5.002	1.00	20.86	C	0.077
ATOM	704	C	ASN	A	46	-16.388	-22.189	5.796	1.00	20.07	C	0.076
ATOM	705	O	ASN	A	46	-16.940	-21.188	6.240	1.00	25.65	O	0.086
ATOM	706	CB	ASN	A	46	-13.920	-22.494	5.838	1.00	24.66	C	0.084
ATOM	707	CG	ASN	A	46	-12.618	-22.221	5.129	1.00	23.15	C	0.081
ATOM	708	OD1	ASN	A	46	-11.776	-21.458	5.594	1.00	26.50	O	0.087
ATOM	709	ND2	ASN	A	46	-12.438	-22.856	4.001	1.00	25.32	N	0.085
ATOM	710	H	ASN	A	46	-14.660	-20.195	5.097	1.00	24.87	H	0.084
ATOM	711	HA	ASN	A	46	-15.184	-22.733	4.269	1.00	25.03	H	0.085
ATOM	712	HB2	ASN	A	46	-13.927	-21.984	6.663	1.00	29.59	H	0.092
ATOM	713	HB3	ASN	A	46	-13.965	-23.443	6.033	1.00	29.59	H	0.092
ATOM	714	HD21	ASN	A	46	-11.714	-22.740	3.552	1.00	30.38	H	0.093
ATOM	715	HD22	ASN	A	46	-13.044	-23.391	3.707	1.00	30.38	H	0.093
ATOM	716	N	THR	A	47	-16.891	-23.409	5.932	1.00	27.53	N	0.089
ATOM	717	CA	THR	A	47	-18.146	-23.564	6.658	1.00	38.62	C	0.105
ATOM	718	C	THR	A	47	-18.018	-23.102	8.109	1.00	32.21	C	0.096
ATOM	719	O	THR	A	47	-19.013	-22.653	8.683	1.00	34.75	O	0.100
ATOM	720	CB	THR	A	47	-18.650	-25.007	6.552	1.00	40.34	C	0.108
ATOM	721	OG1	THR	A	47	-17.564	-25.940	6.588	1.00	37.42	O	0.104
ATOM	722	CG2	THR	A	47	-19.428	-25.159	5.257	1.00	44.72	C	0.113
ATOM	723	H	THR	A	47	-16.541	-24.134	5.628	1.00	33.03	H	0.097
ATOM	724	HA	THR	A	47	-18.823	-23.007	6.245	1.00	46.35	H	0.115
ATOM	725	HB	THR	A	47	-19.228	-25.211	7.305	1.00	48.41	H	0.118
ATOM	726	HG1	THR	A	47	-17.852	-26.721	6.470	1.00	44.90	H	0.113
ATOM	727	HG21	THR	A	47	-19.743	-26.072	5.164	1.00	53.67	H	0.124
ATOM	728	HG22	THR	A	47	-20.192	-24.561	5.256	1.00	53.67	H	0.124
ATOM	729	HG23	THR	A	47	-18.860	-24.944	4.501	1.00	53.67	H	0.124
ATOM	730	N	ASP	A	48	-16.814	-23.155	8.702	1.00	29.92	N	0.093
ATOM	731	CA	ASP	A	48	-16.672	-22.675	10.088	1.00	29.56	C	0.092

ATOM	732	C	ASP	A	48	-16.728	-21.137	10.173	1.00	27.87	C	0.089
ATOM	733	O	ASP	A	48	-16.561	-20.537	11.258	1.00	27.42	O	0.089
ATOM	734	CB	ASP	A	48	-15.383	-23.219	10.728	1.00	31.90	C	0.096
ATOM	735	CG	ASP	A	48	-14.097	-22.546	10.215	1.00	29.56	C	0.092
ATOM	736	OD1	ASP	A	48	-14.128	-21.637	9.365	1.00	28.99	O	0.091
ATOM	737	OD2	ASP	A	48	-13.009	-22.935	10.654	1.00	30.76	O	0.094
ATOM	738	H	ASP	A	48	-16.094	-23.453	8.338	1.00	35.91	H	0.101
ATOM	739	HA	ASP	A	48	-17.402	-23.027	10.620	1.00	35.47	H	0.101
ATOM	740	HB2	ASP	A	48	-15.428	-23.078	11.687	1.00	38.28	H	0.105
ATOM	741	HB3	ASP	A	48	-15.316	-24.168	10.535	1.00	38.28	H	0.105
ATOM	742	N	GLY	A	49	-16.988	-20.495	9.034	1.00	25.87	N	0.086
ATOM	743	CA	GLY	A	49	-17.016	-19.054	8.956	1.00	27.82	C	0.089
ATOM	744	C	GLY	A	49	-15.668	-18.405	8.824	1.00	24.74	C	0.084
ATOM	745	O	GLY	A	49	-15.591	-17.176	8.743	1.00	22.32	O	0.080
ATOM	746	H	GLY	A	49	-17.152	-20.886	8.287	1.00	31.04	H	0.094
ATOM	747	HA2	GLY	A	49	-17.546	-18.795	8.186	1.00	33.38	H	0.098
ATOM	748	HA3	GLY	A	49	-17.434	-18.707	9.759	1.00	33.38	H	0.098
ATOM	749	N	SER	A	50	-14.589	-19.181	8.851	1.00	18.51	N	0.073
ATOM	750	CA	SER	A	50	-13.322	-18.543	8.601	1.00	17.01	C	0.070
ATOM	751	C	SER	A	50	-13.210	-18.351	7.069	1.00	14.86	C	0.065
ATOM	752	O	SER	A	50	-14.009	-18.888	6.299	1.00	15.45	O	0.067
ATOM	753	CB	SER	A	50	-12.193	-19.385	9.146	1.00	18.49	C	0.073
ATOM	754	OG	SER	A	50	-12.168	-20.615	8.447	1.00	20.31	O	0.076
ATOM	755	H	SER	A	50	-14.575	-20.027	9.003	1.00	22.21	H	0.080
ATOM	756	HA	SER	A	50	-13.252	-17.684	9.045	1.00	20.41	H	0.076
ATOM	757	HB2	SER	A	50	-11.351	-18.922	9.017	1.00	22.19	H	0.080
ATOM	758	HB3	SER	A	50	-12.339	-19.552	10.090	1.00	22.19	H	0.080
ATOM	759	HG	SER	A	50	-12.936	-20.953	8.432	1.00	24.37	H	0.084
ATOM	760	N	THR	A	51	-12.176	-17.626	6.641	1.00	16.92	N	0.070
ATOM	761	CA	THR	A	51	-11.931	-17.412	5.205	1.00	12.53	C	0.060
ATOM	762	C	THR	A	51	-10.463	-17.696	4.921	1.00	15.18	C	0.066
ATOM	763	O	THR	A	51	-9.579	-17.344	5.727	1.00	16.32	O	0.068
ATOM	764	CB	THR	A	51	-12.342	-15.991	4.835	1.00	14.45	C	0.064
ATOM	765	OG1	THR	A	51	-13.736	-15.822	5.110	1.00	15.11	O	0.066
ATOM	766	CG2	THR	A	51	-12.136	-15.695	3.324	1.00	15.14	C	0.066
ATOM	767	H	THR	A	51	-11.602	-17.248	7.158	1.00	20.30	H	0.076
ATOM	768	HA	THR	A	51	-12.439	-18.016	4.642	1.00	15.04	H	0.066
ATOM	769	HB	THR	A	51	-11.794	-15.377	5.348	1.00	17.34	H	0.070
ATOM	770	HG1	THR	A	51	-13.868	-15.828	5.939	1.00	18.13	H	0.072
ATOM	771	HG21	THR	A	51	-12.548	-14.848	3.093	1.00	18.16	H	0.072
ATOM	772	HG22	THR	A	51	-11.189	-15.651	3.120	1.00	18.16	H	0.072
ATOM	773	HG23	THR	A	51	-12.541	-16.397	2.791	1.00	18.16	H	0.072
ATOM	774	N	ASP	A	52	-10.211	-18.321	3.770	1.00	13.64	N	0.063
ATOM	775	CA	ASP	A	52	-8.875	-18.532	3.259	1.00	11.06	C	0.056
ATOM	776	C	ASP	A	52	-8.525	-17.433	2.264	1.00	14.80	C	0.065
ATOM	777	O	ASP	A	52	-9.333	-17.099	1.394	1.00	14.75	O	0.065
ATOM	778	CB	ASP	A	52	-8.743	-19.905	2.606	1.00	16.64	C	0.069
ATOM	779	CG	ASP	A	52	-9.018	-21.013	3.568	1.00	20.34	C	0.076
ATOM	780	OD1	ASP	A	52	-8.669	-20.809	4.751	1.00	18.62	O	0.073
ATOM	781	OD2	ASP	A	52	-9.507	-22.063	3.138	1.00	28.36	O	0.090
ATOM	782	H	ASP	A	52	-10.823	-18.639	3.257	1.00	16.37	H	0.068
ATOM	783	HA	ASP	A	52	-8.238	-18.494	3.990	1.00	13.27	H	0.062
ATOM	784	HB2	ASP	A	52	-9.379	-19.973	1.877	1.00	19.97	H	0.076
ATOM	785	HB3	ASP	A	52	-7.840	-20.013	2.269	1.00	19.97	H	0.076
ATOM	786	N	TYR	A	53	-7.294	-16.926	2.344	1.00	15.50	N	0.067
ATOM	787	CA	TYR	A	53	-6.856	-15.799	1.534	1.00	16.74	C	0.069
ATOM	788	C	TYR	A	53	-5.544	-16.003	0.779	1.00	14.08	C	0.064
ATOM	789	O	TYR	A	53	-4.548	-16.412	1.347	1.00	11.57	O	0.058
ATOM	790	CB	TYR	A	53	-6.661	-14.564	2.402	1.00	17.31	C	0.070
ATOM	791	CG	TYR	A	53	-7.878	-14.078	3.144	1.00	16.55	C	0.069
ATOM	792	CD1	TYR	A	53	-8.316	-14.689	4.330	1.00	16.58	C	0.069
ATOM	793	CD2	TYR	A	53	-8.590	-13.000	2.663	1.00	18.83	C	0.073
ATOM	794	CE1	TYR	A	53	-9.402	-14.209	5.015	1.00	16.38	C	0.069
ATOM	795	CE2	TYR	A	53	-9.727	-12.562	3.298	1.00	15.73	C	0.067
ATOM	796	CZ	TYR	A	53	-10.101	-13.133	4.506	1.00	13.21	C	0.062
ATOM	797	OH	TYR	A	53	-11.212	-12.652	5.129	1.00	16.77	O	0.069
ATOM	798	H	TYR	A	53	-6.685	-17.228	2.872	1.00	18.60	H	0.073
ATOM	799	HA	TYR	A	53	-7.546	-15.693	0.860	1.00	20.08	H	0.076
ATOM	800	HB2	TYR	A	53	-5.983	-14.764	3.065	1.00	20.77	H	0.077
ATOM	801	HB3	TYR	A	53	-6.364	-13.837	1.832	1.00	20.77	H	0.077
ATOM	802	HD1	TYR	A	53	-7.862	-15.433	4.656	1.00	19.89	H	0.075
ATOM	803	HD2	TYR	A	53	-8.297	-12.563	1.896	1.00	22.60	H	0.080
ATOM	804	HE1	TYR	A	53	-9.666	-14.604	5.815	1.00	19.65	H	0.075
ATOM	805	HE2	TYR	A	53	-10.243	-11.886	2.921	1.00	18.87	H	0.074
ATOM	806	HH	TYR	A	53	-11.434	-13.165	5.756	1.00	20.13	H	0.076
ATOM	807	N	GLY	A	54	-5.486	-15.561	-0.476	1.00	16.12	N	0.068
ATOM	808	CA	GLY	A	54	-4.195	-15.382	-1.127	1.00	18.26	C	0.072
ATOM	809	C	GLY	A	54	-3.749	-16.587	-1.938	1.00	16.17	C	0.068

```
ATOM    810  O   GLY A  54      -4.450 -17.587  -2.035  1.00 16.30           O  0.068
ATOM    811  H   GLY A  54      -6.168 -15.362  -0.960  1.00 19.34           H  0.074
ATOM    812  HA2 GLY A  54      -4.245 -14.620  -1.725  1.00 21.91           H  0.079
ATOM    813  HA3 GLY A  54      -3.521 -15.209  -0.451  1.00 21.91           H  0.079
ATOM    814  N   ILE A  55      -2.523 -16.485  -2.467  1.00 17.91           N  0.072
ATOM    815  CA  ILE A  55      -1.924 -17.543  -3.286  1.00 16.99           C  0.070
ATOM    816  C   ILE A  55      -1.879 -18.891  -2.574  1.00 17.17           C  0.070
ATOM    817  O   ILE A  55      -1.863 -19.943  -3.224  1.00 16.86           O  0.070
ATOM    818  CB  ILE A  55      -0.483 -17.234  -3.789  1.00 24.68           C  0.084
ATOM    819  CG1 ILE A  55       0.388 -16.546  -2.728  1.00 23.13           C  0.081
ATOM    820  CG2 ILE A  55      -0.486 -16.565  -5.229  1.00 26.51           C  0.087
ATOM    821  CD1 ILE A  55       1.879 -16.819  -2.999  1.00 28.86           C  0.091
ATOM    822  H   ILE A  55      -2.009 -15.803  -2.364  1.00 21.50           H  0.078
ATOM    823  HA  ILE A  55      -2.520 -17.576  -4.050  1.00 20.39           H  0.076
ATOM    824  HB  ILE A  55      -0.031 -18.082  -3.922  1.00 29.61           H  0.092
ATOM    825 HG12 ILE A  55       0.238 -15.588  -2.756  1.00 27.75           H  0.089
ATOM    826 HG13 ILE A  55       0.163 -16.892  -1.850  1.00 27.75           H  0.089
ATOM    827 HG21 ILE A  55       0.430 -16.455  -5.528  1.00 31.82           H  0.095
ATOM    828 HG22 ILE A  55      -0.967 -17.141  -5.844  1.00 31.82           H  0.095
ATOM    829 HG23 ILE A  55      -0.923 -15.701  -5.175  1.00 31.82           H  0.095
ATOM    830 HD11 ILE A  55       2.409 -16.386  -2.313  1.00 34.63           H  0.100
ATOM    831 HD12 ILE A  55       2.033 -17.777  -2.981  1.00 34.63           H  0.100
ATOM    832 HD13 ILE A  55       2.112 -16.464  -3.871  1.00 34.63           H  0.100
ATOM    833  N   LEU A  56      -1.763 -18.885  -1.258  1.00 15.45           N  0.067
ATOM    834  CA  LEU A  56      -1.603 -20.098  -0.453  1.00 18.71           C  0.073
ATOM    835  C   LEU A  56      -2.772 -20.267   0.517  1.00 19.27           C  0.074
ATOM    836  O   LEU A  56      -2.712 -21.132   1.423  1.00 15.72           O  0.067
ATOM    837  CB  LEU A  56      -0.254 -20.057   0.278  1.00 18.33           C  0.072
ATOM    838  CG  LEU A  56       0.995 -20.396  -0.621  1.00 19.62           C  0.075
ATOM    839  CD1 LEU A  56       2.377 -20.173   0.122  1.00 16.42           C  0.069
ATOM    840  CD2 LEU A  56       0.918 -21.866  -1.131  1.00 18.60           C  0.073
ATOM    841  H   LEU A  56      -1.773 -18.167  -0.785  1.00 18.54           H  0.073
ATOM    842  HA  LEU A  56      -1.585 -20.885  -1.019  1.00 22.45           H  0.080
ATOM    843  HB2 LEU A  56      -0.124 -19.164   0.633  1.00 22.00           H  0.079
ATOM    844  HB3 LEU A  56      -0.277 -20.702   1.001  1.00 22.00           H  0.079
ATOM    845  HG  LEU A  56       0.972 -19.783  -1.372  1.00 23.54           H  0.082
ATOM    846 HD11 LEU A  56       3.100 -20.422  -0.474  1.00 19.71           H  0.075
ATOM    847 HD12 LEU A  56       2.458 -19.237   0.366  1.00 19.71           H  0.075
ATOM    848 HD13 LEU A  56       2.400 -20.725   0.919  1.00 19.71           H  0.075
ATOM    849 HD21 LEU A  56       1.767 -22.105  -1.535  1.00 22.32           H  0.080
ATOM    850 HD22 LEU A  56       0.732 -22.451  -0.381  1.00 22.32           H  0.080
ATOM    851 HD23 LEU A  56       0.209 -21.935  -1.789  1.00 22.32           H  0.080
ATOM    852  N   GLN A  57      -3.860 -19.504   0.315  1.00 15.03           N  0.066
ATOM    853  CA  GLN A  57      -5.145 -19.759   0.956  1.00 13.17           C  0.061
ATOM    854  C   GLN A  57      -4.997 -19.909   2.483  1.00 16.63           C  0.069
ATOM    855  O   GLN A  57      -5.464 -20.872   3.101  1.00 15.94           O  0.068
ATOM    856  CB  GLN A  57      -5.808 -20.973   0.331  1.00 18.61           C  0.073
ATOM    857  CG  GLN A  57      -6.327 -20.643  -1.049  1.00 17.97           C  0.072
ATOM    858  CD  GLN A  57      -7.506 -19.683  -0.993  1.00 14.31           C  0.064
ATOM    859  OE1 GLN A  57      -8.674 -20.080  -0.860  1.00 14.84           O  0.065
ATOM    860  NE2 GLN A  57      -7.195 -18.398  -1.119  1.00 16.69           N  0.069
ATOM    861  H   GLN A  57      -3.872 -18.818  -0.203  1.00 18.04           H  0.072
ATOM    862  HA  GLN A  57      -5.721 -18.992   0.812  1.00 15.80           H  0.067
ATOM    863  HB2 GLN A  57      -5.162 -21.692   0.256  1.00 22.33           H  0.080
ATOM    864  HB3 GLN A  57      -6.555 -21.254   0.883  1.00 22.33           H  0.080
ATOM    865  HG2 GLN A  57      -5.620 -20.227  -1.566  1.00 21.56           H  0.079
ATOM    866  HG3 GLN A  57      -6.620 -21.459  -1.485  1.00 21.56           H  0.079
ATOM    867 HE21 GLN A  57      -6.374 -18.163  -1.223  1.00 20.03           H  0.076
ATOM    868 HE22 GLN A  57      -7.813 -17.801  -1.096  1.00 20.03           H  0.076
ATOM    869  N   ILE A  58      -4.387 -18.891   3.064  1.00 15.44           N  0.067
ATOM    870  CA  ILE A  58      -4.120 -18.837   4.504  1.00 13.71           C  0.063
ATOM    871  C   ILE A  58      -5.375 -18.448   5.272  1.00 20.41           C  0.076
ATOM    872  O   ILE A  58      -6.082 -17.498   4.926  1.00 14.32           O  0.064
ATOM    873  CB  ILE A  58      -2.941 -17.888   4.775  1.00 15.58           C  0.067
ATOM    874  CG1 ILE A  58      -1.659 -18.501   4.204  1.00 18.96           C  0.074
ATOM    875  CG2 ILE A  58      -2.737 -17.534   6.289  1.00 16.24           C  0.068
ATOM    876  CD1 ILE A  58      -0.552 -17.568   4.207  1.00 14.92           C  0.065
ATOM    877  H   ILE A  58      -4.108 -18.197   2.642  1.00 18.53           H  0.073
ATOM    878  HA  ILE A  58      -3.885 -19.724   4.819  1.00 16.45           H  0.069
ATOM    879  HB  ILE A  58      -3.154 -17.051   4.332  1.00 18.70           H  0.073
ATOM    880 HG12 ILE A  58      -1.408 -19.269   4.742  1.00 22.75           H  0.081
ATOM    881 HG13 ILE A  58      -1.819 -18.776   3.288  1.00 22.75           H  0.081
ATOM    882 HG21 ILE A  58      -1.942 -16.987   6.381  1.00 19.49           H  0.075
ATOM    883 HG22 ILE A  58      -3.513 -17.046   6.606  1.00 19.49           H  0.075
ATOM    884 HG23 ILE A  58      -2.634 -18.356   6.794  1.00 19.49           H  0.075
ATOM    885 HD11 ILE A  58       0.147 -17.906   3.626  1.00 17.90           H  0.072
ATOM    886 HD12 ILE A  58      -0.864 -16.708   3.885  1.00 17.90           H  0.072
ATOM    887 HD13 ILE A  58      -0.216 -17.480   5.113  1.00 17.90           H  0.072
```

```
ATOM    888  N   ASN A  59      -5.662 -19.227   6.313  1.00 18.74           N  0.073
ATOM    889  CA  ASN A  59      -6.931 -19.224   6.994  1.00 19.01           C  0.074
ATOM    890  C   ASN A  59      -6.965 -18.192   8.104  1.00 16.42           C  0.069
ATOM    891  O   ASN A  59      -6.014 -18.064   8.865  1.00 21.69           O  0.079
ATOM    892  CB  ASN A  59      -7.203 -20.577   7.641  1.00 18.93           C  0.074
ATOM    893  CG  ASN A  59      -8.589 -20.674   8.121  1.00 22.38           C  0.080
ATOM    894  OD1 ASN A  59      -8.843 -20.664   9.317  1.00 28.37           O  0.090
ATOM    895  ND2 ASN A  59      -9.507 -20.703   7.211  1.00 24.07           N  0.083
ATOM    896  H   ASN A  59      -5.105 -19.789   6.652  1.00 22.49           H  0.080
ATOM    897  HA  ASN A  59      -7.612 -19.016   6.335  1.00 22.81           H  0.081
ATOM    898  HB2 ASN A  59      -7.055 -21.280   6.989  1.00 22.72           H  0.081
ATOM    899  HB3 ASN A  59      -6.608 -20.696   8.398  1.00 22.72           H  0.081
ATOM    900 HD21 ASN A  59     -10.335 -20.759   7.436  1.00 28.89           H  0.091
ATOM    901 HD22 ASN A  59      -9.289 -20.665   6.380  1.00 28.89           H  0.091
ATOM    902  N   SER A  60      -8.090 -17.479   8.203  1.00 18.14           N  0.072
ATOM    903  CA  SER A  60      -8.303 -16.456   9.209  1.00 18.55           C  0.073
ATOM    904  C   SER A  60      -8.590 -17.040  10.597  1.00 23.94           C  0.083
ATOM    905  O   SER A  60      -8.695 -16.276  11.564  1.00 24.23           O  0.083
ATOM    906  CB  SER A  60      -9.483 -15.576   8.821  1.00 15.49           C  0.067
ATOM    907  OG  SER A  60     -10.670 -16.361   8.760  1.00 15.90           O  0.067
ATOM    908  H   SER A  60      -8.765 -17.579   7.679  1.00 21.77           H  0.079
ATOM    909  HA  SER A  60      -7.503 -15.909   9.254  1.00 22.26           H  0.080
ATOM    910  HB2 SER A  60      -9.595 -14.878   9.486  1.00 18.58           H  0.073
ATOM    911  HB3 SER A  60      -9.316 -15.181   7.951  1.00 18.58           H  0.073
ATOM    912  HG  SER A  60     -11.329 -15.872   8.582  1.00 19.08           H  0.074
ATOM    913  N   ARG A  61      -8.762 -18.354  10.721  1.00 21.35           N  0.078
ATOM    914  CA  ARG A  61      -9.018 -18.928  12.059  1.00 25.22           C  0.085
ATOM    915  C   ARG A  61      -7.792 -18.797  12.940  1.00 29.02           C  0.091
ATOM    916  O   ARG A  61      -7.912 -18.567  14.143  1.00 26.91           O  0.088
ATOM    917  CB  ARG A  61      -9.448 -20.390  11.934  1.00 27.97           C  0.090
ATOM    918  CG  ARG A  61     -10.217 -20.991  13.142  1.00 47.15           C  0.116
ATOM    919  CD  ARG A  61     -11.732 -20.613  13.109  1.00 60.39           C  0.132
ATOM    920  NE  ARG A  61     -12.227 -20.143  14.405  1.00 72.11           N  0.144
ATOM    921  CZ  ARG A  61     -13.504 -20.125  14.780  1.00 63.28           C  0.135
ATOM    922  NH1 ARG A  61     -14.477 -20.519  13.972  1.00 56.46           N  0.127
ATOM    923  NH2 ARG A  61     -13.813 -19.707  15.998  1.00 61.50           N  0.133
ATOM    924  H   ARG A  61      -8.737 -18.923  10.076  1.00 25.63           H  0.086
ATOM    925  HA  ARG A  61      -9.749 -18.452  12.482  1.00 30.26           H  0.093
ATOM    926  HB2 ARG A  61     -10.028 -20.468  11.160  1.00 33.56           H  0.098
ATOM    927  HB3 ARG A  61      -8.651 -20.929  11.807  1.00 33.56           H  0.098
ATOM    928  HG2 ARG A  61     -10.145 -21.958  13.119  1.00 56.58           H  0.127
ATOM    929  HG3 ARG A  61      -9.837 -20.649  13.966  1.00 56.58           H  0.127
ATOM    930  HD2 ARG A  61     -11.867 -19.904  12.461  1.00 72.46           H  0.144
ATOM    931  HD3 ARG A  61     -12.248 -21.395  12.857  1.00 72.46           H  0.144
ATOM    932  HE  ARG A  61     -11.646 -19.854  14.969  1.00 86.53           H  0.157
ATOM    933 HH11 ARG A  61     -14.292 -20.796  13.179  1.00 67.75           H  0.139
ATOM    934 HH12 ARG A  61     -15.294 -20.497  14.240  1.00 67.75           H  0.139
ATOM    935 HH21 ARG A  61     -13.192 -19.452  16.535  1.00 73.80           H  0.145
ATOM    936 HH22 ARG A  61     -14.634 -19.691  16.252  1.00 73.80           H  0.145
ATOM    937  N   TRP A  62      -6.601 -18.846  12.334  1.00 24.23           N  0.083
ATOM    938  CA  TRP A  62      -5.363 -18.904  13.086  1.00 24.37           C  0.084
ATOM    939  C   TRP A  62      -4.385 -17.811  12.699  1.00 24.09           C  0.083
ATOM    940  O   TRP A  62      -3.692 -17.281  13.562  1.00 21.89           O  0.079
ATOM    941  CB  TRP A  62      -4.676 -20.258  12.876  1.00 25.31           C  0.085
ATOM    942  CG  TRP A  62      -5.526 -21.386  13.271  1.00 26.80           C  0.088
ATOM    943  CD1 TRP A  62      -6.290 -22.139  12.452  1.00 31.86           C  0.096
ATOM    944  CD2 TRP A  62      -5.740 -21.871  14.594  1.00 31.45           C  0.095
ATOM    945  NE1 TRP A  62      -6.984 -23.066  13.183  1.00 35.89           N  0.101
ATOM    946  CE2 TRP A  62      -6.645 -22.938  14.498  1.00 34.91           C  0.100
ATOM    947  CE3 TRP A  62      -5.257 -21.498  15.860  1.00 33.91           C  0.099
ATOM    948  CZ2 TRP A  62      -7.087 -23.655  15.615  1.00 50.18           C  0.120
ATOM    949  CZ3 TRP A  62      -5.677 -22.228  16.974  1.00 41.19           C  0.109
ATOM    950  CH2 TRP A  62      -6.596 -23.288  16.839  1.00 49.20           C  0.119
ATOM    951  H   TRP A  62      -6.492 -18.847  11.481  1.00 29.08           H  0.091
ATOM    952  HA  TRP A  62      -5.596 -18.782  14.020  1.00 29.24           H  0.092
ATOM    953  HB2 TRP A  62      -4.457 -20.360  11.936  1.00 30.38           H  0.093
ATOM    954  HB3 TRP A  62      -3.867 -20.288  13.410  1.00 30.38           H  0.093
ATOM    955  HD1 TRP A  62      -6.338 -22.043  11.528  1.00 38.24           H  0.105
ATOM    956  HE1 TRP A  62      -7.543 -23.636  12.864  1.00 43.07           H  0.111
ATOM    957  HE3 TRP A  62      -4.672 -20.781  15.954  1.00 40.69           H  0.108
ATOM    958  HZ2 TRP A  62      -7.692 -24.357  15.530  1.00 60.22           H  0.131
ATOM    959  HZ3 TRP A  62      -5.346 -22.011  17.816  1.00 49.43           H  0.119
ATOM    960  HH2 TRP A  62      -6.875 -23.747  17.598  1.00 59.04           H  0.130
ATOM    961  N   TRP A  63      -4.351 -17.429  11.420  1.00 17.82           N  0.071
ATOM    962  CA  TRP A  63      -3.154 -16.826  10.862  1.00 18.01           C  0.072
ATOM    963  C   TRP A  63      -3.282 -15.344  10.543  1.00 16.66           C  0.069
ATOM    964  O   TRP A  63      -2.287 -14.635  10.625  1.00 20.55           O  0.077
ATOM    965  CB  TRP A  63      -2.771 -17.602   9.592  1.00 18.93           C  0.074
```

ATOM	966	CG	TRP	A	63	-2.563	-19.011	9.905	1.00	16.12	C	0.068
ATOM	967	CD1	TRP	A	63	-3.330	-20.077	9.517	1.00	18.00	C	0.072
ATOM	968	CD2	TRP	A	63	-1.504	-19.552	10.726	1.00	16.78	C	0.069
ATOM	969	NE1	TRP	A	63	-2.783	-21.220	10.004	1.00	20.03	N	0.076
ATOM	970	CE2	TRP	A	63	-1.688	-20.930	10.773	1.00	21.24	C	0.078
ATOM	971	CE3	TRP	A	63	-0.416	-18.995	11.393	1.00	16.52	C	0.069
ATOM	972	CZ2	TRP	A	63	-0.832	-21.784	11.493	1.00	22.34	C	0.080
ATOM	973	CZ3	TRP	A	63	0.454	-19.863	12.123	1.00	19.16	C	0.074
ATOM	974	CH2	TRP	A	63	0.225	-21.220	12.146	1.00	19.59	C	0.075
ATOM	975	H	TRP	A	63	-5.004	-17.511	10.868	1.00	21.39	H	0.078
ATOM	976	HA	TRP	A	63	-2.427	-16.893	11.501	1.00	21.61	H	0.079
ATOM	977	HB2	TRP	A	63	-3.484	-17.530	8.939	1.00	22.72	H	0.081
ATOM	978	HB3	TRP	A	63	-1.948	-17.240	9.225	1.00	22.72	H	0.081
ATOM	979	HD1	TRP	A	63	-4.103	-20.026	9.003	1.00	21.60	H	0.079
ATOM	980	HE1	TRP	A	63	-3.081	-22.012	9.852	1.00	24.04	H	0.083
ATOM	981	HE3	TRP	A	63	-0.260	-18.078	11.365	1.00	19.82	H	0.075
ATOM	982	HZ2	TRP	A	63	-0.982	-22.702	11.522	1.00	26.81	H	0.088
ATOM	983	HZ3	TRP	A	63	1.179	-19.509	12.585	1.00	22.99	H	0.081
ATOM	984	HH2	TRP	A	63	0.808	-21.769	12.620	1.00	23.51	H	0.082
ATOM	985	N	CYS	A	64	-4.473	-14.840	10.257	1.00	17.44	N	0.071
ATOM	986	CA	CYS	A	64	-4.613	-13.423	9.949	1.00	19.73	C	0.075
ATOM	987	C	CYS	A	64	-5.904	-12.916	10.554	1.00	17.15	C	0.070
ATOM	988	O	CYS	A	64	-6.799	-13.695	10.906	1.00	17.46	O	0.071
ATOM	989	CB	CYS	A	64	-4.573	-13.179	8.417	1.00	16.43	C	0.069
ATOM	990	SG	CYS	A	64	-5.859	-14.060	7.430	1.00	19.17	S	0.074
ATOM	991	H	CYS	A	64	-5.205	-15.292	10.234	1.00	20.92	H	0.077
ATOM	992	HA	CYS	A	64	-3.884	-12.924	10.351	1.00	23.68	H	0.082
ATOM	993	HB2	CYS	A	64	-4.688	-12.229	8.258	1.00	19.71	H	0.075
ATOM	994	HB3	CYS	A	64	-3.708	-13.468	8.086	1.00	19.71	H	0.075
ATOM	995	N	ASN	A	65	-6.027	-11.587	10.609	1.00	20.28	N	0.076
ATOM	996	CA	ASN	A	65	-7.236	-10.969	11.118	1.00	24.78	C	0.084
ATOM	997	C	ASN	A	65	-8.082	-10.413	9.969	1.00	16.07	C	0.068
ATOM	998	O	ASN	A	65	-7.584	-9.619	9.172	1.00	20.76	O	0.077
ATOM	999	CB	ASN	A	65	-6.922	-9.835	12.079	1.00	24.24	C	0.083
ATOM	1000	CG	ASN	A	65	-8.173	-9.137	12.507	1.00	26.68	C	0.087
ATOM	1001	OD1	ASN	A	65	-9.116	-9.781	13.026	1.00	21.81	O	0.079
ATOM	1002	ND2	ASN	A	65	-8.251	-7.850	12.199	1.00	29.45	N	0.092
ATOM	1003	H	ASN	A	65	-5.422	-11.031	10.355	1.00	24.34	H	0.084
ATOM	1004	HA	ASN	A	65	-7.739	-11.660	11.578	1.00	29.73	H	0.092
ATOM	1005	HB2	ASN	A	65	-6.484	-10.191	12.868	1.00	29.09	H	0.091
ATOM	1006	HB3	ASN	A	65	-6.345	-9.191	11.641	1.00	29.09	H	0.091
ATOM	1007	HD21	ASN	A	65	-8.948	-7.396	12.420	1.00	35.34	H	0.101
ATOM	1008	HD22	ASN	A	65	-7.606	-7.468	11.778	1.00	35.34	H	0.101
ATOM	1009	N	ASP	A	66	-9.320	-10.860	9.881	1.00	18.09	N	0.072
ATOM	1010	CA	ASP	A	66	-10.277	-10.293	8.938	1.00	17.27	C	0.070
ATOM	1011	C	ASP	A	66	-11.463	-9.713	9.685	1.00	18.14	C	0.072
ATOM	1012	O	ASP	A	66	-12.453	-9.330	9.058	1.00	19.34	O	0.074
ATOM	1013	CB	ASP	A	66	-10.714	-11.324	7.902	1.00	15.86	C	0.067
ATOM	1014	CG	ASP	A	66	-11.540	-12.430	8.465	1.00	17.56	C	0.071
ATOM	1015	OD1	ASP	A	66	-11.943	-12.384	9.658	1.00	20.60	O	0.077
ATOM	1016	OD2	ASP	A	66	-11.766	-13.404	7.686	1.00	14.68	O	0.065
ATOM	1017	H	ASP	A	66	-9.638	-11.499	10.361	1.00	21.71	H	0.079
ATOM	1018	HA	ASP	A	66	-9.857	-9.584	8.426	1.00	20.72	H	0.077
ATOM	1019	HB2	ASP	A	66	-11.242	-10.877	7.222	1.00	19.03	H	0.074
ATOM	1020	HB3	ASP	A	66	-9.924	-11.719	7.502	1.00	19.03	H	0.074
ATOM	1021	N	GLY	A	67	-11.389	-9.663	11.003	1.00	21.05	N	0.078
ATOM	1022	CA	GLY	A	67	-12.400	-8.991	11.789	1.00	20.07	C	0.076
ATOM	1023	C	GLY	A	67	-13.724	-9.678	11.894	1.00	29.18	C	0.091
ATOM	1024	O	GLY	A	67	-14.632	-9.128	12.541	1.00	31.85	O	0.096
ATOM	1025	H	GLY	A	67	-10.757	-10.014	11.468	1.00	25.26	H	0.085
ATOM	1026	HA2	GLY	A	67	-12.060	-8.878	12.691	1.00	24.09	H	0.083
ATOM	1027	HA3	GLY	A	67	-12.557	-8.116	11.400	1.00	24.09	H	0.083
ATOM	1028	N	ARG	A	68	-13.871	-10.855	11.288	1.00	21.75	N	0.079
ATOM	1029	CA	ARG	A	68	-15.161	-11.529	11.212	1.00	19.95	C	0.076
ATOM	1030	C	ARG	A	68	-14.996	-13.018	11.385	1.00	25.56	C	0.086
ATOM	1031	O	ARG	A	68	-15.798	-13.809	10.863	1.00	23.05	O	0.081
ATOM	1032	CB	ARG	A	68	-15.832	-11.180	9.889	1.00	24.11	C	0.083
ATOM	1033	CG	ARG	A	68	-15.109	-11.805	8.670	1.00	21.10	C	0.078
ATOM	1034	CD	ARG	A	68	-16.132	-12.064	7.580	1.00	20.23	C	0.076
ATOM	1035	NE	ARG	A	68	-15.776	-13.025	6.553	1.00	25.42	N	0.085
ATOM	1036	CZ	ARG	A	68	-16.114	-12.883	5.271	1.00	32.55	C	0.097
ATOM	1037	NH1	ARG	A	68	-16.864	-11.871	4.877	1.00	27.68	N	0.089
ATOM	1038	NH2	ARG	A	68	-15.695	-13.787	4.358	1.00	17.73	N	0.071
ATOM	1039	H	ARG	A	68	-13.231	-11.287	10.909	1.00	26.10	H	0.086
ATOM	1040	HA	ARG	A	68	-15.746	-11.236	11.928	1.00	23.94	H	0.083
ATOM	1041	HB2	ARG	A	68	-16.744	-11.511	9.900	1.00	28.93	H	0.091
ATOM	1042	HB3	ARG	A	68	-15.831	-10.216	9.778	1.00	28.93	H	0.091
ATOM	1043	HG2	ARG	A	68	-14.436	-11.192	8.333	1.00	25.33	H	0.085

ATOM	1044	HG3	ARG	A	68	-14.696	-12.645	8.923	1.00	25.33	H	0.085
ATOM	1045	HD2	ARG	A	68	-16.942	-12.390	8.001	1.00	24.28	H	0.083
ATOM	1046	HD3	ARG	A	68	-16.308	-11.223	7.128	1.00	24.28	H	0.083
ATOM	1047	HE	ARG	A	68	-15.324	-13.721	6.782	1.00	30.50	H	0.093
ATOM	1048	HH11	ARG	A	68	-17.141	-11.292	5.450	1.00	33.21	H	0.098
ATOM	1049	HH12	ARG	A	68	-17.075	-11.790	4.047	1.00	33.21	H	0.098
ATOM	1050	HH21	ARG	A	68	-15.210	-14.453	4.604	1.00	21.27	H	0.078
ATOM	1051	HH22	ARG	A	68	-15.913	-13.695	3.531	1.00	21.27	H	0.078
ATOM	1052	N	THR	A	69	-13.939	-13.437	12.096	1.00	20.84	N	0.077
ATOM	1053	CA	THR	A	69	-13.764	-14.853	12.395	1.00	21.74	C	0.079
ATOM	1054	C	THR	A	69	-13.760	-14.971	13.919	1.00	20.11	C	0.076
ATOM	1055	O	THR	A	69	-12.709	-14.908	14.551	1.00	19.19	O	0.074
ATOM	1056	CB	THR	A	69	-12.492	-15.411	11.770	1.00	24.92	C	0.085
ATOM	1057	OG1	THR	A	69	-12.461	-15.067	10.352	1.00	19.97	O	0.076
ATOM	1058	CG2	THR	A	69	-12.477	-16.942	11.924	1.00	25.93	C	0.086
ATOM	1059	H	THR	A	69	-13.325	-12.922	12.407	1.00	25.01	H	0.085
ATOM	1060	HA	THR	A	69	-14.498	-15.374	12.034	1.00	26.09	H	0.086
ATOM	1061	HB	THR	A	69	-11.709	-15.040	12.205	1.00	29.91	H	0.093
ATOM	1062	HG1	THR	A	69	-12.477	-14.232	10.259	1.00	23.96	H	0.083
ATOM	1063	HG21	THR	A	69	-11.653	-17.305	11.562	1.00	31.11	H	0.094
ATOM	1064	HG22	THR	A	69	-12.540	-17.182	12.862	1.00	31.11	H	0.094
ATOM	1065	HG23	THR	A	69	-13.228	-17.331	11.449	1.00	31.11	H	0.094
ATOM	1066	N	PRO	A	70	-14.921	-15.095	14.542	1.00	22.20	N	0.080
ATOM	1067	CA	PRO	A	70	-14.953	-15.058	16.005	1.00	32.05	C	0.096
ATOM	1068	C	PRO	A	70	-14.019	-16.059	16.660	1.00	30.52	C	0.094
ATOM	1069	O	PRO	A	70	-13.886	-17.214	16.239	1.00	31.46	O	0.095
ATOM	1070	CB	PRO	A	70	-16.415	-15.337	16.316	1.00	33.31	C	0.098
ATOM	1071	CG	PRO	A	70	-17.109	-14.603	15.237	1.00	30.25	C	0.093
ATOM	1072	CD	PRO	A	70	-16.210	-14.681	13.978	1.00	26.25	C	0.087
ATOM	1073	HA	PRO	A	70	-14.702	-14.175	16.318	1.00	38.46	H	0.105
ATOM	1074	HB2	PRO	A	70	-16.597	-16.288	16.275	1.00	39.98	H	0.107
ATOM	1075	HB3	PRO	A	70	-16.650	-14.991	17.191	1.00	39.98	H	0.107
ATOM	1076	HG2	PRO	A	70	-17.968	-15.020	15.066	1.00	36.29	H	0.102
ATOM	1077	HG3	PRO	A	70	-17.236	-13.680	15.507	1.00	36.29	H	0.102
ATOM	1078	HD2	PRO	A	70	-16.544	-15.340	13.350	1.00	31.50	H	0.095
ATOM	1079	HD3	PRO	A	70	-16.140	-13.816	13.545	1.00	31.50	H	0.095
ATOM	1080	N	GLY	A	71	-13.315	-15.574	17.685	1.00	25.91	N	0.086
ATOM	1081	CA	GLY	A	71	-12.470	-16.435	18.462	1.00	40.00	C	0.107
ATOM	1082	C	GLY	A	71	-11.166	-16.753	17.790	1.00	36.89	C	0.103
ATOM	1083	O	GLY	A	71	-10.372	-17.512	18.343	1.00	43.17	O	0.111
ATOM	1084	H	GLY	A	71	-13.321	-14.752	17.938	1.00	31.09	H	0.094
ATOM	1085	HA2	GLY	A	71	-12.276	-16.006	19.310	1.00	48.00	H	0.117
ATOM	1086	HA3	GLY	A	71	-12.935	-17.269	18.629	1.00	48.00	H	0.117
ATOM	1087	N	SER	A	72	-10.903	-16.167	16.630	1.00	28.55	N	0.090
ATOM	1088	CA	SER	A	72	-9.716	-16.537	15.899	1.00	29.97	C	0.093
ATOM	1089	C	SER	A	72	-8.489	-15.856	16.486	1.00	36.86	C	0.103
ATOM	1090	O	SER	A	72	-8.553	-14.924	17.303	1.00	35.46	O	0.101
ATOM	1091	CB	SER	A	72	-9.883	-16.187	14.410	1.00	29.89	C	0.093
ATOM	1092	OG	SER	A	72	-9.955	-14.782	14.244	1.00	34.33	O	0.099
ATOM	1093	H	SER	A	72	-11.390	-15.565	16.258	1.00	34.27	H	0.099
ATOM	1094	HA	SER	A	72	-9.577	-17.495	15.968	1.00	35.97	H	0.102
ATOM	1095	HB2	SER	A	72	-9.122	-16.528	13.915	1.00	35.87	H	0.101
ATOM	1096	HB3	SER	A	72	-10.702	-16.589	14.079	1.00	35.87	H	0.101
ATOM	1097	HG	SER	A	72	-10.526	-14.459	14.769	1.00	41.20	H	0.109
ATOM	1098	N	ARG	A	73	-7.356	-16.338	16.025	1.00	25.53	N	0.086
ATOM	1099	CA	ARG	A	73	-6.063	-15.768	16.289	1.00	27.28	C	0.088
ATOM	1100	C	ARG	A	73	-5.573	-15.174	14.973	1.00	27.74	C	0.089
ATOM	1101	O	ARG	A	73	-6.123	-15.397	13.885	1.00	31.33	O	0.095
ATOM	1102	CB	ARG	A	73	-5.120	-16.848	16.822	1.00	32.30	C	0.096
ATOM	1103	CG	ARG	A	73	-5.783	-17.806	17.827	1.00	34.84	C	0.100
ATOM	1104	CD	ARG	A	73	-5.707	-17.274	19.252	1.00	45.74	C	0.114
ATOM	1105	NE	ARG	A	73	-4.320	-16.942	19.555	1.00	49.99	N	0.120
ATOM	1106	CZ	ARG	A	73	-3.600	-17.497	20.515	1.00	54.43	C	0.125
ATOM	1107	NH1	ARG	A	73	-4.146	-18.317	21.401	1.00	62.63	N	0.134
ATOM	1108	NH2	ARG	A	73	-2.293	-17.233	20.582	1.00	48.18	N	0.117
ATOM	1109	H	ARG	A	73	-7.312	-17.038	15.528	1.00	30.64	H	0.094
ATOM	1110	HA	ARG	A	73	-6.091	-15.070	16.962	1.00	32.73	H	0.097
ATOM	1111	HB2	ARG	A	73	-4.797	-17.377	16.076	1.00	38.76	H	0.105
ATOM	1112	HB3	ARG	A	73	-4.375	-16.418	17.271	1.00	38.76	H	0.105
ATOM	1113	HG2	ARG	A	73	-6.718	-17.916	17.594	1.00	41.81	H	0.109
ATOM	1114	HG3	ARG	A	73	-5.331	-18.663	17.798	1.00	41.81	H	0.109
ATOM	1115	HD2	ARG	A	73	-6.249	-16.474	19.336	1.00	54.89	H	0.125
ATOM	1116	HD3	ARG	A	73	-6.014	-17.951	19.875	1.00	54.89	H	0.125
ATOM	1117	HE	ARG	A	73	-3.942	-16.339	19.073	1.00	59.98	H	0.131
ATOM	1118	HH11	ARG	A	73	-4.986	-18.497	21.359	1.00	75.15	H	0.147
ATOM	1119	HH12	ARG	A	73	-3.662	-18.668	22.019	1.00	75.15	H	0.147
ATOM	1120	HH21	ARG	A	73	-1.931	-16.708	20.006	1.00	57.82	H	0.129
ATOM	1121	HH22	ARG	A	73	-1.814	-17.588	21.202	1.00	57.82	H	0.129

```
ATOM   1122  N   ASN A  74      -4.489 -14.430  15.112  1.00 22.98           N  0.081
ATOM   1123  CA  ASN A  74      -3.891 -13.663  14.082  1.00 20.30           C  0.076
ATOM   1124  C   ASN A  74      -2.392 -13.858  14.238  1.00 23.86           C  0.083
ATOM   1125  O   ASN A  74      -1.651 -12.927  14.534  1.00 26.03           O  0.086
ATOM   1126  CB  ASN A  74      -4.252 -12.192  14.213  1.00 21.27           C  0.078
ATOM   1127  CG  ASN A  74      -3.523 -11.314  13.172  1.00 19.36           C  0.074
ATOM   1128  OD1 ASN A  74      -2.805 -11.808  12.290  1.00 22.41           O  0.080
ATOM   1129  ND2 ASN A  74      -3.732 -10.008  13.266  1.00 26.17           N  0.087
ATOM   1130  H   ASN A  74      -4.064 -14.359  15.856  1.00 27.58           H  0.089
ATOM   1131  HA  ASN A  74      -4.196 -13.965  13.212  1.00 24.36           H  0.084
ATOM   1132  HB2 ASN A  74      -5.207 -12.086  14.081  1.00 25.53           H  0.086
ATOM   1133  HB3 ASN A  74      -4.002 -11.880  15.097  1.00 25.53           H  0.086
ATOM   1134 HD21 ASN A  74      -3.351  -9.471  12.713  1.00 31.40           H  0.095
ATOM   1135 HD22 ASN A  74      -4.247  -9.698  13.881  1.00 31.40           H  0.095
ATOM   1136  N   LEU A  75      -1.964 -15.114  14.084  1.00 23.79           N  0.083
ATOM   1137  CA  LEU A  75      -0.607 -15.520  14.414  1.00 26.65           C  0.087
ATOM   1138  C   LEU A  75       0.425 -14.931  13.461  1.00 27.84           C  0.089
ATOM   1139  O   LEU A  75       1.592 -14.804  13.844  1.00 26.37           O  0.087
ATOM   1140  CB  LEU A  75      -0.575 -17.052  14.445  1.00 26.46           C  0.087
ATOM   1141  CG  LEU A  75      -1.343 -17.642  15.642  1.00 33.61           C  0.098
ATOM   1142  CD1 LEU A  75      -1.384 -19.137  15.600  1.00 29.68           C  0.092
ATOM   1143  CD2 LEU A  75      -0.733 -17.208  16.978  1.00 35.00           C  0.100
ATOM   1144  H   LEU A  75      -2.451 -15.757  13.786  1.00 28.55           H  0.090
ATOM   1145  HA  LEU A  75      -0.349 -15.199  15.292  1.00 31.97           H  0.096
ATOM   1146  HB2 LEU A  75      -0.979 -17.392  13.632  1.00 31.76           H  0.095
ATOM   1147  HB3 LEU A  75       0.347 -17.346  14.505  1.00 31.76           H  0.095
ATOM   1148  HG  LEU A  75      -2.249 -17.301  15.581  1.00 40.33           H  0.108
ATOM   1149 HD11 LEU A  75      -2.307 -19.429  15.658  1.00 35.61           H  0.101
ATOM   1150 HD12 LEU A  75      -0.993 -19.441  14.766  1.00 35.61           H  0.101
ATOM   1151 HD13 LEU A  75      -0.879 -19.488  16.350  1.00 35.61           H  0.101
ATOM   1152 HD21 LEU A  75      -1.075 -17.781  17.682  1.00 42.01           H  0.110
ATOM   1153 HD22 LEU A  75       0.232 -17.289  16.925  1.00 42.01           H  0.110
ATOM   1154 HD23 LEU A  75      -0.978 -16.286  17.153  1.00 42.01           H  0.110
ATOM   1155  N   CYS A  76       0.047 -14.509  12.253  1.00 21.75           N  0.079
ATOM   1156  CA  CYS A  76       1.035 -13.821  11.439  1.00 17.49           C  0.071
ATOM   1157  C   CYS A  76       1.023 -12.312  11.657  1.00 22.63           C  0.081
ATOM   1158  O   CYS A  76       1.779 -11.600  10.990  1.00 26.09           O  0.086
ATOM   1159  CB  CYS A  76       0.823 -14.130   9.934  1.00 17.80           C  0.071
ATOM   1160  SG  CYS A  76       1.099 -15.885   9.664  1.00 20.64           S  0.077
ATOM   1161  H   CYS A  76      -0.734 -14.606  11.907  1.00 26.10           H  0.086
ATOM   1162  HA  CYS A  76       1.914 -14.156  11.679  1.00 20.99           H  0.078
ATOM   1163  HB2 CYS A  76      -0.085 -13.908   9.676  1.00 21.36           H  0.078
ATOM   1164  HB3 CYS A  76       1.453 -13.623   9.400  1.00 21.36           H  0.078
ATOM   1165  N   ASN A  77       0.157 -11.840  12.540  1.00 23.17           N  0.081
ATOM   1166  CA  ASN A  77       0.040 -10.438  12.893  1.00 23.59           C  0.082
ATOM   1167  C   ASN A  77      -0.117  -9.587  11.643  1.00 24.70           C  0.084
ATOM   1168  O   ASN A  77       0.669  -8.675  11.384  1.00 29.95           O  0.093
ATOM   1169  CB  ASN A  77       1.270 -10.049  13.722  1.00 26.22           C  0.087
ATOM   1170  CG  ASN A  77       1.399 -10.896  15.007  1.00 34.67           C  0.100
ATOM   1171  OD1 ASN A  77       2.432 -11.523  15.259  1.00 35.10           O  0.100
ATOM   1172  ND2 ASN A  77       0.358 -10.891  15.827  1.00 37.95           N  0.104
ATOM   1173  H   ASN A  77      -0.399 -12.337  12.968  1.00 27.80           H  0.089
ATOM   1174  HA  ASN A  77      -0.755 -10.279  13.424  1.00 28.31           H  0.090
ATOM   1175  HB2 ASN A  77       2.069 -10.184  13.190  1.00 31.47           H  0.095
ATOM   1176  HB3 ASN A  77       1.198  -9.117  13.981  1.00 31.47           H  0.095
ATOM   1177 HD21 ASN A  77      -0.341 -10.431  15.630  1.00 45.54           H  0.114
ATOM   1178 HD22 ASN A  77       0.382 -11.347  16.555  1.00 45.54           H  0.114
ATOM   1179  N   ILE A  78      -1.132  -9.932  10.845  1.00 23.26           N  0.082
ATOM   1180  CA  ILE A  78      -1.430  -9.196   9.613  1.00 27.32           C  0.088
ATOM   1181  C   ILE A  78      -2.932  -9.163   9.357  1.00 22.93           C  0.081
ATOM   1182  O   ILE A  78      -3.646 -10.125   9.674  1.00 21.43           O  0.078
ATOM   1183  CB  ILE A  78      -0.792  -9.798   8.329  1.00 24.42           C  0.084
ATOM   1184  CG1 ILE A  78      -0.994 -11.324   8.283  1.00 22.37           C  0.080
ATOM   1185  CG2 ILE A  78       0.617  -9.238   8.034  1.00 26.74           C  0.088
ATOM   1186  CD1 ILE A  78      -0.737 -11.954   6.876  1.00 21.35           C  0.078
ATOM   1187  H   ILE A  78      -1.664 -10.590  10.997  1.00 27.92           H  0.089
ATOM   1188  HA  ILE A  78      -1.070  -8.312   9.782  1.00 32.78           H  0.097
ATOM   1189  HB  ILE A  78      -1.274  -9.485   7.547  1.00 29.31           H  0.092
ATOM   1190 HG12 ILE A  78      -0.379 -11.739   8.909  1.00 26.85           H  0.088
ATOM   1191 HG13 ILE A  78      -1.909 -11.525   8.534  1.00 26.85           H  0.088
ATOM   1192 HG21 ILE A  78       0.927  -9.592   7.186  1.00 32.09           H  0.096
ATOM   1193 HG22 ILE A  78       0.569  -8.270   7.991  1.00 32.09           H  0.096
ATOM   1194 HG23 ILE A  78       1.219  -9.508   8.745  1.00 32.09           H  0.096
ATOM   1195 HD11 ILE A  78      -0.940 -12.902   6.912  1.00 25.62           H  0.086
ATOM   1196 HD12 ILE A  78      -1.310 -11.518   6.225  1.00 25.62           H  0.086
ATOM   1197 HD13 ILE A  78       0.194 -11.824   6.636  1.00 25.62           H  0.086
ATOM   1198  N   PRO A  79      -3.436  -8.126   8.711  1.00 26.56           N  0.087
ATOM   1199  CA  PRO A  79      -4.788  -8.210   8.163  1.00 16.85           C  0.069
```

```
ATOM   1200  C   PRO A  79      -4.821  -9.255   7.075  1.00 17.16           C  0.070
ATOM   1201  O   PRO A  79      -3.874  -9.377   6.296  1.00 20.78           O  0.077
ATOM   1202  CB  PRO A  79      -5.034  -6.814   7.579  1.00 21.62           C  0.079
ATOM   1203  CG  PRO A  79      -3.688  -6.173   7.421  1.00 28.77           C  0.091
ATOM   1204  CD  PRO A  79      -2.732  -6.871   8.371  1.00 24.72           C  0.084
ATOM   1205  HA  PRO A  79      -5.446  -8.391   8.853  1.00 20.22           H  0.076
ATOM   1206  HB2 PRO A  79      -5.475  -6.896   6.719  1.00 25.94           H  0.086
ATOM   1207  HB3 PRO A  79      -5.587  -6.300   8.188  1.00 25.94           H  0.086
ATOM   1208  HG2 PRO A  79      -3.389  -6.278   6.505  1.00 34.53           H  0.099
ATOM   1209  HG3 PRO A  79      -3.754  -5.231   7.643  1.00 34.53           H  0.099
ATOM   1210  HD2 PRO A  79      -1.887  -7.059   7.933  1.00 29.66           H  0.092
ATOM   1211  HD3 PRO A  79      -2.582  -6.335   9.165  1.00 29.66           H  0.092
ATOM   1212  N   CYS A  80      -5.924 -10.008   7.013  1.00 18.41           N  0.073
ATOM   1213  CA  CYS A  80      -5.982 -10.979   5.927  1.00 15.32           C  0.066
ATOM   1214  C   CYS A  80      -5.860 -10.275   4.589  1.00 15.18           C  0.066
ATOM   1215  O   CYS A  80      -5.279 -10.823   3.654  1.00 17.44           O  0.071
ATOM   1216  CB  CYS A  80      -7.251 -11.833   5.993  1.00 14.65           C  0.065
ATOM   1217  SG  CYS A  80      -7.342 -12.695   7.524  1.00 15.54           S  0.067
ATOM   1218  H   CYS A  80      -6.597  -9.977   7.547  1.00 22.10           H  0.080
ATOM   1219  HA  CYS A  80      -5.244 -11.602   6.023  1.00 18.38           H  0.073
ATOM   1220  HB2 CYS A  80      -8.030 -11.261   5.913  1.00 17.57           H  0.071
ATOM   1221  HB3 CYS A  80      -7.241 -12.483   5.273  1.00 17.57           H  0.071
ATOM   1222  N   SER A  81      -6.279  -9.013   4.503  1.00 17.33           N  0.070
ATOM   1223  CA  SER A  81      -6.156  -8.349   3.196  1.00 17.54           C  0.071
ATOM   1224  C   SER A  81      -4.713  -8.223   2.712  1.00 21.92           C  0.079
ATOM   1225  O   SER A  81      -4.467  -8.144   1.503  1.00 20.53           O  0.077
ATOM   1226  CB  SER A  81      -6.812  -6.990   3.252  1.00 22.16           C  0.080
ATOM   1227  OG  SER A  81      -6.261  -6.209   4.282  1.00 21.62           O  0.079
ATOM   1228  H   SER A  81      -6.617  -8.545   5.140  1.00 20.80           H  0.077
ATOM   1229  HA  SER A  81      -6.619  -8.895   2.542  1.00 21.05           H  0.078
ATOM   1230  HB2 SER A  81      -6.674  -6.537   2.405  1.00 26.59           H  0.087
ATOM   1231  HB3 SER A  81      -7.761  -7.104   3.416  1.00 26.59           H  0.087
ATOM   1232  HG  SER A  81      -5.428  -6.155   4.187  1.00 25.95           H  0.086
ATOM   1233  N   ALA A  82      -3.741  -8.195   3.616  1.00 18.99           N  0.074
ATOM   1234  CA  ALA A  82      -2.341  -8.163   3.190  1.00 22.06           C  0.080
ATOM   1235  C   ALA A  82      -1.963  -9.393   2.388  1.00 26.14           C  0.087
ATOM   1236  O   ALA A  82      -0.973  -9.372   1.661  1.00 23.00           O  0.081
ATOM   1237  CB  ALA A  82      -1.393  -8.038   4.385  1.00 30.74           C  0.094
ATOM   1238  H   ALA A  82      -3.859  -8.195   4.467  1.00 22.79           H  0.081
ATOM   1239  HA  ALA A  82      -2.223  -7.374   2.639  1.00 26.47           H  0.087
ATOM   1240  HB1 ALA A  82      -0.478  -8.016   4.062  1.00 36.89           H  0.103
ATOM   1241  HB2 ALA A  82      -1.594  -7.219   4.864  1.00 36.89           H  0.103
ATOM   1242  HB3 ALA A  82      -1.519  -8.803   4.970  1.00 36.89           H  0.103
ATOM   1243  N   LEU A  83      -2.744 -10.449   2.479  1.00 21.48           N  0.078
ATOM   1244  CA  LEU A  83      -2.425 -11.685   1.778  1.00 23.23           C  0.082
ATOM   1245  C   LEU A  83      -2.965 -11.707   0.375  1.00 23.97           C  0.083
ATOM   1246  O   LEU A  83      -2.804 -12.727  -0.307  1.00 25.19           O  0.085
ATOM   1247  CB  LEU A  83      -2.962 -12.875   2.562  1.00 21.54           C  0.079
ATOM   1248  CG  LEU A  83      -2.445 -12.903   4.009  1.00 20.61           C  0.077
ATOM   1249  CD1 LEU A  83      -3.062 -14.013   4.831  1.00 22.04           C  0.079
ATOM   1250  CD2 LEU A  83      -0.904 -12.992   3.978  1.00 31.07           C  0.094
ATOM   1251  H   LEU A  83      -3.469 -10.480   2.940  1.00 25.77           H  0.086
ATOM   1252  HA  LEU A  83      -1.461 -11.781   1.723  1.00 27.87           H  0.089
ATOM   1253  HB2 LEU A  83      -3.931 -12.827   2.587  1.00 25.85           H  0.086
ATOM   1254  HB3 LEU A  83      -2.683 -13.695   2.125  1.00 25.85           H  0.086
ATOM   1255  HG  LEU A  83      -2.710 -12.086   4.461  1.00 24.73           H  0.084
ATOM   1256 HD11 LEU A  83      -2.715 -13.967   5.735  1.00 26.45           H  0.087
ATOM   1257 HD12 LEU A  83      -4.026 -13.901   4.840  1.00 26.45           H  0.087
ATOM   1258 HD13 LEU A  83      -2.832 -14.867   4.431  1.00 26.45           H  0.087
ATOM   1259 HD21 LEU A  83      -0.605 -13.536   4.723  1.00 37.29           H  0.103
ATOM   1260 HD22 LEU A  83      -0.629 -13.397   3.141  1.00 37.29           H  0.103
ATOM   1261 HD23 LEU A  83      -0.535 -12.098   4.051  1.00 37.29           H  0.103
ATOM   1262  N   LEU A  84      -3.620 -10.636  -0.062  1.00 18.38           N  0.073
ATOM   1263  CA  LEU A  84      -4.160 -10.602  -1.401  1.00 23.08           C  0.081
ATOM   1264  C   LEU A  84      -3.341  -9.700  -2.315  1.00 21.15           C  0.078
ATOM   1265  O   LEU A  84      -3.742  -9.486  -3.466  1.00 19.53           O  0.075
ATOM   1266  CB  LEU A  84      -5.608 -10.141  -1.363  1.00 21.62           C  0.079
ATOM   1267  CG  LEU A  84      -6.553 -10.851  -0.415  1.00 23.52           C  0.082
ATOM   1268  CD1 LEU A  84      -7.874 -10.125  -0.414  1.00 28.08           C  0.090
ATOM   1269  CD2 LEU A  84      -6.781 -12.294  -0.837  1.00 24.78           C  0.084
ATOM   1270  H   LEU A  84      -3.760  -9.925   0.401  1.00 22.05           H  0.079
ATOM   1271  HA  LEU A  84      -4.155 -11.495  -1.779  1.00 27.69           H  0.089
ATOM   1272  HB2 LEU A  84      -5.613  -9.204  -1.112  1.00 25.94           H  0.086
ATOM   1273  HB3 LEU A  84      -5.975 -10.247  -2.255  1.00 25.94           H  0.086
ATOM   1274  HG  LEU A  84      -6.165 -10.858   0.474  1.00 28.22           H  0.090
ATOM   1275 HD11 LEU A  84      -8.494 -10.599   0.161  1.00 33.70           H  0.098
ATOM   1276 HD12 LEU A  84      -7.738  -9.224  -0.083  1.00 33.70           H  0.098
ATOM   1277 HD13 LEU A  84      -8.219 -10.097  -1.320  1.00 33.70           H  0.098
```

```
ATOM   1278 HD21 LEU A  84      -7.366 -12.723  -0.193  1.00 29.74           H  0.092
ATOM   1279 HD22 LEU A  84      -7.191 -12.306  -1.716  1.00 29.74           H  0.092
ATOM   1280 HD23 LEU A  84      -5.927 -12.754  -0.864  1.00 29.74           H  0.092
ATOM   1281  N   SER A  85      -2.240  -9.160  -1.816  1.00 21.81           N  0.079
ATOM   1282  CA  SER A  85      -1.418  -8.215  -2.541  1.00 25.19           C  0.085
ATOM   1283  C   SER A  85      -0.743  -8.843  -3.761  1.00 23.03           C  0.081
ATOM   1284  O   SER A  85      -0.414 -10.031  -3.782  1.00 20.28           O  0.076
ATOM   1285  CB  SER A  85      -0.329  -7.665  -1.631  1.00 24.87           C  0.084
ATOM   1286  OG  SER A  85       0.564  -6.889  -2.417  1.00 34.29           O  0.099
ATOM   1287  H   SER A  85      -1.937  -9.333  -1.029  1.00 26.17           H  0.087
ATOM   1288  HA  SER A  85      -1.994  -7.498  -2.850  1.00 30.22           H  0.093
ATOM   1289  HB2 SER A  85      -0.729  -7.107  -0.946  1.00 29.84           H  0.092
ATOM   1290  HB3 SER A  85       0.153  -8.400  -1.221  1.00 29.84           H  0.092
ATOM   1291  HG  SER A  85       0.145  -6.272  -2.804  1.00 41.15           H  0.109
ATOM   1292  N   SER A  86      -0.462  -8.001  -4.767  1.00 22.98           N  0.081
ATOM   1293  CA  SER A  86       0.424  -8.453  -5.833  1.00 22.26           C  0.080
ATOM   1294  C   SER A  86       1.741  -8.941  -5.258  1.00 24.64           C  0.084
ATOM   1295  O   SER A  86       2.332  -9.909  -5.758  1.00 31.37           O  0.095
ATOM   1296  CB  SER A  86       0.701  -7.314  -6.801  1.00 29.80           C  0.092
ATOM   1297  OG  SER A  86      -0.470  -6.980  -7.491  1.00 33.94           O  0.099
ATOM   1298  H   SER A  86      -0.761  -7.199  -4.847  1.00 27.58           H  0.089
ATOM   1299  HA  SER A  86      -0.012  -9.174  -6.314  1.00 26.71           H  0.087
ATOM   1300  HB2 SER A  86       1.011  -6.540  -6.304  1.00 35.76           H  0.101
ATOM   1301  HB3 SER A  86       1.379  -7.592  -7.437  1.00 35.76           H  0.101
ATOM   1302  HG  SER A  86      -1.092  -6.831  -6.947  1.00 40.73           H  0.108
ATOM   1303  N   ASP A  87       2.243  -8.233  -4.255  1.00 25.75           N  0.086
ATOM   1304  CA  ASP A  87       3.507  -8.546  -3.619  1.00 24.71           C  0.084
ATOM   1305  C   ASP A  87       3.249  -9.655  -2.625  1.00 24.18           C  0.083
ATOM   1306  O   ASP A  87       2.473  -9.473  -1.687  1.00 24.52           O  0.084
ATOM   1307  CB  ASP A  87       4.070  -7.342  -2.878  1.00 36.06           C  0.102
ATOM   1308  CG  ASP A  87       5.515  -7.547  -2.467  1.00 39.81           C  0.107
ATOM   1309  OD1 ASP A  87       5.789  -8.425  -1.631  1.00 30.54           O  0.094
ATOM   1310  OD2 ASP A  87       6.388  -6.882  -3.074  1.00 44.67           O  0.113
ATOM   1311  H   ASP A  87       1.856  -7.544  -3.916  1.00 30.90           H  0.094
ATOM   1312  HA  ASP A  87       4.152  -8.814  -4.292  1.00 29.65           H  0.092
ATOM   1313  HB2 ASP A  87       4.028  -6.565  -3.456  1.00 43.27           H  0.111
ATOM   1314  HB3 ASP A  87       3.547  -7.189  -2.076  1.00 43.27           H  0.111
ATOM   1315  N   ILE A  88       3.942 -10.786  -2.818  1.00 30.57           N  0.094
ATOM   1316  CA  ILE A  88       3.669 -11.993  -2.057  1.00 23.53           C  0.082
ATOM   1317  C   ILE A  88       4.412 -12.041  -0.763  1.00 26.00           C  0.086
ATOM   1318  O   ILE A  88       4.341 -13.065  -0.053  1.00 26.29           O  0.087
ATOM   1319  CB  ILE A  88       4.024 -13.211  -2.920  1.00 19.74           C  0.075
ATOM   1320  CG1 ILE A  88       5.541 -13.376  -3.082  1.00 25.49           C  0.085
ATOM   1321  CG2 ILE A  88       3.326 -13.105  -4.303  1.00 24.46           C  0.084
ATOM   1322  CD1 ILE A  88       5.921 -14.753  -3.680  1.00 25.65           C  0.086
ATOM   1323  H   ILE A  88       4.579 -10.872  -3.389  1.00 36.68           H  0.103
ATOM   1324  HA  ILE A  88       2.726 -12.019  -1.831  1.00 28.23           H  0.090
ATOM   1325  HB  ILE A  88       3.701 -14.002  -2.461  1.00 23.68           H  0.082
ATOM   1326 HG12 ILE A  88       5.874 -12.686  -3.677  1.00 30.59           H  0.094
ATOM   1327 HG13 ILE A  88       5.964 -13.297  -2.212  1.00 30.59           H  0.094
ATOM   1328 HG21 ILE A  88       3.400 -13.958  -4.759  1.00 29.36           H  0.092
ATOM   1329 HG22 ILE A  88       2.392 -12.881  -4.169  1.00 29.36           H  0.092
ATOM   1330 HG23 ILE A  88       3.762 -12.412  -4.823  1.00 29.36           H  0.092
ATOM   1331 HD11 ILE A  88       6.887 -14.833  -3.699  1.00 30.78           H  0.094
ATOM   1332 HD12 ILE A  88       5.542 -15.454  -3.125  1.00 30.78           H  0.094
ATOM   1333 HD13 ILE A  88       5.565 -14.816  -4.580  1.00 30.78           H  0.094
ATOM   1334  N   THR A  89       5.123 -10.978  -0.399  1.00 26.24           N  0.087
ATOM   1335  CA  THR A  89       5.926 -11.026   0.800  1.00 25.75           C  0.086
ATOM   1336  C   THR A  89       5.104 -11.449   2.022  1.00 23.85           C  0.083
ATOM   1337  O   THR A  89       5.523 -12.324   2.763  1.00 24.22           O  0.083
ATOM   1338  CB  THR A  89       6.623  -9.681   1.067  1.00 29.43           C  0.092
ATOM   1339  OG1 THR A  89       7.511  -9.391  -0.023  1.00 36.12           O  0.102
ATOM   1340  CG2 THR A  89       7.352  -9.742   2.412  1.00 32.85           C  0.097
ATOM   1341  H   THR A  89       5.152 -10.233  -0.826  1.00 31.49           H  0.095
ATOM   1342  HA  THR A  89       6.619 -11.689   0.653  1.00 30.90           H  0.094
ATOM   1343  HB  THR A  89       5.985  -8.952   1.125  1.00 35.32           H  0.101
ATOM   1344  HG1 THR A  89       7.854  -8.631   0.081  1.00 43.35           H  0.111
ATOM   1345 HG21 THR A  89       7.811  -8.904   2.579  1.00 39.42           H  0.106
ATOM   1346 HG22 THR A  89       6.717  -9.901   3.127  1.00 39.42           H  0.106
ATOM   1347 HG23 THR A  89       8.003 -10.461   2.404  1.00 39.42           H  0.106
ATOM   1348  N   ALA A  90       3.922 -10.857   2.235  1.00 25.23           N  0.085
ATOM   1349  CA  ALA A  90       3.142 -11.206   3.427  1.00 25.20           C  0.085
ATOM   1350  C   ALA A  90       2.748 -12.681   3.410  1.00 20.34           C  0.076
ATOM   1351  O   ALA A  90       2.862 -13.376   4.426  1.00 19.03           O  0.074
ATOM   1352  CB  ALA A  90       1.886 -10.336   3.544  1.00 27.70           C  0.089
ATOM   1353  H   ALA A  90       3.560 -10.271   1.720  1.00 30.27           H  0.093
ATOM   1354  HA  ALA A  90       3.695 -11.031   4.205  1.00 30.25           H  0.093
ATOM   1355  HB1 ALA A  90       1.366 -10.634   4.306  1.00 33.24           H  0.098
```

```
ATOM   1356  HB2 ALA A  90       2.152  -9.411   3.666  1.00 33.24           H  0.098
ATOM   1357  HB3 ALA A  90       1.363 -10.425   2.732  1.00 33.24           H  0.098
ATOM   1358  N   SER A  91       2.287 -13.169   2.255  1.00 23.63           N  0.082
ATOM   1359  CA  SER A  91       1.885 -14.570   2.136  1.00 18.81           C  0.073
ATOM   1360  C   SER A  91       3.067 -15.488   2.381  1.00 21.01           C  0.078
ATOM   1361  O   SER A  91       2.941 -16.496   3.088  1.00 18.57           O  0.073
ATOM   1362  CB  SER A  91       1.250 -14.814   0.776  1.00 20.34           C  0.076
ATOM   1363  OG  SER A  91      -0.045 -14.236   0.821  1.00 17.72           O  0.071
ATOM   1364  H   SER A  91       2.198 -12.713   1.531  1.00 28.35           H  0.090
ATOM   1365  HA  SER A  91       1.212 -14.776   2.804  1.00 22.57           H  0.080
ATOM   1366  HB2 SER A  91       1.779 -14.390   0.082  1.00 24.40           H  0.084
ATOM   1367  HB3 SER A  91       1.183 -15.766   0.606  1.00 24.40           H  0.084
ATOM   1368  HG  SER A  91      -0.471 -14.430   0.124  1.00 21.26           H  0.078
ATOM   1369  N   VAL A  92       4.242 -15.139   1.862  1.00 19.11           N  0.074
ATOM   1370  CA  VAL A  92       5.408 -15.991   2.087  1.00 19.66           C  0.075
ATOM   1371  C   VAL A  92       5.814 -15.993   3.567  1.00 19.17           C  0.074
ATOM   1372  O   VAL A  92       6.169 -17.033   4.147  1.00 19.59           O  0.075
ATOM   1373  CB  VAL A  92       6.589 -15.523   1.209  1.00 20.15           C  0.076
ATOM   1374  CG1 VAL A  92       7.900 -16.319   1.656  1.00 19.82           C  0.075
ATOM   1375  CG2 VAL A  92       6.236 -15.746  -0.177  1.00 22.88           C  0.081
ATOM   1376  H   VAL A  92       4.387 -14.435   1.390  1.00 22.94           H  0.081
ATOM   1377  HA  VAL A  92       5.165 -16.897   1.837  1.00 23.59           H  0.082
ATOM   1378  HB  VAL A  92       6.779 -14.577   1.312  1.00 24.18           H  0.083
ATOM   1379 HG11 VAL A  92       8.505 -16.381   0.900  1.00 23.78           H  0.083
ATOM   1380 HG12 VAL A  92       8.328 -15.840   2.383  1.00 23.78           H  0.083
ATOM   1381 HG13 VAL A  92       7.646 -17.207   1.950  1.00 23.78           H  0.083
ATOM   1382 HG21 VAL A  92       6.955 -15.422  -0.742  1.00 27.46           H  0.089
ATOM   1383 HG22 VAL A  92       6.104 -16.696  -0.320  1.00 27.46           H  0.089
ATOM   1384 HG23 VAL A  92       5.417 -15.265  -0.376  1.00 27.46           H  0.089
ATOM   1385  N   ASN A  93       5.846 -14.814   4.177  1.00 24.67           N  0.084
ATOM   1386  CA  ASN A  93       6.276 -14.732   5.569  1.00 25.50           C  0.085
ATOM   1387  C   ASN A  93       5.371 -15.550   6.457  1.00 23.45           C  0.082
ATOM   1388  O   ASN A  93       5.836 -16.189   7.411  1.00 25.20           O  0.085
ATOM   1389  CB  ASN A  93       6.295 -13.284   6.076  1.00 28.57           C  0.090
ATOM   1390  CG  ASN A  93       7.503 -12.468   5.563  1.00 31.90           C  0.096
ATOM   1391  OD1 ASN A  93       8.444 -13.019   4.999  1.00 34.73           O  0.100
ATOM   1392  ND2 ASN A  93       7.435 -11.133   5.723  1.00 36.15           N  0.102
ATOM   1393  H   ASN A  93       5.629 -14.064   3.817  1.00 29.61           H  0.092
ATOM   1394  HA  ASN A  93       7.183 -15.073   5.611  1.00 30.60           H  0.094
ATOM   1395  HB2 ASN A  93       5.487 -12.838   5.779  1.00 34.29           H  0.099
ATOM   1396  HB3 ASN A  93       6.332 -13.292   7.045  1.00 34.29           H  0.099
ATOM   1397 HD21 ASN A  93       8.082 -10.634   5.454  1.00 43.38           H  0.111
ATOM   1398 HD22 ASN A  93       6.744 -10.779   6.094  1.00 43.38           H  0.111
ATOM   1399  N   CYS A  94       4.065 -15.518   6.171  1.00 21.43           N  0.078
ATOM   1400  CA  CYS A  94       3.092 -16.212   6.980  1.00 18.76           C  0.073
ATOM   1401  C   CYS A  94       3.182 -17.704   6.749  1.00 19.25           C  0.074
ATOM   1402  O   CYS A  94       3.187 -18.485   7.703  1.00 18.28           O  0.072
ATOM   1403  CB  CYS A  94       1.692 -15.673   6.659  1.00 23.79           C  0.083
ATOM   1404  SG  CYS A  94       0.380 -16.258   7.810  1.00 21.04           S  0.078
ATOM   1405  H   CYS A  94       3.724 -15.094   5.504  1.00 25.72           H  0.086
ATOM   1406  HA  CYS A  94       3.263 -16.051   7.921  1.00 22.52           H  0.080
ATOM   1407  HB2 CYS A  94       1.714 -14.704   6.705  1.00 28.55           H  0.090
ATOM   1408  HB3 CYS A  94       1.447 -15.957   5.764  1.00 28.55           H  0.090
ATOM   1409  N   ALA A  95       3.233 -18.108   5.487  1.00 15.87           N  0.067
ATOM   1410  CA  ALA A  95       3.513 -19.493   5.128  1.00 18.63           C  0.073
ATOM   1411  C   ALA A  95       4.696 -20.069   5.855  1.00 17.68           C  0.071
ATOM   1412  O   ALA A  95       4.727 -21.269   6.154  1.00 17.26           O  0.070
ATOM   1413  CB  ALA A  95       3.768 -19.590   3.646  1.00 17.90           C  0.072
ATOM   1414  H   ALA A  95       3.109 -17.594   4.809  1.00 19.04           H  0.074
ATOM   1415  HA  ALA A  95       2.731 -20.014   5.372  1.00 22.36           H  0.080
ATOM   1416  HB1 ALA A  95       3.948 -20.515   3.416  1.00 21.48           H  0.078
ATOM   1417  HB2 ALA A  95       2.983 -19.278   3.169  1.00 21.48           H  0.078
ATOM   1418  HB3 ALA A  95       4.533 -19.037   3.421  1.00 21.48           H  0.078
ATOM   1419  N   LYS A  96       5.751 -19.269   6.006  1.00 22.43           N  0.080
ATOM   1420  CA  LYS A  96       6.901 -19.742   6.752  1.00 19.06           C  0.074
ATOM   1421  C   LYS A  96       6.489 -20.123   8.150  1.00 19.96           C  0.076
ATOM   1422  O   LYS A  96       6.977 -21.100   8.698  1.00 20.97           O  0.078
ATOM   1423  CB  LYS A  96       7.991 -18.671   6.764  1.00 23.19           C  0.082
ATOM   1424  CG  LYS A  96       8.765 -18.625   5.409  1.00 23.79           C  0.083
ATOM   1425  CD  LYS A  96       9.869 -17.581   5.421  1.00 26.02           C  0.086
ATOM   1426  CE  LYS A  96      10.493 -17.534   4.065  1.00 25.09           C  0.085
ATOM   1427  NZ  LYS A  96      11.400 -16.383   3.920  1.00 31.36           N  0.095
ATOM   1428  H   LYS A  96       5.819 -18.471   5.693  1.00 26.92           H  0.088
ATOM   1429  HA  LYS A  96       7.275 -20.526   6.322  1.00 22.87           H  0.081
ATOM   1430  HB2 LYS A  96       7.586 -17.803   6.914  1.00 27.82           H  0.089
ATOM   1431  HB3 LYS A  96       8.625 -18.868   7.471  1.00 27.82           H  0.089
ATOM   1432  HG2 LYS A  96       9.169 -19.491   5.241  1.00 28.55           H  0.090
ATOM   1433  HG3 LYS A  96       8.147 -18.403   4.695  1.00 28.55           H  0.090
```

```
ATOM   1434  HD2 LYS A  96       9.500 -16.709   5.631  1.00 31.22           H  0.095
ATOM   1435  HD3 LYS A  96      10.546 -17.820   6.074  1.00 31.22           H  0.095
ATOM   1436  HE2 LYS A  96      11.006 -18.345   3.921  1.00 30.10           H  0.093
ATOM   1437  HE3 LYS A  96       9.797 -17.459   3.394  1.00 30.10           H  0.093
ATOM   1438  HZ1 LYS A  96      11.742 -16.366   3.099  1.00 37.64           H  0.104
ATOM   1439  HZ2 LYS A  96      10.955 -15.625   4.063  1.00 37.64           H  0.104
ATOM   1440  HZ3 LYS A  96      12.065 -16.443   4.509  1.00 37.64           H  0.104
ATOM   1441  N   LYS A  97       5.635 -19.322   8.763  1.00 20.97           N  0.078
ATOM   1442  CA  LYS A  97       5.231 -19.619  10.126  1.00 18.34           C  0.072
ATOM   1443  C   LYS A  97       4.348 -20.848  10.129  1.00 21.01           C  0.078
ATOM   1444  O   LYS A  97       4.512 -21.728  10.961  1.00 19.09           O  0.074
ATOM   1445  CB  LYS A  97       4.522 -18.421  10.738  1.00 23.57           C  0.082
ATOM   1446  CG  LYS A  97       5.485 -17.404  11.351  1.00 42.29           C  0.110
ATOM   1447  CD  LYS A  97       4.805 -16.066  11.661  1.00 43.80           C  0.112
ATOM   1448  CE  LYS A  97       4.859 -15.766  13.176  1.00 50.03           C  0.120
ATOM   1449  NZ  LYS A  97       5.302 -14.370  13.441  1.00 51.72           N  0.122
ATOM   1450  H   LYS A  97       5.281 -18.616   8.422  1.00 25.16           H  0.085
ATOM   1451  HA  LYS A  97       6.009 -19.792  10.679  1.00 22.01           H  0.079
ATOM   1452  HB2 LYS A  97       4.012 -17.971  10.047  1.00 28.28           H  0.090
ATOM   1453  HB3 LYS A  97       3.928 -18.732  11.440  1.00 28.28           H  0.090
ATOM   1454  HG2 LYS A  97       5.838 -17.761  12.181  1.00 50.75           H  0.121
ATOM   1455  HG3 LYS A  97       6.208 -17.236  10.727  1.00 50.75           H  0.121
ATOM   1456  HD2 LYS A  97       5.262 -15.353  11.189  1.00 52.56           H  0.123
ATOM   1457  HD3 LYS A  97       3.875 -16.103  11.387  1.00 52.56           H  0.123
ATOM   1458  HE2 LYS A  97       3.975 -15.883  13.557  1.00 60.03           H  0.131
ATOM   1459  HE3 LYS A  97       5.486 -16.372  13.601  1.00 60.03           H  0.131
ATOM   1460  HZ1 LYS A  97       5.355 -14.229  14.318  1.00 62.06           H  0.133
ATOM   1461  HZ2 LYS A  97       6.102 -14.230  13.077  1.00 62.06           H  0.133
ATOM   1462  HZ3 LYS A  97       4.721 -13.794  13.090  1.00 62.06           H  0.133
ATOM   1463  N   ILE A  98       3.442 -20.920   9.152  1.00 19.21           N  0.074
ATOM   1464  CA  ILE A  98       2.532 -22.044   9.025  1.00 15.76           C  0.067
ATOM   1465  C   ILE A  98       3.302 -23.330   8.831  1.00 18.15           C  0.072
ATOM   1466  O   ILE A  98       3.027 -24.324   9.488  1.00 18.84           O  0.073
ATOM   1467  CB  ILE A  98       1.526 -21.839   7.881  1.00 18.11           C  0.072
ATOM   1468  CG1 ILE A  98       0.714 -20.608   8.149  1.00 16.19           C  0.068
ATOM   1469  CG2 ILE A  98       0.640 -23.076   7.817  1.00 14.05           C  0.063
ATOM   1470  CD1 ILE A  98      -0.126 -20.126   6.925  1.00 18.42           C  0.073
ATOM   1471  H   ILE A  98       3.337 -20.320   8.544  1.00 23.05           H  0.081
ATOM   1472  HA  ILE A  98       2.031 -22.099   9.853  1.00 18.91           H  0.074
ATOM   1473  HB  ILE A  98       1.980 -21.718   7.033  1.00 21.73           H  0.079
ATOM   1474 HG12 ILE A  98       0.099 -20.793   8.876  1.00 19.43           H  0.075
ATOM   1475 HG13 ILE A  98       1.314 -19.888   8.398  1.00 19.43           H  0.075
ATOM   1476 HG21 ILE A  98      -0.109 -22.898   7.227  1.00 16.86           H  0.070
ATOM   1477 HG22 ILE A  98       1.161 -23.820   7.475  1.00 16.86           H  0.070
ATOM   1478 HG23 ILE A  98       0.317 -23.281   8.708  1.00 16.86           H  0.070
ATOM   1479 HD11 ILE A  98      -0.500 -19.253   7.123  1.00 22.11           H  0.080
ATOM   1480 HD12 ILE A  98       0.451 -20.070   6.148  1.00 22.11           H  0.080
ATOM   1481 HD13 ILE A  98      -0.840 -20.762   6.762  1.00 22.11           H  0.080
ATOM   1482  N   VAL A  99       4.257 -23.340   7.904  1.00 16.44           N  0.069
ATOM   1483  CA  VAL A  99       4.954 -24.585   7.639  1.00 15.72           C  0.067
ATOM   1484  C   VAL A  99       5.856 -24.970   8.783  1.00 21.12           C  0.078
ATOM   1485  O   VAL A  99       6.233 -26.145   8.874  1.00 19.96           O  0.076
ATOM   1486  CB  VAL A  99       5.656 -24.440   6.271  1.00 17.62           C  0.071
ATOM   1487  CG1 VAL A  99       6.922 -23.505   6.373  1.00 15.99           C  0.068
ATOM   1488  CG2 VAL A  99       5.967 -25.858   5.720  1.00 18.03           C  0.072
ATOM   1489  H   VAL A  99       4.508 -22.664   7.436  1.00 19.73           H  0.075
ATOM   1490  HA  VAL A  99       4.355 -25.343   7.555  1.00 18.86           H  0.074
ATOM   1491  HB  VAL A  99       5.076 -23.999   5.631  1.00 21.14           H  0.078
ATOM   1492 HG11 VAL A  99       7.234 -23.297   5.479  1.00 19.19           H  0.074
ATOM   1493 HG12 VAL A  99       6.675 -22.689   6.836  1.00 19.19           H  0.074
ATOM   1494 HG13 VAL A  99       7.617 -23.967   6.867  1.00 19.19           H  0.074
ATOM   1495 HG21 VAL A  99       6.462 -25.773   4.890  1.00 21.64           H  0.079
ATOM   1496 HG22 VAL A  99       6.496 -26.341   6.373  1.00 21.64           H  0.079
ATOM   1497 HG23 VAL A  99       5.132 -26.325   5.561  1.00 21.64           H  0.079
ATOM   1498  N   SER A 100       6.107 -24.053   9.713  1.00 18.27           N  0.072
ATOM   1499  CA  SER A 100       6.973 -24.338  10.867  1.00 25.42           C  0.085
ATOM   1500  C   SER A 100       6.218 -24.888  12.051  1.00 24.45           C  0.084
ATOM   1501  O   SER A 100       6.817 -25.209  13.087  1.00 20.19           O  0.076
ATOM   1502  CB  SER A 100       7.697 -23.085  11.275  1.00 22.81           C  0.081
ATOM   1503  OG  SER A 100       8.558 -22.754  10.204  1.00 23.49           O  0.082
ATOM   1504  H   SER A 100       5.789 -23.255   9.702  1.00 21.93           H  0.079
ATOM   1505  HA  SER A 100       7.631 -25.000  10.604  1.00 30.51           H  0.094
ATOM   1506  HB2 SER A 100       7.062 -22.367  11.427  1.00 27.37           H  0.089
ATOM   1507  HB3 SER A 100       8.213 -23.246  12.081  1.00 27.37           H  0.089
ATOM   1508  HG  SER A 100       8.135 -22.772   9.479  1.00 28.19           H  0.090
ATOM   1509  N   ASP A 101       4.897 -24.894  11.990  1.00 28.37           N  0.090
ATOM   1510  CA  ASP A 101       4.050 -25.183  13.176  1.00 28.16           C  0.090
ATOM   1511  C   ASP A 101       3.965 -26.651  13.587  1.00 30.05           C  0.093
```

ATOM	1512	O	ASP	A	101	3.288	-26.891	14.583	1.00	30.32	O	0.093
ATOM	1513	CB	ASP	A	101	2.627	-24.686	12.949	1.00	35.71	C	0.101
ATOM	1514	CG	ASP	A	101	1.876	-24.427	14.228	1.00	41.64	C	0.109
ATOM	1515	OD1	ASP	A	101	2.417	-23.713	15.068	1.00	43.02	O	0.111
ATOM	1516	OD2	ASP	A	101	0.790	-24.982	14.367	1.00	47.99	O	0.117
ATOM	1517	H	ASP	A	101	4.364	-24.700	11.123	1.00	34.04	H	0.099
ATOM	1518	HA	ASP	A	101	4.463	-24.626	14.015	1.00	33.79	H	0.098
ATOM	1519	HB2	ASP	A	101	2.661	-23.761	12.373	1.00	42.85	H	0.111
ATOM	1520	HB3	ASP	A	101	2.081	-25.428	12.369	1.00	42.85	H	0.111
ATOM	1521	N	GLY	A	102	4.595	-27.580	12.877	1.00	27.79	N	0.089
ATOM	1522	CA	GLY	A	102	4.631	-28.972	13.311	1.00	29.57	C	0.092
ATOM	1523	C	GLY	A	102	4.119	-29.973	12.292	1.00	21.11	C	0.078
ATOM	1524	O	GLY	A	102	4.530	-31.130	12.334	1.00	26.25	O	0.087
ATOM	1525	H	GLY	A	102	5.033	-27.440	12.150	1.00	33.34	H	0.098
ATOM	1526	HA2	GLY	A	102	5.548	-29.208	13.522	1.00	35.48	H	0.101
ATOM	1527	HA3	GLY	A	102	4.090	-29.062	14.111	1.00	35.48	H	0.101
ATOM	1528	N	ASN	A	103	3.250	-29.568	11.353	1.00	17.29	N	0.070
ATOM	1529	CA	ASN	A	103	2.732	-30.526	10.376	1.00	17.36	C	0.071
ATOM	1530	C	ASN	A	103	3.281	-30.228	8.985	1.00	13.29	C	0.062
ATOM	1531	O	ASN	A	103	2.896	-30.892	8.018	1.00	15.27	O	0.066
ATOM	1532	CB	ASN	A	103	1.217	-30.530	10.417	1.00	19.77	C	0.075
ATOM	1533	CG	ASN	A	103	0.671	-31.014	11.762	1.00	28.27	C	0.090
ATOM	1534	OD1	ASN	A	103	-0.176	-30.378	12.366	1.00	31.97	O	0.096
ATOM	1535	ND2	ASN	A	103	1.179	-32.140	12.234	1.00	24.74	N	0.084
ATOM	1536	H	ASN	A	103	2.957	-28.764	11.267	1.00	20.75	H	0.077
ATOM	1537	HA	ASN	A	103	3.004	-31.429	10.601	1.00	20.83	H	0.077
ATOM	1538	HB2	ASN	A	103	0.893	-29.628	10.267	1.00	23.72	H	0.082
ATOM	1539	HB3	ASN	A	103	0.883	-31.122	9.725	1.00	23.72	H	0.082
ATOM	1540	HD21	ASN	A	103	0.904	-32.453	12.987	1.00	29.68	H	0.092
ATOM	1541	HD22	ASN	A	103	1.784	-32.559	11.789	1.00	29.68	H	0.092
ATOM	1542	N	GLY	A	104	4.273	-29.361	8.912	1.00	14.46	N	0.064
ATOM	1543	CA	GLY	A	104	4.893	-29.087	7.636	1.00	16.62	C	0.069
ATOM	1544	C	GLY	A	104	3.837	-28.642	6.673	1.00	12.48	C	0.060
ATOM	1545	O	GLY	A	104	2.882	-27.961	7.044	1.00	13.76	O	0.063
ATOM	1546	H	GLY	A	104	4.601	-28.925	9.577	1.00	17.35	H	0.071
ATOM	1547	HA2	GLY	A	104	5.557	-28.387	7.730	1.00	19.94	H	0.076
ATOM	1548	HA3	GLY	A	104	5.322	-29.887	7.294	1.00	19.94	H	0.076
ATOM	1549	N	MET	A	105	3.996	-29.035	5.416	1.00	13.61	N	0.062
ATOM	1550	CA	MET	A	105	3.066	-28.531	4.427	1.00	18.61	C	0.073
ATOM	1551	C	MET	A	105	1.712	-29.237	4.438	1.00	14.89	C	0.065
ATOM	1552	O	MET	A	105	0.840	-28.841	3.660	1.00	14.30	O	0.064
ATOM	1553	CB	MET	A	105	3.657	-28.629	3.045	1.00	15.89	C	0.067
ATOM	1554	CG	MET	A	105	4.647	-27.485	2.823	1.00	13.06	C	0.061
ATOM	1555	SD	MET	A	105	5.198	-27.436	1.081	1.00	16.83	S	0.069
ATOM	1556	CE	MET	A	105	3.883	-26.562	0.394	1.00	13.60	C	0.062
ATOM	1557	H	MET	A	105	4.607	-29.569	5.129	1.00	16.33	H	0.068
ATOM	1558	HA	MET	A	105	2.924	-27.592	4.625	1.00	22.33	H	0.080
ATOM	1559	HB2	MET	A	105	4.127	-29.472	2.950	1.00	19.07	H	0.074
ATOM	1560	HB3	MET	A	105	2.952	-28.565	2.383	1.00	19.07	H	0.074
ATOM	1561	HG2	MET	A	105	4.219	-26.641	3.035	1.00	15.67	H	0.067
ATOM	1562	HG3	MET	A	105	5.424	-27.615	3.389	1.00	15.67	H	0.067
ATOM	1563	HE1	MET	A	105	4.021	-26.484	-0.562	1.00	16.33	H	0.068
ATOM	1564	HE2	MET	A	105	3.059	-27.042	0.571	1.00	16.33	H	0.068
ATOM	1565	HE3	MET	A	105	3.846	-25.680	0.796	1.00	16.33	H	0.068
ATOM	1566	N	ASN	A	106	1.498	-30.217	5.344	1.00	16.03	N	0.068
ATOM	1567	CA	ASN	A	106	0.191	-30.848	5.475	1.00	13.48	C	0.062
ATOM	1568	C	ASN	A	106	-0.880	-29.868	5.965	1.00	13.28	C	0.062
ATOM	1569	O	ASN	A	106	-2.076	-30.149	5.842	1.00	16.91	O	0.070
ATOM	1570	CB	ASN	A	106	0.263	-32.002	6.435	1.00	17.30	C	0.070
ATOM	1571	CG	ASN	A	106	1.069	-33.139	5.884	1.00	14.24	C	0.064
ATOM	1572	OD1	ASN	A	106	0.696	-33.793	4.898	1.00	18.16	O	0.072
ATOM	1573	ND2	ASN	A	106	2.221	-33.326	6.460	1.00	17.55	N	0.071
ATOM	1574	H	ASN	A	106	2.093	-30.522	5.884	1.00	19.23	H	0.074
ATOM	1575	HA	ASN	A	106	-0.070	-31.185	4.604	1.00	16.18	H	0.068
ATOM	1576	HB2	ASN	A	106	0.679	-31.706	7.260	1.00	20.76	H	0.077
ATOM	1577	HB3	ASN	A	106	-0.634	-32.325	6.614	1.00	20.76	H	0.077
ATOM	1578	HD21	ASN	A	106	2.736	-33.961	6.194	1.00	21.06	H	0.078
ATOM	1579	HD22	ASN	A	106	2.467	-32.814	7.106	1.00	21.06	H	0.078
ATOM	1580	N	ALA	A	107	-0.451	-28.736	6.488	1.00	12.59	N	0.060
ATOM	1581	CA	ALA	A	107	-1.342	-27.646	6.822	1.00	16.15	C	0.068
ATOM	1582	C	ALA	A	107	-2.104	-27.148	5.609	1.00	14.91	C	0.065
ATOM	1583	O	ALA	A	107	-3.114	-26.444	5.753	1.00	16.94	O	0.070
ATOM	1584	CB	ALA	A	107	-0.493	-26.536	7.438	1.00	16.98	C	0.070
ATOM	1585	H	ALA	A	107	0.374	-28.568	6.665	1.00	15.11	H	0.066
ATOM	1586	HA	ALA	A	107	-2.010	-27.924	7.468	1.00	19.38	H	0.075
ATOM	1587	HB1	ALA	A	107	-1.067	-25.788	7.663	1.00	20.37	H	0.076
ATOM	1588	HB2	ALA	A	107	-0.064	-26.876	8.239	1.00	20.37	H	0.076
ATOM	1589	HB3	ALA	A	107	0.178	-26.258	6.796	1.00	20.37	H	0.076

```
ATOM   1590  N   TRP A 108      -1.633 -27.471   4.401  1.00 14.35           N  0.064
ATOM   1591  CA  TRP A 108      -2.335 -27.087   3.187  1.00 14.99           C  0.066
ATOM   1592  C   TRP A 108      -2.971 -28.352   2.645  1.00 17.37           C  0.071
ATOM   1593  O   TRP A 108      -2.283 -29.226   2.107  1.00 18.15           O  0.072
ATOM   1594  CB  TRP A 108      -1.442 -26.452   2.169  1.00 17.07           C  0.070
ATOM   1595  CG  TRP A 108      -1.058 -25.099   2.488  1.00 14.02           C  0.063
ATOM   1596  CD1 TRP A 108      -1.806 -23.946   2.283  1.00 19.02           C  0.074
ATOM   1597  CD2 TRP A 108       0.105 -24.697   3.192  1.00 16.53           C  0.069
ATOM   1598  NE1 TRP A 108      -1.126 -22.866   2.759  1.00 15.03           N  0.066
ATOM   1599  CE2 TRP A 108       0.035 -23.299   3.338  1.00 17.82           C  0.071
ATOM   1600  CE3 TRP A 108       1.191 -25.383   3.735  1.00 13.08           C  0.061
ATOM   1601  CZ2 TRP A 108       1.034 -22.576   3.925  1.00 15.34           C  0.066
ATOM   1602  CZ3 TRP A 108       2.169 -24.660   4.325  1.00 17.21           C  0.070
ATOM   1603  CH2 TRP A 108       2.094 -23.278   4.418  1.00 20.11           C  0.076
ATOM   1604  H   TRP A 108      -0.907 -27.911   4.266  1.00 17.22           H  0.070
ATOM   1605  HA  TRP A 108      -3.014 -26.429   3.403  1.00 17.98           H  0.072
ATOM   1606  HB2 TRP A 108      -0.631 -26.979   2.094  1.00 20.49           H  0.077
ATOM   1607  HB3 TRP A 108      -1.905 -26.433   1.317  1.00 20.49           H  0.077
ATOM   1608  HD1 TRP A 108      -2.645 -23.916   1.881  1.00 22.83           H  0.081
ATOM   1609  HE1 TRP A 108      -1.385 -22.047   2.705  1.00 18.03           H  0.072
ATOM   1610  HE3 TRP A 108       1.242 -26.310   3.692  1.00 15.70           H  0.067
ATOM   1611  HZ2 TRP A 108       0.993 -21.649   3.985  1.00 18.41           H  0.073
ATOM   1612  HZ3 TRP A 108       2.909 -25.100   4.678  1.00 20.66           H  0.077
ATOM   1613  HH2 TRP A 108       2.788 -22.816   4.830  1.00 24.14           H  0.083
ATOM   1614  N   VAL A 109      -4.263 -28.469   2.810  1.00 19.96           N  0.076
ATOM   1615  CA  VAL A 109      -4.843 -29.764   2.517  1.00 20.51           C  0.077
ATOM   1616  C   VAL A 109      -4.689 -30.048   1.035  1.00 17.81           C  0.071
ATOM   1617  O   VAL A 109      -4.525 -31.202   0.625  1.00 20.07           O  0.076
ATOM   1618  CB  VAL A 109      -6.311 -29.871   2.955  1.00 30.38           C  0.093
ATOM   1619  CG1 VAL A 109      -6.531 -29.577   4.460  1.00 32.23           C  0.096
ATOM   1620  CG2 VAL A 109      -7.131 -28.976   2.145  1.00 29.31           C  0.092
ATOM   1621  H   VAL A 109      -4.802 -27.853   3.074  1.00 23.95           H  0.083
ATOM   1622  HA  VAL A 109      -4.362 -30.428   3.036  1.00 24.62           H  0.084
ATOM   1623  HB  VAL A 109      -6.583 -30.792   2.818  1.00 36.46           H  0.102
ATOM   1624 HG11 VAL A 109      -7.478 -29.641   4.658  1.00 38.68           H  0.105
ATOM   1625 HG12 VAL A 109      -6.039 -30.228   4.984  1.00 38.68           H  0.105
ATOM   1626 HG13 VAL A 109      -6.210 -28.683   4.657  1.00 38.68           H  0.105
ATOM   1627 HG21 VAL A 109      -8.037 -28.977   2.493  1.00 35.17           H  0.100
ATOM   1628 HG22 VAL A 109      -6.760 -28.081   2.190  1.00 35.17           H  0.100
ATOM   1629 HG23 VAL A 109      -7.129 -29.290   1.227  1.00 35.17           H  0.100
ATOM   1630  N   ALA A 110      -4.795 -29.003   0.203  1.00 16.33           N  0.068
ATOM   1631  CA  ALA A 110      -4.535 -29.205  -1.219  1.00 18.02           C  0.072
ATOM   1632  C   ALA A 110      -3.114 -29.711  -1.489  1.00 16.42           C  0.069
ATOM   1633  O   ALA A 110      -2.916 -30.502  -2.403  1.00 20.66           O  0.077
ATOM   1634  CB  ALA A 110      -4.789 -27.953  -2.019  1.00 24.02           C  0.083
ATOM   1635  H   ALA A 110      -5.007 -28.201   0.431  1.00 19.60           H  0.075
ATOM   1636  HA  ALA A 110      -5.163 -29.881  -1.519  1.00 21.63           H  0.079
ATOM   1637  HB1 ALA A 110      -4.725 -28.163  -2.964  1.00 28.83           H  0.091
ATOM   1638  HB2 ALA A 110      -5.677 -27.622  -1.813  1.00 28.83           H  0.091
ATOM   1639  HB3 ALA A 110      -4.125 -27.287  -1.783  1.00 28.83           H  0.091
ATOM   1640  N   TRP A 111      -2.101 -29.267  -0.728  1.00 14.59           N  0.065
ATOM   1641  CA  TRP A 111      -0.770 -29.857  -0.897  1.00 13.41           C  0.062
ATOM   1642  C   TRP A 111      -0.810 -31.333  -0.530  1.00 18.53           C  0.073
ATOM   1643  O   TRP A 111      -0.294 -32.201  -1.247  1.00 15.82           O  0.067
ATOM   1644  CB  TRP A 111       0.279 -29.103  -0.048  1.00 14.68           C  0.065
ATOM   1645  CG  TRP A 111       1.612 -29.694  -0.176  1.00 12.28           C  0.059
ATOM   1646  CD1 TRP A 111       2.530 -29.451  -1.175  1.00 13.33           C  0.062
ATOM   1647  CD2 TRP A 111       2.186 -30.686   0.647  1.00 16.84           C  0.069
ATOM   1648  NE1 TRP A 111       3.603 -30.250  -1.028  1.00 12.92           N  0.061
ATOM   1649  CE2 TRP A 111       3.438 -31.002   0.108  1.00 15.79           C  0.067
ATOM   1650  CE3 TRP A 111       1.744 -31.370   1.792  1.00 12.68           C  0.060
ATOM   1651  CZ2 TRP A 111       4.269 -31.925   0.688  1.00 13.54           C  0.062
ATOM   1652  CZ3 TRP A 111       2.550 -32.241   2.356  1.00 14.91           C  0.065
ATOM   1653  CH2 TRP A 111       3.804 -32.538   1.821  1.00 14.14           C  0.064
ATOM   1654  H   TRP A 111      -2.159 -28.650  -0.131  1.00 17.50           H  0.071
ATOM   1655  HA  TRP A 111      -0.497 -29.769  -1.823  1.00 16.10           H  0.068
ATOM   1656  HB2 TRP A 111       0.326 -28.181  -0.345  1.00 17.62           H  0.071
ATOM   1657  HB3 TRP A 111       0.019 -29.138   0.886  1.00 17.62           H  0.071
ATOM   1658  HD1 TRP A 111       2.423 -28.825  -1.855  1.00 16.00           H  0.068
ATOM   1659  HE1 TRP A 111       4.280 -30.284  -1.557  1.00 15.51           H  0.067
ATOM   1660  HE3 TRP A 111       0.899 -31.208   2.145  1.00 15.22           H  0.066
ATOM   1661  HZ2 TRP A 111       5.104 -32.125   0.331  1.00 16.25           H  0.068
ATOM   1662  HZ3 TRP A 111       2.277 -32.673   3.133  1.00 17.89           H  0.072
ATOM   1663  HH2 TRP A 111       4.340 -33.169   2.246  1.00 16.97           H  0.070
ATOM   1664  N   ARG A 112      -1.403 -31.652   0.616  1.00 14.44           N  0.064
ATOM   1665  CA  ARG A 112      -1.421 -33.042   0.999  1.00 15.81           C  0.067
ATOM   1666  C   ARG A 112      -2.086 -33.878  -0.082  1.00 15.71           C  0.067
ATOM   1667  O   ARG A 112      -1.603 -34.955  -0.429  1.00 17.26           O  0.070
```

```
ATOM   1668  CB  ARG A 112      -2.158 -33.223   2.321  1.00 15.42           C  0.066
ATOM   1669  CG  ARG A 112      -2.073 -34.610   2.770  1.00 18.16           C  0.072
ATOM   1670  CD  ARG A 112      -2.588 -34.877   4.224  1.00 26.31           C  0.087
ATOM   1671  NE  ARG A 112      -3.870 -34.240   4.499  1.00 34.40           N  0.099
ATOM   1672  CZ  ARG A 112      -5.056 -34.710   4.117  1.00 38.55           C  0.105
ATOM   1673  NH1 ARG A 112      -5.165 -35.825   3.417  1.00 26.21           N  0.087
ATOM   1674  NH2 ARG A 112      -6.154 -34.036   4.444  1.00 33.58           N  0.098
ATOM   1675  H   ARG A 112      -1.783 -31.104   1.159  1.00 17.33           H  0.070
ATOM   1676  HA  ARG A 112      -0.508 -33.345   1.123  1.00 18.98           H  0.074
ATOM   1677  HB2 ARG A 112      -1.757 -32.654   2.996  1.00 18.50           H  0.073
ATOM   1678  HB3 ARG A 112      -3.093 -32.992   2.206  1.00 18.50           H  0.073
ATOM   1679  HG2 ARG A 112      -2.602 -35.160   2.171  1.00 21.79           H  0.079
ATOM   1680  HG3 ARG A 112      -1.143 -34.886   2.738  1.00 21.79           H  0.079
ATOM   1681  HD2 ARG A 112      -2.697 -35.833   4.350  1.00 31.58           H  0.095
ATOM   1682  HD3 ARG A 112      -1.940 -34.531   4.857  1.00 31.58           H  0.095
ATOM   1683  HE  ARG A 112      -3.859 -33.503   4.943  1.00 41.28           H  0.109
ATOM   1684 HH11 ARG A 112      -4.460 -36.265   3.198  1.00 31.46           H  0.095
ATOM   1685 HH12 ARG A 112      -5.941 -36.110   3.181  1.00 31.46           H  0.095
ATOM   1686 HH21 ARG A 112      -6.093 -33.307   4.896  1.00 40.30           H  0.107
ATOM   1687 HH22 ARG A 112      -6.926 -34.330   4.203  1.00 40.30           H  0.107
ATOM   1688  N   ASN A 113      -3.204 -33.400  -0.626  1.00 18.47           N  0.073
ATOM   1689  CA  ASN A 113      -4.010 -34.280  -1.459  1.00 17.08           C  0.070
ATOM   1690  C   ASN A 113      -3.591 -34.258  -2.899  1.00 15.34           C  0.066
ATOM   1691  O   ASN A 113      -3.995 -35.142  -3.668  1.00 19.34           O  0.074
ATOM   1692  CB  ASN A 113      -5.460 -33.871  -1.363  1.00 19.30           C  0.074
ATOM   1693  CG  ASN A 113      -6.061 -34.301  -0.052  1.00 18.08           C  0.072
ATOM   1694  OD1 ASN A 113      -5.659 -35.310   0.522  1.00 20.20           O  0.076
ATOM   1695  ND2 ASN A 113      -7.030 -33.557   0.421  1.00 17.84           N  0.071
ATOM   1696  H   ASN A 113      -3.502 -32.599  -0.529  1.00 22.16           H  0.080
ATOM   1697  HA  ASN A 113      -3.926 -35.191  -1.138  1.00 20.49           H  0.077
ATOM   1698  HB2 ASN A 113      -5.527 -32.905  -1.429  1.00 23.16           H  0.081
ATOM   1699  HB3 ASN A 113      -5.961 -34.288  -2.081  1.00 23.16           H  0.081
ATOM   1700 HD21 ASN A 113      -7.405 -33.763   1.167  1.00 21.40           H  0.078
ATOM   1701 HD22 ASN A 113      -7.291 -32.863  -0.015  1.00 21.40           H  0.078
ATOM   1702  N   ARG A 114      -2.857 -33.257  -3.301  1.00 15.72           N  0.067
ATOM   1703  CA  ARG A 114      -2.628 -33.078  -4.728  1.00 15.38           C  0.066
ATOM   1704  C   ARG A 114      -1.175 -32.875  -5.119  1.00 16.45           C  0.069
ATOM   1705  O   ARG A 114      -0.870 -32.923  -6.321  1.00 19.00           O  0.074
ATOM   1706  CB  ARG A 114      -3.475 -31.890  -5.222  1.00 17.48           C  0.071
ATOM   1707  CG  ARG A 114      -4.944 -32.084  -4.939  1.00 19.55           C  0.075
ATOM   1708  CD  ARG A 114      -5.766 -30.863  -5.414  1.00 20.46           C  0.077
ATOM   1709  NE  ARG A 114      -5.920 -30.832  -6.871  1.00 16.74           N  0.069
ATOM   1710  CZ  ARG A 114      -6.712 -30.004  -7.529  1.00 20.13           C  0.076
ATOM   1711  NH1 ARG A 114      -7.396 -29.077  -6.895  1.00 23.18           N  0.081
ATOM   1712  NH2 ARG A 114      -6.762 -30.072  -8.873  1.00 17.42           N  0.071
ATOM   1713  H   ARG A 114      -2.485 -32.675  -2.788  1.00 18.87           H  0.074
ATOM   1714  HA  ARG A 114      -2.914 -33.875  -5.201  1.00 18.46           H  0.073
ATOM   1715  HB2 ARG A 114      -3.183 -31.082  -4.772  1.00 20.98           H  0.078
ATOM   1716  HB3 ARG A 114      -3.361 -31.795  -6.181  1.00 20.98           H  0.078
ATOM   1717  HG2 ARG A 114      -5.263 -32.870  -5.408  1.00 23.45           H  0.082
ATOM   1718  HG3 ARG A 114      -5.078 -32.191  -3.984  1.00 23.45           H  0.082
ATOM   1719  HD2 ARG A 114      -6.650 -30.900  -5.017  1.00 24.55           H  0.084
ATOM   1720  HD3 ARG A 114      -5.314 -30.049  -5.141  1.00 24.55           H  0.084
ATOM   1721  HE  ARG A 114      -5.461 -31.395  -7.331  1.00 20.09           H  0.076
ATOM   1722 HH11 ARG A 114      -7.331 -29.004  -6.041  1.00 27.82           H  0.089
ATOM   1723 HH12 ARG A 114      -7.908 -28.545  -7.335  1.00 27.82           H  0.089
ATOM   1724 HH21 ARG A 114      -6.282 -30.650  -9.293  1.00 20.90           H  0.077
ATOM   1725 HH22 ARG A 114      -7.274 -29.538  -9.312  1.00 20.90           H  0.077
ATOM   1726  N   CYS A 115      -0.276 -32.662  -4.167  1.00 15.10           N  0.066
ATOM   1727  CA  CYS A 115       1.140 -32.401  -4.424  1.00 15.39           C  0.066
ATOM   1728  C   CYS A 115       2.049 -33.411  -3.751  1.00 16.88           C  0.070
ATOM   1729  O   CYS A 115       3.058 -33.852  -4.330  1.00 17.55           O  0.071
ATOM   1730  CB  CYS A 115       1.562 -30.993  -3.909  1.00 13.88           C  0.063
ATOM   1731  SG  CYS A 115       0.669 -29.651  -4.655  1.00 14.41           S  0.064
ATOM   1732  H   CYS A 115      -0.467 -32.662  -3.328  1.00 18.12           H  0.072
ATOM   1733  HA  CYS A 115       1.258 -32.435  -5.386  1.00 18.47           H  0.073
ATOM   1734  HB2 CYS A 115       1.409 -30.953  -2.952  1.00 16.66           H  0.069
ATOM   1735  HB3 CYS A 115       2.504 -30.862  -4.100  1.00 16.66           H  0.069
ATOM   1736  N   LYS A 116       1.795 -33.668  -2.483  1.00 18.38           N  0.073
ATOM   1737  CA  LYS A 116       2.585 -34.602  -1.700  1.00 18.11           C  0.072
ATOM   1738  C   LYS A 116       2.739 -35.920  -2.426  1.00 15.76           C  0.067
ATOM   1739  O   LYS A 116       1.763 -36.502  -2.903  1.00 17.40           O  0.071
ATOM   1740  CB  LYS A 116       1.876 -34.814  -0.361  1.00 16.55           C  0.069
ATOM   1741  CG  LYS A 116       2.617 -35.635   0.681  1.00 16.26           C  0.068
ATOM   1742  CD  LYS A 116       1.726 -35.759   1.886  1.00 18.12           C  0.072
ATOM   1743  CE  LYS A 116       2.478 -36.362   3.101  1.00 19.01           C  0.074
ATOM   1744  NZ  LYS A 116       1.605 -36.542   4.305  1.00 18.72           N  0.073
ATOM   1745  H   LYS A 116       1.155 -33.306  -2.038  1.00 22.06           H  0.080
```

```
ATOM   1746  HA  LYS A 116       3.475 -34.247  -1.546  1.00 21.73           H  0.079
ATOM   1747  HB2 LYS A 116       1.708 -33.943   0.031  1.00 19.87           H  0.075
ATOM   1748  HB3 LYS A 116       1.036 -35.267  -0.535  1.00 19.87           H  0.075
ATOM   1749  HG2 LYS A 116       2.812 -36.519   0.334  1.00 19.51           H  0.075
ATOM   1750  HG3 LYS A 116       3.440 -35.189   0.935  1.00 19.51           H  0.075
ATOM   1751  HD2 LYS A 116       1.401 -34.880   2.136  1.00 21.74           H  0.079
ATOM   1752  HD3 LYS A 116       0.979 -36.340   1.673  1.00 21.74           H  0.079
ATOM   1753  HE2 LYS A 116       2.828 -37.232   2.854  1.00 22.81           H  0.081
ATOM   1754  HE3 LYS A 116       3.205 -35.769   3.347  1.00 22.81           H  0.081
ATOM   1755  HZ1 LYS A 116       2.068 -36.923   4.962  1.00 22.46           H  0.080
ATOM   1756  HZ2 LYS A 116       1.303 -35.753   4.583  1.00 22.46           H  0.080
ATOM   1757  HZ3 LYS A 116       0.911 -37.062   4.102  1.00 22.46           H  0.080
ATOM   1758  N   GLY A 117       3.965 -36.399  -2.482  1.00 17.09           N  0.070
ATOM   1759  CA  GLY A 117       4.265 -37.655  -3.122  1.00 23.47           C  0.082
ATOM   1760  C   GLY A 117       4.378 -37.599  -4.631  1.00 24.96           C  0.085
ATOM   1761  O   GLY A 117       4.747 -38.610  -5.239  1.00 34.28           O  0.099
ATOM   1762  H   GLY A 117       4.653 -36.006  -2.148  1.00 20.51           H  0.077
ATOM   1763  HA2 GLY A 117       5.109 -37.985  -2.776  1.00 28.16           H  0.090
ATOM   1764  HA3 GLY A 117       3.564 -38.289  -2.903  1.00 28.16           H  0.090
ATOM   1765  N   THR A 118       4.009 -36.489  -5.250  1.00 18.97           N  0.074
ATOM   1766  CA  THR A 118       4.087 -36.312  -6.688  1.00 20.79           C  0.077
ATOM   1767  C   THR A 118       5.435 -35.727  -7.097  1.00 20.70           C  0.077
ATOM   1768  O   THR A 118       6.271 -35.331  -6.283  1.00 20.62           O  0.077
ATOM   1769  CB  THR A 118       2.956 -35.401  -7.210  1.00 21.80           C  0.079
ATOM   1770  OG1 THR A 118       3.228 -34.017  -6.887  1.00 21.90           O  0.079
ATOM   1771  CG2 THR A 118       1.581 -35.895  -6.710  1.00 20.28           C  0.076
ATOM   1772  H   THR A 118       3.699 -35.798  -4.843  1.00 22.76           H  0.081
ATOM   1773  HA  THR A 118       3.986 -37.186  -7.095  1.00 24.94           H  0.085
ATOM   1774  HB  THR A 118       2.909 -35.443  -8.178  1.00 26.16           H  0.087
ATOM   1775  HG1 THR A 118       3.484 -33.953  -6.090  1.00 26.28           H  0.087
ATOM   1776 HG21 THR A 118       0.873 -35.373  -7.118  1.00 24.34           H  0.084
ATOM   1777 HG22 THR A 118       1.458 -36.828  -6.944  1.00 24.34           H  0.084
ATOM   1778 HG23 THR A 118       1.526 -35.802  -5.746  1.00 24.34           H  0.084
ATOM   1779  N   ASP A 119       5.652 -35.731  -8.410  1.00 21.23           N  0.078
ATOM   1780  CA  ASP A 119       6.884 -35.184  -9.007  1.00 20.32           C  0.076
ATOM   1781  C   ASP A 119       6.691 -33.685  -9.009  1.00 21.87           C  0.079
ATOM   1782  O   ASP A 119       6.214 -33.152 -10.009  1.00 24.43           O  0.084
ATOM   1783  CB  ASP A 119       7.079 -35.781 -10.392  1.00 30.92           C  0.094
ATOM   1784  CG  ASP A 119       8.287 -35.288 -11.158  1.00 33.44           C  0.098
ATOM   1785  OD1 ASP A 119       9.072 -34.498 -10.612  1.00 28.32           O  0.090
ATOM   1786  OD2 ASP A 119       8.418 -35.721 -12.295  1.00 40.43           O  0.108
ATOM   1787  H   ASP A 119       4.978 -36.111  -9.100  1.00 25.47           H  0.085
ATOM   1788  HA  ASP A 119       7.734 -35.440  -8.377  1.00 24.38           H  0.084
ATOM   1789  HB2 ASP A 119       7.165 -36.862 -10.291  1.00 37.10           H  0.103
ATOM   1790  HB3 ASP A 119       6.193 -35.569 -10.989  1.00 37.10           H  0.103
ATOM   1791  N   VAL A 120       6.975 -33.060  -7.884  1.00 23.01           N  0.081
ATOM   1792  CA  VAL A 120       6.724 -31.629  -7.726  1.00 21.30           C  0.078
ATOM   1793  C   VAL A 120       7.743 -30.799  -8.456  1.00 24.58           C  0.084
ATOM   1794  O   VAL A 120       7.517 -29.617  -8.690  1.00 23.89           O  0.083
ATOM   1795  CB  VAL A 120       6.716 -31.198  -6.247  1.00 17.65           C  0.071
ATOM   1796  CG1 VAL A 120       5.441 -31.863  -5.549  1.00 18.93           C  0.074
ATOM   1797  CG2 VAL A 120       8.036 -31.498  -5.590  1.00 26.52           C  0.087
ATOM   1798  H   VAL A 120       7.320 -33.439  -7.194  1.00 27.61           H  0.089
ATOM   1799  HA  VAL A 120       5.842 -31.462  -8.093  1.00 25.56           H  0.086
ATOM   1800  HB  VAL A 120       6.626 -30.237  -6.148  1.00 21.18           H  0.078
ATOM   1801 HG11 VAL A 120       5.393 -31.564  -4.628  1.00 22.72           H  0.081
ATOM   1802 HG12 VAL A 120       4.642 -31.590  -6.027  1.00 22.72           H  0.081
ATOM   1803 HG13 VAL A 120       5.531 -32.828  -5.580  1.00 22.72           H  0.081
ATOM   1804 HG21 VAL A 120       7.993 -31.228  -4.659  1.00 31.82           H  0.095
ATOM   1805 HG22 VAL A 120       8.209 -32.450  -5.650  1.00 31.82           H  0.095
ATOM   1806 HG23 VAL A 120       8.735 -31.004  -6.046  1.00 31.82           H  0.095
ATOM   1807  N   GLN A 121       8.856 -31.393  -8.848  1.00 24.58           N  0.084
ATOM   1808  CA  GLN A 121       9.862 -30.675  -9.656  1.00 17.60           C  0.071
ATOM   1809  C   GLN A 121       9.253 -30.285 -11.001  1.00 26.34           C  0.087
ATOM   1810  O   GLN A 121       9.740 -29.334 -11.597  1.00 24.94           O  0.085
ATOM   1811  CB  GLN A 121      11.084 -31.555  -9.875  1.00 32.11           C  0.096
ATOM   1812  CG  GLN A 121      12.376 -30.772 -10.028  1.00 41.30           C  0.109
ATOM   1813  CD  GLN A 121      12.500 -30.092 -11.369  1.00 58.41           C  0.129
ATOM   1814  OE1 GLN A 121      12.889 -28.933 -11.463  1.00 55.78           O  0.126
ATOM   1815  NE2 GLN A 121      12.179 -30.817 -12.427  1.00 53.78           N  0.124
ATOM   1816  H   GLN A 121       9.094 -32.376  -8.623  1.00 29.50           H  0.092
ATOM   1817  HA  GLN A 121      10.160 -29.772  -9.128  1.00 21.11           H  0.078
ATOM   1818  HB2 GLN A 121      11.178 -32.230  -9.026  1.00 38.53           H  0.105
ATOM   1819  HB3 GLN A 121      10.920 -32.161 -10.764  1.00 38.53           H  0.105
ATOM   1820  HG2 GLN A 121      12.428 -30.017  -9.245  1.00 49.56           H  0.119
ATOM   1821  HG3 GLN A 121      13.219 -31.448  -9.896  1.00 49.56           H  0.119
ATOM   1822 HE21 GLN A 121      12.592 -31.736 -12.556  1.00 64.54           H  0.136
ATOM   1823 HE22 GLN A 121      11.508 -30.462 -13.102  1.00 64.54           H  0.136
```

```
ATOM   1824  N   ALA A 122       8.255 -31.016 -11.468  1.00 20.40           N  0.076
ATOM   1825  CA  ALA A 122       7.572 -30.643 -12.706  1.00 23.10           C  0.081
ATOM   1826  C   ALA A 122       7.072 -29.221 -12.665  1.00 26.68           C  0.087
ATOM   1827  O   ALA A 122       7.041 -28.517 -13.686  1.00 22.15           O  0.080
ATOM   1828  CB  ALA A 122       6.392 -31.567 -12.962  1.00 24.38           C  0.084
ATOM   1829  H   ALA A 122       7.945 -31.725 -11.092  1.00 24.48           H  0.084
ATOM   1830  HA  ALA A 122       8.207 -30.736 -13.434  1.00 27.71           H  0.089
ATOM   1831  HB1 ALA A 122       5.951 -31.295 -13.782  1.00 29.26           H  0.092
ATOM   1832  HB2 ALA A 122       6.716 -32.477 -13.047  1.00 29.26           H  0.092
ATOM   1833  HB3 ALA A 122       5.774 -31.504 -12.217  1.00 29.26           H  0.092
ATOM   1834  N   TRP A 123       6.702 -28.764 -11.480  1.00 19.75           N  0.075
ATOM   1835  CA  TRP A 123       6.134 -27.435 -11.347  1.00 23.40           C  0.082
ATOM   1836  C   TRP A 123       7.145 -26.327 -11.599  1.00 22.42           C  0.080
ATOM   1837  O   TRP A 123       6.738 -25.209 -11.914  1.00 27.80           O  0.089
ATOM   1838  CB  TRP A 123       5.479 -27.294  -9.936  1.00 21.17           C  0.078
ATOM   1839  CG  TRP A 123       4.222 -28.063  -9.924  1.00 13.41           C  0.062
ATOM   1840  CD1 TRP A 123       4.002 -29.282  -9.383  1.00 20.62           C  0.077
ATOM   1841  CD2 TRP A 123       3.005 -27.671 -10.547  1.00 18.26           C  0.072
ATOM   1842  NE1 TRP A 123       2.693 -29.689  -9.614  1.00 21.08           N  0.078
ATOM   1843  CE2 TRP A 123       2.070 -28.716 -10.352  1.00 18.50           C  0.073
ATOM   1844  CE3 TRP A 123       2.609 -26.531 -11.275  1.00 30.13           C  0.093
ATOM   1845  CZ2 TRP A 123       0.789 -28.658 -10.842  1.00 26.17           C  0.087
ATOM   1846  CZ3 TRP A 123       1.321 -26.486 -11.791  1.00 31.19           C  0.095
ATOM   1847  CH2 TRP A 123       0.430 -27.541 -11.563  1.00 30.00           C  0.093
ATOM   1848  H   TRP A 123       6.769 -29.200 -10.742  1.00 23.70           H  0.082
ATOM   1849  HA  TRP A 123       5.436 -27.318 -12.011  1.00 28.08           H  0.090
ATOM   1850  HB2 TRP A 123       6.074 -27.649  -9.257  1.00 25.40           H  0.085
ATOM   1851  HB3 TRP A 123       5.283 -26.362  -9.752  1.00 25.40           H  0.085
ATOM   1852  HD1 TRP A 123       4.638 -29.779  -8.921  1.00 24.75           H  0.084
ATOM   1853  HE1 TRP A 123       2.336 -30.422  -9.342  1.00 25.30           H  0.085
ATOM   1854  HE3 TRP A 123       3.199 -25.824 -11.408  1.00 36.15           H  0.102
ATOM   1855  HZ2 TRP A 123       0.185 -29.348 -10.691  1.00 31.41           H  0.095
ATOM   1856  HZ3 TRP A 123       1.048 -25.750 -12.290  1.00 37.43           H  0.104
ATOM   1857  HH2 TRP A 123      -0.431 -27.486 -11.910  1.00 36.00           H  0.102
ATOM   1858  N   ILE A 124       8.443 -26.600 -11.456  1.00 26.46           N  0.087
ATOM   1859  CA  ILE A 124       9.450 -25.607 -11.794  1.00 36.04           C  0.102
ATOM   1860  C   ILE A 124      10.242 -25.992 -13.043  1.00 29.74           C  0.092
ATOM   1861  O   ILE A 124      11.155 -25.257 -13.414  1.00 25.89           O  0.086
ATOM   1862  CB  ILE A 124      10.404 -25.323 -10.611  1.00 33.95           C  0.099
ATOM   1863  CG1 ILE A 124      11.181 -26.573 -10.219  1.00 27.73           C  0.089
ATOM   1864  CG2 ILE A 124       9.634 -24.766  -9.351  1.00 25.84           C  0.086
ATOM   1865  CD1 ILE A 124      12.409 -26.319  -9.339  1.00 39.26           C  0.106
ATOM   1866  H   ILE A 124       8.757 -27.347 -11.169  1.00 31.75           H  0.095
ATOM   1867  HA  ILE A 124       8.991 -24.775 -11.988  1.00 43.25           H  0.111
ATOM   1868  HB  ILE A 124      11.024 -24.643 -10.918  1.00 40.74           H  0.108
ATOM   1869 HG12 ILE A 124      10.588 -27.162  -9.727  1.00 33.28           H  0.098
ATOM   1870 HG13 ILE A 124      11.488 -27.011 -11.028  1.00 33.28           H  0.098
ATOM   1871 HG21 ILE A 124      10.277 -24.562  -8.654  1.00 31.00           H  0.094
ATOM   1872 HG22 ILE A 124       9.154 -23.962  -9.604  1.00 31.00           H  0.094
ATOM   1873 HG23 ILE A 124       9.010 -25.440  -9.039  1.00 31.00           H  0.094
ATOM   1874 HD11 ILE A 124      12.888 -27.153  -9.217  1.00 47.12           H  0.116
ATOM   1875 HD12 ILE A 124      12.982 -25.670  -9.777  1.00 47.12           H  0.116
ATOM   1876 HD13 ILE A 124      12.117 -25.976  -8.480  1.00 47.12           H  0.116
ATOM   1877  N   ARG A 125       9.904 -27.089 -13.710  1.00 26.15           N  0.087
ATOM   1878  CA  ARG A 125      10.671 -27.516 -14.888  1.00 34.73           C  0.100
ATOM   1879  C   ARG A 125      10.580 -26.454 -15.977  1.00 33.83           C  0.098
ATOM   1880  O   ARG A 125       9.489 -26.025 -16.354  1.00 28.41           O  0.090
ATOM   1881  CB  ARG A 125      10.164 -28.881 -15.423  1.00 45.03           C  0.114
ATOM   1882  CG  ARG A 125      11.116 -29.588 -16.461  1.00 56.26           C  0.127
ATOM   1883  CD  ARG A 125      11.041 -31.156 -16.519  1.00 55.23           C  0.126
ATOM   1884  NE  ARG A 125       9.975 -31.673 -17.386  1.00 80.71           N  0.152
ATOM   1885  CZ  ARG A 125       9.865 -32.931 -17.818  1.00 70.51           C  0.142
ATOM   1886  NH1 ARG A 125      10.706 -33.879 -17.426  1.00 53.06           N  0.123
ATOM   1887  NH2 ARG A 125       8.895 -33.241 -18.683  1.00 58.63           N  0.130
ATOM   1888  H   ARG A 125       9.244 -27.603 -13.509  1.00 31.38           H  0.095
ATOM   1889  HA  ARG A 125      11.600 -27.633 -14.636  1.00 41.67           H  0.109
ATOM   1890  HB2 ARG A 125      10.056 -29.484 -14.671  1.00 54.04           H  0.124
ATOM   1891  HB3 ARG A 125       9.311 -28.741 -15.861  1.00 54.04           H  0.124
ATOM   1892  HG2 ARG A 125      10.896 -29.260 -17.347  1.00 67.51           H  0.139
ATOM   1893  HG3 ARG A 125      12.032 -29.355 -16.239  1.00 67.51           H  0.139
ATOM   1894  HD2 ARG A 125      11.885 -31.496 -16.857  1.00 66.27           H  0.138
ATOM   1895  HD3 ARG A 125      10.882 -31.494 -15.624  1.00 66.27           H  0.138
ATOM   1896  HE  ARG A 125       9.368 -31.117 -17.637  1.00 96.86           H  0.167
ATOM   1897 HH11 ARG A 125      11.342 -33.689 -16.879  1.00 63.68           H  0.135
ATOM   1898 HH12 ARG A 125      10.615 -34.682 -17.718  1.00 63.68           H  0.135
ATOM   1899 HH21 ARG A 125       8.351 -32.633 -18.955  1.00 70.36           H  0.142
ATOM   1900 HH22 ARG A 125       8.815 -34.048 -18.968  1.00 70.36           H  0.142
ATOM   1901  N   GLY A 126      11.727 -26.019 -16.498  1.00 43.73           N  0.112
```

```
ATOM   1902  CA  GLY A 126      11.727 -25.026 -17.545  1.00 37.51           C  0.104
ATOM   1903  C   GLY A 126      11.884 -23.587 -17.095  1.00 39.52           C  0.106
ATOM   1904  O   GLY A 126      12.092 -22.708 -17.939  1.00 37.63           O  0.104
ATOM   1905  H   GLY A 126      12.507 -26.287 -16.257  1.00 52.48           H  0.123
ATOM   1906  HA2 GLY A 126      12.457 -25.222 -18.153  1.00 45.01           H  0.114
ATOM   1907  HA3 GLY A 126      10.888 -25.090 -18.028  1.00 45.01           H  0.114
ATOM   1908  N   CYS A 127      11.783 -23.298 -15.801  1.00 33.16           N  0.097
ATOM   1909  CA  CYS A 127      11.892 -21.912 -15.355  1.00 35.72           C  0.101
ATOM   1910  C   CYS A 127      13.345 -21.473 -15.193  1.00 34.22           C  0.099
ATOM   1911  O   CYS A 127      14.171 -22.177 -14.600  1.00 33.05           O  0.097
ATOM   1912  CB  CYS A 127      11.149 -21.717 -14.028  1.00 34.63           C  0.100
ATOM   1913  SG  CYS A 127       9.533 -22.509 -14.059  1.00 30.32           S  0.093
ATOM   1914  H   CYS A 127      11.655 -23.873 -15.175  1.00 39.79           H  0.107
ATOM   1915  HA  CYS A 127      11.489 -21.346 -16.031  1.00 42.86           H  0.111
ATOM   1916  HB2 CYS A 127      11.669 -22.108 -13.309  1.00 41.56           H  0.109
ATOM   1917  HB3 CYS A 127      11.022 -20.768 -13.868  1.00 41.56           H  0.109
ATOM   1918  N   ARG A 128      13.629 -20.247 -15.635  1.00 38.72           N  0.105
ATOM   1919  CA  ARG A 128      14.962 -19.639 -15.421  1.00 39.12           C  0.106
ATOM   1920  C   ARG A 128      15.031 -19.211 -13.963  1.00 45.19           C  0.114
ATOM   1921  O   ARG A 128      14.615 -18.085 -13.678  1.00 53.25           O  0.124
ATOM   1922  CB  ARG A 128      15.246 -18.452 -16.351  1.00 42.86           C  0.111
ATOM   1923  CG  ARG A 128      16.424 -17.587 -15.933  1.00 49.35           C  0.119
ATOM   1924  CD  ARG A 128      17.249 -17.136 -17.116  1.00 49.11           C  0.119
ATOM   1925  NE  ARG A 128      16.400 -16.660 -18.196  1.00 56.80           N  0.128
ATOM   1926  CZ  ARG A 128      16.700 -16.697 -19.491  1.00 65.42           C  0.137
ATOM   1927  NH1 ARG A 128      17.855 -17.183 -19.920  1.00 59.07           N  0.130
ATOM   1928  NH2 ARG A 128      15.828 -16.239 -20.368  1.00 63.38           N  0.135
ATOM   1929  H   ARG A 128      12.955 -19.647 -16.144  1.00 46.46           H  0.115
ATOM   1930  HA  ARG A 128      15.718 -20.403 -15.593  1.00 46.94           H  0.116
ATOM   1931  HB2 ARG A 128      15.435 -18.839 -17.351  1.00 51.43           H  0.121
ATOM   1932  HB3 ARG A 128      14.354 -17.829 -16.398  1.00 51.43           H  0.121
ATOM   1933  HG2 ARG A 128      16.056 -16.710 -15.403  1.00 59.22           H  0.130
ATOM   1934  HG3 ARG A 128      17.061 -18.150 -15.253  1.00 59.22           H  0.130
ATOM   1935  HD2 ARG A 128      17.924 -16.341 -16.807  1.00 58.94           H  0.130
ATOM   1936  HD3 ARG A 128      17.848 -17.975 -17.463  1.00 58.94           H  0.130
ATOM   1937  HE  ARG A 128      15.494 -16.279 -17.943  1.00 68.16           H  0.140
ATOM   1938 HH11 ARG A 128      18.546 -17.541 -19.269  1.00 70.89           H  0.143
ATOM   1939 HH12 ARG A 128      18.050 -17.190 -20.917  1.00 70.89           H  0.143
ATOM   1940 HH21 ARG A 128      14.940 -15.864 -20.048  1.00 76.06           H  0.148
ATOM   1941 HH22 ARG A 128      16.051 -16.258 -21.359  1.00 76.06           H  0.148
ATOM   1942  N   LEU A 129      15.505 -20.083 -13.088  1.00 49.94           N  0.120
ATOM   1943  CA  LEU A 129      15.639 -19.796 -11.643  1.00 50.65           C  0.120
ATOM   1944  C   LEU A 129      17.056 -20.215 -11.243  1.00 69.44           C  0.141
ATOM   1945  O   LEU A 129      17.806 -20.633 -12.118  1.00 70.93           O  0.143
ATOM   1946  CB  LEU A 129      14.567 -20.566 -10.867  1.00 43.69           C  0.112
ATOM   1947  CG  LEU A 129      13.193 -20.665 -11.524  1.00 45.84           C  0.115
ATOM   1948  CD1 LEU A 129      12.258 -21.529 -10.697  1.00 49.05           C  0.119
ATOM   1949  CD2 LEU A 129      12.578 -19.296 -11.740  1.00 39.01           C  0.106
ATOM   1950  OXT LEU A 129      17.381 -20.111 -10.061  1.00 64.96           O  0.136
ATOM   1951  H   LEU A 129      15.802 -21.040 -13.352  1.00 59.93           H  0.131
ATOM   1952  HA  LEU A 129      15.521 -18.727 -11.478  1.00 60.78           H  0.132
ATOM   1953  HB2 LEU A 129      14.935 -21.575 -10.693  1.00 52.42           H  0.123
ATOM   1954  HB3 LEU A 129      14.446 -20.090  -9.895  1.00 52.42           H  0.123
ATOM   1955  HG  LEU A 129      13.318 -21.137 -12.496  1.00 55.01           H  0.126
ATOM   1956 HD11 LEU A 129      11.282 -21.568 -11.178  1.00 58.86           H  0.130
ATOM   1957 HD12 LEU A 129      12.665 -22.536 -10.619  1.00 58.86           H  0.130
ATOM   1958 HD13 LEU A 129      12.154 -21.100  -9.701  1.00 58.86           H  0.130
ATOM   1959 HD21 LEU A 129      12.372 -19.155 -12.800  1.00 46.81           H  0.116
ATOM   1960 HD22 LEU A 129      11.647 -19.225 -11.178  1.00 46.81           H  0.116
ATOM   1961 HD23 LEU A 129      13.268 -18.527 -11.397  1.00 46.81           H  0.116
HETATM 1962 CL    CL A1131      11.112 -29.051  -6.734  0.77 21.55          Cl  0.079
HETATM 1963 CL    CL A1132     -11.893 -11.882  13.833  0.99 25.26          Cl  0.085
HETATM 1964 CL    CL A1134       0.413 -24.061 -14.634  1.00 38.52          Cl  0.105
HETATM 1965 CL    CL A1135      -5.706  -6.158  11.219  1.00 39.42          Cl  0.106
HETATM 1966 CL    CL A1137     -18.890 -10.272   6.123  0.80 33.93          Cl  0.099
HETATM 1967  C1  RI3 A1139       9.853  -8.263  -5.340  0.56 60.50           C  0.132
HETATM 1968  C2  RI3 A1139       8.737  -8.357  -2.973  0.56 45.18           C  0.114
HETATM 1969  C3  RI3 A1139      10.852  -9.639  -3.234  0.56 53.47           C  0.124
HETATM 1970  C7  RI3 A1139       7.391 -10.553  -6.637  0.56 43.02           C  0.111
HETATM 1971  C8  RI3 A1139       5.404 -10.446  -5.891  0.56 36.97           C  0.103
HETATM 1972  C9  RI3 A1139       6.245 -10.078  -4.920  0.56 36.73           C  0.103
HETATM 1973  N3  RI3 A1139       7.494 -10.129  -5.401  0.56 43.26           N  0.111
HETATM 1974  N4  RI3 A1139       6.116 -10.739  -6.952  0.56 41.32           N  0.109
HETATM 1975  O1  RI3 A1139      10.220  -7.382  -6.009  0.56 58.06           O  0.129
HETATM 1976  O2  RI3 A1139       8.568  -7.601  -2.148  0.56 43.31           O  0.111
HETATM 1977  O3  RI3 A1139      11.730  -9.692  -2.525  0.56 47.81           O  0.117
HETATM 1978  O4  RI3 A1139       9.973 -11.196  -5.624  0.56 32.47           O  0.096
HETATM 1979 RE1  RI3 A1139       9.247  -9.669  -4.251  0.56 73.68          Re  0.145
```

```
HETATM 1980  H42 RI3 A1139       5.815 -11.005  -7.712  0.56 49.59           H  0.119
HETATM 1981  H71 RI3 A1139       8.102 -10.692  -7.217  0.56 51.63           H  0.122
HETATM 1982  H81 RI3 A1139       4.493 -10.488  -5.823  0.56 44.36           H  0.113
HETATM 1983  H91 RI3 A1139       6.034  -9.806  -4.063  0.56 44.07           H  0.112
HETATM 1984  C11 RIW A1141      -1.683 -22.594  14.483  0.54 34.29           C  0.099
HETATM 1985  C2  RIW A1141       0.998 -21.410  15.664  0.54 37.70           C  0.104
HETATM 1986  C3  RIW A1141      -2.774 -24.448  14.172  0.54 32.56           C  0.097
HETATM 1987  C5  RIW A1141      -0.487 -22.106  17.725  0.54 41.29           C  0.109
HETATM 1988  C6  RIW A1141       2.036 -22.612  17.748  0.54 49.06           C  0.119
HETATM 1989  C7  RIW A1141      -1.776 -24.661  15.058  0.54 19.21           C  0.074
HETATM 1990  N1  RIW A1141      -1.095 -23.493  15.239  0.54 29.10           N  0.091
HETATM 1991  N2  RIW A1141      -2.700 -23.143  13.822  0.54 35.02           N  0.100
HETATM 1992  O1  RIW A1141       0.295 -25.038  17.200  0.54 27.03           O  0.088
HETATM 1993  O2  RIW A1141      -1.168 -21.541  18.450  0.54 37.01           O  0.103
HETATM 1994  O4  RIW A1141       2.862 -22.312  18.479  0.54 38.81           O  0.105
HETATM 1995  O5  RIW A1141       1.254 -20.417  15.142  0.54 38.22           O  0.105
HETATM 1996 RE1  RIW A1141       0.627 -23.055  16.515  0.54 46.36          Re  0.115
HETATM 1997  H21 RIW A1141      -3.167 -22.725  13.305  0.54 42.02           H  0.110
HETATM 1998  H31 RIW A1141      -3.391 -25.070  13.865  0.54 39.07           H  0.106
HETATM 1999  H71 RIW A1141      -1.584 -25.471  15.469  0.54 23.05           H  0.081
HETATM 2000 H111 RIW A1141      -1.425 -21.704  14.409  0.54 41.14           H  0.109
HETATM 2001  C1  RI3 A1142      12.140 -34.931 -14.248  0.44 31.79           C  0.095
HETATM 2002  C2  RI3 A1142       9.627 -35.313 -14.716  0.44 31.04           C  0.094
HETATM 2003  C3  RI3 A1142      10.334 -33.333 -13.131  0.44 25.58           C  0.086
HETATM 2004  C7  RI3 A1142      10.233 -38.182 -12.187  0.44 23.73           C  0.082
HETATM 2005  C8  RI3 A1142      11.542 -39.427 -13.351  0.44 31.14           C  0.094
HETATM 2006  C9  RI3 A1142      11.709 -38.143 -13.724  0.44 29.17           C  0.091
HETATM 2007  N3  RI3 A1142      10.871 -37.351 -12.969  0.44 22.26           N  0.080
HETATM 2008  N4  RI3 A1142      10.628 -39.435 -12.401  0.44 23.80           N  0.083
HETATM 2009  O1  RI3 A1142      13.044 -34.768 -14.962  0.44 34.01           O  0.099
HETATM 2010  O2  RI3 A1142       9.000 -35.392 -15.658  0.44 28.33           O  0.090
HETATM 2011  O3  RI3 A1142      10.126 -32.235 -13.165  0.44 29.84           O  0.092
HETATM 2012  O4  RI3 A1142      11.915 -35.052 -11.327  0.44 28.89           O  0.091
HETATM 2013 RE1  RI3 A1142      10.665 -35.193 -13.110  0.44 29.62          Re  0.092
HETATM 2014  H42 RI3 A1142      10.336 -40.130 -11.987  0.44 28.56           H  0.090
HETATM 2015  H71 RI3 A1142       9.593 -37.932 -11.560  0.44 28.47           H  0.090
HETATM 2016  H81 RI3 A1142      11.988 -40.164 -13.696  0.44 37.36           H  0.103
HETATM 2017  H91 RI3 A1142      12.293 -37.839 -14.379  0.44 35.00           H  0.100
TER
HETATM 2018 NA    NA C   1      -9.899 -13.075  11.707  1.00 27.69          Na1+  0.089
HETATM 2019 CL    CL B   1       8.148 -31.471   7.534  1.00 17.25          Cl  0.070
HETATM 2020  O   HOH S   1     -13.768 -14.968   7.678  1.00 15.29           O  0.066
HETATM 2021  O   HOH S   2     -11.549 -13.498  -3.958  1.00 16.75           O  0.069
HETATM 2022  O   HOH S   3      -4.655 -32.962  -8.657  1.00 14.11           O  0.064
HETATM 2023  O   HOH S   4      10.527 -31.075   5.160  1.00 19.93           O  0.076
HETATM 2024  O   HOH S   5      -5.771 -11.177  -9.380  1.00 17.32           O  0.070
HETATM 2025  O   HOH S   6       0.893 -11.421   0.195  1.00 20.51           O  0.077
HETATM 2026  O   HOH S   7      -8.260 -28.004  -9.970  1.00 21.61           O  0.079
HETATM 2027  O   HOH S   8      -1.768 -16.704   0.753  1.00 16.39           O  0.069
HETATM 2028  O   HOH S  10       6.258 -28.247  10.750  1.00 20.96           O  0.077
HETATM 2029  O   HOH S  11       0.328 -12.147  -2.491  1.00 24.94           O  0.085
HETATM 2030  O   HOH S  12       3.234 -38.232   6.096  1.00 21.29           O  0.078
HETATM 2031  O   HOH S  13     -18.439  -8.306   7.973  1.00 21.36           O  0.078
HETATM 2032  O   HOH S  14      -1.384 -13.990  -1.978  1.00 21.56           O  0.079
HETATM 2033  O   HOH S  15       2.198 -27.104   9.867  1.00 19.66           O  0.075
HETATM 2034  O   HOH S  16       5.795 -17.400 -15.624  1.00 26.28           O  0.087
HETATM 2035  O   HOH S  17     -10.507  -5.958  12.124  1.00 23.19           O  0.082
HETATM 2036  O   HOH S  18      -3.223  -7.101  11.951  1.00 25.43           O  0.085
HETATM 2037  O   HOH S  19      -1.721 -33.434  -8.755  1.00 21.51           O  0.079
HETATM 2038  O   HOH S  20       2.642 -32.518  -8.912  1.00 25.84           O  0.086
HETATM 2039  O   HOH S  21       2.954  -8.542   0.660  1.00 24.83           O  0.084
HETATM 2040  O   HOH S  22      -9.333 -22.568   0.084  1.00 23.99           O  0.083
HETATM 2041  O   HOH S  23       0.385 -36.616   6.894  1.00 31.67           O  0.095
HETATM 2042  O   HOH S  24       2.726 -11.897   6.996  1.00 27.74           O  0.089
HETATM 2043  O   HOH S  25     -10.528  -9.219  15.170  1.00 29.49           O  0.092
HETATM 2044  O   HOH S  26      14.298 -31.587   1.891  1.00 26.42           O  0.087
HETATM 2045  O   HOH S  27       7.194 -34.969  -4.080  1.00 25.16           O  0.085
HETATM 2046  O   HOH S  28       0.388 -38.933   0.768  1.00 27.42           O  0.089
HETATM 2047  O   HOH S  29       1.901  -6.078   4.292  1.00 31.04           O  0.094
HETATM 2048  O   HOH S  30      -1.344  -5.296  -4.352  1.00 26.03           O  0.086
HETATM 2049  O   HOH S  31      -8.137 -14.259  12.691  1.00 22.19           O  0.080
HETATM 2050  O   HOH S  32      -3.739  -7.612  -7.987  1.00 29.30           O  0.092
HETATM 2051  O   HOH S  33      -0.350 -38.167   3.556  1.00 28.66           O  0.091
HETATM 2052  O   HOH S  34      -3.171 -19.918 -13.424  1.00 31.11           O  0.094
HETATM 2053  O   HOH S  35       0.722  -7.121   1.754  1.00 31.19           O  0.095
HETATM 2054  O   HOH S  36      -4.350  -6.497  -0.673  1.00 26.11           O  0.086
HETATM 2055  O   HOH S  37      -5.934 -24.643  -9.430  1.00 22.96           O  0.081
HETATM 2056  O   HOH S  38       6.354 -33.578  -1.727  1.00 26.44           O  0.087
```

```
HETATM 2057  O   HOH S  39      -8.086 -12.294  14.209  1.00 24.91           O  0.084
HETATM 2058  O   HOH S  40       8.035 -15.525   8.650  1.00 31.46           O  0.095
HETATM 2059  O   HOH S  41      -3.821 -31.968   5.770  1.00 29.30           O  0.092
HETATM 2060  O   HOH S  42      -7.705 -26.274  -3.073  1.00 22.24           O  0.080
HETATM 2061  O   HOH S  43       5.403 -19.998 -16.618  1.00 25.78           O  0.086
HETATM 2062  O   HOH S  44      -4.088 -21.644   6.599  1.00 19.86           O  0.075
HETATM 2063  O   HOH S  45      -2.652 -10.771 -13.378  1.00 31.46           O  0.095
HETATM 2064  O   HOH S  46       3.550 -12.759   9.255  1.00 26.75           O  0.088
HETATM 2065  O   HOH S  47      -7.859 -24.108   1.081  1.00 31.35           O  0.095
HETATM 2066  O   HOH S  49      -5.719 -26.179   3.837  1.00 22.15           O  0.080
HETATM 2067  O   HOH S  50      -3.159 -14.056  17.705  1.00 30.28           O  0.093
HETATM 2068  O   HOH S  51      -0.280 -35.967  -9.695  1.00 23.92           O  0.083
HETATM 2069  O   HOH S  52     -13.113 -13.113  18.488  0.54 25.99           O  0.086
HETATM 2070  O   HOH S  53      -2.406  -4.788  -2.153  1.00 35.90           O  0.101
HETATM 2071  O   HOH S  54      -5.520  -9.285  15.538  1.00 25.32           O  0.085
HETATM 2072  O   HOH S  55      -0.102 -32.178 -10.555  1.00 24.63           O  0.084
HETATM 2073  O   HOH S  56      -1.491 -37.552   0.132  1.00 29.97           O  0.093
HETATM 2074  O   HOH S  57      -3.767  -5.126   3.118  1.00 30.71           O  0.094
HETATM 2075  O   HOH S  60      -4.397 -37.871   0.719  1.00 28.45           O  0.090
HETATM 2076  O   HOH S  61       0.904  -5.075   8.718  1.00 40.55           O  0.108
HETATM 2077  O   HOH S  62      -1.729  -3.744   4.866  1.00 29.60           O  0.092
HETATM 2078  O   HOH S  63       0.019  -5.516   6.214  1.00 37.27           O  0.103
HETATM 2079  O   HOH S  64     -15.938 -18.495  -0.730  1.00 30.20           O  0.093
HETATM 2080  O   HOH S  65      11.238 -19.002 -17.120  1.00 32.58           O  0.097
HETATM 2081  O   HOH S  67       4.289  -9.772   6.650  1.00 25.87           O  0.086
HETATM 2082  O   HOH S  68      -3.806  -7.253  -5.206  1.00 30.76           O  0.094
HETATM 2083  O   HOH S  73      -4.137  -8.826 -12.399  1.00 33.24           O  0.098
HETATM 2084  O   HOH S  74      13.532 -37.644 -16.679  1.00 34.03           O  0.099
HETATM 2085  O   HOH S  75      -0.971 -18.687 -14.269  1.00 25.59           O  0.086
HETATM 2086  O   HOH S  76      11.465 -22.220  10.597  1.00 35.26           O  0.101
HETATM 2087  O   HOH S  77      -9.594 -23.994  -2.618  1.00 38.51           O  0.105
HETATM 2088  O   HOH S  78       0.284 -39.984   5.948  1.00 36.54           O  0.102
HETATM 2089  O   HOH S  81      15.819 -25.047  -5.919  1.00 22.50           O  0.080
HETATM 2090  O   HOH S  83       8.345 -29.044  14.164  1.00 31.40           O  0.095
HETATM 2091  O   HOH S  84       0.200 -41.928   0.399  1.00 32.24           O  0.096
HETATM 2092  O   HOH S  85       3.824 -37.312 -10.181  1.00 24.48           O  0.084
HETATM 2093  O   HOH S  86       0.222 -26.692 -15.690  1.00 38.24           O  0.105
HETATM 2094  O   HOH S  87      14.272 -23.851   6.696  1.00 31.80           O  0.095
HETATM 2095  O   HOH S  88       2.688 -29.789 -15.617  1.00 33.58           O  0.098
HETATM 2096  O   HOH S  89      10.467 -37.112 -17.386  1.00 30.39           O  0.093
HETATM 2097  O   HOH S  90     -14.269 -11.678  15.523  1.00 32.30           O  0.096
HETATM 2098  O   HOH S  92       9.564 -32.900  -3.036  1.00 29.40           O  0.092
HETATM 2099  O   HOH S  94     -19.224 -19.224  -0.000  0.58 36.60           O  0.102
HETATM 2100  O   HOH S  96     -14.651  -7.736  14.428  1.00 31.88           O  0.096
HETATM 2101  O   HOH S  97     -15.878 -20.991  17.104  1.00 44.74           O  0.113
HETATM 2102  O   HOH S 100     -10.602 -22.662   9.779  1.00 36.60           O  0.102
HETATM 2103  O   HOH S 103       3.246  -7.749 -11.918  1.00 26.73           O  0.088
HETATM 2104  O   HOH S 108       8.512 -19.233  10.842  1.00 32.86           O  0.097
HETATM 2105  O   HOH S 110     -12.179  -6.747  14.708  1.00 34.53           O  0.099
HETATM 2106  O   HOH S 111      -6.334 -18.194 -11.407  1.00 32.47           O  0.096
HETATM 2107  O   HOH S 113       9.787 -34.053  -7.117  1.00 28.44           O  0.090
HETATM 2108  O   HOH S 114      -5.875 -26.508   1.434  1.00 27.29           O  0.088
END
```
