# Supplementary material for: Time-series analysis of rhenium(I) organometallic covalent binding to a model protein for drug development
Source: IUCrJ. 2024 Apr 19;11(Pt 3):359–73. doi: 10.1107/S2052252524002598 (PMC11067751; doi:10.1107/S2052252524002598)
Supplement: Supplementary file 2 [file m-11-00359-sup2.zip › Week 3 - P2P1_1/P2P1_1_refine_41.pdf]

```
REMARK   3
REMARK   3 REFINEMENT.
REMARK   3   PROGRAM     : PHENIX (1.20.1_4487: ???)
REMARK   3   AUTHORS     : Adams,Afonine,Bunkoczi,Burnley,Chen,Dar,Davis,
REMARK   3               : Draizen,Echols,Gildea,Gros,Grosse-Kunstleve,Headd,
REMARK   3               : Hintze,Hung,Ioerger,Liebschner,McCoy,McKee,Moriarty,
REMARK   3               : Oeffner,Poon,Read,Richardson,Richardson,Sacchettini,
REMARK   3               : Sauter,Sobolev,Storoni,Terwilliger,Williams,Zwart
REMARK   3
REMARK   3  X-RAY DATA.
REMARK   3
REMARK   3  REFINEMENT TARGET : ML
REMARK   3
REMARK   3  DATA USED IN REFINEMENT.
REMARK   3   RESOLUTION RANGE HIGH (ANGSTROMS) : 1.68
REMARK   3   RESOLUTION RANGE LOW  (ANGSTROMS) : 40.49
REMARK   3   MIN(FOBS/SIGMA_FOBS)              : 1.33
REMARK   3   COMPLETENESS FOR RANGE        (%) : 98.27
REMARK   3   NUMBER OF REFLECTIONS             : 26427
REMARK   3   NUMBER OF REFLECTIONS (NON-ANOMALOUS) : 14397
REMARK   3
REMARK   3  FIT TO DATA USED IN REFINEMENT.
REMARK   3   R VALUE     (WORKING + TEST SET) : 0.1982
REMARK   3   R VALUE            (WORKING SET) : 0.1963
REMARK   3   FREE R VALUE                     : 0.2320
REMARK   3   FREE R VALUE TEST SET SIZE   (%) : 4.93
REMARK   3   FREE R VALUE TEST SET COUNT      : 1303
REMARK   3
REMARK   3  FIT TO DATA USED IN REFINEMENT (IN BINS).
REMARK   3   BIN  RESOLUTION RANGE  COMPL.    NWORK NFREE   RWORK  RFREE  CCWORK CCFREE
REMARK   3     1   40.49 -    3.49    1.00     2864   131  0.1570 0.1883   0.932  0.927
REMARK   3     2    3.49 -    2.77    1.00     2833   167  0.1958 0.2302   0.917  0.846
REMARK   3     3    2.77 -    2.42    1.00     2816   173  0.2044 0.2124   0.895  0.889
REMARK   3     4    2.42 -    2.20    1.00     2820   144  0.2042 0.2683   0.895  0.803
REMARK   3     5    2.20 -    2.04    1.00     2848   142  0.2209 0.3147   0.883  0.757
REMARK   3     6    2.04 -    1.92    1.00     2847   135  0.2373 0.2827   0.850  0.792
REMARK   3     7    1.92 -    1.83    0.98     2792   138  0.2783 0.2736   0.794  0.738
REMARK   3     8    1.83 -    1.75    0.98     2768   134  0.2977 0.3332   0.735  0.655
REMARK   3     9    1.75 -    1.68    0.90     2536   139  0.3278 0.3282   0.544  0.519
REMARK   3
REMARK   3  BULK SOLVENT MODELLING.
REMARK   3   METHOD USED        : FLAT BULK SOLVENT MODEL
REMARK   3   SOLVENT RADIUS     : 1.10
REMARK   3   SHRINKAGE RADIUS   : 0.90
REMARK   3   GRID STEP FACTOR   : 4.00
REMARK   3
REMARK   3  ERROR ESTIMATES.
REMARK   3   COORDINATE ERROR (MAXIMUM-LIKELIHOOD BASED)     : 0.25
REMARK   3   PHASE ERROR (DEGREES, MAXIMUM-LIKELIHOOD BASED) : 29.96
REMARK   3
REMARK   3  STRUCTURE FACTORS CALCULATION ALGORITHM : FFT
REMARK   3  B VALUES.
REMARK   3   FROM WILSON PLOT           (A**2) : 28.74
REMARK   3
REMARK   3  GEOMETRY RESTRAINTS LIBRARY: GEOSTD + MONOMER LIBRARY + CDL V1.2
REMARK   3  DEVIATIONS FROM IDEAL VALUES - RMSD. RMSZ FOR BONDS AND ANGLES.
REMARK   3    BOND      :  0.015   0.078   1105  Z= 0.950
REMARK   3    ANGLE     :  1.399   7.601   1503  Z= 0.810
REMARK   3    CHIRALITY :  0.075   0.285    147
REMARK   3    PLANARITY :  0.015   0.094    195
REMARK   3    DIHEDRAL  : 16.430  83.689    391
REMARK   3    MIN NONBONDED DISTANCE : 2.090
REMARK   3
REMARK   3  MOLPROBITY STATISTICS.
REMARK   3    ALL-ATOM CLASHSCORE : 2.39
REMARK   3    RAMACHANDRAN PLOT:
REMARK   3      OUTLIERS :  0.00 %
REMARK   3      ALLOWED  :  1.57 %
REMARK   3      FAVORED  : 98.43 %
REMARK   3    ROTAMER OUTLIERS :  1.85 %
REMARK   3    CBETA DEVIATIONS :  0.00 %
REMARK   3    PEPTIDE PLANE:
REMARK   3      CIS-PROLINE     : 0.00 %
REMARK   3      CIS-GENERAL     : 0.00 %
REMARK   3      TWISTED PROLINE : 0.00 %
REMARK   3      TWISTED GENERAL : 0.00 %
REMARK   3
REMARK   3  RAMA-Z (RAMACHANDRAN PLOT Z-SCORE):
```

```
REMARK   3  INTERPRETATION: BAD |RAMA-Z| > 3; SUSPICIOUS 2 < |RAMA-Z| < 3; GOOD |RAMA-Z| < 2.
REMARK   3  SCORES FOR WHOLE/HELIX/SHEET/LOOP ARE SCALED INDEPENDENTLY;
REMARK   3  THEREFORE, THE VALUES ARE NOT RELATED IN A SIMPLE MANNER.
REMARK   3    WHOLE: -1.21 (0.64), RESIDUES: 134
REMARK   3    HELIX: -1.63 (0.62), RESIDUES: 51
REMARK   3    SHEET: -0.68 (1.00), RESIDUES: 14
REMARK   3    LOOP :  0.05 (0.71), RESIDUES: 69
REMARK   3
REMARK   3                  min    max   mean <Bi,j>   iso aniso
REMARK   3     Overall:   18.39  84.54  33.68   5.99  1160     0
REMARK   3     Protein:   18.39  84.20  32.94   5.75  1031     0
REMARK   3     Water:     21.87  47.60  34.08    N/A    64     0
REMARK   3     Other:     26.41  84.54  44.95    N/A    65     0
REMARK   3     Chain  A:  18.39  84.20  33.54    N/A  1149     0
REMARK   3     Chain  C:  43.19  84.54  59.84    N/A     5     0
REMARK   3     Chain  B:  32.54  47.60  38.34    N/A     6     0
REMARK   3     Histogram:
REMARK   3         Values      Number of atoms
REMARK   3      18.39 - 25.00       182
REMARK   3      25.00 - 31.62       451
REMARK   3      31.62 - 38.23       274
REMARK   3      38.23 - 44.85       107
REMARK   3      44.85 - 51.46        56
REMARK   3      51.46 - 58.08        38
REMARK   3      58.08 - 64.69        15
REMARK   3      64.69 - 71.31        19
REMARK   3      71.31 - 77.92        12
REMARK   3      77.92 - 84.54         6
REMARK   3
REMARK   3
LINK         NE2 HIS A  15                 RE1  RI3 A2153
LINK         OD2 ASP A 119                 RE1  RII A2147
SSBOND   1 CYS A    6    CYS A  127
SSBOND   2 CYS A   30    CYS A  115
SSBOND   3 CYS A   64    CYS A   80
SSBOND   4 CYS A   76    CYS A   94
CRYST1   80.990   80.990   37.140  90.00  90.00  90.00 P 43 21 2
SCALE1      0.012347  0.000000  0.000000        0.00000
SCALE2      0.000000  0.012347  0.000000        0.00000
SCALE3      0.000000  0.000000  0.026925        0.00000
ATOM      1  N   LYS A   1       4.748  11.449   9.835  1.00 33.72           N  0.072
ATOM      2  CA  LYS A   1       3.747  11.872   8.796  1.00 36.05           C  0.075
ATOM      3  C   LYS A   1       3.783  13.362   8.582  1.00 27.00           C  0.065
ATOM      4  O   LYS A   1       3.740  14.136   9.583  1.00 30.62           O  0.069
ATOM      5  CB  LYS A   1       2.337  11.465   9.231  1.00 33.75           C  0.072
ATOM      6  CG  LYS A   1       1.206  12.159   8.455  1.00 35.63           C  0.074
ATOM      7  CD  LYS A   1      -0.100  11.448   8.851  1.00 39.85           C  0.079
ATOM      8  CE  LYS A   1      -1.273  11.985   8.076  1.00 51.64           C  0.090
ATOM      9  NZ  LYS A   1      -1.432  11.320   6.769  1.00 52.73           N  0.091
ATOM     10  H1  LYS A   1       4.718  10.565   9.933  1.00 40.46           H  0.079
ATOM     11  H2  LYS A   1       5.564  11.691   9.576  1.00 40.46           H  0.079
ATOM     12  H3  LYS A   1       4.554  11.840  10.610  1.00 40.46           H  0.079
ATOM     13  HA  LYS A   1       3.949  11.434   7.954  1.00 43.25           H  0.082
ATOM     14  HB2 LYS A   1       2.238  10.509   9.104  1.00 40.49           H  0.079
ATOM     15  HB3 LYS A   1       2.227  11.683  10.170  1.00 40.49           H  0.079
ATOM     16  HG2 LYS A   1       1.143  13.093   8.710  1.00 42.75           H  0.082
ATOM     17  HG3 LYS A   1       1.345  12.063   7.500  1.00 42.75           H  0.082
ATOM     18  HD2 LYS A   1      -0.020  10.500   8.662  1.00 47.81           H  0.086
ATOM     19  HD3 LYS A   1      -0.269  11.590   9.796  1.00 47.81           H  0.086
ATOM     20  HE2 LYS A   1      -2.084  11.839   8.587  1.00 61.97           H  0.098
ATOM     21  HE3 LYS A   1      -1.143  12.933   7.919  1.00 61.97           H  0.098
ATOM     22  HZ1 LYS A   1      -2.133  11.660   6.338  1.00 63.27           H  0.099
ATOM     23  HZ2 LYS A   1      -0.702  11.442   6.275  1.00 63.27           H  0.099
ATOM     24  HZ3 LYS A   1      -1.560  10.447   6.886  1.00 63.27           H  0.099
ATOM     25  N   VAL A   2       3.872  13.801   7.333  1.00 31.17           N  0.070
ATOM     26  CA  VAL A   2       3.745  15.201   6.978  1.00 28.80           C  0.067
ATOM     27  C   VAL A   2       2.319  15.375   6.491  1.00 45.60           C  0.084
ATOM     28  O   VAL A   2       1.947  14.828   5.447  1.00 32.17           O  0.071
ATOM     29  CB  VAL A   2       4.739  15.650   5.907  1.00 32.09           C  0.071
ATOM     30  CG1 VAL A   2       4.392  17.047   5.530  1.00 35.58           C  0.074
ATOM     31  CG2 VAL A   2       6.177  15.574   6.452  1.00 31.02           C  0.069
ATOM     32  H   VAL A   2       4.010  13.289   6.656  1.00 37.40           H  0.076
ATOM     33  HA  VAL A   2       3.872  15.751   7.767  1.00 34.56           H  0.073
ATOM     34  HB  VAL A   2       4.665  15.082   5.124  1.00 38.50           H  0.077
ATOM     35 HG11 VAL A   2       5.173  17.467   5.137  1.00 42.68           H  0.082
ATOM     36 HG12 VAL A   2       3.667  17.034   4.886  1.00 42.68           H  0.082
ATOM     37 HG13 VAL A   2       4.124  17.533   6.326  1.00 42.68           H  0.082
ATOM     38 HG21 VAL A   2       6.793  15.858   5.758  1.00 37.22           H  0.076
```

ATOM	39	HG22	VAL	A	2	6.254	16.159	7.222	1.00	37.22	H	0.076
ATOM	40	HG23	VAL	A	2	6.368	14.659	6.711	1.00	37.22	H	0.076
ATOM	41	N	PHE	A	3	1.531	16.146	7.238	1.00	33.40	N	0.072
ATOM	42	CA	PHE	A	3	0.132	16.368	6.876	1.00	33.98	C	0.073
ATOM	43	C	PHE	A	3	0.007	17.446	5.808	1.00	35.21	C	0.074
ATOM	44	O	PHE	A	3	0.819	18.377	5.704	1.00	31.65	O	0.070
ATOM	45	CB	PHE	A	3	-0.711	16.839	8.078	1.00	31.20	C	0.070
ATOM	46	CG	PHE	A	3	-1.217	15.767	8.942	1.00	27.19	C	0.065
ATOM	47	CD1	PHE	A	3	-0.426	15.234	9.957	1.00	29.26	C	0.067
ATOM	48	CD2	PHE	A	3	-2.552	15.293	8.819	1.00	25.31	C	0.063
ATOM	49	CE1	PHE	A	3	-0.930	14.247	10.770	1.00	32.17	C	0.071
ATOM	50	CE2	PHE	A	3	-3.040	14.309	9.631	1.00	30.53	C	0.069
ATOM	51	CZ	PHE	A	3	-2.214	13.773	10.622	1.00	36.57	C	0.075
ATOM	52	H	PHE	A	3	1.780	16.550	7.955	1.00	40.07	H	0.079
ATOM	53	HA	PHE	A	3	-0.250	15.545	6.533	1.00	40.76	H	0.080
ATOM	54	HB2	PHE	A	3	-0.167	17.425	8.627	1.00	37.44	H	0.076
ATOM	55	HB3	PHE	A	3	-1.477	17.328	7.740	1.00	37.44	H	0.076
ATOM	56	HD1	PHE	A	3	0.448	15.530	10.070	1.00	35.10	H	0.074
ATOM	57	HD2	PHE	A	3	-3.088	15.651	8.154	1.00	30.36	H	0.069
ATOM	58	HE1	PHE	A	3	-0.387	13.885	11.433	1.00	38.60	H	0.078
ATOM	59	HE2	PHE	A	3	-3.911	14.000	9.526	1.00	36.63	H	0.076
ATOM	60	HZ	PHE	A	3	-2.534	13.107	11.187	1.00	43.88	H	0.083
ATOM	61	N	GLY	A	4	-1.020	17.324	4.996	1.00	35.66	N	0.075
ATOM	62	CA	GLY	A	4	-1.454	18.450	4.237	1.00	32.44	C	0.071
ATOM	63	C	GLY	A	4	-2.323	19.358	5.087	1.00	27.53	C	0.065
ATOM	64	O	GLY	A	4	-2.928	18.927	6.055	1.00	32.62	O	0.071
ATOM	65	H	GLY	A	4	-1.474	16.604	4.872	1.00	42.78	H	0.082
ATOM	66	HA2	GLY	A	4	-0.688	18.954	3.920	1.00	38.92	H	0.078
ATOM	67	HA3	GLY	A	4	-1.970	18.151	3.472	1.00	38.92	H	0.078
ATOM	68	N	ARG	A	5	-2.346	20.635	4.724	1.00	30.63	N	0.069
ATOM	69	CA	ARG	A	5	-3.143	21.613	5.451	1.00	30.07	C	0.068
ATOM	70	C	ARG	A	5	-4.576	21.128	5.660	1.00	35.28	C	0.074
ATOM	71	O	ARG	A	5	-5.041	21.009	6.800	1.00	27.14	O	0.065
ATOM	72	CB	ARG	A	5	-3.072	22.913	4.673	1.00	36.89	C	0.076
ATOM	73	CG	ARG	A	5	-3.904	24.042	5.192	1.00	35.55	C	0.074
ATOM	74	CD	ARG	A	5	-3.645	25.261	4.301	1.00	43.63	C	0.082
ATOM	75	NE	ARG	A	5	-4.225	25.140	2.967	1.00	45.76	N	0.084
ATOM	76	CZ	ARG	A	5	-5.495	25.409	2.657	1.00	38.15	C	0.077
ATOM	77	NH1	ARG	A	5	-6.377	25.740	3.576	1.00	37.02	N	0.076
ATOM	78	NH2	ARG	A	5	-5.888	25.351	1.384	1.00	45.92	N	0.085
ATOM	79	H	ARG	A	5	-1.908	20.961	4.059	1.00	36.75	H	0.076
ATOM	80	HA	ARG	A	5	-2.746	21.762	6.323	1.00	36.08	H	0.075
ATOM	81	HB2	ARG	A	5	-2.149	23.213	4.668	1.00	44.26	H	0.083
ATOM	82	HB3	ARG	A	5	-3.358	22.738	3.763	1.00	44.26	H	0.083
ATOM	83	HG2	ARG	A	5	-4.845	23.812	5.144	1.00	42.65	H	0.081
ATOM	84	HG3	ARG	A	5	-3.644	24.255	6.102	1.00	42.65	H	0.081
ATOM	85	HD2	ARG	A	5	-4.029	26.045	4.723	1.00	52.35	H	0.090
ATOM	86	HD3	ARG	A	5	-2.688	25.378	4.199	1.00	52.35	H	0.090
ATOM	87	HE	ARG	A	5	-3.709	24.875	2.332	1.00	54.90	H	0.092
ATOM	88	HH11	ARG	A	5	-6.143	25.787	4.402	1.00	44.41	H	0.083
ATOM	89	HH12	ARG	A	5	-7.190	25.908	3.350	1.00	44.41	H	0.083
ATOM	90	HH21	ARG	A	5	-5.326	25.135	0.770	1.00	55.09	H	0.093
ATOM	91	HH22	ARG	A	5	-6.706	25.519	1.178	1.00	55.09	H	0.093
ATOM	92	N	CYS	A	6	-5.315	20.849	4.578	1.00	32.12	N	0.071
ATOM	93	CA	CYS	A	6	-6.730	20.492	4.774	1.00	35.75	C	0.075
ATOM	94	C	CYS	A	6	-6.901	19.157	5.513	1.00	30.55	C	0.069
ATOM	95	O	CYS	A	6	-7.826	19.009	6.307	1.00	31.75	O	0.070
ATOM	96	CB	CYS	A	6	-7.474	20.494	3.428	1.00	35.04	C	0.074
ATOM	97	SG	CYS	A	6	-7.775	22.251	2.813	1.00	35.93	S	0.075
ATOM	98	H	CYS	A	6	-5.041	20.857	3.763	1.00	38.53	H	0.077
ATOM	99	HA	CYS	A	6	-7.138	21.176	5.327	1.00	42.90	H	0.082
ATOM	100	HB2	CYS	A	6	-6.941	20.027	2.766	1.00	42.05	H	0.081
ATOM	101	HB3	CYS	A	6	-8.334	20.058	3.536	1.00	42.05	H	0.081
ATOM	102	N	GLU	A	7	-6.021	18.173	5.282	1.00	29.84	N	0.068
ATOM	103	CA	GLU	A	7	-6.133	16.918	6.015	1.00	37.45	C	0.076
ATOM	104	C	GLU	A	7	-5.923	17.136	7.512	1.00	26.33	C	0.064
ATOM	105	O	GLU	A	7	-6.589	16.503	8.361	1.00	28.79	O	0.067
ATOM	106	CB	GLU	A	7	-5.130	15.909	5.468	1.00	43.19	C	0.082
ATOM	107	CG	GLU	A	7	-4.904	14.729	6.441	1.00	55.87	C	0.093
ATOM	108	CD	GLU	A	7	-3.863	13.706	5.928	1.00	61.25	C	0.098
ATOM	109	OE1	GLU	A	7	-2.835	14.140	5.297	1.00	45.68	O	0.084
ATOM	110	OE2	GLU	A	7	-4.094	12.486	6.176	1.00	57.29	O	0.094
ATOM	111	H	GLU	A	7	-5.370	18.210	4.721	1.00	35.81	H	0.075
ATOM	112	HA	GLU	A	7	-7.024	16.556	5.887	1.00	44.94	H	0.084
ATOM	113	HB2	GLU	A	7	-5.464	15.551	4.631	1.00	51.82	H	0.090
ATOM	114	HB3	GLU	A	7	-4.278	16.351	5.327	1.00	51.82	H	0.090
ATOM	115	HG2	GLU	A	7	-4.616	15.045	7.301	1.00	67.04	H	0.102
ATOM	116	HG3	GLU	A	7	-5.745	14.256	6.541	1.00	67.04	H	0.102

ATOM	117	N	LEU	A	8	-4.978	18.004	7.868	1.00	34.48	N	0.073
ATOM	118	CA	LEU	A	8	-4.760	18.246	9.291	1.00	27.98	C	0.066
ATOM	119	C	LEU	A	8	-5.947	18.982	9.904	1.00	26.00	C	0.064
ATOM	120	O	LEU	A	8	-6.350	18.686	11.039	1.00	28.99	O	0.067
ATOM	121	CB	LEU	A	8	-3.457	19.047	9.531	1.00	27.89	C	0.066
ATOM	122	CG	LEU	A	8	-3.133	19.316	11.021	1.00	28.38	C	0.066
ATOM	123	CD1	LEU	A	8	-2.997	17.999	11.807	1.00	33.67	C	0.072
ATOM	124	CD2	LEU	A	8	-1.867	20.164	11.080	1.00	29.13	C	0.067
ATOM	125	H	LEU	A	8	-4.471	18.447	7.333	1.00	41.38	H	0.080
ATOM	126	HA	LEU	A	8	-4.674	17.392	9.742	1.00	33.57	H	0.072
ATOM	127	HB2	LEU	A	8	-2.714	18.551	9.155	1.00	33.46	H	0.072
ATOM	128	HB3	LEU	A	8	-3.535	19.906	9.087	1.00	33.46	H	0.072
ATOM	129	HG	LEU	A	8	-3.855	19.829	11.417	1.00	34.05	H	0.073
ATOM	130	HD11	LEU	A	8	-2.666	18.197	12.697	1.00	40.40	H	0.079
ATOM	131	HD12	LEU	A	8	-3.867	17.573	11.865	1.00	40.40	H	0.079
ATOM	132	HD13	LEU	A	8	-2.373	17.418	11.344	1.00	40.40	H	0.079
ATOM	133	HD21	LEU	A	8	-1.583	20.244	12.004	1.00	34.95	H	0.074
ATOM	134	HD22	LEU	A	8	-1.174	19.732	10.556	1.00	34.95	H	0.074
ATOM	135	HD23	LEU	A	8	-2.058	21.042	10.714	1.00	34.95	H	0.074
ATOM	136	N	ALA	A	9	-6.460	19.997	9.205	1.00	29.26	N	0.067
ATOM	137	CA	ALA	A	9	-7.652	20.659	9.701	1.00	23.61	C	0.061
ATOM	138	C	ALA	A	9	-8.767	19.634	9.976	1.00	27.79	C	0.066
ATOM	139	O	ALA	A	9	-9.480	19.710	10.990	1.00	28.82	O	0.067
ATOM	140	CB	ALA	A	9	-8.144	21.677	8.685	1.00	31.85	C	0.070
ATOM	141	H	ALA	A	9	-6.145	20.308	8.467	1.00	35.10	H	0.074
ATOM	142	HA	ALA	A	9	-7.447	21.122	10.528	1.00	28.33	H	0.066
ATOM	143	HB1	ALA	A	9	-8.940	22.110	9.032	1.00	38.22	H	0.077
ATOM	144	HB2	ALA	A	9	-7.447	22.335	8.535	1.00	38.22	H	0.077
ATOM	145	HB3	ALA	A	9	-8.351	21.219	7.855	1.00	38.22	H	0.077
ATOM	146	N	ALA	A	10	-8.962	18.676	9.061	1.00	34.06	N	0.073
ATOM	147	CA	ALA	A	10	-10.019	17.691	9.244	1.00	31.86	C	0.070
ATOM	148	C	ALA	A	10	-9.783	16.828	10.474	1.00	35.13	C	0.074
ATOM	149	O	ALA	A	10	-10.702	16.561	11.263	1.00	29.41	O	0.068
ATOM	150	CB	ALA	A	10	-10.105	16.821	7.987	1.00	35.13	C	0.074
ATOM	151	H	ALA	A	10	-8.502	18.580	8.341	1.00	40.87	H	0.080
ATOM	152	HA	ALA	A	10	-10.867	18.150	9.354	1.00	38.22	H	0.077
ATOM	153	HB1	ALA	A	10	-10.808	16.164	8.106	1.00	42.15	H	0.081
ATOM	154	HB2	ALA	A	10	-10.306	17.387	7.225	1.00	42.15	H	0.081
ATOM	155	HB3	ALA	A	10	-9.254	16.375	7.853	1.00	42.15	H	0.081
ATOM	156	N	ALA	A	11	-8.566	16.329	10.619	1.00	31.83	N	0.070
ATOM	157	CA	ALA	A	11	-8.248	15.510	11.770	1.00	29.29	C	0.068
ATOM	158	C	ALA	A	11	-8.408	16.286	13.046	1.00	28.80	C	0.067
ATOM	159	O	ALA	A	11	-8.858	15.759	14.069	1.00	33.30	O	0.072
ATOM	160	CB	ALA	A	11	-6.789	15.016	11.661	1.00	35.39	C	0.074
ATOM	161	H	ALA	A	11	-7.914	16.449	10.071	1.00	38.19	H	0.077
ATOM	162	HA	ALA	A	11	-8.838	14.741	11.797	1.00	35.15	H	0.074
ATOM	163	HB1	ALA	A	11	-6.583	14.468	12.435	1.00	42.46	H	0.081
ATOM	164	HB2	ALA	A	11	-6.693	14.493	10.850	1.00	42.46	H	0.081
ATOM	165	HB3	ALA	A	11	-6.197	15.784	11.632	1.00	42.46	H	0.081
ATOM	166	N	MET	A	12	-7.910	17.524	13.048	1.00	27.31	N	0.065
ATOM	167	CA	MET	A	12	-8.014	18.348	14.248	1.00	28.48	C	0.067
ATOM	168	C	MET	A	12	-9.467	18.580	14.599	1.00	28.97	C	0.067
ATOM	169	O	MET	A	12	-9.818	18.627	15.782	1.00	30.09	O	0.068
ATOM	170	CB	MET	A	12	-7.271	19.612	13.912	1.00	26.85	C	0.065
ATOM	171	CG	MET	A	12	-5.803	19.597	14.435	1.00	24.04	C	0.061
ATOM	172	SD	MET	A	12	-4.986	21.156	13.915	1.00	26.24	S	0.064
ATOM	173	CE	MET	A	12	-3.378	20.986	14.725	1.00	24.91	C	0.062
ATOM	174	H	MET	A	12	-7.516	17.895	12.381	1.00	32.76	H	0.071
ATOM	175	HA	MET	A	12	-7.565	17.909	14.987	1.00	34.17	H	0.073
ATOM	176	HB2	MET	A	12	-7.253	19.742	12.952	1.00	32.22	H	0.071
ATOM	177	HB3	MET	A	12	-7.725	20.359	14.333	1.00	32.22	H	0.071
ATOM	178	HG2	MET	A	12	-5.796	19.544	15.403	1.00	28.84	H	0.067
ATOM	179	HG3	MET	A	12	-5.324	18.849	14.046	1.00	28.84	H	0.067
ATOM	180	HE1	MET	A	12	-2.839	21.766	14.522	1.00	29.88	H	0.068
ATOM	181	HE2	MET	A	12	-3.513	20.917	15.683	1.00	29.88	H	0.068
ATOM	182	HE3	MET	A	12	-2.941	20.186	14.393	1.00	29.88	H	0.068
ATOM	183	N	LYS	A	13	-10.332	18.742	13.593	1.00	27.00	N	0.065
ATOM	184	CA	LYS	A	13	-11.779	18.838	13.870	1.00	31.05	C	0.070
ATOM	185	C	LYS	A	13	-12.348	17.527	14.438	1.00	27.93	C	0.066
ATOM	186	O	LYS	A	13	-13.155	17.567	15.369	1.00	32.82	O	0.071
ATOM	187	CB	LYS	A	13	-12.556	19.265	12.616	1.00	45.63	C	0.084
ATOM	188	CG	LYS	A	13	-14.023	19.811	12.881	1.00	40.00	C	0.079
ATOM	189	CD	LYS	A	13	-14.810	20.029	11.532	1.00	45.21	C	0.084
ATOM	190	CE	LYS	A	13	-16.312	20.363	11.666	1.00	55.28	C	0.093
ATOM	191	NZ	LYS	A	13	-16.669	21.635	10.903	1.00	65.56	N	0.101
ATOM	192	H	LYS	A	13	-10.119	18.798	12.762	1.00	32.39	H	0.071
ATOM	193	HA	LYS	A	13	-11.915	19.525	14.541	1.00	37.25	H	0.076
ATOM	194	HB2	LYS	A	13	-12.059	19.969	12.171	1.00	54.75	H	0.092

ATOM	195	HB3	LYS	A	13	-12.632	18.499	12.026	1.00	54.75	H	0.092
ATOM	196	HG2	LYS	A	13	-14.512	19.168	13.418	1.00	47.99	H	0.086
ATOM	197	HG3	LYS	A	13	-13.969	20.662	13.343	1.00	47.99	H	0.086
ATOM	198	HD2	LYS	A	13	-14.393	20.761	11.051	1.00	54.25	H	0.092
ATOM	199	HD3	LYS	A	13	-14.741	19.218	11.005	1.00	54.25	H	0.092
ATOM	200	HE2	LYS	A	13	-16.836	19.633	11.302	1.00	66.33	H	0.102
ATOM	201	HE3	LYS	A	13	-16.528	20.499	12.602	1.00	66.33	H	0.102
ATOM	202	HZ1	LYS	A	13	-17.537	21.810	10.993	1.00	78.66	H	0.111
ATOM	203	HZ2	LYS	A	13	-16.201	22.322	11.222	1.00	78.66	H	0.111
ATOM	204	HZ3	LYS	A	13	-16.482	21.534	10.039	1.00	78.66	H	0.111
ATOM	205	N	ARG	A	14	-12.032	16.365	13.831	1.00	37.07	N	0.076
ATOM	206	CA	ARG	A	14	-12.492	15.092	14.408	1.00	34.34	C	0.073
ATOM	207	C	ARG	A	14	-12.103	14.992	15.882	1.00	46.19	C	0.085
ATOM	208	O	ARG	A	14	-12.789	14.367	16.701	1.00	39.31	O	0.078
ATOM	209	CB	ARG	A	14	-11.890	13.883	13.676	1.00	44.76	C	0.083
ATOM	210	CG	ARG	A	14	-12.504	13.515	12.317	1.00	64.13	C	0.100
ATOM	211	CD	ARG	A	14	-13.831	12.711	12.436	1.00	75.78	C	0.109
ATOM	212	NE	ARG	A	14	-13.723	11.324	12.905	1.00	76.39	N	0.109
ATOM	213	CZ	ARG	A	14	-14.675	10.685	13.582	1.00	78.56	C	0.111
ATOM	214	NH1	ARG	A	14	-15.773	11.308	13.985	1.00	64.87	N	0.101
ATOM	215	NH2	ARG	A	14	-14.528	9.387	13.853	1.00	64.85	N	0.100
ATOM	216	H	ARG	A	14	-11.569	16.291	13.110	1.00	44.48	H	0.083
ATOM	217	HA	ARG	A	14	-13.458	15.040	14.342	1.00	41.20	H	0.080
ATOM	218	HB2	ARG	A	14	-10.948	14.060	13.526	1.00	53.71	H	0.091
ATOM	219	HB3	ARG	A	14	-11.981	13.107	14.250	1.00	53.71	H	0.091
ATOM	220	HG2	ARG	A	14	-12.693	14.330	11.827	1.00	76.94	H	0.109
ATOM	221	HG3	ARG	A	14	-11.870	12.972	11.823	1.00	76.94	H	0.109
ATOM	222	HD2	ARG	A	14	-14.405	13.189	13.053	1.00	90.94	H	0.119
ATOM	223	HD3	ARG	A	14	-14.250	12.687	11.562	1.00	90.94	H	0.119
ATOM	224	HE	ARG	A	14	-12.997	10.896	12.731	1.00	91.67	H	0.119
ATOM	225	HH11	ARG	A	14	-15.890	12.142	13.815	1.00	77.84	H	0.110
ATOM	226	HH12	ARG	A	14	-16.374	10.874	14.421	1.00	77.84	H	0.110
ATOM	227	HH21	ARG	A	14	-13.819	8.972	13.599	1.00	77.81	H	0.110
ATOM	228	HH22	ARG	A	14	-15.135	8.969	14.295	1.00	77.81	H	0.110
ATOM	229	N	HIS	A	15	-10.950	15.566	16.205	1.00	38.73	N	0.078
ATOM	230	CA	HIS	A	15	-10.376	15.440	17.560	1.00	31.37	C	0.070
ATOM	231	C	HIS	A	15	-10.831	16.556	18.499	1.00	36.75	C	0.076
ATOM	232	O	HIS	A	15	-10.219	16.651	19.550	1.00	33.31	O	0.072
ATOM	233	CB	HIS	A	15	-8.860	15.279	17.402	1.00	42.77	C	0.082
ATOM	234	CG	HIS	A	15	-8.438	13.907	16.990	1.00	31.80	C	0.070
ATOM	235	ND1	HIS	A	15	-8.407	12.858	17.861	1.00	46.93	N	0.085
ATOM	236	CD2	HIS	A	15	-8.051	13.394	15.808	1.00	49.83	C	0.088
ATOM	237	CE1	HIS	A	15	-7.934	11.790	17.271	1.00	51.64	C	0.090
ATOM	238	NE2	HIS	A	15	-7.702	12.098	16.016	1.00	47.86	N	0.086
ATOM	239	H	HIS	A	15	-10.473	16.035	15.665	1.00	46.47	H	0.085
ATOM	240	HA	HIS	A	15	-10.702	14.611	17.943	1.00	37.63	H	0.077
ATOM	241	HB2	HIS	A	15	-8.547	15.902	16.727	1.00	51.32	H	0.089
ATOM	242	HB3	HIS	A	15	-8.436	15.474	18.251	1.00	51.32	H	0.089
ATOM	243	HD1	HIS	A	15	-8.591	12.913	18.699	1.00	56.31	H	0.094
ATOM	244	HD2	HIS	A	15	-7.949	13.867	15.014	1.00	59.79	H	0.096
ATOM	245	HE1	HIS	A	15	-7.828	10.951	17.658	1.00	61.96	H	0.098
ATOM	246	N	GLY	A	16	-11.826	17.390	18.153	1.00	32.39	N	0.071
ATOM	247	CA	GLY	A	16	-12.392	18.285	19.131	1.00	28.42	C	0.067
ATOM	248	C	GLY	A	16	-11.667	19.576	19.371	1.00	28.57	C	0.067
ATOM	249	O	GLY	A	16	-11.927	20.226	20.374	1.00	32.26	O	0.071
ATOM	250	H	GLY	A	16	-12.175	17.445	17.369	1.00	38.86	H	0.078
ATOM	251	HA2	GLY	A	16	-13.297	18.505	18.859	1.00	34.10	H	0.073
ATOM	252	HA3	GLY	A	16	-12.446	17.819	19.980	1.00	34.10	H	0.073
ATOM	253	N	LEU	A	17	-10.788	19.978	18.457	1.00	33.21	N	0.072
ATOM	254	CA	LEU	A	17	-10.013	21.241	18.601	1.00	30.63	C	0.069
ATOM	255	C	LEU	A	17	-10.760	22.443	17.995	1.00	34.46	C	0.073
ATOM	256	O	LEU	A	17	-10.349	23.559	18.311	1.00	34.39	O	0.073
ATOM	257	CB	LEU	A	17	-8.640	21.093	17.940	1.00	26.22	C	0.064
ATOM	258	CG	LEU	A	17	-7.538	20.464	18.787	1.00	27.70	C	0.066
ATOM	259	CD1	LEU	A	17	-6.204	20.605	18.077	1.00	24.69	C	0.062
ATOM	260	CD2	LEU	A	17	-7.472	21.085	20.173	1.00	35.44	C	0.074
ATOM	261	H	LEU	A	17	-10.612	19.544	17.736	1.00	39.84	H	0.079
ATOM	262	HA	LEU	A	17	-9.895	21.425	19.544	1.00	36.76	H	0.076
ATOM	263	HB2	LEU	A	17	-8.743	20.542	17.148	1.00	31.46	H	0.070
ATOM	264	HB3	LEU	A	17	-8.333	21.975	17.679	1.00	31.46	H	0.070
ATOM	265	HG	LEU	A	17	-7.722	19.517	18.892	1.00	33.24	H	0.072
ATOM	266	HD11	LEU	A	17	-5.698	19.785	18.188	1.00	29.63	H	0.068
ATOM	267	HD12	LEU	A	17	-6.367	20.771	17.136	1.00	29.63	H	0.068
ATOM	268	HD13	LEU	A	17	-5.719	21.351	18.463	1.00	29.63	H	0.068
ATOM	269	HD21	LEU	A	17	-6.600	20.904	20.558	1.00	42.52	H	0.081
ATOM	270	HD22	LEU	A	17	-7.608	22.042	20.096	1.00	42.52	H	0.081
ATOM	271	HD23	LEU	A	17	-8.166	20.696	20.728	1.00	42.52	H	0.081
ATOM	272	N	ASP	A	18	-11.784	22.252	17.157	1.00	31.40	N	0.070

ATOM	273	CA	ASP	A	18	-12.534	23.398	16.620	1.00	37.17	C	0.076
ATOM	274	C	ASP	A	18	-13.281	24.070	17.763	1.00	33.22	C	0.072
ATOM	275	O	ASP	A	18	-14.182	23.484	18.399	1.00	30.22	O	0.069
ATOM	276	CB	ASP	A	18	-13.483	23.016	15.497	1.00	40.04	C	0.079
ATOM	277	CG	ASP	A	18	-14.098	24.247	14.775	1.00	53.51	C	0.091
ATOM	278	OD1	ASP	A	18	-13.533	25.395	14.835	1.00	43.26	O	0.082
ATOM	279	OD2	ASP	A	18	-15.148	24.011	14.103	1.00	55.95	O	0.093
ATOM	280	H	ASP	A	18	-12.062	21.484	16.887	1.00	37.68	H	0.077
ATOM	281	HA	ASP	A	18	-11.902	24.041	16.262	1.00	44.60	H	0.083
ATOM	282	HB2	ASP	A	18	-12.998	22.495	14.838	1.00	48.04	H	0.086
ATOM	283	HB3	ASP	A	18	-14.211	22.491	15.865	1.00	48.04	H	0.086
ATOM	284	N	ASN	A	19	-12.852	25.287	18.030	1.00	28.11	N	0.066
ATOM	285	CA	ASN	A	19	-13.337	26.158	19.084	1.00	31.89	C	0.070
ATOM	286	C	ASN	A	19	-13.038	25.630	20.483	1.00	36.82	C	0.076
ATOM	287	O	ASN	A	19	-13.700	26.014	21.452	1.00	29.96	O	0.068
ATOM	288	CB	ASN	A	19	-14.825	26.441	18.953	1.00	31.05	C	0.070
ATOM	289	CG	ASN	A	19	-15.105	27.944	18.943	1.00	46.91	C	0.085
ATOM	290	OD1	ASN	A	19	-14.447	28.716	18.215	1.00	57.89	O	0.095
ATOM	291	ND2	ASN	A	19	-16.010	28.381	19.815	1.00	60.53	N	0.097
ATOM	292	H	ASN	A	19	-12.226	25.660	17.573	1.00	33.72	H	0.072
ATOM	293	HA	ASN	A	19	-12.878	27.008	19.001	1.00	38.26	H	0.077
ATOM	294	HB2	ASN	A	19	-15.149	26.069	18.118	1.00	37.25	H	0.076
ATOM	295	HB3	ASN	A	19	-15.300	26.046	19.701	1.00	37.25	H	0.076
ATOM	296	HD21	ASN	A	19	-16.433	27.821	20.312	1.00	72.63	H	0.106
ATOM	297	HD22	ASN	A	19	-16.199	29.219	19.855	1.00	72.63	H	0.106
ATOM	298	N	TYR	A	20	-12.016	24.797	20.631	1.00	28.00	N	0.066
ATOM	299	CA	TYR	A	20	-11.650	24.333	21.970	1.00	25.03	C	0.062
ATOM	300	C	TYR	A	20	-11.047	25.503	22.735	1.00	31.22	C	0.070
ATOM	301	O	TYR	A	20	-10.115	26.176	22.243	1.00	27.56	O	0.066
ATOM	302	CB	TYR	A	20	-10.674	23.184	21.913	1.00	27.98	C	0.066
ATOM	303	CG	TYR	A	20	-10.429	22.561	23.278	1.00	28.67	C	0.067
ATOM	304	CD1	TYR	A	20	-9.428	23.035	24.115	1.00	29.82	C	0.068
ATOM	305	CD2	TYR	A	20	-11.274	21.543	23.752	1.00	35.09	C	0.074
ATOM	306	CE1	TYR	A	20	-9.233	22.484	25.397	1.00	30.27	C	0.069
ATOM	307	CE2	TYR	A	20	-11.087	20.995	25.023	1.00	32.39	C	0.071
ATOM	308	CZ	TYR	A	20	-10.087	21.497	25.856	1.00	28.42	C	0.067
ATOM	309	OH	TYR	A	20	-9.892	20.941	27.080	1.00	35.31	O	0.074
ATOM	310	H	TYR	A	20	-11.527	24.490	19.993	1.00	33.59	H	0.072
ATOM	311	HA	TYR	A	20	-12.446	24.037	22.438	1.00	30.04	H	0.068
ATOM	312	HB2	TYR	A	20	-11.028	22.496	21.328	1.00	33.57	H	0.072
ATOM	313	HB3	TYR	A	20	-9.825	23.506	21.572	1.00	33.57	H	0.072
ATOM	314	HD1	TYR	A	20	-8.871	23.719	23.821	1.00	35.78	H	0.075
ATOM	315	HD2	TYR	A	20	-11.961	21.226	23.211	1.00	42.10	H	0.081
ATOM	316	HE1	TYR	A	20	-8.556	22.808	25.946	1.00	36.32	H	0.075
ATOM	317	HE2	TYR	A	20	-11.649	20.319	25.326	1.00	38.86	H	0.078
ATOM	318	HH	TYR	A	20	-10.480	20.360	27.229	1.00	42.36	H	0.081
ATOM	319	N	ARG	A	21	-11.515	25.697	23.956	1.00	31.14	N	0.070
ATOM	320	CA	ARG	A	21	-11.157	26.880	24.754	1.00	27.39	C	0.065
ATOM	321	C	ARG	A	21	-11.347	28.163	23.968	1.00	29.39	C	0.068
ATOM	322	O	ARG	A	21	-10.636	29.135	24.205	1.00	26.86	O	0.065
ATOM	323	CB	ARG	A	21	-9.712	26.845	25.286	1.00	31.12	C	0.070
ATOM	324	CG	ARG	A	21	-9.617	26.088	26.555	1.00	37.31	C	0.076
ATOM	325	CD	ARG	A	21	-10.019	26.869	27.764	1.00	35.74	C	0.075
ATOM	326	NE	ARG	A	21	-10.372	25.851	28.754	1.00	66.08	N	0.101
ATOM	327	CZ	ARG	A	21	-10.393	26.027	30.065	1.00	77.22	C	0.110
ATOM	328	NH1	ARG	A	21	-10.010	27.169	30.613	1.00	62.44	N	0.099
ATOM	329	NH2	ARG	A	21	-10.781	25.019	30.848	1.00	66.93	N	0.102
ATOM	330	H	ARG	A	21	-12.049	25.156	24.358	1.00	37.36	H	0.076
ATOM	331	HA	ARG	A	21	-11.748	26.920	25.522	1.00	32.87	H	0.072
ATOM	332	HB2	ARG	A	21	-9.142	26.412	24.632	1.00	37.33	H	0.076
ATOM	333	HB3	ARG	A	21	-9.397	27.745	25.460	1.00	37.33	H	0.076
ATOM	334	HG2	ARG	A	21	-10.195	25.312	26.494	1.00	44.77	H	0.083
ATOM	335	HG3	ARG	A	21	-8.698	25.803	26.680	1.00	44.77	H	0.083
ATOM	336	HD2	ARG	A	21	-9.274	27.397	28.091	1.00	42.88	H	0.082
ATOM	337	HD3	ARG	A	21	-10.794	27.420	27.574	1.00	42.88	H	0.082
ATOM	338	HE	ARG	A	21	-10.642	25.090	28.459	1.00	79.29	H	0.111
ATOM	339	HH11	ARG	A	21	-9.757	27.823	30.116	1.00	74.93	H	0.108
ATOM	340	HH12	ARG	A	21	-10.026	27.261	31.468	1.00	74.93	H	0.108
ATOM	341	HH21	ARG	A	21	-11.026	24.272	30.498	1.00	80.31	H	0.112
ATOM	342	HH22	ARG	A	21	-10.793	25.118	31.702	1.00	80.31	H	0.112
ATOM	343	N	GLY	A	22	-12.286	28.152	23.016	1.00	32.85	N	0.072
ATOM	344	CA	GLY	A	22	-12.599	29.316	22.216	1.00	26.43	C	0.064
ATOM	345	C	GLY	A	22	-11.666	29.571	21.041	1.00	28.97	C	0.067
ATOM	346	O	GLY	A	22	-11.770	30.631	20.386	1.00	27.63	O	0.066
ATOM	347	H	GLY	A	22	-12.761	27.463	22.819	1.00	39.41	H	0.078
ATOM	348	HA2	GLY	A	22	-13.499	29.222	21.866	1.00	31.71	H	0.070
ATOM	349	HA3	GLY	A	22	-12.581	30.101	22.785	1.00	31.71	H	0.070
ATOM	350	N	TYR	A	23	-10.791	28.625	20.714	1.00	25.89	N	0.063

ATOM	351	CA	TYR	A	23	-9.850	28.805	19.621	1.00	22.46	C	0.059
ATOM	352	C	TYR	A	23	-10.343	28.085	18.373	1.00	23.39	C	0.060
ATOM	353	O	TYR	A	23	-10.361	26.849	18.320	1.00	26.66	O	0.064
ATOM	354	CB	TYR	A	23	-8.462	28.304	20.011	1.00	23.70	C	0.061
ATOM	355	CG	TYR	A	23	-7.779	29.266	20.986	1.00	22.36	C	0.059
ATOM	356	CD1	TYR	A	23	-7.012	30.361	20.538	1.00	23.29	C	0.060
ATOM	357	CD2	TYR	A	23	-7.946	29.084	22.361	1.00	23.00	C	0.060
ATOM	358	CE1	TYR	A	23	-6.420	31.227	21.450	1.00	20.90	C	0.057
ATOM	359	CE2	TYR	A	23	-7.358	29.906	23.236	1.00	22.36	C	0.059
ATOM	360	CZ	TYR	A	23	-6.595	30.969	22.789	1.00	25.31	C	0.063
ATOM	361	OH	TYR	A	23	-6.026	31.849	23.681	1.00	25.91	O	0.064
ATOM	362	H	TYR	A	23	-10.724	27.866	21.113	1.00	31.06	H	0.070
ATOM	363	HA	TYR	A	23	-9.778	29.749	19.413	1.00	26.94	H	0.065
ATOM	364	HB2	TYR	A	23	-8.543	27.439	20.442	1.00	28.44	H	0.067
ATOM	365	HB3	TYR	A	23	-7.911	28.234	19.216	1.00	28.44	H	0.067
ATOM	366	HD1	TYR	A	23	-6.900	30.504	19.626	1.00	27.94	H	0.066
ATOM	367	HD2	TYR	A	23	-8.446	28.364	22.672	1.00	27.59	H	0.066
ATOM	368	HE1	TYR	A	23	-5.907	31.948	21.163	1.00	25.07	H	0.062
ATOM	369	HE2	TYR	A	23	-7.475	29.772	24.149	1.00	26.83	H	0.065
ATOM	370	HH	TYR	A	23	-6.240	31.640	24.466	1.00	31.09	H	0.070
ATOM	371	N	SER	A	24	-10.727	28.866	17.371	1.00	25.93	N	0.064
ATOM	372	CA	SER	A	24	-11.200	28.317	16.110	1.00	28.42	C	0.067
ATOM	373	C	SER	A	24	-10.114	27.464	15.440	1.00	31.34	C	0.070
ATOM	374	O	SER	A	24	-8.896	27.655	15.638	1.00	25.26	O	0.063
ATOM	375	CB	SER	A	24	-11.622	29.467	15.194	1.00	27.44	C	0.065
ATOM	376	OG	SER	A	24	-10.475	30.123	14.715	1.00	28.79	O	0.067
ATOM	377	H	SER	A	24	-10.723	29.726	17.398	1.00	31.11	H	0.070
ATOM	378	HA	SER	A	24	-11.974	27.755	16.273	1.00	34.09	H	0.073
ATOM	379	HB2	SER	A	24	-12.125	29.112	14.444	1.00	32.92	H	0.072
ATOM	380	HB3	SER	A	24	-12.164	30.095	15.696	1.00	32.92	H	0.072
ATOM	381	HG	SER	A	24	-10.085	29.645	14.144	1.00	34.54	H	0.073
ATOM	382	N	LEU	A	25	-10.592	26.542	14.597	1.00	25.99	N	0.064
ATOM	383	CA	LEU	A	25	-9.746	25.560	13.937	1.00	21.17	C	0.057
ATOM	384	C	LEU	A	25	-8.536	26.169	13.266	1.00	20.08	C	0.056
ATOM	385	O	LEU	A	25	-7.455	25.539	13.225	1.00	25.32	O	0.063
ATOM	386	CB	LEU	A	25	-10.556	24.806	12.879	1.00	32.95	C	0.072
ATOM	387	CG	LEU	A	25	-9.915	23.524	12.439	1.00	33.39	C	0.072
ATOM	388	CD1	LEU	A	25	-9.855	22.550	13.625	1.00	30.68	C	0.069
ATOM	389	CD2	LEU	A	25	-10.822	22.940	11.336	1.00	30.66	C	0.069
ATOM	390	H	LEU	A	25	-11.424	26.469	14.391	1.00	31.19	H	0.070
ATOM	391	HA	LEU	A	25	-9.432	24.928	14.601	1.00	25.40	H	0.063
ATOM	392	HB2	LEU	A	25	-11.429	24.594	13.244	1.00	39.53	H	0.078
ATOM	393	HB3	LEU	A	25	-10.655	25.373	12.098	1.00	39.53	H	0.078
ATOM	394	HG	LEU	A	25	-9.024	23.680	12.089	1.00	40.07	H	0.079
ATOM	395	HD11	LEU	A	25	-9.749	21.648	13.284	1.00	36.81	H	0.076
ATOM	396	HD12	LEU	A	25	-9.100	22.780	14.188	1.00	36.81	H	0.076
ATOM	397	HD13	LEU	A	25	-10.680	22.615	14.130	1.00	36.81	H	0.076
ATOM	398	HD21	LEU	A	25	-10.441	22.104	11.025	1.00	36.79	H	0.076
ATOM	399	HD22	LEU	A	25	-11.706	22.785	11.704	1.00	36.79	H	0.076
ATOM	400	HD23	LEU	A	25	-10.875	23.573	10.603	1.00	36.79	H	0.076
ATOM	401	N	GLY	A	26	-8.701	27.341	12.648	1.00	25.85	N	0.063
ATOM	402	CA	GLY	A	26	-7.576	27.907	11.927	1.00	26.13	C	0.064
ATOM	403	C	GLY	A	26	-6.413	28.295	12.822	1.00	24.70	C	0.062
ATOM	404	O	GLY	A	26	-5.248	28.284	12.378	1.00	24.73	O	0.062
ATOM	405	H	GLY	A	26	-9.424	27.806	12.633	1.00	31.02	H	0.069
ATOM	406	HA2	GLY	A	26	-7.256	27.262	11.277	1.00	31.34	H	0.070
ATOM	407	HA3	GLY	A	26	-7.869	28.699	11.450	1.00	31.34	H	0.070
ATOM	408	N	ASN	A	27	-6.696	28.643	14.083	1.00	23.91	N	0.061
ATOM	409	CA	ASN	A	27	-5.618	28.903	15.018	1.00	21.89	C	0.058
ATOM	410	C	ASN	A	27	-4.719	27.676	15.196	1.00	20.56	C	0.057
ATOM	411	O	ASN	A	27	-3.494	27.808	15.285	1.00	25.39	O	0.063
ATOM	412	CB	ASN	A	27	-6.181	29.345	16.354	1.00	21.20	C	0.057
ATOM	413	CG	ASN	A	27	-6.641	30.759	16.313	1.00	23.62	C	0.061
ATOM	414	OD1	ASN	A	27	-5.828	31.681	16.333	1.00	21.84	O	0.058
ATOM	415	ND2	ASN	A	27	-7.968	30.957	16.258	1.00	29.71	N	0.068
ATOM	416	H	ASN	A	27	-7.487	28.732	14.408	1.00	28.68	H	0.067
ATOM	417	HA	ASN	A	27	-5.070	29.625	14.672	1.00	26.26	H	0.064
ATOM	418	HB2	ASN	A	27	-6.938	28.785	16.584	1.00	25.44	H	0.063
ATOM	419	HB3	ASN	A	27	-5.491	29.271	17.031	1.00	25.44	H	0.063
ATOM	420	HD21	ASN	A	27	-8.283	31.757	16.233	1.00	35.65	H	0.075
ATOM	421	HD22	ASN	A	27	-8.503	30.284	16.246	1.00	35.65	H	0.075
ATOM	422	N	TRP	A	28	-5.335	26.490	15.356	1.00	22.58	N	0.059
ATOM	423	CA	TRP	A	28	-4.612	25.236	15.593	1.00	19.87	C	0.056
ATOM	424	C	TRP	A	28	-3.836	24.820	14.346	1.00	20.44	C	0.056
ATOM	425	O	TRP	A	28	-2.699	24.354	14.436	1.00	22.53	O	0.059
ATOM	426	CB	TRP	A	28	-5.610	24.155	16.038	1.00	23.11	C	0.060
ATOM	427	CG	TRP	A	28	-6.312	24.471	17.327	1.00	23.29	C	0.060
ATOM	428	CD1	TRP	A	28	-7.575	24.942	17.469	1.00	24.35	C	0.062

ATOM	429	CD2	TRP	A	28	-5.764	24.364	18.633	1.00	22.65	C	0.059
ATOM	430	NE1	TRP	A	28	-7.848	25.133	18.800	1.00	25.35	N	0.063
ATOM	431	CE2	TRP	A	28	-6.731	24.787	19.527	1.00	25.02	C	0.062
ATOM	432	CE3	TRP	A	28	-4.520	23.976	19.116	1.00	22.60	C	0.059
ATOM	433	CZ2	TRP	A	28	-6.521	24.792	20.925	1.00	24.52	C	0.062
ATOM	434	CZ3	TRP	A	28	-4.274	23.977	20.491	1.00	24.25	C	0.061
ATOM	435	CH2	TRP	A	28	-5.293	24.407	21.396	1.00	22.26	C	0.059
ATOM	436	H	TRP	A	28	-6.189	26.390	15.330	1.00	27.09	H	0.065
ATOM	437	HA	TRP	A	28	-3.975	25.370	16.312	1.00	23.84	H	0.061
ATOM	438	HB2	TRP	A	28	-6.286	24.052	15.350	1.00	27.73	H	0.066
ATOM	439	HB3	TRP	A	28	-5.132	23.319	16.158	1.00	27.73	H	0.066
ATOM	440	HD1	TRP	A	28	-8.168	25.105	16.771	1.00	29.21	H	0.067
ATOM	441	HE1	TRP	A	28	-8.593	25.414	19.125	1.00	30.41	H	0.069
ATOM	442	HE3	TRP	A	28	-3.858	23.699	18.525	1.00	27.11	H	0.065
ATOM	443	HZ2	TRP	A	28	-7.188	25.078	21.507	1.00	29.41	H	0.068
ATOM	444	HZ3	TRP	A	28	-3.445	23.708	20.816	1.00	29.09	H	0.067
ATOM	445	HH2	TRP	A	28	-5.129	24.404	22.311	1.00	26.70	H	0.064
ATOM	446	N	VAL	A	29	-4.405	25.021	13.168	1.00	24.80	N	0.062
ATOM	447	CA	VAL	A	29	-3.675	24.708	11.924	1.00	24.39	C	0.062
ATOM	448	C	VAL	A	29	-2.498	25.640	11.726	1.00	26.05	C	0.064
ATOM	449	O	VAL	A	29	-1.380	25.203	11.410	1.00	22.02	O	0.059
ATOM	450	CB	VAL	A	29	-4.630	24.751	10.716	1.00	21.64	C	0.058
ATOM	451	CG1	VAL	A	29	-3.839	24.525	9.427	1.00	24.74	C	0.062
ATOM	452	CG2	VAL	A	29	-5.701	23.718	10.856	1.00	26.26	C	0.064
ATOM	453	H	VAL	A	29	-5.198	25.332	13.050	1.00	29.75	H	0.068
ATOM	454	HA	VAL	A	29	-3.326	23.805	11.989	1.00	29.27	H	0.068
ATOM	455	HB	VAL	A	29	-5.050	25.624	10.668	1.00	25.96	H	0.064
ATOM	456	HG11	VAL	A	29	-4.457	24.290	8.717	1.00	29.69	H	0.068
ATOM	457	HG12	VAL	A	29	-3.367	25.340	9.195	1.00	29.69	H	0.068
ATOM	458	HG13	VAL	A	29	-3.208	23.802	9.567	1.00	29.69	H	0.068
ATOM	459	HG21	VAL	A	29	-6.270	23.745	10.071	1.00	31.50	H	0.070
ATOM	460	HG22	VAL	A	29	-5.288	22.844	10.935	1.00	31.50	H	0.070
ATOM	461	HG23	VAL	A	29	-6.224	23.910	11.650	1.00	31.50	H	0.070
ATOM	462	N	CYS	A	30	-2.714	26.930	11.964	1.00	23.03	N	0.060
ATOM	463	CA	CYS	A	30	-1.630	27.901	11.892	1.00	23.18	C	0.060
ATOM	464	C	CYS	A	30	-0.527	27.573	12.883	1.00	34.84	C	0.074
ATOM	465	O	CYS	A	30	0.642	27.589	12.524	1.00	22.52	O	0.059
ATOM	466	CB	CYS	A	30	-2.179	29.325	12.128	1.00	23.19	C	0.060
ATOM	467	SG	CYS	A	30	-0.954	30.609	11.902	1.00	28.25	S	0.066
ATOM	468	H	CYS	A	30	-3.478	27.267	12.169	1.00	27.63	H	0.066
ATOM	469	HA	CYS	A	30	-1.246	27.875	11.002	1.00	27.81	H	0.066
ATOM	470	HB2	CYS	A	30	-2.913	29.489	11.521	1.00	27.82	H	0.066
ATOM	471	HB3	CYS	A	30	-2.497	29.384	13.043	1.00	27.82	H	0.066
ATOM	472	N	ALA	A	31	-0.878	27.235	14.125	1.00	24.65	N	0.062
ATOM	473	CA	ALA	A	31	0.154	26.902	15.101	1.00	20.03	C	0.056
ATOM	474	C	ALA	A	31	0.940	25.688	14.643	1.00	20.21	C	0.056
ATOM	475	O	ALA	A	31	2.167	25.644	14.769	1.00	23.94	O	0.061
ATOM	476	CB	ALA	A	31	-0.492	26.667	16.469	1.00	23.26	C	0.060
ATOM	477	H	ALA	A	31	-1.685	27.192	14.420	1.00	29.58	H	0.068
ATOM	478	HA	ALA	A	31	0.769	27.648	15.181	1.00	24.03	H	0.061
ATOM	479	HB1	ALA	A	31	0.201	26.446	17.111	1.00	27.90	H	0.066
ATOM	480	HB2	ALA	A	31	-0.953	27.475	16.743	1.00	27.90	H	0.066
ATOM	481	HB3	ALA	A	31	-1.123	25.934	16.398	1.00	27.90	H	0.066
ATOM	482	N	ALA	A	32	0.247	24.669	14.095	1.00	22.87	N	0.060
ATOM	483	CA	ALA	A	32	0.994	23.487	13.644	1.00	25.67	C	0.063
ATOM	484	C	ALA	A	32	1.909	23.822	12.458	1.00	21.10	C	0.057
ATOM	485	O	ALA	A	32	3.040	23.285	12.359	1.00	21.87	O	0.058
ATOM	486	CB	ALA	A	32	0.032	22.355	13.281	1.00	27.56	C	0.066
ATOM	487	H	ALA	A	32	-0.605	24.640	13.980	1.00	27.44	H	0.065
ATOM	488	HA	ALA	A	32	1.555	23.176	14.372	1.00	30.80	H	0.069
ATOM	489	HB1	ALA	A	32	0.544	21.607	12.937	1.00	33.07	H	0.072
ATOM	490	HB2	ALA	A	32	-0.456	22.087	14.075	1.00	33.07	H	0.072
ATOM	491	HB3	ALA	A	32	-0.585	22.671	12.603	1.00	33.07	H	0.072
ATOM	492	N	LYS	A	33	1.430	24.684	11.527	1.00	24.40	N	0.062
ATOM	493	CA	LYS	A	33	2.266	25.064	10.390	1.00	21.21	C	0.057
ATOM	494	C	LYS	A	33	3.606	25.564	10.861	1.00	28.21	C	0.066
ATOM	495	O	LYS	A	33	4.654	25.129	10.385	1.00	30.10	O	0.068
ATOM	496	CB	LYS	A	33	1.591	26.151	9.536	1.00	29.33	C	0.068
ATOM	497	CG	LYS	A	33	2.520	26.742	8.492	1.00	27.97	C	0.066
ATOM	498	CD	LYS	A	33	2.895	25.753	7.445	1.00	30.53	C	0.069
ATOM	499	CE	LYS	A	33	3.717	26.410	6.310	1.00	41.77	C	0.081
ATOM	500	NZ	LYS	A	33	3.973	25.379	5.271	1.00	45.61	N	0.084
ATOM	501	H	LYS	A	33	0.649	25.045	11.540	1.00	29.28	H	0.068
ATOM	502	HA	LYS	A	33	2.413	24.286	9.829	1.00	25.44	H	0.063
ATOM	503	HB2	LYS	A	33	0.830	25.763	9.075	1.00	35.19	H	0.074
ATOM	504	HB3	LYS	A	33	1.296	26.870	10.116	1.00	35.19	H	0.074
ATOM	505	HG2	LYS	A	33	2.071	27.475	8.054	1.00	33.56	H	0.072
ATOM	506	HG3	LYS	A	33	3.332	27.072	8.904	1.00	33.56	H	0.072

ATOM	507	HD2	LYS	A	33	3.435	25.053	7.844	1.00	36.63	H	0.076
ATOM	508	HD3	LYS	A	33	2.089	25.376	7.058	1.00	36.63	H	0.076
ATOM	509	HE2	LYS	A	33	3.212	27.137	5.914	1.00	50.12	H	0.088
ATOM	510	HE3	LYS	A	33	4.566	26.725	6.656	1.00	50.12	H	0.088
ATOM	511	HZ1	LYS	A	33	4.446	25.731	4.604	1.00	54.72	H	0.092
ATOM	512	HZ2	LYS	A	33	4.429	24.700	5.622	1.00	54.72	H	0.092
ATOM	513	HZ3	LYS	A	33	3.201	25.074	4.949	1.00	54.72	H	0.092
ATOM	514	N	PHE	A	34	3.598	26.546	11.748	1.00	25.49	N	0.063
ATOM	515	CA	PHE	A	34	4.853	27.155	12.148	1.00	27.51	C	0.065
ATOM	516	C	PHE	A	34	5.568	26.456	13.297	1.00	27.04	C	0.065
ATOM	517	O	PHE	A	34	6.787	26.680	13.484	1.00	33.05	O	0.072
ATOM	518	CB	PHE	A	34	4.535	28.599	12.514	1.00	21.06	C	0.057
ATOM	519	CG	PHE	A	34	4.009	29.380	11.357	1.00	23.81	C	0.061
ATOM	520	CD1	PHE	A	34	4.734	29.397	10.150	1.00	26.30	C	0.064
ATOM	521	CD2	PHE	A	34	2.786	29.995	11.432	1.00	27.32	C	0.065
ATOM	522	CE1	PHE	A	34	4.246	30.107	9.066	1.00	29.74	C	0.068
ATOM	523	CE2	PHE	A	34	2.300	30.718	10.367	1.00	30.63	C	0.069
ATOM	524	CZ	PHE	A	34	3.036	30.778	9.167	1.00	28.23	C	0.066
ATOM	525	H	PHE	A	34	2.897	26.872	12.125	1.00	30.58	H	0.069
ATOM	526	HA	PHE	A	34	5.458	27.159	11.391	1.00	33.00	H	0.072
ATOM	527	HB2	PHE	A	34	3.863	28.608	13.213	1.00	25.27	H	0.063
ATOM	528	HB3	PHE	A	34	5.345	29.032	12.826	1.00	25.27	H	0.063
ATOM	529	HD1	PHE	A	34	5.556	28.966	10.093	1.00	31.55	H	0.070
ATOM	530	HD2	PHE	A	34	2.311	29.978	12.231	1.00	32.77	H	0.071
ATOM	531	HE1	PHE	A	34	4.734	30.143	8.275	1.00	35.68	H	0.075
ATOM	532	HE2	PHE	A	34	1.480	31.152	10.433	1.00	36.75	H	0.076
ATOM	533	HZ	PHE	A	34	2.699	31.244	8.436	1.00	33.87	H	0.073
ATOM	534	N	GLU	A	35	4.883	25.605	14.049	1.00	24.28	N	0.061
ATOM	535	CA	GLU	A	35	5.632	24.803	15.007	1.00	23.47	C	0.060
ATOM	536	C	GLU	A	35	6.428	23.677	14.315	1.00	29.12	C	0.067
ATOM	537	O	GLU	A	35	7.600	23.433	14.638	1.00	24.98	O	0.062
ATOM	538	CB	GLU	A	35	4.705	24.246	16.097	1.00	22.81	C	0.060
ATOM	539	CG	GLU	A	35	4.149	25.320	17.007	1.00	22.82	C	0.060
ATOM	540	CD	GLU	A	35	5.164	25.855	17.996	1.00	33.83	C	0.073
ATOM	541	OE1	GLU	A	35	6.344	25.414	17.995	1.00	27.59	O	0.066
ATOM	542	OE2	GLU	A	35	4.842	26.897	18.646	1.00	30.04	O	0.068
ATOM	543	H	GLU	A	35	4.033	25.476	14.030	1.00	29.14	H	0.067
ATOM	544	HA	GLU	A	35	6.276	25.379	15.449	1.00	28.16	H	0.066
ATOM	545	HB2	GLU	A	35	3.957	23.796	15.674	1.00	27.37	H	0.065
ATOM	546	HB3	GLU	A	35	5.203	23.618	16.644	1.00	27.37	H	0.065
ATOM	547	HG2	GLU	A	35	3.844	26.064	16.466	1.00	27.37	H	0.065
ATOM	548	HG3	GLU	A	35	3.407	24.952	17.511	1.00	27.37	H	0.065
ATOM	549	N	SER	A	36	5.813	22.962	13.342	1.00	24.39	N	0.062
ATOM	550	CA	SER	A	36	6.351	21.696	12.866	1.00	28.21	C	0.066
ATOM	551	C	SER	A	36	6.331	21.574	11.354	1.00	24.84	C	0.062
ATOM	552	O	SER	A	36	6.786	20.552	10.819	1.00	25.82	O	0.063
ATOM	553	CB	SER	A	36	5.586	20.531	13.445	1.00	24.74	C	0.062
ATOM	554	OG	SER	A	36	4.278	20.561	12.906	1.00	27.29	O	0.065
ATOM	555	H	SER	A	36	5.085	23.201	12.952	1.00	29.27	H	0.068
ATOM	556	HA	SER	A	36	7.273	21.616	13.154	1.00	33.84	H	0.073
ATOM	557	HB2	SER	A	36	6.021	19.700	13.197	1.00	29.68	H	0.068
ATOM	558	HB3	SER	A	36	5.542	20.618	14.410	1.00	29.68	H	0.068
ATOM	559	HG	SER	A	36	3.900	21.283	13.110	1.00	32.74	H	0.071
ATOM	560	N	ASN	A	37	5.836	22.575	10.651	1.00	27.29	N	0.065
ATOM	561	CA	ASN	A	37	5.579	22.469	9.218	1.00	26.15	C	0.064
ATOM	562	C	ASN	A	37	4.733	21.238	8.920	1.00	25.09	C	0.063
ATOM	563	O	ASN	A	37	4.873	20.599	7.873	1.00	31.63	O	0.070
ATOM	564	CB	ASN	A	37	6.884	22.489	8.430	1.00	34.16	C	0.073
ATOM	565	CG	ASN	A	37	6.663	22.851	6.996	1.00	45.07	C	0.084
ATOM	566	OD1	ASN	A	37	5.575	23.303	6.611	1.00	40.30	O	0.079
ATOM	567	ND2	ASN	A	37	7.700	22.679	6.180	1.00	55.86	N	0.093
ATOM	568	H	ASN	A	37	5.635	23.343	10.981	1.00	32.74	H	0.071
ATOM	569	HA	ASN	A	37	5.064	23.243	8.945	1.00	31.37	H	0.070
ATOM	570	HB2	ASN	A	37	7.482	23.146	8.820	1.00	40.99	H	0.080
ATOM	571	HB3	ASN	A	37	7.289	21.608	8.461	1.00	40.99	H	0.080
ATOM	572	HD21	ASN	A	37	8.440	22.366	6.486	1.00	67.03	H	0.102
ATOM	573	HD22	ASN	A	37	7.627	22.873	5.345	1.00	67.03	H	0.102
ATOM	574	N	PHE	A	38	3.847	20.889	9.847	1.00	25.74	N	0.063
ATOM	575	CA	PHE	A	38	2.878	19.810	9.716	1.00	30.09	C	0.068
ATOM	576	C	PHE	A	38	3.517	18.423	9.827	1.00	25.72	C	0.063
ATOM	577	O	PHE	A	38	2.852	17.436	9.514	1.00	26.35	O	0.064
ATOM	578	CB	PHE	A	38	2.120	19.913	8.382	1.00	26.28	C	0.064
ATOM	579	CG	PHE	A	38	1.237	21.170	8.232	1.00	23.67	C	0.061
ATOM	580	CD1	PHE	A	38	0.577	21.728	9.320	1.00	24.95	C	0.062
ATOM	581	CD2	PHE	A	38	0.989	21.709	6.982	1.00	27.01	C	0.065
ATOM	582	CE1	PHE	A	38	-0.251	22.857	9.100	1.00	23.91	C	0.061
ATOM	583	CE2	PHE	A	38	0.163	22.810	6.796	1.00	26.01	C	0.064
ATOM	584	CZ	PHE	A	38	-0.463	23.350	7.838	1.00	27.26	C	0.065

```
ATOM    585  H   PHE A  38       3.786  21.290  10.605  1.00 30.88           H  0.069
ATOM    586  HA  PHE A  38       2.231  19.887  10.434  1.00 36.11           H  0.075
ATOM    587  HB2 PHE A  38       2.747  19.901   7.645  1.00 31.53           H  0.070
ATOM    588  HB3 PHE A  38       1.532  19.145   8.311  1.00 31.53           H  0.070
ATOM    589  HD1 PHE A  38       0.702  21.390  10.177  1.00 29.93           H  0.068
ATOM    590  HD2 PHE A  38       1.405  21.331   6.241  1.00 32.40           H  0.071
ATOM    591  HE1 PHE A  38      -0.689  23.248   9.821  1.00 28.69           H  0.067
ATOM    592  HE2 PHE A  38       0.030  23.154   5.942  1.00 31.21           H  0.070
ATOM    593  HZ  PHE A  38      -1.001  24.099   7.718  1.00 32.71           H  0.071
ATOM    594  N   ASN A  39       4.782  18.338  10.255  1.00 27.04           N  0.065
ATOM    595  CA  ASN A  39       5.508  17.077  10.397  1.00 22.77           C  0.060
ATOM    596  C   ASN A  39       5.333  16.522  11.813  1.00 30.03           C  0.068
ATOM    597  O   ASN A  39       5.875  17.077  12.773  1.00 28.12           O  0.066
ATOM    598  CB  ASN A  39       6.960  17.321  10.084  1.00 24.46           C  0.062
ATOM    599  CG  ASN A  39       7.773  16.037  10.025  1.00 24.56           C  0.062
ATOM    600  OD1 ASN A  39       7.275  14.973  10.370  1.00 22.17           O  0.059
ATOM    601  ND2 ASN A  39       9.049  16.153   9.622  1.00 31.26           N  0.070
ATOM    602  H   ASN A  39       5.253  19.022  10.476  1.00 32.44           H  0.071
ATOM    603  HA  ASN A  39       5.160  16.428   9.765  1.00 27.32           H  0.065
ATOM    604  HB2 ASN A  39       7.028  17.759   9.221  1.00 29.34           H  0.068
ATOM    605  HB3 ASN A  39       7.341  17.886  10.774  1.00 29.34           H  0.068
ATOM    606 HD21 ASN A  39       9.550  15.456   9.575  1.00 37.50           H  0.076
ATOM    607 HD22 ASN A  39       9.360  16.922   9.396  1.00 37.50           H  0.076
ATOM    608  N   THR A  40       4.631  15.397  11.932  1.00 23.00           N  0.060
ATOM    609  CA  THR A  40       4.396  14.818  13.263  1.00 25.00           C  0.062
ATOM    610  C   THR A  40       5.705  14.428  13.927  1.00 25.19           C  0.063
ATOM    611  O   THR A  40       5.783  14.310  15.154  1.00 25.43           O  0.063
ATOM    612  CB  THR A  40       3.478  13.602  13.249  1.00 34.19           C  0.073
ATOM    613  OG1 THR A  40       4.082  12.486  12.554  1.00 29.59           O  0.068
ATOM    614  CG2 THR A  40       2.137  13.934  12.580  1.00 30.62           C  0.069
ATOM    615  H   THR A  40       4.287  14.956  11.279  1.00 27.60           H  0.066
ATOM    616  HA  THR A  40       3.976  15.492  13.820  1.00 30.00           H  0.068
ATOM    617  HB  THR A  40       3.295  13.338  14.164  1.00 41.03           H  0.080
ATOM    618  HG1 THR A  40       4.766  12.225  12.966  1.00 35.50           H  0.074
ATOM    619 HG21 THR A  40       1.553  13.160  12.608  1.00 36.74           H  0.076
ATOM    620 HG22 THR A  40       1.708  14.669  13.045  1.00 36.74           H  0.076
ATOM    621 HG23 THR A  40       2.281  14.187  11.655  1.00 36.74           H  0.076
ATOM    622  N   GLN A  41       6.716  14.101  13.136  1.00 26.29           N  0.064
ATOM    623  CA  GLN A  41       7.979  13.614  13.714  1.00 26.95           C  0.065
ATOM    624  C   GLN A  41       8.963  14.704  14.036  1.00 28.25           C  0.066
ATOM    625  O   GLN A  41      10.126  14.406  14.339  1.00 31.50           O  0.070
ATOM    626  CB  GLN A  41       8.620  12.585  12.764  1.00 23.41           C  0.060
ATOM    627  CG  GLN A  41       7.763  11.363  12.505  1.00 29.40           C  0.068
ATOM    628  CD  GLN A  41       8.557  10.282  11.802  1.00 27.39           C  0.065
ATOM    629  OE1 GLN A  41       9.361   9.564  12.456  1.00 32.34           O  0.071
ATOM    630  NE2 GLN A  41       8.489  10.275  10.469  1.00 27.04           N  0.065
ATOM    631  H   GLN A  41       6.707  14.147  12.277  1.00 31.55           H  0.070
ATOM    632  HA  GLN A  41       7.779  13.151  14.543  1.00 32.34           H  0.071
ATOM    633  HB2 GLN A  41       8.790  13.013  11.910  1.00 28.09           H  0.066
ATOM    634  HB3 GLN A  41       9.456  12.282  13.151  1.00 28.09           H  0.066
ATOM    635  HG2 GLN A  41       7.445  11.008  13.350  1.00 35.28           H  0.074
ATOM    636  HG3 GLN A  41       7.014  11.609  11.939  1.00 35.28           H  0.074
ATOM    637 HE21 GLN A  41       7.947  10.806  10.064  1.00 32.44           H  0.071
ATOM    638 HE22 GLN A  41       8.933   9.691  10.020  1.00 32.44           H  0.071
ATOM    639  N   ALA A  42       8.569  15.969  13.976  1.00 24.70           N  0.062
ATOM    640  CA  ALA A  42       9.518  17.046  14.258  1.00 23.60           C  0.061
ATOM    641  C   ALA A  42       9.953  17.056  15.736  1.00 24.41           C  0.062
ATOM    642  O   ALA A  42       9.153  16.862  16.653  1.00 23.14           O  0.060
ATOM    643  CB  ALA A  42       8.882  18.394  13.892  1.00 29.58           C  0.068
ATOM    644  H   ALA A  42       7.774  16.231  13.779  1.00 29.63           H  0.068
ATOM    645  HA  ALA A  42      10.309  16.925  13.710  1.00 28.32           H  0.066
ATOM    646  HB1 ALA A  42       9.493  19.107  14.136  1.00 35.49           H  0.074
ATOM    647  HB2 ALA A  42       8.711  18.415  12.937  1.00 35.49           H  0.074
ATOM    648  HB3 ALA A  42       8.050  18.492  14.381  1.00 35.49           H  0.074
ATOM    649  N   THR A  43      11.256  17.236  15.956  1.00 28.56           N  0.067
ATOM    650  CA  THR A  43      11.813  17.430  17.294  1.00 24.27           C  0.061
ATOM    651  C   THR A  43      12.806  18.584  17.257  1.00 25.82           C  0.063
ATOM    652  O   THR A  43      13.498  18.804  16.246  1.00 34.14           O  0.073
ATOM    653  CB  THR A  43      12.484  16.166  17.827  1.00 30.83           C  0.069
ATOM    654  OG1 THR A  43      13.603  15.866  16.976  1.00 31.90           O  0.070
ATOM    655  CG2 THR A  43      11.511  15.007  17.867  1.00 28.50           C  0.067
ATOM    656  H   THR A  43      11.847  17.241  15.336  1.00 34.27           H  0.073
ATOM    657  HA  THR A  43      11.095  17.667  17.897  1.00 29.11           H  0.067
ATOM    658  HB  THR A  43      12.802  16.328  18.729  1.00 36.99           H  0.076
ATOM    659  HG1 THR A  43      13.339  15.740  16.188  1.00 38.28           H  0.077
ATOM    660 HG21 THR A  43      11.943  14.225  18.245  1.00 34.19           H  0.073
ATOM    661 HG22 THR A  43      10.743  15.238  18.413  1.00 34.19           H  0.073
ATOM    662 HG23 THR A  43      11.208  14.797  16.970  1.00 34.19           H  0.073
```

```
ATOM    663  N  AASN A  44      12.916  19.301  18.374  0.52 25.44           N  0.063
ATOM    664  CA AASN A  44      13.937  20.329  18.452  0.52 22.64           C  0.059
ATOM    665  C  AASN A  44      14.399  20.429  19.894  0.52 25.96           C  0.064
ATOM    666  O  AASN A  44      13.583  20.455  20.812  0.52 23.04           O  0.060
ATOM    667  CB AASN A  44      13.417  21.656  17.888  0.52 26.59           C  0.064
ATOM    668  CG AASN A  44      13.220  21.597  16.342  0.52 26.87           C  0.065
ATOM    669  OD1AASN A  44      14.193  21.716  15.583  0.52 27.07           O  0.065
ATOM    670  ND2AASN A  44      11.959  21.430  15.888  0.52 33.31           N  0.072
ATOM    671  H  AASN A  44      12.427  19.213  19.076  0.52 30.52           H  0.069
ATOM    672  HA AASN A  44      14.699  20.058  17.917  0.52 27.16           H  0.065
ATOM    673  HB2AASN A  44      12.560  21.861  18.294  0.52 31.90           H  0.070
ATOM    674  HB3AASN A  44      14.057  22.358  18.085  0.52 31.90           H  0.070
ATOM    675 HD21AASN A  44      11.808  21.394  15.042  0.52 39.96           H  0.079
ATOM    676 HD22AASN A  44      11.308  21.354  16.445  0.52 39.96           H  0.079
ATOM    677  N  BASN A  44      12.847  19.353  18.360  0.48 25.45           N  0.063
ATOM    678  CA BASN A  44      13.749  20.495  18.497  0.48 22.53           C  0.059
ATOM    679  C  BASN A  44      14.288  20.494  19.915  0.48 26.01           C  0.064
ATOM    680  O  BASN A  44      13.512  20.522  20.871  0.48 23.08           O  0.060
ATOM    681  CB BASN A  44      13.043  21.815  18.176  0.48 26.41           C  0.064
ATOM    682  CG BASN A  44      13.827  23.058  18.632  0.48 34.42           C  0.073
ATOM    683  OD1BASN A  44      13.520  23.655  19.689  0.48 34.39           O  0.073
ATOM    684  ND2BASN A  44      14.816  23.461  17.832  0.48 34.01           N  0.073
ATOM    685  H  BASN A  44      12.350  19.225  19.050  0.48 30.53           H  0.069
ATOM    686  HA BASN A  44      14.495  20.391  17.886  0.48 27.03           H  0.065
ATOM    687  HB2BASN A  44      12.918  21.878  17.216  0.48 31.68           H  0.070
ATOM    688  HB3BASN A  44      12.182  21.826  18.622  0.48 31.68           H  0.070
ATOM    689 HD21BASN A  44      15.284  24.152  18.041  0.48 40.81           H  0.080
ATOM    690 HD22BASN A  44      14.992  23.024  17.112  0.48 40.81           H  0.080
ATOM    691  N  AARG A  45      15.703  20.425  20.084  0.52 26.84           N  0.065
ATOM    692  CA AARG A  45      16.285  20.513  21.411  0.52 28.85           C  0.067
ATOM    693  C  AARG A  45      16.450  21.975  21.805  0.52 31.04           C  0.070
ATOM    694  O  AARG A  45      16.947  22.799  21.022  0.52 33.63           O  0.072
ATOM    695  CB AARG A  45      17.626  19.784  21.488  0.52 32.25           C  0.071
ATOM    696  CG AARG A  45      18.160  19.640  22.942  0.52 33.35           C  0.072
ATOM    697  CD AARG A  45      18.750  18.288  23.226  0.52 38.77           C  0.078
ATOM    698  NE AARG A  45      18.571  17.865  24.615  0.52 34.27           N  0.073
ATOM    699  CZ AARG A  45      19.191  16.817  25.142  0.52 35.47           C  0.074
ATOM    700  NH1AARG A  45      20.043  16.105  24.430  0.52 33.35           N  0.072
ATOM    701  NH2AARG A  45      18.961  16.491  26.416  0.52 34.45           N  0.073
ATOM    702  H  AARG A  45      16.284  20.371  19.452  0.52 32.20           H  0.071
ATOM    703  HA AARG A  45      15.683  20.098  22.048  0.52 34.61           H  0.073
ATOM    704  HB2AARG A  45      17.521  18.897  21.110  0.52 38.70           H  0.078
ATOM    705  HB3AARG A  45      18.283  20.283  20.978  0.52 38.70           H  0.078
ATOM    706  HG2AARG A  45      18.851  20.304  23.090  0.52 40.01           H  0.079
ATOM    707  HG3AARG A  45      17.428  19.782  23.562  0.52 40.01           H  0.079
ATOM    708  HD2AARG A  45      18.325  17.626  22.660  0.52 46.52           H  0.085
ATOM    709  HD3AARG A  45      19.702  18.317  23.043  0.52 46.52           H  0.085
ATOM    710  HE AARG A  45      18.160  18.400  25.149  0.52 41.12           H  0.080
ATOM    711 HH11AARG A  45      20.199  16.308  23.609  0.52 40.01           H  0.079
ATOM    712 HH12AARG A  45      20.439  15.430  24.787  0.52 40.01           H  0.079
ATOM    713 HH21AARG A  45      18.409  16.954  26.885  0.52 41.33           H  0.080
ATOM    714 HH22AARG A  45      19.361  15.815  26.766  0.52 41.33           H  0.080
ATOM    715  N  BARG A  45      15.598  20.449  20.057  0.48 26.73           N  0.065
ATOM    716  CA BARG A  45      16.202  20.532  21.371  0.48 28.84           C  0.067
ATOM    717  C  BARG A  45      16.396  21.988  21.779  0.48 31.01           C  0.069
ATOM    718  O  BARG A  45      16.854  22.825  20.989  0.48 33.64           O  0.072
ATOM    719  CB BARG A  45      17.542  19.805  21.437  0.48 32.17           C  0.071
ATOM    720  CG BARG A  45      18.098  19.877  22.855  0.48 34.39           C  0.073
ATOM    721  CD BARG A  45      18.717  18.609  23.345  0.48 36.81           C  0.076
ATOM    722  NE BARG A  45      17.779  17.500  23.468  0.48 37.42           N  0.076
ATOM    723  CZ BARG A  45      17.744  16.454  22.660  0.48 34.72           C  0.074
ATOM    724  NH1BARG A  45      18.594  16.333  21.651  0.48 43.79           N  0.083
ATOM    725  NH2BARG A  45      16.830  15.500  22.869  0.48 33.89           N  0.073
ATOM    726  H  BARG A  45      16.160  20.371  19.410  0.48 32.07           H  0.071
ATOM    727  HA BARG A  45      15.608  20.118  22.017  0.48 34.60           H  0.073
ATOM    728  HB2BARG A  45      17.419  18.873  21.199  0.48 38.60           H  0.078
ATOM    729  HB3BARG A  45      18.173  20.230  20.835  0.48 38.60           H  0.078
ATOM    730  HG2BARG A  45      18.778  20.568  22.887  0.48 41.26           H  0.080
ATOM    731  HG3BARG A  45      17.377  20.100  23.463  0.48 41.26           H  0.080
ATOM    732  HD2BARG A  45      19.436  18.361  22.747  0.48 44.16           H  0.083
ATOM    733  HD3BARG A  45      19.081  18.774  24.229  0.48 44.16           H  0.083
ATOM    734  HE BARG A  45      17.209  17.528  24.111  0.48 44.89           H  0.084
ATOM    735 HH11BARG A  45      18.550  15.643  21.140  0.48 52.55           H  0.090
ATOM    736 HH12BARG A  45      19.187  16.935  21.500  0.48 52.55           H  0.090
ATOM    737 HH21BARG A  45      16.275  15.572  23.522  0.48 40.66           H  0.080
ATOM    738 HH22BARG A  45      16.798  14.815  22.351  0.48 40.66           H  0.080
ATOM    739  N   ASN A  46      16.020  22.288  23.020  1.00 27.95           N  0.066
ATOM    740  CA  ASN A  46      16.158  23.617  23.580  1.00 30.71           C  0.069
```

ATOM	741	C	ASN	A	46	17.439	23.735	24.387	1.00	32.92	C	0.072
ATOM	742	O	ASN	A	46	17.991	22.728	24.851	1.00	33.83	O	0.073
ATOM	743	CB	ASN	A	46	14.950	23.912	24.429	1.00	35.08	C	0.074
ATOM	744	CG	ASN	A	46	13.673	23.778	23.651	1.00	38.95	C	0.078
ATOM	745	OD1	ASN	A	46	12.772	23.068	24.049	1.00	41.28	O	0.080
ATOM	746	ND2	ASN	A	46	13.635	24.393	22.463	1.00	38.37	N	0.077
ATOM	747	H	ASN	A	46	15.654	21.727	23.560	1.00	33.53	H	0.072
ATOM	748	HA	ASN	A	46	16.193	24.268	22.862	1.00	36.85	H	0.076
ATOM	749	HB2	ASN	A	46	14.920	23.286	25.169	1.00	42.10	H	0.081
ATOM	750	HB3	ASN	A	46	15.008	24.821	24.761	1.00	42.10	H	0.081
ATOM	751	HD21	ASN	A	46	12.929	24.340	21.975	1.00	46.04	H	0.085
ATOM	752	HD22	ASN	A	46	14.300	24.874	22.205	1.00	46.04	H	0.085
ATOM	753	N	THR	A	47	17.945	24.977	24.485	1.00	35.27	N	0.074
ATOM	754	CA	THR	A	47	19.260	25.220	25.087	1.00	43.61	C	0.082
ATOM	755	C	THR	A	47	19.331	24.732	26.535	1.00	41.61	C	0.080
ATOM	756	O	THR	A	47	20.412	24.335	27.008	1.00	50.99	O	0.089
ATOM	757	CB	THR	A	47	19.564	26.709	24.996	1.00	46.23	C	0.085
ATOM	758	OG1	THR	A	47	18.424	27.460	25.437	1.00	53.50	O	0.091
ATOM	759	CG2	THR	A	47	19.826	27.093	23.537	1.00	52.27	C	0.090
ATOM	760	H	THR	A	47	17.546	25.688	24.211	1.00	42.32	H	0.081
ATOM	761	HA	THR	A	47	19.934	24.744	24.577	1.00	52.32	H	0.090
ATOM	762	HB	THR	A	47	20.342	26.929	25.532	1.00	55.47	H	0.093
ATOM	763	HG1	THR	A	47	18.586	28.283	25.389	1.00	64.19	H	0.100
ATOM	764	HG21	THR	A	47	20.031	28.039	23.475	1.00	62.72	H	0.099
ATOM	765	HG22	THR	A	47	20.576	26.584	23.190	1.00	62.72	H	0.099
ATOM	766	HG23	THR	A	47	19.041	26.905	22.999	1.00	62.72	H	0.099
ATOM	767	N	ASP	A	48	18.178	24.696	27.221	1.00	42.64	N	0.081
ATOM	768	CA	ASP	A	48	17.991	24.195	28.583	1.00	43.04	C	0.082
ATOM	769	C	ASP	A	48	17.927	22.680	28.675	1.00	45.37	C	0.084
ATOM	770	O	ASP	A	48	17.711	22.158	29.769	1.00	39.87	O	0.079
ATOM	771	CB	ASP	A	48	16.686	24.766	29.203	1.00	41.50	C	0.080
ATOM	772	CG	ASP	A	48	15.406	24.096	28.633	1.00	51.97	C	0.090
ATOM	773	OD1	ASP	A	48	15.499	23.334	27.636	1.00	43.38	O	0.082
ATOM	774	OD2	ASP	A	48	14.300	24.345	29.182	1.00	50.66	O	0.089
ATOM	775	H	ASP	A	48	17.439	24.981	26.885	1.00	51.16	H	0.089
ATOM	776	HA	ASP	A	48	18.735	24.494	29.130	1.00	51.65	H	0.090
ATOM	777	HB2	ASP	A	48	16.701	24.618	30.162	1.00	49.79	H	0.088
ATOM	778	HB3	ASP	A	48	16.636	25.716	29.015	1.00	49.79	H	0.088
ATOM	779	N	GLY	A	49	18.086	21.959	27.567	1.00	34.79	N	0.074
ATOM	780	CA	GLY	A	49	18.152	20.520	27.609	1.00	36.37	C	0.075
ATOM	781	C	GLY	A	49	16.821	19.825	27.428	1.00	26.81	C	0.065
ATOM	782	O	GLY	A	49	16.790	18.588	27.207	1.00	31.59	O	0.070
ATOM	783	H	GLY	A	49	18.158	22.290	26.777	1.00	41.75	H	0.081
ATOM	784	HA2	GLY	A	49	18.748	20.211	26.909	1.00	43.64	H	0.082
ATOM	785	HA3	GLY	A	49	18.522	20.244	28.462	1.00	43.64	H	0.082
ATOM	786	N	SER	A	50	15.700	20.566	27.513	1.00	28.96	N	0.067
ATOM	787	CA	SER	A	50	14.451	19.955	27.152	1.00	33.88	C	0.073
ATOM	788	C	SER	A	50	14.367	19.877	25.631	1.00	28.91	C	0.067
ATOM	789	O	SER	A	50	15.138	20.520	24.886	1.00	28.07	O	0.066
ATOM	790	CB	SER	A	50	13.255	20.728	27.685	1.00	36.87	C	0.076
ATOM	791	OG	SER	A	50	13.163	22.008	27.113	1.00	29.88	O	0.068
ATOM	792	H	SER	A	50	15.654	21.386	27.767	1.00	34.74	H	0.074
ATOM	793	HA	SER	A	50	14.416	19.054	27.509	1.00	40.65	H	0.080
ATOM	794	HB2	SER	A	50	12.446	20.234	27.478	1.00	44.24	H	0.083
ATOM	795	HB3	SER	A	50	13.346	20.821	28.646	1.00	44.24	H	0.083
ATOM	796	HG	SER	A	50	12.799	22.537	27.655	1.00	35.86	H	0.075
ATOM	797	N	THR	A	51	13.376	19.109	25.183	1.00	25.70	N	0.063
ATOM	798	CA	THR	A	51	13.127	18.873	23.774	1.00	23.95	C	0.061
ATOM	799	C	THR	A	51	11.655	19.107	23.464	1.00	23.50	C	0.060
ATOM	800	O	THR	A	51	10.779	18.772	24.267	1.00	23.71	O	0.061
ATOM	801	CB	THR	A	51	13.513	17.469	23.400	1.00	25.44	C	0.063
ATOM	802	OG1	THR	A	51	14.914	17.247	23.671	1.00	26.94	O	0.065
ATOM	803	CG2	THR	A	51	13.264	17.222	21.893	1.00	23.02	C	0.060
ATOM	804	H	THR	A	51	12.820	18.703	25.698	1.00	30.83	H	0.069
ATOM	805	HA	THR	A	51	13.655	19.489	23.242	1.00	28.73	H	0.067
ATOM	806	HB	THR	A	51	12.987	16.840	23.914	1.00	30.52	H	0.069
ATOM	807	HG1	THR	A	51	15.070	17.359	24.489	1.00	32.32	H	0.071
ATOM	808	HG21	THR	A	51	13.699	16.400	21.618	1.00	27.62	H	0.066
ATOM	809	HG22	THR	A	51	12.313	17.147	21.719	1.00	27.62	H	0.066
ATOM	810	HG23	THR	A	51	13.625	17.957	21.373	1.00	27.62	H	0.066
ATOM	811	N	ASP	A	52	11.391	19.670	22.272	1.00	21.59	N	0.058
ATOM	812	CA	ASP	A	52	10.031	19.902	21.800	1.00	21.33	C	0.058
ATOM	813	C	ASP	A	52	9.667	18.785	20.808	1.00	24.95	C	0.062
ATOM	814	O	ASP	A	52	10.454	18.474	19.911	1.00	26.20	O	0.064
ATOM	815	CB	ASP	A	52	9.890	21.235	21.068	1.00	23.80	C	0.061
ATOM	816	CG	ASP	A	52	10.051	22.411	21.995	1.00	29.92	C	0.068
ATOM	817	OD1	ASP	A	52	9.802	22.285	23.193	1.00	31.56	O	0.070
ATOM	818	OD2	ASP	A	52	10.340	23.508	21.444	1.00	42.39	O	0.081

ATOM	819	H	ASP	A	52	11.997	19.927	21.718	1.00	25.91	H	0.064
ATOM	820	HA	ASP	A	52	9.409	19.881	22.544	1.00	25.59	H	0.063
ATOM	821	HB2	ASP	A	52	10.571	21.295	20.380	1.00	28.56	H	0.067
ATOM	822	HB3	ASP	A	52	9.008	21.281	20.671	1.00	28.56	H	0.067
ATOM	823	N	TYR	A	53	8.473	18.256	20.932	1.00	23.17	N	0.060
ATOM	824	CA	TYR	A	53	8.046	17.119	20.132	1.00	29.43	C	0.068
ATOM	825	C	TYR	A	53	6.736	17.297	19.349	1.00	27.07	C	0.065
ATOM	826	O	TYR	A	53	5.725	17.791	19.855	1.00	23.56	O	0.061
ATOM	827	CB	TYR	A	53	7.863	15.921	21.053	1.00	25.28	C	0.063
ATOM	828	CG	TYR	A	53	9.117	15.439	21.760	1.00	25.46	C	0.063
ATOM	829	CD1	TYR	A	53	9.558	16.033	22.927	1.00	22.24	C	0.059
ATOM	830	CD2	TYR	A	53	9.859	14.422	21.239	1.00	23.80	C	0.061
ATOM	831	CE1	TYR	A	53	10.690	15.580	23.604	1.00	25.98	C	0.064
ATOM	832	CE2	TYR	A	53	10.974	14.002	21.881	1.00	21.63	C	0.058
ATOM	833	CZ	TYR	A	53	11.360	14.541	23.064	1.00	21.54	C	0.058
ATOM	834	OH	TYR	A	53	12.586	14.076	23.642	1.00	26.15	O	0.064
ATOM	835	H	TYR	A	53	7.875	18.538	21.482	1.00	27.79	H	0.066
ATOM	836	HA	TYR	A	53	8.743	16.899	19.494	1.00	35.31	H	0.074
ATOM	837	HB2	TYR	A	53	7.216	16.156	21.737	1.00	30.33	H	0.069
ATOM	838	HB3	TYR	A	53	7.523	15.180	20.528	1.00	30.33	H	0.069
ATOM	839	HD1	TYR	A	53	9.054	16.719	23.301	1.00	26.68	H	0.064
ATOM	840	HD2	TYR	A	53	9.592	14.008	20.450	1.00	28.55	H	0.067
ATOM	841	HE1	TYR	A	53	10.975	15.982	24.393	1.00	31.17	H	0.070
ATOM	842	HE2	TYR	A	53	11.465	13.299	21.522	1.00	25.96	H	0.064
ATOM	843	HH	TYR	A	53	12.779	14.538	24.317	1.00	31.37	H	0.070
ATOM	844	N	GLY	A	54	6.744	16.861	18.103	1.00	25.50	N	0.063
ATOM	845	CA	GLY	A	54	5.508	16.685	17.357	1.00	24.55	C	0.062
ATOM	846	C	GLY	A	54	4.970	17.905	16.666	1.00	20.98	C	0.057
ATOM	847	O	GLY	A	54	5.586	18.971	16.616	1.00	22.75	O	0.060
ATOM	848	H	GLY	A	54	7.454	16.658	17.662	1.00	30.59	H	0.069
ATOM	849	HA2	GLY	A	54	5.650	16.006	16.683	1.00	29.46	H	0.068
ATOM	850	HA3	GLY	A	54	4.822	16.363	17.963	1.00	29.46	H	0.068
ATOM	851	N	ILE	A	55	3.769	17.747	16.105	1.00	22.56	N	0.059
ATOM	852	CA	ILE	A	55	3.113	18.791	15.276	1.00	22.69	C	0.059
ATOM	853	C	ILE	A	55	2.940	20.118	16.025	1.00	24.96	C	0.062
ATOM	854	O	ILE	A	55	2.873	21.141	15.354	1.00	26.98	O	0.065
ATOM	855	CB	ILE	A	55	1.764	18.236	14.774	1.00	29.53	C	0.068
ATOM	856	CG1	ILE	A	55	1.219	19.034	13.589	1.00	50.96	C	0.089
ATOM	857	CG2	ILE	A	55	0.756	18.137	15.906	1.00	33.65	C	0.072
ATOM	858	CD1	ILE	A	55	0.812	18.174	12.419	1.00	47.79	C	0.086
ATOM	859	H	ILE	A	55	3.297	17.033	16.186	1.00	27.07	H	0.065
ATOM	860	HA	ILE	A	55	3.669	18.957	14.501	1.00	27.23	H	0.065
ATOM	861	HB	ILE	A	55	1.927	17.333	14.458	1.00	35.43	H	0.074
ATOM	862	HG12	ILE	A	55	0.437	19.531	13.875	1.00	61.14	H	0.098
ATOM	863	HG13	ILE	A	55	1.903	19.648	13.281	1.00	61.14	H	0.098
ATOM	864	HG21	ILE	A	55	0.113	17.442	15.695	1.00	40.38	H	0.079
ATOM	865	HG22	ILE	A	55	1.223	17.918	16.727	1.00	40.38	H	0.079
ATOM	866	HG23	ILE	A	55	0.303	18.987	16.001	1.00	40.38	H	0.079
ATOM	867	HD11	ILE	A	55	0.073	17.605	12.687	1.00	57.34	H	0.094
ATOM	868	HD12	ILE	A	55	0.540	18.748	11.686	1.00	57.34	H	0.094
ATOM	869	HD13	ILE	A	55	1.569	17.630	12.149	1.00	57.34	H	0.094
ATOM	870	N	LEU	A	56	2.816	20.091	17.349	1.00	24.28	N	0.061
ATOM	871	CA	LEU	A	56	2.654	21.315	18.154	1.00	23.76	C	0.061
ATOM	872	C	LEU	A	56	3.826	21.513	19.118	1.00	26.67	C	0.064
ATOM	873	O	LEU	A	56	3.740	22.364	20.010	1.00	28.70	O	0.067
ATOM	874	CB	LEU	A	56	1.321	21.197	18.909	1.00	25.86	C	0.063
ATOM	875	CG	LEU	A	56	0.081	21.303	18.030	1.00	27.07	C	0.065
ATOM	876	CD1	LEU	A	56	-1.171	21.287	18.960	1.00	30.81	C	0.069
ATOM	877	CD2	LEU	A	56	0.003	22.586	17.264	1.00	28.42	C	0.067
ATOM	878	H	LEU	A	56	2.822	19.369	17.817	1.00	29.13	H	0.067
ATOM	879	HA	LEU	A	56	2.614	22.086	17.568	1.00	28.51	H	0.067
ATOM	880	HB2	LEU	A	56	1.293	20.336	19.354	1.00	31.03	H	0.070
ATOM	881	HB3	LEU	A	56	1.275	21.907	19.569	1.00	31.03	H	0.070
ATOM	882	HG	LEU	A	56	0.033	20.556	17.416	1.00	32.48	H	0.071
ATOM	883	HD11	LEU	A	56	-1.971	21.330	18.414	1.00	36.97	H	0.076
ATOM	884	HD12	LEU	A	56	-1.168	20.467	19.478	1.00	36.97	H	0.076
ATOM	885	HD13	LEU	A	56	-1.133	22.054	19.553	1.00	36.97	H	0.076
ATOM	886	HD21	LEU	A	56	-0.882	22.668	16.875	1.00	34.10	H	0.073
ATOM	887	HD22	LEU	A	56	0.168	23.325	17.870	1.00	34.10	H	0.073
ATOM	888	HD23	LEU	A	56	0.673	22.578	16.563	1.00	34.10	H	0.073
ATOM	889	N	GLN	A	57	4.949	20.816	18.887	1.00	23.20	N	0.060
ATOM	890	CA	GLN	A	57	6.199	21.037	19.552	1.00	18.39	C	0.054
ATOM	891	C	GLN	A	57	6.015	21.176	21.059	1.00	26.07	C	0.064
ATOM	892	O	GLN	A	57	6.456	22.128	21.697	1.00	25.02	O	0.062
ATOM	893	CB	GLN	A	57	6.907	22.243	18.950	1.00	21.86	C	0.058
ATOM	894	CG	GLN	A	57	7.419	21.969	17.527	1.00	24.98	C	0.062
ATOM	895	CD	GLN	A	57	8.594	21.065	17.570	1.00	25.27	C	0.063
ATOM	896	OE1	GLN	A	57	9.713	21.501	17.771	1.00	25.75	O	0.063

ATOM	897	NE2	GLN	A	57	8.362	19.813	17.344	1.00	23.31	N	0.060
ATOM	898	H	GLN	A	57	4.993	20.178	18.313	1.00	27.83	H	0.066
ATOM	899	HA	GLN	A	57	6.764	20.263	19.403	1.00	22.06	H	0.059
ATOM	900	HB2	GLN	A	57	6.282	22.984	18.904	1.00	26.22	H	0.064
ATOM	901	HB3	GLN	A	57	7.664	22.484	19.504	1.00	26.22	H	0.064
ATOM	902	HG2	GLN	A	57	6.720	21.542	17.007	1.00	29.97	H	0.068
ATOM	903	HG3	GLN	A	57	7.688	22.804	17.113	1.00	29.97	H	0.068
ATOM	904	HE21	GLN	A	57	7.558	19.544	17.199	1.00	27.97	H	0.066
ATOM	905	HE22	GLN	A	57	9.010	19.247	17.354	1.00	27.97	H	0.066
ATOM	906	N	ILE	A	58	5.455	20.163	21.627	1.00	21.74	N	0.058
ATOM	907	CA	ILE	A	58	5.199	20.115	23.101	1.00	23.23	C	0.060
ATOM	908	C	ILE	A	58	6.477	19.767	23.843	1.00	25.96	C	0.064
ATOM	909	O	ILE	A	58	7.165	18.810	23.493	1.00	28.13	O	0.066
ATOM	910	CB	ILE	A	58	4.062	19.100	23.370	1.00	27.87	C	0.066
ATOM	911	CG1	ILE	A	58	2.698	19.725	22.996	1.00	25.37	C	0.063
ATOM	912	CG2	ILE	A	58	4.014	18.701	24.825	1.00	27.65	C	0.066
ATOM	913	CD1	ILE	A	58	1.605	18.730	22.836	1.00	31.55	C	0.070
ATOM	914	H	ILE	A	58	5.195	19.459	21.207	1.00	26.08	H	0.064
ATOM	915	HA	ILE	A	58	4.903	20.988	23.402	1.00	27.87	H	0.066
ATOM	916	HB	ILE	A	58	4.208	18.309	22.828	1.00	33.44	H	0.072
ATOM	917	HG12	ILE	A	58	2.438	20.346	23.693	1.00	30.44	H	0.069
ATOM	918	HG13	ILE	A	58	2.792	20.198	22.154	1.00	30.44	H	0.069
ATOM	919	HG21	ILE	A	58	3.172	18.257	25.007	1.00	33.17	H	0.072
ATOM	920	HG22	ILE	A	58	4.749	18.096	25.014	1.00	33.17	H	0.072
ATOM	921	HG23	ILE	A	58	4.090	19.498	25.373	1.00	33.17	H	0.072
ATOM	922	HD11	ILE	A	58	1.908	18.019	22.250	1.00	37.85	H	0.077
ATOM	923	HD12	ILE	A	58	1.377	18.368	23.706	1.00	37.85	H	0.077
ATOM	924	HD13	ILE	A	58	0.832	19.171	22.450	1.00	37.85	H	0.077
ATOM	925	N	ASN	A	59	6.762	20.487	24.939	1.00	26.92	N	0.065
ATOM	926	CA	ASN	A	59	8.073	20.504	25.573	1.00	28.19	C	0.066
ATOM	927	C	ASN	A	59	8.139	19.504	26.721	1.00	38.12	C	0.077
ATOM	928	O	ASN	A	59	7.196	19.380	27.494	1.00	32.50	O	0.071
ATOM	929	CB	ASN	A	59	8.347	21.926	26.051	1.00	29.04	C	0.067
ATOM	930	CG	ASN	A	59	9.657	22.065	26.753	1.00	45.60	C	0.084
ATOM	931	OD1	ASN	A	59	9.755	21.847	27.949	1.00	48.33	O	0.087
ATOM	932	ND2	ASN	A	59	10.670	22.481	26.014	1.00	49.04	N	0.087
ATOM	933	H	ASN	A	59	6.188	20.986	25.339	1.00	32.30	H	0.071
ATOM	934	HA	ASN	A	59	8.749	20.265	24.920	1.00	33.82	H	0.073
ATOM	935	HB2	ASN	A	59	8.354	22.520	25.284	1.00	34.84	H	0.074
ATOM	936	HB3	ASN	A	59	7.647	22.191	26.669	1.00	34.84	H	0.074
ATOM	937	HD21	ASN	A	59	11.447	22.583	26.367	1.00	58.84	H	0.096
ATOM	938	HD22	ASN	A	59	10.556	22.632	25.175	1.00	58.84	H	0.096
ATOM	939	N	SER	A	60	9.283	18.797	26.825	1.00	26.55	N	0.064
ATOM	940	CA	SER	A	60	9.525	17.777	27.833	1.00	27.59	C	0.066
ATOM	941	C	SER	A	60	9.838	18.340	29.221	1.00	28.34	C	0.066
ATOM	942	O	SER	A	60	9.992	17.563	30.167	1.00	34.45	O	0.073
ATOM	943	CB	SER	A	60	10.702	16.900	27.417	1.00	24.40	C	0.062
ATOM	944	OG	SER	A	60	11.900	17.672	27.416	1.00	25.96	O	0.064
ATOM	945	H	SER	A	60	9.951	18.906	26.295	1.00	31.85	H	0.070
ATOM	946	HA	SER	A	60	8.740	17.213	27.907	1.00	33.10	H	0.072
ATOM	947	HB2	SER	A	60	10.794	16.169	28.048	1.00	29.27	H	0.068
ATOM	948	HB3	SER	A	60	10.544	16.555	26.524	1.00	29.27	H	0.068
ATOM	949	HG	SER	A	60	12.551	17.192	27.187	1.00	31.15	H	0.070
ATOM	950	N	ARG	A	61	9.998	19.647	29.363	1.00	32.20	N	0.071
ATOM	951	CA	ARG	A	61	10.179	20.213	30.706	1.00	38.68	C	0.078
ATOM	952	C	ARG	A	61	8.905	20.088	31.512	1.00	41.02	C	0.080
ATOM	953	O	ARG	A	61	8.951	19.904	32.722	1.00	38.23	O	0.077
ATOM	954	CB	ARG	A	61	10.597	21.671	30.615	1.00	27.78	C	0.066
ATOM	955	CG	ARG	A	61	10.496	22.496	31.925	1.00	49.55	C	0.088
ATOM	956	CD	ARG	A	61	11.460	21.994	33.021	1.00	65.08	C	0.101
ATOM	957	NE	ARG	A	61	11.309	22.697	34.300	1.00	69.81	N	0.104
ATOM	958	CZ	ARG	A	61	10.463	22.364	35.272	1.00	57.67	C	0.095
ATOM	959	NH1	ARG	A	61	9.679	21.305	35.185	1.00	55.07	N	0.093
ATOM	960	NH2	ARG	A	61	10.404	23.118	36.365	1.00	66.56	N	0.102
ATOM	961	H	ARG	A	61	10.007	20.220	28.722	1.00	38.63	H	0.078
ATOM	962	HA	ARG	A	61	10.879	19.725	31.167	1.00	46.41	H	0.085
ATOM	963	HB2	ARG	A	61	11.511	21.722	30.294	1.00	33.33	H	0.072
ATOM	964	HB3	ARG	A	61	10.005	22.120	30.004	1.00	33.33	H	0.072
ATOM	965	HG2	ARG	A	61	10.729	23.417	31.729	1.00	59.46	H	0.096
ATOM	966	HG3	ARG	A	61	9.592	22.456	32.273	1.00	59.46	H	0.096
ATOM	967	HD2	ARG	A	61	11.311	21.048	33.168	1.00	78.09	H	0.110
ATOM	968	HD3	ARG	A	61	12.361	22.152	32.708	1.00	78.09	H	0.110
ATOM	969	HE	ARG	A	61	11.810	23.383	34.433	1.00	83.76	H	0.114
ATOM	970	HH11	ARG	A	61	9.702	20.807	34.485	1.00	66.08	H	0.101
ATOM	971	HH12	ARG	A	61	9.143	21.114	35.830	1.00	66.08	H	0.101
ATOM	972	HH21	ARG	A	61	10.909	23.810	36.438	1.00	79.87	H	0.112
ATOM	973	HH22	ARG	A	61	9.863	22.912	37.001	1.00	79.87	H	0.112
ATOM	974	N	TRP	A	62	7.767	20.149	30.867	1.00	29.35	N	0.068

ATOM	975	CA	TRP	A	62	6.514	20.190	31.600	1.00	30.43	C	0.069
ATOM	976	C	TRP	A	62	5.555	19.072	31.283	1.00	33.82	C	0.073
ATOM	977	O	TRP	A	62	4.870	18.573	32.201	1.00	31.94	O	0.071
ATOM	978	CB	TRP	A	62	5.816	21.528	31.286	1.00	36.26	C	0.075
ATOM	979	CG	TRP	A	62	6.524	22.709	31.889	1.00	33.99	C	0.073
ATOM	980	CD1	TRP	A	62	7.194	23.731	31.231	1.00	41.14	C	0.080
ATOM	981	CD2	TRP	A	62	6.573	23.019	33.268	1.00	36.83	C	0.076
ATOM	982	NE1	TRP	A	62	7.679	24.622	32.151	1.00	40.83	N	0.080
ATOM	983	CE2	TRP	A	62	7.301	24.217	33.408	1.00	40.55	C	0.079
ATOM	984	CE3	TRP	A	62	6.083	22.383	34.411	1.00	54.80	C	0.092
ATOM	985	CZ2	TRP	A	62	7.558	24.780	34.647	1.00	56.45	C	0.094
ATOM	986	CZ3	TRP	A	62	6.330	22.949	35.637	1.00	54.99	C	0.093
ATOM	987	CH2	TRP	A	62	7.053	24.142	35.747	1.00	58.58	C	0.096
ATOM	988	H	TRP	A	62	7.684	20.168	30.011	1.00	35.21	H	0.074
ATOM	989	HA	TRP	A	62	6.690	20.166	32.553	1.00	36.50	H	0.075
ATOM	990	HB2	TRP	A	62	5.794	21.654	30.325	1.00	43.51	H	0.082
ATOM	991	HB3	TRP	A	62	4.913	21.505	31.640	1.00	43.51	H	0.082
ATOM	992	HD1	TRP	A	62	7.312	23.791	30.310	1.00	49.36	H	0.088
ATOM	993	HE1	TRP	A	62	8.129	25.332	31.969	1.00	48.98	H	0.087
ATOM	994	HE3	TRP	A	62	5.599	21.592	34.343	1.00	65.75	H	0.101
ATOM	995	HZ2	TRP	A	62	8.038	25.572	34.727	1.00	67.73	H	0.103
ATOM	996	HZ3	TRP	A	62	6.005	22.538	36.405	1.00	65.99	H	0.101
ATOM	997	HH2	TRP	A	62	7.209	24.501	36.591	1.00	70.30	H	0.105
ATOM	998	N	TRP	A	63	5.491	18.648	30.012	1.00	28.52	N	0.067
ATOM	999	CA	TRP	A	63	4.305	17.984	29.523	1.00	28.37	C	0.066
ATOM	1000	C	TRP	A	63	4.456	16.516	29.222	1.00	28.86	C	0.067
ATOM	1001	O	TRP	A	63	3.501	15.781	29.354	1.00	26.69	O	0.064
ATOM	1002	CB	TRP	A	63	3.799	18.692	28.254	1.00	26.02	C	0.064
ATOM	1003	CG	TRP	A	63	3.540	20.133	28.520	1.00	31.04	C	0.070
ATOM	1004	CD1	TRP	A	63	4.257	21.205	28.080	1.00	27.80	C	0.066
ATOM	1005	CD2	TRP	A	63	2.501	20.654	29.361	1.00	27.69	C	0.066
ATOM	1006	NE1	TRP	A	63	3.713	22.391	28.605	1.00	27.34	N	0.065
ATOM	1007	CE2	TRP	A	63	2.633	22.059	29.384	1.00	32.14	C	0.071
ATOM	1008	CE3	TRP	A	63	1.466	20.067	30.082	1.00	28.18	C	0.066
ATOM	1009	CZ2	TRP	A	63	1.752	22.892	30.125	1.00	29.68	C	0.068
ATOM	1010	CZ3	TRP	A	63	0.592	20.885	30.802	1.00	34.15	C	0.073
ATOM	1011	CH2	TRP	A	63	0.733	22.279	30.793	1.00	28.81	C	0.067
ATOM	1012	H	TRP	A	63	6.118	18.737	29.430	1.00	34.22	H	0.073
ATOM	1013	HA	TRP	A	63	3.610	18.068	30.195	1.00	34.04	H	0.073
ATOM	1014	HB2	TRP	A	63	4.472	18.625	27.559	1.00	31.22	H	0.070
ATOM	1015	HB3	TRP	A	63	2.970	18.280	27.963	1.00	31.22	H	0.070
ATOM	1016	HD1	TRP	A	63	5.009	21.158	27.535	1.00	33.35	H	0.072
ATOM	1017	HE1	TRP	A	63	4.000	23.187	28.454	1.00	32.81	H	0.071
ATOM	1018	HE3	TRP	A	63	1.358	19.143	30.085	1.00	33.81	H	0.073
ATOM	1019	HZ2	TRP	A	63	1.839	23.818	30.117	1.00	35.61	H	0.074
ATOM	1020	HZ3	TRP	A	63	-0.102	20.499	31.285	1.00	40.97	H	0.080
ATOM	1021	HH2	TRP	A	63	0.144	22.798	31.291	1.00	34.56	H	0.073
ATOM	1022	N	CYS	A	64	5.611	16.057	28.779	1.00	29.67	N	0.068
ATOM	1023	CA	CYS	A	64	5.782	14.635	28.491	1.00	27.47	C	0.065
ATOM	1024	C	CYS	A	64	7.102	14.182	29.093	1.00	26.31	C	0.064
ATOM	1025	O	CYS	A	64	7.952	14.993	29.447	1.00	28.23	O	0.066
ATOM	1026	CB	CYS	A	64	5.727	14.408	26.981	1.00	26.06	C	0.064
ATOM	1027	SG	CYS	A	64	7.058	15.312	26.084	1.00	27.69	S	0.066
ATOM	1028	H	CYS	A	64	6.309	16.538	28.636	1.00	35.60	H	0.074
ATOM	1029	HA	CYS	A	64	5.069	14.124	28.903	1.00	32.96	H	0.072
ATOM	1030	HB2	CYS	A	64	5.831	13.461	26.799	1.00	31.27	H	0.070
ATOM	1031	HB3	CYS	A	64	4.872	14.720	26.645	1.00	31.27	H	0.070
ATOM	1032	N	ASN	A	65	7.277	12.864	29.187	1.00	25.41	N	0.063
ATOM	1033	CA	ASN	A	65	8.506	12.273	29.687	1.00	27.48	C	0.065
ATOM	1034	C	ASN	A	65	9.377	11.733	28.568	1.00	24.59	C	0.062
ATOM	1035	O	ASN	A	65	8.935	10.923	27.778	1.00	23.98	O	0.061
ATOM	1036	CB	ASN	A	65	8.247	11.128	30.652	1.00	28.46	C	0.067
ATOM	1037	CG	ASN	A	65	9.548	10.501	31.118	1.00	31.43	C	0.070
ATOM	1038	OD1	ASN	A	65	10.430	11.206	31.635	1.00	32.21	O	0.071
ATOM	1039	ND2	ASN	A	65	9.694	9.217	30.918	1.00	39.09	N	0.078
ATOM	1040	H	ASN	A	65	6.683	12.284	28.961	1.00	30.49	H	0.069
ATOM	1041	HA	ASN	A	65	9.014	12.951	30.160	1.00	32.97	H	0.072
ATOM	1042	HB2	ASN	A	65	7.774	11.464	31.430	1.00	34.15	H	0.073
ATOM	1043	HB3	ASN	A	65	7.721	10.445	30.207	1.00	34.15	H	0.073
ATOM	1044	HD21	ASN	A	65	10.418	8.823	31.165	1.00	46.91	H	0.085
ATOM	1045	HD22	ASN	A	65	9.061	8.764	30.551	1.00	46.91	H	0.085
ATOM	1046	N	ASP	A	66	10.634	12.192	28.504	1.00	26.86	N	0.065
ATOM	1047	CA	ASP	A	66	11.581	11.614	27.557	1.00	28.31	C	0.066
ATOM	1048	C	ASP	A	66	12.753	10.974	28.290	1.00	26.73	C	0.065
ATOM	1049	O	ASP	A	66	13.650	10.420	27.637	1.00	31.33	O	0.070
ATOM	1050	CB	ASP	A	66	12.068	12.621	26.504	1.00	22.46	C	0.059
ATOM	1051	CG	ASP	A	66	12.784	13.834	27.029	1.00	27.90	C	0.066
ATOM	1052	OD1	ASP	A	66	13.187	13.890	28.186	1.00	28.47	O	0.067

```
ATOM   1053  OD2 ASP A  66      13.093  14.718  26.192  1.00 24.06           O  0.061
ATOM   1054  H   ASP A  66      10.953  12.825  28.990  1.00 32.22           H  0.071
ATOM   1055  HA  ASP A  66      11.135  10.903  27.073  1.00 33.96           H  0.073
ATOM   1056  HB2 ASP A  66      12.679  12.163  25.906  1.00 26.95           H  0.065
ATOM   1057  HB3 ASP A  66      11.300  12.933  26.001  1.00 26.95           H  0.065
ATOM   1058  N   GLY A  67      12.758  11.021  29.626  1.00 31.69           N  0.070
ATOM   1059  CA  GLY A  67      13.794  10.364  30.402  1.00 37.86           C  0.077
ATOM   1060  C   GLY A  67      15.137  11.054  30.402  1.00 36.14           C  0.075
ATOM   1061  O   GLY A  67      16.091  10.514  30.984  1.00 37.95           O  0.077
ATOM   1062  H   GLY A  67      12.171  11.429  30.103  1.00 38.03           H  0.077
ATOM   1063  HA2 GLY A  67      13.497  10.292  31.323  1.00 45.43           H  0.084
ATOM   1064  HA3 GLY A  67      13.921   9.466  30.058  1.00 45.43           H  0.084
ATOM   1065  N  AARG A  68      15.247  12.236  29.777  0.56 29.62           N  0.068
ATOM   1066  CA AARG A  68      16.532  12.911  29.689  0.56 27.96           C  0.066
ATOM   1067  C  AARG A  68      16.434  14.391  30.008  0.56 31.47           C  0.070
ATOM   1068  O  AARG A  68      17.348  15.154  29.653  0.56 34.65           O  0.073
ATOM   1069  CB AARG A  68      17.110  12.693  28.294  0.56 32.16           C  0.071
ATOM   1070  CG AARG A  68      16.325  13.348  27.187  0.56 30.03           C  0.068
ATOM   1071  CD AARG A  68      17.104  13.210  25.903  0.56 24.67           C  0.062
ATOM   1072  NE AARG A  68      16.872  14.327  25.007  0.56 24.17           N  0.061
ATOM   1073  CZ AARG A  68      17.269  14.330  23.744  0.56 35.41           C  0.074
ATOM   1074  NH1AARG A  68      18.033  13.363  23.269  0.56 32.36           N  0.071
ATOM   1075  NH2AARG A  68      16.882  15.312  22.942  0.56 32.61           N  0.071
ATOM   1076  H  AARG A  68      14.597  12.657  29.403  0.56 35.53           H  0.074
ATOM   1077  HA AARG A  68      17.144  12.514  30.328  0.56 33.55           H  0.072
ATOM   1078  HB2AARG A  68      18.010  13.055  28.277  0.56 38.58           H  0.078
ATOM   1079  HB3AARG A  68      17.136  11.740  28.113  0.56 38.58           H  0.078
ATOM   1080  HG2AARG A  68      15.469  12.904  27.083  0.56 36.03           H  0.075
ATOM   1081  HG3AARG A  68      16.204  14.292  27.374  0.56 36.03           H  0.075
ATOM   1082  HD2AARG A  68      18.053  13.176  26.100  0.56 29.60           H  0.068
ATOM   1083  HD3AARG A  68      16.828  12.398  25.450  0.56 29.60           H  0.068
ATOM   1084  HE AARG A  68      16.314  14.932  25.255  0.56 29.00           H  0.067
ATOM   1085 HH11AARG A  68      18.284  12.717  23.778  0.56 38.82           H  0.078
ATOM   1086 HH12AARG A  68      18.283  13.381  22.447  0.56 38.82           H  0.078
ATOM   1087 HH21AARG A  68      16.387  15.946  23.247  0.56 39.13           H  0.078
ATOM   1088 HH22AARG A  68      17.142  15.326  22.122  0.56 39.13           H  0.078
ATOM   1089  N  BARG A  68      15.251  12.229  29.772  0.44 29.63           N  0.068
ATOM   1090  CA BARG A  68      16.536  12.906  29.688  0.44 28.01           C  0.066
ATOM   1091  C  BARG A  68      16.410  14.397  29.958  0.44 31.53           C  0.070
ATOM   1092  O  BARG A  68      17.275  15.173  29.517  0.44 34.46           O  0.073
ATOM   1093  CB BARG A  68      17.175  12.633  28.315  0.44 32.21           C  0.071
ATOM   1094  CG BARG A  68      16.260  12.792  27.112  0.44 30.16           C  0.069
ATOM   1095  CD BARG A  68      16.518  14.142  26.472  0.44 34.53           C  0.073
ATOM   1096  NE BARG A  68      17.784  14.140  25.755  0.44 34.09           N  0.073
ATOM   1097  CZ BARG A  68      18.678  15.115  25.800  0.44 31.29           C  0.070
ATOM   1098  NH1BARG A  68      18.464  16.230  26.489  0.44 36.94           N  0.076
ATOM   1099  NH2BARG A  68      19.835  14.961  25.167  0.44 34.87           N  0.074
ATOM   1100  H  BARG A  68      14.603  12.647  29.392  0.44 35.56           H  0.074
ATOM   1101  HA BARG A  68      17.128  12.540  30.363  0.44 33.61           H  0.072
ATOM   1102  HB2BARG A  68      17.921  13.241  28.197  0.44 38.65           H  0.078
ATOM   1103  HB3BARG A  68      17.502  11.720  28.309  0.44 38.65           H  0.078
ATOM   1104  HG2BARG A  68      16.455  12.099  26.461  0.44 36.19           H  0.075
ATOM   1105  HG3BARG A  68      15.331  12.753  27.384  0.44 36.19           H  0.075
ATOM   1106  HD2BARG A  68      15.812  14.322  25.833  0.44 41.43           H  0.080
ATOM   1107  HD3BARG A  68      16.537  14.817  27.165  0.44 41.43           H  0.080
ATOM   1108  HE BARG A  68      17.965  13.456  25.266  0.44 40.90           H  0.080
ATOM   1109 HH11BARG A  68      17.725  16.349  26.911  0.44 44.33           H  0.083
ATOM   1110 HH12BARG A  68      19.065  16.845  26.502  0.44 44.33           H  0.083
ATOM   1111 HH21BARG A  68      19.988  14.243  24.719  0.44 41.84           H  0.081
ATOM   1112 HH22BARG A  68      20.424  15.588  25.189  0.44 41.84           H  0.081
ATOM   1113  N   THR A  69      15.332  14.822  30.629  1.00 26.42           N  0.064
ATOM   1114  CA  THR A  69      15.107  16.226  30.959  1.00 31.50           C  0.070
ATOM   1115  C   THR A  69      15.018  16.296  32.476  1.00 36.16           C  0.075
ATOM   1116  O   THR A  69      13.934  16.152  33.050  1.00 36.22           O  0.075
ATOM   1117  CB  THR A  69      13.820  16.744  30.318  1.00 30.41           C  0.069
ATOM   1118  OG1 THR A  69      13.856  16.453  28.924  1.00 25.72           O  0.063
ATOM   1119  CG2 THR A  69      13.685  18.260  30.466  1.00 29.47           C  0.068
ATOM   1120  H   THR A  69      14.696  14.307  30.892  1.00 31.70           H  0.070
ATOM   1121  HA  THR A  69      15.842  16.771  30.643  1.00 37.79           H  0.077
ATOM   1122  HB  THR A  69      13.050  16.317  30.725  1.00 36.48           H  0.075
ATOM   1123  HG1 THR A  69      13.882  15.622  28.804  1.00 30.85           H  0.069
ATOM   1124 HG21 THR A  69      12.871  18.564  30.035  1.00 35.36           H  0.074
ATOM   1125 HG22 THR A  69      13.652  18.500  31.405  1.00 35.36           H  0.074
ATOM   1126 HG23 THR A  69      14.444  18.702  30.054  1.00 35.36           H  0.074
ATOM   1127  N   PRO A  70      16.136  16.470  33.159  1.00 33.01           N  0.072
ATOM   1128  CA  PRO A  70      16.142  16.302  34.619  1.00 36.35           C  0.075
ATOM   1129  C   PRO A  70      15.313  17.359  35.318  1.00 40.27           C  0.079
ATOM   1130  O   PRO A  70      15.345  18.547  34.966  1.00 43.43           O  0.082
```

ATOM	1131	CB	PRO	A	70	17.624	16.441	34.991	1.00	44.67	C	0.083
ATOM	1132	CG	PRO	A	70	18.278	17.065	33.796	1.00	47.99	C	0.086
ATOM	1133	CD	PRO	A	70	17.501	16.591	32.614	1.00	36.13	C	0.075
ATOM	1134	HA	PRO	A	70	15.824	15.419	34.865	1.00	43.62	H	0.082
ATOM	1135	HB2	PRO	A	70	17.713	17.014	35.769	1.00	53.60	H	0.091
ATOM	1136	HB3	PRO	A	70	18.000	15.564	35.166	1.00	53.60	H	0.091
ATOM	1137	HG2	PRO	A	70	18.237	18.030	33.872	1.00	57.59	H	0.095
ATOM	1138	HG3	PRO	A	70	19.199	16.767	33.739	1.00	57.59	H	0.095
ATOM	1139	HD2	PRO	A	70	17.534	17.251	31.905	1.00	43.35	H	0.082
ATOM	1140	HD3	PRO	A	70	17.827	15.727	32.317	1.00	43.35	H	0.082
ATOM	1141	N	GLY	A	71	14.543	16.902	36.308	1.00	45.37	N	0.084
ATOM	1142	CA	GLY	A	71	13.720	17.802	37.082	1.00	55.00	C	0.093
ATOM	1143	C	GLY	A	71	12.451	18.201	36.384	1.00	49.29	C	0.088
ATOM	1144	O	GLY	A	71	11.860	19.237	36.716	1.00	46.86	O	0.085
ATOM	1145	H	GLY	A	71	14.486	16.077	36.543	1.00	54.44	H	0.092
ATOM	1146	HA2	GLY	A	71	13.484	17.377	37.922	1.00	65.99	H	0.101
ATOM	1147	HA3	GLY	A	71	14.224	18.607	37.279	1.00	65.99	H	0.101
ATOM	1148	N	SER	A	72	12.018	17.399	35.424	1.00	39.38	N	0.078
ATOM	1149	CA	SER	A	72	10.868	17.706	34.602	1.00	53.44	C	0.091
ATOM	1150	C	SER	A	72	9.620	17.022	35.148	1.00	42.91	C	0.082
ATOM	1151	O	SER	A	72	9.664	15.999	35.841	1.00	37.98	O	0.077
ATOM	1152	CB	SER	A	72	11.097	17.240	33.156	1.00	51.99	C	0.090
ATOM	1153	OG	SER	A	72	11.157	15.809	33.108	1.00	45.92	O	0.085
ATOM	1154	H	SER	A	72	12.387	16.648	35.226	1.00	47.25	H	0.086
ATOM	1155	HA	SER	A	72	10.718	18.664	34.597	1.00	64.12	H	0.100
ATOM	1156	HB2	SER	A	72	10.361	17.546	32.604	1.00	62.38	H	0.099
ATOM	1157	HB3	SER	A	72	11.934	17.606	32.831	1.00	62.38	H	0.099
ATOM	1158	N	ARG	A	73	8.494	17.565	34.768	1.00	36.11	N	0.075
ATOM	1159	CA	ARG	A	73	7.231	16.902	34.997	1.00	35.37	C	0.074
ATOM	1160	C	ARG	A	73	6.783	16.318	33.668	1.00	43.83	C	0.083
ATOM	1161	O	ARG	A	73	7.422	16.498	32.601	1.00	37.15	O	0.076
ATOM	1162	CB	ARG	A	73	6.210	17.886	35.575	1.00	39.05	C	0.078
ATOM	1163	CG	ARG	A	73	6.728	18.627	36.846	1.00	54.59	C	0.092
ATOM	1164	CD	ARG	A	73	6.655	17.747	38.143	1.00	55.68	C	0.093
ATOM	1165	NE	ARG	A	73	5.269	17.526	38.526	1.00	68.65	N	0.103
ATOM	1166	CZ	ARG	A	73	4.500	18.475	39.034	1.00	71.04	C	0.105
ATOM	1167	NH1	ARG	A	73	4.985	19.683	39.282	1.00	50.59	N	0.089
ATOM	1168	NH2	ARG	A	73	3.212	18.210	39.276	1.00	59.32	N	0.096
ATOM	1169	H	ARG	A	73	8.428	18.325	34.371	1.00	43.32	H	0.082
ATOM	1170	HA	ARG	A	73	7.346	16.176	35.629	1.00	42.44	H	0.081
ATOM	1171	HB2	ARG	A	73	6.001	18.554	34.904	1.00	46.85	H	0.085
ATOM	1172	HB3	ARG	A	73	5.407	17.400	35.819	1.00	46.85	H	0.085
ATOM	1173	HG2	ARG	A	73	7.653	18.887	36.713	1.00	65.50	H	0.101
ATOM	1174	HG3	ARG	A	73	6.184	19.416	36.990	1.00	65.50	H	0.101
ATOM	1175	HD2	ARG	A	73	7.067	16.886	37.972	1.00	66.81	H	0.102
ATOM	1176	HD3	ARG	A	73	7.109	18.201	38.870	1.00	66.81	H	0.102
ATOM	1177	HE	ARG	A	73	4.934	16.742	38.417	1.00	82.38	H	0.113
ATOM	1178	HH11	ARG	A	73	5.811	19.861	39.123	1.00	60.70	H	0.097
ATOM	1179	HH12	ARG	A	73	4.472	20.290	39.611	1.00	60.70	H	0.097
ATOM	1180	HH21	ARG	A	73	2.897	17.426	39.115	1.00	71.17	H	0.105
ATOM	1181	HH22	ARG	A	73	2.703	18.818	39.608	1.00	71.17	H	0.105
ATOM	1182	N	ASN	A	74	5.666	15.627	33.752	1.00	34.47	N	0.073
ATOM	1183	CA	ASN	A	74	5.068	14.849	32.700	1.00	28.39	C	0.066
ATOM	1184	C	ASN	A	74	3.564	15.062	32.915	1.00	31.31	C	0.070
ATOM	1185	O	ASN	A	74	2.816	14.156	33.233	1.00	30.66	O	0.069
ATOM	1186	CB	ASN	A	74	5.455	13.374	32.791	1.00	28.01	C	0.066
ATOM	1187	CG	ASN	A	74	4.775	12.552	31.767	1.00	32.39	C	0.071
ATOM	1188	OD1	ASN	A	74	4.150	13.086	30.898	1.00	29.21	O	0.067
ATOM	1189	ND2	ASN	A	74	4.882	11.209	31.858	1.00	31.74	N	0.070
ATOM	1190	H	ASN	A	74	5.202	15.596	34.475	1.00	41.35	H	0.080
ATOM	1191	HA	ASN	A	74	5.323	15.198	31.832	1.00	34.06	H	0.073
ATOM	1192	HB2	ASN	A	74	6.412	13.288	32.660	1.00	33.61	H	0.072
ATOM	1193	HB3	ASN	A	74	5.206	13.034	33.665	1.00	33.61	H	0.072
ATOM	1194	HD21	ASN	A	74	4.496	10.707	31.276	1.00	38.08	H	0.077
ATOM	1195	HD22	ASN	A	74	5.338	10.856	32.496	1.00	38.08	H	0.077
ATOM	1196	N	LEU	A	75	3.158	16.327	32.853	1.00	27.70	N	0.066
ATOM	1197	CA	LEU	A	75	1.798	16.714	33.165	1.00	31.92	C	0.071
ATOM	1198	C	LEU	A	75	0.795	16.122	32.188	1.00	39.11	C	0.078
ATOM	1199	O	LEU	A	75	-0.366	16.035	32.545	1.00	33.63	O	0.072
ATOM	1200	CB	LEU	A	75	1.733	18.232	33.210	1.00	31.54	C	0.070
ATOM	1201	CG	LEU	A	75	2.481	18.861	34.399	1.00	36.77	C	0.076
ATOM	1202	CD1	LEU	A	75	2.481	20.361	34.277	1.00	34.43	C	0.073
ATOM	1203	CD2	LEU	A	75	1.854	18.507	35.722	1.00	41.55	C	0.080
ATOM	1204	H	LEU	A	75	3.664	16.985	32.629	1.00	33.24	H	0.072
ATOM	1205	HA	LEU	A	75	1.575	16.379	34.047	1.00	38.30	H	0.077
ATOM	1206	HB2	LEU	A	75	2.125	18.584	32.396	1.00	37.84	H	0.077
ATOM	1207	HB3	LEU	A	75	0.803	18.502	33.269	1.00	37.84	H	0.077
ATOM	1208	HG	LEU	A	75	3.401	18.553	34.402	1.00	44.12	H	0.083

ATOM	1209	HD11	LEU	A	75	2.819	20.607	33.402	1.00	41.32	H	0.080
ATOM	1210	HD12	LEU	A	75	1.574	20.684	34.386	1.00	41.32	H	0.080
ATOM	1211	HD13	LEU	A	75	3.050	20.734	34.969	1.00	41.32	H	0.080
ATOM	1212	HD21	LEU	A	75	2.260	19.048	36.418	1.00	49.86	H	0.088
ATOM	1213	HD22	LEU	A	75	0.902	18.686	35.677	1.00	49.86	H	0.088
ATOM	1214	HD23	LEU	A	75	2.006	17.567	35.904	1.00	49.86	H	0.088
ATOM	1215	N	CYS	A	76	1.189	15.689	30.985	1.00	32.62	N	0.071
ATOM	1216	CA	CYS	A	76	0.229	15.007	30.092	1.00	30.39	C	0.069
ATOM	1217	C	CYS	A	76	0.226	13.487	30.246	1.00	31.66	C	0.070
ATOM	1218	O	CYS	A	76	-0.573	12.791	29.598	1.00	34.51	O	0.073
ATOM	1219	CB	CYS	A	76	0.519	15.370	28.630	1.00	28.96	C	0.067
ATOM	1220	SG	CYS	A	76	0.049	17.111	28.322	1.00	28.44	S	0.067
ATOM	1221	H	CYS	A	76	1.983	15.772	30.665	1.00	39.13	H	0.078
ATOM	1222	HA	CYS	A	76	-0.665	15.322	30.296	1.00	36.46	H	0.075
ATOM	1223	HB2	CYS	A	76	1.462	15.261	28.441	1.00	34.74	H	0.074
ATOM	1224	HB3	CYS	A	76	-0.010	14.803	28.047	1.00	34.74	H	0.074
ATOM	1225	N	ASN	A	77	1.131	12.975	31.072	1.00	29.01	N	0.067
ATOM	1226	CA	ASN	A	77	1.241	11.547	31.391	1.00	26.88	C	0.065
ATOM	1227	C	ASN	A	77	1.414	10.720	30.137	1.00	30.37	C	0.069
ATOM	1228	O	ASN	A	77	0.683	9.752	29.896	1.00	31.12	O	0.070
ATOM	1229	CB	ASN	A	77	0.055	11.088	32.193	1.00	27.13	C	0.065
ATOM	1230	CG	ASN	A	77	0.181	11.522	33.605	1.00	42.66	C	0.082
ATOM	1231	OD1	ASN	A	77	-0.502	12.441	34.042	1.00	49.41	O	0.088
ATOM	1232	ND2	ASN	A	77	1.188	10.980	34.291	1.00	50.34	N	0.089
ATOM	1233	H	ASN	A	77	1.720	13.450	31.479	1.00	34.81	H	0.074
ATOM	1234	HA	ASN	A	77	2.033	11.415	31.935	1.00	32.25	H	0.071
ATOM	1235	HB2	ASN	A	77	-0.756	11.472	31.825	1.00	32.55	H	0.071
ATOM	1236	HB3	ASN	A	77	0.008	10.119	32.177	1.00	32.55	H	0.071
ATOM	1237	HD21	ASN	A	77	1.663	10.358	33.936	1.00	60.40	H	0.097
ATOM	1238	HD22	ASN	A	77	1.319	11.205	35.111	1.00	60.40	H	0.097
ATOM	1239	N	ILE	A	78	2.422	11.090	29.341	1.00	28.33	N	0.066
ATOM	1240	CA	ILE	A	78	2.748	10.354	28.115	1.00	27.92	C	0.066
ATOM	1241	C	ILE	A	78	4.246	10.354	27.914	1.00	35.60	C	0.074
ATOM	1242	O	ILE	A	78	4.974	11.305	28.267	1.00	30.04	O	0.068
ATOM	1243	CB	ILE	A	78	2.073	10.968	26.885	1.00	32.52	C	0.071
ATOM	1244	CG1	ILE	A	78	2.268	12.504	26.941	1.00	38.58	C	0.078
ATOM	1245	CG2	ILE	A	78	0.611	10.548	26.791	1.00	39.11	C	0.078
ATOM	1246	CD1	ILE	A	78	2.390	13.135	25.589	1.00	40.05	C	0.079
ATOM	1247	H	ILE	A	78	2.933	11.765	29.489	1.00	33.99	H	0.073
ATOM	1248	HA	ILE	A	78	2.433	9.444	28.215	1.00	33.50	H	0.072
ATOM	1249	HB	ILE	A	78	2.530	10.637	26.096	1.00	39.02	H	0.078
ATOM	1250	HG12	ILE	A	78	1.502	12.900	27.385	1.00	46.29	H	0.085
ATOM	1251	HG13	ILE	A	78	3.077	12.708	27.435	1.00	46.29	H	0.085
ATOM	1252	HG21	ILE	A	78	0.217	10.951	26.002	1.00	46.92	H	0.085
ATOM	1253	HG22	ILE	A	78	0.564	9.581	26.728	1.00	46.92	H	0.085
ATOM	1254	HG23	ILE	A	78	0.144	10.851	27.585	1.00	46.92	H	0.085
ATOM	1255	HD11	ILE	A	78	2.491	14.094	25.696	1.00	48.05	H	0.086
ATOM	1256	HD12	ILE	A	78	3.168	12.768	25.141	1.00	48.05	H	0.086
ATOM	1257	HD13	ILE	A	78	1.590	12.941	25.076	1.00	48.05	H	0.086
ATOM	1258	N	PRO	A	79	4.743	9.308	27.267	1.00	38.37	N	0.077
ATOM	1259	CA	PRO	A	79	6.094	9.406	26.701	1.00	27.67	C	0.066
ATOM	1260	C	PRO	A	79	6.107	10.432	25.575	1.00	29.12	C	0.067
ATOM	1261	O	PRO	A	79	5.177	10.513	24.778	1.00	28.13	O	0.066
ATOM	1262	CB	PRO	A	79	6.382	8.007	26.189	1.00	25.74	C	0.063
ATOM	1263	CG	PRO	A	79	4.983	7.396	25.965	1.00	37.47	C	0.076
ATOM	1264	CD	PRO	A	79	4.119	7.984	27.024	1.00	31.27	C	0.070
ATOM	1265	HA	PRO	A	79	6.740	9.650	27.382	1.00	33.20	H	0.072
ATOM	1266	HB2	PRO	A	79	6.875	8.056	25.355	1.00	30.88	H	0.069
ATOM	1267	HB3	PRO	A	79	6.877	7.505	26.855	1.00	30.88	H	0.069
ATOM	1268	HG2	PRO	A	79	4.659	7.639	25.084	1.00	44.96	H	0.084
ATOM	1269	HG3	PRO	A	79	5.030	6.432	26.058	1.00	44.96	H	0.084
ATOM	1270	HD2	PRO	A	79	3.213	8.085	26.693	1.00	37.52	H	0.076
ATOM	1271	HD3	PRO	A	79	4.149	7.441	27.827	1.00	37.52	H	0.076
ATOM	1272	N	CYS	A	80	7.127	11.269	25.561	1.00	29.58	N	0.068
ATOM	1273	CA	CYS	A	80	7.256	12.246	24.481	1.00	26.81	C	0.065
ATOM	1274	C	CYS	A	80	7.185	11.568	23.121	1.00	29.28	C	0.068
ATOM	1275	O	CYS	A	80	6.607	12.130	22.181	1.00	27.08	O	0.065
ATOM	1276	CB	CYS	A	80	8.563	13.048	24.634	1.00	24.11	C	0.061
ATOM	1277	SG	CYS	A	80	8.695	13.998	26.150	1.00	26.44	S	0.064
ATOM	1278	H	CYS	A	80	7.752	11.298	26.151	1.00	35.49	H	0.074
ATOM	1279	HA	CYS	A	80	6.517	12.872	24.538	1.00	32.16	H	0.071
ATOM	1280	HB2	CYS	A	80	9.310	12.429	24.610	1.00	28.92	H	0.067
ATOM	1281	HB3	CYS	A	80	8.630	13.667	23.892	1.00	28.92	H	0.067
ATOM	1282	N	SER	A	81	7.654	10.310	22.997	1.00	26.64	N	0.064
ATOM	1283	CA	SER	A	81	7.544	9.606	21.714	1.00	20.68	C	0.057
ATOM	1284	C	SER	A	81	6.099	9.498	21.241	1.00	24.78	C	0.062
ATOM	1285	O	SER	A	81	5.832	9.544	20.025	1.00	29.29	O	0.068
ATOM	1286	CB	SER	A	81	8.142	8.195	21.835	1.00	32.92	C	0.072

```
ATOM   1287  OG  SER A  81       7.472   7.490  22.882  1.00 35.60           O  0.074
ATOM   1288  H   SER A  81       8.029   9.858  23.625  1.00 31.96           H  0.071
ATOM   1289  HA  SER A  81       8.047  10.091  21.041  1.00 24.81           H  0.062
ATOM   1290  HB2 SER A  81       8.018   7.721  20.998  1.00 39.50           H  0.078
ATOM   1291  HB3 SER A  81       9.086   8.263  22.047  1.00 39.50           H  0.078
ATOM   1292  HG  SER A  81       6.660   7.394  22.691  1.00 42.71           H  0.082
ATOM   1293  N   ALA A  82       5.160   9.331  22.179  1.00 27.00           N  0.065
ATOM   1294  CA  ALA A  82       3.724   9.342  21.838  1.00 29.19           C  0.067
ATOM   1295  C   ALA A  82       3.337  10.537  20.980  1.00 32.71           C  0.071
ATOM   1296  O   ALA A  82       2.416  10.458  20.149  1.00 32.72           O  0.071
ATOM   1297  CB  ALA A  82       2.902   9.329  23.115  1.00 46.11           C  0.085
ATOM   1298  H   ALA A  82       5.323   9.210  23.015  1.00 32.40           H  0.071
ATOM   1299  HA  ALA A  82       3.514   8.537  21.339  1.00 35.02           H  0.074
ATOM   1300  HB1 ALA A  82       1.961   9.298  22.883  1.00 55.32           H  0.093
ATOM   1301  HB2 ALA A  82       3.140   8.546  23.635  1.00 55.32           H  0.093
ATOM   1302  HB3 ALA A  82       3.092  10.134  23.621  1.00 55.32           H  0.093
ATOM   1303  N   LEU A  83       4.061  11.634  21.120  1.00 29.16           N  0.067
ATOM   1304  CA  LEU A  83       3.684  12.833  20.378  1.00 27.27           C  0.065
ATOM   1305  C   LEU A  83       4.222  12.888  18.970  1.00 28.83           C  0.067
ATOM   1306  O   LEU A  83       3.996  13.887  18.301  1.00 29.51           O  0.068
ATOM   1307  CB  LEU A  83       4.157  14.062  21.139  1.00 29.12           C  0.067
ATOM   1308  CG  LEU A  83       3.563  14.096  22.549  1.00 33.66           C  0.072
ATOM   1309  CD1 LEU A  83       4.311  15.201  23.292  1.00 32.95           C  0.072
ATOM   1310  CD2 LEU A  83       2.077  14.430  22.406  1.00 33.67           C  0.072
ATOM   1311  H   LEU A  83       4.755  11.716  21.622  1.00 34.98           H  0.074
ATOM   1312  HA  LEU A  83       2.717  12.871  20.318  1.00 32.71           H  0.071
ATOM   1313  HB2 LEU A  83       5.124  14.038  21.216  1.00 34.93           H  0.074
ATOM   1314  HB3 LEU A  83       3.878  14.861  20.666  1.00 34.93           H  0.074
ATOM   1315  HG  LEU A  83       3.678  13.248  23.005  1.00 40.38           H  0.079
ATOM   1316 HD11 LEU A  83       3.932  15.297  24.180  1.00 39.53           H  0.078
ATOM   1317 HD12 LEU A  83       5.248  14.960  23.357  1.00 39.53           H  0.078
ATOM   1318 HD13 LEU A  83       4.216  16.031  22.800  1.00 39.53           H  0.078
ATOM   1319 HD21 LEU A  83       1.719  14.656  23.279  1.00 40.39           H  0.079
ATOM   1320 HD22 LEU A  83       1.989  15.186  21.807  1.00 40.39           H  0.079
ATOM   1321 HD23 LEU A  83       1.608  13.662  22.045  1.00 40.39           H  0.079
ATOM   1322  N   LEU A  84       4.941  11.871  18.510  1.00 27.87           N  0.066
ATOM   1323  CA  LEU A  84       5.496  11.827  17.163  1.00 30.80           C  0.069
ATOM   1324  C   LEU A  84       4.683  10.959  16.251  1.00 38.66           C  0.078
ATOM   1325  O   LEU A  84       5.059  10.759  15.096  1.00 31.01           O  0.069
ATOM   1326  CB  LEU A  84       6.924  11.291  17.225  1.00 28.72           C  0.067
ATOM   1327  CG  LEU A  84       7.863  12.122  18.089  1.00 28.18           C  0.066
ATOM   1328  CD1 LEU A  84       9.238  11.475  18.057  1.00 31.97           C  0.071
ATOM   1329  CD2 LEU A  84       7.919  13.618  17.676  1.00 28.13           C  0.066
ATOM   1330  H   LEU A  84       5.127  11.172  18.975  1.00 33.44           H  0.072
ATOM   1331  HA  LEU A  84       5.516  12.721  16.795  1.00 36.96           H  0.076
ATOM   1332  HB2 LEU A  84       6.903  10.393  17.590  1.00 34.45           H  0.073
ATOM   1333  HB3 LEU A  84       7.289  11.271  16.326  1.00 34.45           H  0.073
ATOM   1334  HG  LEU A  84       7.548  12.087  19.006  1.00 33.81           H  0.073
ATOM   1335 HD11 LEU A  84       9.756  11.793  18.813  1.00 38.35           H  0.077
ATOM   1336 HD12 LEU A  84       9.129  10.514  18.109  1.00 38.35           H  0.077
ATOM   1337 HD13 LEU A  84       9.679  11.712  17.226  1.00 38.35           H  0.077
ATOM   1338 HD21 LEU A  84       8.606  14.063  18.197  1.00 33.75           H  0.072
ATOM   1339 HD22 LEU A  84       8.130  13.677  16.731  1.00 33.75           H  0.072
ATOM   1340 HD23 LEU A  84       7.057  14.027  17.849  1.00 33.75           H  0.072
ATOM   1341  N   SER A  85       3.604  10.399  16.764  1.00 32.54           N  0.071
ATOM   1342  CA  SER A  85       2.837   9.431  15.992  1.00 35.54           C  0.074
ATOM   1343  C   SER A  85       2.139  10.106  14.810  1.00 33.97           C  0.073
ATOM   1344  O   SER A  85       1.808  11.302  14.832  1.00 32.81           O  0.071
ATOM   1345  CB  SER A  85       1.811   8.742  16.884  1.00 44.50           C  0.083
ATOM   1346  OG  SER A  85       0.836   8.161  16.052  1.00 43.09           O  0.082
ATOM   1347  H   SER A  85       3.289  10.556  17.548  1.00 39.04           H  0.078
ATOM   1348  HA  SER A  85       3.438   8.754  15.643  1.00 42.64           H  0.081
ATOM   1349  HB2 SER A  85       2.247   8.050  17.406  1.00 53.40           H  0.091
ATOM   1350  HB3 SER A  85       1.394   9.397  17.465  1.00 53.40           H  0.091
ATOM   1351  HG  SER A  85       0.255   7.773  16.518  1.00 51.70           H  0.090
ATOM   1352  N   SER A  86       1.827   9.312  13.777  1.00 33.39           N  0.072
ATOM   1353  CA  SER A  86       0.985   9.901  12.739  1.00 40.72           C  0.080
ATOM   1354  C   SER A  86      -0.467  10.001  13.192  1.00 40.26           C  0.079
ATOM   1355  O   SER A  86      -1.248  10.786  12.619  1.00 35.41           O  0.074
ATOM   1356  CB  SER A  86       1.106   9.129  11.423  1.00 49.77           C  0.088
ATOM   1357  OG  SER A  86       0.852   7.758  11.601  1.00 39.30           O  0.078
ATOM   1358  H   SER A  86       2.069   8.495  13.661  1.00 40.07           H  0.079
ATOM   1359  HA  SER A  86       1.296  10.804  12.567  1.00 48.86           H  0.087
ATOM   1360  HB2 SER A  86       0.464   9.487  10.790  1.00 59.72           H  0.096
ATOM   1361  HB3 SER A  86       2.006   9.238  11.077  1.00 59.72           H  0.096
ATOM   1362  HG  SER A  86       0.924   7.355  10.868  1.00 47.15           H  0.086
ATOM   1363  N   ASP A  87      -0.839   9.276  14.235  1.00 36.19           N  0.075
ATOM   1364  CA  ASP A  87      -2.133   9.524  14.864  1.00 36.98           C  0.076
```

```
ATOM   1365  C   ASP A  87      -1.984  10.687  15.847  1.00 42.04           C  0.081
ATOM   1366  O   ASP A  87      -1.133  10.650  16.757  1.00 36.18           O  0.075
ATOM   1367  CB  ASP A  87      -2.623   8.288  15.591  1.00 40.48           C  0.079
ATOM   1368  CG  ASP A  87      -4.014   8.460  16.128  1.00 45.73           C  0.084
ATOM   1369  OD1 ASP A  87      -4.156   8.974  17.258  1.00 40.15           O  0.079
ATOM   1370  OD2 ASP A  87      -4.971   8.131  15.405  1.00 53.30           O  0.091
ATOM   1371  H   ASP A  87      -0.376   8.647  14.594  1.00 43.42           H  0.082
ATOM   1372  HA  ASP A  87      -2.787   9.767  14.190  1.00 44.37           H  0.083
ATOM   1373  HB2 ASP A  87      -2.628   7.538  14.976  1.00 48.57           H  0.087
ATOM   1374  HB3 ASP A  87      -2.032   8.103  16.338  1.00 48.57           H  0.087
ATOM   1375  N   ILE A  88      -2.822  11.722  15.686  1.00 36.08           N  0.075
ATOM   1376  CA  ILE A  88      -2.570  12.954  16.435  1.00 29.25           C  0.067
ATOM   1377  C   ILE A  88      -3.222  12.962  17.808  1.00 36.63           C  0.076
ATOM   1378  O   ILE A  88      -3.160  13.957  18.535  1.00 29.43           O  0.068
ATOM   1379  CB  ILE A  88      -3.004  14.182  15.601  1.00 29.75           C  0.068
ATOM   1380  CG1 ILE A  88      -4.519  14.202  15.345  1.00 38.02           C  0.077
ATOM   1381  CG2 ILE A  88      -2.255  14.270  14.304  1.00 29.28           C  0.068
ATOM   1382  CD1 ILE A  88      -4.969  15.601  14.780  1.00 32.76           C  0.071
ATOM   1383  H   ILE A  88      -3.512  11.735  15.173  1.00 43.29           H  0.082
ATOM   1384  HA  ILE A  88      -1.614  13.031  16.575  1.00 35.09           H  0.074
ATOM   1385  HB  ILE A  88      -2.786  14.974  16.117  1.00 35.69           H  0.075
ATOM   1386 HG12 ILE A  88      -4.744  13.522  14.691  1.00 45.62           H  0.084
ATOM   1387 HG13 ILE A  88      -4.995  14.044  16.175  1.00 45.62           H  0.084
ATOM   1388 HG21 ILE A  88      -2.406  15.142  13.907  1.00 35.13           H  0.074
ATOM   1389 HG22 ILE A  88      -1.309  14.147  14.479  1.00 35.13           H  0.074
ATOM   1390 HG23 ILE A  88      -2.573  13.573  13.708  1.00 35.13           H  0.074
ATOM   1391 HD11 ILE A  88      -5.938  15.633  14.753  1.00 39.31           H  0.078
ATOM   1392 HD12 ILE A  88      -4.631  16.299  15.362  1.00 39.31           H  0.078
ATOM   1393 HD13 ILE A  88      -4.610  15.712  13.886  1.00 39.31           H  0.078
ATOM   1394  N   THR A  89      -3.792  11.834  18.215  1.00 31.13           N  0.070
ATOM   1395  CA  THR A  89      -4.550  11.797  19.468  1.00 31.76           C  0.070
ATOM   1396  C   THR A  89      -3.753  12.315  20.657  1.00 26.16           C  0.064
ATOM   1397  O   THR A  89      -4.226  13.160  21.421  1.00 28.41           O  0.067
ATOM   1398  CB  THR A  89      -5.007  10.363  19.759  1.00 36.28           C  0.075
ATOM   1399  OG1 THR A  89      -5.941   9.994  18.760  1.00 35.52           O  0.074
ATOM   1400  CG2 THR A  89      -5.640  10.299  21.130  1.00 36.31           C  0.075
ATOM   1401  H   THR A  89      -3.759  11.084  17.796  1.00 37.35           H  0.076
ATOM   1402  HA  THR A  89      -5.341  12.350  19.374  1.00 38.11           H  0.077
ATOM   1403  HB  THR A  89      -4.245   9.763  19.736  1.00 43.53           H  0.082
ATOM   1404  HG1 THR A  89      -5.581  10.032  18.002  1.00 42.62           H  0.081
ATOM   1405 HG21 THR A  89      -6.223   9.526  21.186  1.00 43.57           H  0.082
ATOM   1406 HG22 THR A  89      -4.955  10.224  21.813  1.00 43.57           H  0.082
ATOM   1407 HG23 THR A  89      -6.165  11.099  21.291  1.00 43.57           H  0.082
ATOM   1408  N   ALA A  90      -2.551  11.784  20.852  1.00 25.15           N  0.063
ATOM   1409  CA  ALA A  90      -1.788  12.169  22.038  1.00 36.14           C  0.075
ATOM   1410  C   ALA A  90      -1.388  13.646  21.990  1.00 27.85           C  0.066
ATOM   1411  O   ALA A  90      -1.449  14.344  23.016  1.00 27.20           O  0.065
ATOM   1412  CB  ALA A  90      -0.556  11.262  22.211  1.00 36.88           C  0.076
ATOM   1413  H   ALA A  90      -2.164  11.218  20.333  1.00 30.17           H  0.069
ATOM   1414  HA  ALA A  90      -2.351  12.046  22.818  1.00 43.37           H  0.082
ATOM   1415  HB1 ALA A  90      -0.069  11.539  23.003  1.00 44.26           H  0.083
ATOM   1416  HB2 ALA A  90      -0.852  10.343  22.308  1.00 44.26           H  0.083
ATOM   1417  HB3 ALA A  90       0.010  11.347  21.428  1.00 44.26           H  0.083
ATOM   1418  N   SER A  91      -0.985  14.149  20.822  1.00 27.84           N  0.066
ATOM   1419  CA  SER A  91      -0.654  15.565  20.715  1.00 22.94           C  0.060
ATOM   1420  C   SER A  91      -1.888  16.427  20.945  1.00 22.26           C  0.059
ATOM   1421  O   SER A  91      -1.851  17.447  21.660  1.00 27.38           O  0.065
ATOM   1422  CB  SER A  91      -0.064  15.873  19.337  1.00 25.09           C  0.063
ATOM   1423  OG  SER A  91       1.314  15.600  19.303  1.00 26.52           O  0.064
ATOM   1424  H   SER A  91      -0.897  13.701  20.093  1.00 33.40           H  0.072
ATOM   1425  HA  SER A  91      -0.001  15.799  21.390  1.00 27.52           H  0.065
ATOM   1426  HB2 SER A  91      -0.510  15.324  18.673  1.00 30.10           H  0.068
ATOM   1427  HB3 SER A  91      -0.204  16.812  19.138  1.00 30.10           H  0.068
ATOM   1428  HG  SER A  91       1.623  15.774  18.541  1.00 31.81           H  0.070
ATOM   1429  N   VAL A  92      -3.036  15.974  20.464  1.00 26.42           N  0.064
ATOM   1430  CA  VAL A  92      -4.259  16.751  20.677  1.00 26.62           C  0.064
ATOM   1431  C   VAL A  92      -4.661  16.776  22.150  1.00 28.98           C  0.067
ATOM   1432  O   VAL A  92      -4.956  17.840  22.689  1.00 27.99           O  0.066
ATOM   1433  CB  VAL A  92      -5.413  16.236  19.797  1.00 34.20           C  0.073
ATOM   1434  CG1 VAL A  92      -6.721  16.967  20.249  1.00 35.50           C  0.074
ATOM   1435  CG2 VAL A  92      -5.115  16.465  18.376  1.00 31.60           C  0.070
ATOM   1436  H   VAL A  92      -3.139  15.243  20.023  1.00 31.70           H  0.070
ATOM   1437  HA  VAL A  92      -4.084  17.668  20.412  1.00 31.94           H  0.071
ATOM   1438  HB  VAL A  92      -5.528  15.283  19.938  1.00 41.04           H  0.080
ATOM   1439 HG11 VAL A  92      -7.360  16.946  19.520  1.00 42.59           H  0.081
ATOM   1440 HG12 VAL A  92      -7.090  16.513  21.023  1.00 42.59           H  0.081
ATOM   1441 HG13 VAL A  92      -6.509  17.887  20.470  1.00 42.59           H  0.081
ATOM   1442 HG21 VAL A  92      -5.808  16.051  17.838  1.00 37.91           H  0.077
```

ATOM	1443	HG22	VAL	A	92	-5.093	17.420	18.209	1.00	37.91	H	0.077
ATOM	1444	HG23	VAL	A	92	-4.254	16.073	18.164	1.00	37.91	H	0.077
ATOM	1445	N	ASN	A	93	-4.733	15.625	22.814	1.00	29.65	N	0.068
ATOM	1446	CA	ASN	A	93	-5.169	15.611	24.203	1.00	31.77	C	0.070
ATOM	1447	C	ASN	A	93	-4.220	16.400	25.089	1.00	29.05	C	0.067
ATOM	1448	O	ASN	A	93	-4.654	17.080	26.016	1.00	28.74	O	0.067
ATOM	1449	CB	ASN	A	93	-5.277	14.165	24.726	1.00	33.03	C	0.072
ATOM	1450	CG	ASN	A	93	-6.339	13.370	23.999	1.00	40.83	C	0.080
ATOM	1451	OD1	ASN	A	93	-7.157	13.931	23.258	1.00	48.31	O	0.087
ATOM	1452	ND2	ASN	A	93	-6.346	12.048	24.226	1.00	51.71	N	0.090
ATOM	1453	H	ASN	A	93	-4.538	14.853	22.489	1.00	35.57	H	0.074
ATOM	1454	HA	ASN	A	93	-6.047	16.019	24.264	1.00	38.11	H	0.077
ATOM	1455	HB2	ASN	A	93	-4.426	13.717	24.600	1.00	39.62	H	0.079
ATOM	1456	HB3	ASN	A	93	-5.507	14.184	25.668	1.00	39.62	H	0.079
ATOM	1457	HD21	ASN	A	93	-6.930	11.548	23.840	1.00	62.05	H	0.098
ATOM	1458	HD22	ASN	A	93	-5.762	11.699	24.751	1.00	62.05	H	0.098
ATOM	1459	N	CYS	A	94	-2.910	16.315	24.830	1.00	27.91	N	0.066
ATOM	1460	CA	CYS	A	94	-1.980	17.162	25.577	1.00	21.89	C	0.058
ATOM	1461	C	CYS	A	94	-2.208	18.650	25.275	1.00	29.63	C	0.068
ATOM	1462	O	CYS	A	94	-2.263	19.471	26.194	1.00	31.43	O	0.070
ATOM	1463	CB	CYS	A	94	-0.524	16.723	25.283	1.00	28.76	C	0.067
ATOM	1464	SG	CYS	A	94	0.664	17.427	26.391	1.00	32.38	S	0.071
ATOM	1465	H	CYS	A	94	-2.547	15.795	24.249	1.00	33.49	H	0.072
ATOM	1466	HA	CYS	A	94	-2.140	17.030	26.524	1.00	26.26	H	0.064
ATOM	1467	HB2	CYS	A	94	-0.467	15.758	25.360	1.00	34.51	H	0.073
ATOM	1468	HB3	CYS	A	94	-0.291	16.995	24.382	1.00	34.51	H	0.073
ATOM	1469	N	ALA	A	95	-2.356	19.016	24.003	1.00	27.34	N	0.065
ATOM	1470	CA	ALA	A	95	-2.589	20.422	23.616	1.00	20.27	C	0.056
ATOM	1471	C	ALA	A	95	-3.807	21.023	24.323	1.00	28.03	C	0.066
ATOM	1472	O	ALA	A	95	-3.795	22.195	24.738	1.00	23.79	O	0.061
ATOM	1473	CB	ALA	A	95	-2.744	20.531	22.097	1.00	23.93	C	0.061
ATOM	1474	H	ALA	A	95	-2.327	18.472	23.337	1.00	32.81	H	0.071
ATOM	1475	HA	ALA	A	95	-1.814	20.946	23.871	1.00	24.32	H	0.062
ATOM	1476	HB1	ALA	A	95	-2.894	21.460	21.861	1.00	28.71	H	0.067
ATOM	1477	HB2	ALA	A	95	-1.933	20.209	21.674	1.00	28.71	H	0.067
ATOM	1478	HB3	ALA	A	95	-3.500	19.992	21.818	1.00	28.71	H	0.067
ATOM	1479	N	LYS	A	96	-4.845	20.202	24.539	1.00	29.57	N	0.068
ATOM	1480	CA	LYS	A	96	-6.013	20.660	25.286	1.00	26.07	C	0.064
ATOM	1481	C	LYS	A	96	-5.650	21.009	26.715	1.00	28.46	C	0.067
ATOM	1482	O	LYS	A	96	-6.128	22.009	27.256	1.00	29.95	O	0.068
ATOM	1483	CB	LYS	A	96	-7.073	19.565	25.298	1.00	25.37	C	0.063
ATOM	1484	CG	LYS	A	96	-7.842	19.448	23.981	1.00	24.88	C	0.062
ATOM	1485	CD	LYS	A	96	-8.758	18.201	23.897	1.00	30.54	C	0.069
ATOM	1486	CE	LYS	A	96	-9.651	18.280	22.665	1.00	35.95	C	0.075
ATOM	1487	NZ	LYS	A	96	-10.439	17.037	22.397	1.00	43.00	N	0.082
ATOM	1488	H	LYS	A	96	-4.893	19.388	24.266	1.00	35.48	H	0.074
ATOM	1489	HA	LYS	A	96	-6.385	21.448	24.859	1.00	31.27	H	0.070
ATOM	1490	HB2	LYS	A	96	-6.642	18.713	25.468	1.00	30.44	H	0.069
ATOM	1491	HB3	LYS	A	96	-7.714	19.756	26.001	1.00	30.44	H	0.069
ATOM	1492	HG2	LYS	A	96	-8.401	20.234	23.874	1.00	29.85	H	0.068
ATOM	1493	HG3	LYS	A	96	-7.205	19.397	23.251	1.00	29.85	H	0.068
ATOM	1494	HD2	LYS	A	96	-8.212	17.402	23.830	1.00	36.64	H	0.076
ATOM	1495	HD3	LYS	A	96	-9.323	18.161	24.684	1.00	36.64	H	0.076
ATOM	1496	HE2	LYS	A	96	-10.280	19.009	22.783	1.00	43.13	H	0.082
ATOM	1497	HE3	LYS	A	96	-9.095	18.450	21.888	1.00	43.13	H	0.082
ATOM	1498	HZ1	LYS	A	96	-10.971	16.859	23.088	1.00	51.59	H	0.090
ATOM	1499	HZ2	LYS	A	96	-10.938	17.145	21.668	1.00	51.59	H	0.090
ATOM	1500	HZ3	LYS	A	96	-9.888	16.349	22.271	1.00	51.59	H	0.090
ATOM	1501	N	LYS	A	97	-4.831	20.179	27.362	1.00	30.05	N	0.068
ATOM	1502	CA	LYS	A	97	-4.410	20.497	28.714	1.00	29.97	C	0.068
ATOM	1503	C	LYS	A	97	-3.611	21.788	28.730	1.00	28.52	C	0.067
ATOM	1504	O	LYS	A	97	-3.880	22.689	29.538	1.00	31.76	O	0.070
ATOM	1505	CB	LYS	A	97	-3.603	19.348	29.313	1.00	24.97	C	0.062
ATOM	1506	CG	LYS	A	97	-3.445	19.403	30.854	1.00	53.19	C	0.091
ATOM	1507	CD	LYS	A	97	-4.769	19.507	31.664	1.00	71.38	C	0.105
ATOM	1508	CE	LYS	A	97	-4.647	20.573	32.740	1.00	75.64	C	0.109
ATOM	1509	NZ	LYS	A	97	-4.474	21.944	32.093	1.00	64.74	N	0.100
ATOM	1510	H	LYS	A	97	-4.515	19.445	27.045	1.00	36.05	H	0.075
ATOM	1511	HA	LYS	A	97	-5.202	20.638	29.251	1.00	35.95	H	0.075
ATOM	1512	HB2	LYS	A	97	-4.044	18.512	29.093	1.00	29.96	H	0.068
ATOM	1513	HB3	LYS	A	97	-2.714	19.360	28.926	1.00	29.96	H	0.068
ATOM	1514	HG2	LYS	A	97	-2.994	18.594	31.142	1.00	63.82	H	0.100
ATOM	1515	HG3	LYS	A	97	-2.899	20.172	31.079	1.00	63.82	H	0.100
ATOM	1516	HD2	LYS	A	97	-5.510	19.736	31.085	1.00	85.65	H	0.115
ATOM	1517	HD3	LYS	A	97	-4.940	18.658	32.101	1.00	85.65	H	0.115
ATOM	1518	HE2	LYS	A	97	-5.453	20.585	33.279	1.00	90.77	H	0.119
ATOM	1519	HE3	LYS	A	97	-3.870	20.391	33.292	1.00	90.77	H	0.119
ATOM	1520	HZ1	LYS	A	97	-5.135	22.093	31.516	1.00	77.68	H	0.110

```
ATOM   1521  HZ2 LYS A  97      -4.488  22.578  32.717  1.00 77.68           H  0.110
ATOM   1522  HZ3 LYS A  97      -3.698  21.982  31.659  1.00 77.68           H  0.110
ATOM   1523  N   ILE A  98      -2.635  21.909  27.814  1.00 27.55           N  0.065
ATOM   1524  CA  ILE A  98      -1.790  23.093  27.743  1.00 27.00           C  0.065
ATOM   1525  C   ILE A  98      -2.602  24.356  27.584  1.00 26.87           C  0.065
ATOM   1526  O   ILE A  98      -2.396  25.345  28.297  1.00 29.26           O  0.067
ATOM   1527  CB  ILE A  98      -0.769  22.990  26.593  1.00 25.80           C  0.063
ATOM   1528  CG1 ILE A  98       0.310  21.950  26.848  1.00 28.75           C  0.067
ATOM   1529  CG2 ILE A  98       0.028  24.293  26.377  1.00 27.25           C  0.065
ATOM   1530  CD1 ILE A  98       0.866  21.285  25.603  1.00 32.34           C  0.071
ATOM   1531  H   ILE A  98      -2.449  21.312  27.224  1.00 33.05           H  0.072
ATOM   1532  HA  ILE A  98      -1.292  23.167  28.572  1.00 32.39           H  0.071
ATOM   1533  HB  ILE A  98      -1.237  22.766  25.773  1.00 30.95           H  0.069
ATOM   1534 HG12 ILE A  98       1.049  22.386  27.295  1.00 34.50           H  0.073
ATOM   1535 HG13 ILE A  98      -0.054  21.255  27.418  1.00 34.50           H  0.073
ATOM   1536 HG21 ILE A  98       0.789  24.106  25.805  1.00 32.69           H  0.071
ATOM   1537 HG22 ILE A  98      -0.546  24.951  25.955  1.00 32.69           H  0.071
ATOM   1538 HG23 ILE A  98       0.335  24.621  27.237  1.00 32.69           H  0.071
ATOM   1539 HD11 ILE A  98       1.548  20.647  25.865  1.00 38.80           H  0.078
ATOM   1540 HD12 ILE A  98       0.146  20.828  25.141  1.00 38.80           H  0.078
ATOM   1541 HD13 ILE A  98       1.251  21.964  25.027  1.00 38.80           H  0.078
ATOM   1542  N   VAL A  99      -3.508  24.364  26.618  1.00 20.16           N  0.056
ATOM   1543  CA  VAL A  99      -4.222  25.605  26.313  1.00 22.78           C  0.060
ATOM   1544  C   VAL A  99      -5.210  25.970  27.393  1.00 34.14           C  0.073
ATOM   1545  O   VAL A  99      -5.702  27.096  27.388  1.00 28.58           O  0.067
ATOM   1546  CB  VAL A  99      -4.884  25.497  24.940  1.00 24.56           C  0.062
ATOM   1547  CG1 VAL A  99      -6.011  24.470  25.036  1.00 25.74           C  0.063
ATOM   1548  CG2 VAL A  99      -5.398  26.772  24.497  1.00 27.81           C  0.066
ATOM   1549  H   VAL A  99      -3.724  23.688  26.133  1.00 24.18           H  0.061
ATOM   1550  HA  VAL A  99      -3.573  26.324  26.264  1.00 27.33           H  0.065
ATOM   1551  HB  VAL A  99      -4.235  25.183  24.291  1.00 29.47           H  0.068
ATOM   1552 HG11 VAL A  99      -6.303  24.242  24.140  1.00 30.88           H  0.069
ATOM   1553 HG12 VAL A  99      -5.685  23.676  25.486  1.00 30.88           H  0.069
ATOM   1554 HG13 VAL A  99      -6.751  24.851  25.534  1.00 30.88           H  0.069
ATOM   1555 HG21 VAL A  99      -5.646  26.701  23.562  1.00 33.37           H  0.072
ATOM   1556 HG22 VAL A  99      -6.176  27.005  25.027  1.00 33.37           H  0.072
ATOM   1557 HG23 VAL A  99      -4.708  27.445  24.604  1.00 33.37           H  0.072
ATOM   1558  N   SER A 100      -5.506  25.032  28.301  1.00 31.60           N  0.070
ATOM   1559  CA  SER A 100      -6.423  25.252  29.402  1.00 33.58           C  0.072
ATOM   1560  C   SER A 100      -5.731  25.734  30.650  1.00 33.18           C  0.072
ATOM   1561  O   SER A 100      -6.409  25.974  31.645  1.00 35.18           O  0.074
ATOM   1562  CB  SER A 100      -7.180  23.948  29.705  1.00 28.85           C  0.067
ATOM   1563  OG  SER A 100      -7.925  23.480  28.582  1.00 32.51           O  0.071
ATOM   1564  H   SER A 100      -5.175  24.239  28.292  1.00 37.91           H  0.077
ATOM   1565  HA  SER A 100      -7.073  25.923  29.142  1.00 40.29           H  0.079
ATOM   1566  HB2 SER A 100      -6.537  23.267  29.957  1.00 34.61           H  0.073
ATOM   1567  HB3 SER A 100      -7.793  24.108  30.440  1.00 34.61           H  0.073
ATOM   1568  HG  SER A 100      -7.409  23.332  27.936  1.00 39.00           H  0.078
ATOM   1569  N   ASP A 101      -4.413  25.877  30.612  1.00 31.41           N  0.070
ATOM   1570  CA  ASP A 101      -3.646  26.167  31.845  1.00 38.72           C  0.078
ATOM   1571  C   ASP A 101      -3.486  27.654  32.151  1.00 43.55           C  0.082
ATOM   1572  O   ASP A 101      -2.717  27.923  33.083  1.00 42.20           O  0.081
ATOM   1573  CB  ASP A 101      -2.296  25.461  31.813  1.00 39.63           C  0.079
ATOM   1574  CG  ASP A 101      -1.869  25.009  33.195  1.00 61.51           C  0.098
ATOM   1575  OD1 ASP A 101      -2.445  24.036  33.700  1.00 73.01           O  0.107
ATOM   1576  OD2 ASP A 101      -1.015  25.671  33.766  1.00 46.28           O  0.085
ATOM   1577  H   ASP A 101      -3.932  25.815  29.902  1.00 37.69           H  0.077
ATOM   1578  HA  ASP A 101      -4.137  25.786  32.589  1.00 46.45           H  0.085
ATOM   1579  HB2 ASP A 101      -2.358  24.678  31.243  1.00 47.55           H  0.086
ATOM   1580  HB3 ASP A 101      -1.624  26.072  31.472  1.00 47.55           H  0.086
ATOM   1581  N   GLY A 102      -4.103  28.587  31.411  1.00 36.57           N  0.075
ATOM   1582  CA  GLY A 102      -4.092  29.960  31.884  1.00 26.83           C  0.065
ATOM   1583  C   GLY A 102      -3.743  31.014  30.862  1.00 33.62           C  0.072
ATOM   1584  O   GLY A 102      -4.332  32.102  30.845  1.00 29.30           O  0.068
ATOM   1585  H   GLY A 102      -4.514  28.452  30.668  1.00 43.87           H  0.083
ATOM   1586  HA2 GLY A 102      -4.972  30.172  32.233  1.00 32.19           H  0.071
ATOM   1587  HA3 GLY A 102      -3.459  30.044  32.613  1.00 32.19           H  0.071
ATOM   1588  N   ASN A 103      -2.769  30.712  30.013  1.00 30.08           N  0.068
ATOM   1589  CA  ASN A 103      -2.305  31.656  28.985  1.00 29.46           C  0.068
ATOM   1590  C   ASN A 103      -2.845  31.341  27.594  1.00 27.91           C  0.066
ATOM   1591  O   ASN A 103      -2.428  31.990  26.616  1.00 24.12           O  0.061
ATOM   1592  CB  ASN A 103      -0.795  31.678  28.963  1.00 33.03           C  0.072
ATOM   1593  CG  ASN A 103      -0.253  32.186  30.239  1.00 41.65           C  0.081
ATOM   1594  OD1 ASN A 103      -0.841  33.088  30.834  1.00 33.06           O  0.072
ATOM   1595  ND2 ASN A 103       0.782  31.561  30.739  1.00 45.15           N  0.084
ATOM   1596  H   ASN A 103      -2.352  29.960  30.006  1.00 36.09           H  0.075
ATOM   1597  HA  ASN A 103      -2.608  32.547  29.220  1.00 35.34           H  0.074
ATOM   1598  HB2 ASN A 103      -0.462  30.777  28.827  1.00 39.63           H  0.079
```

ATOM	1599	HB3	ASN	A	103	-0.492	32.261	28.249	1.00	39.63	H	0.079
ATOM	1600	HD21	ASN	A	103	1.120	31.820	31.486	1.00	54.17	H	0.092
ATOM	1601	HD22	ASN	A	103	1.151	30.921	30.299	1.00	54.17	H	0.092
ATOM	1602	N	GLY	A	104	-3.795	30.425	27.504	1.00	27.64	N	0.066
ATOM	1603	CA	GLY	A	104	-4.362	30.083	26.206	1.00	23.66	C	0.061
ATOM	1604	C	GLY	A	104	-3.301	29.685	25.217	1.00	25.70	C	0.063
ATOM	1605	O	GLY	A	104	-2.315	28.984	25.554	1.00	26.81	O	0.065
ATOM	1606	H	GLY	A	104	-4.128	29.990	28.167	1.00	33.16	H	0.072
ATOM	1607	HA2	GLY	A	104	-4.983	29.345	26.307	1.00	28.38	H	0.066
ATOM	1608	HA3	GLY	A	104	-4.844	30.846	25.852	1.00	28.38	H	0.066
ATOM	1609	N	MET	A	105	-3.505	30.094	23.961	1.00	22.91	N	0.060
ATOM	1610	CA	MET	A	105	-2.516	29.655	22.988	1.00	19.72	C	0.055
ATOM	1611	C	MET	A	105	-1.208	30.417	23.018	1.00	23.20	C	0.060
ATOM	1612	O	MET	A	105	-0.313	30.016	22.274	1.00	24.48	O	0.062
ATOM	1613	CB	MET	A	105	-3.020	29.584	21.511	1.00	25.24	C	0.063
ATOM	1614	CG	MET	A	105	-4.037	28.417	21.317	1.00	20.22	C	0.056
ATOM	1615	SD	MET	A	105	-4.571	28.254	19.569	1.00	23.39	S	0.060
ATOM	1616	CE	MET	A	105	-3.204	27.301	19.044	1.00	21.76	C	0.058
ATOM	1617	H	MET	A	105	-4.149	30.583	23.670	1.00	27.48	H	0.065
ATOM	1618	HA	MET	A	105	-2.300	28.738	23.219	1.00	23.66	H	0.061
ATOM	1619	HB2	MET	A	105	-3.464	30.416	21.283	1.00	30.29	H	0.069
ATOM	1620	HB3	MET	A	105	-2.266	29.432	20.920	1.00	30.29	H	0.069
ATOM	1621	HG2	MET	A	105	-3.620	27.583	21.585	1.00	24.26	H	0.061
ATOM	1622	HG3	MET	A	105	-4.823	28.587	21.859	1.00	24.26	H	0.061
ATOM	1623	HE1	MET	A	105	-3.308	27.093	18.102	1.00	26.11	H	0.064
ATOM	1624	HE2	MET	A	105	-2.391	27.812	19.183	1.00	26.11	H	0.064
ATOM	1625	HE3	MET	A	105	-3.173	26.482	19.562	1.00	26.11	H	0.064
ATOM	1626	N	ASN	A	106	-1.056	31.400	23.921	1.00	21.08	N	0.057
ATOM	1627	CA	ASN	A	106	0.227	32.042	24.098	1.00	21.70	C	0.058
ATOM	1628	C	ASN	A	106	1.282	31.073	24.595	1.00	24.72	C	0.062
ATOM	1629	O	ASN	A	106	2.476	31.391	24.538	1.00	23.84	O	0.061
ATOM	1630	CB	ASN	A	106	0.078	33.209	25.058	1.00	27.02	C	0.065
ATOM	1631	CG	ASN	A	106	-0.867	34.251	24.549	1.00	27.93	C	0.066
ATOM	1632	OD1	ASN	A	106	-0.561	34.946	23.548	1.00	25.83	O	0.063
ATOM	1633	ND2	ASN	A	106	-2.045	34.360	25.189	1.00	25.52	N	0.063
ATOM	1634	H	ASN	A	106	-1.680	31.701	24.431	1.00	25.29	H	0.063
ATOM	1635	HA	ASN	A	106	0.523	32.394	23.244	1.00	26.03	H	0.064
ATOM	1636	HB2	ASN	A	106	-0.261	32.882	25.906	1.00	32.42	H	0.071
ATOM	1637	HB3	ASN	A	106	0.944	33.626	25.185	1.00	32.42	H	0.071
ATOM	1638	HD21	ASN	A	106	-2.622	34.943	24.930	1.00	30.61	H	0.069
ATOM	1639	HD22	ASN	A	106	-2.215	33.858	25.866	1.00	30.61	H	0.069
ATOM	1640	N	ALA	A	107	0.858	29.899	25.062	1.00	22.29	N	0.059
ATOM	1641	CA	ALA	A	107	1.777	28.828	25.421	1.00	19.55	C	0.055
ATOM	1642	C	ALA	A	107	2.636	28.434	24.260	1.00	28.23	C	0.066
ATOM	1643	O	ALA	A	107	3.729	27.904	24.460	1.00	30.14	O	0.069
ATOM	1644	CB	ALA	A	107	0.998	27.609	25.926	1.00	21.41	C	0.058
ATOM	1645	H	ALA	A	107	0.031	29.698	25.184	1.00	26.74	H	0.065
ATOM	1646	HA	ALA	A	107	2.357	29.133	26.136	1.00	23.45	H	0.060
ATOM	1647	HB1	ALA	A	107	1.626	26.908	26.160	1.00	25.69	H	0.063
ATOM	1648	HB2	ALA	A	107	0.483	27.865	26.707	1.00	25.69	H	0.063
ATOM	1649	HB3	ALA	A	107	0.404	27.301	25.223	1.00	25.69	H	0.063
ATOM	1650	N	TRP	A	108	2.128	28.649	23.026	1.00	21.10	N	0.057
ATOM	1651	CA	TRP	A	108	2.872	28.386	21.803	1.00	24.96	C	0.062
ATOM	1652	C	TRP	A	108	3.445	29.692	21.297	1.00	26.65	C	0.064
ATOM	1653	O	TRP	A	108	2.739	30.499	20.692	1.00	24.91	O	0.062
ATOM	1654	CB	TRP	A	108	1.982	27.727	20.741	1.00	25.83	C	0.063
ATOM	1655	CG	TRP	A	108	1.712	26.306	21.063	1.00	21.78	C	0.058
ATOM	1656	CD1	TRP	A	108	2.531	25.212	20.774	1.00	23.26	C	0.060
ATOM	1657	CD2	TRP	A	108	0.560	25.779	21.748	1.00	19.10	C	0.055
ATOM	1658	NE1	TRP	A	108	1.971	24.076	21.324	1.00	26.77	N	0.065
ATOM	1659	CE2	TRP	A	108	0.758	24.396	21.883	1.00	23.00	C	0.060
ATOM	1660	CE3	TRP	A	108	-0.597	26.355	22.303	1.00	24.30	C	0.062
ATOM	1661	CZ2	TRP	A	108	-0.169	23.569	22.523	1.00	25.11	C	0.063
ATOM	1662	CZ3	TRP	A	108	-1.505	25.542	22.912	1.00	25.40	C	0.063
ATOM	1663	CH2	TRP	A	108	-1.266	24.176	23.070	1.00	23.68	C	0.061
ATOM	1664	H	TRP	A	108	1.336	28.953	22.883	1.00	25.32	H	0.063
ATOM	1665	HA	TRP	A	108	3.608	27.785	21.997	1.00	29.94	H	0.068
ATOM	1666	HB2	TRP	A	108	1.134	28.196	20.699	1.00	30.99	H	0.069
ATOM	1667	HB3	TRP	A	108	2.429	27.764	19.881	1.00	30.99	H	0.069
ATOM	1668	HD1	TRP	A	108	3.358	25.255	20.351	1.00	27.90	H	0.066
ATOM	1669	HE1	TRP	A	108	2.290	23.281	21.252	1.00	32.11	H	0.071
ATOM	1670	HE3	TRP	A	108	-0.745	27.271	22.246	1.00	29.16	H	0.067
ATOM	1671	HZ2	TRP	A	108	-0.029	22.653	22.604	1.00	30.12	H	0.068
ATOM	1672	HZ3	TRP	A	108	-2.270	25.916	23.286	1.00	30.47	H	0.069
ATOM	1673	HH2	TRP	A	108	-1.921	23.648	23.467	1.00	28.41	H	0.067
ATOM	1674	N	VAL	A	109	4.760	29.854	21.431	1.00	27.66	N	0.066
ATOM	1675	CA	VAL	A	109	5.351	31.143	21.068	1.00	33.88	C	0.073
ATOM	1676	C	VAL	A	109	5.165	31.425	19.578	1.00	29.85	C	0.068

ATOM	1677	O	VAL	A	109	4.871	32.569	19.194	1.00	32.37	O	0.071
ATOM	1678	CB	VAL	A	109	6.828	31.189	21.518	1.00	39.88	C	0.079
ATOM	1679	CG1	VAL	A	109	7.512	32.446	21.012	1.00	44.33	C	0.083
ATOM	1680	CG2	VAL	A	109	6.870	31.190	23.063	1.00	40.70	C	0.080
ATOM	1681	H	VAL	A	109	5.313	29.261	21.717	1.00	33.19	H	0.072
ATOM	1682	HA	VAL	A	109	4.881	31.840	21.552	1.00	40.65	H	0.080
ATOM	1683	HB	VAL	A	109	7.304	30.411	21.187	1.00	47.85	H	0.086
ATOM	1684	HG11	VAL	A	109	8.360	32.549	21.472	1.00	53.19	H	0.091
ATOM	1685	HG12	VAL	A	109	7.663	32.364	20.057	1.00	53.19	H	0.091
ATOM	1686	HG13	VAL	A	109	6.943	33.210	21.195	1.00	53.19	H	0.091
ATOM	1687	HG21	VAL	A	109	7.794	31.235	23.353	1.00	48.83	H	0.087
ATOM	1688	HG22	VAL	A	109	6.382	31.962	23.391	1.00	48.83	H	0.087
ATOM	1689	HG23	VAL	A	109	6.459	30.375	23.391	1.00	48.83	H	0.087
ATOM	1690	N	ALA	A	110	5.251	30.383	18.730	1.00	22.22	N	0.059
ATOM	1691	CA	ALA	A	110	5.013	30.606	17.297	1.00	23.69	C	0.061
ATOM	1692	C	ALA	A	110	3.583	31.064	17.017	1.00	27.88	C	0.066
ATOM	1693	O	ALA	A	110	3.336	31.848	16.077	1.00	24.69	O	0.062
ATOM	1694	CB	ALA	A	110	5.268	29.371	16.484	1.00	26.48	C	0.064
ATOM	1695	H	ALA	A	110	5.437	29.573	18.950	1.00	26.65	H	0.064
ATOM	1696	HA	ALA	A	110	5.615	31.299	16.984	1.00	28.42	H	0.067
ATOM	1697	HB1	ALA	A	110	5.099	29.568	15.549	1.00	31.77	H	0.070
ATOM	1698	HB2	ALA	A	110	6.192	29.100	16.603	1.00	31.77	H	0.070
ATOM	1699	HB3	ALA	A	110	4.674	28.666	16.787	1.00	31.77	H	0.070
ATOM	1700	N	TRP	A	111	2.621	30.579	17.777	1.00	25.62	N	0.063
ATOM	1701	CA	TRP	A	111	1.258	31.064	17.563	1.00	24.61	C	0.062
ATOM	1702	C	TRP	A	111	1.158	32.539	17.954	1.00	27.75	C	0.066
ATOM	1703	O	TRP	A	111	0.570	33.351	17.236	1.00	25.67	O	0.063
ATOM	1704	CB	TRP	A	111	0.248	30.241	18.384	1.00	23.17	C	0.060
ATOM	1705	CG	TRP	A	111	-1.134	30.801	18.291	1.00	23.80	C	0.061
ATOM	1706	CD1	TRP	A	111	-2.066	30.511	17.318	1.00	22.94	C	0.060
ATOM	1707	CD2	TRP	A	111	-1.751	31.785	19.162	1.00	24.80	C	0.062
ATOM	1708	NE1	TRP	A	111	-3.206	31.263	17.549	1.00	20.91	N	0.057
ATOM	1709	CE2	TRP	A	111	-3.039	32.013	18.686	1.00	25.80	C	0.063
ATOM	1710	CE3	TRP	A	111	-1.334	32.439	20.315	1.00	27.10	C	0.065
ATOM	1711	CZ2	TRP	A	111	-3.930	32.911	19.301	1.00	26.37	C	0.064
ATOM	1712	CZ3	TRP	A	111	-2.196	33.318	20.936	1.00	23.90	C	0.061
ATOM	1713	CH2	TRP	A	111	-3.503	33.549	20.425	1.00	31.40	C	0.070
ATOM	1714	H	TRP	A	111	2.715	29.993	18.400	1.00	30.74	H	0.069
ATOM	1715	HA	TRP	A	111	1.030	30.980	16.624	1.00	29.52	H	0.068
ATOM	1716	HB2	TRP	A	111	0.229	29.332	18.047	1.00	27.79	H	0.066
ATOM	1717	HB3	TRP	A	111	0.515	30.247	19.317	1.00	27.79	H	0.066
ATOM	1718	HD1	TRP	A	111	-1.938	29.929	16.604	1.00	27.53	H	0.065
ATOM	1719	HE1	TRP	A	111	-3.924	31.233	17.076	1.00	25.09	H	0.063
ATOM	1720	HE3	TRP	A	111	-0.483	32.290	20.660	1.00	32.52	H	0.071
ATOM	1721	HZ2	TRP	A	111	-4.782	33.056	18.958	1.00	31.64	H	0.070
ATOM	1722	HZ3	TRP	A	111	-1.920	33.763	21.704	1.00	28.68	H	0.067
ATOM	1723	HH2	TRP	A	111	-4.068	34.148	20.858	1.00	37.67	H	0.077
ATOM	1724	N	ARG	A	112	1.685	32.900	19.132	1.00	23.93	N	0.061
ATOM	1725	CA	ARG	A	112	1.613	34.297	19.534	1.00	29.36	C	0.068
ATOM	1726	C	ARG	A	112	2.268	35.192	18.471	1.00	25.23	C	0.063
ATOM	1727	O	ARG	A	112	1.747	36.286	18.116	1.00	24.88	O	0.062
ATOM	1728	CB	ARG	A	112	2.280	34.528	20.889	1.00	27.57	C	0.066
ATOM	1729	CG	ARG	A	112	2.160	36.000	21.290	1.00	27.68	C	0.066
ATOM	1730	CD	ARG	A	112	2.534	36.227	22.756	1.00	37.56	C	0.076
ATOM	1731	NE	ARG	A	112	3.780	35.545	23.079	1.00	42.12	N	0.081
ATOM	1732	CZ	ARG	A	112	4.998	36.021	22.829	1.00	60.41	C	0.097
ATOM	1733	NH1	ARG	A	112	5.179	37.171	22.170	1.00	38.86	N	0.078
ATOM	1734	NH2	ARG	A	112	6.069	35.341	23.272	1.00	42.63	N	0.081
ATOM	1735	H	ARG	A	112	2.073	32.374	19.691	1.00	28.71	H	0.067
ATOM	1736	HA	ARG	A	112	0.681	34.555	19.611	1.00	35.22	H	0.074
ATOM	1737	HB2	ARG	A	112	1.840	33.988	21.564	1.00	33.08	H	0.072
ATOM	1738	HB3	ARG	A	112	3.221	34.300	20.830	1.00	33.08	H	0.072
ATOM	1739	HG2	ARG	A	112	2.760	36.532	20.744	1.00	33.22	H	0.072
ATOM	1740	HG3	ARG	A	112	1.244	36.292	21.163	1.00	33.22	H	0.072
ATOM	1741	HD2	ARG	A	112	2.652	37.176	22.917	1.00	45.07	H	0.084
ATOM	1742	HD3	ARG	A	112	1.834	35.872	23.326	1.00	45.07	H	0.084
ATOM	1743	HE	ARG	A	112	3.725	34.776	23.460	1.00	50.54	H	0.089
ATOM	1744	HH11	ARG	A	112	4.501	37.618	21.888	1.00	46.63	H	0.085
ATOM	1745	HH12	ARG	A	112	5.975	37.460	22.021	1.00	46.63	H	0.085
ATOM	1746	HH21	ARG	A	112	5.963	34.601	23.697	1.00	51.15	H	0.089
ATOM	1747	HH22	ARG	A	112	6.861	35.636	23.115	1.00	51.15	H	0.089
ATOM	1748	N	ASN	A	113	3.408	34.749	17.947	1.00	27.88	N	0.066
ATOM	1749	CA	ASN	A	113	4.173	35.634	17.086	1.00	25.63	C	0.063
ATOM	1750	C	ASN	A	113	3.831	35.536	15.606	1.00	24.55	C	0.062
ATOM	1751	O	ASN	A	113	4.218	36.439	14.842	1.00	26.13	O	0.064
ATOM	1752	CB	ASN	A	113	5.653	35.344	17.213	1.00	26.55	C	0.064
ATOM	1753	CG	ASN	A	113	6.201	35.812	18.490	1.00	27.72	C	0.066
ATOM	1754	OD1	ASN	A	113	5.793	36.852	19.017	1.00	31.28	O	0.070

ATOM	1755	ND2	ASN	A	113	7.205	35.116	18.961	1.00	27.76	N	0.066
ATOM	1756	H	ASN	A	113	3.748	33.969	18.070	1.00	33.44	H	0.072
ATOM	1757	HA	ASN	A	113	4.024	36.551	17.364	1.00	30.75	H	0.069
ATOM	1758	HB2	ASN	A	113	5.795	34.386	17.156	1.00	31.86	H	0.070
ATOM	1759	HB3	ASN	A	113	6.128	35.795	16.497	1.00	31.86	H	0.070
ATOM	1760	HD21	ASN	A	113	7.576	35.345	19.702	1.00	33.31	H	0.072
ATOM	1761	HD22	ASN	A	113	7.467	34.408	18.550	1.00	33.31	H	0.072
ATOM	1762	N	ARG	A	114	3.121	34.481	15.154	1.00	24.74	N	0.062
ATOM	1763	CA	ARG	A	114	2.877	34.379	13.728	1.00	25.66	C	0.063
ATOM	1764	C	ARG	A	114	1.422	34.108	13.387	1.00	25.04	C	0.062
ATOM	1765	O	ARG	A	114	1.050	34.219	12.208	1.00	23.08	O	0.060
ATOM	1766	CB	ARG	A	114	3.778	33.290	13.119	1.00	30.87	C	0.069
ATOM	1767	CG	ARG	A	114	5.233	33.464	13.568	1.00	32.32	C	0.071
ATOM	1768	CD	ARG	A	114	6.103	32.364	13.095	1.00	31.39	C	0.070
ATOM	1769	NE	ARG	A	114	6.261	32.372	11.639	1.00	27.82	N	0.066
ATOM	1770	CZ	ARG	A	114	7.097	31.576	10.984	1.00	25.60	C	0.063
ATOM	1771	NH1	ARG	A	114	7.815	30.664	11.621	1.00	26.21	N	0.064
ATOM	1772	NH2	ARG	A	114	7.224	31.716	9.667	1.00	28.78	N	0.067
ATOM	1773	H	ARG	A	114	2.791	33.849	15.636	1.00	29.68	H	0.068
ATOM	1774	HA	ARG	A	114	3.113	35.216	13.302	1.00	30.78	H	0.069
ATOM	1775	HB2	ARG	A	114	3.470	32.418	13.411	1.00	37.04	H	0.076
ATOM	1776	HB3	ARG	A	114	3.748	33.352	12.151	1.00	37.04	H	0.076
ATOM	1777	HG2	ARG	A	114	5.579	34.297	13.211	1.00	38.77	H	0.078
ATOM	1778	HG3	ARG	A	114	5.276	33.472	14.536	1.00	38.77	H	0.078
ATOM	1779	HD2	ARG	A	114	6.982	32.459	13.495	1.00	37.66	H	0.077
ATOM	1780	HD3	ARG	A	114	5.710	31.515	13.351	1.00	37.66	H	0.077
ATOM	1781	HE	ARG	A	114	5.785	32.924	11.182	1.00	33.38	H	0.072
ATOM	1782	HH11	ARG	A	114	7.741	30.572	12.473	1.00	31.45	H	0.070
ATOM	1783	HH12	ARG	A	114	8.353	30.158	11.180	1.00	31.45	H	0.070
ATOM	1784	HH21	ARG	A	114	6.759	32.308	9.251	1.00	34.53	H	0.073
ATOM	1785	HH22	ARG	A	114	7.760	31.205	9.230	1.00	34.53	H	0.073
ATOM	1786	N	CYS	A	115	0.594	33.852	14.358	1.00	23.92	N	0.061
ATOM	1787	CA	CYS	A	115	-0.820	33.522	14.109	1.00	25.55	C	0.063
ATOM	1788	C	CYS	A	115	-1.763	34.479	14.806	1.00	28.42	C	0.067
ATOM	1789	O	CYS	A	115	-2.737	34.936	14.220	1.00	25.76	O	0.063
ATOM	1790	CB	CYS	A	115	-1.121	32.092	14.618	1.00	24.41	C	0.062
ATOM	1791	SG	CYS	A	115	-0.153	30.778	13.852	1.00	25.82	S	0.063
ATOM	1792	H	CYS	A	115	0.807	33.857	15.191	1.00	28.70	H	0.067
ATOM	1793	HA	CYS	A	115	-1.003	33.552	13.157	1.00	30.66	H	0.069
ATOM	1794	HB2	CYS	A	115	-0.928	32.074	15.566	1.00	29.28	H	0.068
ATOM	1795	HB3	CYS	A	115	-2.059	31.897	14.468	1.00	29.28	H	0.068
ATOM	1796	N	LYS	A	116	-1.518	34.748	16.062	1.00	26.51	N	0.064
ATOM	1797	CA	LYS	A	116	-2.370	35.617	16.841	1.00	25.09	C	0.063
ATOM	1798	C	LYS	A	116	-2.648	36.915	16.099	1.00	27.45	C	0.065
ATOM	1799	O	LYS	A	116	-1.733	37.589	15.634	1.00	24.21	O	0.061
ATOM	1800	CB	LYS	A	116	-1.666	35.931	18.184	1.00	22.99	C	0.060
ATOM	1801	CG	LYS	A	116	-2.547	36.744	19.139	1.00	26.14	C	0.064
ATOM	1802	CD	LYS	A	116	-1.695	36.985	20.422	1.00	22.68	C	0.059
ATOM	1803	CE	LYS	A	116	-2.493	37.522	21.581	1.00	27.27	C	0.065
ATOM	1804	NZ	LYS	A	116	-1.596	37.576	22.766	1.00	24.38	N	0.062
ATOM	1805	H	LYS	A	116	-0.849	34.434	16.501	1.00	31.81	H	0.070
ATOM	1806	HA	LYS	A	116	-3.213	35.174	17.025	1.00	30.10	H	0.068
ATOM	1807	HB2	LYS	A	116	-1.437	35.097	18.623	1.00	27.58	H	0.066
ATOM	1808	HB3	LYS	A	116	-0.862	36.444	18.007	1.00	27.58	H	0.066
ATOM	1809	HG2	LYS	A	116	-2.775	37.600	18.743	1.00	31.36	H	0.070
ATOM	1810	HG3	LYS	A	116	-3.342	36.241	19.373	1.00	31.36	H	0.070
ATOM	1811	HD2	LYS	A	116	-1.299	36.144	20.698	1.00	27.21	H	0.065
ATOM	1812	HD3	LYS	A	116	-0.997	37.627	20.219	1.00	27.21	H	0.065
ATOM	1813	HE2	LYS	A	116	-2.808	38.418	21.381	1.00	32.72	H	0.071
ATOM	1814	HE3	LYS	A	116	-3.234	36.927	21.774	1.00	32.72	H	0.071
ATOM	1815	HZ1	LYS	A	116	-1.294	36.761	22.956	1.00	29.25	H	0.067
ATOM	1816	HZ2	LYS	A	116	-0.904	38.110	22.599	1.00	29.25	H	0.067
ATOM	1817	HZ3	LYS	A	116	-2.041	37.891	23.470	1.00	29.25	H	0.067
ATOM	1818	N	GLY	A	117	-3.916	37.237	15.954	1.00	25.44	N	0.063
ATOM	1819	CA	GLY	A	117	-4.254	38.512	15.395	1.00	33.19	C	0.072
ATOM	1820	C	GLY	A	117	-4.289	38.518	13.887	1.00	41.93	C	0.081
ATOM	1821	O	GLY	A	117	-4.651	39.531	13.293	1.00	40.37	O	0.079
ATOM	1822	H	GLY	A	117	-4.585	36.742	16.169	1.00	30.52	H	0.069
ATOM	1823	HA2	GLY	A	117	-5.128	38.780	15.719	1.00	39.83	H	0.079
ATOM	1824	HA3	GLY	A	117	-3.606	39.172	15.686	1.00	39.83	H	0.079
ATOM	1825	N	THR	A	118	-3.892	37.426	13.249	1.00	26.52	N	0.064
ATOM	1826	CA	THR	A	118	-3.949	37.335	11.809	1.00	28.94	C	0.067
ATOM	1827	C	THR	A	118	-5.264	36.693	11.380	1.00	29.50	C	0.068
ATOM	1828	O	THR	A	118	-6.109	36.283	12.177	1.00	26.77	O	0.065
ATOM	1829	CB	THR	A	118	-2.760	36.542	11.272	1.00	29.77	C	0.068
ATOM	1830	OG1	THR	A	118	-2.988	35.143	11.526	1.00	30.96	O	0.069
ATOM	1831	CG2	THR	A	118	-1.473	37.031	11.923	1.00	26.72	C	0.065
ATOM	1832	H	THR	A	118	-3.584	36.721	13.633	1.00	31.82	H	0.070

ATOM	1833	HA	THR	A	118	-3.910	38.226	11.429	1.00	34.72	H	0.074
ATOM	1834	HB	THR	A	118	-2.688	36.684	10.315	1.00	35.72	H	0.075
ATOM	1835	HG1	THR	A	118	-3.066	35.010	12.352	1.00	37.14	H	0.076
ATOM	1836	HG21	THR	A	118	-0.706	36.661	11.458	1.00	32.06	H	0.071
ATOM	1837	HG22	THR	A	118	-1.429	37.999	11.882	1.00	32.06	H	0.071
ATOM	1838	HG23	THR	A	118	-1.443	36.753	12.852	1.00	32.06	H	0.071
ATOM	1839	N	ASP	A	119	-5.455	36.589	10.067	1.00	31.38	N	0.070
ATOM	1840	CA	ASP	A	119	-6.673	35.963	9.508	1.00	26.02	C	0.064
ATOM	1841	C	ASP	A	119	-6.449	34.459	9.525	1.00	27.41	C	0.065
ATOM	1842	O	ASP	A	119	-6.012	33.924	8.516	1.00	33.98	O	0.073
ATOM	1843	CB	ASP	A	119	-6.961	36.479	8.102	1.00	38.43	C	0.077
ATOM	1844	CG	ASP	A	119	-8.257	35.956	7.517	1.00	47.54	C	0.086
ATOM	1845	OD1	ASP	A	119	-8.752	34.920	7.985	1.00	36.74	O	0.076
ATOM	1846	OD2	ASP	A	119	-8.749	36.599	6.606	1.00	33.42	O	0.072
ATOM	1847	H	ASP	A	119	-4.901	36.870	9.472	1.00	37.65	H	0.077
ATOM	1848	HA	ASP	A	119	-7.434	36.169	10.074	1.00	31.22	H	0.070
ATOM	1849	HB2	ASP	A	119	-7.017	37.447	8.129	1.00	46.11	H	0.085
ATOM	1850	HB3	ASP	A	119	-6.239	36.208	7.514	1.00	46.11	H	0.085
ATOM	1851	N	VAL	A	120	-6.727	33.810	10.643	1.00	30.77	N	0.069
ATOM	1852	CA	VAL	A	120	-6.431	32.388	10.749	1.00	34.65	C	0.073
ATOM	1853	C	VAL	A	120	-7.420	31.555	9.958	1.00	28.02	C	0.066
ATOM	1854	O	VAL	A	120	-7.143	30.381	9.682	1.00	29.25	O	0.067
ATOM	1855	CB	VAL	A	120	-6.380	31.976	12.233	1.00	32.97	C	0.072
ATOM	1856	CG1	VAL	A	120	-5.159	32.635	12.897	1.00	34.11	C	0.073
ATOM	1857	CG2	VAL	A	120	-7.696	32.376	12.960	1.00	35.72	C	0.075
ATOM	1858	H	VAL	A	120	-7.079	34.164	11.342	1.00	36.92	H	0.076
ATOM	1859	HA	VAL	A	120	-5.551	32.229	10.373	1.00	41.57	H	0.080
ATOM	1860	HB	VAL	A	120	-6.278	31.014	12.295	1.00	39.56	H	0.078
ATOM	1861	HG11	VAL	A	120	-5.119	32.360	13.826	1.00	40.92	H	0.080
ATOM	1862	HG12	VAL	A	120	-4.356	32.351	12.432	1.00	40.92	H	0.080
ATOM	1863	HG13	VAL	A	120	-5.251	33.599	12.841	1.00	40.92	H	0.080
ATOM	1864	HG21	VAL	A	120	-7.732	31.918	13.814	1.00	42.86	H	0.082
ATOM	1865	HG22	VAL	A	120	-7.704	33.335	13.101	1.00	42.86	H	0.082
ATOM	1866	HG23	VAL	A	120	-8.454	32.113	12.414	1.00	42.86	H	0.082
ATOM	1867	N	GLN	A	121	-8.573	32.130	9.602	1.00	33.31	N	0.072
ATOM	1868	CA	GLN	A	121	-9.565	31.416	8.808	1.00	28.36	C	0.066
ATOM	1869	C	GLN	A	121	-8.984	31.004	7.472	1.00	33.34	C	0.072
ATOM	1870	O	GLN	A	121	-9.356	29.977	6.922	1.00	30.83	O	0.069
ATOM	1871	CB	GLN	A	121	-10.797	32.310	8.624	1.00	34.78	C	0.074
ATOM	1872	CG	GLN	A	121	-11.905	31.733	7.770	1.00	56.87	C	0.094
ATOM	1873	CD	GLN	A	121	-13.039	31.206	8.651	1.00	69.71	C	0.104
ATOM	1874	OE1	GLN	A	121	-13.636	31.967	9.418	1.00	76.71	O	0.109
ATOM	1875	NE2	GLN	A	121	-13.357	29.917	8.532	1.00	72.53	N	0.106
ATOM	1876	H	GLN	A	121	-8.802	32.932	9.810	1.00	39.97	H	0.079
ATOM	1877	HA	GLN	A	121	-9.838	30.615	9.281	1.00	34.03	H	0.073
ATOM	1878	HB2	GLN	A	121	-11.172	32.498	9.499	1.00	41.73	H	0.081
ATOM	1879	HB3	GLN	A	121	-10.514	33.140	8.209	1.00	41.73	H	0.081
ATOM	1880	HG2	GLN	A	121	-12.261	32.415	7.184	1.00	68.24	H	0.103
ATOM	1881	HG3	GLN	A	121	-11.567	30.990	7.247	1.00	68.24	H	0.103
ATOM	1882	HE21	GLN	A	121	-12.923	29.416	7.985	1.00	87.03	H	0.116
ATOM	1883	HE22	GLN	A	121	-13.991	29.585	9.009	1.00	87.03	H	0.116
ATOM	1884	N	ALA	A	122	-8.038	31.767	6.948	1.00	24.10	N	0.061
ATOM	1885	CA	ALA	A	122	-7.375	31.371	5.716	1.00	24.64	C	0.062
ATOM	1886	C	ALA	A	122	-6.738	29.991	5.751	1.00	31.02	C	0.069
ATOM	1887	O	ALA	A	122	-6.637	29.333	4.711	1.00	29.95	O	0.068
ATOM	1888	CB	ALA	A	122	-6.297	32.420	5.387	1.00	32.12	C	0.071
ATOM	1889	H	ALA	A	122	-7.763	32.511	7.281	1.00	28.92	H	0.067
ATOM	1890	HA	ALA	A	122	-8.025	31.378	4.996	1.00	29.56	H	0.068
ATOM	1891	HB1	ALA	A	122	-5.849	32.164	4.566	1.00	38.53	H	0.077
ATOM	1892	HB2	ALA	A	122	-6.722	33.285	5.277	1.00	38.53	H	0.077
ATOM	1893	HB3	ALA	A	122	-5.658	32.455	6.116	1.00	38.53	H	0.077
ATOM	1894	N	TRP	A	123	-6.290	29.538	6.914	1.00	28.16	N	0.066
ATOM	1895	CA	TRP	A	123	-5.655	28.226	7.006	1.00	24.45	C	0.062
ATOM	1896	C	TRP	A	123	-6.618	27.072	6.737	1.00	29.47	C	0.068
ATOM	1897	O	TRP	A	123	-6.158	25.936	6.505	1.00	32.62	O	0.071
ATOM	1898	CB	TRP	A	123	-5.019	28.067	8.391	1.00	31.29	C	0.070
ATOM	1899	CG	TRP	A	123	-3.754	28.901	8.454	1.00	26.30	C	0.064
ATOM	1900	CD1	TRP	A	123	-3.603	30.089	9.104	1.00	31.36	C	0.070
ATOM	1901	CD2	TRP	A	123	-2.496	28.616	7.833	1.00	24.41	C	0.062
ATOM	1901	NE1	TRP	A	123	-2.328	30.572	8.907	1.00	31.73	N	0.070
ATOM	1903	CE2	TRP	A	123	-1.621	29.683	8.148	1.00	26.71	C	0.064
ATOM	1904	CE3	TRP	A	123	-2.027	27.562	7.036	1.00	26.71	C	0.064
ATOM	1905	CZ2	TRP	A	123	-0.302	29.728	7.712	1.00	36.47	C	0.075
ATOM	1906	CZ3	TRP	A	123	-0.694	27.601	6.600	1.00	38.14	C	0.077
ATOM	1907	CH2	TRP	A	123	0.141	28.702	6.927	1.00	34.83	C	0.074
ATOM	1908	H	TRP	A	123	-6.339	29.964	7.659	1.00	33.79	H	0.073
ATOM	1909	HA	TRP	A	123	-4.945	28.177	6.347	1.00	29.33	H	0.068
ATOM	1910	HB2	TRP	A	123	-5.634	28.381	9.072	1.00	37.54	H	0.076

ATOM	1911	HB3	TRP	A	123	-4.787	27.137	8.540	1.00	37.54	H	0.076
ATOM	1912	HD1	TRP	A	123	-4.270	30.523	9.585	1.00	37.62	H	0.077
ATOM	1913	HE1	TRP	A	123	-2.016	31.301	9.239	1.00	38.06	H	0.077
ATOM	1914	HE3	TRP	A	123	-2.580	26.848	6.816	1.00	32.05	H	0.071
ATOM	1915	HZ2	TRP	A	123	0.252	30.443	7.928	1.00	43.76	H	0.083
ATOM	1916	HZ3	TRP	A	123	-0.363	26.914	6.067	1.00	45.76	H	0.084
ATOM	1917	HH2	TRP	A	123	1.022	28.707	6.630	1.00	41.80	H	0.081
ATOM	1918	N	ILE	A	124	-7.921	27.305	6.854	1.00	32.56	N	0.071
ATOM	1919	CA	ILE	A	124	-8.925	26.274	6.585	1.00	30.88	C	0.069
ATOM	1920	C	ILE	A	124	-9.772	26.591	5.349	1.00	30.69	C	0.069
ATOM	1921	O	ILE	A	124	-10.687	25.811	5.030	1.00	32.97	O	0.072
ATOM	1922	CB	ILE	A	124	-9.838	26.017	7.800	1.00	32.82	C	0.071
ATOM	1923	CG1	ILE	A	124	-10.771	27.194	8.069	1.00	35.72	C	0.075
ATOM	1924	CG2	ILE	A	124	-9.015	25.655	9.082	1.00	27.95	C	0.066
ATOM	1925	CD1	ILE	A	124	-11.845	26.916	9.156	1.00	41.36	C	0.080
ATOM	1926	H	ILE	A	124	-8.256	28.061	7.091	1.00	39.07	H	0.078
ATOM	1927	HA	ILE	A	124	-8.463	25.442	6.402	1.00	37.05	H	0.076
ATOM	1928	HB	ILE	A	124	-10.393	25.251	7.587	1.00	39.38	H	0.078
ATOM	1929	HG12	ILE	A	124	-10.240	27.950	8.365	1.00	42.85	H	0.082
ATOM	1930	HG13	ILE	A	124	-11.245	27.426	7.256	1.00	42.85	H	0.082
ATOM	1931	HG21	ILE	A	124	-9.625	25.383	9.785	1.00	33.53	H	0.072
ATOM	1932	HG22	ILE	A	124	-8.409	24.928	8.871	1.00	33.53	H	0.072
ATOM	1933	HG23	ILE	A	124	-8.511	26.434	9.363	1.00	33.53	H	0.072
ATOM	1934	HD11	ILE	A	124	-12.455	27.667	9.199	1.00	49.63	H	0.088
ATOM	1935	HD12	ILE	A	124	-12.330	26.110	8.920	1.00	49.63	H	0.088
ATOM	1936	HD13	ILE	A	124	-11.405	26.798	10.012	1.00	49.63	H	0.088
ATOM	1937	N	ARG	A	125	-9.464	27.674	4.618	1.00	36.51	N	0.075
ATOM	1938	CA	ARG	A	125	-10.226	28.081	3.424	1.00	40.61	C	0.080
ATOM	1939	C	ARG	A	125	-10.147	27.010	2.342	1.00	33.51	C	0.072
ATOM	1940	O	ARG	A	125	-9.077	26.434	2.068	1.00	32.21	O	0.071
ATOM	1941	CB	ARG	A	125	-9.714	29.435	2.888	1.00	50.96	C	0.089
ATOM	1942	CG	ARG	A	125	-10.528	30.071	1.722	1.00	56.40	C	0.094
ATOM	1943	CD	ARG	A	125	-9.670	30.815	0.675	1.00	67.91	C	0.103
ATOM	1944	NE	ARG	A	125	-9.067	32.084	1.102	1.00	83.10	N	0.114
ATOM	1945	CZ	ARG	A	125	-7.767	32.296	1.305	1.00	69.19	C	0.104
ATOM	1946	NH1	ARG	A	125	-6.891	31.299	1.337	1.00	65.35	N	0.101
ATOM	1947	NH2	ARG	A	125	-7.329	33.545	1.466	1.00	50.43	N	0.089
ATOM	1948	H	ARG	A	125	-8.806	28.199	4.796	1.00	43.81	H	0.083
ATOM	1949	HA	ARG	A	125	-11.158	28.190	3.668	1.00	48.73	H	0.087
ATOM	1950	HB2	ARG	A	125	-9.715	30.071	3.620	1.00	61.14	H	0.098
ATOM	1951	HB3	ARG	A	125	-8.805	29.312	2.571	1.00	61.14	H	0.098
ATOM	1952	HG2	ARG	A	125	-11.013	29.369	1.260	1.00	67.68	H	0.103
ATOM	1953	HG3	ARG	A	125	-11.156	30.710	2.095	1.00	67.68	H	0.103
ATOM	1954	HD2	ARG	A	125	-8.957	30.224	0.390	1.00	81.49	H	0.113
ATOM	1955	HD3	ARG	A	125	-10.237	31.015	-0.086	1.00	81.49	H	0.113
ATOM	1956	HE	ARG	A	125	-9.598	32.747	1.232	1.00	99.71	H	0.125
ATOM	1957	HH11	ARG	A	125	-7.152	30.488	1.226	1.00	78.41	H	0.110
ATOM	1958	HH12	ARG	A	125	-6.058	31.465	1.473	1.00	78.41	H	0.110
ATOM	1959	HH21	ARG	A	125	-7.885	34.201	1.452	1.00	60.52	H	0.097
ATOM	1960	HH22	ARG	A	125	-6.494	33.693	1.609	1.00	60.52	H	0.097
ATOM	1961	N	GLY	A	126	-11.320	26.668	1.790	1.00	39.06	N	0.078
ATOM	1962	CA	GLY	A	126	-11.420	25.681	0.744	1.00	43.05	C	0.082
ATOM	1963	C	GLY	A	126	-11.512	24.237	1.193	1.00	43.78	C	0.083
ATOM	1964	O	GLY	A	126	-11.881	23.381	0.371	1.00	46.90	O	0.085
ATOM	1965	H	GLY	A	126	-12.076	27.008	2.018	1.00	46.86	H	0.085
ATOM	1966	HA2	GLY	A	126	-12.207	25.874	0.210	1.00	51.65	H	0.090
ATOM	1967	HA3	GLY	A	126	-10.644	25.761	0.167	1.00	51.65	H	0.090
ATOM	1968	N	CYS	A	127	-11.180	23.939	2.460	1.00	35.49	N	0.074
ATOM	1969	CA	CYS	A	127	-11.098	22.558	2.934	1.00	33.23	C	0.072
ATOM	1970	C	CYS	A	127	-12.500	21.972	3.101	1.00	42.09	C	0.081
ATOM	1971	O	CYS	A	127	-13.423	22.664	3.546	1.00	39.57	O	0.078
ATOM	1972	CB	CYS	A	127	-10.388	22.510	4.280	1.00	27.05	C	0.065
ATOM	1973	SG	CYS	A	127	-8.768	23.279	4.272	1.00	35.36	S	0.074
ATOM	1974	H	CYS	A	127	-10.998	24.525	3.062	1.00	42.59	H	0.081
ATOM	1975	HA	CYS	A	127	-10.605	22.018	2.297	1.00	39.87	H	0.079
ATOM	1976	HB2	CYS	A	127	-10.932	22.972	4.937	1.00	32.45	H	0.071
ATOM	1977	HB3	CYS	A	127	-10.275	21.583	4.541	1.00	32.45	H	0.071
ATOM	1978	N	ARG	A	128	-12.656	20.682	2.765	1.00	38.23	N	0.077
ATOM	1979	CA	ARG	A	128	-13.903	19.968	3.047	1.00	45.25	C	0.084
ATOM	1980	C	ARG	A	128	-13.940	19.615	4.526	1.00	45.20	C	0.084
ATOM	1981	O	ARG	A	128	-13.110	18.825	5.010	1.00	49.15	O	0.087
ATOM	1982	CB	ARG	A	128	-14.035	18.720	2.177	1.00	51.40	C	0.089
ATOM	1983	CG	ARG	A	128	-15.504	18.435	1.814	1.00	71.19	C	0.105
ATOM	1984	CD	ARG	A	128	-15.842	16.956	1.537	1.00	72.56	C	0.106
ATOM	1985	NE	ARG	A	128	-15.207	16.582	0.278	1.00	73.37	N	0.107
ATOM	1986	CZ	ARG	A	128	-15.853	16.209	-0.817	1.00	76.04	C	0.109
ATOM	1987	NH1	ARG	A	128	-17.177	16.222	-0.879	1.00	72.63	N	0.106
ATOM	1988	NH2	ARG	A	128	-15.152	15.855	-1.888	1.00	80.91	N	0.112

```
ATOM   1989  H   ARG A 128     -12.056  20.204   2.375  1.00 45.87           H  0.085
ATOM   1990  HA  ARG A 128     -14.654  20.551   2.856  1.00 54.29           H  0.092
ATOM   1991  HB2 ARG A 128     -13.539  18.850   1.354  1.00 61.68           H  0.098
ATOM   1992  HB3 ARG A 128     -13.688  17.954   2.661  1.00 61.68           H  0.098
ATOM   1993  HG2 ARG A 128     -16.064  18.728   2.550  1.00 85.43           H  0.115
ATOM   1994  HG3 ARG A 128     -15.727  18.937   1.020  1.00 85.43           H  0.115
ATOM   1995  HD2 ARG A 128     -15.475  16.402   2.244  1.00 87.06           H  0.116
ATOM   1996  HD3 ARG A 128     -16.802  16.844   1.481  1.00 87.06           H  0.116
ATOM   1997  HE  ARG A 128     -14.348  16.605   0.244  1.00 88.04           H  0.117
ATOM   1998 HH11 ARG A 128     -17.646  16.452  -0.198  1.00 87.14           H  0.116
ATOM   1999 HH12 ARG A 128     -17.571  15.982  -1.605  1.00 87.14           H  0.116
ATOM   2000 HH21 ARG A 128     -14.293  15.851  -1.857  1.00 97.09           H  0.123
ATOM   2001 HH22 ARG A 128     -15.558  15.618  -2.608  1.00 97.09           H  0.123
ATOM   2002  N   LEU A 129     -14.893  20.206   5.247  1.00 48.85           N  0.087
ATOM   2003  CA  LEU A 129     -15.044  20.015   6.697  1.00 55.87           C  0.093
ATOM   2004  C   LEU A 129     -16.519  19.683   7.024  1.00 71.18           C  0.105
ATOM   2005  O   LEU A 129     -17.429  19.867   6.193  1.00 84.20           O  0.115
ATOM   2006  CB  LEU A 129     -14.574  21.264   7.460  1.00 54.53           C  0.092
ATOM   2007  CG  LEU A 129     -13.107  21.633   7.160  1.00 35.92           C  0.075
ATOM   2008  CD1 LEU A 129     -12.735  23.056   7.572  1.00 47.36           C  0.086
ATOM   2009  CD2 LEU A 129     -12.171  20.640   7.789  1.00 49.43           C  0.088
ATOM   2010  OXT LEU A 129     -16.830  19.202   8.124  1.00 78.06           O  0.110
ATOM   2011  H   LEU A 129     -15.481  20.737   4.912  1.00 58.61           H  0.096
ATOM   2012  HA  LEU A 129     -14.498  19.265   6.979  1.00 67.04           H  0.102
ATOM   2013  HB2 LEU A 129     -15.130  22.017   7.206  1.00 65.43           H  0.101
ATOM   2014  HB3 LEU A 129     -14.653  21.100   8.413  1.00 65.43           H  0.101
ATOM   2015  HG  LEU A 129     -12.973  21.578   6.201  1.00 43.10           H  0.082
ATOM   2016 HD11 LEU A 129     -11.805  23.216   7.348  1.00 56.83           H  0.094
ATOM   2017 HD12 LEU A 129     -13.303  23.681   7.095  1.00 56.83           H  0.094
ATOM   2018 HD13 LEU A 129     -12.866  23.152   8.528  1.00 56.83           H  0.094
ATOM   2019 HD21 LEU A 129     -11.258  20.934   7.647  1.00 59.31           H  0.096
ATOM   2020 HD22 LEU A 129     -12.358  20.589   8.739  1.00 59.31           H  0.096
ATOM   2021 HD23 LEU A 129     -12.307  19.772   7.378  1.00 59.31           H  0.096
TER
HETATM 2022 CL    CL A1130      -7.779  32.198  26.163  1.00 26.41          Cl  0.064
HETATM 2023 CL    CL A1131     -10.727  29.516  11.760  1.00 41.96          Cl  0.081
HETATM 2024 CL    CL A1132      13.242  13.206  32.525  1.00 43.66          Cl  0.082
HETATM 2025 CL    CL A1134       0.479  25.438   3.692  1.00 64.43          Cl  0.100
HETATM 2026 CL    CL A1135       7.375   7.523  29.506  1.00 48.82          Cl  0.087
HETATM 2027 CL    CL A1137      20.019  11.461  24.588  1.00 56.46          Cl  0.094
HETATM 2028 NA    NA A1138       9.541  15.663  31.385  1.00 38.56          Na  0.077
HETATM 2029  O   HOH A2001       7.242  12.509   9.002  1.00 27.36           O  0.065
HETATM 2030  O   HOH A2002       4.621   8.856  10.690  1.00 34.89           O  0.074
HETATM 2031  O   HOH A2008      -4.237  21.091   1.629  1.00 35.47           O  0.074
HETATM 2032  O   HOH A2012      -4.645  18.038   2.681  1.00 31.47           O  0.070
HETATM 2033  O   HOH A2027     -13.488  24.252  25.119  1.00 32.09           O  0.071
HETATM 2034  O   HOH A2028     -10.389  32.127  23.678  1.00 27.47           O  0.065
HETATM 2035  O   HOH A2029      16.047  12.891  34.142  1.00 38.45           O  0.077
HETATM 2036  O   HOH A2030      -6.064  34.424  16.599  1.00 34.27           O  0.073
HETATM 2037  O   HOH A2031      -9.093  33.569  15.847  1.00 36.21           O  0.075
HETATM 2038  O   HOH A2037      -2.754  10.584  25.345  1.00 38.38           O  0.077
HETATM 2039  O   HOH A2038       8.363  27.808  15.371  1.00 30.31           O  0.069
HETATM 2040  O   HOH A2039       8.389  26.497  11.167  1.00 32.10           O  0.071
HETATM 2041  O   HOH A2044       9.427  19.880  10.438  1.00 37.02           O  0.076
HETATM 2042  O   HOH A2045       4.467  20.693   5.267  1.00 34.56           O  0.073
HETATM 2043  O   HOH A2047      -0.709  40.170  19.275  1.00 34.89           O  0.074
HETATM 2044  O   HOH A2051      12.711  14.723  14.611  1.00 26.08           O  0.064
HETATM 2045  O   HOH A2065      15.161  16.478  26.199  1.00 27.99           O  0.066
HETATM 2046  O   HOH A2067       2.909  17.936  19.345  1.00 21.87           O  0.058
HETATM 2047  O   HOH A2068      10.284  23.944  18.594  1.00 32.63           O  0.071
HETATM 2048  O   HOH A2069       6.472  24.959  21.384  1.00 37.02           O  0.076
HETATM 2049  O   HOH A2070       5.523  23.120  25.025  1.00 31.24           O  0.070
HETATM 2050  O   HOH A2074       5.135  25.140  28.247  1.00 43.56           O  0.082
HETATM 2051  O   HOH A2076      11.943   7.347  30.838  1.00 29.23           O  0.067
HETATM 2052  O   HOH A2077      11.257  14.384  30.298  1.00 27.23           O  0.065
HETATM 2053  O   HOH A2078      18.977   9.722  28.933  1.00 32.98           O  0.072
HETATM 2054  O   HOH A2081      14.145  14.145  37.140  0.56 35.63           O  0.074
HETATM 2055  O   HOH A2082       9.039  13.281  32.769  1.00 38.72           O  0.078
HETATM 2056  O   HOH A2085       4.205   8.528  30.593  1.00 38.05           O  0.077
HETATM 2057  O   HOH A2086       6.831  10.462  34.020  1.00 35.86           O  0.075
HETATM 2058  O   HOH A2087      -2.339  13.425  27.711  1.00 37.94           O  0.077
HETATM 2059  O   HOH A2090       5.470   7.405  18.368  1.00 35.78           O  0.075
HETATM 2060  O   HOH A2092       0.603   8.255  20.321  1.00 35.29           O  0.074
HETATM 2061  O   HOH A2093       2.538  15.161  16.690  1.00 31.46           O  0.070
HETATM 2062  O   HOH A2094       5.605   8.781  12.952  1.00 36.67           O  0.076
HETATM 2063  O   HOH A2095       0.840  13.277  16.179  1.00 29.28           O  0.068
HETATM 2064  O   HOH A2096       3.111   6.586  13.682  1.00 38.61           O  0.078
HETATM 2065  O   HOH A2097      -2.975  10.323  10.706  1.00 41.52           O  0.080
```

```
HETATM 2066  O   HOH A2098      -1.537   9.365  19.534  1.00 33.21           O  0.072
HETATM 2067  O   HOH A2099       0.350  12.262  18.717  1.00 31.12           O  0.070
HETATM 2068  O   HOH A2102      -1.499  12.847  25.316  1.00 29.95           O  0.068
HETATM 2069  O   HOH A2106      -9.416  14.787  23.791  1.00 34.03           O  0.073
HETATM 2070  O   HOH A2107      -7.921  20.226  29.843  1.00 41.98           O  0.081
HETATM 2071  O   HOH A2109      -5.723  29.034  29.366  1.00 27.61           O  0.066
HETATM 2072  O   HOH A2110      -1.735  28.389  28.428  1.00 36.81           O  0.076
HETATM 2073  O   HOH A2112       6.363  27.556  22.570  1.00 31.89           O  0.070
HETATM 2074  O   HOH A2114       1.721  38.739  18.496  1.00 36.99           O  0.076
HETATM 2075  O   HOH A2116       8.624  29.581   8.305  1.00 26.99           O  0.065
HETATM 2076  O   HOH A2117       2.123  34.784   9.603  1.00 29.51           O  0.068
HETATM 2077  O   HOH A2118       4.910  34.296   9.827  1.00 26.13           O  0.064
HETATM 2078  O   HOH A2119       1.226  37.227  15.481  1.00 29.03           O  0.067
HETATM 2079  O   HOH A2121       0.115  39.610  21.783  1.00 32.08           O  0.071
HETATM 2080  O   HOH A2123      -3.399  40.201  17.974  1.00 34.45           O  0.073
HETATM 2081  O   HOH A2127      -2.120  33.616   9.383  1.00 36.74           O  0.076
HETATM 2082  O   HOH A2128      -6.961  35.965  14.891  1.00 34.91           O  0.074
HETATM 2083  O   HOH A2129      -3.852  38.253   8.270  1.00 34.29           O  0.073
HETATM 2084  O   HOH A2131      -9.709  34.992  11.290  1.00 38.26           O  0.077
HETATM 2085  O   HOH A2136     -20.102  13.635   1.949  1.00 34.74           O  0.074
HETATM 2086  O   HOH A2139     -10.428  19.294   1.305  1.00 40.89           O  0.080
HETATM 2087 RE    RE A2145     -15.422  25.279  12.464  0.28 42.60          Re  0.081
HETATM 2088  C1  RII A2147     -11.959  35.660   4.173  0.64 37.65      A    C  0.077
HETATM 2089  C2  RII A2147      -9.330  36.050   3.815  0.64 33.00      A    C  0.072
HETATM 2090  C3  RII A2147     -10.315  34.040   5.295  0.64 36.27      A    C  0.075
HETATM 2091  C4  RII A2147     -11.571  36.397   8.230  0.64 44.48      A    C  0.083
HETATM 2092  C5  RII A2147     -13.321  35.136   8.483  0.64 43.59      A    C  0.082
HETATM 2093  C6  RII A2147     -12.846  34.965   7.268  0.64 40.05      A    C  0.079
HETATM 2094  C7  RII A2147     -10.493  38.918   6.327  0.64 41.67      A    C  0.081
HETATM 2095  C8  RII A2147     -11.559  40.083   4.870  0.64 37.09      A    C  0.076
HETATM 2096  C9  RII A2147     -11.541  38.797   4.475  0.64 33.42      A    C  0.072
HETATM 2097  N1  RII A2147     -11.731  35.763   7.089  0.64 37.25      A    N  0.076
HETATM 2098  N2  RII A2147     -12.515  36.048   9.095  0.64 42.33      A    N  0.081
HETATM 2099  N3  RII A2147     -10.858  38.057   5.416  0.64 34.53      A    N  0.073
HETATM 2100  N4  RII A2147     -10.909  40.143   6.013  0.64 37.94      A    N  0.077
HETATM 2101  O1  RII A2147     -12.863  35.496   3.469  0.64 37.02      A    O  0.076
HETATM 2102  O2  RII A2147      -8.627  36.139   2.928  0.64 40.67      A    O  0.080
HETATM 2103  O3  RII A2147     -10.225  32.921   5.273  0.64 33.77      A    O  0.073
HETATM 2104 RE1  RII A2147     -10.497  35.923   5.316  0.64 32.45      A   Re  0.071
HETATM 2105  H21 RII A2147     -12.601  36.343   9.898  0.64 50.80      A    H  0.089
HETATM 2106  H41 RII A2147     -10.893  37.009   8.404  0.64 53.38      A    H  0.091
HETATM 2107  H42 RII A2147     -10.776  40.852   6.481  0.64 45.53      A    H  0.084
HETATM 2108  H51 RII A2147     -14.066  34.720   8.849  0.64 52.30      A    H  0.090
HETATM 2109  H61 RII A2147     -13.205  34.394   6.628  0.64 48.05      A    H  0.086
HETATM 2110  H71 RII A2147     -10.009  38.706   7.092  0.64 50.00      A    H  0.088
HETATM 2111  H81 RII A2147     -11.956  40.793   4.422  0.64 44.50      A    H  0.083
HETATM 2112  H91 RII A2147     -11.925  38.462   3.698  0.64 40.10      A    H  0.079
HETATM 2113 RE    RE A2149      11.289  25.380  22.398  0.21 42.25          Re  0.081
HETATM 2114  C1  RI3 A2153      -7.876   9.047  13.120  0.75 53.96      A    C  0.092
HETATM 2115  C2  RI3 A2153      -8.357  11.636  13.022  0.75 37.13      A    C  0.076
HETATM 2116  C3  RI3 A2153      -6.053  10.775  13.841  0.75 30.58      A    C  0.069
HETATM 2117  C7  RI3 A2153     -10.579  11.089  15.760  0.75 55.48      A    C  0.093
HETATM 2118  C8  RI3 A2153     -11.865   9.397  15.461  0.75 59.94      A    C  0.097
HETATM 2119  C9  RI3 A2153     -10.675   9.151  14.884  0.75 54.30      A    C  0.092
HETATM 2120  N3  RI3 A2153      -9.851  10.235  15.086  0.75 46.32      A    N  0.085
HETATM 2121  N4  RI3 A2153     -11.792  10.594  15.998  0.75 67.70      A    N  0.103
HETATM 2122  O1  RI3 A2153      -7.895   8.191  12.333  0.75 44.17      A    O  0.083
HETATM 2123  O2  RI3 A2153      -8.685  12.360  12.223  0.75 32.51      A    O  0.071
HETATM 2124  O3  RI3 A2153      -5.018  11.011  13.478  0.75 39.83      A    O  0.079
HETATM 2125  O4  RI3 A2153      -7.306   9.073  16.007  0.75 37.58      A    O  0.076
HETATM 2126 RE1  RI3 A2153      -7.818  10.426  14.383  0.75 39.14      A   Re  0.078
HETATM 2127  H42 RI3 A2153     -12.420  10.993  16.428  0.75 81.24      A    H  0.112
HETATM 2128  H71 RI3 A2153     -10.287  11.925  16.044  0.75 66.57      A    H  0.102
HETATM 2129  H81 RI3 A2153     -12.599   8.828  15.477  0.75 71.92      A    H  0.106
HETATM 2130  H91 RI3 A2153     -10.443   8.374  14.431  0.75 65.15      A    H  0.101
HETATM 2131  C1  RII A2154       1.289  23.161  36.318  0.68 57.86      A    C  0.095
HETATM 2132  C2  RII A2154      -1.269  23.643  36.441  0.68 67.96      A    C  0.103
HETATM 2133  C3  RII A2154      -0.355  22.438  34.324  0.68 45.99      A    C  0.085
HETATM 2134  C4  RII A2154       1.782  25.533  32.927  0.68 28.89      A    C  0.067
HETATM 2135  C5  RII A2154       3.596  24.406  32.612  0.68 33.00      A    C  0.072
HETATM 2136  C6  RII A2154       2.883  23.805  33.546  0.68 34.03      A    C  0.073
HETATM 2137  C7  RII A2154       0.691  27.160  35.470  0.68 48.12      A    C  0.087
HETATM 2138  C8  RII A2154       0.883  27.556  37.569  0.68 55.13      A    C  0.093
HETATM 2139  C9  RII A2154       0.633  26.247  37.384  0.68 58.59      A    C  0.096
HETATM 2140  N1  RII A2154       1.710  24.515  33.769  0.68 35.45      A    N  0.074
HETATM 2141  N2  RII A2154       2.896  25.505  32.216  0.68 32.15      A    N  0.071
HETATM 2142  N3  RII A2154       0.510  25.997  36.037  0.68 27.04      A    N  0.065
HETATM 2143  N4  RII A2154       0.917  28.108  36.377  0.68 44.78      A    N  0.084
```

```
HETATM 2144  O1  RII A2154       2.026  22.621  37.038  0.68 41.82      A    O  0.081
HETATM 2145  O2  RII A2154      -2.084  23.380  37.188  0.68 47.43      A    O  0.086
HETATM 2146  O3  RII A2154      -0.676  21.483  33.819  0.68 45.00      A    O  0.084
HETATM 2147 RE1  RII A2154       0.100  24.059  35.176  0.68 44.73      A   Re  0.083
HETATM 2148  H21 RII A2154       3.134  26.072  31.617  0.68 38.57      A    H  0.077
HETATM 2149  H41 RII A2154       1.136  26.191  32.837  0.68 34.66      A    H  0.073
HETATM 2150  H42 RII A2154       1.061  28.938  36.209  0.68 53.73      A    H  0.091
HETATM 2151  H51 RII A2154       4.413  24.134  32.282  0.68 39.59      A    H  0.079
HETATM 2152  H61 RII A2154       3.132  23.026  33.988  0.68 40.83      A    H  0.080
HETATM 2153  H71 RII A2154       0.667  27.320  34.557  0.68 57.73      A    H  0.095
HETATM 2154  H81 RII A2154       1.011  27.986  38.381  0.68 66.15      A    H  0.101
HETATM 2155  H91 RII A2154       0.554  25.611  38.056  0.68 70.30      A    H  0.105
HETATM 2156  C   ACT A2155     -19.051  21.087   8.236  1.00 53.55           C  0.091
HETATM 2157  O   ACT A2155     -19.634  20.430   7.365  1.00 62.48           O  0.099
HETATM 2158  CH3 ACT A2155     -18.406  22.420   7.855  1.00 52.60           C  0.091
HETATM 2159  OXT ACT A2155     -18.948  20.724   9.390  1.00 59.16           O  0.096
HETATM 2160  H1  ACT A2155     -19.111  23.017   7.279  1.00 63.12           H  0.099
HETATM 2161  H2  ACT A2155     -18.128  22.960   8.759  1.00 63.12           H  0.099
HETATM 2162  H3  ACT A2155     -17.514  22.237   7.258  1.00 63.12           H  0.099
HETATM 2163  O   HOH B   2       7.458  19.363   6.922  1.00 39.52           O  0.078
HETATM 2164  O   HOH B   3     -13.725  37.837   1.581  1.00 33.85           O  0.073
HETATM 2165  O   HOH B   4       8.129  13.580   4.309  1.00 39.42           O  0.078
HETATM 2166  O   HOH B   5      17.451  19.755  17.714  1.00 32.54           O  0.071
HETATM 2167  O   HOH B   6      -7.383  38.396   5.347  1.00 37.12           O  0.076
HETATM 2168  O   HOH B   7       4.418  14.946  36.206  1.00 47.60           O  0.086
HETATM 2169 RE    RE C   1       0.259  27.740   3.017  0.27 84.54          Re  0.115
HETATM 2170 RE    RE C   2     -10.650  10.650   9.285  0.19 58.80          Re  0.096
HETATM 2171 RE    RE C   3     -15.977  16.069   8.194  0.29 49.60          Re  0.088
HETATM 2172 RE    RE C   4     -11.200  36.662  11.705  0.20 63.06          Re  0.099
HETATM 2173 RE    RE C   5      15.276  21.730  33.542  0.20 43.19          Re  0.082
END
```
