# Supplementary material for: Time-series analysis of rhenium(I) organometallic covalent binding to a model protein for drug development
Source: IUCrJ. 2024 Apr 19;11(Pt 3):359–73. doi: 10.1107/S2052252524002598 (PMC11067751; doi:10.1107/S2052252524002598)
Supplement: Supplementary file 3 [file m-11-00359-sup3.zip › Week 9 - P1P16_2/P1P16_2_refine_55.pdf]

```
REMARK   3
REMARK   3 REFINEMENT.
REMARK   3   PROGRAM     : PHENIX (1.20.1_4487: ???)
REMARK   3   AUTHORS     : Adams,Afonine,Bunkoczi,Burnley,Chen,Dar,Davis,
REMARK   3               : Draizen,Echols,Gildea,Gros,Grosse-Kunstleve,Headd,
REMARK   3               : Hintze,Hung,Ioerger,Liebschner,McCoy,McKee,Moriarty,
REMARK   3               : Oeffner,Poon,Read,Richardson,Richardson,Sacchettini,
REMARK   3               : Sauter,Sobolev,Storoni,Terwilliger,Williams,Zwart
REMARK   3
REMARK   3  X-RAY DATA.
REMARK   3
REMARK   3  REFINEMENT TARGET : ML
REMARK   3
REMARK   3  DATA USED IN REFINEMENT.
REMARK   3   RESOLUTION RANGE HIGH (ANGSTROMS) : 1.23
REMARK   3   RESOLUTION RANGE LOW  (ANGSTROMS) : 40.54
REMARK   3   MIN(FOBS/SIGMA_FOBS)              : 1.36
REMARK   3   COMPLETENESS FOR RANGE        (%) : 97.07
REMARK   3   NUMBER OF REFLECTIONS             : 66601
REMARK   3   NUMBER OF REFLECTIONS (NON-ANOMALOUS) : 35363
REMARK   3
REMARK   3  FIT TO DATA USED IN REFINEMENT.
REMARK   3   R VALUE     (WORKING + TEST SET) : 0.1551
REMARK   3   R VALUE            (WORKING SET) : 0.1536
REMARK   3   FREE R VALUE                     : 0.1822
REMARK   3   FREE R VALUE TEST SET SIZE   (%) : 5.09
REMARK   3   FREE R VALUE TEST SET COUNT      : 3392
REMARK   3
REMARK   3  FIT TO DATA USED IN REFINEMENT (IN BINS).
REMARK   3   BIN  RESOLUTION RANGE  COMPL.    NWORK NFREE   RWORK  RFREE  CCWORK CCFREE
REMARK   3     1   40.54 -    3.55    1.00     2714   142  0.1599 0.2048   0.926  0.864
REMARK   3     2    3.55 -    2.82    1.00     2732   141  0.1597 0.1759   0.938  0.908
REMARK   3     3    2.82 -    2.46    1.00     2701   153  0.1558 0.1754   0.935  0.911
REMARK   3     4    2.46 -    2.24    1.00     2702   149  0.1437 0.1904   0.945  0.907
REMARK   3     5    2.23 -    2.07    1.00     2725   125  0.1409 0.1420   0.947  0.956
REMARK   3     6    2.07 -    1.95    1.00     2710   159  0.1415 0.1704   0.949  0.921
REMARK   3     7    1.95 -    1.85    1.00     2697   150  0.1354 0.1556   0.954  0.934
REMARK   3     8    1.85 -    1.77    1.00     2720   142  0.1386 0.1673   0.955  0.934
REMARK   3     9    1.77 -    1.71    1.00     2711   176  0.1381 0.1799   0.951  0.885
REMARK   3    10    1.71 -    1.65    1.00     2689   137  0.1301 0.1411   0.958  0.948
REMARK   3    11    1.65 -    1.60    1.00     2675   164  0.1285 0.1458   0.957  0.935
REMARK   3    12    1.60 -    1.55    1.00     2714   147  0.1322 0.1582   0.954  0.950
REMARK   3    13    1.55 -    1.51    0.99     2714   124  0.1380 0.1632   0.950  0.942
REMARK   3    14    1.51 -    1.47    0.99     2681   172  0.1286 0.1700   0.954  0.930
REMARK   3    15    1.47 -    1.44    1.00     2691   160  0.1412 0.1668   0.949  0.939
REMARK   3    16    1.44 -    1.41    0.99     2696   131  0.1481 0.2179   0.949  0.911
REMARK   3    17    1.41 -    1.38    1.00     2704   165  0.1562 0.1791   0.925  0.900
REMARK   3    18    1.38 -    1.35    0.98     2638   152  0.1752 0.2281   0.907  0.828
REMARK   3    19    1.35 -    1.33    1.00     2713   133  0.1834 0.2130   0.904  0.880
REMARK   3    20    1.33 -    1.31    0.97     2690   120  0.1966 0.2518   0.882  0.823
REMARK   3    21    1.31 -    1.29    0.96     2628   131  0.2248 0.2919   0.828  0.797
REMARK   3    22    1.29 -    1.27    0.89     2404   119  0.2488 0.2674   0.808  0.774
REMARK   3    23    1.27 -    1.25    0.80     2180   102  0.2919 0.3556   0.716  0.785
REMARK   3    24    1.25 -    1.23    0.73     1980    98  0.3040 0.3004   0.757  0.801
REMARK   3
REMARK   3  BULK SOLVENT MODELLING.
REMARK   3   METHOD USED        : FLAT BULK SOLVENT MODEL
REMARK   3   SOLVENT RADIUS     : 1.10
REMARK   3   SHRINKAGE RADIUS   : 0.90
REMARK   3   GRID STEP FACTOR   : 4.00
REMARK   3
REMARK   3  ERROR ESTIMATES.
REMARK   3   COORDINATE ERROR (MAXIMUM-LIKELIHOOD BASED)     : 0.14
REMARK   3   PHASE ERROR (DEGREES, MAXIMUM-LIKELIHOOD BASED) : 18.46
REMARK   3
REMARK   3  STRUCTURE FACTORS CALCULATION ALGORITHM : FFT
REMARK   3  B VALUES.
REMARK   3   FROM WILSON PLOT           (A**2) : 15.16
REMARK   3
REMARK   3  GEOMETRY RESTRAINTS LIBRARY: GEOSTD + MONOMER LIBRARY + CDL V1.2
REMARK   3  DEVIATIONS FROM IDEAL VALUES - RMSD. RMSZ FOR BONDS AND ANGLES.
REMARK   3    BOND      :  0.012   0.108   1135  Z= 0.792
REMARK   3    ANGLE     :  1.247   7.034   1553  Z= 0.732
REMARK   3    CHIRALITY :  0.087   0.241    151
REMARK   3    PLANARITY :  0.015   0.094    202
REMARK   3    DIHEDRAL  : 14.609  81.861    402
REMARK   3    MIN NONBONDED DISTANCE : 2.158
REMARK   3
```

```
REMARK   3  MOLPROBITY STATISTICS.
REMARK   3    ALL-ATOM CLASHSCORE : 3.74
REMARK   3    RAMACHANDRAN PLOT:
REMARK   3      OUTLIERS :  0.00 %
REMARK   3      ALLOWED  :  2.36 %
REMARK   3      FAVORED  : 97.64 %
REMARK   3    ROTAMER OUTLIERS :  0.00 %
REMARK   3    CBETA DEVIATIONS :  0.00 %
REMARK   3    PEPTIDE PLANE:
REMARK   3      CIS-PROLINE     : 0.00 %
REMARK   3      CIS-GENERAL     : 0.00 %
REMARK   3      TWISTED PROLINE : 0.00 %
REMARK   3      TWISTED GENERAL : 0.00 %
REMARK   3
REMARK   3  RAMA-Z (RAMACHANDRAN PLOT Z-SCORE):
REMARK   3  INTERPRETATION: BAD |RAMA-Z| > 3; SUSPICIOUS 2 < |RAMA-Z| < 3; GOOD |RAMA-Z| < 2.
REMARK   3  SCORES FOR WHOLE/HELIX/SHEET/LOOP ARE SCALED INDEPENDENTLY;
REMARK   3  THEREFORE, THE VALUES ARE NOT RELATED IN A SIMPLE MANNER.
REMARK   3    WHOLE:  0.44 (0.67), RESIDUES: 143
REMARK   3    HELIX: -0.08 (0.74), RESIDUES: 45
REMARK   3    SHEET: -0.98 (0.98), RESIDUES: 14
REMARK   3    LOOP :  0.96 (0.68), RESIDUES: 84
REMARK   3
REMARK   3                   min    max   mean <Bi,j>   iso aniso
REMARK   3     Overall:   11.19 119.27  20.90   1.70    15  1217
REMARK   3     Protein:   11.19  61.01  19.78   1.60     0  1053
REMARK   3     Water:     14.44  44.79  29.46    N/A     2   106
REMARK   3     Other:     14.95 119.27  24.52    N/A    13    58
REMARK   3     Chain  A:  11.19  72.86  19.91    N/A     9  1107
REMARK   3     Chain  C:  24.01  36.72  27.88    N/A     0     4
REMARK   3     Chain  B:  24.02  24.02  24.02    N/A     1     0
REMARK   3     Chain  S:  14.44  44.79  29.46    N/A     2   106
REMARK   3     Chain  D:  32.51 119.27  70.29    N/A     3     0
REMARK   3     Histogram:
REMARK   3         Values      Number of atoms
REMARK   3      11.19 - 22.00       875
REMARK   3      22.00 - 32.81       253
REMARK   3      32.81 - 43.61        86
REMARK   3      43.61 - 54.42        11
REMARK   3      54.42 - 65.23         5
REMARK   3      65.23 - 76.04         1
REMARK   3      76.04 - 86.84         0
REMARK   3      86.84 - 97.65         0
REMARK   3      97.65 - 108.46        0
REMARK   3     108.46 - 119.27        1
REMARK   3
REMARK   3
LINK         NE2 HIS A  15                RE1  RI3 A2145
LINK         OD2 ASP A  18                RE2  RRE A2147
LINK         OD2 ASP A 101                RE1  RII A2144
LINK         OD2 ASP A 119                RE1  RII A2143
SSBOND   1 CYS A    6    CYS A  127
SSBOND   2 CYS A   30    CYS A  115
SSBOND   3 CYS A   64    CYS A   80
SSBOND   4 CYS A   76    CYS A   94
CRYST1   81.080   81.080   37.089  90.00  90.00  90.00 P 43 21 2
SCALE1      0.012333  0.000000  0.000000        0.00000
SCALE2      0.000000  0.012333  0.000000        0.00000
SCALE3      0.000000  0.000000  0.026962        0.00000
ATOM      1  N   LYS A   1       4.806  11.775   9.845  1.00 21.10           N  0.026
ANISOU    1  N   LYS A   1     2687   2542   2787    503   -628   -775       N
ATOM      2  CA  LYS A   1       3.850  12.143   8.766  1.00 21.47           C  0.026
ANISOU    2  CA  LYS A   1     2643   2745   2768    383   -808   -713       C
ATOM      3  C   LYS A   1       3.836  13.628   8.571  1.00 20.24           C  0.026
ANISOU    3  C   LYS A   1     2633   2789   2267    447   -959   -837       C
ATOM      4  O   LYS A   1       3.824  14.352   9.555  1.00 20.10           O  0.026
ANISOU    4  O   LYS A   1     2669   2717   2253    441   -919   -770       O
ATOM      5  CB  LYS A   1       2.435  11.685   9.110  1.00 22.85           C  0.027
ANISOU    5  CB  LYS A   1     2715   2722   3246    289   -784   -345       C
ATOM      6  CG  LYS A   1       1.406  12.093   8.080  1.00 23.74           C  0.028
ANISOU    6  CG  LYS A   1     2715   2749   3557    269   -944   -403       C
ATOM      7  CD  LYS A   1       0.045  11.510   8.445  1.00 26.52           C  0.029
ANISOU    7  CD  LYS A   1     2848   2948   4279    267  -1066   -478       C
ATOM      8  CE  LYS A   1      -1.066  12.029   7.547  1.00 30.01           C  0.031
ANISOU    8  CE  LYS A   1     3058   3266   5077    282  -1027   -313       C
ATOM      9  NZ  LYS A   1      -2.261  11.164   7.655  1.00 32.92           N  0.033
ANISOU    9  NZ  LYS A   1     3359   3547   5601    193  -1005   -365       N
ATOM     10  H1  LYS A   1       4.612  10.960  10.148  1.00 25.30           H  0.029
```

```
ATOM     11  H2  LYS A   1       5.635  11.781   9.522  1.00 25.30           H  0.029
ATOM     12  H3  LYS A   1       4.742  12.362  10.511  1.00 25.30           H  0.029
ATOM     13  HA  LYS A   1       4.124  11.706   7.945  1.00 25.75           H  0.029
ATOM     14  HB2 LYS A   1       2.425  10.717   9.172  1.00 27.41           H  0.030
ATOM     15  HB3 LYS A   1       2.177  12.074   9.959  1.00 27.41           H  0.030
ATOM     16  HG2 LYS A   1       1.334  13.060   8.056  1.00 28.48           H  0.030
ATOM     17  HG3 LYS A   1       1.665  11.758   7.208  1.00 28.48           H  0.030
ATOM     18  HD2 LYS A   1       0.079  10.544   8.356  1.00 31.81           H  0.032
ATOM     19  HD3 LYS A   1      -0.169  11.751   9.360  1.00 31.81           H  0.032
ATOM     20  HE2 LYS A   1      -1.309  12.928   7.816  1.00 36.00           H  0.034
ATOM     21  HE3 LYS A   1      -0.765  12.029   6.624  1.00 36.00           H  0.034
ATOM     22  HZ1 LYS A   1      -2.918  11.488   7.150  1.00 39.49           H  0.036
ATOM     23  HZ2 LYS A   1      -2.066  10.340   7.381  1.00 39.49           H  0.036
ATOM     24  HZ3 LYS A   1      -2.537  11.129   8.500  1.00 39.49           H  0.036
ATOM     25  N   VAL A   2       3.855  14.075   7.321  1.00 19.45           N  0.025
ANISOU   25  N   VAL A   2     2531   2712   2146    483   -734   -994       N
ATOM     26  CA  VAL A   2       3.727  15.489   6.998  1.00 19.96           C  0.025
ANISOU   26  CA  VAL A   2     2712   2883   1989    480   -771   -719       C
ATOM     27  C   VAL A   2       2.326  15.679   6.467  1.00 20.22           C  0.026
ANISOU   27  C   VAL A   2     2860   2842   1981    508   -835   -792       C
ATOM     28  O   VAL A   2       2.041  15.252   5.341  1.00 21.39           O  0.026
ANISOU   28  O   VAL A   2     3132   3049   1946    464   -796   -869       O
ATOM     29  CB  VAL A   2       4.778  15.939   5.978  1.00 20.59           C  0.026
ANISOU   29  CB  VAL A   2     2778   3155   1891    417   -480   -670       C
ATOM     30  CG1 VAL A   2       4.519  17.405   5.658  1.00 21.67           C  0.027
ANISOU   30  CG1 VAL A   2     2890   3436   1906    443   -324   -345       C
ATOM     31  CG2 VAL A   2       6.189  15.768   6.526  1.00 21.85           C  0.027
ANISOU   31  CG2 VAL A   2     2836   3276   2191    424   -260   -600       C
ATOM     32  H   VAL A   2       3.941  13.568   6.632  1.00 23.33           H  0.027
ATOM     33  HA  VAL A   2       3.844  16.023   7.799  1.00 23.94           H  0.028
ATOM     34  HB  VAL A   2       4.713  15.397   5.176  1.00 24.70           H  0.028
ATOM     35 HG11 VAL A   2       5.298  17.772   5.211  1.00 25.99           H  0.029
ATOM     36 HG12 VAL A   2       3.744  17.469   5.078  1.00 25.99           H  0.029
ATOM     37 HG13 VAL A   2       4.355  17.884   6.485  1.00 25.99           H  0.029
ATOM     38 HG21 VAL A   2       6.825  16.091   5.868  1.00 26.21           H  0.029
ATOM     39 HG22 VAL A   2       6.274  16.278   7.346  1.00 26.21           H  0.029
ATOM     40 HG23 VAL A   2       6.347  14.827   6.703  1.00 26.21           H  0.029
ATOM     41  N   PHE A   3       1.479  16.332   7.271  1.00 18.22           N  0.024
ANISOU   41  N   PHE A   3     2653   2629   1642    664   -758   -563       N
ATOM     42  CA  PHE A   3       0.101  16.568   6.892  1.00 17.93           C  0.024
ANISOU   42  CA  PHE A   3     2580   2601   1631    565   -851   -798       C
ATOM     43  C   PHE A   3       0.027  17.688   5.862  1.00 18.24           C  0.024
ANISOU   43  C   PHE A   3     2565   2788   1576    493   -888   -731       C
ATOM     44  O   PHE A   3       0.820  18.633   5.868  1.00 19.82           O  0.025
ANISOU   44  O   PHE A   3     2595   2911   2026    601   -816   -679       O
ATOM     45  CB  PHE A   3      -0.737  17.019   8.090  1.00 18.95           C  0.025
ANISOU   45  CB  PHE A   3     2596   2558   2049    529   -738   -613       C
ATOM     46  CG  PHE A   3      -1.239  15.915   8.963  1.00 19.55           C  0.025
ANISOU   46  CG  PHE A   3     2549   2591   2289    283   -741   -580       C
ATOM     47  CD1 PHE A   3      -0.450  15.369   9.942  1.00 20.10           C  0.026
ANISOU   47  CD1 PHE A   3     2630   2672   2337    166   -717   -404       C
ATOM     48  CD2 PHE A   3      -2.542  15.483   8.855  1.00 20.56           C  0.026
ANISOU   48  CD2 PHE A   3     2627   2643   2543    220   -604   -546       C
ATOM     49  CE1 PHE A   3      -0.919  14.371  10.762  1.00 20.21           C  0.026
ANISOU   49  CE1 PHE A   3     2715   2658   2306    287   -610   -242       C
ATOM     50  CE2 PHE A   3      -3.030  14.488   9.690  1.00 21.23           C  0.026
ANISOU   50  CE2 PHE A   3     2717   2687   2664    210   -581   -289       C
ATOM     51  CZ  PHE A   3      -2.200  13.927  10.662  1.00 20.84           C  0.026
ANISOU   51  CZ  PHE A   3     2738   2615   2563    305   -606   -317       C
ATOM     52  H   PHE A   3       1.687  16.648   8.043  1.00 21.85           H  0.027
ATOM     53  HA  PHE A   3      -0.259  15.741   6.536  1.00 21.50           H  0.026
ATOM     54  HB2 PHE A   3      -0.193  17.601   8.643  1.00 22.73           H  0.027
ATOM     55  HB3 PHE A   3      -1.510  17.502   7.759  1.00 22.73           H  0.027
ATOM     56  HD1 PHE A   3       0.419  15.680  10.054  1.00 24.11           H  0.028
ATOM     57  HD2 PHE A   3      -3.101  15.863   8.216  1.00 24.66           H  0.028
ATOM     58  HE1 PHE A   3      -0.351  13.993  11.395  1.00 24.24           H  0.028
ATOM     59  HE2 PHE A   3      -3.909  14.195   9.603  1.00 25.47           H  0.029
ATOM     60  HZ  PHE A   3      -2.517  13.263  11.229  1.00 24.99           H  0.028
ATOM     61  N   GLY A   4      -0.987  17.602   4.998  1.00 18.95           N  0.025
ANISOU   61  N   GLY A   4     2557   2953   1691    515   -852   -403       N
ATOM     62  CA  GLY A   4      -1.442  18.758   4.269  1.00 19.55           C  0.025
ANISOU   62  CA  GLY A   4     2671   2982   1776    407   -869   -146       C
ATOM     63  C   GLY A   4      -2.313  19.615   5.147  1.00 18.44           C  0.024
ANISOU   63  C   GLY A   4     2594   2853   1560    336   -887   -155       C
ATOM     64  O   GLY A   4      -2.839  19.166   6.160  1.00 19.52           O  0.025
ANISOU   64  O   GLY A   4     2499   2877   2042    320   -773   -190       O
ATOM     65  H   GLY A   4      -1.422  16.881   4.825  1.00 22.73           H  0.027
ATOM     66  HA2 GLY A   4      -0.681  19.281   3.972  1.00 23.45           H  0.028
```

```
ATOM     67  HA3 GLY A   4      -1.954  18.478   3.494  1.00 23.45           H  0.028
ATOM     68  N   ARG A   5      -2.445  20.874   4.740  1.00 18.62           N  0.025
ANISOU   68  N   ARG A   5     2740   2843   1492    127   -572   -267       N
ATOM     69  CA  ARG A   5      -3.169  21.851   5.540  1.00 19.32           C  0.025
ANISOU   69  CA  ARG A   5     2630   2766   1944     -6   -392   -361       C
ATOM     70  C   ARG A   5      -4.616  21.414   5.783  1.00 19.47           C  0.025
ANISOU   70  C   ARG A   5     2617   2655   2126      9   -528   -339       C
ATOM     71  O   ARG A   5      -5.085  21.360   6.932  1.00 18.57           O  0.025
ANISOU   71  O   ARG A   5     2618   2530   1908     -0   -819   -240       O
ATOM     72  CB  ARG A   5      -3.067  23.205   4.836  1.00 20.95           C  0.026
ANISOU   72  CB  ARG A   5     2716   2933   2311    -74   -176   -335       C
ATOM     73  CG  ARG A   5      -3.990  24.249   5.350  1.00 21.49           C  0.026
ANISOU   73  CG  ARG A   5     2824   2964   2378   -208    191   -510       C
ATOM     74  CD  ARG A   5      -3.782  25.525   4.552  1.00 21.09           C  0.026
ANISOU   74  CD  ARG A   5     2922   2950   2141   -302    245   -701       C
ATOM     75  NE  ARG A   5      -4.283  25.435   3.186  1.00 22.24           N  0.027
ANISOU   75  NE  ARG A   5     3101   3180   2170   -264    146   -703       N
ATOM     76  CZ  ARG A   5      -5.548  25.612   2.827  1.00 22.48           C  0.027
ANISOU   76  CZ  ARG A   5     3153   3431   1958   -334   -239   -335       C
ATOM     77  NH1 ARG A   5      -6.510  25.798   3.720  1.00 22.06           N  0.027
ANISOU   77  NH1 ARG A   5     3018   3305   2060   -281   -355   -152       N
ATOM     78  NH2 ARG A   5      -5.857  25.586   1.540  1.00 24.39           N  0.028
ANISOU   78  NH2 ARG A   5     3255   3793   2217   -181   -119   -127       N
ATOM     79  H   ARG A   5      -2.125  21.186   4.006  1.00 22.33           H  0.027
ATOM     80  HA  ARG A   5      -2.772  21.938   6.421  1.00 23.17           H  0.027
ATOM     81  HB2 ARG A   5      -2.163  23.539   4.940  1.00 25.13           H  0.029
ATOM     82  HB3 ARG A   5      -3.267  23.077   3.895  1.00 25.13           H  0.029
ATOM     83  HG2 ARG A   5      -4.910  23.959   5.247  1.00 25.78           H  0.029
ATOM     84  HG3 ARG A   5      -3.801  24.427   6.285  1.00 25.78           H  0.029
ATOM     85  HD2 ARG A   5      -4.248  26.252   4.993  1.00 25.30           H  0.029
ATOM     86  HD3 ARG A   5      -2.832  25.717   4.509  1.00 25.30           H  0.029
ATOM     87  HE  ARG A   5      -3.716  25.254   2.565  1.00 26.68           H  0.029
ATOM     88 HH11 ARG A   5      -6.322  25.806   4.559  1.00 26.46           H  0.029
ATOM     89 HH12 ARG A   5      -7.322  25.910   3.460  1.00 26.46           H  0.029
ATOM     90 HH21 ARG A   5      -5.243  25.456   0.952  1.00 29.25           H  0.031
ATOM     91 HH22 ARG A   5      -6.673  25.698   1.291  1.00 29.25           H  0.031
ATOM     92  N   CYS A   6      -5.334  21.064   4.730  1.00 20.38           N  0.026
ANISOU   92  N   CYS A   6     2597   2775   2371     47   -738   -154       N
ATOM     93  CA  CYS A   6      -6.737  20.718   4.940  1.00 19.54           C  0.025
ANISOU   93  CA  CYS A   6     2724   2763   1938     92   -999   -251       C
ATOM     94  C   CYS A   6      -6.893  19.380   5.641  1.00 19.60           C  0.025
ANISOU   94  C   CYS A   6     2763   2640   2045    116   -930   -285       C
ATOM     95  O   CYS A   6      -7.838  19.196   6.415  1.00 19.75           O  0.025
ANISOU   95  O   CYS A   6     2814   2384   2308     -5   -827     36       O
ATOM     96  CB  CYS A   6      -7.497  20.705   3.627  1.00 20.73           C  0.026
ANISOU   96  CB  CYS A   6     2995   2935   1947    128  -1136   -129       C
ATOM     97  SG  CYS A   6      -7.785  22.350   2.937  1.00 21.81           S  0.027
ANISOU   97  SG  CYS A   6     3170   3041   2075     29   -950     -1       S
ATOM     98  H   CYS A   6      -5.051  21.020   3.919  1.00 24.44           H  0.028
ATOM     99  HA  CYS A   6      -7.129  21.405   5.501  1.00 23.44           H  0.028
ATOM    100  HB2 CYS A   6      -6.987  20.196   2.977  1.00 24.87           H  0.028
ATOM    101  HB3 CYS A   6      -8.361  20.288   3.771  1.00 24.87           H  0.028
ATOM    102  N   GLU A   7      -6.001  18.433   5.347  1.00 20.06           N  0.025
ANISOU  102  N   GLU A   7     2806   2658   2158    174   -900   -460       N
ATOM    103  CA  GLU A   7      -6.030  17.137   6.002  1.00 20.88           C  0.026
ANISOU  103  CA  GLU A   7     2732   2608   2594    172  -1136   -543       C
ATOM    104  C   GLU A   7      -5.852  17.319   7.508  1.00 19.72           C  0.025
ANISOU  104  C   GLU A   7     2496   2370   2626    -50  -1004   -680       C
ATOM    105  O   GLU A   7      -6.556  16.687   8.312  1.00 20.28           O  0.026
ANISOU  105  O   GLU A   7     2469   2427   2809     63   -920   -554       O
ATOM    106  CB  GLU A   7      -4.900  16.280   5.420  1.00 24.58           C  0.028
ANISOU  106  CB  GLU A   7     3113   2726   3498    256  -1461   -711       C
ATOM    107  CG  GLU A   7      -4.697  14.987   6.098  1.00 28.35           C  0.030
ANISOU  107  CG  GLU A   7     3503   2919   4351    211  -1735   -680       C
ATOM    108  CD  GLU A   7      -3.455  14.201   5.582  1.00 30.94           C  0.032
ANISOU  108  CD  GLU A   7     3719   3044   4993    149  -1823   -793       C
ATOM    109  OE1 GLU A   7      -2.406  14.783   5.103  1.00 30.80           O  0.032
ANISOU  109  OE1 GLU A   7     3807   3015   4882    181  -2034   -665       O
ATOM    110  OE2 GLU A   7      -3.561  12.961   5.636  1.00 33.32           O  0.033
ANISOU  110  OE2 GLU A   7     3840   3131   5688    147  -1578   -702       O
ATOM    111  H   GLU A   7      -5.370  18.520   4.770  1.00 24.06           H  0.028
ATOM    112  HA  GLU A   7      -6.873  16.685   5.845  1.00 25.04           H  0.028
ATOM    113  HB2 GLU A   7      -5.103  16.093   4.490  1.00 29.48           H  0.031
ATOM    114  HB3 GLU A   7      -4.070  16.778   5.486  1.00 29.48           H  0.031
ATOM    115  HG2 GLU A   7      -4.573  15.147   7.047  1.00 34.01           H  0.033
ATOM    116  HG3 GLU A   7      -5.480  14.432   5.955  1.00 34.01           H  0.033
ATOM    117  N   LEU A   8      -4.901  18.170   7.908  1.00 18.22           N  0.024
ANISOU  117  N   LEU A   8     2456   2166   2302   -102   -771   -602       N
```

```
ATOM    118  CA  LEU A   8      -4.709  18.405   9.332  1.00 18.21           C  0.024
ANISOU  118  CA  LEU A   8     2464   2300   2156    -24   -708   -411       C
ATOM    119  C   LEU A   8      -5.912  19.125   9.926  1.00 18.03           C  0.024
ANISOU  119  C   LEU A   8     2287   2302   2261    -32   -700   -278       C
ATOM    120  O   LEU A   8      -6.321  18.833  11.054  1.00 18.34           O  0.024
ANISOU  120  O   LEU A   8     2267   2414   2287     46   -793    -76       O
ATOM    121  CB  LEU A   8      -3.422  19.199   9.591  1.00 18.01           C  0.024
ANISOU  121  CB  LEU A   8     2420   2363   2061     13   -727   -215       C
ATOM    122  CG  LEU A   8      -3.128  19.469  11.045  1.00 18.09           C  0.024
ANISOU  122  CG  LEU A   8     2376   2379   2118   -275   -681   -200       C
ATOM    123  CD1 LEU A   8      -2.906  18.171  11.823  1.00 18.54           C  0.025
ANISOU  123  CD1 LEU A   8     2333   2416   2294   -387   -721   -294       C
ATOM    124  CD2 LEU A   8      -1.913  20.352  11.233  1.00 19.03           C  0.025
ANISOU  124  CD2 LEU A   8     2473   2566   2191   -275   -567   -512       C
ATOM    125  H   LEU A   8      -4.373  18.607   7.388  1.00 21.85           H  0.027
ATOM    126  HA  LEU A   8      -4.608  17.548   9.775  1.00 21.84           H  0.027
ATOM    127  HB2 LEU A   8      -2.674  18.699   9.229  1.00 21.60           H  0.026
ATOM    128  HB3 LEU A   8      -3.496  20.057   9.144  1.00 21.60           H  0.026
ATOM    129  HG  LEU A   8      -3.904  19.930  11.399  1.00 21.69           H  0.027
ATOM    130 HD11 LEU A   8      -2.589  18.387  12.714  1.00 22.23           H  0.027
ATOM    131 HD12 LEU A   8      -3.746  17.690  11.879  1.00 22.23           H  0.027
ATOM    132 HD13 LEU A   8      -2.247  17.633  11.358  1.00 22.23           H  0.027
ATOM    133 HD21 LEU A   8      -1.780  20.509  12.181  1.00 22.82           H  0.027
ATOM    134 HD22 LEU A   8      -1.137  19.905  10.859  1.00 22.82           H  0.027
ATOM    135 HD23 LEU A   8      -2.061  21.194  10.775  1.00 22.82           H  0.027
ATOM    136  N   ALA A   9      -6.481  20.094   9.206  1.00 17.64           N  0.024
ANISOU  136  N   ALA A   9     2166   2273   2262     -4   -560   -493       N
ATOM    137  CA  ALA A   9      -7.664  20.783   9.723  1.00 17.87           C  0.024
ANISOU  137  CA  ALA A   9     2204   2378   2208     21   -527   -360       C
ATOM    138  C   ALA A   9      -8.773  19.778  10.025  1.00 17.57           C  0.024
ANISOU  138  C   ALA A   9     2231   2414   2032    -42   -710   -219       C
ATOM    139  O   ALA A   9      -9.425  19.818  11.080  1.00 18.54           O  0.025
ANISOU  139  O   ALA A   9     2324   2507   2214   -101   -636    -51       O
ATOM    140  CB  ALA A   9      -8.153  21.834   8.727  1.00 18.80           C  0.025
ANISOU  140  CB  ALA A   9     2424   2547   2174    -68   -525   -130       C
ATOM    141  H   ALA A   9      -6.209  20.365   8.437  1.00 21.15           H  0.026
ATOM    142  HA  ALA A   9      -7.424  21.244  10.542  1.00 21.43           H  0.026
ATOM    143  HB1 ALA A   9      -8.931  22.281   9.096  1.00 22.55           H  0.027
ATOM    144  HB2 ALA A   9      -7.443  22.477   8.574  1.00 22.55           H  0.027
ATOM    145  HB3 ALA A   9      -8.387  21.395   7.894  1.00 22.55           H  0.027
ATOM    146  N   ALA A  10      -9.013  18.864   9.091  1.00 19.25           N  0.025
ANISOU  146  N   ALA A  10     2370   2621   2325   -151   -828   -321       N
ATOM    147  CA  ALA A  10     -10.071  17.880   9.281  1.00 20.66           C  0.026
ANISOU  147  CA  ALA A  10     2484   2591   2774   -315   -867   -278       C
ATOM    148  C   ALA A  10      -9.770  16.965  10.468  1.00 19.96           C  0.025
ANISOU  148  C   ALA A  10     2342   2508   2735   -336  -1038   -199       C
ATOM    149  O   ALA A  10     -10.658  16.638  11.265  1.00 21.32           O  0.026
ANISOU  149  O   ALA A  10     2354   2583   3166   -295  -1080     55       O
ATOM    150  CB  ALA A  10     -10.237  17.025   8.021  1.00 22.18           C  0.027
ANISOU  150  CB  ALA A  10     2756   2522   3148   -285   -881   -358       C
ATOM    151  H   ALA A  10      -8.585  18.792   8.349  1.00 23.09           H  0.027
ATOM    152  HA  ALA A  10     -10.899  18.357   9.449  1.00 24.78           H  0.028
ATOM    153  HB1 ALA A  10     -10.933  16.367   8.175  1.00 26.60           H  0.029
ATOM    154  HB2 ALA A  10     -10.484  17.600   7.280  1.00 26.60           H  0.029
ATOM    155  HB3 ALA A  10      -9.397  16.580   7.828  1.00 26.60           H  0.029
ATOM    156  N   ALA A  11      -8.510  16.548  10.606  1.00 18.85           N  0.025
ANISOU  156  N   ALA A  11     2301   2413   2448   -230   -892   -254       N
ATOM    157  CA  ALA A  11      -8.131  15.680  11.718  1.00 19.38           C  0.025
ANISOU  157  CA  ALA A  11     2345   2422   2596   -164   -881   -360       C
ATOM    158  C   ALA A  11      -8.271  16.425  13.043  1.00 18.79           C  0.025
ANISOU  158  C   ALA A  11     2161   2301   2676   -134   -751   -184       C
ATOM    159  O   ALA A  11      -8.799  15.875  14.020  1.00 19.08           O  0.025
ANISOU  159  O   ALA A  11     2068   2362   2820    -82   -694    158       O
ATOM    160  CB  ALA A  11      -6.713  15.125  11.540  1.00 20.42           C  0.026
ANISOU  160  CB  ALA A  11     2519   2464   2774   -164   -782   -343       C
ATOM    161  H   ALA A  11      -7.864  16.752  10.077  1.00 22.61           H  0.027
ATOM    162  HA  ALA A  11      -8.722  14.911  11.728  1.00 23.24           H  0.027
ATOM    163  HB1 ALA A  11      -6.501  14.552  12.293  1.00 24.49           H  0.028
ATOM    164  HB2 ALA A  11      -6.675  14.615  10.716  1.00 24.49           H  0.028
ATOM    165  HB3 ALA A  11      -6.087  15.865  11.501  1.00 24.49           H  0.028
ATOM    166  N   MET A  12      -7.783  17.675  13.087  1.00 17.04           N  0.024
ANISOU  166  N   MET A  12     2071   2109   2297   -183   -504   -115       N
ATOM    167  CA  MET A  12      -7.922  18.451  14.306  1.00 17.41           C  0.024
ANISOU  167  CA  MET A  12     2078   2224   2312   -168   -379     84       C
ATOM    168  C   MET A  12      -9.396  18.650  14.661  1.00 18.14           C  0.024
ANISOU  168  C   MET A  12     2119   2442   2333   -211   -481   -107       C
ATOM    169  O   MET A  12      -9.758  18.608  15.850  1.00 18.40           O  0.024
ANISOU  169  O   MET A  12     2237   2476   2277   -139    -44    -46       O
```

```
ATOM    170  CB  MET A  12      -7.204  19.770  14.317  1.00 17.10           C  0.024
ANISOU  170  CB  MET A  12     2077   2164   2257    -97   -364    -87       C
ATOM    171  CG  MET A  12      -5.665  19.586  14.280  1.00 16.15           C  0.023
ANISOU  171  CG  MET A  12     2026   2131   1979   -151   -501    -93       C
ATOM    172  SD  MET A  12      -4.938  21.223  14.038  1.00 15.99           S  0.023
ANISOU  172  SD  MET A  12     2317   1995   1762    -10   -224    -39       S
ATOM    173  CE  MET A  12      -3.370  20.983  14.935  1.00 16.38           C  0.023
ANISOU  173  CE  MET A  12     2230   2022   1973     32   -403     58       C
ATOM    174  H   MET A  12      -7.381  18.075  12.441  1.00 20.44           H  0.026
ATOM    175  HA  MET A  12      -7.462  17.936  14.986  1.00 20.88           H  0.026
ATOM    176  HB2 MET A  12      -7.468  20.284  13.538  1.00 20.51           H  0.026
ATOM    177  HB3 MET A  12      -7.434  20.252  15.127  1.00 20.51           H  0.026
ATOM    178  HG2 MET A  12      -5.349  19.213  15.119  1.00 19.37           H  0.025
ATOM    179  HG3 MET A  12      -5.412  19.008  13.544  1.00 19.37           H  0.025
ATOM    180  HE1 MET A  12      -2.844  21.796  14.873  1.00 19.65           H  0.025
ATOM    181  HE2 MET A  12      -3.565  20.785  15.864  1.00 19.65           H  0.025
ATOM    182  HE3 MET A  12      -2.887  20.244  14.534  1.00 19.65           H  0.025
ATOM    183  N   LYS A  13     -10.267  18.810  13.659  1.00 19.15           N  0.025
ANISOU  183  N   LYS A  13     2025   2664   2588   -159   -425     23       N
ATOM    184  CA  LYS A  13     -11.701  18.921  13.923  1.00 19.65           C  0.025
ANISOU  184  CA  LYS A  13     1948   2827   2691   -290   -476    170       C
ATOM    185  C   LYS A  13     -12.279  17.628  14.487  1.00 21.37           C  0.026
ANISOU  185  C   LYS A  13     2181   2895   3042   -375   -592    132       C
ATOM    186  O   LYS A  13     -13.026  17.642  15.474  1.00 21.65           O  0.026
ANISOU  186  O   LYS A  13     2173   2877   3175   -246   -715   -122       O
ATOM    187  CB  LYS A  13     -12.403  19.362  12.643  1.00 22.47           C  0.027
ANISOU  187  CB  LYS A  13     2092   3267   3179   -148   -451    280       C
ATOM    188  CG  LYS A  13     -13.835  19.926  12.894  1.00 27.54           C  0.030
ANISOU  188  CG  LYS A  13     2377   3834   4255     93   -342    496       C
ATOM    189  CD  LYS A  13     -14.673  19.951  11.610  1.00 32.40           C  0.032
ANISOU  189  CD  LYS A  13     2671   4322   5318    361   -145    722       C
ATOM    190  CE  LYS A  13     -16.129  20.332  11.853  1.00 36.18           C  0.034
ANISOU  190  CE  LYS A  13     2934   4691   6121    522    -64    815       C
ATOM    191  NZ  LYS A  13     -16.463  21.660  11.238  1.00 39.17           N  0.036
ANISOU  191  NZ  LYS A  13     3271   4944   6669    520    165    878       N
ATOM    192  H   LYS A  13     -10.055  18.857  12.827  1.00 22.97           H  0.027
ATOM    193  HA  LYS A  13     -11.861  19.598  14.599  1.00 23.56           H  0.028
ATOM    194  HB2 LYS A  13     -11.877  20.059  12.221  1.00 26.95           H  0.030
ATOM    195  HB3 LYS A  13     -12.482  18.599  12.050  1.00 26.95           H  0.030
ATOM    196  HG2 LYS A  13     -14.290  19.367  13.542  1.00 33.04           H  0.033
ATOM    197  HG3 LYS A  13     -13.767  20.834  13.228  1.00 33.04           H  0.033
ATOM    198  HD2 LYS A  13     -14.293  20.601  10.999  1.00 38.87           H  0.035
ATOM    199  HD3 LYS A  13     -14.659  19.069  11.208  1.00 38.87           H  0.035
ATOM    200  HE2 LYS A  13     -16.706  19.660  11.459  1.00 43.40           H  0.038
ATOM    201  HE3 LYS A  13     -16.290  20.389  12.808  1.00 43.40           H  0.038
ATOM    202  HZ1 LYS A  13     -17.313  21.867  11.403  1.00 47.00           H  0.039
ATOM    203  HZ2 LYS A  13     -15.941  22.294  11.579  1.00 47.00           H  0.039
ATOM    204  HZ3 LYS A  13     -16.340  21.627  10.357  1.00 47.00           H  0.039
ATOM    205  N   ARG A  14     -11.885  16.497  13.918  1.00 22.36           N  0.027
ANISOU  205  N   ARG A  14     2391   2885   3220   -455   -712    182       N
ATOM    206  CA  ARG A  14     -12.378  15.218  14.419  1.00 25.08           C  0.029
ANISOU  206  CA  ARG A  14     2668   3213   3648   -575   -672    252       C
ATOM    207  C   ARG A  14     -11.965  15.025  15.872  1.00 24.04           C  0.028
ANISOU  207  C   ARG A  14     2529   2957   3646   -484   -564    318       C
ATOM    208  O   ARG A  14     -12.695  14.395  16.654  1.00 24.88           O  0.028
ANISOU  208  O   ARG A  14     2419   3007   4027   -621   -412    549       O
ATOM    209  CB  ARG A  14     -11.784  14.080  13.584  1.00 29.84           C  0.031
ANISOU  209  CB  ARG A  14     3193   3816   4330   -813   -875     -9       C
ATOM    210  CG  ARG A  14     -12.274  13.984  12.146  1.00 36.48           C  0.034
ANISOU  210  CG  ARG A  14     3742   4503   5616   -979   -518    127       C
ATOM    211  CD  ARG A  14     -12.937  12.654  11.908  1.00 42.62           C  0.037
ANISOU  211  CD  ARG A  14     4324   5076   6793  -1101   -423    -24       C
ATOM    212  NE  ARG A  14     -14.129  12.547  12.735  1.00 47.14           N  0.039
ANISOU  212  NE  ARG A  14     4779   5530   7603  -1207   -452   -165       N
ATOM    213  CZ  ARG A  14     -14.711  11.408  13.080  1.00 49.89           C  0.040
ANISOU  213  CZ  ARG A  14     5113   5844   8001  -1362   -502   -365       C
ATOM    214  NH1 ARG A  14     -14.218  10.234  12.702  1.00 50.90           N  0.041
ANISOU  214  NH1 ARG A  14     5244   5951   8144  -1379   -539   -427       N
ATOM    215  NH2 ARG A  14     -15.820  11.447  13.818  1.00 50.91           N  0.041
ANISOU  215  NH2 ARG A  14     5264   6011   8068  -1365   -545   -433       N
ATOM    216  H   ARG A  14     -11.343  16.441  13.253  1.00 26.82           H  0.029
ATOM    217  HA  ARG A  14     -13.345  15.195  14.347  1.00 30.08           H  0.031
ATOM    218  HB2 ARG A  14     -10.822  14.197  13.552  1.00 35.80           H  0.034
ATOM    219  HB3 ARG A  14     -12.002  13.240  14.018  1.00 35.80           H  0.034
ATOM    220  HG2 ARG A  14     -12.920  14.687  11.975  1.00 43.76           H  0.038
ATOM    221  HG3 ARG A  14     -11.522  14.069  11.540  1.00 43.76           H  0.038
ATOM    222  HD2 ARG A  14     -13.194  12.577  10.976  1.00 51.13           H  0.041
ATOM    223  HD3 ARG A  14     -12.327  11.937  12.144  1.00 51.13           H  0.041
```

```
ATOM    224  HE  ARG A  14     -14.483  13.277  13.021  1.00 56.56           H  0.043
ATOM    225 HH11 ARG A  14     -13.506  10.202  12.221  1.00 61.07           H  0.044
ATOM    226 HH12 ARG A  14     -14.612   9.507  12.937  1.00 61.07           H  0.044
ATOM    227 HH21 ARG A  14     -16.147  12.204  14.063  1.00 61.08           H  0.044
ATOM    228 HH22 ARG A  14     -16.208  10.715  14.050  1.00 61.08           H  0.044
ATOM    229  N   HIS A  15     -10.797  15.565  16.211  1.00 22.15           N  0.027
ANISOU  229  N   HIS A  15     2377   2673   3364   -312   -487    411       N
ATOM    230  CA  HIS A  15     -10.227  15.409  17.569  1.00 21.68           C  0.027
ANISOU  230  CA  HIS A  15     2300   2706   3232   -237   -413    419       C
ATOM    231  C   HIS A  15     -10.722  16.510  18.508  1.00 20.66           C  0.026
ANISOU  231  C   HIS A  15     2113   2831   2907   -278   -350    334       C
ATOM    232  O   HIS A  15     -10.150  16.597  19.578  1.00 22.13           O  0.027
ANISOU  232  O   HIS A  15     2182   2854   3373   -209   -417    470       O
ATOM    233  CB  HIS A  15      -8.703  15.290  17.444  1.00 23.14           C  0.027
ANISOU  233  CB  HIS A  15     2479   2792   3522   -235   -635    298       C
ATOM    234  CG  HIS A  15      -8.250  13.963  16.950  1.00 26.20           C  0.029
ANISOU  234  CG  HIS A  15     2970   2892   4094    -95   -317    669       C
ATOM    235  ND1 HIS A  15      -8.154  12.897  17.784  1.00 29.94           N  0.031
ANISOU  235  ND1 HIS A  15     3548   3101   4727     51     22    566       N
ATOM    236  CD2 HIS A  15      -7.865  13.518  15.739  1.00 27.93           C  0.030
ANISOU  236  CD2 HIS A  15     3314   2877   4421    -66   -328    425       C
ATOM    237  CE1 HIS A  15      -7.732  11.851  17.129  1.00 29.54           C  0.031
ANISOU  237  CE1 HIS A  15     3559   2873   4791      9    -98    373       C
ATOM    238  NE2 HIS A  15      -7.544  12.204  15.880  1.00 27.87           N  0.030
ANISOU  238  NE2 HIS A  15     3398   2745   4448   -201   -493    140       N
ATOM    239  H   HIS A  15     -10.195  16.119  15.574  1.00 26.56           H  0.029
ATOM    240  HA  HIS A  15     -10.590  14.462  17.964  1.00 26.01           H  0.029
ATOM    241  HB2 HIS A  15      -8.343  16.061  16.764  1.00 27.76           H  0.030
ATOM    242  HB3 HIS A  15      -8.252  15.471  18.418  1.00 27.76           H  0.030
ATOM    243  HD1 HIS A  15      -8.408  12.900  18.756  1.00 35.92           H  0.034
ATOM    244  HD2 HIS A  15      -7.776  14.088  14.829  1.00 33.50           H  0.033
ATOM    245  HE1 HIS A  15      -7.590  10.865  17.544  1.00 35.43           H  0.034
ATOM    246  N   GLY A  16     -11.674  17.371  18.112  1.00 20.31           N  0.026
ANISOU  246  N   GLY A  16     2016   2910   2792   -269   -173    400       N
ATOM    247  CA  GLY A  16     -12.313  18.272  19.027  1.00 19.91           C  0.025
ANISOU  247  CA  GLY A  16     2112   2891   2560   -309     37    303       C
ATOM    248  C   GLY A  16     -11.575  19.556  19.294  1.00 19.43           C  0.025
ANISOU  248  C   GLY A  16     2096   2927   2358   -289    -62    274       C
ATOM    249  O   GLY A  16     -11.858  20.212  20.302  1.00 20.34           O  0.026
ANISOU  249  O   GLY A  16     2135   3033   2561   -313     13    177       O
ATOM    250  H   GLY A  16     -11.954  17.426  17.301  1.00 24.36           H  0.028
ATOM    251  HA2 GLY A  16     -13.186  18.504  18.673  1.00 23.87           H  0.028
ATOM    252  HA3 GLY A  16     -12.428  17.818  19.877  1.00 23.87           H  0.028
ATOM    253  N   LEU A  17     -10.633  19.950  18.424  1.00 18.88           N  0.025
ANISOU  253  N   LEU A  17     2146   2864   2163   -199      0    182       N
ATOM    254  CA  LEU A  17      -9.926  21.211  18.659  1.00 18.56           C  0.025
ANISOU  254  CA  LEU A  17     2115   2720   2216   -112     -3    169       C
ATOM    255  C   LEU A  17     -10.698  22.432  18.174  1.00 19.08           C  0.025
ANISOU  255  C   LEU A  17     2029   2784   2435    -56    -80    128       C
ATOM    256  O   LEU A  17     -10.361  23.527  18.605  1.00 19.32           O  0.025
ANISOU  256  O   LEU A  17     2007   2577   2756    -62     32      0       O
ATOM    257  CB  LEU A  17      -8.555  21.194  17.997  1.00 18.16           C  0.024
ANISOU  257  CB  LEU A  17     2252   2515   2134    -71    -21     80       C
ATOM    258  CG  LEU A  17      -7.516  20.448  18.836  1.00 17.62           C  0.024
ANISOU  258  CG  LEU A  17     2290   2395   2012    -38   -102     -2       C
ATOM    259  CD1 LEU A  17      -6.218  20.530  18.062  1.00 17.71           C  0.024
ANISOU  259  CD1 LEU A  17     2220   2354   2157    118    -48   -243       C
ATOM    260  CD2 LEU A  17      -7.295  20.986  20.255  1.00 17.48           C  0.024
ANISOU  260  CD2 LEU A  17     2279   2341   2022    -66     46    -67       C
ATOM    261  H   LEU A  17     -10.396  19.519  17.719  1.00 22.64           H  0.027
ATOM    262  HA  LEU A  17      -9.795  21.300  19.616  1.00 22.26           H  0.027
ATOM    263  HB2 LEU A  17      -8.624  20.752  17.136  1.00 21.78           H  0.027
ATOM    264  HB3 LEU A  17      -8.249  22.107  17.878  1.00 21.78           H  0.027
ATOM    265  HG  LEU A  17      -7.835  19.542  18.977  1.00 21.14           H  0.026
ATOM    266 HD11 LEU A  17      -5.525  20.067  18.558  1.00 21.25           H  0.026
ATOM    267 HD12 LEU A  17      -6.340  20.112  17.195  1.00 21.25           H  0.026
ATOM    268 HD13 LEU A  17      -5.976  21.463  17.949  1.00 21.25           H  0.026
ATOM    269 HD21 LEU A  17      -6.458  20.637  20.598  1.00 20.97           H  0.026
ATOM    270 HD22 LEU A  17      -7.261  21.955  20.223  1.00 20.97           H  0.026
ATOM    271 HD23 LEU A  17      -8.029  20.699  20.819  1.00 20.97           H  0.026
ATOM    272  N   ASP A  18     -11.695  22.315  17.307  1.00 18.44           N  0.024
ANISOU  272  N   ASP A  18     2092   2813   2102     94    -63    180       N
ATOM    273  CA  ASP A  18     -12.371  23.530  16.785  1.00 20.50           C  0.026
ANISOU  273  CA  ASP A  18     2106   2996   2687    -11     21     17       C
ATOM    274  C   ASP A  18     -13.055  24.290  17.933  1.00 19.70           C  0.025
ANISOU  274  C   ASP A  18     1835   2925   2724    -56    175    400       C
ATOM    275  O   ASP A  18     -13.991  23.701  18.496  1.00 20.50           O  0.026
ANISOU  275  O   ASP A  18     2075   2947   2766     -7     57    245       O
```

```
ATOM    276  CB  ASP A  18     -13.349  23.157  15.685  1.00 23.30           C  0.027
ANISOU  276  CB  ASP A  18     2327   3307   3221   -130   -273   -105       C
ATOM    277  CG  ASP A  18     -13.970  24.356  15.007  1.00 26.05           C  0.029
ANISOU  277  CG  ASP A  18     2601   3464   3835   -310   -422   -449       C
ATOM    278  OD1 ASP A  18     -13.381  25.449  15.067  1.00 25.53           O  0.029
ANISOU  278  OD1 ASP A  18     2520   3627   3554   -186   -159   -374       O
ATOM    279  OD2 ASP A  18     -15.047  24.179  14.450  1.00 29.29           O  0.031
ANISOU  279  OD2 ASP A  18     3049   3640   4439   -220  -1035   -655       O
ATOM    280  H   ASP A  18     -12.048  21.412  16.940  1.00 22.12           H  0.027
ATOM    281  HA  ASP A  18     -11.609  24.176  16.352  1.00 24.59           H  0.028
ATOM    282  HB2 ASP A  18     -12.827  22.567  14.932  1.00 27.95           H  0.030
ATOM    283  HB3 ASP A  18     -14.146  22.546  16.105  1.00 27.95           H  0.030
ATOM    284  N  AASN A  19     -12.700  25.420  18.242  0.50 18.85           N  0.025
ANISOU  284  N  AASN A  19     1784   2837   2541     -7    -66    480       N
ATOM    285  CA AASN A  19     -13.281  26.228  19.303  0.50 19.44           C  0.025
ANISOU  285  CA AASN A  19     1693   2895   2797     30   -139    447       C
ATOM    286  C  AASN A  19     -12.964  25.684  20.689  0.50 19.95           C  0.025
ANISOU  286  C  AASN A  19     1762   2908   2908    -22     -5    401       C
ATOM    287  O  AASN A  19     -13.580  26.082  21.676  0.50 19.83           O  0.025
ANISOU  287  O  AASN A  19     1887   2942   2706   -114     -9    410       O
ATOM    288  CB AASN A  19     -14.782  26.447  19.084  0.50 21.00           C  0.026
ANISOU  288  CB AASN A  19     1771   3014   3194    134   -355    340       C
ATOM    289  CG AASN A  19     -15.243  27.759  19.647  0.50 23.30           C  0.027
ANISOU  289  CG AASN A  19     2036   3150   3666    109   -362     78       C
ATOM    290  OD1AASN A  19     -14.436  28.684  19.824  0.50 24.42           O  0.028
ANISOU  290  OD1AASN A  19     2195   3224   3860    178   -525     88       O
ATOM    291  ND2AASN A  19     -16.534  27.871  19.903  0.50 24.71           N  0.028
ANISOU  291  ND2AASN A  19     2118   3262   4009     33   -306   -112       N
ATOM    292  H  AASN A  19     -12.053  25.816  17.835  0.50 22.61           H  0.027
ATOM    293  HA AASN A  19     -12.881  27.111  19.279  0.50 23.31           H  0.027
ATOM    294  HB2AASN A  19     -14.971  26.441  18.132  0.50 25.19           H  0.029
ATOM    295  HB3AASN A  19     -15.276  25.737  19.524  0.50 25.19           H  0.029
ATOM    296 HD21AASN A  19     -17.062  27.211  19.746  0.50 29.64           H  0.031
ATOM    297 HD22AASN A  19     -16.846  28.604  20.227  0.50 29.64           H  0.031
ATOM    298  N  BASN A  19     -12.715  25.430  18.236  0.50 19.49           N  0.025
ANISOU  298  N  BASN A  19     1772   2940   2694     37    109    653       N
ATOM    299  CA BASN A  19     -13.295  26.230  19.304  0.50 20.65           C  0.026
ANISOU  299  CA BASN A  19     1668   3099   3078     92    186    766       C
ATOM    300  C  BASN A  19     -12.977  25.673  20.686  0.50 20.55           C  0.026
ANISOU  300  C  BASN A  19     1750   3004   3054      3    150    574       C
ATOM    301  O  BASN A  19     -13.600  26.062  21.674  0.50 20.48           O  0.026
ANISOU  301  O  BASN A  19     1879   3025   2876   -111    167    628       O
ATOM    302  CB BASN A  19     -14.793  26.472  19.067  0.50 23.22           C  0.027
ANISOU  302  CB BASN A  19     1723   3429   3672    254    240    867       C
ATOM    303  CG BASN A  19     -15.073  27.044  17.676  0.50 26.24           C  0.029
ANISOU  303  CG BASN A  19     1968   3769   4233    249    511    861       C
ATOM    304  OD1BASN A  19     -14.445  28.010  17.255  0.50 28.54           O  0.030
ANISOU  304  OD1BASN A  19     2309   3875   4660    263    441    955       O
ATOM    305  ND2BASN A  19     -16.014  26.439  16.954  0.50 26.94           N  0.030
ANISOU  305  ND2BASN A  19     1987   3927   4322    261    463    787       N
ATOM    306  H  BASN A  19     -12.080  25.836  17.821  0.50 23.38           H  0.028
ATOM    307  HA BASN A  19     -12.893  27.113  19.301  0.50 24.76           H  0.028
ATOM    308  HB2BASN A  19     -15.268  25.631  19.149  0.50 27.85           H  0.030
ATOM    309  HB3BASN A  19     -15.121  27.105  19.726  0.50 27.85           H  0.030
ATOM    310 HD21BASN A  19     -16.431  25.760  17.276  0.50 32.32           H  0.032
ATOM    311 HD22BASN A  19     -16.203  26.728  16.167  0.50 32.32           H  0.032
ATOM    312  N   TYR A  20     -11.978  24.798  20.787  1.00 19.19           N  0.025
ANISOU  312  N   TYR A  20     1697   2848   2745     20     81    414       N
ATOM    313  CA  TYR A  20     -11.552  24.302  22.089  1.00 17.72           C  0.024
ANISOU  313  CA  TYR A  20     1790   2831   2113      4    101    319       C
ATOM    314  C   TYR A  20     -10.940  25.461  22.862  1.00 17.40           C  0.024
ANISOU  314  C   TYR A  20     1901   2653   2058     87    331    327       C
ATOM    315  O   TYR A  20     -10.055  26.162  22.348  1.00 17.14           O  0.024
ANISOU  315  O   TYR A  20     1914   2516   2081    159    381    462       O
ATOM    316  CB  TYR A  20     -10.571  23.125  21.953  1.00 18.70           C  0.025
ANISOU  316  CB  TYR A  20     1832   2864   2411     49    124    341       C
ATOM    317  CG  TYR A  20     -10.341  22.476  23.294  1.00 17.88           C  0.024
ANISOU  317  CG  TYR A  20     1816   2777   2201     88     72    322       C
ATOM    318  CD1 TYR A  20     -11.174  21.448  23.758  1.00 19.17           C  0.025
ANISOU  318  CD1 TYR A  20     2117   2905   2263    104    206    360       C
ATOM    319  CD2 TYR A  20      -9.332  22.931  24.130  1.00 17.42           C  0.024
ANISOU  319  CD2 TYR A  20     1820   2728   2070    155    147    472       C
ATOM    320  CE1 TYR A  20     -11.005  20.919  25.022  1.00 19.99           C  0.025
ANISOU  320  CE1 TYR A  20     2195   2992   2408     94    260    481       C
ATOM    321  CE2 TYR A  20      -9.147  22.418  25.390  1.00 18.26           C  0.024
ANISOU  321  CE2 TYR A  20     1985   2871   2083    341    353    428       C
ATOM    322  CZ  TYR A  20      -9.993  21.416  25.838  1.00 19.55           C  0.025
ANISOU  322  CZ  TYR A  20     2178   3085   2165    205    362    773       C
```

```
ATOM    323  OH  TYR A  20      -9.774  20.903  27.087  1.00 22.20           O  0.027
ANISOU  323  OH  TYR A  20     2489   3466   2480    328    378    756       O
ATOM    324  H   TYR A  20     -11.538  24.481  20.120  1.00 23.01           H  0.027
ATOM    325  HA  TYR A  20     -12.310  23.952  22.582  1.00 21.26           H  0.026
ATOM    326  HB2 TYR A  20     -10.940  22.464  21.347  1.00 22.43           H  0.027
ATOM    327  HB3 TYR A  20      -9.721  23.448  21.614  1.00 22.43           H  0.027
ATOM    328  HD1 TYR A  20     -11.848  21.120  23.208  1.00 22.99           H  0.027
ATOM    329  HD2 TYR A  20      -8.766  23.604  23.829  1.00 20.89           H  0.026
ATOM    330  HE1 TYR A  20     -11.562  20.239  25.324  1.00 23.97           H  0.028
ATOM    331  HE2 TYR A  20      -8.464  22.738  25.935  1.00 21.90           H  0.027
ATOM    332  HH  TYR A  20      -9.169  21.338  27.474  1.00 26.63           H  0.029
ATOM    333  N   ARG A  21     -11.437  25.680  24.080  1.00 18.08           N  0.024
ANISOU  333  N   ARG A  21     1997   2724   2149     76    330    315       N
ATOM    334  CA  ARG A  21     -11.043  26.831  24.895  1.00 17.74           C  0.024
ANISOU  334  CA  ARG A  21     1973   2729   2038     15    434    576       C
ATOM    335  C   ARG A  21     -11.240  28.141  24.127  1.00 17.04           C  0.024
ANISOU  335  C   ARG A  21     1911   2810   1755     97    142    227       C
ATOM    336  O   ARG A  21     -10.535  29.133  24.373  1.00 17.86           O  0.024
ANISOU  336  O   ARG A  21     1978   2887   1920    -42    257    -44       O
ATOM    337  CB  ARG A  21      -9.624  26.702  25.465  1.00 21.70           C  0.027
ANISOU  337  CB  ARG A  21     2355   2972   2918    -52    554   1245       C
ATOM    338  CG  ARG A  21      -9.540  26.183  26.912  1.00 28.66           C  0.030
ANISOU  338  CG  ARG A  21     2832   3809   4248    -46    155   1225       C
ATOM    339  CD  ARG A  21     -10.371  26.990  27.897  1.00 33.75           C  0.033
ANISOU  339  CD  ARG A  21     3416   4452   4954   -236     91   1214       C
ATOM    340  NE  ARG A  21      -9.680  27.150  29.176  1.00 37.72           N  0.035
ANISOU  340  NE  ARG A  21     3625   4862   5843   -178   -270   1431       N
ATOM    341  CZ  ARG A  21      -9.889  26.425  30.271  1.00 39.92           C  0.036
ANISOU  341  CZ  ARG A  21     3829   5248   6090   -349   -334   1526       C
ATOM    342  NH1 ARG A  21     -10.669  25.352  30.262  1.00 41.83           N  0.037
ANISOU  342  NH1 ARG A  21     3910   5440   6542   -388   -481   1550       N
ATOM    343  NH2 ARG A  21      -9.274  26.773  31.400  1.00 40.42           N  0.036
ANISOU  343  NH2 ARG A  21     3955   5358   6043   -457   -141   1396       N
ATOM    344  H   ARG A  21     -12.013  25.168  24.463  1.00 21.68           H  0.027
ATOM    345  HA  ARG A  21     -11.628  26.860  25.668  1.00 21.27           H  0.026
ATOM    346  HB2 ARG A  21      -9.125  26.085  24.907  1.00 26.03           H  0.029
ATOM    347  HB3 ARG A  21      -9.206  27.577  25.446  1.00 26.03           H  0.029
ATOM    348  HG2 ARG A  21      -9.859  25.268  26.935  1.00 34.38           H  0.033
ATOM    349  HG3 ARG A  21      -8.616  26.220  27.204  1.00 34.38           H  0.033
ATOM    350  HD2 ARG A  21     -10.540  27.871  27.529  1.00 40.48           H  0.036
ATOM    351  HD3 ARG A  21     -11.210  26.531  28.060  1.00 40.48           H  0.036
ATOM    352  HE  ARG A  21      -9.085  27.769  29.225  1.00 45.25           H  0.038
ATOM    353 HH11 ARG A  21     -11.057  25.108  29.534  1.00 50.18           H  0.040
ATOM    354 HH12 ARG A  21     -10.788  24.901  30.984  1.00 50.18           H  0.040
ATOM    355 HH21 ARG A  21      -8.752  27.457  31.413  1.00 48.49           H  0.040
ATOM    356 HH22 ARG A  21      -9.399  26.314  32.116  1.00 48.49           H  0.040
ATOM    357  N   GLY A  22     -12.175  28.148  23.165  1.00 16.01           N  0.023
ANISOU  357  N   GLY A  22     1803   2776   1503    172    450    144       N
ATOM    358  CA  GLY A  22     -12.474  29.354  22.408  1.00 16.61           C  0.023
ANISOU  358  CA  GLY A  22     1720   2684   1908    288     61    279       C
ATOM    359  C   GLY A  22     -11.589  29.589  21.201  1.00 16.40           C  0.023
ANISOU  359  C   GLY A  22     1860   2543   1827    284     98     15       C
ATOM    360  O   GLY A  22     -11.736  30.628  20.548  1.00 15.88           O  0.023
ANISOU  360  O   GLY A  22     1887   2230   1919    141    153    101       O
ATOM    361  H   GLY A  22     -12.644  27.464  22.938  1.00 19.20           H  0.025
ATOM    362  HA2 GLY A  22     -13.392  29.305  22.096  1.00 19.92           H  0.025
ATOM    363  HA3 GLY A  22     -12.381  30.120  22.995  1.00 19.92           H  0.025
ATOM    364  N   TYR A  23     -10.667  28.670  20.869  1.00 15.36           N  0.022
ANISOU  364  N   TYR A  23     1769   2540   1529    220    231    147       N
ATOM    365  CA  TYR A  23      -9.719  28.867  19.769  1.00 14.30           C  0.022
ANISOU  365  CA  TYR A  23     1773   2348   1314    113    110    -29       C
ATOM    366  C   TYR A  23     -10.242  28.151  18.524  1.00 14.70           C  0.022
ANISOU  366  C   TYR A  23     1787   2351   1448     92    211     66       C
ATOM    367  O   TYR A  23     -10.329  26.917  18.473  1.00 14.84           O  0.022
ANISOU  367  O   TYR A  23     1786   2345   1508     26     57    119       O
ATOM    368  CB  TYR A  23      -8.333  28.377  20.175  1.00 13.36           C  0.021
ANISOU  368  CB  TYR A  23     1869   2125   1081    176     29    -99       C
ATOM    369  CG  TYR A  23      -7.680  29.305  21.180  1.00 12.67           C  0.020
ANISOU  369  CG  TYR A  23     1832   1990    992    215    106    -23       C
ATOM    370  CD1 TYR A  23      -6.989  30.431  20.753  1.00 13.14           C  0.021
ANISOU  370  CD1 TYR A  23     1908   2074   1011    187     69    151       C
ATOM    371  CD2 TYR A  23      -7.772  29.087  22.551  1.00 13.53           C  0.021
ANISOU  371  CD2 TYR A  23     1933   2052   1156    212    110    129       C
ATOM    372  CE1 TYR A  23      -6.389  31.248  21.649  1.00 12.99           C  0.021
ANISOU  372  CE1 TYR A  23     1857   2005   1073     91     10    187       C
ATOM    373  CE2 TYR A  23      -7.207  29.931  23.455  1.00 13.32           C  0.021
ANISOU  373  CE2 TYR A  23     1811   2069   1180    327    101    122       C
ATOM    374  CZ  TYR A  23      -6.523  31.015  22.988  1.00 13.48           C  0.021
```

```
ANISOU  374  CZ  TYR A  23     1927   1987   1206    222    -69     93       C
ATOM    375  OH  TYR A  23      -5.875  31.871  23.874  1.00 14.35           O  0.022
ANISOU  375  OH  TYR A  23     1992   1988   1470    171    -54    120       O
ATOM    376  H   TYR A  23     -10.573  27.918  21.274  1.00 18.42           H  0.024
ATOM    377  HA  TYR A  23      -9.658  29.810  19.552  1.00 17.15           H  0.024
ATOM    378  HB2 TYR A  23      -8.411  27.498  20.578  1.00 16.02           H  0.023
ATOM    379  HB3 TYR A  23      -7.767  28.332  19.389  1.00 16.02           H  0.023
ATOM    380  HD1 TYR A  23      -6.938  30.626  19.845  1.00 15.76           H  0.023
ATOM    381  HD2 TYR A  23      -8.234  28.340  22.859  1.00 16.23           H  0.023
ATOM    382  HE1 TYR A  23      -5.883  31.970  21.353  1.00 15.57           H  0.022
ATOM    383  HE2 TYR A  23      -7.284  29.774  24.368  1.00 15.97           H  0.023
ATOM    384  HH  TYR A  23      -6.164  31.751  24.653  1.00 17.20           H  0.024
ATOM    385  N   SER A  24     -10.610  28.944  17.525  1.00 15.58           N  0.022
ANISOU  385  N   SER A  24     2140   2460   1320     -9    284    125       N
ATOM    386  CA  SER A  24     -11.096  28.382  16.281  1.00 14.94           C  0.022
ANISOU  386  CA  SER A  24     2024   2533   1121    -90    160     -9       C
ATOM    387  C   SER A  24     -10.053  27.468  15.624  1.00 14.57           C  0.022
ANISOU  387  C   SER A  24     1794   2523   1218   -187     81    -90       C
ATOM    388  O   SER A  24      -8.836  27.600  15.810  1.00 15.51           O  0.022
ANISOU  388  O   SER A  24     1868   2806   1217   -241     58    -30       O
ATOM    389  CB  SER A  24     -11.487  29.505  15.323  1.00 17.11           C  0.024
ANISOU  389  CB  SER A  24     2293   2611   1596    -65    -37    133       C
ATOM    390  OG  SER A  24     -10.324  30.209  14.919  1.00 19.17           O  0.025
ANISOU  390  OG  SER A  24     2672   2735   1878   -463   -213    183       O
ATOM    391  H   SER A  24     -10.587  29.804  17.545  1.00 18.68           H  0.025
ATOM    392  HA  SER A  24     -11.877  27.837  16.463  1.00 17.92           H  0.024
ATOM    393  HB2 SER A  24     -11.920  29.126  14.543  1.00 20.52           H  0.026
ATOM    394  HB3 SER A  24     -12.092  30.115  15.774  1.00 20.52           H  0.026
ATOM    395  HG  SER A  24      -9.839  29.714  14.445  1.00 23.00           H  0.027
ATOM    396  N   LEU A  25     -10.549  26.588  14.750  1.00 15.79           N  0.023
ANISOU  396  N   LEU A  25     2021   2421   1560   -108   -151     66       N
ATOM    397  CA  LEU A  25      -9.694  25.607  14.101  1.00 15.32           C  0.022
ANISOU  397  CA  LEU A  25     1980   2206   1635   -208   -198    -48       C
ATOM    398  C   LEU A  25      -8.499  26.225  13.423  1.00 14.01           C  0.021
ANISOU  398  C   LEU A  25     1838   2060   1424      9    -88     36       C
ATOM    399  O   LEU A  25      -7.396  25.683  13.493  1.00 14.89           O  0.022
ANISOU  399  O   LEU A  25     1846   1884   1930     99   -225     94       O
ATOM    400  CB  LEU A  25     -10.528  24.847  13.081  1.00 17.67           C  0.024
ANISOU  400  CB  LEU A  25     2263   2420   2031   -306   -246    160       C
ATOM    401  CG  LEU A  25      -9.916  23.600  12.465  1.00 17.68           C  0.024
ANISOU  401  CG  LEU A  25     2256   2547   1915   -159   -223    179       C
ATOM    402  CD1 LEU A  25      -9.518  22.575  13.531  1.00 18.64           C  0.025
ANISOU  402  CD1 LEU A  25     2413   2460   2210   -180   -488    271       C
ATOM    403  CD2 LEU A  25     -10.938  23.000  11.476  1.00 18.11           C  0.024
ANISOU  403  CD2 LEU A  25     2293   2675   1912   -194   -501    149       C
ATOM    404  H   LEU A  25     -11.376  26.542  14.519  1.00 18.94           H  0.025
ATOM    405  HA  LEU A  25      -9.351  25.001  14.776  1.00 18.37           H  0.024
ATOM    406  HB2 LEU A  25     -11.350  24.571  13.516  1.00 21.19           H  0.026
ATOM    407  HB3 LEU A  25     -10.727  25.452  12.349  1.00 21.19           H  0.026
ATOM    408  HG  LEU A  25      -9.099  23.830  11.996  1.00 21.20           H  0.026
ATOM    409 HD11 LEU A  25      -9.250  21.752  13.094  1.00 22.36           H  0.027
ATOM    410 HD12 LEU A  25      -8.779  22.930  14.050  1.00 22.36           H  0.027
ATOM    411 HD13 LEU A  25     -10.279  22.409  14.110  1.00 22.36           H  0.027
ATOM    412 HD21 LEU A  25     -10.563  22.196  11.084  1.00 21.72           H  0.027
ATOM    413 HD22 LEU A  25     -11.753  22.785  11.956  1.00 21.72           H  0.027
ATOM    414 HD23 LEU A  25     -11.125  23.651  10.782  1.00 21.72           H  0.027
ATOM    415  N   GLY A  26      -8.684  27.359  12.762  1.00 13.44           N  0.021
ANISOU  415  N   GLY A  26     1850   1882   1377     57    -32   -103       N
ATOM    416  CA  GLY A  26      -7.553  27.975  12.067  1.00 13.13           C  0.021
ANISOU  416  CA  GLY A  26     1855   1818   1317    119   -330    164       C
ATOM    417  C   GLY A  26      -6.384  28.305  12.983  1.00 12.35           C  0.020
ANISOU  417  C   GLY A  26     1776   1908   1010    156   -116    160       C
ATOM    418  O   GLY A  26      -5.235  28.351  12.529  1.00 13.34           O  0.021
ANISOU  418  O   GLY A  26     1956   1968   1144    -23   -177    112       O
ATOM    419  H   GLY A  26      -9.429  27.783  12.699  1.00 16.12           H  0.023
ATOM    420  HA2 GLY A  26      -7.235  27.369  11.380  1.00 15.75           H  0.023
ATOM    421  HA3 GLY A  26      -7.849  28.799  11.648  1.00 15.75           H  0.023
ATOM    422  N   ASN A  27      -6.667  28.721  14.233  1.00 12.55           N  0.020
ANISOU  422  N   ASN A  27     1674   1986   1107    226     -5   -188       N
ATOM    423  CA  ASN A  27      -5.590  28.989  15.191  1.00 12.88           C  0.020
ANISOU  423  CA  ASN A  27     1739   1895   1261    126    -35    -67       C
ATOM    424  C   ASN A  27      -4.695  27.768  15.366  1.00 12.83           C  0.020
ANISOU  424  C   ASN A  27     1768   1877   1231    180     10     -9       C
ATOM    425  O   ASN A  27      -3.464  27.880  15.488  1.00 12.94           O  0.020
ANISOU  425  O   ASN A  27     1763   1757   1398    199    -73   -105       O
ATOM    426  CB  ASN A  27      -6.181  29.385  16.530  1.00 13.55           C  0.021
ANISOU  426  CB  ASN A  27     2004   1793   1352    188     16   -219       C
ATOM    427  CG  ASN A  27      -6.586  30.833  16.520  1.00 13.38           C  0.021
```

```
ANISOU  427  CG  ASN A  27     1842   1863   1378    221    -86   -132       C
ATOM    428  OD1 ASN A  27      -5.741  31.710  16.568  1.00 13.26           O  0.021
ANISOU  428  OD1 ASN A  27     1796   1830   1412    126   -224    -38       O
ATOM    429  ND2 ASN A  27      -7.897  31.098  16.478  1.00 14.64           N  0.022
ANISOU  429  ND2 ASN A  27     1852   2235   1475    322     88   -219       N
ATOM    430  H   ASN A  27      -7.460  28.851  14.540  1.00 15.05           H  0.022
ATOM    431  HA  ASN A  27      -5.050  29.723  14.857  1.00 15.45           H  0.022
ATOM    432  HB2 ASN A  27      -6.967  28.845  16.710  1.00 16.25           H  0.023
ATOM    433  HB3 ASN A  27      -5.522  29.253  17.229  1.00 16.25           H  0.023
ATOM    434 HD21 ASN A  27      -8.171  31.913  16.470  1.00 17.55           H  0.024
ATOM    435 HD22 ASN A  27      -8.465  30.453  16.458  1.00 17.55           H  0.024
ATOM    436  N   TRP A  28      -5.322  26.598  15.502  1.00 12.68           N  0.020
ANISOU  436  N   TRP A  28     1642   1891   1283     71   -139    114       N
ATOM    437  CA  TRP A  28      -4.614  25.338  15.711  1.00 13.15           C  0.021
ANISOU  437  CA  TRP A  28     1882   1807   1309    169   -124     56       C
ATOM    438  C   TRP A  28      -3.802  24.946  14.493  1.00 13.64           C  0.021
ANISOU  438  C   TRP A  28     1939   1818   1425    110   -299    127       C
ATOM    439  O   TRP A  28      -2.670  24.483  14.610  1.00 13.36           O  0.021
ANISOU  439  O   TRP A  28     1860   1853   1362     58   -200     78       O
ATOM    440  CB  TRP A  28      -5.620  24.247  16.086  1.00 14.41           C  0.022
ANISOU  440  CB  TRP A  28     1904   1816   1754    172   -225    119       C
ATOM    441  CG  TRP A  28      -6.282  24.518  17.387  1.00 13.80           C  0.021
ANISOU  441  CG  TRP A  28     1805   1836   1603     91   -106    140       C
ATOM    442  CD1 TRP A  28      -7.550  24.983  17.589  1.00 14.45           C  0.022
ANISOU  442  CD1 TRP A  28     1822   2104   1562    102     39    -37       C
ATOM    443  CD2 TRP A  28      -5.684  24.412  18.694  1.00 13.31           C  0.021
ANISOU  443  CD2 TRP A  28     1774   2006   1277     94     34    127       C
ATOM    444  NE1 TRP A  28      -7.796  25.134  18.926  1.00 15.03           N  0.022
ANISOU  444  NE1 TRP A  28     1780   2186   1743    110    -13    -29       N
ATOM    445  CE2 TRP A  28      -6.668  24.788  19.625  1.00 13.20           C  0.021
ANISOU  445  CE2 TRP A  28     1706   2092   1215     -5    175    161       C
ATOM    446  CE3 TRP A  28      -4.455  23.941  19.168  1.00 13.64           C  0.021
ANISOU  446  CE3 TRP A  28     1948   2008   1225     62     67     61       C
ATOM    447  CZ2 TRP A  28      -6.446  24.774  21.002  1.00 14.04           C  0.021
ANISOU  447  CZ2 TRP A  28     1749   2515   1071    -12    158    106       C
ATOM    448  CZ3 TRP A  28      -4.217  23.943  20.538  1.00 14.58           C  0.022
ANISOU  448  CZ3 TRP A  28     1999   2259   1284     63    -90     82       C
ATOM    449  CH2 TRP A  28      -5.191  24.383  21.444  1.00 15.23           C  0.022
ANISOU  449  CH2 TRP A  28     1982   2500   1303    -73    117     74       C
ATOM    450  H   TRP A  28      -6.176  26.504  15.477  1.00 15.20           H  0.022
ATOM    451  HA  TRP A  28      -3.990  25.439  16.447  1.00 15.77           H  0.023
ATOM    452  HB2 TRP A  28      -6.305  24.198  15.402  1.00 17.28           H  0.024
ATOM    453  HB3 TRP A  28      -5.157  23.397  16.154  1.00 17.28           H  0.024
ATOM    454  HD1 TRP A  28      -8.159  25.172  16.913  1.00 17.32           H  0.024
ATOM    455  HE1 TRP A  28      -8.537  25.402  19.271  1.00 18.02           H  0.024
ATOM    456  HE3 TRP A  28      -3.807  23.633  18.576  1.00 16.35           H  0.023
ATOM    457  HZ2 TRP A  28      -7.114  25.018  21.601  1.00 16.84           H  0.023
ATOM    458  HZ3 TRP A  28      -3.396  23.646  20.860  1.00 17.49           H  0.024
ATOM    459  HH2 TRP A  28      -4.994  24.413  22.352  1.00 18.26           H  0.024
ATOM    460  N   VAL A  29      -4.382  25.090  13.308  1.00 12.77           N  0.020
ANISOU  460  N   VAL A  29     1995   1845   1013     82   -345    -93       N
ATOM    461  CA  VAL A  29      -3.658  24.788  12.062  1.00 12.25           C  0.020
ANISOU  461  CA  VAL A  29     1968   1783    904    190   -328   -296       C
ATOM    462  C   VAL A  29      -2.474  25.719  11.889  1.00 13.49           C  0.021
ANISOU  462  C   VAL A  29     1907   1917   1301    136   -359   -413       C
ATOM    463  O   VAL A  29      -1.373  25.282  11.536  1.00 13.74           O  0.021
ANISOU  463  O   VAL A  29     1880   1976   1364    212   -289   -279       O
ATOM    464  CB  VAL A  29      -4.610  24.799  10.849  1.00 13.25           C  0.021
ANISOU  464  CB  VAL A  29     2037   1980   1016     42   -382   -253       C
ATOM    465  CG1 VAL A  29      -3.849  24.592   9.539  1.00 13.46           C  0.021
ANISOU  465  CG1 VAL A  29     2120   2097    898    -10   -285   -232       C
ATOM    466  CG2 VAL A  29      -5.716  23.762  11.032  1.00 14.70           C  0.022
ANISOU  466  CG2 VAL A  29     2038   2123   1423    -17   -472   -389       C
ATOM    467  H   VAL A  29      -5.190  25.358  13.190  1.00 15.31           H  0.022
ATOM    468  HA  VAL A  29      -3.302  23.889  12.130  1.00 14.69           H  0.022
ATOM    469  HB  VAL A  29      -5.030  25.671  10.790  1.00 15.88           H  0.023
ATOM    470 HG11 VAL A  29      -4.486  24.422   8.827  1.00 16.14           H  0.023
ATOM    471 HG12 VAL A  29      -3.337  25.392   9.342  1.00 16.14           H  0.023
ATOM    472 HG13 VAL A  29      -3.253  23.833   9.637  1.00 16.14           H  0.023
ATOM    473 HG21 VAL A  29      -6.283  23.763  10.245  1.00 17.63           H  0.024
ATOM    474 HG22 VAL A  29      -5.313  22.888  11.148  1.00 17.63           H  0.024
ATOM    475 HG23 VAL A  29      -6.238  23.993  11.816  1.00 17.63           H  0.024
ATOM    476  N   CYS A  30      -2.692  27.016  12.130  1.00 13.51           N  0.021
ANISOU  476  N   CYS A  30     1880   1803   1452    107   -412   -156       N
ATOM    477  CA  CYS A  30      -1.622  27.993  12.033  1.00 12.76           C  0.020
ANISOU  477  CA  CYS A  30     1862   1875   1111    124   -242   -257       C
ATOM    478  C   CYS A  30      -0.501  27.677  13.019  1.00 12.09           C  0.020
ANISOU  478  C   CYS A  30     1760   1777   1056     94   -193    -72       C
```

```
ATOM    479  O   CYS A  30       0.685  27.722  12.672  1.00 12.40           O  0.020
ANISOU  479  O   CYS A  30     1714   1954   1046    111    -98     29       O
ATOM    480  CB  CYS A  30      -2.207  29.385  12.284  1.00 14.21           C  0.021
ANISOU  480  CB  CYS A  30     1833   2059   1509    -63   -175    -89       C
ATOM    481  SG  CYS A  30      -1.002  30.735  12.107  1.00 13.99           S  0.021
ANISOU  481  SG  CYS A  30     1963   1994   1360     16    -53     50       S
ATOM    482  H   CYS A  30      -3.453  27.350  12.350  1.00 16.20           H  0.023
ATOM    483  HA  CYS A  30      -1.234  27.979  11.144  1.00 15.30           H  0.022
ATOM    484  HB2 CYS A  30      -2.921  29.541  11.646  1.00 17.05           H  0.024
ATOM    485  HB3 CYS A  30      -2.556  29.417  13.188  1.00 17.05           H  0.024
ATOM    486  N   ALA A  31      -0.847  27.293  14.253  1.00 12.33           N  0.020
ANISOU  486  N   ALA A  31     1846   1846    992     62   -140    -16       N
ATOM    487  CA  ALA A  31       0.164  26.967  15.244  1.00 11.83           C  0.020
ANISOU  487  CA  ALA A  31     1769   1972    755     45   -212   -139       C
ATOM    488  C   ALA A  31       0.987  25.771  14.778  1.00 12.54           C  0.020
ANISOU  488  C   ALA A  31     1831   1963    970    105   -367   -119       C
ATOM    489  O   ALA A  31       2.216  25.769  14.886  1.00 12.43           O  0.020
ANISOU  489  O   ALA A  31     1678   1957   1089    125   -173   -114       O
ATOM    490  CB  ALA A  31      -0.460  26.675  16.615  1.00 12.96           C  0.020
ANISOU  490  CB  ALA A  31     1826   1934   1163     24   -205    -36       C
ATOM    491  H   ALA A  31      -1.656  27.217  14.534  1.00 14.78           H  0.022
ATOM    492  HA  ALA A  31       0.742  27.738  15.357  1.00 14.19           H  0.021
ATOM    493  HB1 ALA A  31       0.247  26.469  17.246  1.00 15.54           H  0.022
ATOM    494  HB2 ALA A  31      -0.951  27.458  16.911  1.00 15.54           H  0.022
ATOM    495  HB3 ALA A  31      -1.062  25.919  16.533  1.00 15.54           H  0.022
ATOM    496  N   ALA A  32       0.320  24.739  14.251  1.00 12.71           N  0.020
ANISOU  496  N   ALA A  32     1717   1905   1206     73   -188   -160       N
ATOM    497  CA  ALA A  32       1.024  23.552  13.774  1.00 13.53           C  0.021
ANISOU  497  CA  ALA A  32     1839   1834   1467    114   -124   -146       C
ATOM    498  C   ALA A  32       1.939  23.898  12.604  1.00 12.42           C  0.020
ANISOU  498  C   ALA A  32     1782   1823   1113     89   -145   -181       C
ATOM    499  O   ALA A  32       3.056  23.374  12.494  1.00 13.48           O  0.021
ANISOU  499  O   ALA A  32     1758   1965   1396    147    -90    -33       O
ATOM    500  CB  ALA A  32       0.034  22.456  13.381  1.00 14.38           C  0.022
ANISOU  500  CB  ALA A  32     2157   1760   1546     92    -98    -90       C
ATOM    501  H   ALA A  32      -0.534  24.704  14.159  1.00 15.24           H  0.022
ATOM    502  HA  ALA A  32       1.568  23.202  14.497  1.00 16.22           H  0.023
ATOM    503  HB1 ALA A  32       0.527  21.677  13.081  1.00 17.24           H  0.024
ATOM    504  HB2 ALA A  32      -0.507  22.228  14.153  1.00 17.24           H  0.024
ATOM    505  HB3 ALA A  32      -0.533  22.785  12.666  1.00 17.24           H  0.024
ATOM    506  N   LYS A  33       1.476  24.769  11.702  1.00 12.96           N  0.020
ANISOU  506  N   LYS A  33     1979   1906   1041     45   -113     26       N
ATOM    507  CA  LYS A  33       2.295  25.163  10.565  1.00 12.44           C  0.020
ANISOU  507  CA  LYS A  33     2105   1876    747     74   -255     86       C
ATOM    508  C   LYS A  33       3.606  25.727  11.056  1.00 13.52           C  0.021
ANISOU  508  C   LYS A  33     1988   1865   1285     41     65   -100       C
ATOM    509  O   LYS A  33       4.668  25.357  10.551  1.00 14.84           O  0.022
ANISOU  509  O   LYS A  33     1969   2030   1640      4   -229   -162       O
ATOM    510  CB  LYS A  33       1.550  26.208   9.708  1.00 14.38           C  0.022
ANISOU  510  CB  LYS A  33     2430   2170    864     35   -284     67       C
ATOM    511  CG  LYS A  33       2.409  26.860   8.657  1.00 16.59           C  0.023
ANISOU  511  CG  LYS A  33     2690   2538   1077    143   -122    173       C
ATOM    512  CD  LYS A  33       2.904  25.913   7.659  1.00 18.82           C  0.025
ANISOU  512  CD  LYS A  33     3038   2861   1252    175    166    353       C
ATOM    513  CE  LYS A  33       3.636  26.572   6.538  1.00 22.25           C  0.027
ANISOU  513  CE  LYS A  33     3438   3133   1884    495    566    569       C
ATOM    514  NZ  LYS A  33       4.083  25.632   5.500  1.00 25.14           N  0.029
ANISOU  514  NZ  LYS A  33     3713   3347   2493    600    568    438       N
ATOM    515  H   LYS A  33       0.700  25.140  11.730  1.00 15.54           H  0.022
ATOM    516  HA  LYS A  33       2.465  24.390  10.005  1.00 14.92           H  0.022
ATOM    517  HB2 LYS A  33       0.811  25.771   9.257  1.00 17.25           H  0.024
ATOM    518  HB3 LYS A  33       1.216  26.908  10.291  1.00 17.25           H  0.024
ATOM    519  HG2 LYS A  33       1.887  27.535   8.196  1.00 19.90           H  0.025
ATOM    520  HG3 LYS A  33       3.177  27.269   9.086  1.00 19.90           H  0.025
ATOM    521  HD2 LYS A  33       3.513  25.292   8.088  1.00 22.57           H  0.027
ATOM    522  HD3 LYS A  33       2.151  25.432   7.281  1.00 22.57           H  0.027
ATOM    523  HE2 LYS A  33       3.050  27.221   6.119  1.00 26.69           H  0.029
ATOM    524  HE3 LYS A  33       4.422  27.016   6.895  1.00 26.69           H  0.029
ATOM    525  HZ1 LYS A  33       4.639  25.033   5.852  1.00 30.16           H  0.031
ATOM    526  HZ2 LYS A  33       3.383  25.208   5.152  1.00 30.16           H  0.031
ATOM    527  HZ3 LYS A  33       4.505  26.073   4.852  1.00 30.16           H  0.031
ATOM    528  N   PHE A  34       3.542  26.692  11.978  1.00 13.37           N  0.021
ANISOU  528  N   PHE A  34     1903   1875   1304     24   -193     26       N
ATOM    529  CA  PHE A  34       4.766  27.384  12.368  1.00 13.45           C  0.021
ANISOU  529  CA  PHE A  34     1975   1938   1197   -149    -97     77       C
ATOM    530  C   PHE A  34       5.552  26.660  13.445  1.00 14.02           C  0.021
ANISOU  530  C   PHE A  34     1874   2213   1240   -154   -124    122       C
ATOM    531  O   PHE A  34       6.753  26.904  13.568  1.00 16.14           O  0.023
```

```
ANISOU  531  O   PHE A  34     1983   2497   1651   -454   -306    338       O
ATOM    532  CB  PHE A  34       4.452  28.825  12.719  1.00 13.29           C  0.021
ANISOU  532  CB  PHE A  34     2044   1962   1044   -175    134     -9       C
ATOM    533  CG  PHE A  34       3.965  29.611  11.528  1.00 13.76           C  0.021
ANISOU  533  CG  PHE A  34     2124   1949   1156   -266    104     70       C
ATOM    534  CD1 PHE A  34       4.688  29.627  10.336  1.00 14.70           C  0.022
ANISOU  534  CD1 PHE A  34     2187   1985   1413   -366    -40    255       C
ATOM    535  CD2 PHE A  34       2.741  30.268  11.564  1.00 16.26           C  0.023
ANISOU  535  CD2 PHE A  34     2285   2196   1697   -265    -49    418       C
ATOM    536  CE1 PHE A  34       4.214  30.320   9.248  1.00 15.89           C  0.023
ANISOU  536  CE1 PHE A  34     2168   2144   1726   -351    -57    239       C
ATOM    537  CE2 PHE A  34       2.288  30.966  10.450  1.00 16.59           C  0.023
ANISOU  537  CE2 PHE A  34     2234   2299   1769   -416    -24    547       C
ATOM    538  CZ  PHE A  34       3.049  31.004   9.312  1.00 16.41           C  0.023
ANISOU  538  CZ  PHE A  34     2253   2241   1741   -390   -131    511       C
ATOM    539  H   PHE A  34       2.826  26.952  12.376  1.00 16.04           H  0.023
ATOM    540  HA  PHE A  34       5.377  27.434  11.616  1.00 16.13           H  0.023
ATOM    541  HB2 PHE A  34       3.758  28.842  13.396  1.00 15.94           H  0.023
ATOM    542  HB3 PHE A  34       5.255  29.252  13.055  1.00 15.94           H  0.023
ATOM    543  HD1 PHE A  34       5.495  29.168  10.278  1.00 17.63           H  0.024
ATOM    544  HD2 PHE A  34       2.224  30.241  12.337  1.00 19.50           H  0.025
ATOM    545  HE1 PHE A  34       4.701  30.318   8.455  1.00 19.06           H  0.025
ATOM    546  HE2 PHE A  34       1.469  31.406  10.477  1.00 19.89           H  0.025
ATOM    547  HZ  PHE A  34       2.764  31.503   8.580  1.00 19.68           H  0.025
ATOM    548  N   GLU A  35       4.925  25.791  14.245  1.00 13.13           N  0.021
ANISOU  548  N   GLU A  35     1832   2164    993    -74    -77     65       N
ATOM    549  CA  GLU A  35       5.687  24.977  15.180  1.00 12.82           C  0.020
ANISOU  549  CA  GLU A  35     1779   2212    880    -32   -179     47       C
ATOM    550  C   GLU A  35       6.460  23.894  14.442  1.00 13.98           C  0.021
ANISOU  550  C   GLU A  35     1865   2344   1103     54   -384    -74       C
ATOM    551  O   GLU A  35       7.616  23.629  14.792  1.00 15.14           O  0.022
ANISOU  551  O   GLU A  35     1836   2576   1340     88   -263   -225       O
ATOM    552  CB  GLU A  35       4.763  24.387  16.224  1.00 13.04           C  0.021
ANISOU  552  CB  GLU A  35     1905   2268    781   -176   -240    -52       C
ATOM    553  CG  GLU A  35       4.225  25.391  17.196  1.00 14.90           C  0.022
ANISOU  553  CG  GLU A  35     2179   2360   1122   -200   -152   -179       C
ATOM    554  CD  GLU A  35       5.248  26.025  18.080  1.00 14.80           C  0.022
ANISOU  554  CD  GLU A  35     2063   2257   1303      5   -314      2       C
ATOM    555  OE1 GLU A  35       6.414  25.606  18.087  1.00 15.47           O  0.022
ANISOU  555  OE1 GLU A  35     2103   2322   1454    258   -422   -208       O
ATOM    556  OE2 GLU A  35       4.906  26.971  18.805  1.00 15.26           O  0.022
ANISOU  556  OE2 GLU A  35     2136   2484   1179    -24    113    -88       O
ATOM    557  H   GLU A  35       4.075  25.660  14.262  1.00 15.74           H  0.023
ATOM    558  HA  GLU A  35       6.330  25.533  15.646  1.00 15.37           H  0.022
ATOM    559  HB2 GLU A  35       4.008  23.976  15.775  1.00 15.63           H  0.023
ATOM    560  HB3 GLU A  35       5.252  23.718  16.730  1.00 15.63           H  0.023
ATOM    561  HG2 GLU A  35       3.793  26.101  16.696  1.00 17.87           H  0.024
ATOM    562  HG3 GLU A  35       3.580  24.949  17.769  1.00 17.87           H  0.024
ATOM    563  N   SER A  36       5.838  23.205  13.486  1.00 14.79           N  0.022
ANISOU  563  N   SER A  36     1994   2319   1305    133   -352    -82       N
ATOM    564  CA  SER A  36       6.404  21.961  12.961  1.00 15.10           C  0.022
ANISOU  564  CA  SER A  36     2082   2445   1212    280   -341   -155       C
ATOM    565  C   SER A  36       6.390  21.847  11.439  1.00 14.99           C  0.022
ANISOU  565  C   SER A  36     2075   2404   1217    353   -257   -181       C
ATOM    566  O   SER A  36       6.818  20.804  10.907  1.00 15.94           O  0.023
ANISOU  566  O   SER A  36     2204   2397   1454    520   -106   -208       O
ATOM    567  CB  SER A  36       5.664  20.733  13.502  1.00 14.64           C  0.022
ANISOU  567  CB  SER A  36     2111   2502    948    391   -296     60       C
ATOM    568  OG  SER A  36       4.341  20.751  12.974  1.00 14.03           O  0.021
ANISOU  568  OG  SER A  36     2112   2080   1139    367   -239    -79       O
ATOM    569  H   SER A  36       5.091  23.436  13.126  1.00 17.73           H  0.024
ATOM    570  HA  SER A  36       7.324  21.938  13.267  1.00 18.11           H  0.024
ATOM    571  HB2 SER A  36       6.119  19.925  13.218  1.00 17.55           H  0.024
ATOM    572  HB3 SER A  36       5.631  20.772  14.471  1.00 17.55           H  0.024
ATOM    573  HG  SER A  36       3.932  21.433  13.246  1.00 16.82           H  0.023
ATOM    574  N   ASN A  37       5.878  22.837  10.720  1.00 15.32           N  0.022
ANISOU  574  N   ASN A  37     2239   2309   1272    338    -27    -17       N
ATOM    575  CA  ASN A  37       5.625  22.711   9.288  1.00 16.16           C  0.023
ANISOU  575  CA  ASN A  37     2491   2394   1254    456    -68    -63       C
ATOM    576  C   ASN A  37       4.783  21.470   8.963  1.00 15.46           C  0.022
ANISOU  576  C   ASN A  37     2390   2447   1038    453   -169   -123       C
ATOM    577  O   ASN A  37       4.974  20.833   7.926  1.00 15.76           O  0.023
ANISOU  577  O   ASN A  37     2485   2561    943    389    -68   -212       O
ATOM    578  CB  ASN A  37       6.869  22.761   8.403  1.00 18.24           C  0.024
ANISOU  578  CB  ASN A  37     2727   2713   1492    369    150     24       C
ATOM    579  CG  ASN A  37       6.552  23.262   7.006  1.00 20.63           C  0.026
ANISOU  579  CG  ASN A  37     2860   2968   2011    540    361     99       C
ATOM    580  OD1 ASN A  37       5.457  23.735   6.749  1.00 22.40           O  0.027
```

```
ANISOU  580  OD1 ASN A  37     3125   3210   2176    499     16    116       O
ATOM    581  ND2 ASN A  37       7.495  23.050   6.072  1.00 23.87           N  0.028
ANISOU  581  ND2 ASN A  37     3078   3192   2800    569    561    281       N
ATOM    582  H   ASN A  37       5.664  23.605  11.042  1.00 18.37           H  0.024
ATOM    583  HA  ASN A  37       5.116  23.502   9.054  1.00 19.38           H  0.025
ATOM    584  HB2 ASN A  37       7.519  23.361   8.800  1.00 21.88           H  0.027
ATOM    585  HB3 ASN A  37       7.243  21.869   8.327  1.00 21.88           H  0.027
ATOM    586 HD21 ASN A  37       8.227  22.650   6.282  1.00 28.63           H  0.030
ATOM    587 HD22 ASN A  37       7.367  23.314   5.264  1.00 28.63           H  0.030
ATOM    588  N   PHE A  38       3.885  21.107   9.897  1.00 14.20           N  0.021
ANISOU  588  N   PHE A  38     2017   2315   1065    442   -348   -131       N
ATOM    589  CA  PHE A  38       2.926  20.019   9.763  1.00 14.53           C  0.022
ANISOU  589  CA  PHE A  38     2101   2109   1310    427   -426   -363       C
ATOM    590  C   PHE A  38       3.569  18.636   9.843  1.00 14.65           C  0.022
ANISOU  590  C   PHE A  38     2116   2073   1376    417   -434   -288       C
ATOM    591  O   PHE A  38       2.966  17.650   9.459  1.00 15.14           O  0.022
ANISOU  591  O   PHE A  38     2077   2269   1407    455   -453   -301       O
ATOM    592  CB  PHE A  38       2.138  20.129   8.469  1.00 15.47           C  0.022
ANISOU  592  CB  PHE A  38     2238   2158   1482    489   -459   -348       C
ATOM    593  CG  PHE A  38       1.287  21.395   8.337  1.00 14.50           C  0.022
ANISOU  593  CG  PHE A  38     2236   2257   1015    364   -430   -139       C
ATOM    594  CD1 PHE A  38       0.601  21.991   9.411  1.00 14.50           C  0.022
ANISOU  594  CD1 PHE A  38     2123   2203   1184    264   -371   -201       C
ATOM    595  CD2 PHE A  38       1.092  21.935   7.095  1.00 17.07           C  0.024
ANISOU  595  CD2 PHE A  38     2601   2503   1382    322   -408     97       C
ATOM    596  CE1 PHE A  38      -0.235  23.081   9.211  1.00 14.74           C  0.022
ANISOU  596  CE1 PHE A  38     2063   2388   1150    417   -194   -298       C
ATOM    597  CE2 PHE A  38       0.269  23.028   6.898  1.00 16.97           C  0.023
ANISOU  597  CE2 PHE A  38     2578   2464   1406    378   -540    124       C
ATOM    598  CZ  PHE A  38      -0.384  23.603   7.967  1.00 15.88           C  0.023
ANISOU  598  CZ  PHE A  38     2360   2290   1383    443   -442   -235       C
ATOM    599  H   PHE A  38       3.817  21.505  10.656  1.00 17.03           H  0.023
ATOM    600  HA  PHE A  38       2.308  20.093  10.507  1.00 17.42           H  0.024
ATOM    601  HB2 PHE A  38       2.762  20.119   7.727  1.00 18.55           H  0.025
ATOM    602  HB3 PHE A  38       1.539  19.368   8.409  1.00 18.55           H  0.025
ATOM    603  HD1 PHE A  38       0.709  21.649  10.269  1.00 17.39           H  0.024
ATOM    604  HD2 PHE A  38       1.526  21.557   6.364  1.00 20.47           H  0.026
ATOM    605  HE1 PHE A  38      -0.693  23.453   9.929  1.00 17.68           H  0.024
ATOM    606  HE2 PHE A  38       0.155  23.375   6.043  1.00 20.35           H  0.026
ATOM    607  HZ  PHE A  38      -0.925  24.348   7.837  1.00 19.04           H  0.025
ATOM    608  N   ASN A  39       4.794  18.538  10.312  1.00 14.58           N  0.022
ANISOU  608  N   ASN A  39     2192   2041   1305    444   -455    -30       N
ATOM    609  CA  ASN A  39       5.528  17.277  10.393  1.00 14.31           C  0.022
ANISOU  609  CA  ASN A  39     2170   2079   1187    392   -497   -195       C
ATOM    610  C   ASN A  39       5.365  16.709  11.813  1.00 13.73           C  0.021
ANISOU  610  C   ASN A  39     2141   2123    951    391   -410    -66       C
ATOM    611  O   ASN A  39       5.906  17.247  12.780  1.00 13.74           O  0.021
ANISOU  611  O   ASN A  39     2239   2162    819    349   -284   -103       O
ATOM    612  CB  ASN A  39       6.988  17.530  10.047  1.00 14.88           C  0.022
ANISOU  612  CB  ASN A  39     2168   2149   1337    408   -111   -230       C
ATOM    613  CG  ASN A  39       7.808  16.240   9.961  1.00 15.43           C  0.022
ANISOU  613  CG  ASN A  39     2058   2120   1683    348   -100    -66       C
ATOM    614  OD1 ASN A  39       7.366  15.163  10.366  1.00 14.78           O  0.022
ANISOU  614  OD1 ASN A  39     2103   2064   1448    257   -285   -235       O
ATOM    615  ND2 ASN A  39       8.999  16.339   9.401  1.00 17.74           N  0.024
ANISOU  615  ND2 ASN A  39     2044   2246   2449    431    197     53       N
ATOM    616  H   ASN A  39       5.245  19.209  10.604  1.00 17.48           H  0.024
ATOM    617  HA  ASN A  39       5.165  16.624   9.775  1.00 17.16           H  0.024
ATOM    618  HB2 ASN A  39       7.037  17.975   9.187  1.00 17.85           H  0.024
ATOM    619  HB3 ASN A  39       7.383  18.091  10.734  1.00 17.85           H  0.024
ATOM    620 HD21 ASN A  39       9.499  15.643   9.329  1.00 21.27           H  0.026
ATOM    621 HD22 ASN A  39       9.276  17.099   9.109  1.00 21.27           H  0.026
ATOM    622  N   THR A  40       4.670  15.579  11.942  1.00 15.81           N  0.023
ANISOU  622  N   THR A  40     2211   2254   1543    329   -304    -69       N
ATOM    623  CA  THR A  40       4.503  14.977  13.271  1.00 15.75           C  0.023
ANISOU  623  CA  THR A  40     2107   2268   1609    346   -389   -203       C
ATOM    624  C   THR A  40       5.814  14.568  13.933  1.00 14.11           C  0.021
ANISOU  624  C   THR A  40     2092   2224   1047    446   -248    -99       C
ATOM    625  O   THR A  40       5.851  14.418  15.161  1.00 15.48           O  0.022
ANISOU  625  O   THR A  40     2334   2188   1358    463   -246    -85       O
ATOM    626  CB  THR A  40       3.569  13.754  13.238  1.00 17.70           C  0.024
ANISOU  626  CB  THR A  40     1954   2238   2534    332   -114   -228       C
ATOM    627  OG1 THR A  40       4.190  12.691  12.514  1.00 19.54           O  0.025
ANISOU  627  OG1 THR A  40     2151   2365   2908    256   -256   -536       O
ATOM    628  CG2 THR A  40       2.248  14.104  12.616  1.00 16.94           C  0.023
ANISOU  628  CG2 THR A  40     2016   2248   2173    342   -361    -12       C
ATOM    629  H   THR A  40       4.294  15.151  11.298  1.00 18.96           H  0.025
ATOM    630  HA  THR A  40       4.085  15.663  13.816  1.00 18.89           H  0.025
```

```
ATOM    631  HB  THR A  40       3.396  13.456  14.145  1.00 21.23           H  0.026
ATOM    632  HG1 THR A  40       3.686  12.020  12.499  1.00 23.43           H  0.028
ATOM    633 HG21 THR A  40       1.641  13.350  12.679  1.00 20.32           H  0.026
ATOM    634 HG22 THR A  40       1.856  14.863  13.074  1.00 20.32           H  0.026
ATOM    635 HG23 THR A  40       2.372  14.331  11.681  1.00 20.32           H  0.026
ATOM    636  N   GLN A  41       6.865  14.310  13.154  1.00 14.84           N  0.022
ANISOU  636  N   GLN A  41     2047   2253   1340    433   -189   -194       N
ATOM    637  CA  GLN A  41       8.124  13.806  13.688  1.00 15.20           C  0.022
ANISOU  637  CA  GLN A  41     1988   2313   1472    479   -226   -236       C
ATOM    638  C   GLN A  41       9.086  14.914  14.038  1.00 15.44           C  0.022
ANISOU  638  C   GLN A  41     1931   2467   1467    432   -350   -418       C
ATOM    639  O   GLN A  41      10.204  14.623  14.465  1.00 17.96           O  0.024
ANISOU  639  O   GLN A  41     2137   2708   1978    564   -759   -459       O
ATOM    640  CB  GLN A  41       8.780  12.818  12.699  1.00 15.58           C  0.022
ANISOU  640  CB  GLN A  41     2016   2326   1578    498   -267    -50       C
ATOM    641  CG  GLN A  41       7.887  11.630  12.408  1.00 15.74           C  0.023
ANISOU  641  CG  GLN A  41     2188   2282   1509    602   -229   -214       C
ATOM    642  CD  GLN A  41       8.617  10.517  11.773  1.00 15.67           C  0.023
ANISOU  642  CD  GLN A  41     2265   2182   1508    534    -58   -197       C
ATOM    643  OE1 GLN A  41       9.309   9.765  12.455  1.00 19.39           O  0.025
ANISOU  643  OE1 GLN A  41     2612   2263   2492    861   -432   -201       O
ATOM    644  NE2 GLN A  41       8.538  10.413  10.464  1.00 14.61           N  0.022
ANISOU  644  NE2 GLN A  41     2214   2018   1318    357      6   -351       N
ATOM    645  H   GLN A  41       6.871  14.422  12.302  1.00 17.80           H  0.024
ATOM    646  HA  GLN A  41       7.939  13.306  14.499  1.00 18.22           H  0.024
ATOM    647  HB2 GLN A  41       8.959  13.276  11.863  1.00 18.68           H  0.025
ATOM    648  HB3 GLN A  41       9.609  12.489  13.081  1.00 18.68           H  0.025
ATOM    649  HG2 GLN A  41       7.509  11.306  13.241  1.00 18.87           H  0.025
ATOM    650  HG3 GLN A  41       7.178  11.907  11.807  1.00 18.87           H  0.025
ATOM    651 HE21 GLN A  41       8.075  10.981  10.015  1.00 17.52           H  0.024
ATOM    652 HE22 GLN A  41       8.951   9.777  10.058  1.00 17.52           H  0.024
ATOM    653  N   ALA A  42       8.688  16.176  13.916  1.00 15.48           N  0.022
ANISOU  653  N   ALA A  42     2102   2478   1301    319   -376   -206       N
ATOM    654  CA  ALA A  42       9.573  17.279  14.272  1.00 15.96           C  0.023
ANISOU  654  CA  ALA A  42     2103   2538   1424    181   -229   -245       C
ATOM    655  C   ALA A  42      10.034  17.210  15.735  1.00 15.00           C  0.022
ANISOU  655  C   ALA A  42     1890   2469   1342    331   -271   -268       C
ATOM    656  O   ALA A  42       9.242  16.979  16.652  1.00 14.63           O  0.022
ANISOU  656  O   ALA A  42     2083   2106   1369    280   -288    -86       O
ATOM    657  CB  ALA A  42       8.839  18.622  14.029  1.00 16.23           C  0.023
ANISOU  657  CB  ALA A  42     2271   2521   1376    116   -153   -301       C
ATOM    658  H   ALA A  42       7.915  16.420  13.630  1.00 18.56           H  0.025
ATOM    659  HA  ALA A  42      10.366  17.224  13.715  1.00 19.14           H  0.025
ATOM    660  HB1 ALA A  42       9.431  19.353  14.267  1.00 19.47           H  0.025
ATOM    661  HB2 ALA A  42       8.597  18.685  13.091  1.00 19.47           H  0.025
ATOM    662  HB3 ALA A  42       8.041  18.650  14.579  1.00 19.47           H  0.025
ATOM    663  N   THR A  43      11.330  17.453  15.943  1.00 15.96           N  0.023
ANISOU  663  N   THR A  43     1921   2776   1366    157   -185   -383       N
ATOM    664  CA  THR A  43      11.899  17.601  17.277  1.00 16.51           C  0.023
ANISOU  664  CA  THR A  43     2009   2897   1366    206    -93   -192       C
ATOM    665  C   THR A  43      12.857  18.782  17.281  1.00 16.06           C  0.023
ANISOU  665  C   THR A  43     2038   2985   1077     54     46    -22       C
ATOM    666  O   THR A  43      13.554  19.059  16.289  1.00 18.89           O  0.025
ANISOU  666  O   THR A  43     2350   3560   1265   -307    -99   -269       O
ATOM    667  CB  THR A  43      12.652  16.371  17.761  1.00 17.83           C  0.024
ANISOU  667  CB  THR A  43     2011   3022   1741    392      9   -287       C
ATOM    668  OG1 THR A  43      13.755  16.114  16.879  1.00 17.67           O  0.024
ANISOU  668  OG1 THR A  43     1855   3169   1690    403   -162   -276       O
ATOM    669  CG2 THR A  43      11.722  15.160  17.883  1.00 18.05           C  0.024
ANISOU  669  CG2 THR A  43     2257   2879   1723    576     10   -126       C
ATOM    670  H   THR A  43      11.908  17.539  15.312  1.00 19.14           H  0.025
ATOM    671  HA  THR A  43      11.169  17.756  17.897  1.00 19.80           H  0.025
ATOM    672  HB  THR A  43      13.005  16.525  18.651  1.00 21.38           H  0.026
ATOM    673  HG1 THR A  43      13.473  15.977  16.100  1.00 21.19           H  0.026
ATOM    674 HG21 THR A  43      12.221  14.389  18.196  1.00 21.65           H  0.026
ATOM    675 HG22 THR A  43      11.010  15.351  18.514  1.00 21.65           H  0.026
ATOM    676 HG23 THR A  43      11.331  14.953  17.020  1.00 21.65           H  0.026
ATOM    677  N  AASN A  44      12.990  19.419  18.432  0.40 15.16           N  0.022
ANISOU  677  N  AASN A  44     1944   2780   1037    100     56   -108       N
ATOM    678  CA AASN A  44      13.984  20.477  18.527  0.40 15.15           C  0.022
ANISOU  678  CA AASN A  44     1987   2785    983     78    111   -271       C
ATOM    679  C  AASN A  44      14.469  20.582  19.962  0.40 14.56           C  0.022
ANISOU  679  C  AASN A  44     2009   2628    895    129    295   -193       C
ATOM    680  O  AASN A  44      13.675  20.812  20.878  0.40 13.96           O  0.021
ANISOU  680  O  AASN A  44     1833   2578    894    171    486    -39       O
ATOM    681  CB AASN A  44      13.415  21.786  17.987  0.40 17.56           C  0.024
ANISOU  681  CB AASN A  44     2136   2964   1573     25     17   -212       C
ATOM    682  CG AASN A  44      13.224  21.745  16.475  0.40 18.11           C  0.024
```

```
ANISOU  682  CG AASN A  44     2189   3016   1677    -51    384   -192       C
ATOM    683  OD1AASN A  44      14.185  21.853  15.709  0.40 19.08           O  0.025
ANISOU  683  OD1AASN A  44     2278   3333   1640   -305    300   -270       O
ATOM    684  ND2AASN A  44      11.975  21.581  16.041  0.40 19.01           N  0.025
ANISOU  684  ND2AASN A  44     2281   2823   2118    -84    603   -127       N
ATOM    685  H  AASN A  44      12.535  19.264  19.145  0.40 18.18           H  0.024
ATOM    686  HA AASN A  44      14.766  20.258  17.997  0.40 18.17           H  0.024
ATOM    687  HB2AASN A  44      12.552  21.952  18.397  0.40 21.06           H  0.026
ATOM    688  HB3AASN A  44      14.027  22.509  18.195  0.40 21.06           H  0.026
ATOM    689 HD21AASN A  44      11.330  21.505  16.605  0.40 22.80           H  0.027
ATOM    690 HD22AASN A  44      11.813  21.551  15.197  0.40 22.80           H  0.027
ATOM    691  N  BASN A  44      12.916  19.463  18.423  0.60 15.67           N  0.023
ANISOU  691  N  BASN A  44     2105   2695   1154     27    -32     96       N
ATOM    692  CA BASN A  44      13.808  20.604  18.585  0.60 16.69           C  0.023
ANISOU  692  CA BASN A  44     2397   2695   1251    -90    -11     81       C
ATOM    693  C  BASN A  44      14.380  20.558  19.992  0.60 15.80           C  0.023
ANISOU  693  C  BASN A  44     2413   2524   1068    -54    -69    182       C
ATOM    694  O  BASN A  44      13.628  20.625  20.971  0.60 15.64           O  0.023
ANISOU  694  O  BASN A  44     2308   2506   1128    103   -188    258       O
ATOM    695  CB BASN A  44      13.086  21.935  18.342  0.60 20.89           C  0.026
ANISOU  695  CB BASN A  44     2780   2931   2226   -172    -24    272       C
ATOM    696  CG BASN A  44      13.990  23.113  18.600  0.60 24.07           C  0.028
ANISOU  696  CG BASN A  44     3207   3038   2899   -263    165    243       C
ATOM    697  OD1BASN A  44      14.833  23.443  17.776  0.60 25.62           O  0.029
ANISOU  697  OD1BASN A  44     3362   2982   3392   -497    439    390       O
ATOM    698  ND2BASN A  44      13.795  23.787  19.730  0.60 25.24           N  0.029
ANISOU  698  ND2BASN A  44     3423   3097   3071   -191    254    222       N
ATOM    699  H  BASN A  44      12.447  19.281  19.120  0.60 18.79           H  0.025
ATOM    700  HA BASN A  44      14.533  20.540  17.944  0.60 20.02           H  0.025
ATOM    701  HB2BASN A  44      12.789  21.975  17.419  0.60 25.05           H  0.028
ATOM    702  HB3BASN A  44      12.323  22.000  18.938  0.60 25.05           H  0.028
ATOM    703 HD21BASN A  44      13.175  23.544  20.274  0.60 30.28           H  0.031
ATOM    704 HD22BASN A  44      14.289  24.466  19.916  0.60 30.28           H  0.031
ATOM    705  N  AARG A  45      15.783  20.415  20.140  0.40 15.02           N  0.022
ANISOU  705  N  AARG A  45     2177   2559    970    192    279   -286       N
ATOM    706  CA AARG A  45      16.396  20.485  21.459  0.40 16.16           C  0.023
ANISOU  706  CA AARG A  45     2503   2567   1072    157     -3   -131       C
ATOM    707  C  AARG A  45      16.637  21.940  21.818  0.40 17.64           C  0.024
ANISOU  707  C  AARG A  45     2982   2504   1218   -160    -55    178       C
ATOM    708  O  AARG A  45      17.256  22.688  21.049  0.40 19.13           O  0.025
ANISOU  708  O  AARG A  45     3097   2564   1607   -110    203     56       O
ATOM    709  CB AARG A  45      17.728  19.718  21.474  0.40 17.36           C  0.024
ANISOU  709  CB AARG A  45     2494   2805   1297    289   -436   -173       C
ATOM    710  CG AARG A  45      18.356  19.521  22.874  0.40 18.88           C  0.025
ANISOU  710  CG AARG A  45     2593   2944   1636    352   -500   -228       C
ATOM    711  CD AARG A  45      18.418  18.089  23.286  0.40 19.58           C  0.025
ANISOU  711  CD AARG A  45     2608   3081   1751    399   -376   -201       C
ATOM    712  NE AARG A  45      18.621  17.934  24.723  0.40 19.78           N  0.025
ANISOU  712  NE AARG A  45     2541   3081   1894    348   -358   -287       N
ATOM    713  CZ AARG A  45      19.324  16.958  25.269  0.40 19.37           C  0.025
ANISOU  713  CZ AARG A  45     2416   3137   1808    387   -354   -383       C
ATOM    714  NH1AARG A  45      20.041  16.138  24.527  0.40 18.30           N  0.024
ANISOU  714  NH1AARG A  45     2273   3096   1583    582    -38   -431       N
ATOM    715  NH2AARG A  45      19.277  16.779  26.587  0.40 19.73           N  0.025
ANISOU  715  NH2AARG A  45     2404   3185   1907    356   -484   -253       N
ATOM    716  H  AARG A  45      16.341  20.259  19.505  0.40 18.01           H  0.024
ATOM    717  HA AARG A  45      15.807  20.084  22.118  0.40 19.38           H  0.025
ATOM    718  HB2AARG A  45      17.579  18.838  21.095  0.40 20.82           H  0.026
ATOM    719  HB3AARG A  45      18.369  20.207  20.934  0.40 20.82           H  0.026
ATOM    720  HG2AARG A  45      19.262  19.869  22.866  0.40 22.64           H  0.027
ATOM    721  HG3AARG A  45      17.824  19.998  23.529  0.40 22.64           H  0.027
ATOM    722  HD2AARG A  45      17.583  17.654  23.051  0.40 23.49           H  0.028
ATOM    723  HD3AARG A  45      19.157  17.657  22.829  0.40 23.49           H  0.028
ATOM    724  HE AARG A  45      18.260  18.514  25.246  0.40 23.73           H  0.028
ATOM    725 HH11AARG A  45      20.057  16.234  23.672  0.40 21.95           H  0.027
ATOM    726 HH12AARG A  45      20.494  15.508  24.897  0.40 21.95           H  0.027
ATOM    727 HH21AARG A  45      18.792  17.296  27.074  0.40 23.66           H  0.028
ATOM    728 HH22AARG A  45      19.731  16.147  26.952  0.40 23.66           H  0.028
ATOM    729  N  BARG A  45      15.713  20.503  20.087  0.60 15.97           N  0.023
ANISOU  729  N  BARG A  45     2530   2418   1118    -75    -11    150       N
ATOM    730  CA BARG A  45      16.397  20.543  21.375  0.60 16.77           C  0.023
ANISOU  730  CA BARG A  45     2815   2421   1137   -182   -136    336       C
ATOM    731  C  BARG A  45      16.591  21.992  21.778  0.60 18.05           C  0.024
ANISOU  731  C  BARG A  45     3231   2440   1186   -426   -114    392       C
ATOM    732  O  BARG A  45      17.108  22.802  20.990  0.60 20.18           O  0.026
ANISOU  732  O  BARG A  45     3548   2540   1581   -493    127    227       O
ATOM    733  CB BARG A  45      17.754  19.835  21.232  0.60 19.30           C  0.025
ANISOU  733  CB BARG A  45     2883   2650   1799   -199   -439    283       C
```

```
ATOM    734  CG BARG A  45      18.667  19.872  22.445  0.60 21.01           C  0.026
ANISOU  734  CG BARG A  45     3040   2693   2247   -263   -624    307       C
ATOM    735  CD BARG A  45      18.139  19.009  23.563  0.60 19.69           C  0.025
ANISOU  735  CD BARG A  45     3091   2362   2027   -500   -612    275       C
ATOM    736  NE BARG A  45      17.585  17.729  23.097  0.60 21.11           N  0.026
ANISOU  736  NE BARG A  45     3130   2414   2478   -532   -622    257       N
ATOM    737  CZ BARG A  45      18.300  16.635  22.902  0.60 23.10           C  0.027
ANISOU  737  CZ BARG A  45     3298   2546   2933   -504   -485    351       C
ATOM    738  NH1BARG A  45      19.618  16.630  23.076  0.60 24.23           N  0.028
ANISOU  738  NH1BARG A  45     3348   2579   3280   -369   -306    185       N
ATOM    739  NH2BARG A  45      17.680  15.521  22.529  0.60 23.93           N  0.028
ANISOU  739  NH2BARG A  45     3358   2680   3054   -496   -484    312       N
ATOM    740  H  BARG A  45      16.244  20.442  19.413  0.60 19.15           H  0.025
ATOM    741  HA BARG A  45      15.883  20.096  22.065  0.60 20.12           H  0.026
ATOM    742  HB2BARG A  45      17.586  18.901  21.031  0.60 23.14           H  0.027
ATOM    743  HB3BARG A  45      18.235  20.251  20.500  0.60 23.14           H  0.027
ATOM    744  HG2BARG A  45      19.545  19.544  22.196  0.60 25.19           H  0.029
ATOM    745  HG3BARG A  45      18.733  20.784  22.769  0.60 25.19           H  0.029
ATOM    746  HD2BARG A  45      18.863  18.813  24.178  0.60 23.61           H  0.028
ATOM    747  HD3BARG A  45      17.432  19.488  24.024  0.60 23.61           H  0.028
ATOM    748  HE BARG A  45      16.740  17.690  22.941  0.60 25.33           H  0.029
ATOM    749 HH11BARG A  45      20.022  17.349  23.320  0.60 29.07           H  0.031
ATOM    750 HH12BARG A  45      20.067  15.908  22.945  0.60 29.07           H  0.031
ATOM    751 HH21BARG A  45      16.827  15.521  22.417  0.60 28.70           H  0.031
ATOM    752 HH22BARG A  45      18.131  14.801  22.398  0.60 28.70           H  0.031
ATOM    753  N   ASN A  46      16.161  22.322  22.990  1.00 18.67           N  0.025
ANISOU  753  N   ASN A  46     3305   2426   1362   -373   -267     25       N
ATOM    754  CA  ASN A  46      16.287  23.669  23.518  1.00 21.72           C  0.027
ANISOU  754  CA  ASN A  46     3851   2590   1811   -404   -553   -191       C
ATOM    755  C   ASN A  46      17.616  23.799  24.244  1.00 24.29           C  0.028
ANISOU  755  C   ASN A  46     4214   2772   2244   -866   -939     67       C
ATOM    756  O   ASN A  46      18.218  22.805  24.662  1.00 25.19           O  0.029
ANISOU  756  O   ASN A  46     4096   2967   2508  -1046  -1031    259       O
ATOM    757  CB  ASN A  46      15.132  23.923  24.484  1.00 22.84           C  0.027
ANISOU  757  CB  ASN A  46     3947   2663   2066     -4   -494   -439       C
ATOM    758  CG  ASN A  46      13.791  23.736  23.813  1.00 24.30           C  0.028
ANISOU  758  CG  ASN A  46     4034   2881   2317    325   -429    -58       C
ATOM    759  OD1 ASN A  46      12.901  23.049  24.339  1.00 24.71           O  0.028
ANISOU  759  OD1 ASN A  46     4048   2999   2342    469   -782   -231       O
ATOM    760  ND2 ASN A  46      13.637  24.345  22.637  1.00 25.42           N  0.029
ANISOU  760  ND2 ASN A  46     4108   2967   2582    500   -489    227       N
ATOM    761  HA  ASN A  46      16.259  24.332  22.811  1.00 26.05           H  0.029
ATOM    762  HB2 ASN A  46      15.191  23.299  25.225  1.00 27.39           H  0.030
ATOM    763  HB3 ASN A  46      15.184  24.834  24.813  1.00 27.39           H  0.030
ATOM    764 HD21 ASN A  46      12.894  24.274  22.209  1.00 30.49           H  0.031
ATOM    765 HD22 ASN A  46      14.281  24.809  22.305  1.00 30.49           H  0.031
ATOM    766  H  AASN A  46      15.742  21.795  23.524  0.40 22.39           H  0.027
ATOM    767  H  BASN A  46      15.787  21.771  23.534  0.60 22.39           H  0.027
ATOM    768  N   THR A  47      18.102  25.041  24.352  1.00 27.73           N  0.030
ANISOU  768  N   THR A  47     4677   3109   2750  -1022   -833    134       N
ATOM    769  CA  THR A  47      19.417  25.255  24.944  1.00 30.71           C  0.032
ANISOU  769  CA  THR A  47     5026   3473   3169  -1064   -614    130       C
ATOM    770  C   THR A  47      19.475  24.787  26.393  1.00 30.52           C  0.031
ANISOU  770  C   THR A  47     4945   3648   3003  -1016   -764     51       C
ATOM    771  O   THR A  47      20.569  24.489  26.892  1.00 31.27           O  0.032
ANISOU  771  O   THR A  47     4911   3754   3215  -1060   -641     -6       O
ATOM    772  CB  THR A  47      19.824  26.732  24.843  1.00 34.76           C  0.034
ANISOU  772  CB  THR A  47     5400   3684   4122  -1073   -350     89       C
ATOM    773  OG1 THR A  47      18.881  27.569  25.517  1.00 36.60           O  0.034
ANISOU  773  OG1 THR A  47     5587   3662   4656  -1063   -209     27       O
ATOM    774  CG2 THR A  47      19.876  27.158  23.370  1.00 36.32           C  0.034
ANISOU  774  CG2 THR A  47     5531   3788   4481  -1099   -339    199       C
ATOM    775  H   THR A  47      17.698  25.755  24.094  1.00 33.26           H  0.033
ATOM    776  HA  THR A  47      20.068  24.743  24.439  1.00 36.84           H  0.035
ATOM    777  HB  THR A  47      20.696  26.843  25.253  1.00 41.70           H  0.037
ATOM    778  HG1 THR A  47      19.091  28.376  25.418  1.00 43.90           H  0.038
ATOM    779 HG21 THR A  47      20.139  28.089  23.303  1.00 43.57           H  0.038
ATOM    780 HG22 THR A  47      20.520  26.614  22.890  1.00 43.57           H  0.038
ATOM    781 HG23 THR A  47      19.004  27.046  22.962  1.00 43.57           H  0.038
ATOM    782  N   ASP A  48      18.334  24.710  27.071  1.00 28.25           N  0.030
ANISOU  782  N   ASP A  48     4837   3662   2236   -881   -853   -223       N
ATOM    783  CA  ASP A  48      18.256  24.224  28.448  1.00 26.59           C  0.029
ANISOU  783  CA  ASP A  48     4648   3509   1944   -622   -895   -157       C
ATOM    784  C   ASP A  48      18.196  22.715  28.574  1.00 25.94           C  0.029
ANISOU  784  C   ASP A  48     4460   3448   1948   -544   -771    -42       C
ATOM    785  O   ASP A  48      18.087  22.220  29.700  1.00 27.49           O  0.030
ANISOU  785  O   ASP A  48     4636   3553   2253   -590   -736    217       O
ATOM    786  CB  ASP A  48      17.057  24.886  29.132  1.00 27.37           C  0.030
```

```
ANISOU  786  CB  ASP A  48     4693   3686   2020   -221   -698   -344       C
ATOM    787  CG  ASP A  48      15.720  24.286  28.724  1.00 29.14           C  0.031
ANISOU  787  CG  ASP A  48     4821   3869   2381    162   -555   -545       C
ATOM    788  OD1 ASP A  48      15.654  23.456  27.796  1.00 27.77           O  0.030
ANISOU  788  OD1 ASP A  48     4785   3702   2066    191   -760   -590       O
ATOM    789  OD2 ASP A  48      14.711  24.746  29.318  1.00 30.56           O  0.031
ANISOU  789  OD2 ASP A  48     4930   4077   2605    441   -110   -693       O
ATOM    790  H   ASP A  48      17.570  24.939  26.748  1.00 33.89           H  0.033
ATOM    791  HA  ASP A  48      19.060  24.485  28.924  1.00 31.89           H  0.032
ATOM    792  HB2 ASP A  48      17.148  24.783  30.092  1.00 32.83           H  0.033
ATOM    793  HB3 ASP A  48      17.044  25.828  28.899  1.00 32.83           H  0.033
ATOM    794  N   GLY A  49      18.317  21.984  27.465  1.00 24.79           N  0.028
ANISOU  794  N   GLY A  49     3900   3297   2223   -470   -910   -190       N
ATOM    795  CA  GLY A  49      18.356  20.549  27.474  1.00 22.75           C  0.027
ANISOU  795  CA  GLY A  49     3271   3175   2199   -213  -1026    -69       C
ATOM    796  C   GLY A  49      17.009  19.880  27.348  1.00 19.90           C  0.025
ANISOU  796  C   GLY A  49     2822   2845   1895    -97   -852   -183       C
ATOM    797  O   GLY A  49      16.972  18.670  27.082  1.00 19.96           O  0.025
ANISOU  797  O   GLY A  49     2678   2910   1994    158   -504    -68       O
ATOM    798  H   GLY A  49      18.379  22.320  26.677  1.00 29.74           H  0.031
ATOM    799  HA2 GLY A  49      18.907  20.249  26.735  1.00 27.29           H  0.030
ATOM    800  HA3 GLY A  49      18.756  20.254  28.307  1.00 27.29           H  0.030
ATOM    801  N   SER A  50      15.901  20.609  27.547  1.00 17.68           N  0.024
ANISOU  801  N   SER A  50     2846   2556   1314    -58   -648   -193       N
ATOM    802  CA  SER A  50      14.599  20.062  27.174  1.00 16.89           C  0.023
ANISOU  802  CA  SER A  50     2716   2362   1338     21   -431    -69       C
ATOM    803  C   SER A  50      14.489  19.943  25.651  1.00 14.82           C  0.022
ANISOU  803  C   SER A  50     2271   2306   1056     -6   -371     78       C
ATOM    804  O   SER A  50      15.269  20.541  24.903  1.00 15.85           O  0.023
ANISOU  804  O   SER A  50     2419   2396   1209   -145   -276     65       O
ATOM    805  CB  SER A  50      13.467  20.935  27.719  1.00 17.92           C  0.024
ANISOU  805  CB  SER A  50     2996   2311   1503    336   -283   -275       C
ATOM    806  OG  SER A  50      13.413  22.165  27.049  1.00 18.92           O  0.025
ANISOU  806  OG  SER A  50     3229   2511   1448    537   -405   -202       O
ATOM    807  H   SER A  50      15.883  21.399  27.887  1.00 21.20           H  0.026
ATOM    808  HA  SER A  50      14.506  19.176  27.559  1.00 20.25           H  0.026
ATOM    809  HB2 SER A  50      12.624  20.471  27.594  1.00 21.50           H  0.026
ATOM    810  HB3 SER A  50      13.620  21.097  28.663  1.00 21.50           H  0.026
ATOM    811  HG  SER A  50      14.135  22.582  27.154  1.00 22.69           H  0.027
ATOM    812  N   THR A  51      13.461  19.202  25.203  1.00 13.78           N  0.021
ANISOU  812  N   THR A  51     1972   2164   1099    140   -160     42       N
ATOM    813  CA  THR A  51      13.206  18.978  23.781  1.00 13.16           C  0.021
ANISOU  813  CA  THR A  51     1894   2158    947    268    -23    -34       C
ATOM    814  C   THR A  51      11.718  19.166  23.531  1.00 12.72           C  0.020
ANISOU  814  C   THR A  51     1923   2088    822    259     65    -62       C
ATOM    815  O   THR A  51      10.887  18.845  24.385  1.00 12.78           O  0.020
ANISOU  815  O   THR A  51     1844   2285    726    373     67    -37       O
ATOM    816  CB  THR A  51      13.648  17.577  23.338  1.00 13.96           C  0.021
ANISOU  816  CB  THR A  51     1884   2375   1045    224    -21     -2       C
ATOM    817  OG1 THR A  51      15.043  17.436  23.643  1.00 14.91           O  0.022
ANISOU  817  OG1 THR A  51     2025   2631   1011    435    -19     68       O
ATOM    818  CG2 THR A  51      13.485  17.288  21.863  1.00 13.76           C  0.021
ANISOU  818  CG2 THR A  51     1863   2261   1103    305   -115   -251       C
ATOM    819  H   THR A  51      12.892  18.814  25.717  1.00 16.52           H  0.023
ATOM    820  HA  THR A  51      13.698  19.620  23.246  1.00 15.78           H  0.023
ATOM    821  HB  THR A  51      13.080  16.944  23.804  1.00 16.74           H  0.023
ATOM    822  HG1 THR A  51      15.155  17.414  24.475  1.00 17.88           H  0.024
ATOM    823 HG21 THR A  51      13.842  16.410  21.656  1.00 16.50           H  0.023
ATOM    824 HG22 THR A  51      12.546  17.311  21.622  1.00 16.50           H  0.023
ATOM    825 HG23 THR A  51      13.961  17.953  21.340  1.00 16.50           H  0.023
ATOM    826  N   ASP A  52      11.414  19.730  22.374  1.00 13.45           N  0.021
ANISOU  826  N   ASP A  52     1916   2272    922    136    -73     38       N
ATOM    827  CA  ASP A  52      10.056  19.927  21.895  1.00 13.01           C  0.021
ANISOU  827  CA  ASP A  52     1898   2069    976    241    -75    142       C
ATOM    828  C   ASP A  52       9.733  18.844  20.872  1.00 13.27           C  0.021
ANISOU  828  C   ASP A  52     1917   2205    922    208      6     44       C
ATOM    829  O   ASP A  52      10.540  18.559  19.986  1.00 13.79           O  0.021
ANISOU  829  O   ASP A  52     1877   2182   1178    165    140   -264       O
ATOM    830  CB  ASP A  52       9.926  21.277  21.194  1.00 14.94           C  0.022
ANISOU  830  CB  ASP A  52     2279   1978   1418    110   -201    199       C
ATOM    831  CG  ASP A  52      10.127  22.466  22.111  1.00 20.58           C  0.026
ANISOU  831  CG  ASP A  52     3242   2287   2291    232   -312     83       C
ATOM    832  OD1 ASP A  52       9.979  22.328  23.326  1.00 20.40           O  0.026
ANISOU  832  OD1 ASP A  52     3202   2421   2130    442    -40    -76       O
ATOM    833  OD2 ASP A  52      10.314  23.595  21.562  1.00 24.93           O  0.028
ANISOU  833  OD2 ASP A  52     4072   2465   2936    104   -367   -114       O
ATOM    834  H   ASP A  52      12.005  20.021  21.822  1.00 16.13           H  0.023
ATOM    835  HA  ASP A  52       9.439  19.885  22.642  1.00 15.60           H  0.022
```

```
ATOM    836  HB2 ASP A  52      10.593  21.329  20.492  1.00 17.91           H  0.024
ATOM    837  HB3 ASP A  52       9.036  21.346  20.813  1.00 17.91           H  0.024
ATOM    838  N   TYR A  53       8.527  18.287  20.965  1.00 13.03           N  0.021
ANISOU  838  N   TYR A  53     1763   2136   1053    201    -18    115       N
ATOM    839  CA  TYR A  53       8.174  17.137  20.145  1.00 12.66           C  0.020
ANISOU  839  CA  TYR A  53     1725   1965   1119    229   -231     33       C
ATOM    840  C   TYR A  53       6.851  17.318  19.418  1.00 12.01           C  0.020
ANISOU  840  C   TYR A  53     1776   1988    799    241    -64     45       C
ATOM    841  O   TYR A  53       5.847  17.733  20.023  1.00 13.50           O  0.021
ANISOU  841  O   TYR A  53     1734   2262   1134    301     -5    -67       O
ATOM    842  CB  TYR A  53       7.977  15.902  21.063  1.00 13.49           C  0.021
ANISOU  842  CB  TYR A  53     1803   2004   1319    225   -123    262       C
ATOM    843  CG  TYR A  53       9.240  15.472  21.780  1.00 13.47           C  0.021
ANISOU  843  CG  TYR A  53     1837   2002   1280    197   -370     56       C
ATOM    844  CD1 TYR A  53       9.641  16.064  22.966  1.00 13.67           C  0.021
ANISOU  844  CD1 TYR A  53     1869   2000   1327    197    -87    179       C
ATOM    845  CD2 TYR A  53      10.032  14.435  21.253  1.00 14.23           C  0.021
ANISOU  845  CD2 TYR A  53     2119   2136   1153    363   -106   -154       C
ATOM    846  CE1 TYR A  53      10.782  15.633  23.608  1.00 13.14           C  0.021
ANISOU  846  CE1 TYR A  53     1902   2024   1067    218     50    256       C
ATOM    847  CE2 TYR A  53      11.168  14.032  21.885  1.00 13.77           C  0.021
ANISOU  847  CE2 TYR A  53     2095   2157    978    350   -196   -108       C
ATOM    848  CZ  TYR A  53      11.558  14.621  23.025  1.00 12.93           C  0.020
ANISOU  848  CZ  TYR A  53     1899   2060    953    296   -105    149       C
ATOM    849  OH  TYR A  53      12.718  14.183  23.641  1.00 14.21           O  0.021
ANISOU  849  OH  TYR A  53     2011   2153   1235    473   -157     76       O
ATOM    850  H   TYR A  53       7.904  18.557  21.492  1.00 15.63           H  0.023
ATOM    851  HA  TYR A  53       8.877  17.031  19.485  1.00 15.18           H  0.022
ATOM    852  HB2 TYR A  53       7.311  16.115  21.735  1.00 16.18           H  0.023
ATOM    853  HB3 TYR A  53       7.675  15.156  20.521  1.00 16.18           H  0.023
ATOM    854  HD1 TYR A  53       9.138  16.755  23.332  1.00 16.40           H  0.023
ATOM    855  HD2 TYR A  53       9.775  14.019  20.462  1.00 17.07           H  0.024
ATOM    856  HE1 TYR A  53      11.034  16.010  24.420  1.00 15.76           H  0.023
ATOM    857  HE2 TYR A  53      11.677  13.343  21.523  1.00 16.51           H  0.023
ATOM    858  HH  TYR A  53      12.805  14.555  24.389  1.00 17.04           H  0.024
ATOM    859  N   GLY A  54       6.844  16.945  18.149  1.00 13.15           N  0.021
ANISOU  859  N   GLY A  54     1960   2008   1028    176   -131   -152       N
ATOM    860  CA  GLY A  54       5.626  16.754  17.397  1.00 13.13           C  0.021
ANISOU  860  CA  GLY A  54     2120   2085    785    343   -386   -302       C
ATOM    861  C   GLY A  54       5.056  17.981  16.738  1.00 14.33           C  0.022
ANISOU  861  C   GLY A  54     2079   2180   1185    254   -281   -266       C
ATOM    862  O   GLY A  54       5.684  19.032  16.658  1.00 14.02           O  0.021
ANISOU  862  O   GLY A  54     1977   2061   1290    206   -149   -208       O
ATOM    863  H   GLY A  54       7.555  16.791  17.691  1.00 15.77           H  0.023
ATOM    864  HA2 GLY A  54       5.796  16.103  16.698  1.00 15.75           H  0.023
ATOM    865  HA3 GLY A  54       4.947  16.405  17.995  1.00 15.75           H  0.023
ATOM    866  N   ILE A  55       3.849  17.803  16.215  1.00 14.42           N  0.022
ANISOU  866  N   ILE A  55     2066   2308   1105    210   -336     38       N
ATOM    867  CA  ILE A  55       3.202  18.793  15.358  1.00 16.14           C  0.023
ANISOU  867  CA  ILE A  55     2071   2472   1590    253   -465     63       C
ATOM    868  C   ILE A  55       3.001  20.128  16.084  1.00 14.68           C  0.022
ANISOU  868  C   ILE A  55     2026   2364   1186    296   -108     52       C
ATOM    869  O   ILE A  55       2.965  21.190  15.437  1.00 14.69           O  0.022
ANISOU  869  O   ILE A  55     2086   2359   1135    301    -33    169       O
ATOM    870  CB  ILE A  55       1.897  18.148  14.845  1.00 22.59           C  0.027
ANISOU  870  CB  ILE A  55     2387   3069   3127     73   -893     19       C
ATOM    871  CG1 ILE A  55       1.359  18.893  13.645  1.00 27.10           C  0.030
ANISOU  871  CG1 ILE A  55     2882   3247   4167    -29  -1178    171       C
ATOM    872  CG2 ILE A  55       0.858  17.981  15.972  1.00 22.57           C  0.027
ANISOU  872  CG2 ILE A  55     1947   3204   3423     86   -953   -458       C
ATOM    873  CD1 ILE A  55       0.901  18.036  12.525  1.00 28.36           C  0.030
ANISOU  873  CD1 ILE A  55     3041   3268   4466   -117  -1078    152       C
ATOM    874  H   ILE A  55       3.369  17.101  16.342  1.00 17.29           H  0.024
ATOM    875  HA  ILE A  55       3.761  19.021  14.599  1.00 19.36           H  0.025
ATOM    876  HB  ILE A  55       2.106  17.251  14.543  1.00 27.09           H  0.030
ATOM    877 HG12 ILE A  55       0.602  19.428  13.930  1.00 32.50           H  0.032
ATOM    878 HG13 ILE A  55       2.061  19.468  13.302  1.00 32.50           H  0.032
ATOM    879 HG21 ILE A  55       0.051  17.590  15.602  1.00 27.07           H  0.030
ATOM    880 HG22 ILE A  55       1.225  17.399  16.656  1.00 27.07           H  0.030
ATOM    881 HG23 ILE A  55       0.660  18.852  16.350  1.00 27.07           H  0.030
ATOM    882 HD11 ILE A  55       0.562  18.602  11.813  1.00 34.02           H  0.033
ATOM    883 HD12 ILE A  55       1.649  17.511  12.202  1.00 34.02           H  0.033
ATOM    884 HD13 ILE A  55       0.198  17.449  12.844  1.00 34.02           H  0.033
ATOM    885  N   LEU A  56       2.852  20.098  17.424  1.00 13.87           N  0.021
ANISOU  885  N   LEU A  56     1861   2200   1211    226   -183   -211       N
ATOM    886  CA  LEU A  56       2.721  21.301  18.234  1.00 13.84           C  0.021
ANISOU  886  CA  LEU A  56     1893   2169   1197    342    -64     55       C
ATOM    887  C   LEU A  56       3.907  21.510  19.190  1.00 14.59           C  0.022
```

```
ANISOU  887  C   LEU A  56     1981   2060   1504    418    -89   -264       C
ATOM    888  O   LEU A  56       3.830  22.360  20.101  1.00 15.58           O  0.022
ANISOU  888  O   LEU A  56     2117   2143   1659    518   -175   -161       O
ATOM    889  CB  LEU A  56       1.392  21.284  18.997  1.00 14.92           C  0.022
ANISOU  889  CB  LEU A  56     1972   2342   1354    279     32      8       C
ATOM    890  CG  LEU A  56       0.159  21.534  18.089  1.00 15.65           C  0.023
ANISOU  890  CG  LEU A  56     2078   2480   1389    352     61    207       C
ATOM    891  CD1 LEU A  56      -1.094  21.209  18.891  1.00 17.64           C  0.024
ANISOU  891  CD1 LEU A  56     2094   2735   1875    404    265    283       C
ATOM    892  CD2 LEU A  56       0.117  22.964  17.564  1.00 16.47           C  0.023
ANISOU  892  CD2 LEU A  56     2276   2531   1453    708     -3    220       C
ATOM    893  HA  LEU A  56       2.713  22.068  17.640  1.00 16.60           H  0.023
ATOM    894  HB2 LEU A  56       1.283  20.416  19.417  1.00 17.89           H  0.024
ATOM    895  HB3 LEU A  56       1.410  21.979  19.673  1.00 17.89           H  0.024
ATOM    896  HG  LEU A  56       0.207  20.964  17.306  1.00 18.77           H  0.025
ATOM    897 HD11 LEU A  56      -1.875  21.397  18.347  1.00 21.16           H  0.026
ATOM    898 HD12 LEU A  56      -1.079  20.270  19.134  1.00 21.16           H  0.026
ATOM    899 HD13 LEU A  56      -1.108  21.757  19.691  1.00 21.16           H  0.026
ATOM    900 HD21 LEU A  56      -0.725  23.107  17.103  1.00 19.76           H  0.025
ATOM    901 HD22 LEU A  56       0.193  23.577  18.311  1.00 19.76           H  0.025
ATOM    902 HD23 LEU A  56       0.856  23.097  16.950  1.00 19.76           H  0.025
ATOM    903  H  ALEU A  56       2.825  19.372  17.885  0.36 16.64           H  0.023
ATOM    904  H  BLEU A  56       2.825  19.372  17.885  0.64 16.64           H  0.023
ATOM    905  N   GLN A  57       5.025  20.835  18.960  1.00 13.79           N  0.021
ANISOU  905  N   GLN A  57     1970   2032   1237    231   -142   -162       N
ATOM    906  CA  GLN A  57       6.265  21.132  19.641  1.00 13.12           C  0.021
ANISOU  906  CA  GLN A  57     1791   2030   1164    307   -127   -134       C
ATOM    907  C   GLN A  57       6.072  21.271  21.146  1.00 14.04           C  0.021
ANISOU  907  C   GLN A  57     1953   2161   1220    347    -33   -176       C
ATOM    908  O   GLN A  57       6.422  22.268  21.770  1.00 15.33           O  0.022
ANISOU  908  O   GLN A  57     2214   2465   1144    315   -288   -116       O
ATOM    909  CB  GLN A  57       6.938  22.358  19.032  1.00 12.80           C  0.020
ANISOU  909  CB  GLN A  57     1884   1978   1003    370    -25   -111       C
ATOM    910  CG  GLN A  57       7.427  22.088  17.602  1.00 14.30           C  0.022
ANISOU  910  CG  GLN A  57     1897   2099   1439    199     -4    -52       C
ATOM    911  CD  GLN A  57       8.629  21.155  17.597  1.00 14.66           C  0.022
ANISOU  911  CD  GLN A  57     2004   2039   1528    202    -75    -56       C
ATOM    912  OE1 GLN A  57       9.748  21.581  17.805  1.00 16.21           O  0.023
ANISOU  912  OE1 GLN A  57     2066   2148   1945     51   -280    102       O
ATOM    913  NE2 GLN A  57       8.392  19.883  17.359  1.00 14.12           N  0.021
ANISOU  913  NE2 GLN A  57     2001   2000   1365    394    -37     31       N
ATOM    914  H   GLN A  57       5.087  20.186  18.399  1.00 16.54           H  0.023
ATOM    915  HA  GLN A  57       6.860  20.375  19.517  1.00 15.73           H  0.023
ATOM    916  HB2 GLN A  57       6.302  23.089  19.004  1.00 15.35           H  0.022
ATOM    917  HB3 GLN A  57       7.703  22.604  19.575  1.00 15.35           H  0.022
ATOM    918  HG2 GLN A  57       6.715  21.673  17.091  1.00 17.15           H  0.024
ATOM    919  HG3 GLN A  57       7.688  22.925  17.188  1.00 17.15           H  0.024
ATOM    920 HE21 GLN A  57       7.588  19.615  17.214  1.00 16.94           H  0.023
ATOM    921 HE22 GLN A  57       9.042  19.320  17.346  1.00 16.94           H  0.023
ATOM    922  N   ILE A  58       5.563  20.197  21.731  1.00 14.07           N  0.021
ANISOU  922  N   ILE A  58     1982   2252   1113    248    -35     60       N
ATOM    923  CA  ILE A  58       5.275  20.133  23.168  1.00 14.45           C  0.022
ANISOU  923  CA  ILE A  58     2037   2490    965    219    -35    173       C
ATOM    924  C   ILE A  58       6.549  19.737  23.910  1.00 13.74           C  0.021
ANISOU  924  C   ILE A  58     1856   2570    796    414     40     70       C
ATOM    925  O   ILE A  58       7.290  18.845  23.482  1.00 15.60           O  0.022
ANISOU  925  O   ILE A  58     1998   2504   1425    513    -29   -123       O
ATOM    926  CB  ILE A  58       4.117  19.153  23.398  1.00 16.97           C  0.023
ANISOU  926  CB  ILE A  58     2124   2795   1528    228   -171    230       C
ATOM    927  CG1 ILE A  58       2.827  19.810  22.879  1.00 17.71           C  0.024
ANISOU  927  CG1 ILE A  58     2127   2919   1683    111     35    403       C
ATOM    928  CG2 ILE A  58       3.973  18.748  24.864  1.00 16.66           C  0.023
ANISOU  928  CG2 ILE A  58     2126   2811   1394    336    -58    272       C
ATOM    929  CD1 ILE A  58       1.689  18.891  22.834  1.00 18.60           C  0.025
ANISOU  929  CD1 ILE A  58     2191   3154   1720   -132    174    333       C
ATOM    930  H   ILE A  58       5.368  19.471  21.312  1.00 16.88           H  0.023
ATOM    931  HA  ILE A  58       5.013  21.002  23.513  1.00 17.33           H  0.024
ATOM    932  HB  ILE A  58       4.302  18.334  22.913  1.00 20.35           H  0.026
ATOM    933 HG12 ILE A  58       2.593  20.547  23.464  1.00 21.24           H  0.026
ATOM    934 HG13 ILE A  58       2.982  20.137  21.979  1.00 21.24           H  0.026
ATOM    935 HG21 ILE A  58       3.144  18.257  24.977  1.00 19.98           H  0.025
ATOM    936 HG22 ILE A  58       4.725  18.188  25.112  1.00 19.98           H  0.025
ATOM    937 HG23 ILE A  58       3.961  19.547  25.413  1.00 19.98           H  0.025
ATOM    938 HD11 ILE A  58       0.974  19.298  22.319  1.00 22.30           H  0.027
ATOM    939 HD12 ILE A  58       1.968  18.063  22.414  1.00 22.30           H  0.027
ATOM    940 HD13 ILE A  58       1.387  18.718  23.739  1.00 22.30           H  0.027
ATOM    941  N   ASN A  59       6.846  20.426  25.007  1.00 16.08           N  0.023
ANISOU  941  N   ASN A  59     2041   3076    992    510    -44    -60       N
```

```
ATOM    942  CA  ASN A  59       8.167  20.451  25.639  1.00 18.56           C  0.025
ANISOU  942  CA  ASN A  59     2281   3482   1290    531     98      5       C
ATOM    943  C   ASN A  59       8.255  19.475  26.799  1.00 18.09           C  0.024
ANISOU  943  C   ASN A  59     2047   3643   1182    435    380    154       C
ATOM    944  O   ASN A  59       7.338  19.348  27.614  1.00 20.44           O  0.026
ANISOU  944  O   ASN A  59     2153   4301   1313    783    141    494       O
ATOM    945  CB  ASN A  59       8.409  21.884  26.153  1.00 23.42           C  0.028
ANISOU  945  CB  ASN A  59     2618   3734   2546    521     90     97       C
ATOM    946  CG  ASN A  59       9.786  22.131  26.677  1.00 29.38           C  0.031
ANISOU  946  CG  ASN A  59     3236   4161   3764    376    -22    438       C
ATOM    947  OD1 ASN A  59      10.029  21.976  27.881  1.00 31.21           O  0.032
ANISOU  947  OD1 ASN A  59     3402   4274   4183    323   -313    606       O
ATOM    948  ND2 ASN A  59      10.679  22.559  25.820  1.00 32.61           N  0.033
ANISOU  948  ND2 ASN A  59     3467   4404   4520    469    -63    437       N
ATOM    949  H   ASN A  59       6.274  20.912  25.426  1.00 19.28           H  0.025
ATOM    950  HA  ASN A  59       8.849  20.182  25.003  1.00 22.26           H  0.027
ATOM    951  HB2 ASN A  59       8.259  22.504  25.423  1.00 28.09           H  0.030
ATOM    952  HB3 ASN A  59       7.786  22.063  26.874  1.00 28.09           H  0.030
ATOM    953 HD21 ASN A  59      10.460  22.685  24.998  1.00 39.12           H  0.036
ATOM    954 HD22 ASN A  59      11.484  22.715  26.080  1.00 39.12           H  0.036
ATOM    955  N   SER A  60       9.377  18.772  26.851  1.00 16.40           N  0.023
ANISOU  955  N   SER A  60     1961   3273    997    300    140    -81       N
ATOM    956  CA  SER A  60       9.653  17.806  27.888  1.00 16.47           C  0.023
ANISOU  956  CA  SER A  60     2158   3240    859     48    -65     98       C
ATOM    957  C   SER A  60       9.945  18.394  29.273  1.00 18.21           C  0.024
ANISOU  957  C   SER A  60     2261   3701    958     32   -180     66       C
ATOM    958  O   SER A  60      10.090  17.628  30.218  1.00 20.62           O  0.026
ANISOU  958  O   SER A  60     2641   4083   1109   -425   -142    126       O
ATOM    959  CB  SER A  60      10.850  16.965  27.434  1.00 16.65           C  0.023
ANISOU  959  CB  SER A  60     2143   2958   1225    140    112    401       C
ATOM    960  OG  SER A  60      12.010  17.781  27.358  1.00 15.57           O  0.022
ANISOU  960  OG  SER A  60     2080   2656   1179    279     84    245       O
ATOM    961  H   SER A  60      10.013  18.842  26.276  1.00 19.67           H  0.025
ATOM    962  HA  SER A  60       8.865  17.252  28.003  1.00 19.75           H  0.025
ATOM    963  HB2 SER A  60      11.001  16.252  28.073  1.00 19.97           H  0.025
ATOM    964  HB3 SER A  60      10.664  16.592  26.558  1.00 19.97           H  0.025
ATOM    965  HG  SER A  60      12.664  17.326  27.090  1.00 18.67           H  0.025
ATOM    966  N   ARG A  61      10.086  19.702  29.434  1.00 21.08           N  0.026
ANISOU  966  N   ARG A  61     2408   4189   1411    481    -56   -595       N
ATOM    967  CA  ARG A  61      10.295  20.232  30.787  1.00 24.04           C  0.028
ANISOU  967  CA  ARG A  61     2923   4564   1647    759    160   -909       C
ATOM    968  C   ARG A  61       9.037  20.085  31.627  1.00 22.65           C  0.027
ANISOU  968  C   ARG A  61     2798   4383   1425    973     52   -649       C
ATOM    969  O   ARG A  61       9.115  19.866  32.846  1.00 25.12           O  0.029
ANISOU  969  O   ARG A  61     2843   4934   1767   1256     56   -682       O
ATOM    970  CB  ARG A  61      10.709  21.703  30.728  1.00 28.64           C  0.030
ANISOU  970  CB  ARG A  61     3549   5147   2185    585    232  -1312       C
ATOM    971  CG  ARG A  61      11.089  22.372  32.069  1.00 33.75           C  0.033
ANISOU  971  CG  ARG A  61     4277   5663   2883    521    391  -1630       C
ATOM    972  CD  ARG A  61      11.579  21.414  33.144  1.00 37.18           C  0.035
ANISOU  972  CD  ARG A  61     4858   6092   3176    363    347  -1803       C
ATOM    973  NE  ARG A  61      11.675  22.074  34.450  1.00 40.65           N  0.036
ANISOU  973  NE  ARG A  61     5316   6448   3681    225    288  -1891       N
ATOM    974  CZ  ARG A  61      10.782  21.984  35.429  1.00 41.32           C  0.037
ANISOU  974  CZ  ARG A  61     5583   6610   3507    146    227  -2137       C
ATOM    975  NH1 ARG A  61       9.686  21.259  35.300  1.00 41.39           N  0.037
ANISOU  975  NH1 ARG A  61     5640   6665   3424    166    154  -2089       N
ATOM    976  NH2 ARG A  61      10.999  22.634  36.574  1.00 42.54           N  0.037
ANISOU  976  NH2 ARG A  61     5715   6701   3747    115    153  -2168       N
ATOM    977  H   ARG A  61      10.066  20.288  28.805  1.00 25.28           H  0.029
ATOM    978  HA  ARG A  61      11.015  19.738  31.208  1.00 28.83           H  0.031
ATOM    979  HB2 ARG A  61      11.483  21.773  30.147  1.00 34.35           H  0.033
ATOM    980  HB3 ARG A  61       9.969  22.209  30.358  1.00 34.35           H  0.033
ATOM    981  HG2 ARG A  61      11.799  23.013  31.904  1.00 40.49           H  0.036
ATOM    982  HG3 ARG A  61      10.307  22.827  32.419  1.00 40.49           H  0.036
ATOM    983  HD2 ARG A  61      10.958  20.673  33.222  1.00 44.60           H  0.038
ATOM    984  HD3 ARG A  61      12.459  21.084  32.904  1.00 44.60           H  0.038
ATOM    985  HE  ARG A  61      12.368  22.562  34.594  1.00 48.77           H  0.040
ATOM    986 HH11 ARG A  61       9.538  20.832  34.568  1.00 49.66           H  0.040
ATOM    987 HH12 ARG A  61       9.121  21.215  35.946  1.00 49.66           H  0.040
ATOM    988 HH21 ARG A  61      11.712  23.105  36.672  1.00 51.04           H  0.041
ATOM    989 HH22 ARG A  61      10.426  22.581  37.213  1.00 51.04           H  0.041
ATOM    990  N   TRP A  62       7.870  20.192  30.991  1.00 19.67           N  0.025
ANISOU  990  N   TRP A  62     2509   3604   1360    992    -33   -709       N
ATOM    991  CA  TRP A  62       6.626  20.184  31.741  1.00 20.11           C  0.026
ANISOU  991  CA  TRP A  62     2608   3284   1750    931    162   -284       C
ATOM    992  C   TRP A  62       5.690  19.056  31.359  1.00 17.75           C  0.024
ANISOU  992  C   TRP A  62     2454   2985   1305    910    302    -69       C
```

```
ATOM    993  O   TRP A  62       4.952  18.581  32.215  1.00 18.07           O  0.024
ANISOU  993  O   TRP A  62     2746   3115   1004    869    248     12       O
ATOM    994  CB  TRP A  62       5.904  21.531  31.539  1.00 21.57           C  0.026
ANISOU  994  CB  TRP A  62     2846   3342   2009    744    220   -501       C
ATOM    995  CG  TRP A  62       6.631  22.667  32.168  1.00 24.26           C  0.028
ANISOU  995  CG  TRP A  62     3314   3639   2264    498    172   -658       C
ATOM    996  CD1 TRP A  62       7.263  23.692  31.524  1.00 27.82           C  0.030
ANISOU  996  CD1 TRP A  62     3637   3917   3017    366     59   -860       C
ATOM    997  CD2 TRP A  62       6.758  22.924  33.569  1.00 27.52           C  0.030
ANISOU  997  CD2 TRP A  62     3698   3870   2887    394     -6   -773       C
ATOM    998  NE1 TRP A  62       7.793  24.573  32.451  1.00 29.30           N  0.031
ANISOU  998  NE1 TRP A  62     3826   3985   3323     88   -131   -787       N
ATOM    999  CE2 TRP A  62       7.502  24.111  33.711  1.00 28.96           C  0.031
ANISOU  999  CE2 TRP A  62     3911   4045   3048    201   -115   -840       C
ATOM   1000  CE3 TRP A  62       6.309  22.261  34.720  1.00 27.17           C  0.030
ANISOU 1000  CE3 TRP A  62     3907   3934   2480    368   -155   -873       C
ATOM   1001  CZ2 TRP A  62       7.818  24.649  34.965  1.00 30.10           C  0.031
ANISOU 1001  CZ2 TRP A  62     4055   4134   3247    145    -77   -735       C
ATOM   1002  CZ3 TRP A  62       6.637  22.795  35.969  1.00 29.41           C  0.031
ANISOU 1002  CZ3 TRP A  62     4082   4026   3066    240   -173   -762       C
ATOM   1003  CH2 TRP A  62       7.373  23.977  36.077  1.00 30.20           C  0.031
ANISOU 1003  CH2 TRP A  62     4082   4114   3277    197   -125   -642       C
ATOM   1004  H   TRP A  62       7.781  20.268  30.139  1.00 23.59           H  0.028
ATOM   1005  HA  TRP A  62       6.830  20.077  32.683  1.00 24.12           H  0.028
ATOM   1006  HB2 TRP A  62       5.829  21.711  30.589  1.00 25.88           H  0.029
ATOM   1007  HB3 TRP A  62       5.022  21.481  31.938  1.00 25.88           H  0.029
ATOM   1008  HD1 TRP A  62       7.328  23.784  30.600  1.00 33.37           H  0.033
ATOM   1009  HE1 TRP A  62       8.231  25.289  32.268  1.00 35.15           H  0.034
ATOM   1010  HE3 TRP A  62       5.804  21.483  34.653  1.00 32.59           H  0.033
ATOM   1011  HZ2 TRP A  62       8.313  25.433  35.042  1.00 36.11           H  0.034
ATOM   1012  HZ3 TRP A  62       6.360  22.356  36.741  1.00 35.28           H  0.034
ATOM   1013  HH2 TRP A  62       7.566  24.316  36.921  1.00 36.22           H  0.034
ATOM   1014  N   TRP A  63       5.638  18.661  30.077  1.00 15.30           N  0.022
ANISOU 1014  N   TRP A  63     2247   2635    930    600    304    240       N
ATOM   1015  CA  TRP A  63       4.450  18.002  29.533  1.00 14.84           C  0.022
ANISOU 1015  CA  TRP A  63     2173   2376   1090    467    294     31       C
ATOM   1016  C   TRP A  63       4.606  16.524  29.235  1.00 15.95           C  0.023
ANISOU 1016  C   TRP A  63     2079   2333   1649    533    222     83       C
ATOM   1017  O   TRP A  63       3.613  15.813  29.292  1.00 17.78           O  0.024
ANISOU 1017  O   TRP A  63     2175   2485   2096    380    388    -44       O
ATOM   1018  CB  TRP A  63       3.944  18.737  28.273  1.00 15.07           C  0.022
ANISOU 1018  CB  TRP A  63     2196   2190   1340    391    206     40       C
ATOM   1019  CG  TRP A  63       3.678  20.204  28.576  1.00 14.72           C  0.022
ANISOU 1019  CG  TRP A  63     2168   2154   1271    280    195     95       C
ATOM   1020  CD1 TRP A  63       4.377  21.274  28.136  1.00 16.53           C  0.023
ANISOU 1020  CD1 TRP A  63     2410   2227   1645    324    138     13       C
ATOM   1021  CD2 TRP A  63       2.656  20.705  29.440  1.00 15.12           C  0.022
ANISOU 1021  CD2 TRP A  63     2271   2302   1173    335    110     48       C
ATOM   1022  NE1 TRP A  63       3.839  22.424  28.652  1.00 16.59           N  0.023
ANISOU 1022  NE1 TRP A  63     2477   2230   1594    308      8    -80       N
ATOM   1023  CE2 TRP A  63       2.775  22.105  29.454  1.00 15.63           C  0.023
ANISOU 1023  CE2 TRP A  63     2359   2327   1254    312     -6     21       C
ATOM   1024  CE3 TRP A  63       1.621  20.103  30.163  1.00 16.64           C  0.023
ANISOU 1024  CE3 TRP A  63     2196   2423   1705    459    265    -20       C
ATOM   1025  CZ2 TRP A  63       1.937  22.910  30.196  1.00 17.30           C  0.024
ANISOU 1025  CZ2 TRP A  63     2462   2425   1685    327   -128   -240       C
ATOM   1026  CZ3 TRP A  63       0.776  20.930  30.893  1.00 18.04           C  0.024
ANISOU 1026  CZ3 TRP A  63     2299   2569   1986    543    320   -122       C
ATOM   1027  CH2 TRP A  63       0.954  22.307  30.902  1.00 18.26           C  0.024
ANISOU 1027  CH2 TRP A  63     2297   2622   2019    593    289   -215       C
ATOM   1028  H   TRP A  63       6.276  18.765  29.510  1.00 18.34           H  0.024
ATOM   1029  HA  TRP A  63       3.751  18.065  30.203  1.00 17.80           H  0.024
ATOM   1030  HB2 TRP A  63       4.615  18.683  27.575  1.00 18.07           H  0.024
ATOM   1031  HB3 TRP A  63       3.117  18.329  27.970  1.00 18.07           H  0.024
ATOM   1032  HD1 TRP A  63       5.112  21.235  27.569  1.00 19.83           H  0.025
ATOM   1033  HE1 TRP A  63       4.125  23.220  28.497  1.00 19.89           H  0.025
ATOM   1034  HE3 TRP A  63       1.502  19.180  30.156  1.00 19.96           H  0.025
ATOM   1035  HZ2 TRP A  63       2.045  23.834  30.211  1.00 20.74           H  0.026
ATOM   1036  HZ3 TRP A  63       0.080  20.555  31.384  1.00 21.63           H  0.026
ATOM   1037  HH2 TRP A  63       0.378  22.832  31.409  1.00 21.90           H  0.027
ATOM   1038  N   CYS A  64       5.787  16.054  28.878  1.00 16.08           N  0.023
ANISOU 1038  N   CYS A  64     2013   2252   1846    498    572    309       N
ATOM   1039  CA  CYS A  64       5.968  14.660  28.498  1.00 16.11           C  0.023
ANISOU 1039  CA  CYS A  64     2251   2143   1728    535    476    402       C
ATOM   1040  C   CYS A  64       7.295  14.180  29.047  1.00 16.14           C  0.023
ANISOU 1040  C   CYS A  64     2271   2045   1818    524    435    133       C
ATOM   1041  O   CYS A  64       8.157  14.984  29.404  1.00 16.49           O  0.023
ANISOU 1041  O   CYS A  64     2462   2059   1744    483    479    243       O
```

```
ATOM   1042  CB  CYS A  64       5.913  14.491  26.969  1.00 16.42           C  0.023
ANISOU 1042  CB  CYS A  64     2294   2230   1714    541    450    283       C
ATOM   1043  SG  CYS A  64       7.182  15.354  26.042  1.00 16.35           S  0.023
ANISOU 1043  SG  CYS A  64     2237   2190   1785    342    398    278       S
ATOM   1044  H   CYS A  64       6.506  16.524  28.847  1.00 19.29           H  0.025
ATOM   1045  HA  CYS A  64       5.269  14.109  28.883  1.00 19.32           H  0.025
ATOM   1046  HB2 CYS A  64       5.998  13.547  26.764  1.00 19.69           H  0.025
ATOM   1047  HB3 CYS A  64       5.055  14.821  26.658  1.00 19.69           H  0.025
ATOM   1048  N   ASN A  65       7.435  12.865  29.165  1.00 16.14           N  0.023
ANISOU 1048  N   ASN A  65     2268   2117   1747    565    422    241       N
ATOM   1049  CA  ASN A  65       8.679  12.289  29.651  1.00 16.18           C  0.023
ANISOU 1049  CA  ASN A  65     2236   2156   1755    466    403    430       C
ATOM   1050  C   ASN A  65       9.562  11.767  28.528  1.00 15.51           C  0.022
ANISOU 1050  C   ASN A  65     2144   2069   1681    480    136    311       C
ATOM   1051  O   ASN A  65       9.111  10.953  27.713  1.00 15.74           O  0.023
ANISOU 1051  O   ASN A  65     2104   2229   1645    308    318    161       O
ATOM   1052  CB  ASN A  65       8.445  11.113  30.580  1.00 17.90           C  0.024
ANISOU 1052  CB  ASN A  65     2436   2330   2036    461    559    462       C
ATOM   1053  CG  ASN A  65       9.745  10.509  30.992  1.00 19.62           C  0.025
ANISOU 1053  CG  ASN A  65     2695   2577   2181    567    520    593       C
ATOM   1054  OD1 ASN A  65      10.616  11.210  31.512  1.00 20.25           O  0.026
ANISOU 1054  OD1 ASN A  65     2867   2733   2094    749    391    692       O
ATOM   1055  ND2 ASN A  65       9.940   9.229  30.681  1.00 21.96           N  0.027
ANISOU 1055  ND2 ASN A  65     2924   2742   2678    702    357    510       N
ATOM   1056  H   ASN A  65       6.825  12.291  28.970  1.00 19.35           H  0.025
ATOM   1057  HA  ASN A  65       9.129  13.016  30.110  1.00 19.40           H  0.025
ATOM   1058  HB2 ASN A  65       7.978  11.414  31.375  1.00 21.47           H  0.026
ATOM   1059  HB3 ASN A  65       7.920  10.437  30.123  1.00 21.47           H  0.026
ATOM   1060 HD21 ASN A  65      10.675   8.840  30.899  1.00 26.34           H  0.029
ATOM   1061 HD22 ASN A  65       9.330   8.790  30.262  1.00 26.34           H  0.029
ATOM   1062  N   ASP A  66      10.814  12.247  28.465  1.00 14.67           N  0.022
ANISOU 1062  N   ASP A  66     2147   1965   1460    405    292    172       N
ATOM   1063  CA  ASP A  66      11.785  11.706  27.547  1.00 15.18           C  0.022
ANISOU 1063  CA  ASP A  66     2249   2027   1490    537    379     63       C
ATOM   1064  C   ASP A  66      12.976  11.097  28.271  1.00 15.76           C  0.023
ANISOU 1064  C   ASP A  66     2410   2132   1447    576    272    149       C
ATOM   1065  O   ASP A  66      13.891  10.630  27.594  1.00 16.51           O  0.023
ANISOU 1065  O   ASP A  66     2389   2106   1777    589    379     16       O
ATOM   1066  CB  ASP A  66      12.226  12.707  26.473  1.00 15.36           C  0.022
ANISOU 1066  CB  ASP A  66     2128   2138   1570    426    242    233       C
ATOM   1067  CG  ASP A  66      12.978  13.917  27.009  1.00 14.59           C  0.022
ANISOU 1067  CG  ASP A  66     2121   2284   1139    445    -65    162       C
ATOM   1068  OD1 ASP A  66      13.377  13.964  28.193  1.00 15.29           O  0.022
ANISOU 1068  OD1 ASP A  66     2152   2556   1100    352     94    281       O
ATOM   1069  OD2 ASP A  66      13.196  14.861  26.208  1.00 14.94           O  0.022
ANISOU 1069  OD2 ASP A  66     2081   2332   1264    274    -33    264       O
ATOM   1070  H   ASP A  66      11.111  12.890  28.952  1.00 17.59           H  0.024
ATOM   1071  HA  ASP A  66      11.355  11.003  27.034  1.00 18.20           H  0.024
ATOM   1072  HB2 ASP A  66      12.811  12.253  25.847  1.00 18.42           H  0.024
ATOM   1073  HB3 ASP A  66      11.437  13.034  26.012  1.00 18.42           H  0.024
ATOM   1074  N   GLY A  67      12.994  11.060  29.621  1.00 16.50           N  0.023
ANISOU 1074  N   GLY A  67     2522   2267   1478    715    322    329       N
ATOM   1075  CA  GLY A  67      14.074  10.459  30.385  1.00 17.12           C  0.024
ANISOU 1075  CA  GLY A  67     2406   2521   1576    568    102    446       C
ATOM   1076  C   GLY A  67      15.381  11.212  30.391  1.00 18.16           C  0.024
ANISOU 1076  C   GLY A  67     2422   2672   1805    700   -114    361       C
ATOM   1077  O   GLY A  67      16.346  10.731  30.998  1.00 19.77           O  0.025
ANISOU 1077  O   GLY A  67     2602   2942   1967    688   -474    235       O
ATOM   1078  H   GLY A  67      12.372  11.387  30.116  1.00 19.78           H  0.025
ATOM   1079  HA2 GLY A  67      13.785  10.373  31.307  1.00 20.53           H  0.026
ATOM   1080  HA3 GLY A  67      14.249   9.576  30.025  1.00 20.53           H  0.026
ATOM   1081  N  AARG A  68      15.457  12.371  29.757  0.52 17.84           N  0.024
ANISOU 1081  N  AARG A  68     2395   2709   1676    564   -113    362       N
ATOM   1082  CA AARG A  68      16.726  13.081  29.675  0.52 17.43           C  0.024
ANISOU 1082  CA AARG A  68     2326   2805   1491    454    -33    229       C
ATOM   1083  C  AARG A  68      16.560  14.575  29.946  0.52 17.98           C  0.024
ANISOU 1083  C  AARG A  68     2367   2837   1628    364    156    173       C
ATOM   1084  O  AARG A  68      17.388  15.375  29.490  0.52 20.26           O  0.026
ANISOU 1084  O  AARG A  68     2383   2949   2368    255    348   -154       O
ATOM   1085  CB AARG A  68      17.367  12.815  28.319  0.52 16.55           C  0.023
ANISOU 1085  CB AARG A  68     2207   2812   1270    381     12    101       C
ATOM   1086  CG AARG A  68      16.646  13.447  27.153  0.52 15.64           C  0.023
ANISOU 1086  CG AARG A  68     2085   2837   1021    378    269    115       C
ATOM   1087  CD AARG A  68      17.480  13.404  25.906  0.52 15.10           C  0.022
ANISOU 1087  CD AARG A  68     2065   2816    857    278    347    -66       C
ATOM   1088  NE AARG A  68      16.995  14.369  24.929  0.52 15.82           N  0.023
ANISOU 1088  NE AARG A  68     2062   2836   1113    304    317    -29       N
ATOM   1089  CZ AARG A  68      17.401  14.415  23.670  0.52 16.84           C  0.023
```

```
ANISOU 1089  CZ AARG A  68     2217   2813   1370    143    375    -81       C
ATOM   1090  NH1AARG A  68      18.321  13.595  23.227  0.52 16.91           N  0.023
ANISOU 1090  NH1AARG A  68     2334   2722   1368    101    383    -25       N
ATOM   1091  NH2AARG A  68      16.861  15.300  22.835  0.52 15.83           N  0.023
ANISOU 1091  NH2AARG A  68     2204   2810   1002    133    342   -179       N
ATOM   1092  H  AARG A  68      14.798  12.764  29.370  0.52 21.40           H  0.026
ATOM   1093  HA AARG A  68      17.340  12.759  30.353  0.52 20.90           H  0.026
ATOM   1094  HB2AARG A  68      18.271  13.167  28.330  0.52 19.85           H  0.025
ATOM   1095  HB3AARG A  68      17.385  11.857  28.168  0.52 19.85           H  0.025
ATOM   1096  HG2AARG A  68      15.821  12.965  26.985  0.52 18.76           H  0.025
ATOM   1097  HG3AARG A  68      16.452  14.375  27.359  0.52 18.76           H  0.025
ATOM   1098  HD2AARG A  68      18.400  13.622  26.124  0.52 18.11           H  0.024
ATOM   1099  HD3AARG A  68      17.433  12.518  25.514  0.52 18.11           H  0.024
ATOM   1100  HE AARG A  68      16.409  14.944  25.185  0.52 18.97           H  0.025
ATOM   1101 HH11AARG A  68      18.671  13.015  23.756  0.52 20.28           H  0.026
ATOM   1102 HH12AARG A  68      18.575  13.637  22.406  0.52 20.28           H  0.026
ATOM   1103 HH21AARG A  68      16.252  15.839  23.115  0.52 18.99           H  0.025
ATOM   1104 HH22AARG A  68      17.122  15.333  22.016  0.52 18.99           H  0.025
ATOM   1105  N  BARG A  68      15.457  12.368  29.743  0.48 18.34           N  0.024
ANISOU 1105  N  BARG A  68     2477   2684   1807    568   -151    370       N
ATOM   1106  CA BARG A  68      16.713  13.096  29.620  0.48 18.53           C  0.025
ANISOU 1106  CA BARG A  68     2494   2757   1788    458   -134    279       C
ATOM   1107  C  BARG A  68      16.556  14.584  29.932  0.48 18.46           C  0.024
ANISOU 1107  C  BARG A  68     2460   2817   1736    371    109    186       C
ATOM   1108  O  BARG A  68      17.389  15.391  29.500  0.48 20.51           O  0.026
ANISOU 1108  O  BARG A  68     2483   2922   2388    268    278   -123       O
ATOM   1109  CB BARG A  68      17.314  12.881  28.227  0.48 18.93           C  0.025
ANISOU 1109  CB BARG A  68     2546   2716   1930    383   -218    264       C
ATOM   1110  CG BARG A  68      16.417  13.336  27.082  0.48 19.38           C  0.025
ANISOU 1110  CG BARG A  68     2576   2678   2110    361   -180    404       C
ATOM   1111  CD BARG A  68      17.171  13.649  25.798  0.48 19.69           C  0.025
ANISOU 1111  CD BARG A  68     2651   2626   2204    308   -198    473       C
ATOM   1112  NE BARG A  68      17.649  15.029  25.817  0.48 20.28           N  0.026
ANISOU 1112  NE BARG A  68     2717   2638   2349    381   -285    600       N
ATOM   1113  CZ BARG A  68      18.910  15.396  26.009  0.48 21.29           C  0.026
ANISOU 1113  CZ BARG A  68     2741   2707   2640    426   -217    618       C
ATOM   1114  NH1BARG A  68      19.904  14.521  26.001  0.48 22.62           N  0.027
ANISOU 1114  NH1BARG A  68     2729   2766   3100    429    -95    458       N
ATOM   1115  NH2BARG A  68      19.181  16.669  26.229  0.48 21.23           N  0.026
ANISOU 1115  NH2BARG A  68     2746   2651   2670    493   -398    650       N
ATOM   1116  H  BARG A  68      14.790  12.755  29.363  0.48 22.00           H  0.027
ATOM   1117  HA BARG A  68      17.342  12.748  30.271  0.48 22.22           H  0.027
ATOM   1118  HB2BARG A  68      18.144  13.380  28.167  0.48 22.70           H  0.027
ATOM   1119  HB3BARG A  68      17.487  11.934  28.107  0.48 22.70           H  0.027
ATOM   1120  HG2BARG A  68      15.780  12.631  26.885  0.48 23.24           H  0.027
ATOM   1121  HG3BARG A  68      15.949  14.141  27.353  0.48 23.24           H  0.027
ATOM   1122  HD2BARG A  68      17.935  13.057  25.716  0.48 23.61           H  0.028
ATOM   1123  HD3BARG A  68      16.580  13.536  25.037  0.48 23.61           H  0.028
ATOM   1124  HE BARG A  68      17.069  15.651  25.694  0.48 24.32           H  0.028
ATOM   1125 HH11BARG A  68      19.742  13.686  25.870  0.48 27.13           H  0.030
ATOM   1126 HH12BARG A  68      20.712  14.787  26.129  0.48 27.13           H  0.030
ATOM   1127 HH21BARG A  68      18.545  17.248  26.247  0.48 25.46           H  0.029
ATOM   1128 HH22BARG A  68      19.994  16.920  26.355  0.48 25.46           H  0.029
ATOM   1129  N   THR A  69      15.504  14.980  30.668  1.00 16.74           N  0.023
ANISOU 1129  N   THR A  69     2463   2888   1010    444    182    157       N
ATOM   1130  CA  THR A  69      15.253  16.379  31.018  1.00 17.47           C  0.024
ANISOU 1130  CA  THR A  69     2509   3017   1113    434    -78    -28       C
ATOM   1131  C   THR A  69      15.173  16.458  32.533  1.00 18.22           C  0.024
ANISOU 1131  C   THR A  69     2729   3261    935    395     55    258       C
ATOM   1132  O   THR A  69      14.090  16.432  33.119  1.00 19.97           O  0.025
ANISOU 1132  O   THR A  69     2790   3695   1101    515    254    316       O
ATOM   1133  CB  THR A  69      13.992  16.902  30.336  1.00 16.56           C  0.023
ANISOU 1133  CB  THR A  69     2471   2832    989    427   -198     92       C
ATOM   1134  OG1 THR A  69      14.046  16.595  28.938  1.00 15.59           O  0.022
ANISOU 1134  OG1 THR A  69     2296   2783    844    532   -119    132       O
ATOM   1135  CG2 THR A  69      13.918  18.376  30.512  1.00 17.87           C  0.024
ANISOU 1135  CG2 THR A  69     2684   2877   1230    606   -110     95       C
ATOM   1136  HA  THR A  69      15.989  16.940  30.728  1.00 20.95           H  0.026
ATOM   1137  HB  THR A  69      13.202  16.492  30.723  1.00 19.86           H  0.025
ATOM   1138  HG1 THR A  69      14.041  15.763  28.825  1.00 18.69           H  0.025
ATOM   1139 HG21 THR A  69      13.117  18.721  30.086  1.00 21.43           H  0.026
ATOM   1140 HG22 THR A  69      13.892  18.597  31.456  1.00 21.43           H  0.026
ATOM   1141 HG23 THR A  69      14.694  18.797  30.111  1.00 21.43           H  0.026
ATOM   1142  H  ATHR A  69      14.906  14.443  30.974  0.52 20.08           H  0.026
ATOM   1143  H  BTHR A  69      14.913  14.439  30.981  0.48 20.08           H  0.026
ATOM   1144  N   PRO A  70      16.294  16.561  33.209  1.00 19.56           N  0.025
ANISOU 1144  N   PRO A  70     3073   3441    918    127    291     74       N
ATOM   1145  CA  PRO A  70      16.259  16.485  34.675  1.00 22.76           C  0.027
```

```
ANISOU 1145  CA  PRO A  70     3515   3764   1368     84    194    159       C
ATOM   1146  C   PRO A  70      15.329  17.514  35.286  1.00 26.33           C  0.029
ANISOU 1146  C   PRO A  70     4305   3862   1839    409    435    291       C
ATOM   1147  O   PRO A  70      15.167  18.637  34.790  1.00 27.87           O  0.030
ANISOU 1147  O   PRO A  70     4846   3756   1988    410    823     12       O
ATOM   1148  CB  PRO A  70      17.704  16.778  35.090  1.00 25.17           C  0.029
ANISOU 1148  CB  PRO A  70     3455   4013   2095   -176     31     96       C
ATOM   1149  CG  PRO A  70      18.489  16.364  33.922  1.00 23.38           C  0.028
ANISOU 1149  CG  PRO A  70     3299   3928   1658   -228     68    167       C
ATOM   1150  CD  PRO A  70      17.661  16.714  32.682  1.00 21.58           C  0.026
ANISOU 1150  CD  PRO A  70     3180   3754   1267   -177    143    181       C
ATOM   1151  HA  PRO A  70      15.993  15.592  34.946  1.00 27.30           H  0.030
ATOM   1152  HB2 PRO A  70      17.816  17.724  35.272  1.00 30.19           H  0.031
ATOM   1153  HB3 PRO A  70      17.940  16.259  35.875  1.00 30.19           H  0.031
ATOM   1154  HG2 PRO A  70      19.332  16.842  33.914  1.00 28.05           H  0.030
ATOM   1155  HG3 PRO A  70      18.650  15.408  33.964  1.00 28.05           H  0.030
ATOM   1156  HD2 PRO A  70      17.828  17.624  32.390  1.00 25.89           H  0.029
ATOM   1157  HD3 PRO A  70      17.832  16.095  31.955  1.00 25.89           H  0.029
ATOM   1158  N   GLY A  71      14.702  17.106  36.375  1.00 27.50           N  0.030
ANISOU 1158  N   GLY A  71     4425   4190   1835    717    606    312       N
ATOM   1159  CA  GLY A  71      13.901  18.006  37.147  1.00 29.31           C  0.031
ANISOU 1159  CA  GLY A  71     4512   4417   2207   1057    592    458       C
ATOM   1160  C   GLY A  71      12.577  18.294  36.518  1.00 29.20           C  0.031
ANISOU 1160  C   GLY A  71     4381   4605   2109   1559    453    353       C
ATOM   1161  O   GLY A  71      11.871  19.196  36.988  1.00 30.60           O  0.031
ANISOU 1161  O   GLY A  71     4630   4602   2395   1619    621     72       O
ATOM   1162  H   GLY A  71      14.731  16.303  36.683  1.00 32.99           H  0.033
ATOM   1163  HA2 GLY A  71      13.744  17.621  38.023  1.00 35.16           H  0.034
ATOM   1164  HA3 GLY A  71      14.377  18.845  37.251  1.00 35.16           H  0.034
ATOM   1165  N   SER A  72      12.220  17.578  35.453  1.00 29.02           N  0.031
ANISOU 1165  N   SER A  72     4189   4911   1925   1809    -61    577       N
ATOM   1166  CA  SER A  72      11.071  17.926  34.644  1.00 30.17           C  0.031
ANISOU 1166  CA  SER A  72     4047   5163   2254   1783   -170    237       C
ATOM   1167  C   SER A  72       9.825  17.200  35.141  1.00 30.13           C  0.031
ANISOU 1167  C   SER A  72     3792   5186   2472   1829     73    439       C
ATOM   1168  O   SER A  72       9.872  16.253  35.944  1.00 30.63           O  0.032
ANISOU 1168  O   SER A  72     3833   5359   2446   2108    427    346       O
ATOM   1169  CB  SER A  72      11.346  17.608  33.171  1.00 30.40           C  0.031
ANISOU 1169  CB  SER A  72     4042   5231   2279   1834   -489   -383       C
ATOM   1170  OG  SER A  72      11.361  16.207  32.939  1.00 29.86           O  0.031
ANISOU 1170  OG  SER A  72     4060   5194   2090   1909   -432  -1036       O
ATOM   1171  H   SER A  72      12.637  16.877  35.180  1.00 34.81           H  0.034
ATOM   1172  HA  SER A  72      10.895  18.878  34.710  1.00 36.20           H  0.034
ATOM   1173  HB2 SER A  72      10.649  18.008  32.628  1.00 36.47           H  0.034
ATOM   1174  HB3 SER A  72      12.210  17.975  32.927  1.00 36.47           H  0.034
ATOM   1175  N   ARG A  73       8.699  17.696  34.682  1.00 27.99           N  0.030
ANISOU 1175  N   ARG A  73     3656   4900   2078   1598     51    489       N
ATOM   1176  CA  ARG A  73       7.445  17.031  34.915  1.00 27.57           C  0.030
ANISOU 1176  CA  ARG A  73     3354   4574   2547   1322    128    362       C
ATOM   1177  C   ARG A  73       7.003  16.383  33.618  1.00 25.36           C  0.029
ANISOU 1177  C   ARG A  73     2905   4070   2661    966    417    515       C
ATOM   1178  O   ARG A  73       7.641  16.535  32.551  1.00 26.27           O  0.029
ANISOU 1178  O   ARG A  73     2635   3926   3421    779   -225    525       O
ATOM   1179  CB  ARG A  73       6.401  18.003  35.455  1.00 27.17           C  0.030
ANISOU 1179  CB  ARG A  73     3367   4708   2248   1582    543    226       C
ATOM   1180  CG  ARG A  73       6.875  18.743  36.722  1.00 28.89           C  0.031
ANISOU 1180  CG  ARG A  73     3527   4941   2508   1586    763    224       C
ATOM   1181  CD  ARG A  73       6.743  17.886  38.006  1.00 32.17           C  0.032
ANISOU 1181  CD  ARG A  73     3699   5247   3279   1530    672    440       C
ATOM   1182  NE  ARG A  73       5.368  17.817  38.510  1.00 33.75           N  0.033
ANISOU 1182  NE  ARG A  73     3771   5476   3574   1548    866    462       N
ATOM   1183  CZ  ARG A  73       4.707  18.817  39.075  1.00 35.27           C  0.034
ANISOU 1183  CZ  ARG A  73     3812   5648   3942   1492   1014    326       C
ATOM   1184  NH1 ARG A  73       5.293  19.973  39.340  1.00 36.38           N  0.034
ANISOU 1184  NH1 ARG A  73     3869   5777   4177   1487   1067    279       N
ATOM   1185  NH2 ARG A  73       3.421  18.659  39.363  1.00 35.20           N  0.034
ANISOU 1185  NH2 ARG A  73     3743   5699   3932   1445   1077    313       N
ATOM   1186  H   ARG A  73       8.638  18.424  34.228  1.00 33.57           H  0.033
ATOM   1187  HA  ARG A  73       7.542  16.339  35.587  1.00 33.07           H  0.033
ATOM   1188  HB2 ARG A  73       6.206  18.667  34.775  1.00 32.59           H  0.033
ATOM   1189  HB3 ARG A  73       5.596  17.510  35.679  1.00 32.59           H  0.033
ATOM   1190  HG2 ARG A  73       7.809  18.983  36.618  1.00 34.65           H  0.034
ATOM   1191  HG3 ARG A  73       6.338  19.542  36.841  1.00 34.65           H  0.034
ATOM   1192  HD2 ARG A  73       7.036  16.981  37.812  1.00 38.60           H  0.035
ATOM   1193  HD3 ARG A  73       7.297  18.272  38.701  1.00 38.60           H  0.035
ATOM   1194  HE  ARG A  73       4.957  17.066  38.433  1.00 40.48           H  0.036
ATOM   1195 HH11 ARG A  73       6.123  20.089  39.145  1.00 43.65           H  0.038
ATOM   1196 HH12 ARG A  73       4.845  20.608  39.707  1.00 43.65           H  0.038
```

```
ATOM   1197 HH21 ARG A  73       3.028  17.915  39.183  1.00 42.22           H  0.037
ATOM   1198 HH22 ARG A  73       2.980  19.300  39.729  1.00 42.22           H  0.037
ATOM   1199  N   ASN A  74       5.923  15.632  33.764  1.00 22.99           N  0.027
ANISOU 1199  N   ASN A  74     2546   3615   2576    722    671    831       N
ATOM   1200  CA  ASN A  74       5.270  14.876  32.719  1.00 19.64           C  0.025
ANISOU 1200  CA  ASN A  74     2504   3402   1556    777    589    886       C
ATOM   1201  C   ASN A  74       3.766  15.075  32.971  1.00 19.47           C  0.025
ANISOU 1201  C   ASN A  74     2375   3305   1719    734    685    563       C
ATOM   1202  O   ASN A  74       3.021  14.162  33.290  1.00 19.66           O  0.025
ANISOU 1202  O   ASN A  74     2425   3358   1686    847    715    712       O
ATOM   1203  CB  ASN A  74       5.652  13.404  32.797  1.00 20.45           C  0.026
ANISOU 1203  CB  ASN A  74     2668   3297   1805    787    666    808       C
ATOM   1204  CG  ASN A  74       4.982  12.559  31.769  1.00 21.30           C  0.026
ANISOU 1204  CG  ASN A  74     2685   3278   2129    831    663    804       C
ATOM   1205  OD1 ASN A  74       4.348  13.038  30.845  1.00 21.28           O  0.026
ANISOU 1205  OD1 ASN A  74     2673   3065   2347    759    691    798       O
ATOM   1206  ND2 ASN A  74       5.170  11.246  31.900  1.00 22.49           N  0.027
ANISOU 1206  ND2 ASN A  74     2759   3280   2507    981    263    899       N
ATOM   1207  H   ASN A  74       5.522  15.541  34.519  1.00 27.58           H  0.030
ATOM   1208  HA  ASN A  74       5.514  15.198  31.837  1.00 23.55           H  0.028
ATOM   1209  HB2 ASN A  74       6.610  13.322  32.670  1.00 24.53           H  0.028
ATOM   1210  HB3 ASN A  74       5.403  13.062  33.670  1.00 24.53           H  0.028
ATOM   1211 HD21 ASN A  74       4.811  10.702  31.339  1.00 26.98           H  0.030
ATOM   1212 HD22 ASN A  74       5.651  10.943  32.545  1.00 26.98           H  0.030
ATOM   1213  N   LEU A  75       3.304  16.314  32.811  1.00 18.93           N  0.025
ANISOU 1213  N   LEU A  75     2220   3268   1706    624    499    769       N
ATOM   1214  CA  LEU A  75       1.942  16.657  33.187  1.00 20.80           C  0.026
ANISOU 1214  CA  LEU A  75     2385   3399   2119    574    486    339       C
ATOM   1215  C   LEU A  75       0.916  16.066  32.232  1.00 19.91           C  0.025
ANISOU 1215  C   LEU A  75     2294   3311   1959    530    525    503       C
ATOM   1216  O   LEU A  75      -0.248  15.942  32.616  1.00 22.58           O  0.027
ANISOU 1216  O   LEU A  75     2348   3768   2465    498    644    297       O
ATOM   1217  CB  LEU A  75       1.824  18.176  33.309  1.00 21.33           C  0.026
ANISOU 1217  CB  LEU A  75     2498   3516   2091    484    431    -66       C
ATOM   1218  CG  LEU A  75       2.616  18.752  34.474  1.00 22.74           C  0.027
ANISOU 1218  CG  LEU A  75     2776   3630   2233    431    640   -275       C
ATOM   1219  CD1 LEU A  75       2.589  20.249  34.383  1.00 24.34           C  0.028
ANISOU 1219  CD1 LEU A  75     3023   3654   2572    421    454   -536       C
ATOM   1220  CD2 LEU A  75       1.961  18.259  35.762  1.00 24.25           C  0.028
ANISOU 1220  CD2 LEU A  75     3023   3825   2367    231    351   -266       C
ATOM   1221  H   LEU A  75       3.759  16.969  32.489  1.00 22.71           H  0.027
ATOM   1222  HA  LEU A  75       1.729  16.289  34.059  1.00 24.95           H  0.028
ATOM   1223  HB2 LEU A  75       2.156  18.581  32.493  1.00 25.59           H  0.029
ATOM   1224  HB3 LEU A  75       0.891  18.408  33.439  1.00 25.59           H  0.029
ATOM   1225  HG  LEU A  75       3.545  18.473  34.464  1.00 27.27           H  0.030
ATOM   1226 HD11 LEU A  75       3.415  20.603  34.748  1.00 29.20           H  0.031
ATOM   1227 HD12 LEU A  75       2.501  20.508  33.453  1.00 29.20           H  0.031
ATOM   1228 HD13 LEU A  75       1.833  20.583  34.892  1.00 29.20           H  0.031
ATOM   1229 HD21 LEU A  75       2.233  18.834  36.495  1.00 29.09           H  0.031
ATOM   1230 HD22 LEU A  75       0.997  18.288  35.658  1.00 29.09           H  0.031
ATOM   1231 HD23 LEU A  75       2.247  17.348  35.933  1.00 29.09           H  0.031
ATOM   1232  N   CYS A  76       1.299  15.694  31.018  1.00 18.56           N  0.025
ANISOU 1232  N   CYS A  76     2313   2921   1819    470    407    531       N
ATOM   1233  CA  CYS A  76       0.388  14.979  30.138  1.00 19.06           C  0.025
ANISOU 1233  CA  CYS A  76     2292   2848   2101    410    462    453       C
ATOM   1234  C   CYS A  76       0.424  13.465  30.301  1.00 19.36           C  0.025
ANISOU 1234  C   CYS A  76     2286   2895   2177    323    487    684       C
ATOM   1235  O   CYS A  76      -0.348  12.743  29.637  1.00 19.73           O  0.025
ANISOU 1235  O   CYS A  76     2215   2972   2310    290    460    774       O
ATOM   1236  CB  CYS A  76       0.684  15.361  28.688  1.00 17.41           C  0.024
ANISOU 1236  CB  CYS A  76     2258   2536   1822    303    292    402       C
ATOM   1237  SG  CYS A  76       0.179  17.074  28.344  1.00 17.19           S  0.024
ANISOU 1237  SG  CYS A  76     2176   2425   1931    242    140    244       S
ATOM   1238  H   CYS A  76       2.077  15.844  30.683  1.00 22.26           H  0.027
ATOM   1239  HA  CYS A  76      -0.519  15.250  30.349  1.00 22.86           H  0.027
ATOM   1240  HB2 CYS A  76       1.636  15.283  28.523  1.00 20.88           H  0.026
ATOM   1241  HB3 CYS A  76       0.194  14.772  28.094  1.00 20.88           H  0.026
ATOM   1242  N   ASN A  77       1.309  12.972  31.152  1.00 19.79           N  0.025
ANISOU 1242  N   ASN A  77     2327   2966   2227    359    662    840       N
ATOM   1243  CA  ASN A  77       1.417  11.544  31.463  1.00 20.49           C  0.026
ANISOU 1243  CA  ASN A  77     2497   2937   2350    296    703    992       C
ATOM   1244  C   ASN A  77       1.584  10.695  30.203  1.00 19.71           C  0.025
ANISOU 1244  C   ASN A  77     2473   2713   2305    297    723    813       C
ATOM   1245  O   ASN A  77       0.852   9.727  29.973  1.00 21.77           O  0.027
ANISOU 1245  O   ASN A  77     2621   2869   2781     77    860    454       O
ATOM   1246  CB  ASN A  77       0.207  11.101  32.289  1.00 23.22           C  0.027
ANISOU 1246  CB  ASN A  77     2925   3391   2505    179    835   1156       C
ATOM   1247  CG  ASN A  77       0.139  11.785  33.619  1.00 28.57           C  0.030
```

```
ANISOU 1247  CG  ASN A  77     3336   4084   3436    -19    861    863       C
ATOM   1248  OD1 ASN A  77       1.069  11.707  34.432  1.00 30.88          O  0.032
ANISOU 1248  OD1 ASN A  77     3684   4475   3575   -400   1008    705       O
ATOM   1249  ND2 ASN A  77      -0.920  12.538  33.819  1.00 31.72          N  0.032
ANISOU 1249  ND2 ASN A  77     3655   4313   4082    -18    818    618       N
ATOM   1250  H   ASN A  77       1.879  13.454  31.579  1.00 23.74          H  0.028
ATOM   1251  HA  ASN A  77       2.221  11.404  31.987  1.00 24.57          H  0.028
ATOM   1252  HB2 ASN A  77      -0.604  11.311  31.801  1.00 27.85          H  0.030
ATOM   1253  HB3 ASN A  77       0.263  10.146  32.445  1.00 27.85          H  0.030
ATOM   1254 HD21 ASN A  77      -1.517  12.613  33.204  1.00 38.05          H  0.035
ATOM   1255 HD22 ASN A  77      -1.016  12.957  34.564  1.00 38.05          H  0.035
ATOM   1256  N  AILE A  78       2.574  11.063  29.391  0.60 19.13          N  0.025
ANISOU 1256  N  AILE A  78     2382   2569   2318    300    781    598       N
ATOM   1257  CA AILE A  78       2.897  10.347  28.155  0.60 18.61          C  0.025
ANISOU 1257  CA AILE A  78     2328   2471   2272    244    710    399       C
ATOM   1258  C  AILE A  78       4.387  10.394  27.920  0.60 18.68          C  0.025
ANISOU 1258  C  AILE A  78     2374   2399   2326    225    540    377       C
ATOM   1259  O  AILE A  78       5.092  11.295  28.398  0.60 18.25          O  0.024
ANISOU 1259  O  AILE A  78     2367   2403   2164    405    531    345       O
ATOM   1260  CB AILE A  78       2.162  10.965  26.937  0.60 19.37          C  0.025
ANISOU 1260  CB AILE A  78     2399   2468   2491    183    497    410       C
ATOM   1261  CG1AILE A  78       2.306  12.496  26.907  0.60 20.14          C  0.026
ANISOU 1261  CG1AILE A  78     2427   2494   2732    199    348    227       C
ATOM   1262  CG2AILE A  78       0.698  10.564  26.945  0.60 19.17          C  0.025
ANISOU 1262  CG2AILE A  78     2410   2373   2501    191    301    753       C
ATOM   1263  CD1AILE A  78       2.161  13.091  25.506  0.60 20.91          C  0.026
ANISOU 1263  CD1AILE A  78     2571   2484   2889    177    254    254       C
ATOM   1264  H  AILE A  78       3.086  11.738  29.536  0.60 22.95          H  0.027
ATOM   1265  HA AILE A  78       2.633   9.417  28.240  0.60 22.32          H  0.027
ATOM   1266  HB AILE A  78       2.579  10.618  26.133  0.60 23.23          H  0.027
ATOM   1267 HG12AILE A  78       1.619  12.887  27.469  0.60 24.16          H  0.028
ATOM   1268 HG13AILE A  78       3.185  12.734  27.243  0.60 24.16          H  0.028
ATOM   1269 HG21AILE A  78       0.263  10.941  26.164  0.60 22.99          H  0.027
ATOM   1270 HG22AILE A  78       0.636   9.596  26.923  0.60 22.99          H  0.027
ATOM   1271 HG23AILE A  78       0.282  10.904  27.752  0.60 22.99          H  0.027
ATOM   1272 HD11AILE A  78       2.832  12.699  24.925  0.60 25.08          H  0.029
ATOM   1273 HD12AILE A  78       1.274  12.891  25.168  0.60 25.08          H  0.029
ATOM   1274 HD13AILE A  78       2.288  14.051  25.556  0.60 25.08          H  0.029
ATOM   1275  N  BILE A  78       2.629  11.008  29.436  0.40 19.63          N  0.025
ANISOU 1275  N  BILE A  78     2504   2582   2371    276    761    637       N
ATOM   1276  CA BILE A  78       2.837  10.408  28.118  0.40 19.56          C  0.025
ANISOU 1276  CA BILE A  78     2555   2507   2369    227    720    456       C
ATOM   1277  C  BILE A  78       4.326  10.449  27.785  0.40 19.80          C  0.025
ANISOU 1277  C  BILE A  78     2549   2437   2536    125    618    303       C
ATOM   1278  O  BILE A  78       4.982  11.469  28.029  0.40 20.15          O  0.026
ANISOU 1278  O  BILE A  78     2597   2459   2601    161    625    169       O
ATOM   1279  CB BILE A  78       1.997  11.151  27.066  0.40 20.34          C  0.026
ANISOU 1279  CB BILE A  78     2738   2553   2437    234    621    526       C
ATOM   1280  CG1BILE A  78       1.895  10.361  25.761  0.40 21.48          C  0.026
ANISOU 1280  CG1BILE A  78     2978   2655   2530    206    595    527       C
ATOM   1281  CG2BILE A  78       2.583  12.541  26.802  0.40 19.90          C  0.025
ANISOU 1281  CG2BILE A  78     2654   2474   2433    277    455    593       C
ATOM   1282  CD1BILE A  78       0.863   9.282  25.754  0.40 21.92          C  0.027
ANISOU 1282  CD1BILE A  78     3047   2756   2526    173    547    515       C
ATOM   1283  H  BILE A  78       3.238  11.571  29.659  0.40 23.54          H  0.028
ATOM   1284  HA BILE A  78       2.569   9.476  28.130  0.40 23.46          H  0.028
ATOM   1285  HB BILE A  78       1.101  11.244  27.423  0.40 24.39          H  0.028
ATOM   1286 HG12BILE A  78       1.675  10.977  25.045  0.40 25.77          H  0.029
ATOM   1287 HG13BILE A  78       2.753   9.943  25.587  0.40 25.77          H  0.029
ATOM   1288 HG21BILE A  78       1.923  13.079  26.339  0.40 23.87          H  0.028
ATOM   1289 HG22BILE A  78       2.811  12.953  27.650  0.40 23.87          H  0.028
ATOM   1290 HG23BILE A  78       3.379  12.450  26.254  0.40 23.87          H  0.028
ATOM   1291 HD11BILE A  78       1.064   8.648  26.460  0.40 26.29          H  0.029
ATOM   1292 HD12BILE A  78      -0.009   9.678  25.904  0.40 26.29          H  0.029
ATOM   1293 HD13BILE A  78       0.879   8.835  24.893  0.40 26.29          H  0.029
ATOM   1294  N   PRO A  79       4.903   9.380  27.240  1.00 19.16          N  0.025
ANISOU 1294  N   PRO A  79     2530   2320   2428     62    441    261       N
ATOM   1295  CA  PRO A  79       6.259   9.486  26.693  1.00 16.70          C  0.023
ANISOU 1295  CA  PRO A  79     2405   2107   1832    214    584    231       C
ATOM   1296  C   PRO A  79       6.256  10.509  25.573  1.00 15.85          C  0.023
ANISOU 1296  C   PRO A  79     2117   2126   1780    198    370    149       C
ATOM   1297  O   PRO A  79       5.314  10.586  24.775  1.00 16.79          O  0.023
ANISOU 1297  O   PRO A  79     2122   2258   1998     53    198    190       O
ATOM   1298  CB  PRO A  79       6.540   8.072  26.177  1.00 18.33          C  0.024
ANISOU 1298  CB  PRO A  79     2574   2028   2363    283    387    186       C
ATOM   1299  CG  PRO A  79       5.221   7.493  25.937  1.00 21.17          C  0.026
ANISOU 1299  CG  PRO A  79     2807   2156   3082    110    320    -76       C
ATOM   1300  CD  PRO A  79       4.274   8.074  26.944  1.00 20.08          C  0.026
```

```
ANISOU 1300  CD  PRO A  79     2648   2210   2770     25    274    227       C
ATOM   1301  HA  PRO A  79       6.918   9.711  27.368  1.00 20.03           H  0.025
ATOM   1302  HB2 PRO A  79       7.055   8.116  25.356  1.00 21.99           H  0.027
ATOM   1303  HB3 PRO A  79       7.026   7.565  26.847  1.00 21.99           H  0.027
ATOM   1304  HG2 PRO A  79       4.933   7.716  25.038  1.00 25.40           H  0.029
ATOM   1305  HG3 PRO A  79       5.271   6.530  26.040  1.00 25.40           H  0.029
ATOM   1306  HD2 PRO A  79       3.388   8.190  26.566  1.00 24.08           H  0.028
ATOM   1307  HD3 PRO A  79       4.225   7.520  27.739  1.00 24.08           H  0.028
ATOM   1308  N   CYS A  80       7.311  11.315  25.516  1.00 15.67           N  0.023
ANISOU 1308  N   CYS A  80     2094   2278   1581    283    261    229       N
ATOM   1309  CA  CYS A  80       7.382  12.324  24.468  1.00 15.68           C  0.023
ANISOU 1309  CA  CYS A  80     2096   2082   1781    235    117    298       C
ATOM   1310  C   CYS A  80       7.309  11.678  23.084  1.00 15.64           C  0.023
ANISOU 1310  C   CYS A  80     2252   2037   1652    362    116    386       C
ATOM   1311  O   CYS A  80       6.815  12.295  22.131  1.00 15.68           O  0.023
ANISOU 1311  O   CYS A  80     2256   2221   1480    433     40    223       O
ATOM   1312  CB  CYS A  80       8.666  13.152  24.620  1.00 16.02           C  0.023
ANISOU 1312  CB  CYS A  80     2100   2085   1903    274     84     76       C
ATOM   1313  SG  CYS A  80       8.792  14.087  26.134  1.00 15.51           S  0.022
ANISOU 1313  SG  CYS A  80     2224   2113   1557    357    305    162       S
ATOM   1314  H   CYS A  80       7.980  11.299  26.057  1.00 18.79           H  0.025
ATOM   1315  HA  CYS A  80       6.632  12.933  24.555  1.00 18.81           H  0.025
ATOM   1316  HB2 CYS A  80       9.425  12.548  24.588  1.00 19.22           H  0.025
ATOM   1317  HB3 CYS A  80       8.712  13.783  23.885  1.00 19.22           H  0.025
ATOM   1318  N   SER A  81       7.809  10.448  22.932  1.00 16.72           N  0.023
ANISOU 1318  N   SER A  81     2508   2146   1699    384     48    432       N
ATOM   1319  CA  SER A  81       7.728   9.762  21.646  1.00 17.76           C  0.024
ANISOU 1319  CA  SER A  81     2775   2199   1776    399    -19    131       C
ATOM   1320  C   SER A  81       6.294   9.630  21.131  1.00 19.31           C  0.025
ANISOU 1320  C   SER A  81     2778   2349   2208    364    -93     29       C
ATOM   1321  O   SER A  81       6.081   9.588  19.912  1.00 19.91           O  0.025
ANISOU 1321  O   SER A  81     2802   2372   2393    417   -233     29       O
ATOM   1322  CB  SER A  81       8.373   8.382  21.753  1.00 19.75           C  0.025
ANISOU 1322  CB  SER A  81     3132   2309   2063    312    199   -114       C
ATOM   1323  OG  SER A  81       7.648   7.585  22.620  1.00 21.09           O  0.026
ANISOU 1323  OG  SER A  81     3287   2386   2339    348    214     32       O
ATOM   1324  H   SER A  81       8.196   9.996  23.552  1.00 20.05           H  0.025
ATOM   1325  HA  SER A  81       8.220  10.289  20.997  1.00 21.30           H  0.026
ATOM   1326  HB2 SER A  81       8.386   7.967  20.876  1.00 23.69           H  0.028
ATOM   1327  HB3 SER A  81       9.278   8.477  22.089  1.00 23.69           H  0.028
ATOM   1328  HG  SER A  81       6.888   7.428  22.299  1.00 25.29           H  0.029
ATOM   1329  N   ALA A  82       5.306   9.544  22.022  1.00 19.21           N  0.025
ANISOU 1329  N   ALA A  82     2632   2313   2354    320    -75    328       N
ATOM   1330  CA  ALA A  82       3.909   9.460  21.588  1.00 20.29           C  0.026
ANISOU 1330  CA  ALA A  82     2666   2341   2701     -4    -35    376       C
ATOM   1331  C   ALA A  82       3.480  10.687  20.807  1.00 20.51           C  0.026
ANISOU 1331  C   ALA A  82     2533   2383   2876     94   -179     83       C
ATOM   1332  O   ALA A  82       2.519  10.625  20.029  1.00 21.49           O  0.026
ANISOU 1332  O   ALA A  82     2450   2532   3182     64   -356    175       O
ATOM   1333  CB  ALA A  82       2.995   9.303  22.789  1.00 22.22           C  0.027
ANISOU 1333  CB  ALA A  82     2922   2511   3008     54    -66    509       C
ATOM   1334  H   ALA A  82       5.414   9.531  22.875  1.00 23.04           H  0.027
ATOM   1335  HA  ALA A  82       3.815   8.679  21.021  1.00 24.33           H  0.028
ATOM   1336  HB1 ALA A  82       2.080   9.216  22.480  1.00 26.65           H  0.029
ATOM   1337  HB2 ALA A  82       3.255   8.508  23.281  1.00 26.65           H  0.029
ATOM   1338  HB3 ALA A  82       3.080  10.085  23.355  1.00 26.65           H  0.029
ATOM   1339  N   LEU A  83       4.141  11.814  21.023  1.00 19.22           N  0.025
ANISOU 1339  N   LEU A  83     2402   2218   2680    112    -51     73       N
ATOM   1340  CA  LEU A  83       3.847  13.057  20.334  1.00 19.39           C  0.025
ANISOU 1340  CA  LEU A  83     2363   2294   2709    295     92    194       C
ATOM   1341  C   LEU A  83       4.360  13.097  18.908  1.00 18.18           C  0.024
ANISOU 1341  C   LEU A  83     2451   2036   2419    318     -5    -48       C
ATOM   1342  O   LEU A  83       4.131  14.102  18.230  1.00 18.33           O  0.024
ANISOU 1342  O   LEU A  83     2552   1975   2437    340   -220    -52       O
ATOM   1343  CB  LEU A  83       4.420  14.215  21.149  1.00 18.80           C  0.025
ANISOU 1343  CB  LEU A  83     2318   2445   2383    529     55     25       C
ATOM   1344  CG  LEU A  83       3.843  14.360  22.553  1.00 20.22           C  0.026
ANISOU 1344  CG  LEU A  83     2454   2553   2674    422     38     65       C
ATOM   1345  CD1 LEU A  83       4.610  15.359  23.357  1.00 21.01           C  0.026
ANISOU 1345  CD1 LEU A  83     2648   2491   2844    483     44    267       C
ATOM   1346  CD2 LEU A  83       2.389  14.766  22.506  1.00 22.42           C  0.027
ANISOU 1346  CD2 LEU A  83     2525   2878   3115    464    166   -136       C
ATOM   1347  H   LEU A  83       4.787  11.886  21.586  1.00 23.05           H  0.027
ATOM   1348  HA  LEU A  83       2.886  13.172  20.275  1.00 23.25           H  0.027
ATOM   1349  HB2 LEU A  83       5.376  14.083  21.238  1.00 22.55           H  0.027
ATOM   1350  HB3 LEU A  83       4.242  15.041  20.673  1.00 22.55           H  0.027
ATOM   1351  HG  LEU A  83       3.912  13.496  22.987  1.00 24.25           H  0.028
ATOM   1352 HD11 LEU A  83       4.135  15.529  24.186  1.00 25.20           H  0.029
```

```
ATOM   1353 HD12 LEU A  83       5.491  15.000  23.549  1.00 25.20           H  0.029
ATOM   1354 HD13 LEU A  83       4.691  16.180  22.848  1.00 25.20           H  0.029
ATOM   1355 HD21 LEU A  83       2.085  14.957  23.407  1.00 26.89           H  0.030
ATOM   1356 HD22 LEU A  83       2.300  15.558  21.952  1.00 26.89           H  0.030
ATOM   1357 HD23 LEU A  83       1.870  14.039  22.129  1.00 26.89           H  0.030
ATOM   1358  N   LEU A  84       5.083  12.065  18.464  1.00 18.48           N  0.024
ANISOU 1358  N   LEU A  84     2433   2080   2508    384    -18     82       N
ATOM   1359  CA  LEU A  84       5.616  12.020  17.113  1.00 18.29           C  0.024
ANISOU 1359  CA  LEU A  84     2468   2189   2293    306    -80     15       C
ATOM   1360  C   LEU A  84       4.798  11.129  16.190  1.00 19.22           C  0.025
ANISOU 1360  C   LEU A  84     2664   2411   2227    171   -142    -96       C
ATOM   1361  O   LEU A  84       5.119  11.025  14.990  1.00 19.31           O  0.025
ANISOU 1361  O   LEU A  84     2702   2514   2119    281    -90   -234       O
ATOM   1362  CB  LEU A  84       7.055  11.508  17.140  1.00 18.24           C  0.024
ANISOU 1362  CB  LEU A  84     2422   2218   2291    353   -165    -13       C
ATOM   1363  CG  LEU A  84       7.999  12.309  18.033  1.00 18.16           C  0.024
ANISOU 1363  CG  LEU A  84     2322   2307   2270    426   -275    110       C
ATOM   1364  CD1 LEU A  84       9.378  11.722  17.886  1.00 19.96           C  0.025
ANISOU 1364  CD1 LEU A  84     2445   2469   2671    553   -240   -122       C
ATOM   1365  CD2 LEU A  84       7.998  13.770  17.713  1.00 17.48           C  0.024
ANISOU 1365  CD2 LEU A  84     2326   2300   2015    432   -248    280       C
ATOM   1366  H   LEU A  84       5.277  11.374  18.938  1.00 22.16           H  0.027
ATOM   1367  HA  LEU A  84       5.615  12.919  16.750  1.00 21.94           H  0.027
ATOM   1368  HB2 LEU A  84       7.051  10.594  17.465  1.00 21.88           H  0.027
ATOM   1369  HB3 LEU A  84       7.410  11.537  16.238  1.00 21.88           H  0.027
ATOM   1370  HG  LEU A  84       7.701  12.252  18.954  1.00 21.78           H  0.027
ATOM   1371 HD11 LEU A  84       9.972  12.147  18.524  1.00 23.94           H  0.028
ATOM   1372 HD12 LEU A  84       9.335  10.768  18.059  1.00 23.94           H  0.028
ATOM   1373 HD13 LEU A  84       9.694  11.880  16.983  1.00 23.94           H  0.028
ATOM   1374 HD21 LEU A  84       8.696  14.206  18.227  1.00 20.96           H  0.026
ATOM   1375 HD22 LEU A  84       8.165  13.886  16.765  1.00 20.96           H  0.026
ATOM   1376 HD23 LEU A  84       7.134  14.144  17.945  1.00 20.96           H  0.026
ATOM   1377  N  ASER A  85       3.756  10.491  16.713  0.72 20.62           N  0.026
ANISOU 1377  N  ASER A  85     2744   2256   2835    -51   -286   -145       N
ATOM   1378  CA ASER A  85       2.981   9.537  15.932  0.72 20.99           C  0.026
ANISOU 1378  CA ASER A  85     2836   2197   2942    -75   -540   -127       C
ATOM   1379  C  ASER A  85       2.268  10.195  14.754  0.72 21.20           C  0.026
ANISOU 1379  C  ASER A  85     2986   2237   2834     48   -610   -152       C
ATOM   1380  O  ASER A  85       1.927  11.379  14.775  0.72 20.82           O  0.026
ANISOU 1380  O  ASER A  85     2830   2135   2944    181   -699   -279       O
ATOM   1381  CB ASER A  85       1.919   8.918  16.840  0.72 22.27           C  0.027
ANISOU 1381  CB ASER A  85     2928   2149   3384   -272   -593     72       C
ATOM   1382  OG ASER A  85       1.101   8.046  16.105  0.72 22.82           O  0.027
ANISOU 1382  OG ASER A  85     2953   2131   3586   -445   -563     83       O
ATOM   1383  H  ASER A  85       3.476  10.593  17.520  0.72 24.73           H  0.028
ATOM   1384  HA ASER A  85       3.583   8.864  15.577  0.72 25.18           H  0.029
ATOM   1385  HB2ASER A  85       2.356   8.423  17.550  0.72 26.71           H  0.029
ATOM   1386  HB3ASER A  85       1.371   9.625  17.217  0.72 26.71           H  0.029
ATOM   1387  HG ASER A  85       0.527   7.697  16.609  0.72 27.37           H  0.030
ATOM   1388  N  BSER A  85       3.764  10.472  16.709  0.28 20.19           N  0.026
ANISOU 1388  N  BSER A  85     2764   2351   2557     91   -208   -100       N
ATOM   1389  CA BSER A  85       3.004   9.503  15.932  0.28 20.45           C  0.026
ANISOU 1389  CA BSER A  85     2869   2333   2566     82   -327    -82       C
ATOM   1390  C  BSER A  85       2.270  10.181  14.772  0.28 21.08           C  0.026
ANISOU 1390  C  BSER A  85     3024   2315   2671    128   -442   -150       C
ATOM   1391  O  BSER A  85       1.947  11.369  14.812  0.28 20.98           O  0.026
ANISOU 1391  O  BSER A  85     2978   2280   2714    160   -456   -230       O
ATOM   1392  CB BSER A  85       1.953   8.854  16.832  0.28 20.64           C  0.026
ANISOU 1392  CB BSER A  85     2881   2372   2590      8   -315     73       C
ATOM   1393  OG BSER A  85       1.217   9.861  17.520  0.28 20.56           O  0.026
ANISOU 1393  OG BSER A  85     2870   2438   2506    -56   -269     80       O
ATOM   1394  H  BSER A  85       3.482  10.571  17.515  0.28 24.22           H  0.028
ATOM   1395  HA BSER A  85       3.610   8.836  15.574  0.28 24.52           H  0.028
ATOM   1396  HB2BSER A  85       1.345   8.330  16.287  0.28 24.76           H  0.028
ATOM   1397  HB3BSER A  85       2.396   8.283  17.479  0.28 24.76           H  0.028
ATOM   1398  HG BSER A  85       0.655   9.505  18.034  0.28 24.66           H  0.028
ATOM   1399  N   SER A  86       1.987   9.399  13.728  1.00 21.87           N  0.027
ANISOU 1399  N   SER A  86     3185   2317   2807    212   -524   -193       N
ATOM   1400  CA  SER A  86       1.142   9.860  12.637  1.00 24.44           C  0.028
ANISOU 1400  CA  SER A  86     3513   2547   3224    220   -672   -578       C
ATOM   1401  C   SER A  86      -0.291  10.087  13.094  1.00 24.30           C  0.028
ANISOU 1401  C   SER A  86     3308   2599   3324    180   -790   -749       C
ATOM   1402  O   SER A  86      -0.998  10.891  12.484  1.00 25.62           O  0.029
ANISOU 1402  O   SER A  86     3301   2702   3732    387  -1019   -587       O
ATOM   1403  CB  SER A  86       1.236   8.860  11.475  1.00 26.64           C  0.029
ANISOU 1403  CB  SER A  86     3933   2883   3306    153   -781   -495       C
ATOM   1404  OG  SER A  86       0.633   7.636  11.811  1.00 29.50           O  0.031
ANISOU 1404  OG  SER A  86     4215   3308   3685    198   -682   -406       O
```

```
ATOM   1405  H   SER A  86       2.272   8.592  13.644  1.00 26.23           H  0.029
ATOM   1406  HA  SER A  86       1.455  10.713  12.297  1.00 29.31           H  0.031
ATOM   1407  HB2 SER A  86       0.783   9.232  10.702  1.00 31.96           H  0.032
ATOM   1408  HB3 SER A  86       2.171   8.704  11.269  1.00 31.96           H  0.032
ATOM   1409  HG  SER A  86       0.631   7.125  11.144  1.00 35.39           H  0.034
ATOM   1410  N   ASP A  87      -0.722   9.417  14.162  1.00 23.70           N  0.028
ANISOU 1410  N   ASP A  87     3131   2606   3269     27   -375   -804       N
ATOM   1411  CA  ASP A  87      -2.022   9.661  14.768  1.00 23.49           C  0.028
ANISOU 1411  CA  ASP A  87     2915   2586   3424   -216   -543   -684       C
ATOM   1412  C   ASP A  87      -1.861  10.794  15.775  1.00 22.38           C  0.027
ANISOU 1412  C   ASP A  87     2688   2476   3341    -18   -734   -494       C
ATOM   1413  O   ASP A  87      -1.008  10.716  16.672  1.00 22.59           O  0.027
ANISOU 1413  O   ASP A  87     2650   2420   3515    128   -750   -261       O
ATOM   1414  CB  ASP A  87      -2.540   8.408  15.475  1.00 25.43           C  0.029
ANISOU 1414  CB  ASP A  87     3203   2862   3596   -430   -538   -655       C
ATOM   1415  CG  ASP A  87      -3.910   8.613  16.046  1.00 27.76           C  0.030
ANISOU 1415  CG  ASP A  87     3454   3173   3922   -536   -291   -584       C
ATOM   1416  OD1 ASP A  87      -3.980   9.171  17.147  1.00 28.16           O  0.030
ANISOU 1416  OD1 ASP A  87     3556   3187   3957   -603      4   -534       O
ATOM   1417  OD2 ASP A  87      -4.904   8.243  15.392  1.00 31.08           O  0.032
ANISOU 1417  OD2 ASP A  87     3670   3575   4564   -380   -468   -398       O
ATOM   1418  H   ASP A  87      -0.268   8.805  14.559  1.00 28.43           H  0.030
ATOM   1419  HA  ASP A  87      -2.671   9.905  14.089  1.00 28.18           H  0.030
ATOM   1420  HB2 ASP A  87      -2.582   7.677  14.839  1.00 30.50           H  0.031
ATOM   1421  HB3 ASP A  87      -1.939   8.181  16.202  1.00 30.50           H  0.031
ATOM   1422  N   ILE A  88      -2.659  11.862  15.615  1.00 21.73           N  0.027
ANISOU 1422  N   ILE A  88     2536   2467   3254     17   -687   -424       N
ATOM   1423  CA  ILE A  88      -2.482  13.070  16.420  1.00 20.55           C  0.026
ANISOU 1423  CA  ILE A  88     2530   2427   2850      4   -772   -347       C
ATOM   1424  C   ILE A  88      -3.136  12.998  17.796  1.00 19.56           C  0.025
ANISOU 1424  C   ILE A  88     2410   2226   2797     22   -534    -84       C
ATOM   1425  O   ILE A  88      -3.068  13.980  18.543  1.00 18.35           O  0.024
ANISOU 1425  O   ILE A  88     2226   2126   2621     14   -389   -148       O
ATOM   1426  CB  ILE A  88      -2.902  14.323  15.615  1.00 20.02           C  0.025
ANISOU 1426  CB  ILE A  88     2689   2457   2460     94   -759   -400       C
ATOM   1427  CG1 ILE A  88      -4.426  14.327  15.370  1.00 20.75           C  0.026
ANISOU 1427  CG1 ILE A  88     2755   2457   2671     99   -857   -286       C
ATOM   1428  CG2 ILE A  88      -2.096  14.414  14.306  1.00 21.03           C  0.026
ANISOU 1428  CG2 ILE A  88     2885   2447   2659    136   -565   -133       C
ATOM   1429  CD1 ILE A  88      -4.935  15.669  14.884  1.00 21.64           C  0.026
ANISOU 1429  CD1 ILE A  88     2861   2484   2878    198   -575   -121       C
ATOM   1430  H   ILE A  88      -3.304  11.907  15.048  1.00 26.07           H  0.029
ATOM   1431  HA  ILE A  88      -1.539  13.176  16.620  1.00 24.64           H  0.028
ATOM   1432  HB  ILE A  88      -2.697  15.115  16.135  1.00 24.01           H  0.028
ATOM   1433 HG12 ILE A  88      -4.641  13.662  14.697  1.00 24.89           H  0.028
ATOM   1434 HG13 ILE A  88      -4.880  14.116  16.201  1.00 24.89           H  0.028
ATOM   1435 HG21 ILE A  88      -2.325  15.240  13.851  1.00 25.23           H  0.029
ATOM   1436 HG22 ILE A  88      -1.149  14.405  14.516  1.00 25.23           H  0.029
ATOM   1437 HG23 ILE A  88      -2.317  13.655  13.745  1.00 25.23           H  0.029
ATOM   1438 HD11 ILE A  88      -5.900  15.626  14.789  1.00 25.96           H  0.029
ATOM   1439 HD12 ILE A  88      -4.697  16.351  15.532  1.00 25.96           H  0.029
ATOM   1440 HD13 ILE A  88      -4.528  15.870  14.027  1.00 25.96           H  0.029
ATOM   1441  N   THR A  89      -3.702  11.859  18.195  1.00 20.24           N  0.026
ANISOU 1441  N   THR A  89     2448   2212   3029   -127   -391   -104       N
ATOM   1442  CA  THR A  89      -4.437  11.813  19.462  1.00 20.82           C  0.026
ANISOU 1442  CA  THR A  89     2509   2153   3249   -175   -190    136       C
ATOM   1443  C   THR A  89      -3.617  12.331  20.644  1.00 19.80           C  0.025
ANISOU 1443  C   THR A  89     2318   2127   3078      4     -9    303       C
ATOM   1444  O   THR A  89      -4.096  13.168  21.428  1.00 19.67           O  0.025
ANISOU 1444  O   THR A  89     2213   2091   3172     42    109    238       O
ATOM   1445  CB  THR A  89      -4.885  10.377  19.758  1.00 23.63           C  0.028
ANISOU 1445  CB  THR A  89     2748   2392   3839   -412     10    284       C
ATOM   1446  OG1 THR A  89      -5.781   9.950  18.734  1.00 25.67           O  0.029
ANISOU 1446  OG1 THR A  89     2769   2616   4370   -565   -349    142       O
ATOM   1447  CG2 THR A  89      -5.607  10.338  21.106  1.00 24.13           C  0.028
ANISOU 1447  CG2 THR A  89     2937   2392   3839   -474    208    572       C
ATOM   1448  H   THR A  89      -3.676  11.116  17.763  1.00 24.27           H  0.028
ATOM   1449  HA  THR A  89      -5.213  12.386  19.360  1.00 24.97           H  0.028
ATOM   1450  HB  THR A  89      -4.120   9.782  19.790  1.00 28.34           H  0.030
ATOM   1451  HG1 THR A  89      -5.400   9.985  17.986  1.00 30.79           H  0.032
ATOM   1452 HG21 THR A  89      -6.166   9.547  21.159  1.00 28.94           H  0.031
ATOM   1453 HG22 THR A  89      -4.960  10.316  21.828  1.00 28.94           H  0.031
ATOM   1454 HG23 THR A  89      -6.164  11.125  21.206  1.00 28.94           H  0.031
ATOM   1455  N   ALA A  90      -2.398  11.793  20.834  1.00 20.12           N  0.026
ANISOU 1455  N   ALA A  90     2194   2235   3215    -54    -43    381       N
ATOM   1456  CA  ALA A  90      -1.656  12.175  22.028  1.00 20.17           C  0.026
ANISOU 1456  CA  ALA A  90     2262   2325   3076    187     -4    513       C
ATOM   1457  C   ALA A  90      -1.313  13.662  21.997  1.00 18.23           C  0.024
```

```
ANISOU 1457  C   ALA A  90     2189   2256   2482    231     41    277       C
ATOM   1458  O   ALA A  90      -1.393  14.339  23.021  1.00 18.83           O  0.025
ANISOU 1458  O   ALA A  90     2397   2593   2165    234     90    619       O
ATOM   1459  CB  ALA A  90      -0.403  11.332  22.171  1.00 21.34           C  0.026
ANISOU 1459  CB  ALA A  90     2398   2407   3302    270   -131    642       C
ATOM   1460  H   ALA A  90      -2.007  11.235  20.309  1.00 24.13           H  0.028
ATOM   1461  HA  ALA A  90      -2.206  12.004  22.808  1.00 24.19           H  0.028
ATOM   1462  HB1 ALA A  90       0.083  11.620  22.960  1.00 25.59           H  0.029
ATOM   1463  HB2 ALA A  90      -0.658  10.400  22.261  1.00 25.59           H  0.029
ATOM   1464  HB3 ALA A  90       0.149  11.450  21.382  1.00 25.59           H  0.029
ATOM   1465  N   SER A  91      -0.889  14.199  20.844  1.00 17.06           N  0.024
ANISOU 1465  N   SER A  91     2196   2075   2213    250    -49    -60       N
ATOM   1466  CA  SER A  91      -0.568  15.621  20.779  1.00 16.54           C  0.023
ANISOU 1466  CA  SER A  91     2162   2129   1994    121   -111     31       C
ATOM   1467  C   SER A  91      -1.811  16.483  21.063  1.00 14.93           C  0.022
ANISOU 1467  C   SER A  91     2023   1995   1656     38     16    140       C
ATOM   1468  O   SER A  91      -1.749  17.504  21.759  1.00 15.87           O  0.023
ANISOU 1468  O   SER A  91     1923   2084   2023     27     76    159       O
ATOM   1469  CB  SER A  91      -0.011  15.951  19.402  1.00 16.86           C  0.023
ANISOU 1469  CB  SER A  91     1980   2296   2129     99    -74    -31       C
ATOM   1470  OG  SER A  91       1.360  15.623  19.313  1.00 16.06           O  0.023
ANISOU 1470  OG  SER A  91     1871   2313   1920    212     72    -86       O
ATOM   1471  H   SER A  91      -0.782  13.769  20.107  1.00 20.46           H  0.026
ATOM   1472  HA  SER A  91       0.103  15.827  21.448  1.00 19.83           H  0.025
ATOM   1473  HB2 SER A  91      -0.500  15.442  18.736  1.00 20.22           H  0.026
ATOM   1474  HB3 SER A  91      -0.119  16.901  19.239  1.00 20.22           H  0.026
ATOM   1475  HG  SER A  91       1.469  14.802  19.449  1.00 19.26           H  0.025
ATOM   1476  N   VAL A  92      -2.952  16.094  20.524  1.00 15.96           N  0.023
ANISOU 1476  N   VAL A  92     2036   1997   2029     72   -224     33       N
ATOM   1477  CA  VAL A  92      -4.170  16.872  20.760  1.00 16.20           C  0.023
ANISOU 1477  CA  VAL A  92     2037   2155   1964     68   -242     69       C
ATOM   1478  C   VAL A  92      -4.553  16.836  22.230  1.00 16.80           C  0.023
ANISOU 1478  C   VAL A  92     1975   2206   2204    -26   -116     95       C
ATOM   1479  O   VAL A  92      -4.871  17.863  22.832  1.00 17.15           O  0.024
ANISOU 1479  O   VAL A  92     1997   2240   2281     13     69    360       O
ATOM   1480  CB  VAL A  92      -5.320  16.364  19.877  1.00 17.78           C  0.024
ANISOU 1480  CB  VAL A  92     2073   2451   2233     44   -270     41       C
ATOM   1481  CG1 VAL A  92      -6.634  16.959  20.356  1.00 18.57           C  0.025
ANISOU 1481  CG1 VAL A  92     2078   2619   2357    -87   -228    130       C
ATOM   1482  CG2 VAL A  92      -5.032  16.649  18.402  1.00 18.52           C  0.025
ANISOU 1482  CG2 VAL A  92     2123   2632   2282    -45   -313   -107       C
ATOM   1483  H   VAL A  92      -3.054  15.399  20.027  1.00 19.14           H  0.025
ATOM   1484  HA  VAL A  92      -3.986  17.794  20.518  1.00 19.43           H  0.025
ATOM   1485  HB  VAL A  92      -5.402  15.400  19.951  1.00 21.33           H  0.026
ATOM   1486 HG11 VAL A  92      -7.283  16.913  19.637  1.00 22.27           H  0.027
ATOM   1487 HG12 VAL A  92      -6.950  16.453  21.121  1.00 22.27           H  0.027
ATOM   1488 HG13 VAL A  92      -6.487  17.884  20.610  1.00 22.27           H  0.027
ATOM   1489 HG21 VAL A  92      -5.777  16.331  17.868  1.00 22.21           H  0.027
ATOM   1490 HG22 VAL A  92      -4.922  17.605  18.281  1.00 22.21           H  0.027
ATOM   1491 HG23 VAL A  92      -4.219  16.187  18.144  1.00 22.21           H  0.027
ATOM   1492  N   ASN A  93      -4.530  15.648  22.834  1.00 17.55           N  0.024
ANISOU 1492  N   ASN A  93     2187   2248   2235    -17    -92    315       N
ATOM   1493  CA  ASN A  93      -4.964  15.555  24.227  1.00 18.54           C  0.025
ANISOU 1493  CA  ASN A  93     2375   2335   2335   -173    -77    517       C
ATOM   1494  C   ASN A  93      -4.048  16.358  25.134  1.00 17.39           C  0.024
ANISOU 1494  C   ASN A  93     2197   2198   2211   -107     68    496       C
ATOM   1495  O   ASN A  93      -4.506  17.029  26.051  1.00 18.30           O  0.024
ANISOU 1495  O   ASN A  93     2225   2396   2332     75    275    597       O
ATOM   1496  CB  ASN A  93      -5.026  14.093  24.679  1.00 21.01           C  0.026
ANISOU 1496  CB  ASN A  93     2757   2503   2723   -429    -15    803       C
ATOM   1497  CG  ASN A  93      -6.131  13.345  24.004  1.00 24.40           C  0.028
ANISOU 1497  CG  ASN A  93     3189   2823   3260   -589    104    690       C
ATOM   1498  OD1 ASN A  93      -6.988  13.953  23.381  1.00 26.38           O  0.029
ANISOU 1498  OD1 ASN A  93     3200   3080   3744   -700     99    256       O
ATOM   1499  ND2 ASN A  93      -6.143  12.029  24.163  1.00 26.96           N  0.030
ANISOU 1499  ND2 ASN A  93     3487   2977   3780   -828    236    454       N
ATOM   1500  H   ASN A  93      -4.276  14.909  22.475  1.00 21.05           H  0.026
ATOM   1501  HA  ASN A  93      -5.861  15.918  24.296  1.00 22.24           H  0.027
ATOM   1502  HB2 ASN A  93      -4.187  13.656  24.462  1.00 25.20           H  0.029
ATOM   1503  HB3 ASN A  93      -5.178  14.061  25.636  1.00 25.20           H  0.029
ATOM   1504 HD21 ASN A  93      -6.760  11.557  23.793  1.00 32.34           H  0.032
ATOM   1505 HD22 ASN A  93      -5.535  11.647  24.635  1.00 32.34           H  0.032
ATOM   1506  N   CYS A  94      -2.746  16.352  24.848  1.00 16.13           N  0.023
ANISOU 1506  N   CYS A  94     1939   2024   2167   -113   -140    301       N
ATOM   1507  CA  CYS A  94      -1.814  17.151  25.640  1.00 16.03           C  0.023
ANISOU 1507  CA  CYS A  94     1923   2160   2009     -2   -136    320       C
ATOM   1508  C   CYS A  94      -2.019  18.644  25.370  1.00 15.06           C  0.022
ANISOU 1508  C   CYS A  94     1965   2131   1627    178    -70    306       C
```

```
ATOM   1509  O   CYS A  94      -2.022  19.450  26.310  1.00 17.81           O  0.024
ANISOU 1509  O   CYS A  94     2124   2284   2359    263    -59    439       O
ATOM   1510  CB  CYS A  94      -0.375  16.713  25.348  1.00 15.84           C  0.023
ANISOU 1510  CB  CYS A  94     2002   2246   1772    199     72    189       C
ATOM   1511  SG  CYS A  94       0.852  17.428  26.430  1.00 16.86           S  0.023
ANISOU 1511  SG  CYS A  94     2144   2409   1852    146    -46    352       S
ATOM   1512  H   CYS A  94      -2.383  15.902  24.212  1.00 19.35           H  0.025
ATOM   1513  HA  CYS A  94      -1.973  17.000  26.585  1.00 19.23           H  0.025
ATOM   1514  HB2 CYS A  94      -0.321  15.749  25.441  1.00 19.00           H  0.025
ATOM   1515  HB3 CYS A  94      -0.152  16.972  24.440  1.00 19.00           H  0.025
ATOM   1516  N   ALA A  95      -2.205  19.025  24.101  1.00 14.65           N  0.022
ANISOU 1516  N   ALA A  95     1977   2154   1437    216     50    243       N
ATOM   1517  CA  ALA A  95      -2.439  20.441  23.774  1.00 14.31           C  0.022
ANISOU 1517  CA  ALA A  95     1989   2143   1306    114      6     91       C
ATOM   1518  C   ALA A  95      -3.683  20.972  24.465  1.00 14.64           C  0.022
ANISOU 1518  C   ALA A  95     1983   2190   1389     56    -28    208       C
ATOM   1519  O   ALA A  95      -3.703  22.137  24.877  1.00 14.89           O  0.022
ANISOU 1519  O   ALA A  95     1817   2224   1618     73    -63    250       O
ATOM   1520  CB  ALA A  95      -2.574  20.632  22.261  1.00 15.78           C  0.023
ANISOU 1520  CB  ALA A  95     2077   2197   1724    -45     78    148       C
ATOM   1521  H   ALA A  95      -2.202  18.497  23.422  1.00 17.57           H  0.024
ATOM   1522  HA  ALA A  95      -1.671  20.947  24.080  1.00 17.16           H  0.024
ATOM   1523  HB1 ALA A  95      -2.701  21.574  22.072  1.00 18.93           H  0.025
ATOM   1524  HB2 ALA A  95      -1.765  20.314  21.830  1.00 18.93           H  0.025
ATOM   1525  HB3 ALA A  95      -3.339  20.125  21.946  1.00 18.93           H  0.025
ATOM   1526  N   LYS A  96      -4.714  20.126  24.614  1.00 14.89           N  0.022
ANISOU 1526  N   LYS A  96     2074   2219   1366     92    116    350       N
ATOM   1527  CA  LYS A  96      -5.906  20.571  25.327  1.00 16.50           C  0.023
ANISOU 1527  CA  LYS A  96     2040   2344   1886     20    128    305       C
ATOM   1528  C   LYS A  96      -5.589  20.914  26.784  1.00 16.71           C  0.023
ANISOU 1528  C   LYS A  96     2153   2348   1848    161    108    577       C
ATOM   1529  O   LYS A  96      -6.125  21.886  27.338  1.00 18.13           O  0.024
ANISOU 1529  O   LYS A  96     2285   2321   2281    364    120    456       O
ATOM   1530  CB  LYS A  96      -7.008  19.510  25.260  1.00 16.96           C  0.023
ANISOU 1530  CB  LYS A  96     2021   2323   2101   -151    -49    247       C
ATOM   1531  CG  LYS A  96      -7.685  19.423  23.895  1.00 18.15           C  0.024
ANISOU 1531  CG  LYS A  96     2048   2466   2384   -108      8     62       C
ATOM   1532  CD  LYS A  96      -8.702  18.284  23.813  1.00 19.08           C  0.025
ANISOU 1532  CD  LYS A  96     1846   2727   2678   -101    -83     44       C
ATOM   1533  CE  LYS A  96      -9.420  18.263  22.489  1.00 20.08           C  0.026
ANISOU 1533  CE  LYS A  96     1892   2857   2879   -241   -122     46       C
ATOM   1534  NZ  LYS A  96     -10.364  17.124  22.403  1.00 22.28           N  0.027
ANISOU 1534  NZ  LYS A  96     2111   3128   3227   -436   -147     49       N
ATOM   1535  H   LYS A  96      -4.742  19.319  24.319  1.00 17.86           H  0.024
ATOM   1536  HA  LYS A  96      -6.238  21.371  24.889  1.00 19.79           H  0.025
ATOM   1537  HB2 LYS A  96      -6.620  18.643  25.456  1.00 20.34           H  0.026
ATOM   1538  HB3 LYS A  96      -7.690  19.724  25.916  1.00 20.34           H  0.026
ATOM   1539  HG2 LYS A  96      -8.151  20.255  23.719  1.00 21.77           H  0.027
ATOM   1540  HG3 LYS A  96      -7.009  19.271  23.216  1.00 21.77           H  0.027
ATOM   1541  HD2 LYS A  96      -8.242  17.436  23.921  1.00 22.89           H  0.027
ATOM   1542  HD3 LYS A  96      -9.363  18.394  24.514  1.00 22.89           H  0.027
ATOM   1543  HE2 LYS A  96      -9.924  19.086  22.384  1.00 24.08           H  0.028
ATOM   1544  HE3 LYS A  96      -8.772  18.177  21.773  1.00 24.08           H  0.028
ATOM   1545  HZ1 LYS A  96      -9.923  16.356  22.490  1.00 26.73           H  0.029
ATOM   1546  HZ2 LYS A  96     -10.972  17.183  23.050  1.00 26.73           H  0.029
ATOM   1547  HZ3 LYS A  96     -10.778  17.132  21.615  1.00 26.73           H  0.029
ATOM   1548  N   LYS A  97      -4.735  20.125  27.421  1.00 15.94           N  0.023
ANISOU 1548  N   LYS A  97     2285   2278   1493    206    160    785       N
ATOM   1549  CA  LYS A  97      -4.321  20.438  28.788  1.00 16.75           C  0.023
ANISOU 1549  CA  LYS A  97     2374   2502   1489    257    170    681       C
ATOM   1550  C   LYS A  97      -3.478  21.723  28.824  1.00 16.39           C  0.023
ANISOU 1550  C   LYS A  97     2248   2453   1527    206    123    503       C
ATOM   1551  O   LYS A  97      -3.748  22.621  29.628  1.00 17.04           O  0.024
ANISOU 1551  O   LYS A  97     2467   2539   1469    209    268    386       O
ATOM   1552  CB  LYS A  97      -3.548  19.268  29.386  1.00 18.96           C  0.025
ANISOU 1552  CB  LYS A  97     2705   2750   1750    355    162    518       C
ATOM   1553  CG  LYS A  97      -3.028  19.530  30.763  1.00 24.66           C  0.028
ANISOU 1553  CG  LYS A  97     3219   3182   2969    527   -191    638       C
ATOM   1554  CD  LYS A  97      -2.714  18.265  31.614  1.00 29.05           C  0.031
ANISOU 1554  CD  LYS A  97     3557   3588   3892    531   -215    762       C
ATOM   1555  CE  LYS A  97      -2.677  18.688  33.104  1.00 32.25           C  0.032
ANISOU 1555  CE  LYS A  97     3821   3865   4569    396   -323    877       C
ATOM   1556  NZ  LYS A  97      -2.119  17.743  34.106  1.00 34.58           N  0.033
ANISOU 1556  NZ  LYS A  97     4001   4092   5047    338   -105    928       N
ATOM   1557  H   LYS A  97      -4.384  19.411  27.094  1.00 19.11           H  0.025
ATOM   1558  HA  LYS A  97      -5.112  20.578  29.333  1.00 20.09           H  0.026
ATOM   1559  HB2 LYS A  97      -4.136  18.498  29.433  1.00 22.74           H  0.027
ATOM   1560  HB3 LYS A  97      -2.789  19.072  28.814  1.00 22.74           H  0.027
```

```
ATOM   1561  HG2 LYS A  97      -2.204  20.037  30.687  1.00 29.58           H  0.031
ATOM   1562  HG3 LYS A  97      -3.691  20.046  31.247  1.00 29.58           H  0.031
ATOM   1563  HD2 LYS A  97      -3.406  17.597  31.489  1.00 34.85           H  0.034
ATOM   1564  HD3 LYS A  97      -1.851  17.900  31.365  1.00 34.85           H  0.034
ATOM   1565  HE2 LYS A  97      -2.145  19.497  33.165  1.00 38.69           H  0.035
ATOM   1566  HE3 LYS A  97      -3.590  18.870  33.378  1.00 38.69           H  0.035
ATOM   1567  HZ1 LYS A  97      -2.166  18.099  34.920  1.00 41.49           H  0.037
ATOM   1568  HZ2 LYS A  97      -2.581  16.982  34.097  1.00 41.49           H  0.037
ATOM   1569  HZ3 LYS A  97      -1.268  17.566  33.917  1.00 41.49           H  0.037
ATOM   1570  N   ILE A  98      -2.528  21.863  27.888  1.00 15.39           N  0.022
ANISOU 1570  N   ILE A  98     2177   2280   1389    176    114    475       N
ATOM   1571  CA  ILE A  98      -1.677  23.061  27.836  1.00 15.44           C  0.022
ANISOU 1571  CA  ILE A  98     2105   2209   1553    162    119    367       C
ATOM   1572  C   ILE A  98      -2.495  24.340  27.697  1.00 14.41           C  0.022
ANISOU 1572  C   ILE A  98     1980   2125   1370    125    152    288       C
ATOM   1573  O   ILE A  98      -2.295  25.322  28.435  1.00 15.05           O  0.022
ANISOU 1573  O   ILE A  98     2156   2234   1328    165    -63    327       O
ATOM   1574  CB  ILE A  98      -0.646  22.949  26.705  1.00 14.42           C  0.022
ANISOU 1574  CB  ILE A  98     1863   2210   1406    150     36    462       C
ATOM   1575  CG1 ILE A  98       0.316  21.783  27.039  1.00 16.15           C  0.023
ANISOU 1575  CG1 ILE A  98     1870   2233   2034    282    -92    400       C
ATOM   1576  CG2 ILE A  98       0.146  24.244  26.510  1.00 15.04           C  0.022
ANISOU 1576  CG2 ILE A  98     1986   2181   1547     15    221    284       C
ATOM   1577  CD1 ILE A  98       1.231  21.364  25.887  1.00 17.30           C  0.024
ANISOU 1577  CD1 ILE A  98     2081   2226   2265    482   -108    415       C
ATOM   1578  H   ILE A  98      -2.356  21.284  27.276  1.00 18.45           H  0.024
ATOM   1579  HA  ILE A  98      -1.209  23.107  28.684  1.00 18.52           H  0.025
ATOM   1580  HB  ILE A  98      -1.119  22.780  25.876  1.00 17.29           H  0.024
ATOM   1581 HG12 ILE A  98       0.883  22.052  27.779  1.00 19.37           H  0.025
ATOM   1582 HG13 ILE A  98      -0.212  21.008  27.289  1.00 19.37           H  0.025
ATOM   1583 HG21 ILE A  98       0.912  24.063  25.944  1.00 18.04           H  0.024
ATOM   1584 HG22 ILE A  98      -0.427  24.904  26.089  1.00 18.04           H  0.024
ATOM   1585 HG23 ILE A  98       0.442  24.566  27.375  1.00 18.04           H  0.024
ATOM   1586 HD11 ILE A  98       1.710  20.560  26.140  1.00 20.74           H  0.026
ATOM   1587 HD12 ILE A  98       0.690  21.194  25.100  1.00 20.74           H  0.026
ATOM   1588 HD13 ILE A  98       1.860  22.081  25.707  1.00 20.74           H  0.026
ATOM   1589  N   VAL A  99      -3.412  24.353  26.728  1.00 14.35           N  0.022
ANISOU 1589  N   VAL A  99     1922   2124   1406    237     32    350       N
ATOM   1590  CA  VAL A  99      -4.178  25.559  26.416  1.00 14.20           C  0.021
ANISOU 1590  CA  VAL A  99     1978   2159   1256    259    292    401       C
ATOM   1591  C   VAL A  99      -5.167  25.925  27.510  1.00 15.11           C  0.022
ANISOU 1591  C   VAL A  99     2273   2282   1186    179    522    367       C
ATOM   1592  O   VAL A  99      -5.653  27.054  27.517  1.00 16.82           O  0.023
ANISOU 1592  O   VAL A  99     2561   2351   1480    357    257    348       O
ATOM   1593  CB  VAL A  99      -4.884  25.410  25.058  1.00 14.82           C  0.022
ANISOU 1593  CB  VAL A  99     2063   2102   1464    114    314    298       C
ATOM   1594  CG1 VAL A  99      -6.079  24.512  25.186  1.00 15.92           C  0.023
ANISOU 1594  CG1 VAL A  99     2078   2163   1807     40    367    557       C
ATOM   1595  CG2 VAL A  99      -5.259  26.721  24.448  1.00 14.89           C  0.022
ANISOU 1595  CG2 VAL A  99     2091   2159   1408    156    232    501       C
ATOM   1596  H   VAL A  99      -3.609  23.675  26.237  1.00 17.21           H  0.024
ATOM   1597  HA  VAL A  99      -3.560  26.304  26.339  1.00 17.02           H  0.023
ATOM   1598  HB  VAL A  99      -4.251  25.002  24.446  1.00 17.77           H  0.024
ATOM   1599 HG11 VAL A  99      -6.362  24.235  24.301  1.00 19.09           H  0.025
ATOM   1600 HG12 VAL A  99      -5.835  23.736  25.714  1.00 19.09           H  0.025
ATOM   1601 HG13 VAL A  99      -6.794  24.999  25.625  1.00 19.09           H  0.025
ATOM   1602 HG21 VAL A  99      -5.679  26.561  23.588  1.00 17.86           H  0.024
ATOM   1603 HG22 VAL A  99      -5.879  27.179  25.038  1.00 17.86           H  0.024
ATOM   1604 HG23 VAL A  99      -4.458  27.256  24.330  1.00 17.86           H  0.024
ATOM   1605  N   SER A 100      -5.432  25.024  28.441  1.00 16.59           N  0.023
ANISOU 1605  N   SER A 100     2449   2555   1300    221    731    520       N
ATOM   1606  CA  SER A 100      -6.378  25.210  29.524  1.00 17.67           C  0.024
ANISOU 1606  CA  SER A 100     2569   2910   1234    182    669    599       C
ATOM   1607  C   SER A 100      -5.718  25.712  30.789  1.00 20.19           C  0.026
ANISOU 1607  C   SER A 100     2967   3003   1700    299    458    205       C
ATOM   1608  O   SER A 100      -6.414  25.996  31.760  1.00 22.02           O  0.027
ANISOU 1608  O   SER A 100     3273   3245   1849    218    414      7       O
ATOM   1609  CB  SER A 100      -7.088  23.892  29.839  1.00 18.70           C  0.025
ANISOU 1609  CB  SER A 100     2439   3168   1500      5    747    533       C
ATOM   1610  OG  SER A 100      -7.831  23.475  28.717  1.00 20.82           O  0.026
ANISOU 1610  OG  SER A 100     2447   3396   2066    -13    498    250       O
ATOM   1611  H   SER A 100      -5.054  24.252  28.467  1.00 19.90           H  0.025
ATOM   1612  HA  SER A 100      -7.043  25.856  29.241  1.00 21.19           H  0.026
ATOM   1613  HB2 SER A 100      -6.428  23.216  30.055  1.00 22.43           H  0.027
ATOM   1614  HB3 SER A 100      -7.688  24.023  30.590  1.00 22.43           H  0.027
ATOM   1615  HG  SER A 100      -7.320  23.363  28.060  1.00 24.97           H  0.028
ATOM   1616  N   ASP A 101      -4.395  25.836  30.776  1.00 19.53           N  0.025
ANISOU 1616  N   ASP A 101     3026   2881   1512    192    -50    371       N
```

```
ATOM   1617  CA  ASP A 101      -3.605  26.085  32.012  1.00 21.33           C  0.026
ANISOU 1617  CA  ASP A 101     3171   2958   1976    193   -276    545       C
ATOM   1618  C   ASP A 101      -3.419  27.538  32.409  1.00 21.73           C  0.027
ANISOU 1618  C   ASP A 101     3292   3023   1941    268    -73    418       C
ATOM   1619  O   ASP A 101      -2.722  27.759  33.403  1.00 23.36           O  0.028
ANISOU 1619  O   ASP A 101     3475   3040   2362    476   -150    318       O
ATOM   1620  CB  ASP A 101      -2.250  25.401  31.887  1.00 23.43           C  0.028
ANISOU 1620  CB  ASP A 101     3165   3023   2714    303   -660    476       C
ATOM   1621  CG  ASP A 101      -1.811  24.833  33.221  1.00 26.92           C  0.030
ANISOU 1621  CG  ASP A 101     3292   3318   3617    146   -229    496       C
ATOM   1622  OD1 ASP A 101      -2.640  24.227  33.915  1.00 30.81           O  0.032
ANISOU 1622  OD1 ASP A 101     3739   3706   4260     85   -529    682       O
ATOM   1623  OD2 ASP A 101      -0.679  25.048  33.565  1.00 24.82           O  0.028
ANISOU 1623  OD2 ASP A 101     2887   3222   3322    379    -78    349       O
ATOM   1624  H   ASP A 101      -3.823  25.769  29.914  1.00 23.42           H  0.028
ATOM   1625  HA  ASP A 101      -4.140  25.600  32.825  1.00 25.59           H  0.029
ATOM   1626  HB2 ASP A 101      -2.316  24.593  31.160  1.00 28.10           H  0.030
ATOM   1627  HB3 ASP A 101      -1.508  26.119  31.543  1.00 28.10           H  0.030
ATOM   1628  N   GLY A 102      -3.938  28.495  31.649  1.00 19.71           N  0.025
ANISOU 1628  N   GLY A 102     3301   2893   1296    204    198    495       N
ATOM   1629  CA  GLY A 102      -3.989  29.865  32.131  1.00 20.31           C  0.026
ANISOU 1629  CA  GLY A 102     3289   2810   1620    232    358    165       C
ATOM   1630  C   GLY A 102      -3.723  30.912  31.068  1.00 18.05           C  0.024
ANISOU 1630  C   GLY A 102     3076   2670   1113    355    214    142       C
ATOM   1631  O   GLY A 102      -4.320  31.996  31.062  1.00 19.46           O  0.025
ANISOU 1631  O   GLY A 102     3232   2862   1299    451    369     33       O
ATOM   1632  H   GLY A 102      -4.279  28.376  30.869  1.00 23.64           H  0.028
ATOM   1633  HA2 GLY A 102      -4.870  30.034  32.501  1.00 24.36           H  0.028
ATOM   1634  HA3 GLY A 102      -3.326  29.976  32.830  1.00 24.36           H  0.028
ATOM   1635  N   ASN A 103      -2.778  30.643  30.183  1.00 16.45           N  0.023
ANISOU 1635  N   ASN A 103     2827   2514    909    526     46    252       N
ATOM   1636  CA  ASN A 103      -2.381  31.637  29.187  1.00 16.22           C  0.023
ANISOU 1636  CA  ASN A 103     2571   2462   1128    297    -47    240       C
ATOM   1637  C   ASN A 103      -2.877  31.300  27.783  1.00 14.56           C  0.022
ANISOU 1637  C   ASN A 103     2407   2090   1034    204     10    182       C
ATOM   1638  O   ASN A 103      -2.455  31.936  26.804  1.00 14.41           O  0.022
ANISOU 1638  O   ASN A 103     2379   2077   1019    233     34     72       O
ATOM   1639  CB  ASN A 103      -0.864  31.698  29.248  1.00 18.51           C  0.024
ANISOU 1639  CB  ASN A 103     2685   2727   1621    245   -480     51       C
ATOM   1640  CG  ASN A 103      -0.350  32.413  30.472  1.00 19.69           C  0.025
ANISOU 1640  CG  ASN A 103     3023   2850   1608    496   -326   -167       C
ATOM   1641  OD1 ASN A 103      -1.071  33.147  31.153  1.00 21.00           O  0.026
ANISOU 1641  OD1 ASN A 103     3287   2859   1835    490   -408   -345       O
ATOM   1642  ND2 ASN A 103       0.842  32.050  30.844  1.00 21.76           N  0.027
ANISOU 1642  ND2 ASN A 103     3084   2956   2227    541   -300   -191       N
ATOM   1643  H   ASN A 103      -2.351  29.898  30.135  1.00 19.73           H  0.025
ATOM   1644  HA  ASN A 103      -2.757  32.510  29.380  1.00 19.45           H  0.025
ATOM   1645  HB2 ASN A 103      -0.513  30.794  29.261  1.00 22.20           H  0.027
ATOM   1646  HB3 ASN A 103      -0.536  32.170  28.466  1.00 22.20           H  0.027
ATOM   1647 HD21 ASN A 103       1.266  31.448  30.400  1.00 26.10           H  0.029
ATOM   1648 HD22 ASN A 103       1.206  32.411  31.534  1.00 26.10           H  0.029
ATOM   1649  N   GLY A 104      -3.806  30.359  27.669  1.00 13.89           N  0.021
ANISOU 1649  N   GLY A 104     2349   1961    968    218     70    271       N
ATOM   1650  CA  GLY A 104      -4.349  30.063  26.372  1.00 12.24           C  0.020
ANISOU 1650  CA  GLY A 104     1992   2012    647    125     -2    120       C
ATOM   1651  C   GLY A 104      -3.253  29.637  25.395  1.00 12.46           C  0.020
ANISOU 1651  C   GLY A 104     1776   2001    958    137    244    129       C
ATOM   1652  O   GLY A 104      -2.308  28.929  25.756  1.00 14.00           O  0.021
ANISOU 1652  O   GLY A 104     2015   2183   1121    200     91     71       O
ATOM   1653  H   GLY A 104      -4.127  29.893  28.317  1.00 16.66           H  0.023
ATOM   1654  HA2 GLY A 104      -4.995  29.344  26.446  1.00 14.68           H  0.022
ATOM   1655  HA3 GLY A 104      -4.791  30.850  26.017  1.00 14.68           H  0.022
ATOM   1656  N   MET A 105      -3.393  30.024  24.128  1.00 12.94           N  0.020
ANISOU 1656  N   MET A 105     1708   1992   1216    214     88     66       N
ATOM   1657  CA  MET A 105      -2.449  29.580  23.105  1.00 11.19           C  0.019
ANISOU 1657  CA  MET A 105     1666   1852    734    174    114   -181       C
ATOM   1658  C   MET A 105      -1.175  30.398  23.145  1.00 11.91           C  0.020
ANISOU 1658  C   MET A 105     1743   1831    951    157     11    178       C
ATOM   1659  O   MET A 105      -0.238  30.051  22.428  1.00 12.27           O  0.020
ANISOU 1659  O   MET A 105     1769   1980    914    124     11    146       O
ATOM   1660  CB  MET A 105      -3.144  29.688  21.747  1.00 12.57           C  0.020
ANISOU 1660  CB  MET A 105     1822   1915   1041     92    -61   -131       C
ATOM   1661  CG  MET A 105      -4.137  28.530  21.477  1.00 13.50           C  0.021
ANISOU 1661  CG  MET A 105     1878   1811   1440    101   -117    168       C
ATOM   1662  SD  MET A 105      -4.565  28.280  19.755  1.00 13.44           S  0.021
ANISOU 1662  SD  MET A 105     1973   2057   1075     85     13     -2       S
ATOM   1663  CE  MET A 105      -3.115  27.516  19.185  1.00 13.58           C  0.021
ANISOU 1663  CE  MET A 105     2269   1949    940    124    337   -178       C
```

```
ATOM   1664  H   MET A 105      -4.019  30.538  23.839  1.00 15.51           H  0.022
ATOM   1665  HA  MET A 105      -2.203  28.649  23.227  1.00 13.42           H  0.021
ATOM   1666  HB2 MET A 105      -3.641  30.521  21.714  1.00 15.08           H  0.022
ATOM   1667  HB3 MET A 105      -2.472  29.675  21.048  1.00 15.08           H  0.022
ATOM   1668  HG2 MET A 105      -3.741  27.705  21.800  1.00 16.19           H  0.023
ATOM   1669  HG3 MET A 105      -4.960  28.713  21.957  1.00 16.19           H  0.023
ATOM   1670  HE1 MET A 105      -3.231  27.273  18.254  1.00 16.28           H  0.023
ATOM   1671  HE2 MET A 105      -2.375  28.136  19.275  1.00 16.28           H  0.023
ATOM   1672  HE3 MET A 105      -2.947  26.722  19.716  1.00 16.28           H  0.023
ATOM   1673  N   ASN A 106      -1.065  31.362  24.082  1.00 13.34           N  0.021
ANISOU 1673  N   ASN A 106     1809   1822   1438    -19      0     19       N
ATOM   1674  CA  ASN A 106       0.238  32.006  24.252  1.00 13.73           C  0.021
ANISOU 1674  CA  ASN A 106     1997   2038   1181     51   -288   -215       C
ATOM   1675  C   ASN A 106       1.292  31.026  24.751  1.00 14.05           C  0.021
ANISOU 1675  C   ASN A 106     2024   2130   1183    119    -67     89       C
ATOM   1676  O   ASN A 106       2.486  31.338  24.670  1.00 16.99           O  0.023
ANISOU 1676  O   ASN A 106     1977   2214   2265    130   -308    -74       O
ATOM   1677  CB  ASN A 106       0.131  33.210  25.190  1.00 13.76           C  0.021
ANISOU 1677  CB  ASN A 106     1968   1949   1311     57   -271    -24       C
ATOM   1678  CG  ASN A 106      -0.773  34.273  24.643  1.00 13.95           C  0.021
ANISOU 1678  CG  ASN A 106     2122   2031   1148    200   -119     70       C
ATOM   1679  OD1 ASN A 106      -0.463  34.946  23.660  1.00 13.71           O  0.021
ANISOU 1679  OD1 ASN A 106     2183   1935   1092     19    -53     95       O
ATOM   1680  ND2 ASN A 106      -1.892  34.468  25.300  1.00 15.23           N  0.022
ANISOU 1680  ND2 ASN A 106     2161   2141   1483    372     72    133       N
ATOM   1681  H   ASN A 106      -1.695  31.638  24.597  1.00 16.00           H  0.023
ATOM   1682  HA  ASN A 106       0.532  32.354  23.395  1.00 16.46           H  0.023
ATOM   1683  HB2 ASN A 106      -0.226  32.918  26.043  1.00 16.50           H  0.023
ATOM   1684  HB3 ASN A 106       1.012  33.596  25.315  1.00 16.50           H  0.023
ATOM   1685 HD21 ASN A 106      -2.450  35.066  25.031  1.00 18.26           H  0.024
ATOM   1686 HD22 ASN A 106      -2.068  33.999  25.998  1.00 18.26           H  0.024
ATOM   1687  N   ALA A 107       0.888  29.870  25.259  1.00 13.75           N  0.021
ANISOU 1687  N   ALA A 107     2024   2068   1131    376     94    174       N
ATOM   1688  CA  ALA A 107       1.833  28.806  25.588  1.00 14.36           C  0.022
ANISOU 1688  CA  ALA A 107     2051   2296   1110    352     32    197       C
ATOM   1689  C   ALA A 107       2.725  28.400  24.405  1.00 15.00           C  0.022
ANISOU 1689  C   ALA A 107     2042   2389   1267    435     11    375       C
ATOM   1690  O   ALA A 107       3.826  27.848  24.611  1.00 18.65           O  0.025
ANISOU 1690  O   ALA A 107     2284   2911   1893    711      8     98       O
ATOM   1691  CB  ALA A 107       1.101  27.550  26.077  1.00 15.73           C  0.023
ANISOU 1691  CB  ALA A 107     2234   2303   1440    283      0    290       C
ATOM   1692  H   ALA A 107       0.068  29.671  25.426  1.00 16.48           H  0.023
ATOM   1693  HA  ALA A 107       2.393  29.146  26.303  1.00 17.22           H  0.024
ATOM   1694  HB1 ALA A 107       1.754  26.863  26.281  1.00 18.86           H  0.025
ATOM   1695  HB2 ALA A 107       0.593  27.771  26.873  1.00 18.86           H  0.025
ATOM   1696  HB3 ALA A 107       0.503  27.241  25.378  1.00 18.86           H  0.025
ATOM   1697  N   TRP A 108       2.236  28.616  23.178  1.00 13.22           N  0.021
ANISOU 1697  N   TRP A 108     1857   2200    966    214    150    153       N
ATOM   1698  CA  TRP A 108       3.009  28.379  21.967  1.00 13.99           C  0.021
ANISOU 1698  CA  TRP A 108     1858   2277   1179    257    139    315       C
ATOM   1699  C   TRP A 108       3.519  29.721  21.483  1.00 14.52           C  0.022
ANISOU 1699  C   TRP A 108     1700   2464   1354    120    -20    483       C
ATOM   1700  O   TRP A 108       2.755  30.558  20.979  1.00 13.98           O  0.021
ANISOU 1700  O   TRP A 108     1714   2316   1281     75     13    357       O
ATOM   1701  CB  TRP A 108       2.144  27.714  20.910  1.00 13.04           C  0.021
ANISOU 1701  CB  TRP A 108     1952   1943   1058    317    155     92       C
ATOM   1702  CG  TRP A 108       1.850  26.278  21.249  1.00 13.37           C  0.021
ANISOU 1702  CG  TRP A 108     1951   1853   1277    274    208     81       C
ATOM   1703  CD1 TRP A 108       2.654  25.217  21.012  1.00 15.06           C  0.022
ANISOU 1703  CD1 TRP A 108     2064   2058   1601    302    394     42       C
ATOM   1704  CD2 TRP A 108       0.677  25.773  21.905  1.00 12.40           C  0.020
ANISOU 1704  CD2 TRP A 108     1958   1869    885    236      8     -7       C
ATOM   1705  NE1 TRP A 108       2.068  24.073  21.500  1.00 14.32           N  0.022
ANISOU 1705  NE1 TRP A 108     2082   1966   1394    254    255     14       N
ATOM   1706  CE2 TRP A 108       0.836  24.384  22.027  1.00 13.63           C  0.021
ANISOU 1706  CE2 TRP A 108     1935   1928   1316    246     47     70       C
ATOM   1707  CE3 TRP A 108      -0.480  26.379  22.426  1.00 13.75           C  0.021
ANISOU 1707  CE3 TRP A 108     2026   2007   1191     55    161    -99       C
ATOM   1708  CZ2 TRP A 108      -0.117  23.597  22.636  1.00 14.12           C  0.021
ANISOU 1708  CZ2 TRP A 108     1839   1981   1547    272    100    169       C
ATOM   1709  CZ3 TRP A 108      -1.442  25.590  22.991  1.00 13.60           C  0.021
ANISOU 1709  CZ3 TRP A 108     1933   1834   1400    145     37     -5       C
ATOM   1710  CH2 TRP A 108      -1.259  24.220  23.091  1.00 13.44           C  0.021
ANISOU 1710  CH2 TRP A 108     1899   1837   1370    259    178     79       C
ATOM   1711  H   TRP A 108       1.441  28.905  23.025  1.00 15.85           H  0.023
ATOM   1712  HA  TRP A 108       3.764  27.799  22.156  1.00 16.77           H  0.023
ATOM   1713  HB2 TRP A 108       1.302  28.189  20.842  1.00 15.63           H  0.023
ATOM   1714  HB3 TRP A 108       2.608  27.736  20.058  1.00 15.63           H  0.023
```

```
ATOM   1715  HD1 TRP A 108       3.479  25.256  20.584  1.00 18.06           H  0.024
ATOM   1716  HE1 TRP A 108       2.416  23.287  21.479  1.00 17.18           H  0.024
ATOM   1717  HE3 TRP A 108      -0.588  27.302  22.387  1.00 16.49           H  0.023
ATOM   1718  HZ2 TRP A 108       0.006  22.681  22.735  1.00 16.94           H  0.023
ATOM   1719  HZ3 TRP A 108      -2.226  25.975  23.311  1.00 16.31           H  0.023
ATOM   1720  HH2 TRP A 108      -1.928  23.704  23.479  1.00 16.11           H  0.023
ATOM   1721  N   VAL A 109       4.808  29.965  21.696  1.00 16.99           N  0.023
ANISOU 1721  N   VAL A 109     1812   2821   1822   -203   -167    955       N
ATOM   1722  CA  VAL A 109       5.376  31.258  21.331  1.00 18.66           C  0.025
ANISOU 1722  CA  VAL A 109     1938   3159   1991   -316   -415   1223       C
ATOM   1723  C   VAL A 109       5.184  31.546  19.854  1.00 17.35           C  0.024
ANISOU 1723  C   VAL A 109     1792   2853   1949   -167   -304   1059       C
ATOM   1724  O   VAL A 109       4.885  32.685  19.468  1.00 17.00           O  0.023
ANISOU 1724  O   VAL A 109     1852   2788   1819   -364   -488    861       O
ATOM   1725  CB  VAL A 109       6.876  31.262  21.704  1.00 21.68           C  0.027
ANISOU 1725  CB  VAL A 109     2185   3590   2463   -564   -743   1555       C
ATOM   1726  CG1 VAL A 109       7.603  32.469  21.073  1.00 24.90           C  0.028
ANISOU 1726  CG1 VAL A 109     2418   3825   3218   -572   -934   1577       C
ATOM   1727  CG2 VAL A 109       7.064  31.226  23.199  1.00 24.45           C  0.028
ANISOU 1727  CG2 VAL A 109     2481   3753   3055   -694   -652   1458       C
ATOM   1728  H   VAL A 109       5.365  29.410  22.044  1.00 20.38           H  0.026
ATOM   1729  HA  VAL A 109       4.912  31.954  21.822  1.00 22.37           H  0.027
ATOM   1730  HB  VAL A 109       7.281  30.458  21.342  1.00 26.01           H  0.029
ATOM   1731 HG11 VAL A 109       8.455  32.592  21.519  1.00 29.87           H  0.031
ATOM   1732 HG12 VAL A 109       7.745  32.294  20.130  1.00 29.87           H  0.031
ATOM   1733 HG13 VAL A 109       7.054  33.261  21.183  1.00 29.87           H  0.031
ATOM   1734 HG21 VAL A 109       8.014  31.231  23.398  1.00 29.33           H  0.031
ATOM   1735 HG22 VAL A 109       6.640  32.006  23.590  1.00 29.33           H  0.031
ATOM   1736 HG23 VAL A 109       6.657  30.418  23.549  1.00 29.33           H  0.031
ATOM   1737  N   ALA A 110       5.299  30.524  18.999  1.00 16.81           N  0.023
ANISOU 1737  N   ALA A 110     1851   2628   1907    -26    115   1013       N
ATOM   1738  CA  ALA A 110       5.120  30.763  17.579  1.00 15.96           C  0.023
ANISOU 1738  CA  ALA A 110     1962   2460   1641     87    221    566       C
ATOM   1739  C   ALA A 110       3.685  31.163  17.272  1.00 13.79           C  0.021
ANISOU 1739  C   ALA A 110     1902   2103   1235    -13     94    483       C
ATOM   1740  O   ALA A 110       3.468  31.926  16.324  1.00 14.47           O  0.022
ANISOU 1740  O   ALA A 110     2001   1990   1507     61    130    292       O
ATOM   1741  CB  ALA A 110       5.519  29.547  16.734  1.00 17.18           C  0.024
ANISOU 1741  CB  ALA A 110     2130   2632   1767    145    271    433       C
ATOM   1742  H   ALA A 110       5.475  29.711  19.216  1.00 20.16           H  0.026
ATOM   1743  HA  ALA A 110       5.718  31.483  17.324  1.00 19.14           H  0.025
ATOM   1744  HB1 ALA A 110       5.397  29.761  15.796  1.00 20.61           H  0.026
ATOM   1745  HB2 ALA A 110       6.449  29.333  16.907  1.00 20.61           H  0.026
ATOM   1746  HB3 ALA A 110       4.956  28.795  16.977  1.00 20.61           H  0.026
ATOM   1747  N   TRP A 111       2.694  30.620  18.003  1.00 14.29           N  0.022
ANISOU 1747  N   TRP A 111     1764   2213   1452     64     84    290       N
ATOM   1748  CA  TRP A 111       1.311  31.094  17.825  1.00 13.60           C  0.021
ANISOU 1748  CA  TRP A 111     1687   2226   1253   -139    -49    130       C
ATOM   1749  C   TRP A 111       1.200  32.581  18.173  1.00 13.31           C  0.021
ANISOU 1749  C   TRP A 111     1693   2248   1115   -211   -319    289       C
ATOM   1750  O   TRP A 111       0.667  33.375  17.386  1.00 13.05           O  0.021
ANISOU 1750  O   TRP A 111     1816   2049   1091    -11   -131    252       O
ATOM   1751  CB  TRP A 111       0.320  30.274  18.642  1.00 13.47           C  0.021
ANISOU 1751  CB  TRP A 111     1771   2207   1140    -31    131    279       C
ATOM   1752  CG  TRP A 111      -1.077  30.803  18.516  1.00 12.65           C  0.020
ANISOU 1752  CG  TRP A 111     1786   2033    986    -20     84    146       C
ATOM   1753  CD1 TRP A 111      -1.995  30.508  17.543  1.00 12.14           C  0.020
ANISOU 1753  CD1 TRP A 111     1776   1810   1026    -12    -10    229       C
ATOM   1754  CD2 TRP A 111      -1.689  31.775  19.352  1.00 12.27           C  0.020
ANISOU 1754  CD2 TRP A 111     1757   1895   1010   -122      8    272       C
ATOM   1755  NE1 TRP A 111      -3.131  31.246  17.709  1.00 12.29           N  0.020
ANISOU 1755  NE1 TRP A 111     1776   1828   1064     37     -1   -145       N
ATOM   1756  CE2 TRP A 111      -2.970  32.042  18.819  1.00 12.25           C  0.020
ANISOU 1756  CE2 TRP A 111     1795   1814   1045    -83     77    102       C
ATOM   1757  CE3 TRP A 111      -1.274  32.447  20.520  1.00 13.82           C  0.021
ANISOU 1757  CE3 TRP A 111     1946   2094   1212   -278     74    287       C
ATOM   1758  CZ2 TRP A 111      -3.864  32.906  19.446  1.00 12.84           C  0.020
ANISOU 1758  CZ2 TRP A 111     2040   1894    945    -93    124    -47       C
ATOM   1759  CZ3 TRP A 111      -2.183  33.308  21.166  1.00 13.83           C  0.021
ANISOU 1759  CZ3 TRP A 111     2053   2214    988   -252     23     62       C
ATOM   1760  CH2 TRP A 111      -3.458  33.541  20.591  1.00 13.98           C  0.021
ANISOU 1760  CH2 TRP A 111     2050   1962   1300   -167    -91     45       C
ATOM   1761  H   TRP A 111       2.794  29.998  18.589  1.00 17.13           H  0.024
ATOM   1762  HA  TRP A 111       1.071  30.968  16.893  1.00 16.30           H  0.023
ATOM   1763  HB2 TRP A 111       0.327  29.356  18.327  1.00 16.15           H  0.023
ATOM   1764  HB3 TRP A 111       0.574  30.305  19.578  1.00 16.15           H  0.023
ATOM   1765  HD1 TRP A 111      -1.861  29.890  16.862  1.00 14.56           H  0.022
ATOM   1766  HE1 TRP A 111      -3.827  31.218  17.206  1.00 14.73           H  0.022
```

```
ATOM   1767  HE3 TRP A 111      -0.416  32.324  20.858  1.00 16.57           H  0.023
ATOM   1768  HZ2 TRP A 111      -4.714  33.050  19.096  1.00 15.40           H  0.022
ATOM   1769  HZ3 TRP A 111      -1.949  33.722  21.965  1.00 16.58           H  0.023
ATOM   1770  HH2 TRP A 111      -4.036  34.142  21.003  1.00 16.76           H  0.023
ATOM   1771  N   ARG A 112       1.779  32.994  19.317  1.00 14.11           N  0.021
ANISOU 1771  N   ARG A 112     1788   2220   1354   -170   -216    186       N
ATOM   1772  CA  ARG A 112       1.661  34.386  19.698  1.00 14.12           C  0.021
ANISOU 1772  CA  ARG A 112     1876   2238   1252   -301    -31     63       C
ATOM   1773  C   ARG A 112       2.329  35.272  18.659  1.00 13.41           C  0.021
ANISOU 1773  C   ARG A 112     1882   2007   1206   -220     13     38       C
ATOM   1774  O   ARG A 112       1.800  36.313  18.278  1.00 14.93           O  0.022
ANISOU 1774  O   ARG A 112     1981   1991   1700    -85    -44    170       O
ATOM   1775  CB  ARG A 112       2.296  34.628  21.061  1.00 16.19           C  0.023
ANISOU 1775  CB  ARG A 112     2015   2689   1447   -578    -13    -38       C
ATOM   1776  CG  ARG A 112       2.338  36.129  21.434  1.00 17.84           C  0.024
ANISOU 1776  CG  ARG A 112     2208   3063   1507   -557    -95   -236       C
ATOM   1777  CD  ARG A 112       2.672  36.340  22.899  1.00 21.27           C  0.026
ANISOU 1777  CD  ARG A 112     2663   3517   1900   -609     49   -146       C
ATOM   1778  NE  ARG A 112       3.973  35.770  23.208  1.00 23.47           N  0.028
ANISOU 1778  NE  ARG A 112     2979   3829   2109   -736    -52   -104       N
ATOM   1779  CZ  ARG A 112       5.154  36.304  22.919  1.00 26.25           C  0.029
ANISOU 1779  CZ  ARG A 112     3281   4153   2539   -761   -131   -361       C
ATOM   1780  NH1 ARG A 112       5.261  37.473  22.297  1.00 26.53           N  0.029
ANISOU 1780  NH1 ARG A 112     3439   4182   2460   -814    -74   -392       N
ATOM   1781  NH2 ARG A 112       6.263  35.639  23.252  1.00 27.90           N  0.030
ANISOU 1781  NH2 ARG A 112     3277   4341   2982   -763   -245   -318       N
ATOM   1782  H   ARG A 112       2.225  32.497  19.860  1.00 16.92           H  0.023
ATOM   1783  HA  ARG A 112       0.720  34.612  19.764  1.00 16.94           H  0.023
ATOM   1784  HB2 ARG A 112       1.780  34.163  21.738  1.00 19.42           H  0.025
ATOM   1785  HB3 ARG A 112       3.206  34.295  21.050  1.00 19.42           H  0.025
ATOM   1786  HG2 ARG A 112       3.017  36.571  20.902  1.00 21.39           H  0.026
ATOM   1787  HG3 ARG A 112       1.470  36.525  21.261  1.00 21.39           H  0.026
ATOM   1788  HD2 ARG A 112       2.696  37.290  23.094  1.00 25.51           H  0.029
ATOM   1789  HD3 ARG A 112       2.004  35.905  23.452  1.00 25.51           H  0.029
ATOM   1790  HE  ARG A 112       3.979  35.014  23.617  1.00 28.15           H  0.030
ATOM   1791 HH11 ARG A 112       4.553  37.905  22.070  1.00 31.83           H  0.032
ATOM   1792 HH12 ARG A 112       6.038  37.797  22.122  1.00 31.83           H  0.032
ATOM   1793 HH21 ARG A 112       6.205  34.877  23.646  1.00 33.46           H  0.033
ATOM   1794 HH22 ARG A 112       7.035  35.972  23.071  1.00 33.46           H  0.033
ATOM   1795  N   ASN A 113       3.495  34.870  18.163  1.00 13.56           N  0.021
ANISOU 1795  N   ASN A 113     1913   2019   1219   -199      5    135       N
ATOM   1796  CA  ASN A 113       4.288  35.736  17.300  1.00 13.31           C  0.021
ANISOU 1796  CA  ASN A 113     2002   2135    921   -257   -102    124       C
ATOM   1797  C   ASN A 113       3.874  35.691  15.835  1.00 13.64           C  0.021
ANISOU 1797  C   ASN A 113     2005   2060   1119   -281     57    168       C
ATOM   1798  O   ASN A 113       4.166  36.651  15.099  1.00 13.66           O  0.021
ANISOU 1798  O   ASN A 113     2056   1854   1281   -382    -17    198       O
ATOM   1799  CB  ASN A 113       5.769  35.385  17.429  1.00 14.50           C  0.022
ANISOU 1799  CB  ASN A 113     2170   2219   1121   -291     68    -38       C
ATOM   1800  CG  ASN A 113       6.351  35.855  18.727  1.00 15.48           C  0.022
ANISOU 1800  CG  ASN A 113     2141   2353   1387   -402   -113    -14       C
ATOM   1801  OD1 ASN A 113       5.914  36.858  19.316  1.00 17.66           O  0.024
ANISOU 1801  OD1 ASN A 113     2328   2752   1629   -387   -290   -240       O
ATOM   1802  ND2 ASN A 113       7.358  35.132  19.186  1.00 15.72           N  0.023
ANISOU 1802  ND2 ASN A 113     2195   2333   1445   -508    -90     87       N
ATOM   1803  H   ASN A 113       3.848  34.101  18.311  1.00 16.26           H  0.023
ATOM   1804  HA  ASN A 113       4.164  36.650  17.598  1.00 15.96           H  0.023
ATOM   1805  HB2 ASN A 113       5.873  34.422  17.382  1.00 17.39           H  0.024
ATOM   1806  HB3 ASN A 113       6.260  35.808  16.707  1.00 17.39           H  0.024
ATOM   1807 HD21 ASN A 113       7.739  35.346  19.927  1.00 18.85           H  0.025
ATOM   1808 HD22 ASN A 113       7.633  34.447  18.744  1.00 18.85           H  0.025
ATOM   1809  N   ARG A 114       3.180  34.622  15.376  1.00 14.72           N  0.022
ANISOU 1809  N   ARG A 114     2072   2151   1370   -277    -91    293       N
ATOM   1810  CA  ARG A 114       2.920  34.447  13.952  1.00 13.78           C  0.021
ANISOU 1810  CA  ARG A 114     1932   2075   1227   -366    -37    -51       C
ATOM   1811  C   ARG A 114       1.469  34.216  13.600  1.00 12.99           C  0.021
ANISOU 1811  C   ARG A 114     1981   2143    811   -165     90     28       C
ATOM   1812  O   ARG A 114       1.116  34.406  12.423  1.00 14.86           O  0.022
ANISOU 1812  O   ARG A 114     2108   2438   1101    -62    148    308       O
ATOM   1813  CB  ARG A 114       3.802  33.305  13.407  1.00 15.50           C  0.022
ANISOU 1813  CB  ARG A 114     1952   2161   1775   -298      8    117       C
ATOM   1814  CG  ARG A 114       5.272  33.542  13.736  1.00 15.68           C  0.023
ANISOU 1814  CG  ARG A 114     1880   2260   1818   -267   -137    310       C
ATOM   1815  CD  ARG A 114       6.165  32.461  13.229  1.00 15.32           C  0.022
ANISOU 1815  CD  ARG A 114     1973   2293   1556   -249   -130    129       C
ATOM   1816  NE  ARG A 114       6.299  32.478  11.790  1.00 15.04           N  0.022
ANISOU 1816  NE  ARG A 114     1838   2353   1525   -304     92    155       N
ATOM   1817  CZ  ARG A 114       7.128  31.692  11.138  1.00 14.49           C  0.022
```

```
ANISOU 1817  CZ  ARG A 114     1897   2467   1141   -397    150     95       C
ATOM   1818  NH1 ARG A 114       7.837  30.778  11.773  1.00 15.47           N  0.022
ANISOU 1818  NH1 ARG A 114     2040   2351   1489   -383     15     25       N
ATOM   1819  NH2 ARG A 114       7.227  31.815   9.825  1.00 15.60           N  0.022
ANISOU 1819  NH2 ARG A 114     2057   2446   1426   -269    129   -218       N
ATOM   1820  H   ARG A 114       2.858  33.999  15.875  1.00 17.65           H  0.024
ATOM   1821  HA  ARG A 114       3.172  35.255  13.478  1.00 16.52           H  0.023
ATOM   1822  HB2 ARG A 114       3.529  32.466  13.810  1.00 18.59           H  0.025
ATOM   1823  HB3 ARG A 114       3.707  33.256  12.443  1.00 18.59           H  0.025
ATOM   1824  HG2 ARG A 114       5.555  34.377  13.331  1.00 18.80           H  0.025
ATOM   1825  HG3 ARG A 114       5.376  33.589  14.699  1.00 18.80           H  0.025
ATOM   1826  HD2 ARG A 114       7.048  32.573  13.614  1.00 18.38           H  0.024
ATOM   1827  HD3 ARG A 114       5.798  31.601  13.486  1.00 18.38           H  0.024
ATOM   1828  HE  ARG A 114       5.815  33.028  11.340  1.00 18.04           H  0.024
ATOM   1829 HH11 ARG A 114       7.761  30.691  12.626  1.00 18.56           H  0.025
ATOM   1830 HH12 ARG A 114       8.376  30.271  11.336  1.00 18.56           H  0.025
ATOM   1831 HH21 ARG A 114       6.754  32.402   9.411  1.00 18.71           H  0.025
ATOM   1832 HH22 ARG A 114       7.765  31.308   9.385  1.00 18.71           H  0.025
ATOM   1833  N   CYS A 115       0.624  33.887  14.551  1.00 12.50           N  0.020
ANISOU 1833  N   CYS A 115     2034   1924    790   -202   -153    -18       N
ATOM   1834  CA  CYS A 115      -0.773  33.594  14.284  1.00 13.73           C  0.021
ANISOU 1834  CA  CYS A 115     1976   1959   1284    -69     11     50       C
ATOM   1835  C   CYS A 115      -1.730  34.538  14.987  1.00 12.24           C  0.020
ANISOU 1835  C   CYS A 115     2002   1927    723    -33    -53    -21       C
ATOM   1836  O   CYS A 115      -2.752  34.902  14.402  1.00 12.53           O  0.020
ANISOU 1836  O   CYS A 115     2001   2064    696    -40   -112     43       O
ATOM   1837  CB  CYS A 115      -1.080  32.178  14.787  1.00 14.07           C  0.021
ANISOU 1837  CB  CYS A 115     1974   2062   1311      9   -268    105       C
ATOM   1838  SG  CYS A 115      -0.136  30.869  13.985  1.00 13.87           S  0.021
ANISOU 1838  SG  CYS A 115     1942   1953   1375     64    -80     17       S
ATOM   1839  H   CYS A 115       0.838  33.823  15.381  1.00 14.98           H  0.022
ATOM   1840  HA  CYS A 115      -0.929  33.655  13.329  1.00 16.47           H  0.023
ATOM   1841  HB2 CYS A 115      -0.885  32.140  15.736  1.00 16.87           H  0.023
ATOM   1842  HB3 CYS A 115      -2.021  31.995  14.634  1.00 16.87           H  0.023
ATOM   1843  N   LYS A 116      -1.491  34.855  16.255  1.00 13.13           N  0.021
ANISOU 1843  N   LYS A 116     2182   2020    785    119     43     45       N
ATOM   1844  CA  LYS A 116      -2.370  35.695  17.040  1.00 13.19           C  0.021
ANISOU 1844  CA  LYS A 116     2225   2178    607     -1    -48   -261       C
ATOM   1845  C   LYS A 116      -2.634  36.995  16.326  1.00 14.82           C  0.022
ANISOU 1845  C   LYS A 116     2333   2363    934    304    -11   -130       C
ATOM   1846  O   LYS A 116      -1.720  37.697  15.882  1.00 15.36           O  0.022
ANISOU 1846  O   LYS A 116     2377   2118   1341    264   -292     78       O
ATOM   1847  CB  LYS A 116      -1.655  35.981  18.345  1.00 13.16           C  0.021
ANISOU 1847  CB  LYS A 116     2326   2046    628     -1   -148   -191       C
ATOM   1848  CG  LYS A 116      -2.422  36.750  19.380  1.00 13.45           C  0.021
ANISOU 1848  CG  LYS A 116     2309   2110    693    -78     95   -372       C
ATOM   1849  CD  LYS A 116      -1.650  36.904  20.680  1.00 13.75           C  0.021
ANISOU 1849  CD  LYS A 116     2267   2248    708   -148    180   -333       C
ATOM   1850  CE  LYS A 116      -2.431  37.526  21.786  1.00 14.64           C  0.022
ANISOU 1850  CE  LYS A 116     2203   2336   1024      8    -15   -321       C
ATOM   1851  NZ  LYS A 116      -1.633  37.639  23.064  1.00 14.31           N  0.022
ANISOU 1851  NZ  LYS A 116     1945   2251   1244    -12   -209   -394       N
ATOM   1852  H   LYS A 116      -0.803  34.585  16.694  1.00 15.74           H  0.023
ATOM   1853  HA  LYS A 116      -3.221  35.258  17.198  1.00 15.81           H  0.023
ATOM   1854  HB2 LYS A 116      -1.410  35.132  18.746  1.00 15.78           H  0.023
ATOM   1855  HB3 LYS A 116      -0.857  36.495  18.144  1.00 15.78           H  0.023
ATOM   1856  HG2 LYS A 116      -2.616  37.636  19.038  1.00 16.13           H  0.023
ATOM   1857  HG3 LYS A 116      -3.249  36.281  19.575  1.00 16.13           H  0.023
ATOM   1858  HD2 LYS A 116      -1.367  36.026  20.979  1.00 16.48           H  0.023
ATOM   1859  HD3 LYS A 116      -0.876  37.465  20.516  1.00 16.48           H  0.023
ATOM   1860  HE2 LYS A 116      -2.703  38.418  21.520  1.00 17.56           H  0.024
ATOM   1861  HE3 LYS A 116      -3.213  36.981  21.967  1.00 17.56           H  0.024
ATOM   1862  HZ1 LYS A 116      -1.361  36.833  23.325  1.00 17.17           H  0.024
ATOM   1863  HZ2 LYS A 116      -0.920  38.156  22.932  1.00 17.17           H  0.024
ATOM   1864  HZ3 LYS A 116      -2.134  37.997  23.705  1.00 17.17           H  0.024
ATOM   1865  N   GLY A 117      -3.909  37.306  16.199  1.00 16.29           N  0.023
ANISOU 1865  N   GLY A 117     2393   2747   1052    408   -133     -9       N
ATOM   1866  CA  GLY A 117      -4.329  38.540  15.616  1.00 17.30           C  0.024
ANISOU 1866  CA  GLY A 117     2616   2894   1062    583   -275   -259       C
ATOM   1867  C   GLY A 117      -4.459  38.514  14.117  1.00 17.39           C  0.024
ANISOU 1867  C   GLY A 117     2936   2631   1040    501   -293   -154       C
ATOM   1868  O   GLY A 117      -4.973  39.470  13.541  1.00 19.70           O  0.025
ANISOU 1868  O   GLY A 117     3421   2607   1455    717   -502    -69       O
ATOM   1869  H   GLY A 117      -4.557  36.800  16.452  1.00 19.54           H  0.025
ATOM   1870  HA2 GLY A 117      -5.193  38.781  15.985  1.00 20.74           H  0.026
ATOM   1871  HA3 GLY A 117      -3.685  39.228  15.846  1.00 20.74           H  0.026
ATOM   1872  N   THR A 118      -3.996  37.477  13.463  1.00 16.00           N  0.023
ANISOU 1872  N   THR A 118     2618   2323   1136    266   -208   -222       N
```

```
ATOM   1873  CA  THR A 118      -3.990  37.408  12.012  1.00 15.44           C  0.022
ANISOU 1873  CA  THR A 118     2455   2140   1273    122   -232    132        C
ATOM   1874  C   THR A 118      -5.259  36.720  11.517  1.00 15.73           C  0.023
ANISOU 1874  C   THR A 118     2609   2149   1218    149   -360     45        C
ATOM   1875  O   THR A 118      -6.070  36.235  12.306  1.00 16.17           O  0.023
ANISOU 1875  O   THR A 118     2647   2137   1362     97   -376    181        O
ATOM   1876  CB  THR A 118      -2.778  36.616  11.494  1.00 15.19           C  0.022
ANISOU 1876  CB  THR A 118     2345   2117   1309    144   -114    141        C
ATOM   1877  OG1 THR A 118      -2.922  35.201  11.703  1.00 14.09           O  0.021
ANISOU 1877  OG1 THR A 118     2294   2129    931    104   -171    124        O
ATOM   1878  CG2 THR A 118      -1.479  37.123  12.114  1.00 16.70           C  0.023
ANISOU 1878  CG2 THR A 118     2413   2297   1635    105    257   -116        C
ATOM   1879  H   THR A 118      -3.669  36.777  13.841  1.00 19.18           H  0.025
ATOM   1880  HA  THR A 118      -3.941  38.314  11.669  1.00 18.52           H  0.025
ATOM   1881  HB  THR A 118      -2.726  36.756  10.535  1.00 18.22           H  0.024
ATOM   1882  HG1 THR A 118      -2.949  35.034  12.526  1.00 16.90           H  0.023
ATOM   1883 HG21 THR A 118      -0.720  36.683  11.699  1.00 20.03           H  0.025
ATOM   1884 HG22 THR A 118      -1.398  38.080  11.981  1.00 20.03           H  0.025
ATOM   1885 HG23 THR A 118      -1.470  36.935  13.065  1.00 20.03           H  0.025
ATOM   1886  N   ASP A 119      -5.469  36.722  10.193  1.00 16.68           N  0.023
ANISOU 1886  N   ASP A 119     2742   2118   1477     44   -446    142        N
ATOM   1887  CA  ASP A 119      -6.639  36.048   9.577  1.00 16.81           C  0.023
ANISOU 1887  CA  ASP A 119     2899   2117   1372     55   -492    170        C
ATOM   1888  C   ASP A 119      -6.364  34.550   9.595  1.00 16.01           C  0.023
ANISOU 1888  C   ASP A 119     3011   2049   1024    222   -159    258        C
ATOM   1889  O   ASP A 119      -5.849  34.031   8.627  1.00 18.30           O  0.024
ANISOU 1889  O   ASP A 119     3282   2242   1428    158     65    169        O
ATOM   1890  CB  ASP A 119      -6.900  36.544   8.147  1.00 17.33           C  0.024
ANISOU 1890  CB  ASP A 119     2987   2242   1355   -128   -537    429        C
ATOM   1891  CG  ASP A 119      -8.191  36.012   7.557  1.00 20.14           C  0.026
ANISOU 1891  CG  ASP A 119     3051   2511   2091   -200   -592    346        C
ATOM   1892  OD1 ASP A 119      -8.640  34.934   7.970  1.00 20.87           O  0.026
ANISOU 1892  OD1 ASP A 119     3158   2914   1857   -248   -364    285        O
ATOM   1893  OD2 ASP A 119      -8.730  36.692   6.699  1.00 21.28           O  0.026
ANISOU 1893  OD2 ASP A 119     3021   2728   2335     75   -708    427        O
ATOM   1894  H   ASP A 119      -4.838  37.185   9.516  1.00 20.00           H  0.025
ATOM   1895  HA  ASP A 119      -7.520  36.252  10.184  1.00 20.16           H  0.026
ATOM   1896  HB2 ASP A 119      -6.949  37.631   8.155  1.00 20.78           H  0.026
ATOM   1897  HB3 ASP A 119      -6.073  36.245   7.505  1.00 20.78           H  0.026
ATOM   1898  N   VAL A 120      -6.671  33.889  10.703  1.00 16.39           N  0.023
ANISOU 1898  N   VAL A 120     3091   1927   1208    233   -307    313        N
ATOM   1899  CA  VAL A 120      -6.408  32.455  10.812  1.00 16.79           C  0.023
ANISOU 1899  CA  VAL A 120     3038   1993   1347    231   -335    383        C
ATOM   1900  C   VAL A 120      -7.431  31.609  10.077  1.00 16.63           C  0.023
ANISOU 1900  C   VAL A 120     3073   2056   1188    239    -53    193        C
ATOM   1901  O   VAL A 120      -7.157  30.437   9.776  1.00 17.25           O  0.024
ANISOU 1901  O   VAL A 120     3150   2211   1195    242   -234   -148        O
ATOM   1902  CB  VAL A 120      -6.227  32.011  12.272  1.00 17.21           C  0.024
ANISOU 1902  CB  VAL A 120     3113   2148   1277    195   -499    162        C
ATOM   1903  CG1 VAL A 120      -5.037  32.691  12.904  1.00 16.95           C  0.023
ANISOU 1903  CG1 VAL A 120     2995   2215   1232    241   -685    -43        C
ATOM   1904  CG2 VAL A 120      -7.461  32.301  13.073  1.00 17.52           C  0.024
ANISOU 1904  CG2 VAL A 120     3240   2214   1203    182   -133      6        C
ATOM   1905  H   VAL A 120      -7.049  34.238  11.392  1.00 19.65           H  0.025
ATOM   1906  HA  VAL A 120      -5.558  32.284  10.376  1.00 20.13           H  0.026
ATOM   1907  HB  VAL A 120      -6.069  31.054  12.276  1.00 20.63           H  0.026
ATOM   1908 HG11 VAL A 120      -4.931  32.364  13.811  1.00 20.33           H  0.026
ATOM   1909 HG12 VAL A 120      -4.244  32.488  12.384  1.00 20.33           H  0.026
ATOM   1910 HG13 VAL A 120      -5.190  33.649  12.914  1.00 20.33           H  0.026
ATOM   1911 HG21 VAL A 120      -7.354  31.930  13.963  1.00 21.01           H  0.026
ATOM   1912 HG22 VAL A 120      -7.584  33.262  13.128  1.00 21.01           H  0.026
ATOM   1913 HG23 VAL A 120      -8.224  31.894  12.634  1.00 21.01           H  0.026
ATOM   1914  N   GLN A 121      -8.576  32.194   9.730  1.00 17.94           N  0.024
ANISOU 1914  N   GLN A 121     2985   2196   1634    182    126     74        N
ATOM   1915  CA  GLN A 121      -9.528  31.490   8.884  1.00 20.03           C  0.025
ANISOU 1915  CA  GLN A 121     2920   2381   2311    -32    -11     71        C
ATOM   1916  C   GLN A 121      -8.901  31.072   7.559  1.00 17.52           C  0.024
ANISOU 1916  C   GLN A 121     2583   2218   1855     -4   -211    244        C
ATOM   1917  O   GLN A 121      -9.316  30.081   6.968  1.00 17.97           O  0.024
ANISOU 1917  O   GLN A 121     2500   2376   1952   -116   -438     -1        O
ATOM   1918  CB  GLN A 121     -10.755  32.361   8.578  1.00 26.03           C  0.029
ANISOU 1918  CB  GLN A 121     3315   2902   3673    -87     76   -443        C
ATOM   1919  CG  GLN A 121     -11.880  32.288   9.596  1.00 34.05           C  0.033
ANISOU 1919  CG  GLN A 121     3998   3679   5262    -36     34   -688        C
ATOM   1920  CD  GLN A 121     -12.831  31.105   9.353  1.00 39.66           C  0.036
ANISOU 1920  CD  GLN A 121     4494   4267   6307    -55     21  -1054        C
ATOM   1921  OE1 GLN A 121     -12.734  30.389   8.352  1.00 42.62           O  0.037
ANISOU 1921  OE1 GLN A 121     4820   4537   6837     58    -47  -1009        O
```

```
ATOM   1922  NE2 GLN A 121     -13.749  30.895  10.296  1.00 40.90           N  0.036
ANISOU 1922  NE2 GLN A 121     4549   4403   6587   -181    138  -1271       N
ATOM   1923  H   GLN A 121      -8.819  32.983   9.968  1.00 21.51           H  0.026
ATOM   1924  HA  GLN A 121      -9.819  30.705   9.374  1.00 24.03           H  0.028
ATOM   1925  HB2 GLN A 121     -10.468  33.286   8.531  1.00 31.22           H  0.032
ATOM   1926  HB3 GLN A 121     -11.120  32.083   7.724  1.00 31.22           H  0.032
ATOM   1927  HG2 GLN A 121     -11.498  32.188  10.482  1.00 40.85           H  0.036
ATOM   1928  HG3 GLN A 121     -12.401  33.105   9.549  1.00 40.85           H  0.036
ATOM   1929 HE21 GLN A 121     -13.782  31.407  10.986  1.00 49.06           H  0.040
ATOM   1930 HE22 GLN A 121     -14.309  30.247  10.214  1.00 49.06           H  0.040
ATOM   1931  N   ALA A 122      -7.888  31.805   7.090  1.00 15.37           N  0.022
ANISOU 1931  N   ALA A 122     2388   2231   1220    -15   -330     91       N
ATOM   1932  CA  ALA A 122      -7.261  31.460   5.830  1.00 16.85           C  0.023
ANISOU 1932  CA  ALA A 122     2495   2426   1481   -104   -346    113       C
ATOM   1933  C   ALA A 122      -6.719  30.048   5.873  1.00 16.67           C  0.023
ANISOU 1933  C   ALA A 122     2450   2471   1414    -86   -695    -64       C
ATOM   1934  O   ALA A 122      -6.688  29.379   4.840  1.00 17.95           O  0.024
ANISOU 1934  O   ALA A 122     2623   2545   1652    -41   -666    133       O
ATOM   1935  CB  ALA A 122      -6.135  32.443   5.493  1.00 17.05           C  0.024
ANISOU 1935  CB  ALA A 122     2424   2423   1633    106   -164      4       C
ATOM   1936  H   ALA A 122      -7.556  32.495   7.481  1.00 18.43           H  0.024
ATOM   1937  HA  ALA A 122      -7.923  31.526   5.124  1.00 20.21           H  0.026
ATOM   1938  HB1 ALA A 122      -5.755  32.204   4.633  1.00 20.45           H  0.026
ATOM   1939  HB2 ALA A 122      -6.500  33.341   5.456  1.00 20.45           H  0.026
ATOM   1940  HB3 ALA A 122      -5.454  32.391   6.181  1.00 20.45           H  0.026
ATOM   1941  N   TRP A 123      -6.292  29.579   7.062  1.00 16.07           N  0.023
ANISOU 1941  N   TRP A 123     2342   2480   1282    -13   -486   -166       N
ATOM   1942  CA  TRP A 123      -5.696  28.251   7.204  1.00 16.38           C  0.023
ANISOU 1942  CA  TRP A 123     2308   2286   1629    -52   -425   -106       C
ATOM   1943  C   TRP A 123      -6.692  27.134   6.933  1.00 17.29           C  0.024
ANISOU 1943  C   TRP A 123     2332   2289   1950    -94   -416   -246       C
ATOM   1944  O   TRP A 123      -6.280  26.022   6.622  1.00 18.45           O  0.024
ANISOU 1944  O   TRP A 123     2446   2397   2168    -22   -350   -455       O
ATOM   1945  CB  TRP A 123      -5.069  28.093   8.611  1.00 16.59           C  0.023
ANISOU 1945  CB  TRP A 123     2441   2252   1609     51   -529    203       C
ATOM   1946  CG  TRP A 123      -3.844  28.942   8.671  1.00 17.19           C  0.024
ANISOU 1946  CG  TRP A 123     2403   2429   1699   -170   -498    177       C
ATOM   1947  CD1 TRP A 123      -3.741  30.149   9.277  1.00 18.57           C  0.025
ANISOU 1947  CD1 TRP A 123     2423   2645   1986   -129   -548    -51       C
ATOM   1948  CD2 TRP A 123      -2.581  28.695   8.034  1.00 17.95           C  0.024
ANISOU 1948  CD2 TRP A 123     2533   2572   1716    -91   -626    421       C
ATOM   1949  NE1 TRP A 123      -2.496  30.682   9.069  1.00 18.15           N  0.024
ANISOU 1949  NE1 TRP A 123     2458   2645   1795   -238   -677    125       N
ATOM   1950  CE2 TRP A 123      -1.763  29.804   8.320  1.00 18.60           C  0.025
ANISOU 1950  CE2 TRP A 123     2472   2616   1980   -126   -807    570       C
ATOM   1951  CE3 TRP A 123      -2.058  27.645   7.262  1.00 19.47           C  0.025
ANISOU 1951  CE3 TRP A 123     2489   2488   2422     40   -519    504       C
ATOM   1952  CZ2 TRP A 123      -0.450  29.900   7.855  1.00 20.18           C  0.026
ANISOU 1952  CZ2 TRP A 123     2424   2659   2586   -132   -592    504       C
ATOM   1953  CZ3 TRP A 123      -0.764  27.747   6.798  1.00 20.76           C  0.026
ANISOU 1953  CZ3 TRP A 123     2489   2511   2888     40   -429    249       C
ATOM   1954  CH2 TRP A 123       0.016  28.879   7.082  1.00 21.76           C  0.027
ANISOU 1954  CH2 TRP A 123     2413   2659   3196   -117   -263    430       C
ATOM   1955  H   TRP A 123      -6.339  30.017   7.801  1.00 19.27           H  0.025
ATOM   1956  HA  TRP A 123      -4.980  28.161   6.555  1.00 19.64           H  0.025
ATOM   1957  HB2 TRP A 123      -5.696  28.387   9.290  1.00 19.89           H  0.025
ATOM   1958  HB3 TRP A 123      -4.824  27.167   8.765  1.00 19.89           H  0.025
ATOM   1959  HD1 TRP A 123      -4.418  30.558   9.765  1.00 22.27           H  0.027
ATOM   1960  HE1 TRP A 123      -2.222  31.443   9.361  1.00 21.77           H  0.027
ATOM   1961  HE3 TRP A 123      -2.574  26.896   7.068  1.00 23.35           H  0.028
ATOM   1962  HZ2 TRP A 123       0.085  30.632   8.064  1.00 24.21           H  0.028
ATOM   1963  HZ3 TRP A 123      -0.403  27.057   6.290  1.00 24.90           H  0.028
ATOM   1964  HH2 TRP A 123       0.876  28.933   6.733  1.00 26.10           H  0.029
ATOM   1965  N   ILE A 124      -7.991  27.394   7.039  1.00 17.23           N  0.024
ANISOU 1965  N   ILE A 124     2238   2380   1930   -136   -473    -91       N
ATOM   1966  CA  ILE A 124      -8.992  26.364   6.801  1.00 18.91           C  0.025
ANISOU 1966  CA  ILE A 124     2442   2586   2156   -376   -710    -33       C
ATOM   1967  C   ILE A 124      -9.806  26.666   5.546  1.00 20.31           C  0.026
ANISOU 1967  C   ILE A 124     2781   2638   2300   -516  -1056     12       C
ATOM   1968  O   ILE A 124     -10.726  25.925   5.216  1.00 20.46           O  0.026
ANISOU 1968  O   ILE A 124     2906   2599   2268   -490   -957    -84       O
ATOM   1969  CB  ILE A 124      -9.885  26.111   8.035  1.00 20.28           C  0.026
ANISOU 1969  CB  ILE A 124     2618   2751   2338   -357   -539    150       C
ATOM   1970  CG1 ILE A 124     -10.741  27.340   8.373  1.00 23.65           C  0.028
ANISOU 1970  CG1 ILE A 124     2907   3000   3080   -332   -220    203       C
ATOM   1971  CG2 ILE A 124      -9.036  25.599   9.221  1.00 20.01           C  0.025
ANISOU 1971  CG2 ILE A 124     2721   2794   2087   -168   -559     62       C
ATOM   1972  CD1 ILE A 124     -11.841  27.049   9.433  1.00 24.41           C  0.028
```

```
ANISOU 1972  CD1 ILE A 124     3181   3039   3056   -369    -65    184       C
ATOM   1973  H   ILE A 124      -8.317  28.162   7.247  1.00 20.67           H  0.026
ATOM   1974  HA  ILE A 124      -8.529  25.526   6.641  1.00 22.68           H  0.027
ATOM   1975  HB  ILE A 124     -10.518  25.408   7.822  1.00 24.33           H  0.028
ATOM   1976 HG12 ILE A 124     -10.165  28.037   8.724  1.00 28.37           H  0.030
ATOM   1977 HG13 ILE A 124     -11.180  27.648   7.564  1.00 28.37           H  0.030
ATOM   1978 HG21 ILE A 124      -9.626  25.379   9.959  1.00 24.00           H  0.028
ATOM   1979 HG22 ILE A 124      -8.547  24.810   8.941  1.00 24.00           H  0.028
ATOM   1980 HG23 ILE A 124      -8.416  26.295   9.489  1.00 24.00           H  0.028
ATOM   1981 HD11 ILE A 124     -12.388  27.842   9.546  1.00 29.28           H  0.031
ATOM   1982 HD12 ILE A 124     -12.389  26.310   9.125  1.00 29.28           H  0.031
ATOM   1983 HD13 ILE A 124     -11.416  26.817  10.273  1.00 29.28           H  0.031
ATOM   1984  N   ARG A 125      -9.479  27.728   4.813  1.00 22.22           N  0.027
ANISOU 1984  N   ARG A 125     3180   3008   2256   -616  -1356    355       N
ATOM   1985  CA  ARG A 125     -10.323  28.155   3.700  1.00 24.65           C  0.028
ANISOU 1985  CA  ARG A 125     3585   3377   2402   -707  -1608    323       C
ATOM   1986  C   ARG A 125     -10.262  27.114   2.585  1.00 24.53           C  0.028
ANISOU 1986  C   ARG A 125     3702   3570   2048   -596  -1412    445       C
ATOM   1987  O   ARG A 125      -9.202  26.573   2.265  1.00 25.33           O  0.029
ANISOU 1987  O   ARG A 125     3794   3807   2023   -698  -1231    129       O
ATOM   1988  CB  ARG A 125      -9.840  29.525   3.222  1.00 28.59           C  0.030
ANISOU 1988  CB  ARG A 125     4103   3670   3089   -790  -1903    518       C
ATOM   1989  CG  ARG A 125     -10.084  29.886   1.768  1.00 33.64           C  0.033
ANISOU 1989  CG  ARG A 125     4548   4067   4165   -895  -1926    286       C
ATOM   1990  CD  ARG A 125      -9.574  31.310   1.472  1.00 37.34           C  0.035
ANISOU 1990  CD  ARG A 125     4938   4431   4820   -930  -1824     47       C
ATOM   1991  NE  ARG A 125      -8.116  31.289   1.428  1.00 40.11           N  0.036
ANISOU 1991  NE  ARG A 125     5240   4797   5202   -865  -1659   -268       N
ATOM   1992  CZ  ARG A 125      -7.326  32.301   1.765  1.00 41.12           C  0.037
ANISOU 1992  CZ  ARG A 125     5399   5101   5123   -816  -1669   -535       C
ATOM   1993  NH1 ARG A 125      -7.813  33.498   2.065  1.00 41.47           N  0.037
ANISOU 1993  NH1 ARG A 125     5439   5105   5212   -797  -1728   -632       N
ATOM   1994  NH2 ARG A 125      -6.008  32.112   1.804  1.00 41.20           N  0.037
ANISOU 1994  NH2 ARG A 125     5504   5310   4840   -661  -1553   -613       N
ATOM   1995  H   ARG A 125      -8.779  28.213   4.937  1.00 26.65           H  0.029
ATOM   1996  HA  ARG A 125     -11.252  28.241   3.965  1.00 29.56           H  0.031
ATOM   1997  HB2 ARG A 125     -10.286  30.201   3.757  1.00 34.29           H  0.033
ATOM   1998  HB3 ARG A 125      -8.881  29.568   3.364  1.00 34.29           H  0.033
ATOM   1999  HG2 ARG A 125      -9.611  29.263   1.194  1.00 40.35           H  0.036
ATOM   2000  HG3 ARG A 125     -11.035  29.854   1.580  1.00 40.35           H  0.036
ATOM   2001  HD2 ARG A 125      -9.911  31.611   0.613  1.00 44.80           H  0.038
ATOM   2002  HD3 ARG A 125      -9.860  31.917   2.172  1.00 44.80           H  0.038
ATOM   2003  HE  ARG A 125      -7.738  30.563   1.163  1.00 48.12           H  0.039
ATOM   2004 HH11 ARG A 125      -8.662  33.635   2.043  1.00 49.75           H  0.040
ATOM   2005 HH12 ARG A 125      -7.280  34.138   2.280  1.00 49.75           H  0.040
ATOM   2006 HH21 ARG A 125      -5.678  31.342   1.611  1.00 49.43           H  0.040
ATOM   2007 HH22 ARG A 125      -5.487  32.761   2.021  1.00 49.43           H  0.040
ATOM   2008  N   GLY A 126     -11.429  26.793   2.034  1.00 24.91           N  0.028
ANISOU 2008  N   GLY A 126     3806   3547   2112   -630  -1436    465       N
ATOM   2009  CA  GLY A 126     -11.548  25.817   0.971  1.00 24.86           C  0.028
ANISOU 2009  CA  GLY A 126     3820   3504   2121   -668  -1164    438       C
ATOM   2010  C   GLY A 126     -11.615  24.368   1.413  1.00 24.40           C  0.028
ANISOU 2010  C   GLY A 126     3561   3457   2253   -657  -1202    259       C
ATOM   2011  O   GLY A 126     -12.008  23.518   0.613  1.00 25.35           O  0.029
ANISOU 2011  O   GLY A 126     3620   3569   2443   -565  -1079    -41       O
ATOM   2012  H   GLY A 126     -12.181  27.138   2.268  1.00 29.88           H  0.031
ATOM   2013  HA2 GLY A 126     -12.355  26.007   0.469  1.00 29.82           H  0.031
ATOM   2014  HA3 GLY A 126     -10.781  25.908   0.384  1.00 29.82           H  0.031
ATOM   2015  N   CYS A 127     -11.261  24.062   2.666  1.00 23.43           N  0.028
ANISOU 2015  N   CYS A 127     3158   3361   2383   -560  -1475    325       N
ATOM   2016  CA  CYS A 127     -11.130  22.674   3.096  1.00 23.66           C  0.028
ANISOU 2016  CA  CYS A 127     2856   3339   2795   -417  -1434    194       C
ATOM   2017  C   CYS A 127     -12.514  22.064   3.259  1.00 26.92           C  0.030
ANISOU 2017  C   CYS A 127     3055   3736   3438   -549  -1340    268       C
ATOM   2018  O   CYS A 127     -13.418  22.706   3.799  1.00 26.97           O  0.030
ANISOU 2018  O   CYS A 127     2958   3612   3679   -459  -1394    459       O
ATOM   2019  CB  CYS A 127     -10.398  22.634   4.429  1.00 22.04           C  0.027
ANISOU 2019  CB  CYS A 127     2627   3075   2673   -183  -1288    -25       C
ATOM   2020  SG  CYS A 127      -8.769  23.361   4.434  1.00 21.77           S  0.027
ANISOU 2020  SG  CYS A 127     2736   2978   2555     41  -1120     52       S
ATOM   2021  H   CYS A 127     -11.093  24.640   3.281  1.00 28.10           H  0.030
ATOM   2022  HA  CYS A 127     -10.630  22.159   2.444  1.00 28.38           H  0.030
ATOM   2023  HB2 CYS A 127     -10.930  23.113   5.084  1.00 26.44           H  0.029
ATOM   2024  HB3 CYS A 127     -10.301  21.706   4.696  1.00 26.44           H  0.029
ATOM   2025  N   ARG A 128     -12.652  20.783   2.871  1.00 29.63           N  0.031
ANISOU 2025  N   ARG A 128     3303   4216   3738   -729  -1138     90       N
ATOM   2026  CA  ARG A 128     -13.853  20.012   3.175  1.00 35.25           C  0.034
ANISOU 2026  CA  ARG A 128     3630   4992   4772   -909  -1103     24       C
```

```
ATOM   2027  C   ARG A 128     -13.824  19.671   4.656  1.00 35.66           C  0.034
ANISOU 2027  C   ARG A 128     3725   5135   4688  -1060  -1152     55       C
ATOM   2028  O   ARG A 128     -12.877  19.035   5.136  1.00 35.66           O  0.034
ANISOU 2028  O   ARG A 128     3794   5076   4680  -1066  -1208     12       O
ATOM   2029  CB  ARG A 128     -13.905  18.741   2.329  1.00 40.76           C  0.036
ANISOU 2029  CB  ARG A 128     3852   5563   6072  -1120   -934    -97       C
ATOM   2030  CG  ARG A 128     -15.127  17.834   2.609  1.00 46.47           C  0.039
ANISOU 2030  CG  ARG A 128     4132   6200   7324  -1157   -872   -140       C
ATOM   2031  CD  ARG A 128     -16.146  17.808   1.456  1.00 51.99           C  0.041
ANISOU 2031  CD  ARG A 128     4440   6792   8523  -1135   -628   -163       C
ATOM   2032  NE  ARG A 128     -17.404  17.138   1.794  1.00 56.43           N  0.043
ANISOU 2032  NE  ARG A 128     4702   7231   9509  -1087   -373   -327       N
ATOM   2033  CZ  ARG A 128     -18.404  17.694   2.469  1.00 59.63           C  0.044
ANISOU 2033  CZ  ARG A 128     4869   7546  10243  -1080   -145   -491       C
ATOM   2034  NH1 ARG A 128     -18.345  18.953   2.892  1.00 61.01           N  0.044
ANISOU 2034  NH1 ARG A 128     4933   7650  10598  -1056    -11   -516       N
ATOM   2035  NH2 ARG A 128     -19.494  16.972   2.722  1.00 60.49           N  0.044
ANISOU 2035  NH2 ARG A 128     4945   7653  10384  -1068   -124   -552       N
ATOM   2036  H   ARG A 128     -12.058  20.344   2.430  1.00 35.54           H  0.034
ATOM   2037  HA  ARG A 128     -14.648  20.528   2.969  1.00 42.29           H  0.037
ATOM   2038  HB2 ARG A 128     -13.936  18.993   1.393  1.00 48.90           H  0.040
ATOM   2039  HB3 ARG A 128     -13.107  18.219   2.506  1.00 48.90           H  0.040
ATOM   2040  HG2 ARG A 128     -14.818  16.926   2.752  1.00 55.75           H  0.043
ATOM   2041  HG3 ARG A 128     -15.584  18.158   3.401  1.00 55.75           H  0.043
ATOM   2042  HD2 ARG A 128     -16.356  18.720   1.204  1.00 62.38           H  0.045
ATOM   2043  HD3 ARG A 128     -15.754  17.337   0.704  1.00 62.38           H  0.045
ATOM   2044  HE  ARG A 128     -17.502  16.324   1.535  1.00 67.71           H  0.047
ATOM   2045 HH11 ARG A 128     -17.647  19.429   2.730  1.00 73.20           H  0.049
ATOM   2046 HH12 ARG A 128     -19.005  19.292   3.327  1.00 73.20           H  0.049
ATOM   2047 HH21 ARG A 128     -19.543  16.158   2.449  1.00 72.57           H  0.049
ATOM   2048 HH22 ARG A 128     -20.149  17.320   3.158  1.00 72.57           H  0.049
ATOM   2049  N   LEU A 129     -14.817  20.152   5.389  1.00 36.68           N  0.034
ANISOU 2049  N   LEU A 129     3807   5407   4722  -1053  -1086    221       N
ATOM   2050  CA  LEU A 129     -14.845  19.955   6.826  1.00 38.96           C  0.036
ANISOU 2050  CA  LEU A 129     4056   5806   4943   -893  -1068    293       C
ATOM   2051  C   LEU A 129     -16.255  19.650   7.315  1.00 42.96           C  0.037
ANISOU 2051  C   LEU A 129     4315   6235   5774   -876  -1143    295       C
ATOM   2052  O   LEU A 129     -17.216  19.646   6.546  1.00 44.41           O  0.038
ANISOU 2052  O   LEU A 129     4382   6384   6108   -858  -1300    260       O
ATOM   2053  CB  LEU A 129     -14.339  21.199   7.574  1.00 37.62           C  0.035
ANISOU 2053  CB  LEU A 129     4066   5783   4445   -725   -936    464       C
ATOM   2054  CG  LEU A 129     -12.902  21.621   7.341  1.00 36.30           C  0.034
ANISOU 2054  CG  LEU A 129     4049   5736   4009   -583   -943    596       C
ATOM   2055  CD1 LEU A 129     -12.582  23.031   7.872  1.00 36.77           C  0.035
ANISOU 2055  CD1 LEU A 129     4210   5750   4012   -556   -739    548       C
ATOM   2056  CD2 LEU A 129     -11.949  20.613   7.998  1.00 35.97           C  0.034
ANISOU 2056  CD2 LEU A 129     3990   5752   3926   -441   -993    684       C
ATOM   2057  OXT LEU A 129     -16.441  19.421   8.508  1.00 44.43           O  0.038
ANISOU 2057  OXT LEU A 129     4429   6399   6054   -874  -1109    209       O
ATOM   2058  H   LEU A 129     -15.485  20.595   5.076  1.00 44.00           H  0.038
ATOM   2059  HA  LEU A 129     -14.261  19.208   7.029  1.00 46.75           H  0.039
ATOM   2060  HB2 LEU A 129     -14.897  21.948   7.315  1.00 45.13           H  0.038
ATOM   2061  HB3 LEU A 129     -14.431  21.030   8.525  1.00 45.13           H  0.038
ATOM   2062  HG  LEU A 129     -12.768  21.645   6.380  1.00 43.55           H  0.038
ATOM   2063 HD11 LEU A 129     -11.702  23.294   7.561  1.00 44.11           H  0.038
ATOM   2064 HD12 LEU A 129     -13.249  23.653   7.541  1.00 44.11           H  0.038
ATOM   2065 HD13 LEU A 129     -12.598  23.014   8.842  1.00 44.11           H  0.038
ATOM   2066 HD21 LEU A 129     -11.035  20.910   7.866  1.00 43.15           H  0.037
ATOM   2067 HD22 LEU A 129     -12.147  20.562   8.946  1.00 43.15           H  0.037
ATOM   2068 HD23 LEU A 129     -12.077  19.743   7.587  1.00 43.15           H  0.037
TER
HETATM 2069 CL    CL A1130      -7.784  32.278  26.220  1.00 18.20          Cl  0.024
ANISOU 2069 CL    CL A1130     2574   2273   2066    161    356   -274      Cl
HETATM 2070 CL    CL A1131     -10.557  29.769  11.934  0.56 17.44          Cl  0.024
ANISOU 2070 CL    CL A1131     1952   2911   1762    601   -131    365      Cl
HETATM 2071 CL    CL A1132      13.514  13.308  32.434  0.68 18.54          Cl  0.025
ANISOU 2071 CL    CL A1132     2593   2647   1803    512    506    517      Cl
HETATM 2072 CL    CL A1134       0.546  25.087   3.779  1.00 72.86          Cl  0.049
ANISOU 2072 CL    CL A1134    11566   8215   7903   2929   3298   3974      Cl
HETATM 2073 CL    CL A1135       7.451   7.524  29.606  1.00 41.33          Cl  0.037
ANISOU 2073 CL    CL A1135     7061   3539   5103    818  -1641    710      Cl
HETATM 2074 NA    NA A1138       9.655  15.845  31.353  1.00 20.85          Na  0.026
ANISOU 2074 NA    NA A1138     2715   2960   2249    417    253    273      Na
HETATM 2075  C1  RII A2143     -11.985  35.731   4.437  0.48 16.93      A    C  0.023
ANISOU 2075  C1  RII A2143     2659   2489   1283    232   -538    322  A    C
HETATM 2076  C2  RII A2143      -9.383  36.187   3.935  0.48 14.95      A    C  0.022
ANISOU 2076  C2  RII A2143     2256   2282   1140   -115    -45    174  A    C
HETATM 2077  C3  RII A2143     -10.227  34.150   5.414  0.48 16.11      A    C  0.023
```

```
ANISOU 2077  C3  RII A2143     2282   2360   1480    -22   -881    -29  A    C
HETATM 2078  C4  RII A2143     -11.432  36.191   8.454  0.48 17.85      A    C  0.024
ANISOU 2078  C4  RII A2143     2430   2661   1692    -46     93    279  A    C
HETATM 2079  C5  RII A2143     -13.539  35.619   8.477  0.48 18.50      A    C  0.024
ANISOU 2079  C5  RII A2143     2467   2934   1629   -123    274    209  A    C
HETATM 2080  C6  RII A2143     -13.078  35.562   7.236  0.48 18.29      A    C  0.024
ANISOU 2080  C6  RII A2143     2353   2834   1763    -34     22    275  A    C
HETATM 2081  C7  RII A2143     -10.355  39.047   6.349  0.48 16.05      A    C  0.023
ANISOU 2081  C7  RII A2143     2544   2155   1400    220   -747     47  A    C
HETATM 2082  C8  RII A2143     -11.548  40.230   4.979  0.48 16.70      A    C  0.023
ANISOU 2082  C8  RII A2143     2395   2270   1680    113   -474    143  A    C
HETATM 2083  C9  RII A2143     -11.597  38.932   4.606  0.48 16.66      A    C  0.023
ANISOU 2083  C9  RII A2143     2397   2166   1766    244   -490    249  A    C
HETATM 2084  N1  RII A2143     -11.735  35.941   7.219  0.48 17.05      A    N  0.024
ANISOU 2084  N1  RII A2143     2282   2514   1681     68   -545    333  A    N
HETATM 2085  N2  RII A2143     -12.497  36.060   9.241  0.48 18.50      A    N  0.024
ANISOU 2085  N2  RII A2143     2567   2929   1532    -97    320    132  A    N
HETATM 2086  N3  RII A2143     -10.841  38.176   5.497  0.48 15.63      A    N  0.023
ANISOU 2086  N3  RII A2143     2453   2089   1398    223   -566    120  A    N
HETATM 2087  N4  RII A2143     -10.793  40.286   6.055  0.48 17.20      A    N  0.024
ANISOU 2087  N4  RII A2143     2624   2246   1664    172   -489    104  A    N
HETATM 2088  O1  RII A2143     -12.896  35.565   3.762  0.48 17.68      A    O  0.024
ANISOU 2088  O1  RII A2143     2707   2599   1410    187   -740    219  A    O
HETATM 2089  O2  RII A2143      -8.687  36.279   3.017  0.48 16.88      A    O  0.023
ANISOU 2089  O2  RII A2143     2497   2408   1508   -104    243    422  A    O
HETATM 2090  O3  RII A2143     -10.098  33.025   5.317  0.48 18.29      A    O  0.024
ANISOU 2090  O3  RII A2143     2412   2567   1972      5   -682    -17  A    O
HETATM 2091 RE1  RII A2143     -10.492  36.038   5.472  0.48 15.85      A   Re  0.023
ANISOU 2091 RE1  RII A2143     2331   2307   1384    125   -467    221  A   Re
HETATM 2092  H21 RII A2143     -12.523  36.225  10.085  0.48 22.18      A    H  0.027
HETATM 2093  H41 RII A2143     -10.584  36.430   8.750  0.48 21.41      A    H  0.026
HETATM 2094  H42 RII A2143     -10.608  40.998   6.500  0.48 20.63      A    H  0.026
HETATM 2095  H51 RII A2143     -14.394  35.404   8.767  0.48 22.19      A    H  0.027
HETATM 2096  H61 RII A2143     -13.573  35.308   6.491  0.48 21.94      A    H  0.027
HETATM 2097  H71 RII A2143      -9.787  38.841   7.057  0.48 19.25      A    H  0.025
HETATM 2098  H81 RII A2143     -11.966  40.944   4.557  0.48 20.02      A    H  0.025
HETATM 2099  H91 RII A2143     -12.059  38.598   3.873  0.48 19.98      A    H  0.025
HETATM 2100  C1  RII A2144       1.345  23.041  36.499  0.44 17.51      A    C  0.024
ANISOU 2100  C1  RII A2144     2590   2906   1156    581    -74    687  A    C
HETATM 2101  C2  RII A2144      -1.216  23.557  36.513  0.44 18.40      A    C  0.024
ANISOU 2101  C2  RII A2144     2558   2876   1559    487    489    562  A    C
HETATM 2102  C3  RII A2144      -0.173  22.243  34.452  0.44 18.55      A    C  0.025
ANISOU 2102  C3  RII A2144     2604   2624   1818    382    272    260  A    C
HETATM 2103  C4  RII A2144       2.023  25.528  33.287  0.44 16.47      A    C  0.023
ANISOU 2103  C4  RII A2144     1648   2418   2192    866    505    461  A    C
HETATM 2104  C5  RII A2144       3.748  24.322  32.801  0.44 16.92      A    C  0.023
ANISOU 2104  C5  RII A2144     1867   2694   1870    475    295    582  A    C
HETATM 2105  C6  RII A2144       2.987  23.643  33.640  0.44 18.44      A    C  0.024
ANISOU 2105  C6  RII A2144     2045   2698   2264    541    265    551  A    C
HETATM 2106  C7  RII A2144      -0.119  26.994  35.810  0.44 18.86      A    C  0.025
ANISOU 2106  C7  RII A2144     2843   2787   1536    658    -71    288  A    C
HETATM 2107  C8  RII A2144       1.357  27.578  37.256  0.44 21.18      A    C  0.026
ANISOU 2107  C8  RII A2144     3023   2922   2104    593   -137    306  A    C
HETATM 2108  C9  RII A2144       1.479  26.260  37.010  0.44 20.69      A    C  0.026
ANISOU 2108  C9  RII A2144     2869   2906   2085    583   -255    385  A    C
HETATM 2109  N1  RII A2144       1.869  24.400  33.960  0.44 18.80      A    N  0.025
ANISOU 2109  N1  RII A2144     2195   2658   2289    509     32    388  A    N
HETATM 2110  N2  RII A2144       3.134  25.515  32.566  0.44 16.41      A    N  0.023
ANISOU 2110  N2  RII A2144     1727   2608   1899    367    915     59  A    N
HETATM 2111  N3  RII A2144       0.529  25.891  36.082  0.44 17.46      A    N  0.024
ANISOU 2111  N3  RII A2144     2634   2872   1126    661    -99    501  A    N
HETATM 2112  N4  RII A2144       0.370  28.018  36.506  0.44 20.37      A    N  0.026
ANISOU 2112  N4  RII A2144     3018   2898   1823    561   -102    171  A    N
HETATM 2113  O1  RII A2144       2.045  22.507  37.264  0.44 19.49      A    O  0.025
ANISOU 2113  O1  RII A2144     2855   3179   1373    610   -217    635  A    O
HETATM 2114  O2  RII A2144      -2.052  23.348  37.258  0.44 20.11      A    O  0.026
ANISOU 2114  O2  RII A2144     2733   3053   1856    329    590    780  A    O
HETATM 2115  O3  RII A2144      -0.422  21.254  33.976  0.44 19.61      A    O  0.025
ANISOU 2115  O3  RII A2144     2744   2581   2124    352    334    378  A    O
HETATM 2116 RE1  RII A2144       0.204  23.910  35.277  0.44 17.47      A   Re  0.024
ANISOU 2116 RE1  RII A2144     2487   2642   1508    509    278    451  A   Re
HETATM 2117  H21 RII A2144       3.424  26.147  32.062  0.44 19.67      A    H  0.025
HETATM 2118  H41 RII A2144       1.422  26.236  33.302  0.44 19.75      A    H  0.025
HETATM 2119  H42 RII A2144       0.085  28.828  36.472  0.44 24.43      A    H  0.028
HETATM 2120  H51 RII A2144       4.544  24.040  32.431  0.44 20.30      A    H  0.026
HETATM 2121  H61 RII A2144       3.172  22.790  33.959  0.44 22.12      A    H  0.027
HETATM 2122  H71 RII A2144      -0.825  27.056  35.208  0.44 22.62      A    H  0.027
HETATM 2123  H81 RII A2144       1.875  28.082  37.839  0.44 25.41      A    H  0.029
```

```
HETATM 2124  H91 RII A2144       2.098  25.690  37.402  0.44 24.81      A    H  0.028
HETATM 2125  C1  RI3 A2145      -7.708   9.142  13.073  0.68 27.17      A    C  0.030
ANISOU 2125  C1  RI3 A2145     3538   2559   4228   -186  -1303   -195  A    C
HETATM 2126  C2  RI3 A2145      -8.309  11.711  12.980  0.68 25.13      A    C  0.029
ANISOU 2126  C2  RI3 A2145     3375   2356   3818   -357  -1653   -174  A    C
HETATM 2127  C3  RI3 A2145      -5.937  10.920  13.868  0.68 24.79      A    C  0.028
ANISOU 2127  C3  RI3 A2145     3330   2347   3741   -257  -1430   -307  A    C
HETATM 2128  C7  RI3 A2145     -10.501  11.017  15.752  0.68 30.29      A    C  0.031
ANISOU 2128  C7  RI3 A2145     3510   2818   5179   -292   -945   -186  A    C
HETATM 2129  C8  RI3 A2145     -11.763   9.328  15.345  0.68 30.16      A    C  0.031
ANISOU 2129  C8  RI3 A2145     3432   2763   5263   -319   -821   -159  A    C
HETATM 2130  C9  RI3 A2145     -10.570   9.137  14.754  0.68 29.82      A    C  0.031
ANISOU 2130  C9  RI3 A2145     3464   2773   5093   -291   -957   -169  A    C
HETATM 2131  N3  RI3 A2145      -9.762  10.222  15.027  0.68 28.08      A    N  0.030
ANISOU 2131  N3  RI3 A2145     3416   2606   4649   -258  -1258   -220  A    N
HETATM 2132  N4  RI3 A2145     -11.705  10.490  15.954  0.68 31.12      A    N  0.032
ANISOU 2132  N4  RI3 A2145     3524   2894   5406   -256   -830   -131  A    N
HETATM 2133  O1  RI3 A2145      -7.672   8.297  12.275  0.68 28.45      A    O  0.030
ANISOU 2133  O1  RI3 A2145     3721   2751   4338   -200  -1217   -278  A    O
HETATM 2134  O2  RI3 A2145      -8.663  12.413  12.172  0.68 26.21      A    O  0.029
ANISOU 2134  O2  RI3 A2145     3532   2385   4040   -437  -1508   -159  A    O
HETATM 2135  O3  RI3 A2145      -4.903  11.170  13.523  0.68 25.22      A    O  0.029
ANISOU 2135  O3  RI3 A2145     3473   2583   3526   -180  -1516   -450  A    O
HETATM 2136  O4  RI3 A2145      -7.197   9.154  15.961  0.68 27.36      A    O  0.030
ANISOU 2136  O4  RI3 A2145     3347   2538   4511   -161  -1113   -194  A    O
HETATM 2137 RE1  RI3 A2145      -7.724  10.519  14.354  0.68 25.99      A   Re  0.029
ANISOU 2137 RE1  RI3 A2145     3357   2390   4127   -218  -1346   -124  A   Re
HETATM 2138  H42 RI3 A2145     -12.339  10.853  16.407  0.68 37.33      A    H  0.035
HETATM 2139  H71 RI3 A2145     -10.226  11.841  16.088  0.68 36.33      A    H  0.034
HETATM 2140  H81 RI3 A2145     -12.489   8.751  15.323  0.68 36.18      A    H  0.034
HETATM 2141  H91 RI3 A2145     -10.325   8.396  14.253  0.68 35.77      A    H  0.034
HETATM 2142 RE    RE A2146      11.528  25.511  22.356  0.08 28.89          Re  0.031
ANISOU 2142 RE    RE A2146     4701   3549   2727   1072  -1103   -506          Re
HETATM 2143  C4  RRE A2147     -13.947  26.381  12.374  0.17 20.46      A    C  0.026
HETATM 2144  C5  RRE A2147     -14.522  24.106  11.264  0.17 21.13      A    C  0.026
HETATM 2145  C6  RRE A2147     -16.045  26.208  10.910  0.17 17.60      A    C  0.024
HETATM 2146  O11 RRE A2147     -16.551  26.397  13.946  0.17 20.09      A    O  0.026
HETATM 2147  O12 RRE A2147     -17.205  24.025  12.750  0.17 23.06      A    O  0.027
HETATM 2148  O4  RRE A2147     -13.022  27.098  12.358  0.17 19.50      A    O  0.025
HETATM 2149  O5  RRE A2147     -13.968  23.385  10.549  0.17 34.12      A    O  0.033
HETATM 2150  O6  RRE A2147     -16.376  26.812   9.963  0.17 29.76      A    O  0.031
HETATM 2151 RE2  RRE A2147     -15.436  25.250  12.449  0.17 25.15      A   Re  0.029
HETATM 2152 CL    CL C   1       2.686  26.707  29.517  0.50 24.56          Cl  0.028
ANISOU 2152 CL    CL C   1     3427   3676   2229   -249   -293    317          Cl
HETATM 2153 CL    CL C   2       7.342  19.483   6.983  0.65 26.25          Cl  0.029
ANISOU 2153 CL    CL C   2     3579   3555   2839    747    140     31          Cl
HETATM 2154 CL    CL C   3      20.107  11.805  24.379  0.57 24.01          Cl  0.028
ANISOU 2154 CL    CL C   3     3586   2972   2564   -190    704   -179          Cl
HETATM 2155 CL    CL C   4      -9.729  34.808  11.516  1.00 36.72          Cl  0.035
ANISOU 2155 CL    CL C   4     3788   4518   5647    575   -766    164          Cl
HETATM 2156  O   HOH S   1       7.273  12.713   9.058  1.00 17.83           O  0.024
ANISOU 2156  O   HOH S   1     2514   2251   2009    564   -812   -317           O
HETATM 2157  O   HOH S   2       4.892   9.020  10.375  1.00 37.40           O  0.035
ANISOU 2157  O   HOH S   2     4220   2725   7267    -36  -2921    516           O
HETATM 2158  O   HOH S   3       3.991  12.195   5.071  1.00 33.84           O  0.033
ANISOU 2158  O   HOH S   3     6137   3483   3237     22    338  -1786           O
HETATM 2159  O   HOH S   4       1.932  20.123   4.118  1.00 30.65           O  0.032
ANISOU 2159  O   HOH S   4     3914   5426   2305   -441    -51  -1417           O
HETATM 2160  O   HOH S   5      -5.991  21.923  -0.115  1.00 36.98           O  0.035
ANISOU 2160  O   HOH S   5     6760   5200   2092   1012   -268   -139           O
HETATM 2161  O   HOH S   6      -4.314  21.090   1.844  1.00 29.27           O  0.031
ANISOU 2161  O   HOH S   6     3208   5472   2442    377   -432  -1378           O
HETATM 2162  O   HOH S   8      -4.623  18.501   2.643  1.00 24.51           O  0.028
ANISOU 2162  O   HOH S   8     3537   3851   1925    759   -330   -263           O
HETATM 2163  O   HOH S  10     -13.568  24.112  25.190  1.00 27.58           O  0.030
ANISOU 2163  O   HOH S  10     2680   4099   3701   -381   1111    793           O
HETATM 2164  O   HOH S  11     -10.103  31.898  23.913  1.00 20.69           O  0.026
ANISOU 2164  O   HOH S  11     2925   2679   2259    189    256    117           O
HETATM 2165  O   HOH S  12      -5.781  34.534  16.896  1.00 20.59           O  0.026
ANISOU 2165  O   HOH S  12     3942   2307   1575    180   -545   -232           O
HETATM 2166  O   HOH S  13      -8.953  33.847  16.033  1.00 27.35           O  0.030
ANISOU 2166  O   HOH S  13     5127   3034   2231   1726   1190    354           O
HETATM 2167  O   HOH S  14       6.848  26.290   9.106  1.00 38.04           O  0.035
ANISOU 2167  O   HOH S  14     4523   3636   6294    239   1925    590           O
HETATM 2168  O   HOH S  15      -2.851  10.580  25.041  1.00 33.61           O  0.033
ANISOU 2168  O   HOH S  15     4843   3321   4605   -987    980    372           O
HETATM 2169  O   HOH S  16       8.498  27.757  15.590  1.00 20.32           O  0.026
ANISOU 2169  O   HOH S  16     2691   3105   1926   -285   -208     45           O
```

```
HETATM 2170  O   HOH S  17       8.295  26.640  11.449  1.00 23.84           O  0.028
ANISOU 2170  O   HOH S  17     3300   3172   2585  -1111    626   -380       O
HETATM 2171  O   HOH S  18       8.599  25.806  19.830  1.00 25.80           O  0.029
ANISOU 2171  O   HOH S  18     2437   3773   3594   -290   -186   -510       O
HETATM 2172  O   HOH S  19       6.659  28.240  19.950  1.00 21.33           O  0.026
ANISOU 2172  O   HOH S  19     3255   2647   2203    327     97   -130       O
HETATM 2173  O   HOH S  20       9.607  20.183  10.726  1.00 26.63           O  0.029
ANISOU 2173  O   HOH S  20     2774   4734   2610    722    -61    394       O
HETATM 2174  O   HOH S  21       4.274  21.316   5.077  1.00 29.94           O  0.031
ANISOU 2174  O   HOH S  21     3775   5289   2311    -85     -3  -1202       O
HETATM 2175  O   HOH S  22      -0.759  40.241  19.385  1.00 21.39           O  0.026
ANISOU 2175  O   HOH S  22     3062   2913   2154    235     83   -211       O
HETATM 2176  O   HOH S  23      -2.585  32.678   5.311  1.00 23.56           O  0.028
ANISOU 2176  O   HOH S  23     2570   3688   2693   -494   -370     80       O
HETATM 2177  O   HOH S  24      -1.950  36.754   7.388  1.00 26.87           O  0.030
ANISOU 2177  O   HOH S  24     3086   4316   2806   -653   -115   -188       O
HETATM 2178  O   HOH S  25      16.288  17.398  17.371  1.00 25.52           O  0.029
ANISOU 2178  O   HOH S  25     2439   3567   3690    257    175   1125       O
HETATM 2179  O   HOH S  26      12.971  14.970  14.502  1.00 16.99           O  0.023
ANISOU 2179  O   HOH S  26     2623   2490   1344    593   -140   -187       O
HETATM 2180  O   HOH S  28      17.268  19.995  17.630  1.00 22.59           O  0.027
ANISOU 2180  O   HOH S  28     3845   2870   1867    480   1127    -29       O
HETATM 2181  O   HOH S  30      17.454  19.984  31.129  1.00 36.22           O  0.034
ANISOU 2181  O   HOH S  30     3898   4307   5558   -566   -610    366       O
HETATM 2182  O   HOH S  31      15.356  16.552  26.223  1.00 14.97           O  0.022
ANISOU 2182  O   HOH S  31     2175   2435   1077    346     79    134       O
HETATM 2183  O   HOH S  32       9.666  25.309  23.741  1.00 41.84           O  0.037
ANISOU 2183  O   HOH S  32     7359   3966   4574   1574   -320  -1965       O
HETATM 2184  O   HOH S  33       3.030  17.995  19.442  1.00 14.44           O  0.022
ANISOU 2184  O   HOH S  33     2018   2199   1270    222    -50    123       O
HETATM 2185  O   HOH S  34      10.336  24.196  18.429  1.00 22.72           O  0.027
ANISOU 2185  O   HOH S  34     2583   2553   3497   -151   -398   -530       O
HETATM 2186  O   HOH S  35       6.699  24.960  21.449  1.00 32.55           O  0.032
ANISOU 2186  O   HOH S  35     5079   2430   4857    641   1981    282       O
HETATM 2187  O   HOH S  36       5.444  23.025  25.040  1.00 27.37           O  0.030
ANISOU 2187  O   HOH S  36     4040   3048   3312    836   -772    270       O
HETATM 2188  O   HOH S  37       4.741  25.151  28.048  1.00 39.69           O  0.036
ANISOU 2188  O   HOH S  37     6441   2947   5693   -136   2087    838       O
HETATM 2189  O   HOH S  38      12.414  10.680  33.901  1.00 42.13           O  0.037
ANISOU 2189  O   HOH S  38     5883   6969   3154   2325   1055   1350       O
HETATM 2190  O   HOH S  39      12.280   7.410  30.854  1.00 20.79           O  0.026
ANISOU 2190  O   HOH S  39     3135   2710   2056    903    882    706       O
HETATM 2191  O   HOH S  40      11.404  14.438  30.364  1.00 18.04           O  0.024
ANISOU 2191  O   HOH S  40     2936   2609   1310    749    142    128       O
HETATM 2192  O   HOH S  41      16.416   9.000  33.097  1.00 30.10           O  0.031
ANISOU 2192  O   HOH S  41     5093   3548   2796    909    719    706       O
HETATM 2193  O   HOH S  42       9.592  13.977  32.679  1.00 26.07           O  0.029
ANISOU 2193  O   HOH S  42     2656   5062   2188   1222    275    689       O
HETATM 2194  O   HOH S  43       6.882  10.267  34.098  1.00 30.43           O  0.031
ANISOU 2194  O   HOH S  43     5078   3909   2575    768   -821    391       O
HETATM 2195  O   HOH S  44       5.326   7.609  18.112  1.00 27.55           O  0.030
ANISOU 2195  O   HOH S  44     4274   3096   3098   -186   -562     82       O
HETATM 2196  O   HOH S  45       0.702   8.255  20.147  1.00 28.20           O  0.030
ANISOU 2196  O   HOH S  45     3455   4130   3128    530   -252   -196       O
HETATM 2197  O   HOH S  46       2.697  15.251  16.501  1.00 27.05           O  0.030
ANISOU 2197  O   HOH S  46     4367   3483   2429  -1560  -1482    717       O
HETATM 2198  O   HOH S  47       5.670   8.939  13.112  1.00 26.02           O  0.029
ANISOU 2198  O   HOH S  47     3014   3313   3560    609   -461   -192       O
HETATM 2199  O   HOH S  48       0.846  13.434  16.132  1.00 21.04           O  0.026
ANISOU 2199  O   HOH S  48     2501   2488   3007   -251   -483   -217       O
HETATM 2200  O   HOH S  49       3.148   6.729  13.939  1.00 35.81           O  0.034
ANISOU 2200  O   HOH S  49     5472   3187   4948    967  -2037  -1386       O
HETATM 2201  O   HOH S  50      -3.015  10.511  10.708  1.00 34.40           O  0.033
ANISOU 2201  O   HOH S  50     3527   6045   3497   -385   -769   -751       O
HETATM 2202  O   HOH S  51      -1.442   9.494  19.294  1.00 27.55           O  0.030
ANISOU 2202  O   HOH S  51     3471   3028   3969    543   -184   -376       O
HETATM 2203  O   HOH S  52       0.428  12.420  18.768  1.00 22.38           O  0.027
ANISOU 2203  O   HOH S  52     2947   3242   2314    721    -56    342       O
HETATM 2204  O   HOH S  53      -1.477  12.897  25.451  1.00 25.73           O  0.029
ANISOU 2204  O   HOH S  53     3198   3241   3337    287    336   1310       O
HETATM 2205  O   HOH S  54      -6.705  16.430  27.560  1.00 29.41           O  0.031
ANISOU 2205  O   HOH S  54     2897   5562   2714   -482    362   1068       O
HETATM 2206  O   HOH S  55      -7.619  13.480  20.619  1.00 27.32           O  0.030
ANISOU 2206  O   HOH S  55     4212   2734   3435    -76   -488    366       O
HETATM 2207  O   HOH S  56      -9.362  14.866  23.664  1.00 31.40           O  0.032
ANISOU 2207  O   HOH S  56     3228   2957   5744   -222   -850    965       O
HETATM 2208  O   HOH S  57      -4.787  22.273  32.096  1.00 33.13           O  0.033
ANISOU 2208  O   HOH S  57     5651   5044   1894    -20    731    912       O
```

```
HETATM 2209  O   HOH S  58      -5.684  28.946  29.416  1.00 19.59           O  0.025
ANISOU 2209  O   HOH S  58     2821   2536   2087     54    173    495       O
HETATM 2210  O   HOH S  59      -1.709  28.241  28.554  1.00 27.87           O  0.030
ANISOU 2210  O   HOH S  59     4271   3458   2861   1243    879   1028       O
HETATM 2211  O   HOH S  60       4.158  33.461  24.422  1.00 32.99           O  0.033
ANISOU 2211  O   HOH S  60     4420   5226   2889  -1286   -650    399       O
HETATM 2212  O   HOH S  61       6.545  27.793  22.677  1.00 28.54           O  0.030
ANISOU 2212  O   HOH S  61     3213   5145   2486   1726   -245    606       O
HETATM 2213  O   HOH S  63       1.644  38.997  18.585  1.00 27.37           O  0.030
ANISOU 2213  O   HOH S  63     3661   2591   4147    102   -583   -644       O
HETATM 2214  O   HOH S  64       4.562  39.151  19.282  1.00 34.50           O  0.033
ANISOU 2214  O   HOH S  64     6381   3337   3392    233   -421   -494       O
HETATM 2215  O   HOH S  65       8.740  29.621   8.547  1.00 18.18           O  0.024
ANISOU 2215  O   HOH S  65     2655   2193   2058   -246    150   -173       O
HETATM 2216  O   HOH S  66       2.009  34.901   9.977  1.00 20.47           O  0.026
ANISOU 2216  O   HOH S  66     3144   2838   1797    -63    184    336       O
HETATM 2217  O   HOH S  67       4.886  34.364  10.047  1.00 16.93           O  0.023
ANISOU 2217  O   HOH S  67     2513   2580   1341   -424   -153    307       O
HETATM 2218  O   HOH S  68       1.096  37.336  15.518  1.00 16.39           O  0.023
ANISOU 2218  O   HOH S  68     2418   2239   1569   -140    152    156       O
HETATM 2219  O   HOH S  69      -1.136  40.223  14.795  1.00 23.84           O  0.028
ANISOU 2219  O   HOH S  69     3382   2923   2755    105    325    563       O
HETATM 2220  O   HOH S  70       0.086  39.751  22.067  1.00 21.04           O  0.026
ANISOU 2220  O   HOH S  70     3161   2702   2133   -433    249     27       O
HETATM 2221  O   HOH S  71      -3.387  40.183  18.282  1.00 21.59           O  0.026
ANISOU 2221  O   HOH S  71     2913   3297   1995    278    106   -511       O
HETATM 2222  O   HOH S  72      -3.228  41.809  14.122  1.00 35.05           O  0.034
ANISOU 2222  O   HOH S  72     5750   2954   4615   1287   -224    718       O
HETATM 2223  O   HOH S  73      -7.230  40.524  14.928  1.00 35.55           O  0.034
ANISOU 2223  O   HOH S  73     6205   4191   3111   1982  -1077   -139       O
HETATM 2224  O   HOH S  74      -2.226  33.523   9.697  1.00 18.35           O  0.024
ANISOU 2224  O   HOH S  74     2690   2426   1858    -68   -111   -235       O
HETATM 2225  O   HOH S  75      -6.863  36.090  14.891  1.00 20.52           O  0.026
ANISOU 2225  O   HOH S  75     3758   2384   1656    380    -83    357       O
HETATM 2226  O   HOH S  76      -3.783  38.408   8.499  1.00 22.65           O  0.027
ANISOU 2226  O   HOH S  76     3776   3025   1806   -457    -90    424       O
HETATM 2227  O   HOH S  77      -3.553  34.350   7.442  1.00 25.51           O  0.029
ANISOU 2227  O   HOH S  77     3275   3994   2422   -504    179    -52       O
HETATM 2228  O   HOH S  78       0.312  33.375   8.459  1.00 23.72           O  0.028
ANISOU 2228  O   HOH S  78     3213   3510   2290    229   -566     -5       O
HETATM 2229  O   HOH S  79     -10.400  18.909   4.931  1.00 36.24           O  0.034
ANISOU 2229  O   HOH S  79     4337   4525   4907    250  -1922   -366       O
HETATM 2230  O   HOH S  80     -10.326  19.501   1.325  1.00 33.94           O  0.033
ANISOU 2230  O   HOH S  80     4374   4730   3789    167   -420  -1448       O
HETATM 2231  O   HOH S  81      -2.778  29.998   5.009  1.00 29.01           O  0.031
ANISOU 2231  O   HOH S  81     3999   3188   3835   -596  -1618   1153       O
HETATM 2232  O   HOH S  82       2.455  29.746  29.102  1.00 26.49           O  0.029
ANISOU 2232  O   HOH S  82     4869   3122   2073    472   -660   -668       O
HETATM 2233  O   HOH S  84      -2.237  13.669  27.870  1.00 25.67           O  0.029
ANISOU 2233  O   HOH S  84     2436   4379   2937    211     25   1038       O
HETATM 2234  O   HOH S  85       2.111  33.209  33.266  1.00 30.25           O  0.031
ANISOU 2234  O   HOH S  85     3757   5009   2727    114   -671  -1874       O
HETATM 2235  O   HOH S  86      -0.226  42.856  19.001  1.00 29.71           O  0.031
ANISOU 2235  O   HOH S  86     3184   4156   3950    917      0    541       O
HETATM 2236  O   HOH S  88       9.005  28.488  18.490  1.00 30.63           O  0.032
ANISOU 2236  O   HOH S  88     3082   5179   3377    169    447    387       O
HETATM 2237  O   HOH S  89      14.050   8.059  33.114  1.00 37.86           O  0.035
ANISOU 2237  O   HOH S  89     7470   4122   2792   1149   1530    654       O
HETATM 2238  O   HOH S  90      -0.235  37.634  25.783  1.00 32.94           O  0.033
ANISOU 2238  O   HOH S  90     4730   4351   3435   1109  -1319  -1999       O
HETATM 2239  O   HOH S  91      20.878  21.717  24.435  1.00 38.50           O  0.035
ANISOU 2239  O   HOH S  91     4397   4751   5478   -544    453   -950       O
HETATM 2240  O   HOH S  96     -13.556  20.159  16.782  1.00 28.17           O  0.030
ANISOU 2240  O   HOH S  96     2135   3785   4782   -306   -328  -1092       O
HETATM 2241  O   HOH S  97       7.878  19.275   4.424  1.00 37.69           O  0.035
ANISOU 2241  O   HOH S  97     5877   5493   2949   -420   -306  -1171       O
HETATM 2242  O   HOH S  98       5.868  41.624  19.045  1.00 34.70           O  0.034
ANISOU 2242  O   HOH S  98     5395   3949   3840    757    604    351       O
HETATM 2243  O   HOH S  99      14.815  21.653  31.299  1.00 37.10           O  0.035
ANISOU 2243  O   HOH S  99     5651   3550   4895   -846   -917    308       O
HETATM 2244  O   HOH S 100      -8.334  29.384  30.651  1.00 38.46           O  0.035
ANISOU 2244  O   HOH S 100     4656   4806   5151   1336   1234  -1050       O
HETATM 2245  O   HOH S 101      -7.605  30.131  33.447  1.00 41.31           O  0.037
ANISOU 2245  O   HOH S 101     3716   6123   5858    233   1434    926       O
HETATM 2246  O   HOH S 102      -0.450  21.837   2.730  1.00 43.95           O  0.038
ANISOU 2246  O   HOH S 102     7985   4209   4506   1840   2523   1225       O
HETATM 2247  O   HOH S 106     -12.602  17.515  23.947  1.00 36.74           O  0.035
ANISOU 2247  O   HOH S 106     2980   4572   6406   -588    608    -79       O
```

```
HETATM 2248  O   HOH S 113      19.974  19.974  18.544  1.00 36.66           O  0.034
ANISOU 2248  O   HOH S 113     3813   3813   6304     27   1884  -1884       O
HETATM 2249  O   HOH S 117      14.485  14.485  37.089  1.00 26.44           O  0.029
ANISOU 2249  O   HOH S 117     3322   3322   3401   -506     41    -41       O
HETATM 2250  O   HOH S 118     -13.869  24.687  -1.214  1.00 36.33           O  0.034
ANISOU 2250  O   HOH S 118     4033   7420   2352   -285   -921    690       O
HETATM 2251  O   HOH S 119     -19.289  20.575   8.236  1.00 42.71           O  0.037
ANISOU 2251  O   HOH S 119     2887   6006   7335    398    670   2699       O
HETATM 2252  O   HOH S 120       9.720  19.033   8.382  1.00 22.60           O  0.027
ANISOU 2252  O   HOH S 120     2615   2792   3181    432    501    204       O
HETATM 2253  O   HOH S 122       7.301  24.017   3.105  1.00 37.29           O  0.035
ANISOU 2253  O   HOH S 122     5697   6066   2406   -945  -1080    845       O
HETATM 2254  O   HOH S 129       8.360  21.320   1.966  1.00 38.30           O  0.035
ANISOU 2254  O   HOH S 129     3804   4351   6396   1355   -904   -760       O
HETATM 2255  O   HOH S 134      14.292  12.383  39.009  1.00 44.79           O  0.038
ANISOU 2255  O   HOH S 134     5721   8234   3064   2081    909   1097       O
HETATM 2256  O   HOH S 139       0.027  13.403   4.567  1.00 38.95           O  0.036
ANISOU 2256  O   HOH S 139     5461   5608   3730   -951  -1280  -1099       O
HETATM 2257  O   HOH S 157     -12.912  15.490  21.204  1.00 39.98           O  0.036
ANISOU 2257  O   HOH S 157     3911   4644   6634   -912   -762   1195       O
HETATM 2258  O   HOH S 162      16.312  13.162  34.128  1.00 38.30           O  0.035
ANISOU 2258  O   HOH S 162     7360   3170   4023   1203  -2783   -327       O
HETATM 2259  O   HOH S 163      -7.887  20.303  29.644  1.00 44.36           O  0.038
ANISOU 2259  O   HOH S 163     4088   3913   8855    -83   2529   1532       O
HETATM 2260  O   HOH S 172       4.269   8.409  30.646  1.00 30.64           O  0.032
ANISOU 2260  O   HOH S 172     5169   3017   3455    232   -143    806       O
HETATM 2261  O   HOH S 173     -10.125  12.141  19.908  1.00 40.95           O  0.036
ANISOU 2261  O   HOH S 173     3948   6068   5542  -1606   -327   2967       O
HETATM 2262  O   HOH S 175      16.619  26.930  22.864  1.00 33.80           O  0.033
HETATM 2263  O   HOH S 176      -7.603  14.214   7.686  1.00 32.60           O  0.033
HETATM 2264 RE    RE D   2     -16.019  16.295   8.273  0.21 32.51          Re  0.032
HETATM 2265 RE    RE D   4     -10.815  10.815   9.272  0.19119.27          Re  0.062
HETATM 2266 RE    RE D   5     -18.055  23.263   8.198  0.18 59.10          Re  0.044
HETATM 2267 BR    BR B   1      -8.045  11.671   7.602  0.20 24.02          Br  0.028
END
```
