# Supplementary material for: Time-series analysis of rhenium(I) organometallic covalent binding to a model protein for drug development
Source: IUCrJ. 2024 Apr 19;11(Pt 3):359–73. doi: 10.1107/S2052252524002598 (PMC11067751; doi:10.1107/S2052252524002598)
Supplement: Supplementary file 4 [file m-11-00359-sup4.zip › Week 11 - V1_22Ffj5/D_9100077159_val-report-full_P1.pdf]

Preliminary Full wwPDB X-ray Structure Validation Report (i)

Feb 28, 2023 – 03:17 AM EST

This wwPDB validation report is NOT for manuscript review

This is a Preliminary Full wwPDB X-ray Structure Validation Report.

This report is produced by the standalone wwPDB validation server.
The structure in question has not been deposited to the wwPDB.
This report should not be submitted to journals.

We welcome your comments at *validation@mail.wwpdb.org*
A user guide is available at
https://www.wwpdb.org/validation/2017/XrayValidationReportHelp
with specific help available everywhere you see the (i) symbol.

The types of validation reports are described at
http://www.wwpdb.org/validation/2017/FAQs#types.

Not For Manuscript Review

The following versions of software and data (see references (i)) were used in the production of this report:

MolProbity	:	4.02b-467
Mogul	:	1.8.5 (274361), CSD as541be (2020)
Xtriage (Phenix)	:	1.13
EDS	:	2.32.1
buster-report	:	1.1.7 (2018)
Percentile statistics	:	20191225.v01 (using entries in the PDB archive December 25th 2019)
Refmac	:	5.8.0158
CCP4	:	7.0.044 (Gargrove)
Ideal geometry (proteins)	:	Engh & Huber (2001)
Ideal geometry (DNA, RNA)	:	Parkinson et al. (1996)
Validation Pipeline (wwPDB-VP)	:	2.32.1

1 Overall quality at a glance (i)

The following experimental techniques were used to determine the structure:
X-RAY DIFFRACTION

The reported resolution of this entry is 1.23 Å.

Percentile scores (ranging between 0-100) for global validation metrics of the entry are shown in the following graphic. The table shows the number of entries on which the scores are based.

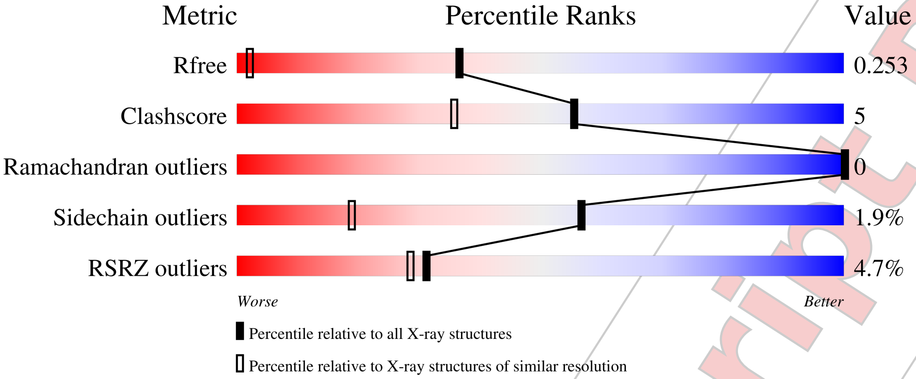

Metric	Whole archive (#Entries)	Similar resolution (#Entries, resolution range(Å))
R_{free}	130704	2024 (1.28-1.20)
Clashscore	141614	1007 (1.26-1.22)
Ramachandran outliers	138981	2053 (1.28-1.20)
Sidechain outliers	138945	2051 (1.28-1.20)
RSRZ outliers	127900	1987 (1.28-1.20)

The table below summarises the geometric issues observed across the polymeric chains and their fit to the electron density. The red, orange, yellow and green segments of the lower bar indicate the fraction of residues that contain outliers for >=3, 2, 1 and 0 types of geometric quality criteria respectively. A grey segment represents the fraction of residues that are not modelled. The numeric value for each fraction is indicated below the corresponding segment, with a dot representing fractions <=5% The upper red bar (where present) indicates the fraction of residues that have poor fit to the electron density. The numeric value is given above the bar.

Mol	Chain	Length	Quality of chain
1	A	129	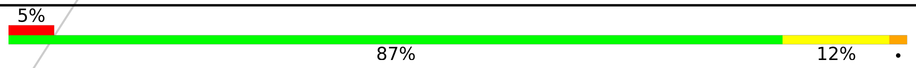

Not For Manuscript Review

2 Entry composition (i)

There are 8 unique types of molecules in this entry. The entry contains 2275 atoms, of which 1048 are hydrogens and 0 are deuteriums.

In the tables below, the ZeroOcc column contains the number of atoms modelled with zero occupancy, the AltConf column contains the number of residues with at least one atom in alternate conformation and the Trace column contains the number of residues modelled with at most 2 atoms.

- Molecule 1 is a protein called Chains: A.

Mol	Chain	Residues	Atoms	ZeroOcc	AltConf	Trace
1	A	129	Total C H N O S<br>2093 647 1028 212 196 10	0	13	0

- Molecule 2 is CHLORIDE ION (three-letter code: CL) (formula: Cl).

Mol	Chain	Residues	Atoms	ZeroOcc	AltConf
2	A	1	Total Cl<br>1 1	0	0
2	D	1	Total Cl<br>1 1	0	0
2	D	1	Total Cl<br>1 1	0	0
2	D	1	Total Cl<br>1 1	0	0

- Molecule 3 is SODIUM ION (three-letter code: NA) (formula: Na).

Mol	Chain	Residues	Atoms	ZeroOcc	AltConf
3	A	1	Total Na<br>1 1	0	0

- Molecule 4 is a ligand with the chemical component id RII but there is no existing wwPDB Chemical Component Dictionary definition for RII. Consequently no firm identification of ligand chemistry can be made. Once the structure is annotated then an identification and diagram will be given here.

Mol	Chain	Residues	Atoms	ZeroOcc	AltConf
4	A	1	Total C H N O Re<br>25 9 8 4 3 1	0	0
4	A	1	Total C H N O Re<br>25 9 8 4 3 1	0	0

Not For Manuscript Review

- Molecule 5 is a ligand with the chemical component id RI3 but its atom names do not match the existing wwPDB Chemical Component Dictionary definition for RI3. Consequently no firm identification of ligand chemistry can be made. Once the structure is annotated then an identification and diagram will be given here.

Mol	Chain	Residues	Atoms	ZeroOcc	AltConf
5	A	1	Total C H N O Re<br>17 6 4 2 4 1	0	0

- Molecule 6 is RHENIUM (three-letter code: RE) (formula: Re).

Mol	Chain	Residues	Atoms	ZeroOcc	AltConf
6	B	1	Total Re<br>1 1	0	0
6	B	1	Total Re<br>1 1	0	0
6	B	1	Total Re<br>1 1	0	0
6	B	1	Total Re<br>1 1	0	0
6	B	1	Total Re<br>1 1	0	0

- Molecule 7 is BROMIDE ION (three-letter code: BR) (formula: Br).

Mol	Chain	Residues	Atoms	ZeroOcc	AltConf
7	C	1	Total Br<br>1 1	0	0

- Molecule 8 is water.

Mol	Chain	Residues	Atoms	ZeroOcc	AltConf
8	S	104	Total O<br>104 104	0	0

Not For Manuscript Review

3 Residue-property plots (i)

These plots are drawn for all protein, RNA, DNA and oligosaccharide chains in the entry. The first graphic for a chain summarises the proportions of the various outlier classes displayed in the second graphic. The second graphic shows the sequence view annotated by issues in geometry and electron density. Residues are color-coded according to the number of geometric quality criteria for which they contain at least one outlier: green = 0, yellow = 1, orange = 2 and red = 3 or more. A red dot above a residue indicates a poor fit to the electron density (RSRZ > 2). Stretches of 2 or more consecutive residues without any outlier are shown as a green connector. Residues present in the sample, but not in the model, are shown in grey.

- Molecule 1: Chains: A

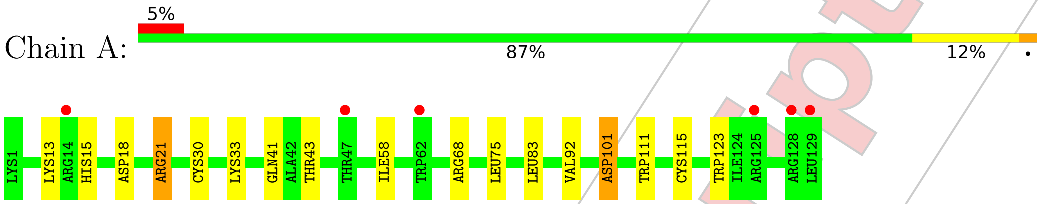

Not For Manuscript Review

4 Data and refinement statistics (i)

Property	Value	Source
Space group	P 43 21 2	Depositor
Cell constants<br>a, b, c, α, β, γ	80.75Å 80.75Å 37.02Å<br>90.00° 90.00° 90.00°	Depositor
Resolution (Å)	22.61 – 1.23<br>22.61 – 1.23	Depositor<br>EDS
% Data completeness<br>(in resolution range)	85.6 (22.61-1.23)<br>72.9 (22.61-1.23)	Depositor<br>EDS
R_{merge}	(Not available)	Depositor
R_{sym}	(Not available)	Depositor
$< I/\sigma(I) >$ [1]	1.29 (at 1.23Å)	Xtriage
Refinement program	phenix.refine 1.20.1_4487, PHENIX 1.20.1_4487	Depositor
R, R_{free}	0.220 , 0.249<br>0.230 , 0.253	Depositor<br>DCC
R_{free} test set	1921 reflections (5.41%)	wwPDB-VP
Wilson B-factor (Å^2)	4.9	Xtriage
Anisotropy	0.820	Xtriage
Bulk solvent k_{sol}(e/Å^3), B_{sol}(Å^2)	0.38 , 41.0	EDS
L-test for twinning[2]	$< \|L\| > = 0.49$, $< L^2 > = 0.32$	Xtriage
Estimated twinning fraction	No twinning to report.	Xtriage
F_o,F_c correlation	0.93	EDS
Total number of atoms	2275	wwPDB-VP
Average B, all atoms (Å^2)	20.0	wwPDB-VP

Xtriage's analysis on translational NCS is as follows: *The largest off-origin peak in the Patterson function is 9.86% of the height of the origin peak. No significant pseudotranslation is detected.*

[1] Intensities estimated from amplitudes.
[2] Theoretical values of $< |L| >$, $< L^2 >$ for acentric reflections are 0.5, 0.333 respectively for untwinned datasets, and 0.375, 0.2 for perfectly twinned datasets.

Not For Manuscript Review

5 Model quality (i)

5.1 Standard geometry (i)

Bond lengths and bond angles in the following residue types are not validated in this section: RII, CL, RI3, BR, RE, NA

The Z score for a bond length (or angle) is the number of standard deviations the observed value is removed from the expected value. A bond length (or angle) with $|Z| > 5$ is considered an outlier worth inspection. RMSZ is the root-mean-square of all Z scores of the bond lengths (or angles).

Mol	Chain	Bond lengths		Bond angles	
		RMSZ	$\#\|Z\| > 5$	RMSZ	$\#\|Z\| > 5$
1	A	0.58	0/1128	0.83	0/1523

There are no bond length outliers.

There are no bond angle outliers.

There are no chirality outliers.

There are no planarity outliers.

5.2 Too-close contacts (i)

In the following table, the Non-H and H(model) columns list the number of non-hydrogen atoms and hydrogen atoms in the chain respectively. The H(added) column lists the number of hydrogen atoms added and optimized by MolProbity. The Clashes column lists the number of clashes within the asymmetric unit, whereas Symm-Clashes lists symmetry-related clashes.

Mol	Chain	Non-H	H(model)	H(added)	Clashes	Symm-Clashes
1	A	1065	1028	987	11	1
2	A	1	0	0	0	0
2	D	3	0	0	1	0
3	A	1	0	0	0	0
4	A	34	16	0	2	0
5	A	13	4	0	0	0
6	B	5	0	0	0	0
7	C	1	0	0	0	0
8	S	104	0	0	1	0
All	All	1227	1048	987	11	1

The all-atom clashscore is defined as the number of clashes found per 1000 atoms (including hydrogen atoms). The all-atom clashscore for this structure is 5.

Not For Manuscript Review

All (11) close contacts within the same asymmetric unit are listed below, sorted by their clash magnitude.

Atom-1	Atom-2	Interatomic distance (Å)	Clash overlap (Å)
1:A:21[B]:ARG:NH1	8:S:93:HOH:O	2.21	0.72
1:A:13:LYS:NZ	1:A:18:ASP:OD2	2.27	0.67
1:A:21[A]:ARG:HE	1:A:21[A]:ARG:HA	1.72	0.54
1:A:75:LEU:HD13	4:A:1139:RII:C3	2.43	0.48
1:A:101:ASP:OD2	4:A:1139:RII:C4	2.64	0.46
1:A:41:GLN:O	2:D:1:CL:CL	2.72	0.45
1:A:58:ILE:HB	1:A:83:LEU:HD13	1.99	0.45
1:A:15:HIS:HB3	1:A:92:VAL:HG11	2.00	0.44
1:A:30:CYS:HB2	1:A:123:TRP:CD1	2.55	0.41
1:A:33:LYS:HG2	1:A:123:TRP:CH2	2.55	0.41
1:A:111:TRP:CD1	1:A:115:CYS:HB2	2.57	0.40

All (1) symmetry-related close contacts are listed below. The label for Atom-2 includes the symmetry operator and encoded unit-cell translations to be applied.

Atom-1	Atom-2	Interatomic distance (Å)	Clash overlap (Å)
1:A:43:THR:O	1:A:68[A]:ARG:HH12[8_554]	1.60	0.00

5.3 Torsion angles (i)

5.3.1 Protein backbone (i)

In the following table, the Percentiles column shows the percent Ramachandran outliers of the chain as a percentile score with respect to all X-ray entries followed by that with respect to entries of similar resolution.

The Analysed column shows the number of residues for which the backbone conformation was analysed, and the total number of residues.

Mol	Chain	Analysed	Favoured	Allowed	Outliers	Percentiles	
1	A	140/129 (108%)	138 (99%)	2 (1%)	0	100	100

There are no Ramachandran outliers to report.

5.3.2 Protein sidechains (i)

In the following table, the Percentiles column shows the percent sidechain outliers of the chain as a percentile score with respect to all X-ray entries followed by that with respect to entries of similar

Not For Manuscript Review

resolution.

The Analysed column shows the number of residues for which the sidechain conformation was analysed, and the total number of residues.

Mol	Chain	Analysed	Rotameric	Outliers	Percentiles	
1	A	116/105 (110%)	113 (97%)	3 (3%)	46	9

All (3) residues with a non-rotameric sidechain are listed below:

Mol	Chain	Res	Type
1	A	21[A]	ARG
1	A	21[B]	ARG
1	A	101	ASP

Sometimes sidechains can be flipped to improve hydrogen bonding and reduce clashes. There are no such sidechains identified.

5.3.3 RNA (i)

There are no RNA molecules in this entry.

5.4 Non-standard residues in protein, DNA, RNA chains (i)

There are no non-standard protein/DNA/RNA residues in this entry.

5.5 Carbohydrates (i)

There are no monosaccharides in this entry.

5.6 Ligand geometry (i)

Of 14 ligands modelled in this entry, 11 are monoatomic, 2 could not be matched to an existing wwPDB Chemical Component Dictionary definition at this stage and 1 could not be matched to an existing wwPDB Chemical Component Dictionary definition at this stage - leaving 0 for Mogul analysis.

There are no bond length outliers.

There are no bond angle outliers.

There are no chirality outliers.

There are no torsion outliers.

Not For Manuscript Review

There are no ring outliers.

No monomer is involved in short contacts.

5.7 Other polymers (i)

There are no such residues in this entry.

5.8 Polymer linkage issues (i)

There are no chain breaks in this entry.

Not For Manuscript Review

6 Fit of model and data (i)

6.1 Protein, DNA and RNA chains (i)

In the following table, the column labelled '#RSRZ> 2' contains the number (and percentage) of RSRZ outliers, followed by percent RSRZ outliers for the chain as percentile scores relative to all X-ray entries and entries of similar resolution. The OWAB column contains the minimum, median, 95th percentile and maximum values of the occupancy-weighted average B-factor per residue. The column labelled 'Q< 0.9' lists the number of (and percentage) of residues with an average occupancy less than 0.9.

Mol	Chain	Analysed	<RSRZ>	#RSRZ>2			OWAB(Å^2)	Q<0.9
1	A	129/129 (100%)	0.48	6 (4%)	31	28	10, 17, 26, 39	0

All (6) RSRZ outliers are listed below:

Mol	Chain	Res	Type	RSRZ
1	A	129	LEU	5.4
1	A	128	ARG	3.4
1	A	47	THR	3.3
1	A	14	ARG	2.6
1	A	125	ARG	2.3
1	A	62	TRP	2.2

6.2 Non-standard residues in protein, DNA, RNA chains (i)

There are no non-standard protein/DNA/RNA residues in this entry.

6.3 Carbohydrates (i)

There are no monosaccharides in this entry.

6.4 Ligands (i)

In the following table, the Atoms column lists the number of modelled atoms in the group and the number defined in the chemical component dictionary. The B-factors column lists the minimum, median, 95th percentile and maximum values of B factors of atoms in the group. The column labelled 'Q< 0.9' lists the number of atoms with occupancy less than 0.9.

Mol	Type	Chain	Res	Atoms	RSCC	RSR	B-factors(Å^2)	Q<0.9
2	CL	D	3	1/?	0.93	0.05	38,38,38,38	0

Continued on next page...

Not For Manuscript Review

Continued from previous page...

Mol	Type	Chain	Res	Atoms	RSCC	RSR	B-factors(Å^2)	Q<0.9
2	CL	D	2	1/?	0.94	0.07	37,37,37,37	0
6	RE	B	5	1/?	0.96	0.08	17,17,17,17	1
3	NA	A	1138	1/?	0.98	0.16	21,21,21,21	0
4	RII	A	1139	17/?	0.98	0.14	15,21,24,24	25
6	RE	B	2	1/?	0.98	0.10	17,17,17,17	1
2	CL	D	1	1/?	0.98	0.05	26,26,26,26	0
7	BR	C	1	1/?	0.98	0.08	27,27,27,27	1
4	RII	A	1140	17/?	0.99	0.10	16,18,21,22	25
6	RE	B	3	1/?	0.99	0.03	25,25,25,25	1
5	RI3	A	1143	13/?	0.99	0.08	26,30,37,37	17
6	RE	B	1	1/?	0.99	0.08	20,20,20,20	1
2	CL	A	1131	1/?	1.00	0.05	15,15,15,15	1
6	RE	B	4	1/?	1.00	0.05	19,19,19,19	1

The following is a graphical depiction of the model fit to experimental electron density of all instances of the Ligand of Interest. In addition, ligands with molecular weight > 250 and outliers as shown on the geometry validation Tables will also be included. Each fit is shown from different orientation to approximate a three-dimensional view.

Not For Manuscript Review

Electron density around RII A 1139:

2mF$_o$-DF$_c$ (at 0.7 rmsd) in gray
mF$_o$-DF$_c$ (at 3 rmsd) in purple (negative)
and green (positive)

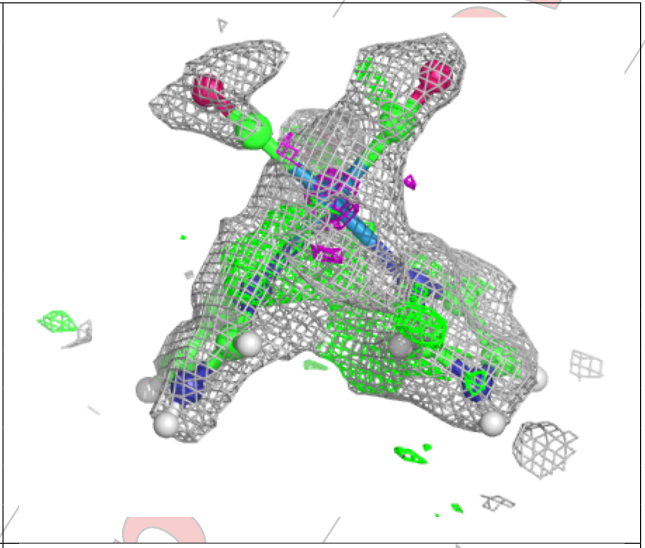

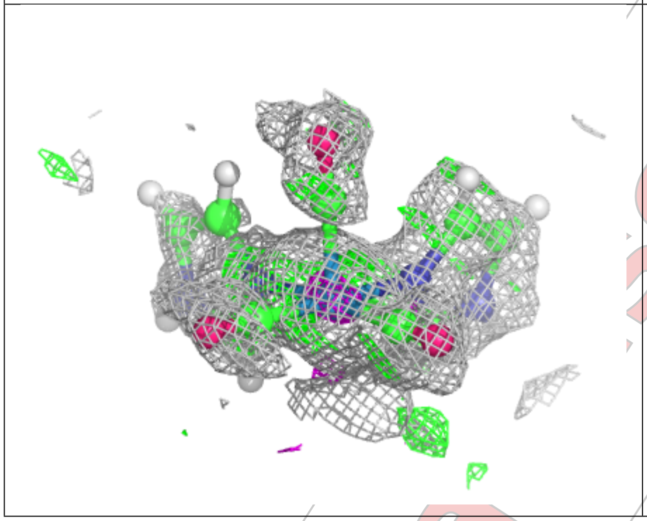

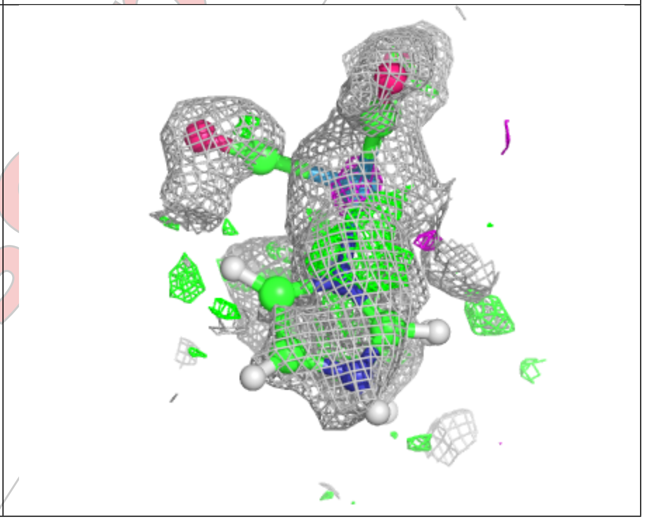

Not For Manuscript Review

Electron density around RII A 1140:

$2mF_o$-DF_c (at 0.7 rmsd) in gray
mF_o-DF_c (at 3 rmsd) in purple (negative)
and green (positive)

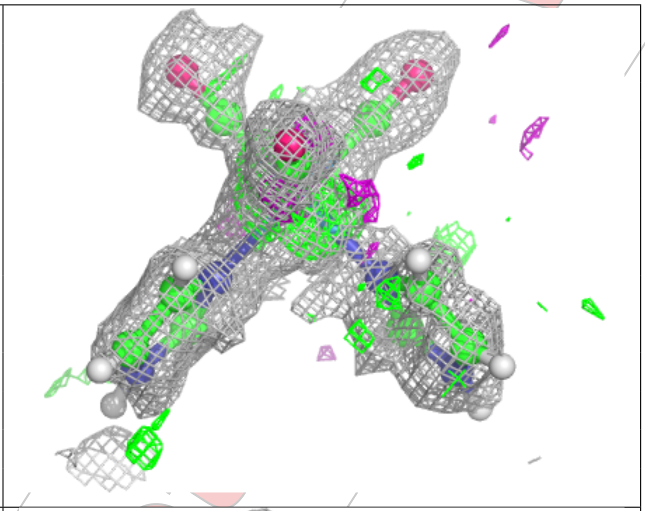

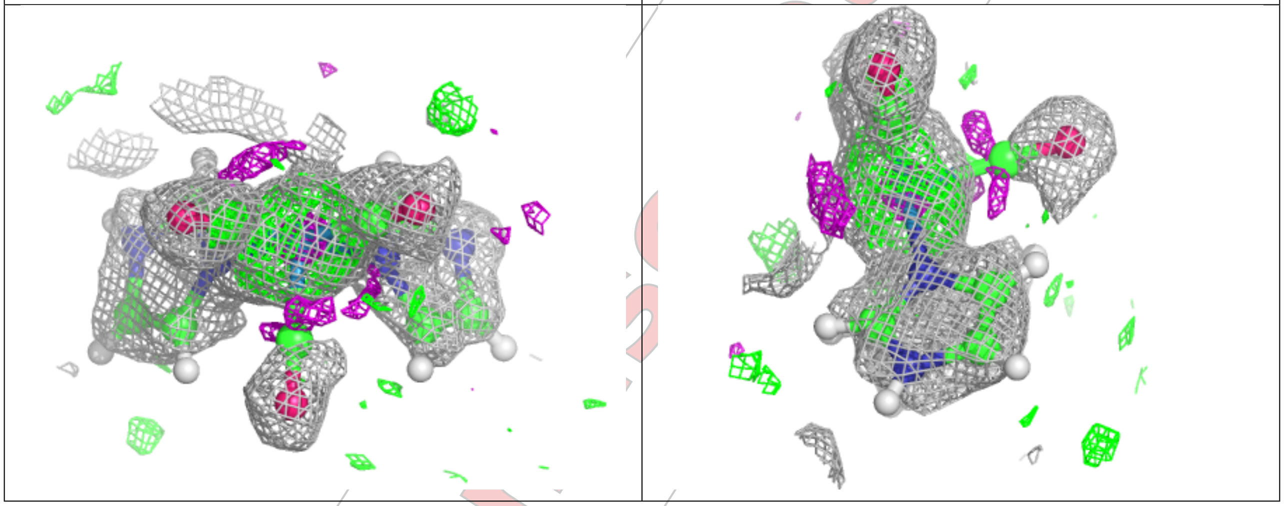

6.5 Other polymers (i)

There are no such residues in this entry.

Not For Manuscript Review
