# Supplementary material for: Time-series analysis of rhenium(I) organometallic covalent binding to a model protein for drug development
Source: IUCrJ. 2024 Apr 19;11(Pt 3):359–73. doi: 10.1107/S2052252524002598 (PMC11067751; doi:10.1107/S2052252524002598)
Supplement: Supplementary file 4 [file m-11-00359-sup4.zip › Week 11 - V1_22Ffj5/Lab_Week11_refine_29.pdf]

```
REMARK   3
REMARK   3 REFINEMENT.
REMARK   3   PROGRAM     : PHENIX (1.20.1_4487: ???)
REMARK   3   AUTHORS     : Adams,Afonine,Bunkoczi,Burnley,Chen,Dar,Davis,
REMARK   3               : Draizen,Echols,Gildea,Gros,Grosse-Kunstleve,Headd,
REMARK   3               : Hintze,Hung,Ioerger,Liebschner,McCoy,McKee,Moriarty,
REMARK   3               : Oeffner,Poon,Read,Richardson,Richardson,Sacchettini,
REMARK   3               : Sauter,Sobolev,Storoni,Terwilliger,Williams,Zwart
REMARK   3
REMARK   3  X-RAY DATA.
REMARK   3
REMARK   3  REFINEMENT TARGET : ML
REMARK   3
REMARK   3  DATA USED IN REFINEMENT.
REMARK   3   RESOLUTION RANGE HIGH (ANGSTROMS) : 1.23
REMARK   3   RESOLUTION RANGE LOW  (ANGSTROMS) : 22.61
REMARK   3   MIN(FOBS/SIGMA_FOBS)              : 1.33
REMARK   3   COMPLETENESS FOR RANGE        (%) : 85.57
REMARK   3   NUMBER OF REFLECTIONS             : 58726
REMARK   3   NUMBER OF REFLECTIONS (NON-ANOMALOUS) : 32871
REMARK   3
REMARK   3  FIT TO DATA USED IN REFINEMENT.
REMARK   3   R VALUE     (WORKING + TEST SET) : 0.2217
REMARK   3   R VALUE            (WORKING SET) : 0.2199
REMARK   3   FREE R VALUE                     : 0.2491
REMARK   3   FREE R VALUE TEST SET SIZE   (%) : 6.08
REMARK   3   FREE R VALUE TEST SET COUNT      : 3573
REMARK   3
REMARK   3  FIT TO DATA USED IN REFINEMENT (IN BINS).
REMARK   3   BIN  RESOLUTION RANGE  COMPL.    NWORK NFREE   RWORK  RFREE  CCWORK CCFREE
REMARK   3     1   22.61 -    3.63    1.00     2481   161  0.1640 0.1863   0.923  0.909
REMARK   3     2    3.63 -    2.88    1.00     2464   171  0.1565 0.1930   0.934  0.900
REMARK   3     3    2.88 -    2.52    1.00     2486   163  0.1599 0.2247   0.932  0.896
REMARK   3     4    2.52 -    2.29    1.00     2466   156  0.1553 0.1840   0.937  0.909
REMARK   3     5    2.29 -    2.12    1.00     2477   159  0.1598 0.1946   0.938  0.906
REMARK   3     6    2.12 -    2.00    0.99     2463   161  0.1649 0.1810   0.934  0.916
REMARK   3     7    2.00 -    1.90    0.99     2477   156  0.1628 0.1784   0.936  0.898
REMARK   3     8    1.90 -    1.82    1.00     2468   162  0.1662 0.2380   0.934  0.857
REMARK   3     9    1.82 -    1.75    1.00     2473   158  0.1579 0.2162   0.936  0.889
REMARK   3    10    1.75 -    1.69    1.00     2469   159  0.1692 0.2093   0.925  0.862
REMARK   3    11    1.69 -    1.63    1.00     2491   161  0.1784 0.2116   0.919  0.888
REMARK   3    12    1.63 -    1.59    1.00     2466   158  0.1914 0.2129   0.903  0.861
REMARK   3    13    1.59 -    1.54    1.00     2477   161  0.2092 0.2693   0.883  0.776
REMARK   3    14    1.54 -    1.51    0.99     2466   158  0.3153 0.3769   0.782  0.640
REMARK   3    15    1.51 -    1.47    0.99     2467   160  0.3084 0.3303   0.750  0.670
REMARK   3    16    1.47 -    1.44    0.97     2364   155  0.4021 0.4577   0.647  0.542
REMARK   3    17    1.44 -    1.41    0.89     2215   143  0.4815 0.4852   0.560  0.566
REMARK   3    18    1.41 -    1.39    0.80     1947   125  0.4568 0.5697   0.494  0.522
REMARK   3    19    1.39 -    1.36    0.27      663    45  0.7264 0.8077   0.347  -0.335
REMARK   3    20    1.36 -    1.34    0.36      746    48  0.4250 0.4075   0.406  0.211
REMARK   3    21    1.34 -    1.32    0.59     1448    97  0.3861 0.3189   0.227  0.469
REMARK   3    22    1.32 -    1.30    0.51     1261    87  0.3830 0.4105   0.267  0.259
REMARK   3    23    1.30 -    1.28    0.88     2150   134  0.4215 0.4140   0.223  0.351
REMARK   3    24    1.28 -    1.26    0.84     2107   140  0.4959 0.4838   0.193  0.176
REMARK   3    25    1.26 -    1.24    0.91     2252   139  0.6157 0.5860   0.166  0.104
REMARK   3    26    1.24 -    1.23    0.37      909    56  0.7714 0.7615   0.143  0.011
REMARK   3
REMARK   3  BULK SOLVENT MODELLING.
REMARK   3   METHOD USED        : FLAT BULK SOLVENT MODEL
REMARK   3   SOLVENT RADIUS     : 1.10
REMARK   3   SHRINKAGE RADIUS   : 0.90
REMARK   3   GRID STEP FACTOR   : 4.00
REMARK   3
REMARK   3  ERROR ESTIMATES.
REMARK   3   COORDINATE ERROR (MAXIMUM-LIKELIHOOD BASED)     : 0.24
REMARK   3   PHASE ERROR (DEGREES, MAXIMUM-LIKELIHOOD BASED) : 32.36
REMARK   3
REMARK   3  STRUCTURE FACTORS CALCULATION ALGORITHM : FFT
REMARK   3  B VALUES.
REMARK   3   FROM WILSON PLOT           (A**2) : 9.48
REMARK   3
REMARK   3  GEOMETRY RESTRAINTS LIBRARY: GEOSTD + MONOMER LIBRARY + CDL V1.2
REMARK   3  DEVIATIONS FROM IDEAL VALUES - RMSD. RMSZ FOR BONDS AND ANGLES.
REMARK   3    BOND      :  0.011   0.077   1140  Z= 0.718
REMARK   3    ANGLE     :  1.345   9.285   1557  Z= 0.756
REMARK   3    CHIRALITY :  0.102   0.537    151
REMARK   3    PLANARITY :  0.019   0.119    205
REMARK   3    DIHEDRAL  : 14.151  82.085    410
```

```
REMARK   3    MIN NONBONDED DISTANCE : 1.993
REMARK   3
REMARK   3  MOLPROBITY STATISTICS.
REMARK   3    ALL-ATOM CLASHSCORE : 5.11
REMARK   3    RAMACHANDRAN PLOT:
REMARK   3      OUTLIERS :  0.00 %
REMARK   3      ALLOWED  :  1.57 %
REMARK   3      FAVORED  : 98.43 %
REMARK   3    ROTAMER OUTLIERS :  0.89 %
REMARK   3    CBETA DEVIATIONS :  0.00 %
REMARK   3    PEPTIDE PLANE:
REMARK   3      CIS-PROLINE     : 0.00 %
REMARK   3      CIS-GENERAL     : 0.00 %
REMARK   3      TWISTED PROLINE : 0.00 %
REMARK   3      TWISTED GENERAL : 0.00 %
REMARK   3
REMARK   3  RAMA-Z (RAMACHANDRAN PLOT Z-SCORE):
REMARK   3  INTERPRETATION: BAD |RAMA-Z| > 3; SUSPICIOUS 2 < |RAMA-Z| < 3; GOOD |RAMA-Z| < 2.
REMARK   3  SCORES FOR WHOLE/HELIX/SHEET/LOOP ARE SCALED INDEPENDENTLY;
REMARK   3  THEREFORE, THE VALUES ARE NOT RELATED IN A SIMPLE MANNER.
REMARK   3    WHOLE:  0.19 (0.67), RESIDUES: 145
REMARK   3    HELIX: -0.89 (0.64), RESIDUES: 45
REMARK   3    SHEET: -0.82 (0.86), RESIDUES: 14
REMARK   3    LOOP :  1.13 (0.72), RESIDUES: 86
REMARK   3
REMARK   3                  min    max   mean <Bi,j>   iso aniso
REMARK   3     Overall:    9.01  46.77  18.89   1.37     5  1222
REMARK   3     Protein:    9.01  46.77  17.91   1.36     0  1065
REMARK   3     Water:     12.76  42.64  26.96    N/A     3   101
REMARK   3     Other:     15.66  38.40  22.41    N/A     2    56
REMARK   3     Chain  A:   9.01  46.77  18.08    N/A     0  1114
REMARK   3     Chain  C:  27.79  27.79  27.79    N/A     1     0
REMARK   3     Chain  S:  12.76  42.64  26.96    N/A     3   101
REMARK   3     Chain  B:  17.56  25.41  20.15    N/A     1     4
REMARK   3     Chain  D:  26.11  38.40  33.90    N/A     0     3
REMARK   3     Histogram:
REMARK   3         Values      Number of atoms
REMARK   3       9.01 - 12.79       170
REMARK   3      12.79 - 16.56       366
REMARK   3      16.56 - 20.34       311
REMARK   3      20.34 - 24.12       155
REMARK   3      24.12 - 27.89       110
REMARK   3      27.89 - 31.67        48
REMARK   3      31.67 - 35.44        32
REMARK   3      35.44 - 39.22        21
REMARK   3      39.22 - 43.00         9
REMARK   3      43.00 - 46.77         5
REMARK   3
REMARK   3
LINK         NE2 HIS A  15                 RE1  RI3 A1143
LINK         OD2 ASP A 101                 RE1  RII A1139
LINK         OD2 ASP A 119                 RE1  RII A1140
SSBOND   1 CYS A    6    CYS A  127
SSBOND   2 CYS A   30    CYS A  115
SSBOND   3 CYS A   64    CYS A   80
SSBOND   4 CYS A   76    CYS A   94
CRYST1   80.753   80.753   37.018  90.00  90.00  90.00 P 43 21 2
SCALE1      0.012383  0.000000  0.000000        0.00000
SCALE2      0.000000  0.012383  0.000000        0.00000
SCALE3      0.000000  0.000000  0.027014        0.00000
ATOM      1  N   LYS A   1      11.589  -4.738 -17.970  1.00 20.97           N  0.044
ANISOU    1  N   LYS A   1     2019   1943   4005   -589   -925    953       N
ATOM      2  CA  LYS A   1      11.959  -3.782 -19.019  1.00 21.37           C  0.045
ANISOU    2  CA  LYS A   1     2135   1901   4083   -472   -926    848       C
ATOM      3  C   LYS A   1      13.463  -3.781 -19.207  1.00 20.31           C  0.044
ANISOU    3  C   LYS A   1     2118   2027   3573   -563   -815    826       C
ATOM      4  O   LYS A   1      14.207  -3.752 -18.225  1.00 20.22           O  0.044
ANISOU    4  O   LYS A   1     1858   1989   3834   -584   -750   1054       O
ATOM      5  CB  LYS A   1      11.486  -2.365 -18.693  1.00 21.91           C  0.045
ANISOU    5  CB  LYS A   1     2263   1744   4319   -327   -938    960       C
ATOM      6  CG  LYS A   1      11.985  -1.312 -19.658  1.00 21.86           C  0.045
ANISOU    6  CG  LYS A   1     2485   1610   4210   -259   -881   1056       C
ATOM      7  CD  LYS A   1      11.383   0.034 -19.314  1.00 22.30           C  0.046
ANISOU    7  CD  LYS A   1     2624   1639   4210   -229   -820   1096       C
ATOM      8  CE  LYS A   1      11.741   1.078 -20.364  1.00 24.45           C  0.048
ANISOU    8  CE  LYS A   1     2850   1755   4685   -222   -666   1197       C
ATOM      9  NZ  LYS A   1      11.223   2.450 -20.052  1.00 25.97           N  0.049
ANISOU    9  NZ  LYS A   1     2958   1963   4946    -88   -830   1330       N
```

```
ATOM     10  H1  LYS A   1      10.703  -4.756 -17.884  1.00 25.31           H  0.049
ATOM     11  H2  LYS A   1      11.882  -5.549 -18.191  1.00 25.31           H  0.049
ATOM     12  H3  LYS A   1      11.957  -4.489 -17.199  1.00 25.31           H  0.049
ATOM     13  HA  LYS A   1      11.549  -4.053 -19.856  1.00 25.79           H  0.049
ATOM     14  HB2 LYS A   1      10.516  -2.348 -18.712  1.00 26.45           H  0.050
ATOM     15  HB3 LYS A   1      11.801  -2.127 -17.807  1.00 26.45           H  0.050
ATOM     16  HG2 LYS A   1      12.950  -1.235 -19.594  1.00 26.38           H  0.050
ATOM     17  HG3 LYS A   1      11.721  -1.547 -20.561  1.00 26.38           H  0.050
ATOM     18  HD2 LYS A   1      10.417  -0.045 -19.280  1.00 26.91           H  0.050
ATOM     19  HD3 LYS A   1      11.727   0.331 -18.457  1.00 26.91           H  0.050
ATOM     20  HE2 LYS A   1      12.707   1.134 -20.433  1.00 29.49           H  0.053
ATOM     21  HE3 LYS A   1      11.366   0.806 -21.216  1.00 29.49           H  0.053
ATOM     22  HZ1 LYS A   1      11.460   3.018 -20.695  1.00 31.31           H  0.054
ATOM     23  HZ2 LYS A   1      10.335   2.434 -19.993  1.00 31.31           H  0.054
ATOM     24  HZ3 LYS A   1      11.559   2.733 -19.278  1.00 31.31           H  0.054
ATOM     25  N   VAL A   2      13.922  -3.838 -20.455  1.00 18.35           N  0.041
ANISOU   25  N   VAL A   2     2166   2146   2662   -656  -1256    625       N
ATOM     26  CA  VAL A   2      15.334  -3.691 -20.772  1.00 19.81           C  0.043
ANISOU   26  CA  VAL A   2     2326   2205   2995   -779   -821    727       C
ATOM     27  C   VAL A   2      15.513  -2.280 -21.299  1.00 19.22           C  0.042
ANISOU   27  C   VAL A   2     2317   2202   2784   -908   -800    703       C
ATOM     28  O   VAL A   2      15.058  -1.962 -22.404  1.00 20.61           O  0.044
ANISOU   28  O   VAL A   2     2443   2365   3023  -1003   -878    646       O
ATOM     29  CB  VAL A   2      15.807  -4.739 -21.788  1.00 20.02           C  0.043
ANISOU   29  CB  VAL A   2     2421   2243   2944   -744   -966    834       C
ATOM     30  CG1 VAL A   2      17.279  -4.464 -22.082  1.00 21.00           C  0.044
ANISOU   30  CG1 VAL A   2     2621   2267   3092   -606   -691   1118       C
ATOM     31  CG2 VAL A   2      15.585  -6.182 -21.278  1.00 20.65           C  0.044
ANISOU   31  CG2 VAL A   2     2369   2253   3224   -703  -1028    915       C
ATOM     32  H   VAL A   2      13.424  -3.963 -21.145  1.00 22.17           H  0.046
ATOM     33  HA  VAL A   2      15.862  -3.787 -19.965  1.00 23.92           H  0.047
ATOM     34  HB  VAL A   2      15.309  -4.630 -22.613  1.00 24.18           H  0.048
ATOM     35 HG11 VAL A   2      17.663  -5.244 -22.512  1.00 25.35           H  0.049
ATOM     36 HG12 VAL A   2      17.349  -3.696 -22.670  1.00 25.35           H  0.049
ATOM     37 HG13 VAL A   2      17.740  -4.288 -21.247  1.00 25.35           H  0.049
ATOM     38 HG21 VAL A   2      15.912  -6.806 -21.945  1.00 24.93           H  0.048
ATOM     39 HG22 VAL A   2      16.071  -6.301 -20.447  1.00 24.93           H  0.048
ATOM     40 HG23 VAL A   2      14.637  -6.322 -21.130  1.00 24.93           H  0.048
ATOM     41  N   PHE A   3      16.153  -1.429 -20.492  1.00 17.57           N  0.041
ANISOU   41  N   PHE A   3     2085   2013   2577  -1070   -545    766       N
ATOM     42  CA  PHE A   3      16.419  -0.049 -20.885  1.00 17.83           C  0.041
ANISOU   42  CA  PHE A   3     1977   2005   2791   -714   -651    910       C
ATOM     43  C   PHE A   3      17.515   0.025 -21.929  1.00 18.88           C  0.042
ANISOU   43  C   PHE A   3     1984   2017   3170   -368   -693    835       C
ATOM     44  O   PHE A   3      18.451  -0.780 -21.952  1.00 19.32           O  0.043
ANISOU   44  O   PHE A   3     1977   2055   3309   -155   -487    748       O
ATOM     45  CB  PHE A   3      16.864   0.778 -19.684  1.00 18.38           C  0.041
ANISOU   45  CB  PHE A   3     1913   1986   3083   -628   -524    859       C
ATOM     46  CG  PHE A   3      15.747   1.304 -18.833  1.00 18.96           C  0.042
ANISOU   46  CG  PHE A   3     1811   2059   3333   -559   -660    756       C
ATOM     47  CD1 PHE A   3      15.130   0.494 -17.878  1.00 19.22           C  0.042
ANISOU   47  CD1 PHE A   3     1840   2101   3360   -410   -550    813       C
ATOM     48  CD2 PHE A   3      15.365   2.646 -18.938  1.00 17.94           C  0.041
ANISOU   48  CD2 PHE A   3     1654   2039   3124   -549   -841    792       C
ATOM     49  CE1 PHE A   3      14.113   1.013 -17.077  1.00 19.02           C  0.042
ANISOU   49  CE1 PHE A   3     1922   2029   3275   -412   -397    970       C
ATOM     50  CE2 PHE A   3      14.375   3.155 -18.130  1.00 19.82           C  0.043
ANISOU   50  CE2 PHE A   3     1762   2166   3604   -395   -799    742       C
ATOM     51  CZ  PHE A   3      13.733   2.341 -17.210  1.00 20.19           C  0.043
ANISOU   51  CZ  PHE A   3     1846   2061   3763   -354   -610    754       C
ATOM     52  H   PHE A   3      16.443  -1.631 -19.708  1.00 21.23           H  0.045
ATOM     53  HA  PHE A   3      15.613   0.346 -21.253  1.00 21.54           H  0.045
ATOM     54  HB2 PHE A   3      17.428   0.226 -19.120  1.00 22.20           H  0.046
ATOM     55  HB3 PHE A   3      17.370   1.540 -20.006  1.00 22.20           H  0.046
ATOM     56  HD1 PHE A   3      15.379  -0.398 -17.794  1.00 23.21           H  0.047
ATOM     57  HD2 PHE A   3      15.785   3.198 -19.557  1.00 21.68           H  0.045
ATOM     58  HE1 PHE A   3      13.690   0.469 -16.452  1.00 22.97           H  0.046
ATOM     59  HE2 PHE A   3      14.114   4.043 -18.222  1.00 23.94           H  0.047
ATOM     60  HZ  PHE A   3      13.059   2.688 -16.672  1.00 24.37           H  0.048
ATOM     61  N   GLY A   4      17.421   1.067 -22.766  1.00 19.82           N  0.043
ANISOU   61  N   GLY A   4     2046   1932   3553   -468   -795    763       N
ATOM     62  CA  GLY A   4      18.570   1.522 -23.498  1.00 19.72           C  0.043
ANISOU   62  CA  GLY A   4     2070   1964   3460   -291   -737    745       C
ATOM     63  C   GLY A   4      19.460   2.383 -22.624  1.00 19.14           C  0.042
ANISOU   63  C   GLY A   4     2094   1834   3346   -347   -603    806       C
ATOM     64  O   GLY A   4      19.029   2.924 -21.603  1.00 19.40           O  0.043
ANISOU   64  O   GLY A   4     2025   1765   3580   -524   -225   1005       O
ATOM     65  H   GLY A   4      16.702   1.514 -22.916  1.00 23.93           H  0.047
```

```
ATOM     66  HA2 GLY A   4      19.083   0.761 -23.812  1.00 23.82           H  0.047
ATOM     67  HA3 GLY A   4      18.285   2.044 -24.264  1.00 23.82           H  0.047
ATOM     68  N   ARG A   5      20.718   2.495 -23.047  1.00 18.22           N  0.041
ANISOU   68  N   ARG A   5     2199   1823   2902   -181   -527    744       N
ATOM     69  CA  ARG A   5      21.718   3.235 -22.289  1.00 18.77           C  0.042
ANISOU   69  CA  ARG A   5     2230   1833   3068     33   -512    480       C
ATOM     70  C   ARG A   5      21.260   4.670 -21.999  1.00 17.75           C  0.041
ANISOU   70  C   ARG A   5     2111   1645   2989    183   -374    635       C
ATOM     71  O   ARG A   5      21.169   5.100 -20.836  1.00 15.71           O  0.038
ANISOU   71  O   ARG A   5     1974   1365   2632    295   -494    798       O
ATOM     72  CB  ARG A   5      23.014   3.225 -23.101  1.00 20.76           C  0.044
ANISOU   72  CB  ARG A   5     2449   1979   3458     57   -357    569       C
ATOM     73  CG  ARG A   5      24.134   3.846 -22.437  1.00 20.78           C  0.044
ANISOU   73  CG  ARG A   5     2563   1989   3344    124   -282    530       C
ATOM     74  CD  ARG A   5      25.378   3.739 -23.301  1.00 22.64           C  0.046
ANISOU   74  CD  ARG A   5     2673   2100   3828    295   -163    468       C
ATOM     75  NE  ARG A   5      25.227   4.309 -24.638  1.00 23.95           N  0.047
ANISOU   75  NE  ARG A   5     2921   2212   3968    331   -446    640       N
ATOM     76  CZ  ARG A   5      25.493   5.572 -24.957  1.00 24.63           C  0.048
ANISOU   76  CZ  ARG A   5     3145   2376   3838    381   -581    891       C
ATOM     77  NH1 ARG A   5      25.710   6.490 -24.032  1.00 23.94           N  0.047
ANISOU   77  NH1 ARG A   5     3101   2275   3719    419   -765    967       N
ATOM     78  NH2 ARG A   5      25.549   5.925 -26.239  1.00 26.72           N  0.050
ANISOU   78  NH2 ARG A   5     3367   2608   4177    360   -589    841       N
ATOM     79  H   ARG A   5      21.017   2.148 -23.775  1.00 22.02           H  0.045
ATOM     80  HA  ARG A   5      21.881   2.787 -21.444  1.00 22.67           H  0.046
ATOM     81  HB2 ARG A   5      23.256   2.305 -23.290  1.00 25.06           H  0.048
ATOM     82  HB3 ARG A   5      22.860   3.700 -23.932  1.00 25.06           H  0.048
ATOM     83  HG2 ARG A   5      23.946   4.786 -22.287  1.00 25.09           H  0.048
ATOM     84  HG3 ARG A   5      24.303   3.395 -21.595  1.00 25.09           H  0.048
ATOM     85  HD2 ARG A   5      26.106   4.203 -22.859  1.00 27.31           H  0.051
ATOM     86  HD3 ARG A   5      25.604   2.801 -23.405  1.00 27.31           H  0.051
ATOM     87  HE  ARG A   5      24.946   3.790 -25.264  1.00 28.89           H  0.052
ATOM     88 HH11 ARG A   5      25.687   6.278 -23.199  1.00 28.87           H  0.052
ATOM     89 HH12 ARG A   5      25.876   7.301 -24.264  1.00 28.87           H  0.052
ATOM     90 HH21 ARG A   5      25.403   5.340 -26.853  1.00 32.21           H  0.055
ATOM     91 HH22 ARG A   5      25.707   6.743 -26.453  1.00 32.21           H  0.055
ATOM     92  N   CYS A   6      20.966   5.427 -23.052  1.00 18.52           N  0.042
ANISOU   92  N   CYS A   6     2123   1673   3241    221   -150    590       N
ATOM     93  CA  CYS A   6      20.601   6.822 -22.856  1.00 17.73           C  0.041
ANISOU   93  CA  CYS A   6     2093   1756   2887    211   -390    803       C
ATOM     94  C   CYS A   6      19.261   6.972 -22.165  1.00 17.60           C  0.041
ANISOU   94  C   CYS A   6     1907   1763   3016      8   -313    965       C
ATOM     95  O   CYS A   6      19.066   7.926 -21.411  1.00 18.65           O  0.042
ANISOU   95  O   CYS A   6     1881   1838   3367     98   -136    895       O
ATOM     96  CB  CYS A   6      20.581   7.556 -24.183  1.00 17.83           C  0.041
ANISOU   96  CB  CYS A   6     2241   1811   2722     92   -316    902       C
ATOM     97  SG  CYS A   6      22.206   7.800 -24.871  1.00 18.96           S  0.042
ANISOU   97  SG  CYS A   6     2270   1909   3024     20   -104    681       S
ATOM     98  H   CYS A   6      20.970   5.164 -23.871  1.00 22.37           H  0.046
ATOM     99  HA  CYS A   6      21.266   7.239 -22.288  1.00 21.42           H  0.045
ATOM    100  HB2 CYS A   6      20.061   7.041 -24.820  1.00 21.54           H  0.045
ATOM    101  HB3 CYS A   6      20.177   8.428 -24.054  1.00 21.54           H  0.045
ATOM    102  N   GLU A   7      18.317   6.071 -22.450  1.00 17.97           N  0.041
ANISOU  102  N   GLU A   7     1796   1728   3306   -145   -255    946       N
ATOM    103  CA  GLU A   7      17.019   6.058 -21.771  1.00 18.06           C  0.041
ANISOU  103  CA  GLU A   7     1772   1743   3346   -171   -581   1144       C
ATOM    104  C   GLU A   7      17.182   5.899 -20.262  1.00 17.64           C  0.041
ANISOU  104  C   GLU A   7     1702   1582   3418   -119   -413   1158       C
ATOM    105  O   GLU A   7      16.509   6.586 -19.475  1.00 18.22           O  0.041
ANISOU  105  O   GLU A   7     1915   1548   3458   -318   -636   1100       O
ATOM    106  CB  GLU A   7      16.138   4.939 -22.332  1.00 22.46           C  0.046
ANISOU  106  CB  GLU A   7     2013   2113   4407   -239   -635   1270       C
ATOM    107  CG  GLU A   7      14.717   4.883 -21.775  1.00 25.56           C  0.049
ANISOU  107  CG  GLU A   7     2200   2394   5119   -274   -598   1442       C
ATOM    108  CD  GLU A   7      13.903   3.615 -22.214  1.00 27.58           C  0.051
ANISOU  108  CD  GLU A   7     2349   2494   5638   -305   -448   1805       C
ATOM    109  OE1 GLU A   7      14.521   2.560 -22.581  1.00 28.36           O  0.052
ANISOU  109  OE1 GLU A   7     2433   2694   5647   -261   -567   1997       O
ATOM    110  OE2 GLU A   7      12.651   3.675 -22.131  1.00 28.54           O  0.052
ANISOU  110  OE2 GLU A   7     2373   2532   5939   -283   -150   1793       O
ATOM    111  H   GLU A   7      18.406   5.451 -23.039  1.00 21.72           H  0.045
ATOM    112  HA  GLU A   7      16.570   6.902 -21.935  1.00 21.82           H  0.045
ATOM    113  HB2 GLU A   7      16.070   5.054 -23.293  1.00 27.10           H  0.050
ATOM    114  HB3 GLU A   7      16.561   4.088 -22.137  1.00 27.10           H  0.050
ATOM    115  HG2 GLU A   7      14.758   4.883 -20.806  1.00 30.83           H  0.054
ATOM    116  HG3 GLU A   7      14.232   5.665 -22.081  1.00 30.83           H  0.054
ATOM    117  N   LEU A   8      18.021   4.952 -19.833  1.00 18.16           N  0.041
```

```
ANISOU  117  N   LEU A   8     1533   1605   3763     10   -304    853       N
ATOM    118  CA  LEU A   8      18.224   4.778 -18.404  1.00 16.79           C  0.040
ANISOU  118  CA  LEU A   8     1335   1531   3513    216   -106    704       C
ATOM    119  C   LEU A   8      18.941   5.982 -17.811  1.00 16.25           C  0.039
ANISOU  119  C   LEU A   8     1242   1575   3357     74    -49    752       C
ATOM    120  O   LEU A   8      18.583   6.452 -16.722  1.00 16.42           O  0.039
ANISOU  120  O   LEU A   8     1338   1680   3220   -207    167    745       O
ATOM    121  CB  LEU A   8      19.063   3.524 -18.153  1.00 16.38           C  0.039
ANISOU  121  CB  LEU A   8     1388   1535   3301    174    -67    471       C
ATOM    122  CG  LEU A   8      19.301   3.187 -16.688  1.00 17.33           C  0.040
ANISOU  122  CG  LEU A   8     1473   1730   3380    202   -380    357       C
ATOM    123  CD1 LEU A   8      17.987   2.997 -15.949  1.00 18.22           C  0.041
ANISOU  123  CD1 LEU A   8     1619   1757   3548    135   -461    272       C
ATOM    124  CD2 LEU A   8      20.134   1.936 -16.538  1.00 17.10           C  0.040
ANISOU  124  CD2 LEU A   8     1527   1840   3130    202   -691    380       C
ATOM    125  H   LEU A   8      18.469   4.416 -20.335  1.00 21.95           H  0.045
ATOM    126  HA  LEU A   8      17.365   4.678 -17.965  1.00 20.30           H  0.044
ATOM    127  HB2 LEU A   8      18.613   2.766 -18.557  1.00 19.80           H  0.043
ATOM    128  HB3 LEU A   8      19.931   3.646 -18.569  1.00 19.80           H  0.043
ATOM    129  HG  LEU A   8      19.780   3.917 -16.266  1.00 20.94           H  0.044
ATOM    130 HD11 LEU A   8      18.170   2.626 -15.072  1.00 22.02           H  0.045
ATOM    131 HD12 LEU A   8      17.547   3.856 -15.858  1.00 22.02           H  0.045
ATOM    132 HD13 LEU A   8      17.426   2.388 -16.454  1.00 22.02           H  0.045
ATOM    133 HD21 LEU A   8      20.280   1.765 -15.594  1.00 20.67           H  0.044
ATOM    134 HD22 LEU A   8      19.660   1.191 -16.940  1.00 20.67           H  0.044
ATOM    135 HD23 LEU A   8      20.984   2.068 -16.986  1.00 20.67           H  0.044
ATOM    136  N   ALA A   9      19.947   6.501 -18.511  1.00 16.88           N  0.040
ANISOU  136  N   ALA A   9     1356   1631   3428    339   -458    586       N
ATOM    137  CA  ALA A   9      20.640   7.679 -18.014  1.00 16.39           C  0.039
ANISOU  137  CA  ALA A   9     1268   1606   3354    370   -206    432       C
ATOM    138  C   ALA A   9      19.654   8.812 -17.745  1.00 16.65           C  0.039
ANISOU  138  C   ALA A   9     1357   1549   3419    305    -92    715       C
ATOM    139  O   ALA A   9      19.706   9.463 -16.695  1.00 17.30           O  0.040
ANISOU  139  O   ALA A   9     1399   1605   3568    253   -259    636       O
ATOM    140  CB  ALA A   9      21.663   8.145 -19.038  1.00 17.36           C  0.040
ANISOU  140  CB  ALA A   9     1181   1769   3647    449    -77    286       C
ATOM    141  H   ALA A   9      20.240   6.196 -19.260  1.00 20.41           H  0.044
ATOM    142  HA  ALA A   9      21.101   7.464 -17.188  1.00 19.82           H  0.043
ATOM    143  HB1 ALA A   9      22.119   8.929 -18.694  1.00 20.98           H  0.044
ATOM    144  HB2 ALA A   9      22.302   7.432 -19.193  1.00 20.98           H  0.044
ATOM    145  HB3 ALA A   9      21.205   8.364 -19.864  1.00 20.98           H  0.044
ATOM    146  N   ALA A  10      18.700   9.023 -18.655  1.00 17.49           N  0.040
ANISOU  146  N   ALA A  10     1483   1495   3667    266    301    992       N
ATOM    147  CA  ALA A  10      17.745  10.103 -18.446  1.00 17.65           C  0.041
ANISOU  147  CA  ALA A  10     1514   1504   3686    187     -0    927       C
ATOM    148  C   ALA A  10      16.845   9.816 -17.254  1.00 18.35           C  0.041
ANISOU  148  C   ALA A  10     1580   1599   3792    319   -112    947       C
ATOM    149  O   ALA A  10      16.524  10.729 -16.466  1.00 19.43           O  0.043
ANISOU  149  O   ALA A  10     1874   1584   3926    467   -143    807       O
ATOM    150  CB  ALA A  10      16.937  10.325 -19.714  1.00 17.92           C  0.041
ANISOU  150  CB  ALA A  10     1461   1395   3953    -67   -277    821       C
ATOM    151  H   ALA A  10      18.589   8.570 -19.377  1.00 21.14           H  0.044
ATOM    152  HA  ALA A  10      18.233  10.920 -18.259  1.00 21.33           H  0.045
ATOM    153  HB1 ALA A  10      16.305  11.045 -19.563  1.00 21.65           H  0.045
ATOM    154  HB2 ALA A  10      17.541  10.560 -20.436  1.00 21.65           H  0.045
ATOM    155  HB3 ALA A  10      16.462   9.508 -19.932  1.00 21.65           H  0.045
ATOM    156  N   ALA A  11      16.384   8.569 -17.133  1.00 18.24           N  0.041
ANISOU  156  N   ALA A  11     1430   1757   3745    263   -294    939       N
ATOM    157  CA  ALA A  11      15.513   8.216 -16.022  1.00 17.88           C  0.041
ANISOU  157  CA  ALA A  11     1356   1681   3758    192   -167    947       C
ATOM    158  C   ALA A  11      16.259   8.322 -14.708  1.00 18.11           C  0.041
ANISOU  158  C   ALA A  11     1299   1682   3901    229    -11    770       C
ATOM    159  O   ALA A  11      15.707   8.819 -13.717  1.00 19.91           O  0.043
ANISOU  159  O   ALA A  11     1460   1710   4396    322    124    572       O
ATOM    160  CB  ALA A  11      14.968   6.794 -16.205  1.00 17.52           C  0.040
ANISOU  160  CB  ALA A  11     1348   1716   3593    150   -301   1059       C
ATOM    161  H   ALA A  11      16.559   7.923 -17.673  1.00 22.04           H  0.045
ATOM    162  HA  ALA A  11      14.762   8.829 -15.996  1.00 21.61           H  0.045
ATOM    163  HB1 ALA A  11      14.391   6.579 -15.456  1.00 21.17           H  0.045
ATOM    164  HB2 ALA A  11      14.464   6.753 -17.033  1.00 21.17           H  0.045
ATOM    165  HB3 ALA A  11      15.712   6.173 -16.239  1.00 21.17           H  0.045
ATOM    166  N   MET A  12      17.508   7.843 -14.672  1.00 15.96           N  0.039
ANISOU  166  N   MET A  12     1098   1660   3307    157   -448    619       N
ATOM    167  CA  MET A  12      18.278   7.966 -13.440  1.00 15.28           C  0.038
ANISOU  167  CA  MET A  12     1126   1602   3078    138   -537    627       C
ATOM    168  C   MET A  12      18.532   9.430 -13.108  1.00 16.56           C  0.039
ANISOU  168  C   MET A  12     1291   1734   3268     77   -363    553       C
ATOM    169  O   MET A  12      18.580   9.807 -11.928  1.00 16.82           O  0.040
```

```
ANISOU  169  O   MET A  12     1447   1735   3210     -7   -202    382       O
ATOM    170  CB  MET A  12      19.613   7.217 -13.495  1.00 14.95           C  0.037
ANISOU  170  CB  MET A  12     1018   1511   3150    124   -377    627       C
ATOM    171  CG  MET A  12      19.481   5.709 -13.346  1.00 14.23           C  0.036
ANISOU  171  CG  MET A  12      950   1340   3119    -47    -99    593       C
ATOM    172  SD  MET A  12      21.035   4.978 -13.789  1.00 14.41           S  0.037
ANISOU  172  SD  MET A  12     1139   1236   3100   -132   -157    261       S
ATOM    173  CE  MET A  12      20.849   3.623 -12.581  1.00 15.74           C  0.038
ANISOU  173  CE  MET A  12     1195   1276   3511   -305    -47    445       C
ATOM    174  H   MET A  12      17.916   7.457 -15.324  1.00 19.30           H  0.043
ATOM    175  HA  MET A  12      17.760   7.576 -12.719  1.00 18.49           H  0.042
ATOM    176  HB2 MET A  12      20.035   7.394 -14.350  1.00 18.09           H  0.041
ATOM    177  HB3 MET A  12      20.180   7.537 -12.776  1.00 18.09           H  0.041
ATOM    178  HG2 MET A  12      19.272   5.480 -12.428  1.00 17.23           H  0.040
ATOM    179  HG3 MET A  12      18.797   5.378 -13.948  1.00 17.23           H  0.040
ATOM    180  HE1 MET A  12      21.588   3.004 -12.686  1.00 19.04           H  0.042
ATOM    181  HE2 MET A  12      20.854   3.995 -11.685  1.00 19.04           H  0.042
ATOM    182  HE3 MET A  12      20.009   3.167 -12.746  1.00 19.04           H  0.042
ATOM    183  N   LYS A  13      18.742  10.274 -14.131  1.00 17.90           N  0.041
ANISOU  183  N   LYS A  13     1490   1753   3558     25   -324    587       N
ATOM    184  CA  LYS A  13      18.845  11.711 -13.876  1.00 18.97           C  0.042
ANISOU  184  CA  LYS A  13     1721   1623   3866    -90   -134    819       C
ATOM    185  C   LYS A  13      17.543  12.265 -13.313  1.00 20.60           C  0.044
ANISOU  185  C   LYS A  13     1872   1679   4277    214      4    710       C
ATOM    186  O   LYS A  13      17.562  13.060 -12.364  1.00 20.85           O  0.044
ANISOU  186  O   LYS A  13     1828   1518   4575    316     81    790       O
ATOM    187  CB  LYS A  13      19.275  12.516 -15.121  1.00 20.43           C  0.044
ANISOU  187  CB  LYS A  13     2030   1538   4194   -284   -177    960       C
ATOM    188  CG  LYS A  13      19.732  13.984 -14.830  1.00 22.61           C  0.046
ANISOU  188  CG  LYS A  13     2300   1642   4649   -403   -246    990       C
ATOM    189  CD  LYS A  13      20.310  14.645 -16.094  1.00 25.60           C  0.049
ANISOU  189  CD  LYS A  13     2576   1734   5417   -476   -335    821       C
ATOM    190  CE  LYS A  13      21.192  15.889 -15.824  1.00 28.17           C  0.051
ANISOU  190  CE  LYS A  13     2757   1972   5976   -366   -553    584       C
ATOM    191  NZ  LYS A  13      22.382  15.661 -14.933  1.00 30.11           N  0.053
ANISOU  191  NZ  LYS A  13     2868   2270   6304   -371   -615    440       N
ATOM    192  H   LYS A  13      18.825  10.044 -14.956  1.00 21.63           H  0.045
ATOM    193  HA  LYS A  13      19.528  11.847 -13.200  1.00 22.92           H  0.046
ATOM    194  HB2 LYS A  13      20.017  12.057 -15.545  1.00 24.67           H  0.048
ATOM    195  HB3 LYS A  13      18.525  12.559 -15.735  1.00 24.67           H  0.048
ATOM    196  HG2 LYS A  13      18.969  14.505 -14.534  1.00 27.28           H  0.051
ATOM    197  HG3 LYS A  13      20.421  13.977 -14.147  1.00 27.28           H  0.051
ATOM    198  HD2 LYS A  13      20.856  13.994 -16.563  1.00 30.87           H  0.054
ATOM    199  HD3 LYS A  13      19.575  14.924 -16.662  1.00 30.87           H  0.054
ATOM    200  HE2 LYS A  13      21.524  16.219 -16.673  1.00 33.96           H  0.056
ATOM    201  HE3 LYS A  13      20.642  16.570 -15.405  1.00 33.96           H  0.056
ATOM    202  HZ1 LYS A  13      22.837  16.420 -14.833  1.00 36.29           H  0.058
ATOM    203  HZ2 LYS A  13      22.113  15.378 -14.133  1.00 36.29           H  0.058
ATOM    204  HZ3 LYS A  13      22.918  15.048 -15.292  1.00 36.29           H  0.058
ATOM    205  N   ARG A  14      16.397  11.855 -13.870  1.00 21.62           N  0.045
ANISOU  205  N   ARG A  14     1992   1783   4439    347    103    964       N
ATOM    206  CA  ARG A  14      15.137  12.379 -13.353  1.00 24.72           C  0.048
ANISOU  206  CA  ARG A  14     2286   2098   5008    578    118   1067       C
ATOM    207  C   ARG A  14      14.926  11.949 -11.899  1.00 23.95           C  0.047
ANISOU  207  C   ARG A  14     2145   1988   4968    597    313    612       C
ATOM    208  O   ARG A  14      14.217  12.623 -11.149  1.00 24.37           O  0.048
ANISOU  208  O   ARG A  14     2252   1874   5136    703    296    530       O
ATOM    209  CB  ARG A  14      13.976  11.927 -14.241  1.00 28.88           C  0.052
ANISOU  209  CB  ARG A  14     2593   2662   5720    830     79   1179       C
ATOM    210  CG  ARG A  14      12.612  12.533 -13.917  1.00 33.74           C  0.056
ANISOU  210  CG  ARG A  14     2957   3230   6634    966     15   1080       C
ATOM    211  CD  ARG A  14      12.357  13.846 -14.684  1.00 36.99           C  0.059
ANISOU  211  CD  ARG A  14     3226   3614   7215   1185   -171   1075       C
ATOM    212  NE  ARG A  14      10.922  14.078 -14.845  1.00 39.66           N  0.061
ANISOU  212  NE  ARG A  14     3491   3936   7641   1378   -364   1045       N
ATOM    213  CZ  ARG A  14      10.175  14.758 -13.986  1.00 41.88           C  0.063
ANISOU  213  CZ  ARG A  14     3682   4304   7927   1391   -684    956       C
ATOM    214  NH1 ARG A  14      10.715  15.408 -12.965  1.00 43.36           N  0.064
ANISOU  214  NH1 ARG A  14     3822   4467   8186   1327   -722    800       N
ATOM    215  NH2 ARG A  14       8.854  14.783 -14.150  1.00 41.73           N  0.062
ANISOU  215  NH2 ARG A  14     3657   4370   7830   1519   -885   1018       N
ATOM    216  H   ARG A  14      16.326  11.298 -14.521  1.00 26.09           H  0.049
ATOM    217  HA  ARG A  14      15.168  13.348 -13.375  1.00 29.81           H  0.053
ATOM    218  HB2 ARG A  14      14.188  12.154 -15.160  1.00 34.81           H  0.057
ATOM    219  HB3 ARG A  14      13.889  10.964 -14.160  1.00 34.81           H  0.057
ATOM    220  HG2 ARG A  14      11.919  11.901 -14.164  1.00 40.64           H  0.062
ATOM    221  HG3 ARG A  14      12.562  12.725 -12.968  1.00 40.64           H  0.062
ATOM    222  HD2 ARG A  14      12.750  14.588 -14.201  1.00 44.54           H  0.065
```

```
ATOM    223  HD3 ARG A  14      12.749  13.781 -15.569  1.00 44.54           H  0.065
ATOM    224  HE  ARG A  14      10.538  13.752 -15.542  1.00 47.74           H  0.067
ATOM    225 HH11 ARG A  14      11.565  15.397 -12.840  1.00 52.18           H  0.070
ATOM    226 HH12 ARG A  14      10.210  15.844 -12.422  1.00 52.18           H  0.070
ATOM    227 HH21 ARG A  14       8.495  14.368 -14.812  1.00 50.23           H  0.069
ATOM    228 HH22 ARG A  14       8.361  15.228 -13.603  1.00 50.23           H  0.069
ATOM    229  N   HIS A  15      15.533  10.817 -11.516  1.00 22.13           N  0.046
ANISOU  229  N   HIS A  15     1912   1745   4752    537    301    472       N
ATOM    230  CA  HIS A  15      15.425  10.320 -10.125  1.00 21.52           C  0.045
ANISOU  230  CA  HIS A  15     1848   1765   4562    396    600    544       C
ATOM    231  C   HIS A  15      16.532  10.815  -9.196  1.00 21.16           C  0.045
ANISOU  231  C   HIS A  15     2048   1695   4297    388    547    417       C
ATOM    232  O   HIS A  15      16.607  10.344  -8.079  1.00 22.03           O  0.045
ANISOU  232  O   HIS A  15     2192   1558   4620    195    658    592       O
ATOM    233  CB  HIS A  15      15.266   8.797 -10.182  1.00 21.98           C  0.045
ANISOU  233  CB  HIS A  15     1782   1885   4686    291    550    912       C
ATOM    234  CG  HIS A  15      13.926   8.363 -10.660  1.00 24.06           C  0.047
ANISOU  234  CG  HIS A  15     1856   2154   5130    176    407   1127       C
ATOM    235  ND1 HIS A  15      12.980   7.831  -9.815  1.00 25.93           N  0.049
ANISOU  235  ND1 HIS A  15     1882   2378   5591    168    473    988       N
ATOM    236  CD2 HIS A  15      13.387   8.347 -11.894  1.00 25.25           C  0.049
ANISOU  236  CD2 HIS A  15     1879   2249   5466    219    112   1505       C
ATOM    237  CE1 HIS A  15      11.899   7.545 -10.507  1.00 26.49           C  0.050
ANISOU  237  CE1 HIS A  15     1832   2462   5771    216    183   1209       C
ATOM    238  NE2 HIS A  15      12.127   7.837 -11.780  1.00 26.83           N  0.050
ANISOU  238  NE2 HIS A  15     1842   2565   5786    223   -214   1540       N
ATOM    239  H   HIS A  15      16.008  10.319 -12.032  1.00 26.71           H  0.050
ATOM    240  HA  HIS A  15      14.595  10.664  -9.759  1.00 25.97           H  0.049
ATOM    241  HB2 HIS A  15      15.933   8.431 -10.783  1.00 26.53           H  0.050
ATOM    242  HB3 HIS A  15      15.389   8.438  -9.291  1.00 26.53           H  0.050
ATOM    243  HD1 HIS A  15      13.069   7.732  -8.965  1.00 31.27           H  0.054
ATOM    244  HD2 HIS A  15      13.791   8.643 -12.677  1.00 30.45           H  0.053
ATOM    245  HE1 HIS A  15      11.118   7.174 -10.167  1.00 31.94           H  0.055
ATOM    246  N   GLY A  16      17.365  11.767  -9.645  1.00 20.82           N  0.044
ANISOU  246  N   GLY A  16     2020   1519   4373    425    444    156       N
ATOM    247  CA  GLY A  16      18.257  12.415  -8.722  1.00 20.93           C  0.044
ANISOU  247  CA  GLY A  16     2003   1527   4421    350    289     22       C
ATOM    248  C   GLY A  16      19.527  11.673  -8.447  1.00 20.02           C  0.043
ANISOU  248  C   GLY A  16     1955   1497   4155    331    178    144       C
ATOM    249  O   GLY A  16      20.211  11.999  -7.467  1.00 20.99           O  0.044
ANISOU  249  O   GLY A  16     2072   1493   4412    366   -117    197       O
ATOM    250  H   GLY A  16      17.421  12.038 -10.459  1.00 25.14           H  0.049
ATOM    251  HA2 GLY A  16      18.492  13.289  -9.072  1.00 25.26           H  0.049
ATOM    252  HA3 GLY A  16      17.797  12.544  -7.878  1.00 25.26           H  0.049
ATOM    253  N   LEU A  17      19.877  10.703  -9.291  1.00 18.86           N  0.042
ANISOU  253  N   LEU A  17     1924   1365   3879    170    325    351       N
ATOM    254  CA  LEU A  17      21.154  10.013  -9.116  1.00 16.74           C  0.040
ANISOU  254  CA  LEU A  17     1789   1188   3383    207    167     76       C
ATOM    255  C   LEU A  17      22.385  10.758  -9.635  1.00 17.42           C  0.040
ANISOU  255  C   LEU A  17     1858   1225   3537    160    -37     30       C
ATOM    256  O   LEU A  17      23.502  10.362  -9.275  1.00 17.36           O  0.040
ANISOU  256  O   LEU A  17     1666   1302   3628    191   -276    -41       O
ATOM    257  CB  LEU A  17      21.118   8.664  -9.812  1.00 16.22           C  0.039
ANISOU  257  CB  LEU A  17     1801   1233   3130    149     38     10       C
ATOM    258  CG  LEU A  17      20.393   7.636  -8.977  1.00 16.99           C  0.040
ANISOU  258  CG  LEU A  17     1898   1250   3308    -45    108    143       C
ATOM    259  CD1 LEU A  17      20.411   6.318  -9.739  1.00 16.04           C  0.039
ANISOU  259  CD1 LEU A  17     1832   1166   3098   -297    225    237       C
ATOM    260  CD2 LEU A  17      21.070   7.484  -7.610  1.00 16.90           C  0.040
ANISOU  260  CD2 LEU A  17     1911   1176   3335    -29    279    152       C
ATOM    261  H   LEU A  17      19.406  10.431  -9.957  1.00 22.79           H  0.046
ATOM    262  HA  LEU A  17      21.294   9.883  -8.168  1.00 20.24           H  0.044
ATOM    263  HB2 LEU A  17      20.653   8.752 -10.659  1.00 19.62           H  0.043
ATOM    264  HB3 LEU A  17      22.025   8.354  -9.958  1.00 19.62           H  0.043
ATOM    265  HG  LEU A  17      19.472   7.909  -8.843  1.00 20.54           H  0.044
ATOM    266 HD11 LEU A  17      19.911   5.655  -9.237  1.00 19.40           H  0.043
ATOM    267 HD12 LEU A  17      20.002   6.454 -10.607  1.00 19.40           H  0.043
ATOM    268 HD13 LEU A  17      21.330   6.029  -9.848  1.00 19.40           H  0.043
ATOM    269 HD21 LEU A  17      20.938   6.577  -7.292  1.00 20.43           H  0.044
ATOM    270 HD22 LEU A  17      22.018   7.666  -7.706  1.00 20.43           H  0.044
ATOM    271 HD23 LEU A  17      20.674   8.114  -6.988  1.00 20.43           H  0.044
ATOM    272  N   ASP A  18      22.219  11.769 -10.502  1.00 17.00           N  0.040
ANISOU  272  N   ASP A  18     1982   1193   3282     60    144    172       N
ATOM    273  CA  ASP A  18      23.397  12.433 -11.054  1.00 19.08           C  0.042
ANISOU  273  CA  ASP A  18     2126   1283   3840    110   -126     70       C
ATOM    274  C   ASP A  18      24.107  13.164  -9.929  1.00 18.26           C  0.041
ANISOU  274  C   ASP A  18     2065   1195   3679     92     13    -17       C
ATOM    275  O   ASP A  18      23.567  14.116  -9.349  1.00 17.72           O  0.041
```

```
ANISOU  275  O   ASP A  18     1988   1286   3456    378    164    -45       O
ATOM    276  CB  ASP A  18      23.059  13.415 -12.165  1.00 21.55           C  0.045
ANISOU  276  CB  ASP A  18     2278   1469   4440    256   -306    233       C
ATOM    277  CG  ASP A  18      24.300  13.848 -12.977  1.00 23.19           C  0.047
ANISOU  277  CG  ASP A  18     2442   1678   4690    364   -644    343       C
ATOM    278  OD1 ASP A  18      25.444  13.398 -12.704  1.00 23.05           O  0.046
ANISOU  278  OD1 ASP A  18     2383   1760   4617    453   -757      2       O
ATOM    279  OD2 ASP A  18      24.142  14.727 -13.845  1.00 24.81           O  0.048
ANISOU  279  OD2 ASP A  18     2672   1868   4888    269   -733    865       O
ATOM    280  H   ASP A  18      21.463  12.076 -10.774  1.00 20.55           H  0.044
ATOM    281  HA  ASP A  18      24.002  11.765 -11.413  1.00 23.04           H  0.046
ATOM    282  HB2 ASP A  18      22.432  12.998 -12.776  1.00 26.00           H  0.049
ATOM    283  HB3 ASP A  18      22.663  14.210 -11.775  1.00 26.00           H  0.049
ATOM    284  N  AASN A  19      25.329  12.731  -9.631  0.51 17.46           N  0.040
ANISOU  284  N  AASN A  19     2030   1020   3582    151     96    165       N
ATOM    285  CA AASN A  19      26.139  13.279  -8.555  0.51 17.39           C  0.040
ANISOU  285  CA AASN A  19     2051    939   3619    114    123    217       C
ATOM    286  C  AASN A  19      25.588  12.944  -7.178  0.51 17.61           C  0.041
ANISOU  286  C  AASN A  19     1955    955   3781     99     -1    -29       C
ATOM    287  O  AASN A  19      26.023  13.535  -6.185  0.51 18.29           O  0.041
ANISOU  287  O  AASN A  19     1962   1057   3930    197   -177    -61       O
ATOM    288  CB AASN A  19      26.370  14.790  -8.701  0.51 17.94           C  0.041
ANISOU  288  CB AASN A  19     2091    994   3731    146    221    299       C
ATOM    289  CG AASN A  19      27.682  15.227  -8.109  0.51 18.72           C  0.042
ANISOU  289  CG AASN A  19     2179   1046   3888    209    295    271       C
ATOM    290  OD1AASN A  19      28.608  14.421  -7.987  0.51 19.26           O  0.042
ANISOU  290  OD1AASN A  19     2233   1122   3964    226    417    436       O
ATOM    291  ND2AASN A  19      27.796  16.512  -7.768  0.51 19.73           N  0.043
ANISOU  291  ND2AASN A  19     2226   1102   4168    250    188    103       N
ATOM    292  H  AASN A  19      25.722  12.096 -10.057  0.51 21.10           H  0.044
ATOM    293  HA AASN A  19      27.009  12.855  -8.612  0.51 21.02           H  0.044
ATOM    294  HB2AASN A  19      26.373  15.021  -9.643  0.51 21.68           H  0.045
ATOM    295  HB3AASN A  19      25.659  15.266  -8.243  0.51 21.68           H  0.045
ATOM    296 HD21AASN A  19      28.531  16.803  -7.429  0.51 23.82           H  0.047
ATOM    297 HD22AASN A  19      27.130  17.046  -7.874  0.51 23.82           H  0.047
ATOM    298  N  BASN A  19      25.318  12.713  -9.584  0.49 17.96           N  0.041
ANISOU  298  N  BASN A  19     2046   1147   3633     -6     19     64       N
ATOM    299  CA BASN A  19      26.151  13.329  -8.507  0.49 18.26           C  0.041
ANISOU  299  CA BASN A  19     2075   1115   3750   -196    -52     43       C
ATOM    300  C  BASN A  19      25.603  12.996  -7.118  0.49 17.48           C  0.040
ANISOU  300  C  BASN A  19     1926   1039   3678    -69   -145    -43       C
ATOM    301  O  BASN A  19      25.886  13.716  -6.162  0.49 17.15           O  0.040
ANISOU  301  O  BASN A  19     1856   1127   3535     22   -422     84       O
ATOM    302  CB BASN A  19      26.305  14.841  -8.696  0.49 19.92           C  0.043
ANISOU  302  CB BASN A  19     2188   1275   4106   -483    -91     17       C
ATOM    303  CG BASN A  19      27.590  15.224  -9.384  0.49 21.69           C  0.045
ANISOU  303  CG BASN A  19     2350   1522   4367   -707   -130   -119       C
ATOM    304  OD1BASN A  19      28.532  15.675  -8.736  0.49 22.91           O  0.046
ANISOU  304  OD1BASN A  19     2499   1585   4619   -748   -111   -140       O
ATOM    305  ND2BASN A  19      27.623  15.061 -10.696  0.49 21.21           N  0.045
ANISOU  305  ND2BASN A  19     2379   1594   4087   -833   -126   -305       N
ATOM    306  H  BASN A  19      25.930  12.294 -10.308  0.49 21.71           H  0.045
ATOM    307  HA BASN A  19      27.149  12.898  -8.581  0.49 22.07           H  0.045
ATOM    308  HB2BASN A  19      25.482  15.224  -9.293  0.49 24.06           H  0.047
ATOM    309  HB3BASN A  19      26.269  15.334  -7.727  0.49 24.06           H  0.047
ATOM    310 HD21BASN A  19      28.184  15.681 -11.273  0.49 25.60           H  0.049
ATOM    311 HD22BASN A  19      27.088  14.311 -11.126  0.49 25.60           H  0.049
ATOM    312  N   TYR A  20      24.660  11.996  -7.078  1.00 17.35           N  0.040
ANISOU  312  N   TYR A  20     1861    966   3766    -84    242     50       N
ATOM    313  CA  TYR A  20      24.215  11.553  -5.767  1.00 16.25           C  0.039
ANISOU  313  CA  TYR A  20     1885   1073   3214    -57    376    -99       C
ATOM    314  C   TYR A  20      25.395  10.967  -4.995  1.00 14.95           C  0.037
ANISOU  314  C   TYR A  20     1929    996   2754    -89    296   -278       C
ATOM    315  O   TYR A  20      26.108  10.075  -5.487  1.00 14.27           O  0.037
ANISOU  315  O   TYR A  20     1693    916   2815    -79    659   -286       O
ATOM    316  CB  TYR A  20      23.041  10.585  -5.865  1.00 17.77           C  0.041
ANISOU  316  CB  TYR A  20     1954   1236   3562   -100    235   -335       C
ATOM    317  CG  TYR A  20      22.431  10.350  -4.501  1.00 17.19           C  0.040
ANISOU  317  CG  TYR A  20     1889   1328   3317   -109    254   -524       C
ATOM    318  CD1 TYR A  20      21.483  11.226  -3.989  1.00 17.79           C  0.041
ANISOU  318  CD1 TYR A  20     1831   1369   3561    -67    341   -349       C
ATOM    319  CD2 TYR A  20      22.861   9.295  -3.693  1.00 18.02           C  0.041
ANISOU  319  CD2 TYR A  20     1808   1342   3696   -205     71   -453       C
ATOM    320  CE1 TYR A  20      20.970  11.060  -2.715  1.00 17.73           C  0.041
ANISOU  320  CE1 TYR A  20     1793   1382   3563     26    486   -392       C
ATOM    321  CE2 TYR A  20      22.372   9.138  -2.409  1.00 17.97           C  0.041
ANISOU  321  CE2 TYR A  20     1741   1390   3696   -254    123   -441       C
ATOM    322  CZ  TYR A  20      21.412  10.012  -1.934  1.00 17.70           C  0.041
```

```
ANISOU  322  CZ  TYR A  20     1810   1389   3525    -94    397   -363       C
ATOM    323  OH  TYR A  20      20.890   9.872  -0.675  1.00 18.90           O  0.042
ANISOU  323  OH  TYR A  20     1950   1436   3795     45    517   -204       O
ATOM    324  HA  TYR A  20      23.906  12.329  -5.273  1.00 19.64           H  0.043
ATOM    325  HB2 TYR A  20      22.360  10.962  -6.444  1.00 21.47           H  0.045
ATOM    326  HB3 TYR A  20      23.351   9.735  -6.214  1.00 21.47           H  0.045
ATOM    327  HD1 TYR A  20      21.194  11.941  -4.508  1.00 21.50           H  0.045
ATOM    328  HD2 TYR A  20      23.518   8.716  -4.007  1.00 21.77           H  0.045
ATOM    329  HE1 TYR A  20      20.331  11.651  -2.387  1.00 21.43           H  0.045
ATOM    330  HE2 TYR A  20      22.657   8.425  -1.884  1.00 21.71           H  0.045
ATOM    331  HH  TYR A  20      21.231   9.205  -0.295  1.00 22.83           H  0.046
ATOM    332  H  ATYR A  20      24.280  11.601  -7.741  0.51 20.97           H  0.044
ATOM    333  H  BTYR A  20      24.317  11.569  -7.741  0.49 20.97           H  0.044
ATOM    334  N  AARG A  21      25.626  11.496  -3.797  0.47 16.12           N  0.039
ANISOU  334  N  AARG A  21     1990   1137   3000   -186    114   -295       N
ATOM    335  CA AARG A  21      26.767  11.080  -2.987  0.47 16.86           C  0.040
ANISOU  335  CA AARG A  21     2042   1209   3154   -177     76   -366       C
ATOM    336  C  AARG A  21      28.089  11.276  -3.732  0.47 15.50           C  0.038
ANISOU  336  C  AARG A  21     1943   1063   2883    -77    -30   -457       C
ATOM    337  O  AARG A  21      29.072  10.565  -3.486  0.47 14.60           O  0.037
ANISOU  337  O  AARG A  21     1835    955   2758    -49   -143   -640       O
ATOM    338  CB AARG A  21      26.609   9.645  -2.470  0.47 19.50           C  0.043
ANISOU  338  CB AARG A  21     2247   1437   3726   -245      7   -168       C
ATOM    339  CG AARG A  21      25.759   9.509  -1.215  0.47 21.61           C  0.045
ANISOU  339  CG AARG A  21     2459   1570   4181   -400    -84     67       C
ATOM    340  CD AARG A  21      26.589   9.758   0.034  0.47 22.85           C  0.046
ANISOU  340  CD AARG A  21     2585   1671   4424   -565   -196    239       C
ATOM    341  NE AARG A  21      27.170  11.095   0.064  0.47 24.61           N  0.048
ANISOU  341  NE AARG A  21     2769   1774   4807   -599   -202    385       N
ATOM    342  CZ AARG A  21      28.144  11.481   0.874  0.47 25.63           C  0.049
ANISOU  342  CZ AARG A  21     2895   1818   5026   -616   -138    562       C
ATOM    343  NH1AARG A  21      28.756  10.622   1.679  0.47 26.74           N  0.050
ANISOU  343  NH1AARG A  21     2993   1978   5189   -554   -137    547       N
ATOM    344  NH2AARG A  21      28.517  12.757   0.871  0.47 25.22           N  0.049
ANISOU  344  NH2AARG A  21     2875   1729   4978   -727    -14    666       N
ATOM    345  H  AARG A  21      25.136  12.099  -3.429  0.47 19.50           H  0.043
ATOM    346  HA AARG A  21      26.797  11.669  -2.222  0.47 20.38           H  0.044
ATOM    347  HB2AARG A  21      26.195   9.108  -3.163  0.47 23.55           H  0.047
ATOM    348  HB3AARG A  21      27.487   9.288  -2.265  0.47 23.55           H  0.047
ATOM    349  HG2AARG A  21      25.043  10.162  -1.240  0.47 26.08           H  0.049
ATOM    350  HG3AARG A  21      25.395   8.613  -1.161  0.47 26.08           H  0.049
ATOM    351  HD2AARG A  21      26.023   9.659   0.815  0.47 27.56           H  0.051
ATOM    352  HD3AARG A  21      27.313   9.114   0.065  0.47 27.56           H  0.051
ATOM    353  HE AARG A  21      26.684  11.724  -0.262  0.47 29.68           H  0.053
ATOM    354 HH11AARG A  21      28.519   9.796   1.690  0.47 32.24           H  0.055
ATOM    355 HH12AARG A  21      29.388  10.894   2.195  0.47 32.24           H  0.055
ATOM    356 HH21AARG A  21      28.127  13.318   0.348  0.47 30.41           H  0.053
ATOM    357 HH22AARG A  21      29.153  13.020   1.387  0.47 30.41           H  0.053
ATOM    358  N  BARG A  21      25.613  11.485  -3.783  0.53 16.22           N  0.039
ANISOU  358  N  BARG A  21     1965   1150   3048   -147    158   -275       N
ATOM    359  CA BARG A  21      26.770  11.114  -2.955  0.53 16.93           C  0.040
ANISOU  359  CA BARG A  21     1986   1232   3216    -97    192   -338       C
ATOM    360  C  BARG A  21      28.094  11.306  -3.704  0.53 15.61           C  0.038
ANISOU  360  C  BARG A  21     1898   1081   2954    -27     12   -409       C
ATOM    361  O  BARG A  21      29.086  10.618  -3.438  0.53 14.90           O  0.037
ANISOU  361  O  BARG A  21     1760    962   2941      7   -154   -602       O
ATOM    362  CB BARG A  21      26.668   9.694  -2.384  0.53 19.45           C  0.043
ANISOU  362  CB BARG A  21     2163   1460   3767   -115    220   -156       C
ATOM    363  CG BARG A  21      26.119   9.559  -0.964  0.53 21.22           C  0.045
ANISOU  363  CG BARG A  21     2349   1580   4135   -236    224     95       C
ATOM    364  CD BARG A  21      26.913  10.337   0.067  0.53 21.86           C  0.045
ANISOU  364  CD BARG A  21     2483   1689   4133   -249    275    274       C
ATOM    365  NE BARG A  21      27.109   9.617   1.324  0.53 22.42           N  0.046
ANISOU  365  NE BARG A  21     2559   1846   4116   -254    263    439       N
ATOM    366  CZ BARG A  21      26.476   9.862   2.465  0.53 21.93           C  0.045
ANISOU  366  CZ BARG A  21     2579   1873   3879   -271    288    516       C
ATOM    367  NH1BARG A  21      25.423  10.658   2.517  0.53 21.14           N  0.044
ANISOU  367  NH1BARG A  21     2575   1855   3602   -268    378    722       N
ATOM    368  NH2BARG A  21      26.884   9.249   3.575  0.53 22.44           N  0.046
ANISOU  368  NH2BARG A  21     2588   1984   3955   -268     37    335       N
ATOM    369  H  BARG A  21      25.097  12.063  -3.411  0.53 19.62           H  0.043
ATOM    370  HA BARG A  21      26.791  11.721  -2.199  0.53 20.47           H  0.044
ATOM    371  HB2BARG A  21      26.085   9.179  -2.963  0.53 23.49           H  0.047
ATOM    372  HB3BARG A  21      27.550   9.292  -2.383  0.53 23.49           H  0.047
ATOM    373  HG2BARG A  21      25.207   9.889  -0.948  0.53 25.62           H  0.049
ATOM    374  HG3BARG A  21      26.136   8.623  -0.709  0.53 25.62           H  0.049
ATOM    375  HD2BARG A  21      27.790  10.529  -0.300  0.53 26.38           H  0.050
ATOM    376  HD3BARG A  21      26.452  11.168   0.258  0.53 26.38           H  0.050
```

```
ATOM    377  HE BARG A  21      27.685   8.978   1.324  0.53 27.06           H  0.050
ATOM    378 HH11BARG A  21      25.149  11.060   1.809  0.53 25.52           H  0.049
ATOM    379 HH12BARG A  21      25.024  10.792   3.267  0.53 25.52           H  0.049
ATOM    380 HH21BARG A  21      27.561   8.719   3.550  0.53 27.08           H  0.050
ATOM    381 HH22BARG A  21      26.472   9.385   4.317  0.53 27.08           H  0.050
ATOM    382  N   GLY A  22      28.107  12.228  -4.668  1.00 15.77           N  0.038
ANISOU  382  N   GLY A  22     1986   1056   2951    -48    -51   -443       N
ATOM    383  CA  GLY A  22      29.280  12.511  -5.461  1.00 16.46           C  0.039
ANISOU  383  CA  GLY A  22     1935    972   3347    -73    108   -246       C
ATOM    384  C   GLY A  22      29.480  11.611  -6.665  1.00 16.58           C  0.039
ANISOU  384  C   GLY A  22     1709   1013   3576    -81     90    -14       C
ATOM    385  O   GLY A  22      30.522  11.722  -7.327  1.00 15.99           O  0.039
ANISOU  385  O   GLY A  22     1636   1140   3297    -83     59    -79       O
ATOM    386  HA2 GLY A  22      29.230  13.426  -5.778  1.00 19.90           H  0.043
ATOM    387  HA3 GLY A  22      30.066  12.433  -4.898  1.00 19.90           H  0.043
ATOM    388  H  AGLY A  22      27.434  12.728  -4.858  0.47 19.08           H  0.042
ATOM    389  H  BGLY A  22      27.427  12.710  -4.879  0.53 19.08           H  0.042
ATOM    390  N   TYR A  23      28.520  10.721  -6.991  1.00 15.53           N  0.038
ANISOU  390  N   TYR A  23     1449    961   3489   -222    -79     79       N
ATOM    391  CA  TYR A  23      28.731   9.767  -8.081  1.00 14.22           C  0.036
ANISOU  391  CA  TYR A  23     1394    979   3029   -297     59    246       C
ATOM    392  C   TYR A  23      28.053  10.271  -9.348  1.00 13.24           C  0.035
ANISOU  392  C   TYR A  23     1452   1088   2491   -195    113     98       C
ATOM    393  O   TYR A  23      26.823  10.311  -9.430  1.00 12.78           O  0.035
ANISOU  393  O   TYR A  23     1422   1012   2422   -388    151    202       O
ATOM    394  CB  TYR A  23      28.251   8.372  -7.690  1.00 13.18           C  0.035
ANISOU  394  CB  TYR A  23     1290   1029   2687   -457     70    125       C
ATOM    395  CG  TYR A  23      29.182   7.740  -6.679  1.00 12.65           C  0.034
ANISOU  395  CG  TYR A  23     1103   1186   2516   -505   -116    -43       C
ATOM    396  CD1 TYR A  23      30.330   7.062  -7.111  1.00 11.05           C  0.032
ANISOU  396  CD1 TYR A  23      913   1125   2161   -436   -240     85       C
ATOM    397  CD2 TYR A  23      28.929   7.822  -5.313  1.00 13.00           C  0.035
ANISOU  397  CD2 TYR A  23     1050   1251   2638   -441    316    -96       C
ATOM    398  CE1 TYR A  23      31.170   6.463  -6.217  1.00  9.69           C  0.030
ANISOU  398  CE1 TYR A  23      728   1135   1820   -375    -86   -221       C
ATOM    399  CE2 TYR A  23      29.743   7.246  -4.408  1.00 12.30           C  0.034
ANISOU  399  CE2 TYR A  23      937   1383   2352   -501    181   -177       C
ATOM    400  CZ  TYR A  23      30.884   6.569  -4.868  1.00 11.68           C  0.033
ANISOU  400  CZ  TYR A  23      831   1313   2294   -430     68   -118       C
ATOM    401  OH  TYR A  23      31.746   5.970  -3.991  1.00 12.29           O  0.034
ANISOU  401  OH  TYR A  23      904   1489   2276   -267     22     -7       O
ATOM    402  H   TYR A  23      27.756  10.656  -6.601  1.00 18.78           H  0.042
ATOM    403  HA  TYR A  23      29.681   9.705  -8.263  1.00 17.21           H  0.040
ATOM    404  HB2 TYR A  23      27.368   8.435  -7.294  1.00 15.96           H  0.039
ATOM    405  HB3 TYR A  23      28.228   7.807  -8.478  1.00 15.96           H  0.039
ATOM    406  HD1 TYR A  23      30.507   6.996  -8.022  1.00 13.41           H  0.035
ATOM    407  HD2 TYR A  23      28.169   8.270  -5.019  1.00 15.75           H  0.038
ATOM    408  HE1 TYR A  23      31.931   6.014  -6.507  1.00 11.78           H  0.033
ATOM    409  HE2 TYR A  23      29.561   7.307  -3.498  1.00 14.90           H  0.037
ATOM    410  HH  TYR A  23      31.568   6.209  -3.206  1.00 14.90           H  0.037
ATOM    411  N   SER A  24      28.864  10.667 -10.331  1.00 14.82           N  0.037
ANISOU  411  N   SER A  24     1547   1275   2809   -108    290     92       N
ATOM    412  CA  SER A  24      28.302  11.153 -11.576  1.00 15.74           C  0.038
ANISOU  412  CA  SER A  24     1677   1331   2973     -7    364    113       C
ATOM    413  C   SER A  24      27.415  10.094 -12.225  1.00 15.07           C  0.038
ANISOU  413  C   SER A  24     1661   1253   2811    -30    285    416       C
ATOM    414  O   SER A  24      27.567   8.877 -12.004  1.00 14.67           O  0.037
ANISOU  414  O   SER A  24     1729   1214   2633    202    321    222       O
ATOM    415  CB  SER A  24      29.402  11.550 -12.553  1.00 16.95           C  0.040
ANISOU  415  CB  SER A  24     1646   1476   3319     67    513    391       C
ATOM    416  OG  SER A  24      30.058  10.373 -12.972  1.00 17.82           O  0.041
ANISOU  416  OG  SER A  24     1636   1735   3401    335    562    367       O
ATOM    417  H   SER A  24      29.723  10.662 -10.298  1.00 17.94           H  0.041
ATOM    418  HA  SER A  24      27.758  11.936 -11.397  1.00 19.04           H  0.042
ATOM    419  HB2 SER A  24      29.010  11.994 -13.321  1.00 20.49           H  0.044
ATOM    420  HB3 SER A  24      30.036  12.134 -12.108  1.00 20.49           H  0.044
ATOM    421  HG  SER A  24      30.672  10.563 -13.513  1.00 21.54           H  0.045
ATOM    422  N   LEU A  25      26.508  10.581 -13.075  1.00 16.28           N  0.039
ANISOU  422  N   LEU A  25     1656   1326   3204    -95    170    474       N
ATOM    423  CA  LEU A  25      25.505   9.724 -13.699  1.00 14.94           C  0.037
ANISOU  423  CA  LEU A  25     1535   1121   3021    -69     70    619       C
ATOM    424  C   LEU A  25      26.126   8.522 -14.389  1.00 14.16           C  0.036
ANISOU  424  C   LEU A  25     1333   1051   2997    -79     43    458       C
ATOM    425  O   LEU A  25      25.538   7.437 -14.371  1.00 13.04           O  0.035
ANISOU  425  O   LEU A  25     1228    962   2763     -6    353     79       O
ATOM    426  CB  LEU A  25      24.736  10.543 -14.727  1.00 15.97           C  0.039
ANISOU  426  CB  LEU A  25     1657   1340   3071    -64    -49    469       C
ATOM    427  CG  LEU A  25      23.515   9.912 -15.329  1.00 15.52           C  0.038
```

```
ANISOU  427  CG  LEU A  25     1644   1237   3015   -183   -181    651       C
ATOM    428  CD1 LEU A  25      22.519   9.547 -14.235  1.00 17.23           C  0.040
ANISOU  428  CD1 LEU A  25     1720   1474   3351   -137   -160    546       C
ATOM    429  CD2 LEU A  25      22.878  10.925 -16.280  1.00 15.85           C  0.039
ANISOU  429  CD2 LEU A  25     1620   1232   3169   -339   -402    762       C
ATOM    430  H   LEU A  25      26.455  11.408 -13.306  1.00 19.69           H  0.043
ATOM    431  HA  LEU A  25      24.888   9.407 -13.021  1.00 18.08           H  0.041
ATOM    432  HB2 LEU A  25      24.451  11.368 -14.303  1.00 19.31           H  0.043
ATOM    433  HB3 LEU A  25      25.339  10.752 -15.458  1.00 19.31           H  0.043
ATOM    434  HG  LEU A  25      23.756   9.114 -15.824  1.00 18.77           H  0.042
ATOM    435 HD11 LEU A  25      21.666   9.334 -14.645  1.00 20.82           H  0.044
ATOM    436 HD12 LEU A  25      22.851   8.778 -13.747  1.00 20.82           H  0.044
ATOM    437 HD13 LEU A  25      22.417  10.303 -13.636  1.00 20.82           H  0.044
ATOM    438 HD21 LEU A  25      22.090  10.528 -16.682  1.00 19.16           H  0.042
ATOM    439 HD22 LEU A  25      22.630  11.717 -15.778  1.00 19.16           H  0.042
ATOM    440 HD23 LEU A  25      23.520  11.157 -16.969  1.00 19.16           H  0.042
ATOM    441  N   GLY A  26      27.282   8.683 -15.017  1.00 13.56           N  0.036
ANISOU  441  N   GLY A  26     1162   1005   2985   -211     55    441       N
ATOM    442  CA  GLY A  26      27.848   7.582 -15.765  1.00 11.86           C  0.033
ANISOU  442  CA  GLY A  26     1177   1185   2144   -355    218    586       C
ATOM    443  C   GLY A  26      28.227   6.436 -14.861  1.00 12.60           C  0.034
ANISOU  443  C   GLY A  26     1243   1326   2219   -343     -7    502       C
ATOM    444  O   GLY A  26      28.258   5.279 -15.294  1.00 13.64           O  0.036
ANISOU  444  O   GLY A  26     1261   1621   2300   -316   -227    407       O
ATOM    445  H   GLY A  26      27.748   9.406 -15.024  1.00 16.42           H  0.039
ATOM    446  HA2 GLY A  26      27.203   7.264 -16.416  1.00 14.38           H  0.037
ATOM    447  HA3 GLY A  26      28.641   7.882 -16.235  1.00 14.38           H  0.037
ATOM    448  N   ASN A  27      28.502   6.730 -13.591  1.00 12.66           N  0.034
ANISOU  448  N   ASN A  27     1165   1256   2389   -344   -156    -77       N
ATOM    449  CA  ASN A  27      28.820   5.663 -12.657  1.00 12.75           C  0.035
ANISOU  449  CA  ASN A  27      908   1177   2759   -157   -258    184       C
ATOM    450  C   ASN A  27      27.598   4.791 -12.436  1.00 11.50           C  0.033
ANISOU  450  C   ASN A  27      844    999   2525    -91   -221    173       C
ATOM    451  O   ASN A  27      27.721   3.563 -12.320  1.00 11.27           O  0.032
ANISOU  451  O   ASN A  27      751    926   2605     37    161    -19       O
ATOM    452  CB  ASN A  27      29.266   6.237 -11.315  1.00 11.72           C  0.033
ANISOU  452  CB  ASN A  27      928   1151   2372   -158    -60    304       C
ATOM    453  CG  ASN A  27      30.690   6.683 -11.323  1.00 12.63           C  0.034
ANISOU  453  CG  ASN A  27     1162    974   2663   -223   -145    117       C
ATOM    454  OD1 ASN A  27      31.586   5.855 -11.261  1.00 12.51           O  0.034
ANISOU  454  OD1 ASN A  27     1263    765   2725   -127   -325    292       O
ATOM    455  ND2 ASN A  27      30.915   7.992 -11.387  1.00 14.00           N  0.036
ANISOU  455  ND2 ASN A  27     1278   1092   2949   -395   -244   -148       N
ATOM    456  H   ASN A  27      28.511   7.521 -13.254  1.00 15.34           H  0.038
ATOM    457  HA  ASN A  27      29.535   5.114 -13.016  1.00 15.45           H  0.038
ATOM    458  HB2 ASN A  27      28.712   7.001 -11.095  1.00 14.21           H  0.036
ATOM    459  HB3 ASN A  27      29.172   5.553 -10.634  1.00 14.21           H  0.036
ATOM    460 HD21 ASN A  27      31.722   8.290 -11.393  1.00 16.95           H  0.040
ATOM    461 HD22 ASN A  27      30.254   8.541 -11.427  1.00 16.95           H  0.040
ATOM    462  N   TRP A  28      26.415   5.416 -12.360  1.00 12.80           N  0.035
ANISOU  462  N   TRP A  28      743   1280   2841   -134     -1     87       N
ATOM    463  CA  TRP A  28      25.184   4.662 -12.123  1.00 12.06           C  0.034
ANISOU  463  CA  TRP A  28      698   1206   2678   -112     -5    168       C
ATOM    464  C   TRP A  28      24.777   3.858 -13.351  1.00 11.85           C  0.033
ANISOU  464  C   TRP A  28      728   1200   2574   -106    -52    502       C
ATOM    465  O   TRP A  28      24.318   2.716 -13.232  1.00 11.19           O  0.032
ANISOU  465  O   TRP A  28      813    990   2449   -203     -6    440       O
ATOM    466  CB  TRP A  28      24.086   5.644 -11.714  1.00 12.55           C  0.034
ANISOU  466  CB  TRP A  28      848   1126   2795    -62    432    -99       C
ATOM    467  CG  TRP A  28      24.381   6.296 -10.408  1.00 12.11           C  0.034
ANISOU  467  CG  TRP A  28     1073   1126   2402     31    550   -105       C
ATOM    468  CD1 TRP A  28      24.883   7.550 -10.232  1.00 11.74           C  0.033
ANISOU  468  CD1 TRP A  28     1023    936   2502     26    249    -74       C
ATOM    469  CD2 TRP A  28      24.290   5.716  -9.099  1.00 11.46           C  0.033
ANISOU  469  CD2 TRP A  28     1210   1047   2099     82    371    135       C
ATOM    470  NE1 TRP A  28      25.073   7.809  -8.888  1.00 11.86           N  0.033
ANISOU  470  NE1 TRP A  28     1150    853   2504   -128    192    185       N
ATOM    471  CE2 TRP A  28      24.713   6.699  -8.173  1.00 10.19           C  0.031
ANISOU  471  CE2 TRP A  28     1051    697   2124     73    103    446       C
ATOM    472  CE3 TRP A  28      23.876   4.465  -8.616  1.00 13.20           C  0.035
ANISOU  472  CE3 TRP A  28     1354   1159   2503    107    477    235       C
ATOM    473  CZ2 TRP A  28      24.720   6.477  -6.796  1.00 10.88           C  0.032
ANISOU  473  CZ2 TRP A  28     1304    749   2081     95     54    515       C
ATOM    474  CZ3 TRP A  28      23.898   4.231  -7.260  1.00 14.47           C  0.037
ANISOU  474  CZ3 TRP A  28     1529   1136   2833    109    232    283       C
ATOM    475  CH2 TRP A  28      24.333   5.229  -6.354  1.00 13.41           C  0.035
ANISOU  475  CH2 TRP A  28     1550    902   2646    168     95    291       C
ATOM    476  H   TRP A  28      26.303   6.265 -12.441  1.00 15.51           H  0.038
```

```
ATOM    477  HA  TRP A  28      25.325   4.043 -11.390  1.00 14.62           H  0.037
ATOM    478  HB2 TRP A  28      24.011   6.337 -12.388  1.00 15.21           H  0.038
ATOM    479  HB3 TRP A  28      23.246   5.166 -11.631  1.00 15.21           H  0.038
ATOM    480  HD1 TRP A  28      25.048   8.156 -10.918  1.00 14.24           H  0.037
ATOM    481  HE1 TRP A  28      25.360   8.548  -8.556  1.00 14.38           H  0.037
ATOM    482  HE3 TRP A  28      23.593   3.803  -9.204  1.00 15.99           H  0.039
ATOM    483  HZ2 TRP A  28      25.016   7.129  -6.203  1.00 13.21           H  0.035
ATOM    484  HZ3 TRP A  28      23.618   3.407  -6.933  1.00 17.52           H  0.040
ATOM    485  HH2 TRP A  28      24.323   5.052  -5.441  1.00 16.25           H  0.039
ATOM    486  N   VAL A  29      24.944   4.429 -14.534  1.00 11.74           N  0.033
ANISOU  486  N   VAL A  29      839   1316   2308   -172    -11    664       N
ATOM    487  CA  VAL A  29      24.653   3.711 -15.775  1.00 12.32           C  0.034
ANISOU  487  CA  VAL A  29      988   1282   2413   -242   -246    360       C
ATOM    488  C   VAL A  29      25.586   2.514 -15.946  1.00 11.96           C  0.033
ANISOU  488  C   VAL A  29     1035   1139   2370    -46   -458    523       C
ATOM    489  O   VAL A  29      25.129   1.397 -16.233  1.00 12.62           O  0.034
ANISOU  489  O   VAL A  29     1281   1198   2317    -40   -449    359       O
ATOM    490  CB  VAL A  29      24.707   4.663 -16.986  1.00 13.43           C  0.035
ANISOU  490  CB  VAL A  29     1254   1326   2522   -323     51    278       C
ATOM    491  CG1 VAL A  29      24.530   3.881 -18.266  1.00 14.78           C  0.037
ANISOU  491  CG1 VAL A  29     1485   1433   2699   -413    348    259       C
ATOM    492  CG2 VAL A  29      23.631   5.692 -16.884  1.00 13.65           C  0.036
ANISOU  492  CG2 VAL A  29     1393   1476   2317   -333    -45    496       C
ATOM    493  H   VAL A  29      25.225   5.233 -14.651  1.00 14.24           H  0.037
ATOM    494  HA  VAL A  29      23.748   3.365 -15.721  1.00 14.94           H  0.037
ATOM    495  HB  VAL A  29      25.566   5.112 -17.012  1.00 16.26           H  0.039
ATOM    496 HG11 VAL A  29      24.330   4.499 -18.986  1.00 17.89           H  0.041
ATOM    497 HG12 VAL A  29      25.349   3.401 -18.463  1.00 17.89           H  0.041
ATOM    498 HG13 VAL A  29      23.796   3.256 -18.155  1.00 17.89           H  0.041
ATOM    499 HG21 VAL A  29      23.682   6.279 -17.654  1.00 16.53           H  0.039
ATOM    500 HG22 VAL A  29      22.770   5.246 -16.863  1.00 16.53           H  0.039
ATOM    501 HG23 VAL A  29      23.759   6.203 -16.070  1.00 16.53           H  0.039
ATOM    502  N   CYS A  30      26.904   2.736 -15.787  1.00 12.05           N  0.034
ANISOU  502  N   CYS A  30     1006   1185   2386   -175   -286    438       N
ATOM    503  CA  CYS A  30      27.883   1.660 -15.837  1.00 10.86           C  0.032
ANISOU  503  CA  CYS A  30     1061    913   2152   -280    105    461       C
ATOM    504  C   CYS A  30      27.502   0.554 -14.858  1.00 10.86           C  0.032
ANISOU  504  C   CYS A  30     1106    905   2116   -150    183    509       C
ATOM    505  O   CYS A  30      27.517  -0.630 -15.217  1.00 11.39           O  0.033
ANISOU  505  O   CYS A  30     1156    912   2259     16    105    299       O
ATOM    506  CB  CYS A  30      29.300   2.233 -15.593  1.00 12.41           C  0.034
ANISOU  506  CB  CYS A  30     1118    982   2617   -279    165    380       C
ATOM    507  SG  CYS A  30      30.557   1.046 -15.782  1.00 11.65           S  0.033
ANISOU  507  SG  CYS A  30     1061   1159   2208   -149     93    202       S
ATOM    508  H   CYS A  30      27.248   3.512 -15.648  1.00 14.61           H  0.037
ATOM    509  HA  CYS A  30      27.874   1.276 -16.728  1.00 13.18           H  0.035
ATOM    510  HB2 CYS A  30      29.463   2.959 -16.207  1.00 15.05           H  0.038
ATOM    511  HB3 CYS A  30      29.345   2.563 -14.682  1.00 15.05           H  0.038
ATOM    512  N   ALA A  31      27.156   0.925 -13.617  1.00 10.15           N  0.031
ANISOU  512  N   ALA A  31     1033    793   2030    -17     95    155       N
ATOM    513  CA  ALA A  31      26.846  -0.106 -12.636  1.00  9.62           C  0.030
ANISOU  513  CA  ALA A  31      941    765   1951   -100   -149    205       C
ATOM    514  C   ALA A  31      25.652  -0.920 -13.095  1.00  9.22           C  0.029
ANISOU  514  C   ALA A  31      971    764   1769   -141    -97     83       C
ATOM    515  O   ALA A  31      25.641  -2.143 -12.962  1.00 10.26           O  0.031
ANISOU  515  O   ALA A  31     1061    700   2136   -254   -318     14       O
ATOM    516  CB  ALA A  31      26.543   0.490 -11.260  1.00 11.59           C  0.033
ANISOU  516  CB  ALA A  31     1047    704   2652    -71    -64    214       C
ATOM    517  H   ALA A  31      27.097   1.735 -13.333  1.00 12.33           H  0.034
ATOM    518  HA  ALA A  31      27.606  -0.703 -12.548  1.00 11.70           H  0.033
ATOM    519  HB1 ALA A  31      26.343  -0.230 -10.642  1.00 14.06           H  0.036
ATOM    520  HB2 ALA A  31      27.319   0.985 -10.953  1.00 14.06           H  0.036
ATOM    521  HB3 ALA A  31      25.780   1.084 -11.335  1.00 14.06           H  0.036
ATOM    522  N   ALA A  32      24.617  -0.249 -13.609  1.00 10.08           N  0.031
ANISOU  522  N   ALA A  32      961    776   2091    -78    -80    282       N
ATOM    523  CA  ALA A  32      23.424  -0.981 -14.049  1.00  9.81           C  0.030
ANISOU  523  CA  ALA A  32      989    801   1938   -132   -136    289       C
ATOM    524  C   ALA A  32      23.739  -1.873 -15.238  1.00  9.41           C  0.030
ANISOU  524  C   ALA A  32      829    734   2013   -160   -192     81       C
ATOM    525  O   ALA A  32      23.190  -2.985 -15.345  1.00 11.97           O  0.033
ANISOU  525  O   ALA A  32      929    865   2754   -181   -307     82       O
ATOM    526  CB  ALA A  32      22.275  -0.016 -14.373  1.00  9.43           C  0.030
ANISOU  526  CB  ALA A  32      944    849   1789   -171    -42    326       C
ATOM    527  H   ALA A  32      24.579   0.604 -13.712  1.00 12.24           H  0.034
ATOM    528  HA  ALA A  32      23.129  -1.554 -13.324  1.00 11.92           H  0.033
ATOM    529  HB1 ALA A  32      21.508  -0.529 -14.671  1.00 11.46           H  0.033
ATOM    530  HB2 ALA A  32      22.049   0.485 -13.574  1.00 11.46           H  0.033
ATOM    531  HB3 ALA A  32      22.561   0.590 -15.075  1.00 11.46           H  0.033
```

```
ATOM    532  N   LYS A  33      24.617  -1.397 -16.141  1.00 10.03           N  0.031
ANISOU  532  N   LYS A  33      876    981   1955   -169    -19     92         N
ATOM    533  CA  LYS A  33      25.016  -2.201 -17.283  1.00 11.86           C  0.033
ANISOU  533  CA  LYS A  33     1240   1130   2134    -79    -41     44         C
ATOM    534  C   LYS A  33      25.587  -3.519 -16.810  1.00 11.26           C  0.032
ANISOU  534  C   LYS A  33     1307    910   2060   -216    207     73         C
ATOM    535  O   LYS A  33      25.167  -4.595 -17.262  1.00 12.59           O  0.034
ANISOU  535  O   LYS A  33     1380    854   2549   -240   -302    266         O
ATOM    536  CB  LYS A  33      26.072  -1.459 -18.108  1.00 13.57           C  0.036
ANISOU  536  CB  LYS A  33     1578   1469   2109     26    -95    249         C
ATOM    537  CG  LYS A  33      26.704  -2.334 -19.158  1.00 16.12           C  0.039
ANISOU  537  CG  LYS A  33     1732   1838   2556    -93    197    334         C
ATOM    538  CD  LYS A  33      25.750  -2.698 -20.264  1.00 17.38           C  0.040
ANISOU  538  CD  LYS A  33     1882   2070   2650   -215    304    155         C
ATOM    539  CE  LYS A  33      26.506  -3.548 -21.293  1.00 19.96           C  0.043
ANISOU  539  CE  LYS A  33     2087   2346   3150   -218    381   -265         C
ATOM    540  NZ  LYS A  33      25.586  -4.121 -22.304  1.00 22.22           N  0.046
ANISOU  540  NZ  LYS A  33     2333   2515   3596   -261    437   -214         N
ATOM    541  H   LYS A  33      24.985  -0.620 -16.106  1.00 12.19           H  0.034
ATOM    542  HA  LYS A  33      24.246  -2.377 -17.846  1.00 14.38           H  0.037
ATOM    543  HB2 LYS A  33      25.653  -0.707 -18.555  1.00 16.43           H  0.039
ATOM    544  HB3 LYS A  33      26.774  -1.145 -17.517  1.00 16.43           H  0.039
ATOM    545  HG2 LYS A  33      27.439  -1.853 -19.556  1.00 19.50           H  0.043
ATOM    546  HG3 LYS A  33      27.042  -3.153 -18.768  1.00 19.50           H  0.043
ATOM    547  HD2 LYS A  33      25.014  -3.219 -19.907  1.00 21.00           H  0.044
ATOM    548  HD3 LYS A  33      25.426  -1.894 -20.699  1.00 21.00           H  0.044
ATOM    549  HE2 LYS A  33      27.155  -2.992 -21.753  1.00 24.10           H  0.047
ATOM    550  HE3 LYS A  33      26.952  -4.280 -20.838  1.00 24.10           H  0.047
ATOM    551  HZ1 LYS A  33      26.044  -4.609 -22.890  1.00 26.82           H  0.050
ATOM    552  HZ2 LYS A  33      24.981  -4.639 -21.906  1.00 26.82           H  0.050
ATOM    553  HZ3 LYS A  33      25.166  -3.467 -22.738  1.00 26.82           H  0.050
ATOM    554  N   PHE A  34      26.470  -3.466 -15.836  1.00 11.24           N  0.032
ANISOU  554  N   PHE A  34     1457    843   1968    -90     63    345         N
ATOM    555  CA  PHE A  34      27.152  -4.696 -15.478  1.00  9.63           C  0.030
ANISOU  555  CA  PHE A  34     1355    810   1493    106   -368    229         C
ATOM    556  C   PHE A  34      26.464  -5.487 -14.386  1.00 12.76           C  0.035
ANISOU  556  C   PHE A  34     1392   1075   2381    215    165    335         C
ATOM    557  O   PHE A  34      26.766  -6.678 -14.209  1.00 14.46           O  0.037
ANISOU  557  O   PHE A  34     1320   1215   2961    445    529    462         O
ATOM    558  CB  PHE A  34      28.621  -4.419 -15.126  1.00 11.32           C  0.033
ANISOU  558  CB  PHE A  34     1384    937   1982    230   -312    128         C
ATOM    559  CG  PHE A  34      29.416  -3.950 -16.297  1.00 12.11           C  0.034
ANISOU  559  CG  PHE A  34     1297    948   2357    291    104    -35         C
ATOM    560  CD1 PHE A  34      29.407  -4.638 -17.517  1.00 12.73           C  0.035
ANISOU  560  CD1 PHE A  34     1368   1121   2348    278    379   -293         C
ATOM    561  CD2 PHE A  34      30.133  -2.789 -16.215  1.00 14.07           C  0.036
ANISOU  561  CD2 PHE A  34     1374   1058   2914    296    552    -17         C
ATOM    562  CE1 PHE A  34      30.151  -4.163 -18.598  1.00 12.22           C  0.034
ANISOU  562  CE1 PHE A  34     1416    939   2286    298    445   -284         C
ATOM    563  CE2 PHE A  34      30.893  -2.314 -17.305  1.00 14.05           C  0.036
ANISOU  563  CE2 PHE A  34     1438   1003   2896    317    651     25         C
ATOM    564  CZ  PHE A  34      30.891  -3.012 -18.498  1.00 12.77           C  0.035
ANISOU  564  CZ  PHE A  34     1346    931   2577    198    573    -10         C
ATOM    565  H   PHE A  34      26.688  -2.768 -15.383  1.00 13.63           H  0.036
ATOM    566  HA  PHE A  34      27.157  -5.270 -16.259  1.00 11.70           H  0.033
ATOM    567  HB2 PHE A  34      28.658  -3.731 -14.443  1.00 13.74           H  0.036
ATOM    568  HB3 PHE A  34      29.026  -5.236 -14.796  1.00 13.74           H  0.036
ATOM    569  HD1 PHE A  34      28.919  -5.425 -17.601  1.00 15.43           H  0.038
ATOM    570  HD2 PHE A  34      30.146  -2.319 -15.413  1.00 17.03           H  0.040
ATOM    571  HE1 PHE A  34      30.148  -4.636 -19.398  1.00 14.81           H  0.037
ATOM    572  HE2 PHE A  34      31.384  -1.528 -17.225  1.00 17.01           H  0.040
ATOM    573  HZ  PHE A  34      31.380  -2.702 -19.226  1.00 15.48           H  0.038
ATOM    574  N   GLU A  35      25.601  -4.859 -13.612  1.00 12.72           N  0.035
ANISOU  574  N   GLU A  35     1284   1103   2447    102     67    327         N
ATOM    575  CA  GLU A  35      24.810  -5.648 -12.678  1.00 11.96           C  0.033
ANISOU  575  CA  GLU A  35     1433    964   2148    113   -280    356         C
ATOM    576  C   GLU A  35      23.687  -6.417 -13.389  1.00 13.35           C  0.035
ANISOU  576  C   GLU A  35     1785   1009   2280    125   -498    276         C
ATOM    577  O   GLU A  35      23.415  -7.579 -13.054  1.00 12.69           O  0.034
ANISOU  577  O   GLU A  35     1878    934   2011    166   -216    243         O
ATOM    578  CB  GLU A  35      24.209  -4.724 -11.615  1.00 11.95           C  0.033
ANISOU  578  CB  GLU A  35     1251   1020   2270    211   -165    599         C
ATOM    579  CG  GLU A  35      25.210  -4.135 -10.613  1.00 12.83           C  0.035
ANISOU  579  CG  GLU A  35     1315   1250   2310    106   -334    408         C
ATOM    580  CD  GLU A  35      25.901  -5.177  -9.725  1.00 13.82           C  0.036
ANISOU  580  CD  GLU A  35     1297   1344   2608   -150   -123    276         C
ATOM    581  OE1 GLU A  35      25.479  -6.348  -9.747  1.00 13.62           O  0.036
ANISOU  581  OE1 GLU A  35     1459   1269   2447   -338   -281    558         O
```

```
ATOM    582  OE2 GLU A  35      26.888  -4.825  -9.045  1.00 13.63           O  0.036
ANISOU  582  OE2 GLU A  35     1339   1506   2332   -130   -179   -116       O
ATOM    583  H   GLU A  35      25.454  -4.012 -13.602  1.00 15.42           H  0.038
ATOM    584  HA  GLU A  35      25.384  -6.291 -12.233  1.00 14.50           H  0.037
ATOM    585  HB2 GLU A  35      23.775  -3.981 -12.064  1.00 14.49           H  0.037
ATOM    586  HB3 GLU A  35      23.551  -5.226 -11.109  1.00 14.49           H  0.037
ATOM    587  HG2 GLU A  35      25.901  -3.664 -11.103  1.00 15.55           H  0.038
ATOM    588  HG3 GLU A  35      24.741  -3.516 -10.032  1.00 15.55           H  0.038
ATOM    589  N   SER A  36      22.954  -5.774 -14.320  1.00 13.10           N  0.035
ANISOU  589  N   SER A  36     1758   1027   2192    -36   -298    363       N
ATOM    590  CA  SER A  36      21.705  -6.339 -14.822  1.00 14.23           C  0.036
ANISOU  590  CA  SER A  36     1737   1150   2519   -159   -518    373       C
ATOM    591  C   SER A  36      21.602  -6.337 -16.338  1.00 14.88           C  0.037
ANISOU  591  C   SER A  36     1726   1189   2739   -346   -352    255       C
ATOM    592  O   SER A  36      20.581  -6.817 -16.864  1.00 14.57           O  0.037
ANISOU  592  O   SER A  36     1733   1173   2631   -524   -432     79       O
ATOM    593  CB  SER A  36      20.512  -5.531 -14.297  1.00 13.20           C  0.035
ANISOU  593  CB  SER A  36     1660   1070   2286   -250   -235    169       C
ATOM    594  OG  SER A  36      20.507  -4.243 -14.887  1.00 12.95           O  0.035
ANISOU  594  OG  SER A  36     1344   1119   2457   -135   -282     73       O
ATOM    595  H   SER A  36      23.164  -5.016 -14.668  1.00 15.87           H  0.039
ATOM    596  HA  SER A  36      21.612  -7.254 -14.514  1.00 17.22           H  0.040
ATOM    597  HB2 SER A  36      19.689  -5.991 -14.527  1.00 15.99           H  0.039
ATOM    598  HB3 SER A  36      20.587  -5.441 -13.334  1.00 15.99           H  0.039
ATOM    599  HG  SER A  36      21.217  -3.837 -14.697  1.00 15.69           H  0.038
ATOM    600  N   ASN A  37      22.610  -5.788 -17.042  1.00 14.78           N  0.037
ANISOU  600  N   ASN A  37     1805   1305   2506   -433   -303    250       N
ATOM    601  CA  ASN A  37      22.523  -5.573 -18.471  1.00 15.31           C  0.038
ANISOU  601  CA  ASN A  37     1829   1462   2524   -530   -204    311       C
ATOM    602  C   ASN A  37      21.285  -4.755 -18.823  1.00 14.88           C  0.037
ANISOU  602  C   ASN A  37     1817   1564   2274   -456   -169    341       C
ATOM    603  O   ASN A  37      20.683  -4.944 -19.877  1.00 15.65           O  0.038
ANISOU  603  O   ASN A  37     2001   1693   2253   -339   -200    419       O
ATOM    604  CB  ASN A  37      22.680  -6.848 -19.318  1.00 17.89           C  0.041
ANISOU  604  CB  ASN A  37     2101   1673   3023   -558   -107    259       C
ATOM    605  CG  ASN A  37      23.043  -6.529 -20.778  1.00 19.94           C  0.043
ANISOU  605  CG  ASN A  37     2380   1893   3304   -647   -185    255       C
ATOM    606  OD1 ASN A  37      23.537  -5.461 -21.064  1.00 19.94           O  0.043
ANISOU  606  OD1 ASN A  37     2502   2025   3048   -793    -95    560       O
ATOM    607  ND2 ASN A  37      22.808  -7.469 -21.686  1.00 21.74           N  0.045
ANISOU  607  ND2 ASN A  37     2512   2165   3584   -580   -278    240       N
ATOM    608  H   ASN A  37      23.356  -5.534 -16.699  1.00 17.89           H  0.041
ATOM    609  HA  ASN A  37      23.277  -5.012 -18.709  1.00 18.52           H  0.042
ATOM    610  HB2 ASN A  37      23.388  -7.394 -18.943  1.00 21.62           H  0.045
ATOM    611  HB3 ASN A  37      21.843  -7.338 -19.315  1.00 21.62           H  0.045
ATOM    612 HD21 ASN A  37      23.000  -7.326 -22.512  1.00 26.24           H  0.050
ATOM    613 HD22 ASN A  37      22.458  -8.218 -21.449  1.00 26.24           H  0.050
ATOM    614  N   PHE A  38      20.936  -3.816 -17.931  1.00 13.74           N  0.036
ANISOU  614  N   PHE A  38     1678   1483   2062   -494    -61    189       N
ATOM    615  CA  PHE A  38      19.841  -2.860 -18.050  1.00 13.29           C  0.035
ANISOU  615  CA  PHE A  38     1554   1324   2170   -588    280    312       C
ATOM    616  C   PHE A  38      18.469  -3.494 -17.944  1.00 14.84           C  0.037
ANISOU  616  C   PHE A  38     1522   1489   2627   -663   -112    401       C
ATOM    617  O   PHE A  38      17.478  -2.853 -18.297  1.00 15.09           O  0.038
ANISOU  617  O   PHE A  38     1557   1644   2531   -754   -441    289       O
ATOM    618  CB  PHE A  38      19.896  -2.078 -19.356  1.00 12.33           C  0.034
ANISOU  618  CB  PHE A  38     1464   1183   2037   -446    276    576       C
ATOM    619  CG  PHE A  38      21.121  -1.211 -19.512  1.00 13.68           C  0.036
ANISOU  619  CG  PHE A  38     1620   1174   2403   -274     71    460       C
ATOM    620  CD1 PHE A  38      21.710  -0.575 -18.424  1.00 13.35           C  0.035
ANISOU  620  CD1 PHE A  38     1790   1332   1952   -146    179    273       C
ATOM    621  CD2 PHE A  38      21.657  -1.029 -20.765  1.00 14.97           C  0.037
ANISOU  621  CD2 PHE A  38     1671   1226   2789   -515     26    332       C
ATOM    622  CE1 PHE A  38      22.818   0.269 -18.611  1.00 13.57           C  0.036
ANISOU  622  CE1 PHE A  38     1879   1369   1909    -86   -352    263       C
ATOM    623  CE2 PHE A  38      22.741  -0.198 -20.963  1.00 13.78           C  0.036
ANISOU  623  CE2 PHE A  38     1732   1296   2206   -537   -287    435       C
ATOM    624  CZ  PHE A  38      23.348   0.431 -19.872  1.00 12.93           C  0.035
ANISOU  624  CZ  PHE A  38     1657   1305   1952   -389   -581    582       C
ATOM    625  H   PHE A  38      21.362  -3.714 -17.191  1.00 16.64           H  0.039
ATOM    626  HA  PHE A  38      19.912  -2.224 -17.321  1.00 16.09           H  0.039
ATOM    627  HB2 PHE A  38      19.879  -2.702 -20.098  1.00 14.94           H  0.037
ATOM    628  HB3 PHE A  38      19.122  -1.496 -19.402  1.00 14.94           H  0.037
ATOM    629  HD1 PHE A  38      21.350  -0.683 -17.573  1.00 16.18           H  0.039
ATOM    630  HD2 PHE A  38      21.258  -1.441 -21.497  1.00 18.11           H  0.041
ATOM    631  HE1 PHE A  38      23.216   0.686 -17.881  1.00 16.44           H  0.039
ATOM    632  HE2 PHE A  38      23.097  -0.094 -21.816  1.00 16.68           H  0.040
ATOM    633  HZ  PHE A  38      24.088   0.979 -20.001  1.00 15.67           H  0.038
```

```
ATOM    634  N   ASN A  39      18.387  -4.735 -17.467  1.00 13.97           N  0.036
ANISOU  634  N   ASN A  39     1506   1462   2338   -693   -172    527       N
ATOM    635  CA  ASN A  39      17.125  -5.471 -17.376  1.00 13.35           C  0.035
ANISOU  635  CA  ASN A  39     1383   1505   2186   -619     -5    535       C
ATOM    636  C   ASN A  39      16.546  -5.282 -15.973  1.00 13.49           C  0.036
ANISOU  636  C   ASN A  39     1322   1389   2413   -555     61    418       C
ATOM    637  O   ASN A  39      17.095  -5.806 -14.994  1.00 12.83           O  0.035
ANISOU  637  O   ASN A  39     1248   1310   2316   -300     21     -1       O
ATOM    638  CB  ASN A  39      17.367  -6.944 -17.691  1.00 13.25           C  0.035
ANISOU  638  CB  ASN A  39     1388   1490   2157   -618    252    374       C
ATOM    639  CG  ASN A  39      16.056  -7.744 -17.794  1.00 13.92           C  0.036
ANISOU  639  CG  ASN A  39     1387   1343   2561   -509    185    180       C
ATOM    640  OD1 ASN A  39      14.982  -7.271 -17.399  1.00 13.65           O  0.036
ANISOU  640  OD1 ASN A  39     1343   1149   2693   -379    -38    223       O
ATOM    641  ND2 ASN A  39      16.134  -8.932 -18.349  1.00 15.75           N  0.038
ANISOU  641  ND2 ASN A  39     1519   1429   3036   -545     60     61       N
ATOM    642  H   ASN A  39      19.064  -5.183 -17.183  1.00 16.91           H  0.040
ATOM    643  HA  ASN A  39      16.494  -5.117 -18.022  1.00 16.17           H  0.039
ATOM    644  HB2 ASN A  39      17.831  -7.015 -18.540  1.00 16.05           H  0.039
ATOM    645  HB3 ASN A  39      17.904  -7.335 -16.984  1.00 16.05           H  0.039
ATOM    646 HD21 ASN A  39      15.427  -9.415 -18.431  1.00 19.05           H  0.042
ATOM    647 HD22 ASN A  39      16.894  -9.231 -18.619  1.00 19.05           H  0.042
ATOM    648  N   THR A  40      15.395  -4.583 -15.864  1.00 13.49           N  0.036
ANISOU  648  N   THR A  40     1310   1215   2601   -473    -15    493       N
ATOM    649  CA  THR A  40      14.852  -4.384 -14.533  1.00 13.87           C  0.036
ANISOU  649  CA  THR A  40     1292   1192   2784   -503   -170    348       C
ATOM    650  C   THR A  40      14.406  -5.700 -13.886  1.00 13.01           C  0.035
ANISOU  650  C   THR A  40     1237   1172   2532   -504   -190    346       C
ATOM    651  O   THR A  40      14.261  -5.758 -12.654  1.00 14.31           O  0.037
ANISOU  651  O   THR A  40     1371   1487   2580   -733   -127    303       O
ATOM    652  CB  THR A  40      13.652  -3.451 -14.585  1.00 16.27           C  0.039
ANISOU  652  CB  THR A  40     1471   1390   3319   -599    -22      0       C
ATOM    653  OG1 THR A  40      12.557  -4.130 -15.234  1.00 18.10           O  0.041
ANISOU  653  OG1 THR A  40     1630   1439   3807   -562   -169    226       O
ATOM    654  CG2 THR A  40      14.006  -2.137 -15.286  1.00 16.33           C  0.039
ANISOU  654  CG2 THR A  40     1557   1369   3278   -628    133     51       C
ATOM    655  H   THR A  40      14.944  -4.241 -16.512  1.00 16.34           H  0.039
ATOM    656  HA  THR A  40      15.529  -3.980 -13.968  1.00 16.79           H  0.040
ATOM    657  HB  THR A  40      13.388  -3.236 -13.677  1.00 19.67           H  0.043
ATOM    658  HG1 THR A  40      12.232  -4.711 -14.722  1.00 21.87           H  0.045
ATOM    659 HG21 THR A  40      13.269  -1.511 -15.206  1.00 19.74           H  0.043
ATOM    660 HG22 THR A  40      14.796  -1.748 -14.879  1.00 19.74           H  0.043
ATOM    661 HG23 THR A  40      14.183  -2.296 -16.226  1.00 19.74           H  0.043
ATOM    662  N   GLN A  41      14.163  -6.739 -14.681  1.00 12.81           N  0.035
ANISOU  662  N   GLN A  41     1234   1084   2550   -472   -336    162       N
ATOM    663  CA  GLN A  41      13.663  -8.004 -14.150  1.00 13.59           C  0.036
ANISOU  663  CA  GLN A  41     1380   1197   2589   -597   -268    234       C
ATOM    664  C   GLN A  41      14.765  -8.977 -13.772  1.00 14.39           C  0.037
ANISOU  664  C   GLN A  41     1596   1391   2482   -717   -428    456       C
ATOM    665  O   GLN A  41      14.471 -10.115 -13.379  1.00 17.79           O  0.041
ANISOU  665  O   GLN A  41     1926   1611   3222   -764   -581    628       O
ATOM    666  CB  GLN A  41      12.684  -8.668 -15.128  1.00 13.30           C  0.035
ANISOU  666  CB  GLN A  41     1436   1135   2481   -571   -315    378       C
ATOM    667  CG  GLN A  41      11.476  -7.803 -15.398  1.00 14.28           C  0.037
ANISOU  667  CG  GLN A  41     1421   1304   2699   -457   -446    573       C
ATOM    668  CD  GLN A  41      10.402  -8.584 -16.115  1.00 15.42           C  0.038
ANISOU  668  CD  GLN A  41     1524   1469   2865   -263   -810    485       C
ATOM    669  OE1 GLN A  41      10.265  -8.517 -17.345  1.00 16.51           O  0.039
ANISOU  669  OE1 GLN A  41     1749   1396   3128     79   -591    322       O
ATOM    670  NE2 GLN A  41       9.667  -9.357 -15.362  1.00 15.89           N  0.039
ANISOU  670  NE2 GLN A  41     1455   1651   2933   -406   -574    751       N
ATOM    671  H   GLN A  41      14.279  -6.738 -15.533  1.00 15.52           H  0.038
ATOM    672  HA  GLN A  41      13.165  -7.813 -13.340  1.00 16.46           H  0.039
ATOM    673  HB2 GLN A  41      13.136  -8.827 -15.971  1.00 16.11           H  0.039
ATOM    674  HB3 GLN A  41      12.376  -9.507 -14.750  1.00 16.11           H  0.039
ATOM    675  HG2 GLN A  41      11.113  -7.486 -14.556  1.00 17.28           H  0.040
ATOM    676  HG3 GLN A  41      11.735  -7.054 -15.958  1.00 17.28           H  0.040
ATOM    677 HE21 GLN A  41       9.800  -9.384 -14.513  1.00 19.22           H  0.042
ATOM    678 HE22 GLN A  41       9.045  -9.830 -15.719  1.00 19.22           H  0.042
ATOM    679  N   ALA A  42      16.016  -8.562 -13.870  1.00 14.32           N  0.037
ANISOU  679  N   ALA A  42     1610   1401   2431   -546   -494    421       N
ATOM    680  CA  ALA A  42      17.112  -9.467 -13.552  1.00 13.92           C  0.036
ANISOU  680  CA  ALA A  42     1642   1248   2401   -279   -162    166       C
ATOM    681  C   ALA A  42      17.053  -9.936 -12.096  1.00 12.94           C  0.035
ANISOU  681  C   ALA A  42     1635   1053   2227   -147     -5   -111       C
ATOM    682  O   ALA A  42      16.831  -9.142 -11.177  1.00 11.79           O  0.033
ANISOU  682  O   ALA A  42     1396    888   2195    -53    -96    -90       O
ATOM    683  CB  ALA A  42      18.439  -8.746 -13.776  1.00 14.62           C  0.037
```

```
ANISOU  683  CB  ALA A  42     1708   1292   2554   -199   -228     94       C
ATOM    684  H   ALA A  42      16.259  -7.774 -14.114  1.00 17.34           H  0.040
ATOM    685  HA  ALA A  42      17.076 -10.243 -14.132  1.00 16.86           H  0.040
ATOM    686  HB1 ALA A  42      19.167  -9.351 -13.563  1.00 17.69           H  0.041
ATOM    687  HB2 ALA A  42      18.496  -8.472 -14.705  1.00 17.69           H  0.041
ATOM    688  HB3 ALA A  42      18.476  -7.968 -13.199  1.00 17.69           H  0.041
ATOM    689  N   THR A  43      17.291 -11.237 -11.887  1.00 12.70           N  0.034
ANISOU  689  N   THR A  43     1833   1060   1933   -139   -462   -439       N
ATOM    690  CA  THR A  43      17.443 -11.799 -10.548  1.00 14.47           C  0.037
ANISOU  690  CA  THR A  43     1840   1182   2474   -294   -506   -133       C
ATOM    691  C   THR A  43      18.625 -12.754 -10.540  1.00 14.54           C  0.037
ANISOU  691  C   THR A  43     1896   1254   2372   -159   -169   -165       C
ATOM    692  O   THR A  43      18.900 -13.432 -11.536  1.00 16.66           O  0.039
ANISOU  692  O   THR A  43     2217   1420   2692    205    -83    137       O
ATOM    693  CB  THR A  43      16.203 -12.566 -10.062  1.00 14.47           C  0.037
ANISOU  693  CB  THR A  43     1863   1232   2403   -532   -425    125       C
ATOM    694  OG1 THR A  43      15.946 -13.659 -10.958  1.00 15.37           O  0.038
ANISOU  694  OG1 THR A  43     2112   1360   2367   -350   -197    224       O
ATOM    695  CG2 THR A  43      14.967 -11.633  -9.975  1.00 13.33           C  0.035
ANISOU  695  CG2 THR A  43     1920   1190   1956   -708   -493   -142       C
ATOM    696  H   THR A  43      17.365 -11.817 -12.515  1.00 15.39           H  0.038
ATOM    697  HA  THR A  43      17.624 -11.083  -9.921  1.00 17.51           H  0.040
ATOM    698  HB  THR A  43      16.376 -12.918  -9.175  1.00 17.52           H  0.040
ATOM    699  HG1 THR A  43      15.872 -13.374 -11.744  1.00 18.59           H  0.042
ATOM    700 HG21 THR A  43      14.196 -12.133  -9.666  1.00 16.15           H  0.039
ATOM    701 HG22 THR A  43      15.142 -10.908  -9.355  1.00 16.15           H  0.039
ATOM    702 HG23 THR A  43      14.771 -11.260 -10.849  1.00 16.15           H  0.039
ATOM    703  N  AASN A  44      19.301 -12.809  -9.393  0.52 13.71           N  0.036
ANISOU  703  N  AASN A  44     1827   1357   2025   -132     52    -22       N
ATOM    704  CA AASN A  44      20.461 -13.669  -9.193  0.52 13.79           C  0.036
ANISOU  704  CA AASN A  44     1783   1463   1992    -92    128    -20       C
ATOM    705  C  AASN A  44      20.341 -14.274  -7.806  0.52 12.50           C  0.034
ANISOU  705  C  AASN A  44     1535   1402   1811   -175    170     -2       C
ATOM    706  O  AASN A  44      20.221 -13.548  -6.813  0.52 12.83           O  0.035
ANISOU  706  O  AASN A  44     1529   1354   1990    -86     62    280       O
ATOM    707  CB AASN A  44      21.766 -12.871  -9.309  0.52 16.54           C  0.039
ANISOU  707  CB AASN A  44     1871   1722   2691    -12     51    -37       C
ATOM    708  CG AASN A  44      23.003 -13.742  -9.176  0.52 18.52           C  0.042
ANISOU  708  CG AASN A  44     1941   1960   3133    -24     31    145       C
ATOM    709  OD1AASN A  44      23.676 -13.751  -8.139  0.52 20.36           O  0.044
ANISOU  709  OD1AASN A  44     2015   2165   3558     22    -64    396       O
ATOM    710  ND2AASN A  44      23.310 -14.478 -10.229  0.52 19.55           N  0.043
ANISOU  710  ND2AASN A  44     1994   1999   3435     15     97    -61       N
ATOM    711  H  AASN A  44      19.099 -12.343  -8.699  0.52 16.60           H  0.039
ATOM    712  HA AASN A  44      20.466 -14.382  -9.851  0.52 16.69           H  0.040
ATOM    713  HB2AASN A  44      21.796 -12.437 -10.176  0.52 20.00           H  0.043
ATOM    714  HB3AASN A  44      21.795 -12.207  -8.605  0.52 20.00           H  0.043
ATOM    715 HD21AASN A  44      24.001 -14.990 -10.209  0.52 23.61           H  0.047
ATOM    716 HD22AASN A  44      22.819 -14.446 -10.934  0.52 23.61           H  0.047
ATOM    717  N  BASN A  44      19.296 -12.875  -9.400  0.48 12.11           N  0.034
ANISOU  717  N  BASN A  44     1640   1230   1731   -281   -151   -209       N
ATOM    718  CA BASN A  44      20.383 -13.851  -9.323  0.48 10.56           C  0.031
ANISOU  718  CA BASN A  44     1409   1204   1399   -337   -414   -400       C
ATOM    719  C  BASN A  44      20.514 -14.394  -7.909  0.48  9.97           C  0.031
ANISOU  719  C  BASN A  44     1163   1259   1364   -434   -429   -376       C
ATOM    720  O  BASN A  44      20.772 -13.646  -6.957  0.48 11.60           O  0.033
ANISOU  720  O  BASN A  44     1081   1280   2046   -348   -187   -443       O
ATOM    721  CB BASN A  44      21.707 -13.301  -9.870  0.48 13.35           C  0.035
ANISOU  721  CB BASN A  44     1387   1327   2358   -253   -460   -570       C
ATOM    722  CG BASN A  44      21.685 -13.153 -11.399  0.48 14.82           C  0.037
ANISOU  722  CG BASN A  44     1272   1377   2983   -140   -414   -606       C
ATOM    723  OD1BASN A  44      21.827 -14.153 -12.143  0.48 15.83           O  0.038
ANISOU  723  OD1BASN A  44     1357   1415   3244   -142   -184   -601       O
ATOM    724  ND2BASN A  44      21.407 -11.915 -11.873  0.48 15.27           N  0.038
ANISOU  724  ND2BASN A  44     1141   1426   3233     58   -601   -761       N
ATOM    725  H  BASN A  44      19.152 -12.425  -8.682  0.48 14.68           H  0.037
ATOM    726  HA BASN A  44      20.145 -14.605  -9.884  0.48 12.82           H  0.035
ATOM    727  HB2BASN A  44      21.872 -12.426  -9.484  0.48 16.17           H  0.039
ATOM    728  HB3BASN A  44      22.425 -13.910  -9.636  0.48 16.17           H  0.039
ATOM    729 HD21BASN A  44      21.374 -11.777 -12.721  0.48 18.47           H  0.042
ATOM    730 HD22BASN A  44      21.300 -11.260 -11.326  0.48 18.47           H  0.042
ATOM    731  N  AARG A  45      20.375 -15.614  -7.775  0.52 14.67           N  0.037
ANISOU  731  N  AARG A  45     1485   1572   2518   -266    185    -12       N
ATOM    732  CA AARG A  45      20.384 -16.312  -6.472  0.52 16.27           C  0.039
ANISOU  732  CA AARG A  45     1493   1819   2871   -228    144     24       C
ATOM    733  C  AARG A  45      21.837 -16.530  -6.057  0.52 16.51           C  0.039
ANISOU  733  C  AARG A  45     1349   2069   2856     48    243     26       C
ATOM    734  O  AARG A  45      22.588 -17.146  -6.836  0.52 17.42           O  0.040
```

```
ANISOU  734  O  AARG A  45     1448   2177   2995     63    129   -103       O
ATOM    735  CB AARG A  45      19.575 -17.620  -6.527  0.52 17.68           C  0.041
ANISOU  735  CB AARG A  45     1781   1835   3100   -458    284    260       C
ATOM    736  CG AARG A  45      19.314 -18.235  -5.155  0.52 19.76           C  0.043
ANISOU  736  CG AARG A  45     1987   1904   3615   -582    308    151       C
ATOM    737  CD AARG A  45      17.888 -17.999  -4.699  0.52 20.85           C  0.044
ANISOU  737  CD AARG A  45     2189   1916   3817   -737    274   -163       C
ATOM    738  NE AARG A  45      17.602 -18.122  -3.274  0.52 22.22           N  0.046
ANISOU  738  NE AARG A  45     2388   1875   4179   -786     21   -241       N
ATOM    739  CZ AARG A  45      16.808 -19.048  -2.742  0.52 22.49           C  0.046
ANISOU  739  CZ AARG A  45     2475   1716   4356   -737   -120   -370       C
ATOM    740  NH1AARG A  45      16.241 -19.955  -3.520  0.52 22.28           N  0.046
ANISOU  740  NH1AARG A  45     2563   1503   4398   -632    -59   -547       N
ATOM    741  NH2AARG A  45      16.583 -19.064  -1.440  0.52 22.99           N  0.046
ANISOU  741  NH2AARG A  45     2450   1779   4507   -774   -212   -403       N
ATOM    742  H  AARG A  45      20.393 -16.128  -8.464  0.52 17.76           H  0.041
ATOM    743  HA AARG A  45      19.970 -15.740  -5.807  0.52 19.68           H  0.043
ATOM    744  HB2AARG A  45      18.717 -17.441  -6.942  0.52 21.36           H  0.045
ATOM    745  HB3AARG A  45      20.066 -18.269  -7.054  0.52 21.36           H  0.045
ATOM    746  HG2AARG A  45      19.464 -19.192  -5.200  0.52 23.86           H  0.047
ATOM    747  HG3AARG A  45      19.911 -17.833  -4.505  0.52 23.86           H  0.047
ATOM    748  HD2AARG A  45      17.668 -17.087  -4.939  0.52 25.17           H  0.049
ATOM    749  HD3AARG A  45      17.313 -18.592  -5.199  0.52 25.17           H  0.049
ATOM    750  HE AARG A  45      17.972 -17.557  -2.741  0.52 26.81           H  0.050
ATOM    751 HH11AARG A  45      16.374 -19.962  -4.366  0.52 26.88           H  0.050
ATOM    752 HH12AARG A  45      15.730 -20.551  -3.169  0.52 26.88           H  0.050
ATOM    753 HH21AARG A  45      16.951 -18.475  -0.933  0.52 27.74           H  0.051
ATOM    754 HH22AARG A  45      16.070 -19.665  -1.100  0.52 27.74           H  0.051
ATOM    755  N  BARG A  45      20.324 -15.704  -7.774  0.48 12.25           N  0.034
ANISOU  755  N  BARG A  45     1153   1358   2144   -380   -273   -221       N
ATOM    756  CA BARG A  45      20.388 -16.336  -6.472  0.48 14.10           C  0.036
ANISOU  756  CA BARG A  45     1224   1663   2472   -120   -168     42       C
ATOM    757  C  BARG A  45      21.847 -16.525  -6.075  0.48 15.25           C  0.038
ANISOU  757  C  BARG A  45     1194   2011   2590    121     47     34       C
ATOM    758  O  BARG A  45      22.657 -17.038  -6.858  0.48 15.82           O  0.038
ANISOU  758  O  BARG A  45     1258   2154   2598    163   -142    -77       O
ATOM    759  CB BARG A  45      19.654 -17.675  -6.553  0.48 14.75           C  0.037
ANISOU  759  CB BARG A  45     1503   1637   2463    -77    -26    532       C
ATOM    760  CG BARG A  45      19.627 -18.545  -5.298  0.48 15.91           C  0.039
ANISOU  760  CG BARG A  45     1684   1644   2718     59     14    692       C
ATOM    761  CD BARG A  45      18.862 -17.897  -4.155  0.48 15.65           C  0.038
ANISOU  761  CD BARG A  45     1751   1650   2545    271    101    670       C
ATOM    762  NE BARG A  45      17.570 -17.320  -4.543  0.48 16.39           N  0.039
ANISOU  762  NE BARG A  45     1817   1629   2781    209    353    784       N
ATOM    763  CZ BARG A  45      16.527 -18.013  -4.978  0.48 16.63           C  0.039
ANISOU  763  CZ BARG A  45     1926   1686   2706    172    207    520       C
ATOM    764  NH1BARG A  45      16.568 -19.333  -5.084  0.48 18.15           N  0.041
ANISOU  764  NH1BARG A  45     2059   1731   3106    143    -16    510       N
ATOM    765  NH2BARG A  45      15.405 -17.363  -5.284  0.48 16.04           N  0.039
ANISOU  765  NH2BARG A  45     1875   1686   2536    101    164    423       N
ATOM    766  H  BARG A  45      20.157 -16.244  -8.423  0.48 14.85           H  0.037
ATOM    767  HA BARG A  45      19.951 -15.777  -5.811  0.48 17.08           H  0.040
ATOM    768  HB2BARG A  45      18.733 -17.499  -6.801  0.48 17.85           H  0.041
ATOM    769  HB3BARG A  45      20.073 -18.202  -7.251  0.48 17.85           H  0.041
ATOM    770  HG2BARG A  45      19.201 -19.390  -5.508  0.48 19.24           H  0.042
ATOM    771  HG3BARG A  45      20.537 -18.699  -5.000  0.48 19.24           H  0.042
ATOM    772  HD2BARG A  45      18.693 -18.568  -3.475  0.48 18.93           H  0.042
ATOM    773  HD3BARG A  45      19.404 -17.185  -3.784  0.48 18.93           H  0.042
ATOM    774  HE BARG A  45      17.516 -16.463  -4.578  0.48 19.82           H  0.043
ATOM    775 HH11BARG A  45      17.285 -19.761  -4.884  0.48 21.93           H  0.045
ATOM    776 HH12BARG A  45      15.877 -19.762  -5.364  0.48 21.93           H  0.045
ATOM    777 HH21BARG A  45      15.372 -16.507  -5.214  0.48 19.40           H  0.043
ATOM    778 HH22BARG A  45      14.719 -17.799  -5.564  0.48 19.40           H  0.043
ATOM    779  N   ASN A  46      22.177 -16.091  -4.865  1.00 16.22           N  0.039
ANISOU  779  N   ASN A  46     1216   2192   2755    252    311     39       N
ATOM    780  CA  ASN A  46      23.518 -16.208  -4.316  1.00 18.37           C  0.041
ANISOU  780  CA  ASN A  46     1390   2626   2966    246    298    516       C
ATOM    781  C   ASN A  46      23.660 -17.515  -3.555  1.00 20.82           C  0.044
ANISOU  781  C   ASN A  46     1832   2839   3239    503    178    852       C
ATOM    782  O   ASN A  46      22.675 -18.099  -3.100  1.00 22.54           O  0.046
ANISOU  782  O   ASN A  46     1966   2719   3878    744    177    864       O
ATOM    783  CB  ASN A  46      23.707 -15.026  -3.375  1.00 19.19           C  0.042
ANISOU  783  CB  ASN A  46     1209   2868   3214    -17    207    357       C
ATOM    784  CG  ASN A  46      23.551 -13.704  -4.092  1.00 21.60           C  0.045
ANISOU  784  CG  ASN A  46     1388   3148   3670   -198    383    389       C
ATOM    785  OD1 ASN A  46      22.904 -12.778  -3.597  1.00 24.28           O  0.048
ANISOU  785  OD1 ASN A  46     1587   3341   4299   -317     85    527       O
ATOM    786  ND2 ASN A  46      24.211 -13.583  -5.237  1.00 22.56           N  0.046
```

```
ANISOU  786  ND2 ASN A  46     1450   3275   3848   -369    553    614       N
ATOM    787  HA  ASN A  46      24.181 -16.174  -5.023  1.00 22.20           H  0.046
ATOM    788  HB2 ASN A  46      23.041 -15.069  -2.671  1.00 23.18           H  0.047
ATOM    789  HB3 ASN A  46      24.599 -15.061  -2.995  1.00 23.18           H  0.047
ATOM    790 HD21 ASN A  46      24.163 -12.850  -5.684  1.00 27.23           H  0.050
ATOM    791 HD22 ASN A  46      24.664 -14.244  -5.547  1.00 27.23           H  0.050
ATOM    792  H  AASN A  46      21.627 -15.704  -4.329  0.52 19.61           H  0.043
ATOM    793  H  BASN A  46      21.622 -15.713  -4.328  0.48 19.61           H  0.043
ATOM    794  N   THR A  47      24.900 -18.001  -3.446  1.00 24.00           N  0.047
ANISOU  794  N   THR A  47     2274   3145   3698    567    163    772       N
ATOM    795  CA  THR A  47      25.091 -19.294  -2.805  1.00 27.65           C  0.051
ANISOU  795  CA  THR A  47     2622   3455   4431    544     20    800       C
ATOM    796  C   THR A  47      24.629 -19.299  -1.348  1.00 27.89           C  0.051
ANISOU  796  C   THR A  47     2766   3589   4241    509   -240    987       C
ATOM    797  O   THR A  47      24.308 -20.369  -0.814  1.00 30.22           O  0.053
ANISOU  797  O   THR A  47     2841   3644   4997    492   -355    911       O
ATOM    798  CB  THR A  47      26.535 -19.786  -2.970  1.00 31.80           C  0.055
ANISOU  798  CB  THR A  47     2822   3612   5649    528    -31    542       C
ATOM    799  OG1 THR A  47      26.644 -21.106  -2.431  1.00 33.89           O  0.056
ANISOU  799  OG1 THR A  47     3031   3736   6109    505    -75    469       O
ATOM    800  CG2 THR A  47      27.509 -18.870  -2.259  1.00 32.68           C  0.055
ANISOU  800  CG2 THR A  47     2749   3690   5980    559      2    389       C
ATOM    801  H   THR A  47      25.617 -17.616  -3.725  1.00 28.94           H  0.052
ATOM    802  HA  THR A  47      24.530 -19.933  -3.271  1.00 33.33           H  0.056
ATOM    803  HB  THR A  47      26.764 -19.803  -3.913  1.00 38.31           H  0.060
ATOM    804  HG1 THR A  47      27.431 -21.387  -2.516  1.00 40.82           H  0.062
ATOM    805 HG21 THR A  47      28.419 -19.165  -2.421  1.00 39.37           H  0.061
ATOM    806 HG22 THR A  47      27.410 -17.962  -2.586  1.00 39.37           H  0.061
ATOM    807 HG23 THR A  47      27.340 -18.881  -1.304  1.00 39.37           H  0.061
ATOM    808  N   ASP A  48      24.556 -18.132  -0.707  1.00 25.29           N  0.049
ANISOU  808  N   ASP A  48     2771   3626   3212    568   -355   1013       N
ATOM    809  CA  ASP A  48      24.104 -18.028   0.677  1.00 25.11           C  0.048
ANISOU  809  CA  ASP A  48     2780   3607   3154    351   -195   1216       C
ATOM    810  C   ASP A  48      22.587 -18.012   0.824  1.00 24.88           C  0.048
ANISOU  810  C   ASP A  48     2767   3453   3233    312    -89   1080       C
ATOM    811  O   ASP A  48      22.091 -17.855   1.947  1.00 25.40           O  0.049
ANISOU  811  O   ASP A  48     2854   3629   3168    389     -2    950       O
ATOM    812  CB  ASP A  48      24.757 -16.820   1.373  1.00 24.78           C  0.048
ANISOU  812  CB  ASP A  48     2818   3691   2908    204   -301   1113       C
ATOM    813  CG  ASP A  48      24.195 -15.471   0.905  1.00 25.90           C  0.049
ANISOU  813  CG  ASP A  48     2847   3821   3173      2   -451    994       C
ATOM    814  OD1 ASP A  48      23.323 -15.425   0.005  1.00 25.21           O  0.049
ANISOU  814  OD1 ASP A  48     2658   3794   3125     49   -570   1050       O
ATOM    815  OD2 ASP A  48      24.632 -14.441   1.462  1.00 27.42           O  0.051
ANISOU  815  OD2 ASP A  48     2953   3939   3526   -180   -624    794       O
ATOM    816  H   ASP A  48      24.767 -17.376  -1.059  1.00 30.50           H  0.053
ATOM    817  HA  ASP A  48      24.417 -18.818   1.146  1.00 30.28           H  0.053
ATOM    818  HB2 ASP A  48      24.607 -16.890   2.329  1.00 29.89           H  0.053
ATOM    819  HB3 ASP A  48      25.709 -16.827   1.187  1.00 29.89           H  0.053
ATOM    820  N   GLY A  49      21.841 -18.192  -0.263  1.00 23.96           N  0.047
ANISOU  820  N   GLY A  49     2572   3049   3482    187   -113   1003       N
ATOM    821  CA  GLY A  49      20.402 -18.187  -0.205  1.00 22.57           C  0.046
ANISOU  821  CA  GLY A  49     2417   2676   3483     58   -162    895       C
ATOM    822  C   GLY A  49      19.758 -16.838  -0.408  1.00 19.45           C  0.043
ANISOU  822  C   GLY A  49     2172   2264   2955      3   -118    569       C
ATOM    823  O   GLY A  49      18.563 -16.786  -0.700  1.00 18.68           O  0.042
ANISOU  823  O   GLY A  49     2250   2190   2659    -78    -58    559       O
ATOM    824  H   GLY A  49      22.157 -18.320  -1.052  1.00 28.90           H  0.052
ATOM    825  HA2 GLY A  49      20.059 -18.786  -0.887  1.00 27.24           H  0.050
ATOM    826  HA3 GLY A  49      20.120 -18.526   0.659  1.00 27.24           H  0.050
ATOM    827  N   SER A  50      20.504 -15.743  -0.295  1.00 17.02           N  0.040
ANISOU  827  N   SER A  50     1918   2003   2545    -26   -269    442       N
ATOM    828  CA  SER A  50      19.901 -14.478  -0.650  1.00 15.80           C  0.038
ANISOU  828  CA  SER A  50     1877   1858   2269    -39   -233    157       C
ATOM    829  C   SER A  50      19.779 -14.378  -2.168  1.00 13.29           C  0.035
ANISOU  829  C   SER A  50     1625   1456   1969    -16    -61    195       C
ATOM    830  O   SER A  50      20.374 -15.185  -2.917  1.00 13.23           O  0.035
ANISOU  830  O   SER A  50     1616   1301   2111    -34    -70   -132       O
ATOM    831  CB  SER A  50      20.737 -13.312  -0.127  1.00 16.76           C  0.040
ANISOU  831  CB  SER A  50     2030   1989   2349    -60   -294    377       C
ATOM    832  OG  SER A  50      21.969 -13.152  -0.827  1.00 17.95           O  0.041
ANISOU  832  OG  SER A  50     2169   2007   2645   -192   -351    537       O
ATOM    833  H   SER A  50      21.321 -15.709  -0.028  1.00 20.57           H  0.044
ATOM    834  HA  SER A  50      19.014 -14.419  -0.263  1.00 19.11           H  0.042
ATOM    835  HB2 SER A  50      20.221 -12.496  -0.220  1.00 20.26           H  0.044
ATOM    836  HB3 SER A  50      20.932 -13.468   0.810  1.00 20.26           H  0.044
ATOM    837  HG  SER A  50      22.434 -13.848  -0.753  1.00 21.69           H  0.045
ATOM    838  N   THR A  51      19.018 -13.352  -2.611  1.00 11.62           N  0.033
```

```
ANISOU  838  N   THR A  51     1479   1228   1709    -81   -181    193       N
ATOM    839  CA  THR A  51      18.815 -13.063  -4.020  1.00 11.55           C  0.033
ANISOU  839  CA  THR A  51     1502   1022   1864   -160    169    343       C
ATOM    840  C   THR A  51      19.057 -11.578  -4.286  1.00 11.03           C  0.032
ANISOU  840  C   THR A  51     1265    932   1996   -201    198    315       C
ATOM    841  O   THR A  51      18.696 -10.706  -3.478  1.00 10.77           O  0.032
ANISOU  841  O   THR A  51     1135    978   1979   -342    -83    114       O
ATOM    842  CB  THR A  51      17.408 -13.488  -4.443  1.00 12.35           C  0.034
ANISOU  842  CB  THR A  51     1864   1119   1708   -273    258     95       C
ATOM    843  OG1 THR A  51      17.224 -14.869  -4.122  1.00 12.70           O  0.034
ANISOU  843  OG1 THR A  51     1935   1021   1871   -502    -45    181       O
ATOM    844  CG2 THR A  51      17.126 -13.269  -5.938  1.00 14.05           C  0.036
ANISOU  844  CG2 THR A  51     1943   1321   2076   -443    432    176       C
ATOM    845  H   THR A  51      18.606 -12.806  -2.090  1.00 14.10           H  0.036
ATOM    846  HA  THR A  51      19.454 -13.572  -4.542  1.00 14.01           H  0.036
ATOM    847  HB  THR A  51      16.764 -12.970  -3.939  1.00 14.96           H  0.037
ATOM    848  HG1 THR A  51      17.279 -14.981  -3.291  1.00 15.39           H  0.038
ATOM    849 HG21 THR A  51      16.234 -13.583  -6.155  1.00 17.01           H  0.040
ATOM    850 HG22 THR A  51      17.189 -12.326  -6.154  1.00 17.01           H  0.040
ATOM    851 HG23 THR A  51      17.770 -13.759  -6.472  1.00 17.01           H  0.040
ATOM    852  N   ASP A  52      19.639 -11.285  -5.442  1.00 11.76           N  0.033
ANISOU  852  N   ASP A  52     1550   1301   1619   -127    235    -92       N
ATOM    853  CA  ASP A  52      19.803  -9.927  -5.944  1.00 12.43           C  0.034
ANISOU  853  CA  ASP A  52     1437   1449   1836   -224     46   -106       C
ATOM    854  C   ASP A  52      18.716  -9.617  -6.953  1.00 11.47           C  0.033
ANISOU  854  C   ASP A  52     1414   1130   1814   -300     47   -234       C
ATOM    855  O   ASP A  52      18.396 -10.459  -7.802  1.00 11.92           O  0.033
ANISOU  855  O   ASP A  52     1571   1056   1902   -411   -261   -194       O
ATOM    856  CB  ASP A  52      21.103  -9.890  -6.718  1.00 14.97           C  0.037
ANISOU  856  CB  ASP A  52     1462   1856   2369    -82     94    241       C
ATOM    857  CG  ASP A  52      22.328  -9.952  -5.820  1.00 18.58           C  0.042
ANISOU  857  CG  ASP A  52     1623   2509   2928     10     51    188       C
ATOM    858  OD1 ASP A  52      22.193  -9.857  -4.585  1.00 18.79           O  0.042
ANISOU  858  OD1 ASP A  52     1807   2534   2798   -208     21    254       O
ATOM    859  OD2 ASP A  52      23.420 -10.256  -6.339  1.00 21.21           O  0.045
ANISOU  859  OD2 ASP A  52     1579   3080   3400    190   -629    308       O
ATOM    860  H   ASP A  52      19.959 -11.881  -5.973  1.00 14.27           H  0.037
ATOM    861  HA  ASP A  52      19.809  -9.273  -5.228  1.00 15.06           H  0.038
ATOM    862  HB2 ASP A  52      21.131 -10.648  -7.319  1.00 18.11           H  0.041
ATOM    863  HB3 ASP A  52      21.143  -9.064  -7.224  1.00 18.11           H  0.041
ATOM    864  N   TYR A  53      18.177  -8.406  -6.884  1.00 11.56           N  0.033
ANISOU  864  N   TYR A  53     1226    963   2203   -180    229    -15       N
ATOM    865  CA  TYR A  53      16.985  -8.079  -7.646  1.00 12.04           C  0.034
ANISOU  865  CA  TYR A  53     1149    881   2546   -185     24    -62       C
ATOM    866  C   TYR A  53      17.160  -6.773  -8.412  1.00 12.22           C  0.034
ANISOU  866  C   TYR A  53     1307    762   2575   -155     66   -209       C
ATOM    867  O   TYR A  53      17.523  -5.744  -7.827  1.00 11.68           O  0.033
ANISOU  867  O   TYR A  53     1531    629   2279   -232    -58    -41       O
ATOM    868  CB  TYR A  53      15.815  -7.858  -6.716  1.00 12.20           C  0.034
ANISOU  868  CB  TYR A  53     1088   1037   2511   -101    -76     65       C
ATOM    869  CG  TYR A  53      15.389  -9.097  -5.994  1.00 12.38           C  0.034
ANISOU  869  CG  TYR A  53     1231   1102   2372   -105   -218    -56       C
ATOM    870  CD1 TYR A  53      15.967  -9.480  -4.816  1.00 11.27           C  0.032
ANISOU  870  CD1 TYR A  53     1243   1037   2002   -384     46   -499       C
ATOM    871  CD2 TYR A  53      14.376  -9.900  -6.530  1.00 11.97           C  0.033
ANISOU  871  CD2 TYR A  53     1114   1319   2113    -46   -324     84       C
ATOM    872  CE1 TYR A  53      15.516 -10.614  -4.134  1.00 11.26           C  0.032
ANISOU  872  CE1 TYR A  53     1062   1276   1940   -374    223   -310       C
ATOM    873  CE2 TYR A  53      13.936 -11.067  -5.899  1.00 12.18           C  0.034
ANISOU  873  CE2 TYR A  53      957   1478   2195   -288    -66     43       C
ATOM    874  CZ  TYR A  53      14.505 -11.410  -4.705  1.00 10.89           C  0.032
ANISOU  874  CZ  TYR A  53     1032   1453   1653   -310    297    183       C
ATOM    875  OH  TYR A  53      14.053 -12.546  -4.117  1.00 12.13           O  0.034
ANISOU  875  OH  TYR A  53     1101   1404   2103   -379   -120    467       O
ATOM    876  H   TYR A  53      18.482  -7.760  -6.405  1.00 14.02           H  0.036
ATOM    877  HA  TYR A  53      16.770  -8.790  -8.269  1.00 14.60           H  0.037
ATOM    878  HB2 TYR A  53      16.063  -7.196  -6.052  1.00 14.79           H  0.037
ATOM    879  HB3 TYR A  53      15.059  -7.540  -7.233  1.00 14.79           H  0.037
ATOM    880  HD1 TYR A  53      16.626  -8.946  -4.434  1.00 13.67           H  0.036
ATOM    881  HD2 TYR A  53      13.988  -9.653  -7.338  1.00 14.51           H  0.037
ATOM    882  HE1 TYR A  53      15.917 -10.869  -3.335  1.00 13.66           H  0.036
ATOM    883  HE2 TYR A  53      13.257 -11.583  -6.270  1.00 14.77           H  0.037
ATOM    884  HH  TYR A  53      14.464 -12.680  -3.397  1.00 14.70           H  0.037
ATOM    885  N   GLY A  54      16.835  -6.788  -9.688  1.00 12.09           N  0.034
ANISOU  885  N   GLY A  54     1461    977   2155   -160   -198     31       N
ATOM    886  CA  GLY A  54      16.571  -5.560 -10.402  1.00 13.99           C  0.036
ANISOU  886  CA  GLY A  54     1517   1267   2533   -132   -136    172       C
ATOM    887  C   GLY A  54      17.792  -4.994 -11.090  1.00 14.52           C  0.037
```

```
ANISOU  887  C   GLY A  54     1509   1285   2722   -233    -80     39       C
ATOM    888  O   GLY A  54      18.825  -5.654 -11.231  1.00 13.83           O  0.036
ANISOU  888  O   GLY A  54     1454   1206   2596   -265     90    -34       O
ATOM    889  H   GLY A  54      16.760  -7.499 -10.166  1.00 14.65           H  0.037
ATOM    890  HA2 GLY A  54      15.893  -5.722 -11.071  1.00 16.94           H  0.040
ATOM    891  HA3 GLY A  54      16.236  -4.894  -9.782  1.00 16.94           H  0.040
ATOM    892  N   ILE A  55      17.641  -3.756 -11.554  1.00 13.48           N  0.036
ANISOU  892  N   ILE A  55     1364   1249   2510   -228   -249    219       N
ATOM    893  CA  ILE A  55      18.659  -3.140 -12.403  1.00 14.89           C  0.037
ANISOU  893  CA  ILE A  55     1606   1315   2736   -270   -333    338       C
ATOM    894  C   ILE A  55      19.997  -2.923 -11.708  1.00 14.91           C  0.037
ANISOU  894  C   ILE A  55     1600   1368   2697   -350     26    402       C
ATOM    895  O   ILE A  55      21.035  -2.845 -12.381  1.00 13.52           O  0.036
ANISOU  895  O   ILE A  55     1495   1340   2303   -439    214    368       O
ATOM    896  CB  ILE A  55      18.045  -1.878 -13.032  1.00 19.95           C  0.043
ANISOU  896  CB  ILE A  55     2176   1640   3766   -249   -351    720       C
ATOM    897  CG1 ILE A  55      18.503  -1.700 -14.490  1.00 22.30           C  0.046
ANISOU  897  CG1 ILE A  55     2519   1829   4123   -161   -449   1031       C
ATOM    898  CG2 ILE A  55      18.104  -0.729 -12.092  1.00 21.57           C  0.045
ANISOU  898  CG2 ILE A  55     2295   1655   4245   -237   -670    647       C
ATOM    899  CD1 ILE A  55      17.849  -0.597 -15.190  1.00 23.54           C  0.047
ANISOU  899  CD1 ILE A  55     2699   1834   4410   -218   -528   1257       C
ATOM    900  H   ILE A  55      16.962  -3.254 -11.391  1.00 16.33           H  0.039
ATOM    901  HA  ILE A  55      18.831  -3.752 -13.134  1.00 18.02           H  0.041
ATOM    902  HB  ILE A  55      17.098  -2.075 -13.106  1.00 24.09           H  0.047
ATOM    903 HG12 ILE A  55      19.458  -1.531 -14.498  1.00 26.91           H  0.050
ATOM    904 HG13 ILE A  55      18.310  -2.515 -14.980  1.00 26.91           H  0.050
ATOM    905 HG21 ILE A  55      17.523  -0.024 -12.419  1.00 26.03           H  0.049
ATOM    906 HG22 ILE A  55      17.807  -1.023 -11.216  1.00 26.03           H  0.049
ATOM    907 HG23 ILE A  55      19.018  -0.407 -12.041  1.00 26.03           H  0.049
ATOM    908 HD11 ILE A  55      18.173  -0.567 -16.104  1.00 28.39           H  0.052
ATOM    909 HD12 ILE A  55      16.890  -0.743 -15.183  1.00 28.39           H  0.052
ATOM    910 HD13 ILE A  55      18.060   0.234 -14.737  1.00 28.39           H  0.052
ATOM    911  N   LEU A  56      19.993  -2.769 -10.385  1.00 13.65           N  0.036
ANISOU  911  N   LEU A  56     1562   1276   2348   -440    -40    531       N
ATOM    912  CA  LEU A  56      21.200  -2.646  -9.585  1.00 12.93           C  0.035
ANISOU  912  CA  LEU A  56     1556   1026   2330   -438   -152    244       C
ATOM    913  C   LEU A  56      21.417  -3.853  -8.679  1.00 13.02           C  0.035
ANISOU  913  C   LEU A  56     1433   1333   2182   -337   -419    307       C
ATOM    914  O   LEU A  56      22.242  -3.792  -7.764  1.00 12.51           O  0.034
ANISOU  914  O   LEU A  56     1409   1391   1951   -522   -221    -20       O
ATOM    915  CB  LEU A  56      21.192  -1.311  -8.809  1.00 11.31           C  0.033
ANISOU  915  CB  LEU A  56     1689    784   1824   -489   -201    285       C
ATOM    916  CG  LEU A  56      21.372  -0.093  -9.723  1.00 14.63           C  0.037
ANISOU  916  CG  LEU A  56     1809    899   2850   -353     38    299       C
ATOM    917  CD1 LEU A  56      21.084   1.223  -8.992  1.00 15.62           C  0.038
ANISOU  917  CD1 LEU A  56     1875    956   3104   -259    238    400       C
ATOM    918  CD2 LEU A  56      22.791  -0.121 -10.268  1.00 15.74           C  0.038
ANISOU  918  CD2 LEU A  56     1951   1064   2963   -479    -13    -62       C
ATOM    919  H   LEU A  56      19.273  -2.732  -9.916  1.00 16.53           H  0.039
ATOM    920  HA  LEU A  56      21.957  -2.623 -10.189  1.00 15.66           H  0.038
ATOM    921  HB2 LEU A  56      20.342  -1.218  -8.351  1.00 13.72           H  0.036
ATOM    922  HB3 LEU A  56      21.916  -1.315  -8.164  1.00 13.72           H  0.036
ATOM    923  HG  LEU A  56      20.759  -0.163 -10.472  1.00 17.70           H  0.041
ATOM    924 HD11 LEU A  56      21.208   1.961  -9.610  1.00 18.89           H  0.042
ATOM    925 HD12 LEU A  56      20.169   1.211  -8.670  1.00 18.89           H  0.042
ATOM    926 HD13 LEU A  56      21.697   1.310  -8.245  1.00 18.89           H  0.042
ATOM    927 HD21 LEU A  56      23.014   0.760 -10.608  1.00 19.03           H  0.042
ATOM    928 HD22 LEU A  56      23.401  -0.363  -9.554  1.00 19.03           H  0.042
ATOM    929 HD23 LEU A  56      22.844  -0.773 -10.984  1.00 19.03           H  0.042
ATOM    930  N   GLN A  57      20.672  -4.935  -8.891  1.00 13.82           N  0.036
ANISOU  930  N   GLN A  57     1417   1485   2349   -166   -211    299       N
ATOM    931  CA  GLN A  57      20.960  -6.199  -8.231  1.00 12.70           C  0.034
ANISOU  931  CA  GLN A  57     1313   1568   1946   -226    -88    136       C
ATOM    932  C   GLN A  57      21.067  -6.056  -6.712  1.00 14.12           C  0.036
ANISOU  932  C   GLN A  57     1510   1567   2287   -287   -196     19       C
ATOM    933  O   GLN A  57      22.076  -6.377  -6.089  1.00 13.95           O  0.036
ANISOU  933  O   GLN A  57     1631   1716   1955   -422   -189    239       O
ATOM    934  CB  GLN A  57      22.168  -6.875  -8.871  1.00 13.10           C  0.035
ANISOU  934  CB  GLN A  57     1204   1545   2231   -276    170    441       C
ATOM    935  CG  GLN A  57      21.871  -7.349 -10.284  1.00 11.55           C  0.033
ANISOU  935  CG  GLN A  57     1101   1324   1963   -339    262    625       C
ATOM    936  CD  GLN A  57      20.934  -8.515 -10.273  1.00 11.92           C  0.033
ANISOU  936  CD  GLN A  57     1173   1112   2242   -452    -10    483       C
ATOM    937  OE1 GLN A  57      21.393  -9.637 -10.071  1.00 12.96           O  0.035
ANISOU  937  OE1 GLN A  57     1326   1114   2485   -399    135    257       O
ATOM    938  NE2 GLN A  57      19.663  -8.274 -10.531  1.00 12.12           N  0.034
ANISOU  938  NE2 GLN A  57     1120   1232   2253   -556    266     87       N
```

```
ATOM    939  H   GLN A  57      19.991  -4.962  -9.415  1.00 16.73           H  0.040
ATOM    940  HA  GLN A  57      20.202  -6.783  -8.389  1.00 15.39           H  0.038
ATOM    941  HB2 GLN A  57      22.901  -6.241  -8.914  1.00 15.88           H  0.039
ATOM    942  HB3 GLN A  57      22.425  -7.645  -8.340  1.00 15.88           H  0.039
ATOM    943  HG2 GLN A  57      21.458  -6.629 -10.786  1.00 14.01           H  0.036
ATOM    944  HG3 GLN A  57      22.697  -7.626 -10.709  1.00 14.01           H  0.036
ATOM    945 HE21 GLN A  57      19.398  -7.468 -10.674  1.00 14.69           H  0.037
ATOM    946 HE22 GLN A  57      19.096  -8.920 -10.538  1.00 14.69           H  0.037
ATOM    947  N   ILE A  58      19.996  -5.519  -6.128  1.00 13.55           N  0.036
ANISOU  947  N   ILE A  58     1668   1444   2038   -110   -260   -261       N
ATOM    948  CA  ILE A  58      19.984  -5.252  -4.697  1.00 13.55           C  0.036
ANISOU  948  CA  ILE A  58     1675   1373   2101   -232    195   -254       C
ATOM    949  C   ILE A  58      19.608  -6.507  -3.939  1.00 13.87           C  0.036
ANISOU  949  C   ILE A  58     1860   1250   2162   -328    -70   -121       C
ATOM    950  O   ILE A  58      18.705  -7.239  -4.352  1.00 16.06           O  0.039
ANISOU  950  O   ILE A  58     1812   1459   2832   -345   -220     18       O
ATOM    951  CB  ILE A  58      19.061  -4.070  -4.392  1.00 14.34           C  0.037
ANISOU  951  CB  ILE A  58     1738   1502   2208   -158    536   -155       C
ATOM    952  CG1 ILE A  58      19.713  -2.796  -4.916  1.00 13.41           C  0.035
ANISOU  952  CG1 ILE A  58     1771   1762   1564    -63    309   -213       C
ATOM    953  CG2 ILE A  58      18.784  -3.921  -2.912  1.00 14.08           C  0.036
ANISOU  953  CG2 ILE A  58     1651   1249   2449   -227    665   -576       C
ATOM    954  CD1 ILE A  58      18.805  -1.641  -4.931  1.00 14.65           C  0.037
ANISOU  954  CD1 ILE A  58     1764   1842   1961    -62    270    -95       C
ATOM    955  H   ILE A  58      19.271  -5.303  -6.537  1.00 16.41           H  0.039
ATOM    956  HA  ILE A  58      20.879  -5.001  -4.421  1.00 16.41           H  0.039
ATOM    957  HB  ILE A  58      18.221  -4.203  -4.856  1.00 17.36           H  0.040
ATOM    958 HG12 ILE A  58      20.468  -2.574  -4.349  1.00 16.24           H  0.039
ATOM    959 HG13 ILE A  58      20.016  -2.947  -5.825  1.00 16.24           H  0.039
ATOM    960 HG21 ILE A  58      18.299  -3.096  -2.758  1.00 17.04           H  0.040
ATOM    961 HG22 ILE A  58      18.249  -4.672  -2.610  1.00 17.04           H  0.040
ATOM    962 HG23 ILE A  58      19.628  -3.901  -2.434  1.00 17.04           H  0.040
ATOM    963 HD11 ILE A  58      19.188  -0.947  -5.490  1.00 17.73           H  0.041
ATOM    964 HD12 ILE A  58      17.948  -1.919  -5.290  1.00 17.73           H  0.041
ATOM    965 HD13 ILE A  58      18.694  -1.312  -4.025  1.00 17.73           H  0.041
ATOM    966  N   ASN A  59      20.352  -6.733  -2.868  1.00 14.93           N  0.037
ANISOU  966  N   ASN A  59     2086   1226   2359   -499   -193   -136       N
ATOM    967  CA  ASN A  59      20.310  -8.022  -2.138  1.00 16.45           C  0.039
ANISOU  967  CA  ASN A  59     2408   1419   2422   -505     91   -153       C
ATOM    968  C   ASN A  59      19.257  -8.080  -1.045  1.00 17.05           C  0.040
ANISOU  968  C   ASN A  59     2683   1246   2548   -514    282    -65       C
ATOM    969  O   ASN A  59      19.046  -7.109  -0.313  1.00 17.73           O  0.041
ANISOU  969  O   ASN A  59     2961   1276   2501   -819    377   -331       O
ATOM    970  CB  ASN A  59      21.709  -8.292  -1.570  1.00 19.26           C  0.042
ANISOU  970  CB  ASN A  59     2562   1659   3099   -436    -60     -1       C
ATOM    971  CG  ASN A  59      21.906  -9.723  -1.126  1.00 23.01           C  0.046
ANISOU  971  CG  ASN A  59     2896   2052   3793   -200   -185     63       C
ATOM    972  OD1 ASN A  59      21.767 -10.028   0.049  1.00 25.14           O  0.049
ANISOU  972  OD1 ASN A  59     3144   2366   4041   -134   -312     57       O
ATOM    973  ND2 ASN A  59      22.226 -10.596  -2.062  1.00 25.02           N  0.048
ANISOU  973  ND2 ASN A  59     2959   2122   4426    -21   -338    134       N
ATOM    974  H   ASN A  59      20.898  -6.161  -2.530  1.00 18.06           H  0.041
ATOM    975  HA  ASN A  59      20.110  -8.728  -2.772  1.00 19.89           H  0.043
ATOM    976  HB2 ASN A  59      22.369  -8.098  -2.254  1.00 23.27           H  0.047
ATOM    977  HB3 ASN A  59      21.852  -7.719  -0.801  1.00 23.27           H  0.047
ATOM    978 HD21 ASN A  59      22.348 -11.422  -1.856  1.00 30.17           H  0.053
ATOM    979 HD22 ASN A  59      22.314 -10.339  -2.878  1.00 30.17           H  0.053
ATOM    980  N   SER A  60      18.667  -9.270  -0.927  1.00 15.74           N  0.038
ANISOU  980  N   SER A  60     2662   1076   2242   -247    244    -87       N
ATOM    981  CA  SER A  60      17.685  -9.533   0.109  1.00 14.81           C  0.037
ANISOU  981  CA  SER A  60     2586    993   2050    -44    182   -104       C
ATOM    982  C   SER A  60      18.298  -9.873   1.470  1.00 16.22           C  0.039
ANISOU  982  C   SER A  60     2888   1486   1788    -46    -20    -61       C
ATOM    983  O   SER A  60      17.544 -10.056   2.419  1.00 18.43           O  0.042
ANISOU  983  O   SER A  60     3080   1883   2039    236    280    104       O
ATOM    984  CB  SER A  60      16.843 -10.734  -0.314  1.00 12.21           C  0.034
ANISOU  984  CB  SER A  60     2006    692   1940   -190    607   -191       C
ATOM    985  OG  SER A  60      17.621 -11.934  -0.389  1.00 13.34           O  0.035
ANISOU  985  OG  SER A  60     1889    713   2467   -207    174   -282       O
ATOM    986  H   SER A  60      18.823  -9.942  -1.441  1.00 19.04           H  0.042
ATOM    987  HA  SER A  60      17.103  -8.764   0.212  1.00 17.93           H  0.041
ATOM    988  HB2 SER A  60      16.134 -10.862   0.335  1.00 14.80           H  0.037
ATOM    989  HB3 SER A  60      16.460 -10.555  -1.187  1.00 14.80           H  0.037
ATOM    990  HG  SER A  60      17.135 -12.578  -0.623  1.00 16.16           H  0.039
ATOM    991  N   ARG A  61      19.626  -9.948   1.621  1.00 18.52           N  0.042
ANISOU  991  N   ARG A  61     3122   1708   2205   -354   -460   -175       N
ATOM    992  CA  ARG A  61      20.153 -10.135   2.975  1.00 20.40           C  0.044
ANISOU  992  CA  ARG A  61     3436   2069   2247   -557   -602   -396       C
```

```
ATOM    993  C   ARG A  61      19.969  -8.876   3.805  1.00 20.24           C  0.044
ANISOU  993  C   ARG A  61     3287   2164   2241   -701   -259   -348       C
ATOM    994  O   ARG A  61      19.671  -8.946   5.005  1.00 21.25           O  0.045
ANISOU  994  O   ARG A  61     3431   2241   2404   -874   -265    -31       O
ATOM    995  CB  ARG A  61      21.623 -10.565   2.954  1.00 23.40           C  0.047
ANISOU  995  CB  ARG A  61     3942   2342   2606   -438   -906   -246       C
ATOM    996  CG  ARG A  61      22.317 -10.769   4.324  1.00 27.44           C  0.051
ANISOU  996  CG  ARG A  61     4417   2739   3271   -365  -1140   -207       C
ATOM    997  CD  ARG A  61      21.460 -11.466   5.389  1.00 30.68           C  0.054
ANISOU  997  CD  ARG A  61     4816   3072   3771   -317  -1302   -341       C
ATOM    998  NE  ARG A  61      21.918 -11.272   6.772  1.00 32.92           N  0.056
ANISOU  998  NE  ARG A  61     5146   3331   4031   -182  -1501   -288       N
ATOM    999  CZ  ARG A  61      21.695 -10.211   7.543  1.00 33.90           C  0.056
ANISOU  999  CZ  ARG A  61     5366   3461   4054    -89  -1637   -381       C
ATOM   1000  NH1 ARG A  61      21.011  -9.166   7.112  1.00 34.46           N  0.057
ANISOU 1000  NH1 ARG A  61     5483   3526   4085    -23  -1557   -465       N
ATOM   1001  NH2 ARG A  61      22.153 -10.213   8.790  1.00 36.02           N  0.058
ANISOU 1001  NH2 ARG A  61     5496   3542   4646    -82  -1532   -235       N
ATOM   1002  H   ARG A  61      20.212  -9.897   0.994  1.00 22.37           H  0.046
ATOM   1003  HA  ARG A  61      19.646 -10.844   3.399  1.00 24.63           H  0.048
ATOM   1004  HB2 ARG A  61      21.685 -11.406   2.474  1.00 28.22           H  0.051
ATOM   1005  HB3 ARG A  61      22.128  -9.887   2.478  1.00 28.22           H  0.051
ATOM   1006  HG2 ARG A  61      23.112 -11.308   4.190  1.00 33.08           H  0.056
ATOM   1007  HG3 ARG A  61      22.569  -9.900   4.674  1.00 33.08           H  0.056
ATOM   1008  HD2 ARG A  61      20.547 -11.149   5.341  1.00 36.97           H  0.059
ATOM   1009  HD3 ARG A  61      21.487 -12.411   5.223  1.00 36.97           H  0.059
ATOM   1010  HE  ARG A  61      22.453 -11.867   7.086  1.00 39.65           H  0.061
ATOM   1011 HH11 ARG A  61      20.705  -9.153   6.309  1.00 41.51           H  0.062
ATOM   1012 HH12 ARG A  61      20.879  -8.495   7.633  1.00 41.51           H  0.062
ATOM   1013 HH21 ARG A  61      22.598 -10.888   9.084  1.00 43.37           H  0.064
ATOM   1014 HH22 ARG A  61      22.015  -9.534   9.300  1.00 43.37           H  0.064
ATOM   1015  N   TRP A  62      20.015  -7.717   3.167  1.00 18.86           N  0.042
ANISOU 1015  N   TRP A  62     2826   2077   2262   -615   -190   -126       N
ATOM   1016  CA  TRP A  62      19.980  -6.484   3.980  1.00 18.39           C  0.041
ANISOU 1016  CA  TRP A  62     2487   1995   2507   -850    -80   -156       C
ATOM   1017  C   TRP A  62      18.948  -5.473   3.537  1.00 16.07           C  0.039
ANISOU 1017  C   TRP A  62     2203   1825   2079   -903   -199   -679       C
ATOM   1018  O   TRP A  62      18.430  -4.798   4.392  1.00 16.01           O  0.039
ANISOU 1018  O   TRP A  62     2365   1810   1910   -941    254   -506       O
ATOM   1019  CB  TRP A  62      21.356  -5.838   3.768  1.00 21.86           C  0.045
ANISOU 1019  CB  TRP A  62     2647   2362   3297   -739   -313   -139       C
ATOM   1020  CG  TRP A  62      22.501  -6.545   4.411  1.00 24.69           C  0.048
ANISOU 1020  CG  TRP A  62     2851   2686   3843   -517   -417     -5       C
ATOM   1021  CD1 TRP A  62      23.535  -7.178   3.786  1.00 26.27           C  0.050
ANISOU 1021  CD1 TRP A  62     2972   2886   4123   -409   -482    100       C
ATOM   1022  CD2 TRP A  62      22.753  -6.651   5.817  1.00 26.80           C  0.050
ANISOU 1022  CD2 TRP A  62     3036   2869   4277   -480   -385     70       C
ATOM   1023  NE1 TRP A  62      24.406  -7.687   4.709  1.00 27.15           N  0.050
ANISOU 1023  NE1 TRP A  62     3044   2971   4302   -291   -447    174       N
ATOM   1024  CE2 TRP A  62      23.950  -7.380   5.963  1.00 27.81           C  0.051
ANISOU 1024  CE2 TRP A  62     3132   3006   4428   -363   -482     50       C
ATOM   1025  CE3 TRP A  62      22.079  -6.214   6.959  1.00 27.13           C  0.050
ANISOU 1025  CE3 TRP A  62     3130   2945   4232   -510   -482      0       C
ATOM   1026  CZ2 TRP A  62      24.480  -7.683   7.213  1.00 29.06           C  0.052
ANISOU 1026  CZ2 TRP A  62     3236   3134   4671   -347   -468    -36       C
ATOM   1027  CZ3 TRP A  62      22.609  -6.506   8.194  1.00 28.58           C  0.052
ANISOU 1027  CZ3 TRP A  62     3221   3082   4556   -420   -474     -3       C
ATOM   1028  CH2 TRP A  62      23.795  -7.227   8.315  1.00 29.30           C  0.052
ANISOU 1028  CH2 TRP A  62     3277   3159   4695   -383   -487    -59       C
ATOM   1029  H   TRP A  62      20.064  -7.606   2.316  1.00 22.78           H  0.046
ATOM   1030  HA  TRP A  62      19.858  -6.679   4.921  1.00 22.22           H  0.046
ATOM   1031  HB2 TRP A  62      21.534  -5.801   2.815  1.00 26.38           H  0.050
ATOM   1032  HB3 TRP A  62      21.331  -4.937   4.128  1.00 26.38           H  0.050
ATOM   1033  HD1 TRP A  62      23.631  -7.257   2.864  1.00 31.67           H  0.054
ATOM   1034  HE1 TRP A  62      25.121  -8.131   4.531  1.00 32.74           H  0.055
ATOM   1035  HE3 TRP A  62      21.288  -5.731   6.886  1.00 32.70           H  0.055
ATOM   1036  HZ2 TRP A  62      25.273  -8.161   7.300  1.00 35.02           H  0.057
ATOM   1037  HZ3 TRP A  62      22.167  -6.220   8.961  1.00 34.45           H  0.057
ATOM   1038  HH2 TRP A  62      24.127  -7.414   9.164  1.00 35.31           H  0.057
ATOM   1039  N   TRP A  63      18.581  -5.523   2.264  1.00 14.79           N  0.037
ANISOU 1039  N   TRP A  63     1908   1696   2014   -823   -291   -536       N
ATOM   1040  CA  TRP A  63      17.942  -4.334   1.727  1.00 14.76           C  0.037
ANISOU 1040  CA  TRP A  63     1718   1648   2242   -687   -440   -347       C
ATOM   1041  C   TRP A  63      16.468  -4.482   1.404  1.00 15.66           C  0.038
ANISOU 1041  C   TRP A  63     1577   1700   2672   -540   -272   -444       C
ATOM   1042  O   TRP A  63      15.742  -3.493   1.493  1.00 15.89           O  0.039
ANISOU 1042  O   TRP A  63     1550   1626   2862   -406   -330   -418       O
ATOM   1043  CB  TRP A  63      18.663  -3.884   0.462  1.00 14.01           C  0.036
```

```
ANISOU 1043  CB  TRP A  63     1674   1682   1968   -591   -220    109       C
ATOM   1044  CG  TRP A  63      20.083  -3.618   0.740  1.00 13.63           C  0.036
ANISOU 1044  CG  TRP A  63     1639   1811   1729   -421   -141   -104       C
ATOM   1045  CD1 TRP A  63      21.157  -4.343   0.288  1.00 15.20           C  0.038
ANISOU 1045  CD1 TRP A  63     1520   1753   2500   -440     19   -154       C
ATOM   1046  CD2 TRP A  63      20.606  -2.590   1.581  1.00 14.91           C  0.037
ANISOU 1046  CD2 TRP A  63     1573   1852   2240   -516   -365    -71       C
ATOM   1047  NE1 TRP A  63      22.336  -3.797   0.782  1.00 15.34           N  0.038
ANISOU 1047  NE1 TRP A  63     1448   1740   2640   -463    -16   -236       N
ATOM   1048  CE2 TRP A  63      22.011  -2.718   1.575  1.00 14.39           C  0.037
ANISOU 1048  CE2 TRP A  63     1454   1783   2232   -487   -337   -213       C
ATOM   1049  CE3 TRP A  63      20.028  -1.564   2.334  1.00 15.29           C  0.038
ANISOU 1049  CE3 TRP A  63     1640   1846   2322   -397   -558    -55       C
ATOM   1050  CZ2 TRP A  63      22.845  -1.842   2.290  1.00 15.47           C  0.038
ANISOU 1050  CZ2 TRP A  63     1573   1818   2488   -359   -509   -165       C
ATOM   1051  CZ3 TRP A  63      20.865  -0.694   3.033  1.00 15.75           C  0.038
ANISOU 1051  CZ3 TRP A  63     1668   1824   2494   -328   -771   -208       C
ATOM   1052  CH2 TRP A  63      22.245  -0.850   3.017  1.00 16.16           C  0.039
ANISOU 1052  CH2 TRP A  63     1625   1808   2708   -372   -652   -350       C
ATOM   1053  H   TRP A  63      18.681  -6.184   1.723  1.00 17.89           H  0.041
ATOM   1054  HA  TRP A  63      18.025  -3.618   2.376  1.00 17.86           H  0.041
ATOM   1055  HB2 TRP A  63      18.608  -4.584  -0.207  1.00 16.96           H  0.040
ATOM   1056  HB3 TRP A  63      18.257  -3.068   0.132  1.00 16.96           H  0.040
ATOM   1057  HD1 TRP A  63      21.105  -5.079  -0.278  1.00 18.38           H  0.041
ATOM   1058  HE1 TRP A  63      23.131  -4.081   0.617  1.00 18.56           H  0.042
ATOM   1059  HE3 TRP A  63      19.105  -1.447   2.347  1.00 18.49           H  0.042
ATOM   1060  HZ2 TRP A  63      23.769  -1.943   2.284  1.00 18.72           H  0.042
ATOM   1061  HZ3 TRP A  63      20.490  -0.007   3.535  1.00 19.05           H  0.042
ATOM   1062  HH2 TRP A  63      22.775  -0.257   3.499  1.00 19.55           H  0.043
ATOM   1063  N   CYS A  64      16.004  -5.674   1.033  1.00 16.31           N  0.039
ANISOU 1063  N   CYS A  64     1443   1646   3109   -554   -111   -663       N
ATOM   1064  CA  CYS A  64      14.607  -5.841   0.667  1.00 15.64           C  0.038
ANISOU 1064  CA  CYS A  64     1355   1542   3047   -522     90   -361       C
ATOM   1065  C   CYS A  64      14.092  -7.160   1.223  1.00 15.39           C  0.038
ANISOU 1065  C   CYS A  64     1493   1443   2910   -495    244   -438       C
ATOM   1066  O   CYS A  64      14.877  -8.053   1.535  1.00 15.57           O  0.038
ANISOU 1066  O   CYS A  64     1550   1647   2719   -539   -100   -298       O
ATOM   1067  CB  CYS A  64      14.424  -5.779  -0.855  1.00 14.66           C  0.037
ANISOU 1067  CB  CYS A  64     1286   1606   2677   -447    171   -456       C
ATOM   1068  SG  CYS A  64      15.258  -7.064  -1.766  1.00 14.67           S  0.037
ANISOU 1068  SG  CYS A  64     1130   1576   2868   -492    291   -257       S
ATOM   1069  H   CYS A  64      16.475  -6.392   0.986  1.00 19.72           H  0.043
ATOM   1070  HA  CYS A  64      14.083  -5.127   1.060  1.00 18.92           H  0.042
ATOM   1071  HB2 CYS A  64      13.477  -5.846  -1.055  1.00 17.74           H  0.041
ATOM   1072  HB3 CYS A  64      14.764  -4.928  -1.172  1.00 17.74           H  0.041
ATOM   1073  N   ASN A  65      12.771  -7.288   1.381  1.00 15.97           N  0.039
ANISOU 1073  N   ASN A  65     1554   1454   3060   -485    387   -435       N
ATOM   1074  CA  ASN A  65      12.177  -8.528   1.874  1.00 16.63           C  0.039
ANISOU 1074  CA  ASN A  65     1620   1515   3184   -514    351   -540       C
ATOM   1075  C   ASN A  65      11.662  -9.400   0.730  1.00 16.20           C  0.039
ANISOU 1075  C   ASN A  65     1673   1391   3092   -621    243   -506       C
ATOM   1076  O   ASN A  65      10.876  -8.935  -0.100  1.00 17.60           O  0.041
ANISOU 1076  O   ASN A  65     1764   1519   3404   -509    256   -398       O
ATOM   1077  CB  ASN A  65      10.991  -8.275   2.803  1.00 17.81           C  0.041
ANISOU 1077  CB  ASN A  65     1626   1778   3364   -406    273   -624       C
ATOM   1078  CG  ASN A  65      10.325  -9.585   3.220  1.00 18.65           C  0.042
ANISOU 1078  CG  ASN A  65     1746   2034   3305   -456    589   -686       C
ATOM   1079  OD1 ASN A  65      11.013 -10.468   3.744  1.00 18.40           O  0.041
ANISOU 1079  OD1 ASN A  65     1849   2104   3039   -448    577   -695       O
ATOM   1080  ND2 ASN A  65       9.016  -9.729   2.965  1.00 19.93           N  0.043
ANISOU 1080  ND2 ASN A  65     1665   2215   3694   -503    882   -391       N
ATOM   1081  H   ASN A  65      12.199  -6.669   1.209  1.00 19.32           H  0.043
ATOM   1082  HA  ASN A  65      12.844  -9.033   2.365  1.00 20.10           H  0.043
ATOM   1083  HB2 ASN A  65      11.301  -7.822   3.603  1.00 21.52           H  0.045
ATOM   1084  HB3 ASN A  65      10.333  -7.732   2.342  1.00 21.52           H  0.045
ATOM   1085 HD21 ASN A  65       8.613 -10.456   3.185  1.00 24.07           H  0.047
ATOM   1086 HD22 ASN A  65       8.578  -9.089   2.593  1.00 24.07           H  0.047
ATOM   1087  N   ASP A  66      12.111 -10.659   0.688  1.00 14.63           N  0.037
ANISOU 1087  N   ASP A  66     1699   1263   2597   -705    157   -413       N
ATOM   1088  CA  ASP A  66      11.564 -11.658  -0.226  1.00 13.62           C  0.036
ANISOU 1088  CA  ASP A  66     1746   1148   2282   -666    103   -452       C
ATOM   1089  C   ASP A  66      10.994 -12.851   0.505  1.00 14.88           C  0.037
ANISOU 1089  C   ASP A  66     1759   1237   2659   -641    142   -525       C
ATOM   1090  O   ASP A  66      10.588 -13.790  -0.148  1.00 15.32           O  0.038
ANISOU 1090  O   ASP A  66     1687   1411   2721   -585     -2   -720       O
ATOM   1091  CB  ASP A  66      12.567 -12.069  -1.323  1.00 13.15           C  0.035
ANISOU 1091  CB  ASP A  66     1833   1128   2038   -428    143   -386       C
ATOM   1092  CG  ASP A  66      13.803 -12.818  -0.796  1.00 13.54           C  0.036
```

```
ANISOU 1092  CG  ASP A  66     1760   1362   2021   -475    272   -445       C
ATOM   1093  OD1 ASP A  66      13.865 -13.192   0.414  1.00 13.50           O  0.036
ANISOU 1093  OD1 ASP A  66     1671   1197   2263   -644    120   -272       O
ATOM   1094  OD2 ASP A  66      14.719 -13.037  -1.624  1.00 13.31           O  0.035
ANISOU 1094  OD2 ASP A  66     1807   1493   1756   -369    543    -31       O
ATOM   1095  H   ASP A  66      12.742 -10.960   1.189  1.00 17.71           H  0.041
ATOM   1096  HA  ASP A  66      10.817 -11.247  -0.688  1.00 16.50           H  0.039
ATOM   1097  HB2 ASP A  66      12.116 -12.652  -1.954  1.00 15.94           H  0.039
ATOM   1098  HB3 ASP A  66      12.876 -11.271  -1.779  1.00 15.94           H  0.039
ATOM   1099  N   GLY A  67      10.934 -12.825   1.837  1.00 16.10           N  0.039
ANISOU 1099  N   GLY A  67     1948   1324   2844   -510     13   -400       N
ATOM   1100  CA  GLY A  67      10.313 -13.914   2.564  1.00 16.27           C  0.039
ANISOU 1100  CA  GLY A  67     2046   1273   2863   -503     36   -152       C
ATOM   1101  C   GLY A  67      11.085 -15.209   2.602  1.00 16.89           C  0.040
ANISOU 1101  C   GLY A  67     2244   1331   2843   -626    335     26       C
ATOM   1102  O   GLY A  67      10.650 -16.139   3.283  1.00 18.38           O  0.041
ANISOU 1102  O   GLY A  67     2302   1500   3182   -863    425    332       O
ATOM   1103  H   GLY A  67      11.243 -12.194   2.333  1.00 19.46           H  0.043
ATOM   1104  HA2 GLY A  67      10.166 -13.632   3.480  1.00 19.67           H  0.043
ATOM   1105  HA3 GLY A  67       9.447 -14.098   2.167  1.00 19.67           H  0.043
ATOM   1106  N  AARG A  68      12.223 -15.310   1.912  0.47 15.82           N  0.038
ANISOU 1106  N  AARG A  68     2193   1296   2522   -514    259   -121       N
ATOM   1107  CA AARG A  68      12.948 -16.575   1.848  0.47 15.85           C  0.039
ANISOU 1107  CA AARG A  68     2234   1258   2532   -467     59   -135       C
ATOM   1108  C  AARG A  68      14.445 -16.386   2.073  0.47 16.18           C  0.039
ANISOU 1108  C  AARG A  68     2303   1290   2556   -422   -113   -292       C
ATOM   1109  O  AARG A  68      15.261 -17.152   1.550  0.47 17.09           O  0.040
ANISOU 1109  O  AARG A  68     2346   1386   2760   -229   -407    -13       O
ATOM   1110  CB AARG A  68      12.684 -17.307   0.535  0.47 15.25           C  0.038
ANISOU 1110  CB AARG A  68     2144   1229   2420   -458    275    -57       C
ATOM   1111  CG AARG A  68      13.363 -16.655  -0.641  0.47 14.00           C  0.036
ANISOU 1111  CG AARG A  68     2041   1172   2109   -492    385    -27       C
ATOM   1112  CD AARG A  68      13.311 -17.458  -1.905  0.47 12.65           C  0.034
ANISOU 1112  CD AARG A  68     1887   1134   1786   -597    370   -146       C
ATOM   1113  NE AARG A  68      14.138 -16.805  -2.899  0.47 11.67           N  0.033
ANISOU 1113  NE AARG A  68     1730   1085   1620   -685    318   -410       N
ATOM   1114  CZ AARG A  68      14.288 -17.212  -4.147  0.47 11.78           C  0.033
ANISOU 1114  CZ AARG A  68     1731   1055   1688   -680    281   -265       C
ATOM   1115  NH1AARG A  68      13.520 -18.144  -4.658  0.47 11.81           N  0.033
ANISOU 1115  NH1AARG A  68     1684   1254   1549   -550    151    -22       N
ATOM   1116  NH2AARG A  68      15.215 -16.640  -4.907  0.47 10.69           N  0.032
ANISOU 1116  NH2AARG A  68     1504    897   1661   -538   -155   -351       N
ATOM   1117  H  AARG A  68      12.592 -14.666   1.477  0.47 19.13           H  0.042
ATOM   1118  HA AARG A  68      12.628 -17.149   2.561  0.47 19.17           H  0.042
ATOM   1119  HB2AARG A  68      13.016 -18.216   0.607  0.47 18.44           H  0.042
ATOM   1120  HB3AARG A  68      11.729 -17.315   0.364  0.47 18.44           H  0.042
ATOM   1121  HG2AARG A  68      12.927 -15.807  -0.816  0.47 16.95           H  0.040
ATOM   1122  HG3AARG A  68      14.299 -16.503  -0.448  0.47 16.95           H  0.040
ATOM   1123  HD2AARG A  68      13.659 -18.349  -1.744  0.47 15.33           H  0.038
ATOM   1124  HD3AARG A  68      12.399 -17.497  -2.233  0.47 15.33           H  0.038
ATOM   1125  HE AARG A  68      14.564 -16.098  -2.658  0.47 14.16           H  0.036
ATOM   1126 HH11AARG A  68      12.919 -18.523  -4.174  0.47 14.32           H  0.037
ATOM   1127 HH12AARG A  68      13.629 -18.388  -5.474  0.47 14.32           H  0.037
ATOM   1128 HH21AARG A  68      15.715 -16.021  -4.582  0.47 12.98           H  0.035
ATOM   1129 HH22AARG A  68      15.311 -16.885  -5.726  0.47 12.98           H  0.035
ATOM   1130  N  BARG A  68      12.226 -15.297   1.916  0.53 16.13           N  0.039
ANISOU 1130  N  BARG A  68     2211   1297   2622   -524    291   -121       N
ATOM   1131  CA BARG A  68      12.938 -16.566   1.807  0.53 16.62           C  0.039
ANISOU 1131  CA BARG A  68     2282   1261   2771   -493     97   -100       C
ATOM   1132  C  BARG A  68      14.412 -16.457   2.182  0.53 16.74           C  0.040
ANISOU 1132  C  BARG A  68     2353   1298   2711   -476    -39   -309       C
ATOM   1133  O  BARG A  68      15.177 -17.377   1.879  0.53 18.66           O  0.042
ANISOU 1133  O  BARG A  68     2474   1439   3176   -348   -270   -223       O
ATOM   1134  CB BARG A  68      12.779 -17.186   0.413  0.53 16.76           C  0.040
ANISOU 1134  CB BARG A  68     2225   1228   2915   -476    263     80       C
ATOM   1135  CG BARG A  68      13.060 -16.227  -0.730  0.53 16.35           C  0.039
ANISOU 1135  CG BARG A  68     2213   1161   2839   -458    416    231       C
ATOM   1136  CD BARG A  68      13.455 -16.942  -2.020  0.53 16.23           C  0.039
ANISOU 1136  CD BARG A  68     2266   1133   2769   -441    425    392       C
ATOM   1137  NE BARG A  68      14.830 -17.416  -1.947  0.53 17.51           N  0.040
ANISOU 1137  NE BARG A  68     2274   1173   3206   -408    437    343       N
ATOM   1138  CZ BARG A  68      15.192 -18.685  -1.789  0.53 18.97           C  0.042
ANISOU 1138  CZ BARG A  68     2225   1235   3749   -340    459     45       C
ATOM   1139  NH1BARG A  68      14.331 -19.672  -1.951  0.53 19.02           N  0.042
ANISOU 1139  NH1BARG A  68     2273   1152   3801   -306    668    -16       N
ATOM   1140  NH2BARG A  68      16.464 -18.968  -1.485  0.53 19.48           N  0.043
ANISOU 1140  NH2BARG A  68     2186   1320   3896   -288    511   -148       N
ATOM   1141  H  BARG A  68      12.605 -14.641   1.509  0.53 19.51           H  0.043
```

```
ATOM   1142  HA BARG A  68      12.541 -17.190   2.434  0.53 20.09           H  0.043
ATOM   1143  HB2BARG A  68      13.392 -17.933   0.330  0.53 20.26           H  0.044
ATOM   1144  HB3BARG A  68      11.866 -17.500   0.316  0.53 20.26           H  0.044
ATOM   1145  HG2BARG A  68      12.262 -15.706  -0.909  0.53 19.77           H  0.043
ATOM   1146  HG3BARG A  68      13.790 -15.640  -0.477  0.53 19.77           H  0.043
ATOM   1147  HD2BARG A  68      12.857 -17.689  -2.166  0.53 19.63           H  0.043
ATOM   1148  HD3BARG A  68      13.392 -16.317  -2.759  0.53 19.63           H  0.043
ATOM   1149  HE BARG A  68      15.456 -16.830  -2.011  0.53 21.16           H  0.045
ATOM   1150 HH11BARG A  68      13.510 -19.510  -2.141  0.53 22.97           H  0.046
ATOM   1151 HH12BARG A  68      14.592 -20.485  -1.852  0.53 22.97           H  0.046
ATOM   1152 HH21BARG A  68      17.035 -18.332  -1.388  0.53 23.53           H  0.047
ATOM   1153 HH22BARG A  68      16.714 -19.786  -1.399  0.53 23.53           H  0.047
ATOM   1154  N   THR A  69      14.827 -15.360   2.836  1.00 15.36           N  0.038
ANISOU 1154  N   THR A  69     2320   1284   2233   -591   -100   -422       N
ATOM   1155  CA  THR A  69      16.220 -15.117   3.216  1.00 14.70           C  0.037
ANISOU 1155  CA  THR A  69     2491   1520   1574   -525   -124   -170       C
ATOM   1156  C   THR A  69      16.301 -15.026   4.735  1.00 16.32           C  0.039
ANISOU 1156  C   THR A  69     2832   1777   1591   -541     82   -125       C
ATOM   1157  O   THR A  69      16.198 -13.939   5.310  1.00 18.18           O  0.041
ANISOU 1157  O   THR A  69     3158   1869   1882   -607    242    137       O
ATOM   1158  CB  THR A  69      16.732 -13.835   2.551  1.00 14.36           C  0.037
ANISOU 1158  CB  THR A  69     2327   1419   1708   -509    252     99       C
ATOM   1159  OG1 THR A  69      16.444 -13.863   1.138  1.00 13.53           O  0.036
ANISOU 1159  OG1 THR A  69     2148   1516   1478   -382    299    -42       O
ATOM   1160  CG2 THR A  69      18.224 -13.678   2.761  1.00 16.13           C  0.039
ANISOU 1160  CG2 THR A  69     2392   1485   2251   -678    329    200       C
ATOM   1161  HA  THR A  69      16.778 -15.849   2.913  1.00 17.79           H  0.041
ATOM   1162  HB  THR A  69      16.289 -13.070   2.950  1.00 17.38           H  0.040
ATOM   1163  HG1 THR A  69      15.614 -13.891   1.014  1.00 16.39           H  0.039
ATOM   1164 HG21 THR A  69      18.528 -12.847   2.365  1.00 19.51           H  0.043
ATOM   1165 HG22 THR A  69      18.429 -13.668   3.709  1.00 19.51           H  0.043
ATOM   1166 HG23 THR A  69      18.696 -14.416   2.345  1.00 19.51           H  0.043
ATOM   1167  H  ATHR A  69      14.282 -14.776   3.154  0.47 18.59           H  0.042
ATOM   1168  H  BTHR A  69      14.299 -14.725   3.076  0.53 18.59           H  0.042
ATOM   1169  N   PRO A  70      16.465 -16.145   5.429  1.00 18.54           N  0.042
ANISOU 1169  N   PRO A  70     3005   2150   1888   -454    -28    -29       N
ATOM   1170  CA  PRO A  70      16.330 -16.132   6.889  1.00 20.37           C  0.044
ANISOU 1170  CA  PRO A  70     3086   2530   2123   -416    -19     79       C
ATOM   1171  C   PRO A  70      17.338 -15.211   7.543  1.00 22.00           C  0.045
ANISOU 1171  C   PRO A  70     3141   2976   2240   -525    -99    -89       C
ATOM   1172  O   PRO A  70      18.458 -15.049   7.059  1.00 23.46           O  0.047
ANISOU 1172  O   PRO A  70     3282   3332   2300   -345    -23   -272       O
ATOM   1173  CB  PRO A  70      16.627 -17.582   7.281  1.00 21.48           C  0.045
ANISOU 1173  CB  PRO A  70     3215   2473   2473   -364    -11    136       C
ATOM   1174  CG  PRO A  70      16.454 -18.389   6.044  1.00 19.44           C  0.043
ANISOU 1174  CG  PRO A  70     3231   2337   1817   -339    188    157       C
ATOM   1175  CD  PRO A  70      16.257 -17.485   4.867  1.00 19.03           C  0.042
ANISOU 1175  CD  PRO A  70     3200   2206   1825   -426    338    129       C
ATOM   1176  HA  PRO A  70      15.429 -15.890   7.157  1.00 24.59           H  0.048
ATOM   1177  HB2 PRO A  70      17.542 -17.646   7.598  1.00 25.93           H  0.049
ATOM   1178  HB3 PRO A  70      16.015 -17.875   7.967  1.00 25.93           H  0.049
ATOM   1179  HG2 PRO A  70      17.247 -18.932   5.912  1.00 23.47           H  0.047
ATOM   1180  HG3 PRO A  70      15.679 -18.962   6.151  1.00 23.47           H  0.047
ATOM   1181  HD2 PRO A  70      16.919 -17.675   4.184  1.00 22.99           H  0.046
ATOM   1182  HD3 PRO A  70      15.355 -17.572   4.523  1.00 22.99           H  0.046
ATOM   1183  N   GLY A  71      16.924 -14.603   8.653  1.00 23.70           N  0.047
ANISOU 1183  N   GLY A  71     3254   3038   2714   -778    -34   -521       N
ATOM   1184  CA  GLY A  71      17.771 -13.719   9.421  1.00 25.73           C  0.049
ANISOU 1184  CA  GLY A  71     3334   3122   3321  -1001     -0   -572       C
ATOM   1185  C   GLY A  71      18.102 -12.430   8.731  1.00 25.85           C  0.049
ANISOU 1185  C   GLY A  71     3360   3049   3414  -1285    139   -710       C
ATOM   1186  O   GLY A  71      18.968 -11.689   9.204  1.00 26.73           O  0.050
ANISOU 1186  O   GLY A  71     3432   3247   3478  -1286   -261  -1121       O
ATOM   1187  H   GLY A  71      16.135 -14.695   8.983  1.00 28.59           H  0.052
ATOM   1188  HA2 GLY A  71      17.334 -13.509  10.260  1.00 31.03           H  0.054
ATOM   1189  HA3 GLY A  71      18.606 -14.170   9.619  1.00 31.03           H  0.054
ATOM   1190  N   SER A  72      17.456 -12.147   7.611  1.00 25.28           N  0.049
ANISOU 1190  N   SER A  72     3309   2871   3426  -1510    530   -444       N
ATOM   1191  CA  SER A  72      17.790 -10.978   6.824  1.00 25.63           C  0.049
ANISOU 1191  CA  SER A  72     3364   2834   3541  -1649    561   -247       C
ATOM   1192  C   SER A  72      17.108  -9.737   7.374  1.00 26.10           C  0.049
ANISOU 1192  C   SER A  72     3388   2779   3749  -1716    569   -412       C
ATOM   1193  O   SER A  72      16.143  -9.796   8.146  1.00 27.01           O  0.050
ANISOU 1193  O   SER A  72     3532   2819   3913  -1819    487   -344       O
ATOM   1194  CB  SER A  72      17.349 -11.175   5.376  1.00 25.69           C  0.049
ANISOU 1194  CB  SER A  72     3300   2840   3621  -1716    461    357       C
ATOM   1195  OG  SER A  72      15.928 -11.062   5.271  1.00 26.83           O  0.050
ANISOU 1195  OG  SER A  72     3278   2890   4027  -1704    243    644       O
```

```
ATOM   1196  H   SER A  72      16.816 -12.619   7.284  1.00 30.49           H  0.053
ATOM   1197  HA  SER A  72      18.750 -10.837   6.840  1.00 30.91           H  0.054
ATOM   1198  HB2 SER A  72      17.761 -10.492   4.825  1.00 30.98           H  0.054
ATOM   1199  HB3 SER A  72      17.625 -12.054   5.076  1.00 30.98           H  0.054
ATOM   1200  N   ARG A  73      17.632  -8.603   6.954  1.00 24.90           N  0.048
ANISOU 1200  N   ARG A  73     3271   2756   3435  -1596    244   -638       N
ATOM   1201  CA  ARG A  73      16.983  -7.328   7.150  1.00 25.33           C  0.049
ANISOU 1201  CA  ARG A  73     3175   2736   3711  -1361    223   -573       C
ATOM   1202  C   ARG A  73      16.287  -6.912   5.859  1.00 25.41           C  0.049
ANISOU 1202  C   ARG A  73     2891   2605   4157  -1218    319   -651       C
ATOM   1203  O   ARG A  73      16.336  -7.605   4.825  1.00 27.09           O  0.050
ANISOU 1203  O   ARG A  73     2861   2734   4698  -1082    261   -541       O
ATOM   1204  CB  ARG A  73      18.018  -6.311   7.610  1.00 26.44           C  0.050
ANISOU 1204  CB  ARG A  73     3378   2817   3850  -1333    114   -827       C
ATOM   1205  CG  ARG A  73      18.796  -6.807   8.809  1.00 28.33           C  0.051
ANISOU 1205  CG  ARG A  73     3584   2974   4206  -1261    147  -1041       C
ATOM   1206  CD  ARG A  73      17.964  -6.772  10.090  1.00 29.94           C  0.053
ANISOU 1206  CD  ARG A  73     3759   3053   4565  -1265    159  -1118       C
ATOM   1207  NE  ARG A  73      17.873  -5.425  10.654  1.00 31.26           N  0.054
ANISOU 1207  NE  ARG A  73     3931   3197   4749  -1205     73  -1376       N
ATOM   1208  CZ  ARG A  73      18.862  -4.785  11.269  1.00 32.30           C  0.055
ANISOU 1208  CZ  ARG A  73     4042   3218   5011  -1176      7  -1613       C
ATOM   1209  NH1 ARG A  73      20.054  -5.340  11.435  1.00 32.88           N  0.055
ANISOU 1209  NH1 ARG A  73     4131   3233   5129  -1146     68  -1774       N
ATOM   1210  NH2 ARG A  73      18.644  -3.564  11.753  1.00 32.86           N  0.055
ANISOU 1210  NH2 ARG A  73     4046   3239   5198  -1259   -109  -1539       N
ATOM   1211  H   ARG A  73      18.384  -8.546   6.541  1.00 30.03           H  0.053
ATOM   1212  HA  ARG A  73      16.309  -7.411   7.841  1.00 30.54           H  0.053
ATOM   1213  HB2 ARG A  73      18.645  -6.145   6.889  1.00 31.87           H  0.055
ATOM   1214  HB3 ARG A  73      17.569  -5.488   7.859  1.00 31.87           H  0.055
ATOM   1215  HG2 ARG A  73      19.091  -7.719   8.663  1.00 34.15           H  0.057
ATOM   1216  HG3 ARG A  73      19.565  -6.231   8.940  1.00 34.15           H  0.057
ATOM   1217  HD2 ARG A  73      17.065  -7.080   9.899  1.00 36.08           H  0.058
ATOM   1218  HD3 ARG A  73      18.378  -7.347  10.752  1.00 36.08           H  0.058
ATOM   1219  HE  ARG A  73      17.120  -5.015  10.582  1.00 37.66           H  0.059
ATOM   1220 HH11 ARG A  73      20.209  -6.129  11.131  1.00 39.61           H  0.061
ATOM   1221 HH12 ARG A  73      20.677  -4.906  11.839  1.00 39.61           H  0.061
ATOM   1222 HH21 ARG A  73      17.874  -3.194  11.655  1.00 39.58           H  0.061
ATOM   1223 HH22 ARG A  73      19.276  -3.142  12.155  1.00 39.58           H  0.061
ATOM   1224  N   ASN A  74      15.592  -5.776   5.955  1.00 22.48           N  0.046
ANISOU 1224  N   ASN A  74     2605   2256   3681  -1116    497   -675       N
ATOM   1225  CA  ASN A  74      14.814  -5.149   4.889  1.00 20.04           C  0.043
ANISOU 1225  CA  ASN A  74     2509   2151   2956   -903    434   -758       C
ATOM   1226  C   ASN A  74      14.991  -3.646   5.107  1.00 19.36           C  0.043
ANISOU 1226  C   ASN A  74     2497   1977   2881   -874    524   -438       C
ATOM   1227  O   ASN A  74      14.051  -2.932   5.463  1.00 18.33           O  0.041
ANISOU 1227  O   ASN A  74     2510   1743   2711   -913    635   -469       O
ATOM   1228  CB  ASN A  74      13.345  -5.537   5.005  1.00 20.05           C  0.043
ANISOU 1228  CB  ASN A  74     2422   2215   2982   -840    505   -811       C
ATOM   1229  CG  ASN A  74      12.461  -4.857   3.973  1.00 19.87           C  0.043
ANISOU 1229  CG  ASN A  74     2410   2252   2890   -808    531   -744       C
ATOM   1230  OD1 ASN A  74      12.915  -4.277   3.010  1.00 18.99           O  0.042
ANISOU 1230  OD1 ASN A  74     2114   2191   2908   -791    743   -615       O
ATOM   1231  ND2 ASN A  74      11.165  -4.931   4.197  1.00 20.20           N  0.043
ANISOU 1231  ND2 ASN A  74     2493   2306   2876   -912    435   -561       N
ATOM   1232  H   ASN A  74      15.558  -5.322   6.684  1.00 27.13           H  0.050
ATOM   1233  HA  ASN A  74      15.156  -5.400   4.016  1.00 24.20           H  0.048
ATOM   1234  HB2 ASN A  74      13.263  -6.496   4.883  1.00 24.21           H  0.048
ATOM   1235  HB3 ASN A  74      13.022  -5.287   5.885  1.00 24.21           H  0.048
ATOM   1236 HD21 ASN A  74      10.612  -4.565   3.649  1.00 24.39           H  0.048
ATOM   1237 HD22 ASN A  74      10.871  -5.348   4.890  1.00 24.39           H  0.048
ATOM   1238  N   LEU A  75      16.229  -3.173   4.939  1.00 18.45           N  0.042
ANISOU 1238  N   LEU A  75     2435   1933   2642   -825    509   -545       N
ATOM   1239  CA  LEU A  75      16.579  -1.825   5.341  1.00 18.76           C  0.042
ANISOU 1239  CA  LEU A  75     2510   2010   2605   -734    215   -627       C
ATOM   1240  C   LEU A  75      15.990  -0.784   4.397  1.00 18.25           C  0.041
ANISOU 1240  C   LEU A  75     2405   1923   2605   -660    302   -721       C
ATOM   1241  O   LEU A  75      15.867   0.378   4.782  1.00 20.01           O  0.043
ANISOU 1241  O   LEU A  75     2568   1937   3099   -575    307   -792       O
ATOM   1242  CB  LEU A  75      18.102  -1.708   5.455  1.00 20.99           C  0.044
ANISOU 1242  CB  LEU A  75     2603   2117   3255   -695    245   -573       C
ATOM   1243  CG  LEU A  75      18.731  -2.412   6.678  1.00 21.85           C  0.045
ANISOU 1243  CG  LEU A  75     2728   2251   3322   -577    377   -580       C
ATOM   1244  CD1 LEU A  75      20.261  -2.345   6.695  1.00 22.64           C  0.046
ANISOU 1244  CD1 LEU A  75     2785   2330   3486   -467    108   -435       C
ATOM   1245  CD2 LEU A  75      18.183  -1.810   7.969  1.00 21.99           C  0.045
ANISOU 1245  CD2 LEU A  75     2805   2318   3233   -548    364   -676       C
ATOM   1246  H   LEU A  75      16.879  -3.619   4.594  1.00 22.29           H  0.046
```

```
ATOM   1247  HA  LEU A  75      16.200  -1.660   6.217  1.00 22.66           H  0.046
ATOM   1248  HB2 LEU A  75      18.500  -2.096   4.660  1.00 25.34           H  0.049
ATOM   1249  HB3 LEU A  75      18.335  -0.768   5.508  1.00 25.34           H  0.049
ATOM   1250  HG  LEU A  75      18.478  -3.348   6.660  1.00 26.37           H  0.050
ATOM   1251 HD11 LEU A  75      20.610  -3.187   7.025  1.00 27.31           H  0.051
ATOM   1252 HD12 LEU A  75      20.580  -2.184   5.793  1.00 27.31           H  0.051
ATOM   1253 HD13 LEU A  75      20.535  -1.621   7.278  1.00 27.31           H  0.051
ATOM   1254 HD21 LEU A  75      18.747  -2.084   8.709  1.00 26.54           H  0.050
ATOM   1255 HD22 LEU A  75      18.186  -0.843   7.892  1.00 26.54           H  0.050
ATOM   1256 HD23 LEU A  75      17.277  -2.128   8.108  1.00 26.54           H  0.050
ATOM   1257  N   CYS A  76      15.602  -1.176   3.181  1.00 18.45           N  0.042
ANISOU 1257  N   CYS A  76     2174   1961   2875   -605    201   -739       N
ATOM   1258  CA  CYS A  76      14.877  -0.262   2.299  1.00 18.90           C  0.042
ANISOU 1258  CA  CYS A  76     2067   1936   3176   -641    243   -521       C
ATOM   1259  C   CYS A  76      13.362  -0.333   2.483  1.00 18.65           C  0.042
ANISOU 1259  C   CYS A  76     2106   1971   3008   -603    581   -560       C
ATOM   1260  O   CYS A  76      12.629   0.403   1.806  1.00 18.53           O  0.042
ANISOU 1260  O   CYS A  76     2119   1980   2940   -649    609   -382       O
ATOM   1261  CB  CYS A  76      15.253  -0.519   0.837  1.00 17.53           C  0.041
ANISOU 1261  CB  CYS A  76     1850   1832   2979   -568    124   -366       C
ATOM   1262  SG  CYS A  76      16.984  -0.098   0.536  1.00 16.80           S  0.040
ANISOU 1262  SG  CYS A  76     1664   1746   2973   -420    122   -300       S
ATOM   1263  H   CYS A  76      15.745  -1.956   2.847  1.00 22.29           H  0.046
ATOM   1264  HA  CYS A  76      15.149   0.645   2.508  1.00 22.82           H  0.046
ATOM   1265  HB2 CYS A  76      15.118  -1.453   0.622  1.00 21.19           H  0.045
ATOM   1266  HB3 CYS A  76      14.703   0.038   0.264  1.00 21.19           H  0.045
ATOM   1267  N   ASN A  77      12.881  -1.186   3.386  1.00 19.16           N  0.042
ANISOU 1267  N   ASN A  77     2123   1961   3197   -489    986   -564       N
ATOM   1268  CA  ASN A  77      11.456  -1.320   3.673  1.00 20.40           C  0.044
ANISOU 1268  CA  ASN A  77     2077   2044   3630   -327   1163   -660       C
ATOM   1269  C   ASN A  77      10.613  -1.489   2.412  1.00 19.58           C  0.043
ANISOU 1269  C   ASN A  77     1663   1916   3859   -248   1004   -657       C
ATOM   1270  O   ASN A  77       9.642  -0.768   2.185  1.00 20.60           O  0.044
ANISOU 1270  O   ASN A  77     1654   2181   3991   -136    759   -616       O
ATOM   1271  CB  ASN A  77      10.945  -0.183   4.559  1.00 24.34           C  0.048
ANISOU 1271  CB  ASN A  77     2466   2408   4376   -333   1150   -712       C
ATOM   1272  CG  ASN A  77      11.803   0.012   5.775  1.00 27.89           C  0.051
ANISOU 1272  CG  ASN A  77     2782   2797   5018   -430   1221   -636       C
ATOM   1273  OD1 ASN A  77      11.570  -0.628   6.799  1.00 29.72           O  0.053
ANISOU 1273  OD1 ASN A  77     3050   3056   5184   -299   1123   -543       O
ATOM   1274  ND2 ASN A  77      12.781   0.903   5.692  1.00 29.66           N  0.053
ANISOU 1274  ND2 ASN A  77     2831   2933   5506   -502   1171   -749       N
ATOM   1275  H   ASN A  77      13.373  -1.711   3.857  1.00 23.14           H  0.047
ATOM   1276  HA  ASN A  77      11.342  -2.135   4.186  1.00 24.63           H  0.048
ATOM   1277  HB2 ASN A  77      10.949   0.643   4.051  1.00 29.36           H  0.052
ATOM   1278  HB3 ASN A  77      10.044  -0.390   4.854  1.00 29.36           H  0.052
ATOM   1279 HD21 ASN A  77      13.293   1.044   6.369  1.00 35.74           H  0.058
ATOM   1280 HD22 ASN A  77      12.909   1.334   4.959  1.00 35.74           H  0.058
ATOM   1281  N   ILE A  78      11.009  -2.447   1.578  1.00 19.03           N  0.042
ANISOU 1281  N   ILE A  78     1587   1767   3875   -345    667   -625       N
ATOM   1282  CA  ILE A  78      10.280  -2.733   0.346  1.00 19.56           C  0.043
ANISOU 1282  CA  ILE A  78     1704   1634   4094   -332    514   -652       C
ATOM   1283  C   ILE A  78      10.305  -4.221   0.096  1.00 18.46           C  0.042
ANISOU 1283  C   ILE A  78     1500   1484   4029   -219    286   -653       C
ATOM   1284  O   ILE A  78      11.216  -4.934   0.528  1.00 17.45           O  0.040
ANISOU 1284  O   ILE A  78     1299   1470   3862    -54    628   -817       O
ATOM   1285  CB  ILE A  78      10.879  -2.017  -0.878  1.00 22.00           C  0.045
ANISOU 1285  CB  ILE A  78     1978   1820   4560   -397    631   -557       C
ATOM   1286  CG1 ILE A  78      12.367  -2.398  -0.996  1.00 23.90           C  0.047
ANISOU 1286  CG1 ILE A  78     2117   1979   4985   -360    701   -369       C
ATOM   1287  CG2 ILE A  78      10.461  -0.541  -0.904  1.00 23.19           C  0.047
ANISOU 1287  CG2 ILE A  78     2109   1902   4802   -438    658   -381       C
ATOM   1288  CD1 ILE A  78      13.029  -1.834  -2.177  1.00 25.09           C  0.048
ANISOU 1288  CD1 ILE A  78     2207   2056   5270   -413    762   -315       C
ATOM   1289  H   ILE A  78      11.698  -2.946   1.703  1.00 22.98           H  0.046
ATOM   1290  HA  ILE A  78       9.365  -2.436   0.458  1.00 23.62           H  0.047
ATOM   1291  HB  ILE A  78      10.451  -2.416  -1.651  1.00 26.55           H  0.050
ATOM   1292 HG12 ILE A  78      12.837  -2.073  -0.212  1.00 28.83           H  0.052
ATOM   1293 HG13 ILE A  78      12.451  -3.362  -1.053  1.00 28.83           H  0.052
ATOM   1294 HG21 ILE A  78      10.832  -0.122  -1.696  1.00 27.98           H  0.051
ATOM   1295 HG22 ILE A  78       9.493  -0.488  -0.924  1.00 27.98           H  0.051
ATOM   1296 HG23 ILE A  78      10.800  -0.103  -0.108  1.00 27.98           H  0.051
ATOM   1297 HD11 ILE A  78      13.944  -2.155  -2.208  1.00 30.26           H  0.053
ATOM   1298 HD12 ILE A  78      12.551  -2.118  -2.972  1.00 30.26           H  0.053
ATOM   1299 HD13 ILE A  78      13.019  -0.867  -2.112  1.00 30.26           H  0.053
ATOM   1300  N   PRO A  79       9.282  -4.736  -0.554  1.00 16.74           N  0.040
ANISOU 1300  N   PRO A  79     1358   1351   3652   -133    338   -545       N
ATOM   1301  CA  PRO A  79       9.386  -6.095  -1.089  1.00 15.63           C  0.038
```

```
ANISOU 1301  CA  PRO A  79     1398   1327   3212   -196    385   -508       C
ATOM   1302  C   PRO A  79      10.385  -6.103  -2.234  1.00 15.11          C  0.038
ANISOU 1302  C   PRO A  79     1343   1445   2952   -283     68   -200       C
ATOM   1303  O   PRO A  79      10.457  -5.161  -3.030  1.00 16.09          O  0.039
ANISOU 1303  O   PRO A  79     1548   1661   2905    -91     79    -36       O
ATOM   1304  CB  PRO A  79       7.968  -6.394  -1.584  1.00 17.07          C  0.040
ANISOU 1304  CB  PRO A  79     1459   1325   3701   -258    249   -517       C
ATOM   1305  CG  PRO A  79       7.413  -5.020  -1.846  1.00 18.31          C  0.041
ANISOU 1305  CG  PRO A  79     1435   1477   4044   -214    400   -618       C
ATOM   1306  CD  PRO A  79       8.034  -4.049  -0.912  1.00 18.50          C  0.042
ANISOU 1306  CD  PRO A  79     1456   1494   4077   -168    288   -590       C
ATOM   1307  HA  PRO A  79       9.647  -6.728  -0.402  1.00 18.90          H  0.042
ATOM   1308  HB2 PRO A  79       8.003  -6.919  -2.399  1.00 20.63          H  0.044
ATOM   1309  HB3 PRO A  79       7.462  -6.850  -0.893  1.00 20.63          H  0.044
ATOM   1310  HG2 PRO A  79       7.613  -4.770  -2.762  1.00 22.12          H  0.046
ATOM   1311  HG3 PRO A  79       6.453  -5.039  -1.711  1.00 22.12          H  0.046
ATOM   1312  HD2 PRO A  79       8.218  -3.212  -1.366  1.00 22.34          H  0.046
ATOM   1313  HD3 PRO A  79       7.476  -3.921  -0.129  1.00 22.34          H  0.046
ATOM   1314  N   CYS A  80      11.191  -7.167  -2.288  1.00 14.18          N  0.036
ANISOU 1314  N   CYS A  80     1203   1277   2909   -440     91    200       N
ATOM   1315  CA  CYS A  80      12.193  -7.249  -3.344  1.00 13.48          C  0.036
ANISOU 1315  CA  CYS A  80     1089   1291   2744   -469    182     87       C
ATOM   1316  C   CYS A  80      11.549  -7.200  -4.731  1.00 13.15          C  0.035
ANISOU 1316  C   CYS A  80     1079   1292   2626   -411     52    -67       C
ATOM   1317  O   CYS A  80      12.176  -6.723  -5.689  1.00 13.52          O  0.036
ANISOU 1317  O   CYS A  80     1191   1218   2728   -426    211    -89       O
ATOM   1318  CB  CYS A  80      12.993  -8.519  -3.180  1.00 13.91          C  0.036
ANISOU 1318  CB  CYS A  80     1013   1494   2776   -460    306   -163       C
ATOM   1319  SG  CYS A  80      13.967  -8.666  -1.669  1.00 14.24          S  0.037
ANISOU 1319  SG  CYS A  80     1343   1497   2570   -436     83   -226       S
ATOM   1320  H   CYS A  80      11.178  -7.832  -1.743  1.00 17.17          H  0.040
ATOM   1321  HA  CYS A  80      12.800  -6.497  -3.266  1.00 16.33          H  0.039
ATOM   1322  HB2 CYS A  80      12.379  -9.270  -3.201  1.00 16.84          H  0.040
ATOM   1323  HB3 CYS A  80      13.604  -8.584  -3.928  1.00 16.84          H  0.040
ATOM   1324  N   SER A  81      10.309  -7.677  -4.861  1.00 15.44          N  0.038
ANISOU 1324  N   SER A  81     1190   1543   3135   -503     92     94       N
ATOM   1325  CA  SER A  81       9.642  -7.586  -6.150  1.00 16.14          C  0.039
ANISOU 1325  CA  SER A  81     1231   1824   3078   -456    -50    -23       C
ATOM   1326  C   SER A  81       9.520  -6.145  -6.644  1.00 17.41          C  0.040
ANISOU 1326  C   SER A  81     1382   1808   3424   -355    -12    248       C
ATOM   1327  O   SER A  81       9.477  -5.921  -7.864  1.00 18.03          O  0.041
ANISOU 1327  O   SER A  81     1488   1967   3395   -204   -113    381       O
ATOM   1328  CB  SER A  81       8.282  -8.262  -6.048  1.00 17.78          C  0.041
ANISOU 1328  CB  SER A  81     1209   2170   3376   -380   -202   -400       C
ATOM   1329  OG  SER A  81       7.514  -7.551  -5.092  1.00 19.21          O  0.042
ANISOU 1329  OG  SER A  81     1192   2419   3689   -358   -199   -475       O
ATOM   1330  H   SER A  81       9.831  -7.988  -4.235  1.00 18.68          H  0.042
ATOM   1331  HA  SER A  81      10.165  -8.076  -6.804  1.00 19.52          H  0.043
ATOM   1332  HB2 SER A  81       7.838  -8.230  -6.910  1.00 21.48          H  0.045
ATOM   1333  HB3 SER A  81       8.395  -9.180  -5.757  1.00 21.48          H  0.045
ATOM   1334  HG  SER A  81       6.755  -7.903  -5.014  1.00 23.21          H  0.047
ATOM   1335  N   ALA A  82       9.461  -5.156  -5.732  1.00 18.02          N  0.041
ANISOU 1335  N   ALA A  82     1493   1719   3636   -261    179    234       N
ATOM   1336  CA  ALA A  82       9.384  -3.749  -6.132  1.00 18.37          C  0.041
ANISOU 1336  CA  ALA A  82     1537   1601   3843   -337    391    206       C
ATOM   1337  C   ALA A  82      10.590  -3.331  -6.943  1.00 18.49          C  0.042
ANISOU 1337  C   ALA A  82     1590   1524   3910   -162    304    131       C
ATOM   1338  O   ALA A  82      10.496  -2.383  -7.737  1.00 18.80          O  0.042
ANISOU 1338  O   ALA A  82     1611   1531   4002    152    226    104       O
ATOM   1339  CB  ALA A  82       9.274  -2.846  -4.907  1.00 18.66          C  0.042
ANISOU 1339  CB  ALA A  82     1570   1730   3790   -519    502    383       C
ATOM   1340  H   ALA A  82       9.464  -5.279  -4.881  1.00 21.78          H  0.045
ATOM   1341  HA  ALA A  82       8.593  -3.617  -6.677  1.00 22.20          H  0.046
ATOM   1342  HB1 ALA A  82       9.224  -1.922  -5.199  1.00 22.54          H  0.046
ATOM   1343  HB2 ALA A  82       8.473  -3.081  -4.413  1.00 22.54          H  0.046
ATOM   1344  HB3 ALA A  82      10.057  -2.975  -4.349  1.00 22.54          H  0.046
ATOM   1345  N   LEU A  83      11.717  -4.012  -6.746  1.00 18.01          N  0.041
ANISOU 1345  N   LEU A  83     1505   1433   3907   -202    258    142       N
ATOM   1346  CA  LEU A  83      12.954  -3.722  -7.448  1.00 18.41          C  0.042
ANISOU 1346  CA  LEU A  83     1489   1490   4017   -328     96    297       C
ATOM   1347  C   LEU A  83      12.960  -4.250  -8.876  1.00 17.54          C  0.041
ANISOU 1347  C   LEU A  83     1353   1617   3696   -523    -91    303       C
ATOM   1348  O   LEU A  83      13.966  -4.058  -9.573  1.00 17.62          O  0.041
ANISOU 1348  O   LEU A  83     1444   1695   3557   -661    -75    169       O
ATOM   1349  CB  LEU A  83      14.159  -4.301  -6.676  1.00 18.81          C  0.042
ANISOU 1349  CB  LEU A  83     1603   1472   4074   -335    133    274       C
ATOM   1350  CG  LEU A  83      14.345  -3.790  -5.236  1.00 19.96          C  0.043
ANISOU 1350  CG  LEU A  83     1814   1525   4244   -354    -11    488       C
```

```
ATOM   1351  CD1 LEU A  83      15.388  -4.564  -4.485  1.00 19.51           C  0.043
ANISOU 1351  CD1 LEU A  83     1816   1584   4014   -419   -187    577       C
ATOM   1352  CD2 LEU A  83      14.736  -2.308  -5.245  1.00 20.82           C  0.044
ANISOU 1352  CD2 LEU A  83     1925   1565   4421   -418     63    457       C
ATOM   1353  H   LEU A  83      11.788  -4.666  -6.192  1.00 21.77           H  0.045
ATOM   1354  HA  LEU A  83      13.066  -2.760  -7.492  1.00 22.25           H  0.046
ATOM   1355  HB2 LEU A  83      14.058  -5.265  -6.630  1.00 22.73           H  0.046
ATOM   1356  HB3 LEU A  83      14.968  -4.085  -7.166  1.00 22.73           H  0.046
ATOM   1357  HG  LEU A  83      13.505  -3.876  -4.758  1.00 24.10           H  0.047
ATOM   1358 HD11 LEU A  83      15.714  -4.025  -3.747  1.00 23.57           H  0.047
ATOM   1359 HD12 LEU A  83      14.991  -5.382  -4.148  1.00 23.57           H  0.047
ATOM   1360 HD13 LEU A  83      16.116  -4.774  -5.089  1.00 23.57           H  0.047
ATOM   1361 HD21 LEU A  83      14.860  -2.007  -4.331  1.00 25.14           H  0.049
ATOM   1362 HD22 LEU A  83      15.562  -2.204  -5.743  1.00 25.14           H  0.049
ATOM   1363 HD23 LEU A  83      14.028  -1.797  -5.667  1.00 25.14           H  0.049
ATOM   1364  N   LEU A  84      11.909  -4.960  -9.321  1.00 18.03           N  0.041
ANISOU 1364  N   LEU A  84     1433   1645   3773   -568   -201    292       N
ATOM   1365  CA  LEU A  84      11.862  -5.505 -10.678  1.00 17.75           C  0.041
ANISOU 1365  CA  LEU A  84     1360   1663   3722   -537    -85    332       C
ATOM   1366  C   LEU A  84      10.960  -4.692 -11.594  1.00 19.06           C  0.042
ANISOU 1366  C   LEU A  84     1348   1780   4115   -410    -90    211       C
ATOM   1367  O   LEU A  84      10.817  -5.026 -12.779  1.00 19.79           O  0.043
ANISOU 1367  O   LEU A  84     1497   1677   4345   -413   -198    -99       O
ATOM   1368  CB  LEU A  84      11.391  -6.962 -10.659  1.00 16.97           C  0.040
ANISOU 1368  CB  LEU A  84     1483   1611   3354   -666   -136    379       C
ATOM   1369  CG  LEU A  84      12.186  -7.944  -9.794  1.00 15.69           C  0.038
ANISOU 1369  CG  LEU A  84     1540   1615   2804   -770     86    434       C
ATOM   1370  CD1 LEU A  84      11.644  -9.329  -9.961  1.00 16.85           C  0.040
ANISOU 1370  CD1 LEU A  84     1703   1640   3058   -772     -2    555       C
ATOM   1371  CD2 LEU A  84      13.658  -7.952 -10.134  1.00 13.59           C  0.036
ANISOU 1371  CD2 LEU A  84     1506   1534   2124   -774    367    368       C
ATOM   1372  H   LEU A  84      11.211  -5.138  -8.851  1.00 21.79           H  0.045
ATOM   1373  HA  LEU A  84      12.754  -5.486 -11.046  1.00 21.45           H  0.045
ATOM   1374  HB2 LEU A  84      10.475  -6.978 -10.339  1.00 20.51           H  0.044
ATOM   1375  HB3 LEU A  84      11.417  -7.298 -11.568  1.00 20.51           H  0.044
ATOM   1376  HG  LEU A  84      12.093  -7.694  -8.861  1.00 18.97           H  0.042
ATOM   1377 HD11 LEU A  84      12.070  -9.915  -9.316  1.00 20.37           H  0.044
ATOM   1378 HD12 LEU A  84      10.686  -9.311  -9.811  1.00 20.37           H  0.044
ATOM   1379 HD13 LEU A  84      11.831  -9.634 -10.862  1.00 20.37           H  0.044
ATOM   1380 HD21 LEU A  84      14.099  -8.637  -9.607  1.00 16.46           H  0.039
ATOM   1381 HD22 LEU A  84      13.762  -8.143 -11.079  1.00 16.46           H  0.039
ATOM   1382 HD23 LEU A  84      14.035  -7.082  -9.928  1.00 16.46           H  0.039
ATOM   1383  N  ASER A  85      10.385  -3.608 -11.085  0.60 19.60           N  0.043
ANISOU 1383  N  ASER A  85     1197   1972   4278   -242    -62    335       N
ATOM   1384  CA ASER A  85       9.431  -2.839 -11.856  0.60 19.64           C  0.043
ANISOU 1384  CA ASER A  85     1169   2042   4251   -126   -165    405       C
ATOM   1385  C  ASER A  85      10.083  -2.196 -13.073  0.60 19.88           C  0.043
ANISOU 1385  C  ASER A  85     1341   2130   4084   -141   -244    403       C
ATOM   1386  O  ASER A  85      11.291  -1.935 -13.110  0.60 20.68           O  0.044
ANISOU 1386  O  ASER A  85     1489   2093   4276   -222   -240    596       O
ATOM   1387  CB ASER A  85       8.851  -1.719 -11.005  0.60 19.75           C  0.043
ANISOU 1387  CB ASER A  85     1086   2132   4286    -18     48    339       C
ATOM   1388  OG ASER A  85       7.956  -0.953 -11.795  0.60 20.17           O  0.043
ANISOU 1388  OG ASER A  85     1159   2115   4391    199    -84    361       O
ATOM   1389  H  ASER A  85      10.532  -3.298 -10.296  0.60 23.67           H  0.047
ATOM   1390  HA ASER A  85       8.708  -3.413 -12.154  0.60 23.72           H  0.047
ATOM   1391  HB2ASER A  85       8.370  -2.102 -10.255  0.60 23.85           H  0.047
ATOM   1392  HB3ASER A  85       9.570  -1.150 -10.690  0.60 23.85           H  0.047
ATOM   1393  HG ASER A  85       7.333  -1.440 -12.077  0.60 24.36           H  0.048
ATOM   1394  N  BSER A  85      10.372  -3.618 -11.086  0.40 19.17           N  0.042
ANISOU 1394  N  BSER A  85     1233   1929   4123   -370      3    284       N
ATOM   1395  CA BSER A  85       9.399  -2.845 -11.839  0.40 18.92           C  0.042
ANISOU 1395  CA BSER A  85     1203   1985   3999   -376     -9    337       C
ATOM   1396  C  BSER A  85      10.042  -2.113 -13.015  0.40 19.20           C  0.042
ANISOU 1396  C  BSER A  85     1318   2057   3919   -307   -143    345       C
ATOM   1397  O  BSER A  85      11.216  -1.732 -12.977  0.40 19.15           O  0.042
ANISOU 1397  O  BSER A  85     1393   1948   3936   -429    -95    444       O
ATOM   1398  CB BSER A  85       8.773  -1.825 -10.901  0.40 18.58           C  0.042
ANISOU 1398  CB BSER A  85     1185   2058   3815   -423    264    294       C
ATOM   1399  OG BSER A  85       9.795  -1.079 -10.271  0.40 18.43           O  0.042
ANISOU 1399  OG BSER A  85     1206   2032   3766   -438    288    327       O
ATOM   1400  H  BSER A  85      10.521  -3.311 -10.297  0.40 23.16           H  0.047
ATOM   1401  HA BSER A  85       8.703  -3.430 -12.176  0.40 22.85           H  0.046
ATOM   1402  HB2BSER A  85       8.207  -1.225 -11.411  0.40 22.44           H  0.046
ATOM   1403  HB3BSER A  85       8.252  -2.288 -10.226  0.40 22.44           H  0.046
ATOM   1404  HG BSER A  85      10.242  -0.660 -10.846  0.40 22.27           H  0.046
ATOM   1405  N   SER A  86       9.239  -1.890 -14.065  1.00 19.64           N  0.043
ANISOU 1405  N   SER A  86     1323   2176   3964   -248   -308    370       N
```

```
ATOM   1406  CA  SER A  86       9.684  -1.050 -15.175  1.00 22.70           C  0.046
ANISOU 1406  CA  SER A  86     1573   2467   4586   -138   -497    474       C
ATOM   1407  C   SER A  86       9.949   0.392 -14.732  1.00 22.08           C  0.045
ANISOU 1407  C   SER A  86     1485   2292   4613   -223   -514    703       C
ATOM   1408  O   SER A  86      10.821   1.072 -15.296  1.00 21.88           O  0.045
ANISOU 1408  O   SER A  86     1541   2160   4615   -440   -339   1142       O
ATOM   1409  CB  SER A  86       8.707  -1.217 -16.352  1.00 24.93           C  0.048
ANISOU 1409  CB  SER A  86     1842   2745   4886     98   -575    611       C
ATOM   1410  OG  SER A  86       7.599  -0.364 -16.242  1.00 27.96           O  0.051
ANISOU 1410  OG  SER A  86     2211   2983   5429    218   -479    399       O
ATOM   1411  HA  SER A  86      10.536  -1.400 -15.479  1.00 27.39           H  0.051
ATOM   1412  HB2 SER A  86       9.175  -1.015 -17.177  1.00 30.07           H  0.053
ATOM   1413  HB3 SER A  86       8.393  -2.135 -16.369  1.00 30.07           H  0.053
ATOM   1414  HG  SER A  86       7.084  -0.476 -16.896  1.00 33.70           H  0.056
ATOM   1415  H  ASER A  86       8.421  -2.151 -14.116  0.60 23.72           H  0.047
ATOM   1416  H  BSER A  86       8.446  -2.210 -14.154  0.40 23.72           H  0.047
ATOM   1417  N   ASP A  87       9.272   0.843 -13.680  1.00 21.36           N  0.045
ANISOU 1417  N   ASP A  87     1585   2185   4345   -177   -699    685       N
ATOM   1418  CA  ASP A  87       9.552   2.119 -13.046  1.00 21.65           C  0.045
ANISOU 1418  CA  ASP A  87     1687   2036   4503    -93   -806    894       C
ATOM   1419  C   ASP A  87      10.673   1.959 -12.025  1.00 21.52           C  0.045
ANISOU 1419  C   ASP A  87     1626   1983   4569   -198   -656    807       C
ATOM   1420  O   ASP A  87      10.590   1.097 -11.136  1.00 21.54           O  0.045
ANISOU 1420  O   ASP A  87     1548   1972   4665   -284   -490    857       O
ATOM   1421  CB  ASP A  87       8.287   2.604 -12.342  1.00 23.43           C  0.047
ANISOU 1421  CB  ASP A  87     1930   2177   4796    -52   -844    856       C
ATOM   1422  CG  ASP A  87       8.469   3.962 -11.737  1.00 26.11           C  0.049
ANISOU 1422  CG  ASP A  87     2169   2371   5379     43   -790    488       C
ATOM   1423  OD1 ASP A  87       9.052   4.049 -10.637  1.00 26.43           O  0.050
ANISOU 1423  OD1 ASP A  87     2181   2366   5495     83   -900    376       O
ATOM   1424  OD2 ASP A  87       8.125   4.941 -12.413  1.00 28.21           O  0.051
ANISOU 1424  OD2 ASP A  87     2331   2506   5882    -54   -598    357       O
ATOM   1425  H   ASP A  87       8.628   0.412 -13.308  1.00 25.78           H  0.049
ATOM   1426  HA  ASP A  87       9.817   2.773 -13.711  1.00 26.13           H  0.049
ATOM   1427  HB2 ASP A  87       7.563   2.654 -12.986  1.00 28.27           H  0.051
ATOM   1428  HB3 ASP A  87       8.060   1.984 -11.631  1.00 28.27           H  0.051
ATOM   1429  N   ILE A  88      11.709   2.793 -12.140  1.00 21.20           N  0.045
ANISOU 1429  N   ILE A  88     1524   2004   4528   -159   -503    816       N
ATOM   1430  CA  ILE A  88      12.939   2.580 -11.369  1.00 19.66           C  0.043
ANISOU 1430  CA  ILE A  88     1305   1975   4191    -55   -443    664       C
ATOM   1431  C   ILE A  88      12.921   3.228  -9.986  1.00 19.56           C  0.043
ANISOU 1431  C   ILE A  88     1203   1846   4382     -6   -423    578       C
ATOM   1432  O   ILE A  88      13.949   3.232  -9.295  1.00 17.97           O  0.041
ANISOU 1432  O   ILE A  88     1209   1735   3882     56   -620    507       O
ATOM   1433  CB  ILE A  88      14.207   2.981 -12.157  1.00 19.47           C  0.043
ANISOU 1433  CB  ILE A  88     1246   2031   4120     32   -375    588       C
ATOM   1434  CG1 ILE A  88      14.211   4.499 -12.372  1.00 20.33           C  0.044
ANISOU 1434  CG1 ILE A  88     1293   2096   4336    172   -208    603       C
ATOM   1435  CG2 ILE A  88      14.335   2.132 -13.449  1.00 20.23           C  0.044
ANISOU 1435  CG2 ILE A  88     1302   2286   4099      8   -264    513       C
ATOM   1436  CD1 ILE A  88      15.500   5.045 -12.979  1.00 20.65           C  0.044
ANISOU 1436  CD1 ILE A  88     1325   2110   4411     71    -46    410       C
ATOM   1437  H   ILE A  88      11.726   3.484 -12.652  1.00 25.59           H  0.049
ATOM   1438  HA  ILE A  88      13.013   1.626 -11.214  1.00 23.75           H  0.047
ATOM   1439  HB  ILE A  88      14.972   2.769 -11.599  1.00 23.51           H  0.047
ATOM   1440 HG12 ILE A  88      13.484   4.729 -12.971  1.00 24.55           H  0.048
ATOM   1441 HG13 ILE A  88      14.086   4.945 -11.521  1.00 24.55           H  0.048
ATOM   1442 HG21 ILE A  88      15.156   2.374 -13.906  1.00 24.43           H  0.048
ATOM   1443 HG22 ILE A  88      14.355   1.193 -13.208  1.00 24.43           H  0.048
ATOM   1444 HG23 ILE A  88      13.572   2.311 -14.021  1.00 24.43           H  0.048
ATOM   1445 HD11 ILE A  88      15.430   6.010 -13.053  1.00 24.93           H  0.048
ATOM   1446 HD12 ILE A  88      16.244   4.810 -12.403  1.00 24.93           H  0.048
ATOM   1447 HD13 ILE A  88      15.626   4.654 -13.858  1.00 24.93           H  0.048
ATOM   1448  N   THR A  89      11.775   3.786  -9.569  1.00 20.53           N  0.044
ANISOU 1448  N   THR A  89     1306   1825   4668     31   -350    325       N
ATOM   1449  CA  THR A  89      11.730   4.536  -8.308  1.00 20.53           C  0.044
ANISOU 1449  CA  THR A  89     1290   1862   4648     72      2     97       C
ATOM   1450  C   THR A  89      12.243   3.706  -7.144  1.00 18.75           C  0.042
ANISOU 1450  C   THR A  89     1112   1808   4206     83     11     13       C
ATOM   1451  O   THR A  89      13.060   4.172  -6.352  1.00 17.39           O  0.040
ANISOU 1451  O   THR A  89     1007   1714   3889     15   -278     73       O
ATOM   1452  CB  THR A  89      10.301   4.969  -7.989  1.00 22.85           C  0.046
ANISOU 1452  CB  THR A  89     1422   1962   5300    183    -18   -259       C
ATOM   1453  OG1 THR A  89       9.811   5.799  -9.041  1.00 25.31           O  0.049
ANISOU 1453  OG1 THR A  89     1625   2032   5960    270    -11    -49       O
ATOM   1454  CG2 THR A  89      10.261   5.763  -6.672  1.00 23.29           C  0.047
ANISOU 1454  CG2 THR A  89     1408   2018   5424    129   -113   -416       C
ATOM   1455  H   THR A  89      11.025   3.746  -9.989  1.00 24.78           H  0.048
```

```
ATOM   1456  HA  THR A  89      12.281   5.330  -8.385  1.00 24.79           H  0.048
ATOM   1457  HB  THR A  89       9.734   4.187  -7.898  1.00 27.57           H  0.051
ATOM   1458  HG1 THR A  89       9.819   5.377  -9.767  1.00 30.52           H  0.053
ATOM   1459 HG21 THR A  89       9.425   6.250  -6.607  1.00 28.10           H  0.051
ATOM   1460 HG22 THR A  89      10.333   5.161  -5.915  1.00 28.10           H  0.051
ATOM   1461 HG23 THR A  89      10.995   6.396  -6.644  1.00 28.10           H  0.051
ATOM   1462  N   ALA A  90      11.734   2.488  -6.981  1.00 18.32           N  0.041
ANISOU 1462  N   ALA A  90     1115   1671   4176    155     49   -200       N
ATOM   1463  CA  ALA A  90      12.096   1.752  -5.781  1.00 17.56           C  0.041
ANISOU 1463  CA  ALA A  90     1173   1571   3928    -43    216   -255       C
ATOM   1464  C   ALA A  90      13.576   1.419  -5.794  1.00 16.67           C  0.039
ANISOU 1464  C   ALA A  90     1221   1367   3746    -78    180     19       C
ATOM   1465  O   ALA A  90      14.246   1.513  -4.765  1.00 16.43           O  0.039
ANISOU 1465  O   ALA A  90     1434   1265   3545     50    282    -21       O
ATOM   1466  CB  ALA A  90      11.243   0.496  -5.623  1.00 16.85           C  0.040
ANISOU 1466  CB  ALA A  90     1022   1584   3796   -124    216   -395       C
ATOM   1467  H   ALA A  90      11.203   2.084  -7.523  1.00 22.14           H  0.046
ATOM   1468  HA  ALA A  90      11.930   2.317  -5.010  1.00 21.22           H  0.045
ATOM   1469  HB1 ALA A  90      11.512   0.032  -4.815  1.00 20.37           H  0.044
ATOM   1470  HB2 ALA A  90      10.310   0.754  -5.562  1.00 20.37           H  0.044
ATOM   1471  HB3 ALA A  90      11.379  -0.076  -6.394  1.00 20.37           H  0.044
ATOM   1472  N   SER A  91      14.100   1.024  -6.955  1.00 15.71           N  0.038
ANISOU 1472  N   SER A  91     1069   1405   3494   -104    272    104       N
ATOM   1473  CA  SER A  91      15.520   0.686  -7.034  1.00 14.94           C  0.037
ANISOU 1473  CA  SER A  91     1140   1278   3256     56    362    201       C
ATOM   1474  C   SER A  91      16.382   1.900  -6.761  1.00 13.71           C  0.036
ANISOU 1474  C   SER A  91     1272   1057   2880    -22    175    199       C
ATOM   1475  O   SER A  91      17.355   1.818  -6.026  1.00 13.95           O  0.036
ANISOU 1475  O   SER A  91     1376    946   2978    231    365    427       O
ATOM   1476  CB  SER A  91      15.889   0.103  -8.402  1.00 15.90           C  0.039
ANISOU 1476  CB  SER A  91     1378   1332   3330    -47   -151    208       C
ATOM   1477  OG  SER A  91      15.512  -1.281  -8.446  1.00 13.97           O  0.036
ANISOU 1477  OG  SER A  91     1116   1331   2860   -255   -372    132       O
ATOM   1478  H   SER A  91      13.667   0.945  -7.694  1.00 19.00           H  0.042
ATOM   1479  HA  SER A  91      15.723   0.019  -6.360  1.00 18.07           H  0.041
ATOM   1480  HB2 SER A  91      15.414   0.587  -9.095  1.00 19.23           H  0.042
ATOM   1481  HB3 SER A  91      16.847   0.179  -8.535  1.00 19.23           H  0.042
ATOM   1482  HG  SER A  91      14.684  -1.357  -8.326  1.00 16.91           H  0.040
ATOM   1483  N   VAL A  92      16.014   3.044  -7.316  1.00 13.24           N  0.035
ANISOU 1483  N   VAL A  92     1309   1007   2712   -257   -235     67       N
ATOM   1484  CA  VAL A  92      16.795   4.257  -7.066  1.00 13.48           C  0.036
ANISOU 1484  CA  VAL A  92     1257    973   2894   -177    -28    174       C
ATOM   1485  C   VAL A  92      16.783   4.588  -5.582  1.00 15.15           C  0.038
ANISOU 1485  C   VAL A  92     1236   1227   3293   -115    233    229       C
ATOM   1486  O   VAL A  92      17.814   4.919  -4.995  1.00 16.26           O  0.039
ANISOU 1486  O   VAL A  92     1570   1196   3410   -230    425     -7       O
ATOM   1487  CB  VAL A  92      16.263   5.435  -7.909  1.00 15.34           C  0.038
ANISOU 1487  CB  VAL A  92     1429   1073   3325    -51     29    319       C
ATOM   1488  CG1 VAL A  92      16.894   6.756  -7.416  1.00 13.63           C  0.036
ANISOU 1488  CG1 VAL A  92     1277    994   2906     -3    767    375       C
ATOM   1489  CG2 VAL A  92      16.528   5.178  -9.396  1.00 14.56           C  0.037
ANISOU 1489  CG2 VAL A  92     1527    997   3007    -87   -341    365       C
ATOM   1490  H   VAL A  92      15.333   3.150  -7.831  1.00 16.03           H  0.039
ATOM   1491  HA  VAL A  92      17.715   4.097  -7.327  1.00 16.33           H  0.039
ATOM   1492  HB  VAL A  92      15.303   5.500  -7.785  1.00 18.55           H  0.042
ATOM   1493 HG11 VAL A  92      16.894   7.396  -8.143  1.00 16.50           H  0.039
ATOM   1494 HG12 VAL A  92      16.374   7.099  -6.672  1.00 16.50           H  0.039
ATOM   1495 HG13 VAL A  92      17.806   6.584  -7.135  1.00 16.50           H  0.039
ATOM   1496 HG21 VAL A  92      16.144   5.902  -9.914  1.00 17.62           H  0.041
ATOM   1497 HG22 VAL A  92      17.486   5.138  -9.543  1.00 17.62           H  0.041
ATOM   1498 HG23 VAL A  92      16.119   4.336  -9.649  1.00 17.62           H  0.041
ATOM   1499  N   ASN A  93      15.584   4.620  -4.976  1.00 16.96           N  0.040
ANISOU 1499  N   ASN A  93     1191   1536   3715    -50    315    -63       N
ATOM   1500  CA  ASN A  93      15.500   5.064  -3.584  1.00 17.36           C  0.040
ANISOU 1500  CA  ASN A  93     1239   1690   3665     62    567   -142       C
ATOM   1501  C   ASN A  93      16.306   4.140  -2.673  1.00 15.87           C  0.039
ANISOU 1501  C   ASN A  93     1275   1476   3280   -109    455    -54       C
ATOM   1502  O   ASN A  93      16.935   4.594  -1.710  1.00 15.54           O  0.038
ANISOU 1502  O   ASN A  93     1366   1460   3080    -91    599    -37       O
ATOM   1503  CB  ASN A  93      14.035   5.103  -3.124  1.00 19.30           C  0.043
ANISOU 1503  CB  ASN A  93     1411   1967   3954     67    869   -188       C
ATOM   1504  CG  ASN A  93      13.263   6.321  -3.640  1.00 23.19           C  0.047
ANISOU 1504  CG  ASN A  93     1750   2289   4773    -83    904   -110       C
ATOM   1505  OD1 ASN A  93      13.813   7.141  -4.372  1.00 24.86           O  0.048
ANISOU 1505  OD1 ASN A  93     2029   2428   4990    -15    822   -224       O
ATOM   1506  ND2 ASN A  93      11.949   6.380  -3.330  1.00 25.53           N  0.049
ANISOU 1506  ND2 ASN A  93     1892   2504   5306    134    933   -205       N
ATOM   1507  H   ASN A  93      14.835   4.398  -5.336  1.00 20.50           H  0.044
```

```
ATOM   1508  HA  ASN A  93      15.866   5.959  -3.510  1.00 20.98           H  0.044
ATOM   1509  HB2 ASN A  93      13.584   4.307  -3.446  1.00 23.31           H  0.047
ATOM   1510  HB3 ASN A  93      14.012   5.125  -2.155  1.00 23.31           H  0.047
ATOM   1511 HD21 ASN A  93      11.472   7.041  -3.604  1.00 30.79           H  0.054
ATOM   1512 HD22 ASN A  93      11.598   5.776  -2.828  1.00 30.79           H  0.054
ATOM   1513  N   CYS A  94      16.299   2.839  -2.959  1.00 16.18           N  0.039
ANISOU 1513  N   CYS A  94     1335   1414   3401   -147    278     14       N
ATOM   1514  CA  CYS A  94      17.071   1.914  -2.142  1.00 15.09           C  0.038
ANISOU 1514  CA  CYS A  94     1378   1364   2992   -215    308   -148       C
ATOM   1515  C   CYS A  94      18.558   2.101  -2.420  1.00 13.91           C  0.036
ANISOU 1515  C   CYS A  94     1394   1282   2609   -333    265    -61       C
ATOM   1516  O   CYS A  94      19.373   2.162  -1.490  1.00 15.90           O  0.039
ANISOU 1516  O   CYS A  94     1737   1324   2979   -458    659    355       O
ATOM   1517  CB  CYS A  94      16.609   0.470  -2.412  1.00 15.27           C  0.038
ANISOU 1517  CB  CYS A  94     1511   1453   2837   -210    385   -136       C
ATOM   1518  SG  CYS A  94      17.333  -0.766  -1.332  1.00 15.81           S  0.038
ANISOU 1518  SG  CYS A  94     1659   1466   2884   -261    362     52       S
ATOM   1519  H   CYS A  94      15.865   2.476  -3.606  1.00 19.57           H  0.043
ATOM   1520  HA  CYS A  94      16.913   2.113  -1.206  1.00 18.26           H  0.041
ATOM   1521  HB2 CYS A  94      15.646   0.428  -2.302  1.00 18.47           H  0.042
ATOM   1522  HB3 CYS A  94      16.843   0.236  -3.324  1.00 18.47           H  0.042
ATOM   1523  N   ALA A  95      18.918   2.278  -3.696  1.00 13.81           N  0.036
ANISOU 1523  N   ALA A  95     1317   1377   2552   -122    300     22       N
ATOM   1524  CA  ALA A  95      20.324   2.494  -4.043  1.00 11.92           C  0.033
ANISOU 1524  CA  ALA A  95     1240   1195   2092     24    119    -75       C
ATOM   1525  C   ALA A  95      20.850   3.736  -3.348  1.00 10.36           C  0.031
ANISOU 1525  C   ALA A  95     1003   1155   1781     74     52    199       C
ATOM   1526  O   ALA A  95      22.023   3.765  -2.936  1.00 13.04           O  0.035
ANISOU 1526  O   ALA A  95      840   1229   2887    101   -327    182       O
ATOM   1527  CB  ALA A  95      20.486   2.657  -5.550  1.00 15.23           C  0.038
ANISOU 1527  CB  ALA A  95     1429   1395   2962    293    202    -34       C
ATOM   1528  H   ALA A  95      18.378   2.277  -4.366  1.00 16.72           H  0.040
ATOM   1529  HA  ALA A  95      20.851   1.732  -3.756  1.00 14.45           H  0.037
ATOM   1530  HB1 ALA A  95      21.424   2.798  -5.753  1.00 18.42           H  0.042
ATOM   1531  HB2 ALA A  95      20.170   1.853  -5.990  1.00 18.42           H  0.042
ATOM   1532  HB3 ALA A  95      19.965   3.421  -5.842  1.00 18.42           H  0.042
ATOM   1533  N   LYS A  96      20.003   4.763  -3.201  1.00 12.48           N  0.034
ANISOU 1533  N   LYS A  96     1270   1210   2264    -18    584   -207       N
ATOM   1534  CA  LYS A  96      20.467   5.964  -2.519  1.00 14.22           C  0.036
ANISOU 1534  CA  LYS A  96     1563   1214   2626   -166    476   -251       C
ATOM   1535  C   LYS A  96      20.803   5.662  -1.075  1.00 15.57           C  0.038
ANISOU 1535  C   LYS A  96     1643   1431   2841   -179    429   -235       C
ATOM   1536  O   LYS A  96      21.762   6.214  -0.538  1.00 16.00           O  0.039
ANISOU 1536  O   LYS A  96     1556   1586   2939   -307    589   -105       O
ATOM   1537  CB  LYS A  96      19.438   7.092  -2.608  1.00 15.35           C  0.038
ANISOU 1537  CB  LYS A  96     1638   1059   3134   -304    231   -434       C
ATOM   1538  CG  LYS A  96      19.422   7.721  -3.983  1.00 16.53           C  0.039
ANISOU 1538  CG  LYS A  96     1696    927   3657    -58     52   -136       C
ATOM   1539  CD  LYS A  96      18.270   8.678  -4.065  1.00 16.92           C  0.040
ANISOU 1539  CD  LYS A  96     1888    922   3620    111     16   -100       C
ATOM   1540  CE  LYS A  96      18.207   9.458  -5.366  1.00 17.57           C  0.041
ANISOU 1540  CE  LYS A  96     1930    999   3746    229    100    150       C
ATOM   1541  NZ  LYS A  96      17.022  10.408  -5.388  1.00 18.78           N  0.042
ANISOU 1541  NZ  LYS A  96     2080   1197   3859    477    298    225       N
ATOM   1542  H   LYS A  96      19.189   4.786  -3.477  1.00 15.13           H  0.038
ATOM   1543  HA  LYS A  96      21.279   6.272  -2.952  1.00 17.22           H  0.040
ATOM   1544  HB2 LYS A  96      18.555   6.734  -2.428  1.00 18.57           H  0.042
ATOM   1545  HB3 LYS A  96      19.660   7.780  -1.961  1.00 18.57           H  0.042
ATOM   1546  HG2 LYS A  96      20.246   8.211  -4.130  1.00 19.98           H  0.043
ATOM   1547  HG3 LYS A  96      19.305   7.034  -4.657  1.00 19.98           H  0.043
ATOM   1548  HD2 LYS A  96      17.443   8.179  -3.979  1.00 20.46           H  0.044
ATOM   1549  HD3 LYS A  96      18.344   9.318  -3.340  1.00 20.46           H  0.044
ATOM   1550  HE2 LYS A  96      19.018   9.981  -5.465  1.00 21.23           H  0.045
ATOM   1551  HE3 LYS A  96      18.115   8.839  -6.107  1.00 21.23           H  0.045
ATOM   1552  HZ1 LYS A  96      17.004  10.854  -6.158  1.00 22.69           H  0.046
ATOM   1553  HZ2 LYS A  96      16.264   9.950  -5.301  1.00 22.69           H  0.046
ATOM   1554  HZ3 LYS A  96      17.087  10.990  -4.718  1.00 22.69           H  0.046
ATOM   1555  N   LYS A  97      20.021   4.790  -0.425  1.00 15.66           N  0.038
ANISOU 1555  N   LYS A  97     1788   1671   2491   -184    313     -7       N
ATOM   1556  CA  LYS A  97      20.361   4.400   0.959  1.00 16.11           C  0.039
ANISOU 1556  CA  LYS A  97     1769   1730   2623   -296    451   -190       C
ATOM   1557  C   LYS A  97      21.649   3.551   0.966  1.00 14.78           C  0.037
ANISOU 1557  C   LYS A  97     1542   1556   2519   -293    301   -258       C
ATOM   1558  O   LYS A  97      22.458   3.740   1.854  1.00 14.92           O  0.037
ANISOU 1558  O   LYS A  97     1554   1492   2622   -465    217   -361       O
ATOM   1559  CB  LYS A  97      19.174   3.658   1.592  1.00 19.15           C  0.042
ANISOU 1559  CB  LYS A  97     2038   2032   3206   -571    638     51       C
ATOM   1560  CG  LYS A  97      19.500   2.928   2.877  1.00 23.86           C  0.047
```

```
ANISOU 1560  CG  LYS A  97     2311   2404   4350   -673    783    -16       C
ATOM   1561  CD  LYS A  97      18.284   2.580   3.697  1.00 26.80           C  0.050
ANISOU 1561  CD  LYS A  97     2553   2612   5016   -691    949     26       C
ATOM   1562  CE  LYS A  97      18.666   2.444   5.152  1.00 29.53           C  0.053
ANISOU 1562  CE  LYS A  97     2755   2792   5675   -602    894    -34       C
ATOM   1563  NZ  LYS A  97      18.626   3.757   5.836  1.00 31.54           N  0.054
ANISOU 1563  NZ  LYS A  97     2929   2932   6122   -473    839    -80       N
ATOM   1564  H   LYS A  97      19.314   4.420  -0.745  1.00 18.94           H  0.042
ATOM   1565  HA  LYS A  97      20.527   5.200   1.481  1.00 19.48           H  0.043
ATOM   1566  HB2 LYS A  97      18.476   4.303   1.787  1.00 23.13           H  0.047
ATOM   1567  HB3 LYS A  97      18.844   3.003   0.957  1.00 23.13           H  0.047
ATOM   1568  HG2 LYS A  97      19.958   2.101   2.660  1.00 28.78           H  0.052
ATOM   1569  HG3 LYS A  97      20.074   3.491   3.420  1.00 28.78           H  0.052
ATOM   1570  HD2 LYS A  97      17.623   3.285   3.617  1.00 32.31           H  0.055
ATOM   1571  HD3 LYS A  97      17.919   1.734   3.393  1.00 32.31           H  0.055
ATOM   1572  HE2 LYS A  97      18.041   1.849   5.595  1.00 35.59           H  0.058
ATOM   1573  HE3 LYS A  97      19.568   2.094   5.216  1.00 35.59           H  0.058
ATOM   1574  HZ1 LYS A  97      18.853   3.661   6.691  1.00 37.99           H  0.060
ATOM   1575  HZ2 LYS A  97      19.195   4.320   5.447  1.00 37.99           H  0.060
ATOM   1576  HZ3 LYS A  97      17.805   4.098   5.791  1.00 37.99           H  0.060
ATOM   1577  N   ILE A  98      21.767   2.611   0.017  1.00 14.76           N  0.037
ANISOU 1577  N   ILE A  98     1407   1369   2833   -179    225   -420       N
ATOM   1578  CA  ILE A  98      22.946   1.753  -0.028  1.00 14.43           C  0.037
ANISOU 1578  CA  ILE A  98     1271   1140   3072   -287     93   -296       C
ATOM   1579  C   ILE A  98      24.208   2.588  -0.190  1.00 13.12           C  0.035
ANISOU 1579  C   ILE A  98     1166   1083   2736   -249   -125   -450       C
ATOM   1580  O   ILE A  98      25.208   2.387   0.514  1.00 13.18           O  0.035
ANISOU 1580  O   ILE A  98     1586   1126   2294   -102    114     65       O
ATOM   1581  CB  ILE A  98      22.806   0.711  -1.146  1.00 13.38           C  0.035
ANISOU 1581  CB  ILE A  98     1234   1064   2785   -233    380   -128       C
ATOM   1582  CG1 ILE A  98      21.617  -0.209  -0.867  1.00 14.69           C  0.037
ANISOU 1582  CG1 ILE A  98     1255   1160   3168   -362    485   -304       C
ATOM   1583  CG2 ILE A  98      24.082  -0.114  -1.320  1.00 13.69           C  0.036
ANISOU 1583  CG2 ILE A  98     1247   1030   2926   -374    119    128       C
ATOM   1584  CD1 ILE A  98      21.315  -1.133  -2.029  1.00 15.03           C  0.038
ANISOU 1584  CD1 ILE A  98     1244   1270   3198   -348    683   -414       C
ATOM   1585  H   ILE A  98      21.187   2.457  -0.599  1.00 17.87           H  0.041
ATOM   1586  HA  ILE A  98      23.015   1.275   0.813  1.00 17.47           H  0.040
ATOM   1587  HB  ILE A  98      22.635   1.181  -1.977  1.00 16.20           H  0.039
ATOM   1588 HG12 ILE A  98      21.819  -0.749  -0.093  1.00 17.78           H  0.041
ATOM   1589 HG13 ILE A  98      20.828   0.329  -0.700  1.00 17.78           H  0.041
ATOM   1590 HG21 ILE A  98      23.932  -0.805  -1.983  1.00 16.58           H  0.039
ATOM   1591 HG22 ILE A  98      24.800   0.466  -1.617  1.00 16.58           H  0.039
ATOM   1592 HG23 ILE A  98      24.313  -0.518  -0.469  1.00 16.58           H  0.039
ATOM   1593 HD11 ILE A  98      20.422  -1.495  -1.920  1.00 18.19           H  0.041
ATOM   1594 HD12 ILE A  98      21.369  -0.628  -2.855  1.00 18.19           H  0.041
ATOM   1595 HD13 ILE A  98      21.964  -1.853  -2.039  1.00 18.19           H  0.041
ATOM   1596  N   VAL A  99      24.197   3.529  -1.137  1.00 12.14           N  0.034
ANISOU 1596  N   VAL A  99     1088   1094   2432   -186   -127   -296       N
ATOM   1597  CA  VAL A  99      25.435   4.235  -1.453  1.00 12.83           C  0.035
ANISOU 1597  CA  VAL A  99     1124   1188   2562   -224     71   -311       C
ATOM   1598  C   VAL A  99      25.827   5.185  -0.339  1.00 14.27           C  0.037
ANISOU 1598  C   VAL A  99     1371   1411   2639   -222     25   -178       C
ATOM   1599  O   VAL A  99      26.964   5.668  -0.327  1.00 14.97           O  0.037
ANISOU 1599  O   VAL A  99     1406   1747   2536   -396    238     83       O
ATOM   1600  CB  VAL A  99      25.313   4.931  -2.819  1.00 12.40           C  0.034
ANISOU 1600  CB  VAL A  99      932   1295   2483   -175    -75   -228       C
ATOM   1601  CG1 VAL A  99      24.473   6.250  -2.711  1.00 10.83           C  0.032
ANISOU 1601  CG1 VAL A  99      848   1044   2221    -97    549   -226       C
ATOM   1602  CG2 VAL A  99      26.667   5.152  -3.433  1.00 14.93           C  0.037
ANISOU 1602  CG2 VAL A  99     1056   1423   3195   -315     83    -68       C
ATOM   1603  H   VAL A  99      23.510   3.769  -1.595  1.00 14.72           H  0.037
ATOM   1604  HA  VAL A  99      26.145   3.579  -1.530  1.00 15.54           H  0.038
ATOM   1605  HB  VAL A  99      24.828   4.337  -3.413  1.00 15.02           H  0.037
ATOM   1606 HG11 VAL A  99      24.042   6.421  -3.563  1.00 13.14           H  0.035
ATOM   1607 HG12 VAL A  99      23.804   6.143  -2.017  1.00 13.14           H  0.035
ATOM   1608 HG13 VAL A  99      25.069   6.980  -2.489  1.00 13.14           H  0.035
ATOM   1609 HG21 VAL A  99      26.556   5.595  -4.289  1.00 18.07           H  0.041
ATOM   1610 HG22 VAL A  99      27.196   5.707  -2.839  1.00 18.07           H  0.041
ATOM   1611 HG23 VAL A  99      27.101   4.293  -3.558  1.00 18.07           H  0.041
ATOM   1612  N   SER A 100      24.926   5.426   0.612  1.00 15.67           N  0.038
ANISOU 1612  N   SER A 100     1795   1399   2761   -269     78   -588       N
ATOM   1613  CA  SER A 100      25.135   6.381   1.686  1.00 17.26           C  0.040
ANISOU 1613  CA  SER A 100     2196   1602   2760   -146    361   -632       C
ATOM   1614  C   SER A 100      25.675   5.750   2.947  1.00 17.06           C  0.040
ANISOU 1614  C   SER A 100     2238   1903   2342   -118    488   -673       C
ATOM   1615  O   SER A 100      25.936   6.453   3.926  1.00 18.64           O  0.042
ANISOU 1615  O   SER A 100     2276   2164   2641    -54    308   -608       O
```

```
ATOM   1616  CB  SER A 100      23.836   7.112   2.008  1.00 17.98           C  0.041
ANISOU 1616  CB  SER A 100     2507   1571   2756    -44    290   -853        C
ATOM   1617  OG  SER A 100      23.391   7.795   0.858  1.00 19.84           O  0.043
ANISOU 1617  OG  SER A 100     2738   1525   3276    -40    302   -586        O
ATOM   1618  H   SER A 100      24.161   5.036   0.655  1.00 18.96           H  0.042
ATOM   1619  HA  SER A 100      25.779   7.043   1.390  1.00 20.86           H  0.044
ATOM   1620  HB2 SER A 100      23.164   6.467   2.279  1.00 21.73           H  0.045
ATOM   1621  HB3 SER A 100      23.996   7.752   2.720  1.00 21.73           H  0.045
ATOM   1622  HG  SER A 100      23.246   7.248   0.237  1.00 23.96           H  0.047
ATOM   1623  N   ASP A 101      25.842   4.435   2.938  1.00 18.08           N  0.041
ANISOU 1623  N   ASP A 101     2265   2117   2489   -128    782    -91        N
ATOM   1624  CA  ASP A 101      26.105   3.682   4.191  1.00 21.17           C  0.045
ANISOU 1624  CA  ASP A 101     2483   2350   3212   -221    647    140        C
ATOM   1625  C   ASP A 101      27.571   3.499   4.580  1.00 21.42           C  0.045
ANISOU 1625  C   ASP A 101     2462   2473   3203   -121    281    267        C
ATOM   1626  O   ASP A 101      27.811   2.868   5.608  1.00 21.05           O  0.044
ANISOU 1626  O   ASP A 101     2481   2587   2930    -90    242    465        O
ATOM   1627  CB  ASP A 101      25.417   2.313   4.113  1.00 21.89           C  0.045
ANISOU 1627  CB  ASP A 101     2707   2330   3278   -304    879    484        C
ATOM   1628  CG  ASP A 101      24.768   1.978   5.441  1.00 24.63           C  0.048
ANISOU 1628  CG  ASP A 101     2924   2418   4016   -268    974    131        C
ATOM   1629  OD1 ASP A 101      24.129   2.874   6.019  1.00 28.68           O  0.052
ANISOU 1629  OD1 ASP A 101     3169   2610   5118   -128    932    -97        O
ATOM   1630  OD2 ASP A 101      24.938   0.856   5.902  1.00 22.47           O  0.046
ANISOU 1630  OD2 ASP A 101     2870   2243   3425   -591    826    247        O
ATOM   1631  H   ASP A 101      25.813   3.943   2.233  1.00 21.85           H  0.045
ATOM   1632  HA  ASP A 101      25.690   4.173   4.916  1.00 25.56           H  0.049
ATOM   1633  HB2 ASP A 101      24.728   2.334   3.430  1.00 26.41           H  0.050
ATOM   1634  HB3 ASP A 101      26.075   1.630   3.911  1.00 26.41           H  0.050
ATOM   1635  N   GLY A 102      28.503   4.069   3.821  1.00 20.37           N  0.044
ANISOU 1635  N   GLY A 102     2296   2411   3032   -153    316    -34        N
ATOM   1636  CA  GLY A 102      29.889   3.997   4.281  1.00 18.80           C  0.042
ANISOU 1636  CA  GLY A 102     2152   2372   2620   -249    165   -148        C
ATOM   1637  C   GLY A 102      30.933   3.691   3.222  1.00 17.32           C  0.040
ANISOU 1637  C   GLY A 102     2054   2211   2316   -359    340    -50        C
ATOM   1638  O   GLY A 102      32.039   4.251   3.224  1.00 17.51           O  0.040
ANISOU 1638  O   GLY A 102     2037   2177   2440   -526    435   -420        O
ATOM   1639  H   GLY A 102      28.373   4.481   3.077  1.00 24.59           H  0.048
ATOM   1640  HA2 GLY A 102      30.124   4.846   4.686  1.00 22.71           H  0.046
ATOM   1641  HA3 GLY A 102      29.965   3.314   4.964  1.00 22.71           H  0.046
ATOM   1642  N   ASN A 103      30.595   2.796   2.302  1.00 16.06           N  0.039
ANISOU 1642  N   ASN A 103     2024   1952   2127   -342    149     38        N
ATOM   1643  CA  ASN A 103      31.551   2.355   1.285  1.00 15.67           C  0.038
ANISOU 1643  CA  ASN A 103     1934   1846   2174   -280     57     73        C
ATOM   1644  C   ASN A 103      31.194   2.868  -0.105  1.00 13.41           C  0.035
ANISOU 1644  C   ASN A 103     1470   1471   2152   -124   -376     12        C
ATOM   1645  O   ASN A 103      31.777   2.426  -1.094  1.00 13.07           O  0.035
ANISOU 1645  O   ASN A 103     1435   1416   2115   -146   -401   -203        O
ATOM   1646  CB  ASN A 103      31.662   0.832   1.316  1.00 17.06           C  0.040
ANISOU 1646  CB  ASN A 103     2151   2128   2204   -243   -185    337        C
ATOM   1647  CG  ASN A 103      32.266   0.324   2.604  1.00 19.66           C  0.043
ANISOU 1647  CG  ASN A 103     2442   2403   2626   -302    -89    148        C
ATOM   1648  OD1 ASN A 103      32.989   1.063   3.301  1.00 21.86           O  0.045
ANISOU 1648  OD1 ASN A 103     2512   2547   3245   -334   -299    340        O
ATOM   1649  ND2 ASN A 103      31.844  -0.861   3.008  1.00 21.03           N  0.044
ANISOU 1649  ND2 ASN A 103     2624   2475   2893   -306     -8     68        N
ATOM   1650  H   ASN A 103      29.820   2.427   2.242  1.00 19.42           H  0.043
ATOM   1651  HA  ASN A 103      32.426   2.710   1.505  1.00 18.95           H  0.042
ATOM   1652  HB2 ASN A 103      30.776   0.448   1.227  1.00 20.63           H  0.044
ATOM   1653  HB3 ASN A 103      32.227   0.540   0.583  1.00 20.63           H  0.044
ATOM   1654 HD21 ASN A 103      32.143  -1.196   3.742  1.00 25.39           H  0.049
ATOM   1655 HD22 ASN A 103      31.321  -1.324   2.506  1.00 25.39           H  0.049
ATOM   1656  N   GLY A 104      30.304   3.852  -0.189  1.00 13.68           N  0.036
ANISOU 1656  N   GLY A 104     1244   1396   2559     34   -285     27        N
ATOM   1657  CA  GLY A 104      29.960   4.422  -1.477  1.00 12.71           C  0.034
ANISOU 1657  CA  GLY A 104     1165   1174   2492   -224   -113    198        C
ATOM   1658  C   GLY A 104      29.546   3.319  -2.440  1.00 10.44           C  0.031
ANISOU 1658  C   GLY A 104      935    766   2266   -267   -101     76        C
ATOM   1659  O   GLY A 104      28.813   2.362  -2.072  1.00 10.01           O  0.031
ANISOU 1659  O   GLY A 104      955    675   2176   -206    249      5        O
ATOM   1660  H   GLY A 104      29.891   4.203   0.479  1.00 16.57           H  0.039
ATOM   1661  HA2 GLY A 104      29.225   5.047  -1.378  1.00 15.41           H  0.038
ATOM   1662  HA3 GLY A 104      30.725   4.891  -1.845  1.00 15.41           H  0.038
ATOM   1663  N   MET A 105      29.978   3.432  -3.697  1.00 10.81           N  0.032
ANISOU 1663  N   MET A 105      998    808   2301   -137     40   -346        N
ATOM   1664  CA  MET A 105      29.525   2.480  -4.718  1.00  9.78           C  0.030
ANISOU 1664  CA  MET A 105      938    901   1878    -38     -7   -229        C
ATOM   1665  C   MET A 105      30.285   1.184  -4.691  1.00 10.55           C  0.031
```

```
ANISOU 1665  C   MET A 105      900    964   2145     57    243   -186       C
ATOM   1666  O   MET A 105      29.919   0.289  -5.458  1.00 10.83           O  0.032
ANISOU 1666  O   MET A 105      924   1240   1952     14    155    -50       O
ATOM   1667  CB  MET A 105      29.562   3.116  -6.106  1.00 11.45           C  0.033
ANISOU 1667  CB  MET A 105      999   1179   2171   -282    176    -20       C
ATOM   1668  CG  MET A 105      28.421   4.168  -6.357  1.00 11.68           C  0.033
ANISOU 1668  CG  MET A 105      942   1246   2251   -280    239    147       C
ATOM   1669  SD  MET A 105      28.152   4.607  -8.095  1.00 12.38           S  0.034
ANISOU 1669  SD  MET A 105     1130   1317   2258   -191     99    -90       S
ATOM   1670  CE  MET A 105      27.379   3.090  -8.685  1.00 11.24           C  0.032
ANISOU 1670  CE  MET A 105     1088   1294   1890   -326   -304     -2       C
ATOM   1671  H   MET A 105      30.521   4.035  -3.981  1.00 13.12           H  0.035
ATOM   1672  HA  MET A 105      28.595   2.275  -4.534  1.00 11.89           H  0.033
ATOM   1673  HB2 MET A 105      30.412   3.569  -6.219  1.00 13.88           H  0.036
ATOM   1674  HB3 MET A 105      29.471   2.417  -6.772  1.00 13.88           H  0.036
ATOM   1675  HG2 MET A 105      27.588   3.808  -6.014  1.00 14.17           H  0.036
ATOM   1676  HG3 MET A 105      28.643   4.983  -5.880  1.00 14.17           H  0.036
ATOM   1677  HE1 MET A 105      27.155   3.195  -9.623  1.00 13.64           H  0.036
ATOM   1678  HE2 MET A 105      28.001   2.354  -8.574  1.00 13.64           H  0.036
ATOM   1679  HE3 MET A 105      26.575   2.925  -8.169  1.00 13.64           H  0.036
ATOM   1680  N   ASN A 106      31.265   1.064  -3.795  1.00 11.59           N  0.033
ANISOU 1680  N   ASN A 106     1025    918   2461   -136   -201    -32       N
ATOM   1681  CA  ASN A 106      31.930  -0.219  -3.588  1.00 12.03           C  0.034
ANISOU 1681  CA  ASN A 106     1100    895   2575   -282    -29    336       C
ATOM   1682  C   ASN A 106      30.923  -1.268  -3.123  1.00 12.08           C  0.034
ANISOU 1682  C   ASN A 106      938   1137   2515   -357    156    186       C
ATOM   1683  O   ASN A 106      31.222  -2.477  -3.200  1.00 14.86           O  0.037
ANISOU 1683  O   ASN A 106     1257   1294   3095   -412    -26    306       O
ATOM   1684  CB  ASN A 106      33.114  -0.074  -2.631  1.00 13.25           C  0.035
ANISOU 1684  CB  ASN A 106     1366    847   2821   -145   -118    169       C
ATOM   1685  CG  ASN A 106      34.183   0.838  -3.201  1.00 12.91           C  0.035
ANISOU 1685  CG  ASN A 106     1412    898   2596    -40     29     60       C
ATOM   1686  OD1 ASN A 106      34.837   0.503  -4.210  1.00 12.35           O  0.034
ANISOU 1686  OD1 ASN A 106     1182    922   2588     76     80   -193       O
ATOM   1687  ND2 ASN A 106      34.332   2.032  -2.606  1.00 13.26           N  0.035
ANISOU 1687  ND2 ASN A 106     1614   1089   2337   -296    -44   -179       N
ATOM   1688  H   ASN A 106      31.562   1.702  -3.300  1.00 14.06           H  0.036
ATOM   1689  HA  ASN A 106      32.284  -0.519  -4.440  1.00 14.58           H  0.037
ATOM   1690  HB2 ASN A 106      32.805   0.305  -1.794  1.00 16.05           H  0.039
ATOM   1691  HB3 ASN A 106      33.510  -0.946  -2.478  1.00 16.05           H  0.039
ATOM   1692 HD21 ASN A 106      34.922   2.586  -2.897  1.00 16.07           H  0.039
ATOM   1693 HD22 ASN A 106      33.855   2.232  -1.919  1.00 16.07           H  0.039
ATOM   1694  N   ALA A 107      29.737  -0.808  -2.666  1.00 11.64           N  0.033
ANISOU 1694  N   ALA A 107      894   1305   2226   -468    -48   -131       N
ATOM   1695  CA  ALA A 107      28.667  -1.728  -2.299  1.00 12.27           C  0.034
ANISOU 1695  CA  ALA A 107     1321   1281   2061   -242     70    -15       C
ATOM   1696  C   ALA A 107      28.287  -2.617  -3.469  1.00 13.86           C  0.036
ANISOU 1696  C   ALA A 107     1304   1212   2750   -139    235   -204       C
ATOM   1697  O   ALA A 107      27.769  -3.713  -3.256  1.00 14.87           O  0.037
ANISOU 1697  O   ALA A 107     1579   1329   2743   -366    -15   -245       O
ATOM   1698  CB  ALA A 107      27.462  -0.937  -1.809  1.00 13.72           C  0.036
ANISOU 1698  CB  ALA A 107     1594   1506   2113   -208    198   -113       C
ATOM   1699  H   ALA A 107      29.539   0.022  -2.559  1.00 14.12           H  0.036
ATOM   1700  HA  ALA A 107      28.971  -2.296  -1.574  1.00 14.88           H  0.037
ATOM   1701  HB1 ALA A 107      26.756  -1.556  -1.567  1.00 16.62           H  0.039
ATOM   1702  HB2 ALA A 107      27.722  -0.413  -1.035  1.00 16.62           H  0.039
ATOM   1703  HB3 ALA A 107      27.160  -0.351  -2.520  1.00 16.62           H  0.039
ATOM   1704  N   TRP A 108      28.497  -2.150  -4.705  1.00 12.66           N  0.034
ANISOU 1704  N   TRP A 108     1330   1106   2373   -108    -42   -368       N
ATOM   1705  CA  TRP A 108      28.251  -2.934  -5.915  1.00 11.08           C  0.032
ANISOU 1705  CA  TRP A 108     1372    946   1890   -216    230     -9       C
ATOM   1706  C   TRP A 108      29.589  -3.454  -6.436  1.00 11.87           C  0.033
ANISOU 1706  C   TRP A 108     1511    781   2216   -115    545   -163       C
ATOM   1707  O   TRP A 108      30.415  -2.696  -6.944  1.00 13.39           O  0.035
ANISOU 1707  O   TRP A 108     1595    732   2761   -126    243    112       O
ATOM   1708  CB  TRP A 108      27.575  -2.061  -6.965  1.00 10.80           C  0.032
ANISOU 1708  CB  TRP A 108     1321    919   1864   -217    190    147       C
ATOM   1709  CG  TRP A 108      26.163  -1.742  -6.624  1.00 11.66           C  0.033
ANISOU 1709  CG  TRP A 108     1221    978   2230   -127    188    206       C
ATOM   1710  CD1 TRP A 108      25.068  -2.530  -6.862  1.00 12.34           C  0.034
ANISOU 1710  CD1 TRP A 108     1136    911   2641    -96    284   -105       C
ATOM   1711  CD2 TRP A 108      25.678  -0.581  -5.945  1.00 10.51           C  0.031
ANISOU 1711  CD2 TRP A 108     1150   1038   1806   -166    216    140       C
ATOM   1712  NE1 TRP A 108      23.942  -1.942  -6.383  1.00 12.65           N  0.034
ANISOU 1712  NE1 TRP A 108     1200    881   2724   -290    259   -264       N
ATOM   1713  CE2 TRP A 108      24.284  -0.737  -5.824  1.00 11.60           C  0.033
ANISOU 1713  CE2 TRP A 108     1168   1073   2167   -275    287   -208       C
ATOM   1714  CE3 TRP A 108      26.268   0.555  -5.420  1.00 12.75           C  0.035
```

```
ANISOU 1714  CE3 TRP A 108     1282   1208   2356    -31    -24    -10       C
ATOM   1715  CZ2 TRP A 108      23.481   0.192  -5.192  1.00 11.67           C  0.033
ANISOU 1715  CZ2 TRP A 108     1149   1191   2093   -292    466    -38       C
ATOM   1716  CZ3 TRP A 108      25.445   1.501  -4.844  1.00 11.91           C  0.033
ANISOU 1716  CZ3 TRP A 108     1243   1165   2118    -28     64   -109       C
ATOM   1717  CH2 TRP A 108      24.088   1.314  -4.718  1.00 12.32           C  0.034
ANISOU 1717  CH2 TRP A 108     1107   1186   2386   -188    100   -257       C
ATOM   1718  H   TRP A 108      28.790  -1.358  -4.870  1.00 15.34           H  0.038
ATOM   1719  HA  TRP A 108      27.675  -3.688  -5.713  1.00 13.44           H  0.035
ATOM   1720  HB2 TRP A 108      28.061  -1.225  -7.042  1.00 13.11           H  0.035
ATOM   1721  HB3 TRP A 108      27.580  -2.528  -7.815  1.00 13.11           H  0.035
ATOM   1722  HD1 TRP A 108      25.094  -3.358  -7.286  1.00 14.95           H  0.037
ATOM   1723  HE1 TRP A 108      23.146  -2.264  -6.434  1.00 15.33           H  0.038
ATOM   1724  HE3 TRP A 108      27.184   0.697  -5.494  1.00 15.45           H  0.038
ATOM   1725  HZ2 TRP A 108      22.560   0.078  -5.135  1.00 14.15           H  0.036
ATOM   1726  HZ3 TRP A 108      25.827   2.269  -4.484  1.00 14.44           H  0.037
ATOM   1727  HH2 TRP A 108      23.574   1.971  -4.308  1.00 14.93           H  0.037
ATOM   1728  N   VAL A 109      29.809  -4.758  -6.291  1.00 14.91           N  0.037
ANISOU 1728  N   VAL A 109     1743    868   3055     90    773    -13       N
ATOM   1729  CA  VAL A 109      31.100  -5.339  -6.631  1.00 16.39           C  0.039
ANISOU 1729  CA  VAL A 109     2039   1015   3172    193    969    144       C
ATOM   1730  C   VAL A 109      31.378  -5.181  -8.116  1.00 16.27           C  0.039
ANISOU 1730  C   VAL A 109     2001   1035   3147    305    540     11       C
ATOM   1731  O   VAL A 109      32.513  -4.890  -8.525  1.00 17.13           O  0.040
ANISOU 1731  O   VAL A 109     1907   1104   3497    437    401    250       O
ATOM   1732  CB  VAL A 109      31.084  -6.821  -6.191  1.00 20.23           C  0.044
ANISOU 1732  CB  VAL A 109     2422   1280   3982    356   1153    440       C
ATOM   1733  CG1 VAL A 109      32.189  -7.679  -6.837  1.00 21.25           C  0.045
ANISOU 1733  CG1 VAL A 109     2427   1462   4187    375   1165    787       C
ATOM   1734  CG2 VAL A 109      31.150  -6.909  -4.669  1.00 22.71           C  0.046
ANISOU 1734  CG2 VAL A 109     2599   1499   4531    561    998    402       C
ATOM   1735  H   VAL A 109      29.230  -5.323  -5.999  1.00 18.04           H  0.041
ATOM   1736  HA  VAL A 109      31.799  -4.881  -6.139  1.00 19.81           H  0.043
ATOM   1737  HB  VAL A 109      30.235  -7.204  -6.461  1.00 24.42           H  0.048
ATOM   1738 HG11 VAL A 109      32.223  -8.538  -6.388  1.00 25.66           H  0.049
ATOM   1739 HG12 VAL A 109      31.985  -7.806  -7.777  1.00 25.66           H  0.049
ATOM   1740 HG13 VAL A 109      33.039  -7.223  -6.740  1.00 25.66           H  0.049
ATOM   1741 HG21 VAL A 109      31.140  -7.843  -4.406  1.00 27.40           H  0.051
ATOM   1742 HG22 VAL A 109      31.969  -6.488  -4.364  1.00 27.40           H  0.051
ATOM   1743 HG23 VAL A 109      30.382  -6.451  -4.293  1.00 27.40           H  0.051
ATOM   1744  N   ALA A 110      30.346  -5.319  -8.949  1.00 16.70           N  0.040
ANISOU 1744  N   ALA A 110     1895   1107   3344    101    350   -385       N
ATOM   1745  CA  ALA A 110      30.542  -5.057 -10.368  1.00 14.72           C  0.037
ANISOU 1745  CA  ALA A 110     1681   1146   2766    131    538   -649       C
ATOM   1746  C   ALA A 110      30.950  -3.611 -10.635  1.00 13.49           C  0.036
ANISOU 1746  C   ALA A 110     1369   1187   2571    -54    445   -384       C
ATOM   1747  O   ALA A 110      31.697  -3.345 -11.581  1.00 12.99           O  0.035
ANISOU 1747  O   ALA A 110     1282   1446   2208    -76    524     67       O
ATOM   1748  CB  ALA A 110      29.332  -5.481 -11.188  1.00 15.69           C  0.038
ANISOU 1748  CB  ALA A 110     1839   1349   2774     96    408   -741       C
ATOM   1749  H   ALA A 110      29.550  -5.555  -8.725  1.00 20.19           H  0.043
ATOM   1750  HA  ALA A 110      31.282  -5.609 -10.666  1.00 17.81           H  0.041
ATOM   1751  HB1 ALA A 110      29.506  -5.299 -12.125  1.00 18.98           H  0.042
ATOM   1752  HB2 ALA A 110      29.180  -6.430 -11.058  1.00 18.98           H  0.042
ATOM   1753  HB3 ALA A 110      28.558  -4.978 -10.891  1.00 18.98           H  0.042
ATOM   1754  N   TRP A 111      30.439  -2.658  -9.855  1.00 14.21           N  0.036
ANISOU 1754  N   TRP A 111     1360   1110   2928   -180    144   -275       N
ATOM   1755  CA  TRP A 111      30.928  -1.290 -10.039  1.00 13.62           C  0.036
ANISOU 1755  CA  TRP A 111     1268   1185   2721    -69    -15     80       C
ATOM   1756  C   TRP A 111      32.415  -1.190  -9.721  1.00 12.88           C  0.035
ANISOU 1756  C   TRP A 111     1219   1202   2473    165    108    216       C
ATOM   1757  O   TRP A 111      33.187  -0.631 -10.512  1.00 12.42           O  0.034
ANISOU 1757  O   TRP A 111      910   1263   2548    140    143    -48       O
ATOM   1758  CB  TRP A 111      30.121  -0.292  -9.216  1.00 12.51           C  0.034
ANISOU 1758  CB  TRP A 111     1265   1222   2267   -268    -93    212       C
ATOM   1759  CG  TRP A 111      30.671   1.107  -9.357  1.00 11.49           C  0.033
ANISOU 1759  CG  TRP A 111      998   1157   2211   -236     56    135       C
ATOM   1760  CD1 TRP A 111      30.370   2.038 -10.339  1.00 10.53           C  0.031
ANISOU 1760  CD1 TRP A 111      871    995   2136   -382    -81     93       C
ATOM   1761  CD2 TRP A 111      31.634   1.740  -8.501  1.00 11.25           C  0.032
ANISOU 1761  CD2 TRP A 111     1030   1153   2090   -151      6    -77       C
ATOM   1762  NE1 TRP A 111      31.112   3.166 -10.149  1.00  9.01           N  0.029
ANISOU 1762  NE1 TRP A 111      792   1017   1614   -411   -306    211       N
ATOM   1763  CE2 TRP A 111      31.886   3.007  -9.032  1.00  9.40           C  0.030
ANISOU 1763  CE2 TRP A 111      863   1130   1577    -75   -561   -218       C
ATOM   1764  CE3 TRP A 111      32.330   1.341  -7.344  1.00 11.54           C  0.033
ANISOU 1764  CE3 TRP A 111      823   1161   2402    106     29    -98       C
ATOM   1765  CZ2 TRP A 111      32.754   3.897  -8.427  1.00 10.75           C  0.032
```

```
ANISOU 1765  CZ2 TRP A 111      945   1235   1905    -23   -593   -443       C
ATOM   1766  CZ3 TRP A 111      33.190   2.225  -6.748  1.00 10.00           C  0.031
ANISOU 1766  CZ3 TRP A 111      874   1229   1695    172   -410    108       C
ATOM   1767  CH2 TRP A 111      33.402   3.493  -7.295  1.00 11.24           C  0.032
ANISOU 1767  CH2 TRP A 111      813   1238   2219    172   -447   -178       C
ATOM   1768  H   TRP A 111      29.842  -2.766  -9.245  1.00 17.20           H  0.040
ATOM   1769  HA  TRP A 111      30.816  -1.050 -10.972  1.00 16.49           H  0.039
ATOM   1770  HB2 TRP A 111      29.202  -0.290  -9.525  1.00 15.16           H  0.038
ATOM   1771  HB3 TRP A 111      30.161  -0.542  -8.280  1.00 15.16           H  0.038
ATOM   1772  HD1 TRP A 111      29.790   1.894 -11.051  1.00 12.79           H  0.035
ATOM   1773  HE1 TRP A 111      31.077   3.874 -10.636  1.00 10.96           H  0.032
ATOM   1774  HE3 TRP A 111      32.187   0.500  -6.974  1.00 14.00           H  0.036
ATOM   1775  HZ2 TRP A 111      32.899   4.743  -8.786  1.00 13.05           H  0.035
ATOM   1776  HZ3 TRP A 111      33.654   1.971  -5.983  1.00 12.14           H  0.034
ATOM   1777  HH2 TRP A 111      34.003   4.072  -6.883  1.00 13.63           H  0.036
ATOM   1778  N   ARG A 112      32.836  -1.756  -8.580  1.00 11.41           N  0.033
ANISOU 1778  N   ARG A 112     1346   1146   1842    367    263    361       N
ATOM   1779  CA  ARG A 112      34.225  -1.656  -8.186  1.00 12.98           C  0.035
ANISOU 1779  CA  ARG A 112     1631   1261   2040    334    142     68       C
ATOM   1780  C   ARG A 112      35.123  -2.299  -9.218  1.00 12.54           C  0.034
ANISOU 1780  C   ARG A 112     1465   1001   2301    315    143     73       C
ATOM   1781  O   ARG A 112      36.197  -1.770  -9.537  1.00 14.60           O  0.037
ANISOU 1781  O   ARG A 112     1606   1062   2878    199    468     38       O
ATOM   1782  CB  ARG A 112      34.469  -2.340  -6.843  1.00 15.48           C  0.038
ANISOU 1782  CB  ARG A 112     2046   1542   2293    323     79    137       C
ATOM   1783  CG  ARG A 112      35.960  -2.282  -6.433  1.00 16.88           C  0.040
ANISOU 1783  CG  ARG A 112     2273   1758   2382    338     20    238       C
ATOM   1784  CD  ARG A 112      36.186  -2.664  -4.997  1.00 20.60           C  0.044
ANISOU 1784  CD  ARG A 112     2585   2025   3216    430    199     42       C
ATOM   1785  NE  ARG A 112      35.605  -3.951  -4.659  1.00 22.47           N  0.046
ANISOU 1785  NE  ARG A 112     2786   2190   3561    646    225    -86       N
ATOM   1786  CZ  ARG A 112      36.130  -5.126  -4.986  1.00 24.49           C  0.048
ANISOU 1786  CZ  ARG A 112     2796   2492   4017    660    141    -36       C
ATOM   1787  NH1 ARG A 112      37.314  -5.223  -5.585  1.00 24.83           N  0.048
ANISOU 1787  NH1 ARG A 112     2657   2689   4088    672    196     -4       N
ATOM   1788  NH2 ARG A 112      35.459  -6.237  -4.690  1.00 25.33           N  0.049
ANISOU 1788  NH2 ARG A 112     2912   2488   4224    574    195     39       N
ATOM   1789  H   ARG A 112      32.337  -2.194  -8.033  1.00 13.84           H  0.036
ATOM   1790  HA  ARG A 112      34.471  -0.721  -8.104  1.00 15.73           H  0.038
ATOM   1791  HB2 ARG A 112      33.948  -1.893  -6.158  1.00 18.72           H  0.042
ATOM   1792  HB3 ARG A 112      34.208  -3.272  -6.907  1.00 18.72           H  0.042
ATOM   1793  HG2 ARG A 112      36.467  -2.898  -6.985  1.00 20.40           H  0.044
ATOM   1794  HG3 ARG A 112      36.287  -1.378  -6.558  1.00 20.40           H  0.044
ATOM   1795  HD2 ARG A 112      37.140  -2.710  -4.828  1.00 24.86           H  0.048
ATOM   1796  HD3 ARG A 112      35.780  -1.994  -4.425  1.00 24.86           H  0.048
ATOM   1797  HE  ARG A 112      34.868  -3.952  -4.215  1.00 27.11           H  0.050
ATOM   1798 HH11 ARG A 112      37.758  -4.514  -5.783  1.00 29.95           H  0.053
ATOM   1799 HH12 ARG A 112      37.633  -5.997  -5.782  1.00 29.95           H  0.053
ATOM   1800 HH21 ARG A 112      34.695  -6.186  -4.298  1.00 30.55           H  0.053
ATOM   1801 HH22 ARG A 112      35.790  -7.005  -4.889  1.00 30.55           H  0.053
ATOM   1802  N   ASN A 113      34.705  -3.443  -9.734  1.00 12.70           N  0.034
ANISOU 1802  N   ASN A 113     1429   1005   2393    280     22    221       N
ATOM   1803  CA  ASN A 113      35.578  -4.214 -10.596  1.00 13.46           C  0.035
ANISOU 1803  CA  ASN A 113     1417   1114   2583    199    187    121       C
ATOM   1804  C   ASN A 113      35.522  -3.809 -12.063  1.00 13.51           C  0.036
ANISOU 1804  C   ASN A 113     1353   1115   2666    373     51    187       C
ATOM   1805  O   ASN A 113      36.444  -4.105 -12.820  1.00 12.35           O  0.034
ANISOU 1805  O   ASN A 113     1329   1076   2287    328    120   -304       O
ATOM   1806  CB  ASN A 113      35.260  -5.695 -10.461  1.00 13.22           C  0.035
ANISOU 1806  CB  ASN A 113     1473   1157   2394    251    -12   -230       C
ATOM   1807  CG  ASN A 113      35.678  -6.256  -9.119  1.00 14.56           C  0.037
ANISOU 1807  CG  ASN A 113     1570   1136   2827    386     48    -76       C
ATOM   1808  OD1 ASN A 113      36.662  -5.791  -8.531  1.00 17.27           O  0.040
ANISOU 1808  OD1 ASN A 113     1722   1449   3393    327     28    187       O
ATOM   1809  ND2 ASN A 113      34.930  -7.238  -8.618  1.00 14.36           N  0.037
ANISOU 1809  ND2 ASN A 113     1447   1152   2856    522    182   -204       N
ATOM   1810  H   ASN A 113      33.930  -3.791  -9.602  1.00 15.39           H  0.038
ATOM   1811  HA  ASN A 113      36.492  -4.086 -10.298  1.00 16.30           H  0.039
ATOM   1812  HB2 ASN A 113      34.303  -5.824 -10.556  1.00 16.02           H  0.039
ATOM   1813  HB3 ASN A 113      35.732  -6.185 -11.152  1.00 16.02           H  0.039
ATOM   1814 HD21 ASN A 113      35.130  -7.585  -7.857  1.00 17.38           H  0.040
ATOM   1815 HD22 ASN A 113      34.252  -7.530  -9.059  1.00 17.38           H  0.040
ATOM   1816  N   ARG A 114      34.479  -3.140 -12.507  1.00 12.80           N  0.035
ANISOU 1816  N   ARG A 114     1527   1095   2240    343    196    116       N
ATOM   1817  CA  ARG A 114      34.340  -2.871 -13.929  1.00 12.51           C  0.034
ANISOU 1817  CA  ARG A 114     1469    998   2285    166     58    226       C
ATOM   1818  C   ARG A 114      34.069  -1.414 -14.254  1.00 12.63           C  0.034
ANISOU 1818  C   ARG A 114     1455    907   2436    119    199     31       C
```

```
ATOM   1819  O   ARG A 114      34.173  -1.029 -15.425  1.00 11.93           O  0.033
ANISOU 1819  O   ARG A 114     1551    817   2164    158    409     26         O
ATOM   1820  CB  ARG A 114      33.196  -3.709 -14.497  1.00 13.13           C  0.035
ANISOU 1820  CB  ARG A 114     1354   1007   2627     43    447    402         C
ATOM   1821  CG  ARG A 114      33.376  -5.179 -14.105  1.00 14.22           C  0.036
ANISOU 1821  CG  ARG A 114     1366   1094   2941    -24    329    436         C
ATOM   1822  CD  ARG A 114      32.286  -6.054 -14.635  1.00 14.63           C  0.037
ANISOU 1822  CD  ARG A 114     1305   1060   3195    198     79    525         C
ATOM   1823  NE  ARG A 114      32.332  -6.259 -16.070  1.00 13.50           N  0.036
ANISOU 1823  NE  ARG A 114     1232    992   2907    348     92    227         N
ATOM   1824  CZ  ARG A 114      31.495  -7.065 -16.722  1.00 14.00           C  0.036
ANISOU 1824  CZ  ARG A 114     1234   1189   2897    486     30     57         C
ATOM   1825  NH1 ARG A 114      30.584  -7.790 -16.079  1.00 13.23           N  0.035
ANISOU 1825  NH1 ARG A 114     1152   1245   2629    528    167    108         N
ATOM   1826  NH2 ARG A 114      31.582  -7.161 -18.045  1.00 14.15           N  0.036
ANISOU 1826  NH2 ARG A 114     1336   1135   2905    337   -133   -236         N
ATOM   1827  H   ARG A 114      33.843  -2.832 -12.017  1.00 15.51           H  0.038
ATOM   1828  HA  ARG A 114      35.151  -3.127 -14.393  1.00 15.16           H  0.038
ATOM   1829  HB2 ARG A 114      32.353  -3.394 -14.136  1.00 15.90           H  0.039
ATOM   1830  HB3 ARG A 114      33.197  -3.646 -15.465  1.00 15.90           H  0.039
ATOM   1831  HG2 ARG A 114      34.219  -5.501 -14.460  1.00 17.21           H  0.040
ATOM   1832  HG3 ARG A 114      33.370  -5.258 -13.139  1.00 17.21           H  0.040
ATOM   1833  HD2 ARG A 114      32.351  -6.924 -14.210  1.00 17.71           H  0.041
ATOM   1834  HD3 ARG A 114      31.431  -5.649 -14.422  1.00 17.71           H  0.041
ATOM   1835  HE  ARG A 114      32.930  -5.839 -16.523  1.00 16.35           H  0.039
ATOM   1836 HH11 ARG A 114      30.516  -7.739 -15.223  1.00 16.02           H  0.039
ATOM   1837 HH12 ARG A 114      30.057  -8.306 -16.521  1.00 16.02           H  0.039
ATOM   1838 HH21 ARG A 114      32.169  -6.700 -18.472  1.00 17.13           H  0.040
ATOM   1839 HH22 ARG A 114      31.049  -7.682 -18.474  1.00 17.13           H  0.040
ATOM   1840  N   CYS A 115      33.677  -0.607 -13.285  1.00 12.25           N  0.034
ANISOU 1840  N   CYS A 115     1429    906   2320     35     81   -213         N
ATOM   1841  CA  CYS A 115      33.402   0.807 -13.563  1.00 12.43           C  0.034
ANISOU 1841  CA  CYS A 115     1368    943   2412    -64    118   -325         C
ATOM   1842  C   CYS A 115      34.361   1.770 -12.886  1.00 10.57           C  0.031
ANISOU 1842  C   CYS A 115     1235   1003   1775    -20    364    190         C
ATOM   1843  O   CYS A 115      34.827   2.729 -13.515  1.00 13.08           O  0.035
ANISOU 1843  O   CYS A 115     1204   1154   2610     89    334    425         O
ATOM   1844  CB  CYS A 115      31.980   1.173 -13.113  1.00 12.35           C  0.034
ANISOU 1844  CB  CYS A 115     1267   1050   2376   -107     55   -306         C
ATOM   1845  SG  CYS A 115      30.721   0.173 -13.888  1.00 12.99           S  0.035
ANISOU 1845  SG  CYS A 115     1158   1151   2626    -81     94     27         S
ATOM   1846  H   CYS A 115      33.561  -0.840 -12.465  1.00 14.85           H  0.037
ATOM   1847  HA  CYS A 115      33.456   0.964 -14.518  1.00 15.07           H  0.038
ATOM   1848  HB2 CYS A 115      31.924   1.031 -12.158  1.00 14.97           H  0.037
ATOM   1849  HB3 CYS A 115      31.805   2.103 -13.326  1.00 14.97           H  0.037
ATOM   1850  N   LYS A 116      34.663   1.533 -11.608  1.00 12.11           N  0.034
ANISOU 1850  N   LYS A 116     1265   1247   2090    182     31    -63         N
ATOM   1851  CA  LYS A 116      35.540   2.391 -10.833  1.00 12.39           C  0.034
ANISOU 1851  CA  LYS A 116     1178   1297   2231     86     16    -92         C
ATOM   1852  C   LYS A 116      36.852   2.655 -11.564  1.00 14.09           C  0.036
ANISOU 1852  C   LYS A 116     1352   1495   2505   -199    -61   -154         C
ATOM   1853  O   LYS A 116      37.535   1.722 -12.012  1.00 13.02           O  0.035
ANISOU 1853  O   LYS A 116     1220   1479   2247   -331   -114    -88         O
ATOM   1854  CB  LYS A 116      35.819   1.693  -9.492  1.00 12.33           C  0.034
ANISOU 1854  CB  LYS A 116     1166   1298   2221    104     64    -72         C
ATOM   1855  CG  LYS A 116      36.676   2.444  -8.493  1.00 12.59           C  0.034
ANISOU 1855  CG  LYS A 116     1268   1233   2282     15    -12   -201         C
ATOM   1856  CD  LYS A 116      36.849   1.588  -7.245  1.00 12.43           C  0.034
ANISOU 1856  CD  LYS A 116     1231   1092   2400     34    129    148         C
ATOM   1857  CE  LYS A 116      37.379   2.414  -6.085  1.00 13.89           C  0.036
ANISOU 1857  CE  LYS A 116     1349   1195   2732    120    -79    355         C
ATOM   1858  NZ  LYS A 116      37.471   1.562  -4.832  1.00 14.02           N  0.036
ANISOU 1858  NZ  LYS A 116     1371   1065   2890     -6    -71    498         N
ATOM   1859  H   LYS A 116      34.361   0.862 -11.163  1.00 14.68           H  0.037
ATOM   1860  HA  LYS A 116      35.102   3.239 -10.660  1.00 15.01           H  0.037
ATOM   1861  HB2 LYS A 116      34.968   1.515  -9.062  1.00 14.95           H  0.037
ATOM   1862  HB3 LYS A 116      36.266   0.852  -9.677  1.00 14.95           H  0.037
ATOM   1863  HG2 LYS A 116      37.551   2.617  -8.875  1.00 15.25           H  0.038
ATOM   1864  HG3 LYS A 116      36.239   3.273  -8.244  1.00 15.25           H  0.038
ATOM   1865  HD2 LYS A 116      35.991   1.217  -6.988  1.00 15.06           H  0.038
ATOM   1866  HD3 LYS A 116      37.483   0.877  -7.428  1.00 15.06           H  0.038
ATOM   1867  HE2 LYS A 116      38.266   2.744  -6.300  1.00 16.81           H  0.040
ATOM   1868  HE3 LYS A 116      36.774   3.152  -5.912  1.00 16.81           H  0.040
ATOM   1869  HZ1 LYS A 116      37.782   2.049  -4.155  1.00 16.97           H  0.040
ATOM   1870  HZ2 LYS A 116      36.666   1.249  -4.617  1.00 16.97           H  0.040
ATOM   1871  HZ3 LYS A 116      38.021   0.876  -4.971  1.00 16.97           H  0.040
ATOM   1872  N   GLY A 117      37.229   3.928 -11.640  1.00 15.76           N  0.038
ANISOU 1872  N   GLY A 117     1551   1781   2657   -238    -58     96         N
```

```
ATOM   1873  CA  GLY A 117      38.471   4.307 -12.260  1.00 16.91           C  0.040
ANISOU 1873  CA  GLY A 117     1825   2010   2590   -416    -13    634       C
ATOM   1874  C   GLY A 117      38.450   4.341 -13.766  1.00 16.85           C  0.040
ANISOU 1874  C   GLY A 117     1808   2234   2360   -509    154    389       C
ATOM   1875  O   GLY A 117      39.447   4.736 -14.370  1.00 18.69           O  0.042
ANISOU 1875  O   GLY A 117     1960   2633   2510   -629    143    636       O
ATOM   1876  H   GLY A 117      36.772   4.590 -11.334  1.00 19.06           H  0.042
ATOM   1877  HA2 GLY A 117      38.722   5.190 -11.946  1.00 20.44           H  0.044
ATOM   1878  HA3 GLY A 117      39.162   3.684 -11.986  1.00 20.44           H  0.044
ATOM   1879  N   THR A 118      37.350   3.967 -14.398  1.00 15.21           N  0.038
ANISOU 1879  N   THR A 118     1556   1943   2279   -390    122    151       N
ATOM   1880  CA  THR A 118      37.267   4.012 -15.845  1.00 13.98           C  0.036
ANISOU 1880  CA  THR A 118     1440   1564   2310   -465    185    472       C
ATOM   1881  C   THR A 118      36.599   5.297 -16.271  1.00 13.34           C  0.035
ANISOU 1881  C   THR A 118     1605   1569   1894   -433    273    808       C
ATOM   1882  O   THR A 118      36.109   6.082 -15.456  1.00 14.89           O  0.037
ANISOU 1882  O   THR A 118     1616   1528   2513   -406    111    357       O
ATOM   1883  CB  THR A 118      36.445   2.829 -16.372  1.00 14.52           C  0.037
ANISOU 1883  CB  THR A 118     1408   1429   2679   -278    -65    318       C
ATOM   1884  OG1 THR A 118      35.028   3.016 -16.122  1.00 13.84           O  0.036
ANISOU 1884  OG1 THR A 118     1451   1267   2541   -195    -56    237       O
ATOM   1885  CG2 THR A 118      36.962   1.529 -15.748  1.00 16.01           C  0.039
ANISOU 1885  CG2 THR A 118     1317   1619   3147   -192   -153    243       C
ATOM   1886  H   THR A 118      36.636   3.683 -14.012  1.00 18.40           H  0.041
ATOM   1887  HA  THR A 118      38.155   3.974 -16.233  1.00 16.93           H  0.040
ATOM   1888  HB  THR A 118      36.581   2.765 -17.330  1.00 17.57           H  0.041
ATOM   1889  HG1 THR A 118      34.889   3.086 -15.297  1.00 16.76           H  0.040
ATOM   1890 HG21 THR A 118      36.560   0.766 -16.192  1.00 19.36           H  0.043
ATOM   1891 HG22 THR A 118      37.926   1.477 -15.843  1.00 19.36           H  0.043
ATOM   1892 HG23 THR A 118      36.737   1.499 -14.805  1.00 19.36           H  0.043
ATOM   1893  N   ASP A 119      36.564   5.480 -17.593  1.00 14.92           N  0.037
ANISOU 1893  N   ASP A 119     1628   1809   2230      2    262    645       N
ATOM   1894  CA  ASP A 119      35.885   6.688 -18.165  1.00 15.38           C  0.038
ANISOU 1894  CA  ASP A 119     1660   2017   2167    -67    295    809       C
ATOM   1895  C   ASP A 119      34.374   6.402 -18.185  1.00 14.18           C  0.036
ANISOU 1895  C   ASP A 119     1381   1987   2021    -83    180    418       C
ATOM   1896  O   ASP A 119      33.883   6.015 -19.214  1.00 16.41           O  0.039
ANISOU 1896  O   ASP A 119     1260   2266   2709   -262    114     33       O
ATOM   1897  CB  ASP A 119      36.481   7.060 -19.530  1.00 18.34           C  0.041
ANISOU 1897  CB  ASP A 119     1768   2306   2892    145    335    639       C
ATOM   1898  CG  ASP A 119      35.867   8.304 -20.155  1.00 21.42           C  0.045
ANISOU 1898  CG  ASP A 119     1949   2453   3734    202    348    847       C
ATOM   1899  OD1 ASP A 119      34.801   8.731 -19.689  1.00 22.58           O  0.046
ANISOU 1899  OD1 ASP A 119     2166   2632   3783    321    309    888       O
ATOM   1900  OD2 ASP A 119      36.456   8.832 -21.108  1.00 20.44           O  0.044
ANISOU 1900  OD2 ASP A 119     1939   2262   3564    142    367   1339       O
ATOM   1901  H   ASP A 119      36.908   4.949 -18.176  1.00 18.05           H  0.041
ATOM   1902  HA  ASP A 119      36.033   7.438 -17.568  1.00 18.61           H  0.042
ATOM   1903  HB2 ASP A 119      37.431   7.222 -19.422  1.00 22.15           H  0.046
ATOM   1904  HB3 ASP A 119      36.341   6.321 -20.143  1.00 22.15           H  0.046
ATOM   1905  N   VAL A 120      33.749   6.652 -17.044  1.00 14.13           N  0.036
ANISOU 1905  N   VAL A 120     1274   1894   2203   -167    585    420       N
ATOM   1906  CA  VAL A 120      32.313   6.382 -16.972  1.00 15.63           C  0.038
ANISOU 1906  CA  VAL A 120     1359   1791   2791   -215    336    536       C
ATOM   1907  C   VAL A 120      31.464   7.415 -17.701  1.00 15.32           C  0.038
ANISOU 1907  C   VAL A 120     1449   1615   2755   -224    328    444       C
ATOM   1908  O   VAL A 120      30.266   7.173 -17.935  1.00 15.21           O  0.038
ANISOU 1908  O   VAL A 120     1616   1610   2556   -126     95    281       O
ATOM   1909  CB  VAL A 120      31.808   6.199 -15.529  1.00 15.57           C  0.038
ANISOU 1909  CB  VAL A 120     1346   1875   2697   -197    549    582       C
ATOM   1910  CG1 VAL A 120      32.406   5.005 -14.911  1.00 15.52           C  0.038
ANISOU 1910  CG1 VAL A 120     1591   1816   2490   -347    528    872       C
ATOM   1911  CG2 VAL A 120      32.159   7.434 -14.713  1.00 17.74           C  0.041
ANISOU 1911  CG2 VAL A 120     1420   2066   3253   -188    287    486       C
ATOM   1912  H   VAL A 120      34.110   6.962 -16.329  1.00 17.11           H  0.040
ATOM   1913  HA  VAL A 120      32.159   5.537 -17.423  1.00 18.91           H  0.042
ATOM   1914  HB  VAL A 120      30.844   6.094 -15.533  1.00 18.84           H  0.042
ATOM   1915 HG11 VAL A 120      32.063   4.912 -14.008  1.00 18.78           H  0.042
ATOM   1916 HG12 VAL A 120      32.169   4.225 -15.437  1.00 18.78           H  0.042
ATOM   1917 HG13 VAL A 120      33.370   5.110 -14.890  1.00 18.78           H  0.042
ATOM   1918 HG21 VAL A 120      31.743   7.360 -13.840  1.00 21.43           H  0.045
ATOM   1919 HG22 VAL A 120      33.122   7.488 -14.614  1.00 21.43           H  0.045
ATOM   1920 HG23 VAL A 120      31.827   8.223 -15.168  1.00 21.43           H  0.045
ATOM   1921  N  AGLN A 121      32.066   8.541 -18.073  0.50 16.31           N  0.039
ANISOU 1921  N  AGLN A 121     1481   1591   3125   -281    213    150       N
ATOM   1922  CA AGLN A 121      31.402   9.559 -18.864  0.50 17.09           C  0.040
ANISOU 1922  CA AGLN A 121     1552   1617   3324   -248    317    123       C
ATOM   1923  C  AGLN A 121      31.027   9.039 -20.247  0.50 15.61           C  0.038
```

```
ANISOU 1923  C   AGLN A 121     1438   1576   2917   -276    422    471       C
ATOM   1924  O   AGLN A 121      30.149   9.612 -20.902  0.50 14.36           O  0.037
ANISOU 1924  O   AGLN A 121     1503   1578   2374   -362    426    789       O
ATOM   1925  CB AGLN A 121      32.388  10.715 -19.006  0.50 19.21           C  0.042
ANISOU 1925  CB AGLN A 121     1722   1699   3879   -146    261     10       C
ATOM   1926  CG AGLN A 121      32.785  11.487 -17.709  0.50 20.72           C  0.044
ANISOU 1926  CG AGLN A 121     1860   1749   4264   -161    487   -177       C
ATOM   1927  CD AGLN A 121      33.371  10.640 -16.539  0.50 21.25           C  0.045
ANISOU 1927  CD AGLN A 121     1856   1751   4466   -184    558   -265       C
ATOM   1928  OE1AGLN A 121      34.405   9.918 -16.654  0.50 20.53           O  0.044
ANISOU 1928  OE1AGLN A 121     1701   1688   4411   -264    632   -355       O
ATOM   1929  NE2AGLN A 121      32.718  10.775 -15.381  0.50 22.34           N  0.046
ANISOU 1929  NE2AGLN A 121     1928   1794   4765   -207    451   -144       N
ATOM   1930  H   AGLN A 121      32.883   8.727 -17.887  0.50 19.72           H  0.043
ATOM   1931  HA AGLN A 121      30.604   9.871 -18.410  0.50 20.66           H  0.044
ATOM   1932  HB2AGLN A 121      33.205  10.372 -19.395  0.50 23.20           H  0.047
ATOM   1933  HB3AGLN A 121      31.998  11.365 -19.612  0.50 23.20           H  0.047
ATOM   1934  HG2AGLN A 121      33.435  12.164 -17.939  0.50 25.01           H  0.048
ATOM   1935  HG3AGLN A 121      31.987  11.921 -17.368  0.50 25.01           H  0.048
ATOM   1936 HE21AGLN A 121      32.023  11.280 -15.335  0.50 26.95           H  0.050
ATOM   1937 HE22AGLN A 121      32.982  10.342 -14.686  0.50 26.95           H  0.050
ATOM   1938  N   BGLN A 121      32.029   8.576 -18.041  0.50 16.76           N  0.040
ANISOU 1938  N   BGLN A 121     1429   1651   3288   -223     78    376       N
ATOM   1939  CA BGLN A 121      31.289   9.525 -18.862  0.50 17.93           C  0.041
ANISOU 1939  CA BGLN A 121     1466   1733   3615   -128     61    541       C
ATOM   1940  C   BGLN A 121      30.857   8.885 -20.169  0.50 16.80           C  0.040
ANISOU 1940  C   BGLN A 121     1366   1618   3399   -140    290    576       C
ATOM   1941  O   BGLN A 121      29.749   9.159 -20.656  0.50 16.89           O  0.040
ANISOU 1941  O   BGLN A 121     1299   1607   3510    -74    380    689       O
ATOM   1942  CB BGLN A 121      32.156  10.757 -19.112  0.50 20.19           C  0.043
ANISOU 1942  CB BGLN A 121     1611   1955   4104     88   -272    843       C
ATOM   1943  CG BGLN A 121      31.563  11.857 -19.989  0.50 22.72           C  0.046
ANISOU 1943  CG BGLN A 121     1765   2191   4676    185   -310    889       C
ATOM   1944  CD BGLN A 121      30.584  12.751 -19.264  0.50 24.62           C  0.048
ANISOU 1944  CD BGLN A 121     1836   2475   5044    239   -303    811       C
ATOM   1945  OE1BGLN A 121      30.816  13.170 -18.129  0.50 25.48           O  0.049
ANISOU 1945  OE1BGLN A 121     1859   2595   5227    298   -367    911       O
ATOM   1946  NE2BGLN A 121      29.495  13.088 -19.941  0.50 25.44           N  0.049
ANISOU 1946  NE2BGLN A 121     1850   2525   5291    287   -222    702       N
ATOM   1947  H   BGLN A 121      32.819   8.830 -17.814  0.50 20.26           H  0.044
ATOM   1948  HA BGLN A 121      30.493   9.807 -18.385  0.50 21.67           H  0.045
ATOM   1949  HB2BGLN A 121      32.365  11.158 -18.254  0.50 24.37           H  0.048
ATOM   1950  HB3BGLN A 121      32.978  10.466 -19.537  0.50 24.37           H  0.048
ATOM   1951  HG2BGLN A 121      32.285  12.416 -20.316  0.50 27.41           H  0.051
ATOM   1952  HG3BGLN A 121      31.098  11.454 -20.738  0.50 27.41           H  0.051
ATOM   1953 HE21BGLN A 121      29.370  12.782 -20.735  0.50 30.68           H  0.054
ATOM   1954 HE22BGLN A 121      28.905  13.598 -19.579  0.50 30.68           H  0.054
ATOM   1955  N    ALA A 122      31.673   7.964 -20.702  1.00 16.04           N  0.039
ANISOU 1955  N    ALA A 122     1435   1584   3077   -193    397    440       N
ATOM   1956  CA   ALA A 122      31.320   7.358 -21.974  1.00 17.24           C  0.040
ANISOU 1956  CA   ALA A 122     1536   1694   3318    -74    421    216       C
ATOM   1957  C    ALA A 122      29.940   6.722 -21.946  1.00 16.54           C  0.039
ANISOU 1957  C    ALA A 122     1548   1639   3098    -87    215    130       C
ATOM   1958  O    ALA A 122      29.326   6.571 -23.003  1.00 16.39           O  0.039
ANISOU 1958  O    ALA A 122     1696   1665   2868   -278    231    126       O
ATOM   1959  CB   ALA A 122      32.361   6.296 -22.327  1.00 19.02           C  0.042
ANISOU 1959  CB   ALA A 122     1630   1905   3692     41    435    -39       C
ATOM   1960  HA   ALA A 122      31.329   8.037 -22.667  1.00 20.83           H  0.044
ATOM   1961  HB1 ALA A 122      32.124   5.892 -23.177  1.00 22.97           H  0.046
ATOM   1962  HB2 ALA A 122      33.232   6.718 -22.392  1.00 22.97           H  0.046
ATOM   1963  HB3 ALA A 122      32.371   5.621 -21.631  1.00 22.97           H  0.046
ATOM   1964  H   AALA A 122      32.318   7.571 -20.291  0.50 19.40           H  0.043
ATOM   1965  H   BALA A 122      32.409   7.687 -20.355  0.50 19.40           H  0.043
ATOM   1966  N    TRP A 123      29.457   6.352 -20.759  1.00 15.63           N  0.038
ANISOU 1966  N    TRP A 123     1422   1682   2836   -142    340    274       N
ATOM   1967  CA   TRP A 123      28.160   5.710 -20.636  1.00 15.15           C  0.038
ANISOU 1967  CA   TRP A 123     1330   1715   2712     35    192    281       C
ATOM   1968  C    TRP A 123      27.021   6.658 -20.886  1.00 15.42           C  0.038
ANISOU 1968  C    TRP A 123     1315   1642   2901    107    -27    399       C
ATOM   1969  O    TRP A 123      25.915   6.193 -21.167  1.00 17.90           O  0.041
ANISOU 1969  O    TRP A 123     1459   1876   3467    202   -110    301       O
ATOM   1970  CB   TRP A 123      27.996   5.033 -19.274  1.00 15.06           C  0.038
ANISOU 1970  CB   TRP A 123     1427   1790   2507    161    481    170       C
ATOM   1971  CG   TRP A 123      28.837   3.864 -19.193  1.00 15.51           C  0.038
ANISOU 1971  CG   TRP A 123     1603   1723   2566    172    420    372       C
ATOM   1972  CD1 TRP A 123      30.029   3.760 -18.561  1.00 16.20           C  0.039
ANISOU 1972  CD1 TRP A 123     1729   1695   2732    128    337    649       C
ATOM   1973  CD2 TRP A 123      28.589   2.612 -19.819  1.00 15.34           C  0.038
```

```
ANISOU 1973  CD2 TRP A 123     1681   1632   2516     80    537    596       C
ATOM   1974  NE1 TRP A 123      30.553   2.505 -18.757  1.00 16.08           N  0.039
ANISOU 1974  NE1 TRP A 123     1720   1796   2592    210    327    183       N
ATOM   1975  CE2 TRP A 123      29.678   1.780 -19.519  1.00 15.87           C  0.039
ANISOU 1975  CE2 TRP A 123     1747   1633   2651    114    342    382       C
ATOM   1976  CE3 TRP A 123      27.555   2.110 -20.611  1.00 17.99           C  0.041
ANISOU 1976  CE3 TRP A 123     1946   1656   3235     91    453    415       C
ATOM   1977  CZ2 TRP A 123      29.762   0.472 -19.972  1.00 15.82           C  0.038
ANISOU 1977  CZ2 TRP A 123     1770   1553   2686    154    102    153       C
ATOM   1978  CZ3 TRP A 123      27.631   0.808 -21.055  1.00 18.68           C  0.042
ANISOU 1978  CZ3 TRP A 123     1982   1675   3440     38    311    496       C
ATOM   1979  CH2 TRP A 123      28.725  -0.003 -20.737  1.00 18.13           C  0.041
ANISOU 1979  CH2 TRP A 123     1907   1612   3369     48    159    266       C
ATOM   1980  H   TRP A 123      29.866   6.463 -20.011  1.00 18.91           H  0.042
ATOM   1981  HA  TRP A 123      28.104   5.014 -21.309  1.00 18.33           H  0.041
ATOM   1982  HB2 TRP A 123      28.257   5.649 -18.572  1.00 18.23           H  0.041
ATOM   1983  HB3 TRP A 123      27.073   4.758 -19.158  1.00 18.23           H  0.041
ATOM   1984  HD1 TRP A 123      30.441   4.442 -18.082  1.00 19.59           H  0.043
ATOM   1985  HE1 TRP A 123      31.295   2.216 -18.434  1.00 19.44           H  0.043
ATOM   1986  HE3 TRP A 123      26.823   2.642 -20.826  1.00 21.74           H  0.045
ATOM   1987  HZ2 TRP A 123      30.488  -0.067 -19.756  1.00 19.13           H  0.042
ATOM   1988  HZ3 TRP A 123      26.944   0.464 -21.579  1.00 22.57           H  0.046
ATOM   1989  HH2 TRP A 123      28.752  -0.878 -21.052  1.00 21.90           H  0.045
ATOM   1990  N   ILE A 124      27.264   7.964 -20.773  1.00 16.63           N  0.039
ANISOU 1990  N   ILE A 124     1500   1675   3144    104   -325    682       N
ATOM   1991  CA  ILE A 124      26.243   8.955 -21.052  1.00 16.56           C  0.039
ANISOU 1991  CA  ILE A 124     1722   1848   2723     87     -6    788       C
ATOM   1992  C   ILE A 124      26.557   9.782 -22.285  1.00 18.80           C  0.042
ANISOU 1992  C   ILE A 124     2104   2191   2849    191    139    772       C
ATOM   1993  O   ILE A 124      25.767  10.667 -22.637  1.00 18.96           O  0.042
ANISOU 1993  O   ILE A 124     2000   2141   3063    238    112    479       O
ATOM   1994  CB  ILE A 124      25.935   9.854 -19.837  1.00 17.79           C  0.041
ANISOU 1994  CB  ILE A 124     1699   1915   3145    -53    410    437       C
ATOM   1995  CG1 ILE A 124      27.133  10.732 -19.497  1.00 20.00           C  0.043
ANISOU 1995  CG1 ILE A 124     1684   2063   3851     82    380    113       C
ATOM   1996  CG2 ILE A 124      25.525   9.019 -18.613  1.00 17.34           C  0.040
ANISOU 1996  CG2 ILE A 124     1765   1876   2948    -90    488    402       C
ATOM   1997  CD1 ILE A 124      26.802  11.828 -18.472  1.00 20.32           C  0.044
ANISOU 1997  CD1 ILE A 124     1563   2085   4074    248    392     87       C
ATOM   1998  H   ILE A 124      28.019   8.299 -20.534  1.00 20.11           H  0.043
ATOM   1999  HA  ILE A 124      25.421   8.481 -21.249  1.00 20.03           H  0.043
ATOM   2000  HB  ILE A 124      25.193  10.433 -20.071  1.00 21.50           H  0.045
ATOM   2001 HG12 ILE A 124      27.834  10.176 -19.123  1.00 24.15           H  0.048
ATOM   2002 HG13 ILE A 124      27.453  11.172 -20.299  1.00 24.15           H  0.048
ATOM   2003 HG21 ILE A 124      25.196   9.612 -17.920  1.00 20.96           H  0.044
ATOM   2004 HG22 ILE A 124      24.827   8.399 -18.875  1.00 20.96           H  0.044
ATOM   2005 HG23 ILE A 124      26.298   8.529 -18.292  1.00 20.96           H  0.044
ATOM   2006 HD11 ILE A 124      27.586  12.383 -18.338  1.00 24.54           H  0.048
ATOM   2007 HD12 ILE A 124      26.070  12.366 -18.812  1.00 24.54           H  0.048
ATOM   2008 HD13 ILE A 124      26.546  11.412 -17.634  1.00 24.54           H  0.048
ATOM   2009  N   ARG A 125      27.670   9.507 -22.970  1.00 21.81           N  0.045
ANISOU 2009  N   ARG A 125     2421   2551   3316    405    398    897       N
ATOM   2010  CA  ARG A 125      28.059  10.325 -24.117  1.00 24.06           C  0.047
ANISOU 2010  CA  ARG A 125     2781   2926   3436    603    515   1211       C
ATOM   2011  C   ARG A 125      27.016  10.245 -25.227  1.00 23.15           C  0.047
ANISOU 2011  C   ARG A 125     2915   3065   2816    594    464   1356       C
ATOM   2012  O   ARG A 125      26.550   9.160 -25.583  1.00 23.22           O  0.047
ANISOU 2012  O   ARG A 125     3113   3091   2621    645    433   1297       O
ATOM   2013  CB  ARG A 125      29.418   9.866 -24.634  1.00 26.99           C  0.050
ANISOU 2013  CB  ARG A 125     3116   3133   4005    645    892   1451       C
ATOM   2014  CG  ARG A 125      30.097  10.785 -25.645  1.00 30.29           C  0.053
ANISOU 2014  CG  ARG A 125     3449   3318   4740    711    945   1401       C
ATOM   2015  CD  ARG A 125      31.536  10.340 -25.852  1.00 33.55           C  0.056
ANISOU 2015  CD  ARG A 125     3775   3498   5473    787    651   1347       C
ATOM   2016  NE  ARG A 125      31.554   8.997 -26.421  1.00 36.14           N  0.058
ANISOU 2016  NE  ARG A 125     4061   3664   6007    803    453   1155       N
ATOM   2017  CZ  ARG A 125      32.403   8.032 -26.083  1.00 38.32           C  0.060
ANISOU 2017  CZ  ARG A 125     4343   3813   6404    771    242   1055       C
ATOM   2018  NH1 ARG A 125      33.502   8.282 -25.388  1.00 39.32           N  0.061
ANISOU 2018  NH1 ARG A 125     4409   3882   6651    772    236   1130       N
ATOM   2019  NH2 ARG A 125      32.142   6.781 -26.454  1.00 38.87           N  0.060
ANISOU 2019  NH2 ARG A 125     4479   3901   6389    732    173    940       N
ATOM   2020  H   ARG A 125      28.210   8.861 -22.794  1.00 26.33           H  0.050
ATOM   2021  HA  ARG A 125      28.136  11.251 -23.838  1.00 29.02           H  0.052
ATOM   2022  HB2 ARG A 125      30.017   9.776 -23.877  1.00 32.54           H  0.055
ATOM   2023  HB3 ARG A 125      29.305   9.002 -25.059  1.00 32.54           H  0.055
ATOM   2024  HG2 ARG A 125      29.632  10.735 -26.495  1.00 36.49           H  0.058
ATOM   2025  HG3 ARG A 125      30.099  11.695 -25.309  1.00 36.49           H  0.058
```

```
ATOM   2026  HD2 ARG A 125      31.979  10.944 -26.469  1.00 40.40           H  0.061
ATOM   2027  HD3 ARG A 125      31.996  10.325 -24.998  1.00 40.40           H  0.061
ATOM   2028  HE  ARG A 125      30.968   8.815 -27.024  1.00 43.52           H  0.064
ATOM   2029 HH11 ARG A 125      33.679   9.084 -25.133  1.00 47.34           H  0.067
ATOM   2030 HH12 ARG A 125      34.038   7.640 -25.187  1.00 47.34           H  0.067
ATOM   2031 HH21 ARG A 125      31.436   6.607 -26.913  1.00 46.79           H  0.066
ATOM   2032 HH22 ARG A 125      32.692   6.150 -26.256  1.00 46.79           H  0.066
ATOM   2033  N   GLY A 126      26.634  11.405 -25.761  1.00 24.92           N  0.048
ANISOU 2033  N   GLY A 126     2855   3206   3407    538    403   1227       N
ATOM   2034  CA  GLY A 126      25.681  11.477 -26.838  1.00 24.61           C  0.048
ANISOU 2034  CA  GLY A 126     2781   3246   3325    523    481   1181       C
ATOM   2035  C   GLY A 126      24.233  11.548 -26.411  1.00 23.92           C  0.047
ANISOU 2035  C   GLY A 126     2662   3141   3287    538    326   1226       C
ATOM   2036  O   GLY A 126      23.392  11.962 -27.212  1.00 24.77           O  0.048
ANISOU 2036  O   GLY A 126     2609   3272   3531    427     63   1085       O
ATOM   2037  H   GLY A 126      26.925  12.172 -25.504  1.00 30.05           H  0.053
ATOM   2038  HA2 GLY A 126      25.872  12.263 -27.374  1.00 29.68           H  0.053
ATOM   2039  HA3 GLY A 126      25.787  10.696 -27.404  1.00 29.68           H  0.053
ATOM   2040  N   CYS A 127      23.921  11.171 -25.173  1.00 22.64           N  0.046
ANISOU 2040  N   CYS A 127     2606   2934   3063    515    565   1057       N
ATOM   2041  CA  CYS A 127      22.530  11.135 -24.731  1.00 22.96           C  0.046
ANISOU 2041  CA  CYS A 127     2531   2712   3482    383    390    835       C
ATOM   2042  C   CYS A 127      21.951  12.537 -24.557  1.00 24.90           C  0.048
ANISOU 2042  C   CYS A 127     2738   2866   3858    578    212   1211       C
ATOM   2043  O   CYS A 127      22.623  13.448 -24.058  1.00 24.45           O  0.048
ANISOU 2043  O   CYS A 127     2510   2750   4030    559    292   1276       O
ATOM   2044  CB  CYS A 127      22.428  10.416 -23.396  1.00 20.84           C  0.044
ANISOU 2044  CB  CYS A 127     2370   2298   3252    224    189    596       C
ATOM   2045  SG  CYS A 127      23.190   8.789 -23.384  1.00 20.03           S  0.043
ANISOU 2045  SG  CYS A 127     2323   2000   3286     55     47    560       S
ATOM   2046  H   CYS A 127      24.491  10.933 -24.574  1.00 27.32           H  0.051
ATOM   2047  HA  CYS A 127      21.993  10.657 -25.383  1.00 27.71           H  0.051
ATOM   2048  HB2 CYS A 127      22.867  10.953 -22.718  1.00 25.16           H  0.049
ATOM   2049  HB3 CYS A 127      21.491  10.306 -23.172  1.00 25.16           H  0.049
ATOM   2050  N   ARG A 128      20.676  12.709 -24.922  1.00 27.35           N  0.051
ANISOU 2050  N   ARG A 128     3121   3146   4125    692    121   1322       N
ATOM   2051  CA  ARG A 128      19.985  13.939 -24.561  1.00 31.59           C  0.054
ANISOU 2051  CA  ARG A 128     3670   3495   4838    739    219   1214       C
ATOM   2052  C   ARG A 128      19.583  13.848 -23.091  1.00 31.45           C  0.054
ANISOU 2052  C   ARG A 128     3901   3469   4577    705    442   1125       C
ATOM   2053  O   ARG A 128      18.865  12.926 -22.687  1.00 31.96           O  0.055
ANISOU 2053  O   ARG A 128     3912   3475   4755    649    384   1125       O
ATOM   2054  CB  ARG A 128      18.795  14.223 -25.479  1.00 35.72           C  0.058
ANISOU 2054  CB  ARG A 128     3974   3836   5762    890    160   1198       C
ATOM   2055  CG  ARG A 128      17.820  13.089 -25.667  1.00 39.50           C  0.061
ANISOU 2055  CG  ARG A 128     4262   4162   6585   1096     86   1368       C
ATOM   2056  CD  ARG A 128      16.448  13.622 -26.096  1.00 42.53           C  0.063
ANISOU 2056  CD  ARG A 128     4514   4437   7210   1284     24   1547       C
ATOM   2057  NE  ARG A 128      16.485  14.568 -27.213  1.00 44.91           N  0.065
ANISOU 2057  NE  ARG A 128     4685   4670   7709   1482    131   1598       N
ATOM   2058  CZ  ARG A 128      15.783  15.695 -27.276  1.00 46.21           C  0.066
ANISOU 2058  CZ  ARG A 128     4798   4826   7934   1666    272   1596       C
ATOM   2059  NH1 ARG A 128      15.045  16.111 -26.259  1.00 46.77           N  0.066
ANISOU 2059  NH1 ARG A 128     4832   4914   8026   1754    278   1564       N
ATOM   2060  NH2 ARG A 128      15.819  16.422 -28.391  1.00 46.49           N  0.066
ANISOU 2060  NH2 ARG A 128     4814   4878   7974   1724    357   1586       N
ATOM   2061  H   ARG A 128      20.205  12.143 -25.366  1.00 32.97           H  0.056
ATOM   2062  HA  ARG A 128      20.604  14.681 -24.650  1.00 38.06           H  0.060
ATOM   2063  HB2 ARG A 128      18.300  14.972 -25.113  1.00 43.01           H  0.063
ATOM   2064  HB3 ARG A 128      19.136  14.459 -26.356  1.00 43.01           H  0.063
ATOM   2065  HG2 ARG A 128      18.147  12.495 -26.360  1.00 47.55           H  0.067
ATOM   2066  HG3 ARG A 128      17.715  12.609 -24.831  1.00 47.55           H  0.067
ATOM   2067  HD2 ARG A 128      15.898  12.871 -26.369  1.00 51.19           H  0.069
ATOM   2068  HD3 ARG A 128      16.046  14.056 -25.330  1.00 51.19           H  0.069
ATOM   2069  HE  ARG A 128      16.999  14.381 -27.877  1.00 54.04           H  0.071
ATOM   2070 HH11 ARG A 128      15.003  15.657 -25.532  1.00 56.28           H  0.073
ATOM   2071 HH12 ARG A 128      14.602  16.845 -26.328  1.00 56.28           H  0.073
ATOM   2072 HH21 ARG A 128      16.297  16.164 -29.057  1.00 55.94           H  0.072
ATOM   2073 HH22 ARG A 128      15.373  17.156 -28.440  1.00 55.94           H  0.072
ATOM   2074  N   LEU A 129      20.098  14.769 -22.284  1.00 31.70           N  0.054
ANISOU 2074  N   LEU A 129     4165   3372   4508    755    719   1145       N
ATOM   2075  CA  LEU A 129      19.950  14.702 -20.835  1.00 32.77           C  0.055
ANISOU 2075  CA  LEU A 129     4456   3458   4535    840    616    985       C
ATOM   2076  C   LEU A 129      19.582  16.067 -20.299  1.00 34.55           C  0.057
ANISOU 2076  C   LEU A 129     4754   3542   4832    911    672    930       C
ATOM   2077  O   LEU A 129      19.653  17.045 -21.041  1.00 35.63           O  0.058
ANISOU 2077  O   LEU A 129     4886   3565   5089    926    688    880       O
ATOM   2078  CB  LEU A 129      21.242  14.193 -20.171  1.00 31.78           C  0.055
```

```
ANISOU 2078  CB  LEU A 129     4470   3426   4179    822    610   1161       C
ATOM   2079  CG  LEU A 129      21.641  12.744 -20.446  1.00 31.82          C  0.055
ANISOU 2079  CG  LEU A 129     4457   3440   4194    812    569   1117       C
ATOM   2080  CD1 LEU A 129      23.027  12.410 -19.884  1.00 32.21          C  0.055
ANISOU 2080  CD1 LEU A 129     4409   3509   4319    835    604   1095       C
ATOM   2081  CD2 LEU A 129      20.587  11.813 -19.851  1.00 32.76          C  0.055
ANISOU 2081  CD2 LEU A 129     4502   3461   4484    779    550    945       C
ATOM   2082  OXT LEU A 129      19.202  16.209 -19.136  1.00 35.72          O  0.058
ANISOU 2082  OXT LEU A 129     4873   3609   5089    967    577    771       O
ATOM   2083  H   LEU A 129      20.544  15.452 -22.556  1.00 38.19          H  0.060
ATOM   2084  HA  LEU A 129      19.231  14.090 -20.613  1.00 39.47          H  0.061
ATOM   2085  HB2 LEU A 129      21.975  14.753 -20.471  1.00 38.28          H  0.060
ATOM   2086  HB3 LEU A 129      21.143  14.285 -19.210  1.00 38.28          H  0.060
ATOM   2087  HG  LEU A 129      21.663  12.599 -21.405  1.00 38.34          H  0.060
ATOM   2088 HD11 LEU A 129      23.204  11.467 -20.027  1.00 38.80          H  0.060
ATOM   2089 HD12 LEU A 129      23.692  12.946 -20.344  1.00 38.80          H  0.060
ATOM   2090 HD13 LEU A 129      23.039  12.609 -18.935  1.00 38.80          H  0.060
ATOM   2091 HD21 LEU A 129      20.905  10.898 -19.911  1.00 39.46          H  0.061
ATOM   2092 HD22 LEU A 129      20.443  12.053 -18.922  1.00 39.46          H  0.061
ATOM   2093 HD23 LEU A 129      19.761  11.910 -20.350  1.00 39.46          H  0.061
HETATM 2094 CL    CL A1131      29.529  10.659 -15.901  0.69 15.66         Cl  0.038
ANISOU 2094 CL    CL A1131     2216   1209   2525   -810    320     95      Cl
HETATM 2095 NA    NA A1138      15.665  -9.452   3.571  1.00 21.79         Na1+  0.045
ANISOU 2095 NA    NA A1138     2711   2000   3566   -691   -237   -557      Na1+
HETATM 2096  C1  RII A1139      22.977  -1.224   8.690  0.55 21.33          C  0.045
ANISOU 2096  C1  RII A1139     2811   2327   2967   -833    675   -308       C
HETATM 2097  C2  RII A1139      23.518   1.335   8.633  0.55 23.26          C  0.047
ANISOU 2097  C2  RII A1139     2920   2352   3567   -776    584   -443       C
HETATM 2098  C3  RII A1139      22.239   0.317   6.573  0.55 21.88          C  0.045
ANISOU 2098  C3  RII A1139     2763   2362   3189   -665    534   -334       C
HETATM 2099  C4  RII A1139      25.378  -1.657   5.230  0.55 19.02          C  0.042
ANISOU 2099  C4  RII A1139     2340   1858   3029   -966    344   -141       C
HETATM 2100  C5  RII A1139      24.657  -3.657   5.054  0.55 16.59          C  0.039
ANISOU 2100  C5  RII A1139     2185   1821   2296  -1006    344    -76       C
HETATM 2101  C6  RII A1139      23.965  -3.081   6.025  0.55 17.56          C  0.041
ANISOU 2101  C6  RII A1139     2304   1839   2528   -978    396   -122       C
HETATM 2102  C7  RII A1139      26.985  -0.082   7.875  0.55 19.64          C  0.043
ANISOU 2102  C7  RII A1139     2631   2174   2657   -943    735    708       C
HETATM 2103  C8  RII A1139      27.398  -1.271   9.626  0.55 20.17          C  0.043
ANISOU 2103  C8  RII A1139     2677   2124   2862  -1040    522    625       C
HETATM 2104  C9  RII A1139      26.068  -1.272   9.406  0.55 19.66          C  0.043
ANISOU 2104  C9  RII A1139     2702   2149   2618   -990    719    638       C
HETATM 2105  N1  RII A1139      24.418  -1.762   6.123  0.55 18.86          N  0.042
ANISOU 2105  N1  RII A1139     2477   1937   2752   -907    424   -251       N
HETATM 2106  N2  RII A1139      25.545  -2.755   4.537  0.55 18.23          N  0.041
ANISOU 2106  N2  RII A1139     2240   1861   2826   -925    404     17       N
HETATM 2107  N3  RII A1139      25.805  -0.510   8.280  0.55 15.93          N  0.039
ANISOU 2107  N3  RII A1139     2197   2078   1779   -622    732    587       N
HETATM 2108  N4  RII A1139      27.948  -0.534   8.678  0.55 20.59          N  0.044
ANISOU 2108  N4  RII A1139     2710   2163   2950   -981    447    671       N
HETATM 2109  O1  RII A1139      22.443  -1.865   9.491  0.55 22.64          O  0.046
ANISOU 2109  O1  RII A1139     2899   2441   3262   -761    601   -435       O
HETATM 2110  O2  RII A1139      23.314   2.214   9.334  0.55 24.71          O  0.048
ANISOU 2110  O2  RII A1139     2987   2493   3910   -749    810   -258       O
HETATM 2111  O3  RII A1139      21.260   0.597   6.089  0.55 21.99          O  0.045
ANISOU 2111  O3  RII A1139     2712   2436   3206   -640    466   -574       O
HETATM 2112 RE1  RII A1139      23.859  -0.133   7.441  0.55 21.65         Re  0.045
ANISOU 2112 RE1  RII A1139     2792   2288   3146   -783    551   -278      Re
HETATM 2113  H21 RII A1139      26.116  -2.890   3.908  0.55 22.03          H  0.045
HETATM 2114  H41 RII A1139      25.858  -0.877   5.072  0.55 22.97          H  0.046
HETATM 2115  H42 RII A1139      28.788  -0.375   8.588  0.55 24.85          H  0.048
HETATM 2116  H51 RII A1139      24.552  -4.533   4.767  0.55 20.06          H  0.043
HETATM 2117  H61 RII A1139      23.288  -3.472   6.529  0.55 21.22          H  0.045
HETATM 2118  H71 RII A1139      27.127   0.461   7.134  0.55 23.72          H  0.047
HETATM 2119  H81 RII A1139      27.844  -1.708  10.314  0.55 24.35          H  0.048
HETATM 2120  H91 RII A1139      25.433  -1.711   9.922  0.55 23.74          H  0.047
HETATM 2121  C1  RII A1140      35.514  11.996 -23.464  0.60 18.32          C  0.041
ANISOU 2121  C1  RII A1140     1731   2180   3051    -84     88    821       C
HETATM 2122  C2  RII A1140      35.987   9.373 -23.877  0.60 19.15          C  0.042
ANISOU 2122  C2  RII A1140     1896   2085   3295   -142     16    405       C
HETATM 2123  C3  RII A1140      33.955  10.294 -22.409  0.60 18.66          C  0.042
ANISOU 2123  C3  RII A1140     1682   2155   3253   -239    220    653       C
HETATM 2124  C4  RII A1140      35.915  11.390 -19.341  0.60 18.54          C  0.042
ANISOU 2124  C4  RII A1140     1989   2095   2960     41    136    512       C
HETATM 2125  C5  RII A1140      35.479  13.533 -19.286  0.60 17.31          C  0.040
ANISOU 2125  C5  RII A1140     1990   2036   2549    205     65    518       C
HETATM 2126  C6  RII A1140      35.467  13.118 -20.537  0.60 17.45          C  0.040
ANISOU 2126  C6  RII A1140     1987   2007   2638    102    157    558       C
```

```
HETATM 2127  C7  RII A1140      38.872  10.210 -21.568  0.60 17.49           C  0.040
ANISOU 2127  C7  RII A1140     1849   1891   2907   -312    457    598       C
HETATM 2128  C8  RII A1140      40.026  11.568 -22.789  0.60 17.12           C  0.040
ANISOU 2128  C8  RII A1140     1832   1740   2932   -296    336    434       C
HETATM 2129  C9  RII A1140      38.732  11.697 -23.124  0.60 16.89           C  0.040
ANISOU 2129  C9  RII A1140     1828   1773   2817   -190    203    472       C
HETATM 2130  N1  RII A1140      35.751  11.757 -20.591  0.60 16.96           N  0.040
ANISOU 2130  N1  RII A1140     1891   1965   2587    -23    131    691       N
HETATM 2131  N2  RII A1140      35.750  12.427 -18.528  0.60 18.03           N  0.041
ANISOU 2131  N2  RII A1140     2074   2096   2680    188    203    329       N
HETATM 2132  N3  RII A1140      38.005  10.833 -22.325  0.60 16.92           N  0.040
ANISOU 2132  N3  RII A1140     1758   1909   2762   -257    336    509       N
HETATM 2133  N4  RII A1140      40.098  10.667 -21.836  0.60 17.51           N  0.040
ANISOU 2133  N4  RII A1140     1853   1864   2935   -348    301    569       N
HETATM 2134  O1  RII A1140      35.317  12.924 -24.138  0.60 19.42           O  0.043
ANISOU 2134  O1  RII A1140     1670   2381   3328    -75    124    597       O
HETATM 2135  O2  RII A1140      36.077   8.696 -24.786  0.60 19.89           O  0.043
ANISOU 2135  O2  RII A1140     2036   2233   3290   -131    -34    462       O
HETATM 2136  O3  RII A1140      32.847  10.148 -22.485  0.60 19.39           O  0.043
ANISOU 2136  O3  RII A1140     1733   2247   3387   -222    115    619       O
HETATM 2137 RE1  RII A1140      35.851  10.503 -22.346  0.60 17.86          Re  0.041
ANISOU 2137 RE1  RII A1140     1732   2015   3040   -203    214    657      Re
HETATM 2138  H21 RII A1140      35.829  12.411 -17.672  0.60 21.79           H  0.045
HETATM 2139  H41 RII A1140      36.106  10.525 -19.061  0.60 22.40           H  0.046
HETATM 2140  H42 RII A1140      40.821  10.403 -21.452  0.60 21.16           H  0.045
HETATM 2141  H51 RII A1140      35.327  14.397 -18.982  0.60 20.92           H  0.044
HETATM 2142  H61 RII A1140      35.301  13.658 -21.276  0.60 21.09           H  0.044
HETATM 2143  H71 RII A1140      38.667   9.568 -20.927  0.60 21.14           H  0.044
HETATM 2144  H81 RII A1140      40.739  12.037 -23.157  0.60 20.69           H  0.044
HETATM 2145  H91 RII A1140      38.385  12.269 -23.769  0.60 20.42           H  0.044
HETATM 2146  C1  RI3 A1143       9.036   7.868 -14.604  0.75 30.27           C  0.053
ANISOU 2146  C1  RI3 A1143     2042   3040   6420     68   -587   1777       C
HETATM 2147  C2  RI3 A1143      11.604   8.488 -14.678  0.75 26.76           C  0.050
ANISOU 2147  C2  RI3 A1143     1794   2872   5503    146   -301   1770       C
HETATM 2148  C3  RI3 A1143      10.856   6.075 -13.877  0.75 27.47           C  0.051
ANISOU 2148  C3  RI3 A1143     1832   2895   5710     87   -398   1777       C
HETATM 2149  C7  RI3 A1143      10.469  10.393 -11.438  0.75 30.81           C  0.054
ANISOU 2149  C7  RI3 A1143     2194   3015   6496    111   -421   1210       C
HETATM 2150  C8  RI3 A1143       9.196  11.893 -12.314  0.75 30.65           C  0.054
ANISOU 2150  C8  RI3 A1143     2177   2992   6476     98   -378   1118       C
HETATM 2151  C9  RI3 A1143       9.189  10.796 -13.100  0.75 30.63           C  0.054
ANISOU 2151  C9  RI3 A1143     2145   3022   6473    101   -407   1216       C
HETATM 2152  N3  RI3 A1143      10.008   9.832 -12.536  0.75 30.32           N  0.053
ANISOU 2152  N3  RI3 A1143     2063   3002   6454     99   -402   1400       N
HETATM 2153  N4  RI3 A1143       9.982  11.629 -11.293  0.75 30.91           N  0.054
ANISOU 2153  N4  RI3 A1143     2211   3018   6517    117   -392   1068       N
HETATM 2154  O1  RI3 A1143       8.195   7.851 -15.400  0.75 31.43           O  0.054
ANISOU 2154  O1  RI3 A1143     2178   3177   6586     43   -553   1688       O
HETATM 2155  O2  RI3 A1143      12.267   8.856 -15.512  0.75 26.46           O  0.050
ANISOU 2155  O2  RI3 A1143     1871   2950   5232    310   -417   1582       O
HETATM 2156  O3  RI3 A1143      11.154   5.050 -14.240  0.75 27.05           O  0.050
ANISOU 2156  O3  RI3 A1143     1946   2920   5412      6   -658   1806       O
HETATM 2157  O4  RI3 A1143       9.101   7.229 -11.703  0.75 29.06           O  0.052
ANISOU 2157  O4  RI3 A1143     1807   2844   6392     82    -50   1764       O
HETATM 2158 RE1  RI3 A1143      10.427   7.840 -13.322  0.75 29.40          Re  0.052
ANISOU 2158 RE1  RI3 A1143     1855   2915   6402     56   -230   1801      Re
HETATM 2159  H42 RI3 A1143      10.160  12.163 -10.643  0.75 37.24           H  0.059
HETATM 2160  H71 RI3 A1143      11.050   9.985 -10.839  0.75 37.12           H  0.059
HETATM 2161  H81 RI3 A1143       8.728  12.682 -12.461  0.75 36.93           H  0.059
HETATM 2162  H91 RI3 A1143       8.715  10.702 -13.893  0.75 36.91           H  0.059
TER
HETATM 2163 CL    CL D   1      14.826 -12.809 -13.274  1.00 26.11          Cl  0.049
ANISOU 2163 CL    CL D   1     2855   3202   3864  -1072   -277    358      Cl
HETATM 2164 CL    CL D   2       7.437  -7.312   1.850  1.00 37.19          Cl  0.059
ANISOU 2164 CL    CL D   2     2618   6027   5487   -458    919    975      Cl
HETATM 2165 CL    CL D   3      25.067  -0.415 -24.070  1.00 38.40          Cl  0.060
ANISOU 2165 CL    CL D   3     4404   4632   5553  -1376   1394  -1635      Cl
HETATM 2166 RE    RE B   1      25.289  15.334 -15.358  0.13 20.83          Re  0.044
ANISOU 2166 RE    RE B   1     2107   1680   4128    -16    -97   1088      Re
HETATM 2167 RE    RE B   2      10.573  10.573 -18.509  0.09 17.76          Re  0.041
ANISOU 2167 RE    RE B   2     1619   1619   3510     18   -382    382      Re
HETATM 2168 RE    RE B   3      16.134  15.892 -19.538  0.15 25.41          Re  0.049
ANISOU 2168 RE    RE B   3     2405   2575   4674    472   -479     33      Re
HETATM 2169 RE    RE B   4      32.145   7.824  -1.570  0.25 19.17          Re  0.042
ANISOU 2169 RE    RE B   4     1556   2205   3522    -82    -61   -330      Re
HETATM 2170 RE    RE B   5      21.593 -15.095   5.953  0.08 17.56          Re  0.041
HETATM 2171  O   HOH S   2      16.476 -15.225  -1.594  1.00 13.63           O  0.036
ANISOU 2171  O   HOH S   2     1401   1680   2097   -797    234   -196       O
```

```
HETATM 2172  O   HOH S   3      17.915  -2.971  -8.355  1.00 12.76           O  0.035
ANISOU 2172  O   HOH S   3     1453    837   2557   -363    267     55         O
HETATM 2173  O   HOH S   5      34.142  -4.885 -17.887  1.00 15.24           O  0.038
ANISOU 2173  O   HOH S   5     1131   2220   2440    730    279    176         O
HETATM 2174  O   HOH S   6      14.343 -11.187   2.569  1.00 16.53           O  0.039
ANISOU 2174  O   HOH S   6     1635   2438   2208   -710    207   -392         O
HETATM 2175  O   HOH S   7      26.565  -2.641   1.695  1.00 21.66           O  0.045
ANISOU 2175  O   HOH S   7     2353   2528   3349      1    139   1671         O
HETATM 2176  O   HOH S   8      12.559  -7.211 -18.737  1.00 15.84           O  0.039
ANISOU 2176  O   HOH S   8     1650   1556   2811   -806    218    329         O
HETATM 2177  O   HOH S   9      29.005   5.695   1.619  1.00 18.15           O  0.041
ANISOU 2177  O   HOH S   9     1373   2130   3394   -387    801   -525         O
HETATM 2178  O   HOH S  10      29.367  -8.685 -19.234  1.00 16.49           O  0.039
ANISOU 2178  O   HOH S  10     1797   1380   3089    654   -665   -499         O
HETATM 2179  O   HOH S  11      37.179  -1.101 -12.328  1.00 15.33           O  0.038
ANISOU 2179  O   HOH S  11     1233   1594   2998    -52    167   -368         O
HETATM 2180  O   HOH S  12      31.837  10.240  -3.905  1.00 19.66           O  0.043
ANISOU 2180  O   HOH S  12     1631   2555   3285   -428   -593   -384         O
HETATM 2181  O   HOH S  13       7.390 -12.115   3.003  1.00 20.01           O  0.043
ANISOU 2181  O   HOH S  13     2301   2320   2980  -1020    745  -1146         O
HETATM 2182  O   HOH S  14      35.911   6.896 -12.899  1.00 21.88           O  0.045
ANISOU 2182  O   HOH S  14     1840   2910   3566   -904    633    422         O
HETATM 2183  O   HOH S  15      12.293  -0.259  -9.093  1.00 23.15           O  0.047
ANISOU 2183  O   HOH S  15     2233   2301   4259  -1190     23    183         O
HETATM 2184  O   HOH S  16      40.009   0.709  -8.488  1.00 19.90           O  0.043
ANISOU 2184  O   HOH S  16     2081   2365   3117   -513   -145   -453         O
HETATM 2185  O   HOH S  17      11.583 -19.982  -3.376  1.00 18.88           O  0.042
ANISOU 2185  O   HOH S  17     2370   2042   2762    432  -1013    -95         O
HETATM 2186  O   HOH S  18      13.271  -0.743 -11.660  1.00 21.02           O  0.044
ANISOU 2186  O   HOH S  18     2380   1738   3869    298   -317    614         O
HETATM 2187  O   HOH S  19      40.038   3.432  -9.580  1.00 20.78           O  0.044
ANISOU 2187  O   HOH S  19     2513   1973   3411   -414    103   -479         O
HETATM 2188  O   HOH S  20      12.760   1.521  -2.449  1.00 23.26           O  0.047
ANISOU 2188  O   HOH S  20     2052   2260   4524   -340    220    187         O
HETATM 2189  O   HOH S  21      32.112  14.010  -7.264  1.00 21.39           O  0.045
ANISOU 2189  O   HOH S  21     1612   2894   3623   -832   -241    507         O
HETATM 2190  O   HOH S  22      28.127  -6.573  -7.951  1.00 24.18           O  0.048
ANISOU 2190  O   HOH S  22     3766   2581   2842   -401    607     40         O
HETATM 2191  O   HOH S  23      33.500   2.319 -18.257  1.00 20.31           O  0.044
ANISOU 2191  O   HOH S  23     1741   3066   2912    247   -213    269         O
HETATM 2192  O   HOH S  24      27.665  -6.343  -5.022  1.00 24.69           O  0.048
ANISOU 2192  O   HOH S  24     3022   2657   3701  -1613    364    359         O
HETATM 2193  O   HOH S  25      19.274  -7.442 -20.779  1.00 22.48           O  0.046
ANISOU 2193  O   HOH S  25     2604   3033   2904   -787   -438    -87         O
HETATM 2194  O   HOH S  26      28.159   1.710   0.813  1.00 22.57           O  0.046
ANISOU 2194  O   HOH S  26     3220   2138   3219  -1347    980   -573         O
HETATM 2195  O   HOH S  27      19.752 -17.107 -10.147  1.00 19.67           O  0.043
ANISOU 2195  O   HOH S  27     1726   2807   2941   -317   -167   -549         O
HETATM 2196  O   HOH S  28      38.197   3.904 -19.389  1.00 20.62           O  0.044
ANISOU 2196  O   HOH S  28     2420   1537   3878    -46   1251    318         O
HETATM 2197  O   HOH S  29      20.158  13.604 -11.102  1.00 25.87           O  0.049
ANISOU 2197  O   HOH S  29     2819   1982   5030    562   -529    160         O
HETATM 2198  O   HOH S  30      15.145  -2.624 -11.281  1.00 23.73           O  0.047
ANISOU 2198  O   HOH S  30     1725   4234   3056    836    259   1354         O
HETATM 2199  O   HOH S  31      34.304   5.910 -10.985  1.00 20.95           O  0.044
ANISOU 2199  O   HOH S  31     1921   3380   2658  -1143      9     87         O
HETATM 2200  O   HOH S  32      20.688   4.319 -25.891  1.00 24.46           O  0.048
ANISOU 2200  O   HOH S  32     3559   2843   2894   -229   -717    577         O
HETATM 2201  O   HOH S  33      39.520  -0.226  -5.778  1.00 21.34           O  0.045
ANISOU 2201  O   HOH S  33     2526   1690   3891    859    308   -567         O
HETATM 2202  O   HOH S  34      27.472  -8.466 -12.270  1.00 19.93           O  0.043
ANISOU 2202  O   HOH S  34     2465   1893   3212    242   -662    -15         O
HETATM 2203  O   HOH S  35       8.266  -0.800  -7.615  1.00 26.15           O  0.049
ANISOU 2203  O   HOH S  35     2225   2293   5416    -57    680    376         O
HETATM 2204  O   HOH S  36      10.263  -6.662   6.419  1.00 24.30           O  0.048
ANISOU 2204  O   HOH S  36     2860   2781   3594  -1353    707    761         O
HETATM 2205  O   HOH S  37      14.700   9.430  -3.948  1.00 24.46           O  0.048
ANISOU 2205  O   HOH S  37     1563   2780   4951    449    761    108         O
HETATM 2206  O   HOH S  38      22.853  -5.437  -2.856  1.00 26.10           O  0.049
ANISOU 2206  O   HOH S  38     2001   2526   5391   -673   -161    782         O
HETATM 2207  O   HOH S  39      18.930  -9.705 -19.474  1.00 20.25           O  0.044
ANISOU 2207  O   HOH S  39     1937   2265   3491   -607    527   -671         O
HETATM 2208  O   HOH S  40      19.985  -9.603 -17.066  1.00 24.41           O  0.048
ANISOU 2208  O   HOH S  40     3646   1716   3913   -500    507   -344         O
HETATM 2209  O   HOH S  41      13.536   2.459   0.023  1.00 20.93           O  0.044
ANISOU 2209  O   HOH S  41     2912   1413   3626   -118    609   -301         O
HETATM 2210  O   HOH S  42      34.660  -1.921 -17.958  1.00 20.13           O  0.043
ANISOU 2210  O   HOH S  42     2194   3050   2405    625     37   -155         O
```

```
HETATM 2211  O   HOH S  43       6.522  -3.035 -14.002  1.00 27.55           O  0.051
ANISOU 2211  O   HOH S  43      1609   4083   4776    -71   -331   1254       O
HETATM 2212  O   HOH S  44       8.844  -4.713 -17.411  1.00 26.12           O  0.049
ANISOU 2212  O   HOH S  44      2125   2734   5064   -402     65   1800       O
HETATM 2213  O   HOH S  47      17.327 -16.186 -10.316  1.00 22.40           O  0.046
ANISOU 2213  O   HOH S  47      2334   1926   4252   -261    498    267       O
HETATM 2214  O   HOH S  48      24.044 -10.226  -9.403  1.00 21.45           O  0.045
ANISOU 2214  O   HOH S  48      1666   1731   4754   -161   -986    916       O
HETATM 2215  O   HOH S  49      14.400 -14.378   9.253  0.50 25.31           O  0.049
ANISOU 2215  O   HOH S  49      2791   2619   4207    958    778  -1034       O
HETATM 2216  O   HOH S  50       7.487  -5.314  -9.651  1.00 25.32           O  0.049
ANISOU 2216  O   HOH S  50      2853   3050   3718    772   -408     60       O
HETATM 2217  O   HOH S  51      11.638   7.988 -20.088  1.00 24.85           O  0.048
ANISOU 2217  O   HOH S  51      1458   2889   5095   -286    -13  -1090       O
HETATM 2218  O   HOH S  52      39.040  -4.418  -8.673  1.00 28.96           O  0.052
ANISOU 2218  O   HOH S  52      1559   4813   4632   -301   -229   1351       O
HETATM 2219  O   HOH S  53      33.355  -0.286 -19.601  1.00 23.67           O  0.047
ANISOU 2219  O   HOH S  53      2943   2461   3590     -5   -105    602       O
HETATM 2220  O   HOH S  54       9.417   1.542  -8.404  1.00 25.82           O  0.049
ANISOU 2220  O   HOH S  54      2050   2743   5017   -879   -532    985       O
HETATM 2221  O   HOH S  55      29.839   2.790 -22.920  1.00 30.30           O  0.053
ANISOU 2221  O   HOH S  55      3957   3969   3587    522   1899   1417       O
HETATM 2222  O   HOH S  56      10.439 -11.997   5.982  1.00 33.65           O  0.056
ANISOU 2222  O   HOH S  56      4699   5004   3085  -1278    230   -684       O
HETATM 2223  O   HOH S  57      18.163   4.678 -25.107  1.00 25.86           O  0.049
ANISOU 2223  O   HOH S  57      4276   2205   3344   -432   -661    765       O
HETATM 2224  O   HOH S  58      33.575   9.092 -11.935  1.00 25.01           O  0.048
ANISOU 2224  O   HOH S  58      1997   3527   3978  -1234    252  -1008       O
HETATM 2225  O   HOH S  59      20.973  -4.287 -22.600  1.00 28.42           O  0.052
ANISOU 2225  O   HOH S  59      5989   2495   2314   -702  -1391    218       O
HETATM 2226  O   HOH S  60      19.292  -7.992  13.650  1.00 31.38           O  0.054
ANISOU 2226  O   HOH S  60      3358   5651   2915     83   -955  -1047       O
HETATM 2227  O   HOH S  61      24.119  13.646  -2.626  1.00 27.57           O  0.051
ANISOU 2227  O   HOH S  61      3395   2514   4567    435    189   -732       O
HETATM 2228  O   HOH S  62      38.701  -1.626  -9.206  1.00 29.81           O  0.053
ANISOU 2228  O   HOH S  62      2064   2812   6452    -83    607   -140       O
HETATM 2229  O   HOH S  64      34.375   3.657 -20.337  1.00 29.11           O  0.052
ANISOU 2229  O   HOH S  64      4348   1881   4832    961  -1092   -733       O
HETATM 2230  O   HOH S  66      21.414  -8.451  11.206  1.00 39.45           O  0.061
ANISOU 2230  O   HOH S  66      5509   3248   6231   -563   -781   2326       O
HETATM 2231  O   HOH S  67      19.817  -1.973  13.343  1.00 28.03           O  0.051
ANISOU 2231  O   HOH S  67      4098   3536   3015    584   -671    246       O
HETATM 2232  O   HOH S  68      18.503  11.244  -0.287  1.00 36.63           O  0.059
ANISOU 2232  O   HOH S  68      5831   3603   4484    441   2118    -25       O
HETATM 2233  O   HOH S  69       5.562  -8.735  -3.684  1.00 25.13           O  0.048
ANISOU 2233  O   HOH S  69      1977   2791   4779    -23    961    853       O
HETATM 2234  O   HOH S  71      26.174  -6.698 -18.925  1.00 33.42           O  0.056
ANISOU 2234  O   HOH S  71      3391   3218   6089   -607    808   -918       O
HETATM 2235  O   HOH S  72      10.240   3.049 -16.979  1.00 29.85           O  0.053
ANISOU 2235  O   HOH S  72      3272   2407   5662     13   -668   1708       O
HETATM 2236  O   HOH S  76      11.954  -3.786 -22.681  1.00 28.63           O  0.052
ANISOU 2236  O   HOH S  76      2839   4695   3345    117  -1831      1       O
HETATM 2237  O   HOH S  77      40.031   1.202 -13.038  1.00 26.88           O  0.050
ANISOU 2237  O   HOH S  77      1409   3430   5374     -9    234   -425       O
HETATM 2238  O   HOH S  78      36.746   2.055 -20.390  1.00 27.43           O  0.051
ANISOU 2238  O   HOH S  78      3654   2595   4172   1503   -256    807       O
HETATM 2239  O   HOH S  79      40.220   2.249 -18.057  1.00 29.31           O  0.052
ANISOU 2239  O   HOH S  79      2411   3357   5369   1102    567    506       O
HETATM 2240  O   HOH S  80       7.409   0.624  -5.010  1.00 32.96           O  0.056
ANISOU 2240  O   HOH S  80      3445   3560   5517   1491    505   -532       O
HETATM 2241  O   HOH S  81      24.882  -6.631  -6.291  1.00 25.64           O  0.049
ANISOU 2241  O   HOH S  81      1666   3728   4350   -171   -535  -1590       O
HETATM 2242  O   HOH S  82      29.233   8.173   2.955  1.00 38.56           O  0.060
ANISOU 2242  O   HOH S  82      2654   4836   7161  -1054   -238  -1521       O
HETATM 2243  O   HOH S  84      10.463   3.049  -2.627  1.00 35.10           O  0.057
ANISOU 2243  O   HOH S  84      2487   5787   5061   1283    506   -207       O
HETATM 2244  O   HOH S  86      17.629  12.553  -3.663  1.00 35.50           O  0.058
ANISOU 2244  O   HOH S  86      3710   3535   6243   1662   -861  -1525       O
HETATM 2245  O   HOH S  87      25.715  -8.582  -8.069  1.00 25.11           O  0.048
ANISOU 2245  O   HOH S  87      3062   1792   4686   -342   -435    513       O
HETATM 2246  O   HOH S  91      29.729  13.707  -9.918  1.00 37.86           O  0.060
ANISOU 2246  O   HOH S  91      7167   2018   5199   -822    716    105       O
HETATM 2247  O   HOH S  93      23.635  10.683   1.212  1.00 32.23           O  0.055
ANISOU 2247  O   HOH S  93      5492   2087   4667   -684    864   -654       O
HETATM 2248  O   HOH S  96      13.818  -9.481   4.917  1.00 27.45           O  0.051
ANISOU 2248  O   HOH S  96      3373   3243   3813  -1635   1351   -911       O
HETATM 2249  O   HOH S  97      21.551   0.115  11.771  1.00 37.41           O  0.059
ANISOU 2249  O   HOH S  97      3828   6027   4361  -1790   1844   -898       O
```

```
HETATM 2250  O   HOH S  98      27.985  -8.993  -9.405  1.00 32.38           O  0.055
ANISOU 2250  O   HOH S  98     3789   2701   5815    326   1517    227       O
HETATM 2251  O   HOH S 100      13.047 -16.254   6.173  1.00 31.19           O  0.054
ANISOU 2251  O   HOH S 100     2322   5857   3671   -903   -293   2418       O
HETATM 2252  O   HOH S 101      23.760  -7.327  12.375  1.00 29.47           O  0.053
ANISOU 2252  O   HOH S 101     3660   3911   3628    199    661   1424       O
HETATM 2253  O   HOH S 102      22.407   4.716   4.226  1.00 32.09           O  0.055
ANISOU 2253  O   HOH S 102     4605   3764   3825    -93   1734      8       O
HETATM 2254  O   HOH S 103      41.456  -5.721  -8.567  1.00 33.04           O  0.056
ANISOU 2254  O   HOH S 103     4671   3568   4314  -2161    388    833       O
HETATM 2255  O   HOH S 104      40.067  -2.883 -11.205  1.00 32.18           O  0.055
ANISOU 2255  O   HOH S 104     3685   2795   5747    874  -2182  -1732       O
HETATM 2256  O   HOH S 107      21.650   6.041 -28.054  1.00 37.24           O  0.059
ANISOU 2256  O   HOH S 107     5014   6427   2710  -2049   -312   -119       O
HETATM 2257  O   HOH S 108       7.929 -13.712   5.256  1.00 35.53           O  0.058
ANISOU 2257  O   HOH S 108     2951   6764   3785  -1767   1100  -1956       O
HETATM 2258  O   HOH S 111      13.418   7.753  -7.167  1.00 27.66           O  0.051
ANISOU 2258  O   HOH S 111     2692   2872   4946     -5    662    369       O
HETATM 2259  O   HOH S 118      16.724   6.910  -0.019  1.00 33.31           O  0.056
ANISOU 2259  O   HOH S 118     4678   2690   5289    898   1534  -1046       O
HETATM 2260  O   HOH S 121      14.879   9.123  -1.487  1.00 39.05           O  0.060
ANISOU 2260  O   HOH S 121     5131   4600   5106   1806    947  -1345       O
HETATM 2261  O   HOH S 128      15.294  13.127  -6.547  1.00 34.73           O  0.057
ANISOU 2261  O   HOH S 128     4359   2979   5857    877   1437   -815       O
HETATM 2262  O   HOH S 130       8.879 -16.353   5.273  1.00 28.02           O  0.051
ANISOU 2262  O   HOH S 130     3090   3909   3649  -1440    245    428       O
HETATM 2263  O   HOH S 132      20.038   7.860   1.542  1.00 35.81           O  0.058
ANISOU 2263  O   HOH S 132     3396   2752   7457    120   1521  -1395       O
HETATM 2264  O   HOH S 138      21.417 -20.880  -3.294  1.00 34.24           O  0.057
ANISOU 2264  O   HOH S 138     5121   2249   5638    695  -1808    -47       O
HETATM 2265  O   HOH S 139      28.196  10.594 -29.708  1.00 42.64           O  0.063
ANISOU 2265  O   HOH S 139     5783   4538   5879   1445   1914   -832       O
HETATM 2266  O   HOH S 140      29.718  -2.511   1.135  1.00 20.48           O  0.044
ANISOU 2266  O   HOH S 140     1578   2537   3665    -21  -1167    213       O
HETATM 2267  O   HOH S 144      36.633  11.278 -16.259  1.00 35.54           O  0.058
ANISOU 2267  O   HOH S 144     5037   4108   4360  -1732  -1676   1451       O
HETATM 2268  O   HOH S 151       8.256  -4.456   2.860  1.00 25.91           O  0.049
ANISOU 2268  O   HOH S 151     1460   4334   4052   -748    639    150       O
HETATM 2269  O   HOH S 152      18.723  10.270 -23.092  1.00 31.39           O  0.054
ANISOU 2269  O   HOH S 152     2923   3649   5353    649    269   2314       O
HETATM 2270  O   HOH S 153      19.308  10.324 -26.500  1.00 31.12           O  0.054
ANISOU 2270  O   HOH S 153     2990   2965   5869   -637   -917   -234       O
HETATM 2271  O   HOH S 154      12.137  10.321  -7.765  1.00 37.38           O  0.059
ANISOU 2271  O   HOH S 154     4424   4125   5653   1028   2763    297       O
HETATM 2272  O   HOH S 155      22.336 -10.095 -20.515  1.00 33.09           O  0.056
HETATM 2273  O   HOH S 156      21.875 -14.586   3.568  1.00 40.88           O  0.062
HETATM 2274  O   HOH S 157      25.286 -11.188  -5.571  1.00 30.79           O  0.054
HETATM 2275 BR    BR C   1      13.155 -13.347   4.656  0.65 27.79          Br  0.051
END
```
