# Supplementary material for: Time-series analysis of rhenium(I) organometallic covalent binding to a model protein for drug development
Source: IUCrJ. 2024 Apr 19;11(Pt 3):359–73. doi: 10.1107/S2052252524002598 (PMC11067751; doi:10.1107/S2052252524002598)
Supplement: Supplementary file 5 [file m-11-00359-sup5.zip › Week 14 - V1_22Ffj6/Lab_Week14_141_refine_9_dpi.pdf]

```
REMARK   3
REMARK   3 REFINEMENT.
REMARK   3   PROGRAM     : PHENIX (1.20.1_4487: ???)
REMARK   3   AUTHORS     : Adams,Afonine,Bunkoczi,Burnley,Chen,Dar,Davis,
REMARK   3               : Draizen,Echols,Gildea,Gros,Grosse-Kunstleve,Headd,
REMARK   3               : Hintze,Hung,Ioerger,Liebschner,McCoy,McKee,Moriarty,
REMARK   3               : Oeffner,Poon,Read,Richardson,Richardson,Sacchettini,
REMARK   3               : Sauter,Sobolev,Storoni,Terwilliger,Williams,Zwart
REMARK   3
REMARK   3  X-RAY DATA.
REMARK   3
REMARK   3  REFINEMENT TARGET : ML
REMARK   3
REMARK   3  DATA USED IN REFINEMENT.
REMARK   3   RESOLUTION RANGE HIGH (ANGSTROMS) : 1.40
REMARK   3   RESOLUTION RANGE LOW  (ANGSTROMS) : 22.64
REMARK   3   MIN(FOBS/SIGMA_FOBS)              : 1.33
REMARK   3   COMPLETENESS FOR RANGE        (%) : 98.21
REMARK   3   NUMBER OF REFLECTIONS             : 45734
REMARK   3   NUMBER OF REFLECTIONS (NON-ANOMALOUS) : 24582
REMARK   3
REMARK   3  FIT TO DATA USED IN REFINEMENT.
REMARK   3   R VALUE     (WORKING + TEST SET) : 0.1635
REMARK   3   R VALUE            (WORKING SET) : 0.1605
REMARK   3   FREE R VALUE                     : 0.1971
REMARK   3   FREE R VALUE TEST SET SIZE   (%) : 8.17
REMARK   3   FREE R VALUE TEST SET COUNT      : 3737
REMARK   3
REMARK   3  FIT TO DATA USED IN REFINEMENT (IN BINS).
REMARK   3   BIN  RESOLUTION RANGE  COMPL.    NWORK NFREE   RWORK  RFREE  CCWORK CCFREE
REMARK   3     1  22.64 -    4.18    1.00     1584   143  0.1644 0.1638   0.922  0.948
REMARK   3     2   4.18 -    3.33    0.98     1548   140  0.1543 0.1607   0.930  0.882
REMARK   3     3   3.32 -    2.90    1.00     1587   147  0.1436 0.1872   0.945  0.886
REMARK   3     4   2.90 -    2.64    0.99     1549   138  0.1464 0.1872   0.941  0.923
REMARK   3     5   2.64 -    2.45    0.99     1576   136  0.1386 0.1806   0.947  0.894
REMARK   3     6   2.45 -    2.31    0.99     1563   136  0.1362 0.1687   0.947  0.909
REMARK   3     7   2.31 -    2.19    0.97     1548   136  0.1360 0.1829   0.954  0.901
REMARK   3     8   2.19 -    2.10    0.99     1577   141  0.1308 0.1818   0.953  0.904
REMARK   3     9   2.10 -    2.01    0.96     1525   138  0.1440 0.1965   0.944  0.900
REMARK   3    10   2.01 -    1.95    0.99     1534   142  0.1387 0.1762   0.948  0.912
REMARK   3    11   1.95 -    1.88    0.97     1530   134  0.1395 0.2091   0.948  0.896
REMARK   3    12   1.88 -    1.83    1.00     1594   144  0.1374 0.1957   0.950  0.903
REMARK   3    13   1.83 -    1.78    1.00     1587   139  0.1294 0.1612   0.955  0.943
REMARK   3    14   1.78 -    1.74    1.00     1570   145  0.1334 0.1901   0.944  0.884
REMARK   3    15   1.74 -    1.70    1.00     1581   143  0.1434 0.2045   0.935  0.880
REMARK   3    16   1.70 -    1.66    1.00     1597   135  0.1456 0.1873   0.931  0.884
REMARK   3    17   1.66 -    1.63    1.00     1583   146  0.1471 0.1798   0.933  0.930
REMARK   3    18   1.63 -    1.60    1.00     1576   137  0.1604 0.2187   0.912  0.811
REMARK   3    19   1.60 -    1.57    1.00     1607   140  0.1782 0.2280   0.889  0.793
REMARK   3    20   1.57 -    1.54    1.00     1553   142  0.1828 0.2413   0.888  0.790
REMARK   3    21   1.54 -    1.52    1.00     1602   142  0.2218 0.2799   0.819  0.782
REMARK   3    22   1.52 -    1.50    1.00     1558   138  0.2344 0.3194   0.800  0.650
REMARK   3    23   1.50 -    1.47    1.00     1586   141  0.2396 0.2763   0.772  0.785
REMARK   3    24   1.47 -    1.45    1.00     1583   140  0.2524 0.2810   0.735  0.726
REMARK   3    25   1.45 -    1.43    1.00     1559   137  0.2833 0.3167   0.688  0.585
REMARK   3    26   1.43 -    1.41    1.00     1599   138  0.2914 0.2921   0.658  0.682
REMARK   3    27   1.41 -    1.40    0.71     1141    99  0.3126 0.3648   0.562  0.265
REMARK   3
REMARK   3  BULK SOLVENT MODELLING.
REMARK   3   METHOD USED        : FLAT BULK SOLVENT MODEL
REMARK   3   SOLVENT RADIUS     : 1.10
REMARK   3   SHRINKAGE RADIUS   : 0.90
REMARK   3   GRID STEP FACTOR   : 4.00
REMARK   3
REMARK   3  ERROR ESTIMATES.
REMARK   3   COORDINATE ERROR (MAXIMUM-LIKELIHOOD BASED)     : 0.16
REMARK   3   PHASE ERROR (DEGREES, MAXIMUM-LIKELIHOOD BASED) : 19.60
REMARK   3
REMARK   3  STRUCTURE FACTORS CALCULATION ALGORITHM : FFT
REMARK   3  B VALUES.
REMARK   3   FROM WILSON PLOT           (A**2) : 9.48
REMARK   3
REMARK   3  GEOMETRY RESTRAINTS LIBRARY: GEOSTD + MONOMER LIBRARY + CDL V1.2
REMARK   3  DEVIATIONS FROM IDEAL VALUES - RMSD. RMSZ FOR BONDS AND ANGLES.
REMARK   3    BOND      :  0.013   0.070   1114  Z= 0.834
REMARK   3    ANGLE     :  1.305   6.942   1517  Z= 0.772
REMARK   3    CHIRALITY :  0.082   0.278    148
REMARK   3    PLANARITY :  0.015   0.100    197
```

```
REMARK   3    DIHEDRAL  : 15.073  82.834    398
REMARK   3    MIN NONBONDED DISTANCE : 2.135
REMARK   3
REMARK   3  MOLPROBITY STATISTICS.
REMARK   3    ALL-ATOM CLASHSCORE : 2.85
REMARK   3    RAMACHANDRAN PLOT:
REMARK   3      OUTLIERS :  0.00 %
REMARK   3      ALLOWED  :  1.57 %
REMARK   3      FAVORED  : 98.43 %
REMARK   3    ROTAMER OUTLIERS :  0.92 %
REMARK   3    CBETA DEVIATIONS :  0.00 %
REMARK   3    PEPTIDE PLANE:
REMARK   3      CIS-PROLINE     : 0.00 %
REMARK   3      CIS-GENERAL     : 0.00 %
REMARK   3      TWISTED PROLINE : 0.00 %
REMARK   3      TWISTED GENERAL : 0.00 %
REMARK   3
REMARK   3  RAMA-Z (RAMACHANDRAN PLOT Z-SCORE):
REMARK   3  INTERPRETATION: BAD |RAMA-Z| > 3; SUSPICIOUS 2 < |RAMA-Z| < 3; GOOD |RAMA-Z| < 2.
REMARK   3  SCORES FOR WHOLE/HELIX/SHEET/LOOP ARE SCALED INDEPENDENTLY;
REMARK   3  THEREFORE, THE VALUES ARE NOT RELATED IN A SIMPLE MANNER.
REMARK   3    WHOLE: -0.01 (0.70), RESIDUES: 137
REMARK   3    HELIX: -0.66 (0.66), RESIDUES: 45
REMARK   3    SHEET: -0.84 (1.03), RESIDUES: 14
REMARK   3    LOOP :  0.79 (0.77), RESIDUES: 78
REMARK   3
REMARK   3                  min    max   mean <Bi,j>   iso aniso
REMARK   3     Overall:    4.28  48.07  14.63   1.82     0  1235
REMARK   3     Protein:    4.28  48.07  13.18   1.83     0  1042
REMARK   3     Water:      7.45  37.91  23.98    N/A     0   135
REMARK   3     Other:     10.71  32.22  18.92    N/A     0    58
REMARK   3     Chain  A:   4.28  48.07  13.44    N/A     0  1094
REMARK   3     Chain  C:  19.29  27.77  23.53    N/A     0     2
REMARK   3     Chain  B:  13.85  24.58  19.06    N/A     0     4
REMARK   3     Chain  S:   7.45  37.91  23.98    N/A     0   135
REMARK   3     Histogram:
REMARK   3         Values      Number of atoms
REMARK   3       4.28 - 8.66        263
REMARK   3       8.66 - 13.04       387
REMARK   3      13.04 - 17.42       267
REMARK   3      17.42 - 21.80       106
REMARK   3      21.80 - 26.18        86
REMARK   3      26.18 - 30.56        64
REMARK   3      30.56 - 34.94        36
REMARK   3      34.94 - 39.32        14
REMARK   3      39.32 - 43.70         5
REMARK   3      43.70 - 48.07         7
REMARK   3
REMARK   3
LINK         NE2 HIS A  15                RE1  RI3 A1140
LINK         OD2 ASP A 101                RE1  RII A1139
LINK         OD2 ASP A 119                RE1  RII A1141
SSBOND   1 CYS A    6    CYS A  127
SSBOND   2 CYS A   30    CYS A  115
SSBOND   3 CYS A   64    CYS A   80
SSBOND   4 CYS A   76    CYS A   94
CRYST1   80.922   80.922   37.034  90.00  90.00  90.00 P 43 21 2
SCALE1      0.012358  0.000000  0.000000        0.00000
SCALE2      0.000000  0.012358  0.000000        0.00000
SCALE3      0.000000  0.000000  0.027002        0.00000
ATOM      1  N   LYS A   1      11.704  -4.837 -17.980  1.00 14.39           N  0.040
ANISOU    1  N   LYS A   1     1625   1548   2296   -562   -780    848       N
ATOM      2  CA  LYS A   1      12.069  -3.873 -19.044  1.00 14.62           C  0.040
ANISOU    2  CA  LYS A   1     1672   1409   2475   -350   -825    876       C
ATOM      3  C   LYS A   1      13.564  -3.878 -19.247  1.00 13.87           C  0.039
ANISOU    3  C   LYS A   1     1713   1414   2143   -489   -825    827       C
ATOM      4  O   LYS A   1      14.307  -3.846 -18.268  1.00 14.21           O  0.040
ANISOU    4  O   LYS A   1     1805   1500   2097   -573   -818    904       O
ATOM      5  CB  LYS A   1      11.566  -2.494 -18.634  1.00 16.70           C  0.043
ANISOU    5  CB  LYS A   1     1816   1499   3032   -330   -919    857       C
ATOM      6  CG  LYS A   1      12.013  -1.413 -19.581  1.00 17.38           C  0.044
ANISOU    6  CG  LYS A   1     1938   1771   2896   -211   -955   1046       C
ATOM      7  CD  LYS A   1      11.328  -0.109 -19.257  1.00 17.93           C  0.044
ANISOU    7  CD  LYS A   1     2079   1936   2798   -251   -640   1277       C
ATOM      8  CE  LYS A   1      11.849   0.979 -20.212  1.00 20.70           C  0.048
ANISOU    8  CE  LYS A   1     2324   1912   3630   -357   -615   1371       C
ATOM      9  NZ  LYS A   1      11.223   2.301 -19.885  1.00 24.74           N  0.052
ANISOU    9  NZ  LYS A   1     2675   2196   4529   -355   -444   1056       N
```

```
ATOM     10  H1  LYS A   1      10.861  -5.099 -18.093  1.00 17.27           H  0.044
ATOM     11  H2  LYS A   1      12.243  -5.544 -18.023  1.00 17.27           H  0.044
ATOM     12  H3  LYS A   1      11.789  -4.447 -17.184  1.00 17.27           H  0.044
ATOM     13  HA  LYS A   1      11.662  -4.104 -19.893  1.00 17.55           H  0.044
ATOM     14  HB2 LYS A   1      10.596  -2.501 -18.620  1.00 20.05           H  0.047
ATOM     15  HB3 LYS A   1      11.909  -2.281 -17.751  1.00 20.05           H  0.047
ATOM     16  HG2 LYS A   1      12.971  -1.287 -19.501  1.00 20.86           H  0.048
ATOM     17  HG3 LYS A   1      11.786  -1.664 -20.490  1.00 20.86           H  0.048
ATOM     18  HD2 LYS A   1      10.369  -0.200 -19.376  1.00 21.52           H  0.049
ATOM     19  HD3 LYS A   1      11.526   0.152 -18.344  1.00 21.52           H  0.049
ATOM     20  HE2 LYS A   1      12.811   1.060 -20.120  1.00 24.84           H  0.052
ATOM     21  HE3 LYS A   1      11.621   0.746 -21.126  1.00 24.84           H  0.052
ATOM     22  HZ1 LYS A   1      11.481   2.914 -20.476  1.00 29.69           H  0.057
ATOM     23  HZ2 LYS A   1      10.336   2.232 -19.908  1.00 29.69           H  0.057
ATOM     24  HZ3 LYS A   1      11.471   2.562 -19.071  1.00 29.69           H  0.057
ATOM     25  N   VAL A   2      14.010  -3.929 -20.488  1.00 14.02           N  0.039
ANISOU   25  N   VAL A   2     1716   1405   2208   -534   -671    483       N
ATOM     26  CA  VAL A   2      15.422  -3.793 -20.809  1.00 13.06           C  0.038
ANISOU   26  CA  VAL A   2     1897   1266   1799   -647   -501    518       C
ATOM     27  C   VAL A   2      15.623  -2.375 -21.332  1.00 14.43           C  0.040
ANISOU   27  C   VAL A   2     2007   1439   2038   -609   -767    774       C
ATOM     28  O   VAL A   2      15.235  -2.052 -22.466  1.00 15.51           O  0.041
ANISOU   28  O   VAL A   2     1886   1527   2480   -624   -994    861       O
ATOM     29  CB  VAL A   2      15.921  -4.836 -21.813  1.00 13.98           C  0.039
ANISOU   29  CB  VAL A   2     2047   1555   1710   -564   -398    264       C
ATOM     30  CG1 VAL A   2      17.370  -4.559 -22.071  1.00 16.04           C  0.042
ANISOU   30  CG1 VAL A   2     2309   1621   2166   -583   -159    320       C
ATOM     31  CG2 VAL A   2      15.720  -6.267 -21.265  1.00 14.73           C  0.040
ANISOU   31  CG2 VAL A   2     2062   1717   1818   -571   -145    150       C
ATOM     32  H   VAL A   2      13.507  -4.043 -21.176  1.00 16.83           H  0.043
ATOM     33  HA  VAL A   2      15.937  -3.916 -19.997  1.00 15.67           H  0.042
ATOM     34  HB  VAL A   2      15.420  -4.782 -22.642  1.00 16.78           H  0.043
ATOM     35 HG11 VAL A   2      17.773  -5.339 -22.484  1.00 19.25           H  0.046
ATOM     36 HG12 VAL A   2      17.445  -3.795 -22.665  1.00 19.25           H  0.046
ATOM     37 HG13 VAL A   2      17.810  -4.366 -21.228  1.00 19.25           H  0.046
ATOM     38 HG21 VAL A   2      15.970  -6.906 -21.950  1.00 17.68           H  0.044
ATOM     39 HG22 VAL A   2      16.279  -6.386 -20.481  1.00 17.68           H  0.044
ATOM     40 HG23 VAL A   2      14.787  -6.386 -21.026  1.00 17.68           H  0.044
ATOM     41  N   PHE A   3      16.238  -1.542 -20.494  1.00 13.38           N  0.038
ANISOU   41  N   PHE A   3     1911   1315   1856   -672   -463    434       N
ATOM     42  CA  PHE A   3      16.472  -0.156 -20.868  1.00 12.38           C  0.037
ANISOU   42  CA  PHE A   3     1777   1337   1592   -480   -622    445       C
ATOM     43  C   PHE A   3      17.567  -0.047 -21.904  1.00 12.80           C  0.038
ANISOU   43  C   PHE A   3     1922   1436   1503   -345   -715    692       C
ATOM     44  O   PHE A   3      18.513  -0.828 -21.929  1.00 14.19           O  0.039
ANISOU   44  O   PHE A   3     2193   1433   1766   -375   -439    738       O
ATOM     45  CB  PHE A   3      16.957   0.647 -19.663  1.00 10.32           C  0.034
ANISOU   45  CB  PHE A   3     1517   1336   1067   -392   -163    250       C
ATOM     46  CG  PHE A   3      15.886   1.216 -18.829  1.00 11.68           C  0.036
ANISOU   46  CG  PHE A   3     1476   1322   1641   -315   -283    538       C
ATOM     47  CD1 PHE A   3      15.268   0.437 -17.838  1.00 12.16           C  0.037
ANISOU   47  CD1 PHE A   3     1450   1431   1738   -217   -444    644       C
ATOM     48  CD2 PHE A   3      15.493   2.539 -18.984  1.00 13.05           C  0.038
ANISOU   48  CD2 PHE A   3     1527   1562   1867   -121   -436    858       C
ATOM     49  CE1 PHE A   3      14.308   0.985 -17.017  1.00 13.78           C  0.039
ANISOU   49  CE1 PHE A   3     1724   1576   1937   -248   -278    829       C
ATOM     50  CE2 PHE A   3      14.518   3.088 -18.191  1.00 16.01           C  0.042
ANISOU   50  CE2 PHE A   3     1568   1709   2803     -9   -444    943       C
ATOM     51  CZ  PHE A   3      13.906   2.309 -17.182  1.00 15.45           C  0.041
ANISOU   51  CZ  PHE A   3     1624   1678   2568   -134   -362   1015       C
ATOM     52  H   PHE A   3      16.525  -1.755 -19.711  1.00 16.05           H  0.042
ATOM     53  HA  PHE A   3      15.638   0.201 -21.212  1.00 14.86           H  0.040
ATOM     54  HB2 PHE A   3      17.488   0.064 -19.099  1.00 12.38           H  0.037
ATOM     55  HB3 PHE A   3      17.500   1.384 -19.983  1.00 12.38           H  0.037
ATOM     56  HD1 PHE A   3      15.509  -0.456 -17.735  1.00 14.59           H  0.040
ATOM     57  HD2 PHE A   3      15.899   3.062 -19.638  1.00 15.65           H  0.041
ATOM     58  HE1 PHE A   3      13.925   0.467 -16.346  1.00 16.54           H  0.043
ATOM     59  HE2 PHE A   3      14.260   3.972 -18.317  1.00 19.21           H  0.046
ATOM     60  HZ  PHE A   3      13.246   2.674 -16.638  1.00 18.54           H  0.045
ATOM     61  N   GLY A   4      17.456   0.988 -22.753  1.00 13.58           N  0.039
ANISOU   61  N   GLY A   4     1977   1531   1653   -414   -578    843       N
ATOM     62  CA  GLY A   4      18.601   1.437 -23.499  1.00 14.63           C  0.040
ANISOU   62  CA  GLY A   4     2200   1648   1709   -445   -507    683       C
ATOM     63  C   GLY A   4      19.504   2.285 -22.629  1.00 12.66           C  0.037
ANISOU   63  C   GLY A   4     2001   1555   1255   -462   -478    701       C
ATOM     64  O   GLY A   4      19.047   2.901 -21.661  1.00 13.55           O  0.039
ANISOU   64  O   GLY A   4     2096   1638   1414   -655   -382    761       O
ATOM     65  H   GLY A   4      16.733   1.430 -22.900  1.00 16.30           H  0.042
```

```
ATOM     66  HA2 GLY A   4      19.103   0.672 -23.820  1.00 17.55           H  0.044
ATOM     67  HA3 GLY A   4      18.311   1.966 -24.259  1.00 17.55           H  0.044
ATOM     68  N   ARG A   5      20.774   2.337 -23.019  1.00 14.29           N  0.040
ANISOU   68  N   ARG A   5     2335   1607   1489   -307   -143    838       N
ATOM     69  CA  ARG A   5      21.750   3.111 -22.262  1.00 14.56           C  0.040
ANISOU   69  CA  ARG A   5     2272   1488   1772   -226   -147    831       C
ATOM     70  C   ARG A   5      21.274   4.551 -22.020  1.00 14.28           C  0.040
ANISOU   70  C   ARG A   5     2022   1545   1859   -123   -252    680       C
ATOM     71  O   ARG A   5      21.244   5.034 -20.882  1.00 11.31           O  0.035
ANISOU   71  O   ARG A   5     1616   1191   1492   -287   -480    708       O
ATOM     72  CB  ARG A   5      23.097   3.057 -23.001  1.00 15.59           C  0.041
ANISOU   72  CB  ARG A   5     2439   1649   1836   -173    -30    831       C
ATOM     73  CG  ARG A   5      24.125   4.006 -22.493  1.00 17.95           C  0.044
ANISOU   73  CG  ARG A   5     2671   2019   2130     -2   -105    603       C
ATOM     74  CD  ARG A   5      25.436   3.817 -23.246  1.00 19.24           C  0.046
ANISOU   74  CD  ARG A   5     2914   2230   2166    264     40    588       C
ATOM     75  NE  ARG A   5      25.362   4.260 -24.635  1.00 19.79           N  0.047
ANISOU   75  NE  ARG A   5     3190   2391   1938    420   -374    446       N
ATOM     76  CZ  ARG A   5      25.472   5.527 -25.017  1.00 21.44           C  0.049
ANISOU   76  CZ  ARG A   5     3563   2556   2027    393   -447    243       C
ATOM     77  NH1 ARG A   5      25.572   6.512 -24.136  1.00 21.03           N  0.048
ANISOU   77  NH1 ARG A   5     3526   2428   2038    275   -736    145       N
ATOM     78  NH2 ARG A   5      25.509   5.813 -26.317  1.00 20.62           N  0.048
ANISOU   78  NH2 ARG A   5     3878   2650   1305    319   -411    -65       N
ATOM     79  H   ARG A   5      21.092   1.938 -23.711  1.00 17.15           H  0.043
ATOM     80  HA  ARG A   5      21.872   2.717 -21.384  1.00 17.47           H  0.044
ATOM     81  HB2 ARG A   5      23.459   2.161 -22.917  1.00 18.71           H  0.045
ATOM     82  HB3 ARG A   5      22.944   3.266 -23.936  1.00 18.71           H  0.045
ATOM     83  HG2 ARG A   5      23.821   4.918 -22.624  1.00 21.54           H  0.049
ATOM     84  HG3 ARG A   5      24.283   3.842 -21.550  1.00 21.54           H  0.049
ATOM     85  HD2 ARG A   5      26.131   4.330 -22.805  1.00 23.09           H  0.050
ATOM     86  HD3 ARG A   5      25.668   2.875 -23.245  1.00 23.09           H  0.050
ATOM     87  HE  ARG A   5      25.239   3.665 -25.243  1.00 23.75           H  0.051
ATOM     88 HH11 ARG A   5      25.566   6.338 -23.294  1.00 25.24           H  0.053
ATOM     89 HH12 ARG A   5      25.643   7.326 -24.407  1.00 25.24           H  0.053
ATOM     90 HH21 ARG A   5      25.461   5.181 -26.898  1.00 24.74           H  0.052
ATOM     91 HH22 ARG A   5      25.580   6.631 -26.576  1.00 24.74           H  0.052
ATOM     92  N   CYS A   6      20.950   5.288 -23.087  1.00 14.43           N  0.040
ANISOU   92  N   CYS A   6     2244   1640   1599      8     73    548       N
ATOM     93  CA  CYS A   6      20.626   6.699 -22.873  1.00 14.63           C  0.040
ANISOU   93  CA  CYS A   6     2256   1615   1687    -70   -291    483       C
ATOM     94  C   CYS A   6      19.291   6.870 -22.161  1.00 14.05           C  0.039
ANISOU   94  C   CYS A   6     2207   1506   1625    -43   -425    848       C
ATOM     95  O   CYS A   6      19.108   7.817 -21.385  1.00 14.74           O  0.040
ANISOU   95  O   CYS A   6     2251   1466   1884    140   -369    901       O
ATOM     96  CB  CYS A   6      20.611   7.456 -24.201  1.00 14.78           C  0.040
ANISOU   96  CB  CYS A   6     2204   1613   1799   -133   -386    819       C
ATOM     97  SG  CYS A   6      22.260   7.768 -24.883  1.00 14.91           S  0.040
ANISOU   97  SG  CYS A   6     2092   1604   1968    -59   -201    902       S
ATOM     98  H   CYS A   6      20.913   5.010 -23.900  1.00 17.32           H  0.044
ATOM     99  HA  CYS A   6      21.320   7.087 -22.317  1.00 17.55           H  0.044
ATOM    100  HB2 CYS A   6      20.115   6.934 -24.851  1.00 17.74           H  0.044
ATOM    101  HB3 CYS A   6      20.179   8.314 -24.065  1.00 17.74           H  0.044
ATOM    102  N   GLU A   7      18.339   5.983 -22.439  1.00 14.84           N  0.040
ANISOU  102  N   GLU A   7     2237   1434   1967    -17   -641    816       N
ATOM    103  CA  GLU A   7      17.048   6.025 -21.775  1.00 14.68           C  0.040
ANISOU  103  CA  GLU A   7     2032   1450   2097    -90   -536    868       C
ATOM    104  C   GLU A   7      17.214   5.864 -20.270  1.00 13.19           C  0.038
ANISOU  104  C   GLU A   7     1763   1368   1881     34   -489    824       C
ATOM    105  O   GLU A   7      16.605   6.582 -19.465  1.00 14.28           O  0.040
ANISOU  105  O   GLU A   7     1615   1394   2418   -133   -698    876       O
ATOM    106  CB  GLU A   7      16.174   4.921 -22.376  1.00 17.21           C  0.043
ANISOU  106  CB  GLU A   7     2256   1859   2422   -129   -490   1192       C
ATOM    107  CG  GLU A   7      14.846   4.690 -21.675  1.00 21.15           C  0.048
ANISOU  107  CG  GLU A   7     2385   2320   3333   -213   -743   1460       C
ATOM    108  CD  GLU A   7      14.058   3.457 -22.235  1.00 24.46           C  0.052
ANISOU  108  CD  GLU A   7     2598   2683   4013   -251   -857   1749       C
ATOM    109  OE1 GLU A   7      14.665   2.442 -22.703  1.00 24.85           O  0.052
ANISOU  109  OE1 GLU A   7     2793   2899   3751   -179   -699   1861       O
ATOM    110  OE2 GLU A   7      12.811   3.506 -22.194  1.00 26.80           O  0.054
ANISOU  110  OE2 GLU A   7     2598   2938   4646   -457   -671   1769       O
ATOM    111  H   GLU A   7      18.418   5.345 -23.011  1.00 17.81           H  0.044
ATOM    112  HA  GLU A   7      16.610   6.878 -21.921  1.00 17.62           H  0.044
ATOM    113  HB2 GLU A   7      15.979   5.153 -23.297  1.00 20.65           H  0.048
ATOM    114  HB3 GLU A   7      16.669   4.087 -22.342  1.00 20.65           H  0.048
ATOM    115  HG2 GLU A   7      15.011   4.534 -20.732  1.00 25.38           H  0.053
ATOM    116  HG3 GLU A   7      14.289   5.476 -21.789  1.00 25.38           H  0.053
ATOM    117  N   LEU A   8      18.045   4.901 -19.868  1.00 11.68           N  0.036
```

```
ANISOU  117  N   LEU A   8     1630   1435   1372    -89   -494    718       N
ATOM    118  CA  LEU A   8      18.268   4.727 -18.442  1.00 11.19           C  0.035
ANISOU  118  CA  LEU A   8     1444   1356   1451    163   -443    663       C
ATOM    119  C   LEU A   8      19.016   5.915 -17.870  1.00 12.01           C  0.036
ANISOU  119  C   LEU A   8     1226   1319   2019     26   -378    799       C
ATOM    120  O   LEU A   8      18.730   6.353 -16.765  1.00 11.90           O  0.036
ANISOU  120  O   LEU A   8     1060   1418   2045   -135   -283    863       O
ATOM    121  CB  LEU A   8      19.041   3.428 -18.172  1.00  9.69           C  0.033
ANISOU  121  CB  LEU A   8     1551   1343    787    210   -180    319       C
ATOM    122  CG  LEU A   8      19.310   3.100 -16.697  1.00 10.95           C  0.035
ANISOU  122  CG  LEU A   8     1545   1430   1184    311   -264    343       C
ATOM    123  CD1 LEU A   8      18.024   2.913 -15.913  1.00 12.32           C  0.037
ANISOU  123  CD1 LEU A   8     1539   1450   1693    154    -58    858       C
ATOM    124  CD2 LEU A   8      20.202   1.863 -16.556  1.00 13.42           C  0.038
ANISOU  124  CD2 LEU A   8     1862   1543   1695    294   -389    255       C
ATOM    125  H   LEU A   8      18.473   4.360 -20.382  1.00 14.01           H  0.039
ATOM    126  HA  LEU A   8      17.408   4.656 -17.999  1.00 13.43           H  0.038
ATOM    127  HB2 LEU A   8      18.531   2.690 -18.541  1.00 11.62           H  0.036
ATOM    128  HB3 LEU A   8      19.902   3.491 -18.614  1.00 11.62           H  0.036
ATOM    129  HG  LEU A   8      19.778   3.858 -16.314  1.00 13.14           H  0.038
ATOM    130 HD11 LEU A   8      18.227   2.467 -15.076  1.00 14.79           H  0.040
ATOM    131 HD12 LEU A   8      17.631   3.782 -15.739  1.00 14.79           H  0.040
ATOM    132 HD13 LEU A   8      17.412   2.372 -16.436  1.00 14.79           H  0.040
ATOM    133 HD21 LEU A   8      20.263   1.623 -15.618  1.00 16.11           H  0.042
ATOM    134 HD22 LEU A   8      19.810   1.133 -17.060  1.00 16.11           H  0.042
ATOM    135 HD23 LEU A   8      21.084   2.069 -16.903  1.00 16.11           H  0.042
ATOM    136  N   ALA A   9      20.006   6.442 -18.595  1.00 10.98           N  0.035
ANISOU  136  N   ALA A   9     1206   1122   1845    -58   -552    658       N
ATOM    137  CA  ALA A   9      20.676   7.648 -18.110  1.00 11.16           C  0.035
ANISOU  137  CA  ALA A   9     1297   1112   1832    -12   -355    351       C
ATOM    138  C   ALA A   9      19.678   8.770 -17.832  1.00 11.56           C  0.036
ANISOU  138  C   ALA A   9     1314   1292   1786     31      4    395       C
ATOM    139  O   ALA A   9      19.725   9.410 -16.786  1.00 10.57           O  0.034
ANISOU  139  O   ALA A   9     1278   1297   1441    -58    -67    746       O
ATOM    140  CB  ALA A   9      21.729   8.097 -19.109  1.00 11.05           C  0.035
ANISOU  140  CB  ALA A   9     1210    978   2012   -109   -407    497       C
ATOM    141  H   ALA A   9      20.297   6.133 -19.343  1.00 13.18           H  0.038
ATOM    142  HA  ALA A   9      21.126   7.441 -17.276  1.00 13.40           H  0.038
ATOM    143  HB1 ALA A   9      22.168   8.892 -18.769  1.00 13.26           H  0.038
ATOM    144  HB2 ALA A   9      22.377   7.385 -19.225  1.00 13.26           H  0.038
ATOM    145  HB3 ALA A   9      21.297   8.293 -19.955  1.00 13.26           H  0.038
ATOM    146  N   ALA A  10      18.723   8.972 -18.732  1.00 12.24           N  0.037
ANISOU  146  N   ALA A  10     1489   1493   1669     64    106    709       N
ATOM    147  CA  ALA A  10      17.722  10.005 -18.512  1.00 12.94           C  0.038
ANISOU  147  CA  ALA A  10     1494   1348   2075     32     40    935       C
ATOM    148  C   ALA A  10      16.876   9.726 -17.280  1.00 14.34           C  0.040
ANISOU  148  C   ALA A  10     1595   1303   2551    149   -210    904       C
ATOM    149  O   ALA A  10      16.635  10.630 -16.463  1.00 14.74           O  0.040
ANISOU  149  O   ALA A  10     1650   1318   2634    219    -23    938       O
ATOM    150  CB  ALA A  10      16.859  10.112 -19.763  1.00 15.09           C  0.041
ANISOU  150  CB  ALA A  10     1712   1493   2529     -1     33   1089       C
ATOM    151  H   ALA A  10      18.634   8.532 -19.466  1.00 14.69           H  0.040
ATOM    152  HA  ALA A  10      18.163  10.855 -18.355  1.00 15.53           H  0.041
ATOM    153  HB1 ALA A  10      16.215  10.827 -19.643  1.00 18.11           H  0.045
ATOM    154  HB2 ALA A  10      17.428  10.304 -20.525  1.00 18.11           H  0.045
ATOM    155  HB3 ALA A  10      16.396   9.270 -19.900  1.00 18.11           H  0.045
ATOM    156  N   ALA A  11      16.396   8.478 -17.144  1.00 14.49           N  0.040
ANISOU  156  N   ALA A  11     1615   1484   2406    245   -445    858       N
ATOM    157  CA  ALA A  11      15.531   8.144 -16.021  1.00 12.95           C  0.038
ANISOU  157  CA  ALA A  11     1493   1224   2204    235   -119    827       C
ATOM    158  C   ALA A  11      16.276   8.294 -14.711  1.00 13.45           C  0.038
ANISOU  158  C   ALA A  11     1523   1023   2564    283    224    567       C
ATOM    159  O   ALA A  11      15.741   8.809 -13.731  1.00 14.31           O  0.040
ANISOU  159  O   ALA A  11     1433   1010   2996    285    405    411       O
ATOM    160  CB  ALA A  11      14.998   6.714 -16.188  1.00 13.44           C  0.038
ANISOU  160  CB  ALA A  11     1551   1363   2193    285    -19    907       C
ATOM    161  H   ALA A  11      16.557   7.826 -17.681  1.00 17.39           H  0.044
ATOM    162  HA  ALA A  11      14.770   8.745 -16.005  1.00 15.55           H  0.041
ATOM    163  HB1 ALA A  11      14.430   6.498 -15.432  1.00 16.13           H  0.042
ATOM    164  HB2 ALA A  11      14.487   6.662 -17.011  1.00 16.13           H  0.042
ATOM    165  HB3 ALA A  11      15.748   6.100 -16.225  1.00 16.13           H  0.042
ATOM    166  N   MET A  12      17.518   7.840 -14.688  1.00 11.88           N  0.036
ANISOU  166  N   MET A  12     1575    901   2038    272    186    432       N
ATOM    167  CA  MET A  12      18.354   7.928 -13.504  1.00 11.51           C  0.036
ANISOU  167  CA  MET A  12     1403   1047   1922    239    302    254       C
ATOM    168  C   MET A  12      18.601   9.392 -13.135  1.00 11.51           C  0.036
ANISOU  168  C   MET A  12     1360   1127   1885    135    310    427       C
ATOM    169  O   MET A  12      18.541   9.765 -11.944  1.00 12.27           O  0.037
```

```
ANISOU  169  O   MET A  12     1348   1204   2112     94    162    353       O
ATOM    170  CB  MET A  12      19.625   7.193 -13.918  1.00 11.26           C  0.035
ANISOU  170  CB  MET A  12     1344   1043   1890    359    -12     68       C
ATOM    171  CG  MET A  12      19.561   5.722 -13.545  1.00  9.00           C  0.031
ANISOU  171  CG  MET A  12     1174   1053   1192    280   -111    -60       C
ATOM    172  SD  MET A  12      21.174   4.937 -13.805  1.00  9.22           S  0.032
ANISOU  172  SD  MET A  12      881   1165   1456     97    -95    274       S
ATOM    173  CE  MET A  12      20.943   3.298 -13.028  1.00 11.00           C  0.035
ANISOU  173  CE  MET A  12      816    946   2418     82    -79    234       C
ATOM    174  H   MET A  12      17.910   7.469 -15.357  1.00 14.26           H  0.040
ATOM    175  HA  MET A  12      17.970   7.521 -12.712  1.00 13.81           H  0.039
ATOM    176  HB2 MET A  12      19.737   7.261 -14.879  1.00 13.51           H  0.039
ATOM    177  HB3 MET A  12      20.386   7.590 -13.466  1.00 13.51           H  0.039
ATOM    178  HG2 MET A  12      19.320   5.634 -12.610  1.00 10.80           H  0.034
ATOM    179  HG3 MET A  12      18.905   5.274 -14.101  1.00 10.80           H  0.034
ATOM    180  HE1 MET A  12      21.770   2.796 -13.099  1.00 13.20           H  0.038
ATOM    181  HE2 MET A  12      20.709   3.421 -12.095  1.00 13.20           H  0.038
ATOM    182  HE3 MET A  12      20.231   2.828 -13.489  1.00 13.20           H  0.038
ATOM    183  N   LYS A  13      18.790  10.248 -14.130  1.00 11.89           N  0.036
ANISOU  183  N   LYS A  13     1333    980   2206    131     82    324       N
ATOM    184  CA  LYS A  13      18.907  11.674 -13.875  1.00 12.58           C  0.037
ANISOU  184  CA  LYS A  13     1761    943   2075    -25    151    673       C
ATOM    185  C   LYS A  13      17.620  12.271 -13.314  1.00 14.50           C  0.040
ANISOU  185  C   LYS A  13     1828   1120   2561    402     51    595       C
ATOM    186  O   LYS A  13      17.669  13.076 -12.383  1.00 15.94           O  0.042
ANISOU  186  O   LYS A  13     1696   1328   3034    603    287    582       O
ATOM    187  CB  LYS A  13      19.330  12.398 -15.156  1.00 14.35           C  0.040
ANISOU  187  CB  LYS A  13     2377   1148   1928   -252    157    791       C
ATOM    188  CG  LYS A  13      19.884  13.837 -14.855  1.00 18.28           C  0.045
ANISOU  188  CG  LYS A  13     2968   1354   2625   -330    162    951       C
ATOM    189  CD  LYS A  13      20.213  14.604 -16.129  1.00 21.60           C  0.049
ANISOU  189  CD  LYS A  13     3506   1415   3287   -333    415    729       C
ATOM    190  CE  LYS A  13      20.566  16.067 -15.811  1.00 23.04           C  0.050
ANISOU  190  CE  LYS A  13     3886   1612   3255   -461    501    946       C
ATOM    191  NZ  LYS A  13      20.825  16.873 -17.053  1.00 26.58           N  0.054
ANISOU  191  NZ  LYS A  13     4244   2024   3831   -449    401    576       N
ATOM    192  H   LYS A  13      18.855  10.030 -14.960  1.00 14.27           H  0.040
ATOM    193  HA  LYS A  13      19.596  11.806 -13.205  1.00 15.09           H  0.041
ATOM    194  HB2 LYS A  13      20.028  11.888 -15.596  1.00 17.22           H  0.044
ATOM    195  HB3 LYS A  13      18.562  12.484 -15.743  1.00 17.22           H  0.044
ATOM    196  HG2 LYS A  13      19.216  14.339 -14.363  1.00 21.94           H  0.049
ATOM    197  HG3 LYS A  13      20.696  13.762 -14.329  1.00 21.94           H  0.049
ATOM    198  HD2 LYS A  13      20.974  14.192 -16.568  1.00 25.92           H  0.053
ATOM    199  HD3 LYS A  13      19.445  14.595 -16.720  1.00 25.92           H  0.053
ATOM    200  HE2 LYS A  13      19.828  16.477 -15.334  1.00 27.65           H  0.055
ATOM    201  HE3 LYS A  13      21.367  16.090 -15.264  1.00 27.65           H  0.055
ATOM    202  HZ1 LYS A  13      20.933  17.731 -16.842  1.00 31.90           H  0.059
ATOM    203  HZ2 LYS A  13      21.561  16.580 -17.458  1.00 31.90           H  0.059
ATOM    204  HZ3 LYS A  13      20.137  16.798 -17.613  1.00 31.90           H  0.059
ATOM    205  N   ARG A  14      16.482  11.894 -13.863  1.00 15.74           N  0.042
ANISOU  205  N   ARG A  14     1866   1350   2763    554    397    764       N
ATOM    206  CA  ARG A  14      15.166  12.392 -13.379  1.00 17.66           C  0.044
ANISOU  206  CA  ARG A  14     2248   1743   2718    634    228    912       C
ATOM    207  C   ARG A  14      14.961  11.946 -11.932  1.00 16.36           C  0.042
ANISOU  207  C   ARG A  14     2018   1490   2708    442    140    669       C
ATOM    208  O   ARG A  14      14.337  12.668 -11.178  1.00 20.11           O  0.047
ANISOU  208  O   ARG A  14     2193   1460   3989    547    239    478       O
ATOM    209  CB  ARG A  14      14.067  11.877 -14.289  1.00 22.19           C  0.049
ANISOU  209  CB  ARG A  14     2943   2392   3097    992    -44   1211       C
ATOM    210  CG  ARG A  14      14.057  12.523 -15.664  1.00 29.90           C  0.057
ANISOU  210  CG  ARG A  14     3651   3333   4378   1046     96    880       C
ATOM    211  CD  ARG A  14      12.647  12.860 -16.081  1.00 36.98           C  0.064
ANISOU  211  CD  ARG A  14     4312   4179   5559   1143     -7    773       C
ATOM    212  NE  ARG A  14      12.088  13.890 -15.216  1.00 42.18           N  0.068
ANISOU  212  NE  ARG A  14     4885   4807   6334   1103     -5    652       N
ATOM    213  CZ  ARG A  14      10.794  14.150 -15.079  1.00 45.06           C  0.070
ANISOU  213  CZ  ARG A  14     5219   5258   6643   1139    -79    663       C
ATOM    214  NH1 ARG A  14       9.891  13.466 -15.758  1.00 46.76           N  0.072
ANISOU  214  NH1 ARG A  14     5362   5462   6944   1166    -81    701       N
ATOM    215  NH2 ARG A  14      10.400  15.109 -14.265  1.00 45.58           N  0.071
ANISOU  215  NH2 ARG A  14     5423   5429   6468   1056   -120    683       N
ATOM    216  H   ARG A  14      16.423  11.220 -14.648  1.00 18.88           H  0.046
ATOM    217  HA  ARG A  14      15.173  13.480 -13.415  1.00 21.19           H  0.048
ATOM    218  HB2 ARG A  14      14.192  10.801 -14.407  1.00 26.63           H  0.054
ATOM    219  HB3 ARG A  14      13.106  12.052 -13.807  1.00 26.63           H  0.054
ATOM    220  HG2 ARG A  14      14.656  13.431 -15.645  1.00 35.89           H  0.063
ATOM    221  HG3 ARG A  14      14.492  11.839 -16.391  1.00 35.89           H  0.063
ATOM    222  HD2 ARG A  14      12.642  13.208 -17.113  1.00 44.37           H  0.070
```

```
ATOM    223  HD3 ARG A  14      12.035  11.962 -16.020  1.00 44.37           H  0.070
ATOM    224  HE  ARG A  14      12.738  14.446 -14.668  1.00 50.61           H  0.075
ATOM    225 HH11 ARG A  14      10.167  12.725 -16.395  1.00 56.12           H  0.079
ATOM    226 HH12 ARG A  14       8.907  13.686 -15.639  1.00 56.12           H  0.079
ATOM    227 HH21 ARG A  14      11.088  15.640 -13.739  1.00 54.70           H  0.078
ATOM    228 HH22 ARG A  14       9.409  15.309 -14.162  1.00 54.70           H  0.078
ATOM    229  N   HIS A  15      15.494  10.784 -11.563  1.00 13.57           N  0.039
ANISOU  229  N   HIS A  15     1641   1556   1959    346    -69    391       N
ATOM    230  CA  HIS A  15      15.376  10.261 -10.192  1.00 14.14           C  0.039
ANISOU  230  CA  HIS A  15     1580   1291   2500    309    336    556       C
ATOM    231  C   HIS A  15      16.500  10.744  -9.296  1.00 14.52           C  0.040
ANISOU  231  C   HIS A  15     1782   1128   2607    494    381    636       C
ATOM    232  O   HIS A  15      16.602  10.167  -8.276  1.00 14.40           O  0.040
ANISOU  232  O   HIS A  15     1912   1089   2471    402    559    521       O
ATOM    233  CB  HIS A  15      15.289   8.735 -10.285  1.00 15.23           C  0.041
ANISOU  233  CB  HIS A  15     1473   1514   2802    324    397    655       C
ATOM    234  CG  HIS A  15      13.958   8.255 -10.738  1.00 19.73           C  0.047
ANISOU  234  CG  HIS A  15     1550   2104   3842    248    371     93       C
ATOM    235  ND1 HIS A  15      12.872   8.265  -9.915  1.00 22.81           N  0.050
ANISOU  235  ND1 HIS A  15     1799   2643   4223     35    327   -151       N
ATOM    236  CD2 HIS A  15      13.527   7.758 -11.911  1.00 22.17           C  0.049
ANISOU  236  CD2 HIS A  15     1452   2488   4484     34     47    119       C
ATOM    237  CE1 HIS A  15      11.832   7.782 -10.547  1.00 22.38           C  0.050
ANISOU  237  CE1 HIS A  15     1460   2570   4474    -17    379     70       C
ATOM    238  NE2 HIS A  15      12.202   7.475 -11.766  1.00 23.06           N  0.050
ANISOU  238  NE2 HIS A  15     1481   2563   4720    172    -20    510       N
ATOM    239  H   HIS A  15      16.031  10.163 -12.196  1.00 16.28           H  0.042
ATOM    240  HA  HIS A  15      14.438  10.622  -9.775  1.00 16.96           H  0.043
ATOM    241  HB2 HIS A  15      16.052   8.375 -10.974  1.00 18.28           H  0.045
ATOM    242  HB3 HIS A  15      15.492   8.308  -9.306  1.00 18.28           H  0.045
ATOM    243  HD1 HIS A  15      12.853   8.647  -8.980  1.00 27.37           H  0.055
ATOM    244  HD2 HIS A  15      14.119   7.556 -12.790  1.00 26.61           H  0.054
ATOM    245  HE1 HIS A  15      10.830   7.710 -10.148  1.00 26.86           H  0.054
ATOM    246  N   GLY A  16      17.318  11.741  -9.685  1.00 14.47           N  0.040
ANISOU  246  N   GLY A  16     1952   1036   2511    381    484    475       N
ATOM    247  CA  GLY A  16      18.258  12.316  -8.752  1.00 14.75           C  0.040
ANISOU  247  CA  GLY A  16     2059   1040   2504    234    314    263       C
ATOM    248  C   GLY A  16      19.535  11.561  -8.480  1.00 13.56           C  0.039
ANISOU  248  C   GLY A  16     2038   1054   2062    178     28    170       C
ATOM    249  O   GLY A  16      20.223  11.871  -7.499  1.00 14.67           O  0.040
ANISOU  249  O   GLY A  16     2403   1151   2021    404     36    -86       O
ATOM    250  H   GLY A  16      17.333  12.079 -10.475  1.00 17.37           H  0.044
ATOM    251  HA2 GLY A  16      18.514  13.190  -9.087  1.00 17.70           H  0.044
ATOM    252  HA3 GLY A  16      17.807  12.424  -7.901  1.00 17.70           H  0.044
ATOM    253  N   LEU A  17      19.918  10.620  -9.346  1.00 11.91           N  0.036
ANISOU  253  N   LEU A  17     1670   1036   1820    127    -37      9       N
ATOM    254  CA  LEU A  17      21.186   9.922  -9.165  1.00 11.85           C  0.036
ANISOU  254  CA  LEU A  17     1783    845   1873     -2    257     95       C
ATOM    255  C   LEU A  17      22.401  10.679  -9.690  1.00 13.65           C  0.039
ANISOU  255  C   LEU A  17     1970    912   2304    -37     51      4       C
ATOM    256  O   LEU A  17      23.518  10.361  -9.251  1.00 12.84           O  0.038
ANISOU  256  O   LEU A  17     1636    899   2345     23    -13   -101       O
ATOM    257  CB  LEU A  17      21.137   8.542  -9.820  1.00 11.74           C  0.036
ANISOU  257  CB  LEU A  17     1726    901   1835   -170    167     37       C
ATOM    258  CG  LEU A  17      20.441   7.467  -8.977  1.00 12.76           C  0.037
ANISOU  258  CG  LEU A  17     1824   1162   1862   -192    222     92       C
ATOM    259  CD1 LEU A  17      20.556   6.191  -9.771  1.00 12.05           C  0.036
ANISOU  259  CD1 LEU A  17     1855    770   1952   -459    -52   -174       C
ATOM    260  CD2 LEU A  17      21.046   7.250  -7.604  1.00 13.02           C  0.038
ANISOU  260  CD2 LEU A  17     1805   1338   1803     -5    122    -57       C
ATOM    261  H   LEU A  17      19.467  10.373 -10.035  1.00 14.30           H  0.040
ATOM    262  HA  LEU A  17      21.312   9.808  -8.210  1.00 14.22           H  0.040
ATOM    263  HB2 LEU A  17      20.657   8.615 -10.660  1.00 14.09           H  0.039
ATOM    264  HB3 LEU A  17      22.046   8.244  -9.983  1.00 14.09           H  0.039
ATOM    265  HG  LEU A  17      19.527   7.742  -8.805  1.00 15.31           H  0.041
ATOM    266 HD11 LEU A  17      19.905   5.553  -9.441  1.00 14.46           H  0.040
ATOM    267 HD12 LEU A  17      20.385   6.385 -10.706  1.00 14.46           H  0.040
ATOM    268 HD13 LEU A  17      21.452   5.834  -9.666  1.00 14.46           H  0.040
ATOM    269 HD21 LEU A  17      20.762   6.387  -7.267  1.00 15.62           H  0.041
ATOM    270 HD22 LEU A  17      22.013   7.275  -7.678  1.00 15.62           H  0.041
ATOM    271 HD23 LEU A  17      20.741   7.954  -7.010  1.00 15.62           H  0.041
ATOM    272  N   ASP A  18      22.245  11.647 -10.616  1.00 13.32           N  0.038
ANISOU  272  N   ASP A  18     2067    918   2075     74    115     93       N
ATOM    273  CA  ASP A  18      23.438  12.345 -11.099  1.00 13.78           C  0.039
ANISOU  273  CA  ASP A  18     2186   1036   2015    371    236    140       C
ATOM    274  C   ASP A  18      24.094  13.097  -9.946  1.00 13.98           C  0.039
ANISOU  274  C   ASP A  18     2144    976   2194    234    227     -9       C
ATOM    275  O   ASP A  18      23.482  14.002  -9.349  1.00 13.50           O  0.039
```

```
ANISOU  275  O   ASP A  18     2069    992   2068    185    307    -76       O
ATOM    276  CB  ASP A  18      23.116  13.315 -12.241  1.00 15.89           C  0.042
ANISOU  276  CB  ASP A  18     2425   1107   2504    632    309    506       C
ATOM    277  CG  ASP A  18      24.363  13.839 -12.964  1.00 18.52           C  0.045
ANISOU  277  CG  ASP A  18     2813   1410   2813    654   -126    535       C
ATOM    278  OD1 ASP A  18      25.468  13.259 -12.843  1.00 17.76           O  0.044
ANISOU  278  OD1 ASP A  18     2785   1481   2484    824   -514     29       O
ATOM    279  OD2 ASP A  18      24.222  14.860 -13.672  1.00 21.10           O  0.048
ANISOU  279  OD2 ASP A  18     3060   1734   3222    549   -150    874       O
ATOM    280  H   ASP A  18      21.497  11.900 -10.958  1.00 15.98           H  0.042
ATOM    281  HA  ASP A  18      24.051  11.684 -11.455  1.00 16.54           H  0.043
ATOM    282  HB2 ASP A  18      22.563  12.858 -12.894  1.00 19.06           H  0.046
ATOM    283  HB3 ASP A  18      22.639  14.079 -11.879  1.00 19.06           H  0.046
ATOM    284  N   ASN A  19      25.331  12.705  -9.640  1.00 12.24           N  0.037
ANISOU  284  N   ASN A  19     2056    586   2008    183    113    169       N
ATOM    285  CA  ASN A  19      26.142  13.292  -8.545  1.00 13.69           C  0.039
ANISOU  285  CA  ASN A  19     2176    944   2081     62    108    296       C
ATOM    286  C   ASN A  19      25.590  12.955  -7.165  1.00 12.68           C  0.037
ANISOU  286  C   ASN A  19     1886    862   2072    -15   -206     63       C
ATOM    287  O   ASN A  19      26.015  13.573  -6.206  1.00 13.38           O  0.038
ANISOU  287  O   ASN A  19     2069    930   2085    -16    -29    246       O
ATOM    288  CB  ASN A  19      26.344  14.785  -8.776  1.00 17.82           C  0.044
ANISOU  288  CB  ASN A  19     2765   1635   2371    -79    308    675       C
ATOM    289  CG  ASN A  19      27.808  14.954  -9.085  1.00 26.98           C  0.054
ANISOU  289  CG  ASN A  19     3550   2885   3814   -326    357    331       C
ATOM    290  OD1 ASN A  19      28.627  14.987  -8.169  1.00 29.79           O  0.057
ANISOU  290  OD1 ASN A  19     3797   3428   4092   -248    203    154       O
ATOM    291  ND2 ASN A  19      28.146  14.837 -10.358  1.00 27.86           N  0.055
ANISOU  291  ND2 ASN A  19     3863   3259   3463   -465    531    287       N
ATOM    292  H   ASN A  19      25.822  11.949 -10.152  1.00 14.69           H  0.040
ATOM    293  HA  ASN A  19      27.130  12.838  -8.610  1.00 16.42           H  0.042
ATOM    294  HB2 ASN A  19      25.742  15.122  -9.619  1.00 21.38           H  0.048
ATOM    295  HB3 ASN A  19      26.073  15.349  -7.886  1.00 21.38           H  0.048
ATOM    296 HD21 ASN A  19      27.708  14.124 -10.935  1.00 33.43           H  0.061
ATOM    297 HD22 ASN A  19      28.844  15.457 -10.759  1.00 33.43           H  0.061
ATOM    298  N   TYR A  20      24.684  11.979  -7.070  1.00 11.89           N  0.036
ANISOU  298  N   TYR A  20     1610    707   2201   -181     88    259       N
ATOM    299  CA  TYR A  20      24.250  11.525  -5.760  1.00 10.47           C  0.034
ANISOU  299  CA  TYR A  20     1492    895   1592     10      1    -12       C
ATOM    300  C   TYR A  20      25.424  10.920  -4.990  1.00 11.16           C  0.035
ANISOU  300  C   TYR A  20     1544   1078   1617   -231    295   -257       C
ATOM    301  O   TYR A  20      26.116  10.006  -5.478  1.00 11.01           O  0.035
ANISOU  301  O   TYR A  20     1337   1298   1549   -120    315   -466       O
ATOM    302  CB  TYR A  20      23.121  10.494  -5.855  1.00 11.76           C  0.036
ANISOU  302  CB  TYR A  20     1459   1041   1970   -265     -5   -288       C
ATOM    303  CG  TYR A  20      22.480  10.269  -4.501  1.00 12.31           C  0.037
ANISOU  303  CG  TYR A  20     1448   1274   1956   -249    396   -287       C
ATOM    304  CD1 TYR A  20      21.476  11.113  -4.049  1.00 13.56           C  0.039
ANISOU  304  CD1 TYR A  20     1616   1336   2200   -165    323     15       C
ATOM    305  CD2 TYR A  20      22.941   9.274  -3.639  1.00 13.58           C  0.039
ANISOU  305  CD2 TYR A  20     1439   1454   2265   -296    568   -140       C
ATOM    306  CE1 TYR A  20      20.920  10.935  -2.782  1.00 14.47           C  0.040
ANISOU  306  CE1 TYR A  20     1493   1480   2526   -219    492     56       C
ATOM    307  CE2 TYR A  20      22.416   9.105  -2.393  1.00 13.91           C  0.039
ANISOU  307  CE2 TYR A  20     1493   1565   2229   -366    669   -312       C
ATOM    308  CZ  TYR A  20      21.395   9.945  -1.971  1.00 14.85           C  0.040
ANISOU  308  CZ  TYR A  20     1589   1530   2521   -329    639    -42       C
ATOM    309  OH  TYR A  20      20.864   9.782  -0.735  1.00 16.74           O  0.043
ANISOU  309  OH  TYR A  20     1975   1712   2673   -209    437   -124       O
ATOM    310  H   TYR A  20      24.328  11.562  -7.732  1.00 14.27           H  0.040
ATOM    311  HA  TYR A  20      23.906  12.294  -5.280  1.00 12.57           H  0.037
ATOM    312  HB2 TYR A  20      22.441  10.814  -6.468  1.00 14.12           H  0.039
ATOM    313  HB3 TYR A  20      23.480   9.649  -6.170  1.00 14.12           H  0.039
ATOM    314  HD1 TYR A  20      21.172  11.802  -4.594  1.00 16.27           H  0.042
ATOM    315  HD2 TYR A  20      23.625   8.711  -3.922  1.00 16.29           H  0.042
ATOM    316  HE1 TYR A  20      20.230  11.488  -2.491  1.00 17.36           H  0.044
ATOM    317  HE2 TYR A  20      22.737   8.436  -1.832  1.00 16.70           H  0.043
ATOM    318  HH  TYR A  20      20.232  10.322  -0.620  1.00 20.08           H  0.047
ATOM    319  N  AARG A  21      25.676  11.412  -3.779  0.51 10.86           N  0.035
ANISOU  319  N  AARG A  21     1587   1045   1495   -401    437   -439       N
ATOM    320  CA AARG A  21      26.852  10.956  -2.977  0.51 12.20           C  0.037
ANISOU  320  CA AARG A  21     1822   1142   1674   -315    382   -374       C
ATOM    321  C  AARG A  21      28.134  11.154  -3.802  0.51 11.31           C  0.035
ANISOU  321  C  AARG A  21     1729    983   1584   -285     12   -334       C
ATOM    322  O  AARG A  21      29.158  10.551  -3.467  0.51 11.17           O  0.035
ANISOU  322  O  AARG A  21     1693    958   1592    -87   -172   -499       O
ATOM    323  CB AARG A  21      26.652   9.517  -2.472  0.51 15.21           C  0.041
ANISOU  323  CB AARG A  21     2201   1380   2195   -377    563   -254       C
```

```
ATOM    324  CG AARG A  21      25.946   9.403  -1.128  0.51 18.89           C  0.046
ANISOU  324  CG AARG A  21     2741   1732   2706   -420    678   -397       C
ATOM    325  CD AARG A  21      26.566  10.292  -0.068  0.51 21.83           C  0.049
ANISOU  325  CD AARG A  21     3148   2107   3038   -379    929   -268       C
ATOM    326  NE AARG A  21      26.166  10.021   1.303  0.51 25.78           N  0.053
ANISOU  326  NE AARG A  21     3551   2339   3906   -283    889   -175       N
ATOM    327  CZ AARG A  21      24.972  10.292   1.821  0.51 25.53           C  0.053
ANISOU  327  CZ AARG A  21     3688   2384   3627    -68   1158   -421       C
ATOM    328  NH1AARG A  21      23.999  10.799   1.079  0.51 22.87           N  0.050
ANISOU  328  NH1AARG A  21     3597   2158   2935    125   1353   -746       N
ATOM    329  NH2AARG A  21      24.745  10.030   3.093  0.51 27.66           N  0.055
ANISOU  329  NH2AARG A  21     3836   2579   4095    -29    824   -276       N
ATOM    330  H  AARG A  21      24.880  11.611  -3.145  0.51 13.03           H  0.038
ATOM    331  HA AARG A  21      26.925  11.618  -2.118  0.51 14.65           H  0.040
ATOM    332  HB2AARG A  21      26.071   8.969  -3.212  0.51 18.25           H  0.045
ATOM    333  HB3AARG A  21      27.625   9.036  -2.394  0.51 18.25           H  0.045
ATOM    334  HG2AARG A  21      24.899   9.673  -1.254  0.51 22.67           H  0.050
ATOM    335  HG3AARG A  21      25.984   8.369  -0.788  0.51 22.67           H  0.050
ATOM    336  HD2AARG A  21      27.649  10.210  -0.139  0.51 26.19           H  0.054
ATOM    337  HD3AARG A  21      26.304  11.325  -0.288  0.51 26.19           H  0.054
ATOM    338  HE AARG A  21      26.868   9.638   1.929  0.51 30.94           H  0.058
ATOM    339 HH11AARG A  21      24.153  11.024   0.102  0.51 27.44           H  0.055
ATOM    340 HH12AARG A  21      23.096  10.986   1.504  0.51 27.44           H  0.055
ATOM    341 HH21AARG A  21      25.479   9.621   3.663  0.51 33.19           H  0.060
ATOM    342 HH22AARG A  21      23.827  10.209   3.490  0.51 33.19           H  0.060
ATOM    343  N  BARG A  21      25.679  11.417  -3.780  0.49 10.86           N  0.035
ANISOU  343  N  BARG A  21     1593   1029   1506   -425    422   -475       N
ATOM    344  CA BARG A  21      26.853  10.961  -2.975  0.49 12.18           C  0.037
ANISOU  344  CA BARG A  21     1825   1105   1697   -373    354   -453       C
ATOM    345  C  BARG A  21      28.137  11.161  -3.795  0.49 11.31           C  0.035
ANISOU  345  C  BARG A  21     1734    967   1598   -312     13   -373       C
ATOM    346  O  BARG A  21      29.157  10.547  -3.468  0.49 11.14           O  0.035
ANISOU  346  O  BARG A  21     1699    939   1597   -121   -156   -538       O
ATOM    347  CB BARG A  21      26.649   9.524  -2.479  0.49 15.13           C  0.041
ANISOU  347  CB BARG A  21     2189   1300   2259   -504    484   -421       C
ATOM    348  CG BARG A  21      25.726   9.441  -1.277  0.49 18.62           C  0.045
ANISOU  348  CG BARG A  21     2685   1592   2799   -629    536   -673       C
ATOM    349  CD BARG A  21      26.453   9.680   0.024  0.49 21.02           C  0.048
ANISOU  349  CD BARG A  21     2965   1882   3141   -725    699   -683       C
ATOM    350  NE BARG A  21      25.508  10.067   1.058  0.49 24.14           N  0.052
ANISOU  350  NE BARG A  21     3224   2251   3699   -788    553   -579       N
ATOM    351  CZ BARG A  21      25.757  10.065   2.362  0.49 23.90           C  0.051
ANISOU  351  CZ BARG A  21     3335   2433   3311   -795    350   -623       C
ATOM    352  NH1BARG A  21      26.939   9.703   2.827  0.49 23.10           N  0.050
ANISOU  352  NH1BARG A  21     3259   2409   3107   -998     32   -887       N
ATOM    353  NH2BARG A  21      24.810  10.429   3.205  0.49 25.95           N  0.053
ANISOU  353  NH2BARG A  21     3515   2597   3746   -662    134   -423       N
ATOM    354  H  BARG A  21      24.885  11.631  -3.149  0.49 13.04           H  0.038
ATOM    355  HA BARG A  21      26.915  11.606  -2.100  0.49 14.61           H  0.040
ATOM    356  HB2BARG A  21      26.231   8.925  -3.286  0.49 18.16           H  0.045
ATOM    357  HB3BARG A  21      27.616   9.102  -2.210  0.49 18.16           H  0.045
ATOM    358  HG2BARG A  21      24.936  10.183  -1.382  0.49 22.35           H  0.050
ATOM    359  HG3BARG A  21      25.259   8.460  -1.241  0.49 22.35           H  0.050
ATOM    360  HD2BARG A  21      26.968   8.768   0.321  0.49 25.23           H  0.053
ATOM    361  HD3BARG A  21      27.195  10.466  -0.109  0.49 25.23           H  0.053
ATOM    362  HE BARG A  21      24.578  10.348   0.762  0.49 28.97           H  0.056
ATOM    363 HH11BARG A  21      27.680   9.418   2.194  0.49 27.72           H  0.055
ATOM    364 HH12BARG A  21      27.105   9.712   3.829  0.49 27.72           H  0.055
ATOM    365 HH21BARG A  21      23.899  10.707   2.852  0.49 31.14           H  0.059
ATOM    366 HH22BARG A  21      24.997  10.432   4.204  0.49 31.14           H  0.059
ATOM    367  N   GLY A  22      28.086  12.205  -4.693  1.00 11.13           N  0.035
ANISOU  367  N   GLY A  22     1711    797   1720   -353    -38   -468       N
ATOM    368  CA  GLY A  22      29.248  12.499  -5.481  1.00 10.88           C  0.035
ANISOU  368  CA  GLY A  22     1532    756   1847   -292    102   -335       C
ATOM    369  C   GLY A  22      29.486  11.616  -6.688  1.00 11.14           C  0.035
ANISOU  369  C   GLY A  22     1367    943   1922   -225    133    -98       C
ATOM    370  O   GLY A  22      30.533  11.760  -7.325  1.00  8.68           O  0.031
ANISOU  370  O   GLY A  22      991    950   1358   -178     93   -390       O
ATOM    371  HA2 GLY A  22      29.175  13.412  -5.801  1.00 13.06           H  0.038
ATOM    372  HA3 GLY A  22      30.029  12.425  -4.911  1.00 13.06           H  0.038
ATOM    373  H  AGLY A  22      27.405  12.701  -4.861  0.51 13.35           H  0.038
ATOM    374  H  BGLY A  22      27.404  12.698  -4.867  0.49 13.35           H  0.038
ATOM    375  N   TYR A  23      28.569  10.721  -7.039  1.00  9.97           N  0.033
ANISOU  375  N   TYR A  23     1378    792   1617   -121    194   -123       N
ATOM    376  CA  TYR A  23      28.822   9.754  -8.101  1.00  7.31           C  0.028
ANISOU  376  CA  TYR A  23     1278    422   1078   -103     96      4       C
ATOM    377  C   TYR A  23      28.107  10.221  -9.344  1.00  7.02           C  0.028
ANISOU  377  C   TYR A  23     1123    437   1107    -88    234    -53       C
```

```
ATOM    378  O   TYR A  23      26.884  10.287  -9.366  1.00  8.06           O  0.030
ANISOU  378  O   TYR A  23     987    595   1481   -124     40    131       O
ATOM    379  CB  TYR A  23      28.346   8.357  -7.697  1.00  7.71           C  0.029
ANISOU  379  CB  TYR A  23    1135    518   1277    -17     76   -115       C
ATOM    380  CG  TYR A  23      29.268   7.725  -6.697  1.00  7.85           C  0.029
ANISOU  380  CG  TYR A  23    1053    598   1330    -82      2     52       C
ATOM    381  CD1 TYR A  23      30.367   6.993  -7.117  1.00  7.47           C  0.029
ANISOU  381  CD1 TYR A  23    1080    439   1318      8    -30    222       C
ATOM    382  CD2 TYR A  23      29.044   7.813  -5.321  1.00  6.67           C  0.027
ANISOU  382  CD2 TYR A  23     884    542   1109     33    373     72       C
ATOM    383  CE1 TYR A  23      31.232   6.436  -6.199  1.00  7.60           C  0.029
ANISOU  383  CE1 TYR A  23     948    710   1231   -108    -13    247       C
ATOM    384  CE2 TYR A  23      29.881   7.223  -4.433  1.00  7.16           C  0.028
ANISOU  384  CE2 TYR A  23     907    622   1191   -212     30    -19       C
ATOM    385  CZ  TYR A  23      30.972   6.507  -4.885  1.00  6.67           C  0.027
ANISOU  385  CZ  TYR A  23     898    757    880   -234     16     81       C
ATOM    386  OH  TYR A  23      31.834   5.906  -4.030  1.00  7.78           O  0.029
ANISOU  386  OH  TYR A  23     983    920   1055   -182     54   -224       O
ATOM    387  H   TYR A  23      27.792  10.654  -6.677  1.00 11.96           H  0.036
ATOM    388  HA  TYR A  23      29.775   9.714  -8.277  1.00  8.77           H  0.031
ATOM    389  HB2 TYR A  23      27.463   8.422  -7.299  1.00  9.26           H  0.032
ATOM    390  HB3 TYR A  23      28.314   7.790  -8.483  1.00  9.26           H  0.032
ATOM    391  HD1 TYR A  23      30.523   6.877  -8.026  1.00  8.96           H  0.031
ATOM    392  HD2 TYR A  23      28.306   8.285  -5.008  1.00  8.01           H  0.030
ATOM    393  HE1 TYR A  23      32.003   6.006  -6.493  1.00  9.12           H  0.032
ATOM    394  HE2 TYR A  23      29.721   7.300  -3.520  1.00  8.59           H  0.031
ATOM    395  HH  TYR A  23      31.804   6.282  -3.280  1.00  9.34           H  0.032
ATOM    396  N   SER A  24      28.880  10.651 -10.333  1.00  9.06           N  0.032
ANISOU  396  N   SER A  24    1222    880   1338    117    261    -81       N
ATOM    397  CA  SER A  24      28.307  11.130 -11.576  1.00  8.68           C  0.031
ANISOU  397  CA  SER A  24    1386   1024    889    118    276   -136       C
ATOM    398  C   SER A  24      27.436  10.068 -12.247  1.00  8.42           C  0.030
ANISOU  398  C   SER A  24    1277    757   1165    -39     63    243       C
ATOM    399  O   SER A  24      27.610   8.864 -12.052  1.00  8.33           O  0.030
ANISOU  399  O   SER A  24    1312    516   1335   -106   -274    285       O
ATOM    400  CB  SER A  24      29.431  11.531 -12.525  1.00  9.89           C  0.033
ANISOU  400  CB  SER A  24    1164   1359   1237    302    323    310       C
ATOM    401  OG  SER A  24      30.100  10.361 -12.992  1.00 13.18           O  0.038
ANISOU  401  OG  SER A  24    1544   1626   1838    409    538    201       O
ATOM    402  H   SER A  24      29.739  10.675 -10.307  1.00 10.87           H  0.035
ATOM    403  HA  SER A  24      27.741  11.896 -11.390  1.00 10.42           H  0.034
ATOM    404  HB2 SER A  24      29.057  12.010 -13.281  1.00 11.87           H  0.036
ATOM    405  HB3 SER A  24      30.063  12.096 -12.054  1.00 11.87           H  0.036
ATOM    406  HG  SER A  24      30.209  10.404 -13.823  1.00 15.82           H  0.042
ATOM    407  N   LEU A  25      26.518  10.542 -13.082  1.00  8.11           N  0.030
ANISOU  407  N   LEU A  25    1150    816   1115    -37    201    158       N
ATOM    408  CA  LEU A  25      25.522   9.674 -13.714  1.00  7.05           C  0.028
ANISOU  408  CA  LEU A  25     814    524   1341     62    155     37       C
ATOM    409  C   LEU A  25      26.155   8.481 -14.414  1.00  6.91           C  0.028
ANISOU  409  C   LEU A  25     757    423   1446    -85    166    111       C
ATOM    410  O   LEU A  25      25.582   7.390 -14.437  1.00  7.44           O  0.029
ANISOU  410  O   LEU A  25     913    515   1399   -148     36    172       O
ATOM    411  CB  LEU A  25      24.705  10.498 -14.723  1.00  9.76           C  0.033
ANISOU  411  CB  LEU A  25    1327    786   1596     98    100    378       C
ATOM    412  CG  LEU A  25      23.498   9.888 -15.361  1.00 10.16           C  0.033
ANISOU  412  CG  LEU A  25    1220    833   1809   -142    176    196       C
ATOM    413  CD1 LEU A  25      22.412   9.466 -14.354  1.00 10.58           C  0.034
ANISOU  413  CD1 LEU A  25    1121   1024   1873   -157    532    252       C
ATOM    414  CD2 LEU A  25      22.886  10.829 -16.350  1.00 10.10           C  0.033
ANISOU  414  CD2 LEU A  25    1453    847   1538   -276    259    387       C
ATOM    415  H   LEU A  25      26.445  11.369 -13.304  1.00  9.73           H  0.033
ATOM    416  HA  LEU A  25      24.932   9.329 -13.026  1.00  8.46           H  0.030
ATOM    417  HB2 LEU A  25      24.398  11.295 -14.264  1.00 11.72           H  0.036
ATOM    418  HB3 LEU A  25      25.302  10.742 -15.447  1.00 11.72           H  0.036
ATOM    419  HG  LEU A  25      23.811   9.084 -15.805  1.00 12.20           H  0.037
ATOM    420 HD11 LEU A  25      21.605   9.232 -14.839  1.00 12.69           H  0.037
ATOM    421 HD12 LEU A  25      22.729   8.700 -13.850  1.00 12.69           H  0.037
ATOM    422 HD13 LEU A  25      22.233  10.207 -13.753  1.00 12.69           H  0.037
ATOM    423 HD21 LEU A  25      22.214  10.354 -16.865  1.00 12.12           H  0.037
ATOM    424 HD22 LEU A  25      22.475  11.567 -15.873  1.00 12.12           H  0.037
ATOM    425 HD23 LEU A  25      23.580  11.162 -16.940  1.00 12.12           H  0.037
ATOM    426  N   GLY A  26      27.287   8.672 -15.079  1.00  7.85           N  0.029
ANISOU  426  N   GLY A  26     903    509   1570    -72     54      8       N
ATOM    427  CA  GLY A  26      27.892   7.557 -15.791  1.00  7.42           C  0.029
ANISOU  427  CA  GLY A  26    1016    774   1031   -243    -23    255       C
ATOM    428  C   GLY A  26      28.276   6.391 -14.891  1.00  6.39           C  0.027
ANISOU  428  C   GLY A  26     933    667    827   -109    -92    109       C
ATOM    429  O   GLY A  26      28.261   5.233 -15.327  1.00  7.56           O  0.029
```

```
ANISOU  429  O   GLY A  26      1151    801    921    -62    -14    323       O
ATOM    430  H   GLY A  26       27.714   9.417 -15.132  1.00  9.42           H  0.032
ATOM    431  HA2 GLY A  26       27.265   7.229 -16.455  1.00  8.91           H  0.031
ATOM    432  HA3 GLY A  26       28.694   7.866 -16.240  1.00  8.91           H  0.031
ATOM    433  N   ASN A  27       28.624   6.679 -13.624  1.00  5.57           N  0.025
ANISOU  433  N   ASN A  27       785    777    554   -265    -52   -146       N
ATOM    434  CA  ASN A  27       28.888   5.599 -12.666  1.00  5.75           C  0.025
ANISOU  434  CA  ASN A  27       732    608    842   -253   -181    302       C
ATOM    435  C   ASN A  27       27.664   4.699 -12.469  1.00  6.69           C  0.027
ANISOU  435  C   ASN A  27       773    728   1043   -225    121    -81       C
ATOM    436  O   ASN A  27       27.793   3.468 -12.411  1.00  6.19           O  0.026
ANISOU  436  O   ASN A  27       601    987    763   -301   -263     97       O
ATOM    437  CB  ASN A  27       29.325   6.198 -11.320  1.00  5.12           C  0.024
ANISOU  437  CB  ASN A  27       785    553    608   -341   -279    155       C
ATOM    438  CG  ASN A  27       30.759   6.622 -11.338  1.00  6.16           C  0.026
ANISOU  438  CG  ASN A  27      1115    631    594   -381    -15     -7       C
ATOM    439  OD1 ASN A  27       31.638   5.809 -11.308  1.00  6.78           O  0.027
ANISOU  439  OD1 ASN A  27       992    515   1070   -115    -36    -58       O
ATOM    440  ND2 ASN A  27       30.988   7.932 -11.457  1.00  8.17           N  0.030
ANISOU  440  ND2 ASN A  27      1369    685   1052   -456    -23    -35       N
ATOM    441  H   ASN A  27       28.710   7.472 -13.304  1.00  6.68           H  0.027
ATOM    442  HA  ASN A  27       29.607   5.048 -13.013  1.00  6.89           H  0.028
ATOM    443  HB2 ASN A  27       28.781   6.977 -11.125  1.00  6.14           H  0.026
ATOM    444  HB3 ASN A  27       29.214   5.533 -10.623  1.00  6.14           H  0.026
ATOM    445 HD21 ASN A  27       31.796   8.228 -11.473  1.00  9.81           H  0.033
ATOM    446 HD22 ASN A  27       30.329   8.480 -11.518  1.00  9.81           H  0.033
ATOM    447  N   TRP A  28       26.489   5.311 -12.355  1.00  5.92           N  0.026
ANISOU  447  N   TRP A  28       756    641    853   -148    172    277       N
ATOM    448  CA  TRP A  28       25.251   4.580 -12.126  1.00  5.80           C  0.025
ANISOU  448  CA  TRP A  28       606    667    930   -108   -214    365       C
ATOM    449  C   TRP A  28       24.853   3.794 -13.353  1.00  7.00           C  0.028
ANISOU  449  C   TRP A  28       679    903   1077    -54     43    103       C
ATOM    450  O   TRP A  28       24.369   2.676 -13.240  1.00  6.97           O  0.028
ANISOU  450  O   TRP A  28       645    926   1078   -220     47    -28       O
ATOM    451  CB  TRP A  28       24.155   5.553 -11.728  1.00  5.20           C  0.024
ANISOU  451  CB  TRP A  28       655    494    827    -43   -147     98       C
ATOM    452  CG  TRP A  28       24.446   6.237 -10.438  1.00  5.44           C  0.024
ANISOU  452  CG  TRP A  28       823    611    635    -60    -28    261       C
ATOM    453  CD1 TRP A  28       24.921   7.510 -10.271  1.00  6.70           C  0.027
ANISOU  453  CD1 TRP A  28       921    640    983   -178    -54    202       C
ATOM    454  CD2 TRP A  28       24.326   5.687  -9.138  1.00  6.41           C  0.027
ANISOU  454  CD2 TRP A  28       741    735    959    -42    -21    124       C
ATOM    455  NE1 TRP A  28       25.089   7.782  -8.957  1.00  8.38           N  0.030
ANISOU  455  NE1 TRP A  28       855    730   1599    -67   -354    254       N
ATOM    456  CE2 TRP A  28       24.735   6.671  -8.228  1.00  7.96           C  0.030
ANISOU  456  CE2 TRP A  28       833    826   1367    -11     22     80       C
ATOM    457  CE3 TRP A  28       23.932   4.448  -8.656  1.00  6.93           C  0.028
ANISOU  457  CE3 TRP A  28       741   1095    799   -158     11    -32       C
ATOM    458  CZ2 TRP A  28       24.760   6.449  -6.858  1.00  7.36           C  0.028
ANISOU  458  CZ2 TRP A  28       912    961    924   -212    259      8       C
ATOM    459  CZ3 TRP A  28       23.921   4.237  -7.329  1.00  6.73           C  0.027
ANISOU  459  CZ3 TRP A  28       944   1158    457   -338    -33     88       C
ATOM    460  CH2 TRP A  28       24.355   5.217  -6.425  1.00  7.52           C  0.029
ANISOU  460  CH2 TRP A  28      1098   1155    603   -282     30     89       C
ATOM    461  H   TRP A  28       26.380   6.163 -12.408  1.00  7.11           H  0.028
ATOM    462  HA  TRP A  28       25.377   3.952 -11.398  1.00  6.96           H  0.028
ATOM    463  HB2 TRP A  28       24.068   6.232 -12.415  1.00  6.24           H  0.026
ATOM    464  HB3 TRP A  28       23.321   5.069 -11.631  1.00  6.24           H  0.026
ATOM    465  HD1 TRP A  28       25.102   8.103 -10.964  1.00  8.04           H  0.030
ATOM    466  HE1 TRP A  28       25.371   8.527  -8.632  1.00 10.06           H  0.033
ATOM    467  HE3 TRP A  28       23.679   3.773  -9.243  1.00  8.32           H  0.030
ATOM    468  HZ2 TRP A  28       25.039   7.107  -6.263  1.00  8.83           H  0.031
ATOM    469  HZ3 TRP A  28       23.618   3.421  -7.002  1.00  8.08           H  0.030
ATOM    470  HH2 TRP A  28       24.367   5.026  -5.515  1.00  9.02           H  0.031
ATOM    471  N   VAL A  29       24.970   4.388 -14.532  1.00  6.97           N  0.028
ANISOU  471  N   VAL A  29       842    869    938   -253    164    364       N
ATOM    472  CA  VAL A  29       24.694   3.669 -15.777  1.00  6.19           C  0.026
ANISOU  472  CA  VAL A  29       700    859    794   -141   -163    480       C
ATOM    473  C   VAL A  29       25.625   2.483 -15.944  1.00  6.39           C  0.027
ANISOU  473  C   VAL A  29       677    798    954   -240    -42    442       C
ATOM    474  O   VAL A  29       25.192   1.382 -16.294  1.00  6.62           O  0.027
ANISOU  474  O   VAL A  29       688    754   1071   -168   -218     61       O
ATOM    475  CB  VAL A  29       24.753   4.609 -16.998  1.00  6.54           C  0.027
ANISOU  475  CB  VAL A  29       715    711   1061   -198    -23    309       C
ATOM    476  CG1 VAL A  29       24.561   3.800 -18.256  1.00  8.51           C  0.031
ANISOU  476  CG1 VAL A  29       768   1055   1409   -167    -88    514       C
ATOM    477  CG2 VAL A  29       23.661   5.653 -16.874  1.00  8.25           C  0.030
ANISOU  477  CG2 VAL A  29       931    976   1229   -234     81    341       C
```

```
ATOM    478  H   VAL A  29      25.207   5.207 -14.645  1.00  8.37           H  0.030
ATOM    479  HA  VAL A  29      23.789   3.327 -15.714  1.00  7.43           H  0.029
ATOM    480  HB  VAL A  29      25.613   5.057 -17.040  1.00  7.85           H  0.029
ATOM    481 HG11 VAL A  29      24.585   4.395 -19.021  1.00 10.21           H  0.034
ATOM    482 HG12 VAL A  29      25.275   3.146 -18.323  1.00 10.21           H  0.034
ATOM    483 HG13 VAL A  29      23.703   3.349 -18.214  1.00 10.21           H  0.034
ATOM    484 HG21 VAL A  29      23.726   6.268 -17.622  1.00  9.91           H  0.033
ATOM    485 HG22 VAL A  29      22.798   5.210 -16.884  1.00  9.91           H  0.033
ATOM    486 HG23 VAL A  29      23.777   6.134 -16.040  1.00  9.91           H  0.033
ATOM    487  N   CYS A  30      26.930   2.702 -15.736  1.00  6.76           N  0.027
ANISOU  487  N   CYS A  30      681    930    957   -134    -13    448       N
ATOM    488  CA  CYS A  30      27.907   1.624 -15.801  1.00  5.68           C  0.025
ANISOU  488  CA  CYS A  30      641    758    760   -225    152    300       C
ATOM    489  C   CYS A  30      27.558   0.498 -14.826  1.00  4.28           C  0.022
ANISOU  489  C   CYS A  30      610    542    476   -172    112    219       C
ATOM    490  O   CYS A  30      27.614  -0.676 -15.184  1.00  6.13           O  0.026
ANISOU  490  O   CYS A  30      719    519   1091   -286    -82    -13       O
ATOM    491  CB  CYS A  30      29.315   2.206 -15.567  1.00  6.47           C  0.027
ANISOU  491  CB  CYS A  30      881    950    627     15     13     55       C
ATOM    492  SG  CYS A  30      30.635   1.007 -15.760  1.00  7.01           S  0.028
ANISOU  492  SG  CYS A  30      863    862    939   -150     42     99       S
ATOM    493  H   CYS A  30      27.271   3.470 -15.555  1.00  8.11           H  0.030
ATOM    494  HA  CYS A  30      27.904   1.224 -16.684  1.00  6.82           H  0.027
ATOM    495  HB2 CYS A  30      29.468   2.919 -16.207  1.00  7.76           H  0.029
ATOM    496  HB3 CYS A  30      29.362   2.555 -14.663  1.00  7.76           H  0.029
ATOM    497  N   ALA A  31      27.219   0.854 -13.601  1.00  5.50           N  0.025
ANISOU  497  N   ALA A  31      695    627    768   -132    222    293       N
ATOM    498  CA  ALA A  31      26.880  -0.165 -12.615  1.00  5.19           C  0.024
ANISOU  498  CA  ALA A  31      726    510    735   -152    269    179       C
ATOM    499  C   ALA A  31      25.703  -0.987 -13.082  1.00  4.45           C  0.022
ANISOU  499  C   ALA A  31      682    442    567    -80    171    229       C
ATOM    500  O   ALA A  31      25.710  -2.199 -12.960  1.00  5.79           O  0.025
ANISOU  500  O   ALA A  31      763    531    907   -173   -124    353       O
ATOM    501  CB  ALA A  31      26.514   0.476 -11.287  1.00  5.26           C  0.024
ANISOU  501  CB  ALA A  31      827    359    814   -161    -33    213       C
ATOM    502  H   ALA A  31      27.176   1.664 -13.315  1.00  6.60           H  0.027
ATOM    503  HA  ALA A  31      27.658  -0.731 -12.494  1.00  6.22           H  0.026
ATOM    504  HB1 ALA A  31      26.238  -0.218 -10.667  1.00  6.32           H  0.026
ATOM    505  HB2 ALA A  31      27.289   0.943 -10.937  1.00  6.32           H  0.026
ATOM    506  HB3 ALA A  31      25.786   1.101 -11.428  1.00  6.32           H  0.026
ATOM    507  N   ALA A  32      24.656  -0.329 -13.576  1.00  5.41           N  0.024
ANISOU  507  N   ALA A  32      563    535    956   -250   -208    222       N
ATOM    508  CA  ALA A  32      23.477  -1.059 -14.053  1.00  5.24           C  0.024
ANISOU  508  CA  ALA A  32      561    683    745   -304     85    184       C
ATOM    509  C   ALA A  32      23.824  -1.948 -15.215  1.00  4.52           C  0.022
ANISOU  509  C   ALA A  32      660    787    269   -165   -131     36       C
ATOM    510  O   ALA A  32      23.302  -3.060 -15.317  1.00  7.87           O  0.029
ANISOU  510  O   ALA A  32     1071   1011    907   -341   -257     36       O
ATOM    511  CB  ALA A  32      22.394  -0.069 -14.484  1.00  6.78           C  0.027
ANISOU  511  CB  ALA A  32      410    784   1383    -18    168    241       C
ATOM    512  H   ALA A  32      24.600   0.526 -13.648  1.00  6.49           H  0.027
ATOM    513  HA  ALA A  32      23.137  -1.606 -13.327  1.00  6.28           H  0.026
ATOM    514  HB1 ALA A  32      21.637  -0.563 -14.837  1.00  8.14           H  0.030
ATOM    515  HB2 ALA A  32      22.119   0.453 -13.714  1.00  8.14           H  0.030
ATOM    516  HB3 ALA A  32      22.755   0.516 -15.168  1.00  8.14           H  0.030
ATOM    517  N   LYS A  33      24.676  -1.470 -16.134  1.00  6.89           N  0.028
ANISOU  517  N   LYS A  33      795    733   1091   -198     85    337       N
ATOM    518  CA  LYS A  33      25.062  -2.281 -17.279  1.00  7.41           C  0.029
ANISOU  518  CA  LYS A  33      928   1024    862   -168    108    284       C
ATOM    519  C   LYS A  33      25.646  -3.599 -16.816  1.00  7.84           C  0.029
ANISOU  519  C   LYS A  33     1102    712   1163   -171     70    121       C
ATOM    520  O   LYS A  33      25.339  -4.662 -17.360  1.00  8.68           O  0.031
ANISOU  520  O   LYS A  33     1243    600   1454     -1   -236    317       O
ATOM    521  CB  LYS A  33      26.122  -1.546 -18.122  1.00  8.53           C  0.031
ANISOU  521  CB  LYS A  33     1201   1406    633    -34     75    454       C
ATOM    522  CG  LYS A  33      26.727  -2.384 -19.210  1.00 10.72           C  0.034
ANISOU  522  CG  LYS A  33     1496   1822    755   -216    146    493       C
ATOM    523  CD  LYS A  33      25.768  -2.738 -20.278  1.00 12.25           C  0.037
ANISOU  523  CD  LYS A  33     1614   2139    902   -242    169   -179       C
ATOM    524  CE  LYS A  33      26.474  -3.637 -21.299  1.00 15.01           C  0.041
ANISOU  524  CE  LYS A  33     1927   2502   1275   -289    422   -455       C
ATOM    525  NZ  LYS A  33      25.502  -4.038 -22.374  1.00 17.00           N  0.043
ANISOU  525  NZ  LYS A  33     2174   2674   1611   -360    179    -73       N
ATOM    526  H   LYS A  33      25.036  -0.690 -16.112  1.00  8.27           H  0.030
ATOM    527  HA  LYS A  33      24.274  -2.437 -17.823  1.00  8.89           H  0.031
ATOM    528  HB2 LYS A  33      25.707  -0.776 -18.540  1.00 10.23           H  0.034
ATOM    529  HB3 LYS A  33      26.840  -1.259 -17.537  1.00 10.23           H  0.034
ATOM    530  HG2 LYS A  33      27.457  -1.891 -19.617  1.00 12.86           H  0.038
```

```
ATOM    531  HG3 LYS A  33      27.059  -3.209 -18.823  1.00 12.86           H  0.038
ATOM    532  HD2 LYS A  33      25.014  -3.218 -19.901  1.00 14.70           H  0.040
ATOM    533  HD3 LYS A  33      25.458  -1.934 -20.726  1.00 14.70           H  0.040
ATOM    534  HE2 LYS A  33      27.210  -3.156 -21.706  1.00 18.02           H  0.045
ATOM    535  HE3 LYS A  33      26.802  -4.437 -20.859  1.00 18.02           H  0.045
ATOM    536  HZ1 LYS A  33      24.788  -4.425 -22.010  1.00 20.40           H  0.047
ATOM    537  HZ2 LYS A  33      25.241  -3.321 -22.832  1.00 20.40           H  0.047
ATOM    538  HZ3 LYS A  33      25.888  -4.613 -22.934  1.00 20.40           H  0.047
ATOM    539  N   PHE A  34      26.561  -3.537 -15.868  1.00  6.65           N  0.027
ANISOU  539  N   PHE A  34     1101    565    862     -7    163     67       N
ATOM    540  CA  PHE A  34      27.285  -4.745 -15.494  1.00  6.81           C  0.027
ANISOU  540  CA  PHE A  34      964    568   1056    -61    170    322       C
ATOM    541  C   PHE A  34      26.574  -5.542 -14.414  1.00  9.75           C  0.033
ANISOU  541  C   PHE A  34     1164    705   1834    -40    138    493       C
ATOM    542  O   PHE A  34      26.801  -6.758 -14.298  1.00 10.23           O  0.034
ANISOU  542  O   PHE A  34     1538    847   1503    331    527    502       O
ATOM    543  CB  PHE A  34      28.725  -4.408 -15.150  1.00  5.39           C  0.024
ANISOU  543  CB  PHE A  34      790    706    553    195    206   -238       C
ATOM    544  CG  PHE A  34      29.522  -3.909 -16.349  1.00  6.26           C  0.026
ANISOU  544  CG  PHE A  34      926    854    599    362    186   -144       C
ATOM    545  CD1 PHE A  34      29.522  -4.627 -17.544  1.00  6.46           C  0.027
ANISOU  545  CD1 PHE A  34      845    871    737    317    299   -150       C
ATOM    546  CD2 PHE A  34      30.199  -2.704 -16.315  1.00  9.28           C  0.032
ANISOU  546  CD2 PHE A  34     1104    933   1488    261    415   -376       C
ATOM    547  CE1 PHE A  34      30.238  -4.193 -18.601  1.00  7.24           C  0.028
ANISOU  547  CE1 PHE A  34     1016    808    926    268    181     55       C
ATOM    548  CE2 PHE A  34      30.960  -2.291 -17.408  1.00 11.74           C  0.036
ANISOU  548  CE2 PHE A  34     1349   1035   2075    266    518   -222       C
ATOM    549  CZ  PHE A  34      30.914  -3.032 -18.559  1.00  9.94           C  0.033
ANISOU  549  CZ  PHE A  34     1306    938   1533     73     53    124       C
ATOM    550  H   PHE A  34      26.780  -2.827 -15.435  1.00  7.99           H  0.030
ATOM    551  HA  PHE A  34      27.352  -5.349 -16.250  1.00  8.18           H  0.030
ATOM    552  HB2 PHE A  34      28.733  -3.713 -14.474  1.00  6.47           H  0.027
ATOM    553  HB3 PHE A  34      29.162  -5.205 -14.811  1.00  6.47           H  0.027
ATOM    554  HD1 PHE A  34      29.025  -5.411 -17.613  1.00  7.75           H  0.029
ATOM    555  HD2 PHE A  34      30.145  -2.165 -15.558  1.00 11.13           H  0.035
ATOM    556  HE1 PHE A  34      30.265  -4.708 -19.375  1.00  8.69           H  0.031
ATOM    557  HE2 PHE A  34      31.490  -1.529 -17.356  1.00 14.09           H  0.039
ATOM    558  HZ  PHE A  34      31.355  -2.728 -19.319  1.00 11.93           H  0.036
ATOM    559  N   GLU A  35      25.741  -4.913 -13.635  1.00  7.64           N  0.029
ANISOU  559  N   GLU A  35      933    656   1316    -40   -157    287       N
ATOM    560  CA  GLU A  35      24.935  -5.676 -12.704  1.00  7.50           C  0.029
ANISOU  560  CA  GLU A  35     1153    703    995     88   -118     17       C
ATOM    561  C   GLU A  35      23.838  -6.464 -13.421  1.00  7.01           C  0.028
ANISOU  561  C   GLU A  35     1369    691    604    110   -105    110       C
ATOM    562  O   GLU A  35      23.583  -7.615 -13.075  1.00  8.33           O  0.030
ANISOU  562  O   GLU A  35     1769    515    879     23   -107    -12       O
ATOM    563  CB  GLU A  35      24.327  -4.762 -11.670  1.00  6.56           C  0.027
ANISOU  563  CB  GLU A  35      830    740    923    173    185    -16       C
ATOM    564  CG  GLU A  35      25.290  -4.177 -10.689  1.00  7.35           C  0.028
ANISOU  564  CG  GLU A  35      955    898    939    -57    106    289       C
ATOM    565  CD  GLU A  35      25.916  -5.207  -9.748  1.00  9.20           C  0.032
ANISOU  565  CD  GLU A  35     1152   1078   1264   -121   -134     48       C
ATOM    566  OE1 GLU A  35      25.506  -6.385  -9.733  1.00  8.55           O  0.031
ANISOU  566  OE1 GLU A  35     1113   1023   1111   -125   -395    106       O
ATOM    567  OE2 GLU A  35      26.879  -4.845  -9.042  1.00  9.61           O  0.033
ANISOU  567  OE2 GLU A  35     1461   1287    903   -208   -448   -232       O
ATOM    568  H   GLU A  35      25.618  -4.062 -13.619  1.00  9.17           H  0.032
ATOM    569  HA  GLU A  35      25.515  -6.309 -12.253  1.00  9.00           H  0.031
ATOM    570  HB2 GLU A  35      23.898  -4.023 -12.130  1.00  7.87           H  0.029
ATOM    571  HB3 GLU A  35      23.670  -5.266 -11.166  1.00  7.87           H  0.029
ATOM    572  HG2 GLU A  35      26.010  -3.747 -11.176  1.00  8.82           H  0.031
ATOM    573  HG3 GLU A  35      24.822  -3.525 -10.143  1.00  8.82           H  0.031
ATOM    574  N   SER A  36      23.106  -5.835 -14.368  1.00  7.62           N  0.029
ANISOU  574  N   SER A  36     1186   1003    707   -243   -370    182       N
ATOM    575  CA  SER A  36      21.851  -6.397 -14.854  1.00  8.14           C  0.030
ANISOU  575  CA  SER A  36     1450    936    708   -289   -126    -10       C
ATOM    576  C   SER A  36      21.703  -6.407 -16.366  1.00  9.64           C  0.033
ANISOU  576  C   SER A  36     1482    934   1247   -429     24    273       C
ATOM    577  O   SER A  36      20.672  -6.898 -16.864  1.00 10.66           O  0.034
ANISOU  577  O   SER A  36     1567    963   1522   -557     56    342       O
ATOM    578  CB  SER A  36      20.650  -5.606 -14.333  1.00  9.14           C  0.032
ANISOU  578  CB  SER A  36     1418   1038   1016    -72   -116     61       C
ATOM    579  OG  SER A  36      20.647  -4.278 -14.849  1.00  7.58           O  0.029
ANISOU  579  OG  SER A  36     1134    934    811    -82   -326    262       O
ATOM    580  H   SER A  36      23.324  -5.088 -14.735  1.00  9.15           H  0.032
ATOM    581  HA  SER A  36      21.824  -7.307 -14.519  1.00  9.77           H  0.033
ATOM    582  HB2 SER A  36      19.835  -6.053 -14.610  1.00 10.97           H  0.035
```

```
ATOM    583  HB3 SER A  36      20.695  -5.567 -13.365  1.00 10.97           H  0.035
ATOM    584  HG  SER A  36      21.364  -3.890 -14.648  1.00  9.09           H  0.032
ATOM    585  N   ASN A  37      22.707  -5.922 -17.085  1.00  9.92           N  0.033
ANISOU  585  N   ASN A  37     1524    899   1346   -442     88     98       N
ATOM    586  CA  ASN A  37      22.596  -5.630 -18.511  1.00  9.05           C  0.032
ANISOU  586  CA  ASN A  37     1424   1055    958   -715   -123     59       C
ATOM    587  C   ASN A  37      21.359  -4.799 -18.832  1.00  8.13           C  0.030
ANISOU  587  C   ASN A  37     1329   1139    621   -618   -174    167       C
ATOM    588  O   ASN A  37      20.750  -4.946 -19.884  1.00  9.51           O  0.032
ANISOU  588  O   ASN A  37     1378   1159   1076   -409   -499   -203       O
ATOM    589  CB  ASN A  37      22.684  -6.886 -19.371  1.00 11.14           C  0.035
ANISOU  589  CB  ASN A  37     1772   1404   1057   -566   -368    201       C
ATOM    590  CG  ASN A  37      23.093  -6.581 -20.806  1.00 14.88           C  0.040
ANISOU  590  CG  ASN A  37     2233   1635   1787   -620   -151    102       C
ATOM    591  OD1 ASN A  37      23.635  -5.516 -21.096  1.00 17.21           O  0.043
ANISOU  591  OD1 ASN A  37     2539   1960   2039   -752    156    262       O
ATOM    592  ND2 ASN A  37      22.812  -7.507 -21.709  1.00 16.81           N  0.043
ANISOU  592  ND2 ASN A  37     2319   1805   2264   -389   -103   -300       N
ATOM    593  H   ASN A  37      23.485  -5.747 -16.762  1.00 11.90           H  0.036
ATOM    594  HA  ASN A  37      23.361  -5.084 -18.748  1.00 10.86           H  0.035
ATOM    595  HB2 ASN A  37      23.345  -7.485 -18.991  1.00 13.37           H  0.038
ATOM    596  HB3 ASN A  37      21.816  -7.318 -19.391  1.00 13.37           H  0.038
ATOM    597 HD21 ASN A  37      23.024  -7.381 -22.534  1.00 20.18           H  0.047
ATOM    598 HD22 ASN A  37      22.419  -8.234 -21.472  1.00 20.18           H  0.047
ATOM    599  N   PHE A  38      20.995  -3.888 -17.924  1.00  7.64           N  0.029
ANISOU  599  N   PHE A  38     1170   1032    700   -531   -196    -44       N
ATOM    600  CA  PHE A  38      19.920  -2.933 -18.065  1.00  9.35           C  0.032
ANISOU  600  CA  PHE A  38     1192   1108   1254   -572    -31    120       C
ATOM    601  C   PHE A  38      18.543  -3.587 -18.006  1.00  9.96           C  0.033
ANISOU  601  C   PHE A  38     1241   1164   1379   -634   -340    252       C
ATOM    602  O   PHE A  38      17.559  -2.978 -18.406  1.00 10.02           O  0.033
ANISOU  602  O   PHE A  38     1271   1326   1211   -639    -74    471       O
ATOM    603  CB  PHE A  38      20.029  -2.124 -19.351  1.00  8.39           C  0.030
ANISOU  603  CB  PHE A  38     1097    991   1100   -586     33    199       C
ATOM    604  CG  PHE A  38      21.278  -1.275 -19.490  1.00  8.68           C  0.031
ANISOU  604  CG  PHE A  38     1355   1014    930   -367     -1    474       C
ATOM    605  CD1 PHE A  38      21.852  -0.598 -18.423  1.00 10.33           C  0.034
ANISOU  605  CD1 PHE A  38     1482   1267   1174   -443    122    569       C
ATOM    606  CD2 PHE A  38      21.815  -1.108 -20.738  1.00 10.07           C  0.033
ANISOU  606  CD2 PHE A  38     1653   1187    986   -286     15    573       C
ATOM    607  CE1 PHE A  38      22.951   0.237 -18.630  1.00  8.55           C  0.031
ANISOU  607  CE1 PHE A  38     1407   1155    686   -455    132    259       C
ATOM    608  CE2 PHE A  38      22.933  -0.284 -20.962  1.00  9.29           C  0.032
ANISOU  608  CE2 PHE A  38     1580   1221    730   -355   -151    416       C
ATOM    609  CZ  PHE A  38      23.490   0.376 -19.926  1.00  9.44           C  0.032
ANISOU  609  CZ  PHE A  38     1323   1154   1109   -481     21    503       C
ATOM    610  H   PHE A  38      21.392  -3.808 -17.166  1.00  9.17           H  0.032
ATOM    611  HA  PHE A  38      19.995  -2.316 -17.320  1.00 11.23           H  0.035
ATOM    612  HB2 PHE A  38      20.015  -2.740 -20.100  1.00 10.07           H  0.033
ATOM    613  HB3 PHE A  38      19.267  -1.526 -19.399  1.00 10.07           H  0.033
ATOM    614  HD1 PHE A  38      21.503  -0.700 -17.567  1.00 12.39           H  0.037
ATOM    615  HD2 PHE A  38      21.430  -1.552 -21.459  1.00 12.08           H  0.036
ATOM    616  HE1 PHE A  38      23.327   0.699 -17.916  1.00 10.26           H  0.034
ATOM    617  HE2 PHE A  38      23.285  -0.195 -21.818  1.00 11.15           H  0.035
ATOM    618  HZ  PHE A  38      24.229   0.922 -20.067  1.00 11.33           H  0.035
ATOM    619  N   ASN A  39      18.457  -4.825 -17.504  1.00  8.22           N  0.030
ANISOU  619  N   ASN A  39     1099   1064    961   -546   -153    314       N
ATOM    620  CA  ASN A  39      17.182  -5.537 -17.385  1.00  7.08           C  0.028
ANISOU  620  CA  ASN A  39      973   1173    543   -427   -171    263       C
ATOM    621  C   ASN A  39      16.643  -5.356 -15.969  1.00  7.77           C  0.029
ANISOU  621  C   ASN A  39      853   1040   1058   -408   -177    220       C
ATOM    622  O   ASN A  39      17.201  -5.925 -15.025  1.00  8.65           O  0.031
ANISOU  622  O   ASN A  39     1039    955   1294   -400   -204    183       O
ATOM    623  CB  ASN A  39      17.414  -7.010 -17.650  1.00  9.62           C  0.033
ANISOU  623  CB  ASN A  39     1132   1359   1165   -364    121    231       C
ATOM    624  CG  ASN A  39      16.114  -7.799 -17.789  1.00  9.76           C  0.033
ANISOU  624  CG  ASN A  39     1130   1288   1289   -333     17     -1       C
ATOM    625  OD1 ASN A  39      15.058  -7.346 -17.408  1.00  9.37           O  0.032
ANISOU  625  OD1 ASN A  39     1071    971   1518   -173   -157    432       O
ATOM    626  ND2 ASN A  39      16.214  -9.002 -18.336  1.00 11.62           N  0.036
ANISOU  626  ND2 ASN A  39     1196   1340   1881   -220   -202   -204       N
ATOM    627  H   ASN A  39      19.133  -5.277 -17.222  1.00  9.87           H  0.033
ATOM    628  HA  ASN A  39      16.540  -5.170 -18.013  1.00  8.50           H  0.031
ATOM    629  HB2 ASN A  39      17.913  -7.107 -18.476  1.00 11.55           H  0.036
ATOM    630  HB3 ASN A  39      17.917  -7.388 -16.912  1.00 11.55           H  0.036
ATOM    631 HD21 ASN A  39      15.512  -9.488 -18.437  1.00 13.95           H  0.039
ATOM    632 HD22 ASN A  39      16.981  -9.297 -18.589  1.00 13.95           H  0.039
ATOM    633  N   THR A  40      15.514  -4.653 -15.822  1.00  7.84           N  0.029
```

```
ANISOU  633  N   THR A  40      764    957   1256   -310   -175    170       N
ATOM    634  CA  THR A  40      14.908  -4.478 -14.504  1.00  9.25           C  0.032
ANISOU  634  CA  THR A  40      881   1071   1563   -409   -110    506       C
ATOM    635  C   THR A  40      14.511  -5.791 -13.852  1.00  6.78           C  0.027
ANISOU  635  C   THR A  40      946    889    740   -338   -155    348       C
ATOM    636  O   THR A  40      14.410  -5.837 -12.627  1.00  7.76           O  0.029
ANISOU  636  O   THR A  40     1052   1071    825   -459     36    359       O
ATOM    637  CB  THR A  40      13.678  -3.569 -14.567  1.00 10.67           C  0.034
ANISOU  637  CB  THR A  40      999   1002   2053   -251    -76    290       C
ATOM    638  OG1 THR A  40      12.621  -4.218 -15.308  1.00 13.39           O  0.038
ANISOU  638  OG1 THR A  40      986   1238   2864   -207   -594    607       O
ATOM    639  CG2 THR A  40      13.995  -2.254 -15.283  1.00  9.39           C  0.032
ANISOU  639  CG2 THR A  40      835    967   1767   -339    -60    162       C
ATOM    640  H   THR A  40      15.086  -4.273 -16.464  1.00  9.40           H  0.032
ATOM    641  HA  THR A  40      15.582  -4.050 -13.953  1.00 11.10           H  0.035
ATOM    642  HB  THR A  40      13.397  -3.381 -13.657  1.00 12.81           H  0.038
ATOM    643  HG1 THR A  40      12.006  -4.451 -14.786  1.00 16.07           H  0.042
ATOM    644 HG21 THR A  40      13.345  -1.577 -15.038  1.00 11.27           H  0.035
ATOM    645 HG22 THR A  40      14.880  -1.945 -15.034  1.00 11.27           H  0.035
ATOM    646 HG23 THR A  40      13.966  -2.385 -16.244  1.00 11.27           H  0.035
ATOM    647  N   GLN A  41      14.230  -6.830 -14.631  1.00  7.51           N  0.029
ANISOU  647  N   GLN A  41      906    868   1080   -464   -270    299       N
ATOM    648  CA  GLN A  41      13.773  -8.116 -14.098  1.00  7.19           C  0.028
ANISOU  648  CA  GLN A  41     1104    810    816   -508   -302    112       C
ATOM    649  C   GLN A  41      14.888  -9.088 -13.759  1.00  8.73           C  0.031
ANISOU  649  C   GLN A  41     1256    824   1237   -509   -352    314       C
ATOM    650  O   GLN A  41      14.595 -10.228 -13.364  1.00 10.95           O  0.035
ANISOU  650  O   GLN A  41     1539    959   1664   -400   -468    589       O
ATOM    651  CB  GLN A  41      12.775  -8.744 -15.084  1.00  7.71           C  0.029
ANISOU  651  CB  GLN A  41     1295    883    752   -444   -327    280       C
ATOM    652  CG  GLN A  41      11.641  -7.831 -15.398  1.00  8.73           C  0.031
ANISOU  652  CG  GLN A  41     1298   1184    837   -458   -390    443       C
ATOM    653  CD  GLN A  41      10.514  -8.580 -16.142  1.00  9.76           C  0.033
ANISOU  653  CD  GLN A  41     1336   1104   1269   -478   -456    572       C
ATOM    654  OE1 GLN A  41      10.352  -8.514 -17.355  1.00 10.01           O  0.033
ANISOU  654  OE1 GLN A  41     1477   1203   1124   -370   -262     47       O
ATOM    655  NE2 GLN A  41       9.819  -9.364 -15.398  1.00  8.85           N  0.031
ANISOU  655  NE2 GLN A  41      966   1216   1181   -521   -457    326       N
ATOM    656  H   GLN A  41      14.297  -6.818 -15.488  1.00  9.02           H  0.031
ATOM    657  HA  GLN A  41      13.309  -7.958 -13.261  1.00  8.62           H  0.031
ATOM    658  HB2 GLN A  41      13.234  -8.952 -15.912  1.00  9.25           H  0.032
ATOM    659  HB3 GLN A  41      12.411  -9.555 -14.694  1.00  9.25           H  0.032
ATOM    660  HG2 GLN A  41      11.278  -7.471 -14.573  1.00 10.48           H  0.034
ATOM    661  HG3 GLN A  41      11.956  -7.109 -15.964  1.00 10.48           H  0.034
ATOM    662 HE21 GLN A  41      10.001  -9.435 -14.561  1.00 10.62           H  0.034
ATOM    663 HE22 GLN A  41       9.172  -9.818 -15.738  1.00 10.62           H  0.034
ATOM    664  N   ALA A  42      16.152  -8.690 -13.878  1.00  8.87           N  0.031
ANISOU  664  N   ALA A  42     1308    913   1149   -534   -508    416       N
ATOM    665  CA  ALA A  42      17.258  -9.567 -13.547  1.00  9.60           C  0.032
ANISOU  665  CA  ALA A  42     1556   1074   1019   -300   -120    -96       C
ATOM    666  C   ALA A  42      17.170 -10.023 -12.080  1.00 10.14           C  0.033
ANISOU  666  C   ALA A  42     1600   1208   1046   -158     41    -49       C
ATOM    667  O   ALA A  42      16.960  -9.214 -11.179  1.00  7.54           O  0.029
ANISOU  667  O   ALA A  42     1133   1051    679     75   -133     40       O
ATOM    668  CB  ALA A  42      18.566  -8.802 -13.741  1.00  8.43           C  0.030
ANISOU  668  CB  ALA A  42     1539   1085    579   -408    -22     38       C
ATOM    669  H   ALA A  42      16.394  -7.911 -14.151  1.00 10.64           H  0.034
ATOM    670  HA  ALA A  42      17.235 -10.349 -14.120  1.00 11.52           H  0.036
ATOM    671  HB1 ALA A  42      19.302  -9.352 -13.430  1.00 10.12           H  0.033
ATOM    672  HB2 ALA A  42      18.677  -8.602 -14.683  1.00 10.12           H  0.033
ATOM    673  HB3 ALA A  42      18.529  -7.979 -13.229  1.00 10.12           H  0.033
ATOM    674  N   THR A  43      17.339 -11.318 -11.858  1.00  8.95           N  0.031
ANISOU  674  N   THR A  43     1779   1068    556   -268   -299     12       N
ATOM    675  CA  THR A  43      17.535 -11.874 -10.526  1.00  9.40           C  0.032
ANISOU  675  CA  THR A  43     1919   1025    626   -147    -62    159       C
ATOM    676  C   THR A  43      18.726 -12.816 -10.532  1.00  8.57           C  0.031
ANISOU  676  C   THR A  43     1683   1140    432    -78    -10     80       C
ATOM    677  O   THR A  43      19.026 -13.472 -11.534  1.00 11.24           O  0.035
ANISOU  677  O   THR A  43     2263   1321    688    266    114    250       O
ATOM    678  CB  THR A  43      16.287 -12.616 -10.048  1.00 10.09           C  0.033
ANISOU  678  CB  THR A  43     2068    856    909   -343     26    106       C
ATOM    679  OG1 THR A  43      16.029 -13.713 -10.932  1.00 11.33           O  0.035
ANISOU  679  OG1 THR A  43     2087    927   1293   -255    -22     41       O
ATOM    680  CG2 THR A  43      15.069 -11.694  -9.984  1.00  9.84           C  0.033
ANISOU  680  CG2 THR A  43     2026    862    849   -442     76     77       C
ATOM    681  H   THR A  43      17.345 -11.913 -12.479  1.00 10.74           H  0.034
ATOM    682  HA  THR A  43      17.725 -11.159  -9.899  1.00 11.28           H  0.035
ATOM    683  HB  THR A  43      16.436 -12.947  -9.148  1.00 12.11           H  0.036
```

```
ATOM    684  HG1 THR A  43      15.949 -13.434 -11.720  1.00 13.60           H  0.039
ATOM    685 HG21 THR A  43      14.283 -12.200  -9.724  1.00 11.81           H  0.036
ATOM    686 HG22 THR A  43      15.219 -10.990  -9.333  1.00 11.81           H  0.036
ATOM    687 HG23 THR A  43      14.911 -11.291 -10.852  1.00 11.81           H  0.036
ATOM    688  N  AASN A  44      19.395 -12.904  -9.390  0.53  8.14           N  0.030
ANISOU  688  N  AASN A  44     1588   1022    484   -116    149    118       N
ATOM    689  CA AASN A  44      20.526 -13.808  -9.245  0.53  8.11           C  0.030
ANISOU  689  CA AASN A  44     1401   1151    530    -26    338    163       C
ATOM    690  C  AASN A  44      20.463 -14.360  -7.832  0.53  8.53           C  0.031
ANISOU  690  C  AASN A  44     1373   1194    676   -297    336    166       C
ATOM    691  O  AASN A  44      20.495 -13.583  -6.873  0.53  8.98           O  0.031
ANISOU  691  O  AASN A  44     1294   1041   1078   -571     45    -20       O
ATOM    692  CB AASN A  44      21.844 -13.081  -9.492  0.53 11.65           C  0.036
ANISOU  692  CB AASN A  44     1464   1581   1383    262    563    141       C
ATOM    693  CG AASN A  44      23.049 -13.965  -9.286  0.53 14.84           C  0.040
ANISOU  693  CG AASN A  44     1539   1954   2145    302    500     56       C
ATOM    694  OD1AASN A  44      23.280 -14.927 -10.042  0.53 16.63           O  0.043
ANISOU  694  OD1AASN A  44     1573   2153   2591    243    537    -35       O
ATOM    695  ND2AASN A  44      23.842 -13.636  -8.274  0.53 14.62           N  0.040
ANISOU  695  ND2AASN A  44     1665   2126   1765    442    331    243       N
ATOM    696  H  AASN A  44      19.211 -12.450  -8.684  0.53  9.77           H  0.033
ATOM    697  HA AASN A  44      20.473 -14.540  -9.879  0.53  9.73           H  0.033
ATOM    698  HB2AASN A  44      21.861 -12.760 -10.407  0.53 13.99           H  0.039
ATOM    699  HB3AASN A  44      21.912 -12.333  -8.877  0.53 13.99           H  0.039
ATOM    700 HD21AASN A  44      24.545 -14.104  -8.109  0.53 17.55           H  0.044
ATOM    701 HD22AASN A  44      23.654 -12.955  -7.784  0.53 17.55           H  0.044
ATOM    702  N  BASN A  44      19.411 -12.912  -9.400  0.47  8.24           N  0.030
ANISOU  702  N  BASN A  44     1669    913    551   -183     63    -48       N
ATOM    703  CA BASN A  44      20.499 -13.881  -9.310  0.47  8.13           C  0.030
ANISOU  703  CA BASN A  44     1568    904    617   -117    199   -178       C
ATOM    704  C  BASN A  44      20.572 -14.414  -7.888  0.47  8.57           C  0.031
ANISOU  704  C  BASN A  44     1342   1071    842   -127    194    -41       C
ATOM    705  O  BASN A  44      20.731 -13.645  -6.937  0.47  7.86           O  0.029
ANISOU  705  O  BASN A  44     1245    969    773   -161     72   -159       O
ATOM    706  CB BASN A  44      21.828 -13.307  -9.796  0.47 11.81           C  0.036
ANISOU  706  CB BASN A  44     1847   1055   1586   -130    307   -243       C
ATOM    707  CG BASN A  44      21.872 -13.157 -11.338  0.47 13.49           C  0.039
ANISOU  707  CG BASN A  44     2018   1184   1924   -275    429   -490       C
ATOM    708  OD1BASN A  44      22.000 -14.146 -12.070  0.47 13.35           O  0.038
ANISOU  708  OD1BASN A  44     2143   1192   1738   -301    404   -279       O
ATOM    709  ND2BASN A  44      21.749 -11.913 -11.826  0.47 16.27           N  0.042
ANISOU  709  ND2BASN A  44     1984   1397   2799   -214    496   -998       N
ATOM    710  H  BASN A  44      19.271 -12.443  -8.693  0.47  9.89           H  0.033
ATOM    711  HA BASN A  44      20.300 -14.636  -9.886  0.47  9.76           H  0.033
ATOM    712  HB2BASN A  44      21.958 -12.429  -9.404  0.47 14.17           H  0.039
ATOM    713  HB3BASN A  44      22.548 -13.900  -9.530  0.47 14.17           H  0.039
ATOM    714 HD21BASN A  44      21.768 -11.781 -12.676  0.47 19.52           H  0.046
ATOM    715 HD22BASN A  44      21.651 -11.248 -11.289  0.47 19.52           H  0.046
ATOM    716  N  AARG A  45      20.380 -15.688  -7.710  0.53  8.79           N  0.031
ANISOU  716  N  AARG A  45     1348   1411    579   -179    238    211       N
ATOM    717  CA AARG A  45      20.477 -16.359  -6.422  0.53  9.18           C  0.032
ANISOU  717  CA AARG A  45     1421   1597    468    -10    114    230       C
ATOM    718  C  AARG A  45      21.935 -16.579  -6.061  0.53 10.43           C  0.034
ANISOU  718  C  AARG A  45     1286   2117    563    326     59     31       C
ATOM    719  O  AARG A  45      22.717 -17.114  -6.854  0.53 13.08           O  0.038
ANISOU  719  O  AARG A  45     1565   2325   1082    516    129    -37       O
ATOM    720  CB AARG A  45      19.751 -17.710  -6.444  0.53 11.82           C  0.036
ANISOU  720  CB AARG A  45     1907   1789    795    -80    206    421       C
ATOM    721  CG AARG A  45      19.598 -18.383  -5.041  0.53 13.33           C  0.038
ANISOU  721  CG AARG A  45     2193   1780   1091    -44    -39    434       C
ATOM    722  CD AARG A  45      18.154 -18.397  -4.614  0.53 12.14           C  0.037
ANISOU  722  CD AARG A  45     2207   1721    686    -83    131    365       C
ATOM    723  NE AARG A  45      17.945 -18.538  -3.175  0.53 14.25           N  0.040
ANISOU  723  NE AARG A  45     2560   1806   1049    -46   -114    293       N
ATOM    724  CZ AARG A  45      17.078 -19.368  -2.607  0.53 16.18           C  0.042
ANISOU  724  CZ AARG A  45     2628   1839   1680    -46   -470    178       C
ATOM    725  NH1AARG A  45      16.371 -20.231  -3.321  0.53 15.61           N  0.041
ANISOU  725  NH1AARG A  45     2631   1730   1570      6   -723    -50       N
ATOM    726  NH2AARG A  45      16.884 -19.299  -1.291  0.53 16.95           N  0.043
ANISOU  726  NH2AARG A  45     2678   1855   1905   -165   -581    175       N
ATOM    727  H  AARG A  45      20.266 -16.226  -8.371  0.53 10.54           H  0.034
ATOM    728  HA AARG A  45      20.059 -15.798  -5.750  0.53 11.01           H  0.035
ATOM    729  HB2AARG A  45      18.861 -17.577  -6.805  0.53 14.18           H  0.039
ATOM    730  HB3AARG A  45      20.251 -18.320  -7.009  0.53 14.18           H  0.039
ATOM    731  HG2AARG A  45      19.916 -19.298  -5.083  0.53 16.00           H  0.042
ATOM    732  HG3AARG A  45      20.109 -17.884  -4.384  0.53 16.00           H  0.042
ATOM    733  HD2AARG A  45      17.743 -17.563  -4.888  0.53 14.57           H  0.040
ATOM    734  HD3AARG A  45      17.712 -19.144  -5.047  0.53 14.57           H  0.040
```

```
ATOM    735  HE AARG A  45      18.420 -18.045  -2.656  0.53 17.10           H  0.043
ATOM    736 HH11AARG A  45      16.468 -20.264  -4.175  0.53 18.73           H  0.045
ATOM    737 HH12AARG A  45      15.816 -20.759  -2.930  0.53 18.73           H  0.045
ATOM    738 HH21AARG A  45      17.318 -18.723  -0.822  0.53 20.33           H  0.047
ATOM    739 HH22AARG A  45      16.325 -19.830  -0.910  0.53 20.33           H  0.047
ATOM    740  N  BARG A  45      20.480 -15.733  -7.760  0.47  8.98           N  0.031
ANISOU  740  N  BARG A  45     1204   1302    905     35    137     34       N
ATOM    741  CA BARG A  45      20.495 -16.419  -6.477  0.47  9.41           C  0.032
ANISOU  741  CA BARG A  45     1166   1592    817    171    131    123       C
ATOM    742  C  BARG A  45      21.931 -16.670  -6.042  0.47 10.44           C  0.034
ANISOU  742  C  BARG A  45     1159   2017    789    373    162    -84       C
ATOM    743  O  BARG A  45      22.698 -17.316  -6.763  0.47 12.60           O  0.037
ANISOU  743  O  BARG A  45     1417   2048   1322    494    398   -292       O
ATOM    744  CB BARG A  45      19.728 -17.734  -6.634  0.47 12.02           C  0.036
ANISOU  744  CB BARG A  45     1445   1647   1475    190    173    340       C
ATOM    745  CG BARG A  45      19.779 -18.648  -5.433  0.47 12.67           C  0.037
ANISOU  745  CG BARG A  45     1455   1650   1708    314    135    517       C
ATOM    746  CD BARG A  45      18.963 -18.104  -4.292  0.47  9.65           C  0.033
ANISOU  746  CD BARG A  45     1252   1510    904    367    227    609       C
ATOM    747  NE BARG A  45      17.698 -17.474  -4.666  0.47 11.03           N  0.035
ANISOU  747  NE BARG A  45     1279   1613   1299    180    287    633       N
ATOM    748  CZ BARG A  45      16.573 -18.132  -4.905  0.47 10.23           C  0.034
ANISOU  748  CZ BARG A  45     1126   1697   1062    143    -35    585       C
ATOM    749  NH1BARG A  45      16.536 -19.449  -4.925  0.47 11.92           N  0.036
ANISOU  749  NH1BARG A  45     1092   1933   1505      1    188    346       N
ATOM    750  NH2BARG A  45      15.457 -17.447  -5.124  0.47 11.25           N  0.035
ANISOU  750  NH2BARG A  45     1224   1786   1266    -11   -329    493       N
ATOM    751  H  BARG A  45      20.405 -16.271  -8.427  0.47 10.77           H  0.034
ATOM    752  HA BARG A  45      20.083 -15.874  -5.788  0.47 11.29           H  0.035
ATOM    753  HB2BARG A  45      18.795 -17.528  -6.802  0.47 14.42           H  0.040
ATOM    754  HB3BARG A  45      20.102 -18.220  -7.385  0.47 14.42           H  0.040
ATOM    755  HG2BARG A  45      19.423 -19.518  -5.675  0.47 15.20           H  0.041
ATOM    756  HG3BARG A  45      20.698 -18.737  -5.136  0.47 15.20           H  0.041
ATOM    757  HD2BARG A  45      18.753 -18.835  -3.690  0.47 11.58           H  0.036
ATOM    758  HD3BARG A  45      19.492 -17.434  -3.830  0.47 11.58           H  0.036
ATOM    759  HE BARG A  45      17.683 -16.617  -4.735  0.47 13.24           H  0.038
ATOM    760 HH11BARG A  45      17.253 -19.902  -4.781  0.47 14.31           H  0.040
ATOM    761 HH12BARG A  45      15.795 -19.856  -5.082  0.47 14.31           H  0.040
ATOM    762 HH21BARG A  45      15.470 -16.588  -5.110  0.47 13.50           H  0.039
ATOM    763 HH22BARG A  45      14.720 -17.864  -5.280  0.47 13.50           H  0.039
ATOM    764  N   ASN A  46      22.288 -16.152  -4.869  1.00 10.11           N  0.033
ANISOU  764  N   ASN A  46      998   2219    623    340   -101     88       N
ATOM    765  CA  ASN A  46      23.623 -16.339  -4.305  1.00 14.07           C  0.039
ANISOU  765  CA  ASN A  46     1259   2753   1334    414   -286    558       C
ATOM    766  C   ASN A  46      23.718 -17.658  -3.538  1.00 16.61           C  0.043
ANISOU  766  C   ASN A  46     1712   3030   1570    763    -91    806       C
ATOM    767  O   ASN A  46      22.715 -18.244  -3.127  1.00 18.03           O  0.045
ANISOU  767  O   ASN A  46     1993   2956   1902    748     77    683       O
ATOM    768  CB  ASN A  46      23.923 -15.179  -3.377  1.00 15.23           C  0.041
ANISOU  768  CB  ASN A  46     1394   2877   1514     -5   -631    495       C
ATOM    769  CG  ASN A  46      23.750 -13.848  -4.048  1.00 16.98           C  0.043
ANISOU  769  CG  ASN A  46     1750   3197   1505   -337   -280    553       C
ATOM    770  OD1 ASN A  46      23.018 -12.952  -3.593  1.00 19.49           O  0.046
ANISOU  770  OD1 ASN A  46     1940   3356   2111   -294   -486    787       O
ATOM    771  ND2 ASN A  46      24.396 -13.723  -5.196  1.00 17.71           N  0.044
ANISOU  771  ND2 ASN A  46     1858   3286   1585   -740     32    813       N
ATOM    772  HA  ASN A  46      24.284 -16.371  -5.013  1.00 16.88           H  0.043
ATOM    773  HB2 ASN A  46      23.318 -15.215  -2.619  1.00 18.27           H  0.045
ATOM    774  HB3 ASN A  46      24.841 -15.246  -3.072  1.00 18.27           H  0.045
ATOM    775 HD21 ASN A  46      24.346 -12.989  -5.640  1.00 21.25           H  0.048
ATOM    776 HD22 ASN A  46      24.867 -14.377  -5.497  1.00 21.25           H  0.048
ATOM    777  H  AASN A  46      21.757 -15.733  -4.338  0.53 12.13           H  0.037
ATOM    778  H  BASN A  46      21.768 -15.681  -4.372  0.47 12.13           H  0.037
ATOM    779  N   THR A  47      24.955 -18.176  -3.405  1.00 18.87           N  0.046
ANISOU  779  N   THR A  47     2109   3457   1605    900     14    594       N
ATOM    780  CA  THR A  47      25.097 -19.492  -2.781  1.00 24.04           C  0.051
ANISOU  780  CA  THR A  47     2463   3828   2845    811     69    313       C
ATOM    781  C   THR A  47      24.616 -19.481  -1.334  1.00 25.09           C  0.053
ANISOU  781  C   THR A  47     2807   3843   2885    598   -109    626       C
ATOM    782  O   THR A  47      24.193 -20.522  -0.815  1.00 27.31           O  0.055
ANISOU  782  O   THR A  47     2957   3800   3618    386   -223    847       O
ATOM    783  CB  THR A  47      26.542 -19.988  -2.864  1.00 27.27           C  0.055
ANISOU  783  CB  THR A  47     2433   4125   3805    948     29   -109       C
ATOM    784  OG1 THR A  47      27.431 -18.965  -2.405  1.00 28.39           O  0.056
ANISOU  784  OG1 THR A  47     2263   4278   4247    998     21   -243       O
ATOM    785  CG2 THR A  47      26.879 -20.357  -4.315  1.00 28.32           C  0.056
ANISOU  785  CG2 THR A  47     2469   4231   4061   1097    -25   -206       C
ATOM    786  H   THR A  47      25.686 -17.801  -3.657  1.00 22.65           H  0.050
```

```
ATOM    787  HA  THR A  47      24.551 -20.122  -3.277  1.00 28.85           H  0.056
ATOM    788  HB  THR A  47      26.656 -20.774  -2.308  1.00 32.73           H  0.060
ATOM    789  HG1 THR A  47      28.225 -19.236  -2.443  1.00 34.07           H  0.061
ATOM    790 HG21 THR A  47      27.795 -20.673  -4.372  1.00 33.99           H  0.061
ATOM    791 HG22 THR A  47      26.284 -21.057  -4.626  1.00 33.99           H  0.061
ATOM    792 HG23 THR A  47      26.778 -19.580  -4.887  1.00 33.99           H  0.061
ATOM    793  N   ASP A  48      24.661 -18.316  -0.680  1.00 22.70           N  0.050
ANISOU  793  N   ASP A  48     2836   3778   2013    736   -308    564       N
ATOM    794  CA  ASP A  48      24.203 -18.147   0.697  1.00 22.12           C  0.049
ANISOU  794  CA  ASP A  48     2733   3697   1974    655   -216    960       C
ATOM    795  C   ASP A  48      22.692 -18.075   0.808  1.00 21.14           C  0.048
ANISOU  795  C   ASP A  48     2675   3485   1871    648    -23    805       C
ATOM    796  O   ASP A  48      22.171 -17.900   1.921  1.00 22.29           O  0.050
ANISOU  796  O   ASP A  48     2847   3759   1865    774    175    879       O
ATOM    797  CB  ASP A  48      24.831 -16.901   1.343  1.00 22.48           C  0.050
ANISOU  797  CB  ASP A  48     2679   3781   2083    534   -332    742       C
ATOM    798  CG  ASP A  48      24.284 -15.569   0.776  1.00 24.46           C  0.052
ANISOU  798  CG  ASP A  48     2730   3961   2604    204   -353    682       C
ATOM    799  OD1 ASP A  48      23.408 -15.574  -0.116  1.00 21.37           O  0.048
ANISOU  799  OD1 ASP A  48     2277   3850   1994    249   -613   1124       O
ATOM    800  OD2 ASP A  48      24.735 -14.500   1.241  1.00 25.78           O  0.053
ANISOU  800  OD2 ASP A  48     2975   4087   2733    -21   -516    140       O
ATOM    801  H   ASP A  48      24.962 -17.589  -1.024  1.00 27.25           H  0.055
ATOM    802  HA  ASP A  48      24.508 -18.913   1.208  1.00 26.54           H  0.054
ATOM    803  HB2 ASP A  48      24.647 -16.916   2.295  1.00 26.98           H  0.054
ATOM    804  HB3 ASP A  48      25.788 -16.917   1.189  1.00 26.98           H  0.054
ATOM    805  N   GLY A  49      21.979 -18.235  -0.309  1.00 17.57           N  0.044
ANISOU  805  N   GLY A  49     2372   2874   1430    562     49    706       N
ATOM    806  CA  GLY A  49      20.544 -18.258  -0.298  1.00 16.20           C  0.042
ANISOU  806  CA  GLY A  49     2104   2355   1697    411   -188    583       C
ATOM    807  C   GLY A  49      19.880 -16.905  -0.423  1.00 13.57           C  0.039
ANISOU  807  C   GLY A  49     1779   1740   1638    262    -70    556       C
ATOM    808  O   GLY A  49      18.679 -16.868  -0.639  1.00 13.51           O  0.039
ANISOU  808  O   GLY A  49     1868   1520   1744   -233    104    354       O
ATOM    809  H   GLY A  49      22.323 -18.332  -1.091  1.00 21.09           H  0.048
ATOM    810  HA2 GLY A  49      20.237 -18.806  -1.038  1.00 19.44           H  0.046
ATOM    811  HA3 GLY A  49      20.246 -18.656   0.534  1.00 19.44           H  0.046
ATOM    812  N   SER A  50      20.614 -15.803  -0.282  1.00 12.06           N  0.036
ANISOU  812  N   SER A  50     1453   1655   1473    276   -116    639       N
ATOM    813  CA  SER A  50      20.059 -14.498  -0.643  1.00  8.60           C  0.031
ANISOU  813  CA  SER A  50     1240   1425    602     66   -130    289       C
ATOM    814  C   SER A  50      19.897 -14.399  -2.163  1.00  7.94           C  0.030
ANISOU  814  C   SER A  50     1281   1071    667    -40    -21    109       C
ATOM    815  O   SER A  50      20.464 -15.193  -2.919  1.00  9.23           O  0.032
ANISOU  815  O   SER A  50     1367    981   1160     27   -209    234       O
ATOM    816  CB  SER A  50      20.922 -13.349  -0.132  1.00 10.62           C  0.034
ANISOU  816  CB  SER A  50     1368   1824    844    -71    105    151       C
ATOM    817  OG  SER A  50      22.170 -13.249  -0.787  1.00 12.13           O  0.037
ANISOU  817  OG  SER A  50     1338   2010   1260   -124     38    341       O
ATOM    818  H   SER A  50      21.421 -15.782   0.015  1.00 14.47           H  0.040
ATOM    819  HA  SER A  50      19.189 -14.402  -0.224  1.00 10.32           H  0.034
ATOM    820  HB2 SER A  50      20.438 -12.519  -0.269  1.00 12.75           H  0.037
ATOM    821  HB3 SER A  50      21.085 -13.486   0.814  1.00 12.75           H  0.037
ATOM    822  HG  SER A  50      22.537 -14.004  -0.822  1.00 14.55           H  0.040
ATOM    823  N   THR A  51      19.107 -13.399  -2.601  1.00  6.94           N  0.028
ANISOU  823  N   THR A  51     1269    820    546    -89   -120    107       N
ATOM    824  CA  THR A  51      18.933 -13.140  -4.030  1.00  5.95           C  0.026
ANISOU  824  CA  THR A  51     1189    539    535   -305    -28    154       C
ATOM    825  C   THR A  51      19.152 -11.658  -4.310  1.00  5.47           C  0.025
ANISOU  825  C   THR A  51     1061    639    377   -331   -147    -11       C
ATOM    826  O   THR A  51      18.809 -10.790  -3.504  1.00  5.96           O  0.026
ANISOU  826  O   THR A  51     1142    671    452   -444     91   -124       O
ATOM    827  CB  THR A  51      17.547 -13.554  -4.449  1.00  7.58           C  0.029
ANISOU  827  CB  THR A  51     1281    578   1021   -273   -203   -239       C
ATOM    828  OG1 THR A  51      17.348 -14.948  -4.166  1.00  8.29           O  0.030
ANISOU  828  OG1 THR A  51     1306    782   1061   -484    -86     -1       O
ATOM    829  CG2 THR A  51      17.332 -13.342  -5.945  1.00  8.48           C  0.031
ANISOU  829  CG2 THR A  51     1497    559   1167   -338   -268    -42       C
ATOM    830  H   THR A  51      18.670 -12.867  -2.087  1.00  8.32           H  0.030
ATOM    831  HA  THR A  51      19.585 -13.631  -4.553  1.00  7.15           H  0.028
ATOM    832  HB  THR A  51      16.907 -13.014  -3.960  1.00  9.10           H  0.032
ATOM    833  HG1 THR A  51      16.795 -15.038  -3.540  1.00  9.95           H  0.033
ATOM    834 HG21 THR A  51      16.425 -13.584  -6.187  1.00 10.18           H  0.033
ATOM    835 HG22 THR A  51      17.481 -12.410  -6.172  1.00 10.18           H  0.033
ATOM    836 HG23 THR A  51      17.951 -13.892  -6.450  1.00 10.18           H  0.033
ATOM    837  N   ASP A  52      19.723 -11.389  -5.474  1.00  6.74           N  0.027
ANISOU  837  N   ASP A  52     1211    863    488   -298    -36   -165       N
ATOM    838  CA  ASP A  52      19.953 -10.038  -5.957  1.00  8.48           C  0.031
```

```
ANISOU  838  CA  ASP A  52     1090    958   1174   -337   -121    -86       C
ATOM    839  C   ASP A  52      18.867  -9.685  -6.959  1.00  6.99           C  0.028
ANISOU  839  C   ASP A  52      898    710   1047   -427   -124   -162       C
ATOM    840  O   ASP A  52      18.536 -10.509  -7.833  1.00  8.32           O  0.030
ANISOU  840  O   ASP A  52     1004    921   1234   -217   -284     78       O
ATOM    841  CB  ASP A  52      21.295  -9.916  -6.678  1.00  9.24           C  0.032
ANISOU  841  CB  ASP A  52     1036   1503    974   -199    112     65       C
ATOM    842  CG  ASP A  52      22.479 -10.154  -5.792  1.00 14.23           C  0.040
ANISOU  842  CG  ASP A  52     1401   2357   1648    -43    -56    258       C
ATOM    843  OD1 ASP A  52      22.378  -9.922  -4.588  1.00 13.11           O  0.038
ANISOU  843  OD1 ASP A  52     1500   2205   1277   -390   -339    302       O
ATOM    844  OD2 ASP A  52      23.530 -10.575  -6.347  1.00 18.40           O  0.045
ANISOU  844  OD2 ASP A  52     1403   3117   2472    230   -162    599       O
ATOM    845  H   ASP A  52      19.995 -11.994  -6.021  1.00  8.09           H  0.030
ATOM    846  HA  ASP A  52      19.940  -9.434  -5.198  1.00 10.18           H  0.033
ATOM    847  HB2 ASP A  52      21.326 -10.571  -7.394  1.00 11.09           H  0.035
ATOM    848  HB3 ASP A  52      21.374  -9.021  -7.043  1.00 11.09           H  0.035
ATOM    849  N   TYR A  53      18.311  -8.489  -6.835  1.00  6.33           N  0.026
ANISOU  849  N   TYR A  53      850    520   1036   -324   -209     20       N
ATOM    850  CA  TYR A  53      17.147  -8.131  -7.623  1.00  6.47           C  0.027
ANISOU  850  CA  TYR A  53     1035    506    919   -311    120    -13       C
ATOM    851  C   TYR A  53      17.293  -6.830  -8.388  1.00  6.60           C  0.027
ANISOU  851  C   TYR A  53      940    575    993   -392     73   -141       C
ATOM    852  O   TYR A  53      17.653  -5.792  -7.816  1.00  7.08           O  0.028
ANISOU  852  O   TYR A  53      808    694   1189   -325   -239   -172       O
ATOM    853  CB  TYR A  53      15.959  -7.948  -6.705  1.00  6.46           C  0.027
ANISOU  853  CB  TYR A  53      812    485   1156    -42    150     66       C
ATOM    854  CG  TYR A  53      15.478  -9.219  -6.010  1.00  6.81           C  0.027
ANISOU  854  CG  TYR A  53      977    731    878   -107    -29   -139       C
ATOM    855  CD1 TYR A  53      16.050  -9.658  -4.838  1.00  6.60           C  0.027
ANISOU  855  CD1 TYR A  53      885    596   1027   -138   -192   -258       C
ATOM    856  CD2 TYR A  53      14.418  -9.964  -6.538  1.00  7.13           C  0.028
ANISOU  856  CD2 TYR A  53     1071    941    699   -365   -266     83       C
ATOM    857  CE1 TYR A  53      15.606 -10.798  -4.223  1.00  6.74           C  0.027
ANISOU  857  CE1 TYR A  53      820    804    935   -408    307    -90       C
ATOM    858  CE2 TYR A  53      14.001 -11.100  -5.951  1.00  7.58           C  0.029
ANISOU  858  CE2 TYR A  53     1039   1003    840   -291   -185    -21       C
ATOM    859  CZ  TYR A  53      14.585 -11.513  -4.788  1.00  6.61           C  0.027
ANISOU  859  CZ  TYR A  53      875    920    717   -381     -2     49       C
ATOM    860  OH  TYR A  53      14.144 -12.659  -4.183  1.00  7.05           O  0.028
ANISOU  860  OH  TYR A  53      791    843   1046   -314   -137    142       O
ATOM    861  H   TYR A  53      18.591  -7.873  -6.304  1.00  7.60           H  0.029
ATOM    862  HA  TYR A  53      17.037  -8.838  -8.277  1.00  7.77           H  0.029
ATOM    863  HB2 TYR A  53      16.201  -7.312  -6.013  1.00  7.75           H  0.029
ATOM    864  HB3 TYR A  53      15.218  -7.604  -7.228  1.00  7.75           H  0.029
ATOM    865  HD1 TYR A  53      16.748  -9.174  -4.459  1.00  7.92           H  0.030
ATOM    866  HD2 TYR A  53      13.992  -9.667  -7.310  1.00  8.56           H  0.031
ATOM    867  HE1 TYR A  53      15.995 -11.084  -3.428  1.00  8.08           H  0.030
ATOM    868  HE2 TYR A  53      13.319 -11.602  -6.336  1.00  9.10           H  0.032
ATOM    869  HH  TYR A  53      14.798 -13.145  -3.978  1.00  8.46           H  0.030
ATOM    870  N   GLY A  54      16.955  -6.868  -9.665  1.00  6.96           N  0.028
ANISOU  870  N   GLY A  54     1043    701    903   -359   -149    111       N
ATOM    871  CA  GLY A  54      16.730  -5.661 -10.376  1.00  6.33           C  0.026
ANISOU  871  CA  GLY A  54      801    721    883   -274   -206    421       C
ATOM    872  C   GLY A  54      17.948  -5.097 -11.076  1.00  8.55           C  0.031
ANISOU  872  C   GLY A  54     1233    866   1150   -236   -331    482       C
ATOM    873  O   GLY A  54      18.983  -5.724 -11.206  1.00  8.31           O  0.030
ANISOU  873  O   GLY A  54     1446    924    787   -221   -299    126       O
ATOM    874  H   GLY A  54      16.855  -7.586 -10.128  1.00  8.36           H  0.030
ATOM    875  HA2 GLY A  54      16.049  -5.818 -11.049  1.00  7.60           H  0.029
ATOM    876  HA3 GLY A  54      16.410  -4.989  -9.754  1.00  7.60           H  0.029
ATOM    877  N   ILE A  55      17.768  -3.850 -11.563  1.00  9.70           N  0.033
ANISOU  877  N   ILE A  55     1281   1029   1377   -269   -270    560       N
ATOM    878  CA  ILE A  55      18.758  -3.173 -12.419  1.00 10.39           C  0.034
ANISOU  878  CA  ILE A  55     1437   1101   1411   -270   -396    611       C
ATOM    879  C   ILE A  55      20.089  -2.960 -11.718  1.00  9.10           C  0.032
ANISOU  879  C   ILE A  55     1184   1073   1202   -319    -76    615       C
ATOM    880  O   ILE A  55      21.144  -2.940 -12.360  1.00  7.77           O  0.029
ANISOU  880  O   ILE A  55     1120    977    855   -377     43    296       O
ATOM    881  CB  ILE A  55      18.125  -1.877 -12.980  1.00 15.16           C  0.041
ANISOU  881  CB  ILE A  55     2129   1310   2320   -225    -66    895       C
ATOM    882  CG1 ILE A  55      18.896  -1.422 -14.211  1.00 21.18           C  0.048
ANISOU  882  CG1 ILE A  55     2427   1746   3876    -67    -76   1218       C
ATOM    883  CG2 ILE A  55      17.873  -0.822 -11.872  1.00 16.04           C  0.042
ANISOU  883  CG2 ILE A  55     2266   1169   2658   -164   -310    837       C
ATOM    884  CD1 ILE A  55      18.032  -0.690 -15.196  1.00 23.59           C  0.051
ANISOU  884  CD1 ILE A  55     2587   1864   4512   -112   -314   1329       C
ATOM    885  H   ILE A  55      17.072  -3.370 -11.408  1.00 11.64           H  0.036
```

```
ATOM    886  HA  ILE A  55      18.977  -3.747 -13.170  1.00 12.47           H  0.037
ATOM    887  HB  ILE A  55      17.226  -2.050 -13.302  1.00 18.19           H  0.045
ATOM    888 HG12 ILE A  55      19.609  -0.825 -13.934  1.00 25.42           H  0.053
ATOM    889 HG13 ILE A  55      19.267  -2.199 -14.656  1.00 25.42           H  0.053
ATOM    890 HG21 ILE A  55      17.530  -0.011 -12.279  1.00 19.24           H  0.046
ATOM    891 HG22 ILE A  55      17.226  -1.175 -11.241  1.00 19.24           H  0.046
ATOM    892 HG23 ILE A  55      18.710  -0.635 -11.418  1.00 19.24           H  0.046
ATOM    893 HD11 ILE A  55      18.570  -0.439 -15.963  1.00 28.31           H  0.056
ATOM    894 HD12 ILE A  55      17.308  -1.272 -15.475  1.00 28.31           H  0.056
ATOM    895 HD13 ILE A  55      17.672   0.104 -14.771  1.00 28.31           H  0.056
ATOM    896  N   LEU A  56      20.076  -2.854 -10.395  1.00  8.36           N  0.030
ANISOU  896  N   LEU A  56     1074    993   1111   -295   -358    184       N
ATOM    897  CA  LEU A  56      21.292  -2.730  -9.613  1.00  8.66           C  0.031
ANISOU  897  CA  LEU A  56     1123    934   1233   -370   -110    -95       C
ATOM    898  C   LEU A  56      21.511  -3.910  -8.685  1.00  7.92           C  0.030
ANISOU  898  C   LEU A  56     1149    984    877   -438   -238     97       C
ATOM    899  O   LEU A  56      22.317  -3.801  -7.749  1.00  8.20           O  0.030
ANISOU  899  O   LEU A  56     1187   1055    872   -391   -225    142       O
ATOM    900  CB  LEU A  56      21.245  -1.400  -8.831  1.00  9.71           C  0.033
ANISOU  900  CB  LEU A  56     1318    973   1399   -345    100     24       C
ATOM    901  CG  LEU A  56      21.478  -0.216  -9.771  1.00  9.83           C  0.033
ANISOU  901  CG  LEU A  56     1437   1083   1215   -273    385    -64       C
ATOM    902  CD1 LEU A  56      21.114   1.099  -9.055  1.00 12.66           C  0.037
ANISOU  902  CD1 LEU A  56     1446   1124   2241   -499    535    -46       C
ATOM    903  CD2 LEU A  56      22.919  -0.139 -10.303  1.00 10.38           C  0.034
ANISOU  903  CD2 LEU A  56     1728   1219    996   -316    648    -45       C
ATOM    904  H   LEU A  56      19.359  -2.852  -9.920  1.00 10.04           H  0.033
ATOM    905  HA  LEU A  56      22.063  -2.704 -10.201  1.00 10.39           H  0.034
ATOM    906  HB2 LEU A  56      20.374  -1.300  -8.415  1.00 11.65           H  0.036
ATOM    907  HB3 LEU A  56      21.938  -1.399  -8.153  1.00 11.65           H  0.036
ATOM    908  HG  LEU A  56      20.907  -0.343 -10.545  1.00 11.80           H  0.036
ATOM    909 HD11 LEU A  56      21.304   1.844  -9.647  1.00 15.19           H  0.041
ATOM    910 HD12 LEU A  56      20.170   1.086  -8.832  1.00 15.19           H  0.041
ATOM    911 HD13 LEU A  56      21.644   1.176  -8.247  1.00 15.19           H  0.041
ATOM    912 HD21 LEU A  56      23.009   0.651 -10.858  1.00 12.45           H  0.037
ATOM    913 HD22 LEU A  56      23.530  -0.088  -9.552  1.00 12.45           H  0.037
ATOM    914 HD23 LEU A  56      23.105  -0.934 -10.827  1.00 12.45           H  0.037
ATOM    915  N   GLN A  57      20.791  -5.016  -8.889  1.00  7.69           N  0.029
ANISOU  915  N   GLN A  57     1052    807   1061   -458    -90    195       N
ATOM    916  CA  GLN A  57      21.087  -6.274  -8.204  1.00  6.81           C  0.027
ANISOU  916  CA  GLN A  57      990    914    685   -427     87    244       C
ATOM    917  C   GLN A  57      21.218  -6.074  -6.687  1.00  7.44           C  0.029
ANISOU  917  C   GLN A  57     1202    900    724   -329    140    175       C
ATOM    918  O   GLN A  57      22.200  -6.452  -6.071  1.00  8.56           O  0.031
ANISOU  918  O   GLN A  57     1387    948    918   -344   -164    263       O
ATOM    919  CB  GLN A  57      22.331  -6.906  -8.845  1.00  6.16           C  0.026
ANISOU  919  CB  GLN A  57      735    921    686   -437    109    105       C
ATOM    920  CG  GLN A  57      22.022  -7.378 -10.270  1.00  6.46           C  0.027
ANISOU  920  CG  GLN A  57      837    962    654   -466    158     66       C
ATOM    921  CD  GLN A  57      21.123  -8.579 -10.250  1.00  7.58           C  0.029
ANISOU  921  CD  GLN A  57     1008   1013    861   -416    323     54       C
ATOM    922  OE1 GLN A  57      21.582  -9.708 -10.084  1.00  8.04           O  0.030
ANISOU  922  OE1 GLN A  57     1061    864   1128   -277    -63    126       O
ATOM    923  NE2 GLN A  57      19.851  -8.339 -10.423  1.00  7.84           N  0.029
ANISOU  923  NE2 GLN A  57     1090   1049    838   -549    161   -140       N
ATOM    924  H   GLN A  57      20.120  -5.061  -9.425  1.00  9.22           H  0.032
ATOM    925  HA  GLN A  57      20.346  -6.892  -8.309  1.00  8.18           H  0.030
ATOM    926  HB2 GLN A  57      23.044  -6.249  -8.884  1.00  7.40           H  0.029
ATOM    927  HB3 GLN A  57      22.614  -7.670  -8.319  1.00  7.40           H  0.029
ATOM    928  HG2 GLN A  57      21.576  -6.668 -10.756  1.00  7.75           H  0.029
ATOM    929  HG3 GLN A  57      22.848  -7.620 -10.717  1.00  7.75           H  0.029
ATOM    930 HE21 GLN A  57      19.578  -7.532 -10.538  1.00  9.40           H  0.032
ATOM    931 HE22 GLN A  57      19.287  -8.988 -10.422  1.00  9.40           H  0.032
ATOM    932  N   ILE A  58      20.131  -5.569  -6.106  1.00  7.65           N  0.029
ANISOU  932  N   ILE A  58     1261   1065    582   -201     -9    -89       N
ATOM    933  CA  ILE A  58      20.083  -5.258  -4.667  1.00  7.50           C  0.029
ANISOU  933  CA  ILE A  58     1281    951    616   -268    373     87       C
ATOM    934  C   ILE A  58      19.691  -6.525  -3.927  1.00  8.68           C  0.031
ANISOU  934  C   ILE A  58     1397    980    921   -275     74    -18       C
ATOM    935  O   ILE A  58      18.782  -7.247  -4.357  1.00  8.83           O  0.031
ANISOU  935  O   ILE A  58     1488   1064    804   -154   -168    -77       O
ATOM    936  CB  ILE A  58      19.140  -4.080  -4.410  1.00  8.77           C  0.031
ANISOU  936  CB  ILE A  58     1306    922   1103   -197    346     19       C
ATOM    937  CG1 ILE A  58      19.866  -2.801  -4.872  1.00  9.87           C  0.033
ANISOU  937  CG1 ILE A  58     1397    962   1392   -406    -87    106       C
ATOM    938  CG2 ILE A  58      18.676  -4.007  -2.929  1.00  8.44           C  0.030
ANISOU  938  CG2 ILE A  58     1269    922   1015    160    444   -219       C
ATOM    939  CD1 ILE A  58      18.968  -1.622  -4.915  1.00 11.16           C  0.035
```

```
ANISOU  939  CD1 ILE A  58     1563   1014   1664   -262     54    235       C
ATOM    940  H   ILE A  58      19.400  -5.393  -6.523  1.00  9.19           H  0.032
ATOM    941  HA  ILE A  58      20.956  -4.996  -4.335  1.00  9.00           H  0.031
ATOM    942  HB  ILE A  58      18.321  -4.193  -4.916  1.00 10.52           H  0.034
ATOM    943 HG12 ILE A  58      20.589  -2.607  -4.254  1.00 11.85           H  0.036
ATOM    944 HG13 ILE A  58      20.221  -2.943  -5.763  1.00 11.85           H  0.036
ATOM    945 HG21 ILE A  58      18.173  -3.188  -2.795  1.00 10.13           H  0.033
ATOM    946 HG22 ILE A  58      18.115  -4.775  -2.736  1.00 10.13           H  0.033
ATOM    947 HG23 ILE A  58      19.456  -4.014  -2.352  1.00 10.13           H  0.033
ATOM    948 HD11 ILE A  58      19.383  -0.928  -5.452  1.00 13.39           H  0.038
ATOM    949 HD12 ILE A  58      18.122  -1.884  -5.311  1.00 13.39           H  0.038
ATOM    950 HD13 ILE A  58      18.825  -1.300  -4.012  1.00 13.39           H  0.038
ATOM    951  N   ASN A  59      20.411  -6.819  -2.834  1.00  8.97           N  0.031
ANISOU  951  N   ASN A  59     1630   1026    753   -472   -172    180       N
ATOM    952  CA  ASN A  59      20.439  -8.149  -2.217  1.00 10.94           C  0.035
ANISOU  952  CA  ASN A  59     1937   1466    755   -567     54    201       C
ATOM    953  C   ASN A  59      19.466  -8.229  -1.046  1.00 11.72           C  0.036
ANISOU  953  C   ASN A  59     2333   1154    964   -436    308   -230       C
ATOM    954  O   ASN A  59      19.307  -7.280  -0.278  1.00 13.24           O  0.038
ANISOU  954  O   ASN A  59     2855   1271    903   -622    462   -177       O
ATOM    955  CB  ASN A  59      21.871  -8.448  -1.774  1.00 13.29           C  0.038
ANISOU  955  CB  ASN A  59     1988   1983   1077   -712   -517     41       C
ATOM    956  CG  ASN A  59      22.032  -9.801  -1.153  1.00 19.08           C  0.046
ANISOU  956  CG  ASN A  59     2430   2856   1965   -475   -270    396       C
ATOM    957  OD1 ASN A  59      21.798  -9.969   0.052  1.00 21.48           O  0.049
ANISOU  957  OD1 ASN A  59     2707   3252   2201   -507   -526    542       O
ATOM    958  ND2 ASN A  59      22.445 -10.775  -1.947  1.00 22.53           N  0.050
ANISOU  958  ND2 ASN A  59     2618   3030   2912   -419   -452    271       N
ATOM    959  H   ASN A  59      20.904  -6.247  -2.422  1.00 10.77           H  0.034
ATOM    960  HA  ASN A  59      20.151  -8.828  -2.847  1.00 13.13           H  0.038
ATOM    961  HB2 ASN A  59      22.454  -8.405  -2.548  1.00 15.94           H  0.042
ATOM    962  HB3 ASN A  59      22.141  -7.786  -1.118  1.00 15.94           H  0.042
ATOM    963 HD21 ASN A  59      22.551 -11.569  -1.633  1.00 27.03           H  0.055
ATOM    964 HD22 ASN A  59      22.607 -10.614  -2.776  1.00 27.03           H  0.055
ATOM    965  N   SER A  60      18.828  -9.385  -0.924  1.00 10.33           N  0.034
ANISOU  965  N   SER A  60     2092   1022    812   -314    380     25       N
ATOM    966  CA  SER A  60      17.784  -9.645   0.041  1.00  8.33           C  0.030
ANISOU  966  CA  SER A  60     1806    905    455     -2     63    -10       C
ATOM    967  C   SER A  60      18.343  -9.931   1.439  1.00 12.86           C  0.038
ANISOU  967  C   SER A  60     2578   1381    927    167     44     18       C
ATOM    968  O   SER A  60      17.553 -10.084   2.353  1.00 15.43           O  0.041
ANISOU  968  O   SER A  60     2802   1634   1428    664    508     99       O
ATOM    969  CB  SER A  60      16.960 -10.860  -0.397  1.00  8.99           C  0.031
ANISOU  969  CB  SER A  60     1769    797    849   -148    199    -88       C
ATOM    970  OG  SER A  60      17.738 -12.044  -0.381  1.00  7.98           O  0.030
ANISOU  970  OG  SER A  60     1383    821    827    -53    183     21       O
ATOM    971  H   SER A  60      18.993 -10.069  -1.418  1.00 12.40           H  0.037
ATOM    972  HA  SER A  60      17.204  -8.869   0.082  1.00 10.00           H  0.033
ATOM    973  HB2 SER A  60      16.212 -10.967   0.211  1.00 10.78           H  0.034
ATOM    974  HB3 SER A  60      16.635 -10.710  -1.299  1.00 10.78           H  0.034
ATOM    975  HG  SER A  60      17.265 -12.702  -0.603  1.00  9.57           H  0.032
ATOM    976  N   ARG A  61      19.655 -10.022   1.633  1.00 13.39           N  0.038
ANISOU  976  N   ARG A  61     2712   1615    760   -105   -359     -0       N
ATOM    977  CA  ARG A  61      20.159 -10.255   2.994  1.00 16.72           C  0.043
ANISOU  977  CA  ARG A  61     3186   1956   1210   -440   -634   -151       C
ATOM    978  C   ARG A  61      20.057  -8.985   3.816  1.00 13.67           C  0.039
ANISOU  978  C   ARG A  61     2754   1617    824   -686   -222    -49       C
ATOM    979  O   ARG A  61      19.862  -9.044   5.032  1.00 15.11           O  0.041
ANISOU  979  O   ARG A  61     3192   1473   1076  -1052   -183     -4       O
ATOM    980  CB  ARG A  61      21.600 -10.755   2.954  1.00 21.29           C  0.048
ANISOU  980  CB  ARG A  61     3720   2542   1829   -282  -1001   -125       C
ATOM    981  CG  ARG A  61      22.441 -10.556   4.242  1.00 27.76           C  0.055
ANISOU  981  CG  ARG A  61     4284   3219   3045   -204  -1145   -308       C
ATOM    982  CD  ARG A  61      22.165 -11.637   5.308  1.00 32.78           C  0.060
ANISOU  982  CD  ARG A  61     4726   3747   3981   -231  -1303   -409       C
ATOM    983  NE  ARG A  61      22.939 -11.418   6.533  1.00 35.04           N  0.062
ANISOU  983  NE  ARG A  61     5093   4208   4013   -175  -1643   -294       N
ATOM    984  CZ  ARG A  61      22.616 -10.562   7.496  1.00 35.22           C  0.062
ANISOU  984  CZ  ARG A  61     5194   4409   3779   -154  -1867   -244       C
ATOM    985  NH1 ARG A  61      21.520  -9.823   7.436  1.00 31.96           N  0.059
ANISOU  985  NH1 ARG A  61     5016   4459   2668   -178  -1977    -29       N
ATOM    986  NH2 ARG A  61      23.416 -10.443   8.554  1.00 35.76           N  0.063
ANISOU  986  NH2 ARG A  61     5256   4505   3828    -90  -2100    -82       N
ATOM    987  H   ARG A  61      20.256  -9.955   1.021  1.00 16.07           H  0.042
ATOM    988  HA  ARG A  61      19.636 -10.951   3.422  1.00 20.06           H  0.047
ATOM    989  HB2 ARG A  61      21.583 -11.708   2.771  1.00 25.55           H  0.053
ATOM    990  HB3 ARG A  61      22.061 -10.288   2.240  1.00 25.55           H  0.053
ATOM    991  HG2 ARG A  61      23.383 -10.594   4.014  1.00 33.32           H  0.061
```

```
ATOM    992  HG3 ARG A  61      22.228  -9.693   4.629  1.00 33.32           H  0.061
ATOM    993  HD2 ARG A  61      21.223 -11.623   5.539  1.00 39.33           H  0.066
ATOM    994  HD3 ARG A  61      22.406 -12.506   4.950  1.00 39.33           H  0.066
ATOM    995  HE  ARG A  61      23.657 -11.879   6.636  1.00 42.05           H  0.068
ATOM    996 HH11 ARG A  61      20.994  -9.889   6.759  1.00 38.35           H  0.065
ATOM    997 HH12 ARG A  61      21.334  -9.276   8.073  1.00 38.35           H  0.065
ATOM    998 HH21 ARG A  61      24.132 -10.917   8.607  1.00 42.91           H  0.069
ATOM    999 HH22 ARG A  61      23.217  -9.892   9.184  1.00 42.91           H  0.069
ATOM   1000  N   TRP A  62      20.184  -7.843   3.179  1.00 12.59           N  0.037
ANISOU 1000  N   TRP A  62     2131   1520   1133   -592   -240   -151       N
ATOM   1001  CA  TRP A  62      20.171  -6.607   3.905  1.00 12.54           C  0.037
ANISOU 1001  CA  TRP A  62     1735   1588   1440   -572   -186   -206       C
ATOM   1002  C   TRP A  62      19.070  -5.664   3.503  1.00 10.60           C  0.034
ANISOU 1002  C   TRP A  62     1496   1404   1128   -570   -140   -294       C
ATOM   1003  O   TRP A  62      18.574  -4.937   4.357  1.00 10.31           O  0.034
ANISOU 1003  O   TRP A  62     1498   1477    940   -580    336   -279       O
ATOM   1004  CB  TRP A  62      21.517  -5.877   3.729  1.00 15.24           C  0.041
ANISOU 1004  CB  TRP A  62     1857   1854   2078   -313   -159   -196       C
ATOM   1005  CG  TRP A  62      22.707  -6.579   4.369  1.00 17.33           C  0.044
ANISOU 1005  CG  TRP A  62     2154   2317   2114    -49    -20     15       C
ATOM   1006  CD1 TRP A  62      23.735  -7.200   3.732  1.00 20.40           C  0.047
ANISOU 1006  CD1 TRP A  62     2483   2645   2625    120   -358     66       C
ATOM   1007  CD2 TRP A  62      22.946  -6.707   5.764  1.00 20.69           C  0.048
ANISOU 1007  CD2 TRP A  62     2453   2633   2775     18   -253    132       C
ATOM   1008  NE1 TRP A  62      24.621  -7.688   4.653  1.00 20.78           N  0.048
ANISOU 1008  NE1 TRP A  62     2511   2805   2581    468   -347    229       N
ATOM   1009  CE2 TRP A  62      24.153  -7.408   5.911  1.00 22.04           C  0.049
ANISOU 1009  CE2 TRP A  62     2662   2933   2780    234   -463    329       C
ATOM   1010  CE3 TRP A  62      22.258  -6.284   6.910  1.00 22.01           C  0.049
ANISOU 1010  CE3 TRP A  62     2637   2770   2956   -187   -304    273       C
ATOM   1011  CZ2 TRP A  62      24.690  -7.711   7.156  1.00 24.05           C  0.051
ANISOU 1011  CZ2 TRP A  62     2889   3059   3192     99   -466    164       C
ATOM   1012  CZ3 TRP A  62      22.796  -6.598   8.152  1.00 22.80           C  0.050
ANISOU 1012  CZ3 TRP A  62     2753   2901   3009   -208   -244    290       C
ATOM   1013  CH2 TRP A  62      23.991  -7.297   8.261  1.00 23.85           C  0.051
ANISOU 1013  CH2 TRP A  62     2885   2977   3198    -71   -403    127       C
ATOM   1014  H   TRP A  62      20.279  -7.763   2.328  1.00 15.11           H  0.041
ATOM   1015  HA  TRP A  62      20.049  -6.811   4.846  1.00 15.04           H  0.041
ATOM   1016  HB2 TRP A  62      21.702  -5.794   2.780  1.00 18.28           H  0.045
ATOM   1017  HB3 TRP A  62      21.446  -4.997   4.131  1.00 18.28           H  0.045
ATOM   1018  HD1 TRP A  62      23.822  -7.281   2.810  1.00 24.49           H  0.052
ATOM   1019  HE1 TRP A  62      25.353  -8.102   4.473  1.00 24.94           H  0.052
ATOM   1020  HE3 TRP A  62      21.463  -5.807   6.840  1.00 26.41           H  0.054
ATOM   1021  HZ2 TRP A  62      25.491  -8.177   7.237  1.00 28.86           H  0.056
ATOM   1022  HZ3 TRP A  62      22.348  -6.335   8.924  1.00 27.36           H  0.055
ATOM   1023  HH2 TRP A  62      24.327  -7.489   9.107  1.00 28.61           H  0.056
ATOM   1024  N   TRP A  63      18.663  -5.642   2.229  1.00  9.30           N  0.032
ANISOU 1024  N   TRP A  63     1284   1232   1017   -471     -8   -284       N
ATOM   1025  CA  TRP A  63      18.032  -4.443   1.704  1.00  8.06           C  0.030
ANISOU 1025  CA  TRP A  63     1149   1098    817   -519     74   -146       C
ATOM   1026  C   TRP A  63      16.554  -4.572   1.410  1.00  8.77           C  0.031
ANISOU 1026  C   TRP A  63     1084   1041   1205   -445     81   -327       C
ATOM   1027  O   TRP A  63      15.823  -3.592   1.499  1.00  9.37           O  0.032
ANISOU 1027  O   TRP A  63     1226   1042   1290   -291    -41   -371       O
ATOM   1028  CB  TRP A  63      18.764  -3.934   0.449  1.00  9.18           C  0.032
ANISOU 1028  CB  TRP A  63     1041   1084   1363   -576    -45   -273       C
ATOM   1029  CG  TRP A  63      20.179  -3.665   0.724  1.00  8.39           C  0.030
ANISOU 1029  CG  TRP A  63      915   1179   1094   -368   -196    -96       C
ATOM   1030  CD1 TRP A  63      21.235  -4.373   0.282  1.00  9.49           C  0.032
ANISOU 1030  CD1 TRP A  63     1135   1304   1166   -323    124   -139       C
ATOM   1031  CD2 TRP A  63      20.695  -2.631   1.578  1.00  7.83           C  0.029
ANISOU 1031  CD2 TRP A  63      989   1058    927   -271   -163   -183       C
ATOM   1032  NE1 TRP A  63      22.401  -3.836   0.793  1.00  9.79           N  0.033
ANISOU 1032  NE1 TRP A  63     1016   1253   1452   -250    175   -131       N
ATOM   1033  CE2 TRP A  63      22.095  -2.761   1.579  1.00  9.10           C  0.032
ANISOU 1033  CE2 TRP A  63      932   1111   1413   -286    -47   -267       C
ATOM   1034  CE3 TRP A  63      20.109  -1.579   2.278  1.00  9.55           C  0.032
ANISOU 1034  CE3 TRP A  63     1096   1162   1371   -499    -96   -298       C
ATOM   1035  CZ2 TRP A  63      22.926  -1.890   2.305  1.00 10.70           C  0.034
ANISOU 1035  CZ2 TRP A  63     1179   1103   1782   -234   -142   -190       C
ATOM   1036  CZ3 TRP A  63      20.926  -0.724   2.984  1.00 10.89           C  0.035
ANISOU 1036  CZ3 TRP A  63     1343   1333   1461   -396   -595   -311       C
ATOM   1037  CH2 TRP A  63      22.307  -0.888   3.009  1.00 12.18           C  0.037
ANISOU 1037  CH2 TRP A  63     1356   1347   1925   -315   -467   -614       C
ATOM   1038  H   TRP A  63      18.742  -6.292   1.673  1.00 11.16           H  0.035
ATOM   1039  HA  TRP A  63      18.116  -3.754   2.381  1.00  9.68           H  0.033
ATOM   1040  HB2 TRP A  63      18.711  -4.607  -0.248  1.00 11.01           H  0.035
ATOM   1041  HB3 TRP A  63      18.350  -3.111   0.148  1.00 11.01           H  0.035
```

```
ATOM   1042  HD1 TRP A  63      21.187  -5.111  -0.282  1.00 11.38           H  0.035
ATOM   1043  HE1 TRP A  63      23.194  -4.132   0.641  1.00 11.75           H  0.036
ATOM   1044  HE3 TRP A  63      19.187  -1.458   2.269  1.00 11.46           H  0.035
ATOM   1045  HZ2 TRP A  63      23.851  -1.989   2.309  1.00 12.83           H  0.038
ATOM   1046  HZ3 TRP A  63      20.544  -0.020   3.456  1.00 13.07           H  0.038
ATOM   1047  HH2 TRP A  63      22.824  -0.303   3.515  1.00 14.62           H  0.040
ATOM   1048  N   CYS A  64      16.083  -5.760   1.068  1.00 10.54           N  0.034
ANISOU 1048  N   CYS A  64     1163   1010   1833   -521     72   -370       N
ATOM   1049  CA  CYS A  64      14.684  -5.921   0.693  1.00  8.82           C  0.031
ANISOU 1049  CA  CYS A  64      928   1109   1315   -424     93   -535       C
ATOM   1050  C   CYS A  64      14.206  -7.253   1.258  1.00  7.80           C  0.029
ANISOU 1050  C   CYS A  64      807   1027   1131   -386     27   -447       C
ATOM   1051  O   CYS A  64      14.997  -8.119   1.606  1.00  9.79           O  0.033
ANISOU 1051  O   CYS A  64      909   1218   1594   -343     56   -466       O
ATOM   1052  CB  CYS A  64      14.493  -5.852  -0.819  1.00  9.05           C  0.032
ANISOU 1052  CB  CYS A  64      772   1253   1414   -410    101   -403       C
ATOM   1053  SG  CYS A  64      15.323  -7.149  -1.773  1.00  9.74           S  0.033
ANISOU 1053  SG  CYS A  64      917   1134   1652   -401    363   -288       S
ATOM   1054  H   CYS A  64      16.548  -6.483   1.045  1.00 12.65           H  0.037
ATOM   1055  HA  CYS A  64      14.141  -5.213   1.075  1.00 10.59           H  0.034
ATOM   1056  HB2 CYS A  64      13.544  -5.917  -1.009  1.00 10.86           H  0.035
ATOM   1057  HB3 CYS A  64      14.836  -5.000  -1.131  1.00 10.86           H  0.035
ATOM   1058  N   ASN A  65      12.882  -7.401   1.366  1.00  8.35           N  0.030
ANISOU 1058  N   ASN A  65      929   1023   1222   -510    139   -357       N
ATOM   1059  CA  ASN A  65      12.315  -8.649   1.842  1.00  8.93           C  0.031
ANISOU 1059  CA  ASN A  65     1000   1185   1209   -483    416   -351       C
ATOM   1060  C   ASN A  65      11.767  -9.518   0.711  1.00  9.81           C  0.033
ANISOU 1060  C   ASN A  65      936   1108   1681   -521    207    -61       C
ATOM   1061  O   ASN A  65      10.936  -9.077  -0.077  1.00 10.12           O  0.033
ANISOU 1061  O   ASN A  65      907   1378   1559   -234     14   -398       O
ATOM   1062  CB  ASN A  65      11.184  -8.407   2.826  1.00 11.22           C  0.035
ANISOU 1062  CB  ASN A  65     1224   1563   1478   -708    328   -389       C
ATOM   1063  CG  ASN A  65      10.550  -9.701   3.235  1.00 12.72           C  0.037
ANISOU 1063  CG  ASN A  65     1449   1892   1491   -897     59   -428       C
ATOM   1064  OD1 ASN A  65      11.199 -10.547   3.796  1.00 13.83           O  0.039
ANISOU 1064  OD1 ASN A  65     1581   1966   1708   -758     -4   -187       O
ATOM   1065  ND2 ASN A  65       9.291  -9.876   2.905  1.00 16.62           N  0.043
ANISOU 1065  ND2 ASN A  65     1885   2183   2245   -930    296   -795       N
ATOM   1066  H   ASN A  65      12.304  -6.795   1.169  1.00 10.02           H  0.033
ATOM   1067  HA  ASN A  65      13.043  -9.128   2.267  1.00 10.72           H  0.034
ATOM   1068  HB2 ASN A  65      11.533  -7.970   3.618  1.00 13.47           H  0.038
ATOM   1069  HB3 ASN A  65      10.507  -7.850   2.410  1.00 13.47           H  0.038
ATOM   1070 HD21 ASN A  65       8.888 -10.604   3.120  1.00 19.94           H  0.047
ATOM   1071 HD22 ASN A  65       8.871  -9.263   2.473  1.00 19.94           H  0.047
ATOM   1072  N   ASP A  66      12.236 -10.753   0.645  1.00  8.74           N  0.031
ANISOU 1072  N   ASP A  66      942    854   1524   -482    151   -112       N
ATOM   1073  CA  ASP A  66      11.677 -11.724  -0.276  1.00  8.96           C  0.031
ANISOU 1073  CA  ASP A  66     1072    975   1356   -564    185   -259       C
ATOM   1074  C   ASP A  66      11.053 -12.910   0.439  1.00  9.50           C  0.032
ANISOU 1074  C   ASP A  66     1231   1148   1229   -660     30   -205       C
ATOM   1075  O   ASP A  66      10.586 -13.831  -0.252  1.00  9.75           O  0.033
ANISOU 1075  O   ASP A  66     1097   1254   1352   -600    155   -462       O
ATOM   1076  CB  ASP A  66      12.708 -12.148  -1.348  1.00  8.24           C  0.030
ANISOU 1076  CB  ASP A  66     1054    840   1236   -437      2   -317       C
ATOM   1077  CG  ASP A  66      13.891 -12.895  -0.796  1.00  8.97           C  0.031
ANISOU 1077  CG  ASP A  66     1284    981   1144   -403     66   -168       C
ATOM   1078  OD1 ASP A  66      13.933 -13.253   0.424  1.00  9.65           O  0.033
ANISOU 1078  OD1 ASP A  66     1454   1142   1069   -317    358     44       O
ATOM   1079  OD2 ASP A  66      14.836 -13.155  -1.603  1.00  8.42           O  0.030
ANISOU 1079  OD2 ASP A  66     1382    998    820   -396     83    -99       O
ATOM   1080  H   ASP A  66      12.881 -11.053   1.128  1.00 10.48           H  0.034
ATOM   1081  HA  ASP A  66      10.965 -11.309  -0.787  1.00 10.75           H  0.034
ATOM   1082  HB2 ASP A  66      12.268 -12.726  -1.992  1.00  9.88           H  0.033
ATOM   1083  HB3 ASP A  66      13.042 -11.353  -1.791  1.00  9.88           H  0.033
ATOM   1084  N   GLY A  67      11.039 -12.918   1.784  1.00 11.31           N  0.035
ANISOU 1084  N   GLY A  67     1406   1180   1711   -590    103   -148       N
ATOM   1085  CA  GLY A  67      10.436 -13.987   2.556  1.00 11.71           C  0.036
ANISOU 1085  CA  GLY A  67     1516   1182   1752   -391     66   -304       C
ATOM   1086  C   GLY A  67      11.181 -15.295   2.533  1.00 11.21           C  0.035
ANISOU 1086  C   GLY A  67     1752   1153   1355   -565    194    -81       C
ATOM   1087  O   GLY A  67      10.695 -16.270   3.128  1.00 14.15           O  0.039
ANISOU 1087  O   GLY A  67     2046   1283   2048   -822    367    -41       O
ATOM   1088  H   GLY A  67      11.382 -12.297   2.270  1.00 13.57           H  0.039
ATOM   1089  HA2 GLY A  67      10.372 -13.702   3.481  1.00 14.05           H  0.039
ATOM   1090  HA3 GLY A  67       9.543 -14.151   2.214  1.00 14.05           H  0.039
ATOM   1091  N  AARG A  68      12.347 -15.379   1.892  0.47  9.48           N  0.032
ANISOU 1091  N  AARG A  68     1641   1028    933   -476    108    -36       N
ATOM   1092  CA AARG A  68      13.054 -16.655   1.862  0.47  9.93           C  0.033
```

```
ANISOU 1092  CA AARG A  68     1720   1101    955   -517    112     86       C
ATOM   1093  C  AARG A  68      14.519 -16.543   2.250  0.47 11.69           C  0.036
ANISOU 1093  C  AARG A  68     1809   1218   1413   -402    168    149       C
ATOM   1094  O  AARG A  68      15.287 -17.479   2.006  0.47 14.49           O  0.040
ANISOU 1094  O  AARG A  68     1897   1374   2234   -287    -52    214       O
ATOM   1095  CB AARG A  68      12.914 -17.326   0.492  0.47  9.24           C  0.032
ANISOU 1095  CB AARG A  68     1664   1091    756   -636     91    175       C
ATOM   1096  CG AARG A  68      13.513 -16.559  -0.668  0.47  9.09           C  0.032
ANISOU 1096  CG AARG A  68     1650   1018    785   -554     78    117       C
ATOM   1097  CD AARG A  68      13.485 -17.417  -1.892  0.47  9.04           C  0.032
ANISOU 1097  CD AARG A  68     1550   1045    840   -391   -179     -7       C
ATOM   1098  NE AARG A  68      14.424 -16.929  -2.880  0.47  7.13           N  0.028
ANISOU 1098  NE AARG A  68     1329    927    453   -273   -216    -60       N
ATOM   1099  CZ AARG A  68      14.440 -17.342  -4.136  0.47  7.62           C  0.029
ANISOU 1099  CZ AARG A  68     1304   1011    580   -227   -308   -179       C
ATOM   1100  NH1AARG A  68      13.558 -18.228  -4.577  0.47  6.74           N  0.027
ANISOU 1100  NH1AARG A  68     1124    949    488   -124   -288   -170       N
ATOM   1101  NH2AARG A  68      15.354 -16.855  -4.964  0.47  7.37           N  0.028
ANISOU 1101  NH2AARG A  68     1340    991    470   -183   -297     83       N
ATOM   1102  H  AARG A  68      12.736 -14.731   1.482  0.47 11.38           H  0.035
ATOM   1103  HA AARG A  68      12.638 -17.240   2.514  0.47 11.92           H  0.036
ATOM   1104  HB2AARG A  68      13.355 -18.189   0.527  0.47 11.09           H  0.035
ATOM   1105  HB3AARG A  68      11.969 -17.444   0.304  0.47 11.09           H  0.035
ATOM   1106  HG2AARG A  68      12.996 -15.756  -0.836  0.47 10.90           H  0.035
ATOM   1107  HG3AARG A  68      14.433 -16.326  -0.469  0.47 10.90           H  0.035
ATOM   1108  HD2AARG A  68      13.729 -18.326  -1.657  0.47 10.85           H  0.035
ATOM   1109  HD3AARG A  68      12.595 -17.402  -2.278  0.47 10.85           H  0.035
ATOM   1110  HE AARG A  68      15.001 -16.340  -2.637  0.47  8.56           H  0.031
ATOM   1111 HH11AARG A  68      12.963 -18.544  -4.043  0.47  8.09           H  0.030
ATOM   1112 HH12AARG A  68      13.582 -18.487  -5.397  0.47  8.09           H  0.030
ATOM   1113 HH21AARG A  68      15.926 -16.279  -4.682  0.47  8.85           H  0.031
ATOM   1114 HH22AARG A  68      15.374 -17.116  -5.783  0.47  8.85           H  0.031
ATOM   1115  N  BARG A  68      12.336 -15.364   1.851  0.53  9.49           N  0.032
ANISOU 1115  N  BARG A  68     1633   1092    882   -462    120    -63       N
ATOM   1116  CA BARG A  68      13.079 -16.616   1.733  0.53 10.35           C  0.034
ANISOU 1116  CA BARG A  68     1732   1283    916   -502    109    -20       C
ATOM   1117  C  BARG A  68      14.563 -16.450   2.056  0.53 11.48           C  0.036
ANISOU 1117  C  BARG A  68     1798   1277   1288   -462     37    -62       C
ATOM   1118  O  BARG A  68      15.388 -17.245   1.588  0.53 12.26           O  0.037
ANISOU 1118  O  BARG A  68     1837   1357   1463   -465   -282   -232       O
ATOM   1119  CB BARG A  68      12.884 -17.255   0.346  0.53 10.85           C  0.035
ANISOU 1119  CB BARG A  68     1746   1480    897   -565      3    163       C
ATOM   1120  CG BARG A  68      13.148 -16.345  -0.894  0.53 11.28           C  0.035
ANISOU 1120  CG BARG A  68     1764   1593    929   -482    -29    204       C
ATOM   1121  CD BARG A  68      13.548 -17.165  -2.152  0.53 12.66           C  0.037
ANISOU 1121  CD BARG A  68     1679   1684   1447   -356     59     92       C
ATOM   1122  NE BARG A  68      14.926 -17.620  -2.041  0.53 14.83           N  0.040
ANISOU 1122  NE BARG A  68     1663   1811   2162   -360    120    -70       N
ATOM   1123  CZ BARG A  68      15.316 -18.884  -1.909  0.53 14.91           C  0.040
ANISOU 1123  CZ BARG A  68     1602   1848   2215   -242    332   -169       C
ATOM   1124  NH1BARG A  68      14.479 -19.894  -2.073  0.53 13.45           N  0.038
ANISOU 1124  NH1BARG A  68     1537   1812   1761   -303    589   -371       N
ATOM   1125  NH2BARG A  68      16.575 -19.136  -1.570  0.53 15.56           N  0.041
ANISOU 1125  NH2BARG A  68     1584   1868   2460   -174    279   -183       N
ATOM   1126  H  BARG A  68      12.704 -14.697   1.451  0.53 11.39           H  0.035
ATOM   1127  HA BARG A  68      12.721 -17.237   2.386  0.53 12.42           H  0.037
ATOM   1128  HB2BARG A  68      13.489 -18.010   0.276  0.53 13.02           H  0.038
ATOM   1129  HB3BARG A  68      11.965 -17.559   0.283  0.53 13.02           H  0.038
ATOM   1130  HG2BARG A  68      12.341 -15.848  -1.102  0.53 13.54           H  0.039
ATOM   1131  HG3BARG A  68      13.871 -15.732  -0.691  0.53 13.54           H  0.039
ATOM   1132  HD2BARG A  68      12.971 -17.940  -2.232  0.53 15.19           H  0.041
ATOM   1133  HD3BARG A  68      13.468 -16.608  -2.942  0.53 15.19           H  0.041
ATOM   1134  HE BARG A  68      15.541 -17.019  -2.062  0.53 17.80           H  0.044
ATOM   1135 HH11BARG A  68      13.656 -19.742  -2.271  0.53 16.14           H  0.042
ATOM   1136 HH12BARG A  68      14.758 -20.702  -1.982  0.53 16.14           H  0.042
ATOM   1137 HH21BARG A  68      17.124 -18.487  -1.439  0.53 18.67           H  0.045
ATOM   1138 HH22BARG A  68      16.842 -19.949  -1.481  0.53 18.67           H  0.045
ATOM   1139  N   THR A  69      14.936 -15.412   2.813  1.00  9.82           N  0.033
ANISOU 1139  N   THR A  69     1835   1127    769   -453    197    -87       N
ATOM   1140  CA  THR A  69      16.325 -15.185   3.197  1.00  9.89           C  0.033
ANISOU 1140  CA  THR A  69     1813   1193    751   -464    -23      5       C
ATOM   1141  C   THR A  69      16.411 -15.118   4.711  1.00 12.00           C  0.036
ANISOU 1141  C   THR A  69     2089   1377   1094   -452    267    -32       C
ATOM   1142  O   THR A  69      16.391 -14.040   5.301  1.00 12.82           O  0.038
ANISOU 1142  O   THR A  69     2503   1217   1152   -555    217   -228       O
ATOM   1143  CB  THR A  69      16.875 -13.917   2.539  1.00  9.11           C  0.032
ANISOU 1143  CB  THR A  69     1725   1174    562   -484     39     46       C
ATOM   1144  OG1 THR A  69      16.575 -13.971   1.125  1.00  8.45           O  0.030
```

```
ANISOU 1144  OG1 THR A  69     1552    861    797   -463     -4    106       O
ATOM   1145  CG2 THR A  69      18.395 -13.810   2.697  1.00 10.17           C  0.033
ANISOU 1145  CG2 THR A  69     1819   1444    602   -477    -70   -117       C
ATOM   1146  HA  THR A  69      16.867 -15.934   2.902  1.00 11.87           H  0.036
ATOM   1147  HB  THR A  69      16.473 -13.141   2.959  1.00 10.93           H  0.035
ATOM   1148  HG1 THR A  69      15.872 -13.540   0.964  1.00 10.14           H  0.033
ATOM   1149 HG21 THR A  69      18.726 -13.041   2.209  1.00 12.21           H  0.037
ATOM   1150 HG22 THR A  69      18.625 -13.711   3.635  1.00 12.21           H  0.037
ATOM   1151 HG23 THR A  69      18.822 -14.610   2.352  1.00 12.21           H  0.037
ATOM   1152  H  ATHR A  69      14.420 -14.746   2.986  0.47 11.78           H  0.036
ATOM   1153  H  BTHR A  69      14.392 -14.820   3.117  0.53 11.78           H  0.036
ATOM   1154  N   PRO A  70      16.536 -16.243   5.381  1.00 11.59           N  0.036
ANISOU 1154  N   PRO A  70     2091   1725    589   -109    224    142       N
ATOM   1155  CA  PRO A  70      16.456 -16.221   6.844  1.00 14.22           C  0.040
ANISOU 1155  CA  PRO A  70     2428   2238    738    -44    350    264       C
ATOM   1156  C   PRO A  70      17.493 -15.334   7.477  1.00 17.74           C  0.044
ANISOU 1156  C   PRO A  70     2728   3073    940   -201    309    129       C
ATOM   1157  O   PRO A  70      18.662 -15.312   7.083  1.00 20.66           O  0.048
ANISOU 1157  O   PRO A  70     2657   3627   1565   -127    -85   -269       O
ATOM   1158  CB  PRO A  70      16.706 -17.688   7.222  1.00 16.04           C  0.042
ANISOU 1158  CB  PRO A  70     2848   2114   1133    194     73     66       C
ATOM   1159  CG  PRO A  70      16.235 -18.452   6.046  1.00 15.17           C  0.041
ANISOU 1159  CG  PRO A  70     2870   2123    771     94    162    203       C
ATOM   1160  CD  PRO A  70      16.564 -17.610   4.863  1.00 15.28           C  0.041
ANISOU 1160  CD  PRO A  70     2687   1933   1184    -67    257     35       C
ATOM   1161  HA  PRO A  70      15.572 -15.928   7.116  1.00 17.07           H  0.043
ATOM   1162  HB2 PRO A  70      17.652 -17.835   7.379  1.00 19.25           H  0.046
ATOM   1163  HB3 PRO A  70      16.199 -17.918   8.016  1.00 19.25           H  0.046
ATOM   1164  HG2 PRO A  70      16.696 -19.304   6.002  1.00 18.20           H  0.045
ATOM   1165  HG3 PRO A  70      15.278 -18.596   6.110  1.00 18.20           H  0.045
ATOM   1166  HD2 PRO A  70      17.445 -17.829   4.522  1.00 18.33           H  0.045
ATOM   1167  HD3 PRO A  70      15.900 -17.726   4.166  1.00 18.33           H  0.045
ATOM   1168  N   GLY A  71      17.055 -14.616   8.499  1.00 18.86           N  0.046
ANISOU 1168  N   GLY A  71     2989   3181    994   -471    269   -214       N
ATOM   1169  CA  GLY A  71      17.972 -13.839   9.285  1.00 23.25           C  0.051
ANISOU 1169  CA  GLY A  71     3286   3362   2187   -804    288   -455       C
ATOM   1170  C   GLY A  71      18.294 -12.528   8.640  1.00 23.98           C  0.051
ANISOU 1170  C   GLY A  71     3443   3341   2326  -1236    337   -375       C
ATOM   1171  O   GLY A  71      19.274 -11.878   9.024  1.00 26.43           O  0.054
ANISOU 1171  O   GLY A  71     3693   3533   2816  -1347   -123   -914       O
ATOM   1172  H   GLY A  71      16.234 -14.567   8.749  1.00 22.63           H  0.050
ATOM   1173  HA2 GLY A  71      17.583 -13.666  10.156  1.00 27.90           H  0.055
ATOM   1174  HA3 GLY A  71      18.797 -14.336   9.400  1.00 27.90           H  0.055
ATOM   1175  N   SER A  72      17.512 -12.138   7.649  1.00 23.48           N  0.051
ANISOU 1175  N   SER A  72     3449   3216   2255  -1408    374    -37       N
ATOM   1176  CA  SER A  72      17.840 -11.013   6.804  1.00 23.86           C  0.051
ANISOU 1176  CA  SER A  72     3597   3178   2289  -1427     13    190       C
ATOM   1177  C   SER A  72      17.106  -9.774   7.279  1.00 24.00           C  0.051
ANISOU 1177  C   SER A  72     3450   3055   2616  -1567    201    114       C
ATOM   1178  O   SER A  72      16.083  -9.810   7.976  1.00 25.12           O  0.053
ANISOU 1178  O   SER A  72     3570   3229   2745  -1709    151    126       O
ATOM   1179  CB  SER A  72      17.515 -11.298   5.327  1.00 23.60           C  0.051
ANISOU 1179  CB  SER A  72     3797   3224   1945  -1287   -216    731       C
ATOM   1180  OG  SER A  72      16.125 -11.209   5.071  1.00 24.05           O  0.051
ANISOU 1180  OG  SER A  72     3769   3287   2081  -1412   -557    648       O
ATOM   1181  H   SER A  72      16.770 -12.521   7.444  1.00 28.17           H  0.056
ATOM   1182  HA  SER A  72      18.794 -10.845   6.857  1.00 28.63           H  0.056
ATOM   1183  HB2 SER A  72      17.977 -10.647   4.774  1.00 28.32           H  0.056
ATOM   1184  HB3 SER A  72      17.817 -12.193   5.108  1.00 28.32           H  0.056
ATOM   1185  N   ARG A  73      17.676  -8.659   6.919  1.00 19.80           N  0.047
ANISOU 1185  N   ARG A  73     3194   2830   1497  -1491   -111     -6       N
ATOM   1186  CA  ARG A  73      17.026  -7.397   7.141  1.00 18.69           C  0.045
ANISOU 1186  CA  ARG A  73     2879   2609   1614  -1089     -9    234       C
ATOM   1187  C   ARG A  73      16.396  -6.941   5.827  1.00 19.28           C  0.046
ANISOU 1187  C   ARG A  73     2415   2417   2493   -766    116   -271       C
ATOM   1188  O   ARG A  73      16.578  -7.546   4.760  1.00 20.04           O  0.047
ANISOU 1188  O   ARG A  73     2339   2387   2889   -408    340    -88       O
ATOM   1189  CB  ARG A  73      18.063  -6.403   7.667  1.00 19.96           C  0.047
ANISOU 1189  CB  ARG A  73     3136   2666   1780  -1088     50   -164       C
ATOM   1190  CG  ARG A  73      18.798  -6.929   8.905  1.00 22.66           C  0.050
ANISOU 1190  CG  ARG A  73     3429   2737   2443   -807    -90   -535       C
ATOM   1191  CD  ARG A  73      17.934  -6.758  10.176  1.00 23.74           C  0.051
ANISOU 1191  CD  ARG A  73     3649   2787   2584   -877   -107   -779       C
ATOM   1192  NE  ARG A  73      17.946  -5.368  10.626  1.00 24.57           N  0.052
ANISOU 1192  NE  ARG A  73     3921   2951   2464   -699   -290  -1411       N
ATOM   1193  CZ  ARG A  73      18.922  -4.808  11.331  1.00 27.95           C  0.055
ANISOU 1193  CZ  ARG A  73     4104   3197   3320   -693   -520  -1409       C
ATOM   1194  NH1 ARG A  73      19.937  -5.521  11.796  1.00 30.15           N  0.058
```

```
ANISOU 1194  NH1 ARG A  73     4298   3336   3824   -626   -312  -1696       N
ATOM   1195  NH2 ARG A  73      18.866  -3.499  11.586  1.00 28.26           N  0.056
ANISOU 1195  NH2 ARG A  73     4102   3300   3335   -701   -801  -1071       N
ATOM   1196  H   ARG A  73      18.446  -8.604   6.540  1.00 23.75           H  0.051
ATOM   1197  HA  ARG A  73      16.317  -7.457   7.801  1.00 22.43           H  0.050
ATOM   1198  HB2 ARG A  73      18.720  -6.233   6.975  1.00 23.95           H  0.051
ATOM   1199  HB3 ARG A  73      17.616  -5.577   7.910  1.00 23.95           H  0.051
ATOM   1200  HG2 ARG A  73      18.991  -7.873   8.789  1.00 27.19           H  0.055
ATOM   1201  HG3 ARG A  73      19.623  -6.434   9.026  1.00 27.19           H  0.055
ATOM   1202  HD2 ARG A  73      17.018  -7.011   9.981  1.00 28.49           H  0.056
ATOM   1203  HD3 ARG A  73      18.288  -7.316  10.886  1.00 28.49           H  0.056
ATOM   1204  HE  ARG A  73      17.271  -4.877  10.419  1.00 29.48           H  0.057
ATOM   1205 HH11 ARG A  73      19.974  -6.366  11.644  1.00 36.18           H  0.063
ATOM   1206 HH12 ARG A  73      20.558  -5.138  12.251  1.00 36.18           H  0.063
ATOM   1207 HH21 ARG A  73      18.205  -3.032  11.295  1.00 33.91           H  0.061
ATOM   1208 HH22 ARG A  73      19.491  -3.123  12.042  1.00 33.91           H  0.061
ATOM   1209  N   ASN A  74      15.636  -5.869   5.939  1.00 17.01           N  0.043
ANISOU 1209  N   ASN A  74     1948   1974   2540   -656    407   -345       N
ATOM   1210  CA  ASN A  74      14.878  -5.227   4.868  1.00 14.66           C  0.040
ANISOU 1210  CA  ASN A  74     1956   1767   1849   -578    460   -541       C
ATOM   1211  C   ASN A  74      15.052  -3.730   5.065  1.00 13.89           C  0.039
ANISOU 1211  C   ASN A  74     2038   1596   1644   -437    211   -544       C
ATOM   1212  O   ASN A  74      14.121  -3.006   5.378  1.00 13.52           O  0.039
ANISOU 1212  O   ASN A  74     1928   1618   1589   -337    566   -441       O
ATOM   1213  CB  ASN A  74      13.410  -5.624   4.969  1.00 14.26           C  0.040
ANISOU 1213  CB  ASN A  74     1833   1792   1793   -641    756   -494       C
ATOM   1214  CG  ASN A  74      12.552  -4.961   3.937  1.00 13.86           C  0.039
ANISOU 1214  CG  ASN A  74     1796   1621   1848   -607    877   -569       C
ATOM   1215  OD1 ASN A  74      13.057  -4.342   3.018  1.00 13.18           O  0.038
ANISOU 1215  OD1 ASN A  74     1883   1643   1481   -645    600   -421       O
ATOM   1216  ND2 ASN A  74      11.226  -5.047   4.094  1.00 14.23           N  0.040
ANISOU 1216  ND2 ASN A  74     1821   1667   1919   -549    953   -393       N
ATOM   1217  H   ASN A  74      15.530  -5.456   6.686  1.00 20.41           H  0.047
ATOM   1218  HA  ASN A  74      15.214  -5.479   3.993  1.00 17.60           H  0.044
ATOM   1219  HB2 ASN A  74      13.334  -6.584   4.849  1.00 17.11           H  0.043
ATOM   1220  HB3 ASN A  74      13.075  -5.372   5.843  1.00 17.11           H  0.043
ATOM   1221 HD21 ASN A  74      10.700  -4.678   3.522  1.00 17.08           H  0.043
ATOM   1222 HD22 ASN A  74      10.899  -5.470   4.768  1.00 17.08           H  0.043
ATOM   1223  N   LEU A  75      16.288  -3.297   4.894  1.00 12.00           N  0.036
ANISOU 1223  N   LEU A  75     2189   1262   1109   -436     96   -538       N
ATOM   1224  CA  LEU A  75      16.653  -1.949   5.295  1.00 13.78           C  0.039
ANISOU 1224  CA  LEU A  75     2314   1534   1386   -415    169   -766       C
ATOM   1225  C   LEU A  75      16.052  -0.913   4.370  1.00 13.01           C  0.038
ANISOU 1225  C   LEU A  75     2275   1403   1266   -588    501   -606       C
ATOM   1226  O   LEU A  75      15.933   0.230   4.791  1.00 15.49           O  0.041
ANISOU 1226  O   LEU A  75     2592   1425   1867   -641    669   -655       O
ATOM   1227  CB  LEU A  75      18.173  -1.806   5.387  1.00 16.09           C  0.042
ANISOU 1227  CB  LEU A  75     2452   1780   1881   -551     14   -483       C
ATOM   1228  CG  LEU A  75      18.786  -2.572   6.574  1.00 18.53           C  0.045
ANISOU 1228  CG  LEU A  75     2719   2276   2046   -534      6   -307       C
ATOM   1229  CD1 LEU A  75      20.304  -2.596   6.599  1.00 19.82           C  0.047
ANISOU 1229  CD1 LEU A  75     2782   2493   2256   -443    -59   -165       C
ATOM   1230  CD2 LEU A  75      18.287  -1.970   7.875  1.00 20.91           C  0.048
ANISOU 1230  CD2 LEU A  75     2946   2502   2498   -460   -418   -190       C
ATOM   1231  H   LEU A  75      16.928  -3.757   4.551  1.00 14.40           H  0.040
ATOM   1232  HA  LEU A  75      16.314  -1.775   6.187  1.00 16.53           H  0.043
ATOM   1233  HB2 LEU A  75      18.570  -2.151   4.572  1.00 19.31           H  0.046
ATOM   1234  HB3 LEU A  75      18.393  -0.867   5.492  1.00 19.31           H  0.046
ATOM   1235  HG  LEU A  75      18.506  -3.495   6.474  1.00 22.24           H  0.049
ATOM   1236 HD11 LEU A  75      20.601  -3.481   6.863  1.00 23.79           H  0.051
ATOM   1237 HD12 LEU A  75      20.638  -2.385   5.713  1.00 23.79           H  0.051
ATOM   1238 HD13 LEU A  75      20.619  -1.937   7.237  1.00 23.79           H  0.051
ATOM   1239 HD21 LEU A  75      18.789  -2.352   8.612  1.00 25.10           H  0.053
ATOM   1240 HD22 LEU A  75      18.417  -1.009   7.849  1.00 25.10           H  0.053
ATOM   1241 HD23 LEU A  75      17.344  -2.174   7.976  1.00 25.10           H  0.053
ATOM   1242  N   CYS A  76      15.636  -1.284   3.148  1.00 13.43           N  0.038
ANISOU 1242  N   CYS A  76     1997   1521   1584   -523    532   -566       N
ATOM   1243  CA  CYS A  76      14.964  -0.347   2.268  1.00 12.60           C  0.037
ANISOU 1243  CA  CYS A  76     1676   1326   1784   -462    505   -550       C
ATOM   1244  C   CYS A  76      13.449  -0.395   2.447  1.00 13.35           C  0.038
ANISOU 1244  C   CYS A  76     1734   1377   1960   -165    688   -462       C
ATOM   1245  O   CYS A  76      12.723   0.369   1.802  1.00 13.82           O  0.039
ANISOU 1245  O   CYS A  76     1784   1323   2145   -139    629   -294       O
ATOM   1246  CB  CYS A  76      15.346  -0.613   0.813  1.00 12.12           C  0.037
ANISOU 1246  CB  CYS A  76     1482   1187   1936   -487    234   -341       C
ATOM   1247  SG  CYS A  76      17.061  -0.142   0.500  1.00 10.58           S  0.034
ANISOU 1247  SG  CYS A  76     1203   1217   1598   -318     72   -318       S
ATOM   1248  H   CYS A  76      15.737  -2.072   2.819  1.00 16.11           H  0.042
```

```
ATOM   1249  HA  CYS A  76      15.261   0.554   2.467  1.00 15.12           H  0.041
ATOM   1250  HB2 CYS A  76      15.246  -1.558   0.620  1.00 14.54           H  0.040
ATOM   1251  HB3 CYS A  76      14.771  -0.093   0.229  1.00 14.54           H  0.040
ATOM   1252  N   ASN A  77      12.968  -1.267   3.324  1.00 13.22           N  0.038
ANISOU 1252  N   ASN A  77     1760   1351   1914    -92    596   -726       N
ATOM   1253  CA  ASN A  77      11.546  -1.392   3.605  1.00 13.17           C  0.038
ANISOU 1253  CA  ASN A  77     1923   1546   1536   -138    825   -586       C
ATOM   1254  C   ASN A  77      10.741  -1.567   2.335  1.00 14.16           C  0.039
ANISOU 1254  C   ASN A  77     1690   1523   2167   -190    509   -606       C
ATOM   1255  O   ASN A  77       9.806  -0.837   2.062  1.00 14.84           O  0.040
ANISOU 1255  O   ASN A  77     1778   1563   2298    -91    439   -720       O
ATOM   1256  CB  ASN A  77      11.074  -0.195   4.423  1.00 17.31           C  0.044
ANISOU 1256  CB  ASN A  77     2425   1982   2171   -252    898   -379       C
ATOM   1257  CG  ASN A  77      11.770  -0.143   5.740  1.00 22.39           C  0.050
ANISOU 1257  CG  ASN A  77     3067   2517   2923   -274    992   -549       C
ATOM   1258  OD1 ASN A  77      11.520  -0.994   6.608  1.00 25.36           O  0.053
ANISOU 1258  OD1 ASN A  77     3517   3003   3115    -13   1004   -595       O
ATOM   1259  ND2 ASN A  77      12.706   0.797   5.892  1.00 25.66           N  0.053
ANISOU 1259  ND2 ASN A  77     3312   2813   3624   -222    901   -722       N
ATOM   1260  H   ASN A  77      13.458  -1.808   3.779  1.00 15.87           H  0.042
ATOM   1261  HA  ASN A  77      11.395  -2.195   4.128  1.00 15.81           H  0.042
ATOM   1262  HB2 ASN A  77      11.269   0.623   3.940  1.00 20.77           H  0.048
ATOM   1263  HB3 ASN A  77      10.120  -0.267   4.582  1.00 20.77           H  0.048
ATOM   1264 HD21 ASN A  77      12.882   1.337   5.246  1.00 30.79           H  0.058
ATOM   1265 HD22 ASN A  77      13.133   0.863   6.636  1.00 30.79           H  0.058
ATOM   1266  N   ILE A  78      11.120  -2.570   1.549  1.00 13.36           N  0.038
ANISOU 1266  N   ILE A  78     1370   1527   2178   -228    370   -608       N
ATOM   1267  CA  ILE A  78      10.428  -2.842   0.303  1.00 12.85           C  0.038
ANISOU 1267  CA  ILE A  78     1366   1363   2153   -298    441   -577       C
ATOM   1268  C   ILE A  78      10.425  -4.341   0.037  1.00 12.66           C  0.037
ANISOU 1268  C   ILE A  78     1258   1311   2243   -157    238   -561       C
ATOM   1269  O   ILE A  78      11.354  -5.065   0.447  1.00 14.10           O  0.039
ANISOU 1269  O   ILE A  78     1356   1437   2566   -103    169   -347       O
ATOM   1270  CB  ILE A  78      11.028  -2.132  -0.922  1.00 15.23           C  0.041
ANISOU 1270  CB  ILE A  78     1543   1437   2808   -197    773   -579       C
ATOM   1271  CG1 ILE A  78      12.530  -2.411  -1.015  1.00 16.75           C  0.043
ANISOU 1271  CG1 ILE A  78     1685   1634   3044   -280    628   -411       C
ATOM   1272  CG2 ILE A  78      10.662  -0.637  -0.918  1.00 18.56           C  0.045
ANISOU 1272  CG2 ILE A  78     1854   1544   3656    -53    814   -374       C
ATOM   1273  CD1 ILE A  78      13.080  -2.025  -2.337  1.00 18.54           C  0.045
ANISOU 1273  CD1 ILE A  78     1833   1754   3457   -136    697   -310       C
ATOM   1274  H   ILE A  78      11.774  -3.102   1.718  1.00 16.03           H  0.042
ATOM   1275  HA  ILE A  78       9.524  -2.514   0.432  1.00 15.42           H  0.041
ATOM   1276  HB  ILE A  78      10.637  -2.495  -1.732  1.00 18.28           H  0.045
ATOM   1277 HG12 ILE A  78      12.992  -1.900  -0.333  1.00 20.09           H  0.047
ATOM   1278 HG13 ILE A  78      12.687  -3.359  -0.885  1.00 20.09           H  0.047
ATOM   1279 HG21 ILE A  78      11.068  -0.211  -1.690  1.00 22.28           H  0.049
ATOM   1280 HG22 ILE A  78       9.697  -0.548  -0.960  1.00 22.28           H  0.049
ATOM   1281 HG23 ILE A  78      10.997  -0.232  -0.103  1.00 22.28           H  0.049
ATOM   1282 HD11 ILE A  78      14.047  -2.107  -2.312  1.00 22.24           H  0.049
ATOM   1283 HD12 ILE A  78      12.716  -2.615  -3.015  1.00 22.24           H  0.049
ATOM   1284 HD13 ILE A  78      12.831  -1.108  -2.528  1.00 22.24           H  0.049
ATOM   1285  N   PRO A  79       9.381  -4.859  -0.586  1.00 12.66           N  0.037
ANISOU 1285  N   PRO A  79     1199   1340   2272    -81     37   -623       N
ATOM   1286  CA  PRO A  79       9.485  -6.214  -1.129  1.00 12.29           C  0.037
ANISOU 1286  CA  PRO A  79     1216   1253   2199   -337     24   -461       C
ATOM   1287  C   PRO A  79      10.518  -6.213  -2.241  1.00 11.12           C  0.035
ANISOU 1287  C   PRO A  79     1193   1232   1802   -294    117   -279       C
ATOM   1288  O   PRO A  79      10.593  -5.278  -3.047  1.00 11.20           O  0.035
ANISOU 1288  O   PRO A  79     1090   1298   1866   -191    -13    185       O
ATOM   1289  CB  PRO A  79       8.083  -6.488  -1.665  1.00 12.49           C  0.037
ANISOU 1289  CB  PRO A  79     1175   1486   2085   -354    175     24       C
ATOM   1290  CG  PRO A  79       7.449  -5.175  -1.831  1.00 13.68           C  0.039
ANISOU 1290  CG  PRO A  79     1091   1692   2415   -228     -9     34       C
ATOM   1291  CD  PRO A  79       8.083  -4.227  -0.893  1.00 12.36           C  0.037
ANISOU 1291  CD  PRO A  79     1138   1439   2120    -21     63   -304       C
ATOM   1292  HA  PRO A  79       9.698  -6.876  -0.453  1.00 14.75           H  0.040
ATOM   1293  HB2 PRO A  79       8.143  -6.950  -2.516  1.00 14.99           H  0.041
ATOM   1294  HB3 PRO A  79       7.591  -7.029  -1.028  1.00 14.99           H  0.041
ATOM   1295  HG2 PRO A  79       7.577  -4.875  -2.745  1.00 16.42           H  0.042
ATOM   1296  HG3 PRO A  79       6.502  -5.254  -1.637  1.00 16.42           H  0.042
ATOM   1297  HD2 PRO A  79       8.212  -3.362  -1.313  1.00 14.84           H  0.040
ATOM   1298  HD3 PRO A  79       7.549  -4.129  -0.089  1.00 14.84           H  0.040
ATOM   1299  N   CYS A  80      11.356  -7.253  -2.255  1.00 10.49           N  0.034
ANISOU 1299  N   CYS A  80     1321   1277   1387   -321    161    -39       N
ATOM   1300  CA  CYS A  80      12.311  -7.382  -3.348  1.00  7.90           C  0.029
ANISOU 1300  CA  CYS A  80     1022   1197    782   -284    245     -1       C
ATOM   1301  C   CYS A  80      11.647  -7.285  -4.720  1.00  7.83           C  0.029
```

```
ANISOU 1301  C   CYS A  80      933   1255    787   -141    117    -89       C
ATOM   1302  O   CYS A  80      12.272  -6.809  -5.680  1.00 10.06           O  0.033
ANISOU 1302  O   CYS A  80      994   1366   1463   -160    168    -41       O
ATOM   1303  CB  CYS A  80      13.133  -8.642  -3.226  1.00  8.65           C  0.031
ANISOU 1303  CB  CYS A  80      875   1125   1288   -130    456   -216       C
ATOM   1304  SG  CYS A  80      14.078  -8.738  -1.689  1.00  8.95           S  0.031
ANISOU 1304  SG  CYS A  80     1004   1128   1270   -222    217   -337       S
ATOM   1305  H   CYS A  80      11.388  -7.875  -1.662  1.00 12.59           H  0.037
ATOM   1306  HA  CYS A  80      12.934  -6.642  -3.277  1.00  9.48           H  0.032
ATOM   1307  HB2 CYS A  80      12.539  -9.408  -3.259  1.00 10.38           H  0.034
ATOM   1308  HB3 CYS A  80      13.762  -8.678  -3.964  1.00 10.38           H  0.034
ATOM   1309  N   SER A  81      10.403  -7.754  -4.863  1.00 10.00           N  0.033
ANISOU 1309  N   SER A  81      965   1437   1396   -256    265     -8       N
ATOM   1310  CA  SER A  81       9.774  -7.678  -6.174  1.00 11.74           C  0.036
ANISOU 1310  CA  SER A  81     1214   1659   1589   -369    290     35       C
ATOM   1311  C   SER A  81       9.652  -6.231  -6.649  1.00 13.40           C  0.038
ANISOU 1311  C   SER A  81     1169   1804   2119   -410    -46    166       C
ATOM   1312  O   SER A  81       9.618  -5.983  -7.862  1.00 15.00           O  0.041
ANISOU 1312  O   SER A  81     1175   2040   2483   -545     96    109       O
ATOM   1313  CB  SER A  81       8.392  -8.342  -6.120  1.00 14.60           C  0.040
ANISOU 1313  CB  SER A  81     1240   1797   2510   -341    -27    -49       C
ATOM   1314  OG  SER A  81       7.553  -7.641  -5.215  1.00 15.81           O  0.042
ANISOU 1314  OG  SER A  81     1260   1904   2842   -253    248   -159       O
ATOM   1315  H   SER A  81       9.926  -8.106  -4.240  1.00 11.99           H  0.036
ATOM   1316  HA  SER A  81      10.316  -8.159  -6.818  1.00 14.09           H  0.039
ATOM   1317  HB2 SER A  81       7.994  -8.323  -7.005  1.00 17.52           H  0.044
ATOM   1318  HB3 SER A  81       8.490  -9.259  -5.820  1.00 17.52           H  0.044
ATOM   1319  HG  SER A  81       6.939  -8.146  -4.942  1.00 18.97           H  0.046
ATOM   1320  N   ALA A  82       9.556  -5.265  -5.724  1.00 11.54           N  0.036
ANISOU 1320  N   ALA A  82     1097   1509   1777   -193    -59    264       N
ATOM   1321  CA  ALA A  82       9.458  -3.855  -6.125  1.00 13.44           C  0.038
ANISOU 1321  CA  ALA A  82     1276   1517   2312   -229    -53    176       C
ATOM   1322  C   ALA A  82      10.667  -3.439  -6.945  1.00 13.56           C  0.039
ANISOU 1322  C   ALA A  82     1334   1306   2514   -343   -439    174       C
ATOM   1323  O   ALA A  82      10.598  -2.505  -7.750  1.00 14.84           O  0.040
ANISOU 1323  O   ALA A  82     1362   1548   2730   -443   -342    153       O
ATOM   1324  CB  ALA A  82       9.344  -2.944  -4.918  1.00 14.92           C  0.040
ANISOU 1324  CB  ALA A  82     1248   1672   2749   -169    394    358       C
ATOM   1325  H   ALA A  82       9.545  -5.397  -4.874  1.00 13.85           H  0.039
ATOM   1326  HA  ALA A  82       8.650  -3.751  -6.652  1.00 16.12           H  0.042
ATOM   1327  HB1 ALA A  82       9.348  -2.021  -5.217  1.00 17.91           H  0.044
ATOM   1328  HB2 ALA A  82       8.516  -3.138  -4.452  1.00 17.91           H  0.044
ATOM   1329  HB3 ALA A  82      10.099  -3.104  -4.329  1.00 17.91           H  0.044
ATOM   1330  N   LEU A  83      11.777  -4.120  -6.746  1.00 10.59           N  0.034
ANISOU 1330  N   LEU A  83     1014   1057   1953   -339   -230     40       N
ATOM   1331  CA  LEU A  83      13.021  -3.812  -7.443  1.00 12.05           C  0.036
ANISOU 1331  CA  LEU A  83     1286   1136   2157   -457   -228    282       C
ATOM   1332  C   LEU A  83      13.053  -4.329  -8.869  1.00 11.16           C  0.035
ANISOU 1332  C   LEU A  83     1182   1196   1861   -441   -108    213       C
ATOM   1333  O   LEU A  83      14.071  -4.171  -9.557  1.00  9.88           O  0.033
ANISOU 1333  O   LEU A  83     1230   1188   1337   -411   -107    146       O
ATOM   1334  CB  LEU A  83      14.151  -4.384  -6.616  1.00  8.75           C  0.031
ANISOU 1334  CB  LEU A  83     1226    957   1143   -539   -322    -86       C
ATOM   1335  CG  LEU A  83      14.329  -3.856  -5.199  1.00 11.23           C  0.035
ANISOU 1335  CG  LEU A  83     1759    990   1517   -577   -375     94       C
ATOM   1336  CD1 LEU A  83      15.324  -4.607  -4.403  1.00 13.07           C  0.038
ANISOU 1336  CD1 LEU A  83     1745   1174   2047   -726   -320    109       C
ATOM   1337  CD2 LEU A  83      14.749  -2.374  -5.283  1.00 12.73           C  0.037
ANISOU 1337  CD2 LEU A  83     1962   1179   1696   -625   -514    188       C
ATOM   1338  H   LEU A  83      11.843  -4.781  -6.200  1.00 12.71           H  0.037
ATOM   1339  HA  LEU A  83      13.148  -2.853  -7.519  1.00 14.46           H  0.040
ATOM   1340  HB2 LEU A  83      14.009  -5.341  -6.543  1.00 10.50           H  0.034
ATOM   1341  HB3 LEU A  83      14.981  -4.207  -7.087  1.00 10.50           H  0.034
ATOM   1342  HG  LEU A  83      13.485  -3.962  -4.733  1.00 13.47           H  0.038
ATOM   1343 HD11 LEU A  83      15.428  -4.175  -3.540  1.00 15.68           H  0.042
ATOM   1344 HD12 LEU A  83      15.010  -5.517  -4.282  1.00 15.68           H  0.042
ATOM   1345 HD13 LEU A  83      16.170  -4.609  -4.876  1.00 15.68           H  0.042
ATOM   1346 HD21 LEU A  83      14.947  -2.051  -4.390  1.00 15.28           H  0.041
ATOM   1347 HD22 LEU A  83      15.537  -2.300  -5.844  1.00 15.28           H  0.041
ATOM   1348 HD23 LEU A  83      14.021  -1.860  -5.666  1.00 15.28           H  0.041
ATOM   1349  N   LEU A  84      11.991  -4.989  -9.341  1.00 11.47           N  0.036
ANISOU 1349  N   LEU A  84     1159   1443   1758   -615    -74    275       N
ATOM   1350  CA  LEU A  84      11.967  -5.583 -10.669  1.00 11.06           C  0.035
ANISOU 1350  CA  LEU A  84     1000   1359   1842   -673    -86    -91       C
ATOM   1351  C   LEU A  84      11.077  -4.807 -11.614  1.00 13.10           C  0.038
ANISOU 1351  C   LEU A  84     1287   1369   2321   -316     67   -114       C
ATOM   1352  O   LEU A  84      10.973  -5.158 -12.803  1.00 12.89           O  0.038
ANISOU 1352  O   LEU A  84     1492   1274   2132   -283    133   -251       O
```

```
ATOM   1353  CB  LEU A  84      11.481  -7.033 -10.591  1.00 11.24           C  0.035
ANISOU 1353  CB  LEU A  84     1039   1458   1773   -671   -211   -130       C
ATOM   1354  CG  LEU A  84      12.303  -8.014  -9.761  1.00 11.30           C  0.035
ANISOU 1354  CG  LEU A  84     1099   1463   1733   -664   -242     80       C
ATOM   1355  CD1 LEU A  84      11.708  -9.423  -9.971  1.00 15.13           C  0.041
ANISOU 1355  CD1 LEU A  84     1439   1724   2586   -860   -245    190       C
ATOM   1356  CD2 LEU A  84      13.756  -8.000 -10.085  1.00 10.27           C  0.034
ANISOU 1356  CD2 LEU A  84     1128   1582   1191   -493   -182     -3       C
ATOM   1357  H   LEU A  84      11.262  -5.105  -8.898  1.00 13.77           H  0.039
ATOM   1358  HA  LEU A  84      12.868  -5.594 -11.028  1.00 13.27           H  0.038
ATOM   1359  HB2 LEU A  84      10.588  -7.026 -10.212  1.00 13.49           H  0.039
ATOM   1360  HB3 LEU A  84      11.455  -7.385 -11.495  1.00 13.49           H  0.039
ATOM   1361  HG  LEU A  84      12.255  -7.751  -8.828  1.00 13.57           H  0.039
ATOM   1362 HD11 LEU A  84      11.951  -9.983  -9.218  1.00 18.16           H  0.045
ATOM   1363 HD12 LEU A  84      10.743  -9.352 -10.033  1.00 18.16           H  0.045
ATOM   1364 HD13 LEU A  84      12.066  -9.797 -10.791  1.00 18.16           H  0.045
ATOM   1365 HD21 LEU A  84      14.215  -8.630  -9.506  1.00 12.32           H  0.037
ATOM   1366 HD22 LEU A  84      13.875  -8.257 -11.013  1.00 12.32           H  0.037
ATOM   1367 HD23 LEU A  84      14.103  -7.106  -9.942  1.00 12.32           H  0.037
ATOM   1368  N   SER A  85      10.468  -3.740 -11.111  1.00 13.92           N  0.039
ANISOU 1368  N   SER A  85     1296   1575   2418   -139   -126    191       N
ATOM   1369  CA  SER A  85       9.518  -2.929 -11.870  1.00 13.78           C  0.039
ANISOU 1369  CA  SER A  85     1154   1648   2432    -17    -22    663       C
ATOM   1370  C   SER A  85      10.150  -2.239 -13.071  1.00 14.47           C  0.040
ANISOU 1370  C   SER A  85     1188   1874   2437   -131   -165    656       C
ATOM   1371  O   SER A  85      11.326  -1.869 -13.054  1.00 14.31           O  0.040
ANISOU 1371  O   SER A  85     1178   1708   2553   -289   -269    767       O
ATOM   1372  CB  SER A  85       8.950  -1.854 -10.945  1.00 16.88           C  0.043
ANISOU 1372  CB  SER A  85     1324   1914   3178    243    203    674       C
ATOM   1373  OG  SER A  85       8.083  -0.989 -11.676  1.00 19.64           O  0.046
ANISOU 1373  OG  SER A  85     1468   2235   3760    165    -51    444       O
ATOM   1374  H   SER A  85      10.588  -3.453 -10.309  1.00 16.70           H  0.043
ATOM   1375  HA  SER A  85       8.821  -3.513 -12.209  1.00 16.53           H  0.043
ATOM   1376  HB2 SER A  85       8.450  -2.279 -10.231  1.00 20.26           H  0.047
ATOM   1377  HB3 SER A  85       9.681  -1.334 -10.574  1.00 20.26           H  0.047
ATOM   1378  HG  SER A  85       7.622  -1.435 -12.218  1.00 23.57           H  0.051
ATOM   1379  N   SER A  86       9.335  -1.997 -14.112  1.00 14.26           N  0.040
ANISOU 1379  N   SER A  86     1237   2043   2137   -261   -366    613       N
ATOM   1380  CA  SER A  86       9.770  -1.110 -15.197  1.00 17.47           C  0.044
ANISOU 1380  CA  SER A  86     1404   2545   2688   -281   -579    834       C
ATOM   1381  C   SER A  86      10.016   0.318 -14.704  1.00 18.43           C  0.045
ANISOU 1381  C   SER A  86     1453   2464   3085   -240   -736    853       C
ATOM   1382  O   SER A  86      10.830   1.053 -15.291  1.00 20.61           O  0.048
ANISOU 1382  O   SER A  86     1541   2526   3762   -413   -862   1257       O
ATOM   1383  CB  SER A  86       8.778  -1.143 -16.379  1.00 20.12           C  0.047
ANISOU 1383  CB  SER A  86     1644   3043   2959   -193   -411   1092       C
ATOM   1384  OG  SER A  86       7.501  -0.648 -16.026  1.00 25.86           O  0.053
ANISOU 1384  OG  SER A  86     2262   3455   4110   -191   -392   1037       O
ATOM   1385  H   SER A  86       8.546  -2.326 -14.209  1.00 17.11           H  0.043
ATOM   1386  HA  SER A  86      10.613  -1.443 -15.541  1.00 20.96           H  0.048
ATOM   1387  HB2 SER A  86       9.134  -0.597 -17.097  1.00 24.15           H  0.052
ATOM   1388  HB3 SER A  86       8.683  -2.061 -16.677  1.00 24.15           H  0.052
ATOM   1389  HG  SER A  86       7.196  -1.081 -15.373  1.00 31.04           H  0.058
ATOM   1390  N   ASP A  87       9.370   0.722 -13.621  1.00 15.49           N  0.041
ANISOU 1390  N   ASP A  87     1292   2240   2353    -12   -811    347       N
ATOM   1391  CA  ASP A  87       9.598   2.032 -13.037  1.00 16.21           C  0.042
ANISOU 1391  CA  ASP A  87     1499   1990   2670     86   -786    646       C
ATOM   1392  C   ASP A  87      10.736   1.881 -12.048  1.00 14.29           C  0.040
ANISOU 1392  C   ASP A  87     1379   1669   2381    -18   -499    739       C
ATOM   1393  O   ASP A  87      10.658   1.054 -11.128  1.00 14.65           O  0.040
ANISOU 1393  O   ASP A  87     1380   1596   2591   -137   -333    613       O
ATOM   1394  CB  ASP A  87       8.354   2.563 -12.332  1.00 19.10           C  0.046
ANISOU 1394  CB  ASP A  87     1745   2269   3245    177   -865    285       C
ATOM   1395  CG  ASP A  87       8.577   3.947 -11.741  1.00 22.21           C  0.049
ANISOU 1395  CG  ASP A  87     2128   2415   3894    446   -739    219       C
ATOM   1396  OD1 ASP A  87       9.153   4.011 -10.647  1.00 21.59           O  0.049
ANISOU 1396  OD1 ASP A  87     2015   2335   3854    412   -852     81       O
ATOM   1397  OD2 ASP A  87       8.248   4.972 -12.373  1.00 25.38           O  0.053
ANISOU 1397  OD2 ASP A  87     2507   2687   4450    598   -371    171       O
ATOM   1398  H   ASP A  87       8.788   0.249 -13.201  1.00 18.59           H  0.045
ATOM   1399  HA  ASP A  87       9.822   2.678 -13.725  1.00 19.45           H  0.046
ATOM   1400  HB2 ASP A  87       7.625   2.620 -12.970  1.00 22.92           H  0.050
ATOM   1401  HB3 ASP A  87       8.115   1.960 -11.610  1.00 22.92           H  0.050
ATOM   1402  N   ILE A  88      11.778   2.699 -12.216  1.00 13.70           N  0.039
ANISOU 1402  N   ILE A  88     1250   1450   2505     79   -390    875       N
ATOM   1403  CA  ILE A  88      12.992   2.519 -11.417  1.00 12.30           C  0.037
ANISOU 1403  CA  ILE A  88      964   1304   2407     24   -402    747       C
ATOM   1404  C   ILE A  88      12.944   3.137 -10.024  1.00 11.61           C  0.036
```

```
ANISOU 1404  C   ILE A  88      863   1212   2336    180   -186    552       C
ATOM   1405  O   ILE A  88      13.961   3.093  -9.309  1.00 11.99           O  0.036
ANISOU 1405  O   ILE A  88      891   1208   2456    208   -370    360       O
ATOM   1406  CB  ILE A  88      14.249   2.968 -12.197  1.00 11.66           C  0.036
ANISOU 1406  CB  ILE A  88     1076   1325   2030    -43   -353    611       C
ATOM   1407  CG1 ILE A  88      14.270   4.485 -12.393  1.00 13.34           C  0.038
ANISOU 1407  CG1 ILE A  88     1428   1497   2143    -79    -57    752       C
ATOM   1408  CG2 ILE A  88      14.344   2.199 -13.525  1.00 13.89           C  0.039
ANISOU 1408  CG2 ILE A  88     1173   1689   2417    -69   -756    392       C
ATOM   1409  CD1 ILE A  88      15.663   5.023 -12.887  1.00 13.64           C  0.039
ANISOU 1409  CD1 ILE A  88     1440   1611   2131    -64    366    720       C
ATOM   1410  H   ILE A  88      11.805   3.352 -12.774  1.00 16.44           H  0.043
ATOM   1411  HA  ILE A  88      13.080   1.567 -11.253  1.00 14.76           H  0.040
ATOM   1412  HB  ILE A  88      15.036   2.752 -11.673  1.00 14.00           H  0.039
ATOM   1413 HG12 ILE A  88      13.604   4.726 -13.056  1.00 16.01           H  0.042
ATOM   1414 HG13 ILE A  88      14.067   4.914 -11.547  1.00 16.01           H  0.042
ATOM   1415 HG21 ILE A  88      15.263   2.213 -13.833  1.00 16.67           H  0.043
ATOM   1416 HG22 ILE A  88      14.057   1.283 -13.381  1.00 16.67           H  0.043
ATOM   1417 HG23 ILE A  88      13.769   2.626 -14.179  1.00 16.67           H  0.043
ATOM   1418 HD11 ILE A  88      15.676   5.990 -12.804  1.00 16.37           H  0.042
ATOM   1419 HD12 ILE A  88      16.364   4.635 -12.340  1.00 16.37           H  0.042
ATOM   1420 HD13 ILE A  88      15.789   4.769 -13.815  1.00 16.37           H  0.042
ATOM   1421  N   THR A  89      11.816   3.708  -9.580  1.00 13.93           N  0.039
ANISOU 1421  N   THR A  89     1078   1391   2822    419    -72    207       N
ATOM   1422  CA  THR A  89      11.802   4.414  -8.292  1.00 14.20           C  0.040
ANISOU 1422  CA  THR A  89     1001   1570   2826    385    207     33       C
ATOM   1423  C   THR A  89      12.347   3.597  -7.118  1.00 12.75           C  0.037
ANISOU 1423  C   THR A  89      984   1272   2590     80    251    -27       C
ATOM   1424  O   THR A  89      13.181   4.068  -6.325  1.00 12.86           O  0.038
ANISOU 1424  O   THR A  89     1067   1154   2666     64    282   -404       O
ATOM   1425  CB  THR A  89      10.369   4.875  -7.928  1.00 16.66           C  0.043
ANISOU 1425  CB  THR A  89     1111   1979   3238    585     60    165       C
ATOM   1426  OG1 THR A  89       9.899   5.779  -8.908  1.00 19.69           O  0.047
ANISOU 1426  OG1 THR A  89     1330   2350   3800    548   -297    566       O
ATOM   1427  CG2 THR A  89      10.400   5.554  -6.552  1.00 18.03           C  0.045
ANISOU 1427  CG2 THR A  89     1390   2186   3274    463    356    334       C
ATOM   1428  H   THR A  89      11.063   3.700  -9.996  1.00 16.71           H  0.043
ATOM   1429  HA  THR A  89      12.382   5.180  -8.422  1.00 17.05           H  0.043
ATOM   1430  HB  THR A  89       9.763   4.119  -7.892  1.00 19.99           H  0.047
ATOM   1431  HG1 THR A  89       9.985   5.444  -9.673  1.00 23.62           H  0.051
ATOM   1432 HG21 THR A  89       9.602   6.092  -6.430  1.00 21.63           H  0.049
ATOM   1433 HG22 THR A  89      10.437   4.883  -5.852  1.00 21.63           H  0.049
ATOM   1434 HG23 THR A  89      11.180   6.126  -6.482  1.00 21.63           H  0.049
ATOM   1435  N   ALA A  90      11.825   2.385  -6.926  1.00 12.04           N  0.036
ANISOU 1435  N   ALA A  90     1040   1051   2483   -148    327    -71       N
ATOM   1436  CA  ALA A  90      12.211   1.668  -5.725  1.00 11.78           C  0.036
ANISOU 1436  CA  ALA A  90     1113   1233   2130   -227    344    300       C
ATOM   1437  C   ALA A  90      13.698   1.335  -5.749  1.00 10.08           C  0.033
ANISOU 1437  C   ALA A  90      978   1144   1709   -126     50    212       C
ATOM   1438  O   ALA A  90      14.369   1.426  -4.712  1.00 11.07           O  0.035
ANISOU 1438  O   ALA A  90     1210   1049   1947   -252     86     48       O
ATOM   1439  CB  ALA A  90      11.321   0.436  -5.500  1.00 12.43           C  0.037
ANISOU 1439  CB  ALA A  90     1074   1473   2178   -351    566    297       C
ATOM   1440  H   ALA A  90      11.274   1.982  -7.450  1.00 14.45           H  0.040
ATOM   1441  HA  ALA A  90      12.058   2.234  -4.952  1.00 14.13           H  0.039
ATOM   1442  HB1 ALA A  90      11.734  -0.135  -4.833  1.00 14.92           H  0.040
ATOM   1443  HB2 ALA A  90      10.449   0.728  -5.191  1.00 14.92           H  0.040
ATOM   1444  HB3 ALA A  90      11.231  -0.046  -6.337  1.00 14.92           H  0.040
ATOM   1445  N   SER A  91      14.218   0.928  -6.918  1.00  9.41           N  0.032
ANISOU 1445  N   SER A  91      951   1185   1440   -242   -242    184       N
ATOM   1446  CA  SER A  91      15.636   0.593  -7.026  1.00  7.25           C  0.028
ANISOU 1446  CA  SER A  91      850    908    996     36     37     41       C
ATOM   1447  C   SER A  91      16.499   1.819  -6.773  1.00  7.92           C  0.030
ANISOU 1447  C   SER A  91     1051    897   1060    -29     65    -26       C
ATOM   1448  O   SER A  91      17.494   1.752  -6.052  1.00  9.48           O  0.032
ANISOU 1448  O   SER A  91     1175    865   1564     31     28    229       O
ATOM   1449  CB  SER A  91      15.952   0.050  -8.418  1.00  8.86           C  0.031
ANISOU 1449  CB  SER A  91     1086    940   1340   -195    -59     88       C
ATOM   1450  OG  SER A  91      15.626  -1.331  -8.466  1.00  8.41           O  0.030
ANISOU 1450  OG  SER A  91     1109    766   1320   -184   -144    -35       O
ATOM   1451  H   SER A  91      13.774   0.841  -7.650  1.00 11.29           H  0.035
ATOM   1452  HA  SER A  91      15.837  -0.091  -6.368  1.00  8.70           H  0.031
ATOM   1453  HB2 SER A  91      15.426   0.528  -9.078  1.00 10.63           H  0.034
ATOM   1454  HB3 SER A  91      16.898   0.164  -8.602  1.00 10.63           H  0.034
ATOM   1455  HG  SER A  91      14.858  -1.456  -8.151  1.00 10.09           H  0.033
ATOM   1456  N   VAL A  92      16.079   2.959  -7.284  1.00  9.30           N  0.032
ANISOU 1456  N   VAL A  92     1086   1026   1423    -20     40    208       N
ATOM   1457  CA  VAL A  92      16.831   4.186  -7.047  1.00  8.96           C  0.031
```

```
ANISOU 1457  CA  VAL A  92     1033    701   1671    -61     47    510       C
ATOM   1458  C   VAL A  92      16.810   4.565  -5.582  1.00 10.16           C  0.033
ANISOU 1458  C   VAL A  92     1189    782   1890    -40    428    309       C
ATOM   1459  O   VAL A  92      17.844   4.827  -4.990  1.00 10.68           O  0.034
ANISOU 1459  O   VAL A  92     1252    784   2022   -182    228   -226       O
ATOM   1460  CB  VAL A  92      16.325   5.333  -7.939  1.00 10.25           C  0.034
ANISOU 1460  CB  VAL A  92     1033    960   1903   -165     57    657       C
ATOM   1461  CG1 VAL A  92      16.923   6.659  -7.458  1.00 11.81           C  0.036
ANISOU 1461  CG1 VAL A  92     1286    956   2246     10    187    450       C
ATOM   1462  CG2 VAL A  92      16.668   5.092  -9.339  1.00  9.83           C  0.033
ANISOU 1462  CG2 VAL A  92     1118    923   1694    -61    254    353       C
ATOM   1463  H   VAL A  92      15.373   3.054  -7.765  1.00 11.17           H  0.035
ATOM   1464  HA  VAL A  92      17.754   4.012  -7.290  1.00 10.75           H  0.034
ATOM   1465  HB  VAL A  92      15.358   5.382  -7.879  1.00 12.30           H  0.037
ATOM   1466 HG11 VAL A  92      16.742   7.346  -8.120  1.00 14.18           H  0.039
ATOM   1467 HG12 VAL A  92      16.516   6.901  -6.612  1.00 14.18           H  0.039
ATOM   1468 HG13 VAL A  92      17.881   6.552  -7.347  1.00 14.18           H  0.039
ATOM   1469 HG21 VAL A  92      16.532   5.910  -9.843  1.00 11.80           H  0.036
ATOM   1470 HG22 VAL A  92      17.598   4.820  -9.394  1.00 11.80           H  0.036
ATOM   1471 HG23 VAL A  92      16.097   4.389  -9.688  1.00 11.80           H  0.036
ATOM   1472  N   ASN A  93      15.640   4.557  -4.956  1.00 11.01           N  0.035
ANISOU 1472  N   ASN A  93     1289   1068   1826    144    678     40       N
ATOM   1473  CA  ASN A  93      15.579   5.000  -3.570  1.00 11.41           C  0.035
ANISOU 1473  CA  ASN A  93     1213   1258   1863    223    775   -136       C
ATOM   1474  C   ASN A  93      16.394   4.074  -2.682  1.00  9.32           C  0.032
ANISOU 1474  C   ASN A  93     1147    988   1407     12    467    -70       C
ATOM   1475  O   ASN A  93      17.032   4.502  -1.710  1.00 10.72           O  0.034
ANISOU 1475  O   ASN A  93     1289   1124   1658     75    323    236       O
ATOM   1476  CB  ASN A  93      14.124   5.036  -3.104  1.00 14.00           C  0.039
ANISOU 1476  CB  ASN A  93     1386   1712   2221    471    802   -133       C
ATOM   1477  CG  ASN A  93      13.324   6.167  -3.740  1.00 17.98           C  0.044
ANISOU 1477  CG  ASN A  93     1590   2232   3008    502    676     -7       C
ATOM   1478  OD1 ASN A  93      13.865   7.043  -4.377  1.00 17.73           O  0.044
ANISOU 1478  OD1 ASN A  93     1933   2279   2525    369    369    -85       O
ATOM   1479  ND2 ASN A  93      12.007   6.131  -3.552  1.00 20.34           N  0.047
ANISOU 1479  ND2 ASN A  93     1767   2567   3396    822    827   -204       N
ATOM   1480  H   ASN A  93      14.891   4.308  -5.299  1.00 13.21           H  0.038
ATOM   1481  HA  ASN A  93      15.942   5.897  -3.501  1.00 13.69           H  0.039
ATOM   1482  HB2 ASN A  93      13.697   4.198  -3.339  1.00 16.80           H  0.043
ATOM   1483  HB3 ASN A  93      14.105   5.160  -2.142  1.00 16.80           H  0.043
ATOM   1484 HD21 ASN A  93      11.507   6.745  -3.889  1.00 24.41           H  0.052
ATOM   1485 HD22 ASN A  93      11.655   5.495  -3.093  1.00 24.41           H  0.052
ATOM   1486  N   CYS A  94      16.376   2.777  -2.986  1.00  9.86           N  0.033
ANISOU 1486  N   CYS A  94     1109    697   1939    -67    324   -299       N
ATOM   1487  CA  CYS A  94      17.162   1.863  -2.184  1.00  9.83           C  0.033
ANISOU 1487  CA  CYS A  94     1178   1040   1516   -121     84   -321       C
ATOM   1488  C   CYS A  94      18.642   2.071  -2.429  1.00  9.12           C  0.032
ANISOU 1488  C   CYS A  94     1035   1047   1382   -243    180    100       C
ATOM   1489  O   CYS A  94      19.425   2.037  -1.478  1.00 10.57           O  0.034
ANISOU 1489  O   CYS A  94      886   1355   1775   -533     60    -29       O
ATOM   1490  CB  CYS A  94      16.755   0.416  -2.498  1.00 10.38           C  0.034
ANISOU 1490  CB  CYS A  94     1315   1212   1418   -184     68   -229       C
ATOM   1491  SG  CYS A  94      17.443  -0.830  -1.379  1.00 10.46           S  0.034
ANISOU 1491  SG  CYS A  94     1204   1228   1541   -179    119     29       S
ATOM   1492  H   CYS A  94      15.931   2.419  -3.629  1.00 11.83           H  0.036
ATOM   1493  HA  CYS A  94      16.986   2.025  -1.244  1.00 11.79           H  0.036
ATOM   1494  HB2 CYS A  94      15.788   0.351  -2.447  1.00 12.46           H  0.037
ATOM   1495  HB3 CYS A  94      17.055   0.199  -3.395  1.00 12.46           H  0.037
ATOM   1496  N   ALA A  95      19.017   2.204  -3.702  1.00  9.51           N  0.032
ANISOU 1496  N   ALA A  95     1015    988   1610   -208    180    114       N
ATOM   1497  CA  ALA A  95      20.411   2.472  -4.067  1.00  8.81           C  0.031
ANISOU 1497  CA  ALA A  95      781    970   1597   -168     42   -294       C
ATOM   1498  C   ALA A  95      20.963   3.694  -3.337  1.00  9.09           C  0.032
ANISOU 1498  C   ALA A  95     1021    917   1515    -83    238   -313       C
ATOM   1499  O   ALA A  95      22.122   3.683  -2.879  1.00  9.85           O  0.033
ANISOU 1499  O   ALA A  95      942    919   1882    -58    126    -63       O
ATOM   1500  CB  ALA A  95      20.568   2.624  -5.576  1.00  8.92           C  0.031
ANISOU 1500  CB  ALA A  95      768   1108   1513    283    -99   -217       C
ATOM   1501  H   ALA A  95      18.485   2.145  -4.376  1.00 11.41           H  0.035
ATOM   1502  HA  ALA A  95      20.940   1.703  -3.801  1.00 10.57           H  0.034
ATOM   1503  HB1 ALA A  95      21.507   2.546  -5.806  1.00 10.71           H  0.034
ATOM   1504  HB2 ALA A  95      20.060   1.926  -6.018  1.00 10.71           H  0.034
ATOM   1505  HB3 ALA A  95      20.234   3.495  -5.842  1.00 10.71           H  0.034
ATOM   1506  N   LYS A  96      20.148   4.756  -3.211  1.00  8.88           N  0.031
ANISOU 1506  N   LYS A  96      786   1083   1507      7    424   -312       N
ATOM   1507  CA  LYS A  96      20.583   5.942  -2.447  1.00  8.96           C  0.031
ANISOU 1507  CA  LYS A  96     1030    929   1445    -93    306   -281       C
ATOM   1508  C   LYS A  96      20.898   5.605  -0.998  1.00  9.58           C  0.032
```

```
ANISOU 1508  C   LYS A  96     1120    992   1527   -270    267   -298       C
ATOM   1509  O   LYS A  96      21.870   6.102  -0.439  1.00  9.72           O  0.033
ANISOU 1509  O   LYS A  96     1158   1131   1405   -444    404   -127       O
ATOM   1510  CB  LYS A  96      19.530   7.038  -2.548  1.00  9.71           C  0.033
ANISOU 1510  CB  LYS A  96     1085    930   1675    165    224   -259       C
ATOM   1511  CG  LYS A  96      19.424   7.648  -3.939  1.00 10.36           C  0.034
ANISOU 1511  CG  LYS A  96     1026    876   2036    186     50    139       C
ATOM   1512  CD  LYS A  96      18.266   8.644  -4.038  1.00 12.20           C  0.037
ANISOU 1512  CD  LYS A  96     1306    847   2481    190      6    -94       C
ATOM   1513  CE  LYS A  96      18.282   9.332  -5.393  1.00 13.33           C  0.038
ANISOU 1513  CE  LYS A  96     1362   1047   2657    399    -79    -86       C
ATOM   1514  NZ  LYS A  96      17.083  10.269  -5.512  1.00 16.53           N  0.043
ANISOU 1514  NZ  LYS A  96     1691   1255   3337    451    244    -58       N
ATOM   1515  H   LYS A  96      19.360   4.815  -3.548  1.00 10.66           H  0.034
ATOM   1516  HA  LYS A  96      21.404   6.280  -2.839  1.00 10.75           H  0.034
ATOM   1517  HB2 LYS A  96      18.664   6.663  -2.321  1.00 11.65           H  0.036
ATOM   1518  HB3 LYS A  96      19.757   7.749  -1.928  1.00 11.65           H  0.036
ATOM   1519  HG2 LYS A  96      20.247   8.118  -4.145  1.00 12.44           H  0.037
ATOM   1520  HG3 LYS A  96      19.273   6.942  -4.587  1.00 12.44           H  0.037
ATOM   1521  HD2 LYS A  96      17.423   8.175  -3.938  1.00 14.64           H  0.040
ATOM   1522  HD3 LYS A  96      18.355   9.318  -3.346  1.00 14.64           H  0.040
ATOM   1523  HE2 LYS A  96      19.095   9.853  -5.487  1.00 16.00           H  0.042
ATOM   1524  HE3 LYS A  96      18.230   8.668  -6.099  1.00 16.00           H  0.042
ATOM   1525  HZ1 LYS A  96      17.042  10.608  -6.334  1.00 19.84           H  0.047
ATOM   1526  HZ2 LYS A  96      16.331   9.823  -5.346  1.00 19.84           H  0.047
ATOM   1527  HZ3 LYS A  96      17.159  10.936  -4.927  1.00 19.84           H  0.047
ATOM   1528  N   LYS A  97      20.110   4.732  -0.370  1.00  9.55           N  0.032
ANISOU 1528  N   LYS A  97     1126   1055   1446   -125    262   -158       N
ATOM   1529  CA  LYS A  97      20.437   4.318   0.990  1.00  8.71           C  0.031
ANISOU 1529  CA  LYS A  97     1281   1107    920   -444    316    -84       C
ATOM   1530  C   LYS A  97      21.721   3.482   1.016  1.00  8.84           C  0.031
ANISOU 1530  C   LYS A  97     1285   1207    866   -369    141    -55       C
ATOM   1531  O   LYS A  97      22.595   3.692   1.857  1.00  9.84           O  0.033
ANISOU 1531  O   LYS A  97     1473   1288    979   -505    294   -272       O
ATOM   1532  CB  LYS A  97      19.247   3.561   1.574  1.00 11.18           C  0.035
ANISOU 1532  CB  LYS A  97     1658   1472   1117   -824    132    238       C
ATOM   1533  CG  LYS A  97      19.497   3.057   2.951  1.00 18.13           C  0.045
ANISOU 1533  CG  LYS A  97     2155   2185   2548   -861    593    272       C
ATOM   1534  CD  LYS A  97      18.184   2.773   3.716  1.00 23.10           C  0.050
ANISOU 1534  CD  LYS A  97     2629   2636   3511   -899   1022    295       C
ATOM   1535  CE  LYS A  97      17.367   4.050   3.900  1.00 28.51           C  0.056
ANISOU 1535  CE  LYS A  97     3059   3134   4638   -745    902    248       C
ATOM   1536  NZ  LYS A  97      16.019   3.847   4.480  1.00 31.82           N  0.059
ANISOU 1536  NZ  LYS A  97     3293   3392   5406   -793    754    203       N
ATOM   1537  H   LYS A  97      19.401   4.375  -0.700  1.00 11.45           H  0.035
ATOM   1538  HA  LYS A  97      20.597   5.093   1.550  1.00 10.45           H  0.034
ATOM   1539  HB2 LYS A  97      18.482   4.156   1.608  1.00 13.41           H  0.038
ATOM   1540  HB3 LYS A  97      19.050   2.799   1.007  1.00 13.41           H  0.038
ATOM   1541  HG2 LYS A  97      20.002   2.231   2.901  1.00 21.75           H  0.049
ATOM   1542  HG3 LYS A  97      19.998   3.723   3.448  1.00 21.75           H  0.049
ATOM   1543  HD2 LYS A  97      17.649   2.137   3.214  1.00 27.72           H  0.055
ATOM   1544  HD3 LYS A  97      18.392   2.413   4.592  1.00 27.72           H  0.055
ATOM   1545  HE2 LYS A  97      17.853   4.643   4.494  1.00 34.21           H  0.061
ATOM   1546  HE3 LYS A  97      17.251   4.470   3.033  1.00 34.21           H  0.061
ATOM   1547  HZ1 LYS A  97      15.603   4.630   4.564  1.00 38.19           H  0.065
ATOM   1548  HZ2 LYS A  97      15.536   3.319   3.950  1.00 38.19           H  0.065
ATOM   1549  HZ3 LYS A  97      16.086   3.468   5.282  1.00 38.19           H  0.065
ATOM   1550  N   ILE A  98      21.878   2.569   0.050  1.00  8.67           N  0.031
ANISOU 1550  N   ILE A  98     1110   1048   1137   -174     60   -175       N
ATOM   1551  CA  ILE A  98      23.048   1.690   0.016  1.00  9.25           C  0.032
ANISOU 1551  CA  ILE A  98     1059   1104   1349   -274    -31   -106       C
ATOM   1552  C   ILE A  98      24.318   2.509  -0.161  1.00  8.82           C  0.031
ANISOU 1552  C   ILE A  98      877   1076   1396   -162      7     -4       C
ATOM   1553  O   ILE A  98      25.298   2.345   0.580  1.00  9.54           O  0.032
ANISOU 1553  O   ILE A  98     1308   1254   1062     90    231   -142       O
ATOM   1554  CB  ILE A  98      22.904   0.662  -1.120  1.00  7.89           C  0.029
ANISOU 1554  CB  ILE A  98      944   1031   1023   -259    -57      1       C
ATOM   1555  CG1 ILE A  98      21.736  -0.303  -0.863  1.00  9.67           C  0.033
ANISOU 1555  CG1 ILE A  98      918   1241   1516   -595   -107     80       C
ATOM   1556  CG2 ILE A  98      24.198  -0.148  -1.327  1.00  9.03           C  0.032
ANISOU 1556  CG2 ILE A  98      858   1107   1466   -206    -99     88       C
ATOM   1557  CD1 ILE A  98      21.305  -1.118  -2.111  1.00 10.06           C  0.033
ANISOU 1557  CD1 ILE A  98      938   1232   1653   -590    180   -145       C
ATOM   1558  H   ILE A  98      21.323   2.440  -0.594  1.00 10.41           H  0.034
ATOM   1559  HA  ILE A  98      23.102   1.218   0.861  1.00 11.09           H  0.035
ATOM   1560  HB  ILE A  98      22.721   1.173  -1.924  1.00  9.47           H  0.032
ATOM   1561 HG12 ILE A  98      22.000  -0.934  -0.176  1.00 11.61           H  0.036
ATOM   1562 HG13 ILE A  98      20.969   0.210  -0.566  1.00 11.61           H  0.036
```

```
ATOM   1563 HG21 ILE A  98      24.056  -0.793  -2.037  1.00 10.84           H  0.035
ATOM   1564 HG22 ILE A  98      24.915   0.459  -1.570  1.00 10.84           H  0.035
ATOM   1565 HG23 ILE A  98      24.419  -0.607  -0.502  1.00 10.84           H  0.035
ATOM   1566 HD11 ILE A  98      20.484  -1.594  -1.909  1.00 12.07           H  0.036
ATOM   1567 HD12 ILE A  98      21.160  -0.509  -2.852  1.00 12.07           H  0.036
ATOM   1568 HD13 ILE A  98      22.006  -1.750  -2.334  1.00 12.07           H  0.036
ATOM   1569  N   VAL A  99      24.312   3.432  -1.124  1.00  8.13           N  0.030
ANISOU 1569  N   VAL A  99     898   1191   1001   -206    -82     21       N
ATOM   1570  CA  VAL A  99      25.530   4.174  -1.443  1.00  7.57           C  0.029
ANISOU 1570  CA  VAL A  99    1020   1016    840   -127    -33   -294       C
ATOM   1571  C   VAL A  99      25.920   5.134  -0.331  1.00  8.35           C  0.030
ANISOU 1571  C   VAL A  99    1006   1232    936   -194      6   -262       C
ATOM   1572  O   VAL A  99      27.044   5.641  -0.337  1.00  9.12           O  0.032
ANISOU 1572  O   VAL A  99    1100   1261   1104   -350    264    -63       O
ATOM   1573  CB  VAL A  99      25.353   4.861  -2.809  1.00  7.50           C  0.029
ANISOU 1573  CB  VAL A  99    1185    732    931      8     10   -199       C
ATOM   1574  CG1 VAL A  99      24.475   6.099  -2.721  1.00  9.27           C  0.032
ANISOU 1574  CG1 VAL A  99    1444    750   1328    102    406   -331       C
ATOM   1575  CG2 VAL A  99      26.707   5.255  -3.397  1.00  9.15           C  0.032
ANISOU 1575  CG2 VAL A  99    1386    767   1322    -38    367     83       C
ATOM   1576  H   VAL A  99      23.627   3.642  -1.600  1.00  9.76           H  0.033
ATOM   1577  HA  VAL A  99      26.286   3.573  -1.534  1.00  9.08           H  0.032
ATOM   1578  HB  VAL A  99      24.917   4.221  -3.392  1.00  9.00           H  0.031
ATOM   1579 HG11 VAL A  99      24.351   6.462  -3.612  1.00 11.12           H  0.035
ATOM   1580 HG12 VAL A  99      23.617   5.851  -2.342  1.00 11.12           H  0.035
ATOM   1581 HG13 VAL A  99      24.910   6.755  -2.154  1.00 11.12           H  0.035
ATOM   1582 HG21 VAL A  99      26.580   5.554  -4.311  1.00 10.97           H  0.035
ATOM   1583 HG22 VAL A  99      27.086   5.972  -2.865  1.00 10.97           H  0.035
ATOM   1584 HG23 VAL A  99      27.295   4.484  -3.380  1.00 10.97           H  0.035
ATOM   1585  N   SER A 100      25.019   5.372   0.624  1.00 10.24           N  0.034
ANISOU 1585  N   SER A 100    1279   1537   1076   -334    378   -498       N
ATOM   1586  CA  SER A 100      25.243   6.330   1.692  1.00 12.12           C  0.037
ANISOU 1586  CA  SER A 100    1488   1707   1411   -261    442   -431       C
ATOM   1587  C   SER A 100      25.755   5.672   2.949  1.00 14.16           C  0.039
ANISOU 1587  C   SER A 100    1728   2060   1591   -242    557   -334       C
ATOM   1588  O   SER A 100      26.049   6.369   3.926  1.00 16.45           O  0.043
ANISOU 1588  O   SER A 100    1894   2332   2026   -283    476   -550       O
ATOM   1589  CB  SER A 100      23.951   7.094   2.014  1.00 12.95           C  0.038
ANISOU 1589  CB  SER A 100    1520   1719   1682   -230    177   -467       C
ATOM   1590  OG  SER A 100      23.439   7.804   0.906  1.00 14.30           O  0.040
ANISOU 1590  OG  SER A 100    1867   1718   1849   -324    371   -549       O
ATOM   1591  H   SER A 100      24.256   4.979   0.671  1.00 12.29           H  0.037
ATOM   1592  HA  SER A 100      25.898   6.978   1.391  1.00 14.55           H  0.040
ATOM   1593  HB2 SER A 100      23.281   6.457   2.306  1.00 15.54           H  0.041
ATOM   1594  HB3 SER A 100      24.138   7.728   2.724  1.00 15.54           H  0.041
ATOM   1595  HG  SER A 100      23.259   7.271   0.282  1.00 17.16           H  0.043
ATOM   1596  N   ASP A 101      25.875   4.354   2.930  1.00 13.09           N  0.038
ANISOU 1596  N   ASP A 101    1793   1973   1209   -110    787    175       N
ATOM   1597  CA  ASP A 101      26.126   3.590   4.173  1.00 15.17           C  0.041
ANISOU 1597  CA  ASP A 101    2073   2232   1458   -116    523    188       C
ATOM   1598  C   ASP A 101      27.585   3.383   4.521  1.00 15.91           C  0.042
ANISOU 1598  C   ASP A 101    2065   2323   1657   -195    243    455       C
ATOM   1599  O   ASP A 101      27.827   2.681   5.516  1.00 17.04           O  0.043
ANISOU 1599  O   ASP A 101    2268   2228   1978   -213    351    608       O
ATOM   1600  CB  ASP A 101      25.385   2.274   4.096  1.00 16.07           C  0.042
ANISOU 1600  CB  ASP A 101    2336   2251   1519   -217    438    564       C
ATOM   1601  CG  ASP A 101      24.841   1.898   5.446  1.00 20.75           C  0.048
ANISOU 1601  CG  ASP A 101    2749   2385   2750     -7    702    207       C
ATOM   1602  OD1 ASP A 101      24.275   2.765   6.130  1.00 26.15           O  0.054
ANISOU 1602  OD1 ASP A 101    3329   2727   3879    191    741    232       O
ATOM   1603  OD2 ASP A 101      25.072   0.787   5.810  1.00 18.46           O  0.045
ANISOU 1603  OD2 ASP A 101    2629   2055   2330   -449    382    205       O
ATOM   1604  H   ASP A 101      25.808   3.778   2.071  1.00 15.71           H  0.042
ATOM   1605  HA  ASP A 101      25.684   4.165   4.984  1.00 18.20           H  0.045
ATOM   1606  HB2 ASP A 101      24.568   2.346   3.378  1.00 19.29           H  0.046
ATOM   1607  HB3 ASP A 101      26.071   1.496   3.771  1.00 19.29           H  0.046
ATOM   1608  N   GLY A 102      28.508   3.961   3.780  1.00 14.99           N  0.041
ANISOU 1608  N   GLY A 102    1953   2320   1421   -181    248    100       N
ATOM   1609  CA  GLY A 102      29.888   3.989   4.230  1.00 13.61           C  0.039
ANISOU 1609  CA  GLY A 102    1842   2257   1074   -419     37   -134       C
ATOM   1610  C   GLY A 102      30.940   3.734   3.169  1.00 10.58           C  0.034
ANISOU 1610  C   GLY A 102    1510   1893    618   -447     61   -280       C
ATOM   1611  O   GLY A 102      32.034   4.328   3.177  1.00 13.05           O  0.038
ANISOU 1611  O   GLY A 102    1614   2239   1104   -784    216    -50       O
ATOM   1612  H   GLY A 102      28.367   4.330   3.016  1.00 17.98           H  0.044
ATOM   1613  HA2 GLY A 102      30.067   4.862   4.612  1.00 16.34           H  0.042
ATOM   1614  HA3 GLY A 102      30.000   3.314   4.917  1.00 16.34           H  0.042
ATOM   1615  N   ASN A 103      30.660   2.769   2.305  1.00  9.25           N  0.032
```

```
ANISOU 1615  N   ASN A 103     1415   1604    496   -433     73    -61       N
ATOM   1616  CA  ASN A 103      31.619   2.329   1.297  1.00  9.69           C  0.033
ANISOU 1616  CA  ASN A 103     1447   1548    687   -289    161    172       C
ATOM   1617  C   ASN A 103      31.235   2.809  -0.098  1.00  8.47           C  0.031
ANISOU 1617  C   ASN A 103     1214   1405    598   -126   -196     10       C
ATOM   1618  O   ASN A 103      31.882   2.421  -1.086  1.00  8.93           O  0.031
ANISOU 1618  O   ASN A 103     1265   1248    878   -225   -385   -417       O
ATOM   1619  CB  ASN A 103      31.731   0.810   1.316  1.00 12.99           C  0.038
ANISOU 1619  CB  ASN A 103     1794   1693   1449   -152    137    674       C
ATOM   1620  CG  ASN A 103      32.336   0.314   2.607  1.00 14.47           C  0.040
ANISOU 1620  CG  ASN A 103     1983   1902   1612   -133    -57    565       C
ATOM   1621  OD1 ASN A 103      33.130   1.006   3.248  1.00 15.70           O  0.042
ANISOU 1621  OD1 ASN A 103     2081   2072   1812   -286   -509    872       O
ATOM   1622  ND2 ASN A 103      31.928  -0.856   3.018  1.00 15.94           N  0.042
ANISOU 1622  ND2 ASN A 103     2120   1996   1940    -75      2    483       N
ATOM   1623  H   ASN A 103      29.911   2.347   2.280  1.00 11.10           H  0.035
ATOM   1624  HA  ASN A 103      32.493   2.693   1.511  1.00 11.63           H  0.036
ATOM   1625  HB2 ASN A 103      30.846   0.423   1.224  1.00 15.59           H  0.041
ATOM   1626  HB3 ASN A 103      32.297   0.522   0.583  1.00 15.59           H  0.041
ATOM   1627 HD21 ASN A 103      32.239  -1.188   3.747  1.00 19.13           H  0.046
ATOM   1628 HD22 ASN A 103      31.348  -1.295   2.559  1.00 19.13           H  0.046
ATOM   1629  N   GLY A 104      30.298   3.739  -0.192  1.00  8.34           N  0.030
ANISOU 1629  N   GLY A 104     1119   1333    717   -151    176   -158       N
ATOM   1630  CA  GLY A 104      29.978   4.334  -1.469  1.00  6.82           C  0.027
ANISOU 1630  CA  GLY A 104      946   1118    526   -157    -67   -358       C
ATOM   1631  C   GLY A 104      29.556   3.270  -2.456  1.00  7.02           C  0.028
ANISOU 1631  C   GLY A 104      878    895    894   -142   -348   -339       C
ATOM   1632  O   GLY A 104      28.876   2.298  -2.096  1.00  7.12           O  0.028
ANISOU 1632  O   GLY A 104     1083    685    935    -43    138     71       O
ATOM   1633  H   GLY A 104      29.836   4.040   0.468  1.00 10.01           H  0.033
ATOM   1634  HA2 GLY A 104      29.253   4.969  -1.363  1.00  8.18           H  0.030
ATOM   1635  HA3 GLY A 104      30.755   4.798  -1.820  1.00  8.18           H  0.030
ATOM   1636  N   MET A 105      29.974   3.436  -3.714  1.00  6.50           N  0.027
ANISOU 1636  N   MET A 105      757    723    989   -133   -153   -332       N
ATOM   1637  CA  MET A 105      29.567   2.482  -4.761  1.00  5.67           C  0.025
ANISOU 1637  CA  MET A 105      835    578    743    -19   -219   -192       C
ATOM   1638  C   MET A 105      30.335   1.178  -4.724  1.00  5.64           C  0.025
ANISOU 1638  C   MET A 105      851    505    788    -59    -63   -224       C
ATOM   1639  O   MET A 105      29.994   0.271  -5.487  1.00  5.52           O  0.025
ANISOU 1639  O   MET A 105      809    564    726    147    115   -148       O
ATOM   1640  CB  MET A 105      29.592   3.112  -6.164  1.00  6.72           C  0.027
ANISOU 1640  CB  MET A 105     1013    456   1087    167     10     24       C
ATOM   1641  CG  MET A 105      28.445   4.113  -6.369  1.00  5.45           C  0.024
ANISOU 1641  CG  MET A 105      750    507    813    103   -219    122       C
ATOM   1642  SD  MET A 105      28.206   4.598  -8.110  1.00  6.87           S  0.027
ANISOU 1642  SD  MET A 105      797    862    953    -64    -68    -47       S
ATOM   1643  CE  MET A 105      27.482   3.058  -8.670  1.00  5.96           C  0.026
ANISOU 1643  CE  MET A 105      860    867    536   -258   -164    -64       C
ATOM   1644  H   MET A 105      30.482   4.076  -3.983  1.00  7.80           H  0.029
ATOM   1645  HA  MET A 105      28.633   2.272  -4.603  1.00  6.81           H  0.027
ATOM   1646  HB2 MET A 105      30.431   3.583  -6.286  1.00  8.07           H  0.030
ATOM   1647  HB3 MET A 105      29.506   2.411  -6.829  1.00  8.07           H  0.030
ATOM   1648  HG2 MET A 105      27.619   3.711  -6.057  1.00  6.54           H  0.027
ATOM   1649  HG3 MET A 105      28.636   4.917  -5.861  1.00  6.54           H  0.027
ATOM   1650  HE1 MET A 105      27.460   3.053  -9.640  1.00  7.15           H  0.028
ATOM   1651  HE2 MET A 105      28.022   2.319  -8.348  1.00  7.15           H  0.028
ATOM   1652  HE3 MET A 105      26.581   2.986  -8.318  1.00  7.15           H  0.028
ATOM   1653  N   ASN A 106      31.329   1.038  -3.837  1.00  5.94           N  0.026
ANISOU 1653  N   ASN A 106      812    562    882   -133   -116    -80       N
ATOM   1654  CA  ASN A 106      31.971  -0.253  -3.648  1.00  7.63           C  0.029
ANISOU 1654  CA  ASN A 106      954    644   1299   -145    -69     45       C
ATOM   1655  C   ASN A 106      31.002  -1.304  -3.125  1.00  8.19           C  0.030
ANISOU 1655  C   ASN A 106      904    860   1347   -308    104    -51       C
ATOM   1656  O   ASN A 106      31.306  -2.493  -3.229  1.00  9.72           O  0.033
ANISOU 1656  O   ASN A 106      960    939   1797   -217    261    121       O
ATOM   1657  CB  ASN A 106      33.131  -0.118  -2.692  1.00  9.01           C  0.031
ANISOU 1657  CB  ASN A 106     1106    666   1650    -78     29    195       C
ATOM   1658  CG  ASN A 106      34.217   0.794  -3.226  1.00  9.06           C  0.032
ANISOU 1658  CG  ASN A 106     1203    869   1371   -118      9    237       C
ATOM   1659  OD1 ASN A 106      34.885   0.464  -4.197  1.00  7.35           O  0.028
ANISOU 1659  OD1 ASN A 106     1030    831    934   -195   -117    -52       O
ATOM   1660  ND2 ASN A 106      34.335   1.986  -2.629  1.00  9.44           N  0.032
ANISOU 1660  ND2 ASN A 106     1412    890   1286   -433     13    172       N
ATOM   1661  H   ASN A 106      31.641   1.670  -3.344  1.00  7.13           H  0.028
ATOM   1662  HA  ASN A 106      32.320  -0.556  -4.500  1.00  9.15           H  0.032
ATOM   1663  HB2 ASN A 106      32.811   0.252  -1.854  1.00 10.81           H  0.034
ATOM   1664  HB3 ASN A 106      33.520  -0.994  -2.540  1.00 10.81           H  0.034
ATOM   1665 HD21 ASN A 106      34.936   2.542  -2.893  1.00 11.33           H  0.035
```

```
ATOM   1666 HD22 ASN A 106      33.810   2.196  -1.980  1.00 11.33           H  0.035
ATOM   1667  N   ALA A 107      29.824  -0.881  -2.665  1.00  7.42           N  0.029
ANISOU 1667  N   ALA A 107      947    935    939   -282    125   -120       N
ATOM   1668  CA  ALA A 107      28.763  -1.818  -2.313  1.00  6.35           C  0.026
ANISOU 1668  CA  ALA A 107     1093    843    475   -197    214     45       C
ATOM   1669  C   ALA A 107      28.352  -2.679  -3.493  1.00  6.68           C  0.027
ANISOU 1669  C   ALA A 107     1185    544    811   -303    296     31       C
ATOM   1670  O   ALA A 107      27.811  -3.785  -3.291  1.00 12.07           O  0.036
ANISOU 1670  O   ALA A 107     1947    772   1865   -402    197     45       O
ATOM   1671  CB  ALA A 107      27.574  -1.005  -1.852  1.00  7.50           C  0.029
ANISOU 1671  CB  ALA A 107     1038   1049    762   -193    497   -145       C
ATOM   1672  H   ALA A 107      29.615  -0.056  -2.546  1.00  8.91           H  0.031
ATOM   1673  HA  ALA A 107      29.060  -2.411  -1.605  1.00  7.62           H  0.029
ATOM   1674  HB1 ALA A 107      26.800  -1.587  -1.788  1.00  9.00           H  0.031
ATOM   1675  HB2 ALA A 107      27.771  -0.621  -0.984  1.00  9.00           H  0.031
ATOM   1676  HB3 ALA A 107      27.405  -0.299  -2.496  1.00  9.00           H  0.031
ATOM   1677  N   TRP A 108      28.583  -2.197  -4.711  1.00  6.46           N  0.027
ANISOU 1677  N   TRP A 108     1067    668    721    -65    133   -106       N
ATOM   1678  CA  TRP A 108      28.332  -2.943  -5.951  1.00  7.07           C  0.028
ANISOU 1678  CA  TRP A 108     1087    666    933    -16    351   -174       C
ATOM   1679  C   TRP A 108      29.674  -3.472  -6.451  1.00  7.61           C  0.029
ANISOU 1679  C   TRP A 108     1271    598   1023    221    425    128       C
ATOM   1680  O   TRP A 108      30.485  -2.725  -7.003  1.00  8.07           O  0.030
ANISOU 1680  O   TRP A 108     1122    729   1217    -19    230    -52       O
ATOM   1681  CB  TRP A 108      27.630  -2.073  -6.995  1.00  6.35           C  0.026
ANISOU 1681  CB  TRP A 108     1013    816    585    -20    221   -211       C
ATOM   1682  CG  TRP A 108      26.220  -1.786  -6.654  1.00  6.46           C  0.027
ANISOU 1682  CG  TRP A 108      797    764    894     17    232    -59       C
ATOM   1683  CD1 TRP A 108      25.141  -2.605  -6.870  1.00  7.31           C  0.028
ANISOU 1683  CD1 TRP A 108      769    679   1329    -52    117   -247       C
ATOM   1684  CD2 TRP A 108      25.724  -0.629  -5.973  1.00  6.28           C  0.026
ANISOU 1684  CD2 TRP A 108      710    776    900    -74     27    -70       C
ATOM   1685  NE1 TRP A 108      24.002  -2.015  -6.359  1.00  7.59           N  0.029
ANISOU 1685  NE1 TRP A 108      798    800   1286   -269    277   -225       N
ATOM   1686  CE2 TRP A 108      24.328  -0.786  -5.832  1.00  6.74           C  0.027
ANISOU 1686  CE2 TRP A 108      824    902    837   -226     84    -62       C
ATOM   1687  CE3 TRP A 108      26.321   0.527  -5.481  1.00  7.80           C  0.029
ANISOU 1687  CE3 TRP A 108      858   1013   1092     16     17   -378       C
ATOM   1688  CZ2 TRP A 108      23.544   0.145  -5.200  1.00  6.64           C  0.027
ANISOU 1688  CZ2 TRP A 108      807    955    761     -3   -295   -124       C
ATOM   1689  CZ3 TRP A 108      25.506   1.492  -4.891  1.00  7.38           C  0.028
ANISOU 1689  CZ3 TRP A 108      811    788   1206    101   -171   -198       C
ATOM   1690  CH2 TRP A 108      24.142   1.287  -4.744  1.00  7.29           C  0.028
ANISOU 1690  CH2 TRP A 108      760    912   1097    -46   -376   -386       C
ATOM   1691  H   TRP A 108      28.897  -1.410  -4.855  1.00  7.76           H  0.029
ATOM   1692  HA  TRP A 108      27.748  -3.697  -5.771  1.00  8.48           H  0.031
ATOM   1693  HB2 TRP A 108      28.099  -1.227  -7.066  1.00  7.62           H  0.029
ATOM   1694  HB3 TRP A 108      27.644  -2.532  -7.849  1.00  7.62           H  0.029
ATOM   1695  HD1 TRP A 108      25.172  -3.431  -7.295  1.00  8.77           H  0.031
ATOM   1696  HE1 TRP A 108      23.214  -2.360  -6.368  1.00  9.11           H  0.032
ATOM   1697  HE3 TRP A 108      27.240   0.652  -5.544  1.00  9.36           H  0.032
ATOM   1698  HZ2 TRP A 108      22.632   0.004  -5.085  1.00  7.97           H  0.030
ATOM   1699  HZ3 TRP A 108      25.883   2.288  -4.592  1.00  8.86           H  0.031
ATOM   1700  HH2 TRP A 108      23.625   1.937  -4.328  1.00  8.75           H  0.031
ATOM   1701  N   VAL A 109      29.893  -4.773  -6.267  1.00 10.67           N  0.034
ANISOU 1701  N   VAL A 109     1533    801   1719    334    808    400       N
ATOM   1702  CA  VAL A 109      31.174  -5.374  -6.603  1.00 12.11           C  0.036
ANISOU 1702  CA  VAL A 109     1877    876   1847    417    839    378       C
ATOM   1703  C   VAL A 109      31.476  -5.189  -8.080  1.00 10.83           C  0.035
ANISOU 1703  C   VAL A 109     1694    636   1787    156    521    257       C
ATOM   1704  O   VAL A 109      32.634  -4.901  -8.457  1.00 11.41           O  0.035
ANISOU 1704  O   VAL A 109     1713    706   1917    294    512    282       O
ATOM   1705  CB  VAL A 109      31.103  -6.864  -6.189  1.00 14.77           C  0.040
ANISOU 1705  CB  VAL A 109     2194   1204   2214    624    938    609       C
ATOM   1706  CG1 VAL A 109      32.280  -7.653  -6.742  1.00 17.57           C  0.044
ANISOU 1706  CG1 VAL A 109     2373   1609   2695    581    736   1032       C
ATOM   1707  CG2 VAL A 109      31.047  -6.973  -4.667  1.00 19.42           C  0.046
ANISOU 1707  CG2 VAL A 109     2631   1546   3203    826    821    740       C
ATOM   1708  H   VAL A 109      29.316  -5.326  -5.949  1.00 12.80           H  0.038
ATOM   1709  HA  VAL A 109      31.899  -4.942  -6.124  1.00 14.53           H  0.040
ATOM   1710  HB  VAL A 109      30.298  -7.254  -6.564  1.00 17.72           H  0.044
ATOM   1711 HG11 VAL A 109      32.211  -8.573  -6.444  1.00 21.09           H  0.048
ATOM   1712 HG12 VAL A 109      32.258  -7.616  -7.711  1.00 21.09           H  0.048
ATOM   1713 HG13 VAL A 109      33.105  -7.260  -6.416  1.00 21.09           H  0.048
ATOM   1714 HG21 VAL A 109      31.009  -7.910  -4.419  1.00 23.31           H  0.051
ATOM   1715 HG22 VAL A 109      31.841  -6.563  -4.292  1.00 23.31           H  0.051
ATOM   1716 HG23 VAL A 109      30.254  -6.514  -4.347  1.00 23.31           H  0.051
ATOM   1717  N   ALA A 110      30.451  -5.271  -8.922  1.00 10.21           N  0.034
```

```
ANISOU 1717  N   ALA A 110     1560    564   1756     87    503    142       N
ATOM   1718  CA  ALA A 110      30.638  -5.055 -10.345  1.00  9.92           C  0.033
ANISOU 1718  CA  ALA A 110     1425    618   1727      9    522    114       C
ATOM   1719  C   ALA A 110      31.062  -3.617 -10.616  1.00  7.11           C  0.028
ANISOU 1719  C   ALA A 110     1014    544   1144     78    393     45       C
ATOM   1720  O   ALA A 110      31.847  -3.361 -11.540  1.00  8.35           O  0.030
ANISOU 1720  O   ALA A 110      963    827   1382     61    192   -120       O
ATOM   1721  CB  ALA A 110      29.395  -5.436 -11.142  1.00 12.25           C  0.037
ANISOU 1721  CB  ALA A 110     1576    863   2216     76    317   -237       C
ATOM   1722  H   ALA A 110      29.641  -5.449  -8.693  1.00 12.26           H  0.037
ATOM   1723  HA  ALA A 110      31.341  -5.645 -10.657  1.00 11.90           H  0.036
ATOM   1724  HB1 ALA A 110      29.490  -5.108 -12.050  1.00 14.70           H  0.040
ATOM   1725  HB2 ALA A 110      29.307  -6.402 -11.146  1.00 14.70           H  0.040
ATOM   1726  HB3 ALA A 110      28.617  -5.034 -10.724  1.00 14.70           H  0.040
ATOM   1727  N   TRP A 111      30.484  -2.653  -9.910  1.00  6.56           N  0.027
ANISOU 1727  N   TRP A 111      982    475   1034    -54    332   -135       N
ATOM   1728  CA  TRP A 111      31.015  -1.293 -10.061  1.00  6.56           C  0.027
ANISOU 1728  CA  TRP A 111     1012    719    760   -151    237   -116       C
ATOM   1729  C   TRP A 111      32.509  -1.230  -9.740  1.00  7.22           C  0.028
ANISOU 1729  C   TRP A 111     1030    921    794     88    392     39       C
ATOM   1730  O   TRP A 111      33.290  -0.660 -10.493  1.00  6.90           O  0.028
ANISOU 1730  O   TRP A 111      826    866    928    105    229    226       O
ATOM   1731  CB  TRP A 111      30.217  -0.278  -9.235  1.00  6.05           C  0.026
ANISOU 1731  CB  TRP A 111      866    809    624    -87    157    -33       C
ATOM   1732  CG  TRP A 111      30.770   1.107  -9.389  1.00  6.05           C  0.026
ANISOU 1732  CG  TRP A 111      885    682    730    -31    -54   -112       C
ATOM   1733  CD1 TRP A 111      30.490   2.011 -10.386  1.00  5.71           C  0.025
ANISOU 1733  CD1 TRP A 111      891    622    657   -104    128   -118       C
ATOM   1734  CD2 TRP A 111      31.745   1.710  -8.562  1.00  5.94           C  0.026
ANISOU 1734  CD2 TRP A 111     1026    778    453     -5   -206     -8       C
ATOM   1735  NE1 TRP A 111      31.205   3.169 -10.176  1.00  4.81           N  0.023
ANISOU 1735  NE1 TRP A 111      712    562    554   -114   -250    -31       N
ATOM   1736  CE2 TRP A 111      32.004   2.986  -9.084  1.00  4.86           C  0.023
ANISOU 1736  CE2 TRP A 111      797    461    590    -16   -237   -223       C
ATOM   1737  CE3 TRP A 111      32.417   1.310  -7.400  1.00  6.34           C  0.026
ANISOU 1737  CE3 TRP A 111      965    658    785    -91   -128    104       C
ATOM   1738  CZ2 TRP A 111      32.885   3.864  -8.486  1.00  5.63           C  0.025
ANISOU 1738  CZ2 TRP A 111      871    659    608    -68   -281   -253       C
ATOM   1739  CZ3 TRP A 111      33.260   2.138  -6.830  1.00  6.43           C  0.027
ANISOU 1739  CZ3 TRP A 111      642    798   1004     32   -238    -31       C
ATOM   1740  CH2 TRP A 111      33.509   3.430  -7.351  1.00  6.04           C  0.026
ANISOU 1740  CH2 TRP A 111      709    720    864    129   -394   -241       C
ATOM   1741  H   TRP A 111      29.824  -2.748  -9.367  1.00  7.87           H  0.029
ATOM   1742  HA  TRP A 111      30.904  -1.039 -10.990  1.00  7.87           H  0.029
ATOM   1743  HB2 TRP A 111      29.295  -0.274  -9.534  1.00  7.26           H  0.028
ATOM   1744  HB3 TRP A 111      30.261  -0.522  -8.297  1.00  7.26           H  0.028
ATOM   1745  HD1 TRP A 111      29.906   1.863 -11.094  1.00  6.85           H  0.027
ATOM   1746  HE1 TRP A 111      31.156   3.884 -10.650  1.00  5.77           H  0.025
ATOM   1747  HE3 TRP A 111      32.267   0.467  -7.036  1.00  7.61           H  0.029
ATOM   1748  HZ2 TRP A 111      33.045   4.709  -8.838  1.00  6.75           H  0.027
ATOM   1749  HZ3 TRP A 111      33.707   1.868  -6.060  1.00  7.72           H  0.029
ATOM   1750  HH2 TRP A 111      34.107   3.993  -6.914  1.00  7.24           H  0.028
ATOM   1751  N   ARG A 112      32.918  -1.716  -8.563  1.00  7.18           N  0.028
ANISOU 1751  N   ARG A 112     1111    876    741    177    145    113       N
ATOM   1752  CA  ARG A 112      34.317  -1.646  -8.210  1.00  7.55           C  0.029
ANISOU 1752  CA  ARG A 112     1255    856    756    323    -99    248       C
ATOM   1753  C   ARG A 112      35.197  -2.338  -9.245  1.00  7.52           C  0.029
ANISOU 1753  C   ARG A 112     1189    912    754    297     90    166       C
ATOM   1754  O   ARG A 112      36.217  -1.783  -9.647  1.00  9.23           O  0.032
ANISOU 1754  O   ARG A 112     1251   1044   1210    526    406    117       O
ATOM   1755  CB  ARG A 112      34.544  -2.254  -6.825  1.00  9.24           C  0.032
ANISOU 1755  CB  ARG A 112     1708   1202    600    563   -128     36       C
ATOM   1756  CG  ARG A 112      36.011  -2.340  -6.429  1.00 11.85           C  0.036
ANISOU 1756  CG  ARG A 112     2119   1473    910    540   -244    334       C
ATOM   1757  CD  ARG A 112      36.145  -2.699  -4.953  1.00 17.28           C  0.044
ANISOU 1757  CD  ARG A 112     2595   1952   2019    419   -128     79       C
ATOM   1758  NE  ARG A 112      35.589  -4.011  -4.672  1.00 19.75           N  0.047
ANISOU 1758  NE  ARG A 112     2869   2196   2437    550     12    182       N
ATOM   1759  CZ  ARG A 112      36.154  -5.174  -4.987  1.00 21.99           C  0.049
ANISOU 1759  CZ  ARG A 112     2937   2542   2875    546     81   -239       C
ATOM   1760  NH1 ARG A 112      37.343  -5.241  -5.590  1.00 20.88           N  0.048
ANISOU 1760  NH1 ARG A 112     2741   2608   2585    770    429   -576       N
ATOM   1761  NH2 ARG A 112      35.518  -6.306  -4.672  1.00 21.51           N  0.049
ANISOU 1761  NH2 ARG A 112     2967   2480   2724    386    -75   -151       N
ATOM   1762  H   ARG A 112      32.410  -2.080  -7.973  1.00  8.62           H  0.031
ATOM   1763  HA  ARG A 112      34.576  -0.712  -8.177  1.00  9.05           H  0.032
ATOM   1764  HB2 ARG A 112      34.091  -1.706  -6.166  1.00 11.08           H  0.035
ATOM   1765  HB3 ARG A 112      34.181  -3.153  -6.816  1.00 11.08           H  0.035
```

```
ATOM   1766  HG2 ARG A 112      36.450  -3.026  -6.955  1.00 14.22           H  0.040
ATOM   1767  HG3 ARG A 112      36.439  -1.482  -6.579  1.00 14.22           H  0.040
ATOM   1768  HD2 ARG A 112      37.084  -2.707  -4.708  1.00 20.74           H  0.048
ATOM   1769  HD3 ARG A 112      35.668  -2.045  -4.419  1.00 20.74           H  0.048
ATOM   1770  HE  ARG A 112      34.830  -4.038  -4.268  1.00 23.70           H  0.051
ATOM   1771 HH11 ARG A 112      37.767  -4.519  -5.786  1.00 25.06           H  0.052
ATOM   1772 HH12 ARG A 112      37.685  -6.006  -5.783  1.00 25.06           H  0.052
ATOM   1773 HH21 ARG A 112      34.758  -6.275  -4.273  1.00 25.81           H  0.053
ATOM   1774 HH22 ARG A 112      35.870  -7.065  -4.869  1.00 25.81           H  0.053
ATOM   1775  N   ASN A 113      34.767  -3.494  -9.749  1.00  7.42           N  0.029
ANISOU 1775  N   ASN A 113     1268    820    733    415    124    -27       N
ATOM   1776  CA  ASN A 113      35.641  -4.264 -10.641  1.00  7.16           C  0.028
ANISOU 1776  CA  ASN A 113     1142    911    668    312     72   -141       C
ATOM   1777  C   ASN A 113      35.602  -3.821 -12.103  1.00  7.60           C  0.029
ANISOU 1777  C   ASN A 113     1058    898    933    234     73    204       C
ATOM   1778  O   ASN A 113      36.533  -4.125 -12.856  1.00  7.18           O  0.028
ANISOU 1778  O   ASN A 113      748   1053    928    136    208     58       O
ATOM   1779  CB  ASN A 113      35.342  -5.737 -10.536  1.00  7.70           C  0.029
ANISOU 1779  CB  ASN A 113     1145   1097    683    350    -31   -267       C
ATOM   1780  CG  ASN A 113      35.786  -6.307  -9.210  1.00  9.81           C  0.033
ANISOU 1780  CG  ASN A 113     1304   1151   1272    376     24   -197       C
ATOM   1781  OD1 ASN A 113      36.789  -5.841  -8.614  1.00 12.33           O  0.037
ANISOU 1781  OD1 ASN A 113     1286   1203   2196    405    -73    -32       O
ATOM   1782  ND2 ASN A 113      35.035  -7.290  -8.727  1.00 10.20           N  0.033
ANISOU 1782  ND2 ASN A 113     1291   1122   1462    376     -9    -16       N
ATOM   1783  H   ASN A 113      33.998  -3.846  -9.596  1.00  8.91           H  0.031
ATOM   1784  HA  ASN A 113      36.553  -4.126 -10.340  1.00  8.59           H  0.031
ATOM   1785  HB2 ASN A 113      34.386  -5.876 -10.621  1.00  9.24           H  0.032
ATOM   1786  HB3 ASN A 113      35.811  -6.210 -11.242  1.00  9.24           H  0.032
ATOM   1787 HD21 ASN A 113      35.237  -7.656  -7.975  1.00 12.24           H  0.037
ATOM   1788 HD22 ASN A 113      34.347  -7.561  -9.166  1.00 12.24           H  0.037
ATOM   1789  N   ARG A 114      34.531  -3.187 -12.561  1.00  7.93           N  0.030
ANISOU 1789  N   ARG A 114     1308    852    855    170     61    239       N
ATOM   1790  CA  ARG A 114      34.395  -2.918 -13.979  1.00  6.58           C  0.027
ANISOU 1790  CA  ARG A 114     1094    722    683     68    -59    412       C
ATOM   1791  C   ARG A 114      34.152  -1.445 -14.288  1.00  7.44           C  0.029
ANISOU 1791  C   ARG A 114     1159    692    978    -49     87    172       C
ATOM   1792  O   ARG A 114      34.256  -1.062 -15.460  1.00  7.04           O  0.028
ANISOU 1792  O   ARG A 114     1413    694    568    -94    210     54       O
ATOM   1793  CB  ARG A 114      33.247  -3.764 -14.539  1.00  8.12           C  0.030
ANISOU 1793  CB  ARG A 114     1299    730   1056    100    -84    196       C
ATOM   1794  CG  ARG A 114      33.479  -5.254 -14.164  1.00  8.19           C  0.030
ANISOU 1794  CG  ARG A 114     1448    888    773    232   -139    252       C
ATOM   1795  CD  ARG A 114      32.439  -6.146 -14.668  1.00  7.85           C  0.029
ANISOU 1795  CD  ARG A 114     1233    820    929    385    279    197       C
ATOM   1796  NE  ARG A 114      32.408  -6.248 -16.108  1.00  8.83           N  0.031
ANISOU 1796  NE  ARG A 114     1142    723   1491    325    172    112       N
ATOM   1797  CZ  ARG A 114      31.612  -7.077 -16.758  1.00  9.70           C  0.033
ANISOU 1797  CZ  ARG A 114     1126    978   1582    165    -22     73       C
ATOM   1798  NH1 ARG A 114      30.699  -7.797 -16.123  1.00  9.36           N  0.032
ANISOU 1798  NH1 ARG A 114      944    982   1629    314    -35    -61       N
ATOM   1799  NH2 ARG A 114      31.730  -7.182 -18.063  1.00  9.01           N  0.031
ANISOU 1799  NH2 ARG A 114     1179    903   1341     10   -322     15       N
ATOM   1800  H   ARG A 114      33.879  -2.907 -12.075  1.00  9.52           H  0.032
ATOM   1801  HA  ARG A 114      35.205  -3.173 -14.449  1.00  7.89           H  0.029
ATOM   1802  HB2 ARG A 114      32.405  -3.471 -14.157  1.00  9.74           H  0.033
ATOM   1803  HB3 ARG A 114      33.219  -3.683 -15.505  1.00  9.74           H  0.033
ATOM   1804  HG2 ARG A 114      34.326  -5.543 -14.539  1.00  9.82           H  0.033
ATOM   1805  HG3 ARG A 114      33.498  -5.335 -13.198  1.00  9.82           H  0.033
ATOM   1806  HD2 ARG A 114      32.590  -7.036 -14.312  1.00  9.42           H  0.032
ATOM   1807  HD3 ARG A 114      31.575  -5.815 -14.377  1.00  9.42           H  0.032
ATOM   1808  HE  ARG A 114      32.934  -5.744 -16.564  1.00 10.60           H  0.034
ATOM   1809 HH11 ARG A 114      30.616  -7.730 -15.269  1.00 11.23           H  0.035
ATOM   1810 HH12 ARG A 114      30.190  -8.331 -16.564  1.00 11.23           H  0.035
ATOM   1811 HH21 ARG A 114      32.319  -6.715 -18.480  1.00 10.81           H  0.034
ATOM   1812 HH22 ARG A 114      31.218  -7.718 -18.499  1.00 10.81           H  0.034
ATOM   1813  N   CYS A 115      33.832  -0.618 -13.303  1.00  6.13           N  0.026
ANISOU 1813  N   CYS A 115      925    498    906     43    -10   -113       N
ATOM   1814  CA  CYS A 115      33.505   0.786 -13.590  1.00  6.33           C  0.026
ANISOU 1814  CA  CYS A 115      790    655    959     37    221   -178       C
ATOM   1815  C   CYS A 115      34.447   1.765 -12.922  1.00  6.86           C  0.027
ANISOU 1815  C   CYS A 115      835   1076    694    -29     83   -152       C
ATOM   1816  O   CYS A 115      34.891   2.736 -13.530  1.00  7.41           O  0.029
ANISOU 1816  O   CYS A 115      965   1086    764   -155     13     91       O
ATOM   1817  CB  CYS A 115      32.068   1.106 -13.120  1.00  6.91           C  0.028
ANISOU 1817  CB  CYS A 115      880    862    883   -152    428      3       C
ATOM   1818  SG  CYS A 115      30.802   0.128 -13.920  1.00  7.18           S  0.028
ANISOU 1818  SG  CYS A 115      777    820   1131   -133     81     91       S
```

```
ATOM   1819  H   CYS A 115      33.796  -0.836 -12.472  1.00  7.36           H  0.028
ATOM   1820  HA  CYS A 115      33.553   0.916 -14.550  1.00  7.59           H  0.029
ATOM   1821  HB2 CYS A 115      32.009   0.941 -12.166  1.00  8.29           H  0.030
ATOM   1822  HB3 CYS A 115      31.881   2.040 -13.307  1.00  8.29           H  0.030
ATOM   1823  N   LYS A 116      34.777   1.506 -11.675  1.00  7.47           N  0.029
ANISOU 1823  N   LYS A 116      987   1266    584     74     32   -168       N
ATOM   1824  CA  LYS A 116      35.634   2.366 -10.899  1.00  7.44           C  0.029
ANISOU 1824  CA  LYS A 116     1113   1150    562    119   -195    -80       C
ATOM   1825  C   LYS A 116      36.934   2.631 -11.625  1.00  8.48           C  0.031
ANISOU 1825  C   LYS A 116     1434   1281    505   -154   -105      7       C
ATOM   1826  O   LYS A 116      37.654   1.720 -12.025  1.00  9.61           O  0.033
ANISOU 1826  O   LYS A 116     1308   1264   1079   -115    153    167       O
ATOM   1827  CB  LYS A 116      35.965   1.642  -9.597  1.00  8.15           C  0.030
ANISOU 1827  CB  LYS A 116     1031   1271    795     56   -308    274       C
ATOM   1828  CG  LYS A 116      36.732   2.446  -8.572  1.00  6.66           C  0.027
ANISOU 1828  CG  LYS A 116      914   1197    419    158   -236     14       C
ATOM   1829  CD  LYS A 116      36.886   1.589  -7.298  1.00  6.51           C  0.027
ANISOU 1829  CD  LYS A 116      946   1118    412    272    -79   -143       C
ATOM   1830  CE  LYS A 116      37.367   2.402  -6.085  1.00  7.20           C  0.028
ANISOU 1830  CE  LYS A 116     1096   1042    597    184   -108    -13       C
ATOM   1831  NZ  LYS A 116      37.552   1.601  -4.856  1.00  8.67           N  0.031
ANISOU 1831  NZ  LYS A 116     1091    807   1396    253   -330     52       N
ATOM   1832  H   LYS A 116      34.507   0.814 -11.241  1.00  8.96           H  0.031
ATOM   1833  HA  LYS A 116      35.186   3.210 -10.732  1.00  8.92           H  0.031
ATOM   1834  HB2 LYS A 116      35.132   1.368  -9.182  1.00  9.78           H  0.033
ATOM   1835  HB3 LYS A 116      36.502   0.863  -9.810  1.00  9.78           H  0.033
ATOM   1836  HG2 LYS A 116      37.612   2.669  -8.914  1.00  7.99           H  0.030
ATOM   1837  HG3 LYS A 116      36.246   3.257  -8.353  1.00  7.99           H  0.030
ATOM   1838  HD2 LYS A 116      36.027   1.198  -7.074  1.00  7.82           H  0.029
ATOM   1839  HD3 LYS A 116      37.536   0.889  -7.465  1.00  7.82           H  0.029
ATOM   1840  HE2 LYS A 116      38.220   2.809  -6.302  1.00  8.64           H  0.031
ATOM   1841  HE3 LYS A 116      36.710   3.090  -5.891  1.00  8.64           H  0.031
ATOM   1842  HZ1 LYS A 116      36.791   1.570  -4.396  1.00 10.40           H  0.034
ATOM   1843  HZ2 LYS A 116      37.796   0.772  -5.067  1.00 10.40           H  0.034
ATOM   1844  HZ3 LYS A 116      38.183   1.968  -4.347  1.00 10.40           H  0.034
ATOM   1845  N   GLY A 117      37.251   3.898 -11.742  1.00 10.83           N  0.035
ANISOU 1845  N   GLY A 117     1825   1623    669   -409   -169    221       N
ATOM   1846  CA  GLY A 117      38.493   4.288 -12.324  1.00 13.26           C  0.038
ANISOU 1846  CA  GLY A 117     1880   2083   1075   -636   -208    502       C
ATOM   1847  C   GLY A 117      38.491   4.313 -13.830  1.00 12.75           C  0.037
ANISOU 1847  C   GLY A 117     1655   2379    811   -725    -83    363       C
ATOM   1848  O   GLY A 117      39.503   4.700 -14.418  1.00 16.27           O  0.042
ANISOU 1848  O   GLY A 117     1822   3091   1269  -1012     -8    581       O
ATOM   1849  H   GLY A 117      36.753   4.551 -11.488  1.00 13.00           H  0.038
ATOM   1850  HA2 GLY A 117      38.718   5.179 -12.011  1.00 15.91           H  0.042
ATOM   1851  HA3 GLY A 117      39.182   3.669 -12.036  1.00 15.91           H  0.042
ATOM   1852  N   THR A 118      37.392   3.940 -14.467  1.00 10.49           N  0.034
ANISOU 1852  N   THR A 118     1272   1941    771   -459   -116    331       N
ATOM   1853  CA  THR A 118      37.294   3.994 -15.913  1.00  9.12           C  0.032
ANISOU 1853  CA  THR A 118     1214   1620    632   -115    217    216       C
ATOM   1854  C   THR A 118      36.617   5.287 -16.335  1.00  8.79           C  0.031
ANISOU 1854  C   THR A 118     1207   1484    648   -101    345    296       C
ATOM   1855  O   THR A 118      36.171   6.088 -15.527  1.00  9.90           O  0.033
ANISOU 1855  O   THR A 118     1414   1283   1067   -296    247    155       O
ATOM   1856  CB  THR A 118      36.537   2.773 -16.425  1.00  9.97           C  0.033
ANISOU 1856  CB  THR A 118     1202   1556   1029     38    260    213       C
ATOM   1857  OG1 THR A 118      35.124   2.953 -16.225  1.00  9.61           O  0.033
ANISOU 1857  OG1 THR A 118     1137   1391   1123    172    172    279       O
ATOM   1858  CG2 THR A 118      37.066   1.471 -15.766  1.00 12.50           C  0.037
ANISOU 1858  CG2 THR A 118     1483   1584   1682     11    -62     80       C
ATOM   1859  H   THR A 118      36.683   3.649 -14.078  1.00 12.58           H  0.037
ATOM   1860  HA  THR A 118      38.184   3.984 -16.299  1.00 10.95           H  0.035
ATOM   1861  HB  THR A 118      36.684   2.668 -17.378  1.00 11.96           H  0.036
ATOM   1862  HG1 THR A 118      34.988   3.431 -15.548  1.00 11.53           H  0.036
ATOM   1863 HG21 THR A 118      36.826   0.704 -16.309  1.00 15.00           H  0.041
ATOM   1864 HG22 THR A 118      38.031   1.509 -15.684  1.00 15.00           H  0.041
ATOM   1865 HG23 THR A 118      36.679   1.365 -14.883  1.00 15.00           H  0.041
ATOM   1866  N   ASP A 119      36.615   5.503 -17.641  1.00 10.41           N  0.034
ANISOU 1866  N   ASP A 119     1142   1825    987    122    398    364       N
ATOM   1867  CA  ASP A 119      35.939   6.691 -18.188  1.00 10.29           C  0.034
ANISOU 1867  CA  ASP A 119     1153   1959    798    -84     93    563       C
ATOM   1868  C   ASP A 119      34.445   6.418 -18.182  1.00  9.71           C  0.033
ANISOU 1868  C   ASP A 119     1091   1998    600    -52    144    264       C
ATOM   1869  O   ASP A 119      33.957   5.902 -19.133  1.00 13.48           O  0.038
ANISOU 1869  O   ASP A 119     1228   2246   1649    -80   -123    159       O
ATOM   1870  CB  ASP A 119      36.429   6.958 -19.605  1.00 13.30           C  0.038
ANISOU 1870  CB  ASP A 119     1414   2287   1354    -21    253    638       C
ATOM   1871  CG  ASP A 119      35.851   8.236 -20.167  1.00 16.44           C  0.043
```

```
ANISOU 1871  CG  ASP A 119     1831   2471   1943    -32    763    915       C
ATOM   1872  OD1 ASP A 119      34.817   8.689 -19.668  1.00 19.55           O  0.046
ANISOU 1872  OD1 ASP A 119     2137   2644   2648    186    388    970       O
ATOM   1873  OD2 ASP A 119      36.456   8.761 -21.092  1.00 16.32           O  0.042
ANISOU 1873  OD2 ASP A 119     1945   2311   1943      9    685   1018       O
ATOM   1874  H   ASP A 119      37.064   4.885 -18.340  1.00 12.49           H  0.037
ATOM   1875  HA  ASP A 119      36.156   7.553 -17.559  1.00 12.35           H  0.037
ATOM   1876  HB2 ASP A 119      37.514   7.041 -19.599  1.00 15.97           H  0.042
ATOM   1877  HB3 ASP A 119      36.151   6.128 -20.253  1.00 15.97           H  0.042
ATOM   1878  N   VAL A 120      33.800   6.741 -17.076  1.00  9.31           N  0.032
ANISOU 1878  N   VAL A 120      979   1970    590     -2    165    107       N
ATOM   1879  CA  VAL A 120      32.384   6.414 -17.023  1.00  8.87           C  0.031
ANISOU 1879  CA  VAL A 120      960   1831    579   -233    100    162       C
ATOM   1880  C   VAL A 120      31.546   7.422 -17.791  1.00  9.34           C  0.032
ANISOU 1880  C   VAL A 120     1105   1787    655   -326    166    284       C
ATOM   1881  O   VAL A 120      30.352   7.160 -18.036  1.00 10.48           O  0.034
ANISOU 1881  O   VAL A 120     1385   1866    733   -239    134    296       O
ATOM   1882  CB  VAL A 120      31.906   6.263 -15.567  1.00 10.80           C  0.034
ANISOU 1882  CB  VAL A 120     1013   1914   1175     16     22    302       C
ATOM   1883  CG1 VAL A 120      32.608   5.055 -14.961  1.00 10.27           C  0.034
ANISOU 1883  CG1 VAL A 120     1051   1847   1006   -149   -111    366       C
ATOM   1884  CG2 VAL A 120      32.171   7.506 -14.743  1.00 13.31           C  0.038
ANISOU 1884  CG2 VAL A 120     1092   2291   1675    -91   -333    472       C
ATOM   1885  H   VAL A 120      34.133   7.112 -16.375  1.00 11.17           H  0.035
ATOM   1886  HA  VAL A 120      32.247   5.553 -17.448  1.00 10.64           H  0.034
ATOM   1887  HB  VAL A 120      30.945   6.135 -15.556  1.00 12.96           H  0.038
ATOM   1888 HG11 VAL A 120      32.252   4.895 -14.074  1.00 12.33           H  0.037
ATOM   1889 HG12 VAL A 120      32.451   4.283 -15.526  1.00 12.33           H  0.037
ATOM   1890 HG13 VAL A 120      33.560   5.237 -14.908  1.00 12.33           H  0.037
ATOM   1891 HG21 VAL A 120      31.563   7.520 -13.988  1.00 15.97           H  0.042
ATOM   1892 HG22 VAL A 120      33.089   7.486 -14.428  1.00 15.97           H  0.042
ATOM   1893 HG23 VAL A 120      32.028   8.289 -15.297  1.00 15.97           H  0.042
ATOM   1894  N   GLN A 121      32.115   8.602 -18.113  1.00 12.46           N  0.037
ANISOU 1894  N   GLN A 121     1135   2044   1555   -464    261    393       N
ATOM   1895  CA  GLN A 121      31.388   9.573 -18.924  1.00 12.84           C  0.038
ANISOU 1895  CA  GLN A 121     1314   2099   1465   -155    295    374       C
ATOM   1896  C   GLN A 121      30.955   8.958 -20.239  1.00 10.91           C  0.035
ANISOU 1896  C   GLN A 121     1143   1779   1222    -29    -93    458       C
ATOM   1897  O   GLN A 121      29.952   9.367 -20.819  1.00 13.00           O  0.038
ANISOU 1897  O   GLN A 121     1348   2058   1533     49   -102    535       O
ATOM   1898  CB  GLN A 121      32.275  10.788 -19.201  1.00 19.40           C  0.046
ANISOU 1898  CB  GLN A 121     1917   2736   2718   -189    -64    198       C
ATOM   1899  CG  GLN A 121      32.332  11.837 -18.105  1.00 26.63           C  0.054
ANISOU 1899  CG  GLN A 121     2583   3363   4171   -179    -67    231       C
ATOM   1900  CD  GLN A 121      31.225  12.878 -18.241  1.00 32.16           C  0.059
ANISOU 1900  CD  GLN A 121     3152   3863   5205   -190   -354    316       C
ATOM   1901  OE1 GLN A 121      30.466  12.865 -19.213  1.00 34.19           O  0.061
ANISOU 1901  OE1 GLN A 121     3402   4118   5469   -412   -208    584       O
ATOM   1902  NE2 GLN A 121      31.142  13.793 -17.275  1.00 34.41           N  0.062
ANISOU 1902  NE2 GLN A 121     3421   3991   5661    -32   -516    127       N
ATOM   1903  H   GLN A 121      32.903   8.851 -17.876  1.00 14.95           H  0.041
ATOM   1904  HA  GLN A 121      30.603   9.869 -18.437  1.00 15.41           H  0.041
ATOM   1905  HB2 GLN A 121      33.181  10.475 -19.345  1.00 23.28           H  0.051
ATOM   1906  HB3 GLN A 121      31.945  11.228 -20.000  1.00 23.28           H  0.051
ATOM   1907  HG2 GLN A 121      32.234  11.402 -17.244  1.00 31.95           H  0.059
ATOM   1908  HG3 GLN A 121      33.186  12.296 -18.150  1.00 31.95           H  0.059
ATOM   1909 HE21 GLN A 121      31.697  13.776 -16.618  1.00 41.29           H  0.067
ATOM   1910 HE22 GLN A 121      30.533  14.399 -17.308  1.00 41.29           H  0.067
ATOM   1911  N   ALA A 122      31.672   7.946 -20.696  1.00  8.49           N  0.031
ANISOU 1911  N   ALA A 122     1219   1342    664     -8   -245    353       N
ATOM   1912  CA  ALA A 122      31.354   7.322 -21.967  1.00  9.78           C  0.033
ANISOU 1912  CA  ALA A 122     1503   1270    942    142     16    337       C
ATOM   1913  C   ALA A 122      29.966   6.700 -21.959  1.00 10.67           C  0.034
ANISOU 1913  C   ALA A 122     1604   1226   1226    174    198    493       C
ATOM   1914  O   ALA A 122      29.340   6.547 -23.000  1.00 11.44           O  0.035
ANISOU 1914  O   ALA A 122     1656   1280   1411    -13    -89    457       O
ATOM   1915  CB  ALA A 122      32.372   6.218 -22.247  1.00 10.62           C  0.034
ANISOU 1915  CB  ALA A 122     1493   1517   1023    158    451   -135       C
ATOM   1916  H   ALA A 122      32.348   7.601 -20.291  1.00 10.19           H  0.033
ATOM   1917  HA  ALA A 122      31.390   7.997 -22.663  1.00 11.73           H  0.036
ATOM   1918  HB1 ALA A 122      32.219   5.866 -23.137  1.00 12.74           H  0.037
ATOM   1919  HB2 ALA A 122      33.266   6.591 -22.187  1.00 12.74           H  0.037
ATOM   1920  HB3 ALA A 122      32.263   5.514 -21.589  1.00 12.74           H  0.037
ATOM   1921  N   TRP A 123      29.486   6.291 -20.785  1.00 10.63           N  0.034
ANISOU 1921  N   TRP A 123     1590   1404   1047     52    220    512       N
ATOM   1922  CA  TRP A 123      28.172   5.689 -20.675  1.00 11.09           C  0.035
ANISOU 1922  CA  TRP A 123     1400   1354   1460     48     75    579       C
ATOM   1923  C   TRP A 123      27.030   6.650 -20.953  1.00 11.67           C  0.036
```

```
ANISOU 1923  C   TRP A 123     1553   1358   1522    132   -403    292       C
ATOM   1924  O   TRP A 123      25.927   6.182 -21.241  1.00 12.89           O  0.038
ANISOU 1924  O   TRP A 123     1582   1422   1895    300   -544    177       O
ATOM   1925  CB  TRP A 123      28.047   5.039 -19.271  1.00 10.93           C  0.035
ANISOU 1925  CB  TRP A 123     1351   1466   1338     98    310    164       C
ATOM   1926  CG  TRP A 123      28.898   3.829 -19.216  1.00  9.18           C  0.032
ANISOU 1926  CG  TRP A 123     1349   1222    918    -19     50     72       C
ATOM   1927  CD1 TRP A 123      30.100   3.717 -18.628  1.00  9.82           C  0.033
ANISOU 1927  CD1 TRP A 123     1644   1384    702    199    -50    139       C
ATOM   1928  CD2 TRP A 123      28.625   2.558 -19.826  1.00 10.31           C  0.034
ANISOU 1928  CD2 TRP A 123     1559   1296   1063    109    236     42       C
ATOM   1929  NE1 TRP A 123      30.625   2.467 -18.864  1.00 10.82           N  0.034
ANISOU 1929  NE1 TRP A 123     1598   1442   1070    251   -108    245       N
ATOM   1930  CE2 TRP A 123      29.737   1.725 -19.582  1.00 11.27           C  0.035
ANISOU 1930  CE2 TRP A 123     1488   1406   1386     52    314    205       C
ATOM   1931  CE3 TRP A 123      27.567   2.062 -20.569  1.00 12.22           C  0.037
ANISOU 1931  CE3 TRP A 123     1514   1323   1808      4    306    260       C
ATOM   1932  CZ2 TRP A 123      29.819   0.397 -20.044  1.00 14.01           C  0.039
ANISOU 1932  CZ2 TRP A 123     1509   1605   2209     40    249     19       C
ATOM   1933  CZ3 TRP A 123      27.639   0.735 -21.055  1.00 15.20           C  0.041
ANISOU 1933  CZ3 TRP A 123     1631   1502   2641    -14    382    112       C
ATOM   1934  CH2 TRP A 123      28.768  -0.081 -20.784  1.00 15.60           C  0.041
ANISOU 1934  CH2 TRP A 123     1651   1622   2655     20    372   -254       C
ATOM   1935  H   TRP A 123      29.907   6.353 -20.038  1.00 12.76           H  0.037
ATOM   1936  HA  TRP A 123      28.080   4.994 -21.345  1.00 13.31           H  0.038
ATOM   1937  HB2 TRP A 123      28.344   5.664 -18.591  1.00 13.12           H  0.038
ATOM   1938  HB3 TRP A 123      27.125   4.784 -19.107  1.00 13.12           H  0.038
ATOM   1939  HD1 TRP A 123      30.516   4.386 -18.134  1.00 11.78           H  0.036
ATOM   1940  HE1 TRP A 123      31.397   2.196 -18.598  1.00 12.98           H  0.038
ATOM   1941  HE3 TRP A 123      26.822   2.591 -20.746  1.00 14.67           H  0.040
ATOM   1942  HZ2 TRP A 123      30.558  -0.136 -19.855  1.00 16.81           H  0.043
ATOM   1943  HZ3 TRP A 123      26.936   0.393 -21.560  1.00 18.24           H  0.045
ATOM   1944  HH2 TRP A 123      28.795  -0.951 -21.111  1.00 18.72           H  0.045
ATOM   1945  N   ILE A 124      27.240   7.953 -20.793  1.00 11.45           N  0.035
ANISOU 1945  N   ILE A 124     1546   1403   1399    -82   -413    528       N
ATOM   1946  CA  ILE A 124      26.224   8.977 -21.017  1.00 11.29           C  0.035
ANISOU 1946  CA  ILE A 124     1461   1550   1278     30   -243    676       C
ATOM   1947  C   ILE A 124      26.507   9.799 -22.276  1.00 11.81           C  0.036
ANISOU 1947  C   ILE A 124     1625   1769   1094     58    -39    659       C
ATOM   1948  O   ILE A 124      25.718  10.678 -22.629  1.00 13.72           O  0.039
ANISOU 1948  O   ILE A 124     1738   1777   1698     20   -155    559       O
ATOM   1949  CB  ILE A 124      25.989   9.875 -19.784  1.00 13.51           C  0.039
ANISOU 1949  CB  ILE A 124     1678   1618   1836    106    -11    315       C
ATOM   1950  CG1 ILE A 124      27.236  10.683 -19.440  1.00 16.52           C  0.043
ANISOU 1950  CG1 ILE A 124     1923   1785   2571    219     62      8       C
ATOM   1951  CG2 ILE A 124      25.509   9.060 -18.587  1.00 12.61           C  0.037
ANISOU 1951  CG2 ILE A 124     1810   1662   1320    168   -198    185       C
ATOM   1952  CD1 ILE A 124      26.957  11.839 -18.447  1.00 18.41           C  0.045
ANISOU 1952  CD1 ILE A 124     2074   1895   3027    317   -114    -12       C
ATOM   1953  H   ILE A 124      27.994   8.282 -20.545  1.00 13.74           H  0.039
ATOM   1954  HA  ILE A 124      25.378   8.524 -21.162  1.00 13.54           H  0.039
ATOM   1955  HB  ILE A 124      25.287  10.503 -20.014  1.00 16.21           H  0.042
ATOM   1956 HG12 ILE A 124      27.891  10.094 -19.036  1.00 19.83           H  0.047
ATOM   1957 HG13 ILE A 124      27.594  11.069 -20.254  1.00 19.83           H  0.047
ATOM   1958 HG21 ILE A 124      25.125   9.661 -17.930  1.00 15.14           H  0.041
ATOM   1959 HG22 ILE A 124      24.841   8.424 -18.886  1.00 15.14           H  0.041
ATOM   1960 HG23 ILE A 124      26.265   8.589 -18.203  1.00 15.14           H  0.041
ATOM   1961 HD11 ILE A 124      27.758  12.379 -18.359  1.00 22.09           H  0.049
ATOM   1962 HD12 ILE A 124      26.229  12.381 -18.789  1.00 22.09           H  0.049
ATOM   1963 HD13 ILE A 124      26.715  11.464 -17.586  1.00 22.09           H  0.049
ATOM   1964  N   ARG A 125      27.582   9.480 -22.985  1.00 14.26           N  0.040
ANISOU 1964  N   ARG A 125     1930   2072   1416    329     94    961       N
ATOM   1965  CA  ARG A 125      28.051  10.302 -24.099  1.00 17.50           C  0.044
ANISOU 1965  CA  ARG A 125     2317   2649   1684    502    368   1146       C
ATOM   1966  C   ARG A 125      27.031  10.256 -25.236  1.00 17.92           C  0.044
ANISOU 1966  C   ARG A 125     2508   2775   1527    458    314   1067       C
ATOM   1967  O   ARG A 125      26.500   9.198 -25.580  1.00 18.37           O  0.045
ANISOU 1967  O   ARG A 125     2565   2923   1493    747    144    982       O
ATOM   1968  CB  ARG A 125      29.435   9.803 -24.536  1.00 22.48           C  0.050
ANISOU 1968  CB  ARG A 125     2995   3201   2346    544    859   1428       C
ATOM   1969  CG  ARG A 125      30.250  10.784 -25.415  1.00 27.25           C  0.055
ANISOU 1969  CG  ARG A 125     3617   3608   3130    648    613   1326       C
ATOM   1970  CD  ARG A 125      31.483  10.110 -26.049  1.00 31.68           C  0.059
ANISOU 1970  CD  ARG A 125     4143   3993   3903    800    503   1135       C
ATOM   1971  NE  ARG A 125      31.214   8.718 -26.399  1.00 36.89           N  0.064
ANISOU 1971  NE  ARG A 125     4636   4432   4949    878    244    743       N
ATOM   1972  CZ  ARG A 125      32.031   7.695 -26.173  1.00 39.92           C  0.066
ANISOU 1972  CZ  ARG A 125     4985   4659   5523    932    -85    472       C
```

```
ATOM   1973  NH1 ARG A 125      33.257   7.874 -25.710  1.00 39.75           N  0.066
ANISOU 1973  NH1 ARG A 125     4938   4718   5446    977   -268    507       N
ATOM   1974  NH2 ARG A 125      31.605   6.458 -26.421  1.00 41.30           N  0.067
ANISOU 1974  NH2 ARG A 125     5185   4756   5753    849   -221    354       N
ATOM   1975  H   ARG A 125      28.067   8.785 -22.841  1.00 17.11           H  0.043
ATOM   1976  HA  ARG A 125      28.142  11.233 -23.840  1.00 21.00           H  0.048
ATOM   1977  HB2 ARG A 125      29.960   9.624 -23.740  1.00 26.98           H  0.054
ATOM   1978  HB3 ARG A 125      29.318   8.987 -25.047  1.00 26.98           H  0.054
ATOM   1979  HG2 ARG A 125      29.685  11.115 -26.130  1.00 32.70           H  0.060
ATOM   1980  HG3 ARG A 125      30.557  11.522 -24.866  1.00 32.70           H  0.060
ATOM   1981  HD2 ARG A 125      31.728  10.586 -26.858  1.00 38.02           H  0.065
ATOM   1982  HD3 ARG A 125      32.219  10.128 -25.417  1.00 38.02           H  0.065
ATOM   1983  HE  ARG A 125      30.464   8.546 -26.783  1.00 44.27           H  0.070
ATOM   1984 HH11 ARG A 125      33.543   8.668 -25.547  1.00 47.69           H  0.072
ATOM   1985 HH12 ARG A 125      33.767   7.196 -25.572  1.00 47.69           H  0.072
ATOM   1986 HH21 ARG A 125      30.811   6.330 -26.723  1.00 49.56           H  0.074
ATOM   1987 HH22 ARG A 125      32.125   5.788 -26.279  1.00 49.56           H  0.074
ATOM   1988  N   GLY A 126      26.716  11.436 -25.788  1.00 19.74           N  0.047
ANISOU 1988  N   GLY A 126     2465   3000   2036    439    460   1189       N
ATOM   1989  CA  GLY A 126      25.776  11.533 -26.879  1.00 20.23           C  0.047
ANISOU 1989  CA  GLY A 126     2348   3035   2303    473    509   1045       C
ATOM   1990  C   GLY A 126      24.320  11.566 -26.487  1.00 18.73           C  0.045
ANISOU 1990  C   GLY A 126     2187   2820   2109    488    158   1182       C
ATOM   1991  O   GLY A 126      23.490  11.978 -27.300  1.00 20.05           O  0.047
ANISOU 1991  O   GLY A 126     2151   3121   2346    230    -16    780       O
ATOM   1992  H   GLY A 126      27.043  12.191 -25.537  1.00 23.69           H  0.051
ATOM   1993  HA2 GLY A 126      25.964  12.345 -27.375  1.00 24.27           H  0.052
ATOM   1994  HA3 GLY A 126      25.904  10.769 -27.464  1.00 24.27           H  0.052
ATOM   1995  N   CYS A 127      23.984  11.172 -25.262  1.00 16.13           N  0.042
ANISOU 1995  N   CYS A 127     2042   2314   1772    480     22   1114       N
ATOM   1996  CA  CYS A 127      22.594  11.077 -24.862  1.00 15.43           C  0.041
ANISOU 1996  CA  CYS A 127     2032   1914   1915    424   -119   1045       C
ATOM   1997  C   CYS A 127      21.999  12.473 -24.691  1.00 18.47           C  0.045
ANISOU 1997  C   CYS A 127     2350   2154   2513    526    -12   1114       C
ATOM   1998  O   CYS A 127      22.648  13.378 -24.160  1.00 19.74           O  0.047
ANISOU 1998  O   CYS A 127     2357   2030   3112    650    121   1216       O
ATOM   1999  CB  CYS A 127      22.498  10.391 -23.502  1.00 13.19           C  0.038
ANISOU 1999  CB  CYS A 127     1781   1563   1668    123   -450    537       C
ATOM   2000  SG  CYS A 127      23.228   8.764 -23.397  1.00 14.58           S  0.040
ANISOU 2000  SG  CYS A 127     1989   1630   1922   -133   -171    542       S
ATOM   2001  H   CYS A 127      24.545  10.955 -24.648  1.00 19.35           H  0.046
ATOM   2002  HA  CYS A 127      22.104  10.578 -25.534  1.00 18.51           H  0.045
ATOM   2003  HB2 CYS A 127      22.946  10.949 -22.848  1.00 15.83           H  0.042
ATOM   2004  HB3 CYS A 127      21.559  10.300 -23.274  1.00 15.83           H  0.042
ATOM   2005  N   ARG A 128      20.703  12.607 -25.016  1.00 20.32           N  0.047
ANISOU 2005  N   ARG A 128     2687   2597   2434    559   -530    861       N
ATOM   2006  CA  ARG A 128      19.947  13.802 -24.651  1.00 25.63           C  0.053
ANISOU 2006  CA  ARG A 128     3267   3225   3245    601   -456    748       C
ATOM   2007  C   ARG A 128      19.635  13.763 -23.156  1.00 26.62           C  0.054
ANISOU 2007  C   ARG A 128     3485   3261   3369    550   -280    509       C
ATOM   2008  O   ARG A 128      18.945  12.850 -22.683  1.00 26.38           O  0.054
ANISOU 2008  O   ARG A 128     3560   3095   3366    378   -200    368       O
ATOM   2009  CB  ARG A 128      18.659  13.864 -25.471  1.00 30.75           C  0.058
ANISOU 2009  CB  ARG A 128     3744   3899   4039    693   -586    550       C
ATOM   2010  CG  ARG A 128      17.865  15.161 -25.299  1.00 35.70           C  0.063
ANISOU 2010  CG  ARG A 128     4220   4541   4804    721   -645    443       C
ATOM   2011  CD  ARG A 128      16.629  15.161 -26.210  1.00 40.54           C  0.067
ANISOU 2011  CD  ARG A 128     4719   5133   5553    725   -647    357       C
ATOM   2012  NE  ARG A 128      16.985  14.862 -27.594  1.00 44.22           N  0.070
ANISOU 2012  NE  ARG A 128     5086   5616   6100    764   -580    491       N
ATOM   2013  CZ  ARG A 128      16.162  14.970 -28.630  1.00 47.06           C  0.072
ANISOU 2013  CZ  ARG A 128     5369   5971   6540    898   -463    539       C
ATOM   2014  NH1 ARG A 128      14.890  15.310 -28.471  1.00 48.07           N  0.073
ANISOU 2014  NH1 ARG A 128     5435   6034   6797   1004   -367    606       N
ATOM   2015  NH2 ARG A 128      16.624  14.716 -29.854  1.00 47.56           N  0.072
ANISOU 2015  NH2 ARG A 128     5467   6118   6486    932   -433    462       N
ATOM   2016  H   ARG A 128      20.246  12.019 -25.446  1.00 24.38           H  0.052
ATOM   2017  HA  ARG A 128      20.471  14.597 -24.834  1.00 30.75           H  0.058
ATOM   2018  HB2 ARG A 128      18.885  13.780 -26.410  1.00 36.90           H  0.064
ATOM   2019  HB3 ARG A 128      18.085  13.130 -25.201  1.00 36.90           H  0.064
ATOM   2020  HG2 ARG A 128      17.570  15.243 -24.379  1.00 42.84           H  0.069
ATOM   2021  HG3 ARG A 128      18.423  15.918 -25.537  1.00 42.84           H  0.069
ATOM   2022  HD2 ARG A 128      16.004  14.486 -25.902  1.00 48.65           H  0.073
ATOM   2023  HD3 ARG A 128      16.212  16.036 -26.184  1.00 48.65           H  0.073
ATOM   2024  HE  ARG A 128      17.787  14.596 -27.750  1.00 53.07           H  0.076
ATOM   2025 HH11 ARG A 128      14.582  15.466 -27.684  1.00 57.69           H  0.080
ATOM   2026 HH12 ARG A 128      14.374  15.374 -29.156  1.00 57.69           H  0.080
ATOM   2027 HH21 ARG A 128      17.445  14.486 -29.965  1.00 57.07           H  0.079
```

```
ATOM   2028 HH22 ARG A 128      16.100  14.783 -30.534  1.00 57.07           H  0.079
ATOM   2029  N   LEU A 129      20.178  14.722 -22.407  1.00 27.80           N  0.055
ANISOU 2029  N   LEU A 129     3730   3300   3535    605    -91    516       N
ATOM   2030  CA  LEU A 129      19.958  14.819 -20.963  1.00 30.13           C  0.058
ANISOU 2030  CA  LEU A 129     4047   3716   3687    636   -232    367       C
ATOM   2031  C   LEU A 129      19.662  16.261 -20.553  1.00 34.19           C  0.061
ANISOU 2031  C   LEU A 129     4506   4144   4341    841   -660    305       C
ATOM   2032  O   LEU A 129      18.964  16.998 -21.257  1.00 35.15           O  0.062
ANISOU 2032  O   LEU A 129     4652   4301   4405    979   -878    544       O
ATOM   2033  CB  LEU A 129      21.173  14.293 -20.177  1.00 28.51           C  0.056
ANISOU 2033  CB  LEU A 129     3888   3764   3180    508    -34    234       C
ATOM   2034  CG  LEU A 129      21.628  12.878 -20.539  1.00 29.00           C  0.056
ANISOU 2034  CG  LEU A 129     3814   3831   3373    453     29     32       C
ATOM   2035  CD1 LEU A 129      22.992  12.508 -19.917  1.00 28.28           C  0.056
ANISOU 2035  CD1 LEU A 129     3771   3889   3086    503    172     90       C
ATOM   2036  CD2 LEU A 129      20.587  11.893 -20.055  1.00 30.50           C  0.058
ANISOU 2036  CD2 LEU A 129     3905   3848   3836    439    -28    -87       C
ATOM   2037  OXT LEU A 129      20.116  16.714 -19.495  1.00 36.01           O  0.063
ANISOU 2037  OXT LEU A 129     4707   4273   4704    881   -806    215       O
ATOM   2038  H   LEU A 129      20.688  15.342 -22.717  1.00 33.37           H  0.061
ATOM   2039  HA  LEU A 129      19.194  14.268 -20.730  1.00 36.16           H  0.063
ATOM   2040  HB2 LEU A 129      21.921  14.889 -20.339  1.00 34.21           H  0.061
ATOM   2041  HB3 LEU A 129      20.948  14.293 -19.233  1.00 34.21           H  0.061
ATOM   2042  HG  LEU A 129      21.733  12.835 -21.502  1.00 34.80           H  0.062
ATOM   2043 HD11 LEU A 129      23.290  11.664 -20.290  1.00 33.94           H  0.061
ATOM   2044 HD12 LEU A 129      23.633  13.206 -20.123  1.00 33.94           H  0.061
ATOM   2045 HD13 LEU A 129      22.889  12.426 -18.956  1.00 33.94           H  0.061
ATOM   2046 HD21 LEU A 129      20.876  10.994 -20.277  1.00 36.60           H  0.063
ATOM   2047 HD22 LEU A 129      20.492  11.981 -19.093  1.00 36.60           H  0.063
ATOM   2048 HD23 LEU A 129      19.742  12.086 -20.490  1.00 36.60           H  0.063
HETATM 2049 CL    CL A1131      29.580  10.671 -15.944  1.00 16.13          Cl  0.042
ANISOU 2049 CL    CL A1131     2686   1800   1642   -894    104    259      Cl
HETATM 2050 CL    CL A1134      25.116  -0.477 -23.967  1.00 32.22          Cl  0.060
ANISOU 2050 CL    CL A1134     4295   3975   3973   -639   1272   -564      Cl
HETATM 2051 CL    CL A1135       7.501  -7.392   1.823  1.00 31.52          Cl  0.059
ANISOU 2051 CL    CL A1135     2759   4961   4255   -314    686   1141      Cl
HETATM 2052 CL    CL A1137      11.753 -20.078  -3.451  1.00 27.07          Cl  0.055
ANISOU 2052 CL    CL A1137     3161   3754   3369    758   -790   -136      Cl
HETATM 2053 NA    NA A1138      15.765  -9.571   3.521  1.00 16.93          Na  0.043
ANISOU 2053 NA    NA A1138     2329   1674   2431   -561    227   -277      Na
HETATM 2054  C1  RII A1139      23.026  -1.324   8.608  0.56 15.84           C  0.042
ANISOU 2054  C1  RII A1139     2447   2110   1463  -1137    379   -241       C
HETATM 2055  C2  RII A1139      23.509   1.247   8.642  0.56 19.20           C  0.046
ANISOU 2055  C2  RII A1139     2686   2182   2427   -564    727   -933       C
HETATM 2056  C3  RII A1139      22.218   0.249   6.575  0.56 18.04           C  0.045
ANISOU 2056  C3  RII A1139     2385   2240   2229   -621    389   -260       C
HETATM 2057  C4  RII A1139      25.476  -1.862   5.217  0.56 12.37           C  0.037
ANISOU 2057  C4  RII A1139     1658   1234   1807   -781    367    -46       C
HETATM 2058  C5  RII A1139      24.483  -3.753   4.965  0.56 10.71           C  0.034
ANISOU 2058  C5  RII A1139     1548   1240   1282   -656    464     63       C
HETATM 2059  C6  RII A1139      23.818  -3.051   5.866  0.56 10.84           C  0.035
ANISOU 2059  C6  RII A1139     1713   1196   1211   -794    350   -126       C
HETATM 2060  C7  RII A1139      27.049  -0.173   7.712  0.56 16.16           C  0.042
ANISOU 2060  C7  RII A1139     2442   2242   1458   -994    155    168       C
HETATM 2061  C8  RII A1139      27.494  -1.232   9.524  0.56 16.29           C  0.042
ANISOU 2061  C8  RII A1139     2331   2233   1627  -1073    333    -29       C
HETATM 2062  C9  RII A1139      26.161  -1.207   9.343  0.56 16.91           C  0.043
ANISOU 2062  C9  RII A1139     2417   2236   1774  -1056    495     56       C
HETATM 2063  N1  RII A1139      24.435  -1.817   6.048  0.56 12.43           N  0.037
ANISOU 2063  N1  RII A1139     1976   1357   1388   -896    395    -88       N
HETATM 2064  N2  RII A1139      25.528  -2.994   4.541  0.56 12.34           N  0.037
ANISOU 2064  N2  RII A1139     1658   1247   1785   -798    334    -45       N
HETATM 2065  N3  RII A1139      25.872  -0.524   8.177  0.56 13.62           N  0.039
ANISOU 2065  N3  RII A1139     2177   1930   1067   -849    368    196       N
HETATM 2066  N4  RII A1139      28.030  -0.600   8.506  0.56 15.73           N  0.042
ANISOU 2066  N4  RII A1139     2434   2286   1255  -1021    158    189       N
HETATM 2067  O1  RII A1139      22.491  -2.038   9.357  0.56 17.14           O  0.043
ANISOU 2067  O1  RII A1139     2687   2374   1450  -1003    217   -603       O
HETATM 2068  O2  RII A1139      23.254   2.079   9.385  0.56 21.43           O  0.049
ANISOU 2068  O2  RII A1139     2758   2384   2998   -429   1419   -826       O
HETATM 2069  O3  RII A1139      21.224   0.533   6.123  0.56 18.10           O  0.045
ANISOU 2069  O3  RII A1139     2564   2344   1969   -577    408   -395       O
HETATM 2070 RE1  RII A1139      23.897  -0.163   7.381  0.56 16.97          Re  0.043
ANISOU 2070 RE1  RII A1139     2463   2063   1920   -766    539   -276      Re
HETATM 2071  H21 RII A1139      26.121  -3.218   3.961  0.56 14.81           H  0.040
HETATM 2072  H41 RII A1139      26.094  -1.179   5.102  0.56 14.84           H  0.040
HETATM 2073  H42 RII A1139      28.869  -0.470   8.378  0.56 18.87           H  0.046
HETATM 2074  H51 RII A1139      24.272  -4.598   4.662  0.56 12.86           H  0.038
```

```
HETATM 2075  H61 RII A1139      23.052  -3.339   6.309  0.56 13.01           H  0.038
HETATM 2076  H71 RII A1139      27.188   0.302   6.925  0.56 19.40           H  0.046
HETATM 2077  H81 RII A1139      27.949  -1.631  10.227  0.56 19.55           H  0.046
HETATM 2078  H91 RII A1139      25.533  -1.583   9.913  0.56 20.30           H  0.047
HETATM 2079  C1  RI3 A1140       9.122   7.834 -14.610  0.82 25.34           C  0.053
ANISOU 2079  C1  RI3 A1140     1932   3050   4647    234   -735   1515       C
HETATM 2080  C2  RI3 A1140      11.684   8.428 -14.662  0.82 21.91           C  0.049
ANISOU 2080  C2  RI3 A1140     1458   2778   4088    253    127   1799       C
HETATM 2081  C3  RI3 A1140      10.848   6.037 -13.854  0.82 23.62           C  0.051
ANISOU 2081  C3  RI3 A1140     1691   2667   4617     31   -602   1553       C
HETATM 2082  C7  RI3 A1140      10.944  10.561 -11.787  0.82 28.77           C  0.056
ANISOU 2082  C7  RI3 A1140     2449   2929   5554    434   -347   1182       C
HETATM 2083  C8  RI3 A1140       9.279  11.821 -12.270  0.82 28.10           C  0.056
ANISOU 2083  C8  RI3 A1140     2374   2944   5360    339   -276    960       C
HETATM 2084  C9  RI3 A1140       9.125  10.642 -12.883  0.82 27.43           C  0.055
ANISOU 2084  C9  RI3 A1140     2283   2870   5268    320   -356   1185       C
HETATM 2085  N3  RI3 A1140      10.194   9.838 -12.573  0.82 26.57           N  0.054
ANISOU 2085  N3  RI3 A1140     2081   2796   5219    266   -422   1492       N
HETATM 2086  N4  RI3 A1140      10.403  11.759 -11.596  0.82 29.50           N  0.057
ANISOU 2086  N4  RI3 A1140     2552   2988   5669    472   -219   1030       N
HETATM 2087  O1  RI3 A1140       8.309   7.795 -15.443  0.82 27.56           O  0.055
ANISOU 2087  O1  RI3 A1140     2022   3231   5218    163   -858   1306       O
HETATM 2088  O2  RI3 A1140      12.361   8.803 -15.475  0.82 22.58           O  0.050
ANISOU 2088  O2  RI3 A1140     1526   2785   4269    358   -112   1476       O
HETATM 2089  O3  RI3 A1140      11.060   5.010 -14.254  0.82 21.33           O  0.048
ANISOU 2089  O3  RI3 A1140     1896   2530   3678   -125   -967   1368       O
HETATM 2090  O4  RI3 A1140       9.047   7.289 -11.738  0.82 23.49           O  0.051
ANISOU 2090  O4  RI3 A1140     1410   2790   4725    -52    188   1452       O
HETATM 2091 RE1  RI3 A1140      10.485   7.818 -13.287  0.82 24.93          Re  0.052
ANISOU 2091 RE1  RI3 A1140     1682   2812   4981    124   -268   1776      Re
HETATM 2092  H42 RI3 A1140      10.737  12.386 -11.114  0.82 35.40           H  0.062
HETATM 2093  H71 RI3 A1140      11.749  10.283 -11.414  0.82 34.53           H  0.062
HETATM 2094  H81 RI3 A1140       8.699  12.544 -12.313  0.82 33.73           H  0.061
HETATM 2095  H91 RI3 A1140       8.415  10.406 -13.432  0.82 32.91           H  0.060
HETATM 2096  C1  RII A1141      35.562  11.987 -23.510  0.65 14.71           C  0.040
ANISOU 2096  C1  RII A1141     1790   1983   1817     18    188   1121       C
HETATM 2097  C2  RII A1141      36.043   9.361 -23.918  0.65 12.70           C  0.037
ANISOU 2097  C2  RII A1141     1767   1852   1206   -128    160   -173       C
HETATM 2098  C3  RII A1141      34.040  10.191 -22.436  0.65 13.65           C  0.039
ANISOU 2098  C3  RII A1141     1585   1835   1766   -225    170    750       C
HETATM 2099  C4  RII A1141      36.166  11.447 -19.359  0.65 16.18           C  0.042
ANISOU 2099  C4  RII A1141     2121   2121   1908    314    205     36       C
HETATM 2100  C5  RII A1141      35.328  13.450 -19.252  0.65 16.93           C  0.043
ANISOU 2100  C5  RII A1141     2311   2185   1937    556    202   -142       C
HETATM 2101  C6  RII A1141      35.243  13.018 -20.493  0.65 15.90           C  0.042
ANISOU 2101  C6  RII A1141     2216   2057   1769    518    134    -13       C
HETATM 2102  C7  RII A1141      38.942  10.367 -21.478  0.65 14.79           C  0.040
ANISOU 2102  C7  RII A1141     1870   2105   1643   -523    724    553       C
HETATM 2103  C8  RII A1141      40.113  11.496 -22.885  0.65 15.02           C  0.041
ANISOU 2103  C8  RII A1141     2060   2032   1615   -600    584    539       C
HETATM 2104  C9  RII A1141      38.821  11.549 -23.255  0.65 13.54           C  0.039
ANISOU 2104  C9  RII A1141     1774   1978   1392   -514    619    461       C
HETATM 2105  N1  RII A1141      35.781  11.740 -20.588  0.65 14.20           N  0.040
ANISOU 2105  N1  RII A1141     1967   1951   1477    175     93    335       N
HETATM 2106  N2  RII A1141      35.916  12.454 -18.533  0.65 16.28           N  0.042
ANISOU 2106  N2  RII A1141     2279   2195   1711    529    474   -207       N
HETATM 2107  N3  RII A1141      38.068  10.822 -22.341  0.65 13.19           N  0.038
ANISOU 2107  N3  RII A1141     1726   1939   1347   -411    643    497       N
HETATM 2108  N4  RII A1141      40.174  10.764 -21.794  0.65 15.49           N  0.041
ANISOU 2108  N4  RII A1141     2042   2205   1638   -666    732    416       N
HETATM 2109  O1  RII A1141      35.314  12.916 -24.172  0.65 16.50           O  0.043
ANISOU 2109  O1  RII A1141     1835   2288   2147    125     16   1287       O
HETATM 2110  O2  RII A1141      36.124   8.696 -24.841  0.65 16.29           O  0.042
ANISOU 2110  O2  RII A1141     1904   2071   2215    -18    225    187       O
HETATM 2111  O3  RII A1141      32.927  10.059 -22.521  0.65 15.45           O  0.041
ANISOU 2111  O3  RII A1141     1820   1855   2195   -296    -67    780       O
HETATM 2112 RE1  RII A1141      35.927  10.504 -22.371  0.65 14.05          Re  0.039
ANISOU 2112 RE1  RII A1141     1658   1942   1738   -190    184    677      Re
HETATM 2113  H21 RII A1141      36.087  12.470 -17.692  0.65 19.53           H  0.046
HETATM 2114  H41 RII A1141      36.571  10.649 -19.108  0.65 19.42           H  0.046
HETATM 2115  H42 RII A1141      40.886  10.579 -21.351  0.65 18.59           H  0.045
HETATM 2116  H51 RII A1141      35.039  14.270 -18.930  0.65 20.31           H  0.047
HETATM 2117  H61 RII A1141      34.882  13.498 -21.202  0.65 19.08           H  0.046
HETATM 2118  H71 RII A1141      38.733   9.839 -20.742  0.65 17.74           H  0.044
HETATM 2119  H81 RII A1141      40.827  11.898 -23.319  0.65 18.03           H  0.045
HETATM 2120  H91 RII A1141      38.484  11.997 -23.996  0.65 16.25           H  0.042
TER
HETATM 2121 RE    RE B   4      25.302  15.275 -15.466  0.19 24.58          Re  0.052
```

```
ANISOU 2121 RE    RE B   4      3023   2531   3786   -493   -207   1448       Re
HETATM 2122 RE    RE B   5      10.745  10.492 -18.788  0.15 17.68           Re  0.044
ANISOU 2122 RE    RE B   5      2456   1984   2276    303   -368    163       Re
HETATM 2123 RE    RE B   6      32.223   7.800  -1.573  0.26 13.85           Re  0.039
ANISOU 2123 RE    RE B   6      1360   1717   2184    -30   -184   -244       Re
HETATM 2124 RE    RE B   7      21.667 -15.171   5.978  0.12 20.15           Re  0.047
ANISOU 2124 RE    RE B   7      2368   3614   1672   -121    -34    279       Re
HETATM 2125 BR    BR C   3      13.232 -13.342   4.597  0.50 19.29           Br  0.046
ANISOU 2125 BR    BR C   3      2271   2400   2660   -484    316   -404       Br
HETATM 2126 BR    BR C   4      16.137  16.048 -19.595  0.50 27.77           Br  0.055
ANISOU 2126 BR    BR C   4      3194   3910   3448   -166    125   -306       Br
HETATM 2127  O   HOH S   1      16.550 -15.311  -1.663  1.00  7.45            O  0.029
ANISOU 2127  O   HOH S   1      1425    652    754   -466    -10    -52        O
HETATM 2128  O   HOH S   2      14.902 -12.901 -13.284  1.00  8.74            O  0.031
ANISOU 2128  O   HOH S   2      1256   1463    602   -537   -126    313        O
HETATM 2129  O   HOH S   3      17.928  -2.976  -8.403  1.00  8.14            O  0.030
ANISOU 2129  O   HOH S   3      1284    534   1273   -115    321    256        O
HETATM 2130  O   HOH S   4      14.448 -11.317   2.499  1.00 10.47            O  0.034
ANISOU 2130  O   HOH S   4      1077   2018    885   -652    134     -8        O
HETATM 2131  O   HOH S   5      12.638  -7.292 -18.771  1.00 11.66            O  0.036
ANISOU 2131  O   HOH S   5      1153   1568   1710   -661    -22    507        O
HETATM 2132  O   HOH S   6      37.209  -1.204 -12.292  1.00 10.26            O  0.034
ANISOU 2132  O   HOH S   6      1366   1383   1150    -92    399   -440        O
HETATM 2133  O   HOH S   7      29.489  -8.755 -19.269  1.00 11.87            O  0.036
ANISOU 2133  O   HOH S   7      1262   1588   1659    367   -255   -174        O
HETATM 2134  O   HOH S   8      34.252  -4.870 -17.833  1.00  9.75            O  0.033
ANISOU 2134  O   HOH S   8       696   1586   1424    432     78    249        O
HETATM 2135  O   HOH S   9      28.917   5.640   1.595  1.00 12.99            O  0.038
ANISOU 2135  O   HOH S   9      1510   1831   1596    -94    439   -278        O
HETATM 2136  O   HOH S  10       7.348 -12.198   3.048  1.00 13.61            O  0.039
ANISOU 2136  O   HOH S  10      1463   1833   1877   -696    510   -977        O
HETATM 2137  O   HOH S  11      40.179   0.721  -8.535  1.00 17.78            O  0.044
ANISOU 2137  O   HOH S  11      1775   2104   2875   -221   -142    -68        O
HETATM 2138  O   HOH S  12      26.657  -2.716   1.675  1.00 17.37            O  0.044
ANISOU 2138  O   HOH S  12      1845   3340   1413    105     96    708        O
HETATM 2139  O   HOH S  13      34.813  -2.002 -17.942  1.00 13.62            O  0.039
ANISOU 2139  O   HOH S  13      1606   2242   1329    478    305     26        O
HETATM 2140  O   HOH S  14      23.047  -5.447  -2.838  1.00 19.06            O  0.046
ANISOU 2140  O   HOH S  14      1376   2710   3156   -468   -369    788        O
HETATM 2141  O   HOH S  15      20.132  13.584 -10.949  1.00 19.15            O  0.046
ANISOU 2141  O   HOH S  15      2536   1212   3529    556   -651    109        O
HETATM 2142  O   HOH S  16      32.126  13.869  -7.231  1.00 16.48            O  0.043
ANISOU 2142  O   HOH S  16      1637   2061   2563   -954   -517    -92        O
HETATM 2143  O   HOH S  17      12.872   1.458  -2.419  1.00 16.18            O  0.042
ANISOU 2143  O   HOH S  17      1585   1941   2621   -220    654    726        O
HETATM 2144  O   HOH S  18      33.477   2.288 -18.223  1.00 14.46            O  0.040
ANISOU 2144  O   HOH S  18      1667   2190   1636     82   -553   -132        O
HETATM 2145  O   HOH S  19      19.368  -7.385 -20.743  1.00 16.70            O  0.043
ANISOU 2145  O   HOH S  19      1703   2149   2492   -796   -570   -193        O
HETATM 2146  O   HOH S  20      13.414  -0.820 -11.659  1.00 14.45            O  0.040
ANISOU 2146  O   HOH S  20      1593   1320   2578    358   -166    457        O
HETATM 2147  O   HOH S  21      27.596  -8.493 -12.208  1.00 14.76            O  0.040
ANISOU 2147  O   HOH S  21      2062   1886   1661    521    122    266        O
HETATM 2148  O   HOH S  22      13.927  -9.622   4.855  1.00 18.20            O  0.045
ANISOU 2148  O   HOH S  22      2978   2457   1479  -1208    214   -678        O
HETATM 2149  O   HOH S  23      15.276  -2.467 -11.173  1.00 14.62            O  0.040
ANISOU 2149  O   HOH S  23      1536   2248   1771    595    -21    928        O
HETATM 2150  O   HOH S  24      12.352  -0.394  -9.183  1.00 15.60            O  0.041
ANISOU 2150  O   HOH S  24      2222   1344   2360   -301    309    504        O
HETATM 2151  O   HOH S  25       8.013  11.635 -16.962  1.00 16.32            O  0.042
ANISOU 2151  O   HOH S  25      1818   1814   2569   -356   -435    256        O
HETATM 2152  O   HOH S  26      19.024  -9.689 -19.481  1.00 15.03            O  0.041
ANISOU 2152  O   HOH S  26      1617   1910   2183   -869    214   -464        O
HETATM 2153  O   HOH S  27      36.010   6.804 -12.879  1.00 16.07            O  0.042
ANISOU 2153  O   HOH S  27      1760   2668   1680   -287    657   -187        O
HETATM 2154  O   HOH S  28      14.791   9.336  -3.982  1.00 22.74            O  0.050
ANISOU 2154  O   HOH S  28      1612   2260   4767    362    914     67        O
HETATM 2155  O   HOH S  29      40.021   3.388  -9.616  1.00 14.53            O  0.040
ANISOU 2155  O   HOH S  29      2308   2146   1065   -244   -319   -124        O
HETATM 2156  O   HOH S  30      28.169  -6.608  -7.910  1.00 18.00            O  0.044
ANISOU 2156  O   HOH S  30      2589   2625   1625   -290    165    236        O
HETATM 2157  O   HOH S  31      17.377 -16.204 -10.502  1.00 18.63            O  0.045
ANISOU 2157  O   HOH S  31      2098   1435   3545   -607    812     41        O
HETATM 2158  O   HOH S  32      38.333   3.884 -19.450  1.00 16.96            O  0.043
ANISOU 2158  O   HOH S  32      2906   2190   1347    236    872   -228        O
HETATM 2159  O   HOH S  33      39.602  -0.118  -5.928  1.00 14.39            O  0.040
ANISOU 2159  O   HOH S  33      2012   2190   1267   1044    240   -266        O
HETATM 2160  O   HOH S  34      14.469 -14.469   9.258  1.00 23.84            O  0.051
```

```
ANISOU 2160  O   HOH S  34     3038   3038   2982   -196    345    345       O
HETATM 2161  O   HOH S  35      28.265   1.743   0.595  1.00 18.73           O  0.045
ANISOU 2161  O   HOH S  35     2400   2382   2333  -1089    129   -195       O
HETATM 2162  O   HOH S  36       8.342  -0.802  -7.677  1.00 20.01           O  0.047
ANISOU 2162  O   HOH S  36     2421   2224   2956   -825   -368    888       O
HETATM 2163  O   HOH S  37      34.383   5.901 -11.067  1.00 15.17           O  0.041
ANISOU 2163  O   HOH S  37      976   3521   1265   -289   -156    259       O
HETATM 2164  O   HOH S  38      13.680   2.319   0.003  1.00 16.85           O  0.043
ANISOU 2164  O   HOH S  38     3163   1804   1437    294    655   -332       O
HETATM 2165  O   HOH S  39      20.039  -1.993 -23.731  1.00 22.10           O  0.049
ANISOU 2165  O   HOH S  39     4157   2560   1679     33   -872    136       O
HETATM 2166  O   HOH S  40      18.207   4.611 -25.071  1.00 19.66           O  0.046
ANISOU 2166  O   HOH S  40     3890   1894   1688    -21   -911    417       O
HETATM 2167  O   HOH S  41      19.859 -17.327 -10.033  1.00 16.16           O  0.042
ANISOU 2167  O   HOH S  41     1703   2568   1867   -353   -112   -769       O
HETATM 2168  O   HOH S  42      24.126 -10.343  -9.398  1.00 13.84           O  0.039
ANISOU 2168  O   HOH S  42     1095   1655   2510    252   -392    720       O
HETATM 2169  O   HOH S  43      20.970   4.256 -26.004  1.00 23.07           O  0.050
ANISOU 2169  O   HOH S  43     4103   2722   1940   -906  -1233    737       O
HETATM 2170  O   HOH S  44      19.398  10.240 -26.419  1.00 24.86           O  0.052
ANISOU 2170  O   HOH S  44     2689   3592   3165  -1232   -337    772       O
HETATM 2171  O   HOH S  45      31.866  10.123  -3.945  1.00 14.80           O  0.040
ANISOU 2171  O   HOH S  45     2022   1475   2129    642   -450   -766       O
HETATM 2172  O   HOH S  46       7.621  -5.418  -9.833  1.00 23.63           O  0.051
ANISOU 2172  O   HOH S  46     2689   3648   2642    756   -696    200       O
HETATM 2173  O   HOH S  47      34.289   3.523 -20.390  1.00 22.96           O  0.050
ANISOU 2173  O   HOH S  47     3579   2752   2395    196     -2   -585       O
HETATM 2174  O   HOH S  48      20.232  -9.649 -17.038  1.00 21.42           O  0.049
ANISOU 2174  O   HOH S  48     3046   2228   2865   -904    928   -274       O
HETATM 2175  O   HOH S  49       8.947  -4.858 -17.392  1.00 23.00           O  0.050
ANISOU 2175  O   HOH S  49     2119   2872   3746    199    122   1927       O
HETATM 2176  O   HOH S  50      10.460 -12.087   6.011  1.00 28.30           O  0.056
ANISOU 2176  O   HOH S  50     4663   4111   1978  -1056    229   -624       O
HETATM 2177  O   HOH S  51      39.193  -4.580  -8.774  1.00 28.61           O  0.056
ANISOU 2177  O   HOH S  51     2708   5202   2963   -679     50    375       O
HETATM 2178  O   HOH S  52      29.891   2.749 -22.833  1.00 24.78           O  0.052
ANISOU 2178  O   HOH S  52     3244   3558   2616   1107   1445   1181       O
HETATM 2179  O   HOH S  53      10.403  -6.973   6.215  1.00 23.22           O  0.051
ANISOU 2179  O   HOH S  53     3314   4097   1414  -1148   -143    651       O
HETATM 2180  O   HOH S  54      33.686   8.992 -11.976  1.00 21.30           O  0.048
ANISOU 2180  O   HOH S  54     2057   3443   2591  -1342     29   -786       O
HETATM 2181  O   HOH S  55      33.342  -0.405 -19.578  1.00 20.53           O  0.048
ANISOU 2181  O   HOH S  55     3427   2575   1797    237    114    470       O
HETATM 2182  O   HOH S  56      21.087  -4.354 -22.658  1.00 22.09           O  0.049
ANISOU 2182  O   HOH S  56     3544   2188   2659    353   -892    141       O
HETATM 2183  O   HOH S  57      25.969  -8.573  -8.048  1.00 21.61           O  0.049
ANISOU 2183  O   HOH S  57     3715   1940   2556   -437   -103   -122       O
HETATM 2184  O   HOH S  58      33.143  -2.262   5.123  1.00 26.52           O  0.054
ANISOU 2184  O   HOH S  58     2995   3770   3310  -1127  -1407   1406       O
HETATM 2185  O   HOH S  59      27.660  -6.421  -5.106  1.00 20.44           O  0.047
ANISOU 2185  O   HOH S  59     3003   2203   2560  -1116    355    659       O
HETATM 2186  O   HOH S  60      33.414  -4.116  -3.346  1.00 26.21           O  0.054
ANISOU 2186  O   HOH S  60     4455   3536   1967   1270   -184    435       O
HETATM 2187  O   HOH S  61      24.995  -6.591  -6.388  1.00 24.54           O  0.052
ANISOU 2187  O   HOH S  61     1874   3647   3802   -841    -21  -1083       O
HETATM 2188  O   HOH S  62      40.290   2.387 -17.963  1.00 24.50           O  0.052
ANISOU 2188  O   HOH S  62     1931   3304   4074    -24     18    121       O
HETATM 2189  O   HOH S  63       6.505  -3.101 -13.866  1.00 29.55           O  0.057
ANISOU 2189  O   HOH S  63     2091   4355   4780   -360  -1336   1155       O
HETATM 2190  O   HOH S  64      13.453   7.604  -7.226  1.00 16.28           O  0.042
ANISOU 2190  O   HOH S  64     1882   2085   2220   -184    265    158       O
HETATM 2191  O   HOH S  65       9.428   1.561  -8.543  1.00 19.82           O  0.047
ANISOU 2191  O   HOH S  65     1514   2589   3430   -589     -7   -246       O
HETATM 2192  O   HOH S  66      38.761  -1.755  -9.240  1.00 23.66           O  0.051
ANISOU 2192  O   HOH S  66     1891   2563   4534   -171   -420   1289       O
HETATM 2193  O   HOH S  67      39.962   1.048 -13.251  1.00 24.00           O  0.051
ANISOU 2193  O   HOH S  67     2642   3296   3181    227    728    322       O
HETATM 2194  O   HOH S  68      17.490  12.598  -3.914  1.00 29.73           O  0.057
ANISOU 2194  O   HOH S  68     3847   1949   5501    585   -119  -1181       O
HETATM 2195  O   HOH S  69      24.142  13.582  -2.766  1.00 18.80           O  0.045
ANISOU 2195  O   HOH S  69     2598   1553   2992    482    396   -943       O
HETATM 2196  O   HOH S  70      26.157  -6.824 -18.765  1.00 25.19           O  0.053
ANISOU 2196  O   HOH S  70     2827   3475   3270    175    265   -715       O
HETATM 2197  O   HOH S  71      14.591 -20.085   2.093  1.00 31.63           O  0.059
ANISOU 2197  O   HOH S  71     5455   2704   3857    870     56  -1582       O
HETATM 2198  O   HOH S  72      18.906  10.404 -23.040  1.00 27.08           O  0.055
ANISOU 2198  O   HOH S  72     3656   2931   3701    476    657   1289       O
HETATM 2199  O   HOH S  73      10.472   2.971 -16.978  1.00 27.39           O  0.055
```

```
ANISOU 2199  O   HOH S  73     4596   2516   3293   -387   -940    473       O
HETATM 2200  O   HOH S  74       5.640  -8.943  -3.849  1.00 21.86           O  0.049
ANISOU 2200  O   HOH S  74     2945   2598   2763   -887    502     39       O
HETATM 2201  O   HOH S  75      22.428   4.840   4.313  1.00 23.43           O  0.051
ANISOU 2201  O   HOH S  75     3276   3750   1877   -693    409   -519       O
HETATM 2202  O   HOH S  76      16.529   6.646  -0.213  1.00 22.67           O  0.050
ANISOU 2202  O   HOH S  76     4047   2543   2023    338    792   -527       O
HETATM 2203  O   HOH S  77      32.483   2.528 -22.319  1.00 28.23           O  0.056
ANISOU 2203  O   HOH S  77     4268   2988   3469    849    803    909       O
HETATM 2204  O   HOH S  78       5.533  -9.920   3.194  1.00 35.08           O  0.062
ANISOU 2204  O   HOH S  78     4355   4338   4636  -2133    481   -665       O
HETATM 2205  O   HOH S  79      10.562   2.764  -2.566  1.00 26.00           O  0.053
ANISOU 2205  O   HOH S  79     1972   3380   4527    851   1034   -318       O
HETATM 2206  O   HOH S  80      12.832  15.613 -30.556  1.00 28.15           O  0.056
ANISOU 2206  O   HOH S  80     3213   3358   4124    622    140   -581       O
HETATM 2207  O   HOH S  81      37.559   0.083  -2.187  1.00 24.23           O  0.052
ANISOU 2207  O   HOH S  81     3031   3083   3091   -596  -1068    990       O
HETATM 2208  O   HOH S  82      11.662 -20.024   2.670  1.00 29.95           O  0.057
ANISOU 2208  O   HOH S  82     3232   3925   4221  -1611  -1764   1768       O
HETATM 2209  O   HOH S  84      18.342 -23.220  -5.574  1.00 29.64           O  0.057
ANISOU 2209  O   HOH S  84     3167   4954   3139    411   -111   1479       O
HETATM 2210  O   HOH S  85      12.081  -4.374 -22.664  1.00 27.76           O  0.055
ANISOU 2210  O   HOH S  85     2157   4953   3437   -234  -1379    256       O
HETATM 2211  O   HOH S  86      26.450   0.021   1.972  1.00 27.69           O  0.055
ANISOU 2211  O   HOH S  86     3967   3723   2831  -2076  -1011    281       O
HETATM 2212  O   HOH S  88      16.425  13.269 -21.957  1.00 33.97           O  0.061
ANISOU 2212  O   HOH S  88     3265   4800   4843   1929    868   1863       O
HETATM 2213  O   HOH S  89      26.325  16.683 -13.861  1.00 29.07           O  0.057
ANISOU 2213  O   HOH S  89     2899   3513   4633   1096     42    476       O
HETATM 2214  O   HOH S  92      13.739  -7.748 -23.757  1.00 31.07           O  0.058
ANISOU 2214  O   HOH S  92     6062   3759   1983   -657  -1165    432       O
HETATM 2215  O   HOH S  93      26.901 -16.736  -5.153  1.00 28.42           O  0.056
ANISOU 2215  O   HOH S  93     2132   5604   3063    247   1008   -531       O
HETATM 2216  O   HOH S  94      42.602   3.757 -11.237  1.00 23.32           O  0.051
ANISOU 2216  O   HOH S  94     2427   3439   2994   -406    136    483       O
HETATM 2217  O   HOH S  95      24.727  -3.565  -3.377  1.00 26.47           O  0.054
ANISOU 2217  O   HOH S  95     2132   3156   4769   -922    302  -1558       O
HETATM 2218  O   HOH S  96      28.233  -9.008  -9.219  1.00 28.47           O  0.056
ANISOU 2218  O   HOH S  96     3599   3155   4064    502   1082    -28       O
HETATM 2219  O   HOH S  97      15.208   8.973  -1.432  1.00 31.82           O  0.059
ANISOU 2219  O   HOH S  97     5431   2858   3802   1488   1618    310       O
HETATM 2220  O   HOH S  99      14.127 -12.432   7.433  1.00 30.64           O  0.058
ANISOU 2220  O   HOH S  99     5888   4088   1664   -742    520     -8       O
HETATM 2221  O   HOH S 100      12.749  10.442  -7.243  1.00 31.53           O  0.059
ANISOU 2221  O   HOH S 100     4276   3300   4404   1304   2125    527       O
HETATM 2222  O   HOH S 102      21.715 -14.851   3.584  1.00 30.20           O  0.058
ANISOU 2222  O   HOH S 102     3298   4097   4081    273   -492   -451       O
HETATM 2223  O   HOH S 104      21.908   5.974 -27.882  1.00 32.98           O  0.060
ANISOU 2223  O   HOH S 104     5300   5385   1847  -1533   -328    617       O
HETATM 2224  O   HOH S 105      13.544  -5.333 -24.876  1.00 30.56           O  0.058
ANISOU 2224  O   HOH S 105     4563   4993   2055   -389   -806    588       O
HETATM 2225  O   HOH S 106      25.384  -7.379  -1.958  1.00 34.33           O  0.061
ANISOU 2225  O   HOH S 106     3709   3893   5440   -584   1265    396       O
HETATM 2226  O   HOH S 109      24.582  13.785 -29.025  1.00 31.28           O  0.059
ANISOU 2226  O   HOH S 109     6030   3289   2567   1273    969    730       O
HETATM 2227  O   HOH S 110      19.936 -17.290   3.050  1.00 25.40           O  0.053
ANISOU 2227  O   HOH S 110     3938   2511   3200   -224    -70   -881       O
HETATM 2228  O   HOH S 111       8.968 -16.522   5.230  1.00 25.18           O  0.053
ANISOU 2228  O   HOH S 111     1951   4523   3095  -1108    529    504       O
HETATM 2229  O   HOH S 112      17.891 -18.520   1.735  1.00 30.37           O  0.058
ANISOU 2229  O   HOH S 112     4900   3955   2683    298   1175    232       O
HETATM 2230  O   HOH S 113      29.863  17.636  -9.017  1.00 32.12           O  0.059
ANISOU 2230  O   HOH S 113     4062   1922   6219     47   -214    343       O
HETATM 2231  O   HOH S 115      20.080   7.938   1.590  1.00 31.07           O  0.058
ANISOU 2231  O   HOH S 115     3055   2555   6197    499   1407   -889       O
HETATM 2232  O   HOH S 116       6.026  -4.027 -11.060  1.00 37.01           O  0.064
ANISOU 2232  O   HOH S 116     3192   6276   4594  -1431    824    656       O
HETATM 2233  O   HOH S 118      37.988  10.629 -26.752  1.00 16.43           O  0.042
ANISOU 2233  O   HOH S 118     3083   2038   1121    488    474    514       O
HETATM 2234  O   HOH S 119      10.355 -22.353   2.212  1.00 28.42           O  0.056
ANISOU 2234  O   HOH S 119     2142   3872   4786    842    124    364       O
HETATM 2235  O   HOH S 120      19.168  -8.335 -23.350  1.00 31.54           O  0.059
ANISOU 2235  O   HOH S 120     4306   5353   2326   -242  -1213    335       O
HETATM 2236  O   HOH S 121       7.266   0.647  -5.138  1.00 27.44           O  0.055
ANISOU 2236  O   HOH S 121     2958   3480   3990   1144   -125    -33       O
HETATM 2237  O   HOH S 122      21.860   0.164 -24.914  1.00 30.56           O  0.058
ANISOU 2237  O   HOH S 122     3394   5754   2462  -1704    233   -729       O
HETATM 2238  O   HOH S 123      15.216  -4.209   8.477  1.00 33.03           O  0.060
```

```
ANISOU 2238  O   HOH S 123     3274   4679   4596     68   1611   -636       O
HETATM 2239  O   HOH S 125       4.822  -6.190 -12.568  1.00 34.53           O  0.062
ANISOU 2239  O   HOH S 125     6174   2863   4083   -217   1118     84       O
HETATM 2240  O   HOH S 126      24.470 -12.101   0.770  1.00 29.47           O  0.057
ANISOU 2240  O   HOH S 126     3031   4922   3243  -1725   -611   -800       O
HETATM 2241  O   HOH S 127      25.340  -4.738   0.411  1.00 33.94           O  0.061
ANISOU 2241  O   HOH S 127     3204   5407   4283    270   1264  -2024       O
HETATM 2242  O   HOH S 128      41.553  -5.896  -8.585  1.00 33.64           O  0.061
ANISOU 2242  O   HOH S 128     4501   4187   4093  -1577    376     40       O
HETATM 2243  O   HOH S 129       6.667  -4.955 -19.023  1.00 28.48           O  0.056
ANISOU 2243  O   HOH S 129     3146   2630   5046    639   -179   -531       O
HETATM 2244  O   HOH S 130      21.269 -20.952  -2.934  1.00 36.93           O  0.064
ANISOU 2244  O   HOH S 130     5367   3088   5577    909  -1679    956       O
HETATM 2245  O   HOH S 132       7.087  -1.019   1.586  1.00 31.60           O  0.059
ANISOU 2245  O   HOH S 132     3371   5252   3383    576   1421  -1297       O
HETATM 2246  O   HOH S 136      38.732   8.144 -12.833  1.00 33.62           O  0.061
ANISOU 2246  O   HOH S 136     5527   3786   3462  -1200  -1017   -692       O
HETATM 2247  O   HOH S 139      13.209  -0.041 -23.248  1.00 32.05           O  0.059
ANISOU 2247  O   HOH S 139     4682   3501   3993   -250  -1445    998       O
HETATM 2248  O   HOH S 142       7.577  14.047 -17.200  1.00 30.63           O  0.058
ANISOU 2248  O   HOH S 142     5417   2523   3698   1229   -478    154       O
HETATM 2249  O   HOH S 146      26.162 -15.654  -7.449  1.00 31.91           O  0.059
ANISOU 2249  O   HOH S 146     3926   4337   3859   1263    139    602       O
HETATM 2250  O   HOH S 170      39.020   9.055 -17.993  1.00 30.37           O  0.058
ANISOU 2250  O   HOH S 170     4350   4223   2966  -1129  -1816   1162       O
HETATM 2251  O   HOH S 171      25.748 -11.372  -5.631  1.00 27.46           O  0.055
ANISOU 2251  O   HOH S 171     2322   3597   4513   -557   -240   2278       O
HETATM 2252  O   HOH S 172      39.861  10.022 -15.817  1.00 37.91           O  0.065
ANISOU 2252  O   HOH S 172     6063   4479   3862    829    667    125       O
HETATM 2253  O   HOH S 178       8.364  -4.228   2.701  1.00 22.66           O  0.050
ANISOU 2253  O   HOH S 178     1928   4187   2496    313   1084   -128       O
HETATM 2254  O   HOH S 179      29.969   7.598   5.354  1.00 30.76           O  0.058
ANISOU 2254  O   HOH S 179     3875   3657   4156    840   1243   -297       O
HETATM 2255  O   HOH S 180      25.955  -9.366 -23.180  1.00 34.30           O  0.061
ANISOU 2255  O   HOH S 180     3871   5130   4031   1799    -13   1003       O
HETATM 2256  O   HOH S 181      29.360   8.358   2.911  1.00 35.94           O  0.063
ANISOU 2256  O   HOH S 181     2823   5300   5532  -1479    716  -1869       O
HETATM 2257  O   HOH S 183      21.387  -8.364  11.351  1.00 36.10           O  0.063
ANISOU 2257  O   HOH S 183     4683   3932   5100  -1993    -57   1669       O
HETATM 2258  O   HOH S 189      34.800   9.781 -16.189  1.00 16.37           O  0.042
ANISOU 2258  O   HOH S 189     2093   1460   2668   -327     -3    499       O
HETATM 2259  O   HOH S 191      36.515  11.332 -16.079  1.00 29.15           O  0.057
ANISOU 2259  O   HOH S 191     4114   3714   3249  -1885   -397    337       O
HETATM 2260  O   HOH S 196      23.858  -7.266  12.327  1.00 28.71           O  0.056
ANISOU 2260  O   HOH S 196     3714   4954   2239    939   1062    795       O
HETATM 2261  O   HOH S 199      18.451  11.248  -0.184  1.00 32.04           O  0.059
ANISOU 2261  O   HOH S 199     6385   2530   3258    863   2023   -100       O
END
```
