# Supplementary material for: Time-series analysis of rhenium(I) organometallic covalent binding to a model protein for drug development
Source: IUCrJ. 2024 Apr 19;11(Pt 3):359–73. doi: 10.1107/S2052252524002598 (PMC11067751; doi:10.1107/S2052252524002598)
Supplement: Supplementary file 6 [file m-11-00359-sup6.zip › Week 18 - P1P8_1/P1P8_1_refine_47.pdf]

```
REMARK   3
REMARK   3 REFINEMENT.
REMARK   3   PROGRAM     : PHENIX (1.20.1_4487: ???)
REMARK   3   AUTHORS     : Adams,Afonine,Bunkoczi,Burnley,Chen,Dar,Davis,
REMARK   3               : Draizen,Echols,Gildea,Gros,Grosse-Kunstleve,Headd,
REMARK   3               : Hintze,Hung,Ioerger,Liebschner,McCoy,McKee,Moriarty,
REMARK   3               : Oeffner,Poon,Read,Richardson,Richardson,Sacchettini,
REMARK   3               : Sauter,Sobolev,Storoni,Terwilliger,Williams,Zwart
REMARK   3
REMARK   3  X-RAY DATA.
REMARK   3
REMARK   3  REFINEMENT TARGET : ML
REMARK   3
REMARK   3  DATA USED IN REFINEMENT.
REMARK   3   RESOLUTION RANGE HIGH (ANGSTROMS) : 1.21
REMARK   3   RESOLUTION RANGE LOW  (ANGSTROMS) : 28.72
REMARK   3   MIN(FOBS/SIGMA_FOBS)              : 1.35
REMARK   3   COMPLETENESS FOR RANGE        (%) : 99.42
REMARK   3   NUMBER OF REFLECTIONS             : 72919
REMARK   3   NUMBER OF REFLECTIONS (NON-ANOMALOUS) : 38746
REMARK   3
REMARK   3  FIT TO DATA USED IN REFINEMENT.
REMARK   3   R VALUE     (WORKING + TEST SET) : 0.1497
REMARK   3   R VALUE            (WORKING SET) : 0.1486
REMARK   3   FREE R VALUE                     : 0.1711
REMARK   3   FREE R VALUE TEST SET SIZE   (%) : 5.16
REMARK   3   FREE R VALUE TEST SET COUNT      : 3765
REMARK   3
REMARK   3  FIT TO DATA USED IN REFINEMENT (IN BINS).
REMARK   3   BIN  RESOLUTION RANGE  COMPL.    NWORK NFREE   RWORK  RFREE  CCWORK CCFREE
REMARK   3     1   28.72 -    3.62    0.99     2567   134  0.1628 0.1811   0.924  0.920
REMARK   3     2    3.62 -    2.87    1.00     2564   141  0.1630 0.1797   0.931  0.921
REMARK   3     3    2.87 -    2.51    1.00     2583   142  0.1564 0.1806   0.935  0.904
REMARK   3     4    2.51 -    2.28    1.00     2582   145  0.1476 0.1583   0.942  0.946
REMARK   3     5    2.28 -    2.12    1.00     2570   137  0.1434 0.1534   0.946  0.955
REMARK   3     6    2.12 -    1.99    1.00     2578   142  0.1476 0.1776   0.942  0.913
REMARK   3     7    1.99 -    1.89    1.00     2583   137  0.1395 0.1780   0.949  0.925
REMARK   3     8    1.89 -    1.81    1.00     2581   142  0.1363 0.1926   0.954  0.922
REMARK   3     9    1.81 -    1.74    1.00     2583   139  0.1293 0.1653   0.958  0.941
REMARK   3    10    1.74 -    1.68    1.00     2552   141  0.1377 0.1576   0.952  0.944
REMARK   3    11    1.68 -    1.63    1.00     2573   135  0.1204 0.1221   0.964  0.973
REMARK   3    12    1.63 -    1.58    1.00     2599   140  0.1223 0.1561   0.958  0.942
REMARK   3    13    1.58 -    1.54    1.00     2555   141  0.1263 0.1324   0.961  0.951
REMARK   3    14    1.54 -    1.50    1.00     2579   140  0.1236 0.1451   0.960  0.938
REMARK   3    15    1.50 -    1.47    1.00     2587   139  0.1249 0.1358   0.960  0.948
REMARK   3    16    1.47 -    1.44    1.00     2556   140  0.1300 0.1406   0.959  0.931
REMARK   3    17    1.44 -    1.41    1.00     2608   144  0.1399 0.1793   0.950  0.918
REMARK   3    18    1.41 -    1.38    1.00     2545   133  0.1350 0.1684   0.954  0.924
REMARK   3    19    1.38 -    1.36    1.00     2582   143  0.1413 0.1740   0.947  0.941
REMARK   3    20    1.36 -    1.33    1.00     2556   134  0.1355 0.1725   0.956  0.925
REMARK   3    21    1.33 -    1.31    1.00     2608   144  0.1416 0.1764   0.942  0.913
REMARK   3    22    1.31 -    1.29    1.00     2545   141  0.1442 0.2017   0.944  0.905
REMARK   3    23    1.29 -    1.27    1.00     2620   140  0.1493 0.1438   0.941  0.945
REMARK   3    24    1.27 -    1.25    1.00     2560   140  0.1658 0.1924   0.931  0.915
REMARK   3    25    1.25 -    1.24    0.99     2543   144  0.1716 0.1894   0.929  0.903
REMARK   3    26    1.24 -    1.22    0.97     2494   138  0.1941 0.2435   0.917  0.876
REMARK   3    27    1.22 -    1.21    0.90     2301   129  0.2132 0.2772   0.901  0.877
REMARK   3
REMARK   3  BULK SOLVENT MODELLING.
REMARK   3   METHOD USED        : FLAT BULK SOLVENT MODEL
REMARK   3   SOLVENT RADIUS     : 1.10
REMARK   3   SHRINKAGE RADIUS   : 0.90
REMARK   3   GRID STEP FACTOR   : 4.00
REMARK   3
REMARK   3  ERROR ESTIMATES.
REMARK   3   COORDINATE ERROR (MAXIMUM-LIKELIHOOD BASED)     : 0.10
REMARK   3   PHASE ERROR (DEGREES, MAXIMUM-LIKELIHOOD BASED) : 15.33
REMARK   3
REMARK   3  STRUCTURE FACTORS CALCULATION ALGORITHM : FFT
REMARK   3  B VALUES.
REMARK   3   FROM WILSON PLOT           (A**2) : 14.66
REMARK   3
REMARK   3  GEOMETRY RESTRAINTS LIBRARY: GEOSTD + MONOMER LIBRARY + CDL V1.2
REMARK   3  DEVIATIONS FROM IDEAL VALUES - RMSD. RMSZ FOR BONDS AND ANGLES.
REMARK   3    BOND      :  0.011   0.081   1153  Z= 0.710
REMARK   3    ANGLE     :  1.268   6.693   1574  Z= 0.737
REMARK   3    CHIRALITY :  0.084   0.226    151
REMARK   3    PLANARITY :  0.011   0.076    204
```

```
REMARK   3    DIHEDRAL  : 14.518  83.694    408
REMARK   3    MIN NONBONDED DISTANCE : 2.137
REMARK   3
REMARK   3  MOLPROBITY STATISTICS.
REMARK   3    ALL-ATOM CLASHSCORE : 2.76
REMARK   3    RAMACHANDRAN PLOT:
REMARK   3      OUTLIERS :  0.00 %
REMARK   3      ALLOWED  :  1.57 %
REMARK   3      FAVORED  : 98.43 %
REMARK   3    ROTAMER OUTLIERS :  0.90 %
REMARK   3    CBETA DEVIATIONS :  0.00 %
REMARK   3    PEPTIDE PLANE:
REMARK   3      CIS-PROLINE     : 0.00 %
REMARK   3      CIS-GENERAL     : 0.00 %
REMARK   3      TWISTED PROLINE : 0.00 %
REMARK   3      TWISTED GENERAL : 0.00 %
REMARK   3
REMARK   3  RAMA-Z (RAMACHANDRAN PLOT Z-SCORE):
REMARK   3  INTERPRETATION: BAD |RAMA-Z| > 3; SUSPICIOUS 2 < |RAMA-Z| < 3; GOOD |RAMA-Z| < 2.
REMARK   3  SCORES FOR WHOLE/HELIX/SHEET/LOOP ARE SCALED INDEPENDENTLY;
REMARK   3  THEREFORE, THE VALUES ARE NOT RELATED IN A SIMPLE MANNER.
REMARK   3    WHOLE:  0.55 (0.69), RESIDUES: 143
REMARK   3    HELIX:  0.08 (0.69), RESIDUES: 48
REMARK   3    SHEET: -1.35 (1.09), RESIDUES: 15
REMARK   3    LOOP :  1.16 (0.74), RESIDUES: 80
REMARK   3
REMARK   3                  min    max   mean <Bi,j>   iso aniso
REMARK   3     Overall:   10.61  52.92  19.63   1.50    15  1228
REMARK   3     Protein:   10.61  52.52  18.34   1.47     0  1056
REMARK   3     Water:     13.44  43.72  27.78    N/A     3    99
REMARK   3     Other:     11.29  52.92  25.83    N/A    12    73
REMARK   3     Chain  A:  10.61  52.52  19.45    N/A     5  1217
REMARK   3     Chain  C:  14.70  14.70  14.70    N/A     1     0
REMARK   3     Chain  B:  17.85  52.92  28.05    N/A     6     0
REMARK   3     Chain  W:  20.61  38.38  32.02    N/A     3    11
REMARK   3     Histogram:
REMARK   3         Values      Number of atoms
REMARK   3      10.61 - 14.84       368
REMARK   3      14.84 - 19.07       379
REMARK   3      19.07 - 23.30       213
REMARK   3      23.30 - 27.53       111
REMARK   3      27.53 - 31.76        60
REMARK   3      31.76 - 35.99        60
REMARK   3      35.99 - 40.23        25
REMARK   3      40.23 - 44.46        16
REMARK   3      44.46 - 48.69         6
REMARK   3      48.69 - 52.92         5
REMARK   3
REMARK   3
LINK         NE2 HIS A  15                RE1  RI3 A2152
LINK         OD2 ASP A 101                RE1 ARII A2148
LINK         OD2 ASP A 101                RE1 BRII A2148
LINK         OD2 ASP A 119                RE1  RII A2149
SSBOND   1 CYS A    6    CYS A  127
SSBOND   2 CYS A   30    CYS A  115
SSBOND   3 CYS A   64    CYS A   80
SSBOND   4 CYS A   76    CYS A   94
CRYST1   81.237   81.237   37.221  90.00  90.00  90.00 P 43 21 2
SCALE1      0.012310  0.000000  0.000000        0.00000
SCALE2      0.000000  0.012310  0.000000        0.00000
SCALE3      0.000000  0.000000  0.026867        0.00000
ATOM      1  N   LYS A   1       4.820  11.766   9.861  1.00 20.41           N  0.024
ANISOU    1  N   LYS A   1     2327   2625   2802    581   -644   -723       N
ATOM      2  CA  LYS A   1       3.858  12.111   8.787  1.00 20.21           C  0.024
ANISOU    2  CA  LYS A   1     2335   2591   2754    642   -761   -635       C
ATOM      3  C   LYS A   1       3.866  13.616   8.604  1.00 18.57           C  0.023
ANISOU    3  C   LYS A   1     2233   2509   2315    640   -818   -774       C
ATOM      4  O   LYS A   1       3.852  14.348   9.592  1.00 18.56           O  0.023
ANISOU    4  O   LYS A   1     2266   2463   2323    467   -818   -725       O
ATOM      5  CB  LYS A   1       2.462  11.671   9.198  1.00 21.50           C  0.024
ANISOU    5  CB  LYS A   1     2424   2630   3116    594   -787   -611       C
ATOM      6  CG  LYS A   1       1.358  12.045   8.220  1.00 22.14           C  0.025
ANISOU    6  CG  LYS A   1     2483   2731   3199    635   -797   -789       C
ATOM      7  CD  LYS A   1       0.019  11.441   8.647  1.00 23.60           C  0.025
ANISOU    7  CD  LYS A   1     2681   2860   3424    610   -724   -982       C
ATOM      8  CE  LYS A   1      -1.124  11.956   7.802  1.00 26.12           C  0.027
ANISOU    8  CE  LYS A   1     2948   3171   3806    611   -673   -912       C
ATOM      9  NZ  LYS A   1      -0.878  11.786   6.349  1.00 29.09           N  0.028
```

```
ANISOU    9  NZ  LYS A   1     3192   3450   4411    569   -607   -689       N
ATOM     10  H1  LYS A   1       4.838  10.883   9.971  1.00 24.49           H  0.026
ATOM     11  H2  LYS A   1       5.632  12.053   9.636  1.00 24.49           H  0.026
ATOM     12  H3  LYS A   1       4.568  12.154  10.621  1.00 24.49           H  0.026
ATOM     13  HA  LYS A   1       4.105  11.679   7.954  1.00 24.25           H  0.026
ATOM     14  HB2 LYS A   1       2.457  10.705   9.290  1.00 25.80           H  0.027
ATOM     15  HB3 LYS A   1       2.248  12.080  10.051  1.00 25.80           H  0.027
ATOM     16  HG2 LYS A   1       1.261  13.010   8.195  1.00 26.57           H  0.027
ATOM     17  HG3 LYS A   1       1.580  11.705   7.339  1.00 26.57           H  0.027
ATOM     18  HD2 LYS A   1       0.058  10.477   8.549  1.00 28.32           H  0.028
ATOM     19  HD3 LYS A   1      -0.157  11.677   9.571  1.00 28.32           H  0.028
ATOM     20  HE2 LYS A   1      -1.931  11.468   8.029  1.00 31.35           H  0.029
ATOM     21  HE3 LYS A   1      -1.247  12.902   7.978  1.00 31.35           H  0.029
ATOM     22  HZ1 LYS A   1      -1.586  12.064   5.887  1.00 34.91           H  0.031
ATOM     23  HZ2 LYS A   1      -0.167  12.261   6.103  1.00 34.91           H  0.031
ATOM     24  HZ3 LYS A   1      -0.731  10.928   6.164  1.00 34.91           H  0.031
ATOM     25  N   VAL A   2       3.960  14.060   7.358  1.00 18.99           N  0.023
ANISOU   25  N   VAL A   2     2270   2677   2270    681   -629   -880       N
ATOM     26  CA  VAL A   2       3.806  15.467   7.011  1.00 18.78           C  0.023
ANISOU   26  CA  VAL A   2     2371   2862   1905    585   -708   -856       C
ATOM     27  C   VAL A   2       2.379  15.647   6.506  1.00 19.54           C  0.023
ANISOU   27  C   VAL A   2     2444   2910   2072    507   -723  -1009       C
ATOM     28  O   VAL A   2       2.040  15.197   5.411  1.00 21.08           O  0.024
ANISOU   28  O   VAL A   2     2664   3028   2317    572   -802   -962       O
ATOM     29  CB  VAL A   2       4.848  15.943   5.989  1.00 19.86           C  0.023
ANISOU   29  CB  VAL A   2     2496   3087   1964    617   -426   -673       C
ATOM     30  CG1 VAL A   2       4.575  17.409   5.639  1.00 21.16           C  0.024
ANISOU   30  CG1 VAL A   2     2666   3292   2081    566   -300   -384       C
ATOM     31  CG2 VAL A   2       6.290  15.772   6.536  1.00 20.42           C  0.024
ANISOU   31  CG2 VAL A   2     2564   3203   1990    546   -384   -616       C
ATOM     32  H   VAL A   2       4.116  13.554   6.680  1.00 22.79           H  0.025
ATOM     33  HA  VAL A   2       3.909  16.004   7.812  1.00 22.54           H  0.025
ATOM     34  HB  VAL A   2       4.766  15.416   5.179  1.00 23.84           H  0.026
ATOM     35 HG11 VAL A   2       5.343  17.767   5.168  1.00 25.39           H  0.026
ATOM     36 HG12 VAL A   2       3.788  17.461   5.074  1.00 25.39           H  0.026
ATOM     37 HG13 VAL A   2       4.430  17.907   6.459  1.00 25.39           H  0.026
ATOM     38 HG21 VAL A   2       6.920  16.091   5.870  1.00 24.50           H  0.026
ATOM     39 HG22 VAL A   2       6.381  16.289   7.352  1.00 24.50           H  0.026
ATOM     40 HG23 VAL A   2       6.449  14.833   6.719  1.00 24.50           H  0.026
ATOM     41  N   PHE A   3       1.545  16.311   7.308  1.00 18.68           N  0.023
ANISOU   41  N   PHE A   3     2241   2873   1985    573   -702   -801       N
ATOM     42  CA  PHE A   3       0.156  16.528   6.934  1.00 19.33           C  0.023
ANISOU   42  CA  PHE A   3     2236   2869   2238    542   -768   -924       C
ATOM     43  C   PHE A   3       0.050  17.642   5.897  1.00 19.52           C  0.023
ANISOU   43  C   PHE A   3     2274   3097   2048    547  -1028   -907       C
ATOM     44  O   PHE A   3       0.847  18.583   5.879  1.00 20.06           O  0.023
ANISOU   44  O   PHE A   3     2286   3170   2164    665   -896   -917       O
ATOM     45  CB  PHE A   3      -0.687  16.979   8.131  1.00 20.24           C  0.024
ANISOU   45  CB  PHE A   3     2277   2824   2590    435   -712   -872       C
ATOM     46  CG  PHE A   3      -1.196  15.880   9.019  1.00 20.25           C  0.024
ANISOU   46  CG  PHE A   3     2302   2790   2603    449   -840   -786       C
ATOM     47  CD1 PHE A   3      -0.400  15.303   9.982  1.00 20.82           C  0.024
ANISOU   47  CD1 PHE A   3     2252   2834   2824    425   -796   -644       C
ATOM     48  CD2 PHE A   3      -2.514  15.454   8.907  1.00 20.92           C  0.024
ANISOU   48  CD2 PHE A   3     2409   2777   2762    447   -852   -803       C
ATOM     49  CE1 PHE A   3      -0.904  14.305  10.811  1.00 21.32           C  0.024
ANISOU   49  CE1 PHE A   3     2314   2881   2904    449   -791   -562       C
ATOM     50  CE2 PHE A   3      -3.016  14.472   9.726  1.00 21.59           C  0.024
ANISOU   50  CE2 PHE A   3     2448   2830   2926    382   -896   -792       C
ATOM     51  CZ  PHE A   3      -2.213  13.896  10.688  1.00 21.97           C  0.025
ANISOU   51  CZ  PHE A   3     2436   2833   3078    445   -881   -788       C
ATOM     52  H   PHE A   3       1.762  16.643   8.071  1.00 22.42           H  0.025
ATOM     53  HA  PHE A   3      -0.223  15.714   6.567  1.00 23.19           H  0.025
ATOM     54  HB2 PHE A   3      -0.149  17.571   8.680  1.00 24.29           H  0.026
ATOM     55  HB3 PHE A   3      -1.458  17.461   7.795  1.00 24.29           H  0.026
ATOM     56  HD1 PHE A   3       0.483  15.580  10.078  1.00 24.98           H  0.026
ATOM     57  HD2 PHE A   3      -3.067  15.843   8.268  1.00 25.10           H  0.026
ATOM     58  HE1 PHE A   3      -0.356  13.918  11.455  1.00 25.58           H  0.026
ATOM     59  HE2 PHE A   3      -3.899  14.196   9.632  1.00 25.91           H  0.027
ATOM     60  HZ  PHE A   3      -2.547  13.223  11.236  1.00 26.37           H  0.027
ATOM     61  N   GLY A   4      -0.957  17.538   5.034  1.00 20.08           N  0.023
ANISOU   61  N   GLY A   4     2356   3229   2045    558   -963   -683       N
ATOM     62  CA  GLY A   4      -1.409  18.699   4.310  1.00 20.20           C  0.024
ANISOU   62  CA  GLY A   4     2350   3278   2048    509   -854   -426       C
ATOM     63  C   GLY A   4      -2.293  19.569   5.176  1.00 18.76           C  0.023
ANISOU   63  C   GLY A   4     2232   3082   1812    345   -779   -419       C
ATOM     64  O   GLY A   4      -2.815  19.115   6.192  1.00 19.36           O  0.023
ANISOU   64  O   GLY A   4     2166   3044   2145    332   -646   -224       O
```

```
ATOM     65  H   GLY A   4      -1.385  16.813   4.857  1.00 24.10           H  0.026
ATOM     66  HA2 GLY A   4      -0.646  19.222   4.018  1.00 24.24           H  0.026
ATOM     67  HA3 GLY A   4      -1.914  18.422   3.529  1.00 24.24           H  0.026
ATOM     68  N   ARG A   5      -2.410  20.837   4.773  1.00 19.46           N  0.023
ANISOU   68  N   ARG A   5     2362   3007   2026    194   -632   -405       N
ATOM     69  CA  ARG A   5      -3.149  21.816   5.558  1.00 19.32           C  0.023
ANISOU   69  CA  ARG A   5     2326   2880   2135    -14   -573   -333       C
ATOM     70  C   ARG A   5      -4.589  21.372   5.802  1.00 18.79           C  0.023
ANISOU   70  C   ARG A   5     2230   2687   2221      2   -631   -396       C
ATOM     71  O   ARG A   5      -5.051  21.337   6.947  1.00 18.47           O  0.022
ANISOU   71  O   ARG A   5     2294   2488   2236     -3   -798   -231       O
ATOM     72  CB  ARG A   5      -3.089  23.159   4.835  1.00 20.88           C  0.024
ANISOU   72  CB  ARG A   5     2444   3030   2460   -151   -491   -141       C
ATOM     73  CG  ARG A   5      -3.946  24.228   5.392  1.00 21.16           C  0.024
ANISOU   73  CG  ARG A   5     2518   3115   2406   -291   -111   -200       C
ATOM     74  CD  ARG A   5      -3.727  25.515   4.597  1.00 22.84           C  0.025
ANISOU   74  CD  ARG A   5     2620   3211   2846   -313    -27   -286       C
ATOM     75  NE  ARG A   5      -4.199  25.446   3.213  1.00 23.97           N  0.026
ANISOU   75  NE  ARG A   5     2741   3409   2957   -166      3   -339       N
ATOM     76  CZ  ARG A   5      -5.466  25.585   2.821  1.00 24.61           C  0.026
ANISOU   76  CZ  ARG A   5     2937   3678   2736   -155   -210   -406       C
ATOM     77  NH1 ARG A   5      -6.447  25.794   3.683  1.00 24.07           N  0.026
ANISOU   77  NH1 ARG A   5     2857   3592   2697   -234   -308   -513       N
ATOM     78  NH2 ARG A   5      -5.755  25.516   1.525  1.00 25.80           N  0.027
ANISOU   78  NH2 ARG A   5     3134   3902   2766   -118   -111   -170       N
ATOM     79  H   ARG A   5      -2.069  21.151   4.048  1.00 23.36           H  0.025
ATOM     80  HA  ARG A   5      -2.718  21.920   6.421  1.00 23.19           H  0.025
ATOM     81  HB2 ARG A   5      -2.173  23.479   4.860  1.00 25.06           H  0.026
ATOM     82  HB3 ARG A   5      -3.358  23.022   3.913  1.00 25.06           H  0.026
ATOM     83  HG2 ARG A   5      -4.880  23.977   5.317  1.00 25.39           H  0.026
ATOM     84  HG3 ARG A   5      -3.708  24.390   6.318  1.00 25.39           H  0.026
ATOM     85  HD2 ARG A   5      -4.201  26.238   5.036  1.00 27.41           H  0.027
ATOM     86  HD3 ARG A   5      -2.777  25.711   4.576  1.00 27.41           H  0.027
ATOM     87  HE  ARG A   5      -3.610  25.304   2.602  1.00 28.76           H  0.028
ATOM     88 HH11 ARG A   5      -6.279  25.843   4.525  1.00 28.89           H  0.028
ATOM     89 HH12 ARG A   5      -7.255  25.880   3.401  1.00 28.89           H  0.028
ATOM     90 HH21 ARG A   5      -5.129  25.381   0.951  1.00 30.96           H  0.029
ATOM     91 HH22 ARG A   5      -6.569  25.604   1.261  1.00 30.96           H  0.029
ATOM     92  N   CYS A   6      -5.327  21.056   4.741  1.00 19.03           N  0.023
ANISOU   92  N   CYS A   6     2255   2880   2096     34   -868   -353       N
ATOM     93  CA  CYS A   6      -6.729  20.681   4.946  1.00 19.21           C  0.023
ANISOU   93  CA  CYS A   6     2305   2872   2121     88   -972   -526       C
ATOM     94  C   CYS A   6      -6.866  19.335   5.664  1.00 19.33           C  0.023
ANISOU   94  C   CYS A   6     2233   2743   2370    155   -898   -586       C
ATOM     95  O   CYS A   6      -7.831  19.124   6.411  1.00 19.40           O  0.023
ANISOU   95  O   CYS A   6     2204   2652   2517    169   -933   -447       O
ATOM     96  CB  CYS A   6      -7.476  20.667   3.615  1.00 20.26           C  0.024
ANISOU   96  CB  CYS A   6     2490   3056   2153    199  -1007   -272       C
ATOM     97  SG  CYS A   6      -7.740  22.315   2.897  1.00 20.81           S  0.024
ANISOU   97  SG  CYS A   6     2601   3067   2238    132   -879    -55       S
ATOM     98  H   CYS A   6      -5.057  21.049   3.924  1.00 22.84           H  0.025
ATOM     99  HA  CYS A   6      -7.145  21.351   5.510  1.00 23.05           H  0.025
ATOM    100  HB2 CYS A   6      -6.966  20.145   2.977  1.00 24.32           H  0.026
ATOM    101  HB3 CYS A   6      -8.346  20.261   3.750  1.00 24.32           H  0.026
ATOM    102  N   GLU A   7      -5.984  18.382   5.366  1.00 19.30           N  0.023
ANISOU  102  N   GLU A   7     2272   2710   2349    217   -955   -763       N
ATOM    103  CA  GLU A   7      -6.020  17.101   6.049  1.00 20.33           C  0.024
ANISOU  103  CA  GLU A   7     2280   2720   2724    320   -890   -838       C
ATOM    104  C   GLU A   7      -5.846  17.298   7.549  1.00 19.21           C  0.023
ANISOU  104  C   GLU A   7     2083   2602   2614    215   -911   -776       C
ATOM    105  O   GLU A   7      -6.565  16.691   8.349  1.00 19.60           O  0.023
ANISOU  105  O   GLU A   7     2211   2474   2764    176   -915   -781       O
ATOM    106  CB  GLU A   7      -4.912  16.213   5.486  1.00 23.52           C  0.025
ANISOU  106  CB  GLU A   7     2726   2829   3383    365  -1298  -1106       C
ATOM    107  CG  GLU A   7      -4.798  14.844   6.111  1.00 27.66           C  0.028
ANISOU  107  CG  GLU A   7     3156   3047   4305    314  -1607   -960       C
ATOM    108  CD  GLU A   7      -3.549  14.063   5.649  1.00 30.64           C  0.029
ANISOU  108  CD  GLU A   7     3407   3254   4982    263  -1971   -906       C
ATOM    109  OE1 GLU A   7      -2.507  14.689   5.180  1.00 31.36           O  0.029
ANISOU  109  OE1 GLU A   7     3595   3330   4990    196  -2335   -954       O
ATOM    110  OE2 GLU A   7      -3.550  12.834   5.910  1.00 31.70           O  0.029
ANISOU  110  OE2 GLU A   7     3463   3233   5350    340  -1848   -781       O
ATOM    111  H   GLU A   7      -5.362  18.455   4.777  1.00 23.16           H  0.025
ATOM    112  HA  GLU A   7      -6.874  16.670   5.890  1.00 24.40           H  0.026
ATOM    113  HB2 GLU A   7      -5.071  16.087   4.537  1.00 28.23           H  0.028
ATOM    114  HB3 GLU A   7      -4.063  16.663   5.615  1.00 28.23           H  0.028
ATOM    115  HG2 GLU A   7      -4.746  14.936   7.075  1.00 33.19           H  0.030
ATOM    116  HG3 GLU A   7      -5.580  14.323   5.871  1.00 33.19           H  0.030
```

```
ATOM    117  N   LEU A   8      -4.893  18.138   7.953  1.00 17.22           N  0.022
ANISOU  117  N   LEU A   8     1955   2486   2100     26   -734   -609       N
ATOM    118  CA  LEU A   8      -4.701  18.368   9.379  1.00 16.64           C  0.021
ANISOU  118  CA  LEU A   8     1870   2369   2083     -1   -733   -375       C
ATOM    119  C   LEU A   8      -5.918  19.076   9.966  1.00 16.39           C  0.021
ANISOU  119  C   LEU A   8     1831   2224   2173    -30   -783   -417       C
ATOM    120  O   LEU A   8      -6.324  18.785  11.096  1.00 16.63           O  0.021
ANISOU  120  O   LEU A   8     1977   2133   2210    -44   -616   -395       O
ATOM    121  CB  LEU A   8      -3.421  19.168   9.640  1.00 16.49           C  0.021
ANISOU  121  CB  LEU A   8     1868   2369   2029    -44   -626   -455       C
ATOM    122  CG  LEU A   8      -3.124  19.435  11.099  1.00 16.64           C  0.021
ANISOU  122  CG  LEU A   8     1956   2228   2139   -170   -485   -388       C
ATOM    123  CD1 LEU A   8      -2.928  18.127  11.875  1.00 17.03           C  0.022
ANISOU  123  CD1 LEU A   8     1902   2197   2373   -297   -540   -411       C
ATOM    124  CD2 LEU A   8      -1.898  20.310  11.270  1.00 17.81           C  0.022
ANISOU  124  CD2 LEU A   8     2067   2349   2350   -240   -463   -392       C
ATOM    125  H   LEU A   8      -4.360  18.574   7.437  1.00 20.66           H  0.024
ATOM    126  HA  LEU A   8      -4.614  17.510   9.822  1.00 19.97           H  0.023
ATOM    127  HB2 LEU A   8      -2.669  18.677   9.274  1.00 19.79           H  0.023
ATOM    128  HB3 LEU A   8      -3.497  20.026   9.195  1.00 19.79           H  0.023
ATOM    129  HG  LEU A   8      -3.876  19.904  11.494  1.00 19.97           H  0.023
ATOM    130 HD11 LEU A   8      -2.614  18.335  12.769  1.00 20.44           H  0.024
ATOM    131 HD12 LEU A   8      -3.775  17.657  11.924  1.00 20.44           H  0.024
ATOM    132 HD13 LEU A   8      -2.272  17.582  11.413  1.00 20.44           H  0.024
ATOM    133 HD21 LEU A   8      -1.693  20.386  12.215  1.00 21.37           H  0.024
ATOM    134 HD22 LEU A   8      -1.154  19.903  10.800  1.00 21.37           H  0.024
ATOM    135 HD23 LEU A   8      -2.083  21.187  10.899  1.00 21.37           H  0.024
ATOM    136  N   ALA A   9      -6.494  20.048   9.239  1.00 16.58           N  0.021
ANISOU  136  N   ALA A   9     1838   2243   2218    -38   -669   -386       N
ATOM    137  CA  ALA A   9      -7.672  20.738   9.753  1.00 16.83           C  0.021
ANISOU  137  CA  ALA A   9     1921   2156   2319      5   -578   -357       C
ATOM    138  C   ALA A   9      -8.779  19.747  10.056  1.00 16.93           C  0.022
ANISOU  138  C   ALA A   9     2019   2124   2290      5   -653   -242       C
ATOM    139  O   ALA A   9      -9.420  19.818  11.111  1.00 17.24           O  0.022
ANISOU  139  O   ALA A   9     1999   2106   2447    -74   -781   -348       O
ATOM    140  CB  ALA A   9      -8.147  21.800   8.769  1.00 17.65           C  0.022
ANISOU  140  CB  ALA A   9     2082   2140   2483    -26   -452   -259       C
ATOM    141  H   ALA A   9      -6.225  20.315   8.467  1.00 19.90           H  0.023
ATOM    142  HA  ALA A   9      -7.437  21.184  10.582  1.00 20.20           H  0.024
ATOM    143  HB1 ALA A   9      -8.928  22.243   9.136  1.00 21.18           H  0.024
ATOM    144  HB2 ALA A   9      -7.435  22.444   8.632  1.00 21.18           H  0.024
ATOM    145  HB3 ALA A   9      -8.373  21.372   7.928  1.00 21.18           H  0.024
ATOM    146  N   ALA A  10      -9.030  18.830   9.121  1.00 18.06           N  0.022
ANISOU  146  N   ALA A  10     2108   2278   2476    -67   -811   -253       N
ATOM    147  CA  ALA A  10     -10.072  17.827   9.318  1.00 19.18           C  0.023
ANISOU  147  CA  ALA A  10     2206   2312   2770   -226   -966   -301       C
ATOM    148  C   ALA A  10      -9.768  16.949  10.527  1.00 19.13           C  0.023
ANISOU  148  C   ALA A  10     2055   2209   3004   -274  -1026   -339       C
ATOM    149  O   ALA A  10     -10.670  16.633  11.320  1.00 19.93           O  0.023
ANISOU  149  O   ALA A  10     2061   2247   3264   -295   -966   -107       O
ATOM    150  CB  ALA A  10     -10.231  16.998   8.046  1.00 20.16           C  0.023
ANISOU  150  CB  ALA A  10     2368   2416   2876   -256   -984   -494       C
ATOM    151  H   ALA A  10      -8.614  18.769   8.371  1.00 21.67           H  0.024
ATOM    152  HA  ALA A  10     -10.914  18.279   9.485  1.00 23.02           H  0.025
ATOM    153  HB1 ALA A  10     -10.925  16.335   8.186  1.00 24.19           H  0.026
ATOM    154  HB2 ALA A  10     -10.478  17.586   7.315  1.00 24.19           H  0.026
ATOM    155  HB3 ALA A  10      -9.389  16.559   7.848  1.00 24.19           H  0.026
ATOM    156  N   ALA A  11      -8.510  16.524  10.672  1.00 18.51           N  0.023
ANISOU  156  N   ALA A  11     2054   2130   2849   -176   -933   -443       N
ATOM    157  CA  ALA A  11      -8.160  15.656  11.788  1.00 18.49           C  0.022
ANISOU  157  CA  ALA A  11     2136   2123   2767   -186   -737   -286       C
ATOM    158  C   ALA A  11      -8.304  16.402  13.106  1.00 18.15           C  0.022
ANISOU  158  C   ALA A  11     2010   2081   2806   -145   -646    -45       C
ATOM    159  O   ALA A  11      -8.799  15.847  14.093  1.00 18.16           O  0.022
ANISOU  159  O   ALA A  11     2022   2037   2840   -117   -733      1       O
ATOM    160  CB  ALA A  11      -6.726  15.140  11.625  1.00 19.25           C  0.023
ANISOU  160  CB  ALA A  11     2236   2169   2910   -158   -673   -496       C
ATOM    161  H   ALA A  11      -7.857  16.721  10.148  1.00 22.21           H  0.025
ATOM    162  HA  ALA A  11      -8.759  14.893  11.802  1.00 22.19           H  0.025
ATOM    163  HB1 ALA A  11      -6.511  14.565  12.376  1.00 23.10           H  0.025
ATOM    164  HB2 ALA A  11      -6.663  14.640  10.796  1.00 23.10           H  0.025
ATOM    165  HB3 ALA A  11      -6.119  15.897  11.603  1.00 23.10           H  0.025
ATOM    166  N   MET A  12      -7.835  17.657  13.149  1.00 16.57           N  0.021
ANISOU  166  N   MET A  12     1865   1932   2498   -163   -595   -217       N
ATOM    167  CA  MET A  12      -7.959  18.436  14.369  1.00 16.62           C  0.021
ANISOU  167  CA  MET A  12     1772   1962   2582   -127   -540    -43       C
ATOM    168  C   MET A  12      -9.430  18.645  14.711  1.00 17.37           C  0.022
ANISOU  168  C   MET A  12     1797   2113   2691   -157   -599   -128       C
```

```
ATOM    169  O   MET A  12      -9.800  18.624  15.893  1.00 17.42           O  0.022
ANISOU  169  O   MET A  12     1782   2201   2637   -125   -405   -183       O
ATOM    170  CB  MET A  12      -7.234  19.780  14.264  1.00 15.98           C  0.021
ANISOU  170  CB  MET A  12     1699   1942   2430    -73   -466   -109       C
ATOM    171  CG  MET A  12      -5.709  19.613  14.314  1.00 14.62           C  0.020
ANISOU  171  CG  MET A  12     1683   1731   2142    -58   -494   -167       C
ATOM    172  SD  MET A  12      -4.925  21.239  14.030  1.00 14.53           S  0.020
ANISOU  172  SD  MET A  12     1845   1746   1929     -8   -252   -130       S
ATOM    173  CE  MET A  12      -3.397  20.978  14.933  1.00 15.59           C  0.021
ANISOU  173  CE  MET A  12     1809   1747   2367    -72   -302   -167       C
ATOM    174  H   MET A  12      -7.451  18.065  12.497  1.00 19.88           H  0.023
ATOM    175  HA  MET A  12      -7.545  17.939  15.092  1.00 19.95           H  0.023
ATOM    176  HB2 MET A  12      -7.465  20.202  13.422  1.00 19.17           H  0.023
ATOM    177  HB3 MET A  12      -7.501  20.345  15.006  1.00 19.17           H  0.023
ATOM    178  HG2 MET A  12      -5.443  19.287  15.188  1.00 17.55           H  0.022
ATOM    179  HG3 MET A  12      -5.423  19.003  13.617  1.00 17.55           H  0.022
ATOM    180  HE1 MET A  12      -2.855  21.780  14.874  1.00 18.71           H  0.023
ATOM    181  HE2 MET A  12      -3.607  20.788  15.861  1.00 18.71           H  0.023
ATOM    182  HE3 MET A  12      -2.923  20.228  14.541  1.00 18.71           H  0.023
ATOM    183  N   LYS A  13     -10.282  18.854  13.700  1.00 18.68           N  0.023
ANISOU  183  N   LYS A  13     1857   2382   2859   -170   -626   -141       N
ATOM    184  CA  LYS A  13     -11.715  18.981  13.954  1.00 19.50           C  0.023
ANISOU  184  CA  LYS A  13     1859   2583   2969   -144   -686    230       C
ATOM    185  C   LYS A  13     -12.287  17.684  14.517  1.00 20.57           C  0.024
ANISOU  185  C   LYS A  13     1924   2624   3269   -276   -595    256       C
ATOM    186  O   LYS A  13     -13.053  17.708  15.483  1.00 21.65           O  0.024
ANISOU  186  O   LYS A  13     1972   2611   3644   -190   -652    205       O
ATOM    187  CB  LYS A  13     -12.428  19.392  12.668  1.00 20.86           C  0.024
ANISOU  187  CB  LYS A  13     1923   2808   3195    -95   -628    400       C
ATOM    188  CG  LYS A  13     -13.880  19.831  12.897  1.00 23.93           C  0.026
ANISOU  188  CG  LYS A  13     2003   3271   3817    113   -708    511       C
ATOM    189  CD  LYS A  13     -14.480  20.498  11.656  1.00 27.30           C  0.027
ANISOU  189  CD  LYS A  13     2199   3751   4423    215   -748    673       C
ATOM    190  CE  LYS A  13     -15.875  21.056  11.914  1.00 29.91           C  0.029
ANISOU  190  CE  LYS A  13     2403   4080   4883    408   -755    739       C
ATOM    191  NZ  LYS A  13     -16.440  21.650  10.687  1.00 32.03           N  0.030
ANISOU  191  NZ  LYS A  13     2641   4291   5238    611   -486    748       N
ATOM    192  H   LYS A  13     -10.057  18.924  12.873  1.00 22.42           H  0.025
ATOM    193  HA  LYS A  13     -11.857  19.680  14.611  1.00 23.41           H  0.025
ATOM    194  HB2 LYS A  13     -11.951  20.136  12.268  1.00 25.03           H  0.026
ATOM    195  HB3 LYS A  13     -12.437  18.638  12.058  1.00 25.03           H  0.026
ATOM    196  HG2 LYS A  13     -14.418  19.053  13.112  1.00 28.71           H  0.028
ATOM    197  HG3 LYS A  13     -13.908  20.469  13.627  1.00 28.71           H  0.028
ATOM    198  HD2 LYS A  13     -13.908  21.232  11.382  1.00 32.76           H  0.030
ATOM    199  HD3 LYS A  13     -14.545  19.843  10.944  1.00 32.76           H  0.030
ATOM    200  HE2 LYS A  13     -16.460  20.339  12.204  1.00 35.90           H  0.031
ATOM    201  HE3 LYS A  13     -15.825  21.747  12.593  1.00 35.90           H  0.031
ATOM    202  HZ1 LYS A  13     -17.253  21.971  10.852  1.00 38.44           H  0.032
ATOM    203  HZ2 LYS A  13     -15.919  22.313  10.403  1.00 38.44           H  0.032
ATOM    204  HZ3 LYS A  13     -16.497  21.032  10.049  1.00 38.44           H  0.032
ATOM    205  N   ARG A  14     -11.908  16.532  13.948  1.00 20.73           N  0.024
ANISOU  205  N   ARG A  14     2017   2665   3194   -496   -828    208       N
ATOM    206  CA  ARG A  14     -12.400  15.263  14.475  1.00 23.93           C  0.026
ANISOU  206  CA  ARG A  14     2239   2950   3902   -600   -836    311       C
ATOM    207  C   ARG A  14     -11.974  15.076  15.925  1.00 23.23           C  0.025
ANISOU  207  C   ARG A  14     2081   2766   3979   -553   -639    364       C
ATOM    208  O   ARG A  14     -12.698  14.461  16.720  1.00 24.55           O  0.026
ANISOU  208  O   ARG A  14     2095   2939   4293   -658   -613    503       O
ATOM    209  CB  ARG A  14     -11.851  14.124  13.620  1.00 28.16           C  0.028
ANISOU  209  CB  ARG A  14     2755   3343   4602   -766  -1004    237       C
ATOM    210  CG  ARG A  14     -12.284  14.147  12.145  1.00 34.46           C  0.031
ANISOU  210  CG  ARG A  14     3321   3931   5842   -798   -725    416       C
ATOM    211  CD  ARG A  14     -13.069  12.908  11.743  1.00 39.90           C  0.033
ANISOU  211  CD  ARG A  14     3841   4416   6901   -841   -704    413       C
ATOM    212  NE  ARG A  14     -14.102  12.590  12.720  1.00 43.22           N  0.034
ANISOU  212  NE  ARG A  14     4130   4711   7581  -1019   -739    405       N
ATOM    213  CZ  ARG A  14     -14.411  11.368  13.133  1.00 45.62           C  0.035
ANISOU  213  CZ  ARG A  14     4412   4932   7990  -1130   -712    385       C
ATOM    214  NH1 ARG A  14     -13.808  10.296  12.644  1.00 46.08           N  0.036
ANISOU  214  NH1 ARG A  14     4493   4949   8066  -1175   -788    395       N
ATOM    215  NH2 ARG A  14     -15.354  11.218  14.056  1.00 46.92           N  0.036
ANISOU  215  NH2 ARG A  14     4601   5070   8157  -1132   -654    391       N
ATOM    216  H   ARG A  14     -11.380  16.463  13.273  1.00 24.88           H  0.026
ATOM    217  HA  ARG A  14     -13.369  15.245  14.431  1.00 28.71           H  0.028
ATOM    218  HB2 ARG A  14     -10.882  14.174  13.635  1.00 33.79           H  0.030
ATOM    219  HB3 ARG A  14     -12.133  13.281  14.007  1.00 33.79           H  0.030
ATOM    220  HG2 ARG A  14     -12.848  14.922  11.993  1.00 41.36           H  0.034
ATOM    221  HG3 ARG A  14     -11.494  14.196  11.584  1.00 41.36           H  0.034
```

```
ATOM    222  HD2 ARG A  14     -13.499  13.066  10.888  1.00 47.88           H  0.036
ATOM    223  HD3 ARG A  14     -12.455  12.162  11.677  1.00 47.88           H  0.036
ATOM    224  HE  ARG A  14     -14.546  13.246  13.055  1.00 51.87           H  0.038
ATOM    225 HH11 ARG A  14     -13.196  10.374  12.046  1.00 55.29           H  0.039
ATOM    226 HH12 ARG A  14     -14.029   9.515  12.928  1.00 55.29           H  0.039
ATOM    227 HH21 ARG A  14     -15.753  11.908  14.379  1.00 56.31           H  0.039
ATOM    228 HH22 ARG A  14     -15.566  10.431  14.330  1.00 56.31           H  0.039
ATOM    229  N   HIS A  15     -10.790  15.600  16.268  1.00 21.26           N  0.024
ANISOU  229  N   HIS A  15     1925   2527   3626   -479   -569    265       N
ATOM    230  CA  HIS A  15     -10.222  15.460  17.632  1.00 21.07           C  0.024
ANISOU  230  CA  HIS A  15     1975   2393   3638   -445   -464    480       C
ATOM    231  C   HIS A  15     -10.709  16.567  18.559  1.00 20.29           C  0.024
ANISOU  231  C   HIS A  15     1879   2512   3316   -428   -320    493       C
ATOM    232  O   HIS A  15     -10.153  16.660  19.630  1.00 21.34           O  0.024
ANISOU  232  O   HIS A  15     1935   2651   3523   -319   -331    526       O
ATOM    233  CB  HIS A  15      -8.695  15.326  17.543  1.00 21.75           C  0.024
ANISOU  233  CB  HIS A  15     2105   2199   3960   -512   -912    476       C
ATOM    234  CG  HIS A  15      -8.315  13.999  17.001  1.00 24.38           C  0.026
ANISOU  234  CG  HIS A  15     2352   2210   4701   -473  -1061    669       C
ATOM    235  ND1 HIS A  15      -7.738  13.023  17.755  1.00 26.92           N  0.027
ANISOU  235  ND1 HIS A  15     2594   2427   5209   -477   -732    810       N
ATOM    236  CD2 HIS A  15      -8.507  13.444  15.796  1.00 26.38           C  0.027
ANISOU  236  CD2 HIS A  15     2689   2204   5129   -395  -1245     47       C
ATOM    237  CE1 HIS A  15      -7.613  11.938  17.056  1.00 27.79           C  0.028
ANISOU  237  CE1 HIS A  15     2779   2213   5566   -331   -874    484       C
ATOM    238  NE2 HIS A  15      -7.998  12.198  15.860  1.00 28.32           N  0.028
ANISOU  238  NE2 HIS A  15     3031   2370   5358   -278  -1219    135       N
ATOM    239  H   HIS A  15     -10.288  16.045  15.730  1.00 25.51           H  0.026
ATOM    240  HA  HIS A  15     -10.551  14.626  18.003  1.00 25.28           H  0.026
ATOM    241  HB2 HIS A  15      -8.344  16.011  16.953  1.00 26.10           H  0.027
ATOM    242  HB3 HIS A  15      -8.312  15.413  18.429  1.00 26.10           H  0.027
ATOM    243  HD1 HIS A  15      -7.540  13.097  18.589  1.00 32.31           H  0.030
ATOM    244  HD2 HIS A  15      -8.871  13.855  15.047  1.00 31.66           H  0.029
ATOM    245  HE1 HIS A  15      -7.204  11.153  17.340  1.00 33.34           H  0.030
ATOM    246  N   GLY A  16     -11.683  17.395  18.152  1.00 20.19           N  0.024
ANISOU  246  N   GLY A  16     1867   2584   3221   -324   -416    543       N
ATOM    247  CA  GLY A  16     -12.306  18.297  19.085  1.00 19.97           C  0.023
ANISOU  247  CA  GLY A  16     1881   2574   3131   -200   -250    406       C
ATOM    248  C   GLY A  16     -11.599  19.610  19.325  1.00 18.86           C  0.023
ANISOU  248  C   GLY A  16     1806   2513   2848   -225   -168    259       C
ATOM    249  O   GLY A  16     -11.884  20.267  20.327  1.00 19.97           O  0.023
ANISOU  249  O   GLY A  16     1926   2579   3083   -364   -128    228       O
ATOM    250  H   GLY A  16     -11.987  17.443  17.349  1.00 24.23           H  0.026
ATOM    251  HA2 GLY A  16     -13.201  18.499  18.769  1.00 23.96           H  0.026
ATOM    252  HA3 GLY A  16     -12.389  17.848  19.941  1.00 23.96           H  0.026
ATOM    253  N   LEU A  17     -10.669  20.025  18.443  1.00 17.77           N  0.022
ANISOU  253  N   LEU A  17     1671   2434   2647   -241    -12    120       N
ATOM    254  CA  LEU A  17      -9.956  21.288  18.680  1.00 16.63           C  0.021
ANISOU  254  CA  LEU A  17     1641   2337   2339   -135    -70    116       C
ATOM    255  C   LEU A  17     -10.707  22.507  18.178  1.00 16.97           C  0.022
ANISOU  255  C   LEU A  17     1685   2472   2289   -121    -12    155       C
ATOM    256  O   LEU A  17     -10.375  23.613  18.605  1.00 17.16           O  0.022
ANISOU  256  O   LEU A  17     1631   2398   2490    -42     -8    101       O
ATOM    257  CB  LEU A  17      -8.586  21.260  18.015  1.00 15.90           C  0.021
ANISOU  257  CB  LEU A  17     1797   2190   2052      8    -69    161       C
ATOM    258  CG  LEU A  17      -7.530  20.530  18.831  1.00 16.47           C  0.021
ANISOU  258  CG  LEU A  17     1894   2133   2230    125   -134    182       C
ATOM    259  CD1 LEU A  17      -6.185  20.582  18.082  1.00 16.36           C  0.021
ANISOU  259  CD1 LEU A  17     1789   2228   2199    316   -171    -53       C
ATOM    260  CD2 LEU A  17      -7.306  21.081  20.238  1.00 15.99           C  0.021
ANISOU  260  CD2 LEU A  17     1993   1992   2091     57   -103    194       C
ATOM    261  H   LEU A  17     -10.442  19.608  17.726  1.00 21.33           H  0.024
ATOM    262  HA  LEU A  17      -9.831  21.399  19.634  1.00 19.95           H  0.023
ATOM    263  HB2 LEU A  17      -8.662  20.812  17.158  1.00 19.08           H  0.023
ATOM    264  HB3 LEU A  17      -8.283  22.172  17.884  1.00 19.08           H  0.023
ATOM    265  HG  LEU A  17      -7.788  19.599  18.914  1.00 19.76           H  0.023
ATOM    266 HD11 LEU A  17      -5.524  20.079  18.583  1.00 19.63           H  0.023
ATOM    267 HD12 LEU A  17      -6.300  20.192  17.201  1.00 19.63           H  0.023
ATOM    268 HD13 LEU A  17      -5.905  21.507  18.000  1.00 19.63           H  0.023
ATOM    269 HD21 LEU A  17      -6.470  20.732  20.584  1.00 19.19           H  0.023
ATOM    270 HD22 LEU A  17      -7.268  22.049  20.194  1.00 19.19           H  0.023
ATOM    271 HD23 LEU A  17      -8.040  20.804  20.808  1.00 19.19           H  0.023
ATOM    272  N   ASP A  18     -11.717  22.361  17.310  1.00 17.27           N  0.022
ANISOU  272  N   ASP A  18     1789   2626   2147    -81   -162    165       N
ATOM    273  CA  ASP A  18     -12.399  23.545  16.788  1.00 18.73           C  0.023
ANISOU  273  CA  ASP A  18     1831   2831   2454   -118   -193    238       C
ATOM    274  C   ASP A  18     -13.156  24.246  17.904  1.00 18.39           C  0.022
ANISOU  274  C   ASP A  18     1611   2764   2614   -100     31    342       C
```

```
ATOM    275  O   ASP A  18     -14.088  23.685  18.489  1.00 18.91           O  0.023
ANISOU  275  O   ASP A  18     1609   2786   2792   -245     49    212       O
ATOM    276  CB  ASP A  18     -13.339  23.196  15.637  1.00 20.96           C  0.024
ANISOU  276  CB  ASP A  18     2003   3159   2803   -173   -388    205       C
ATOM    277  CG  ASP A  18     -13.865  24.439  14.895  1.00 22.26           C  0.025
ANISOU  277  CG  ASP A  18     2178   3338   2943   -289   -691    205       C
ATOM    278  OD1 ASP A  18     -13.306  25.557  14.994  1.00 21.81           O  0.024
ANISOU  278  OD1 ASP A  18     2155   3342   2791   -320   -410     20       O
ATOM    279  OD2 ASP A  18     -14.841  24.281  14.151  1.00 25.62           O  0.026
ANISOU  279  OD2 ASP A  18     2555   3532   3649   -273  -1123    254       O
ATOM    280  H   ASP A  18     -12.017  21.610  17.017  1.00 20.72           H  0.024
ATOM    281  HA  ASP A  18     -11.733  24.163  16.448  1.00 22.48           H  0.025
ATOM    282  HB2 ASP A  18     -12.863  22.644  14.997  1.00 25.16           H  0.026
ATOM    283  HB3 ASP A  18     -14.102  22.712  15.989  1.00 25.16           H  0.026
ATOM    284  N  AASN A  19     -12.740  25.469  18.212  0.54 17.65           N  0.022
ANISOU  284  N  AASN A  19     1453   2689   2564   -141   -106    381       N
ATOM    285  CA AASN A  19     -13.328  26.274  19.265  0.54 18.29           C  0.022
ANISOU  285  CA AASN A  19     1532   2665   2752    -72   -173    476       C
ATOM    286  C  AASN A  19     -12.989  25.742  20.648  0.54 17.72           C  0.022
ANISOU  286  C  AASN A  19     1495   2598   2638    -39     53    451       C
ATOM    287  O  AASN A  19     -13.611  26.144  21.632  0.54 18.36           O  0.022
ANISOU  287  O  AASN A  19     1533   2728   2717    -30     49    340       O
ATOM    288  CB AASN A  19     -14.842  26.473  19.063  0.54 19.74           C  0.023
ANISOU  288  CB AASN A  19     1732   2725   3043    -31   -471    384       C
ATOM    289  CG AASN A  19     -15.330  27.745  19.679  0.54 22.28           C  0.025
ANISOU  289  CG AASN A  19     2040   2861   3564    -20   -422    128       C
ATOM    290  OD1AASN A  19     -14.558  28.684  19.894  0.54 23.79           O  0.026
ANISOU  290  OD1AASN A  19     2297   2879   3862    163   -396    189       O
ATOM    291  ND2AASN A  19     -16.618  27.800  19.949  0.54 23.44           N  0.025
ANISOU  291  ND2AASN A  19     2075   2928   3903     21   -207   -112       N
ATOM    292  H  AASN A  19     -12.093  25.865  17.806  0.54 21.18           H  0.024
ATOM    293  HA AASN A  19     -12.923  27.153  19.207  0.54 21.95           H  0.025
ATOM    294  HB2AASN A  19     -15.035  26.504  18.113  0.54 23.69           H  0.025
ATOM    295  HB3AASN A  19     -15.318  25.735  19.476  0.54 23.69           H  0.025
ATOM    296 HD21AASN A  19     -17.122  27.124  19.780  0.54 28.13           H  0.028
ATOM    297 HD22AASN A  19     -16.953  28.509  20.302  0.54 28.13           H  0.028
ATOM    298  N  BASN A  19     -12.743  25.470  18.217  0.46 17.88           N  0.022
ANISOU  298  N  BASN A  19     1469   2760   2565    -93    107    432       N
ATOM    299  CA BASN A  19     -13.331  26.272  19.276  0.46 18.50           C  0.022
ANISOU  299  CA BASN A  19     1529   2794   2708    -12    180    584       C
ATOM    300  C  BASN A  19     -12.993  25.733  20.655  0.46 17.77           C  0.022
ANISOU  300  C  BASN A  19     1486   2662   2605     -1    263    497       C
ATOM    301  O  BASN A  19     -13.619  26.127  21.640  0.46 18.18           O  0.022
ANISOU  301  O  BASN A  19     1492   2765   2650     19    317    361       O
ATOM    302  CB BASN A  19     -14.843  26.486  19.068  0.46 20.03           C  0.023
ANISOU  302  CB BASN A  19     1686   2995   2928     90    124    648       C
ATOM    303  CG BASN A  19     -15.146  27.023  17.694  0.46 22.07           C  0.025
ANISOU  303  CG BASN A  19     1880   3251   3254    156    326    590       C
ATOM    304  OD1BASN A  19     -14.625  28.071  17.314  0.46 23.68           O  0.025
ANISOU  304  OD1BASN A  19     2060   3334   3604    337    183    612       O
ATOM    305  ND2BASN A  19     -15.989  26.313  16.935  0.46 22.63           N  0.025
ANISOU  305  ND2BASN A  19     1889   3409   3300    102    426    577       N
ATOM    306  H  BASN A  19     -12.098  25.869  17.812  0.46 21.46           H  0.024
ATOM    307  HA BASN A  19     -12.926  27.152  19.225  0.46 22.21           H  0.025
ATOM    308  HB2BASN A  19     -15.306  25.641  19.174  0.46 24.03           H  0.026
ATOM    309  HB3BASN A  19     -15.166  27.127  19.720  0.46 24.03           H  0.026
ATOM    310 HD21BASN A  19     -16.332  25.585  17.238  0.46 27.16           H  0.027
ATOM    311 HD22BASN A  19     -16.188  26.585  16.144  0.46 27.16           H  0.027
ATOM    312  N   TYR A  20     -12.001  24.849  20.762  1.00 17.38           N  0.022
ANISOU  312  N   TYR A  20     1510   2573   2522      0    127    509       N
ATOM    313  CA  TYR A  20     -11.578  24.387  22.079  1.00 17.08           C  0.022
ANISOU  313  CA  TYR A  20     1607   2497   2385     22    134    305       C
ATOM    314  C   TYR A  20     -10.982  25.568  22.842  1.00 16.57           C  0.021
ANISOU  314  C   TYR A  20     1602   2493   2200    159    365    345       C
ATOM    315  O   TYR A  20     -10.054  26.241  22.357  1.00 15.67           O  0.021
ANISOU  315  O   TYR A  20     1447   2301   2208    101    284    434       O
ATOM    316  CB  TYR A  20     -10.586  23.213  21.979  1.00 17.07           C  0.022
ANISOU  316  CB  TYR A  20     1667   2407   2414    -47     59    260       C
ATOM    317  CG  TYR A  20     -10.340  22.592  23.335  1.00 16.85           C  0.021
ANISOU  317  CG  TYR A  20     1673   2303   2425    -42    174    306       C
ATOM    318  CD1 TYR A  20     -11.161  21.574  23.814  1.00 18.32           C  0.022
ANISOU  318  CD1 TYR A  20     1791   2483   2688      1    326    302       C
ATOM    319  CD2 TYR A  20      -9.321  23.050  24.161  1.00 17.07           C  0.022
ANISOU  319  CD2 TYR A  20     1649   2396   2442    194     27    410       C
ATOM    320  CE1 TYR A  20     -10.981  21.054  25.080  1.00 18.81           C  0.023
ANISOU  320  CE1 TYR A  20     1937   2600   2611     30    293    467       C
ATOM    321  CE2 TYR A  20      -9.133  22.544  25.423  1.00 17.60           C  0.022
ANISOU  321  CE2 TYR A  20     1751   2525   2412    277    260    327       C
```

```
ATOM    322  CZ  TYR A  20      -9.966  21.545  25.882  1.00 18.03           C  0.022
ANISOU  322  CZ  TYR A  20     1907   2709   2233    281    271    540        C
ATOM    323  OH  TYR A  20      -9.777  21.044  27.142  1.00 20.38           O  0.024
ANISOU  323  OH  TYR A  20     2206   3120   2417    323    388    445        O
ATOM    324  H   TYR A  20     -11.569  24.508  20.101  1.00 20.86           H  0.024
ATOM    325  HA  TYR A  20     -12.356  24.078  22.568  1.00 20.50           H  0.024
ATOM    326  HB2 TYR A  20     -10.952  22.532  21.393  1.00 20.49           H  0.024
ATOM    327  HB3 TYR A  20      -9.739  23.536  21.633  1.00 20.49           H  0.024
ATOM    328  HD1 TYR A  20     -11.860  21.262  23.286  1.00 21.99           H  0.025
ATOM    329  HD2 TYR A  20      -8.775  23.742  23.865  1.00 20.49           H  0.024
ATOM    330  HE1 TYR A  20     -11.540  20.379  25.393  1.00 22.58           H  0.025
ATOM    331  HE2 TYR A  20      -8.449  22.870  25.962  1.00 21.12           H  0.024
ATOM    332  HH  TYR A  20      -9.130  21.432  27.512  1.00 24.45           H  0.026
ATOM    333  N   ARG A  21     -11.453  25.800  24.064  1.00 17.26           N  0.022
ANISOU  333  N   ARG A  21     1733   2635   2189    147    445    467        N
ATOM    334  CA  ARG A  21     -11.025  26.957  24.895  1.00 17.61           C  0.022
ANISOU  334  CA  ARG A  21     1888   2693   2109    294    433    450        C
ATOM    335  C   ARG A  21     -11.245  28.245  24.095  1.00 16.35           C  0.021
ANISOU  335  C   ARG A  21     1732   2542   1938    187    245    142        C
ATOM    336  O   ARG A  21     -10.568  29.222  24.368  1.00 16.77           O  0.021
ANISOU  336  O   ARG A  21     1890   2617   1866     71    324   -136        O
ATOM    337  CB  ARG A  21      -9.589  26.749  25.390  1.00 21.39           C  0.024
ANISOU  337  CB  ARG A  21     2227   3062   2838    446    326    944        C
ATOM    338  CG  ARG A  21      -9.506  26.114  26.769  1.00 27.52           C  0.027
ANISOU  338  CG  ARG A  21     2867   3669   3919    724    286    981        C
ATOM    339  CD  ARG A  21      -9.926  27.094  27.841  1.00 33.36           C  0.030
ANISOU  339  CD  ARG A  21     3564   4190   4919    858    466    819        C
ATOM    340  NE  ARG A  21      -9.515  26.688  29.170  1.00 38.73           N  0.033
ANISOU  340  NE  ARG A  21     4134   4661   5920   1020    562    659        N
ATOM    341  CZ  ARG A  21      -9.494  27.473  30.243  1.00 41.90           C  0.034
ANISOU  341  CZ  ARG A  21     4550   4952   6419   1140    640    689        C
ATOM    342  NH1 ARG A  21      -9.848  28.746  30.166  1.00 43.55           N  0.035
ANISOU  342  NH1 ARG A  21     4741   5104   6704   1081    653    711        N
ATOM    343  NH2 ARG A  21      -9.099  26.980  31.401  1.00 42.57           N  0.034
ANISOU  343  NH2 ARG A  21     4697   5073   6405   1111    750    743        N
ATOM    344  H   ARG A  21     -12.034  25.299  24.453  1.00 20.71           H  0.024
ATOM    345  HA  ARG A  21     -11.599  27.001  25.675  1.00 21.13           H  0.024
ATOM    346  HB2 ARG A  21      -9.124  26.167  24.769  1.00 25.67           H  0.027
ATOM    347  HB3 ARG A  21      -9.141  27.608  25.432  1.00 25.67           H  0.027
ATOM    348  HG2 ARG A  21     -10.099  25.347  26.807  1.00 33.02           H  0.030
ATOM    349  HG3 ARG A  21      -8.591  25.843  26.944  1.00 33.02           H  0.030
ATOM    350  HD2 ARG A  21      -9.525  27.957  27.655  1.00 40.03           H  0.033
ATOM    351  HD3 ARG A  21     -10.893  27.171  27.838  1.00 40.03           H  0.033
ATOM    352  HE  ARG A  21      -9.268  25.871  29.274  1.00 46.48           H  0.036
ATOM    353 HH11 ARG A  21     -10.106  29.078  29.416  1.00 52.27           H  0.038
ATOM    354 HH12 ARG A  21      -9.827  29.240  30.869  1.00 52.27           H  0.038
ATOM    355 HH21 ARG A  21      -9.081  27.483  32.099  1.00 51.08           H  0.037
ATOM    356 HH22 ARG A  21      -8.865  26.155  31.460  1.00 51.08           H  0.037
ATOM    357  N   GLY A  22     -12.197  28.229  23.158  1.00 15.76           N  0.021
ANISOU  357  N   GLY A  22     1656   2387   1944    196    409    267        N
ATOM    358  CA  GLY A  22     -12.497  29.403  22.375  1.00 15.44           C  0.021
ANISOU  358  CA  GLY A  22     1561   2243   2062    196    270    127        C
ATOM    359  C   GLY A  22     -11.618  29.651  21.162  1.00 14.91           C  0.020
ANISOU  359  C   GLY A  22     1556   2141   1967    233    249     89        C
ATOM    360  O   GLY A  22     -11.782  30.684  20.489  1.00 15.28           O  0.020
ANISOU  360  O   GLY A  22     1670   1919   2217    187    182     31        O
ATOM    361  H   GLY A  22     -12.681  27.545  22.964  1.00 18.91           H  0.023
ATOM    362  HA2 GLY A  22     -13.414  29.341  22.065  1.00 18.53           H  0.023
ATOM    363  HA3 GLY A  22     -12.431  30.182  22.949  1.00 18.53           H  0.023
ATOM    364  N   TYR A  23     -10.721  28.740  20.827  1.00 13.32           N  0.019
ANISOU  364  N   TYR A  23     1395   2082   1582    245    272     27        N
ATOM    365  CA  TYR A  23      -9.771  28.929  19.735  1.00 13.24           C  0.019
ANISOU  365  CA  TYR A  23     1415   2018   1597    190    209    -79        C
ATOM    366  C   TYR A  23     -10.283  28.206  18.504  1.00 13.21           C  0.019
ANISOU  366  C   TYR A  23     1470   2011   1537     67    146     35        C
ATOM    367  O   TYR A  23     -10.358  26.975  18.471  1.00 13.74           O  0.019
ANISOU  367  O   TYR A  23     1441   2002   1777    -13     56    -40        O
ATOM    368  CB  TYR A  23      -8.379  28.450  20.147  1.00 12.60           C  0.019
ANISOU  368  CB  TYR A  23     1445   1806   1538     92    208     62        C
ATOM    369  CG  TYR A  23      -7.723  29.388  21.149  1.00 12.08           C  0.018
ANISOU  369  CG  TYR A  23     1487   1590   1515    155    173    173        C
ATOM    370  CD1 TYR A  23      -7.029  30.514  20.727  1.00 12.34           C  0.018
ANISOU  370  CD1 TYR A  23     1492   1725   1473    183    167     77        C
ATOM    371  CD2 TYR A  23      -7.819  29.168  22.508  1.00 12.26           C  0.018
ANISOU  371  CD2 TYR A  23     1605   1638   1416     95     62    197        C
ATOM    372  CE1 TYR A  23      -6.432  31.344  21.626  1.00 11.70           C  0.018
ANISOU  372  CE1 TYR A  23     1461   1565   1420     48    135    161        C
ATOM    373  CE2 TYR A  23      -7.259  30.010  23.403  1.00 12.20           C  0.018
```

```
ANISOU  373  CE2 TYR A  23     1503   1700   1432    168    120     90       C
ATOM    374  CZ  TYR A  23      -6.559  31.117  22.971  1.00 12.03           C  0.018
ANISOU  374  CZ  TYR A  23     1531   1620   1419     76      2     40       C
ATOM    375  OH  TYR A  23      -5.943  31.963  23.849  1.00 12.78           O  0.019
ANISOU  375  OH  TYR A  23     1653   1679   1523     95    -50    216       O
ATOM    376  H   TYR A  23     -10.637  27.983  21.225  1.00 15.98           H  0.021
ATOM    377  HA  TYR A  23      -9.711  29.874  19.525  1.00 15.89           H  0.021
ATOM    378  HB2 TYR A  23      -8.453  27.574  20.557  1.00 15.13           H  0.020
ATOM    379  HB3 TYR A  23      -7.813  28.404  19.361  1.00 15.13           H  0.020
ATOM    380  HD1 TYR A  23      -6.940  30.683  19.817  1.00 14.81           H  0.020
ATOM    381  HD2 TYR A  23      -8.294  28.430  22.815  1.00 14.71           H  0.020
ATOM    382  HE1 TYR A  23      -5.967  32.092  21.326  1.00 14.05           H  0.020
ATOM    383  HE2 TYR A  23      -7.329  29.832  24.313  1.00 14.64           H  0.020
ATOM    384  HH  TYR A  23      -6.162  31.769  24.636  1.00 15.33           H  0.020
ATOM    385  N   SER A  24     -10.661  28.977  17.492  1.00 14.76           N  0.020
ANISOU  385  N   SER A  24     1698   2183   1727    -29     48    284       N
ATOM    386  CA  SER A  24     -11.144  28.406  16.247  1.00 14.91           C  0.020
ANISOU  386  CA  SER A  24     1673   2331   1662    -40    -95    173       C
ATOM    387  C   SER A  24     -10.086  27.517  15.590  1.00 14.37           C  0.020
ANISOU  387  C   SER A  24     1481   2253   1725    -43   -180     -9       C
ATOM    388  O   SER A  24      -8.882  27.674  15.790  1.00 14.27           O  0.020
ANISOU  388  O   SER A  24     1467   2474   1482   -163   -128    -59       O
ATOM    389  CB  SER A  24     -11.578  29.520  15.292  1.00 16.73           C  0.021
ANISOU  389  CB  SER A  24     2062   2481   1815    -98   -156     95       C
ATOM    390  OG  SER A  24     -10.449  30.280  14.907  1.00 18.40           O  0.022
ANISOU  390  OG  SER A  24     2460   2609   1922   -385   -137    187       O
ATOM    391  H   SER A  24     -10.647  29.837  17.503  1.00 17.71           H  0.022
ATOM    392  HA  SER A  24     -11.920  27.855  16.435  1.00 17.90           H  0.022
ATOM    393  HB2 SER A  24     -11.982  29.126  14.503  1.00 20.08           H  0.023
ATOM    394  HB3 SER A  24     -12.214  30.098  15.742  1.00 20.08           H  0.023
ATOM    395  HG  SER A  24      -9.928  29.810  14.446  1.00 22.08           H  0.025
ATOM    396  N   LEU A  25     -10.569  26.624  14.728  1.00 14.50           N  0.020
ANISOU  396  N   LEU A  25     1486   2155   1867    -74   -254      8       N
ATOM    397  CA  LEU A  25      -9.713  25.640  14.082  1.00 13.69           C  0.019
ANISOU  397  CA  LEU A  25     1596   1989   1618    -54   -255     28       C
ATOM    398  C   LEU A  25      -8.499  26.248  13.399  1.00 12.60           C  0.019
ANISOU  398  C   LEU A  25     1532   1852   1403     21   -175    100       C
ATOM    399  O   LEU A  25      -7.402  25.689  13.470  1.00 13.39           O  0.019
ANISOU  399  O   LEU A  25     1497   1641   1948    102   -164     62       O
ATOM    400  CB  LEU A  25     -10.559  24.873  13.076  1.00 15.71           C  0.021
ANISOU  400  CB  LEU A  25     1855   2128   1988   -170   -273    205       C
ATOM    401  CG  LEU A  25      -9.923  23.643  12.474  1.00 15.56           C  0.021
ANISOU  401  CG  LEU A  25     1833   2133   1946    -94   -201     86       C
ATOM    402  CD1 LEU A  25      -9.548  22.615  13.525  1.00 16.63           C  0.021
ANISOU  402  CD1 LEU A  25     1980   1991   2348   -104   -476     92       C
ATOM    403  CD2 LEU A  25     -10.916  23.033  11.473  1.00 15.71           C  0.021
ANISOU  403  CD2 LEU A  25     1942   2220   1808     78   -442    -97       C
ATOM    404  H   LEU A  25     -11.397  26.569  14.501  1.00 17.40           H  0.022
ATOM    405  HA  LEU A  25      -9.396  25.015  14.753  1.00 16.43           H  0.021
ATOM    406  HB2 LEU A  25     -11.375  24.589  13.517  1.00 18.86           H  0.023
ATOM    407  HB3 LEU A  25     -10.780  25.471  12.345  1.00 18.86           H  0.023
ATOM    408  HG  LEU A  25      -9.120  23.897  11.993  1.00 18.67           H  0.023
ATOM    409 HD11 LEU A  25      -9.293  21.791  13.081  1.00 19.96           H  0.023
ATOM    410 HD12 LEU A  25      -8.804  22.952  14.048  1.00 19.96           H  0.023
ATOM    411 HD13 LEU A  25     -10.314  22.457  14.099  1.00 19.96           H  0.023
ATOM    412 HD21 LEU A  25     -10.518  22.242  11.077  1.00 18.86           H  0.023
ATOM    413 HD22 LEU A  25     -11.731  22.795  11.942  1.00 18.86           H  0.023
ATOM    414 HD23 LEU A  25     -11.110  23.687  10.783  1.00 18.86           H  0.023
ATOM    415  N   GLY A  26      -8.672  27.382  12.733  1.00 12.22           N  0.018
ANISOU  415  N   GLY A  26     1463   1700   1479     90    -68     29       N
ATOM    416  CA  GLY A  26      -7.549  27.988  12.037  1.00 12.55           C  0.019
ANISOU  416  CA  GLY A  26     1647   1655   1465     -1   -241     37       C
ATOM    417  C   GLY A  26      -6.392  28.346  12.952  1.00 11.32           C  0.018
ANISOU  417  C   GLY A  26     1475   1599   1228     74    -93    -13       C
ATOM    418  O   GLY A  26      -5.234  28.376  12.510  1.00 11.62           O  0.018
ANISOU  418  O   GLY A  26     1585   1559   1271     24   -150     84       O
ATOM    419  H   GLY A  26      -9.414  27.812  12.669  1.00 14.66           H  0.020
ATOM    420  HA2 GLY A  26      -7.223  27.373  11.361  1.00 15.06           H  0.020
ATOM    421  HA3 GLY A  26      -7.847  28.798  11.594  1.00 15.06           H  0.020
ATOM    422  N   ASN A  27      -6.688  28.726  14.204  1.00 11.80           N  0.018
ANISOU  422  N   ASN A  27     1520   1676   1289    109    -65    -72       N
ATOM    423  CA  ASN A  27      -5.609  29.005  15.150  1.00 11.36           C  0.018
ANISOU  423  CA  ASN A  27     1560   1572   1183    107   -113   -126       C
ATOM    424  C   ASN A  27      -4.711  27.795  15.341  1.00 11.61           C  0.018
ANISOU  424  C   ASN A  27     1541   1518   1354    161   -108     -4       C
ATOM    425  O   ASN A  27      -3.480  27.922  15.437  1.00 11.18           O  0.017
ANISOU  425  O   ASN A  27     1404   1512   1332    180    -83    -75       O
ATOM    426  CB  ASN A  27      -6.180  29.421  16.500  1.00 11.83           C  0.018
```

```
ANISOU  426  CB  ASN A  27     1615   1620   1260     15     54    -33       C
ATOM    427  CG  ASN A  27      -6.643  30.847  16.490  1.00 12.19           C  0.018
ANISOU  427  CG  ASN A  27     1555   1767   1309    164     11   -131       C
ATOM    428  OD1 ASN A  27      -5.803  31.739  16.529  1.00 12.53           O  0.019
ANISOU  428  OD1 ASN A  27     1620   1643   1499    141   -142    -71       O
ATOM    429  ND2 ASN A  27      -7.960  31.080  16.444  1.00 13.96           N  0.020
ANISOU  429  ND2 ASN A  27     1612   2189   1503    366     32   -107       N
ATOM    430  H   ASN A  27      -7.482  28.826  14.519  1.00 14.16           H  0.020
ATOM    431  HA  ASN A  27      -5.067  29.735  14.811  1.00 13.63           H  0.019
ATOM    432  HB2 ASN A  27      -6.939  28.857  16.715  1.00 14.20           H  0.020
ATOM    433  HB3 ASN A  27      -5.493  29.331  17.178  1.00 14.20           H  0.020
ATOM    434 HD21 ASN A  27      -8.254  31.888  16.438  1.00 16.75           H  0.021
ATOM    435 HD22 ASN A  27      -8.513  30.421  16.419  1.00 16.75           H  0.021
ATOM    436  N   TRP A  28      -5.319  26.614  15.440  1.00 11.89           N  0.018
ANISOU  436  N   TRP A  28     1532   1530   1454    114   -116      7       N
ATOM    437  CA  TRP A  28      -4.582  25.383  15.685  1.00 11.93           C  0.018
ANISOU  437  CA  TRP A  28     1589   1562   1382    127   -102    -89       C
ATOM    438  C   TRP A  28      -3.779  24.984  14.469  1.00 11.58           C  0.018
ANISOU  438  C   TRP A  28     1534   1418   1450     49   -178    -81       C
ATOM    439  O   TRP A  28      -2.667  24.479  14.594  1.00 11.90           O  0.018
ANISOU  439  O   TRP A  28     1517   1546   1460    100   -135     62       O
ATOM    440  CB  TRP A  28      -5.583  24.271  16.073  1.00 12.30           C  0.018
ANISOU  440  CB  TRP A  28     1609   1542   1521     59   -145    -29       C
ATOM    441  CG  TRP A  28      -6.272  24.547  17.400  1.00 12.50           C  0.018
ANISOU  441  CG  TRP A  28     1541   1575   1633     45   -152     62       C
ATOM    442  CD1 TRP A  28      -7.536  24.997  17.589  1.00 12.20           C  0.018
ANISOU  442  CD1 TRP A  28     1483   1721   1431     25    -72    -80       C
ATOM    443  CD2 TRP A  28      -5.697  24.440  18.702  1.00 11.92           C  0.018
ANISOU  443  CD2 TRP A  28     1496   1552   1480     79    -72    112       C
ATOM    444  NE1 TRP A  28      -7.776  25.220  18.908  1.00 13.36           N  0.019
ANISOU  444  NE1 TRP A  28     1443   1839   1795     12     19   -134       N
ATOM    445  CE2 TRP A  28      -6.670  24.845  19.622  1.00 12.13           C  0.018
ANISOU  445  CE2 TRP A  28     1434   1639   1536     68    -27     27       C
ATOM    446  CE3 TRP A  28      -4.463  24.016  19.170  1.00 12.17           C  0.018
ANISOU  446  CE3 TRP A  28     1583   1636   1405     84    -28    153       C
ATOM    447  CZ2 TRP A  28      -6.427  24.856  20.995  1.00 13.41           C  0.019
ANISOU  447  CZ2 TRP A  28     1655   1941   1501     32     49     52       C
ATOM    448  CZ3 TRP A  28      -4.222  24.023  20.531  1.00 13.40           C  0.019
ANISOU  448  CZ3 TRP A  28     1794   1894   1405    158    -99    206       C
ATOM    449  CH2 TRP A  28      -5.190  24.445  21.420  1.00 14.16           C  0.020
ANISOU  449  CH2 TRP A  28     1870   2116   1395     -2    -32    222       C
ATOM    450  H   TRP A  28      -6.169  26.501  15.368  1.00 14.26           H  0.020
ATOM    451  HA  TRP A  28      -3.970  25.516  16.426  1.00 14.32           H  0.020
ATOM    452  HB2 TRP A  28      -6.266  24.206  15.387  1.00 14.76           H  0.020
ATOM    453  HB3 TRP A  28      -5.107  23.429  16.150  1.00 14.76           H  0.020
ATOM    454  HD1 TRP A  28      -8.131  25.199  16.904  1.00 14.64           H  0.020
ATOM    455  HE1 TRP A  28      -8.521  25.490  19.243  1.00 16.04           H  0.021
ATOM    456  HE3 TRP A  28      -3.799  23.751  18.575  1.00 14.60           H  0.020
ATOM    457  HZ2 TRP A  28      -7.082  25.123  21.599  1.00 16.10           H  0.021
ATOM    458  HZ3 TRP A  28      -3.392  23.751  20.851  1.00 16.09           H  0.021
ATOM    459  HH2 TRP A  28      -5.007  24.426  22.332  1.00 17.00           H  0.022
ATOM    460  N   VAL A  29      -4.375  25.116  13.285  1.00 11.73           N  0.018
ANISOU  460  N   VAL A  29     1587   1557   1314     45   -252   -127       N
ATOM    461  CA  VAL A  29      -3.664  24.806  12.050  1.00 11.58           C  0.018
ANISOU  461  CA  VAL A  29     1605   1489   1307    114   -332   -145       C
ATOM    462  C   VAL A  29      -2.478  25.738  11.867  1.00 12.13           C  0.018
ANISOU  462  C   VAL A  29     1629   1566   1413    157   -359   -158       C
ATOM    463  O   VAL A  29      -1.371  25.285  11.529  1.00 12.25           O  0.018
ANISOU  463  O   VAL A  29     1559   1659   1436    162   -309   -134       O
ATOM    464  CB  VAL A  29      -4.620  24.804  10.839  1.00 12.52           C  0.019
ANISOU  464  CB  VAL A  29     1660   1696   1402     20   -186   -194       C
ATOM    465  CG1 VAL A  29      -3.850  24.622   9.539  1.00 13.07           C  0.019
ANISOU  465  CG1 VAL A  29     1719   1864   1383    128   -203   -213       C
ATOM    466  CG2 VAL A  29      -5.723  23.781  11.034  1.00 13.45           C  0.019
ANISOU  466  CG2 VAL A  29     1718   1832   1563    -50   -241   -146       C
ATOM    467  H   VAL A  29      -5.185  25.382  13.172  1.00 14.08           H  0.020
ATOM    468  HA  VAL A  29      -3.307  23.908  12.129  1.00 13.90           H  0.020
ATOM    469  HB  VAL A  29      -5.047  25.674  10.794  1.00 15.03           H  0.020
ATOM    470 HG11 VAL A  29      -4.477  24.413   8.830  1.00 15.69           H  0.021
ATOM    471 HG12 VAL A  29      -3.379  25.444   9.330  1.00 15.69           H  0.021
ATOM    472 HG13 VAL A  29      -3.218  23.894   9.646  1.00 15.69           H  0.021
ATOM    473 HG21 VAL A  29      -6.281  23.764  10.241  1.00 16.15           H  0.021
ATOM    474 HG22 VAL A  29      -5.323  22.909  11.176  1.00 16.15           H  0.021
ATOM    475 HG23 VAL A  29      -6.254  24.031  11.806  1.00 16.15           H  0.021
ATOM    476  N   CYS A  30      -2.685  27.051  12.068  1.00 11.43           N  0.018
ANISOU  476  N   CYS A  30     1535   1479   1327    118   -274   -107       N
ATOM    477  CA  CYS A  30      -1.606  28.029  11.990  1.00 11.18           C  0.017
ANISOU  477  CA  CYS A  30     1478   1573   1198    107   -139   -128       C
```

```
ATOM    478  C   CYS A  30      -0.500  27.687  12.987  1.00 11.36           C  0.018
ANISOU  478  C   CYS A  30     1467   1516   1334     97   -124    -97       C
ATOM    479  O   CYS A  30       0.685  27.719  12.642  1.00 11.36           O  0.018
ANISOU  479  O   CYS A  30     1502   1558   1255     97   -185    -41       O
ATOM    480  CB  CYS A  30      -2.181  29.426  12.238  1.00 12.07           C  0.018
ANISOU  480  CB  CYS A  30     1544   1674   1369    -13   -211     61       C
ATOM    481  SG  CYS A  30      -1.015  30.752  12.042  1.00 12.28           S  0.018
ANISOU  481  SG  CYS A  30     1611   1625   1430     41   -106     82       S
ATOM    482  H   CYS A  30      -3.451  27.395  12.252  1.00 13.71           H  0.019
ATOM    483  HA  CYS A  30      -1.225  28.013  11.098  1.00 13.42           H  0.019
ATOM    484  HB2 CYS A  30      -2.914  29.577  11.626  1.00 14.49           H  0.020
ATOM    485  HB3 CYS A  30      -2.510  29.463  13.150  1.00 14.49           H  0.020
ATOM    486  N   ALA A  31      -0.856  27.354  14.230  1.00 11.31           N  0.018
ANISOU  486  N   ALA A  31     1494   1577   1227    110   -146     -7       N
ATOM    487  CA  ALA A  31       0.168  27.004  15.205  1.00 11.47           C  0.018
ANISOU  487  CA  ALA A  31     1477   1731   1150    152   -224   -111       C
ATOM    488  C   ALA A  31       0.984  25.807  14.733  1.00 11.45           C  0.018
ANISOU  488  C   ALA A  31     1391   1785   1173     85   -223   -100       C
ATOM    489  O   ALA A  31       2.206  25.807  14.834  1.00 11.70           O  0.018
ANISOU  489  O   ALA A  31     1354   1781   1310    146   -165   -105       O
ATOM    490  CB  ALA A  31      -0.456  26.725  16.572  1.00 12.65           C  0.019
ANISOU  490  CB  ALA A  31     1567   1778   1462    135   -137   -105       C
ATOM    491  H   ALA A  31      -1.663  27.324  14.525  1.00 13.58           H  0.019
ATOM    492  HA  ALA A  31       0.774  27.755  15.304  1.00 13.76           H  0.019
ATOM    493  HB1 ALA A  31       0.248  26.495  17.199  1.00 15.18           H  0.020
ATOM    494  HB2 ALA A  31      -0.922  27.520  16.874  1.00 15.18           H  0.020
ATOM    495  HB3 ALA A  31      -1.080  25.987  16.489  1.00 15.18           H  0.020
ATOM    496  N   ALA A  32       0.316  24.765  14.238  1.00 11.43           N  0.018
ANISOU  496  N   ALA A  32     1407   1653   1284     87   -221   -181       N
ATOM    497  CA  ALA A  32       1.029  23.579  13.757  1.00 11.94           C  0.018
ANISOU  497  CA  ALA A  32     1549   1679   1309    188   -195    -79       C
ATOM    498  C   ALA A  32       1.939  23.913  12.582  1.00 11.16           C  0.017
ANISOU  498  C   ALA A  32     1553   1555   1132    203   -168   -178       C
ATOM    499  O   ALA A  32       3.059  23.389  12.487  1.00 12.21           O  0.018
ANISOU  499  O   ALA A  32     1534   1716   1387    194   -124   -150       O
ATOM    500  CB  ALA A  32       0.039  22.485  13.380  1.00 12.46           C  0.018
ANISOU  500  CB  ALA A  32     1669   1667   1399    203   -227   -188       C
ATOM    501  H   ALA A  32      -0.540  24.718  14.170  1.00 13.72           H  0.019
ATOM    502  HA  ALA A  32       1.586  23.239  14.474  1.00 14.33           H  0.020
ATOM    503  HB1 ALA A  32       0.529  21.721  13.039  1.00 14.95           H  0.020
ATOM    504  HB2 ALA A  32      -0.466  22.230  14.168  1.00 14.95           H  0.020
ATOM    505  HB3 ALA A  32      -0.561  22.824  12.697  1.00 14.95           H  0.020
ATOM    506  N   LYS A  33       1.479  24.770  11.661  1.00 11.76           N  0.018
ANISOU  506  N   LYS A  33     1656   1640   1171    113   -151    -52       N
ATOM    507  CA  LYS A  33       2.310  25.171  10.528  1.00 12.42           C  0.018
ANISOU  507  CA  LYS A  33     1797   1720   1203     85   -196   -112       C
ATOM    508  C   LYS A  33       3.628  25.757  11.015  1.00 11.91           C  0.018
ANISOU  508  C   LYS A  33     1696   1675   1155      4     -9   -124       C
ATOM    509  O   LYS A  33       4.696  25.370  10.543  1.00 12.82           O  0.019
ANISOU  509  O   LYS A  33     1763   1829   1278    -32    -19   -140       O
ATOM    510  CB  LYS A  33       1.570  26.216   9.663  1.00 13.88           C  0.019
ANISOU  510  CB  LYS A  33     2092   2022   1160      0   -359     86       C
ATOM    511  CG  LYS A  33       2.420  26.867   8.608  1.00 16.26           C  0.021
ANISOU  511  CG  LYS A  33     2461   2361   1355     14   -227    149       C
ATOM    512  CD  LYS A  33       2.910  25.882   7.604  1.00 19.01           C  0.023
ANISOU  512  CD  LYS A  33     2723   2774   1727     96    -47    326       C
ATOM    513  CE  LYS A  33       3.824  26.556   6.605  1.00 21.93           C  0.024
ANISOU  513  CE  LYS A  33     3093   3135   2104    406    287    567       C
ATOM    514  NZ  LYS A  33       4.078  25.674   5.426  1.00 24.05           N  0.026
ANISOU  514  NZ  LYS A  33     3381   3372   2384    548    329    479       N
ATOM    515  H   LYS A  33       0.697  25.128  11.672  1.00 14.11           H  0.020
ATOM    516  HA  LYS A  33       2.502  24.395   9.979  1.00 14.91           H  0.020
ATOM    517  HB2 LYS A  33       0.828  25.780   9.216  1.00 16.66           H  0.021
ATOM    518  HB3 LYS A  33       1.234  26.917  10.243  1.00 16.66           H  0.021
ATOM    519  HG2 LYS A  33       1.892  27.527   8.141  1.00 19.51           H  0.023
ATOM    520  HG3 LYS A  33       3.188  27.296   9.016  1.00 19.51           H  0.023
ATOM    521  HD2 LYS A  33       3.412  25.182   8.050  1.00 22.82           H  0.025
ATOM    522  HD3 LYS A  33       2.155  25.506   7.125  1.00 22.82           H  0.025
ATOM    523  HE2 LYS A  33       3.408  27.374   6.290  1.00 26.32           H  0.027
ATOM    524  HE3 LYS A  33       4.674  26.752   7.029  1.00 26.32           H  0.027
ATOM    525  HZ1 LYS A  33       4.462  24.916   5.691  1.00 28.86           H  0.028
ATOM    526  HZ2 LYS A  33       3.310  25.482   5.019  1.00 28.86           H  0.028
ATOM    527  HZ3 LYS A  33       4.617  26.085   4.849  1.00 28.86           H  0.028
ATOM    528  N   PHE A  34       3.574  26.691  11.952  1.00 11.65           N  0.018
ANISOU  528  N   PHE A  34     1653   1656   1118    -61    -68     41       N
ATOM    529  CA  PHE A  34       4.781  27.408  12.336  1.00 12.31           C  0.018
ANISOU  529  CA  PHE A  34     1637   1736   1304   -249    -99     -1       C
ATOM    530  C   PHE A  34       5.560  26.695  13.425  1.00 13.28           C  0.019
```

```
ANISOU  530  C   PHE A  34     1651   1898   1496   -236   -145    118       C
ATOM    531  O   PHE A  34       6.752  26.922  13.546  1.00 14.98           O  0.020
ANISOU  531  O   PHE A  34     1744   2214   1734   -432   -306    305       O
ATOM    532  CB  PHE A  34       4.450  28.861  12.681  1.00 12.57           C  0.019
ANISOU  532  CB  PHE A  34     1697   1708   1372   -218    111   -121       C
ATOM    533  CG  PHE A  34       3.969  29.630  11.479  1.00 12.92           C  0.019
ANISOU  533  CG  PHE A  34     1744   1741   1426   -325    136     61       C
ATOM    534  CD1 PHE A  34       4.719  29.644  10.310  1.00 13.58           C  0.019
ANISOU  534  CD1 PHE A  34     1766   1821   1572   -326   -155    137       C
ATOM    535  CD2 PHE A  34       2.767  30.302  11.492  1.00 15.10           C  0.020
ANISOU  535  CD2 PHE A  34     1887   1998   1853   -156    237    263       C
ATOM    536  CE1 PHE A  34       4.255  30.324   9.200  1.00 14.65           C  0.020
ANISOU  536  CE1 PHE A  34     1878   1950   1740   -325    -63    334       C
ATOM    537  CE2 PHE A  34       2.342  31.024  10.404  1.00 16.81           C  0.021
ANISOU  537  CE2 PHE A  34     1968   2172   2246   -197     94    469       C
ATOM    538  CZ  PHE A  34       3.076  31.022   9.262  1.00 15.97           C  0.021
ANISOU  538  CZ  PHE A  34     2069   2120   1880   -268    -46    544       C
ATOM    539  H   PHE A  34       2.864  26.927  12.376  1.00 13.98           H  0.020
ATOM    540  HA  PHE A  34       5.368  27.428  11.565  1.00 14.77           H  0.020
ATOM    541  HB2 PHE A  34       3.749  28.878  13.351  1.00 15.09           H  0.020
ATOM    542  HB3 PHE A  34       5.247  29.297  13.020  1.00 15.09           H  0.020
ATOM    543  HD1 PHE A  34       5.522  29.177  10.267  1.00 16.30           H  0.021
ATOM    544  HD2 PHE A  34       2.262  30.316  12.273  1.00 18.12           H  0.022
ATOM    545  HE1 PHE A  34       4.765  30.345   8.423  1.00 17.58           H  0.022
ATOM    546  HE2 PHE A  34       1.528  31.474  10.433  1.00 20.17           H  0.023
ATOM    547  HZ  PHE A  34       2.773  31.488   8.516  1.00 19.17           H  0.023
ATOM    548  N   GLU A  35       4.942  25.804  14.182  1.00 11.90           N  0.018
ANISOU  548  N   GLU A  35     1562   1735   1223   -133   -191    -22       N
ATOM    549  CA  GLU A  35       5.710  25.010  15.136  1.00 12.47           C  0.018
ANISOU  549  CA  GLU A  35     1585   1829   1326    -52   -248     35       C
ATOM    550  C   GLU A  35       6.490  23.902  14.438  1.00 13.19           C  0.019
ANISOU  550  C   GLU A  35     1650   2107   1255    115   -284     81       C
ATOM    551  O   GLU A  35       7.641  23.650  14.781  1.00 13.83           O  0.019
ANISOU  551  O   GLU A  35     1573   2319   1362    117   -229   -135       O
ATOM    552  CB  GLU A  35       4.775  24.404  16.176  1.00 12.18           C  0.018
ANISOU  552  CB  GLU A  35     1623   1757   1249    -32   -155     42       C
ATOM    553  CG  GLU A  35       4.228  25.425  17.167  1.00 13.32           C  0.019
ANISOU  553  CG  GLU A  35     1768   1878   1414    -47   -160    -30       C
ATOM    554  CD  GLU A  35       5.250  26.068  18.059  1.00 13.28           C  0.019
ANISOU  554  CD  GLU A  35     1785   1945   1315     47   -231     70       C
ATOM    555  OE1 GLU A  35       6.424  25.624  18.077  1.00 13.70           O  0.019
ANISOU  555  OE1 GLU A  35     1938   1877   1391    132   -466    -91       O
ATOM    556  OE2 GLU A  35       4.889  27.039  18.769  1.00 14.11           O  0.020
ANISOU  556  OE2 GLU A  35     1843   2203   1316     46     91   -103       O
ATOM    557  H   GLU A  35       4.098  25.639  14.168  1.00 14.28           H  0.020
ATOM    558  HA  GLU A  35       6.344  25.584  15.593  1.00 14.97           H  0.020
ATOM    559  HB2 GLU A  35       4.021  23.997  15.721  1.00 14.62           H  0.020
ATOM    560  HB3 GLU A  35       5.259  23.731  16.679  1.00 14.62           H  0.020
ATOM    561  HG2 GLU A  35       3.791  26.134  16.669  1.00 15.98           H  0.021
ATOM    562  HG3 GLU A  35       3.580  24.983  17.738  1.00 15.98           H  0.021
ATOM    563  N   SER A  36       5.878  23.216  13.483  1.00 13.27           N  0.019
ANISOU  563  N   SER A  36     1674   2011   1356    221   -209   -230       N
ATOM    564  CA  SER A  36       6.438  21.979  12.967  1.00 13.99           C  0.020
ANISOU  564  CA  SER A  36     1708   2173   1435    315   -158   -252       C
ATOM    565  C   SER A  36       6.433  21.846  11.459  1.00 13.76           C  0.019
ANISOU  565  C   SER A  36     1719   2179   1329    324    -94   -222       C
ATOM    566  O   SER A  36       6.864  20.806  10.953  1.00 14.84           O  0.020
ANISOU  566  O   SER A  36     1755   2310   1573    424   -116   -298       O
ATOM    567  CB  SER A  36       5.677  20.778  13.514  1.00 13.95           C  0.020
ANISOU  567  CB  SER A  36     1795   2134   1370    357   -115   -184       C
ATOM    568  OG  SER A  36       4.362  20.768  12.960  1.00 13.21           O  0.019
ANISOU  568  OG  SER A  36     1763   1829   1425    301   -227   -175       O
ATOM    569  H   SER A  36       5.135  23.447  13.117  1.00 15.92           H  0.021
ATOM    570  HA  SER A  36       7.358  21.902  13.261  1.00 16.79           H  0.021
ATOM    571  HB2 SER A  36       6.138  19.963  13.262  1.00 16.74           H  0.021
ATOM    572  HB3 SER A  36       5.618  20.847  14.480  1.00 16.74           H  0.021
ATOM    573  HG  SER A  36       3.957  21.474  13.169  1.00 15.85           H  0.021
ATOM    574  N   ASN A  37       5.897  22.808  10.736  1.00 14.35           N  0.020
ANISOU  574  N   ASN A  37     1921   2189   1341    267    -74   -107       N
ATOM    575  CA  ASN A  37       5.679  22.690   9.308  1.00 15.61           C  0.021
ANISOU  575  CA  ASN A  37     2264   2318   1349    405    -93   -156       C
ATOM    576  C   ASN A  37       4.834  21.454   8.987  1.00 14.75           C  0.020
ANISOU  576  C   ASN A  37     2201   2194   1209    472   -196   -262       C
ATOM    577  O   ASN A  37       5.009  20.812   7.956  1.00 15.63           O  0.021
ANISOU  577  O   ASN A  37     2265   2329   1343    504   -162   -252       O
ATOM    578  CB  ASN A  37       6.960  22.742   8.475  1.00 17.91           C  0.022
ANISOU  578  CB  ASN A  37     2585   2663   1557    250     40     35       C
ATOM    579  CG  ASN A  37       6.677  23.157   7.026  1.00 20.87           C  0.024
```

```
ANISOU  579  CG  ASN A  37     2866   2981   2081    360    208    -20       C
ATOM    580  OD1 ASN A  37       5.595  23.667   6.704  1.00 21.94           O  0.025
ANISOU  580  OD1 ASN A  37     3176   3112   2047    373    211     97       O
ATOM    581  ND2 ASN A  37       7.644  22.938   6.151  1.00 22.39           N  0.025
ANISOU  581  ND2 ASN A  37     2942   3145   2420    318    485     44       N
ATOM    582  H   ASN A  37       5.643  23.563  11.059  1.00 17.22           H  0.022
ATOM    583  HA  ASN A  37       5.149  23.456   9.039  1.00 18.74           H  0.023
ATOM    584  HB2 ASN A  37       7.568  23.391   8.863  1.00 21.49           H  0.024
ATOM    585  HB3 ASN A  37       7.370  21.863   8.464  1.00 21.49           H  0.024
ATOM    586 HD21 ASN A  37       7.534  23.153   5.326  1.00 26.87           H  0.027
ATOM    587 HD22 ASN A  37       8.383  22.582   6.408  1.00 26.87           H  0.027
ATOM    588  N   PHE A  38       3.917  21.112   9.895  1.00 13.68           N  0.019
ANISOU  588  N   PHE A  38     1910   2031   1257    461   -252   -135       N
ATOM    589  CA  PHE A  38       2.957  20.022   9.746  1.00 13.87           C  0.019
ANISOU  589  CA  PHE A  38     1845   2036   1390    424   -346   -291       C
ATOM    590  C   PHE A  38       3.614  18.635   9.840  1.00 13.65           C  0.019
ANISOU  590  C   PHE A  38     1848   2009   1330    360   -383   -273       C
ATOM    591  O   PHE A  38       2.982  17.642   9.498  1.00 15.09           O  0.020
ANISOU  591  O   PHE A  38     1827   2113   1794    439   -411   -450       O
ATOM    592  CB  PHE A  38       2.141  20.145   8.452  1.00 14.53           C  0.020
ANISOU  592  CB  PHE A  38     1933   2030   1559    464   -608   -286       C
ATOM    593  CG  PHE A  38       1.290  21.369   8.327  1.00 13.66           C  0.019
ANISOU  593  CG  PHE A  38     1894   2021   1276    388   -528   -199       C
ATOM    594  CD1 PHE A  38       0.623  21.957   9.403  1.00 13.60           C  0.019
ANISOU  594  CD1 PHE A  38     1783   2009   1376    343   -386   -233       C
ATOM    595  CD2 PHE A  38       1.095  21.893   7.087  1.00 16.11           C  0.021
ANISOU  595  CD2 PHE A  38     2182   2323   1618    472   -415   -174       C
ATOM    596  CE1 PHE A  38      -0.220  23.033   9.208  1.00 14.26           C  0.020
ANISOU  596  CE1 PHE A  38     1885   2051   1483    405   -437   -286       C
ATOM    597  CE2 PHE A  38       0.288  22.989   6.911  1.00 15.73           C  0.021
ANISOU  597  CE2 PHE A  38     2130   2289   1557    515   -472    -99       C
ATOM    598  CZ  PHE A  38      -0.381  23.551   7.962  1.00 15.01           C  0.020
ANISOU  598  CZ  PHE A  38     2009   2074   1620    506   -620   -363       C
ATOM    599  H   PHE A  38       3.830  21.523  10.645  1.00 16.42           H  0.021
ATOM    600  HA  PHE A  38       2.328  20.080  10.482  1.00 16.65           H  0.021
ATOM    601  HB2 PHE A  38       2.750  20.137   7.698  1.00 17.44           H  0.022
ATOM    602  HB3 PHE A  38       1.550  19.378   8.393  1.00 17.44           H  0.022
ATOM    603  HD1 PHE A  38       0.715  21.594  10.254  1.00 16.32           H  0.021
ATOM    604  HD2 PHE A  38       1.525  21.515   6.354  1.00 19.34           H  0.023
ATOM    605  HE1 PHE A  38      -0.648  23.426   9.934  1.00 17.11           H  0.022
ATOM    606  HE2 PHE A  38       0.171  23.337   6.057  1.00 18.88           H  0.023
ATOM    607  HZ  PHE A  38      -0.919  24.298   7.830  1.00 18.01           H  0.022
ATOM    608  N   ASN A  39       4.860  18.536  10.315  1.00 13.51           N  0.019
ANISOU  608  N   ASN A  39     1855   1938   1339    453   -546   -182       N
ATOM    609  CA  ASN A  39       5.581  17.263  10.437  1.00 13.55           C  0.019
ANISOU  609  CA  ASN A  39     1767   1868   1513    514   -426   -334       C
ATOM    610  C   ASN A  39       5.421  16.718  11.854  1.00 12.94           C  0.019
ANISOU  610  C   ASN A  39     1596   1778   1544    414   -284   -381       C
ATOM    611  O   ASN A  39       5.943  17.292  12.820  1.00 13.10           O  0.019
ANISOU  611  O   ASN A  39     1721   1809   1446    287   -222   -253       O
ATOM    612  CB  ASN A  39       7.041  17.508  10.102  1.00 14.53           C  0.020
ANISOU  612  CB  ASN A  39     1922   1948   1652    445   -246   -104       C
ATOM    613  CG  ASN A  39       7.852  16.249  10.020  1.00 14.59           C  0.020
ANISOU  613  CG  ASN A  39     1766   2024   1755    398   -187   -242       C
ATOM    614  OD1 ASN A  39       7.381  15.167  10.400  1.00 14.23           O  0.020
ANISOU  614  OD1 ASN A  39     1783   2007   1616    305   -281   -391       O
ATOM    615  ND2 ASN A  39       9.076  16.351   9.479  1.00 16.44           N  0.021
ANISOU  615  ND2 ASN A  39     1860   2061   2326    344     98   -182       N
ATOM    616  H   ASN A  39       5.321  19.211  10.581  1.00 16.21           H  0.021
ATOM    617  HA  ASN A  39       5.220  16.617   9.810  1.00 16.26           H  0.021
ATOM    618  HB2 ASN A  39       7.095  17.954   9.242  1.00 17.44           H  0.022
ATOM    619  HB3 ASN A  39       7.431  18.069  10.790  1.00 17.44           H  0.022
ATOM    620 HD21 ASN A  39       9.574  15.654   9.408  1.00 19.73           H  0.023
ATOM    621 HD22 ASN A  39       9.369  17.117   9.219  1.00 19.73           H  0.023
ATOM    622  N   THR A  40       4.705  15.592  11.985  1.00 13.74           N  0.019
ANISOU  622  N   THR A  40     1703   1817   1700    353   -360   -228       N
ATOM    623  CA  THR A  40       4.534  15.017  13.308  1.00 14.67           C  0.020
ANISOU  623  CA  THR A  40     1703   1857   2016    363   -261   -319       C
ATOM    624  C   THR A  40       5.847  14.581  13.948  1.00 13.69           C  0.019
ANISOU  624  C   THR A  40     1687   1897   1617    434   -237    -72       C
ATOM    625  O   THR A  40       5.893  14.433  15.172  1.00 14.49           O  0.020
ANISOU  625  O   THR A  40     1884   1981   1641    486   -131   -119       O
ATOM    626  CB  THR A  40       3.607  13.795  13.312  1.00 17.09           C  0.022
ANISOU  626  CB  THR A  40     1639   1929   2924    417    -49   -432       C
ATOM    627  OG1 THR A  40       4.223  12.714  12.594  1.00 18.63           O  0.023
ANISOU  627  OG1 THR A  40     1855   2040   3183    355   -268   -601       O
ATOM    628  CG2 THR A  40       2.274  14.071  12.683  1.00 16.78           C  0.021
ANISOU  628  CG2 THR A  40     1649   1971   2755    398   -381   -206       C
```

```
ATOM    629  H   THR A  40       4.327  15.162  11.344  1.00 16.49           H  0.021
ATOM    630  HA  THR A  40       4.146  15.686  13.889  1.00 17.61           H  0.022
ATOM    631  HB  THR A  40       3.454  13.518  14.229  1.00 20.50           H  0.024
ATOM    632  HG1 THR A  40       3.717  12.043  12.595  1.00 22.36           H  0.025
ATOM    633 HG21 THR A  40       1.826  13.234  12.485  1.00 20.14           H  0.023
ATOM    634 HG22 THR A  40       1.719  14.585  13.289  1.00 20.14           H  0.023
ATOM    635 HG23 THR A  40       2.386  14.567  11.858  1.00 20.14           H  0.023
ATOM    636  N   GLN A  41       6.904  14.339  13.171  1.00 13.76           N  0.019
ANISOU  636  N   GLN A  41     1617   1998   1612    403   -199   -262       N
ATOM    637  CA  GLN A  41       8.175  13.847  13.719  1.00 13.43           C  0.019
ANISOU  637  CA  GLN A  41     1655   1993   1456    416   -288   -294       C
ATOM    638  C   GLN A  41       9.149  14.956  14.063  1.00 13.85           C  0.019
ANISOU  638  C   GLN A  41     1700   2087   1477    373   -346   -391       C
ATOM    639  O   GLN A  41      10.274  14.670  14.468  1.00 16.04           O  0.021
ANISOU  639  O   GLN A  41     1787   2364   1942    482   -544   -541       O
ATOM    640  CB  GLN A  41       8.816  12.840  12.752  1.00 14.39           C  0.020
ANISOU  640  CB  GLN A  41     1635   2033   1801    454   -293   -204       C
ATOM    641  CG  GLN A  41       7.938  11.636  12.478  1.00 14.72           C  0.020
ANISOU  641  CG  GLN A  41     1786   2101   1704    516   -123   -207       C
ATOM    642  CD  GLN A  41       8.677  10.509  11.851  1.00 15.19           C  0.020
ANISOU  642  CD  GLN A  41     1900   2037   1834    498   -154   -196       C
ATOM    643  OE1 GLN A  41       9.387   9.759  12.522  1.00 16.81           O  0.021
ANISOU  643  OE1 GLN A  41     2256   2083   2047    759   -292   -217       O
ATOM    644  NE2 GLN A  41       8.586  10.419  10.532  1.00 14.73           N  0.020
ANISOU  644  NE2 GLN A  41     1808   1973   1815    377    -78   -335       N
ATOM    645  H   GLN A  41       6.913  14.452  12.318  1.00 16.51           H  0.021
ATOM    646  HA  GLN A  41       7.985  13.370  14.542  1.00 16.12           H  0.021
ATOM    647  HB2 GLN A  41       8.989  13.282  11.906  1.00 17.27           H  0.022
ATOM    648  HB3 GLN A  41       9.648  12.522  13.135  1.00 17.27           H  0.022
ATOM    649  HG2 GLN A  41       7.566  11.320  13.316  1.00 17.66           H  0.022
ATOM    650  HG3 GLN A  41       7.224  11.897  11.876  1.00 17.66           H  0.022
ATOM    651 HE21 GLN A  41       8.090  10.970  10.097  1.00 17.68           H  0.022
ATOM    652 HE22 GLN A  41       8.998   9.790  10.115  1.00 17.68           H  0.022
ATOM    653  N   ALA A  42       8.736  16.215  13.981  1.00 13.76           N  0.019
ANISOU  653  N   ALA A  42     1734   2019   1476    371   -253   -379       N
ATOM    654  CA  ALA A  42       9.637  17.311  14.311  1.00 13.30           C  0.019
ANISOU  654  CA  ALA A  42     1631   2152   1271    242    -89   -317       C
ATOM    655  C   ALA A  42      10.085  17.251  15.767  1.00 13.09           C  0.019
ANISOU  655  C   ALA A  42     1625   2107   1244    324   -101   -261       C
ATOM    656  O   ALA A  42       9.273  17.028  16.668  1.00 12.89           O  0.019
ANISOU  656  O   ALA A  42     1679   1825   1395    235    -55   -161       O
ATOM    657  CB  ALA A  42       8.922  18.649  14.090  1.00 14.64           C  0.020
ANISOU  657  CB  ALA A  42     1878   2209   1475    232   -175   -288       C
ATOM    658  H   ALA A  42       7.948  16.461  13.740  1.00 16.51           H  0.021
ATOM    659  HA  ALA A  42      10.421  17.276  13.740  1.00 15.96           H  0.021
ATOM    660  HB1 ALA A  42       9.530  19.372  14.312  1.00 17.57           H  0.022
ATOM    661  HB2 ALA A  42       8.656  18.714  13.160  1.00 17.57           H  0.022
ATOM    662  HB3 ALA A  42       8.139  18.687  14.662  1.00 17.57           H  0.022
ATOM    663  N   THR A  43      11.380  17.500  15.988  1.00 14.10           N  0.020
ANISOU  663  N   THR A  43     1665   2392   1301    253    -48   -380       N
ATOM    664  CA  THR A  43      11.956  17.649  17.319  1.00 14.30           C  0.020
ANISOU  664  CA  THR A  43     1727   2502   1206    152    -71   -379       C
ATOM    665  C   THR A  43      12.906  18.834  17.319  1.00 14.14           C  0.020
ANISOU  665  C   THR A  43     1729   2551   1092     12    -29   -108       C
ATOM    666  O   THR A  43      13.601  19.106  16.326  1.00 17.07           O  0.022
ANISOU  666  O   THR A  43     2055   3149   1284   -268    -13   -106       O
ATOM    667  CB  THR A  43      12.717  16.418  17.803  1.00 15.33           C  0.020
ANISOU  667  CB  THR A  43     1815   2556   1456    328    -46   -337       C
ATOM    668  OG1 THR A  43      13.813  16.158  16.927  1.00 15.66           O  0.021
ANISOU  668  OG1 THR A  43     1664   2667   1619    409   -215   -330       O
ATOM    669  CG2 THR A  43      11.806  15.199  17.924  1.00 15.37           C  0.021
ANISOU  669  CG2 THR A  43     1941   2403   1496    499     65   -156       C
ATOM    670  H   THR A  43      11.958  17.587  15.359  1.00 16.93           H  0.022
ATOM    671  HA  THR A  43      11.244  17.830  17.950  1.00 17.17           H  0.022
ATOM    672  HB  THR A  43      13.070  16.605  18.687  1.00 18.40           H  0.022
ATOM    673  HG1 THR A  43      13.531  16.019  16.148  1.00 18.79           H  0.023
ATOM    674 HG21 THR A  43      12.316  14.434  18.234  1.00 18.44           H  0.022
ATOM    675 HG22 THR A  43      11.093  15.380  18.556  1.00 18.44           H  0.022
ATOM    676 HG23 THR A  43      11.416  14.990  17.061  1.00 18.44           H  0.022
ATOM    677  N  AASN A  44      12.986  19.507  18.452  0.48 13.30           N  0.019
ANISOU  677  N  AASN A  44     1861   2188   1006    -72   -239    -26       N
ATOM    678  CA AASN A  44      13.900  20.635  18.588  0.48 13.96           C  0.020
ANISOU  678  CA AASN A  44     2058   2118   1127   -136   -310    -56       C
ATOM    679  C  AASN A  44      14.465  20.581  20.000  0.48 13.61           C  0.019
ANISOU  679  C  AASN A  44     1986   1954   1231     21   -286   -134       C
ATOM    680  O  AASN A  44      13.705  20.579  20.974  0.48 13.44           O  0.019
ANISOU  680  O  AASN A  44     1960   1879   1268    118   -241   -129       O
ATOM    681  CB AASN A  44      13.165  21.961  18.361  0.48 16.29           C  0.021
```

```
ANISOU  681  CB AASN A  44     2426   2204   1561   -238   -440     76       C
ATOM    682  CG AASN A  44      14.001  23.157  18.692  0.48 18.57           C  0.023
ANISOU  682  CG AASN A  44     2760   2209   2086   -281   -260    -45       C
ATOM    683  OD1AASN A  44      13.854  23.790  19.743  0.48 19.66           O  0.023
ANISOU  683  OD1AASN A  44     2947   2286   2236   -231   -196     85       O
ATOM    684  ND2AASN A  44      14.914  23.461  17.808  0.48 18.96           N  0.023
ANISOU  684  ND2AASN A  44     2840   2111   2252   -456    186     56       N
ATOM    685  H  AASN A  44      12.525  19.335  19.158  0.48 15.97           H  0.021
ATOM    686  HA AASN A  44      14.628  20.560  17.951  0.48 16.75           H  0.021
ATOM    687  HB2AASN A  44      12.909  22.024  17.427  0.48 19.55           H  0.023
ATOM    688  HB3AASN A  44      12.375  21.984  18.923  0.48 19.55           H  0.023
ATOM    689 HD21AASN A  44      14.990  22.993  17.090  0.48 22.75           H  0.025
ATOM    690 HD22AASN A  44      15.431  24.136  17.938  0.48 22.75           H  0.025
ATOM    691  N  BASN A  44      13.003  19.489  18.468  0.52 13.78           N  0.019
ANISOU  691  N  BASN A  44     1714   2343   1177    -47     -2     29       N
ATOM    692  CA BASN A  44      13.912  20.629  18.590  0.52 14.74           C  0.020
ANISOU  692  CA BASN A  44     1769   2412   1419    -95    146    150       C
ATOM    693  C  BASN A  44      14.432  20.676  20.027  0.52 13.50           C  0.019
ANISOU  693  C  BASN A  44     1666   2149   1314    -31    175     55       C
ATOM    694  O  BASN A  44      13.689  20.936  20.979  0.52 12.94           O  0.019
ANISOU  694  O  BASN A  44     1600   2117   1201    -16    325     50       O
ATOM    695  CB BASN A  44      13.209  21.914  18.140  0.52 18.70           C  0.023
ANISOU  695  CB BASN A  44     2033   2736   2336   -146     13    394       C
ATOM    696  CG BASN A  44      13.056  21.994  16.589  0.52 21.36           C  0.024
ANISOU  696  CG BASN A  44     2131   2960   3024   -232    287    427       C
ATOM    697  OD1BASN A  44      14.004  22.307  15.859  0.52 23.92           O  0.026
ANISOU  697  OD1BASN A  44     2190   3244   3656   -359   -155     96       O
ATOM    698  ND2BASN A  44      11.857  21.682  16.096  0.52 22.56           N  0.025
ANISOU  698  ND2BASN A  44     2175   2855   3543   -233    623    449       N
ATOM    699  H  BASN A  44      12.563  19.301  19.183  0.52 16.53           H  0.021
ATOM    700  HA BASN A  44      14.674  20.489  18.006  0.52 17.69           H  0.022
ATOM    701  HB2BASN A  44      12.322  21.945  18.532  0.52 22.44           H  0.025
ATOM    702  HB3BASN A  44      13.729  22.679  18.431  0.52 22.44           H  0.025
ATOM    703 HD21BASN A  44      11.217  21.464  16.627  0.52 27.08           H  0.027
ATOM    704 HD22BASN A  44      11.721  21.708  15.247  0.52 27.08           H  0.027
ATOM    705  N  AARG A  45      15.790  20.567  20.121  0.48 13.82           N  0.019
ANISOU  705  N  AARG A  45     2074   1978   1200     88   -294   -178       N
ATOM    706  CA AARG A  45      16.414  20.580  21.431  0.48 14.77           C  0.020
ANISOU  706  CA AARG A  45     2248   1991   1372     -0   -426     34       C
ATOM    707  C  AARG A  45      16.679  22.018  21.828  0.48 16.00           C  0.021
ANISOU  707  C  AARG A  45     2663   1953   1464   -209   -216    169       C
ATOM    708  O  AARG A  45      17.299  22.779  21.073  0.48 17.60           O  0.022
ANISOU  708  O  AARG A  45     2880   2072   1734   -318    -18    -34       O
ATOM    709  CB AARG A  45      17.732  19.798  21.476  0.48 16.56           C  0.021
ANISOU  709  CB AARG A  45     2193   2224   1876    143   -887    236       C
ATOM    710  CG AARG A  45      18.297  19.635  22.907  0.48 18.17           C  0.022
ANISOU  710  CG AARG A  45     2291   2355   2257    112   -799    397       C
ATOM    711  CD AARG A  45      18.599  18.229  23.308  0.48 18.24           C  0.022
ANISOU  711  CD AARG A  45     2218   2447   2266    100   -479    442       C
ATOM    712  NE AARG A  45      18.629  18.034  24.755  0.48 18.47           N  0.022
ANISOU  712  NE AARG A  45     2067   2468   2481     33   -249    197       N
ATOM    713  CZ AARG A  45      19.343  17.101  25.362  0.48 18.05           C  0.022
ANISOU  713  CZ AARG A  45     1869   2522   2468    127   -116    206       C
ATOM    714  NH1AARG A  45      20.212  16.364  24.698  0.48 17.78           N  0.022
ANISOU  714  NH1AARG A  45     1761   2609   2386    144     57     97       N
ATOM    715  NH2AARG A  45      19.161  16.886  26.662  0.48 15.75           N  0.021
ANISOU  715  NH2AARG A  45     1698   2440   1846    387   -173    790       N
ATOM    716  HA AARG A  45      15.809  20.190  22.081  0.48 17.72           H  0.022
ATOM    717  HB2AARG A  45      17.585  18.913  21.108  0.48 19.87           H  0.023
ATOM    718  HB3AARG A  45      18.394  20.269  20.946  0.48 19.87           H  0.023
ATOM    719  HG2AARG A  45      19.122  20.142  22.973  0.48 21.80           H  0.024
ATOM    720  HG3AARG A  45      17.649  19.986  23.537  0.48 21.80           H  0.024
ATOM    721  HD2AARG A  45      17.917  17.643  22.944  0.48 21.89           H  0.024
ATOM    722  HD3AARG A  45      19.466  17.993  22.946  0.48 21.89           H  0.024
ATOM    723  HE AARG A  45      18.153  18.558  25.243  0.48 22.16           H  0.025
ATOM    724 HH11AARG A  45      20.334  16.483  23.856  0.48 21.34           H  0.024
ATOM    725 HH12AARG A  45      20.665  15.763  25.113  0.48 21.34           H  0.024
ATOM    726 HH21AARG A  45      18.598  17.364  27.102  0.48 18.90           H  0.023
ATOM    727 HH22AARG A  45      19.626  16.287  27.068  0.48 18.90           H  0.023
ATOM    728  N  BARG A  45      15.735  20.453  20.163  0.52 13.48           N  0.019
ANISOU  728  N  BARG A  45     1808   2072   1240     42    177    -90       N
ATOM    729  CA BARG A  45      16.431  20.548  21.437  0.52 14.38           C  0.020
ANISOU  729  CA BARG A  45     2045   1960   1459    -90     80     51       C
ATOM    730  C  BARG A  45      16.661  22.001  21.809  0.52 15.78           C  0.021
ANISOU  730  C  BARG A  45     2589   1947   1461   -259    -13    179       C
ATOM    731  O  BARG A  45      17.247  22.763  21.030  0.52 17.56           O  0.022
ANISOU  731  O  BARG A  45     2852   2091   1730   -361    109    -27       O
ATOM    732  CB BARG A  45      17.784  19.828  21.267  0.52 16.11           C  0.021
```

```
ANISOU  732  CB BARG A  45     1979   2071   2071     42   -219     94       C
ATOM    733  CG BARG A  45      18.771  19.931  22.402  0.52 16.27           C  0.021
ANISOU  733  CG BARG A  45     2032   2082   2068    -16    -65    105       C
ATOM    734  CD BARG A  45      18.343  19.087  23.597  0.52 13.94           C  0.020
ANISOU  734  CD BARG A  45     2075   1844   1378    -96    143    -77       C
ATOM    735  NE BARG A  45      17.624  17.867  23.237  0.52 15.22           N  0.020
ANISOU  735  NE BARG A  45     2052   1929   1802    -10    102   -188       N
ATOM    736  CZ BARG A  45      18.213  16.738  22.885  0.52 15.87           C  0.021
ANISOU  736  CZ BARG A  45     2321   1985   1723    -57     49   -200       C
ATOM    737  NH1BARG A  45      19.526  16.665  22.756  0.52 16.98           N  0.022
ANISOU  737  NH1BARG A  45     2354   2024   2076    141    369    -61       N
ATOM    738  NH2BARG A  45      17.467  15.657  22.663  0.52 15.32           N  0.020
ANISOU  738  NH2BARG A  45     2558   1873   1389     11   -167   -472       N
ATOM    739  H  BARG A  45      16.251  20.239  19.509  0.52 16.17           H  0.021
ATOM    740  HA BARG A  45      15.919  20.112  22.136  0.52 17.26           H  0.022
ATOM    741  HB2BARG A  45      17.615  18.891  21.090  0.52 19.33           H  0.023
ATOM    742  HB3BARG A  45      18.220  20.213  20.491  0.52 19.33           H  0.023
ATOM    743  HG2BARG A  45      19.634  19.607  22.100  0.52 19.53           H  0.023
ATOM    744  HG3BARG A  45      18.846  20.853  22.692  0.52 19.53           H  0.023
ATOM    745  HD2BARG A  45      19.132  18.833  24.100  0.52 16.73           H  0.021
ATOM    746  HD3BARG A  45      17.756  19.619  24.157  0.52 16.73           H  0.021
ATOM    747  HE BARG A  45      16.766  17.903  23.196  0.52 18.26           H  0.022
ATOM    748 HH11BARG A  45      20.016  17.357  22.901  0.52 20.38           H  0.024
ATOM    749 HH12BARG A  45      19.893  15.922  22.525  0.52 20.38           H  0.024
ATOM    750 HH21BARG A  45      16.612  15.701  22.744  0.52 18.38           H  0.022
ATOM    751 HH22BARG A  45      17.840  14.918  22.429  0.52 18.38           H  0.022
ATOM    752  N   ASN A  46      16.204  22.347  23.036  1.00 16.34           N  0.021
ANISOU  752  N   ASN A  46     2850   1903   1453   -292   -270      8       N
ATOM    753  CA  ASN A  46      16.393  23.732  23.554  1.00 19.36           C  0.023
ANISOU  753  CA  ASN A  46     3377   2115   1864   -236   -304    -88       C
ATOM    754  C   ASN A  46      17.731  23.843  24.285  1.00 21.66           C  0.024
ANISOU  754  C   ASN A  46     3617   2348   2265   -635   -572     43       C
ATOM    755  O   ASN A  46      18.317  22.828  24.620  1.00 22.09           O  0.025
ANISOU  755  O   ASN A  46     3468   2678   2248   -773   -549    147       O
ATOM    756  CB  ASN A  46      15.216  24.077  24.446  1.00 20.99           C  0.024
ANISOU  756  CB  ASN A  46     3578   2265   2134    195   -140   -286       C
ATOM    757  CG  ASN A  46      13.888  23.869  23.756  1.00 22.63           C  0.025
ANISOU  757  CG  ASN A  46     3712   2585   2300    550   -315    -97       C
ATOM    758  OD1 ASN A  46      13.013  23.183  24.264  1.00 23.23           O  0.025
ANISOU  758  OD1 ASN A  46     3750   2782   2296    674   -579   -240       O
ATOM    759  ND2 ASN A  46      13.743  24.454  22.584  1.00 24.01           N  0.026
ANISOU  759  ND2 ASN A  46     3835   2695   2592    621   -508    410       N
ATOM    760  HA  ASN A  46      16.402  24.354  22.810  1.00 23.23           H  0.025
ATOM    761  HB2 ASN A  46      15.238  23.511  25.233  1.00 25.19           H  0.026
ATOM    762  HB3 ASN A  46      15.278  25.010  24.705  1.00 25.19           H  0.026
ATOM    763 HD21 ASN A  46      13.007  24.367  22.148  1.00 28.81           H  0.028
ATOM    764 HD22 ASN A  46      14.382  24.927  22.257  1.00 28.81           H  0.028
ATOM    765  H  AASN A  46      15.783  21.816  23.565  0.48 19.60           H  0.023
ATOM    766  H  BASN A  46      15.793  21.817  23.575  0.52 19.60           H  0.023
ATOM    767  N   THR A  47      18.219  25.067  24.446  1.00 24.77           N  0.026
ANISOU  767  N   THR A  47     3963   2602   2845   -837   -538     81       N
ATOM    768  CA  THR A  47      19.509  25.289  25.091  1.00 28.13           C  0.028
ANISOU  768  CA  THR A  47     4238   2985   3465   -876   -588    223       C
ATOM    769  C   THR A  47      19.496  24.794  26.535  1.00 27.88           C  0.028
ANISOU  769  C   THR A  47     4279   3167   3148   -829   -973    159       C
ATOM    770  O   THR A  47      20.549  24.433  27.074  1.00 29.41           O  0.028
ANISOU  770  O   THR A  47     4287   3326   3561   -925   -992   -109       O
ATOM    771  CB  THR A  47      19.919  26.767  25.098  1.00 31.76           C  0.029
ANISOU  771  CB  THR A  47     4457   3144   4466   -864   -308    280       C
ATOM    772  OG1 THR A  47      18.789  27.612  25.311  1.00 33.03           O  0.030
ANISOU  772  OG1 THR A  47     4614   3003   4935   -767   -175    101       O
ATOM    773  CG2 THR A  47      20.567  27.127  23.780  1.00 33.30           C  0.030
ANISOU  773  CG2 THR A  47     4526   3303   4823   -859   -282    320       C
ATOM    774  H   THR A  47      17.824  25.787  24.191  1.00 29.72           H  0.029
ATOM    775  HA  THR A  47      20.190  24.792  24.611  1.00 33.76           H  0.030
ATOM    776  HB  THR A  47      20.567  26.915  25.805  1.00 38.11           H  0.032
ATOM    777  HG1 THR A  47      18.431  27.433  26.050  1.00 39.64           H  0.033
ATOM    778 HG21 THR A  47      20.816  28.065  23.778  1.00 39.96           H  0.033
ATOM    779 HG22 THR A  47      21.363  26.590  23.644  1.00 39.96           H  0.033
ATOM    780 HG23 THR A  47      19.948  26.964  23.051  1.00 39.96           H  0.033
ATOM    781  N   ASP A  48      18.323  24.771  27.180  1.00 26.02           N  0.027
ANISOU  781  N   ASP A  48     4244   3109   2536   -841  -1146     11       N
ATOM    782  CA  ASP A  48      18.220  24.298  28.557  1.00 25.52           C  0.026
ANISOU  782  CA  ASP A  48     4211   3028   2458   -671  -1064    -44       C
ATOM    783  C   ASP A  48      18.165  22.785  28.676  1.00 24.35           C  0.026
ANISOU  783  C   ASP A  48     3939   3023   2290   -711   -895    199       C
ATOM    784  O   ASP A  48      18.032  22.286  29.804  1.00 25.14           O  0.026
ANISOU  784  O   ASP A  48     4225   3102   2225   -748   -987    207       O
```

```
ATOM    785  CB  ASP A  48      17.019  24.921  29.278  1.00 25.27           C  0.026
ANISOU  785  CB  ASP A  48     4385   2986   2231   -438   -925   -329       C
ATOM    786  CG  ASP A  48      15.665  24.387  28.809  1.00 26.03           C  0.027
ANISOU  786  CG  ASP A  48     4623   2962   2307   -121   -694   -571       C
ATOM    787  OD1 ASP A  48      15.593  23.546  27.903  1.00 23.57           O  0.025
ANISOU  787  OD1 ASP A  48     4623   2634   1699    -96   -720   -295       O
ATOM    788  OD2 ASP A  48      14.640  24.849  29.355  1.00 27.50           O  0.027
ANISOU  788  OD2 ASP A  48     4805   3188   2456    178   -295   -820       O
ATOM    789  H   ASP A  48      17.575  25.025  26.839  1.00 31.23           H  0.029
ATOM    790  HA  ASP A  48      19.015  24.589  29.031  1.00 30.63           H  0.029
ATOM    791  HB2 ASP A  48      17.096  24.738  30.227  1.00 30.33           H  0.029
ATOM    792  HB3 ASP A  48      17.027  25.879  29.127  1.00 30.33           H  0.029
ATOM    793  N   GLY A  49      18.293  22.052  27.567  1.00 22.46           N  0.025
ANISOU  793  N   GLY A  49     3222   2869   2443   -755   -937    163       N
ATOM    794  CA  GLY A  49      18.332  20.612  27.583  1.00 20.64           C  0.024
ANISOU  794  CA  GLY A  49     2740   2762   2340   -446   -771    168       C
ATOM    795  C   GLY A  49      16.989  19.934  27.430  1.00 17.86           C  0.022
ANISOU  795  C   GLY A  49     2349   2459   1979   -245   -601     48       C
ATOM    796  O   GLY A  49      16.956  18.718  27.174  1.00 17.94           O  0.022
ANISOU  796  O   GLY A  49     2150   2607   2059    -53   -385     71       O
ATOM    797  H   GLY A  49      18.360  22.386  26.778  1.00 26.96           H  0.027
ATOM    798  HA2 GLY A  49      18.904  20.306  26.862  1.00 24.77           H  0.026
ATOM    799  HA3 GLY A  49      18.722  20.317  28.421  1.00 24.77           H  0.026
ATOM    800  N   SER A  50      15.879  20.675  27.587  1.00 15.91           N  0.021
ANISOU  800  N   SER A  50     2330   2251   1463   -112   -497     -8       N
ATOM    801  CA  SER A  50      14.573  20.121  27.247  1.00 14.35           C  0.020
ANISOU  801  CA  SER A  50     2167   2165   1119     86   -334   -137       C
ATOM    802  C   SER A  50      14.476  19.979  25.723  1.00 13.13           C  0.019
ANISOU  802  C   SER A  50     1943   1905   1139     55   -132     40       C
ATOM    803  O   SER A  50      15.259  20.574  24.965  1.00 14.41           O  0.020
ANISOU  803  O   SER A  50     2045   2077   1354   -169   -158     72       O
ATOM    804  CB  SER A  50      13.422  21.001  27.736  1.00 15.92           C  0.021
ANISOU  804  CB  SER A  50     2504   2189   1356    300   -126   -267       C
ATOM    805  OG  SER A  50      13.400  22.273  27.122  1.00 17.02           O  0.022
ANISOU  805  OG  SER A  50     2798   2145   1523    440   -330   -146       O
ATOM    806  H   SER A  50      15.862  21.482  27.882  1.00 19.09           H  0.023
ATOM    807  HA  SER A  50      14.480  19.242  27.646  1.00 17.22           H  0.022
ATOM    808  HB2 SER A  50      12.585  20.550  27.542  1.00 19.11           H  0.023
ATOM    809  HB3 SER A  50      13.512  21.122  28.694  1.00 19.11           H  0.023
ATOM    810  HG  SER A  50      14.115  22.683  27.284  1.00 20.43           H  0.024
ATOM    811  N   THR A  51      13.449  19.251  25.263  1.00 11.82           N  0.018
ANISOU  811  N   THR A  51     1649   1779   1062     48    -70    -36       N
ATOM    812  CA  THR A  51      13.217  19.054  23.836  1.00 11.21           C  0.018
ANISOU  812  CA  THR A  51     1560   1752    947     98     -6     59       C
ATOM    813  C   THR A  51      11.728  19.256  23.579  1.00 11.55           C  0.018
ANISOU  813  C   THR A  51     1622   1765   1001    228     32     64       C
ATOM    814  O   THR A  51      10.882  18.903  24.415  1.00 11.64           O  0.018
ANISOU  814  O   THR A  51     1592   1823   1008    245     -5    -91       O
ATOM    815  CB  THR A  51      13.647  17.613  23.435  1.00 12.20           C  0.018
ANISOU  815  CB  THR A  51     1500   1990   1146    110     34     55       C
ATOM    816  OG1 THR A  51      15.047  17.487  23.728  1.00 13.53           O  0.019
ANISOU  816  OG1 THR A  51     1594   2334   1212    261      6    127       O
ATOM    817  CG2 THR A  51      13.477  17.366  21.921  1.00 12.56           C  0.019
ANISOU  817  CG2 THR A  51     1532   2000   1239    219     55    -14       C
ATOM    818  H   THR A  51      12.871  18.860  25.766  1.00 14.18           H  0.020
ATOM    819  HA  THR A  51      13.723  19.698  23.316  1.00 13.45           H  0.019
ATOM    820  HB  THR A  51      13.142  16.955  23.935  1.00 14.64           H  0.020
ATOM    821  HG1 THR A  51      15.180  17.600  24.550  1.00 16.23           H  0.021
ATOM    822 HG21 THR A  51      13.811  16.485  21.690  1.00 15.07           H  0.020
ATOM    823 HG22 THR A  51      12.540  17.421  21.678  1.00 15.07           H  0.020
ATOM    824 HG23 THR A  51      13.973  18.032  21.419  1.00 15.07           H  0.020
ATOM    825  N   ASP A  52      11.433  19.796  22.404  1.00 11.90           N  0.018
ANISOU  825  N   ASP A  52     1611   1926    984    127   -112     16       N
ATOM    826  CA  ASP A  52      10.064  19.990  21.934  1.00 11.81           C  0.018
ANISOU  826  CA  ASP A  52     1636   1862    991    239    -40     -2       C
ATOM    827  C   ASP A  52       9.737  18.907  20.911  1.00 11.57           C  0.018
ANISOU  827  C   ASP A  52     1574   1777   1045    227     53     91       C
ATOM    828  O   ASP A  52      10.563  18.603  20.032  1.00 12.25           O  0.018
ANISOU  828  O   ASP A  52     1589   1848   1217    104     66    -99       O
ATOM    829  CB  ASP A  52       9.929  21.334  21.211  1.00 13.85           C  0.019
ANISOU  829  CB  ASP A  52     2037   2021   1203    183   -249    -12       C
ATOM    830  CG  ASP A  52      10.192  22.521  22.090  1.00 18.01           C  0.022
ANISOU  830  CG  ASP A  52     2974   2221   1650    254   -238    -24       C
ATOM    831  OD1 ASP A  52       9.946  22.442  23.300  1.00 18.66           O  0.023
ANISOU  831  OD1 ASP A  52     3061   2293   1735    413   -199   -282       O
ATOM    832  OD2 ASP A  52      10.634  23.569  21.526  1.00 22.85           O  0.025
ANISOU  832  OD2 ASP A  52     3850   2430   2402      9    -96   -198       O
ATOM    833  H   ASP A  52      12.026  20.067  21.843  1.00 14.28           H  0.020
```

```
ATOM    834  HA  ASP A  52       9.435  19.948  22.671  1.00 14.18           H  0.020
ATOM    835  HB2 ASP A  52      10.564  21.361  20.478  1.00 16.62           H  0.021
ATOM    836  HB3 ASP A  52       9.026  21.413  20.867  1.00 16.62           H  0.021
ATOM    837  N   TYR A  53       8.521  18.352  20.988  1.00 11.67           N  0.018
ANISOU  837  N   TYR A  53     1484   1734   1216    256    -30     25       N
ATOM    838  CA  TYR A  53       8.162  17.186  20.190  1.00 11.75           C  0.018
ANISOU  838  CA  TYR A  53     1465   1664   1336    220     18   -111       C
ATOM    839  C   TYR A  53       6.851  17.393  19.441  1.00 11.73           C  0.018
ANISOU  839  C   TYR A  53     1463   1625   1367    256    -23    -55       C
ATOM    840  O   TYR A  53       5.846  17.799  20.027  1.00 12.19           O  0.018
ANISOU  840  O   TYR A  53     1537   1754   1342    193     12     28       O
ATOM    841  CB  TYR A  53       7.975  15.971  21.107  1.00 12.52           C  0.019
ANISOU  841  CB  TYR A  53     1588   1709   1461    203     65     -5       C
ATOM    842  CG  TYR A  53       9.224  15.531  21.826  1.00 12.53           C  0.019
ANISOU  842  CG  TYR A  53     1711   1796   1255    157   -115     82       C
ATOM    843  CD1 TYR A  53       9.645  16.137  23.006  1.00 12.61           C  0.019
ANISOU  843  CD1 TYR A  53     1716   1761   1313    204     78    185       C
ATOM    844  CD2 TYR A  53      10.021  14.507  21.299  1.00 12.81           C  0.019
ANISOU  844  CD2 TYR A  53     1769   1849   1249    271   -112    -86       C
ATOM    845  CE1 TYR A  53      10.806  15.704  23.659  1.00 11.97           C  0.018
ANISOU  845  CE1 TYR A  53     1656   1619   1272    210     40    159       C
ATOM    846  CE2 TYR A  53      11.168  14.093  21.959  1.00 13.31           C  0.019
ANISOU  846  CE2 TYR A  53     1791   1898   1367    351    -12   -111       C
ATOM    847  CZ  TYR A  53      11.574  14.683  23.131  1.00 11.92           C  0.018
ANISOU  847  CZ  TYR A  53     1656   1685   1188    289     27    126       C
ATOM    848  OH  TYR A  53      12.731  14.235  23.711  1.00 13.14           O  0.019
ANISOU  848  OH  TYR A  53     1851   1668   1473    381    -79    160       O
ATOM    849  H   TYR A  53       7.889  18.636  21.497  1.00 14.00           H  0.020
ATOM    850  HA  TYR A  53       8.862  16.990  19.548  1.00 14.10           H  0.020
ATOM    851  HB2 TYR A  53       7.310  16.189  21.779  1.00 15.03           H  0.020
ATOM    852  HB3 TYR A  53       7.665  15.224  20.572  1.00 15.03           H  0.020
ATOM    853  HD1 TYR A  53       9.136  16.820  23.379  1.00 15.13           H  0.020
ATOM    854  HD2 TYR A  53       9.766  14.085  20.511  1.00 15.37           H  0.021
ATOM    855  HE1 TYR A  53      11.071  16.118  24.449  1.00 14.37           H  0.020
ATOM    856  HE2 TYR A  53      11.681  13.409  21.593  1.00 15.97           H  0.021
ATOM    857  HH  TYR A  53      12.887  14.669  24.413  1.00 15.77           H  0.021
ATOM    858  N   GLY A  54       6.855  16.954  18.192  1.00 12.48           N  0.018
ANISOU  858  N   GLY A  54     1657   1705   1379    132    -56   -242       N
ATOM    859  CA  GLY A  54       5.590  16.831  17.443  1.00 13.05           C  0.019
ANISOU  859  CA  GLY A  54     1757   1791   1411    170   -286   -250       C
ATOM    860  C   GLY A  54       5.052  18.025  16.694  1.00 12.68           C  0.019
ANISOU  860  C   GLY A  54     1630   1710   1478    182     33    -75       C
ATOM    861  O   GLY A  54       5.694  19.031  16.613  1.00 12.71           O  0.019
ANISOU  861  O   GLY A  54     1615   1784   1431    170    -17   -122       O
ATOM    862  H   GLY A  54       7.557  16.722  17.753  1.00 14.97           H  0.020
ATOM    863  HA2 GLY A  54       5.695  16.117  16.797  1.00 15.67           H  0.021
ATOM    864  HA3 GLY A  54       4.900  16.554  18.066  1.00 15.67           H  0.021
ATOM    865  N  AILE A  55       3.812  17.860  16.229  0.47 13.68           N  0.019
ANISOU  865  N  AILE A  55     1605   1951   1641    116    -51   -176       N
ATOM    866  CA AILE A  55       3.183  18.902  15.366  0.47 15.43           C  0.021
ANISOU  866  CA AILE A  55     1751   2188   1924    235   -272   -112       C
ATOM    867  C  AILE A  55       3.033  20.225  16.123  0.47 14.05           C  0.020
ANISOU  867  C  AILE A  55     1661   2071   1606    297   -146   -110       C
ATOM    868  O  AILE A  55       2.955  21.254  15.456  0.47 14.36           O  0.020
ANISOU  868  O  AILE A  55     1751   2143   1560    224    -98    124       O
ATOM    869  CB AILE A  55       1.820  18.413  14.824  0.47 18.99           C  0.023
ANISOU  869  CB AILE A  55     1951   2520   2745     64   -464   -248       C
ATOM    870  CG1AILE A  55       0.795  18.109  15.922  0.47 19.32           C  0.023
ANISOU  870  CG1AILE A  55     1800   2497   3044    -24   -592   -391       C
ATOM    871  CG2AILE A  55       2.008  17.230  13.894  0.47 20.36           C  0.024
ANISOU  871  CG2AILE A  55     2045   2647   3044    124   -354   -284       C
ATOM    872  CD1AILE A  55      -0.614  17.945  15.405  0.47 19.71           C  0.023
ANISOU  872  CD1AILE A  55     1810   2460   3220    -10   -556   -146       C
ATOM    873  H  AILE A  55       3.314  17.177  16.386  0.47 16.41           H  0.021
ATOM    874  HA AILE A  55       3.761  19.062  14.605  0.47 18.52           H  0.023
ATOM    875  HB AILE A  55       1.454  19.136  14.291  0.47 22.79           H  0.025
ATOM    876 HG12AILE A  55       1.046  17.283  16.365  0.47 23.19           H  0.025
ATOM    877 HG13AILE A  55       0.789  18.837  16.563  0.47 23.19           H  0.025
ATOM    878 HG21AILE A  55       1.204  17.113  13.364  0.47 24.43           H  0.026
ATOM    879 HG22AILE A  55       2.765  17.402  13.313  0.47 24.43           H  0.026
ATOM    880 HG23AILE A  55       2.171  16.439  14.429  0.47 24.43           H  0.026
ATOM    881 HD11AILE A  55      -1.210  17.796  16.156  0.47 23.66           H  0.025
ATOM    882 HD12AILE A  55      -0.872  18.752  14.932  0.47 23.66           H  0.025
ATOM    883 HD13AILE A  55      -0.643  17.185  14.803  0.47 23.66           H  0.025
ATOM    884  N  BILE A  55       3.818  17.859  16.230  0.53 13.34           N  0.019
ANISOU  884  N  BILE A  55     1610   1955   1503    155   -154    -77       N
ATOM    885  CA BILE A  55       3.187  18.893  15.362  0.53 14.86           C  0.020
ANISOU  885  CA BILE A  55     1803   2200   1644    323   -413     52       C
```

```
ATOM    886  C  BILE A  55       2.998  20.216  16.117  0.53 13.46           C  0.019
ANISOU  886  C  BILE A  55     1660   2056   1400    350   -205    -20       C
ATOM    887  O  BILE A  55       2.967  21.248  15.454  0.53 13.40           O  0.019
ANISOU  887  O  BILE A  55     1724   2102   1265    270   -121    280       O
ATOM    888  CB BILE A  55       1.857  18.318  14.831  0.53 18.99           C  0.023
ANISOU  888  CB BILE A  55     2158   2600   2457    152   -673    -62       C
ATOM    889  CG1BILE A  55       1.285  19.155  13.686  0.53 21.33           C  0.024
ANISOU  889  CG1BILE A  55     2458   2714   2933    139   -810     77       C
ATOM    890  CG2BILE A  55       0.851  18.136  15.955  0.53 19.33           C  0.023
ANISOU  890  CG2BILE A  55     1993   2657   2694    115   -689   -307       C
ATOM    891  CD1BILE A  55       0.976  18.355  12.447  0.53 21.06           C  0.024
ANISOU  891  CD1BILE A  55     2482   2597   2922    266   -567      5       C
ATOM    892  H  BILE A  55       3.322  17.175  16.390  0.53 16.01           H  0.021
ATOM    893  HA BILE A  55       3.766  19.061  14.602  0.53 17.84           H  0.022
ATOM    894  HB BILE A  55       2.049  17.437  14.473  0.53 22.79           H  0.025
ATOM    895 HG12BILE A  55       0.461  19.570  13.984  0.53 25.60           H  0.026
ATOM    896 HG13BILE A  55       1.929  19.839  13.445  0.53 25.60           H  0.026
ATOM    897 HG21BILE A  55       0.087  17.643  15.616  0.53 23.19           H  0.025
ATOM    898 HG22BILE A  55       1.268  17.642  16.678  0.53 23.19           H  0.025
ATOM    899 HG23BILE A  55       0.567  19.007  16.271  0.53 23.19           H  0.025
ATOM    900 HD11BILE A  55       0.683  18.959  11.747  0.53 25.27           H  0.026
ATOM    901 HD12BILE A  55       1.778  17.887  12.166  0.53 25.27           H  0.026
ATOM    902 HD13BILE A  55       0.275  17.717  12.650  0.53 25.27           H  0.026
ATOM    903  N   LEU A  56       2.866  20.158  17.448  1.00 13.30           N  0.019
ANISOU  903  N   LEU A  56     1573   1956   1525    282   -206    -71       N
ATOM    904  CA  LEU A  56       2.707  21.393  18.267  1.00 13.31           C  0.019
ANISOU  904  CA  LEU A  56     1660   1953   1446    448   -132    -65       C
ATOM    905  C   LEU A  56       3.926  21.619  19.174  1.00 13.08           C  0.019
ANISOU  905  C   LEU A  56     1732   1847   1390    428   -102   -133       C
ATOM    906  O   LEU A  56       3.847  22.479  20.029  1.00 13.55           O  0.019
ANISOU  906  O   LEU A  56     1757   1799   1591    445    -85   -328       O
ATOM    907  CB  LEU A  56       1.383  21.312  19.027  1.00 14.00           C  0.020
ANISOU  907  CB  LEU A  56     1610   2153   1556    436    -21     71       C
ATOM    908  CG  LEU A  56       0.155  21.535  18.142  1.00 15.14           C  0.020
ANISOU  908  CG  LEU A  56     1757   2241   1755    464    -69    237       C
ATOM    909  CD1 LEU A  56      -1.125  21.244  18.905  1.00 16.89           C  0.021
ANISOU  909  CD1 LEU A  56     1752   2451   2216    423    264    258       C
ATOM    910  CD2 LEU A  56       0.135  22.947  17.576  1.00 16.20           C  0.021
ANISOU  910  CD2 LEU A  56     1895   2482   1779    478    -44    205       C
ATOM    911  H   LEU A  56       2.852  19.428  17.902  1.00 15.97           H  0.021
ATOM    912  HA  LEU A  56       2.658  22.155  17.671  1.00 15.98           H  0.021
ATOM    913  HB2 LEU A  56       1.305  20.432  19.427  1.00 16.80           H  0.021
ATOM    914  HB3 LEU A  56       1.377  21.991  19.720  1.00 16.80           H  0.021
ATOM    915  HG  LEU A  56       0.197  20.921  17.393  1.00 18.17           H  0.022
ATOM    916 HD11 LEU A  56      -1.882  21.381  18.315  1.00 20.27           H  0.024
ATOM    917 HD12 LEU A  56      -1.106  20.324  19.213  1.00 20.27           H  0.024
ATOM    918 HD13 LEU A  56      -1.184  21.846  19.664  1.00 20.27           H  0.024
ATOM    919 HD21 LEU A  56      -0.756  23.141  17.245  1.00 19.44           H  0.023
ATOM    920 HD22 LEU A  56       0.370  23.572  18.280  1.00 19.44           H  0.023
ATOM    921 HD23 LEU A  56       0.777  23.008  16.852  1.00 19.44           H  0.023
ATOM    922  N   GLN A  57       5.030  20.889  18.976  1.00 12.28           N  0.018
ANISOU  922  N   GLN A  57     1567   1742   1356    357   -157    -60       N
ATOM    923  CA  GLN A  57       6.292  21.200  19.647  1.00 11.47           C  0.018
ANISOU  923  CA  GLN A  57     1470   1777   1110    267    -89    -27       C
ATOM    924  C   GLN A  57       6.102  21.337  21.145  1.00 12.27           C  0.018
ANISOU  924  C   GLN A  57     1677   1773   1213    405    -89   -149       C
ATOM    925  O   GLN A  57       6.484  22.320  21.769  1.00 13.67           O  0.019
ANISOU  925  O   GLN A  57     1995   1865   1335    297   -105   -280       O
ATOM    926  CB  GLN A  57       6.954  22.423  19.007  1.00 12.25           C  0.018
ANISOU  926  CB  GLN A  57     1574   1822   1258    168   -100     24       C
ATOM    927  CG  GLN A  57       7.486  22.136  17.589  1.00 12.41           C  0.018
ANISOU  927  CG  GLN A  57     1666   1729   1321    118    -69    -74       C
ATOM    928  CD  GLN A  57       8.652  21.194  17.588  1.00 13.01           C  0.019
ANISOU  928  CD  GLN A  57     1731   1788   1426    190   -189    -39       C
ATOM    929  OE1 GLN A  57       9.785  21.631  17.788  1.00 14.30           O  0.020
ANISOU  929  OE1 GLN A  57     1713   1936   1783    145   -177    -84       O
ATOM    930  NE2 GLN A  57       8.420  19.903  17.369  1.00 12.42           N  0.018
ANISOU  930  NE2 GLN A  57     1684   1729   1308    244     36   -154       N
ATOM    931  H   GLN A  57       5.072  20.206  18.455  1.00 14.74           H  0.020
ATOM    932  HA  GLN A  57       6.892  20.450  19.511  1.00 13.76           H  0.019
ATOM    933  HB2 GLN A  57       6.302  23.138  18.943  1.00 14.70           H  0.020
ATOM    934  HB3 GLN A  57       7.701  22.704  19.557  1.00 14.70           H  0.020
ATOM    935  HG2 GLN A  57       6.777  21.737  17.060  1.00 14.90           H  0.020
ATOM    936  HG3 GLN A  57       7.774  22.970  17.185  1.00 14.90           H  0.020
ATOM    937 HE21 GLN A  57       7.617  19.628  17.232  1.00 14.91           H  0.020
ATOM    938 HE22 GLN A  57       9.073  19.344  17.364  1.00 14.91           H  0.020
ATOM    939  N   ILE A  58       5.575  20.274  21.723  1.00 12.24           N  0.018
ANISOU  939  N   ILE A  58     1608   1913   1129    342    -94      8       N
```

```
ATOM    940  CA  ILE A  58       5.287  20.209  23.164  1.00 13.23           C  0.019
ANISOU  940  CA  ILE A  58      1651   2143   1234    243     56     -2       C
ATOM    941  C   ILE A  58       6.558  19.821  23.918  1.00 13.15           C  0.019
ANISOU  941  C   ILE A  58      1611   2212   1174    254     26   -126       C
ATOM    942  O   ILE A  58       7.281  18.891  23.527  1.00 13.59           O  0.019
ANISOU  942  O   ILE A  58      1704   2198   1261    367     73    -98       O
ATOM    943  CB  ILE A  58       4.137  19.223  23.398  1.00 14.76           C  0.020
ANISOU  943  CB  ILE A  58      1703   2422   1485    106     70      6       C
ATOM    944  CG1 ILE A  58       2.848  19.891  22.885  1.00 15.29           C  0.020
ANISOU  944  CG1 ILE A  58      1834   2503   1474    109     40     56       C
ATOM    945  CG2 ILE A  58       3.999  18.864  24.856  1.00 14.85           C  0.020
ANISOU  945  CG2 ILE A  58      1769   2462   1412    102    256     51       C
ATOM    946  CD1 ILE A  58       1.631  19.024  22.898  1.00 16.82           C  0.021
ANISOU  946  CD1 ILE A  58      1847   2676   1866     57    140    130       C
ATOM    947  H   ILE A  58       5.367  19.555  21.300  1.00 14.69           H  0.020
ATOM    948  HA  ILE A  58       5.005  21.084  23.474  1.00 15.88           H  0.021
ATOM    949  HB  ILE A  58       4.301  18.418  22.885  1.00 17.72           H  0.022
ATOM    950 HG12 ILE A  58       2.661  20.664  23.440  1.00 18.35           H  0.022
ATOM    951 HG13 ILE A  58       2.991  20.176  21.969  1.00 18.35           H  0.022
ATOM    952 HG21 ILE A  58       3.173  18.375  24.988  1.00 17.82           H  0.022
ATOM    953 HG22 ILE A  58       4.750  18.310  25.121  1.00 17.82           H  0.022
ATOM    954 HG23 ILE A  58       3.986  19.679  25.381  1.00 17.82           H  0.022
ATOM    955 HD11 ILE A  58       0.931  19.456  22.384  1.00 20.18           H  0.023
ATOM    956 HD12 ILE A  58       1.851  18.166  22.502  1.00 20.18           H  0.023
ATOM    957 HD13 ILE A  58       1.339  18.900  23.815  1.00 20.18           H  0.023
ATOM    958  N   ASN A  59       6.852  20.542  24.994  1.00 13.89           N  0.019
ANISOU  958  N   ASN A  59      1650   2524   1102    342     24    -61       N
ATOM    959  CA  ASN A  59       8.152  20.498  25.651  1.00 15.38           C  0.021
ANISOU  959  CA  ASN A  59      1802   2712   1329    167     56   -134       C
ATOM    960  C   ASN A  59       8.230  19.528  26.816  1.00 15.27           C  0.020
ANISOU  960  C   ASN A  59      1647   2861   1294    172    216     39       C
ATOM    961  O   ASN A  59       7.309  19.431  27.625  1.00 17.67           O  0.022
ANISOU  961  O   ASN A  59      1799   3440   1474    430    144    362       O
ATOM    962  CB  ASN A  59       8.434  21.914  26.170  1.00 17.89           C  0.022
ANISOU  962  CB  ASN A  59      2204   2811   1783   -107     -9   -462       C
ATOM    963  CG  ASN A  59       9.811  22.093  26.700  1.00 20.34           C  0.024
ANISOU  963  CG  ASN A  59      2648   2905   2175    -50   -115   -686       C
ATOM    964  OD1 ASN A  59       9.994  22.146  27.924  1.00 22.84           O  0.025
ANISOU  964  OD1 ASN A  59      2971   2997   2708    102   -254   -508       O
ATOM    965  ND2 ASN A  59      10.770  22.249  25.831  1.00 23.69           N  0.025
ANISOU  965  ND2 ASN A  59      2827   3065   3107    110    -32   -509       N
ATOM    966  H   ASN A  59       6.298  21.080  25.372  1.00 16.67           H  0.021
ATOM    967  HA  ASN A  59       8.836  20.262  25.005  1.00 18.46           H  0.022
ATOM    968  HB2 ASN A  59       8.312  22.544  25.443  1.00 21.47           H  0.024
ATOM    969  HB3 ASN A  59       7.812  22.114  26.887  1.00 21.47           H  0.024
ATOM    970 HD21 ASN A  59      11.579  22.361  26.099  1.00 28.42           H  0.028
ATOM    971 HD22 ASN A  59      10.597  22.216  24.989  1.00 28.42           H  0.028
ATOM    972  N   SER A  60       9.386  18.877  26.940  1.00 14.21           N  0.020
ANISOU  972  N   SER A  60      1708   2626   1063    196    103    141       N
ATOM    973  CA  SER A  60       9.659  17.913  27.976  1.00 14.53           C  0.020
ANISOU  973  CA  SER A  60      1876   2542   1101     63    114    100       C
ATOM    974  C   SER A  60       9.957  18.517  29.350  1.00 15.84           C  0.021
ANISOU  974  C   SER A  60      2027   2941   1050     63     62     56       C
ATOM    975  O   SER A  60      10.005  17.771  30.309  1.00 18.48           O  0.022
ANISOU  975  O   SER A  60      2409   3001   1610   -134   -145     66       O
ATOM    976  CB  SER A  60      10.833  17.062  27.511  1.00 14.56           C  0.020
ANISOU  976  CB  SER A  60      1887   2308   1338    145     73    315       C
ATOM    977  OG  SER A  60      12.040  17.826  27.455  1.00 13.65           O  0.019
ANISOU  977  OG  SER A  60      1855   2142   1191    194     79    224       O
ATOM    978  H   SER A  60      10.050  18.991  26.405  1.00 17.05           H  0.022
ATOM    979  HA  SER A  60       8.889  17.331  28.070  1.00 17.43           H  0.022
ATOM    980  HB2 SER A  60      10.955  16.328  28.133  1.00 17.48           H  0.022
ATOM    981  HB3 SER A  60      10.639  16.716  26.626  1.00 17.48           H  0.022
ATOM    982  HG  SER A  60      12.251  18.089  28.224  1.00 16.39           H  0.021
ATOM    983  N   ARG A  61      10.108  19.834  29.490  1.00 17.43           N  0.022
ANISOU  983  N   ARG A  61      2122   3267   1233    351    101   -318       N
ATOM    984  CA  ARG A  61      10.311  20.396  30.833  1.00 19.93           C  0.023
ANISOU  984  CA  ARG A  61      2420   3569   1584    564     17   -648       C
ATOM    985  C   ARG A  61       9.053  20.253  31.683  1.00 18.95           C  0.023
ANISOU  985  C   ARG A  61      2348   3460   1393    785   -176   -462       C
ATOM    986  O   ARG A  61       9.158  20.034  32.889  1.00 20.78           O  0.024
ANISOU  986  O   ARG A  61      2505   3751   1639    930      6   -492       O
ATOM    987  CB  ARG A  61      10.709  21.870  30.739  1.00 24.17           C  0.026
ANISOU  987  CB  ARG A  61      2926   4105   2153    364     14   -959       C
ATOM    988  CG  ARG A  61      10.786  22.650  32.035  1.00 29.28           C  0.028
ANISOU  988  CG  ARG A  61      3605   4682   2839    244    168  -1164       C
ATOM    989  CD  ARG A  61      11.425  21.840  33.147  1.00 33.11           C  0.030
ANISOU  989  CD  ARG A  61      4162   5108   3309    174    281  -1246       C
```

```
ATOM    990  NE  ARG A  61      11.478  22.570  34.420  1.00 35.45           N  0.031
ANISOU  990  NE  ARG A  61     4604   5425   3442    115    358  -1463       N
ATOM    991  CZ  ARG A  61      10.638  22.402  35.434  1.00 35.61           C  0.031
ANISOU  991  CZ  ARG A  61     4867   5559   3103     97    418  -1738       C
ATOM    992  NH1 ARG A  61       9.658  21.516  35.383  1.00 35.10           N  0.031
ANISOU  992  NH1 ARG A  61     4893   5501   2941    114    409  -1897       N
ATOM    993  NH2 ARG A  61      10.803  23.121  36.540  1.00 35.92           N  0.031
ANISOU  993  NH2 ARG A  61     4999   5653   2995     42    369  -1739       N
ATOM    994  H   ARG A  61      10.098  20.409  28.850  1.00 20.92           H  0.024
ATOM    995  HA  ARG A  61      11.030  19.914  31.271  1.00 23.92           H  0.026
ATOM    996  HB2 ARG A  61      11.579  21.926  30.314  1.00 29.01           H  0.028
ATOM    997  HB3 ARG A  61      10.051  22.322  30.191  1.00 29.01           H  0.028
ATOM    998  HG2 ARG A  61      11.321  23.447  31.896  1.00 35.14           H  0.031
ATOM    999  HG3 ARG A  61       9.890  22.893  32.315  1.00 35.14           H  0.031
ATOM   1000  HD2 ARG A  61      10.973  21.008  33.297  1.00 39.73           H  0.033
ATOM   1001  HD3 ARG A  61      12.342  21.656  32.890  1.00 39.73           H  0.033
ATOM   1002  HE  ARG A  61      12.103  23.153  34.516  1.00 42.55           H  0.034
ATOM   1003 HH11 ARG A  61       9.538  21.040  34.678  1.00 42.12           H  0.034
ATOM   1004 HH12 ARG A  61       9.130  21.424  36.055  1.00 42.12           H  0.034
ATOM   1005 HH21 ARG A  61      11.439  23.698  36.587  1.00 43.10           H  0.034
ATOM   1006 HH22 ARG A  61      10.267  23.017  37.204  1.00 43.10           H  0.034
ATOM   1007  N   TRP A  62       7.891  20.295  31.065  1.00 17.37           N  0.022
ANISOU 1007  N   TRP A  62     2173   3011   1415    727   -156   -334       N
ATOM   1008  CA  TRP A  62       6.629  20.277  31.824  1.00 17.62           C  0.022
ANISOU 1008  CA  TRP A  62     2184   2786   1726    808     33   -211       C
ATOM   1009  C   TRP A  62       5.656  19.196  31.412  1.00 15.41           C  0.021
ANISOU 1009  C   TRP A  62     2158   2547   1152    718    300    -70       C
ATOM   1010  O   TRP A  62       5.007  18.707  32.285  1.00 16.45           O  0.021
ANISOU 1010  O   TRP A  62     2343   2616   1291    614    262    219       O
ATOM   1011  CB  TRP A  62       5.941  21.625  31.585  1.00 20.65           C  0.024
ANISOU 1011  CB  TRP A  62     2558   2931   2358    696   -100   -538       C
ATOM   1012  CG  TRP A  62       6.635  22.779  32.230  1.00 24.07           C  0.026
ANISOU 1012  CG  TRP A  62     3005   3230   2911    421   -210   -684       C
ATOM   1013  CD1 TRP A  62       7.280  23.807  31.609  1.00 26.87           C  0.027
ANISOU 1013  CD1 TRP A  62     3347   3375   3487    194   -264   -808       C
ATOM   1014  CD2 TRP A  62       6.758  23.018  33.639  1.00 26.79           C  0.027
ANISOU 1014  CD2 TRP A  62     3289   3447   3441    275   -390   -767       C
ATOM   1015  NE1 TRP A  62       7.791  24.673  32.536  1.00 28.34           N  0.028
ANISOU 1015  NE1 TRP A  62     3565   3464   3740     10   -429   -800       N
ATOM   1016  CE2 TRP A  62       7.487  24.214  33.788  1.00 28.62           C  0.028
ANISOU 1016  CE2 TRP A  62     3593   3568   3713    108   -374   -761       C
ATOM   1017  CE3 TRP A  62       6.319  22.341  34.780  1.00 26.57           C  0.027
ANISOU 1017  CE3 TRP A  62     3354   3539   3202    326   -477   -889       C
ATOM   1018  CZ2 TRP A  62       7.789  24.741  35.041  1.00 29.62           C  0.028
ANISOU 1018  CZ2 TRP A  62     3687   3658   3910    124   -305   -709       C
ATOM   1019  CZ3 TRP A  62       6.618  22.865  36.017  1.00 28.97           C  0.028
ANISOU 1019  CZ3 TRP A  62     3559   3679   3769    337   -365   -778       C
ATOM   1020  CH2 TRP A  62       7.341  24.049  36.141  1.00 29.64           C  0.028
ANISOU 1020  CH2 TRP A  62     3651   3744   3866    239   -303   -710       C
ATOM   1021  H   TRP A  62       7.791  20.334  30.212  1.00 20.84           H  0.024
ATOM   1022  HA  TRP A  62       6.810  20.185  32.772  1.00 21.15           H  0.024
ATOM   1023  HB2 TRP A  62       5.909  21.794  30.630  1.00 24.79           H  0.026
ATOM   1024  HB3 TRP A  62       5.040  21.584  31.942  1.00 24.79           H  0.026
ATOM   1025  HD1 TRP A  62       7.358  23.907  30.688  1.00 32.25           H  0.030
ATOM   1026  HE1 TRP A  62       8.235  25.388  32.360  1.00 34.01           H  0.031
ATOM   1027  HE3 TRP A  62       5.835  21.550  34.705  1.00 31.88           H  0.030
ATOM   1028  HZ2 TRP A  62       8.271  25.532  35.130  1.00 35.55           H  0.031
ATOM   1029  HZ3 TRP A  62       6.331  22.422  36.783  1.00 34.76           H  0.031
ATOM   1030  HH2 TRP A  62       7.528  24.379  36.990  1.00 35.57           H  0.031
ATOM   1031  N   TRP A  63       5.584  18.814  30.132  1.00 14.74           N  0.020
ANISOU 1031  N   TRP A  63     2031   2193   1378    546    465    204       N
ATOM   1032  CA  TRP A  63       4.413  18.063  29.601  1.00 14.22           C  0.020
ANISOU 1032  CA  TRP A  63     1850   2069   1483    394    270    164       C
ATOM   1033  C   TRP A  63       4.602  16.582  29.314  1.00 14.15           C  0.020
ANISOU 1033  C   TRP A  63     1759   2052   1566    353    230     49       C
ATOM   1034  O   TRP A  63       3.637  15.887  29.304  1.00 15.72           O  0.021
ANISOU 1034  O   TRP A  63     1690   2201   2081    336    436     -6       O
ATOM   1035  CB  TRP A  63       3.959  18.781  28.322  1.00 13.56           C  0.019
ANISOU 1035  CB  TRP A  63     1777   1971   1405    369    308     26       C
ATOM   1036  CG  TRP A  63       3.686  20.219  28.609  1.00 14.44           C  0.020
ANISOU 1036  CG  TRP A  63     1911   1956   1619    313     37     51       C
ATOM   1037  CD1 TRP A  63       4.420  21.281  28.187  1.00 16.27           C  0.021
ANISOU 1037  CD1 TRP A  63     2092   1977   2114    468     36     26       C
ATOM   1038  CD2 TRP A  63       2.654  20.749  29.462  1.00 14.77           C  0.020
ANISOU 1038  CD2 TRP A  63     2008   1965   1638    442    -91     65       C
ATOM   1039  NE1 TRP A  63       3.890  22.435  28.684  1.00 16.48           N  0.021
ANISOU 1039  NE1 TRP A  63     2197   2089   1974    347   -163    133       N
ATOM   1040  CE2 TRP A  63       2.812  22.148  29.472  1.00 15.99           C  0.021
```

```
ANISOU 1040  CE2 TRP A  63     2077   2135   1864    509    -39    -32       C
ATOM   1041  CE3 TRP A  63       1.602  20.189  30.189  1.00 15.46           C  0.021
ANISOU 1041  CE3 TRP A  63     2078   2076   1720    568    134     -9       C
ATOM   1042  CZ2 TRP A  63       1.958  22.994  30.177  1.00 16.71           C  0.021
ANISOU 1042  CZ2 TRP A  63     2186   2167   1997    546   -174    -88       C
ATOM   1043  CZ3 TRP A  63       0.760  21.023  30.891  1.00 17.20           C  0.022
ANISOU 1043  CZ3 TRP A  63     2268   2278   1989    586    223    -77       C
ATOM   1044  CH2 TRP A  63       0.937  22.406  30.884  1.00 17.69           C  0.022
ANISOU 1044  CH2 TRP A  63     2353   2405   1962    630    178   -234       C
ATOM   1045  H   TRP A  63       6.192  18.972  29.545  1.00 17.69           H  0.022
ATOM   1046  HA  TRP A  63       3.691  18.137  30.244  1.00 17.07           H  0.022
ATOM   1047  HB2 TRP A  63       4.660  18.728  27.654  1.00 16.28           H  0.021
ATOM   1048  HB3 TRP A  63       3.143  18.372  27.993  1.00 16.28           H  0.021
ATOM   1049  HD1 TRP A  63       5.165  21.230  27.633  1.00 19.53           H  0.023
ATOM   1050  HE1 TRP A  63       4.195  23.225  28.532  1.00 19.77           H  0.023
ATOM   1051  HE3 TRP A  63       1.472  19.268  30.200  1.00 18.55           H  0.023
ATOM   1052  HZ2 TRP A  63       2.076  23.916  30.172  1.00 20.06           H  0.023
ATOM   1053  HZ3 TRP A  63       0.059  20.656  31.380  1.00 20.64           H  0.024
ATOM   1054  HH2 TRP A  63       0.351  22.942  31.368  1.00 21.22           H  0.024
ATOM   1055  N   CYS A  64       5.802  16.141  28.976  1.00 14.57           N  0.020
ANISOU 1055  N   CYS A  64     1754   2021   1760    419    476     72       N
ATOM   1056  CA  CYS A  64       5.981  14.749  28.593  1.00 14.40           C  0.020
ANISOU 1056  CA  CYS A  64     1848   1852   1772    398    459      2       C
ATOM   1057  C   CYS A  64       7.298  14.254  29.162  1.00 14.72           C  0.020
ANISOU 1057  C   CYS A  64     1895   1920   1776    450    491    142       C
ATOM   1058  O   CYS A  64       8.165  15.059  29.512  1.00 15.38           O  0.021
ANISOU 1058  O   CYS A  64     2041   1976   1827    448    426    322       O
ATOM   1059  CB  CYS A  64       5.931  14.573  27.074  1.00 14.39           C  0.020
ANISOU 1059  CB  CYS A  64     1874   1749   1844    359    506    -38       C
ATOM   1060  SG  CYS A  64       7.188  15.413  26.121  1.00 14.53           S  0.020
ANISOU 1060  SG  CYS A  64     1931   1754   1837    290    322    173       S
ATOM   1061  H   CYS A  64       6.519  16.616  28.959  1.00 17.48           H  0.022
ATOM   1062  HA  CYS A  64       5.270  14.215  28.978  1.00 17.28           H  0.022
ATOM   1063  HB2 CYS A  64       6.008  13.626  26.877  1.00 17.27           H  0.022
ATOM   1064  HB3 CYS A  64       5.071  14.896  26.761  1.00 17.27           H  0.022
ATOM   1065  N   ASN A  65       7.442  12.935  29.261  1.00 14.73           N  0.020
ANISOU 1065  N   ASN A  65     1871   1935   1790    420    398    296       N
ATOM   1066  CA  ASN A  65       8.689  12.354  29.731  1.00 14.25           C  0.020
ANISOU 1066  CA  ASN A  65     1897   1955   1560    340    407    478       C
ATOM   1067  C   ASN A  65       9.546  11.846  28.583  1.00 14.17           C  0.020
ANISOU 1067  C   ASN A  65     1840   1907   1638    373    359    319       C
ATOM   1068  O   ASN A  65       9.101  10.994  27.798  1.00 14.59           O  0.020
ANISOU 1068  O   ASN A  65     1804   1872   1865    294    292    226       O
ATOM   1069  CB  ASN A  65       8.431  11.173  30.649  1.00 16.61           C  0.021
ANISOU 1069  CB  ASN A  65     2255   2121   1934    456    487    451       C
ATOM   1070  CG  ASN A  65       9.724  10.543  31.079  1.00 18.32           C  0.022
ANISOU 1070  CG  ASN A  65     2565   2314   2082    547    607    574       C
ATOM   1071  OD1 ASN A  65      10.583  11.237  31.629  1.00 19.41           O  0.023
ANISOU 1071  OD1 ASN A  65     2689   2516   2169    609    498    836       O
ATOM   1072  ND2 ASN A  65       9.933   9.265  30.742  1.00 20.43           N  0.024
ANISOU 1072  ND2 ASN A  65     2769   2456   2538    646    679    470       N
ATOM   1073  H   ASN A  65       6.834  12.360  29.063  1.00 17.67           H  0.022
ATOM   1074  HA  ASN A  65       9.196  13.019  30.223  1.00 17.10           H  0.022
ATOM   1075  HB2 ASN A  65       7.960  11.477  31.441  1.00 19.93           H  0.023
ATOM   1076  HB3 ASN A  65       7.907  10.507  30.178  1.00 19.93           H  0.023
ATOM   1077 HD21 ASN A  65      10.668   8.877  30.964  1.00 24.52           H  0.026
ATOM   1078 HD22 ASN A  65       9.316   8.820  30.341  1.00 24.52           H  0.026
ATOM   1079  N   ASP A  66      10.794  12.309  28.534  1.00 13.71           N  0.019
ANISOU 1079  N   ASP A  66     1803   1820   1586    399    335    224       N
ATOM   1080  CA  ASP A  66      11.786  11.749  27.641  1.00 13.86           C  0.019
ANISOU 1080  CA  ASP A  66     1835   1846   1583    395    424    249       C
ATOM   1081  C   ASP A  66      12.984  11.151  28.354  1.00 14.88           C  0.020
ANISOU 1081  C   ASP A  66     1996   1940   1720    489    397    307       C
ATOM   1082  O   ASP A  66      13.917  10.698  27.674  1.00 15.55           O  0.021
ANISOU 1082  O   ASP A  66     2036   1994   1877    580    424     51       O
ATOM   1083  CB  ASP A  66      12.249  12.744  26.565  1.00 13.17           C  0.019
ANISOU 1083  CB  ASP A  66     1711   1860   1433    386    316    132       C
ATOM   1084  CG  ASP A  66      12.960  13.997  27.111  1.00 12.81           C  0.019
ANISOU 1084  CG  ASP A  66     1822   1870   1173    412     94    332       C
ATOM   1085  OD1 ASP A  66      13.357  14.045  28.313  1.00 13.84           O  0.019
ANISOU 1085  OD1 ASP A  66     1919   2049   1291    399    106    413       O
ATOM   1086  OD2 ASP A  66      13.187  14.904  26.281  1.00 12.86           O  0.019
ANISOU 1086  OD2 ASP A  66     1862   1885   1140    304    -64    148       O
ATOM   1087  H   ASP A  66      11.088  12.956  29.018  1.00 16.45           H  0.021
ATOM   1088  HA  ASP A  66      11.367  11.016  27.165  1.00 16.63           H  0.021
ATOM   1089  HB2 ASP A  66      12.868  12.291  25.971  1.00 15.80           H  0.021
ATOM   1090  HB3 ASP A  66      11.474  13.041  26.064  1.00 15.80           H  0.021
ATOM   1091  N   GLY A  67      12.998  11.131  29.691  1.00 15.62           N  0.021
```

```
ANISOU 1091  N   GLY A  67     2143   2112   1679    598    328    404       N
ATOM   1092  CA  GLY A  67      14.084  10.546  30.467  1.00 17.20           C  0.022
ANISOU 1092  CA  GLY A  67     2309   2326   1902    525    195    423       C
ATOM   1093  C   GLY A  67      15.394  11.297  30.467  1.00 17.13           C  0.022
ANISOU 1093  C   GLY A  67     2303   2387   1817    627     25    412       C
ATOM   1094  O   GLY A  67      16.357  10.840  31.099  1.00 18.55           O  0.023
ANISOU 1094  O   GLY A  67     2445   2492   2111    595   -287    469       O
ATOM   1095  H   GLY A  67      12.371  11.460  30.179  1.00 18.74           H  0.023
ATOM   1096  HA2 GLY A  67      13.794  10.462  31.389  1.00 20.65           H  0.024
ATOM   1097  HA3 GLY A  67      14.258   9.653  30.130  1.00 20.65           H  0.024
ATOM   1098  N  AARG A  68      15.474  12.446  29.805  0.58 15.92           N  0.021
ANISOU 1098  N  AARG A  68     2219   2428   1401    604    296    289       N
ATOM   1099  CA AARG A  68      16.770  13.165  29.726  0.58 16.53           C  0.021
ANISOU 1099  CA AARG A  68     2167   2640   1475    501    176      6       C
ATOM   1100  C  AARG A  68      16.562  14.657  29.988  0.58 17.57           C  0.022
ANISOU 1100  C  AARG A  68     2217   2647   1810    345    222    -78       C
ATOM   1101  O  AARG A  68      17.416  15.443  29.561  0.58 19.71           O  0.023
ANISOU 1101  O  AARG A  68     2222   2752   2515    210    493   -302       O
ATOM   1102  CB AARG A  68      17.432  12.869  28.378  0.58 17.51           C  0.022
ANISOU 1102  CB AARG A  68     2282   2826   1544    446    247    -83       C
ATOM   1103  CG AARG A  68      16.579  13.200  27.165  0.58 18.76           C  0.023
ANISOU 1103  CG AARG A  68     2315   3082   1733    382    619   -194       C
ATOM   1104  CD AARG A  68      17.467  13.366  25.945  0.58 18.34           C  0.022
ANISOU 1104  CD AARG A  68     2182   3196   1591    277    753   -420       C
ATOM   1105  NE AARG A  68      16.862  14.122  24.864  0.58 19.36           N  0.023
ANISOU 1105  NE AARG A  68     2039   3315   2001    334    562   -337       N
ATOM   1106  CZ AARG A  68      17.506  14.584  23.795  0.58 18.52           C  0.023
ANISOU 1106  CZ AARG A  68     1966   3201   1870    427    367   -601       C
ATOM   1107  NH1AARG A  68      18.799  14.367  23.637  0.58 19.74           N  0.023
ANISOU 1107  NH1AARG A  68     2303   3360   1838    363    339   -259       N
ATOM   1108  NH2AARG A  68      16.853  15.273  22.878  0.58 20.69           N  0.024
ANISOU 1108  NH2AARG A  68     2051   3300   2510    368   -230   -304       N
ATOM   1109  H  AARG A  68      14.646  13.041  29.621  0.58 19.10           H  0.023
ATOM   1110  HA AARG A  68      17.422  12.784  30.510  0.58 19.84           H  0.023
ATOM   1111  HB2AARG A  68      18.361  13.433  28.318  0.58 21.01           H  0.024
ATOM   1112  HB3AARG A  68      17.682  11.811  28.345  0.58 21.01           H  0.024
ATOM   1113  HG2AARG A  68      15.865  12.397  26.988  0.58 22.52           H  0.025
ATOM   1114  HG3AARG A  68      16.024  14.115  27.352  0.58 22.52           H  0.025
ATOM   1115  HD2AARG A  68      18.385  13.867  26.248  0.58 22.01           H  0.025
ATOM   1116  HD3AARG A  68      17.739  12.379  25.574  0.58 22.01           H  0.025
ATOM   1117  HE AARG A  68      15.863  14.285  24.910  0.58 23.23           H  0.025
ATOM   1118 HH11AARG A  68      19.329  13.860  24.340  0.58 23.69           H  0.025
ATOM   1119 HH12AARG A  68      19.273  14.748  22.823  0.58 23.69           H  0.025
ATOM   1120 HH21AARG A  68      15.854  15.429  22.978  0.58 24.83           H  0.026
ATOM   1121 HH22AARG A  68      17.347  15.622  22.062  0.58 24.83           H  0.026
ATOM   1122  N  BARG A  68      15.459  12.467  29.833  0.42 16.37           N  0.021
ANISOU 1122  N  BARG A  68     2290   2319   1610    648     75    383       N
ATOM   1123  CA BARG A  68      16.768  13.163  29.736  0.42 16.65           C  0.021
ANISOU 1123  CA BARG A  68     2274   2388   1663    601   -168    300       C
ATOM   1124  C  BARG A  68      16.563  14.656  29.991  0.42 17.46           C  0.022
ANISOU 1124  C  BARG A  68     2269   2508   1859    409     83     58       C
ATOM   1125  O  BARG A  68      17.411  15.442  29.547  0.42 19.30           O  0.023
ANISOU 1125  O  BARG A  68     2302   2603   2427    291    327   -159       O
ATOM   1126  CB BARG A  68      17.422  12.844  28.390  0.42 17.33           C  0.022
ANISOU 1126  CB BARG A  68     2454   2327   1803    630   -412    512       C
ATOM   1127  CG BARG A  68      16.624  13.270  27.171  0.42 19.49           C  0.023
ANISOU 1127  CG BARG A  68     2722   2392   2290    515   -184    323       C
ATOM   1128  CD BARG A  68      17.585  13.721  26.091  0.42 21.08           C  0.024
ANISOU 1128  CD BARG A  68     3025   2416   2567    345    -29    144       C
ATOM   1129  NE BARG A  68      18.245  14.960  26.485  0.42 23.31           N  0.025
ANISOU 1129  NE BARG A  68     3321   2506   3029    176    234   -410       N
ATOM   1130  CZ BARG A  68      19.477  15.346  26.175  0.42 24.09           C  0.026
ANISOU 1130  CZ BARG A  68     3441   2614   3096    -18    192   -364       C
ATOM   1131  NH1BARG A  68      20.290  14.596  25.447  0.42 23.62           N  0.025
ANISOU 1131  NH1BARG A  68     3408   2576   2991    109     11   -356       N
ATOM   1132  NH2BARG A  68      19.897  16.512  26.630  0.42 24.99           N  0.026
ANISOU 1132  NH2BARG A  68     3541   2671   3282    -76    218   -398       N
ATOM   1133  H  BARG A  68      14.798  12.873  29.462  0.42 19.64           H  0.023
ATOM   1134  HA BARG A  68      17.357  12.831  30.431  0.42 19.98           H  0.023
ATOM   1135  HB2BARG A  68      18.283  13.288  28.354  0.42 20.80           H  0.024
ATOM   1136  HB3BARG A  68      17.551  11.884  28.332  0.42 20.80           H  0.024
ATOM   1137  HG2BARG A  68      16.110  12.518  26.837  0.42 23.38           H  0.025
ATOM   1138  HG3BARG A  68      16.041  14.011  27.399  0.42 23.38           H  0.025
ATOM   1139  HD2BARG A  68      18.261  13.039  25.958  0.42 25.29           H  0.026
ATOM   1140  HD3BARG A  68      17.097  13.880  25.268  0.42 25.29           H  0.026
ATOM   1141  HE BARG A  68      17.786  15.500  26.972  0.42 27.97           H  0.028
ATOM   1142 HH11BARG A  68      20.024  13.835  25.149  0.42 28.35           H  0.028
ATOM   1143 HH12BARG A  68      21.083  14.874  25.266  0.42 28.35           H  0.028
```

```
ATOM   1144 HH21BARG A  68      19.374  17.002  27.105  0.42 29.98           H  0.029
ATOM   1145 HH22BARG A  68      20.692  16.785  26.446  0.42 29.98           H  0.029
ATOM   1146  N   THR A  69      15.513  15.025  30.729  1.00 15.74           N  0.021
ANISOU 1146  N   THR A  69     2148   2480   1354    443    116     77       N
ATOM   1147  CA  THR A  69      15.258  16.415  31.118  1.00 15.18           C  0.020
ANISOU 1147  CA  THR A  69     2025   2560   1183    424     34    160       C
ATOM   1148  C   THR A  69      15.188  16.483  32.642  1.00 16.48           C  0.021
ANISOU 1148  C   THR A  69     2295   2952   1014    483    103    290       C
ATOM   1149  O   THR A  69      14.110  16.483  33.235  1.00 17.78           O  0.022
ANISOU 1149  O   THR A  69     2337   3327   1091    603    139    236       O
ATOM   1150  CB  THR A  69      13.989  16.942  30.443  1.00 14.55           C  0.020
ANISOU 1150  CB  THR A  69     2006   2414   1109    386   -123     32       C
ATOM   1151  OG1 THR A  69      14.021  16.642  29.031  1.00 14.08           O  0.020
ANISOU 1151  OG1 THR A  69     1900   2299   1150    419    -71     59       O
ATOM   1152  CG2 THR A  69      13.892  18.426  30.609  1.00 15.49           C  0.021
ANISOU 1152  CG2 THR A  69     2202   2484   1200    503    -98     32       C
ATOM   1153  H   THR A  69      14.913  14.481  31.017  1.00 18.89           H  0.023
ATOM   1154  HA  THR A  69      15.994  16.971  30.820  1.00 18.22           H  0.022
ATOM   1155  HB  THR A  69      13.208  16.532  30.847  1.00 17.47           H  0.022
ATOM   1156  HG1 THR A  69      14.035  15.810  28.914  1.00 16.90           H  0.022
ATOM   1157 HG21 THR A  69      13.074  18.752  30.202  1.00 18.59           H  0.023
ATOM   1158 HG22 THR A  69      13.889  18.656  31.551  1.00 18.59           H  0.023
ATOM   1159 HG23 THR A  69      14.649  18.857  30.182  1.00 18.59           H  0.023
ATOM   1160  N   PRO A  70      16.321  16.602  33.314  1.00 17.26           N  0.022
ANISOU 1160  N   PRO A  70     2498   3203    859    330    -95    188       N
ATOM   1161  CA  PRO A  70      16.323  16.491  34.780  1.00 20.12           C  0.023
ANISOU 1161  CA  PRO A  70     2901   3484   1258    165   -123    289       C
ATOM   1162  C   PRO A  70      15.418  17.508  35.436  1.00 23.27           C  0.025
ANISOU 1162  C   PRO A  70     3480   3593   1769    275    104    201       C
ATOM   1163  O   PRO A  70      15.405  18.680  35.074  1.00 25.37           O  0.026
ANISOU 1163  O   PRO A  70     3912   3629   2098    227    470   -250       O
ATOM   1164  CB  PRO A  70      17.788  16.753  35.154  1.00 21.89           C  0.024
ANISOU 1164  CB  PRO A  70     2820   3663   1832     37   -272    120       C
ATOM   1165  CG  PRO A  70      18.521  16.371  33.989  1.00 20.59           C  0.024
ANISOU 1165  CG  PRO A  70     2700   3607   1517     11   -201    103       C
ATOM   1166  CD  PRO A  70      17.681  16.729  32.780  1.00 19.18           C  0.023
ANISOU 1166  CD  PRO A  70     2615   3482   1193     75    -81    124       C
ATOM   1167  HA  PRO A  70      16.069  15.597  35.058  1.00 24.14           H  0.026
ATOM   1168  HB2 PRO A  70      17.914  17.695  35.346  1.00 26.26           H  0.027
ATOM   1169  HB3 PRO A  70      18.038  16.209  35.916  1.00 26.26           H  0.027
ATOM   1170  HG2 PRO A  70      19.362  16.854  33.968  1.00 24.71           H  0.026
ATOM   1171  HG3 PRO A  70      18.684  15.415  34.013  1.00 24.71           H  0.026
ATOM   1172  HD2 PRO A  70      17.855  17.641  32.499  1.00 23.02           H  0.025
ATOM   1173  HD3 PRO A  70      17.830  16.095  32.062  1.00 23.02           H  0.025
ATOM   1174  N   GLY A  71      14.688  17.047  36.439  1.00 23.49           N  0.025
ANISOU 1174  N   GLY A  71     3571   3729   1626    514    370    115       N
ATOM   1175  CA  GLY A  71      13.860  17.896  37.245  1.00 26.24           C  0.027
ANISOU 1175  CA  GLY A  71     3660   3896   2415    875    327     73       C
ATOM   1176  C   GLY A  71      12.545  18.248  36.614  1.00 24.82           C  0.026
ANISOU 1176  C   GLY A  71     3553   3913   1966   1371    415     57       C
ATOM   1177  O   GLY A  71      11.809  19.082  37.162  1.00 27.30           O  0.027
ANISOU 1177  O   GLY A  71     3687   4055   2632   1432    408   -142       O
ATOM   1178  H   GLY A  71      14.661  16.219  36.672  1.00 28.19           H  0.028
ATOM   1179  HA2 GLY A  71      13.681  17.455  38.090  1.00 31.49           H  0.029
ATOM   1180  HA3 GLY A  71      14.335  18.722  37.428  1.00 31.49           H  0.029
ATOM   1181  N   SER A  72      12.220  17.651  35.473  1.00 24.43           N  0.026
ANISOU 1181  N   SER A  72     3517   3943   1824   1460    125    249       N
ATOM   1182  CA  SER A  72      11.082  18.083  34.701  1.00 23.54           C  0.025
ANISOU 1182  CA  SER A  72     3410   3913   1623   1383    165    127       C
ATOM   1183  C   SER A  72       9.846  17.327  35.198  1.00 23.98           C  0.026
ANISOU 1183  C   SER A  72     3355   3982   1775   1418    141    306       C
ATOM   1184  O   SER A  72       9.906  16.374  35.997  1.00 25.49           O  0.026
ANISOU 1184  O   SER A  72     3483   4124   2077   1524    313    360       O
ATOM   1185  CB  SER A  72      11.294  17.794  33.223  1.00 22.41           C  0.025
ANISOU 1185  CB  SER A  72     3301   3711   1505   1275    175    -39       C
ATOM   1186  OG  SER A  72      11.421  16.405  33.016  1.00 22.18           O  0.025
ANISOU 1186  OG  SER A  72     3206   3653   1570   1162    214    -78       O
ATOM   1187  H   SER A  72      12.649  16.990  35.128  1.00 29.32           H  0.028
ATOM   1188  HA  SER A  72      10.940  19.035  34.820  1.00 28.25           H  0.028
ATOM   1189  HB2 SER A  72      10.531  18.118  32.721  1.00 26.90           H  0.027
ATOM   1190  HB3 SER A  72      12.104  18.237  32.925  1.00 26.90           H  0.027
ATOM   1191  N   ARG A  73       8.715  17.787  34.726  1.00 23.07           N  0.025
ANISOU 1191  N   ARG A  73     3221   3817   1729   1290    173     98       N
ATOM   1192  CA  ARG A  73       7.456  17.135  34.992  1.00 23.32           C  0.025
ANISOU 1192  CA  ARG A  73     3076   3622   2163   1076    197    247       C
ATOM   1193  C   ARG A  73       6.999  16.462  33.704  1.00 20.85           C  0.024
ANISOU 1193  C   ARG A  73     2790   3230   1903    872    556    438       C
ATOM   1194  O   ARG A  73       7.621  16.604  32.636  1.00 21.56           O  0.024
```

```
ANISOU 1194  O   ARG A  73     2757   3090   2344    884    338    457       O
ATOM   1195  CB  ARG A  73       6.441  18.160  35.502  1.00 23.95           C  0.026
ANISOU 1195  CB  ARG A  73     3086   3785   2231   1184    595     80       C
ATOM   1196  CG  ARG A  73       6.951  18.898  36.752  1.00 25.75           C  0.027
ANISOU 1196  CG  ARG A  73     3235   4057   2491   1158   1005    112       C
ATOM   1197  CD  ARG A  73       6.896  18.067  38.039  1.00 29.09           C  0.028
ANISOU 1197  CD  ARG A  73     3410   4378   3266   1189   1143    183       C
ATOM   1198  NE  ARG A  73       5.522  17.905  38.515  1.00 32.32           N  0.030
ANISOU 1198  NE  ARG A  73     3665   4703   3913   1077   1445    290       N
ATOM   1199  CZ  ARG A  73       4.829  18.821  39.181  1.00 34.82           C  0.031
ANISOU 1199  CZ  ARG A  73     3841   4919   4469   1015   1622    281       C
ATOM   1200  NH1 ARG A  73       5.362  19.983  39.519  1.00 35.75           N  0.031
ANISOU 1200  NH1 ARG A  73     3921   5025   4635   1020   1709    159       N
ATOM   1201  NH2 ARG A  73       3.562  18.571  39.500  1.00 35.60           N  0.031
ANISOU 1201  NH2 ARG A  73     3935   5003   4590    988   1671    224       N
ATOM   1202  H   ARG A  73       8.646  18.491  34.237  1.00 27.69           H  0.028
ATOM   1203  HA  ARG A  73       7.572  16.453  35.672  1.00 27.99           H  0.028
ATOM   1204  HB2 ARG A  73       6.276  18.818  34.808  1.00 28.75           H  0.028
ATOM   1205  HB3 ARG A  73       5.617  17.704  35.734  1.00 28.75           H  0.028
ATOM   1206  HG2 ARG A  73       7.872  19.166  36.612  1.00 30.90           H  0.029
ATOM   1207  HG3 ARG A  73       6.402  19.686  36.890  1.00 30.90           H  0.029
ATOM   1208  HD2 ARG A  73       7.263  17.186  37.866  1.00 34.91           H  0.031
ATOM   1209  HD3 ARG A  73       7.408  18.513  38.731  1.00 34.91           H  0.031
ATOM   1210  HE  ARG A  73       5.132  17.156  38.350  1.00 38.79           H  0.033
ATOM   1211 HH11 ARG A  73       6.178  20.160  39.315  1.00 42.90           H  0.034
ATOM   1212 HH12 ARG A  73       4.893  20.561  39.949  1.00 42.90           H  0.034
ATOM   1213 HH21 ARG A  73       3.205  17.819  39.285  1.00 42.73           H  0.034
ATOM   1214 HH22 ARG A  73       3.104  19.156  39.933  1.00 42.73           H  0.034
ATOM   1215  N   ASN A  74       5.910  15.714  33.839  1.00 19.78           N  0.023
ANISOU 1215  N   ASN A  74     2437   2943   2135    692    613    635       N
ATOM   1216  CA  ASN A  74       5.261  14.949  32.779  1.00 18.36           C  0.022
ANISOU 1216  CA  ASN A  74     2360   2889   1727    690    603    435       C
ATOM   1217  C   ASN A  74       3.759  15.140  32.998  1.00 17.87           C  0.022
ANISOU 1217  C   ASN A  74     2212   2879   1698    706    698    392       C
ATOM   1218  O   ASN A  74       3.018  14.222  33.304  1.00 18.24           O  0.022
ANISOU 1218  O   ASN A  74     2189   3008   1733    702    634    634       O
ATOM   1219  CB  ASN A  74       5.666  13.478  32.845  1.00 18.36           C  0.022
ANISOU 1219  CB  ASN A  74     2368   2698   1912    710    659    474       C
ATOM   1220  CG  ASN A  74       4.976  12.616  31.808  1.00 18.38           C  0.022
ANISOU 1220  CG  ASN A  74     2327   2592   2063    655    579    563       C
ATOM   1221  OD1 ASN A  74       4.347  13.108  30.885  1.00 17.75           O  0.022
ANISOU 1221  OD1 ASN A  74     2280   2461   2002    553    423    514       O
ATOM   1222  ND2 ASN A  74       5.148  11.314  31.931  1.00 19.69           N  0.023
ANISOU 1222  ND2 ASN A  74     2429   2622   2430    756    361    575       N
ATOM   1223  H   ASN A  74       5.502  15.630  34.591  1.00 23.73           H  0.025
ATOM   1224  HA  ASN A  74       5.504  15.308  31.911  1.00 22.04           H  0.025
ATOM   1225  HB2 ASN A  74       6.623  13.409  32.700  1.00 22.04           H  0.025
ATOM   1226  HB3 ASN A  74       5.439  13.129  33.721  1.00 22.04           H  0.025
ATOM   1227 HD21 ASN A  74       4.783  10.777  31.367  1.00 23.63           H  0.025
ATOM   1228 HD22 ASN A  74       5.606  11.001  32.588  1.00 23.63           H  0.025
ATOM   1229  N   LEU A  75       3.302  16.379  32.835  1.00 17.48           N  0.022
ANISOU 1229  N   LEU A  75     2090   2865   1688    732    585    389       N
ATOM   1230  CA  LEU A  75       1.941  16.739  33.221  1.00 18.37           C  0.022
ANISOU 1230  CA  LEU A  75     2143   2972   1864    693    491    362       C
ATOM   1231  C   LEU A  75       0.905  16.162  32.267  1.00 17.82           C  0.022
ANISOU 1231  C   LEU A  75     1964   2851   1955    611    452    243       C
ATOM   1232  O   LEU A  75      -0.259  16.038  32.652  1.00 20.28           O  0.024
ANISOU 1232  O   LEU A  75     2007   3180   2518    541    614     55       O
ATOM   1233  CB  LEU A  75       1.822  18.268  33.344  1.00 20.14           C  0.023
ANISOU 1233  CB  LEU A  75     2404   3181   2068    581    403    252       C
ATOM   1234  CG  LEU A  75       2.600  18.889  34.510  1.00 22.45           C  0.025
ANISOU 1234  CG  LEU A  75     2831   3398   2300    447    557    143       C
ATOM   1235  CD1 LEU A  75       2.638  20.413  34.431  1.00 23.84           C  0.026
ANISOU 1235  CD1 LEU A  75     3010   3438   2610    439    508    -81       C
ATOM   1236  CD2 LEU A  75       2.007  18.469  35.862  1.00 24.46           C  0.026
ANISOU 1236  CD2 LEU A  75     3094   3612   2586    386    501     71       C
ATOM   1237  H   LEU A  75       3.759  17.028  32.504  1.00 20.98           H  0.024
ATOM   1238  HA  LEU A  75       1.763  16.361  34.096  1.00 22.04           H  0.025
ATOM   1239  HB2 LEU A  75       2.153  18.669  32.526  1.00 24.17           H  0.026
ATOM   1240  HB3 LEU A  75       0.887  18.496  33.462  1.00 24.17           H  0.026
ATOM   1241  HG  LEU A  75       3.515  18.569  34.478  1.00 26.94           H  0.027
ATOM   1242 HD11 LEU A  75       3.449  20.683  33.972  1.00 28.61           H  0.028
ATOM   1243 HD12 LEU A  75       1.863  20.721  33.937  1.00 28.61           H  0.028
ATOM   1244 HD13 LEU A  75       2.630  20.780  35.329  1.00 28.61           H  0.028
ATOM   1245 HD21 LEU A  75       2.489  18.919  36.573  1.00 29.35           H  0.028
ATOM   1246 HD22 LEU A  75       1.070  18.720  35.887  1.00 29.35           H  0.028
ATOM   1247 HD23 LEU A  75       2.095  17.509  35.963  1.00 29.35           H  0.028
ATOM   1248  N   CYS A  76       1.284  15.779  31.045  1.00 17.03           N  0.022
```

```
ANISOU 1248  N   CYS A  76     1940   2559   1972    465    416    361       N
ATOM   1249  CA  CYS A  76       0.364  15.067  30.164  1.00 17.27           C  0.022
ANISOU 1249  CA  CYS A  76     1954   2374   2233    408    461    523       C
ATOM   1250  C   CYS A  76       0.415  13.553  30.348  1.00 17.97           C  0.022
ANISOU 1250  C   CYS A  76     1992   2471   2364    294    402    688       C
ATOM   1251  O   CYS A  76      -0.385  12.833  29.718  1.00 18.24           O  0.022
ANISOU 1251  O   CYS A  76     1943   2668   2319    130    461    686       O
ATOM   1252  CB  CYS A  76       0.649  15.427  28.700  1.00 16.76           C  0.021
ANISOU 1252  CB  CYS A  76     1981   2235   2152    335    376    516       C
ATOM   1253  SG  CYS A  76       0.174  17.154  28.385  1.00 15.83           S  0.021
ANISOU 1253  SG  CYS A  76     1894   2126   1996    276    146    200       S
ATOM   1254  H   CYS A  76       2.062  15.919  30.707  1.00 20.44           H  0.024
ATOM   1255  HA  CYS A  76      -0.539  15.356  30.362  1.00 20.72           H  0.024
ATOM   1256  HB2 CYS A  76       1.591  15.319  28.507  1.00 20.11           H  0.023
ATOM   1257  HB3 CYS A  76       0.120  14.856  28.121  1.00 20.11           H  0.023
ATOM   1258  N   ASN A  77       1.312  13.067  31.205  1.00 18.39           N  0.022
ANISOU 1258  N   ASN A  77     2083   2529   2377    439    660    752       N
ATOM   1259  CA  ASN A  77       1.379  11.641  31.522  1.00 19.45           C  0.023
ANISOU 1259  CA  ASN A  77     2264   2610   2515    359    828    925       C
ATOM   1260  C   ASN A  77       1.570  10.790  30.272  1.00 19.34           C  0.023
ANISOU 1260  C   ASN A  77     2233   2416   2701    278    682    729       C
ATOM   1261  O   ASN A  77       0.866   9.799  30.061  1.00 20.38           O  0.024
ANISOU 1261  O   ASN A  77     2344   2566   2831    121    820    462       O
ATOM   1262  CB  ASN A  77       0.155  11.225  32.336  1.00 22.74           C  0.025
ANISOU 1262  CB  ASN A  77     2730   3013   2897    382   1129   1026       C
ATOM   1263  CG  ASN A  77       0.120  11.864  33.690  1.00 27.69           C  0.028
ANISOU 1263  CG  ASN A  77     3135   3586   3801    347   1188    970       C
ATOM   1264  OD1 ASN A  77       1.066  11.727  34.477  1.00 30.14           O  0.029
ANISOU 1264  OD1 ASN A  77     3484   3971   3996     68   1202    921       O
ATOM   1265  ND2 ASN A  77      -0.916  12.666  33.934  1.00 30.06           N  0.029
ANISOU 1265  ND2 ASN A  77     3394   3689   4338    435   1077    907       N
ATOM   1266  H   ASN A  77       1.897  13.543  31.618  1.00 22.07           H  0.025
ATOM   1267  HA  ASN A  77       2.156  11.495  32.084  1.00 23.34           H  0.025
ATOM   1268  HB2 ASN A  77      -0.648  11.486  31.859  1.00 27.29           H  0.027
ATOM   1269  HB3 ASN A  77       0.170  10.263  32.459  1.00 27.29           H  0.027
ATOM   1270 HD21 ASN A  77      -1.543  12.748  33.351  1.00 36.07           H  0.031
ATOM   1271 HD22 ASN A  77      -0.980  13.066  34.693  1.00 36.07           H  0.031
ATOM   1272  N   ILE A  78       2.595  11.137  29.490  1.00 17.92           N  0.022
ANISOU 1272  N   ILE A  78     2136   2129   2545    250    667    532       N
ATOM   1273  CA  ILE A  78       2.893  10.455  28.227  1.00 17.84           C  0.022
ANISOU 1273  CA  ILE A  78     2057   2126   2596    202    667    454       C
ATOM   1274  C   ILE A  78       4.389  10.446  27.975  1.00 17.82           C  0.022
ANISOU 1274  C   ILE A  78     2067   2101   2602    145    654    443       C
ATOM   1275  O   ILE A  78       5.084  11.398  28.340  1.00 17.59           O  0.022
ANISOU 1275  O   ILE A  78     2223   2074   2386    153    599    360       O
ATOM   1276  CB  ILE A  78       2.190  11.099  27.020  1.00 20.46           C  0.024
ANISOU 1276  CB  ILE A  78     2252   2320   3203    132    551    550       C
ATOM   1277  CG1 ILE A  78       2.418  12.608  27.003  1.00 20.19           C  0.024
ANISOU 1277  CG1 ILE A  78     2367   2335   2971    277    419    610       C
ATOM   1278  CG2 ILE A  78       0.734  10.725  27.002  1.00 22.53           C  0.025
ANISOU 1278  CG2 ILE A  78     2318   2546   3694     97    412    492       C
ATOM   1279  CD1 ILE A  78       2.200  13.262  25.641  1.00 21.27           C  0.024
ANISOU 1279  CD1 ILE A  78     2460   2465   3155    247    406    634       C
ATOM   1280  H   ILE A  78       3.142  11.775  29.673  1.00 21.51           H  0.024
ATOM   1281  HA  ILE A  78       2.583   9.540  28.299  1.00 21.41           H  0.024
ATOM   1282  HB  ILE A  78       2.595  10.734  26.217  1.00 24.56           H  0.026
ATOM   1283 HG12 ILE A  78       1.802  13.021  27.629  1.00 24.24           H  0.026
ATOM   1284 HG13 ILE A  78       3.330  12.794  27.273  1.00 24.24           H  0.026
ATOM   1285 HG21 ILE A  78       0.319  11.112  26.216  1.00 27.03           H  0.027
ATOM   1286 HG22 ILE A  78       0.657   9.758  26.975  1.00 27.03           H  0.027
ATOM   1287 HG23 ILE A  78       0.310  11.069  27.804  1.00 27.03           H  0.027
ATOM   1288 HD11 ILE A  78       2.373  14.213  25.717  1.00 25.52           H  0.026
ATOM   1289 HD12 ILE A  78       2.809  12.865  24.999  1.00 25.52           H  0.026
ATOM   1290 HD13 ILE A  78       1.283  13.113  25.363  1.00 25.52           H  0.026
ATOM   1291  N   PRO A  79       4.901   9.428  27.312  1.00 17.52           N  0.022
ANISOU 1291  N   PRO A  79     2169   1913   2576    165    532    188       N
ATOM   1292  CA  PRO A  79       6.258   9.535  26.775  1.00 16.54           C  0.021
ANISOU 1292  CA  PRO A  79     2091   1769   2426    291    503    167       C
ATOM   1293  C   PRO A  79       6.262  10.561  25.659  1.00 14.90           C  0.020
ANISOU 1293  C   PRO A  79     1948   1712   2002    211    263    144       C
ATOM   1294  O   PRO A  79       5.315  10.650  24.880  1.00 15.38           O  0.021
ANISOU 1294  O   PRO A  79     1905   1687   2250    100    227    263       O
ATOM   1295  CB  PRO A  79       6.541   8.129  26.235  1.00 17.71           C  0.022
ANISOU 1295  CB  PRO A  79     2234   1804   2691    303    515    232       C
ATOM   1296  CG  PRO A  79       5.200   7.569  25.917  1.00 20.07           C  0.023
ANISOU 1296  CG  PRO A  79     2358   1895   3374    252    361     88       C
ATOM   1297  CD  PRO A  79       4.251   8.154  26.952  1.00 19.13           C  0.023
ANISOU 1297  CD  PRO A  79     2264   1945   3059    172    419    367       C
```

```
ATOM   1298  HA  PRO A  79       6.898   9.773  27.464  1.00 19.85           H  0.023
ATOM   1299  HB2 PRO A  79       7.088   8.188  25.436  1.00 21.25           H  0.024
ATOM   1300  HB3 PRO A  79       6.983   7.599  26.916  1.00 21.25           H  0.024
ATOM   1301  HG2 PRO A  79       4.938   7.840  25.023  1.00 24.09           H  0.026
ATOM   1302  HG3 PRO A  79       5.227   6.602  25.987  1.00 24.09           H  0.026
ATOM   1303  HD2 PRO A  79       3.380   8.315  26.557  1.00 22.96           H  0.025
ATOM   1304  HD3 PRO A  79       4.191   7.572  27.726  1.00 22.96           H  0.025
ATOM   1305  N   CYS A  80       7.318  11.362  25.591  1.00 13.97           N  0.020
ANISOU 1305  N   CYS A  80     1802   1737   1769    280    148    210       N
ATOM   1306  CA  CYS A  80       7.381  12.363  24.526  1.00 13.84           C  0.019
ANISOU 1306  CA  CYS A  80     1825   1710   1724    233    110    248       C
ATOM   1307  C   CYS A  80       7.309  11.722  23.147  1.00 14.01           C  0.020
ANISOU 1307  C   CYS A  80     1892   1657   1775    258     91     51       C
ATOM   1308  O   CYS A  80       6.813  12.331  22.199  1.00 14.35           O  0.020
ANISOU 1308  O   CYS A  80     1883   1790   1780    178     65    167       O
ATOM   1309  CB  CYS A  80       8.659  13.192  24.657  1.00 13.75           C  0.019
ANISOU 1309  CB  CYS A  80     1824   1708   1692    325    231    318       C
ATOM   1310  SG  CYS A  80       8.795  14.143  26.200  1.00 13.80           S  0.019
ANISOU 1310  SG  CYS A  80     1886   1772   1584    310    296    139       S
ATOM   1311  H   CYS A  80       7.990  11.352  26.127  1.00 16.77           H  0.021
ATOM   1312  HA  CYS A  80       6.625  12.964  24.615  1.00 16.61           H  0.021
ATOM   1313  HB2 CYS A  80       9.422  12.595  24.612  1.00 16.50           H  0.021
ATOM   1314  HB3 CYS A  80       8.691  13.817  23.920  1.00 16.50           H  0.021
ATOM   1315  N   SER A  81       7.799  10.504  23.001  1.00 14.99           N  0.020
ANISOU 1315  N   SER A  81     2053   1730   1912    286    212     93       N
ATOM   1316  CA  SER A  81       7.731   9.839  21.704  1.00 16.44           C  0.021
ANISOU 1316  CA  SER A  81     2359   1763   2126    328     48   -105       C
ATOM   1317  C   SER A  81       6.299   9.710  21.189  1.00 17.97           C  0.022
ANISOU 1317  C   SER A  81     2494   1857   2477    232   -220    -22       C
ATOM   1318  O   SER A  81       6.077   9.665  19.968  1.00 17.85           O  0.022
ANISOU 1318  O   SER A  81     2570   1834   2377    217   -349    -71       O
ATOM   1319  CB  SER A  81       8.342   8.442  21.810  1.00 17.83           C  0.022
ANISOU 1319  CB  SER A  81     2514   1972   2290    345     78    -80       C
ATOM   1320  OG  SER A  81       7.625   7.656  22.743  1.00 20.12           O  0.023
ANISOU 1320  OG  SER A  81     2762   1984   2897    322    -28     29       O
ATOM   1321  H   SER A  81       8.172  10.043  23.624  1.00 17.99           H  0.022
ATOM   1322  HA  SER A  81       8.243  10.348  21.056  1.00 19.73           H  0.023
ATOM   1323  HB2 SER A  81       8.305   8.014  20.941  1.00 21.40           H  0.024
ATOM   1324  HB3 SER A  81       9.263   8.521  22.104  1.00 21.40           H  0.024
ATOM   1325  HG  SER A  81       6.839   7.534  22.474  1.00 24.14           H  0.026
ATOM   1326  N   ALA A  82       5.317   9.640  22.089  1.00 17.91           N  0.022
ANISOU 1326  N   ALA A  82     2389   1889   2525    114   -231    188       N
ATOM   1327  CA  ALA A  82       3.924   9.529  21.658  1.00 18.78           C  0.023
ANISOU 1327  CA  ALA A  82     2315   2062   2760     22   -161    272       C
ATOM   1328  C   ALA A  82       3.478  10.749  20.865  1.00 18.00           C  0.022
ANISOU 1328  C   ALA A  82     2088   2013   2737     85   -231     21       C
ATOM   1329  O   ALA A  82       2.517  10.680  20.079  1.00 19.28           O  0.023
ANISOU 1329  O   ALA A  82     2065   2014   3246      6   -286     14       O
ATOM   1330  CB  ALA A  82       3.014   9.374  22.865  1.00 19.79           C  0.023
ANISOU 1330  CB  ALA A  82     2413   2167   2940    -11   -150    313       C
ATOM   1331  H   ALA A  82       5.428   9.654  22.942  1.00 21.49           H  0.024
ATOM   1332  HA  ALA A  82       3.823   8.745  21.096  1.00 22.54           H  0.025
ATOM   1333  HB1 ALA A  82       2.096   9.302  22.560  1.00 23.75           H  0.025
ATOM   1334  HB2 ALA A  82       3.266   8.572  23.349  1.00 23.75           H  0.025
ATOM   1335  HB3 ALA A  82       3.113  10.151  23.437  1.00 23.75           H  0.025
ATOM   1336  N   LEU A  83       4.130  11.878  21.081  1.00 16.42           N  0.021
ANISOU 1336  N   LEU A  83     1926   1896   2417     57    -13    -80       N
ATOM   1337  CA  LEU A  83       3.825  13.117  20.381  1.00 16.77           C  0.021
ANISOU 1337  CA  LEU A  83     1931   1957   2485    143     20     65       C
ATOM   1338  C   LEU A  83       4.366  13.138  18.954  1.00 16.58           C  0.021
ANISOU 1338  C   LEU A  83     2028   1875   2395    265    -45    -63       C
ATOM   1339  O   LEU A  83       4.166  14.135  18.251  1.00 16.67           O  0.021
ANISOU 1339  O   LEU A  83     2201   1834   2301    343   -137    -22       O
ATOM   1340  CB  LEU A  83       4.417  14.289  21.176  1.00 16.57           C  0.021
ANISOU 1340  CB  LEU A  83     1898   2079   2317    306    -20    -69       C
ATOM   1341  CG  LEU A  83       3.883  14.462  22.606  1.00 17.35           C  0.022
ANISOU 1341  CG  LEU A  83     2007   2176   2409    412    -69     97       C
ATOM   1342  CD1 LEU A  83       4.682  15.463  23.394  1.00 18.05           C  0.022
ANISOU 1342  CD1 LEU A  83     2277   2077   2503    426    -50    171       C
ATOM   1343  CD2 LEU A  83       2.438  14.906  22.608  1.00 19.01           C  0.023
ANISOU 1343  CD2 LEU A  83     2118   2488   2616    463   -216     25       C
ATOM   1344  H   LEU A  83       4.773  11.957  21.646  1.00 19.71           H  0.023
ATOM   1345  HA  LEU A  83       2.863  13.232  20.341  1.00 20.13           H  0.023
ATOM   1346  HB2 LEU A  83       5.377  14.163  21.238  1.00 19.88           H  0.023
ATOM   1347  HB3 LEU A  83       4.229  15.110  20.695  1.00 19.88           H  0.023
ATOM   1348  HG  LEU A  83       3.934  13.610  23.067  1.00 20.82           H  0.024
ATOM   1349 HD11 LEU A  83       4.213  15.666  24.218  1.00 21.66           H  0.024
ATOM   1350 HD12 LEU A  83       5.552  15.084  23.593  1.00 21.66           H  0.024
```

```
ATOM   1351 HD13 LEU A  83       4.785  16.268  22.864  1.00 21.66           H  0.024
ATOM   1352 HD21 LEU A  83       2.148  15.041  23.524  1.00 22.81           H  0.025
ATOM   1353 HD22 LEU A  83       2.363  15.736  22.112  1.00 22.81           H  0.025
ATOM   1354 HD23 LEU A  83       1.897  14.220  22.187  1.00 22.81           H  0.025
ATOM   1355  N   LEU A  84       5.074  12.093  18.522  1.00 16.13           N  0.021
ANISOU 1355  N   LEU A  84     2005   1846   2277    351   -118    -77       N
ATOM   1356  CA  LEU A  84       5.628  12.046  17.170  1.00 16.23           C  0.021
ANISOU 1356  CA  LEU A  84     2207   1980   1978    263   -184    -61       C
ATOM   1357  C   LEU A  84       4.817  11.150  16.242  1.00 18.09           C  0.022
ANISOU 1357  C   LEU A  84     2323   2158   2392    251   -244   -292       C
ATOM   1358  O   LEU A  84       5.142  11.042  15.052  1.00 18.39           O  0.022
ANISOU 1358  O   LEU A  84     2424   2235   2330    300   -299   -365       O
ATOM   1359  CB  LEU A  84       7.058  11.525  17.229  1.00 15.77           C  0.021
ANISOU 1359  CB  LEU A  84     2251   1894   1847    399    -94   -115       C
ATOM   1360  CG  LEU A  84       8.009  12.319  18.110  1.00 16.30           C  0.021
ANISOU 1360  CG  LEU A  84     2161   1974   2057    456   -295     87       C
ATOM   1361  CD1 LEU A  84       9.413  11.776  17.996  1.00 19.17           C  0.023
ANISOU 1361  CD1 LEU A  84     2189   2145   2950    599   -227   -124       C
ATOM   1362  CD2 LEU A  84       8.054  13.815  17.792  1.00 15.96           C  0.021
ANISOU 1362  CD2 LEU A  84     2161   1967   1938    342   -330     51       C
ATOM   1363  H   LEU A  84       5.249  11.396  18.994  1.00 19.36           H  0.023
ATOM   1364  HA  LEU A  84       5.641  12.938  16.802  1.00 19.47           H  0.023
ATOM   1365  HB2 LEU A  84       7.039  10.616  17.567  1.00 18.93           H  0.023
ATOM   1366  HB3 LEU A  84       7.423  11.529  16.330  1.00 18.93           H  0.023
ATOM   1367  HG  LEU A  84       7.730  12.223  19.034  1.00 19.56           H  0.023
ATOM   1368 HD11 LEU A  84       9.973  12.204  18.663  1.00 23.01           H  0.025
ATOM   1369 HD12 LEU A  84       9.391  10.818  18.146  1.00 23.01           H  0.025
ATOM   1370 HD13 LEU A  84       9.752  11.963  17.107  1.00 23.01           H  0.025
ATOM   1371 HD21 LEU A  84       8.737  14.233  18.339  1.00 19.16           H  0.023
ATOM   1372 HD22 LEU A  84       8.266  13.931  16.853  1.00 19.16           H  0.023
ATOM   1373 HD23 LEU A  84       7.188  14.206  17.987  1.00 19.16           H  0.023
ATOM   1374  N   SER A  85       3.774  10.501  16.757  1.00 19.01           N  0.023
ANISOU 1374  N   SER A  85     2392   2055   2777    102   -356   -270       N
ATOM   1375  CA  SER A  85       2.985   9.550  15.986  1.00 20.34           C  0.024
ANISOU 1375  CA  SER A  85     2590   2060   3078     35   -459   -419       C
ATOM   1376  C   SER A  85       2.259  10.193  14.801  1.00 20.07           C  0.023
ANISOU 1376  C   SER A  85     2663   2023   2939     77   -507   -433       C
ATOM   1377  O   SER A  85       1.897  11.374  14.818  1.00 19.02           O  0.023
ANISOU 1377  O   SER A  85     2539   1884   2804    140   -508   -334       O
ATOM   1378  CB  SER A  85       1.947   8.930  16.929  1.00 22.71           C  0.025
ANISOU 1378  CB  SER A  85     2815   2165   3649   -118   -658    -91       C
ATOM   1379  OG  SER A  85       1.063   8.071  16.225  1.00 25.61           O  0.026
ANISOU 1379  OG  SER A  85     3151   2307   4274   -154   -660   -129       O
ATOM   1380  H   SER A  85       3.499  10.597  17.566  1.00 22.82           H  0.025
ATOM   1381  HA  SER A  85       3.561   8.845  15.651  1.00 24.41           H  0.026
ATOM   1382  HB2 SER A  85       2.408   8.417  17.611  1.00 27.25           H  0.027
ATOM   1383  HB3 SER A  85       1.432   9.641  17.342  1.00 27.25           H  0.027
ATOM   1384  HG  SER A  85       0.501   7.740  16.755  1.00 30.74           H  0.029
ATOM   1385  N   SER A  86       2.001   9.390  13.764  1.00 21.48           N  0.024
ANISOU 1385  N   SER A  86     2863   2168   3131    155   -439   -462       N
ATOM   1386  CA  SER A  86       1.148   9.848  12.670  1.00 23.50           C  0.025
ANISOU 1386  CA  SER A  86     3107   2301   3522    239   -771   -737       C
ATOM   1387  C   SER A  86      -0.288  10.089  13.145  1.00 23.53           C  0.025
ANISOU 1387  C   SER A  86     2995   2212   3734    293   -884   -774       C
ATOM   1388  O   SER A  86      -1.005  10.918  12.561  1.00 24.72           O  0.026
ANISOU 1388  O   SER A  86     3130   2333   3929    440  -1109   -673       O
ATOM   1389  CB  SER A  86       1.216   8.865  11.493  1.00 26.28           C  0.027
ANISOU 1389  CB  SER A  86     3528   2685   3771    203   -914   -630       C
ATOM   1390  OG  SER A  86       0.786   7.600  11.884  1.00 29.32           O  0.028
ANISOU 1390  OG  SER A  86     3768   3067   4306    188   -829   -499       O
ATOM   1391  H   SER A  86       2.303   8.590  13.673  1.00 25.78           H  0.027
ATOM   1392  HA  SER A  86       1.491  10.698  12.354  1.00 28.21           H  0.028
ATOM   1393  HB2 SER A  86       0.644   9.187  10.779  1.00 31.53           H  0.029
ATOM   1394  HB3 SER A  86       2.133   8.806  11.183  1.00 31.53           H  0.029
ATOM   1395  HG  SER A  86       1.277   7.307  12.499  1.00 35.19           H  0.031
ATOM   1396  N   ASP A  87      -0.707   9.414  14.214  1.00 22.57           N  0.025
ANISOU 1396  N   ASP A  87     2814   2105   3654    140   -779   -965       N
ATOM   1397  CA  ASP A  87      -1.996   9.660  14.845  1.00 22.80           C  0.025
ANISOU 1397  CA  ASP A  87     2676   2108   3879    -76   -920   -848       C
ATOM   1398  C   ASP A  87      -1.851  10.790  15.860  1.00 21.22           C  0.024
ANISOU 1398  C   ASP A  87     2452   2025   3584     41   -805   -664       C
ATOM   1399  O   ASP A  87      -1.012  10.723  16.760  1.00 20.50           O  0.024
ANISOU 1399  O   ASP A  87     2342   2088   3359    229   -798   -512       O
ATOM   1400  CB  ASP A  87      -2.518   8.397  15.533  1.00 25.60           C  0.026
ANISOU 1400  CB  ASP A  87     2944   2266   4516   -342   -689   -919       C
ATOM   1401  CG  ASP A  87      -3.877   8.612  16.158  1.00 27.46           C  0.027
ANISOU 1401  CG  ASP A  87     3079   2432   4924   -543   -525   -874       C
ATOM   1402  OD1 ASP A  87      -3.913   9.207  17.250  1.00 27.94           O  0.028
```

```
ANISOU 1402  OD1 ASP A  87     3111   2550   4956   -618   -258   -842       O
ATOM   1403  OD2 ASP A  87      -4.906   8.197  15.568  1.00 29.91           O  0.029
ANISOU 1403  OD2 ASP A  87     3259   2757   5347   -417   -607   -564       O
ATOM   1404  H   ASP A  87      -0.250   8.795  14.598  1.00 27.08           H  0.027
ATOM   1405  HA  ASP A  87      -2.640   9.932  14.172  1.00 27.36           H  0.027
ATOM   1406  HB2 ASP A  87      -2.596   7.686  14.877  1.00 30.72           H  0.029
ATOM   1407  HB3 ASP A  87      -1.900   8.138  16.235  1.00 30.72           H  0.029
ATOM   1408  N   ILE A  88      -2.686  11.826  15.727  1.00 20.76           N  0.024
ANISOU 1408  N   ILE A  88     2362   1989   3535    126   -752   -641       N
ATOM   1409  CA  ILE A  88      -2.504  13.058  16.504  1.00 19.24           C  0.023
ANISOU 1409  CA  ILE A  88     2216   1863   3233      9   -824   -416       C
ATOM   1410  C   ILE A  88      -3.139  13.018  17.890  1.00 18.80           C  0.023
ANISOU 1410  C   ILE A  88     2117   1720   3305      9   -679   -106       C
ATOM   1411  O   ILE A  88      -3.093  14.028  18.613  1.00 18.17           O  0.022
ANISOU 1411  O   ILE A  88     2110   1657   3139    -12   -507   -127       O
ATOM   1412  CB  ILE A  88      -2.922  14.297  15.676  1.00 19.78           C  0.023
ANISOU 1412  CB  ILE A  88     2287   2004   3226     45   -825   -503       C
ATOM   1413  CG1 ILE A  88      -4.445  14.331  15.474  1.00 20.38           C  0.024
ANISOU 1413  CG1 ILE A  88     2333   2051   3360    -75   -907   -422       C
ATOM   1414  CG2 ILE A  88      -2.143  14.368  14.352  1.00 21.43           C  0.024
ANISOU 1414  CG2 ILE A  88     2547   2245   3352     29   -732   -418       C
ATOM   1415  CD1 ILE A  88      -4.958  15.669  14.949  1.00 20.89           C  0.024
ANISOU 1415  CD1 ILE A  88     2383   2223   3330     79   -770   -256       C
ATOM   1416  H   ILE A  88      -3.363  11.841  15.196  1.00 24.91           H  0.026
ATOM   1417  HA  ILE A  88      -1.551  13.154  16.657  1.00 23.10           H  0.025
ATOM   1418  HB  ILE A  88      -2.683  15.083  16.192  1.00 23.74           H  0.025
ATOM   1419 HG12 ILE A  88      -4.691  13.646  14.832  1.00 24.46           H  0.026
ATOM   1420 HG13 ILE A  88      -4.886  14.162  16.320  1.00 24.46           H  0.026
ATOM   1421 HG21 ILE A  88      -2.365  15.196  13.899  1.00 25.72           H  0.027
ATOM   1422 HG22 ILE A  88      -1.193  14.340  14.544  1.00 25.72           H  0.027
ATOM   1423 HG23 ILE A  88      -2.391  13.611  13.799  1.00 25.72           H  0.027
ATOM   1424 HD11 ILE A  88      -5.926  15.634  14.889  1.00 25.07           H  0.026
ATOM   1425 HD12 ILE A  88      -4.689  16.372  15.561  1.00 25.07           H  0.026
ATOM   1426 HD13 ILE A  88      -4.578  15.831  14.071  1.00 25.07           H  0.026
ATOM   1427  N   THR A  89      -3.695  11.880  18.309  1.00 20.21           N  0.024
ANISOU 1427  N   THR A  89     2231   1903   3544    -83   -490    -71       N
ATOM   1428  CA  THR A  89      -4.417  11.847  19.578  1.00 20.51           C  0.024
ANISOU 1428  CA  THR A  89     2213   1889   3690   -194   -175     -7       C
ATOM   1429  C   THR A  89      -3.592  12.400  20.741  1.00 19.02           C  0.023
ANISOU 1429  C   THR A  89     2072   1782   3372   -166    -15    111       C
ATOM   1430  O   THR A  89      -4.075  13.222  21.527  1.00 18.79           O  0.023
ANISOU 1430  O   THR A  89     2023   1865   3253   -134     77    202       O
ATOM   1431  CB  THR A  89      -4.839  10.415  19.905  1.00 22.45           C  0.025
ANISOU 1431  CB  THR A  89     2401   1994   4136   -376   -218     95       C
ATOM   1432  OG1 THR A  89      -5.766   9.966  18.935  1.00 23.84           O  0.026
ANISOU 1432  OG1 THR A  89     2302   2206   4550   -479   -346     77       O
ATOM   1433  CG2 THR A  89      -5.498  10.385  21.270  1.00 23.40           C  0.025
ANISOU 1433  CG2 THR A  89     2615   1967   4310   -453   -101    391       C
ATOM   1434  H   THR A  89      -3.669  11.131  17.888  1.00 24.25           H  0.026
ATOM   1435  HA  THR A  89      -5.220  12.385  19.498  1.00 24.61           H  0.026
ATOM   1436  HB  THR A  89      -4.062   9.834  19.916  1.00 26.94           H  0.027
ATOM   1437  HG1 THR A  89      -5.415   9.984  18.172  1.00 28.61           H  0.028
ATOM   1438 HG21 THR A  89      -6.063   9.600  21.345  1.00 28.08           H  0.028
ATOM   1439 HG22 THR A  89      -4.824  10.353  21.967  1.00 28.08           H  0.028
ATOM   1440 HG23 THR A  89      -6.045  11.177  21.392  1.00 28.08           H  0.028
ATOM   1441  N   ALA A  90      -2.387  11.870  20.941  1.00 18.54           N  0.023
ANISOU 1441  N   ALA A  90     1985   1818   3240    -79    -98    227       N
ATOM   1442  CA  ALA A  90      -1.617  12.268  22.117  1.00 18.35           C  0.022
ANISOU 1442  CA  ALA A  90     2054   1935   2981     -1    -83    442       C
ATOM   1443  C   ALA A  90      -1.292  13.757  22.070  1.00 16.73           C  0.021
ANISOU 1443  C   ALA A  90     1939   1964   2455      7   -296    290       C
ATOM   1444  O   ALA A  90      -1.365  14.449  23.095  1.00 17.43           O  0.022
ANISOU 1444  O   ALA A  90     2009   2108   2506    121    -45    478       O
ATOM   1445  CB  ALA A  90      -0.347  11.422  22.248  1.00 19.56           C  0.023
ANISOU 1445  CB  ALA A  90     2172   1999   3261    122     28    457       C
ATOM   1446  H   ALA A  90      -2.003  11.296  20.429  1.00 22.25           H  0.025
ATOM   1447  HA  ALA A  90      -2.157  12.111  22.908  1.00 22.02           H  0.025
ATOM   1448  HB1 ALA A  90       0.142  11.708  23.035  1.00 23.47           H  0.025
ATOM   1449  HB2 ALA A  90      -0.597  10.489  22.334  1.00 23.47           H  0.025
ATOM   1450  HB3 ALA A  90       0.198  11.547  21.455  1.00 23.47           H  0.025
ATOM   1451  N   SER A  91      -0.903  14.272  20.900  1.00 15.25           N  0.020
ANISOU 1451  N   SER A  91     1816   1734   2246    127   -233     58       N
ATOM   1452  CA  SER A  91      -0.578  15.691  20.819  1.00 14.21           C  0.020
ANISOU 1452  CA  SER A  91     1713   1711   1975     24   -193     25       C
ATOM   1453  C   SER A  91      -1.819  16.545  21.086  1.00 14.19           C  0.020
ANISOU 1453  C   SER A  91     1621   1726   2045    -27   -120     19       C
ATOM   1454  O   SER A  91      -1.739  17.557  21.782  1.00 14.60           O  0.020
ANISOU 1454  O   SER A  91     1634   1755   2158    -32    -77     53       O
```

```
ATOM   1455  CB  SER A  91      -0.020  16.026  19.441  1.00 14.88           C  0.020
ANISOU 1455  CB  SER A  91     1741   1848   2065     13    -83   -111         C
ATOM   1456  OG  SER A  91       1.346  15.687  19.381  1.00 15.09           O  0.020
ANISOU 1456  OG  SER A  91     1778   1986   1970    140    -14   -194         O
ATOM   1457  H   SER A  91      -0.822  13.835  20.164  1.00 18.31           H  0.022
ATOM   1458  HA  SER A  91       0.093  15.908  21.484  1.00 17.05           H  0.022
ATOM   1459  HB2 SER A  91      -0.504  15.520  18.770  1.00 17.86           H  0.022
ATOM   1460  HB3 SER A  91      -0.120  16.977  19.279  1.00 17.86           H  0.022
ATOM   1461  HG  SER A  91       1.655  15.871  18.622  1.00 18.11           H  0.022
ATOM   1462  N   VAL A  92      -2.973  16.155  20.561  1.00 14.98           N  0.020
ANISOU 1462  N   VAL A  92     1627   1720   2345     37   -184    168         N
ATOM   1463  CA  VAL A  92      -4.186  16.929  20.801  1.00 15.17           C  0.020
ANISOU 1463  CA  VAL A  92     1595   1804   2365     15   -156    147         C
ATOM   1464  C   VAL A  92      -4.571  16.908  22.276  1.00 15.04           C  0.020
ANISOU 1464  C   VAL A  92     1514   1782   2418    -84    -57    182         C
ATOM   1465  O   VAL A  92      -4.874  17.951  22.866  1.00 15.61           O  0.021
ANISOU 1465  O   VAL A  92     1596   1877   2459    -15    -27    179         O
ATOM   1466  CB  VAL A  92      -5.336  16.413  19.923  1.00 16.43           C  0.021
ANISOU 1466  CB  VAL A  92     1662   1924   2658     13   -155    196         C
ATOM   1467  CG1 VAL A  92      -6.648  17.025  20.386  1.00 17.12           C  0.022
ANISOU 1467  CG1 VAL A  92     1558   2120   2827     62   -312    171         C
ATOM   1468  CG2 VAL A  92      -5.085  16.683  18.442  1.00 16.87           C  0.021
ANISOU 1468  CG2 VAL A  92     1779   2055   2575   -180   -248    122         C
ATOM   1469  H   VAL A  92      -3.082  15.458  20.070  1.00 17.98           H  0.022
ATOM   1470  HA  VAL A  92      -4.019  17.852  20.554  1.00 18.21           H  0.022
ATOM   1471  HB  VAL A  92      -5.405  15.452  20.038  1.00 19.72           H  0.023
ATOM   1472 HG11 VAL A  92      -7.288  16.980  19.659  1.00 20.55           H  0.024
ATOM   1473 HG12 VAL A  92      -6.981  16.527  21.149  1.00 20.55           H  0.024
ATOM   1474 HG13 VAL A  92      -6.496  17.951  20.631  1.00 20.55           H  0.024
ATOM   1475 HG21 VAL A  92      -5.827  16.330  17.927  1.00 20.24           H  0.024
ATOM   1476 HG22 VAL A  92      -5.012  17.640  18.304  1.00 20.24           H  0.024
ATOM   1477 HG23 VAL A  92      -4.260  16.246  18.177  1.00 20.24           H  0.024
ATOM   1478  N   ASN A  93      -4.545  15.727  22.908  1.00 16.43           N  0.021
ANISOU 1478  N   ASN A  93     1689   1939   2615   -132     35    437         N
ATOM   1479  CA  ASN A  93      -4.965  15.657  24.305  1.00 17.39           C  0.022
ANISOU 1479  CA  ASN A  93     1918   2014   2674   -161     64    548         C
ATOM   1480  C   ASN A  93      -4.051  16.482  25.195  1.00 15.68           C  0.021
ANISOU 1480  C   ASN A  93     1757   1918   2284    -67    268    388         C
ATOM   1481  O   ASN A  93      -4.498  17.127  26.153  1.00 16.29           O  0.021
ANISOU 1481  O   ASN A  93     1769   2111   2310     78    265    430         O
ATOM   1482  CB  ASN A  93      -5.016  14.199  24.764  1.00 20.15           C  0.023
ANISOU 1482  CB  ASN A  93     2235   2191   3228   -384     39    740         C
ATOM   1483  CG  ASN A  93      -6.130  13.440  24.103  1.00 24.49           C  0.026
ANISOU 1483  CG  ASN A  93     2604   2595   4105   -532     -1    655         C
ATOM   1484  OD1 ASN A  93      -6.971  14.018  23.420  1.00 25.16           O  0.026
ANISOU 1484  OD1 ASN A  93     2414   2840   4305   -711     22    448         O
ATOM   1485  ND2 ASN A  93      -6.128  12.132  24.284  1.00 27.11           N  0.027
ANISOU 1485  ND2 ASN A  93     2942   2662   4698   -822    -74    529         N
ATOM   1486  H   ASN A  93      -4.297  14.980  22.562  1.00 19.72           H  0.023
ATOM   1487  HA  ASN A  93      -5.860  16.023  24.381  1.00 20.87           H  0.024
ATOM   1488  HB2 ASN A  93      -4.179  13.765  24.538  1.00 24.18           H  0.026
ATOM   1489  HB3 ASN A  93      -5.159  14.172  25.723  1.00 24.18           H  0.026
ATOM   1490 HD21 ASN A  93      -5.518  11.766  24.767  1.00 32.54           H  0.030
ATOM   1491 HD22 ASN A  93      -6.742  11.648  23.926  1.00 32.54           H  0.030
ATOM   1492  N   CYS A  94      -2.750  16.449  24.911  1.00 14.89           N  0.020
ANISOU 1492  N   CYS A  94     1626   1818   2215    -97    172    341         N
ATOM   1493  CA  CYS A  94      -1.821  17.246  25.688  1.00 14.51           C  0.020
ANISOU 1493  CA  CYS A  94     1575   1887   2053     70    135    293         C
ATOM   1494  C   CYS A  94      -2.016  18.732  25.414  1.00 13.69           C  0.019
ANISOU 1494  C   CYS A  94     1596   1862   1745    175     60    187         C
ATOM   1495  O   CYS A  94      -2.025  19.533  26.345  1.00 14.48           O  0.020
ANISOU 1495  O   CYS A  94     1714   1896   1892    126    113    211         O
ATOM   1496  CB  CYS A  94      -0.402  16.761  25.427  1.00 14.68           C  0.020
ANISOU 1496  CB  CYS A  94     1741   1888   1949    184     55    328         C
ATOM   1497  SG  CYS A  94       0.847  17.500  26.490  1.00 15.15           S  0.020
ANISOU 1497  SG  CYS A  94     1791   1941   2025    119    110    243         S
ATOM   1498  H   CYS A  94      -2.391  15.981  24.285  1.00 17.87           H  0.022
ATOM   1499  HA  CYS A  94      -2.010  17.100  26.628  1.00 17.42           H  0.022
ATOM   1500  HB2 CYS A  94      -0.373  15.801  25.562  1.00 17.62           H  0.022
ATOM   1501  HB3 CYS A  94      -0.167  16.969  24.509  1.00 17.62           H  0.022
ATOM   1502  N   ALA A  95      -2.195  19.112  24.149  1.00 13.74           N  0.019
ANISOU 1502  N   ALA A  95     1619   1878   1724    109     72    262         N
ATOM   1503  CA  ALA A  95      -2.440  20.523  23.818  1.00 12.82           C  0.019
ANISOU 1503  CA  ALA A  95     1638   1779   1456    -11     77    221         C
ATOM   1504  C   ALA A  95      -3.680  21.055  24.514  1.00 13.52           C  0.019
ANISOU 1504  C   ALA A  95     1661   1789   1685     18    129    286         C
ATOM   1505  O   ALA A  95      -3.711  22.224  24.909  1.00 13.42           O  0.019
ANISOU 1505  O   ALA A  95     1618   1711   1772    116     45    234         O
```

```
ATOM   1506  CB  ALA A  95      -2.590  20.672  22.310  1.00 14.53           C  0.020
ANISOU 1506  CB  ALA A  95     1701   1800   2020    -32    202    141       C
ATOM   1507  H   ALA A  95      -2.180  18.584  23.471  1.00 16.49           H  0.021
ATOM   1508  HA  ALA A  95      -1.680  21.055  24.102  1.00 15.39           H  0.021
ATOM   1509  HB1 ALA A  95      -2.751  21.605  22.100  1.00 17.44           H  0.022
ATOM   1510  HB2 ALA A  95      -1.773  20.372  21.881  1.00 17.44           H  0.022
ATOM   1511  HB3 ALA A  95      -3.338  20.131  22.013  1.00 17.44           H  0.022
ATOM   1512  N   LYS A  96      -4.712  20.222  24.671  1.00 13.76           N  0.019
ANISOU 1512  N   LYS A  96     1624   1730   1874      1    235    229       N
ATOM   1513  CA  LYS A  96      -5.905  20.659  25.392  1.00 14.59           C  0.020
ANISOU 1513  CA  LYS A  96     1713   1913   1919     41    316    229       C
ATOM   1514  C   LYS A  96      -5.583  21.008  26.848  1.00 14.87           C  0.020
ANISOU 1514  C   LYS A  96     1763   1981   1906    122    269    284       C
ATOM   1515  O   LYS A  96      -6.104  21.983  27.392  1.00 15.38           O  0.021
ANISOU 1515  O   LYS A  96     1824   2028   1993    256    280    293       O
ATOM   1516  CB  LYS A  96      -7.013  19.597  25.296  1.00 15.53           C  0.021
ANISOU 1516  CB  LYS A  96     1590   2008   2305    -12     86    410       C
ATOM   1517  CG  LYS A  96      -7.668  19.483  23.922  1.00 16.61           C  0.021
ANISOU 1517  CG  LYS A  96     1533   2113   2667   -118    146    341       C
ATOM   1518  CD  LYS A  96      -8.687  18.353  23.862  1.00 17.66           C  0.022
ANISOU 1518  CD  LYS A  96     1535   2361   2814   -195   -145    235       C
ATOM   1519  CE  LYS A  96      -9.410  18.352  22.546  1.00 19.15           C  0.023
ANISOU 1519  CE  LYS A  96     1737   2509   3031   -316   -151    220       C
ATOM   1520  NZ  LYS A  96     -10.392  17.207  22.443  1.00 21.07           N  0.024
ANISOU 1520  NZ  LYS A  96     1850   2626   3529   -463   -209    254       N
ATOM   1521  H   LYS A  96      -4.745  19.415  24.375  1.00 16.51           H  0.021
ATOM   1522  HA  LYS A  96      -6.239  21.465  24.969  1.00 17.51           H  0.022
ATOM   1523  HB2 LYS A  96      -6.632  18.732  25.512  1.00 18.64           H  0.023
ATOM   1524  HB3 LYS A  96      -7.708  19.816  25.936  1.00 18.64           H  0.023
ATOM   1525  HG2 LYS A  96      -8.126  20.313  23.719  1.00 19.94           H  0.023
ATOM   1526  HG3 LYS A  96      -6.984  19.308  23.257  1.00 19.94           H  0.023
ATOM   1527  HD2 LYS A  96      -8.231  17.503  23.961  1.00 21.19           H  0.024
ATOM   1528  HD3 LYS A  96      -9.340  18.470  24.570  1.00 21.19           H  0.024
ATOM   1529  HE2 LYS A  96      -9.902  19.182  22.452  1.00 22.98           H  0.025
ATOM   1530  HE3 LYS A  96      -8.764  18.263  21.828  1.00 22.98           H  0.025
ATOM   1531  HZ1 LYS A  96     -11.000  17.269  23.090  1.00 25.28           H  0.026
ATOM   1532  HZ2 LYS A  96     -10.805  17.231  21.655  1.00 25.28           H  0.026
ATOM   1533  HZ3 LYS A  96      -9.963  16.431  22.522  1.00 25.28           H  0.026
ATOM   1534  N  ALYS A  97      -4.711  20.228  27.488  0.53 14.65           N  0.020
ANISOU 1534  N  ALYS A  97     1893   1868   1805    194    214    394       N
ATOM   1535  CA ALYS A  97      -4.302  20.566  28.848  0.53 14.94           C  0.020
ANISOU 1535  CA ALYS A  97     1956   1973   1746    310    226    444       C
ATOM   1536  C  ALYS A  97      -3.480  21.852  28.861  0.53 14.63           C  0.020
ANISOU 1536  C  ALYS A  97     1845   2054   1659    334    193    340       C
ATOM   1537  O  ALYS A  97      -3.697  22.725  29.706  0.53 15.27           O  0.020
ANISOU 1537  O  ALYS A  97     1941   2183   1676    318    243    364       O
ATOM   1538  CB ALYS A  97      -3.515  19.403  29.443  0.53 16.27           C  0.021
ANISOU 1538  CB ALYS A  97     2110   2105   1968    427    135    509       C
ATOM   1539  CG ALYS A  97      -2.986  19.693  30.837  0.53 17.97           C  0.022
ANISOU 1539  CG ALYS A  97     2225   2330   2273    481    -41    551       C
ATOM   1540  CD ALYS A  97      -2.591  18.428  31.619  0.53 19.96           C  0.023
ANISOU 1540  CD ALYS A  97     2285   2485   2813    428    -46    486       C
ATOM   1541  CE ALYS A  97      -2.246  18.777  33.067  0.53 22.57           C  0.025
ANISOU 1541  CE ALYS A  97     2485   2701   3389    321    162    317       C
ATOM   1542  NZ ALYS A  97      -2.118  17.601  33.972  0.53 24.18           N  0.026
ANISOU 1542  NZ ALYS A  97     2535   2873   3779    290    288    188       N
ATOM   1543  H  ALYS A  97      -4.351  19.516  27.166  0.53 17.58           H  0.022
ATOM   1544  HA ALYS A  97      -5.092  20.707  29.393  0.53 17.93           H  0.022
ATOM   1545  HB2ALYS A  97      -4.094  18.627  29.499  0.53 19.53           H  0.023
ATOM   1546  HB3ALYS A  97      -2.757  19.210  28.870  0.53 19.53           H  0.023
ATOM   1547  HG2ALYS A  97      -2.198  20.254  30.763  0.53 21.56           H  0.024
ATOM   1548  HG3ALYS A  97      -3.673  20.154  31.343  0.53 21.56           H  0.024
ATOM   1549  HD2ALYS A  97      -3.334  17.805  31.622  0.53 23.95           H  0.026
ATOM   1550  HD3ALYS A  97      -1.812  18.024  31.207  0.53 23.95           H  0.026
ATOM   1551  HE2ALYS A  97      -1.396  19.242  33.074  0.53 27.09           H  0.027
ATOM   1552  HE3ALYS A  97      -2.940  19.353  33.425  0.53 27.09           H  0.027
ATOM   1553  HZ1ALYS A  97      -1.917  17.872  34.796  0.53 29.02           H  0.028
ATOM   1554  HZ2ALYS A  97      -2.885  17.150  33.995  0.53 29.02           H  0.028
ATOM   1555  HZ3ALYS A  97      -1.474  17.061  33.680  0.53 29.02           H  0.028
ATOM   1556  N  BLYS A  97      -4.717  20.219  27.493  0.47 15.40           N  0.021
ANISOU 1556  N  BLYS A  97     1924   1920   2009    145    208    386       N
ATOM   1557  CA BLYS A  97      -4.277  20.557  28.846  0.47 16.29           C  0.021
ANISOU 1557  CA BLYS A  97     2022   2029   2139    208    206    439       C
ATOM   1558  C  BLYS A  97      -3.489  21.860  28.850  0.47 15.42           C  0.021
ANISOU 1558  C  BLYS A  97     1876   2075   1907    277    177    350       C
ATOM   1559  O  BLYS A  97      -3.718  22.742  29.684  0.47 16.08           O  0.021
ANISOU 1559  O  BLYS A  97     1953   2189   1970    272    204    369       O
ATOM   1560  CB BLYS A  97      -3.427  19.419  29.413  0.47 18.65           C  0.023
```

```
ANISOU 1560  CB BLYS A  97     2269   2164   2652    229    150    483       C
ATOM   1561  CG BLYS A  97      -4.137  18.091  29.605  0.47 21.31           C  0.024
ANISOU 1561  CG BLYS A  97     2458   2353   3286    197     69    519       C
ATOM   1562  CD BLYS A  97      -3.409  17.212  30.645  0.47 23.86           C  0.026
ANISOU 1562  CD BLYS A  97     2619   2528   3920    169     84    542       C
ATOM   1563  CE BLYS A  97      -3.272  15.758  30.189  0.47 26.42           C  0.027
ANISOU 1563  CE BLYS A  97     2821   2757   4458    146    -96    518       C
ATOM   1564  NZ BLYS A  97      -2.863  14.884  31.325  0.47 27.60           N  0.027
ANISOU 1564  NZ BLYS A  97     2896   2853   4740    143   -142    438       N
ATOM   1565  H  BLYS A  97      -4.376  19.496  27.174  0.47 18.49           H  0.022
ATOM   1566  HA BLYS A  97      -5.054  20.670  29.416  0.47 19.55           H  0.023
ATOM   1567  HB2BLYS A  97      -2.682  19.265  28.811  0.47 22.38           H  0.025
ATOM   1568  HB3BLYS A  97      -3.089  19.695  30.279  0.47 22.38           H  0.025
ATOM   1569  HG2BLYS A  97      -5.040  18.253  29.921  0.47 25.57           H  0.026
ATOM   1570  HG3BLYS A  97      -4.156  17.612  28.761  0.47 25.57           H  0.026
ATOM   1571  HD2BLYS A  97      -2.518  17.565  30.792  0.47 28.64           H  0.028
ATOM   1572  HD3BLYS A  97      -3.911  17.221  31.475  0.47 28.64           H  0.028
ATOM   1573  HE2BLYS A  97      -4.126  15.444  29.853  0.47 31.70           H  0.029
ATOM   1574  HE3BLYS A  97      -2.595  15.701  29.497  0.47 31.70           H  0.029
ATOM   1575  HZ1BLYS A  97      -2.684  14.064  31.029  0.47 33.13           H  0.030
ATOM   1576  HZ2BLYS A  97      -2.136  15.215  31.718  0.47 33.13           H  0.030
ATOM   1577  HZ3BLYS A  97      -3.519  14.839  31.925  0.47 33.13           H  0.030
ATOM   1578  N   ILE A  98      -2.534  21.988  27.929  1.00 14.31           N  0.020
ANISOU 1578  N   ILE A  98     1716   1998   1724    261    161    346       N
ATOM   1579  CA  ILE A  98      -1.680  23.178  27.880  1.00 14.22           C  0.020
ANISOU 1579  CA  ILE A  98     1701   1975   1729    198     42    335       C
ATOM   1580  C   ILE A  98      -2.500  24.448  27.726  1.00 13.53           C  0.019
ANISOU 1580  C   ILE A  98     1696   1869   1577    171    168    198       C
ATOM   1581  O   ILE A  98      -2.296  25.427  28.447  1.00 14.62           O  0.020
ANISOU 1581  O   ILE A  98     1834   1944   1777    193    -43    212       O
ATOM   1582  CB  ILE A  98      -0.644  23.035  26.752  1.00 13.32           C  0.019
ANISOU 1582  CB  ILE A  98     1584   1862   1615    152     80    330       C
ATOM   1583  CG1 ILE A  98       0.321  21.876  27.054  1.00 14.11           C  0.020
ANISOU 1583  CG1 ILE A  98     1687   1855   1819    269     34    370       C
ATOM   1584  CG2 ILE A  98       0.104  24.344  26.561  1.00 13.49           C  0.019
ANISOU 1584  CG2 ILE A  98     1639   1814   1672      3     60     68       C
ATOM   1585  CD1 ILE A  98       1.214  21.481  25.897  1.00 14.43           C  0.020
ANISOU 1585  CD1 ILE A  98     1816   1890   1775    269    196    340       C
ATOM   1586  H   ILE A  98      -2.364  21.405  27.320  1.00 17.18           H  0.022
ATOM   1587  HA  ILE A  98      -1.194  23.244  28.717  1.00 17.07           H  0.022
ATOM   1588  HB  ILE A  98      -1.116  22.831  25.929  1.00 15.98           H  0.021
ATOM   1589 HG12 ILE A  98       0.888  22.143  27.786  1.00 16.93           H  0.022
ATOM   1590 HG13 ILE A  98      -0.195  21.095  27.307  1.00 16.93           H  0.022
ATOM   1591 HG21 ILE A  98       0.915  24.177  26.056  1.00 16.19           H  0.021
ATOM   1592 HG22 ILE A  98      -0.461  24.966  26.076  1.00 16.19           H  0.021
ATOM   1593 HG23 ILE A  98       0.328  24.708  27.432  1.00 16.19           H  0.021
ATOM   1594 HD11 ILE A  98       1.663  20.649  26.115  1.00 17.31           H  0.022
ATOM   1595 HD12 ILE A  98       0.668  21.364  25.104  1.00 17.31           H  0.022
ATOM   1596 HD13 ILE A  98       1.869  22.181  25.748  1.00 17.31           H  0.022
ATOM   1597  N   VAL A  99      -3.417  24.466  26.755  1.00 12.68           N  0.019
ANISOU 1597  N   VAL A  99     1632   1700   1485    133     39    182       N
ATOM   1598  CA  VAL A  99      -4.178  25.674  26.435  1.00 13.17           C  0.019
ANISOU 1598  CA  VAL A  99     1682   1772   1548    157    305     72       C
ATOM   1599  C   VAL A  99      -5.160  26.045  27.539  1.00 13.87           C  0.019
ANISOU 1599  C   VAL A  99     1867   1986   1417    295    380    116       C
ATOM   1600  O   VAL A  99      -5.644  27.177  27.562  1.00 15.04           O  0.020
ANISOU 1600  O   VAL A  99     1987   2117   1613    453    388    139       O
ATOM   1601  CB  VAL A  99      -4.888  25.509  25.077  1.00 12.65           C  0.019
ANISOU 1601  CB  VAL A  99     1632   1757   1416    120    206    150       C
ATOM   1602  CG1 VAL A  99      -6.101  24.611  25.189  1.00 13.32           C  0.019
ANISOU 1602  CG1 VAL A  99     1606   1870   1585    113    182    375       C
ATOM   1603  CG2 VAL A  99      -5.272  26.863  24.475  1.00 13.16           C  0.019
ANISOU 1603  CG2 VAL A  99     1667   1761   1572    148    126    275       C
ATOM   1604  H   VAL A  99      -3.617  23.788  26.266  1.00 15.21           H  0.020
ATOM   1605  HA  VAL A  99      -3.554  26.411  26.346  1.00 15.80           H  0.021
ATOM   1606  HB  VAL A  99      -4.271  25.084  24.461  1.00 15.18           H  0.020
ATOM   1607 HG11 VAL A  99      -6.378  24.344  24.299  1.00 15.99           H  0.021
ATOM   1608 HG12 VAL A  99      -5.866  23.828  25.711  1.00 15.99           H  0.021
ATOM   1609 HG13 VAL A  99      -6.817  25.097  25.626  1.00 15.99           H  0.021
ATOM   1610 HG21 VAL A  99      -5.701  26.716  23.618  1.00 15.79           H  0.021
ATOM   1611 HG22 VAL A  99      -5.883  27.315  25.078  1.00 15.79           H  0.021
ATOM   1612 HG23 VAL A  99      -4.469  27.394  24.356  1.00 15.79           H  0.021
ATOM   1613  N   SER A 100      -5.445  25.129  28.453  1.00 14.68           N  0.020
ANISOU 1613  N   SER A 100     2006   2169   1403    334    521    334       N
ATOM   1614  CA  SER A 100      -6.373  25.333  29.551  1.00 16.45           C  0.021
ANISOU 1614  CA  SER A 100     2241   2458   1551    228    583    363       C
ATOM   1615  C   SER A 100      -5.707  25.851  30.804  1.00 18.13           C  0.022
ANISOU 1615  C   SER A 100     2760   2430   1697    275    572    142       C
```

```
ATOM   1616  O   SER A 100      -6.399  26.097  31.787  1.00 20.02           O  0.023
ANISOU 1616  O   SER A 100     3154   2616   1838    149    704     66       O
ATOM   1617  CB  SER A 100      -7.069  24.009  29.874  1.00 17.74           C  0.022
ANISOU 1617  CB  SER A 100     2036   2758   1945    109    633    398       C
ATOM   1618  OG  SER A 100      -7.849  23.595  28.764  1.00 18.93           O  0.023
ANISOU 1618  OG  SER A 100     1979   3013   2199    166    535    194       O
ATOM   1619  H   SER A 100      -5.096  24.343  28.456  1.00 17.62           H  0.022
ATOM   1620  HA  SER A 100      -7.049  25.974  29.281  1.00 19.74           H  0.023
ATOM   1621  HB2 SER A 100      -6.399  23.334  30.063  1.00 21.29           H  0.024
ATOM   1622  HB3 SER A 100      -7.648  24.132  30.643  1.00 21.29           H  0.024
ATOM   1623  HG  SER A 100      -7.357  23.488  28.091  1.00 22.71           H  0.025
ATOM   1624  N   ASP A 101      -4.381  25.973  30.803  1.00 18.38           N  0.022
ANISOU 1624  N   ASP A 101     2905   2446   1631    213    158    409       N
ATOM   1625  CA  ASP A 101      -3.604  26.232  32.043  1.00 20.21           C  0.024
ANISOU 1625  CA  ASP A 101     3033   2756   1892    299   -172    530       C
ATOM   1626  C   ASP A 101      -3.402  27.700  32.393  1.00 20.27           C  0.024
ANISOU 1626  C   ASP A 101     3055   2809   1838    378      9    320       C
ATOM   1627  O   ASP A 101      -2.671  27.934  33.358  1.00 22.10           O  0.025
ANISOU 1627  O   ASP A 101     3172   2958   2267    455   -301    294       O
ATOM   1628  CB  ASP A 101      -2.258  25.505  31.998  1.00 23.34           C  0.025
ANISOU 1628  CB  ASP A 101     3082   3006   2782    263   -289    510       C
ATOM   1629  CG  ASP A 101      -1.841  24.977  33.366  1.00 25.33           C  0.026
ANISOU 1629  CG  ASP A 101     3077   3313   3233     68    105    781       C
ATOM   1630  OD1 ASP A 101      -2.714  24.481  34.094  1.00 29.25           O  0.028
ANISOU 1630  OD1 ASP A 101     3279   3664   4172    -50    -46    886       O
ATOM   1631  OD2 ASP A 101      -0.662  25.092  33.709  1.00 24.46           O  0.026
ANISOU 1631  OD2 ASP A 101     2798   3371   3124    305      7    495       O
ATOM   1632  H   ASP A 101      -3.892  25.912  30.098  1.00 22.05           H  0.025
ATOM   1633  HA  ASP A 101      -4.105  25.846  32.778  1.00 24.26           H  0.026
ATOM   1634  HB2 ASP A 101      -2.323  24.752  31.390  1.00 28.02           H  0.028
ATOM   1635  HB3 ASP A 101      -1.575  26.122  31.694  1.00 28.02           H  0.028
ATOM   1636  N   GLY A 102      -3.959  28.653  31.652  1.00 18.96           N  0.023
ANISOU 1636  N   GLY A 102     3025   2623   1555    315    346    465       N
ATOM   1637  CA  GLY A 102      -3.956  30.027  32.125  1.00 18.44           C  0.022
ANISOU 1637  CA  GLY A 102     2951   2628   1426    357    255    102       C
ATOM   1638  C   GLY A 102      -3.681  31.074  31.064  1.00 16.84           C  0.021
ANISOU 1638  C   GLY A 102     2663   2458   1279    463    157    110       C
ATOM   1639  O   GLY A 102      -4.290  32.157  31.063  1.00 17.81           O  0.022
ANISOU 1639  O   GLY A 102     2709   2591   1466    554    371     21       O
ATOM   1640  H   GLY A 102      -4.337  28.532  30.889  1.00 22.75           H  0.025
ATOM   1641  HA2 GLY A 102      -4.820  30.224  32.519  1.00 22.12           H  0.025
ATOM   1642  HA3 GLY A 102      -3.284  30.126  32.817  1.00 22.12           H  0.025
ATOM   1643  N   ASN A 103      -2.779  30.758  30.141  1.00 15.37           N  0.021
ANISOU 1643  N   ASN A 103     2428   2294   1117    445    122    179       N
ATOM   1644  CA  ASN A 103      -2.340  31.750  29.164  1.00 14.73           C  0.020
ANISOU 1644  CA  ASN A 103     2237   2181   1180    391    -41    155       C
ATOM   1645  C   ASN A 103      -2.846  31.403  27.760  1.00 13.38           C  0.019
ANISOU 1645  C   ASN A 103     2011   1911   1161    338    146     67       C
ATOM   1646  O   ASN A 103      -2.423  32.011  26.763  1.00 13.10           O  0.019
ANISOU 1646  O   ASN A 103     2029   1818   1130    197    155     34       O
ATOM   1647  CB  ASN A 103      -0.821  31.876  29.175  1.00 16.70           C  0.021
ANISOU 1647  CB  ASN A 103     2327   2445   1572    239   -317     71       C
ATOM   1648  CG  ASN A 103      -0.325  32.467  30.497  1.00 18.06           C  0.022
ANISOU 1648  CG  ASN A 103     2577   2639   1645    449   -315   -158       C
ATOM   1649  OD1 ASN A 103      -0.937  33.330  31.097  1.00 19.93           O  0.023
ANISOU 1649  OD1 ASN A 103     2872   2780   1922    616   -596   -288       O
ATOM   1650  ND2 ASN A 103       0.797  31.999  30.930  1.00 20.32           N  0.024
ANISOU 1650  ND2 ASN A 103     2673   2901   2145    490   -421   -143       N
ATOM   1651  H   ASN A 103      -2.409  29.986  30.058  1.00 18.44           H  0.022
ATOM   1652  HA  ASN A 103      -2.706  32.615  29.403  1.00 17.68           H  0.022
ATOM   1653  HB2 ASN A 103      -0.425  30.997  29.066  1.00 20.04           H  0.023
ATOM   1654  HB3 ASN A 103      -0.542  32.463  28.455  1.00 20.04           H  0.023
ATOM   1655 HD21 ASN A 103       1.210  31.390  30.484  1.00 24.38           H  0.026
ATOM   1656 HD22 ASN A 103       1.129  32.292  31.667  1.00 24.38           H  0.026
ATOM   1657  N   GLY A 104      -3.783  30.468  27.663  1.00 12.97           N  0.019
ANISOU 1657  N   GLY A 104     1892   1762   1273    242    154    163       N
ATOM   1658  CA  GLY A 104      -4.346  30.153  26.363  1.00 12.27           C  0.018
ANISOU 1658  CA  GLY A 104     1690   1679   1294    236     76    118       C
ATOM   1659  C   GLY A 104      -3.268  29.740  25.377  1.00 11.30           C  0.018
ANISOU 1659  C   GLY A 104     1467   1531   1296    261    136    128       C
ATOM   1660  O   GLY A 104      -2.298  29.062  25.737  1.00 12.20           O  0.018
ANISOU 1660  O   GLY A 104     1678   1692   1264    252    -52     80       O
ATOM   1661  H   GLY A 104      -4.104  30.012  28.317  1.00 15.56           H  0.021
ATOM   1662  HA2 GLY A 104      -4.983  29.426  26.450  1.00 14.73           H  0.020
ATOM   1663  HA3 GLY A 104      -4.807  30.930  26.011  1.00 14.73           H  0.020
ATOM   1664  N   MET A 105      -3.436  30.115  24.105  1.00 11.32           N  0.018
ANISOU 1664  N   MET A 105     1394   1631   1275    250     64    135       N
ATOM   1665  CA  MET A 105      -2.497  29.685  23.070  1.00 10.61           C  0.017
```

```
ANISOU 1665  CA  MET A 105     1340   1535   1155     85     73    -37       C
ATOM   1666  C   MET A 105      -1.187  30.458  23.120  1.00 10.96           C  0.017
ANISOU 1666  C   MET A 105     1451   1525   1189    148    104    -89       C
ATOM   1667  O   MET A 105      -0.258  30.127  22.389  1.00 11.00           O  0.017
ANISOU 1667  O   MET A 105     1416   1588   1175     74    125     -0       O
ATOM   1668  CB  MET A 105      -3.171  29.792  21.702  1.00 11.51           C  0.018
ANISOU 1668  CB  MET A 105     1463   1668   1244     33     79     21       C
ATOM   1669  CG  MET A 105      -4.131  28.588  21.466  1.00 11.13           C  0.017
ANISOU 1669  CG  MET A 105     1389   1643   1199    131    110     63       C
ATOM   1670  SD  MET A 105      -4.594  28.344  19.740  1.00 12.05           S  0.018
ANISOU 1670  SD  MET A 105     1578   1680   1319     91     91     38       S
ATOM   1671  CE  MET A 105      -3.189  27.575  19.140  1.00 12.38           C  0.018
ANISOU 1671  CE  MET A 105     1794   1650   1259     10    184     10       C
ATOM   1672  H   MET A 105      -4.077  30.612  23.821  1.00 13.58           H  0.019
ATOM   1673  HA  MET A 105      -2.295  28.748  23.218  1.00 12.73           H  0.019
ATOM   1674  HB2 MET A 105      -3.688  30.611  21.659  1.00 13.82           H  0.019
ATOM   1675  HB3 MET A 105      -2.493  29.782  21.008  1.00 13.82           H  0.019
ATOM   1676  HG2 MET A 105      -3.697  27.776  21.771  1.00 13.36           H  0.019
ATOM   1677  HG3 MET A 105      -4.945  28.735  21.972  1.00 13.36           H  0.019
ATOM   1678  HE1 MET A 105      -3.313  27.383  18.197  1.00 14.85           H  0.020
ATOM   1679  HE2 MET A 105      -2.430  28.167  19.260  1.00 14.85           H  0.020
ATOM   1680  HE3 MET A 105      -3.047  26.750  19.631  1.00 14.85           H  0.020
ATOM   1681  N   ASN A 106      -1.062  31.446  24.019  1.00 11.46           N  0.018
ANISOU 1681  N   ASN A 106     1398   1621   1334     82    110    -41       N
ATOM   1682  CA  ASN A 106       0.227  32.106  24.195  1.00 11.61           C  0.018
ANISOU 1682  CA  ASN A 106     1491   1677   1245     66    -92      7       C
ATOM   1683  C   ASN A 106       1.283  31.118  24.695  1.00 12.25           C  0.018
ANISOU 1683  C   ASN A 106     1544   1838   1273    123     15      7       C
ATOM   1684  O   ASN A 106       2.471  31.422  24.606  1.00 14.15           O  0.020
ANISOU 1684  O   ASN A 106     1456   1940   1981     81    -86    -12       O
ATOM   1685  CB  ASN A 106       0.122  33.309  25.131  1.00 12.67           C  0.019
ANISOU 1685  CB  ASN A 106     1593   1712   1509     -3   -101     27       C
ATOM   1686  CG  ASN A 106      -0.770  34.389  24.602  1.00 12.26           C  0.018
ANISOU 1686  CG  ASN A 106     1644   1702   1311      6     -7    -33       C
ATOM   1687  OD1 ASN A 106      -0.465  35.053  23.609  1.00 12.62           O  0.019
ANISOU 1687  OD1 ASN A 106     1806   1726   1264    -15     61     -7       O
ATOM   1688  ND2 ASN A 106      -1.929  34.540  25.221  1.00 13.41           N  0.019
ANISOU 1688  ND2 ASN A 106     1836   1788   1469    257    121    136       N
ATOM   1689  H   ASN A 106      -1.692  31.743  24.523  1.00 13.75           H  0.019
ATOM   1690  HA  ASN A 106       0.523  32.435  23.332  1.00 13.94           H  0.020
ATOM   1691  HB2 ASN A 106      -0.236  33.015  25.983  1.00 15.21           H  0.020
ATOM   1692  HB3 ASN A 106       1.006  33.687  25.257  1.00 15.21           H  0.020
ATOM   1693 HD21 ASN A 106      -2.483  35.142  24.955  1.00 16.09           H  0.021
ATOM   1694 HD22 ASN A 106      -2.118  34.054  25.905  1.00 16.09           H  0.021
ATOM   1695  N   ALA A 107       0.883  29.956  25.217  1.00 12.64           N  0.019
ANISOU 1695  N   ALA A 107     1708   1812   1285    286    -10    136       N
ATOM   1696  CA  ALA A 107       1.838  28.909  25.548  1.00 13.60           C  0.019
ANISOU 1696  CA  ALA A 107     1843   1927   1397    332     29    249       C
ATOM   1697  C   ALA A 107       2.680  28.504  24.350  1.00 13.76           C  0.019
ANISOU 1697  C   ALA A 107     1763   1905   1563    441     27    272       C
ATOM   1698  O   ALA A 107       3.764  27.938  24.537  1.00 16.29           O  0.021
ANISOU 1698  O   ALA A 107     1986   2472   1729    589      4    159       O
ATOM   1699  CB  ALA A 107       1.104  27.682  26.065  1.00 14.58           C  0.020
ANISOU 1699  CB  ALA A 107     2131   2051   1359    328    141    317       C
ATOM   1700  H   ALA A 107       0.065  29.754  25.388  1.00 15.17           H  0.020
ATOM   1701  HA  ALA A 107       2.433  29.226  26.246  1.00 16.32           H  0.021
ATOM   1702  HB1 ALA A 107       1.753  26.994  26.281  1.00 17.50           H  0.022
ATOM   1703  HB2 ALA A 107       0.603  27.926  26.859  1.00 17.50           H  0.022
ATOM   1704  HB3 ALA A 107       0.500  27.363  25.377  1.00 17.50           H  0.022
ATOM   1705  N   TRP A 108       2.204  28.716  23.130  1.00 11.86           N  0.018
ANISOU 1705  N   TRP A 108     1552   1736   1216    239     60    102       N
ATOM   1706  CA  TRP A 108       2.986  28.459  21.921  1.00 12.73           C  0.019
ANISOU 1706  CA  TRP A 108     1552   1888   1396    298    171    282       C
ATOM   1707  C   TRP A 108       3.517  29.798  21.426  1.00 13.08           C  0.019
ANISOU 1707  C   TRP A 108     1520   1987   1463    167     16    347       C
ATOM   1708  O   TRP A 108       2.761  30.624  20.885  1.00 12.81           O  0.019
ANISOU 1708  O   TRP A 108     1556   1826   1487     87    -37    388       O
ATOM   1709  CB  TRP A 108       2.141  27.774  20.848  1.00 12.47           C  0.018
ANISOU 1709  CB  TRP A 108     1637   1815   1287    310    206    192       C
ATOM   1710  CG  TRP A 108       1.830  26.343  21.201  1.00 12.10           C  0.018
ANISOU 1710  CG  TRP A 108     1613   1668   1315    257    186    -15       C
ATOM   1711  CD1 TRP A 108       2.625  25.286  20.990  1.00 13.17           C  0.019
ANISOU 1711  CD1 TRP A 108     1678   1897   1430    306    400     -2       C
ATOM   1712  CD2 TRP A 108       0.666  25.843  21.874  1.00 11.53           C  0.018
ANISOU 1712  CD2 TRP A 108     1630   1586   1165    189    145     32       C
ATOM   1713  NE1 TRP A 108       2.046  24.141  21.495  1.00 13.45           N  0.019
ANISOU 1713  NE1 TRP A 108     1759   1828   1523    340    243    161       N
ATOM   1714  CE2 TRP A 108       0.824  24.468  22.029  1.00 11.90           C  0.018
```

```
ANISOU 1714  CE2 TRP A 108     1622   1617   1283    186      5    -17       C
ATOM   1715  CE3 TRP A 108      -0.497  26.446  22.376  1.00 12.11           C  0.018
ANISOU 1715  CE3 TRP A 108     1711   1459   1429    136    146     27       C
ATOM   1716  CZ2 TRP A 108      -0.132  23.690  22.665  1.00 12.69           C  0.019
ANISOU 1716  CZ2 TRP A 108     1625   1570   1627    214     -4     43       C
ATOM   1717  CZ3 TRP A 108      -1.457  25.672  22.966  1.00 12.53           C  0.019
ANISOU 1717  CZ3 TRP A 108     1686   1563   1513     93     80     52       C
ATOM   1718  CH2 TRP A 108      -1.266  24.312  23.130  1.00 12.37           C  0.018
ANISOU 1718  CH2 TRP A 108     1571   1607   1521    188    154    113       C
ATOM   1719  H   TRP A 108       1.414  29.015  22.970  1.00 14.23           H  0.020
ATOM   1720  HA  TRP A 108       3.740  27.886  22.132  1.00 15.28           H  0.020
ATOM   1721  HB2 TRP A 108       1.302  28.251  20.751  1.00 14.97           H  0.020
ATOM   1722  HB3 TRP A 108       2.627  27.779  20.009  1.00 14.97           H  0.020
ATOM   1723  HD1 TRP A 108       3.458  25.322  20.579  1.00 15.81           H  0.021
ATOM   1724  HE1 TRP A 108       2.380  23.350  21.453  1.00 16.14           H  0.021
ATOM   1725  HE3 TRP A 108      -0.627  27.362  22.281  1.00 14.53           H  0.020
ATOM   1726  HZ2 TRP A 108      -0.020  22.771  22.753  1.00 15.23           H  0.020
ATOM   1727  HZ3 TRP A 108      -2.228  26.071  23.300  1.00 15.04           H  0.020
ATOM   1728  HH2 TRP A 108      -1.931  23.804  23.535  1.00 14.84           H  0.020
ATOM   1729  N   VAL A 109       4.816  30.026  21.601  1.00 15.93           N  0.021
ANISOU 1729  N   VAL A 109     1470   2493   2091   -114   -179    927       N
ATOM   1730  CA  VAL A 109       5.372  31.329  21.245  1.00 17.67           C  0.022
ANISOU 1730  CA  VAL A 109     1682   2807   2227   -217   -416   1077       C
ATOM   1731  C   VAL A 109       5.170  31.627  19.772  1.00 16.38           C  0.021
ANISOU 1731  C   VAL A 109     1471   2579   2175   -215   -308    967       C
ATOM   1732  O   VAL A 109       4.884  32.766  19.393  1.00 16.22           O  0.021
ANISOU 1732  O   VAL A 109     1663   2552   1948   -355   -416    833       O
ATOM   1733  CB  VAL A 109       6.862  31.376  21.645  1.00 20.59           C  0.024
ANISOU 1733  CB  VAL A 109     1955   3208   2661   -572   -745   1271       C
ATOM   1734  CG1 VAL A 109       7.522  32.619  21.117  1.00 22.93           C  0.025
ANISOU 1734  CG1 VAL A 109     2110   3429   3174   -690   -908   1196       C
ATOM   1735  CG2 VAL A 109       6.999  31.316  23.159  1.00 23.05           C  0.025
ANISOU 1735  CG2 VAL A 109     2217   3389   3151   -601   -862   1258       C
ATOM   1736  H   VAL A 109       5.383  29.461  21.915  1.00 19.12           H  0.023
ATOM   1737  HA  VAL A 109       4.908  32.014  21.751  1.00 21.21           H  0.024
ATOM   1738  HB  VAL A 109       7.318  30.607  21.269  1.00 24.71           H  0.026
ATOM   1739 HG11 VAL A 109       8.362  32.751  21.584  1.00 27.52           H  0.027
ATOM   1740 HG12 VAL A 109       7.687  32.513  20.167  1.00 27.52           H  0.027
ATOM   1741 HG13 VAL A 109       6.937  33.377  21.272  1.00 27.52           H  0.027
ATOM   1742 HG21 VAL A 109       7.940  31.352  23.392  1.00 27.66           H  0.028
ATOM   1743 HG22 VAL A 109       6.532  32.072  23.548  1.00 27.66           H  0.028
ATOM   1744 HG23 VAL A 109       6.611  30.487  23.479  1.00 27.66           H  0.028
ATOM   1745  N   ALA A 110       5.281  30.610  18.922  1.00 15.48           N  0.021
ANISOU 1745  N   ALA A 110     1444   2330   2107    121    262   1077       N
ATOM   1746  CA  ALA A 110       5.075  30.837  17.501  1.00 14.72           C  0.020
ANISOU 1746  CA  ALA A 110     1705   2160   1727    283    326    722       C
ATOM   1747  C   ALA A 110       3.639  31.231  17.186  1.00 13.76           C  0.019
ANISOU 1747  C   ALA A 110     1731   1995   1501    125    158    490       C
ATOM   1748  O   ALA A 110       3.414  31.977  16.227  1.00 13.37           O  0.019
ANISOU 1748  O   ALA A 110     1790   1896   1394     96    110    384       O
ATOM   1749  CB  ALA A 110       5.489  29.632  16.658  1.00 17.32           C  0.022
ANISOU 1749  CB  ALA A 110     1912   2247   2422    209    446    677       C
ATOM   1750  H   ALA A 110       5.470  29.799  19.138  1.00 18.57           H  0.023
ATOM   1751  HA  ALA A 110       5.639  31.579  17.232  1.00 17.66           H  0.022
ATOM   1752  HB1 ALA A 110       5.333  29.833  15.722  1.00 20.79           H  0.024
ATOM   1753  HB2 ALA A 110       6.431  29.453  16.805  1.00 20.79           H  0.024
ATOM   1754  HB3 ALA A 110       4.960  28.864  16.925  1.00 20.79           H  0.024
ATOM   1755  N   TRP A 111       2.661  30.684  17.921  1.00 13.29           N  0.019
ANISOU 1755  N   TRP A 111     1538   1931   1580     78    194    465       N
ATOM   1756  CA  TRP A 111       1.284  31.154  17.744  1.00 11.97           C  0.018
ANISOU 1756  CA  TRP A 111     1430   1802   1317     26    -75    206       C
ATOM   1757  C   TRP A 111       1.195  32.640  18.088  1.00 11.96           C  0.018
ANISOU 1757  C   TRP A 111     1393   1774   1379   -161   -194    319       C
ATOM   1758  O   TRP A 111       0.628  33.437  17.337  1.00 11.93           O  0.018
ANISOU 1758  O   TRP A 111     1524   1768   1242    -17   -118    264       O
ATOM   1759  CB  TRP A 111       0.276  30.345  18.583  1.00 12.01           C  0.018
ANISOU 1759  CB  TRP A 111     1416   1741   1407    -12    -35    266       C
ATOM   1760  CG  TRP A 111      -1.097  30.894  18.460  1.00 11.42           C  0.018
ANISOU 1760  CG  TRP A 111     1455   1725   1160      3    105    221       C
ATOM   1761  CD1 TRP A 111      -2.020  30.582  17.485  1.00 11.49           C  0.018
ANISOU 1761  CD1 TRP A 111     1568   1564   1233     29    -12    157       C
ATOM   1762  CD2 TRP A 111      -1.713  31.861  19.303  1.00 11.29           C  0.018
ANISOU 1762  CD2 TRP A 111     1463   1734   1093   -120    112    149       C
ATOM   1763  NE1 TRP A 111      -3.173  31.320  17.674  1.00 11.51           N  0.018
ANISOU 1763  NE1 TRP A 111     1500   1675   1200    -71    -35     98       N
ATOM   1764  CE2 TRP A 111      -2.999  32.122  18.777  1.00 11.54           C  0.018
ANISOU 1764  CE2 TRP A 111     1512   1691   1181    -16     66    140       C
ATOM   1765  CE3 TRP A 111      -1.312  32.539  20.453  1.00 12.12           C  0.018
```

```
ANISOU 1765  CE3 TRP A 111     1603   1757   1244    -64     47    286       C
ATOM   1766  CZ2 TRP A 111      -3.885  32.992  19.405  1.00 12.38           C  0.018
ANISOU 1766  CZ2 TRP A 111     1605   1752   1347    -80     23    118       C
ATOM   1767  CZ3 TRP A 111      -2.187  33.386  21.059  1.00 12.63           C  0.019
ANISOU 1767  CZ3 TRP A 111     1804   1782   1212   -127    117    188       C
ATOM   1768  CH2 TRP A 111      -3.448  33.621  20.530  1.00 12.56           C  0.019
ANISOU 1768  CH2 TRP A 111     1782   1613   1377   -160    147     59       C
ATOM   1769  H   TRP A 111       2.764  30.063  18.507  1.00 15.95           H  0.021
ATOM   1770  HA  TRP A 111       1.039  31.052  16.811  1.00 14.37           H  0.020
ATOM   1771  HB2 TRP A 111       0.265  29.426  18.272  1.00 14.41           H  0.020
ATOM   1772  HB3 TRP A 111       0.535  30.381  19.517  1.00 14.41           H  0.020
ATOM   1773  HD1 TRP A 111      -1.869  30.000  16.775  1.00 13.79           H  0.019
ATOM   1774  HE1 TRP A 111      -3.876  31.290  17.180  1.00 13.82           H  0.019
ATOM   1775  HE3 TRP A 111      -0.469  32.391  20.818  1.00 14.54           H  0.020
ATOM   1776  HZ2 TRP A 111      -4.737  33.146  19.064  1.00 14.86           H  0.020
ATOM   1777  HZ3 TRP A 111      -1.916  33.855  21.815  1.00 15.16           H  0.020
ATOM   1778  HH2 TRP A 111      -4.011  34.225  20.957  1.00 15.07           H  0.020
ATOM   1779  N   ARG A 112       1.754  33.045  19.227  1.00 12.68           N  0.019
ANISOU 1779  N   ARG A 112     1553   1852   1411   -225   -217    214       N
ATOM   1780  CA  ARG A 112       1.656  34.448  19.605  1.00 12.95           C  0.019
ANISOU 1780  CA  ARG A 112     1636   1972   1314   -304   -128    274       C
ATOM   1781  C   ARG A 112       2.310  35.323  18.542  1.00 12.44           C  0.018
ANISOU 1781  C   ARG A 112     1548   1869   1310   -240   -131    202       C
ATOM   1782  O   ARG A 112       1.798  36.380  18.192  1.00 13.79           O  0.019
ANISOU 1782  O   ARG A 112     1744   1862   1634   -135   -189    235       O
ATOM   1783  CB  ARG A 112       2.269  34.705  20.992  1.00 15.02           C  0.020
ANISOU 1783  CB  ARG A 112     1926   2300   1481   -454    -63     75       C
ATOM   1784  CG  ARG A 112       2.269  36.189  21.382  1.00 16.54           C  0.021
ANISOU 1784  CG  ARG A 112     2176   2661   1447   -438   -110   -218       C
ATOM   1785  CD  ARG A 112       2.584  36.468  22.818  1.00 19.87           C  0.023
ANISOU 1785  CD  ARG A 112     2404   3101   2046   -522    136   -166       C
ATOM   1786  NE  ARG A 112       3.856  35.874  23.183  1.00 22.11           N  0.025
ANISOU 1786  NE  ARG A 112     2779   3549   2073   -551    -89   -111       N
ATOM   1787  CZ  ARG A 112       5.047  36.376  22.877  1.00 24.47           C  0.026
ANISOU 1787  CZ  ARG A 112     2897   3787   2613   -549     24   -216       C
ATOM   1788  NH1 ARG A 112       5.175  37.513  22.201  1.00 24.18           N  0.026
ANISOU 1788  NH1 ARG A 112     2987   3779   2421   -714     43   -302       N
ATOM   1789  NH2 ARG A 112       6.139  35.722  23.264  1.00 25.45           N  0.026
ANISOU 1789  NH2 ARG A 112     2786   3949   2935   -518     86   -218       N
ATOM   1790  H   ARG A 112       2.181  32.544  19.780  1.00 15.21           H  0.020
ATOM   1791  HA  ARG A 112       0.718  34.692  19.649  1.00 15.54           H  0.021
ATOM   1792  HB2 ARG A 112       1.756  34.221  21.658  1.00 18.02           H  0.022
ATOM   1793  HB3 ARG A 112       3.188  34.396  20.993  1.00 18.02           H  0.022
ATOM   1794  HG2 ARG A 112       2.932  36.650  20.845  1.00 19.85           H  0.023
ATOM   1795  HG3 ARG A 112       1.390  36.556  21.199  1.00 19.85           H  0.023
ATOM   1796  HD2 ARG A 112       2.640  37.426  22.957  1.00 23.85           H  0.026
ATOM   1797  HD3 ARG A 112       1.893  36.084  23.381  1.00 23.85           H  0.026
ATOM   1798  HE  ARG A 112       3.838  35.142  23.633  1.00 26.53           H  0.027
ATOM   1799 HH11 ARG A 112       4.477  37.945  21.946  1.00 29.02           H  0.028
ATOM   1800 HH12 ARG A 112       5.958  37.817  22.017  1.00 29.02           H  0.028
ATOM   1801 HH21 ARG A 112       6.067  34.986  23.703  1.00 30.54           H  0.029
ATOM   1802 HH22 ARG A 112       6.917  36.035  23.075  1.00 30.54           H  0.029
ATOM   1803  N   ASN A 113       3.481  34.924  18.059  1.00 12.72           N  0.019
ANISOU 1803  N   ASN A 113     1522   1909   1402   -262   -152    275       N
ATOM   1804  CA  ASN A 113       4.267  35.778  17.179  1.00 13.11           C  0.019
ANISOU 1804  CA  ASN A 113     1640   1950   1390   -381    -93    192       C
ATOM   1805  C   ASN A 113       3.854  35.734  15.715  1.00 13.31           C  0.019
ANISOU 1805  C   ASN A 113     1620   1869   1570   -371   -126    216       C
ATOM   1806  O   ASN A 113       4.168  36.682  14.983  1.00 13.32           O  0.019
ANISOU 1806  O   ASN A 113     1848   1769   1446   -373    -81    299       O
ATOM   1807  CB  ASN A 113       5.741  35.432  17.325  1.00 13.92           C  0.020
ANISOU 1807  CB  ASN A 113     1727   2044   1516   -424     -7    125       C
ATOM   1808  CG  ASN A 113       6.313  35.902  18.633  1.00 14.78           C  0.020
ANISOU 1808  CG  ASN A 113     1781   2213   1623   -420     39     -3       C
ATOM   1809  OD1 ASN A 113       5.871  36.921  19.189  1.00 16.47           O  0.021
ANISOU 1809  OD1 ASN A 113     1944   2427   1888   -298   -162   -107       O
ATOM   1810  ND2 ASN A 113       7.319  35.200  19.121  1.00 14.90           N  0.020
ANISOU 1810  ND2 ASN A 113     1976   2196   1489   -482   -206    221       N
ATOM   1811  H   ASN A 113       3.843  34.162  18.226  1.00 15.27           H  0.020
ATOM   1812  HA  ASN A 113       4.158  36.695  17.475  1.00 15.73           H  0.021
ATOM   1813  HB2 ASN A 113       5.847  34.469  17.279  1.00 16.70           H  0.021
ATOM   1814  HB3 ASN A 113       6.239  35.856  16.609  1.00 16.70           H  0.021
ATOM   1815 HD21 ASN A 113       7.683  35.430  19.865  1.00 17.88           H  0.022
ATOM   1816 HD22 ASN A 113       7.601  34.504  18.702  1.00 17.88           H  0.022
ATOM   1817  N   ARG A 114       3.151  34.696  15.281  1.00 12.90           N  0.019
ANISOU 1817  N   ARG A 114     1649   1995   1256   -328   -122    268       N
ATOM   1818  CA  ARG A 114       2.893  34.488  13.871  1.00 12.68           C  0.019
ANISOU 1818  CA  ARG A 114     1619   1932   1266   -254    -67    335       C
```

```
ATOM   1819  C   ARG A 114       1.441  34.240  13.526  1.00 12.49           C  0.018
ANISOU 1819  C   ARG A 114     1629   1925   1191   -154    -34    173       C
ATOM   1820  O   ARG A 114       1.086  34.376  12.353  1.00 13.82           O  0.019
ANISOU 1820  O   ARG A 114     1751   2363   1137    -79    -17    293       O
ATOM   1821  CB  ARG A 114       3.779  33.354  13.336  1.00 13.30           C  0.019
ANISOU 1821  CB  ARG A 114     1657   1977   1421   -207    -11    198       C
ATOM   1822  CG  ARG A 114       5.254  33.590  13.625  1.00 13.68           C  0.019
ANISOU 1822  CG  ARG A 114     1618   1999   1580   -227      3     48       C
ATOM   1823  CD  ARG A 114       6.159  32.520  13.139  1.00 13.45           C  0.019
ANISOU 1823  CD  ARG A 114     1661   2000   1452   -237    107     71       C
ATOM   1824  NE  ARG A 114       6.293  32.527  11.700  1.00 13.94           N  0.020
ANISOU 1824  NE  ARG A 114     1674   1986   1638   -314    224    -46       N
ATOM   1825  CZ  ARG A 114       7.125  31.732  11.057  1.00 14.15           C  0.020
ANISOU 1825  CZ  ARG A 114     1711   2037   1627   -353    223    -17       C
ATOM   1826  NH1 ARG A 114       7.854  30.836  11.708  1.00 15.29           N  0.020
ANISOU 1826  NH1 ARG A 114     1807   2047   1955   -358    196    -16       N
ATOM   1827  NH2 ARG A 114       7.244  31.846   9.737  1.00 15.14           N  0.020
ANISOU 1827  NH2 ARG A 114     1831   2151   1769   -264    140    -63       N
ATOM   1828  H   ARG A 114       2.810  34.093  15.791  1.00 15.48           H  0.021
ATOM   1829  HA  ARG A 114       3.154  35.291  13.396  1.00 15.22           H  0.020
ATOM   1830  HB2 ARG A 114       3.518  32.521  13.758  1.00 15.97           H  0.021
ATOM   1831  HB3 ARG A 114       3.667  33.290  12.375  1.00 15.97           H  0.021
ATOM   1832  HG2 ARG A 114       5.524  34.419  13.200  1.00 16.42           H  0.021
ATOM   1833  HG3 ARG A 114       5.377  33.660  14.584  1.00 16.42           H  0.021
ATOM   1834  HD2 ARG A 114       7.039  32.649  13.525  1.00 16.15           H  0.021
ATOM   1835  HD3 ARG A 114       5.802  31.658  13.404  1.00 16.15           H  0.021
ATOM   1836  HE  ARG A 114       5.810  33.073  11.244  1.00 16.73           H  0.021
ATOM   1837 HH11 ARG A 114       7.787  30.758  12.561  1.00 18.34           H  0.022
ATOM   1838 HH12 ARG A 114       8.393  30.326  11.273  1.00 18.34           H  0.022
ATOM   1839 HH21 ARG A 114       6.773  32.426   9.311  1.00 18.16           H  0.022
ATOM   1840 HH22 ARG A 114       7.783  31.331   9.308  1.00 18.16           H  0.022
ATOM   1841  N   CYS A 115       0.593  33.905  14.481  1.00 11.69           N  0.018
ANISOU 1841  N   CYS A 115     1574   1728   1141   -145     -6    151       N
ATOM   1842  CA  CYS A 115      -0.810  33.621  14.211  1.00 11.87           C  0.018
ANISOU 1842  CA  CYS A 115     1464   1717   1331    -63     15     78       C
ATOM   1843  C   CYS A 115      -1.759  34.598  14.894  1.00 11.15           C  0.017
ANISOU 1843  C   CYS A 115     1628   1698    912     47      1     96       C
ATOM   1844  O   CYS A 115      -2.777  34.984  14.315  1.00 12.18           O  0.018
ANISOU 1844  O   CYS A 115     1702   1814   1114     33   -158     37       O
ATOM   1845  CB  CYS A 115      -1.141  32.195  14.707  1.00 12.08           C  0.018
ANISOU 1845  CB  CYS A 115     1489   1667   1436   -123   -189    156       C
ATOM   1846  SG  CYS A 115      -0.153  30.912  13.918  1.00 12.38           S  0.018
ANISOU 1846  SG  CYS A 115     1532   1676   1496      1   -120     22       S
ATOM   1847  H   CYS A 115       0.807  33.833  15.311  1.00 14.03           H  0.020
ATOM   1848  HA  CYS A 115      -0.973  33.656  13.256  1.00 14.25           H  0.020
ATOM   1849  HB2 CYS A 115      -0.965  32.159  15.658  1.00 14.50           H  0.020
ATOM   1850  HB3 CYS A 115      -2.076  32.006  14.533  1.00 14.50           H  0.020
ATOM   1851  N   LYS A 116      -1.499  34.919  16.152  1.00 11.85           N  0.018
ANISOU 1851  N   LYS A 116     1735   1681   1087     73    -25     28       N
ATOM   1852  CA  LYS A 116      -2.360  35.767  16.950  1.00 12.71           C  0.019
ANISOU 1852  CA  LYS A 116     1787   1942   1102     92    -94      5       C
ATOM   1853  C   LYS A 116      -2.611  37.077  16.228  1.00 14.23           C  0.020
ANISOU 1853  C   LYS A 116     1962   2131   1314    300    -87   -224       C
ATOM   1854  O   LYS A 116      -1.681  37.766  15.787  1.00 14.42           O  0.020
ANISOU 1854  O   LYS A 116     2189   1775   1515    124   -113     98       O
ATOM   1855  CB  LYS A 116      -1.667  36.039  18.283  1.00 12.66           C  0.019
ANISOU 1855  CB  LYS A 116     1926   1785   1101    133   -178    -14       C
ATOM   1856  CG  LYS A 116      -2.455  36.825  19.278  1.00 13.11           C  0.019
ANISOU 1856  CG  LYS A 116     1927   1844   1209     47    -40     14       C
ATOM   1857  CD  LYS A 116      -1.670  36.997  20.558  1.00 13.36           C  0.019
ANISOU 1857  CD  LYS A 116     1861   1894   1321   -103     35     84       C
ATOM   1858  CE  LYS A 116      -2.435  37.616  21.706  1.00 13.52           C  0.019
ANISOU 1858  CE  LYS A 116     1858   1978   1301    -16     -2     25       C
ATOM   1859  NZ  LYS A 116      -1.639  37.725  22.954  1.00 13.47           N  0.019
ANISOU 1859  NZ  LYS A 116     1846   1989   1282   -115    -95    -79       N
ATOM   1860  H   LYS A 116      -0.803  34.646  16.578  1.00 14.22           H  0.020
ATOM   1861  HA  LYS A 116      -3.208  35.325  17.115  1.00 15.26           H  0.020
ATOM   1862  HB2 LYS A 116      -1.449  35.187  18.693  1.00 15.20           H  0.020
ATOM   1863  HB3 LYS A 116      -0.849  36.531  18.108  1.00 15.20           H  0.020
ATOM   1864  HG2 LYS A 116      -2.648  37.704  18.917  1.00 15.73           H  0.021
ATOM   1865  HG3 LYS A 116      -3.278  36.354  19.483  1.00 15.73           H  0.021
ATOM   1866  HD2 LYS A 116      -1.362  36.125  20.850  1.00 16.03           H  0.021
ATOM   1867  HD3 LYS A 116      -0.906  37.567  20.376  1.00 16.03           H  0.021
ATOM   1868  HE2 LYS A 116      -2.715  38.509  21.452  1.00 16.22           H  0.021
ATOM   1869  HE3 LYS A 116      -3.212  37.068  21.896  1.00 16.22           H  0.021
ATOM   1870  HZ1 LYS A 116      -0.921  38.231  22.813  1.00 16.16           H  0.021
ATOM   1871  HZ2 LYS A 116      -2.131  38.094  23.597  1.00 16.16           H  0.021
ATOM   1872  HZ3 LYS A 116      -1.376  36.917  23.218  1.00 16.16           H  0.021
```

```
ATOM   1873  N   GLY A 117      -3.885  37.433  16.123  1.00 16.39           N  0.021
ANISOU 1873  N   GLY A 117     2164   2651   1412    696   -200   -101       N
ATOM   1874  CA  GLY A 117      -4.242  38.687  15.502  1.00 17.89           C  0.022
ANISOU 1874  CA  GLY A 117     2576   2661   1560    804   -230   -289       C
ATOM   1875  C   GLY A 117      -4.358  38.645  14.002  1.00 18.52           C  0.023
ANISOU 1875  C   GLY A 117     2920   2411   1706    762   -494   -166       C
ATOM   1876  O   GLY A 117      -4.832  39.617  13.419  1.00 21.10           O  0.024
ANISOU 1876  O   GLY A 117     3516   2344   2158    893   -649   -164       O
ATOM   1877  H   GLY A 117      -4.552  36.967  16.402  1.00 19.67           H  0.023
ATOM   1878  HA2 GLY A 117      -5.095  38.982  15.858  1.00 21.47           H  0.024
ATOM   1879  HA3 GLY A 117      -3.576  39.353  15.731  1.00 21.47           H  0.024
ATOM   1880  N   THR A 118      -3.990  37.550  13.367  1.00 15.64           N  0.021
ANISOU 1880  N   THR A 118     2577   2122   1243    470   -410   -213       N
ATOM   1881  CA  THR A 118      -4.013  37.443  11.921  1.00 15.16           C  0.020
ANISOU 1881  CA  THR A 118     2368   1958   1434    237   -327     68       C
ATOM   1882  C   THR A 118      -5.303  36.760  11.474  1.00 15.01           C  0.020
ANISOU 1882  C   THR A 118     2370   1988   1344    233   -417    267       C
ATOM   1883  O   THR A 118      -6.080  36.244  12.282  1.00 14.79           O  0.020
ANISOU 1883  O   THR A 118     2232   2041   1345    109   -177    181       O
ATOM   1884  CB  THR A 118      -2.801  36.668  11.397  1.00 14.81           C  0.020
ANISOU 1884  CB  THR A 118     2308   1953   1366     84   -233     66       C
ATOM   1885  OG1 THR A 118      -2.975  35.268  11.644  1.00 14.32           O  0.020
ANISOU 1885  OG1 THR A 118     2180   1898   1364     89   -294     -6       O
ATOM   1886  CG2 THR A 118      -1.494  37.175  11.955  1.00 15.85           C  0.021
ANISOU 1886  CG2 THR A 118     2404   2020   1600    -42     14     19       C
ATOM   1887  H   THR A 118      -3.716  36.836  13.760  1.00 18.77           H  0.023
ATOM   1888  HA  THR A 118      -3.992  38.332  11.534  1.00 18.19           H  0.022
ATOM   1889  HB  THR A 118      -2.762  36.796  10.436  1.00 17.77           H  0.022
ATOM   1890  HG1 THR A 118      -3.053  35.129  12.469  1.00 17.19           H  0.022
ATOM   1891 HG21 THR A 118      -0.753  36.702  11.545  1.00 19.03           H  0.023
ATOM   1892 HG22 THR A 118      -1.402  38.123  11.772  1.00 19.03           H  0.023
ATOM   1893 HG23 THR A 118      -1.465  37.035  12.914  1.00 19.03           H  0.023
ATOM   1894  N   ASP A 119      -5.526  36.762  10.153  1.00 15.55           N  0.021
ANISOU 1894  N   ASP A 119     2575   1942   1392    119   -385    255       N
ATOM   1895  CA  ASP A 119      -6.704  36.078   9.568  1.00 16.69           C  0.021
ANISOU 1895  CA  ASP A 119     2859   1961   1524     70   -611    206       C
ATOM   1896  C   ASP A 119      -6.414  34.580   9.594  1.00 16.79           C  0.021
ANISOU 1896  C   ASP A 119     3006   1994   1378     77   -271     96       C
ATOM   1897  O   ASP A 119      -5.904  34.065   8.621  1.00 18.88           O  0.023
ANISOU 1897  O   ASP A 119     3350   2090   1732     22    -49     90       O
ATOM   1898  CB  ASP A 119      -6.988  36.557   8.140  1.00 18.26           C  0.022
ANISOU 1898  CB  ASP A 119     3044   2085   1810     29   -879    353       C
ATOM   1899  CG  ASP A 119      -8.263  35.988   7.548  1.00 19.94           C  0.023
ANISOU 1899  CG  ASP A 119     3074   2258   2246    -55   -905    365       C
ATOM   1900  OD1 ASP A 119      -8.701  34.909   7.974  1.00 21.92           O  0.024
ANISOU 1900  OD1 ASP A 119     3270   2588   2473   -236  -1025    319       O
ATOM   1901  OD2 ASP A 119      -8.811  36.648   6.679  1.00 19.90           O  0.023
ANISOU 1901  OD2 ASP A 119     2973   2464   2123    123   -705    471       O
ATOM   1902  H   ASP A 119      -5.019  37.147   9.574  1.00 18.66           H  0.023
ATOM   1903  HA  ASP A 119      -7.486  36.252  10.114  1.00 20.03           H  0.023
ATOM   1904  HB2 ASP A 119      -7.069  37.524   8.145  1.00 21.92           H  0.024
ATOM   1905  HB3 ASP A 119      -6.251  36.292   7.568  1.00 21.92           H  0.024
ATOM   1906  N   VAL A 120      -6.745  33.906  10.684  1.00 16.02           N  0.021
ANISOU 1906  N   VAL A 120     2893   1860   1336     88   -364    160       N
ATOM   1907  CA  VAL A 120      -6.433  32.489  10.802  1.00 16.01           C  0.021
ANISOU 1907  CA  VAL A 120     2755   1823   1505    145   -278    279       C
ATOM   1908  C   VAL A 120      -7.459  31.640  10.069  1.00 16.22           C  0.021
ANISOU 1908  C   VAL A 120     2777   1871   1514     92   -238    181       C
ATOM   1909  O   VAL A 120      -7.172  30.488   9.749  1.00 16.52           O  0.021
ANISOU 1909  O   VAL A 120     2727   1990   1560    105   -316     98       O
ATOM   1910  CB  VAL A 120      -6.290  32.046  12.268  1.00 16.68           C  0.021
ANISOU 1910  CB  VAL A 120     2663   2035   1639     95   -352    223       C
ATOM   1911  CG1 VAL A 120      -5.073  32.724  12.908  1.00 16.53           C  0.021
ANISOU 1911  CG1 VAL A 120     2543   1982   1755    198   -554     99       C
ATOM   1912  CG2 VAL A 120      -7.534  32.304  13.073  1.00 17.43           C  0.022
ANISOU 1912  CG2 VAL A 120     2616   2240   1768     38   -164    227       C
ATOM   1913  H   VAL A 120      -7.148  34.243  11.365  1.00 19.23           H  0.023
ATOM   1914  HA  VAL A 120      -5.577  32.333  10.374  1.00 19.22           H  0.023
ATOM   1915  HB  VAL A 120      -6.129  31.090  12.283  1.00 20.01           H  0.023
ATOM   1916 HG11 VAL A 120      -4.995  32.429  13.829  1.00 19.84           H  0.023
ATOM   1917 HG12 VAL A 120      -4.277  32.476  12.412  1.00 19.84           H  0.023
ATOM   1918 HG13 VAL A 120      -5.196  33.686  12.878  1.00 19.84           H  0.023
ATOM   1919 HG21 VAL A 120      -7.401  31.967  13.973  1.00 20.92           H  0.024
ATOM   1920 HG22 VAL A 120      -7.702  33.259  13.101  1.00 20.92           H  0.024
ATOM   1921 HG23 VAL A 120      -8.282  31.847  12.658  1.00 20.92           H  0.024
ATOM   1922  N   GLN A 121      -8.620  32.194   9.716  1.00 16.95           N  0.022
ANISOU 1922  N   GLN A 121     2742   1837   1862     68   -165    115       N
ATOM   1923  CA  GLN A 121      -9.580  31.480   8.880  1.00 18.78           C  0.023
```

```
ANISOU 1923  CA  GLN A 121     2740   2076   2320    -33   -284     41       C
ATOM   1924  C   GLN A 121      -8.962  31.097   7.539  1.00 17.02           C  0.022
ANISOU 1924  C   GLN A 121     2404   2074   1987     34   -490    135       C
ATOM   1925  O   GLN A 121      -9.359  30.097   6.936  1.00 17.26           O  0.022
ANISOU 1925  O   GLN A 121     2241   2257   2060    -32   -634    143       O
ATOM   1926  CB  GLN A 121     -10.810  32.365   8.650  1.00 24.00           C  0.026
ANISOU 1926  CB  GLN A 121     3107   2437   3577   -258   -498   -137       C
ATOM   1927  CG  GLN A 121     -11.991  31.711   7.908  1.00 30.80           C  0.029
ANISOU 1927  CG  GLN A 121     3663   2966   5074   -321   -432   -181       C
ATOM   1928  CD  GLN A 121     -12.803  30.740   8.748  1.00 36.10           C  0.031
ANISOU 1928  CD  GLN A 121     4206   3363   6148   -296   -360   -237       C
ATOM   1929  OE1 GLN A 121     -13.095  30.996   9.918  1.00 38.38           O  0.032
ANISOU 1929  OE1 GLN A 121     4429   3587   6566   -282   -367   -252       O
ATOM   1930  NE2 GLN A 121     -13.183  29.619   8.145  1.00 38.43           N  0.032
ANISOU 1930  NE2 GLN A 121     4390   3522   6689   -313   -241   -202       N
ATOM   1931  H   GLN A 121      -8.875  32.982   9.949  1.00 20.34           H  0.024
ATOM   1932  HA  GLN A 121      -9.863  30.670   9.331  1.00 22.54           H  0.025
ATOM   1933  HB2 GLN A 121     -11.139  32.658   9.514  1.00 28.81           H  0.028
ATOM   1934  HB3 GLN A 121     -10.536  33.138   8.131  1.00 28.81           H  0.028
ATOM   1935  HG2 GLN A 121     -12.592  32.410   7.606  1.00 36.96           H  0.032
ATOM   1936  HG3 GLN A 121     -11.649  31.223   7.143  1.00 36.96           H  0.032
ATOM   1937 HE21 GLN A 121     -12.963  29.475   7.326  1.00 46.12           H  0.036
ATOM   1938 HE22 GLN A 121     -13.646  29.035   8.575  1.00 46.12           H  0.036
ATOM   1939  N   ALA A 122      -7.964  31.852   7.075  1.00 15.78           N  0.021
ANISOU 1939  N   ALA A 122     2339   2080   1576     26   -481    199       N
ATOM   1940  CA  ALA A 122      -7.328  31.506   5.811  1.00 17.20           C  0.022
ANISOU 1940  CA  ALA A 122     2494   2320   1720   -119   -484    138       C
ATOM   1941  C   ALA A 122      -6.726  30.109   5.847  1.00 16.28           C  0.021
ANISOU 1941  C   ALA A 122     2277   2405   1504    -93   -654     24       C
ATOM   1942  O   ALA A 122      -6.638  29.448   4.806  1.00 17.56           O  0.022
ANISOU 1942  O   ALA A 122     2468   2575   1627    -74   -558     83       O
ATOM   1943  CB  ALA A 122      -6.223  32.480   5.448  1.00 18.70           C  0.023
ANISOU 1943  CB  ALA A 122     2694   2277   2132   -191   -247    133       C
ATOM   1944  H   ALA A 122      -7.646  32.551   7.463  1.00 18.93           H  0.023
ATOM   1945  HA  ALA A 122      -7.993  31.529   5.105  1.00 20.64           H  0.024
ATOM   1946  HB1 ALA A 122      -5.830  32.211   4.603  1.00 22.44           H  0.025
ATOM   1947  HB2 ALA A 122      -6.601  33.370   5.369  1.00 22.44           H  0.025
ATOM   1948  HB3 ALA A 122      -5.549  32.467   6.146  1.00 22.44           H  0.025
ATOM   1949  N   TRP A 123      -6.332  29.625   7.034  1.00 15.88           N  0.021
ANISOU 1949  N   TRP A 123     2143   2311   1580    -44   -493     28       N
ATOM   1950  CA  TRP A 123      -5.704  28.310   7.145  1.00 15.87           C  0.021
ANISOU 1950  CA  TRP A 123     2076   2201   1755    -39   -538    -58       C
ATOM   1951  C   TRP A 123      -6.687  27.174   6.883  1.00 16.28           C  0.021
ANISOU 1951  C   TRP A 123     2010   2238   1938    -69   -368    -67       C
ATOM   1952  O   TRP A 123      -6.245  26.062   6.597  1.00 17.23           O  0.022
ANISOU 1952  O   TRP A 123     2076   2284   2189    -75   -279   -122       O
ATOM   1953  CB  TRP A 123      -5.069  28.157   8.561  1.00 16.27           C  0.021
ANISOU 1953  CB  TRP A 123     2159   2162   1861   -133   -549    117       C
ATOM   1954  CG  TRP A 123      -3.852  29.005   8.615  1.00 16.17           C  0.021
ANISOU 1954  CG  TRP A 123     2093   2233   1817   -138   -507    202       C
ATOM   1955  CD1 TRP A 123      -3.738  30.210   9.224  1.00 16.75           C  0.021
ANISOU 1955  CD1 TRP A 123     2194   2364   1808   -155   -433     56       C
ATOM   1956  CD2 TRP A 123      -2.590  28.736   8.000  1.00 17.47           C  0.022
ANISOU 1956  CD2 TRP A 123     2115   2372   2150   -185   -456    490       C
ATOM   1957  NE1 TRP A 123      -2.486  30.736   9.002  1.00 17.35           N  0.022
ANISOU 1957  NE1 TRP A 123     2168   2422   2001   -299   -672    277       N
ATOM   1958  CE2 TRP A 123      -1.757  29.846   8.255  1.00 17.61           C  0.022
ANISOU 1958  CE2 TRP A 123     2066   2421   2202   -162   -612    665       C
ATOM   1959  CE3 TRP A 123      -2.085  27.669   7.239  1.00 19.29           C  0.023
ANISOU 1959  CE3 TRP A 123     2143   2448   2739     83   -428    509       C
ATOM   1960  CZ2 TRP A 123      -0.449  29.923   7.778  1.00 19.50           C  0.023
ANISOU 1960  CZ2 TRP A 123     2084   2580   2745   -227   -356    725       C
ATOM   1961  CZ3 TRP A 123      -0.787  27.746   6.761  1.00 20.75           C  0.024
ANISOU 1961  CZ3 TRP A 123     2170   2603   3113     86   -154    389       C
ATOM   1962  CH2 TRP A 123       0.006  28.881   7.013  1.00 21.34           C  0.024
ANISOU 1962  CH2 TRP A 123     2107   2667   3336     -3   -126    564       C
ATOM   1963  H   TRP A 123      -6.418  30.039   7.783  1.00 19.06           H  0.023
ATOM   1964  HA  TRP A 123      -4.992  28.245   6.489  1.00 19.05           H  0.023
ATOM   1965  HB2 TRP A 123      -5.694  28.460   9.238  1.00 19.52           H  0.023
ATOM   1966  HB3 TRP A 123      -4.815  27.233   8.712  1.00 19.52           H  0.023
ATOM   1967  HD1 TRP A 123      -4.419  30.636   9.692  1.00 20.10           H  0.023
ATOM   1968  HE1 TRP A 123      -2.201  31.491   9.299  1.00 20.82           H  0.024
ATOM   1969  HE3 TRP A 123      -2.625  26.942   7.027  1.00 23.15           H  0.025
ATOM   1970  HZ2 TRP A 123       0.092  30.655   7.968  1.00 23.40           H  0.025
ATOM   1971  HZ3 TRP A 123      -0.444  27.051   6.247  1.00 24.91           H  0.026
ATOM   1972  HH2 TRP A 123       0.887  28.890   6.716  1.00 25.61           H  0.026
ATOM   1973  N   ILE A 124      -7.995  27.397   7.019  1.00 16.50           N  0.021
ANISOU 1973  N   ILE A 124     2083   2227   1960   -171   -395    -11       N
```

```
ATOM   1974  CA  ILE A 124      -8.983  26.366   6.755  1.00 17.94           C  0.022
ANISOU 1974  CA  ILE A 124     2320   2404   2092   -299   -687     90       C
ATOM   1975  C   ILE A 124      -9.809  26.673   5.526  1.00 19.46           C  0.023
ANISOU 1975  C   ILE A 124     2520   2626   2249   -338   -942    193       C
ATOM   1976  O   ILE A 124     -10.675  25.891   5.177  1.00 19.26           O  0.023
ANISOU 1976  O   ILE A 124     2491   2675   2153   -301   -880    170       O
ATOM   1977  CB  ILE A 124      -9.864  26.081   7.993  1.00 19.36           C  0.023
ANISOU 1977  CB  ILE A 124     2552   2467   2338   -290   -377     75       C
ATOM   1978  CG1 ILE A 124     -10.712  27.293   8.386  1.00 21.69           C  0.024
ANISOU 1978  CG1 ILE A 124     2853   2552   2835   -293    -30    -91       C
ATOM   1979  CG2 ILE A 124      -8.980  25.572   9.178  1.00 18.58           C  0.023
ANISOU 1979  CG2 ILE A 124     2549   2527   1986   -172   -357    265       C
ATOM   1980  CD1 ILE A 124     -11.809  26.956   9.369  1.00 22.74           C  0.025
ANISOU 1980  CD1 ILE A 124     3054   2601   2986   -267    -25    -87       C
ATOM   1981  H   ILE A 124      -8.334  28.148   7.266  1.00 19.80           H  0.023
ATOM   1982  HA  ILE A 124      -8.510  25.541   6.564  1.00 21.53           H  0.024
ATOM   1983  HB  ILE A 124     -10.474  25.365   7.757  1.00 23.23           H  0.025
ATOM   1984 HG12 ILE A 124     -10.138  27.958   8.797  1.00 26.03           H  0.027
ATOM   1985 HG13 ILE A 124     -11.130  27.663   7.593  1.00 26.03           H  0.027
ATOM   1986 HG21 ILE A 124      -9.556  25.279   9.901  1.00 22.30           H  0.025
ATOM   1987 HG22 ILE A 124      -8.436  24.831   8.868  1.00 22.30           H  0.025
ATOM   1988 HG23 ILE A 124      -8.410  26.297   9.480  1.00 22.30           H  0.025
ATOM   1989 HD11 ILE A 124     -12.245  27.775   9.648  1.00 27.29           H  0.027
ATOM   1990 HD12 ILE A 124     -12.450  26.369   8.938  1.00 27.29           H  0.027
ATOM   1991 HD13 ILE A 124     -11.420  26.513  10.139  1.00 27.29           H  0.027
ATOM   1992  N   ARG A 125      -9.530  27.770   4.832  1.00 21.89           N  0.024
ANISOU 1992  N   ARG A 125     2985   2916   2415   -400  -1307    279       N
ATOM   1993  CA  ARG A 125     -10.359  28.191   3.707  1.00 24.64           C  0.026
ANISOU 1993  CA  ARG A 125     3431   3418   2514   -511  -1492    399       C
ATOM   1994  C   ARG A 125     -10.300  27.149   2.601  1.00 24.35           C  0.026
ANISOU 1994  C   ARG A 125     3515   3542   2195   -573  -1409    396       C
ATOM   1995  O   ARG A 125      -9.234  26.627   2.261  1.00 24.98           O  0.026
ANISOU 1995  O   ARG A 125     3567   3715   2209   -686  -1120    169       O
ATOM   1996  CB  ARG A 125      -9.864  29.557   3.222  1.00 28.25           C  0.028
ANISOU 1996  CB  ARG A 125     3820   3880   3035   -573  -1730    509       C
ATOM   1997  CG  ARG A 125     -10.478  30.102   1.931  1.00 33.12           C  0.030
ANISOU 1997  CG  ARG A 125     4151   4354   4079   -699  -1702    416       C
ATOM   1998  CD  ARG A 125      -9.780  31.423   1.523  1.00 37.78           C  0.032
ANISOU 1998  CD  ARG A 125     4492   4799   5062   -788  -1501    278       C
ATOM   1999  NE  ARG A 125      -8.337  31.229   1.401  1.00 41.55           N  0.034
ANISOU 1999  NE  ARG A 125     4764   5216   5809   -836  -1244     70       N
ATOM   2000  CZ  ARG A 125      -7.414  32.154   1.636  1.00 43.81           C  0.035
ANISOU 2000  CZ  ARG A 125     4935   5523   6186   -859  -1150   -106       C
ATOM   2001  NH1 ARG A 125      -7.738  33.400   1.948  1.00 44.10           N  0.035
ANISOU 2001  NH1 ARG A 125     4989   5575   6191   -856  -1174   -175       N
ATOM   2002  NH2 ARG A 125      -6.128  31.818   1.561  1.00 44.84           N  0.035
ANISOU 2002  NH2 ARG A 125     5005   5678   6355   -808  -1089   -128       N
ATOM   2003  H   ARG A 125      -8.865  28.291   4.990  1.00 26.27           H  0.027
ATOM   2004  HA  ARG A 125     -11.280  28.281   3.998  1.00 29.57           H  0.028
ATOM   2005  HB2 ARG A 125     -10.042  30.207   3.919  1.00 33.90           H  0.030
ATOM   2006  HB3 ARG A 125      -8.907  29.492   3.084  1.00 33.90           H  0.030
ATOM   2007  HG2 ARG A 125     -10.358  29.460   1.215  1.00 39.74           H  0.033
ATOM   2008  HG3 ARG A 125     -11.421  30.282   2.071  1.00 39.74           H  0.033
ATOM   2009  HD2 ARG A 125     -10.123  31.718   0.665  1.00 45.33           H  0.035
ATOM   2010  HD3 ARG A 125      -9.946  32.094   2.203  1.00 45.33           H  0.035
ATOM   2011  HE  ARG A 125      -8.063  30.451   1.157  1.00 49.87           H  0.037
ATOM   2012 HH11 ARG A 125      -8.564  33.631   2.003  1.00 52.92           H  0.038
ATOM   2013 HH12 ARG A 125      -7.120  33.980   2.094  1.00 52.92           H  0.038
ATOM   2014 HH21 ARG A 125      -5.905  31.013   1.358  1.00 53.81           H  0.038
ATOM   2015 HH22 ARG A 125      -5.520  32.409   1.707  1.00 53.81           H  0.038
ATOM   2016  N   GLY A 126     -11.473  26.809   2.078  1.00 25.09           N  0.026
ANISOU 2016  N   GLY A 126     3586   3477   2471   -612  -1433    277       N
ATOM   2017  CA  GLY A 126     -11.598  25.869   0.997  1.00 25.08           C  0.026
ANISOU 2017  CA  GLY A 126     3599   3405   2524   -574  -1259    282       C
ATOM   2018  C   GLY A 126     -11.607  24.419   1.406  1.00 24.32           C  0.026
ANISOU 2018  C   GLY A 126     3331   3304   2604   -583  -1194    266       C
ATOM   2019  O   GLY A 126     -11.989  23.573   0.606  1.00 25.08           O  0.026
ANISOU 2019  O   GLY A 126     3450   3423   2656   -458  -1153     69       O
ATOM   2020  H   GLY A 126     -12.226  27.124   2.347  1.00 30.11           H  0.029
ATOM   2021  HA2 GLY A 126     -12.423  26.049   0.519  1.00 30.09           H  0.029
ATOM   2022  HA3 GLY A 126     -10.861  26.000   0.381  1.00 30.09           H  0.029
ATOM   2023  N   CYS A 127     -11.228  24.102   2.634  1.00 22.31           N  0.025
ANISOU 2023  N   CYS A 127     2906   3132   2439   -499  -1378    384       N
ATOM   2024  CA  CYS A 127     -11.115  22.711   3.040  1.00 21.41           C  0.024
ANISOU 2024  CA  CYS A 127     2645   3003   2487   -373  -1291    248       C
ATOM   2025  C   CYS A 127     -12.487  22.094   3.227  1.00 23.89           C  0.026
ANISOU 2025  C   CYS A 127     2845   3239   2994   -493  -1329    191       C
ATOM   2026  O   CYS A 127     -13.399  22.740   3.741  1.00 23.76           O  0.025
```

```
ANISOU 2026  O   CYS A 127     2665   3090   3274   -509  -1348    298       O
ATOM   2027  CB  CYS A 127     -10.384  22.623   4.370  1.00 19.27           C  0.023
ANISOU 2027  CB  CYS A 127     2352   2741   2227    -66  -1049    164       C
ATOM   2028  SG  CYS A 127      -8.718  23.339   4.375  1.00 19.95           S  0.023
ANISOU 2028  SG  CYS A 127     2381   2836   2364     70   -879      9       S
ATOM   2029  H   CYS A 127     -11.031  24.670   3.249  1.00 26.77           H  0.027
ATOM   2030  HA  CYS A 127     -10.626  22.205   2.372  1.00 25.69           H  0.027
ATOM   2031  HB2 CYS A 127     -10.906  23.091   5.041  1.00 23.12           H  0.025
ATOM   2032  HB3 CYS A 127     -10.302  21.688   4.616  1.00 23.12           H  0.025
ATOM   2033  N   ARG A 128     -12.612  20.815   2.853  1.00 27.14           N  0.027
ANISOU 2033  N   ARG A 128     3216   3688   3407   -487  -1194   -275       N
ATOM   2034  CA  ARG A 128     -13.813  20.036   3.139  1.00 32.23           C  0.030
ANISOU 2034  CA  ARG A 128     3707   4345   4194   -443  -1212   -471       C
ATOM   2035  C   ARG A 128     -13.786  19.678   4.623  1.00 33.38           C  0.030
ANISOU 2035  C   ARG A 128     3734   4544   4403   -624  -1094   -253       C
ATOM   2036  O   ARG A 128     -12.872  18.984   5.084  1.00 34.20           O  0.031
ANISOU 2036  O   ARG A 128     3758   4569   4667   -558   -900   -190       O
ATOM   2037  CB  ARG A 128     -13.852  18.783   2.257  1.00 37.40           C  0.032
ANISOU 2037  CB  ARG A 128     4224   4826   5159   -190  -1090   -746       C
ATOM   2038  CG  ARG A 128     -15.141  17.950   2.350  1.00 41.85           C  0.034
ANISOU 2038  CG  ARG A 128     4689   5311   5901    149  -1146   -884       C
ATOM   2039  CD  ARG A 128     -15.759  17.658   0.955  1.00 45.93           C  0.035
ANISOU 2039  CD  ARG A 128     5122   5735   6593    486  -1132   -995       C
ATOM   2040  NE  ARG A 128     -14.904  16.804   0.135  1.00 49.23           N  0.037
ANISOU 2040  NE  ARG A 128     5485   6062   7159    787  -1146  -1135       N
ATOM   2041  CZ  ARG A 128     -14.920  16.760  -1.193  1.00 51.69           C  0.038
ANISOU 2041  CZ  ARG A 128     5744   6319   7577   1003  -1108  -1255       C
ATOM   2042  NH1 ARG A 128     -15.737  17.520  -1.906  1.00 52.52           N  0.038
ANISOU 2042  NH1 ARG A 128     5840   6421   7696   1058  -1102  -1256       N
ATOM   2043  NH2 ARG A 128     -14.090  15.932  -1.823  1.00 52.43           N  0.038
ANISOU 2043  NH2 ARG A 128     5821   6392   7710   1070  -1100  -1330       N
ATOM   2044  H   ARG A 128     -12.007  20.376   2.428  1.00 32.56           H  0.030
ATOM   2045  HA  ARG A 128     -14.603  20.570   2.960  1.00 38.68           H  0.033
ATOM   2046  HB2 ARG A 128     -13.747  19.055   1.332  1.00 44.88           H  0.035
ATOM   2047  HB3 ARG A 128     -13.114  18.206   2.510  1.00 44.88           H  0.035
ATOM   2048  HG2 ARG A 128     -14.940  17.101   2.774  1.00 50.22           H  0.037
ATOM   2049  HG3 ARG A 128     -15.796  18.437   2.874  1.00 50.22           H  0.037
ATOM   2050  HD2 ARG A 128     -16.611  17.210   1.072  1.00 55.11           H  0.039
ATOM   2051  HD3 ARG A 128     -15.884  18.493   0.501  1.00 55.11           H  0.039
ATOM   2052  HE  ARG A 128     -14.348  16.290   0.543  1.00 59.08           H  0.040
ATOM   2053 HH11 ARG A 128     -16.279  18.060  -1.513  1.00 63.03           H  0.042
ATOM   2054 HH12 ARG A 128     -15.727  17.472  -2.765  1.00 63.03           H  0.042
ATOM   2055 HH21 ARG A 128     -13.554  15.434  -1.371  1.00 62.92           H  0.041
ATOM   2056 HH22 ARG A 128     -14.090  15.895  -2.682  1.00 62.92           H  0.041
ATOM   2057  N   LEU A 129     -14.758  20.186   5.377  1.00 33.58           N  0.030
ANISOU 2057  N   LEU A 129     3734   4727   4299   -758  -1148    -27       N
ATOM   2058  CA  LEU A 129     -14.789  19.972   6.825  1.00 35.05           C  0.031
ANISOU 2058  CA  LEU A 129     3869   4975   4475   -835  -1039     77       C
ATOM   2059  C   LEU A 129     -16.196  19.638   7.324  1.00 37.86           C  0.032
ANISOU 2059  C   LEU A 129     4047   5229   5110   -835   -906     43       C
ATOM   2060  O   LEU A 129     -17.150  19.569   6.547  1.00 38.62           O  0.033
ANISOU 2060  O   LEU A 129     4090   5296   5287   -892   -864    -67       O
ATOM   2061  CB  LEU A 129     -14.258  21.206   7.565  1.00 33.93           C  0.030
ANISOU 2061  CB  LEU A 129     3862   4952   4080   -874   -990    265       C
ATOM   2062  CG  LEU A 129     -12.793  21.561   7.317  1.00 32.55           C  0.030
ANISOU 2062  CG  LEU A 129     3816   4896   3654   -875  -1098    367       C
ATOM   2063  CD1 LEU A 129     -12.436  22.953   7.828  1.00 32.77           C  0.030
ANISOU 2063  CD1 LEU A 129     3973   4872   3605   -919   -880    266       C
ATOM   2064  CD2 LEU A 129     -11.897  20.505   7.982  1.00 33.00           C  0.030
ANISOU 2064  CD2 LEU A 129     3822   4954   3763   -725  -1021    541       C
ATOM   2065  OXT LEU A 129     -16.405  19.422   8.519  1.00 38.87           O  0.033
ANISOU 2065  OXT LEU A 129     4102   5325   5341   -832   -767     53       O
ATOM   2066  H   LEU A 129     -15.411  20.658   5.077  1.00 40.30           H  0.033
ATOM   2067  HA  LEU A 129     -14.215  19.223   7.046  1.00 42.07           H  0.034
ATOM   2068  HB2 LEU A 129     -14.790  21.973   7.299  1.00 40.72           H  0.033
ATOM   2069  HB3 LEU A 129     -14.362  21.058   8.518  1.00 40.72           H  0.033
ATOM   2070  HG  LEU A 129     -12.621  21.541   6.363  1.00 39.06           H  0.033
ATOM   2071 HD11 LEU A 129     -11.499  23.125   7.645  1.00 39.32           H  0.033
ATOM   2072 HD12 LEU A 129     -12.988  23.607   7.372  1.00 39.32           H  0.033
ATOM   2073 HD13 LEU A 129     -12.599  22.990   8.783  1.00 39.32           H  0.033
ATOM   2074 HD21 LEU A 129     -10.970  20.775   7.889  1.00 39.60           H  0.033
ATOM   2075 HD22 LEU A 129     -12.130  20.440   8.921  1.00 39.60           H  0.033
ATOM   2076 HD23 LEU A 129     -12.038  19.650   7.546  1.00 39.60           H  0.033
TER
HETATM 2077 CL    CL A1131     -10.682  29.707  11.879  1.00 20.89          Cl  0.024
ANISOU 2077 CL    CL A1131     2321   3329   2287    766     -9    211      Cl
HETATM 2078 CL    CL A1132      13.442  13.313  32.572  1.00 19.50          Cl  0.023
ANISOU 2078 CL    CL A1132     2734   2524   2153    502    349    317      Cl
```

```
HETATM 2079 CL    CL A1134       0.496  25.305   3.881  1.00 40.69          Cl  0.033
ANISOU 2079 CL    CL A1134     5576   6124   3760    689    328   2092       Cl
HETATM 2080 CL    CL A1135       7.413   7.561  29.742  1.00 35.33          Cl  0.031
ANISOU 2080 CL    CL A1135     5689   3013   4723     47  -1296    880       Cl
HETATM 2081 CL    CL A1137      20.174  11.813  24.470  1.00 28.88          Cl  0.028
ANISOU 2081 CL    CL A1137     4422   3374   3176   -392    523   -109       Cl
HETATM 2082 NA    NA A1138       9.591  15.868  31.428  1.00 18.51          Na  0.023
ANISOU 2082 NA    NA A1138     2584   2624   1826    538    212    145       Na
HETATM 2083  O   HOH A2001       7.304  12.699   9.080  1.00 17.40           O  0.022
ANISOU 2083  O   HOH A2001     2320   2201   2092    772   -606   -507        O
HETATM 2084  O   HOH A2002       4.691   9.030  10.682  1.00 32.51           O  0.030
ANISOU 2084  O   HOH A2002     4175   2479   5700    141  -2617     61        O
HETATM 2085  O   HOH A2003       4.280  12.115   5.109  1.00 33.58           O  0.030
ANISOU 2085  O   HOH A2003     5645   3906   3206   -358    463  -1976        O
HETATM 2086  O   HOH A2004       2.062  19.977   4.169  1.00 28.63           O  0.028
ANISOU 2086  O   HOH A2004     3009   5231   2638    119   -201  -1219        O
HETATM 2087  O   HOH A2007      -0.356  21.805   2.626  1.00 42.69           O  0.034
ANISOU 2087  O   HOH A2007     7982   4070   4168   1098   2726   1257        O
HETATM 2088  O   HOH A2008      -4.263  21.045   1.871  1.00 27.18           O  0.027
ANISOU 2088  O   HOH A2008     2908   5349   2070    277   -228   -813        O
HETATM 2089  O   HOH A2012      -4.601  18.508   2.672  1.00 25.19           O  0.026
ANISOU 2089  O   HOH A2012     2787   4739   2047    846   -353   -487        O
HETATM 2090  O   HOH A2013       0.019  13.500   4.513  1.00 36.48           O  0.032
ANISOU 2090  O   HOH A2013     5421   4678   3762   -874   -719   -636        O
HETATM 2091  O   HOH A2025     -11.135  18.487  27.469  1.00 35.13           O  0.031
ANISOU 2091  O   HOH A2025     4019   5013   4314   -538    673   2019        O
HETATM 2092  O   HOH A2027     -13.628  24.312  25.174  1.00 26.10           O  0.027
ANISOU 2092  O   HOH A2027     2526   3962   3430   -650    932    506        O
HETATM 2093  O   HOH A2028     -10.130  32.002  23.936  1.00 18.31           O  0.022
ANISOU 2093  O   HOH A2028     2466   2334   2155    345    344     -3        O
HETATM 2094  O   HOH A2029      16.449  13.139  34.068  1.00 34.15           O  0.031
ANISOU 2094  O   HOH A2029     6038   2810   4127    526  -2709    134        O
HETATM 2095  O   HOH A2030      -5.865  34.574  16.819  1.00 22.01           O  0.025
ANISOU 2095  O   HOH A2030     4430   2031   1900    431   -492   -165        O
HETATM 2096  O   HOH A2031      -9.003  33.883  15.993  1.00 27.54           O  0.027
ANISOU 2096  O   HOH A2031     5019   2976   2468   1864   1058    355        O
HETATM 2097  O   HOH A2034      -0.204   7.237  22.689  1.00 37.27           O  0.032
ANISOU 2097  O   HOH A2034     5473   4053   4635  -1896    197    878        O
HETATM 2098  O   HOH A2036       6.785  26.334   9.003  1.00 32.56           O  0.030
ANISOU 2098  O   HOH A2036     3515   3438   5416    -80   2076    267        O
HETATM 2099  O   HOH A2037      -2.849  10.674  25.201  1.00 30.89           O  0.029
ANISOU 2099  O   HOH A2037     4036   2993   4706   -851    408    733        O
HETATM 2100  O   HOH A2038       8.498  27.785  15.543  1.00 19.18           O  0.023
ANISOU 2100  O   HOH A2038     2390   2739   2160   -530   -228     -7        O
HETATM 2101  O   HOH A2039       8.296  26.659  11.332  1.00 21.66           O  0.024
ANISOU 2101  O   HOH A2039     2470   3043   2716   -699    512   -186        O
HETATM 2102  O   HOH A2041       8.602  25.966  19.730  1.00 23.53           O  0.025
ANISOU 2102  O   HOH A2041     2422   3584   2934     35    -78   -823        O
HETATM 2103  O   HOH A2043       6.663  28.331  19.941  1.00 19.21           O  0.023
ANISOU 2103  O   HOH A2043     2897   2325   2076    350   -232    -87        O
HETATM 2104  O   HOH A2044       9.634  20.198  10.795  1.00 25.91           O  0.027
ANISOU 2104  O   HOH A2044     2525   4471   2849    710     49    256        O
HETATM 2105  O   HOH A2045       4.413  21.212   5.170  1.00 25.54           O  0.026
ANISOU 2105  O   HOH A2045     3500   4356   1850     -6    -81   -681        O
HETATM 2106  O   HOH A2046       7.447  24.003   3.162  1.00 40.43           O  0.033
ANISOU 2106  O   HOH A2046     6372   6239   2750  -2020   -914    554        O
HETATM 2107  O   HOH A2047      -0.750  40.356  19.326  1.00 21.26           O  0.024
ANISOU 2107  O   HOH A2047     3069   2475   2536    205    240    112        O
HETATM 2108  O   HOH A2049      -2.005  36.869   7.388  1.00 29.94           O  0.029
ANISOU 2108  O   HOH A2049     4000   4024   3352  -1518   -848    198        O
HETATM 2109  O   HOH A2050      16.295  17.405  17.484  0.81 20.72           O  0.024
ANISOU 2109  O   HOH A2050     1957   2565   3352    -57    -26   1061        O
HETATM 2110  O   HOH A2051      13.008  14.996  14.567  1.00 15.14           O  0.020
ANISOU 2110  O   HOH A2051     2214   2165   1373    481   -254   -140        O
HETATM 2111  O   HOH A2052       2.149  32.088   5.742  1.00 37.22           O  0.032
ANISOU 2111  O   HOH A2052     3383   7240   3519  -2313   -545   1588        O
HETATM 2112  O   HOH A2055      17.319  20.014  17.743  1.00 20.74           O  0.024
ANISOU 2112  O   HOH A2055     3563   2399   1917    421   1125      4        O
HETATM 2113  O   HOH A2057      16.800  26.994  22.795  1.00 34.35           O  0.031
ANISOU 2113  O   HOH A2057     6912   2539   3599   -126   -780    624        O
HETATM 2114  O   HOH A2058      15.908  26.725  26.545  1.00 34.26           O  0.031
ANISOU 2114  O   HOH A2058     7335   3131   2552    -11   -457   -927        O
HETATM 2115  O   HOH A2067       3.033  18.026  19.454  1.00 13.44           O  0.019
ANISOU 2115  O   HOH A2067     1615   1998   1493    217    -91    102        O
HETATM 2116  O   HOH A2068      10.367  24.173  18.518  1.00 21.12           O  0.024
ANISOU 2116  O   HOH A2068     2240   2344   3440   -144   -359   -568        O
HETATM 2117  O   HOH A2069       6.623  25.029  21.337  1.00 28.65           O  0.028
ANISOU 2117  O   HOH A2069     4650   2368   3868    386   1152    151        O
```

```
HETATM 2118  O   HOH A2070       5.469  23.133  25.066  1.00 28.00           O  0.028
ANISOU 2118  O   HOH A2070     3921   2497   4221   1105  -1321   -586        O
HETATM 2119  O   HOH A2074       4.863  25.320  28.135  1.00 38.23           O  0.032
ANISOU 2119  O   HOH A2074     6614   2696   5216    -13   2126    885        O
HETATM 2120  O   HOH A2075      12.257  10.662  33.887  1.00 41.06           O  0.034
ANISOU 2120  O   HOH A2075     6153   6425   3021   2359    789    683        O
HETATM 2121  O   HOH A2076      12.232   7.443  30.954  1.00 20.43           O  0.024
ANISOU 2121  O   HOH A2076     2944   2519   2298   1009   1025    491        O
HETATM 2122  O   HOH A2077      11.379  14.486  30.451  1.00 16.39           O  0.021
ANISOU 2122  O   HOH A2077     2572   2299   1357    535    307    225        O
HETATM 2123  O   HOH A2078      19.067   9.778  28.808  1.00 20.20           O  0.024
ANISOU 2123  O   HOH A2078     2686   2438   2550    614   -342   -363        O
HETATM 2124  O   HOH A2079      16.315   9.023  33.190  1.00 29.74           O  0.029
ANISOU 2124  O   HOH A2079     6035   2886   2379    850    281    671        O
HETATM 2125  O   HOH A2081      14.219  14.219  37.221  0.58 35.77           O  0.031
ANISOU 2125  O   HOH A2081     3964   4083   5545     -7    495  -1168        O
HETATM 2126  O   HOH A2082       9.533  13.935  32.736  1.00 24.64           O  0.026
ANISOU 2126  O   HOH A2082     2420   5299   1645    996    127    251        O
HETATM 2127  O   HOH A2084       7.951  19.267  41.582  1.00 36.59           O  0.032
ANISOU 2127  O   HOH A2084     5570   5603   2729   -368    500   -920        O
HETATM 2128  O   HOH A2085       4.510   8.392  30.622  1.00 29.09           O  0.028
ANISOU 2128  O   HOH A2085     5634   2467   2953    -51    287   1011        O
HETATM 2129  O   HOH A2086       6.964  10.393  34.193  1.00 28.56           O  0.028
ANISOU 2129  O   HOH A2086     4048   3912   2890    745   -636    645        O
HETATM 2130  O   HOH A2087      -2.303  13.841  27.961  1.00 24.75           O  0.026
ANISOU 2130  O   HOH A2087     2363   3939   3102   -266    202    920        O
HETATM 2131  O   HOH A2090       5.524   7.601  18.141  1.00 25.79           O  0.027
ANISOU 2131  O   HOH A2090     4082   2606   3111     -8   -481   -198        O
HETATM 2132  O   HOH A2091       4.906   6.275  21.786  1.00 38.03           O  0.032
ANISOU 2132  O   HOH A2091     6722   3183   4543    710   -414     57        O
HETATM 2133  O   HOH A2092       0.736   8.364  20.237  1.00 24.99           O  0.026
ANISOU 2133  O   HOH A2092     2976   2709   3812     99   -264    -79        O
HETATM 2134  O   HOH A2093       2.623  15.340  16.622  1.00 24.05           O  0.026
ANISOU 2134  O   HOH A2093     3611   2750   2776  -1241  -1373    774        O
HETATM 2135  O   HOH A2094       5.738   8.990  13.200  1.00 23.37           O  0.025
ANISOU 2135  O   HOH A2094     3006   2732   3143    892   -702   -516        O
HETATM 2136  O   HOH A2095       0.863  13.438  16.222  1.00 17.78           O  0.022
ANISOU 2136  O   HOH A2095     2137   1937   2682   -140   -163   -293        O
HETATM 2137  O   HOH A2096       3.199   6.747  13.859  1.00 35.20           O  0.031
ANISOU 2137  O   HOH A2096     5104   2881   5389   1137  -1323  -1741        O
HETATM 2138  O   HOH A2097      -2.928  10.328  10.722  1.00 32.52           O  0.030
ANISOU 2138  O   HOH A2097     3232   5207   3917   -364   -862   -821        O
HETATM 2139  O   HOH A2098      -1.487   9.501  19.352  1.00 24.90           O  0.026
ANISOU 2139  O   HOH A2098     3042   2098   4321    246   -324   -468        O
HETATM 2140  O   HOH A2099       0.398  12.421  18.847  1.00 20.34           O  0.024
ANISOU 2140  O   HOH A2099     2233   2988   2509    466   -380    -65        O
HETATM 2141  O   HOH A2102      -1.490  13.093  25.538  1.00 22.78           O  0.025
ANISOU 2141  O   HOH A2102     3045   2474   3136    -90     83    923        O
HETATM 2142  O   HOH A2103      -6.680  16.516  27.650  1.00 28.39           O  0.028
ANISOU 2142  O   HOH A2103     2832   4768   3186   -303    997    625        O
HETATM 2143  O   HOH A2105      -7.603  13.552  20.659  1.00 27.29           O  0.027
ANISOU 2143  O   HOH A2105     3388   3092   3890   -753   -390    507        O
HETATM 2144  O   HOH A2106      -9.447  14.903  23.863  1.00 29.46           O  0.028
ANISOU 2144  O   HOH A2106     2909   2743   5540    -39     27   1291        O
HETATM 2145  O   HOH A2107      -7.844  20.345  29.900  1.00 41.39           O  0.034
ANISOU 2145  O   HOH A2107     4402   3503   7820   -311   2094    709        O
HETATM 2146  O   HOH A2108      -4.836  22.522  32.202  1.00 31.35           O  0.029
ANISOU 2146  O   HOH A2108     4601   4988   2323    610    684   1071        O
HETATM 2147  O   HOH A2109      -5.665  29.047  29.472  1.00 18.17           O  0.022
ANISOU 2147  O   HOH A2109     2613   2423   1866     98    473    548        O
HETATM 2148  O   HOH A2110      -1.768  28.325  28.531  1.00 25.32           O  0.026
ANISOU 2148  O   HOH A2110     3496   3661   2462   1384    516    848        O
HETATM 2149  O   HOH A2111       4.271  33.525  24.278  1.00 37.29           O  0.032
ANISOU 2149  O   HOH A2111     5248   4779   4142  -2309   -884    453        O
HETATM 2150  O   HOH A2112       6.472  27.803  22.685  1.00 25.87           O  0.027
ANISOU 2150  O   HOH A2112     2910   4399   2521   1407   -280    320        O
HETATM 2151  O   HOH A2114       1.623  39.060  18.497  1.00 28.23           O  0.028
ANISOU 2151  O   HOH A2114     3820   2226   4681    401    -70   -283        O
HETATM 2152  O   HOH A2115       4.579  39.319  19.118  1.00 35.97           O  0.031
ANISOU 2152  O   HOH A2115     7310   3170   3188   1422   -765   -311        O
HETATM 2153  O   HOH A2116       8.762  29.635   8.507  1.00 17.39           O  0.022
ANISOU 2153  O   HOH A2116     2259   2094   2255   -190    226   -221        O
HETATM 2154  O   HOH A2117       2.058  34.973   9.898  1.00 17.94           O  0.022
ANISOU 2154  O   HOH A2117     2322   2664   1830   -355    181    461        O
HETATM 2155  O   HOH A2118       4.870  34.401   9.936  1.00 15.48           O  0.021
ANISOU 2155  O   HOH A2118     1962   1929   1991   -405     30    244        O
HETATM 2156  O   HOH A2119       1.142  37.395  15.424  1.00 15.77           O  0.021
ANISOU 2156  O   HOH A2119     2171   2035   1784   -161     97    259        O
```

```
HETATM 2157  O   HOH A2120      -1.194  40.206  14.591  1.00 26.98           O  0.027
ANISOU 2157  O   HOH A2120     3680   2539   4033    274    502   1013       O
HETATM 2158  O   HOH A2121       0.116  39.766  22.002  1.00 19.92           O  0.023
ANISOU 2158  O   HOH A2121     2653   2379   2535   -500    299    -37       O
HETATM 2159  O   HOH A2122      -0.165  37.934  25.559  1.00 29.90           O  0.029
ANISOU 2159  O   HOH A2122     4054   3888   3420    757  -1051  -2009       O
HETATM 2160  O   HOH A2123      -3.405  40.243  18.264  1.00 21.05           O  0.024
ANISOU 2160  O   HOH A2123     3023   3249   1727    212    145   -424       O
HETATM 2161  O   HOH A2127      -2.264  33.592   9.609  1.00 18.79           O  0.023
ANISOU 2161  O   HOH A2127     3004   2380   1755   -189     93   -141       O
HETATM 2162  O   HOH A2128      -6.948  36.113  14.883  1.00 20.12           O  0.023
ANISOU 2162  O   HOH A2128     3533   2175   1938    559    175    271       O
HETATM 2163  O   HOH A2129      -3.863  38.450   8.407  1.00 21.11           O  0.024
ANISOU 2163  O   HOH A2129     2928   3198   1895   -318    -89    701       O
HETATM 2164  O   HOH A2130      -3.642  34.437   7.409  1.00 28.80           O  0.028
ANISOU 2164  O   HOH A2130     3756   3759   3427   -600    466   -200       O
HETATM 2165  O   HOH A2131      -9.829  34.834  11.562  1.00 22.01           O  0.025
ANISOU 2165  O   HOH A2131     2895   2430   3038    345   -855   -208       O
HETATM 2166  O   HOH A2134       0.286  33.445   8.312  1.00 25.80           O  0.027
ANISOU 2166  O   HOH A2134     3402   3629   2771    252   -686    158       O
HETATM 2167  O   HOH A2136     -20.201  13.563   1.833  1.00 24.92           O  0.026
ANISOU 2167  O   HOH A2136     3574   1992   3903   -336   -224   -327       O
HETATM 2168  O   HOH A2137     -13.500  16.583   6.317  1.00 43.72           O  0.035
ANISOU 2168  O   HOH A2137     4427   5185   6999  -1089  -2175    147       O
HETATM 2169  O   HOH A2138     -10.370  18.884   4.909  1.00 32.77           O  0.030
ANISOU 2169  O   HOH A2138     4198   4150   4102    -96  -1928     11       O
HETATM 2170  O   HOH A2139     -10.143  19.469   1.346  1.00 31.35           O  0.029
ANISOU 2170  O   HOH A2139     3731   4650   3532    288   -876  -1389       O
HETATM 2171  C1 ARII A2148       1.190  22.942  36.475  0.29 14.79      A    C  0.020
ANISOU 2171  C1 ARII A2148     2426   2050   1144    556    161    551  A    C
HETATM 2172  C2 ARII A2148      -1.410  23.422  36.328  0.29 17.43      A    C  0.022
ANISOU 2172  C2 ARII A2148     2496   2318   1808    563    831    269  A    C
HETATM 2173  C3 ARII A2148      -0.303  22.279  34.346  0.29 17.60      A    C  0.022
ANISOU 2173  C3 ARII A2148     2592   2164   1932    397    538    339  A    C
HETATM 2174  C4 ARII A2148       1.697  25.271  32.870  0.29 13.52      A    C  0.019
ANISOU 2174  C4 ARII A2148     1931   1920   1287    307    387    -73  A    C
HETATM 2175  C5 ARII A2148       3.792  24.803  33.142  0.29 12.20      A    C  0.018
ANISOU 2175  C5 ARII A2148     1839   1732   1063    276     53   -202  A    C
HETATM 2176  C6 ARII A2148       3.103  24.228  34.111  0.29 12.85      A    C  0.019
ANISOU 2176  C6 ARII A2148     1935   1806   1140    366    151    -80  A    C
HETATM 2177  C7 ARII A2148      -0.109  26.988  35.685  0.29 16.58      A    C  0.021
ANISOU 2177  C7 ARII A2148     2618   1641   2041    812    515    193  A    C
HETATM 2178  C8 ARII A2148       1.127  27.425  37.394  0.29 18.44      A    C  0.022
ANISOU 2178  C8 ARII A2148     2705   2034   2268    600    409    466  A    C
HETATM 2179  C9 ARII A2148       1.174  26.105  37.153  0.29 17.25      A    C  0.022
ANISOU 2179  C9 ARII A2148     2586   1931   2039    713    324    585  A    C
HETATM 2180  N1 ARII A2148       1.752  24.508  33.958  0.29 14.62      A    N  0.020
ANISOU 2180  N1 ARII A2148     2135   1947   1474    348    236     79  A    N
HETATM 2181  N2 ARII A2148       2.907  25.457  32.323  0.29 11.29      A    N  0.018
ANISOU 2181  N2 ARII A2148     1803   1748    738    248    408   -264  A    N
HETATM 2182  N3 ARII A2148       0.403  25.821  36.046  0.29 14.34      A    N  0.020
ANISOU 2182  N3 ARII A2148     2427   1660   1362    769    577    565  A    N
HETATM 2183  N4 ARII A2148       0.340  27.963  36.487  0.29 18.60      A    N  0.023
ANISOU 2183  N4 ARII A2148     2766   1972   2329    618    404    361  A    N
HETATM 2184  O1 ARII A2148       1.838  22.364  37.247  0.29 15.58      A    O  0.021
ANISOU 2184  O1 ARII A2148     2508   2162   1250    456     29    587  A    O
HETATM 2185  O2 ARII A2148      -2.321  23.108  36.945  0.29 19.47      A    O  0.023
ANISOU 2185  O2 ARII A2148     2672   2507   2217    530    935    235  A    O
HETATM 2186  O3 ARII A2148      -0.594  21.300  33.845  0.29 18.03      A    O  0.022
ANISOU 2186  O3 ARII A2148     2675   2129   2047    309    674    201  A    O
HETATM 2187 RE1 ARII A2148       0.111  23.887  35.254  0.29 15.89      A   Re  0.021
ANISOU 2187 RE1 ARII A2148     2423   2096   1516    505    545    259  A   Re
HETATM 2188  H21ARII A2148       3.092  25.913  31.618  0.29 13.55      A    H  0.019
HETATM 2189  H41ARII A2148       0.917  25.617  32.505  0.29 16.23      A    H  0.021
HETATM 2190  H42ARII A2148       0.142  28.797  36.421  0.29 22.32      A    H  0.025
HETATM 2191  H51ARII A2148       4.710  24.746  33.018  0.29 14.64      A    H  0.020
HETATM 2192  H61ARII A2148       3.469  23.720  34.799  0.29 15.42      A    H  0.021
HETATM 2193  H71ARII A2148      -0.679  27.118  34.961  0.29 19.90      A    H  0.023
HETATM 2194  H81ARII A2148       1.570  27.876  38.074  0.29 22.13      A    H  0.025
HETATM 2195  H91ARII A2148       1.663  25.484  37.641  0.29 20.71      A    H  0.024
HETATM 2196  C1 BRII A2148       1.637  23.755  36.710  0.29 31.93      A    C  0.030
ANISOU 2196  C1 BRII A2148     3081   4659   4393    458   -740   1198  A    C
HETATM 2197  C2 BRII A2148      -1.021  24.060  36.815  0.29 32.42      A    C  0.030
ANISOU 2197  C2 BRII A2148     3039   4668   4611    378   -737   1097  A    C
HETATM 2198  C3 BRII A2148      -0.057  22.697  34.869  0.29 32.51      A    C  0.030
ANISOU 2198  C3 BRII A2148     3045   4640   4669    437   -762   1181  A    C
HETATM 2199  C4 BRII A2148       2.014  26.050  33.427  0.29 31.42      A    C  0.029
ANISOU 2199  C4 BRII A2148     2944   4716   4279    611   -861   1015  A    C
```

```
HETATM 2200  C5 BRII A2148       3.687  24.864  32.734  0.29 31.90      A    C  0.030
ANISOU 2200  C5 BRII A2148     3024   4753   4346    590   -797    973  A    C
HETATM 2201  C6 BRII A2148       2.999  24.162  33.625  0.29 31.78      A    C  0.029
ANISOU 2201  C6 BRII A2148     3028   4742   4303    583   -769    979  A    C
HETATM 2202  C7 BRII A2148       0.107  27.580  35.836  0.29 36.13      A    C  0.031
ANISOU 2202  C7 BRII A2148     3450   4922   5355    510   -855   1274  A    C
HETATM 2203  C8 BRII A2148       1.483  28.198  37.375  0.29 36.84      A    C  0.032
ANISOU 2203  C8 BRII A2148     3508   4951   5538    530   -855   1294  A    C
HETATM 2204  C9 BRII A2148       1.566  26.862  37.220  0.29 35.98      A    C  0.031
ANISOU 2204  C9 BRII A2148     3422   4902   5346    541   -869   1321  A    C
HETATM 2205  N1 BRII A2148       1.906  24.910  34.068  0.29 31.45      A    N  0.029
ANISOU 2205  N1 BRII A2148     2985   4714   4249    546   -771    969  A    N
HETATM 2206  N2 BRII A2148       3.046  26.064  32.594  0.29 31.82      A    N  0.030
ANISOU 2206  N2 BRII A2148     3000   4743   4348    600   -854   1014  A    N
HETATM 2207  N3 BRII A2148       0.680  26.467  36.228  0.29 34.41      A    N  0.031
ANISOU 2207  N3 BRII A2148     3288   4828   4958    525   -841   1274  A    N
HETATM 2208  N4 BRII A2148       0.585  28.627  36.514  0.29 36.81      A    N  0.032
ANISOU 2208  N4 BRII A2148     3517   4961   5509    504   -867   1270  A    N
HETATM 2209  O1 BRII A2148       2.476  23.370  37.437  0.29 32.69      A    O  0.030
ANISOU 2209  O1 BRII A2148     3198   4685   4537    443   -743   1307  A    O
HETATM 2210  O2 BRII A2148      -1.829  23.801  37.571  0.29 33.38      A    O  0.030
ANISOU 2210  O2 BRII A2148     3103   4705   4874    325   -829   1118  A    O
HETATM 2211  O3 BRII A2148      -0.323  21.655  34.529  0.29 33.60      A    O  0.030
ANISOU 2211  O3 BRII A2148     3094   4692   4979    428   -825   1276  A    O
HETATM 2212 RE1 BRII A2148       0.338  24.442  35.517  0.29 30.96      A   Re  0.029
ANISOU 2212 RE1 BRII A2148     3022   4610   4133    439   -641   1084  A   Re
HETATM 2213  H21BRII A2148       3.285  26.716  32.089  0.29 38.19      A    H  0.032
HETATM 2214  H41BRII A2148       1.408  26.750  33.515  0.29 37.71      A    H  0.032
HETATM 2215  H42BRII A2148       0.340  29.445  36.408  0.29 44.18      A    H  0.035
HETATM 2216  H51BRII A2148       4.451  24.589  32.285  0.29 38.29      A    H  0.032
HETATM 2217  H61BRII A2148       3.206  23.299  33.903  0.29 38.13      A    H  0.032
HETATM 2218  H71BRII A2148      -0.541  27.635  35.171  0.29 43.35      A    H  0.034
HETATM 2219  H81BRII A2148       1.971  28.718  37.971  0.29 44.21      A    H  0.035
HETATM 2220  H91BRII A2148       2.124  26.296  37.700  0.29 43.17      A    H  0.034
HETATM 2221  C1  RII A2149     -12.012  35.736   4.316  0.58 18.24      A    C  0.022
ANISOU 2221  C1  RII A2149     2642   2434   1854    174   -756    186  A    C
HETATM 2222  C2  RII A2149      -9.366  36.174   3.907  0.58 17.54      A    C  0.022
ANISOU 2222  C2  RII A2149     2501   2409   1756    109    -81    -12  A    C
HETATM 2223  C3  RII A2149     -10.272  34.167   5.363  0.58 17.73      A    C  0.022
ANISOU 2223  C3  RII A2149     2600   2261   1876    128   -934     91  A    C
HETATM 2224  C4  RII A2149     -11.412  36.192   8.420  0.58 20.22      A    C  0.024
ANISOU 2224  C4  RII A2149     2617   2688   2377   -103   -386     50  A    C
HETATM 2225  C5  RII A2149     -13.532  35.693   8.487  0.58 21.57      A    C  0.024
ANISOU 2225  C5  RII A2149     2715   2956   2523   -152   -271     59  A    C
HETATM 2226  C6  RII A2149     -13.124  35.648   7.236  0.58 20.51      A    C  0.024
ANISOU 2226  C6  RII A2149     2637   2861   2297    -50   -482     33  A    C
HETATM 2227  C7  RII A2149     -10.274  39.088   6.210  0.58 18.86      A    C  0.023
ANISOU 2227  C7  RII A2149     2766   2286   2115    -19   -909    164  A    C
HETATM 2228  C8  RII A2149     -11.457  40.261   4.846  0.58 19.40      A    C  0.023
ANISOU 2228  C8  RII A2149     2677   2260   2436   -155   -524    -27  A    C
HETATM 2229  C9  RII A2149     -11.532  38.952   4.511  0.58 18.50      A    C  0.022
ANISOU 2229  C9  RII A2149     2663   2158   2208     -9   -577    106  A    C
HETATM 2230  N1  RII A2149     -11.774  35.976   7.175  0.58 19.03      A    N  0.023
ANISOU 2230  N1  RII A2149     2537   2535   2160     68   -847    217  A    N
HETATM 2231  N2  RII A2149     -12.452  36.048   9.234  0.58 21.13      A    N  0.024
ANISOU 2231  N2  RII A2149     2678   2888   2462   -187   -196     99  A    N
HETATM 2232  N3  RII A2149     -10.798  38.198   5.409  0.58 17.48      A    N  0.022
ANISOU 2232  N3  RII A2149     2688   2168   1785     92   -672    218  A    N
HETATM 2233  N4  RII A2149     -10.672  40.321   5.898  0.58 20.00      A    N  0.023
ANISOU 2233  N4  RII A2149     2863   2369   2365   -110   -654     53  A    N
HETATM 2234  O1  RII A2149     -12.942  35.536   3.626  0.58 19.33      A    O  0.023
ANISOU 2234  O1  RII A2149     2798   2597   1950    218   -891    250  A    O
HETATM 2235  O2  RII A2149      -8.673  36.245   2.979  0.58 20.22      A    O  0.024
ANISOU 2235  O2  RII A2149     2760   2610   2314    153    125    127  A    O
HETATM 2236  O3  RII A2149     -10.178  33.043   5.293  0.58 19.03      A    O  0.023
ANISOU 2236  O3  RII A2149     2566   2261   2402    240   -965    -33  A    O
HETATM 2237 RE1  RII A2149     -10.532  36.041   5.416  0.58 17.19      A   Re  0.022
ANISOU 2237 RE1  RII A2149     2522   2245   1764    201   -682    140  A   Re
HETATM 2238  H21 RII A2149     -12.441  36.151  10.088  0.58 25.36      A    H  0.026
HETATM 2239  H41 RII A2149     -10.556  36.433   8.690  0.58 24.26      A    H  0.026
HETATM 2240  H42 RII A2149     -10.440  41.039   6.311  0.58 24.00      A    H  0.026
HETATM 2241  H51 RII A2149     -14.392  35.531   8.796  0.58 25.88      A    H  0.027
HETATM 2242  H61 RII A2149     -13.657  35.433   6.505  0.58 24.62      A    H  0.026
HETATM 2243  H71 RII A2149      -9.723  38.885   6.931  0.58 22.63      A    H  0.025
HETATM 2244  H81 RII A2149     -11.860  40.974   4.408  0.58 23.28      A    H  0.025
HETATM 2245  H91 RII A2149     -12.033  38.607   3.809  0.58 22.20      A    H  0.025
HETATM 2246  C1  RI3 A2152      -7.730   9.144  13.268  0.84 31.18           C  0.029
ANISOU 2246  C1  RI3 A2152     3645   2424   5779    -55  -1890   -433       C
```

```
HETATM 2247  C2  RI3 A2152      -8.395  11.664  13.137  0.84 26.33           C  0.027
ANISOU 2247  C2  RI3 A2152     3477   1995   4533     66  -2288   -332       C
HETATM 2248  C3  RI3 A2152      -6.060  10.935  13.982  0.84 26.00           C  0.027
ANISOU 2248  C3  RI3 A2152     3251   2073   4554   -506  -1436   -690       C
HETATM 2249  C7  RI3 A2152     -10.487  10.844  16.101  0.84 33.65           C  0.030
ANISOU 2249  C7  RI3 A2152     3483   2735   6568   -433  -1375   -467       C
HETATM 2250  C8  RI3 A2152     -11.865   9.355  15.439  0.84 33.90           C  0.030
ANISOU 2250  C8  RI3 A2152     3456   2806   6620   -326  -1352   -265       C
HETATM 2251  C9  RI3 A2152     -10.716   9.194  14.756  0.84 33.24           C  0.030
ANISOU 2251  C9  RI3 A2152     3454   2730   6445   -323  -1531   -301       C
HETATM 2252  N3  RI3 A2152      -9.832  10.147  15.203  0.84 32.02           N  0.030
ANISOU 2252  N3  RI3 A2152     3460   2535   6172   -305  -1634   -342       N
HETATM 2253  N4  RI3 A2152     -11.718  10.362  16.261  0.84 34.42           N  0.031
ANISOU 2253  N4  RI3 A2152     3483   2867   6726   -357  -1321   -339       N
HETATM 2254  O1  RI3 A2152      -7.697   8.285  12.456  0.84 32.23           O  0.030
ANISOU 2254  O1  RI3 A2152     3697   2731   5818   -118  -2063   -615       O
HETATM 2255  O2  RI3 A2152      -8.735  12.316  12.280  0.84 27.96           O  0.028
ANISOU 2255  O2  RI3 A2152     3670   2035   4917    -74  -2179   -457       O
HETATM 2256  O3  RI3 A2152      -4.995  11.209  13.649  0.84 27.08           O  0.027
ANISOU 2256  O3  RI3 A2152     3136   2324   4830   -401  -1517   -630       O
HETATM 2257  O4  RI3 A2152      -7.237   9.198  16.180  0.84 30.62           O  0.029
ANISOU 2257  O4  RI3 A2152     3508   2302   5822   -180  -1517   -167       O
HETATM 2258 RE1  RI3 A2152      -7.802  10.518  14.542  0.84 29.36          Re  0.028
ANISOU 2258 RE1  RI3 A2152     3451   2228   5475   -170  -1901   -361      Re
HETATM 2259  H42 RI3 A2152     -12.310  10.663  16.807  0.84 41.30           H  0.034
HETATM 2260  H71 RI3 A2152     -10.141  11.563  16.577  0.84 40.38           H  0.033
HETATM 2261  H81 RI3 A2152     -12.630   8.836  15.347  0.84 40.68           H  0.033
HETATM 2262  H91 RI3 A2152     -10.546   8.549  14.110  0.84 39.89           H  0.033
HETATM 2263 BR    BR A2169     -11.734   8.084  10.958  0.51 46.41          Br  0.036
ANISOU 2263 BR    BR A2169     3268   9598   4768    939     48   2157      Br
HETATM 2264 NA    NA A2176      15.346  16.608  26.260  1.00 19.06          Na  0.023
ANISOU 2264 NA    NA A2176     2335   2782   2125    334      4    208      Na
HETATM 2265 CL    CL A2180       7.362  19.470   6.940  1.00 43.18          Cl  0.034
ANISOU 2265 CL    CL A2180     3695   5635   7075   1265   -256  -2705      Cl
HETATM 2266  C2  IMD A2182     -11.813  39.538  12.708  0.60 37.82           C  0.032
HETATM 2267  C4  IMD A2182     -10.721  41.253  11.913  0.60 39.11           C  0.033
HETATM 2268  C5  IMD A2182     -11.955  41.669  12.233  0.60 30.97           C  0.029
HETATM 2269  N1  IMD A2182     -12.664  40.557  12.590  0.60 31.49           N  0.029
HETATM 2270  N3  IMD A2182     -10.647  39.928  12.235  0.60 42.41           N  0.034
HETATM 2271  H2  IMD A2182     -12.051  38.620  12.956  0.60 45.38           H  0.035
HETATM 2272  H4  IMD A2182      -9.983  41.815  11.597  0.60 46.93           H  0.036
HETATM 2273  H5  IMD A2182     -12.326  42.560  12.064  0.60 37.16           H  0.032
HETATM 2274  HN1 IMD A2182     -13.589  40.559  12.906  0.60 37.79           H  0.032
HETATM 2275  HN3 IMD A2182      -9.846  39.368  12.161  0.60 50.89           H  0.037
HETATM 2276  O   HOH W   1       2.729  26.807  29.513  1.00 20.61           O  0.024
ANISOU 2276  O   HOH W   1     2921   2809   2100     11   -467    -90       O
HETATM 2277  O   HOH W   2      -7.232  38.385   5.280  1.00 28.76           O  0.028
ANISOU 2277  O   HOH W   2     4303   3676   2947   -903  -1574   1246       O
HETATM 2278  O   HOH W   5       3.632  24.908  24.685  1.00 38.38           O  0.032
ANISOU 2278  O   HOH W   5     4474   3268   6839   1007   2419    770       O
HETATM 2279  O   HOH W   6     -10.683  38.020   1.026  1.00 21.22           O  0.024
ANISOU 2279  O   HOH W   6     2620   3832   1612   -639   -582    708       O
HETATM 2280  O   HOH W   7      14.001   8.096  33.258  1.00 33.62           O  0.030
ANISOU 2280  O   HOH W   7     6798   3339   2640   1280   1439    860       O
HETATM 2281  O   HOH W   8      -0.171  43.006  18.918  1.00 28.98           O  0.028
ANISOU 2281  O   HOH W   8     3388   3989   3633    817    -58    524       O
HETATM 2282  O   HOH W   9      -2.738  30.143   4.887  1.00 35.16           O  0.031
ANISOU 2282  O   HOH W   9     4359   4966   4034   -886  -2177   1575       O
HETATM 2283  O   HOH W  11      20.054  20.054  18.610  1.00 34.33           O  0.031
ANISOU 2283  O   HOH W  11     3224   3224   6595     21   1694  -1694       O
HETATM 2284  O   HOH W  12     -13.084  15.593  21.269  1.00 36.55           O  0.032
ANISOU 2284  O   HOH W  12     3480   3966   6440  -1062   -150    670       O
HETATM 2285  O   HOH W  13      -0.026  26.663  29.904  1.00 35.48           O  0.031
ANISOU 2285  O   HOH W  13     3913   6541   3027   2201   -663  -1191       O
HETATM 2286  O   HOH W  14       9.030  28.422  18.361  1.00 33.21           O  0.030
ANISOU 2286  O   HOH W  14     3349   5470   3800    372    -49    971       O
HETATM 2287  O   HOH W  19       4.548  15.371  36.641  1.00 36.79           O  0.032
HETATM 2288  O   HOH W  20      -9.114  39.377   9.692  1.00 34.35           O  0.031
HETATM 2289  O   HOH W  21     -10.738  23.750  28.907  1.00 30.81           O  0.029
HETATM 2290 RE    RE B   1      11.358  25.751  22.386  0.20 52.92          Re  0.038
HETATM 2291 RE    RE B   2     -15.401  25.373  12.402  0.21 23.95          Re  0.026
HETATM 2292 RE    RE B   3     -15.963  16.177   8.308  0.26 27.21          Re  0.027
HETATM 2293 RE    RE B   4     -10.644  10.644   9.305  0.10 18.04          Re  0.022
HETATM 2294 RE    RE B   5     -11.337  36.862  11.641  0.11 28.33          Re  0.028
HETATM 2295 RE    RE B   6      15.227  21.797  33.901  0.13 17.85          Re  0.022
HETATM 2296 CL    CL C   1      -7.821  32.341  26.266  1.00 14.70          Cl  0.020
END
```
