# Supplementary material for: Time-series analysis of rhenium(I) organometallic covalent binding to a model protein for drug development
Source: IUCrJ. 2024 Apr 19;11(Pt 3):359–73. doi: 10.1107/S2052252524002598 (PMC11067751; doi:10.1107/S2052252524002598)
Supplement: Supplementary file 7 [file m-11-00359-sup7.zip › Week 38 - P5P1_1 - 1-54/P5P1_2_refine_19.pdf]

```
REMARK   3
REMARK   3 REFINEMENT.
REMARK   3   PROGRAM     : PHENIX (1.20.1_4487: ???)
REMARK   3   AUTHORS     : Adams,Afonine,Bunkoczi,Burnley,Chen,Dar,Davis,
REMARK   3               : Draizen,Echols,Gildea,Gros,Grosse-Kunstleve,Headd,
REMARK   3               : Hintze,Hung,Ioerger,Liebschner,McCoy,McKee,Moriarty,
REMARK   3               : Oeffner,Poon,Read,Richardson,Richardson,Sacchettini,
REMARK   3               : Sauter,Sobolev,Storoni,Terwilliger,Williams,Zwart
REMARK   3
REMARK   3  X-RAY DATA.
REMARK   3
REMARK   3  REFINEMENT TARGET : ML
REMARK   3
REMARK   3  DATA USED IN REFINEMENT.
REMARK   3   RESOLUTION RANGE HIGH (ANGSTROMS) : 1.76
REMARK   3   RESOLUTION RANGE LOW  (ANGSTROMS) : 57.37
REMARK   3   MIN(FOBS/SIGMA_FOBS)              : 1.41
REMARK   3   COMPLETENESS FOR RANGE        (%) : 96.64
REMARK   3   NUMBER OF REFLECTIONS             : 22741
REMARK   3   NUMBER OF REFLECTIONS (NON-ANOMALOUS) : 12514
REMARK   3
REMARK   3  FIT TO DATA USED IN REFINEMENT.
REMARK   3   R VALUE     (WORKING + TEST SET) : 0.1822
REMARK   3   R VALUE            (WORKING SET) : 0.1799
REMARK   3   FREE R VALUE                     : 0.2247
REMARK   3   FREE R VALUE TEST SET SIZE   (%) : 5.11
REMARK   3   FREE R VALUE TEST SET COUNT      : 1163
REMARK   3
REMARK   3  FIT TO DATA USED IN REFINEMENT (IN BINS).
REMARK   3   BIN  RESOLUTION RANGE  COMPL.    NWORK NFREE   RWORK  RFREE  CCWORK CCFREE
REMARK   3     1   57.37 -    3.52    1.00     2812   144  0.1580 0.1744   0.929  0.890
REMARK   3     2    3.52 -    2.79    1.00     2774   149  0.1791 0.2361   0.920  0.846
REMARK   3     3    2.79 -    2.44    1.00     2778   175  0.1829 0.2271   0.912  0.891
REMARK   3     4    2.44 -    2.22    1.00     2820   121  0.1652 0.1948   0.929  0.899
REMARK   3     5    2.22 -    2.06    1.00     2803   123  0.1937 0.2396   0.921  0.890
REMARK   3     6    2.06 -    1.94    1.00     2760   176  0.1962 0.2759   0.913  0.799
REMARK   3     7    1.94 -    1.84    0.97     2724   140  0.2133 0.3391   0.909  0.753
REMARK   3     8    1.84 -    1.76    0.76     2107   135  0.2752 0.3268   0.887  0.824
REMARK   3
REMARK   3  BULK SOLVENT MODELLING.
REMARK   3   METHOD USED        : FLAT BULK SOLVENT MODEL
REMARK   3   SOLVENT RADIUS     : 1.10
REMARK   3   SHRINKAGE RADIUS   : 0.90
REMARK   3   GRID STEP FACTOR   : 4.00
REMARK   3
REMARK   3  ERROR ESTIMATES.
REMARK   3   COORDINATE ERROR (MAXIMUM-LIKELIHOOD BASED)     : 0.25
REMARK   3   PHASE ERROR (DEGREES, MAXIMUM-LIKELIHOOD BASED) : 23.96
REMARK   3
REMARK   3  STRUCTURE FACTORS CALCULATION ALGORITHM : FFT
REMARK   3  B VALUES.
REMARK   3   FROM WILSON PLOT           (A**2) : 19.21
REMARK   3
REMARK   3  GEOMETRY RESTRAINTS LIBRARY: GEOSTD + MONOMER LIBRARY + CDL V1.2
REMARK   3  DEVIATIONS FROM IDEAL VALUES - RMSD. RMSZ FOR BONDS AND ANGLES.
REMARK   3    BOND      :  0.012   0.068   1094  Z= 0.757
REMARK   3    ANGLE     :  1.237   7.944   1489  Z= 0.715
REMARK   3    CHIRALITY :  0.068   0.342    146
REMARK   3    PLANARITY :  0.012   0.061    192
REMARK   3    DIHEDRAL  : 14.978  81.666    388
REMARK   3    MIN NONBONDED DISTANCE : 2.170
REMARK   3
REMARK   3  MOLPROBITY STATISTICS.
REMARK   3    ALL-ATOM CLASHSCORE : 2.90
REMARK   3    RAMACHANDRAN PLOT:
REMARK   3      OUTLIERS :  0.00 %
REMARK   3      ALLOWED  :  2.36 %
REMARK   3      FAVORED  : 97.64 %
REMARK   3    ROTAMER OUTLIERS :  0.93 %
REMARK   3    CBETA DEVIATIONS :  0.00 %
REMARK   3    PEPTIDE PLANE:
REMARK   3      CIS-PROLINE     : 0.00 %
REMARK   3      CIS-GENERAL     : 0.00 %
REMARK   3      TWISTED PROLINE : 0.00 %
REMARK   3      TWISTED GENERAL : 0.00 %
REMARK   3
REMARK   3  RAMA-Z (RAMACHANDRAN PLOT Z-SCORE):
REMARK   3  INTERPRETATION: BAD |RAMA-Z| > 3; SUSPICIOUS 2 < |RAMA-Z| < 3; GOOD |RAMA-Z| < 2.
```

```
REMARK   3  SCORES FOR WHOLE/HELIX/SHEET/LOOP ARE SCALED INDEPENDENTLY;
REMARK   3  THEREFORE, THE VALUES ARE NOT RELATED IN A SIMPLE MANNER.
REMARK   3    WHOLE: -0.76 (0.70), RESIDUES: 133
REMARK   3    HELIX: -1.09 (0.69), RESIDUES: 51
REMARK   3    SHEET: -1.20 (1.03), RESIDUES: 13
REMARK   3    LOOP :  0.34 (0.77), RESIDUES: 69
REMARK   3
REMARK   3                  min    max   mean <Bi,j>   iso aniso
REMARK   3     Overall:    8.20  86.59  21.75   5.95  1166     0
REMARK   3     Protein:    8.20  86.59  20.80   5.66  1023     0
REMARK   3     Water:     11.32  43.40  25.21    N/A    84     0
REMARK   3     Other:     16.45  68.83  33.20    N/A    59     0
REMARK   3     Chain  A:   8.20  86.59  21.35    N/A  1074     0
REMARK   3     Chain  S:  11.32  43.40  25.21    N/A    84     0
REMARK   3     Chain  B:  25.05  68.83  40.86    N/A     7     0
REMARK   3     Chain  C:  24.57  24.57  24.57    N/A     1     0
REMARK   3     Histogram:
REMARK   3         Values      Number of atoms
REMARK   3       8.20 - 16.04       387
REMARK   3      16.04 - 23.88       445
REMARK   3      23.88 - 31.72       169
REMARK   3      31.72 - 39.56        93
REMARK   3      39.56 - 47.40        36
REMARK   3      47.40 - 55.23        20
REMARK   3      55.23 - 63.07         5
REMARK   3      63.07 - 70.91         8
REMARK   3      70.91 - 78.75         2
REMARK   3      78.75 - 86.59         1
REMARK   3
REMARK   3
LINK         NE2 HIS A  15                 RE1  RI3 A1141
LINK         OD2 ASP A 101                 RE1  RII A1142
LINK         OD2 ASP A 119                 RE1  RII A1140
SSBOND   1 CYS A    6    CYS A  127
SSBOND   2 CYS A   30    CYS A  115
SSBOND   3 CYS A   64    CYS A   80
SSBOND   4 CYS A   76    CYS A   94
CRYST1   81.130   81.130   37.200  90.00  90.00  90.00 P 43 21 2
SCALE1      0.012326  0.000000  0.000000        0.00000
SCALE2      0.000000  0.012326  0.000000        0.00000
SCALE3      0.000000  0.000000  0.026882        0.00000
ATOM      1  N   LYS A   1       4.819  11.675   9.793  1.00 21.76           N  0.078
ATOM      2  CA  LYS A   1       3.827  12.050   8.748  1.00 27.51           C  0.088
ATOM      3  C   LYS A   1       3.854  13.548   8.587  1.00 18.11           C  0.071
ATOM      4  O   LYS A   1       3.862  14.252   9.581  1.00 18.45           O  0.072
ATOM      5  CB  LYS A   1       2.445  11.590   9.146  1.00 22.11           C  0.079
ATOM      6  CG  LYS A   1       1.341  12.097   8.246  1.00 23.56           C  0.081
ATOM      7  CD  LYS A   1       0.028  11.412   8.544  1.00 24.32           C  0.083
ATOM      8  CE  LYS A   1      -1.076  12.069   7.728  1.00 27.83           C  0.088
ATOM      9  NZ  LYS A   1      -0.687  12.391   6.327  1.00 46.10           N  0.114
ATOM     10  H1  LYS A   1       4.862  10.789   9.856  1.00 26.00           H  0.085
ATOM     11  H2  LYS A   1       5.619  11.997   9.572  1.00 26.00           H  0.085
ATOM     12  H3  LYS A   1       4.568  12.017  10.576  1.00 26.00           H  0.085
ATOM     13  HA  LYS A   1       4.037  11.625   7.902  1.00 32.89           H  0.096
ATOM     14  HB2 LYS A   1       2.422  10.620   9.123  1.00 26.41           H  0.086
ATOM     15  HB3 LYS A   1       2.261  11.905  10.045  1.00 26.41           H  0.086
ATOM     16  HG2 LYS A   1       1.227  13.050   8.384  1.00 28.15           H  0.089
ATOM     17  HG3 LYS A   1       1.574  11.921   7.321  1.00 28.15           H  0.089
ATOM     18  HD2 LYS A   1       0.082  10.475   8.302  1.00 29.06           H  0.090
ATOM     19  HD3 LYS A   1      -0.183  11.500   9.487  1.00 29.06           H  0.090
ATOM     20  HE2 LYS A   1      -1.836  11.468   7.691  1.00 33.27           H  0.096
ATOM     21  HE3 LYS A   1      -1.331  12.900   8.160  1.00 33.27           H  0.096
ATOM     22  HZ1 LYS A   1      -1.395  12.681   5.873  1.00 55.20           H  0.124
ATOM     23  HZ2 LYS A   1      -0.061  13.024   6.323  1.00 55.20           H  0.124
ATOM     24  HZ3 LYS A   1      -0.365  11.664   5.927  1.00 55.20           H  0.124
ATOM     25  N   VAL A   2       3.918  14.026   7.352  1.00 17.68           N  0.070
ATOM     26  CA  VAL A   2       3.790  15.434   7.049  1.00 19.83           C  0.075
ATOM     27  C   VAL A   2       2.365  15.624   6.544  1.00 26.54           C  0.086
ATOM     28  O   VAL A   2       2.014  15.168   5.446  1.00 20.28           O  0.075
ATOM     29  CB  VAL A   2       4.805  15.908   6.012  1.00 20.04           C  0.075
ATOM     30  CG1 VAL A   2       4.603  17.349   5.766  1.00 27.05           C  0.087
ATOM     31  CG2 VAL A   2       6.269  15.718   6.490  1.00 18.83           C  0.073
ATOM     32  H   VAL A   2       4.038  13.535   6.656  1.00 21.09           H  0.077
ATOM     33  HA  VAL A   2       3.935  15.955   7.854  1.00 23.68           H  0.081
ATOM     34  HB  VAL A   2       4.671  15.381   5.208  1.00 23.92           H  0.082
ATOM     35 HG11 VAL A   2       5.338  17.684   5.228  1.00 32.34           H  0.095
ATOM     36 HG12 VAL A   2       3.765  17.475   5.294  1.00 32.34           H  0.095
ATOM     37 HG13 VAL A   2       4.577  17.814   6.617  1.00 32.34           H  0.095
```

```
ATOM     38 HG21 VAL A   2       6.870  16.074   5.818  1.00 22.48           H  0.079
ATOM     39 HG22 VAL A   2       6.391  16.192   7.328  1.00 22.48           H  0.079
ATOM     40 HG23 VAL A   2       6.439  14.772   6.618  1.00 22.48           H  0.079
ATOM     41  N   PHE A   3       1.552  16.296   7.333  1.00 18.19           N  0.071
ATOM     42  CA  PHE A   3       0.175  16.545   6.945  1.00 17.16           C  0.069
ATOM     43  C   PHE A   3       0.061  17.634   5.906  1.00 19.30           C  0.073
ATOM     44  O   PHE A   3       0.842  18.580   5.873  1.00 16.11           O  0.067
ATOM     45  CB  PHE A   3      -0.658  17.005   8.131  1.00 16.68           C  0.068
ATOM     46  CG  PHE A   3      -1.163  15.913   8.969  1.00 15.87           C  0.067
ATOM     47  CD1 PHE A   3      -0.347  15.326   9.919  1.00 18.08           C  0.071
ATOM     48  CD2 PHE A   3      -2.459  15.457   8.845  1.00 17.62           C  0.070
ATOM     49  CE1 PHE A   3      -0.815  14.339  10.711  1.00 22.94           C  0.080
ATOM     50  CE2 PHE A   3      -2.907  14.479   9.637  1.00 18.29           C  0.072
ATOM     51  CZ  PHE A   3      -2.075  13.898  10.561  1.00 26.23           C  0.086
ATOM     52  H   PHE A   3       1.770  16.620   8.099  1.00 21.70           H  0.078
ATOM     53  HA  PHE A   3      -0.166  15.711   6.588  1.00 20.47           H  0.076
ATOM     54  HB2 PHE A   3      -0.111  17.578   8.691  1.00 19.89           H  0.075
ATOM     55  HB3 PHE A   3      -1.424  17.500   7.799  1.00 19.89           H  0.075
ATOM     56  HD1 PHE A   3       0.532  15.615  10.014  1.00 21.58           H  0.078
ATOM     57  HD2 PHE A   3      -3.023  15.832   8.207  1.00 21.02           H  0.077
ATOM     58  HE1 PHE A   3      -0.265  13.963  11.360  1.00 27.41           H  0.088
ATOM     59  HE2 PHE A   3      -3.787  14.191   9.561  1.00 21.83           H  0.078
ATOM     60  HZ  PHE A   3      -2.386  13.196  11.086  1.00 31.35           H  0.094
ATOM     61  N   GLY A   4      -0.978  17.525   5.083  1.00 24.70           N  0.083
ATOM     62  CA  GLY A   4      -1.386  18.663   4.315  1.00 23.91           C  0.082
ATOM     63  C   GLY A   4      -2.258  19.555   5.162  1.00 14.60           C  0.064
ATOM     64  O   GLY A   4      -2.817  19.133   6.155  1.00 20.88           O  0.076
ATOM     65  H   GLY A   4      -1.446  16.813   4.964  1.00 29.52           H  0.091
ATOM     66  HA2 GLY A   4      -0.607  19.163   4.024  1.00 28.58           H  0.089
ATOM     67  HA3 GLY A   4      -1.886  18.376   3.536  1.00 28.58           H  0.089
ATOM     68  N   ARG A   5      -2.357  20.813   4.747  1.00 20.17           N  0.075
ATOM     69  CA  ARG A   5      -3.133  21.783   5.500  1.00 23.23           C  0.081
ATOM     70  C   ARG A   5      -4.568  21.314   5.751  1.00 19.24           C  0.073
ATOM     71  O   ARG A   5      -5.027  21.229   6.902  1.00 17.95           O  0.071
ATOM     72  CB  ARG A   5      -3.097  23.087   4.721  1.00 28.99           C  0.090
ATOM     73  CG  ARG A   5      -3.940  24.170   5.292  1.00 19.92           C  0.075
ATOM     74  CD  ARG A   5      -3.665  25.473   4.538  1.00 26.36           C  0.086
ATOM     75  NE  ARG A   5      -4.123  25.428   3.153  1.00 33.00           N  0.096
ATOM     76  CZ  ARG A   5      -5.388  25.588   2.785  1.00 33.99           C  0.098
ATOM     77  NH1 ARG A   5      -6.344  25.796   3.673  1.00 27.52           N  0.088
ATOM     78  NH2 ARG A   5      -5.694  25.573   1.492  1.00 37.05           N  0.102
ATOM     79  H   ARG A   5      -1.986  21.127   4.037  1.00 24.08           H  0.082
ATOM     80  HA  ARG A   5      -2.742  21.913   6.378  1.00 27.76           H  0.088
ATOM     81  HB2 ARG A   5      -2.183  23.408   4.698  1.00 34.66           H  0.098
ATOM     82  HB3 ARG A   5      -3.412  22.916   3.819  1.00 34.66           H  0.098
ATOM     83  HG2 ARG A   5      -4.878  23.944   5.197  1.00 23.78           H  0.082
ATOM     84  HG3 ARG A   5      -3.723  24.298   6.229  1.00 23.78           H  0.082
ATOM     85  HD2 ARG A   5      -4.126  26.200   4.983  1.00 31.51           H  0.094
ATOM     86  HD3 ARG A   5      -2.709  25.640   4.533  1.00 31.51           H  0.094
ATOM     87  HE  ARG A   5      -3.538  25.289   2.538  1.00 39.47           H  0.105
ATOM     88 HH11 ARG A   5      -6.153  25.831   4.511  1.00 32.90           H  0.096
ATOM     89 HH12 ARG A   5      -7.157  25.898   3.414  1.00 32.90           H  0.096
ATOM     90 HH21 ARG A   5      -5.076  25.461   0.905  1.00 44.34           H  0.111
ATOM     91 HH22 ARG A   5      -6.511  25.676   1.242  1.00 44.34           H  0.111
ATOM     92  N   CYS A   6      -5.313  21.030   4.686  1.00 23.40           N  0.081
ATOM     93  CA  CYS A   6      -6.722  20.679   4.899  1.00 15.47           C  0.066
ATOM     94  C   CYS A   6      -6.866  19.349   5.606  1.00 17.72           C  0.070
ATOM     95  O   CYS A   6      -7.812  19.149   6.362  1.00 19.33           O  0.074
ATOM     96  CB  CYS A   6      -7.459  20.645   3.561  1.00 25.50           C  0.084
ATOM     97  SG  CYS A   6      -7.742  22.338   2.940  1.00 22.98           S  0.080
ATOM     98  H   CYS A   6      -5.045  21.031   3.869  1.00 27.95           H  0.088
ATOM     99  HA  CYS A   6      -7.132  21.361   5.454  1.00 18.44           H  0.072
ATOM    100  HB2 CYS A   6      -6.926  20.161   2.910  1.00 30.47           H  0.092
ATOM    101  HB3 CYS A   6      -8.318  20.209   3.676  1.00 30.47           H  0.092
ATOM    102  N   GLU A   7      -5.948  18.422   5.335  1.00 21.63           N  0.078
ATOM    103  CA  GLU A   7      -5.948  17.140   6.018  1.00 17.27           C  0.070
ATOM    104  C   GLU A   7      -5.799  17.316   7.515  1.00 20.69           C  0.076
ATOM    105  O   GLU A   7      -6.495  16.667   8.311  1.00 18.05           O  0.071
ATOM    106  CB  GLU A   7      -4.817  16.275   5.456  1.00 26.85           C  0.087
ATOM    107  CG  GLU A   7      -4.643  14.929   6.141  1.00 35.92           C  0.100
ATOM    108  CD  GLU A   7      -3.433  14.128   5.598  1.00 42.25           C  0.109
ATOM    109  OE1 GLU A   7      -2.423  14.728   5.118  1.00 29.97           O  0.092
ATOM    110  OE2 GLU A   7      -3.527  12.878   5.611  1.00 37.06           O  0.102
ATOM    111  H   GLU A   7      -5.316  18.513   4.759  1.00 25.84           H  0.085
ATOM    112  HA  GLU A   7      -6.791  16.687   5.860  1.00 20.61           H  0.076
ATOM    113  HB2 GLU A   7      -4.997  16.104   4.519  1.00 32.10           H  0.095
ATOM    114  HB3 GLU A   7      -3.982  16.760   5.551  1.00 32.10           H  0.095
ATOM    115  HG2 GLU A   7      -4.505  15.073   7.090  1.00 42.99           H  0.110
```

```
ATOM    116  HG3 GLU A   7      -5.442  14.397   5.999  1.00 42.99           H  0.110
ATOM    117  N   LEU A   8      -4.882  18.184   7.928  1.00 20.99           N  0.077
ATOM    118  CA  LEU A   8      -4.712  18.387   9.357  1.00 15.25           C  0.065
ATOM    119  C   LEU A   8      -5.913  19.107   9.948  1.00 14.92           C  0.065
ATOM    120  O   LEU A   8      -6.342  18.795  11.063  1.00 15.48           O  0.066
ATOM    121  CB  LEU A   8      -3.407  19.165   9.618  1.00 13.50           C  0.061
ATOM    122  CG  LEU A   8      -3.069  19.420  11.079  1.00 15.39           C  0.066
ATOM    123  CD1 LEU A   8      -2.834  18.062  11.796  1.00 18.26           C  0.071
ATOM    124  CD2 LEU A   8      -1.860  20.298  11.265  1.00 19.42           C  0.074
ATOM    125  H   LEU A   8      -4.367  18.650   7.421  1.00 25.06           H  0.084
ATOM    126  HA  LEU A   8      -4.636  17.528   9.801  1.00 18.18           H  0.071
ATOM    127  HB2 LEU A   8      -2.671  18.661   9.236  1.00 16.08           H  0.067
ATOM    128  HB3 LEU A   8      -3.477  20.030   9.183  1.00 16.08           H  0.067
ATOM    129  HG  LEU A   8      -3.819  19.892  11.474  1.00 18.34           H  0.072
ATOM    130 HD11 LEU A   8      -2.426  18.228  12.660  1.00 21.79           H  0.078
ATOM    131 HD12 LEU A   8      -3.687  17.615  11.914  1.00 21.79           H  0.078
ATOM    132 HD13 LEU A   8      -2.247  17.514  11.252  1.00 21.79           H  0.078
ATOM    133 HD21 LEU A   8      -1.728  20.458  12.213  1.00 23.18           H  0.081
ATOM    134 HD22 LEU A   8      -1.084  19.850  10.894  1.00 23.18           H  0.081
ATOM    135 HD23 LEU A   8      -2.007  21.140  10.805  1.00 23.18           H  0.081
ATOM    136  N   ALA A   9      -6.463  20.077   9.228  1.00 19.31           N  0.074
ATOM    137  CA  ALA A   9      -7.664  20.741   9.723  1.00 16.82           C  0.069
ATOM    138  C   ALA A   9      -8.756  19.723  10.018  1.00 17.91           C  0.071
ATOM    139  O   ALA A   9      -9.406  19.757  11.065  1.00 16.60           O  0.068
ATOM    140  CB  ALA A   9      -8.166  21.754   8.708  1.00 20.15           C  0.075
ATOM    141  H   ALA A   9      -6.171  20.362   8.471  1.00 23.05           H  0.080
ATOM    142  HA  ALA A   9      -7.440  21.214  10.540  1.00 20.07           H  0.075
ATOM    143  HB1 ALA A   9      -9.006  22.125   9.021  1.00 24.06           H  0.082
ATOM    144  HB2 ALA A   9      -7.507  22.460   8.614  1.00 24.06           H  0.082
ATOM    145  HB3 ALA A   9      -8.298  21.310   7.856  1.00 24.06           H  0.082
ATOM    146  N   ALA A  10      -8.970  18.805   9.089  1.00 20.95           N  0.077
ATOM    147  CA  ALA A  10     -10.006  17.795   9.264  1.00 18.99           C  0.073
ATOM    148  C   ALA A  10      -9.751  16.940  10.494  1.00 19.90           C  0.075
ATOM    149  O   ALA A  10     -10.660  16.681  11.295  1.00 20.70           O  0.076
ATOM    150  CB  ALA A  10     -10.082  16.936   8.004  1.00 24.31           C  0.082
ATOM    151  H   ALA A  10      -8.534  18.742   8.350  1.00 25.02           H  0.084
ATOM    152  HA  ALA A  10     -10.861  18.234   9.394  1.00 22.67           H  0.080
ATOM    153  HB1 ALA A  10     -10.733  16.230   8.143  1.00 29.05           H  0.090
ATOM    154  HB2 ALA A  10     -10.353  17.493   7.258  1.00 29.05           H  0.090
ATOM    155  HB3 ALA A  10      -9.209  16.552   7.830  1.00 29.05           H  0.090
ATOM    156  N   ALA A  11      -8.514  16.473  10.664  1.00 19.89           N  0.075
ATOM    157  CA  ALA A  11      -8.237  15.631  11.816  1.00 16.21           C  0.067
ATOM    158  C   ALA A  11      -8.354  16.419  13.110  1.00 16.45           C  0.068
ATOM    159  O   ALA A  11      -8.910  15.935  14.106  1.00 21.22           O  0.077
ATOM    160  CB  ALA A  11      -6.859  14.987  11.654  1.00 21.55           C  0.078
ATOM    161  H   ALA A  11      -7.846  16.625  10.144  1.00 23.75           H  0.082
ATOM    162  HA  ALA A  11      -8.887  14.913  11.865  1.00 19.33           H  0.074
ATOM    163  HB1 ALA A  11      -6.684  14.419  12.421  1.00 25.74           H  0.085
ATOM    164  HB2 ALA A  11      -6.851  14.458  10.842  1.00 25.74           H  0.085
ATOM    165  HB3 ALA A  11      -6.188  15.686  11.601  1.00 25.74           H  0.085
ATOM    166  N   MET A  12      -7.804  17.628  13.140  1.00 17.09           N  0.069
ATOM    167  CA  MET A  12      -7.938  18.409  14.360  1.00 10.88           C  0.055
ATOM    168  C   MET A  12      -9.407  18.629  14.703  1.00 17.34           C  0.070
ATOM    169  O   MET A  12      -9.774  18.660  15.884  1.00 15.52           O  0.066
ATOM    170  CB  MET A  12      -7.224  19.745  14.190  1.00 12.44           C  0.059
ATOM    171  CG  MET A  12      -5.724  19.664  14.467  1.00 15.52           C  0.066
ATOM    172  SD  MET A  12      -4.894  21.225  14.038  1.00 14.98           S  0.065
ATOM    173  CE  MET A  12      -3.371  21.090  15.095  1.00 11.36           C  0.056
ATOM    174  H   MET A  12      -7.369  18.001  12.499  1.00 20.38           H  0.076
ATOM    175  HA  MET A  12      -7.513  17.933  15.090  1.00 12.94           H  0.060
ATOM    176  HB2 MET A  12      -7.343  20.051  13.278  1.00 14.81           H  0.064
ATOM    177  HB3 MET A  12      -7.607  20.386  14.808  1.00 14.81           H  0.064
ATOM    178  HG2 MET A  12      -5.579  19.489  15.410  1.00 18.50           H  0.072
ATOM    179  HG3 MET A  12      -5.335  18.953  13.934  1.00 18.50           H  0.072
ATOM    180  HE1 MET A  12      -2.814  21.870  14.946  1.00 13.51           H  0.061
ATOM    181  HE2 MET A  12      -3.637  21.046  16.027  1.00 13.51           H  0.061
ATOM    182  HE3 MET A  12      -2.887  20.286  14.851  1.00 13.51           H  0.061
ATOM    183  N   LYS A  13     -10.254  18.812  13.681  1.00 19.51           N  0.074
ATOM    184  CA  LYS A  13     -11.693  18.962  13.935  1.00 16.76           C  0.068
ATOM    185  C   LYS A  13     -12.291  17.677  14.506  1.00 14.65           C  0.064
ATOM    186  O   LYS A  13     -13.088  17.727  15.446  1.00 21.01           O  0.077
ATOM    187  CB  LYS A  13     -12.427  19.355  12.650  1.00 21.85           C  0.078
ATOM    188  CG  LYS A  13     -13.910  19.818  12.869  1.00 32.02           C  0.095
ATOM    189  CD  LYS A  13     -14.544  20.273  11.543  1.00 31.24           C  0.094
ATOM    190  CE  LYS A  13     -16.078  20.336  11.569  1.00 34.60           C  0.098
ATOM    191  NZ  LYS A  13     -16.611  21.524  10.791  1.00 47.51           N  0.115
ATOM    192  H   LYS A  13     -10.027  18.852  12.853  1.00 23.29           H  0.081
ATOM    193  HA  LYS A  13     -11.815  19.674  14.582  1.00 19.99           H  0.075
```

ATOM	194	HB2	LYS	A	13	-11.949	20.089	12.233	1.00	26.10	H	0.085
ATOM	195	HB3	LYS	A	13	-12.443	18.589	12.056	1.00	26.10	H	0.085
ATOM	196	HG2	LYS	A	13	-14.431	19.079	13.222	1.00	38.30	H	0.104
ATOM	197	HG3	LYS	A	13	-13.928	20.563	13.490	1.00	38.30	H	0.104
ATOM	198	HD2	LYS	A	13	-14.217	21.161	11.330	1.00	37.36	H	0.102
ATOM	199	HD3	LYS	A	13	-14.288	19.649	10.845	1.00	37.36	H	0.102
ATOM	200	HE2	LYS	A	13	-16.438	19.528	11.172	1.00	41.39	H	0.108
ATOM	201	HE3	LYS	A	13	-16.379	20.417	12.488	1.00	41.39	H	0.108
ATOM	202	HZ1	LYS	A	13	-17.500	21.540	10.830	1.00	56.88	H	0.126
ATOM	203	HZ2	LYS	A	13	-16.293	22.281	11.136	1.00	56.88	H	0.126
ATOM	204	HZ3	LYS	A	13	-16.359	21.467	9.939	1.00	56.88	H	0.126
ATOM	205	N	ARG	A	14	-11.932	16.534	13.925	1.00	23.24	N	0.081
ATOM	206	CA	ARG	A	14	-12.377	15.259	14.465	1.00	29.02	C	0.090
ATOM	207	C	ARG	A	14	-11.946	15.081	15.915	1.00	32.22	C	0.095
ATOM	208	O	ARG	A	14	-12.633	14.411	16.687	1.00	24.37	O	0.083
ATOM	209	CB	ARG	A	14	-11.811	14.118	13.634	1.00	22.13	C	0.079
ATOM	210	CG	ARG	A	14	-12.248	14.079	12.168	1.00	41.76	C	0.108
ATOM	211	CD	ARG	A	14	-13.107	12.839	11.881	1.00	67.64	C	0.138
ATOM	212	NE	ARG	A	14	-13.994	12.514	12.996	1.00	73.49	N	0.143
ATOM	213	CZ	ARG	A	14	-14.451	11.299	13.273	1.00	61.97	C	0.132
ATOM	214	NH1	ARG	A	14	-14.050	10.240	12.594	1.00	45.47	N	0.113
ATOM	215	NH2	ARG	A	14	-15.330	11.148	14.259	1.00	66.70	N	0.137
ATOM	216	H	ARG	A	14	-11.437	16.472	13.225	1.00	27.77	H	0.088
ATOM	217	HA	ARG	A	14	-13.346	15.231	14.421	1.00	34.71	H	0.099
ATOM	218	HB2	ARG	A	14	-10.843	14.187	13.644	1.00	26.43	H	0.086
ATOM	219	HB3	ARG	A	14	-12.087	13.281	14.039	1.00	26.43	H	0.086
ATOM	220	HG2	ARG	A	14	-12.774	14.869	11.967	1.00	50.00	H	0.118
ATOM	221	HG3	ARG	A	14	-11.464	14.048	11.598	1.00	50.00	H	0.118
ATOM	222	HD2	ARG	A	14	-13.654	13.004	11.097	1.00	81.05	H	0.151
ATOM	223	HD3	ARG	A	14	-12.526	12.078	11.725	1.00	81.05	H	0.151
ATOM	224	HE	ARG	A	14	-14.238	13.157	13.511	1.00	88.06	H	0.157
ATOM	225	HH11	ARG	A	14	-13.480	10.328	11.957	1.00	54.45	H	0.123
ATOM	226	HH12	ARG	A	14	-14.359	9.461	12.790	1.00	54.45	H	0.123
ATOM	227	HH21	ARG	A	14	-15.595	11.832	14.708	1.00	79.92	H	0.150
ATOM	228	HH22	ARG	A	14	-15.634	10.366	14.448	1.00	79.92	H	0.150
ATOM	229	N	HIS	A	15	-10.796	15.633	16.273	1.00	21.57	N	0.078
ATOM	230	CA	HIS	A	15	-10.257	15.492	17.643	1.00	18.75	C	0.072
ATOM	231	C	HIS	A	15	-10.775	16.594	18.564	1.00	23.88	C	0.082
ATOM	232	O	HIS	A	15	-10.234	16.686	19.647	1.00	23.59	O	0.081
ATOM	233	CB	HIS	A	15	-8.731	15.410	17.606	1.00	23.21	C	0.081
ATOM	234	CG	HIS	A	15	-8.217	14.111	17.100	1.00	21.43	C	0.077
ATOM	235	ND1	HIS	A	15	-8.267	12.970	17.851	1.00	34.03	N	0.098
ATOM	236	CD2	HIS	A	15	-7.636	13.766	15.936	1.00	37.04	C	0.102
ATOM	237	CE1	HIS	A	15	-7.744	11.977	17.177	1.00	25.34	C	0.084
ATOM	238	NE2	HIS	A	15	-7.347	12.439	16.005	1.00	24.17	N	0.082
ATOM	239	H	HIS	A	15	-10.185	16.186	15.645	1.00	25.76	H	0.085
ATOM	240	HA	HIS	A	15	-10.618	14.544	18.038	1.00	22.38	H	0.079
ATOM	241	HB2	HIS	A	15	-8.351	16.210	16.973	1.00	27.73	H	0.088
ATOM	242	HB3	HIS	A	15	-8.342	15.567	18.610	1.00	27.73	H	0.088
ATOM	243	HD1	HIS	A	15	-8.697	12.888	18.763	1.00	40.72	H	0.107
ATOM	244	HD2	HIS	A	15	-7.407	14.420	15.108	1.00	44.33	H	0.111
ATOM	245	HE1	HIS	A	15	-7.675	10.955	17.518	1.00	30.29	H	0.092
ATOM	246	N	GLY	A	16	-11.745	17.423	18.154	1.00	18.96	N	0.073
ATOM	247	CA	GLY	A	16	-12.334	18.339	19.090	1.00	18.71	C	0.072
ATOM	248	C	GLY	A	16	-11.584	19.618	19.336	1.00	20.23	C	0.075
ATOM	249	O	GLY	A	16	-11.871	20.284	20.334	1.00	17.84	O	0.071
ATOM	250	H	GLY	A	16	-12.048	17.460	17.350	1.00	22.63	H	0.080
ATOM	251	HA2	GLY	A	16	-13.216	18.579	18.767	1.00	22.33	H	0.079
ATOM	252	HA3	GLY	A	16	-12.423	17.887	19.944	1.00	22.33	H	0.079
ATOM	253	N	LEU	A	17	-10.664	20.021	18.446	1.00	18.60	N	0.072
ATOM	254	CA	LEU	A	17	-9.952	21.285	18.666	1.00	15.55	C	0.066
ATOM	255	C	LEU	A	17	-10.696	22.526	18.172	1.00	20.58	C	0.076
ATOM	256	O	LEU	A	17	-10.382	23.641	18.626	1.00	16.75	O	0.068
ATOM	257	CB	LEU	A	17	-8.581	21.246	18.006	1.00	15.12	C	0.065
ATOM	258	CG	LEU	A	17	-7.497	20.531	18.819	1.00	15.99	C	0.067
ATOM	259	CD1	LEU	A	17	-6.227	20.650	18.024	1.00	15.53	C	0.066
ATOM	260	CD2	LEU	A	17	-7.304	21.038	20.264	1.00	17.62	C	0.070
ATOM	261	H	LEU	A	17	-10.443	19.594	17.733	1.00	22.20	H	0.079
ATOM	262	HA	LEU	A	17	-9.839	21.378	19.625	1.00	18.53	H	0.072
ATOM	263	HB2	LEU	A	17	-8.662	20.785	17.157	1.00	18.03	H	0.071
ATOM	264	HB3	LEU	A	17	-8.284	22.158	17.861	1.00	18.03	H	0.071
ATOM	265	HG	LEU	A	17	-7.764	19.608	18.955	1.00	19.07	H	0.073
ATOM	266	HD11	LEU	A	17	-5.602	19.971	18.323	1.00	18.51	H	0.072
ATOM	267	HD12	LEU	A	17	-6.430	20.522	17.084	1.00	18.51	H	0.072
ATOM	268	HD13	LEU	A	17	-5.849	21.533	18.164	1.00	18.51	H	0.072
ATOM	269	HD21	LEU	A	17	-6.428	20.765	20.581	1.00	21.02	H	0.077
ATOM	270	HD22	LEU	A	17	-7.370	22.006	20.271	1.00	21.02	H	0.077
ATOM	271	HD23	LEU	A	17	-7.994	20.655	20.828	1.00	21.02	H	0.077

```
ATOM    272  N   ASP A  18     -11.680  22.374  17.264  1.00 21.76           N  0.078
ATOM    273  CA  ASP A  18     -12.393  23.548  16.753  1.00 22.75           C  0.080
ATOM    274  C   ASP A  18     -13.173  24.202  17.890  1.00 24.82           C  0.083
ATOM    275  O   ASP A  18     -14.102  23.612  18.464  1.00 20.84           O  0.076
ATOM    276  CB  ASP A  18     -13.330  23.208  15.585  1.00 19.59           C  0.074
ATOM    277  CG  ASP A  18     -13.864  24.466  14.843  1.00 32.73           C  0.096
ATOM    278  OD1 ASP A  18     -13.354  25.608  15.022  1.00 25.60           O  0.085
ATOM    279  OD2 ASP A  18     -14.804  24.300  14.035  1.00 29.63           O  0.091
ATOM    280  H   ASP A  18     -11.941  21.622  16.940  1.00 25.99           H  0.085
ATOM    281  HA  ASP A  18     -11.736  24.171  16.406  1.00 27.17           H  0.087
ATOM    282  HB2 ASP A  18     -12.846  22.665  14.942  1.00 23.38           H  0.081
ATOM    283  HB3 ASP A  18     -14.093  22.716  15.927  1.00 23.38           H  0.081
ATOM    284  N   ASN A  19     -12.762  25.423  18.202  1.00 17.44           N  0.070
ATOM    285  CA  ASN A  19     -13.294  26.244  19.264  1.00 21.28           C  0.077
ATOM    286  C   ASN A  19     -13.009  25.700  20.645  1.00 14.52           C  0.064
ATOM    287  O   ASN A  19     -13.618  26.130  21.627  1.00 20.90           O  0.076
ATOM    288  CB  ASN A  19     -14.791  26.478  19.124  1.00 23.96           C  0.082
ATOM    289  CG  ASN A  19     -15.093  27.866  18.666  1.00 37.46           C  0.102
ATOM    290  OD1 ASN A  19     -14.438  28.397  17.754  1.00 36.88           O  0.102
ATOM    291  ND2 ASN A  19     -15.988  28.531  19.395  1.00 53.60           N  0.122
ATOM    292  H   ASN A  19     -12.129  25.821  17.778  1.00 20.81           H  0.076
ATOM    293  HA  ASN A  19     -12.847  27.102  19.189  1.00 25.42           H  0.084
ATOM    294  HB2 ASN A  19     -15.152  25.857  18.473  1.00 28.62           H  0.090
ATOM    295  HB3 ASN A  19     -15.218  26.343  19.985  1.00 28.62           H  0.090
ATOM    296 HD21 ASN A  19     -16.350  28.155  20.079  1.00 64.20           H  0.134
ATOM    297 HD22 ASN A  19     -16.205  29.335  19.181  1.00 64.20           H  0.134
ATOM    298  N   TYR A  20     -12.073  24.773  20.762  1.00 14.52           N  0.064
ATOM    299  CA  TYR A  20     -11.615  24.386  22.088  1.00 14.33           C  0.063
ATOM    300  C   TYR A  20     -11.001  25.571  22.835  1.00 21.51           C  0.078
ATOM    301  O   TYR A  20     -10.122  26.283  22.326  1.00 17.07           O  0.069
ATOM    302  CB  TYR A  20     -10.598  23.245  21.977  1.00 15.12           C  0.065
ATOM    303  CG  TYR A  20     -10.384  22.606  23.326  1.00 17.06           C  0.069
ATOM    304  CD1 TYR A  20     -11.240  21.597  23.766  1.00 20.47           C  0.076
ATOM    305  CD2 TYR A  20      -9.362  23.013  24.170  1.00 14.79           C  0.064
ATOM    306  CE1 TYR A  20     -11.076  21.013  25.014  1.00 20.82           C  0.076
ATOM    307  CE2 TYR A  20      -9.206  22.459  25.433  1.00 21.38           C  0.077
ATOM    308  CZ  TYR A  20     -10.078  21.449  25.840  1.00 26.30           C  0.086
ATOM    309  OH  TYR A  20      -9.934  20.868  27.052  1.00 22.38           O  0.079
ATOM    310  H   TYR A  20     -11.696  24.362  20.108  1.00 17.30           H  0.070
ATOM    311  HA  TYR A  20     -12.376  24.076  22.604  1.00 17.08           H  0.069
ATOM    312  HB2 TYR A  20     -10.930  22.572  21.363  1.00 18.03           H  0.071
ATOM    313  HB3 TYR A  20      -9.750  23.594  21.661  1.00 18.03           H  0.071
ATOM    314  HD1 TYR A  20     -11.932  21.311  23.215  1.00 24.44           H  0.083
ATOM    315  HD2 TYR A  20      -8.769  23.670  23.884  1.00 17.62           H  0.070
ATOM    316  HE1 TYR A  20     -11.642  20.328  25.288  1.00 24.86           H  0.083
ATOM    317  HE2 TYR A  20      -8.532  22.757  26.000  1.00 25.54           H  0.085
ATOM    318  HH  TYR A  20      -9.341  21.267  27.494  1.00 26.73           H  0.086
ATOM    319  N   ARG A  21     -11.440  25.767  24.075  1.00 17.15           N  0.069
ATOM    320  CA  ARG A  21     -10.999  26.914  24.871  1.00 13.98           C  0.063
ATOM    321  C   ARG A  21     -11.211  28.211  24.106  1.00 18.82           C  0.073
ATOM    322  O   ARG A  21     -10.599  29.236  24.418  1.00 15.65           O  0.066
ATOM    323  CB  ARG A  21      -9.547  26.733  25.338  1.00 18.32           C  0.072
ATOM    324  CG  ARG A  21      -9.150  27.469  26.620  1.00 33.46           C  0.097
ATOM    325  CD  ARG A  21     -10.032  27.127  27.830  1.00 33.86           C  0.097
ATOM    326  NE  ARG A  21     -10.249  25.696  27.952  1.00 47.76           N  0.116
ATOM    327  CZ  ARG A  21     -10.413  25.049  29.102  1.00 49.76           C  0.118
ATOM    328  NH1 ARG A  21     -10.289  25.667  30.272  1.00 45.45           N  0.113
ATOM    329  NH2 ARG A  21     -10.695  23.746  29.072  1.00 34.62           N  0.098
ATOM    330  H   ARG A  21     -11.994  25.249  24.479  1.00 20.46           H  0.076
ATOM    331  HA  ARG A  21     -11.533  26.982  25.678  1.00 16.66           H  0.068
ATOM    332  HB2 ARG A  21      -9.396  25.787  25.494  1.00 21.87           H  0.078
ATOM    333  HB3 ARG A  21      -8.961  27.051  24.634  1.00 21.87           H  0.078
ATOM    334  HG2 ARG A  21      -8.236  27.233  26.845  1.00 40.03           H  0.106
ATOM    335  HG3 ARG A  21      -9.217  28.424  26.466  1.00 40.03           H  0.106
ATOM    336  HD2 ARG A  21      -9.599  27.440  28.640  1.00 40.52           H  0.106
ATOM    337  HD3 ARG A  21     -10.895  27.558  27.728  1.00 40.52           H  0.106
ATOM    338  HE  ARG A  21     -10.273  25.234  27.228  1.00 57.19           H  0.127
ATOM    339 HH11 ARG A  21     -10.098  26.505  30.297  1.00 54.41           H  0.123
ATOM    340 HH12 ARG A  21     -10.399  25.229  31.004  1.00 54.41           H  0.123
ATOM    341 HH21 ARG A  21     -10.768  23.339  28.318  1.00 41.42           H  0.108
ATOM    342 HH22 ARG A  21     -10.804  23.313  29.807  1.00 41.42           H  0.108
ATOM    343  N   GLY A  22     -12.143  28.186  23.148  1.00 21.85           N  0.078
ATOM    344  CA  GLY A  22     -12.495  29.366  22.369  1.00 15.16           C  0.065
ATOM    345  C   GLY A  22     -11.617  29.620  21.164  1.00 17.67           C  0.070
ATOM    346  O   GLY A  22     -11.739  30.663  20.505  1.00 16.27           O  0.067
ATOM    347  H   GLY A  22     -12.591  27.485  22.930  1.00 26.10           H  0.085
ATOM    348  HA2 GLY A  22     -13.408  29.270  22.055  1.00 18.07           H  0.071
ATOM    349  HA3 GLY A  22     -12.440  30.145  22.945  1.00 18.07           H  0.071
```

ATOM	350	N	TYR	A	23	-10.718	28.698	20.848	1.00	13.15	N	0.061
ATOM	351	CA	TYR	A	23	-9.775	28.894	19.752	1.00	12.55	C	0.059
ATOM	352	C	TYR	A	23	-10.279	28.164	18.521	1.00	12.51	C	0.059
ATOM	353	O	TYR	A	23	-10.309	26.931	18.492	1.00	14.70	O	0.064
ATOM	354	CB	TYR	A	23	-8.388	28.396	20.179	1.00	12.86	C	0.060
ATOM	355	CG	TYR	A	23	-7.730	29.303	21.169	1.00	11.51	C	0.057
ATOM	356	CD1	TYR	A	23	-7.004	30.433	20.751	1.00	12.27	C	0.059
ATOM	357	CD2	TYR	A	23	-7.823	29.074	22.549	1.00	9.83	C	0.052
ATOM	358	CE1	TYR	A	23	-6.450	31.289	21.685	1.00	11.59	C	0.057
ATOM	359	CE2	TYR	A	23	-7.244	29.916	23.444	1.00	10.28	C	0.054
ATOM	360	CZ	TYR	A	23	-6.529	31.004	23.021	1.00	12.26	C	0.059
ATOM	361	OH	TYR	A	23	-5.927	31.876	23.923	1.00	14.47	O	0.064
ATOM	362	H	TYR	A	23	-10.634	27.945	21.255	1.00	15.66	H	0.066
ATOM	363	HA	TYR	A	23	-9.719	29.835	19.523	1.00	14.94	H	0.065
ATOM	364	HB2	TYR	A	23	-8.478	27.521	20.588	1.00	15.31	H	0.065
ATOM	365	HB3	TYR	A	23	-7.817	28.340	19.397	1.00	15.31	H	0.065
ATOM	366	HD1	TYR	A	23	-6.896	30.604	19.843	1.00	14.60	H	0.064
ATOM	367	HD2	TYR	A	23	-8.290	28.331	22.857	1.00	11.68	H	0.057
ATOM	368	HE1	TYR	A	23	-6.021	32.064	21.403	1.00	13.78	H	0.062
ATOM	369	HE2	TYR	A	23	-7.334	29.751	24.355	1.00	12.22	H	0.058
ATOM	370	HH	TYR	A	23	-5.990	31.571	24.704	1.00	17.24	H	0.069
ATOM	371	N	SER	A	24	-10.692	28.927	17.517	1.00	15.68	N	0.066
ATOM	372	CA	SER	A	24	-11.132	28.365	16.253	1.00	15.51	C	0.066
ATOM	373	C	SER	A	24	-10.047	27.530	15.572	1.00	12.75	C	0.060
ATOM	374	O	SER	A	24	-8.841	27.737	15.757	1.00	16.29	O	0.068
ATOM	375	CB	SER	A	24	-11.553	29.502	15.323	1.00	20.28	C	0.075
ATOM	376	OG	SER	A	24	-10.412	30.232	14.892	1.00	20.47	O	0.076
ATOM	377	H	SER	A	24	-10.726	29.786	17.545	1.00	18.69	H	0.072
ATOM	378	HA	SER	A	24	-11.880	27.771	16.420	1.00	18.49	H	0.072
ATOM	379	HB2	SER	A	24	-12.004	29.130	14.549	1.00	24.22	H	0.082
ATOM	380	HB3	SER	A	24	-12.151	30.099	15.800	1.00	24.22	H	0.082
ATOM	381	HG	SER	A	24	-9.921	29.743	14.417	1.00	24.44	H	0.083
ATOM	382	N	LEU	A	25	-10.508	26.608	14.724	1.00	14.10	N	0.063
ATOM	383	CA	LEU	A	25	-9.637	25.614	14.087	1.00	10.99	C	0.055
ATOM	384	C	LEU	A	25	-8.445	26.238	13.394	1.00	12.92	C	0.060
ATOM	385	O	LEU	A	25	-7.354	25.664	13.404	1.00	15.74	O	0.066
ATOM	386	CB	LEU	A	25	-10.474	24.842	13.064	1.00	20.92	C	0.077
ATOM	387	CG	LEU	A	25	-9.903	23.570	12.522	1.00	15.81	C	0.067
ATOM	388	CD1	LEU	A	25	-9.556	22.536	13.595	1.00	20.50	C	0.076
ATOM	389	CD2	LEU	A	25	-10.890	22.908	11.508	1.00	17.78	C	0.071
ATOM	390	H	LEU	A	25	-11.334	26.533	14.496	1.00	16.80	H	0.069
ATOM	391	HA	LEU	A	25	-9.289	25.017	14.769	1.00	13.07	H	0.060
ATOM	392	HB2	LEU	A	25	-11.318	24.615	13.483	1.00	24.98	H	0.084
ATOM	393	HB3	LEU	A	25	-10.626	25.427	12.305	1.00	24.98	H	0.084
ATOM	394	HG	LEU	A	25	-9.075	23.823	12.084	1.00	18.84	H	0.073
ATOM	395	HD11	LEU	A	25	-9.382	21.683	13.166	1.00	24.47	H	0.083
ATOM	396	HD12	LEU	A	25	-8.767	22.832	14.075	1.00	24.47	H	0.083
ATOM	397	HD13	LEU	A	25	-10.304	22.452	14.206	1.00	24.47	H	0.083
ATOM	398	HD21	LEU	A	25	-10.478	22.109	11.143	1.00	21.21	H	0.077
ATOM	399	HD22	LEU	A	25	-11.710	22.676	11.971	1.00	21.21	H	0.077
ATOM	400	HD23	LEU	A	25	-11.079	23.538	10.795	1.00	21.21	H	0.077
ATOM	401	N	GLY	A	26	-8.635	27.397	12.744	1.00	12.24	N	0.059
ATOM	402	CA	GLY	A	26	-7.518	28.032	12.046	1.00	14.11	C	0.063
ATOM	403	C	GLY	A	26	-6.332	28.373	12.942	1.00	12.80	C	0.060
ATOM	404	O	GLY	A	26	-5.183	28.328	12.497	1.00	15.81	O	0.067
ATOM	405	H	GLY	A	26	-9.381	27.822	12.695	1.00	14.57	H	0.064
ATOM	406	HA2	GLY	A	26	-7.203	27.435	11.350	1.00	16.80	H	0.069
ATOM	407	HA3	GLY	A	26	-7.829	28.856	11.639	1.00	16.80	H	0.069
ATOM	408	N	ASN	A	27	-6.596	28.706	14.213	1.00	11.54	N	0.057
ATOM	409	CA	ASN	A	27	-5.523	28.975	15.160	1.00	10.47	C	0.054
ATOM	410	C	ASN	A	27	-4.629	27.761	15.366	1.00	11.84	C	0.058
ATOM	411	O	ASN	A	27	-3.398	27.886	15.443	1.00	13.35	O	0.061
ATOM	412	CB	ASN	A	27	-6.125	29.370	16.507	1.00	10.76	C	0.055
ATOM	413	CG	ASN	A	27	-6.590	30.793	16.517	1.00	15.57	C	0.066
ATOM	414	OD1	ASN	A	27	-5.795	31.696	16.613	1.00	11.84	O	0.058
ATOM	415	ND2	ASN	A	27	-7.914	30.992	16.403	1.00	17.34	N	0.070
ATOM	416	H	ASN	A	27	-7.385	28.782	14.545	1.00	13.73	H	0.062
ATOM	417	HA	ASN	A	27	-4.985	29.700	14.807	1.00	12.44	H	0.059
ATOM	418	HB2	ASN	A	27	-6.887	28.800	16.696	1.00	12.79	H	0.060
ATOM	419	HB3	ASN	A	27	-5.453	29.266	17.199	1.00	12.79	H	0.060
ATOM	420	HD21	ASN	A	27	-8.231	31.791	16.403	1.00	20.68	H	0.076
ATOM	421	HD22	ASN	A	27	-8.445	30.319	16.329	1.00	20.68	H	0.076
ATOM	422	N	TRP	A	28	-5.255	26.596	15.490	1.00	11.37	N	0.056
ATOM	423	CA	TRP	A	28	-4.541	25.339	15.700	1.00	8.20	C	0.048
ATOM	424	C	TRP	A	28	-3.744	24.924	14.464	1.00	9.10	C	0.050
ATOM	425	O	TRP	A	28	-2.622	24.401	14.584	1.00	10.71	O	0.055
ATOM	426	CB	TRP	A	28	-5.553	24.268	16.100	1.00	10.65	C	0.055
ATOM	427	CG	TRP	A	28	-6.252	24.568	17.408	1.00	11.95	C	0.058

ATOM	428	CD1	TRP	A	28	-7.513	25.076	17.557	1.00	10.23	C	0.054
ATOM	429	CD2	TRP	A	28	-5.715	24.441	18.719	1.00	10.33	C	0.054
ATOM	430	NE1	TRP	A	28	-7.807	25.211	18.878	1.00	15.37	N	0.066
ATOM	431	CE2	TRP	A	28	-6.703	24.851	19.618	1.00	13.17	C	0.061
ATOM	432	CE3	TRP	A	28	-4.490	23.995	19.217	1.00	11.20	C	0.056
ATOM	433	CZ2	TRP	A	28	-6.512	24.831	21.005	1.00	14.02	C	0.063
ATOM	434	CZ3	TRP	A	28	-4.295	23.977	20.585	1.00	13.68	C	0.062
ATOM	435	CH2	TRP	A	28	-5.286	24.402	21.461	1.00	17.28	C	0.070
ATOM	436	H	TRP	A	28	-6.110	26.501	15.457	1.00	13.53	H	0.062
ATOM	437	HA	TRP	A	28	-3.898	25.443	16.419	1.00	9.72	H	0.052
ATOM	438	HB2	TRP	A	28	-6.229	24.201	15.408	1.00	12.66	H	0.060
ATOM	439	HB3	TRP	A	28	-5.091	23.420	16.196	1.00	12.66	H	0.060
ATOM	440	HD1	TRP	A	28	-8.085	25.296	16.857	1.00	12.15	H	0.058
ATOM	441	HE1	TRP	A	28	-8.560	25.477	19.199	1.00	18.32	H	0.072
ATOM	442	HE3	TRP	A	28	-3.817	23.715	18.639	1.00	13.32	H	0.061
ATOM	443	HZ2	TRP	A	28	-7.183	25.095	21.592	1.00	16.70	H	0.068
ATOM	444	HZ3	TRP	A	28	-3.484	23.673	20.926	1.00	16.29	H	0.068
ATOM	445	HH2	TRP	A	28	-5.117	24.397	22.375	1.00	20.61	H	0.076
ATOM	446	N	VAL	A	29	-4.347	25.066	13.272	1.00	11.60	N	0.057
ATOM	447	CA	VAL	A	29	-3.643	24.777	12.026	1.00	9.43	C	0.051
ATOM	448	C	VAL	A	29	-2.474	25.713	11.843	1.00	12.80	C	0.060
ATOM	449	O	VAL	A	29	-1.362	25.291	11.514	1.00	12.53	O	0.059
ATOM	450	CB	VAL	A	29	-4.586	24.851	10.814	1.00	11.01	C	0.056
ATOM	451	CG1	VAL	A	29	-3.809	24.608	9.536	1.00	11.35	C	0.056
ATOM	452	CG2	VAL	A	29	-5.656	23.791	10.957	1.00	14.28	C	0.063
ATOM	453	H	VAL	A	29	-5.160	25.327	13.164	1.00	13.79	H	0.062
ATOM	454	HA	VAL	A	29	-3.311	23.868	12.093	1.00	11.19	H	0.056
ATOM	455	HB	VAL	A	29	-4.995	25.730	10.771	1.00	13.09	H	0.061
ATOM	456	HG11	VAL	A	29	-4.435	24.468	8.808	1.00	13.50	H	0.061
ATOM	457	HG12	VAL	A	29	-3.255	25.382	9.352	1.00	13.50	H	0.061
ATOM	458	HG13	VAL	A	29	-3.253	23.822	9.650	1.00	13.50	H	0.061
ATOM	459	HG21	VAL	A	29	-6.336	23.935	10.280	1.00	17.01	H	0.069
ATOM	460	HG22	VAL	A	29	-5.252	22.917	10.839	1.00	17.01	H	0.069
ATOM	461	HG23	VAL	A	29	-6.050	23.857	11.841	1.00	17.01	H	0.069
ATOM	462	N	CYS	A	30	-2.691	27.006	12.111	1.00	12.88	N	0.060
ATOM	463	CA	CYS	A	30	-1.609	27.973	12.003	1.00	14.74	C	0.064
ATOM	464	C	CYS	A	30	-0.514	27.668	13.008	1.00	13.41	C	0.061
ATOM	465	O	CYS	A	30	0.674	27.737	12.677	1.00	11.62	O	0.057
ATOM	466	CB	CYS	A	30	-2.169	29.384	12.204	1.00	12.85	C	0.060
ATOM	467	SG	CYS	A	30	-0.984	30.725	12.066	1.00	15.54	S	0.066
ATOM	468	H	CYS	A	30	-3.446	27.339	12.353	1.00	15.33	H	0.066
ATOM	469	HA	CYS	A	30	-1.217	27.930	11.117	1.00	17.56	H	0.070
ATOM	470	HB2	CYS	A	30	-2.854	29.537	11.534	1.00	15.30	H	0.065
ATOM	471	HB3	CYS	A	30	-2.556	29.433	13.092	1.00	15.30	H	0.065
ATOM	472	N	ALA	A	31	-0.889	27.302	14.234	1.00	11.86	N	0.058
ATOM	473	CA	ALA	A	31	0.150	26.962	15.201	1.00	11.19	C	0.056
ATOM	474	C	ALA	A	31	0.948	25.761	14.735	1.00	12.33	C	0.059
ATOM	475	O	ALA	A	31	2.162	25.738	14.871	1.00	10.69	O	0.055
ATOM	476	CB	ALA	A	31	-0.423	26.670	16.577	1.00	13.00	C	0.060
ATOM	477	H	ALA	A	31	-1.699	27.245	14.516	1.00	14.11	H	0.063
ATOM	478	HA	ALA	A	31	0.728	27.736	15.282	1.00	13.30	H	0.061
ATOM	479	HB1	ALA	A	31	0.296	26.393	17.167	1.00	15.48	H	0.066
ATOM	480	HB2	ALA	A	31	-0.843	27.474	16.922	1.00	15.48	H	0.066
ATOM	481	HB3	ALA	A	31	-1.081	25.961	16.502	1.00	15.48	H	0.066
ATOM	482	N	ALA	A	32	0.274	24.724	14.210	1.00	12.40	N	0.059
ATOM	483	CA	ALA	A	32	1.047	23.556	13.758	1.00	17.06	C	0.069
ATOM	484	C	ALA	A	32	1.968	23.913	12.605	1.00	13.95	C	0.062
ATOM	485	O	ALA	A	32	3.080	23.366	12.497	1.00	13.14	O	0.061
ATOM	486	CB	ALA	A	32	0.113	22.416	13.344	1.00	20.62	C	0.076
ATOM	487	H	ALA	A	32	-0.578	24.674	14.110	1.00	14.76	H	0.064
ATOM	488	HA	ALA	A	32	1.588	23.249	14.502	1.00	20.35	H	0.075
ATOM	489	HB1	ALA	A	32	0.644	21.687	12.987	1.00	24.63	H	0.083
ATOM	490	HB2	ALA	A	32	-0.382	22.115	14.122	1.00	24.63	H	0.083
ATOM	491	HB3	ALA	A	32	-0.501	22.741	12.667	1.00	24.63	H	0.083
ATOM	492	N	LYS	A	33	1.482	24.745	11.674	1.00	11.43	N	0.057
ATOM	493	CA	LYS	A	33	2.310	25.152	10.538	1.00	10.79	C	0.055
ATOM	494	C	LYS	A	33	3.641	25.709	10.997	1.00	14.10	C	0.063
ATOM	495	O	LYS	A	33	4.702	25.297	10.498	1.00	14.82	O	0.064
ATOM	496	CB	LYS	A	33	1.578	26.195	9.671	1.00	12.12	C	0.058
ATOM	497	CG	LYS	A	33	2.443	26.783	8.618	1.00	16.28	C	0.068
ATOM	498	CD	LYS	A	33	2.845	25.834	7.559	1.00	18.06	C	0.071
ATOM	499	CE	LYS	A	33	3.772	26.547	6.563	1.00	21.48	C	0.078
ATOM	500	NZ	LYS	A	33	4.054	25.660	5.441	1.00	23.39	N	0.081
ATOM	501	H	LYS	A	33	0.690	25.081	11.680	1.00	13.59	H	0.062
ATOM	502	HA	LYS	A	33	2.471	24.366	9.993	1.00	12.83	H	0.060
ATOM	503	HB2	LYS	A	33	0.823	25.769	9.235	1.00	14.43	H	0.064
ATOM	504	HB3	LYS	A	33	1.268	26.916	10.241	1.00	14.43	H	0.064
ATOM	505	HG2	LYS	A	33	1.963	27.510	8.192	1.00	19.42	H	0.074

ATOM	506	HG3	LYS	A	33	3.253	27.119	9.033	1.00	19.42	H	0.074
ATOM	507	HD2	LYS	A	33	3.321	25.085	7.951	1.00	21.55	H	0.078
ATOM	508	HD3	LYS	A	33	2.060	25.516	7.085	1.00	21.55	H	0.078
ATOM	509	HE2	LYS	A	33	3.340	27.348	6.228	1.00	25.66	H	0.085
ATOM	510	HE3	LYS	A	33	4.607	26.777	6.998	1.00	25.66	H	0.085
ATOM	511	HZ1	LYS	A	33	4.419	24.903	5.735	1.00	27.94	H	0.088
ATOM	512	HZ2	LYS	A	33	3.300	25.468	5.008	1.00	27.94	H	0.088
ATOM	513	HZ3	LYS	A	33	4.619	26.053	4.877	1.00	27.94	H	0.088
ATOM	514	N	PHE	A	34	3.616	26.659	11.944	1.00	15.80	N	0.067
ATOM	515	CA	PHE	A	34	4.847	27.345	12.327	1.00	13.69	C	0.062
ATOM	516	C	PHE	A	34	5.603	26.647	13.454	1.00	16.30	C	0.068
ATOM	517	O	PHE	A	34	6.828	26.835	13.580	1.00	17.01	O	0.069
ATOM	518	CB	PHE	A	34	4.529	28.802	12.669	1.00	11.30	C	0.056
ATOM	519	CG	PHE	A	34	4.017	29.580	11.477	1.00	12.84	C	0.060
ATOM	520	CD1	PHE	A	34	4.712	29.537	10.265	1.00	17.39	C	0.070
ATOM	521	CD2	PHE	A	34	2.849	30.309	11.538	1.00	20.79	C	0.076
ATOM	522	CE1	PHE	A	34	4.249	30.206	9.164	1.00	15.26	C	0.065
ATOM	523	CE2	PHE	A	34	2.395	30.984	10.417	1.00	18.16	C	0.071
ATOM	524	CZ	PHE	A	34	3.121	30.940	9.235	1.00	14.43	C	0.064
ATOM	525	H	PHE	A	34	2.912	26.914	12.367	1.00	18.84	H	0.073
ATOM	526	HA	PHE	A	34	5.464	27.364	11.578	1.00	16.31	H	0.068
ATOM	527	HB2	PHE	A	34	3.846	28.824	13.358	1.00	13.44	H	0.061
ATOM	528	HB3	PHE	A	34	5.335	29.236	12.988	1.00	13.44	H	0.061
ATOM	529	HD1	PHE	A	34	5.501	29.047	10.206	1.00	20.75	H	0.076
ATOM	530	HD2	PHE	A	34	2.367	30.348	12.332	1.00	24.83	H	0.083
ATOM	531	HE1	PHE	A	34	4.716	30.153	8.362	1.00	18.19	H	0.071
ATOM	532	HE2	PHE	A	34	1.601	31.468	10.457	1.00	21.66	H	0.078
ATOM	533	HZ	PHE	A	34	2.830	31.417	8.492	1.00	17.19	H	0.069
ATOM	534	N	GLU	A	35	4.959	25.759	14.200	1.00	12.87	N	0.060
ATOM	535	CA	GLU	A	35	5.737	24.971	15.143	1.00	12.11	C	0.058
ATOM	536	C	GLU	A	35	6.504	23.863	14.434	1.00	20.32	C	0.075
ATOM	537	O	GLU	A	35	7.679	23.629	14.733	1.00	16.64	O	0.068
ATOM	538	CB	GLU	A	35	4.818	24.386	16.213	1.00	13.83	C	0.062
ATOM	539	CG	GLU	A	35	4.233	25.409	17.152	1.00	10.89	C	0.055
ATOM	540	CD	GLU	A	35	5.232	26.030	18.122	1.00	19.92	C	0.075
ATOM	541	OE1	GLU	A	35	6.379	25.590	18.145	1.00	16.97	O	0.069
ATOM	542	OE2	GLU	A	35	4.870	27.026	18.794	1.00	17.35	O	0.070
ATOM	543	H	GLU	A	35	4.114	25.602	14.180	1.00	15.33	H	0.066
ATOM	544	HA	GLU	A	35	6.380	25.547	15.586	1.00	14.40	H	0.063
ATOM	545	HB2	GLU	A	35	4.080	23.934	15.774	1.00	16.48	H	0.068
ATOM	546	HB3	GLU	A	35	5.325	23.754	16.745	1.00	16.48	H	0.068
ATOM	547	HG2	GLU	A	35	3.852	26.129	16.625	1.00	12.95	H	0.060
ATOM	548	HG3	GLU	A	35	3.541	24.982	17.680	1.00	12.95	H	0.060
ATOM	549	N	SER	A	36	5.872	23.176	13.484	1.00	15.32	N	0.065
ATOM	550	CA	SER	A	36	6.432	21.923	12.984	1.00	13.03	C	0.060
ATOM	551	C	SER	A	36	6.460	21.784	11.474	1.00	18.16	C	0.071
ATOM	552	O	SER	A	36	6.888	20.723	10.980	1.00	16.32	O	0.068
ATOM	553	CB	SER	A	36	5.643	20.725	13.522	1.00	12.54	C	0.059
ATOM	554	OG	SER	A	36	4.336	20.692	12.947	1.00	13.96	O	0.063
ATOM	555	H	SER	A	36	5.129	23.412	13.119	1.00	18.27	H	0.072
ATOM	556	HA	SER	A	36	7.346	21.888	13.308	1.00	15.52	H	0.066
ATOM	557	HB2	SER	A	36	6.111	19.907	13.292	1.00	14.93	H	0.065
ATOM	558	HB3	SER	A	36	5.564	20.803	14.485	1.00	14.93	H	0.065
ATOM	559	HG	SER	A	36	3.828	21.218	13.359	1.00	16.62	H	0.068
ATOM	560	N	ASN	A	37	6.011	22.789	10.742	1.00	15.94	N	0.067
ATOM	561	CA	ASN	A	37	5.675	22.686	9.324	1.00	18.13	C	0.071
ATOM	562	C	ASN	A	37	4.879	21.428	9.016	1.00	16.16	C	0.067
ATOM	563	O	ASN	A	37	5.093	20.783	7.983	1.00	17.84	O	0.071
ATOM	564	CB	ASN	A	37	6.937	22.753	8.475	1.00	17.03	C	0.069
ATOM	565	CG	ASN	A	37	6.660	23.235	7.058	1.00	29.80	C	0.091
ATOM	566	OD1	ASN	A	37	5.589	23.774	6.784	1.00	28.65	O	0.090
ATOM	567	ND2	ASN	A	37	7.598	22.982	6.135	1.00	33.78	N	0.097
ATOM	568	H	ASN	A	37	5.884	23.581	11.054	1.00	19.01	H	0.073
ATOM	569	HA	ASN	A	37	5.110	23.438	9.088	1.00	21.64	H	0.078
ATOM	570	HB2	ASN	A	37	7.563	23.369	8.886	1.00	20.31	H	0.075
ATOM	571	HB3	ASN	A	37	7.330	21.868	8.421	1.00	20.31	H	0.075
ATOM	572	HD21	ASN	A	37	7.481	23.237	5.322	1.00	40.42	H	0.106
ATOM	573	HD22	ASN	A	37	8.316	22.564	6.355	1.00	40.42	H	0.106
ATOM	574	N	PHE	A	38	3.931	21.099	9.897	1.00	12.97	N	0.060
ATOM	575	CA	PHE	A	38	2.980	20.010	9.751	1.00	12.23	C	0.059
ATOM	576	C	PHE	A	38	3.623	18.629	9.832	1.00	14.47	C	0.064
ATOM	577	O	PHE	A	38	2.993	17.659	9.455	1.00	15.44	O	0.066
ATOM	578	CB	PHE	A	38	2.201	20.089	8.429	1.00	13.43	C	0.061
ATOM	579	CG	PHE	A	38	1.311	21.309	8.298	1.00	14.14	C	0.063
ATOM	580	CD1	PHE	A	38	0.734	21.887	9.407	1.00	15.00	C	0.065
ATOM	581	CD2	PHE	A	38	1.016	21.834	7.054	1.00	16.49	C	0.068
ATOM	582	CE1	PHE	A	38	-0.146	22.953	9.279	1.00	11.98	C	0.058
ATOM	583	CE2	PHE	A	38	0.152	22.965	6.930	1.00	14.44	C	0.064

```
ATOM    584  CZ  PHE A  38      -0.429  23.482   8.031  1.00 15.54           C  0.066
ATOM    585  H   PHE A  38       3.818  21.525  10.636  1.00 15.44           H  0.066
ATOM    586  HA  PHE A  38       2.357  20.102  10.488  1.00 14.55           H  0.064
ATOM    587  HB2 PHE A  38       2.837  20.110   7.697  1.00 15.99           H  0.067
ATOM    588  HB3 PHE A  38       1.636  19.305   8.356  1.00 15.99           H  0.067
ATOM    589  HD1 PHE A  38       0.937  21.558  10.253  1.00 17.88           H  0.071
ATOM    590  HD2 PHE A  38       1.383  21.447   6.291  1.00 19.66           H  0.074
ATOM    591  HE1 PHE A  38      -0.548  23.314  10.037  1.00 14.25           H  0.063
ATOM    592  HE2 PHE A  38      -0.009  23.342   6.095  1.00 17.20           H  0.069
ATOM    593  HZ  PHE A  38      -1.021  24.194   7.953  1.00 18.53           H  0.072
ATOM    594  N   ASN A  39       4.859  18.521  10.340  1.00 14.21           N  0.063
ATOM    595  CA  ASN A  39       5.570  17.247  10.437  1.00 13.45           C  0.061
ATOM    596  C   ASN A  39       5.409  16.685  11.836  1.00 15.23           C  0.065
ATOM    597  O   ASN A  39       5.899  17.270  12.795  1.00 13.66           O  0.062
ATOM    598  CB  ASN A  39       7.043  17.457  10.130  1.00 14.31           C  0.063
ATOM    599  CG  ASN A  39       7.827  16.164  10.102  1.00 14.02           C  0.063
ATOM    600  OD1 ASN A  39       7.334  15.122  10.459  1.00 12.28           O  0.059
ATOM    601  ND2 ASN A  39       9.055  16.246   9.656  1.00 16.92           N  0.069
ATOM    602  H   ASN A  39       5.311  19.188  10.641  1.00 16.92           H  0.069
ATOM    603  HA  ASN A  39       5.184  16.617   9.808  1.00 16.01           H  0.067
ATOM    604  HB2 ASN A  39       7.128  17.878   9.260  1.00 17.05           H  0.069
ATOM    605  HB3 ASN A  39       7.429  18.027  10.814  1.00 17.05           H  0.069
ATOM    606 HD21 ASN A  39       9.546  15.541   9.617  1.00 20.18           H  0.075
ATOM    607 HD22 ASN A  39       9.370  17.004   9.401  1.00 20.18           H  0.075
ATOM    608  N   THR A  40       4.722  15.551  11.961  1.00 13.81           N  0.062
ATOM    609  CA  THR A  40       4.511  15.008  13.290  1.00 13.01           C  0.060
ATOM    610  C   THR A  40       5.809  14.570  13.941  1.00 12.91           C  0.060
ATOM    611  O   THR A  40       5.886  14.450  15.166  1.00 14.94           O  0.065
ATOM    612  CB  THR A  40       3.562  13.815  13.278  1.00 20.50           C  0.076
ATOM    613  OG1 THR A  40       4.197  12.674  12.677  1.00 19.86           O  0.075
ATOM    614  CG2 THR A  40       2.307  14.128  12.511  1.00 15.03           C  0.065
ATOM    615  H   THR A  40       4.382  15.097  11.314  1.00 16.45           H  0.068
ATOM    616  HA  THR A  40       4.097  15.711  13.816  1.00 15.49           H  0.066
ATOM    617  HB  THR A  40       3.323  13.606  14.195  1.00 24.48           H  0.083
ATOM    618  HG1 THR A  40       4.672  12.278  13.245  1.00 23.71           H  0.081
ATOM    619 HG21 THR A  40       1.722  13.355  12.501  1.00 17.91           H  0.071
ATOM    620 HG22 THR A  40       1.840  14.870  12.927  1.00 17.91           H  0.071
ATOM    621 HG23 THR A  40       2.527  14.368  11.598  1.00 17.91           H  0.071
ATOM    622  N   GLN A  41       6.814  14.281  13.150  1.00 11.54           N  0.057
ATOM    623  CA  GLN A  41       8.083  13.813  13.718  1.00 10.40           C  0.054
ATOM    624  C   GLN A  41       9.046  14.921  14.098  1.00 16.30           C  0.068
ATOM    625  O   GLN A  41      10.168  14.623  14.525  1.00 18.74           O  0.072
ATOM    626  CB  GLN A  41       8.764  12.820  12.760  1.00 11.59           C  0.057
ATOM    627  CG  GLN A  41       7.895  11.642  12.419  1.00 16.80           C  0.069
ATOM    628  CD  GLN A  41       8.680  10.522  11.781  1.00 20.84           C  0.076
ATOM    629  OE1 GLN A  41       9.405   9.821  12.476  1.00 17.81           O  0.071
ATOM    630  NE2 GLN A  41       8.613  10.402  10.465  1.00 12.34           N  0.059
ATOM    631  H   GLN A  41       6.798  14.342  12.293  1.00 13.72           H  0.062
ATOM    632  HA  GLN A  41       7.874  13.342  14.540  1.00 12.35           H  0.059
ATOM    633  HB2 GLN A  41       8.982  13.279  11.934  1.00 13.78           H  0.062
ATOM    634  HB3 GLN A  41       9.573  12.484  13.177  1.00 13.78           H  0.062
ATOM    635  HG2 GLN A  41       7.488  11.301  13.231  1.00 20.04           H  0.075
ATOM    636  HG3 GLN A  41       7.207  11.923  11.796  1.00 20.04           H  0.075
ATOM    637 HE21 GLN A  41       8.134  10.951  10.007  1.00 14.69           H  0.064
ATOM    638 HE22 GLN A  41       9.047   9.776  10.068  1.00 14.69           H  0.064
ATOM    639  N   ALA A  42       8.644  16.185  14.002  1.00 11.53           N  0.057
ATOM    640  CA  ALA A  42       9.574  17.276  14.305  1.00  9.27           C  0.051
ATOM    641  C   ALA A  42      10.030  17.239  15.768  1.00 11.77           C  0.057
ATOM    642  O   ALA A  42       9.238  17.004  16.668  1.00 12.75           O  0.060
ATOM    643  CB  ALA A  42       8.893  18.613  14.021  1.00 15.11           C  0.065
ATOM    644  H   ALA A  42       7.856  16.437  13.767  1.00 13.72           H  0.062
ATOM    645  HA  ALA A  42      10.363  17.184  13.747  1.00 11.00           H  0.055
ATOM    646  HB1 ALA A  42       9.502  19.332  14.248  1.00 18.01           H  0.071
ATOM    647  HB2 ALA A  42       8.665  18.657  13.079  1.00 18.01           H  0.071
ATOM    648  HB3 ALA A  42       8.088  18.678  14.559  1.00 18.01           H  0.071
ATOM    649  N   THR A  43      11.339  17.470  15.990  1.00 16.44           N  0.068
ATOM    650  CA  THR A  43      11.905  17.623  17.330  1.00 12.00           C  0.058
ATOM    651  C   THR A  43      12.833  18.822  17.335  1.00 15.99           C  0.067
ATOM    652  O   THR A  43      13.418  19.195  16.307  1.00 21.58           O  0.078
ATOM    653  CB  THR A  43      12.680  16.365  17.815  1.00 15.28           C  0.065
ATOM    654  OG1 THR A  43      13.811  16.100  16.979  1.00 16.89           O  0.069
ATOM    655  CG2 THR A  43      11.759  15.166  17.883  1.00 15.79           C  0.066
ATOM    656  H   THR A  43      11.924  17.543  15.364  1.00 19.60           H  0.074
ATOM    657  HA  THR A  43      11.182  17.769  17.960  1.00 14.28           H  0.063
ATOM    658  HB  THR A  43      13.018  16.527  18.710  1.00 18.21           H  0.071
ATOM    659  HG1 THR A  43      13.565  16.019  16.179  1.00 20.14           H  0.075
ATOM    660 HG21 THR A  43      12.192  14.441  18.360  1.00 18.83           H  0.073
ATOM    661 HG22 THR A  43      10.940  15.402  18.345  1.00 18.83           H  0.073
```

```
ATOM    662 HG23 THR A  43      11.539  14.866  16.987  1.00 18.83           H  0.073
ATOM    663  N   ASN A  44      12.947  19.455  18.486  1.00 11.76           N  0.057
ATOM    664  CA  ASN A  44      13.890  20.559  18.606  1.00  8.87           C  0.050
ATOM    665  C   ASN A  44      14.453  20.553  20.019  1.00 16.96           C  0.069
ATOM    666  O   ASN A  44      13.681  20.632  20.974  1.00 15.52           O  0.066
ATOM    667  CB  ASN A  44      13.262  21.884  18.293  1.00 22.26           C  0.079
ATOM    668  CG  ASN A  44      14.094  22.665  17.336  1.00 50.93           C  0.119
ATOM    669  OD1 ASN A  44      14.372  22.210  16.210  1.00 47.22           O  0.115
ATOM    670  ND2 ASN A  44      14.639  23.767  17.823  1.00 37.94           N  0.103
ATOM    671  H   ASN A  44      12.501  19.272  19.198  1.00 13.99           H  0.063
ATOM    672  HA  ASN A  44      14.612  20.422  17.973  1.00 10.53           H  0.054
ATOM    673  HB2 ASN A  44      12.390  21.741  17.894  1.00 26.59           H  0.086
ATOM    674  HB3 ASN A  44      13.173  22.398  19.111  1.00 26.59           H  0.086
ATOM    675 HD21 ASN A  44      14.502  23.989  18.643  1.00 45.40           H  0.113
ATOM    676 HD22 ASN A  44      15.130  24.262  17.320  1.00 45.40           H  0.113
ATOM    677  N  AARG A  45      15.780  20.428  20.141  0.51 16.18           N  0.067
ATOM    678  CA AARG A  45      16.446  20.519  21.438  0.51 16.16           C  0.067
ATOM    679  C  AARG A  45      16.677  21.970  21.820  0.51 21.87           C  0.078
ATOM    680  O  AARG A  45      17.251  22.757  21.046  0.51 19.47           O  0.074
ATOM    681  CB AARG A  45      17.783  19.758  21.441  0.51 21.37           C  0.077
ATOM    682  CG AARG A  45      18.420  19.543  22.846  0.51 27.06           C  0.087
ATOM    683  CD AARG A  45      18.246  18.111  23.329  0.51 24.64           C  0.083
ATOM    684  NE AARG A  45      18.429  17.935  24.770  0.51 22.22           N  0.079
ATOM    685  CZ AARG A  45      19.297  17.105  25.332  0.51 24.52           C  0.083
ATOM    686  NH1AARG A  45      20.163  16.412  24.609  0.51 21.58           N  0.078
ATOM    687  NH2AARG A  45      19.284  16.947  26.655  0.51 21.69           N  0.078
ATOM    688  H  AARG A  45      16.317  20.290  19.483  0.51 19.29           H  0.073
ATOM    689  HA AARG A  45      15.868  20.111  22.102  0.51 19.27           H  0.073
ATOM    690  HB2AARG A  45      17.638  18.882  21.051  0.51 25.53           H  0.085
ATOM    691  HB3AARG A  45      18.421  20.258  20.908  0.51 25.53           H  0.085
ATOM    692  HG2AARG A  45      19.370  19.735  22.800  0.51 32.35           H  0.095
ATOM    693  HG3AARG A  45      17.993  20.134  23.486  0.51 32.35           H  0.095
ATOM    694  HD2AARG A  45      17.349  17.817  23.109  0.51 29.44           H  0.091
ATOM    695  HD3AARG A  45      18.898  17.551  22.880  0.51 29.44           H  0.091
ATOM    696  HE AARG A  45      17.935  18.406  25.293  0.51 26.54           H  0.086
ATOM    697 HH11AARG A  45      20.172  16.494  23.753  0.51 25.77           H  0.085
ATOM    698 HH12AARG A  45      20.717  15.879  24.996  0.51 25.77           H  0.085
ATOM    699 HH21AARG A  45      18.716  17.381  27.134  0.51 25.91           H  0.085
ATOM    700 HH22AARG A  45      19.842  16.411  27.030  0.51 25.91           H  0.085
ATOM    701  N  BARG A  45      15.780  20.484  20.120  0.49 16.23           N  0.067
ATOM    702  CA BARG A  45      16.483  20.544  21.397  0.49 16.16           C  0.067
ATOM    703  C  BARG A  45      16.695  21.990  21.815  0.49 21.91           C  0.078
ATOM    704  O  BARG A  45      17.263  22.796  21.058  0.49 19.39           O  0.074
ATOM    705  CB BARG A  45      17.815  19.792  21.286  0.49 21.53           C  0.078
ATOM    706  CG BARG A  45      18.698  19.858  22.518  0.49 29.80           C  0.091
ATOM    707  CD BARG A  45      18.139  19.034  23.658  0.49 19.89           C  0.075
ATOM    708  NE BARG A  45      17.494  17.783  23.252  0.49 19.11           N  0.073
ATOM    709  CZ BARG A  45      18.133  16.659  22.953  0.49 19.32           C  0.074
ATOM    710  NH1BARG A  45      19.454  16.588  22.956  0.49 18.79           N  0.073
ATOM    711  NH2BARG A  45      17.428  15.568  22.682  0.49 19.39           N  0.074
ATOM    712  H  BARG A  45      16.308  20.400  19.446  0.49 19.36           H  0.074
ATOM    713  HA BARG A  45      15.946  20.122  22.085  0.49 19.27           H  0.073
ATOM    714  HB2BARG A  45      17.626  18.857  21.113  0.49 25.71           H  0.085
ATOM    715  HB3BARG A  45      18.319  20.170  20.548  0.49 25.71           H  0.085
ATOM    716  HG2BARG A  45      19.578  19.514  22.299  0.49 35.64           H  0.100
ATOM    717  HG3BARG A  45      18.765  20.779  22.815  0.49 35.64           H  0.100
ATOM    718  HD2BARG A  45      18.866  18.805  24.258  0.49 23.74           H  0.082
ATOM    719  HD3BARG A  45      17.477  19.565  24.127  0.49 23.74           H  0.082
ATOM    720  HE BARG A  45      16.635  17.777  23.204  0.49 22.81           H  0.080
ATOM    721 HH11BARG A  45      19.921  17.282  23.155  0.49 22.42           H  0.079
ATOM    722 HH12BARG A  45      19.845  15.848  22.758  0.49 22.42           H  0.079
ATOM    723 HH21BARG A  45      16.569  15.596  22.703  0.49 23.15           H  0.080
ATOM    724 HH22BARG A  45      17.831  14.834  22.487  0.49 23.15           H  0.080
ATOM    725  N   ASN A  46      16.227  22.316  23.020  1.00 16.34           N  0.068
ATOM    726  CA  ASN A  46      16.342  23.651  23.591  1.00 21.03           C  0.077
ATOM    727  C   ASN A  46      17.671  23.781  24.339  1.00 18.44           C  0.072
ATOM    728  O   ASN A  46      18.301  22.793  24.737  1.00 20.13           O  0.075
ATOM    729  CB  ASN A  46      15.158  23.905  24.515  1.00 23.24           C  0.081
ATOM    730  CG  ASN A  46      13.820  23.774  23.814  1.00 24.69           C  0.083
ATOM    731  OD1 ASN A  46      12.899  23.155  24.327  1.00 26.98           O  0.087
ATOM    732  ND2 ASN A  46      13.715  24.370  22.629  1.00 22.03           N  0.079
ATOM    733  HA  ASN A  46      16.331  24.330  22.898  1.00 25.11           H  0.084
ATOM    734  HB2 ASN A  46      15.180  23.260  25.239  1.00 27.77           H  0.088
ATOM    735  HB3 ASN A  46      15.223  24.805  24.870  1.00 27.77           H  0.088
ATOM    736 HD21 ASN A  46      12.977  24.326  22.189  1.00 26.32           H  0.086
ATOM    737 HD22 ASN A  46      14.385  24.799  22.302  1.00 26.32           H  0.086
ATOM    738  N   THR A  47      18.143  25.029  24.462  1.00 24.08           N  0.082
ATOM    739  CA  THR A  47      19.446  25.221  25.073  1.00 21.13           C  0.077
```

ATOM	740	C	THR	A	47	19.456	24.797	26.542	1.00	30.40	C	0.092
ATOM	741	O	THR	A	47	20.520	24.424	27.062	1.00	33.17	O	0.096
ATOM	742	CB	THR	A	47	19.900	26.681	24.924	1.00	29.96	C	0.092
ATOM	743	OG1	THR	A	47	21.082	26.894	25.706	1.00	50.30	O	0.119
ATOM	744	CG2	THR	A	47	18.829	27.619	25.358	1.00	29.46	C	0.091
ATOM	745	H	THR	A	47	17.740	25.745	24.208	1.00	28.77	H	0.090
ATOM	746	HA	THR	A	47	20.090	24.668	24.604	1.00	25.24	H	0.084
ATOM	747	HB	THR	A	47	20.092	26.866	23.992	1.00	35.83	H	0.100
ATOM	748	HG1	THR	A	47	21.237	27.717	25.774	1.00	60.24	H	0.130
ATOM	749	HG21	THR	A	47	19.183	28.521	25.419	1.00	35.23	H	0.099
ATOM	750	HG22	THR	A	47	18.100	27.610	24.718	1.00	35.23	H	0.099
ATOM	751	HG23	THR	A	47	18.488	27.355	26.227	1.00	35.23	H	0.099
ATOM	752	N	ASP	A	48	18.294	24.829	27.215	1.00	30.10	N	0.092
ATOM	753	CA	ASP	A	48	18.138	24.338	28.591	1.00	34.04	C	0.098
ATOM	754	C	ASP	A	48	18.101	22.815	28.714	1.00	40.25	C	0.106
ATOM	755	O	ASP	A	48	17.948	22.302	29.824	1.00	33.20	O	0.096
ATOM	756	CB	ASP	A	48	16.861	24.917	29.246	1.00	35.54	C	0.100
ATOM	757	CG	ASP	A	48	15.555	24.278	28.723	1.00	46.57	C	0.114
ATOM	758	OD1	ASP	A	48	15.600	23.402	27.836	1.00	31.01	O	0.093
ATOM	759	OD2	ASP	A	48	14.471	24.655	29.221	1.00	43.48	O	0.110
ATOM	760	H	ASP	A	48	17.562	25.139	26.886	1.00	36.00	H	0.100
ATOM	761	HA	ASP	A	48	18.908	24.657	29.088	1.00	40.73	H	0.107
ATOM	762	HB2	ASP	A	48	16.903	24.765	30.203	1.00	42.53	H	0.109
ATOM	763	HB3	ASP	A	48	16.820	25.869	29.063	1.00	42.53	H	0.109
ATOM	764	N	GLY	A	49	18.239	22.077	27.606	1.00	24.23	N	0.082
ATOM	765	CA	GLY	A	49	18.269	20.631	27.641	1.00	24.00	C	0.082
ATOM	766	C	GLY	A	49	16.929	19.950	27.446	1.00	14.00	C	0.063
ATOM	767	O	GLY	A	49	16.898	18.751	27.158	1.00	18.97	O	0.073
ATOM	768	H	GLY	A	49	18.317	22.406	26.815	1.00	28.95	H	0.090
ATOM	769	HA2	GLY	A	49	18.863	20.317	26.941	1.00	28.67	H	0.090
ATOM	770	HA3	GLY	A	49	18.619	20.350	28.502	1.00	28.67	H	0.090
ATOM	771	N	SER	A	50	15.822	20.673	27.585	1.00	17.13	N	0.069
ATOM	772	CA	SER	A	50	14.547	20.093	27.244	1.00	14.78	C	0.064
ATOM	773	C	SER	A	50	14.449	19.942	25.729	1.00	12.33	C	0.059
ATOM	774	O	SER	A	50	15.212	20.544	24.976	1.00	13.65	O	0.062
ATOM	775	CB	SER	A	50	13.399	20.952	27.770	1.00	17.91	C	0.071
ATOM	776	OG	SER	A	50	13.326	22.213	27.124	1.00	21.48	O	0.078
ATOM	777	H	SER	A	50	15.792	21.484	27.868	1.00	20.44	H	0.076
ATOM	778	HA	SER	A	50	14.466	19.218	27.655	1.00	17.61	H	0.070
ATOM	779	HB2	SER	A	50	12.566	20.479	27.622	1.00	21.37	H	0.077
ATOM	780	HB3	SER	A	50	13.533	21.098	28.720	1.00	21.37	H	0.077
ATOM	781	HG	SER	A	50	14.060	22.616	27.197	1.00	25.66	H	0.085
ATOM	782	N	THR	A	51	13.454	19.163	25.281	1.00	12.93	N	0.060
ATOM	783	CA	THR	A	51	13.205	18.956	23.854	1.00	9.14	C	0.051
ATOM	784	C	THR	A	51	11.718	19.171	23.580	1.00	10.75	C	0.055
ATOM	785	O	THR	A	51	10.881	18.872	24.432	1.00	10.95	O	0.055
ATOM	786	CB	THR	A	51	13.653	17.562	23.422	1.00	13.62	C	0.062
ATOM	787	OG1	THR	A	51	15.052	17.416	23.696	1.00	15.61	O	0.066
ATOM	788	CG2	THR	A	51	13.470	17.306	21.927	1.00	15.06	C	0.065
ATOM	789	H	THR	A	51	12.907	18.740	25.793	1.00	15.39	H	0.066
ATOM	790	HA	THR	A	51	13.699	19.597	23.319	1.00	10.85	H	0.055
ATOM	791	HB	THR	A	51	13.110	16.922	23.908	1.00	16.22	H	0.067
ATOM	792	HG1	THR	A	51	15.183	17.409	24.526	1.00	18.61	H	0.072
ATOM	793	HG21	THR	A	51	13.988	16.532	21.657	1.00	17.95	H	0.071
ATOM	794	HG22	THR	A	51	12.534	17.142	21.731	1.00	17.95	H	0.071
ATOM	795	HG23	THR	A	51	13.768	18.077	21.420	1.00	17.95	H	0.071
ATOM	796	N	ASP	A	52	11.417	19.747	22.412	1.00	12.37	N	0.059
ATOM	797	CA	ASP	A	52	10.069	19.973	21.931	1.00	12.23	C	0.059
ATOM	798	C	ASP	A	52	9.729	18.881	20.912	1.00	12.80	C	0.060
ATOM	799	O	ASP	A	52	10.532	18.582	20.026	1.00	17.14	O	0.069
ATOM	800	CB	ASP	A	52	9.942	21.339	21.239	1.00	12.63	C	0.059
ATOM	801	CG	ASP	A	52	10.148	22.508	22.158	1.00	22.37	C	0.079
ATOM	802	OD1	ASP	A	52	10.013	22.374	23.389	1.00	16.83	O	0.069
ATOM	803	OD2	ASP	A	52	10.407	23.598	21.611	1.00	30.12	O	0.092
ATOM	804	H	ASP	A	52	12.014	20.026	21.859	1.00	14.73	H	0.064
ATOM	805	HA	ASP	A	52	9.449	19.949	22.676	1.00	14.56	H	0.064
ATOM	806	HB2	ASP	A	52	10.609	21.395	20.536	1.00	15.03	H	0.065
ATOM	807	HB3	ASP	A	52	9.053	21.415	20.860	1.00	15.03	H	0.065
ATOM	808	N	TYR	A	53	8.520	18.314	21.004	1.00	10.55	N	0.054
ATOM	809	CA	TYR	A	53	8.142	17.161	20.218	1.00	11.36	C	0.056
ATOM	810	C	TYR	A	53	6.835	17.366	19.464	1.00	10.94	C	0.055
ATOM	811	O	TYR	A	53	5.834	17.765	20.055	1.00	11.70	O	0.057
ATOM	812	CB	TYR	A	53	7.970	15.962	21.141	1.00	8.78	C	0.050
ATOM	813	CG	TYR	A	53	9.242	15.522	21.836	1.00	14.80	C	0.064
ATOM	814	CD1	TYR	A	53	9.660	16.091	23.025	1.00	11.75	C	0.057
ATOM	815	CD2	TYR	A	53	10.011	14.516	21.285	1.00	15.00	C	0.065
ATOM	816	CE1	TYR	A	53	10.816	15.656	23.661	1.00	13.79	C	0.062
ATOM	817	CE2	TYR	A	53	11.177	14.097	21.882	1.00	16.54	C	0.068

```
ATOM    818  CZ  TYR A  53      11.573  14.663  23.078  1.00 13.55           C  0.062
ATOM    819  OH  TYR A  53      12.734  14.220  23.657  1.00 15.27           O  0.065
ATOM    820  H   TYR A  53       7.898  18.593  21.528  1.00 12.54           H  0.059
ATOM    821  HA  TYR A  53       8.832  17.018  19.551  1.00 13.51           H  0.061
ATOM    822  HB2 TYR A  53       7.324  16.190  21.828  1.00 10.41           H  0.054
ATOM    823  HB3 TYR A  53       7.647  15.212  20.617  1.00 10.41           H  0.054
ATOM    824  HD1 TYR A  53       9.159  16.776  23.405  1.00 13.97           H  0.063
ATOM    825  HD2 TYR A  53       9.734  14.114  20.493  1.00 17.87           H  0.071
ATOM    826  HE1 TYR A  53      11.076  16.030  24.471  1.00 16.42           H  0.068
ATOM    827  HE2 TYR A  53      11.696  13.436  21.483  1.00 19.73           H  0.074
ATOM    828  HH  TYR A  53      12.908  14.679  24.339  1.00 18.20           H  0.071
ATOM    829  N   GLY A  54       6.839  17.024  18.198  1.00 10.34           N  0.054
ATOM    830  CA  GLY A  54       5.608  16.761  17.448  1.00 10.85           C  0.055
ATOM    831  C   GLY A  54       5.055  17.987  16.746  1.00 15.62           C  0.066
ATOM    832  O   GLY A  54       5.681  19.032  16.649  1.00 12.12           O  0.058
ATOM    833  H   GLY A  54       7.553  16.932  17.727  1.00 12.29           H  0.059
ATOM    834  HA2 GLY A  54       5.785  16.084  16.777  1.00 12.89           H  0.060
ATOM    835  HA3 GLY A  54       4.930  16.432  18.058  1.00 12.89           H  0.060
ATOM    836  N   ILE A  55       3.827  17.822  16.247  1.00 15.46           N  0.066
ATOM    837  CA  ILE A  55       3.145  18.810  15.404  1.00 11.98           C  0.058
ATOM    838  C   ILE A  55       2.962  20.155  16.101  1.00 14.08           C  0.063
ATOM    839  O   ILE A  55       2.856  21.192  15.433  1.00 16.24           O  0.067
ATOM    840  CB  ILE A  55       1.804  18.150  14.960  1.00 18.27           C  0.072
ATOM    841  CG1 ILE A  55       1.179  18.855  13.778  1.00 40.35           C  0.106
ATOM    842  CG2 ILE A  55       0.842  17.894  16.128  1.00 19.24           C  0.073
ATOM    843  CD1 ILE A  55       1.509  18.175  12.520  1.00 30.88           C  0.093
ATOM    844  H   ILE A  55       3.348  17.121  16.386  1.00 18.44           H  0.072
ATOM    845  HA  ILE A  55       3.683  19.028  14.627  1.00 14.25           H  0.063
ATOM    846  HB  ILE A  55       2.029  17.265  14.634  1.00 21.80           H  0.078
ATOM    847 HG12 ILE A  55       0.214  18.861  13.880  1.00 48.30           H  0.116
ATOM    848 HG13 ILE A  55       1.513  19.765  13.733  1.00 48.30           H  0.116
ATOM    849 HG21 ILE A  55       0.031  17.484  15.786  1.00 22.97           H  0.080
ATOM    850 HG22 ILE A  55       1.269  17.299  16.765  1.00 22.97           H  0.080
ATOM    851 HG23 ILE A  55       0.630  18.739  16.555  1.00 22.97           H  0.080
ATOM    852 HD11 ILE A  55       1.093  18.652  11.785  1.00 36.93           H  0.102
ATOM    853 HD12 ILE A  55       2.472  18.169  12.408  1.00 36.93           H  0.102
ATOM    854 HD13 ILE A  55       1.174  17.266  12.555  1.00 36.93           H  0.102
ATOM    855  N   LEU A  56       2.885  20.158  17.425  1.00 10.84           N  0.055
ATOM    856  CA  LEU A  56       2.729  21.364  18.235  1.00 12.06           C  0.058
ATOM    857  C   LEU A  56       3.902  21.597  19.186  1.00 17.49           C  0.070
ATOM    858  O   LEU A  56       3.775  22.401  20.120  1.00 14.27           O  0.063
ATOM    859  CB  LEU A  56       1.421  21.295  19.021  1.00 15.58           C  0.066
ATOM    860  CG  LEU A  56       0.199  21.471  18.108  1.00 15.06           C  0.065
ATOM    861  CD1 LEU A  56      -1.046  21.198  18.916  1.00 18.64           C  0.072
ATOM    862  CD2 LEU A  56       0.070  22.874  17.530  1.00 16.48           C  0.068
ATOM    863  H   LEU A  56       2.921  19.443  17.901  1.00 12.88           H  0.060
ATOM    864  HA  LEU A  56       2.686  22.131  17.642  1.00 14.35           H  0.063
ATOM    865  HB2 LEU A  56       1.355  20.430  19.455  1.00 18.57           H  0.072
ATOM    866  HB3 LEU A  56       1.410  22.001  19.685  1.00 18.57           H  0.072
ATOM    867  HG  LEU A  56       0.302  20.857  17.364  1.00 17.95           H  0.071
ATOM    868 HD11 LEU A  56      -1.824  21.329  18.352  1.00 22.25           H  0.079
ATOM    869 HD12 LEU A  56      -1.020  20.283  19.237  1.00 22.25           H  0.079
ATOM    870 HD13 LEU A  56      -1.076  21.811  19.668  1.00 22.25           H  0.079
ATOM    871 HD21 LEU A  56      -0.818  22.982  17.156  1.00 19.66           H  0.074
ATOM    872 HD22 LEU A  56       0.211  23.522  18.238  1.00 19.66           H  0.074
ATOM    873 HD23 LEU A  56       0.738  22.992  16.836  1.00 19.66           H  0.074
ATOM    874  N   GLN A  57       5.028  20.900  18.987  1.00 12.73           N  0.060
ATOM    875  CA  GLN A  57       6.289  21.181  19.669  1.00  9.85           C  0.053
ATOM    876  C   GLN A  57       6.073  21.330  21.177  1.00 13.52           C  0.062
ATOM    877  O   GLN A  57       6.466  22.300  21.807  1.00 12.93           O  0.060
ATOM    878  CB  GLN A  57       6.959  22.398  19.060  1.00 10.59           C  0.054
ATOM    879  CG  GLN A  57       7.437  22.102  17.629  1.00 12.59           C  0.059
ATOM    880  CD  GLN A  57       8.649  21.196  17.656  1.00 12.02           C  0.058
ATOM    881  OE1 GLN A  57       9.774  21.676  17.859  1.00 13.46           O  0.061
ATOM    882  NE2 GLN A  57       8.439  19.933  17.429  1.00 12.93           N  0.060
ATOM    883  H   GLN A  57       5.083  20.237  18.442  1.00 15.16           H  0.065
ATOM    884  HA  GLN A  57       6.887  20.427  19.548  1.00 11.70           H  0.057
ATOM    885  HB2 GLN A  57       6.327  23.133  19.028  1.00 12.59           H  0.059
ATOM    886  HB3 GLN A  57       7.728  22.645  19.597  1.00 12.59           H  0.059
ATOM    887  HG2 GLN A  57       6.729  21.658  17.135  1.00 14.99           H  0.065
ATOM    888  HG3 GLN A  57       7.679  22.931  17.189  1.00 14.99           H  0.065
ATOM    889 HE21 GLN A  57       7.641  19.651  17.274  1.00 15.40           H  0.066
ATOM    890 HE22 GLN A  57       9.097  19.380  17.434  1.00 15.40           H  0.066
ATOM    891  N   ILE A  58       5.575  20.262  21.746  1.00 13.82           N  0.062
ATOM    892  CA  ILE A  58       5.287  20.183  23.180  1.00 13.36           C  0.061
ATOM    893  C   ILE A  58       6.564  19.800  23.916  1.00 19.73           C  0.074
ATOM    894  O   ILE A  58       7.290  18.901  23.492  1.00 16.18           O  0.067
ATOM    895  CB  ILE A  58       4.138  19.179  23.405  1.00 15.47           C  0.066
```

ATOM	896	CG1	ILE	A	58	2.823	19.829	22.939	1.00	17.81	C	0.071
ATOM	897	CG2	ILE	A	58	4.047	18.703	24.842	1.00	18.17	C	0.071
ATOM	898	CD1	ILE	A	58	1.666	18.866	22.827	1.00	24.25	C	0.082
ATOM	899	H	ILE	A	58	5.383	19.539	21.321	1.00	16.46	H	0.068
ATOM	900	HA	ILE	A	58	5.006	21.044	23.527	1.00	15.91	H	0.067
ATOM	901	HB	ILE	A	58	4.319	18.384	22.879	1.00	18.44	H	0.072
ATOM	902	HG12	ILE	A	58	2.573	20.516	23.577	1.00	21.25	H	0.077
ATOM	903	HG13	ILE	A	58	2.963	20.223	22.065	1.00	21.25	H	0.077
ATOM	904	HG21	ILE	A	58	3.257	18.150	24.942	1.00	21.69	H	0.078
ATOM	905	HG22	ILE	A	58	4.841	18.187	25.055	1.00	21.69	H	0.078
ATOM	906	HG23	ILE	A	58	3.988	19.474	25.427	1.00	21.69	H	0.078
ATOM	907	HD11	ILE	A	58	0.875	19.353	22.551	1.00	28.97	H	0.090
ATOM	908	HD12	ILE	A	58	1.883	18.187	22.169	1.00	28.97	H	0.090
ATOM	909	HD13	ILE	A	58	1.516	18.452	23.691	1.00	28.97	H	0.090
ATOM	910	N	ASN	A	59	6.829	20.491	25.025	1.00	14.67	N	0.064
ATOM	911	CA	ASN	A	59	8.123	20.524	25.677	1.00	16.66	C	0.068
ATOM	912	C	ASN	A	59	8.209	19.523	26.818	1.00	19.82	C	0.074
ATOM	913	O	ASN	A	59	7.266	19.386	27.612	1.00	22.18	O	0.079
ATOM	914	CB	ASN	A	59	8.373	21.920	26.244	1.00	22.93	C	0.080
ATOM	915	CG	ASN	A	59	9.729	22.043	26.869	1.00	28.57	C	0.089
ATOM	916	OD1	ASN	A	59	9.887	21.890	28.073	1.00	31.49	O	0.094
ATOM	917	ND2	ASN	A	59	10.716	22.321	26.056	1.00	33.95	N	0.097
ATOM	918	H	ASN	A	59	6.242	20.969	25.433	1.00	17.49	H	0.070
ATOM	919	HA	ASN	A	59	8.802	20.288	25.026	1.00	19.87	H	0.075
ATOM	920	HB2	ASN	A	59	8.311	22.570	25.527	1.00	27.40	H	0.088
ATOM	921	HB3	ASN	A	59	7.708	22.111	26.924	1.00	27.40	H	0.088
ATOM	922	HD21	ASN	A	59	11.515	22.403	26.361	1.00	40.62	H	0.107
ATOM	923	HD22	ASN	A	59	10.564	22.422	25.215	1.00	40.62	H	0.107
ATOM	924	N	SER	A	60	9.383	18.879	26.922	1.00	15.07	N	0.065
ATOM	925	CA	SER	A	60	9.685	17.890	27.927	1.00	15.15	C	0.065
ATOM	926	C	SER	A	60	9.963	18.478	29.313	1.00	16.05	C	0.067
ATOM	927	O	SER	A	60	10.129	17.712	30.262	1.00	21.30	O	0.077
ATOM	928	CB	SER	A	60	10.882	17.054	27.472	1.00	13.18	C	0.061
ATOM	929	OG	SER	A	60	12.032	17.838	27.439	1.00	12.76	O	0.060
ATOM	930	H	SER	A	60	10.043	19.015	26.389	1.00	17.96	H	0.071
ATOM	931	HA	SER	A	60	8.917	17.305	28.022	1.00	18.06	H	0.071
ATOM	932	HB2	SER	A	60	11.014	16.321	28.094	1.00	15.69	H	0.066
ATOM	933	HB3	SER	A	60	10.708	16.706	26.583	1.00	15.69	H	0.066
ATOM	934	HG	SER	A	60	12.685	17.380	27.174	1.00	15.19	H	0.065
ATOM	935	N	ARG	A	61	10.026	19.784	29.462	1.00	21.12	N	0.077
ATOM	936	CA	ARG	A	61	10.263	20.318	30.809	1.00	23.04	C	0.080
ATOM	937	C	ARG	A	61	9.013	20.216	31.657	1.00	24.07	C	0.082
ATOM	938	O	ARG	A	61	9.108	20.019	32.872	1.00	25.80	O	0.085
ATOM	939	CB	ARG	A	61	10.724	21.763	30.715	1.00	19.37	C	0.074
ATOM	940	CG	ARG	A	61	11.007	22.483	32.055	1.00	40.43	C	0.106
ATOM	941	CD	ARG	A	61	11.790	21.652	33.080	1.00	42.40	C	0.109
ATOM	942	NE	ARG	A	61	11.854	22.297	34.396	1.00	59.42	N	0.129
ATOM	943	CZ	ARG	A	61	10.899	22.265	35.321	1.00	47.04	C	0.115
ATOM	944	NH1	ARG	A	61	9.787	21.568	35.156	1.00	31.49	N	0.094
ATOM	945	NH2	ARG	A	61	11.069	22.944	36.453	1.00	54.74	N	0.124
ATOM	946	H	ARG	A	61	9.941	20.367	28.835	1.00	25.22	H	0.084
ATOM	947	HA	ARG	A	61	10.968	19.813	31.243	1.00	27.53	H	0.088
ATOM	948	HB2	ARG	A	61	11.547	21.784	30.201	1.00	23.12	H	0.080
ATOM	949	HB3	ARG	A	61	10.035	22.271	30.259	1.00	23.12	H	0.080
ATOM	950	HG2	ARG	A	61	11.525	23.282	31.873	1.00	48.39	H	0.116
ATOM	951	HG3	ARG	A	61	10.160	22.722	32.462	1.00	48.39	H	0.116
ATOM	952	HD2	ARG	A	61	11.357	20.791	33.189	1.00	50.76	H	0.119
ATOM	953	HD3	ARG	A	61	12.698	21.528	32.762	1.00	50.76	H	0.119
ATOM	954	HE	ARG	A	61	12.571	22.733	34.586	1.00	71.18	H	0.141
ATOM	955	HH11	ARG	A	61	9.665	21.118	34.433	1.00	37.66	H	0.103
ATOM	956	HH12	ARG	A	61	9.186	21.566	35.771	1.00	37.66	H	0.103
ATOM	957	HH21	ARG	A	61	11.790	23.396	36.579	1.00	65.56	H	0.135
ATOM	958	HH22	ARG	A	61	10.458	22.930	37.058	1.00	65.56	H	0.135
ATOM	959	N	TRP	A	62	7.856	20.283	31.034	1.00	15.20	N	0.065
ATOM	960	CA	TRP	A	62	6.605	20.266	31.769	1.00	17.42	C	0.070
ATOM	961	C	TRP	A	62	5.663	19.140	31.412	1.00	21.12	C	0.077
ATOM	962	O	TRP	A	62	4.991	18.627	32.308	1.00	21.85	O	0.078
ATOM	963	CB	TRP	A	62	5.876	21.593	31.552	1.00	23.76	C	0.082
ATOM	964	CG	TRP	A	62	6.589	22.729	32.179	1.00	21.54	C	0.078
ATOM	965	CD1	TRP	A	62	7.231	23.748	31.543	1.00	35.17	C	0.099
ATOM	966	CD2	TRP	A	62	6.748	22.962	33.584	1.00	33.16	C	0.096
ATOM	967	NE1	TRP	A	62	7.758	24.619	32.469	1.00	28.30	N	0.089
ATOM	968	CE2	TRP	A	62	7.483	24.152	33.727	1.00	30.95	C	0.093
ATOM	969	CE3	TRP	A	62	6.334	22.277	34.739	1.00	32.28	C	0.095
ATOM	970	CZ2	TRP	A	62	7.823	24.673	34.974	1.00	46.04	C	0.114
ATOM	971	CZ3	TRP	A	62	6.667	22.802	35.981	1.00	43.59	C	0.110
ATOM	972	CH2	TRP	A	62	7.403	23.988	36.086	1.00	47.02	C	0.115
ATOM	973	H	TRP	A	62	7.766	20.340	30.180	1.00	18.12	H	0.071

ATOM	974	HA	TRP	A	62	6.820	20.157	32.709	1.00	20.78	H	0.076
ATOM	975	HB2	TRP	A	62	5.808	21.766	30.600	1.00	28.39	H	0.089
ATOM	976	HB3	TRP	A	62	4.990	21.537	31.943	1.00	28.39	H	0.089
ATOM	977	HD1	TRP	A	62	7.302	23.841	30.620	1.00	42.08	H	0.109
ATOM	978	HE1	TRP	A	62	8.190	25.340	32.288	1.00	33.84	H	0.097
ATOM	979	HE3	TRP	A	62	5.846	21.487	34.673	1.00	38.61	H	0.104
ATOM	980	HZ2	TRP	A	62	8.318	25.457	35.050	1.00	55.12	H	0.124
ATOM	981	HZ3	TRP	A	62	6.397	22.360	36.753	1.00	52.19	H	0.121
ATOM	982	HH2	TRP	A	62	7.613	24.319	36.929	1.00	56.30	H	0.126
ATOM	983	N	TRP	A	63	5.606	18.715	30.136	1.00	17.09	N	0.069
ATOM	984	CA	TRP	A	63	4.432	18.031	29.603	1.00	16.88	C	0.069
ATOM	985	C	TRP	A	63	4.599	16.562	29.282	1.00	16.13	C	0.067
ATOM	986	O	TRP	A	63	3.650	15.798	29.437	1.00	14.18	O	0.063
ATOM	987	CB	TRP	A	63	3.955	18.745	28.327	1.00	13.57	C	0.062
ATOM	988	CG	TRP	A	63	3.685	20.184	28.625	1.00	15.28	C	0.065
ATOM	989	CD1	TRP	A	63	4.377	21.251	28.169	1.00	18.09	C	0.071
ATOM	990	CD2	TRP	A	63	2.636	20.714	29.462	1.00	15.87	C	0.067
ATOM	991	NE1	TRP	A	63	3.838	22.420	28.670	1.00	18.85	N	0.073
ATOM	992	CE2	TRP	A	63	2.774	22.111	29.471	1.00	15.31	C	0.065
ATOM	993	CE3	TRP	A	63	1.586	20.147	30.167	1.00	14.99	C	0.065
ATOM	994	CZ2	TRP	A	63	1.877	22.956	30.184	1.00	21.55	C	0.078
ATOM	995	CZ3	TRP	A	63	0.722	20.970	30.892	1.00	22.91	C	0.080
ATOM	996	CH2	TRP	A	63	0.877	22.347	30.894	1.00	17.72	C	0.070
ATOM	997	H	TRP	A	63	6.240	18.815	29.564	1.00	20.38	H	0.076
ATOM	998	HA	TRP	A	63	3.743	18.078	30.284	1.00	20.13	H	0.075
ATOM	999	HB2	TRP	A	63	4.643	18.690	27.646	1.00	16.16	H	0.067
ATOM	1000	HB3	TRP	A	63	3.138	18.333	28.008	1.00	16.16	H	0.067
ATOM	1001	HD1	TRP	A	63	5.111	21.205	27.600	1.00	21.58	H	0.078
ATOM	1002	HE1	TRP	A	63	4.126	23.213	28.505	1.00	22.49	H	0.079
ATOM	1003	HE3	TRP	A	63	1.457	19.226	30.156	1.00	17.86	H	0.071
ATOM	1004	HZ2	TRP	A	63	1.967	23.882	30.168	1.00	25.74	H	0.085
ATOM	1005	HZ3	TRP	A	63	0.031	20.588	31.384	1.00	27.37	H	0.088
ATOM	1006	HH2	TRP	A	63	0.287	22.870	31.388	1.00	21.14	H	0.077
ATOM	1007	N	CYS	A	64	5.783	16.123	28.855	1.00	15.24	N	0.065
ATOM	1008	CA	CYS	A	64	5.945	14.714	28.517	1.00	14.37	C	0.063
ATOM	1009	C	CYS	A	64	7.273	14.254	29.079	1.00	14.54	C	0.064
ATOM	1010	O	CYS	A	64	8.132	15.068	29.422	1.00	14.96	O	0.065
ATOM	1011	CB	CYS	A	64	5.862	14.492	27.012	1.00	16.55	C	0.068
ATOM	1012	SG	CYS	A	64	7.153	15.422	26.103	1.00	16.76	S	0.068
ATOM	1013	H	CYS	A	64	6.486	16.609	28.758	1.00	18.16	H	0.071
ATOM	1014	HA	CYS	A	64	5.245	14.177	28.920	1.00	17.12	H	0.069
ATOM	1015	HB2	CYS	A	64	5.980	13.547	26.823	1.00	19.73	H	0.074
ATOM	1016	HB3	CYS	A	64	4.995	14.788	26.694	1.00	19.73	H	0.074
ATOM	1017	N	ASN	A	65	7.419	12.944	29.212	1.00	14.11	N	0.063
ATOM	1018	CA	ASN	A	65	8.650	12.360	29.716	1.00	14.45	C	0.064
ATOM	1019	C	ASN	A	65	9.525	11.830	28.587	1.00	15.60	C	0.066
ATOM	1020	O	ASN	A	65	9.087	10.984	27.807	1.00	15.93	O	0.067
ATOM	1021	CB	ASN	A	65	8.416	11.208	30.691	1.00	19.86	C	0.075
ATOM	1022	CG	ASN	A	65	9.728	10.622	31.173	1.00	23.51	C	0.081
ATOM	1023	OD1	ASN	A	65	10.567	11.347	31.705	1.00	18.85	O	0.073
ATOM	1024	ND2	ASN	A	65	9.961	9.339	30.891	1.00	20.88	N	0.076
ATOM	1025	H	ASN	A	65	6.812	12.367	29.014	1.00	16.81	H	0.069
ATOM	1026	HA	ASN	A	65	9.104	13.088	30.170	1.00	17.22	H	0.069
ATOM	1027	HB2	ASN	A	65	7.924	11.533	31.462	1.00	23.71	H	0.081
ATOM	1028	HB3	ASN	A	65	7.912	10.508	30.248	1.00	23.71	H	0.081
ATOM	1029	HD21	ASN	A	65	10.694	8.969	31.147	1.00	24.93	H	0.084
ATOM	1030	HD22	ASN	A	65	9.378	8.883	30.454	1.00	24.93	H	0.084
ATOM	1031	N	ASP	A	66	10.777	12.305	28.520	1.00	13.50	N	0.061
ATOM	1032	CA	ASP	A	66	11.740	11.748	27.580	1.00	16.25	C	0.067
ATOM	1033	C	ASP	A	66	12.934	11.129	28.294	1.00	15.58	C	0.066
ATOM	1034	O	ASP	A	66	13.824	10.605	27.620	1.00	17.12	O	0.069
ATOM	1035	CB	ASP	A	66	12.212	12.774	26.527	1.00	14.47	C	0.064
ATOM	1036	CG	ASP	A	66	12.954	13.953	27.095	1.00	16.01	C	0.067
ATOM	1037	OD1	ASP	A	66	13.344	13.959	28.286	1.00	15.58	O	0.066
ATOM	1038	OD2	ASP	A	66	13.224	14.903	26.293	1.00	13.61	O	0.062
ATOM	1039	H	ASP	A	66	11.082	12.945	29.008	1.00	16.07	H	0.067
ATOM	1040	HA	ASP	A	66	11.293	11.059	27.065	1.00	19.38	H	0.074
ATOM	1041	HB2	ASP	A	66	12.805	12.327	25.904	1.00	17.24	H	0.069
ATOM	1042	HB3	ASP	A	66	11.434	13.116	26.058	1.00	17.24	H	0.069
ATOM	1043	N	GLY	A	67	12.929	11.102	29.623	1.00	18.57	N	0.072
ATOM	1044	CA	GLY	A	67	13.990	10.517	30.403	1.00	21.44	C	0.077
ATOM	1045	C	GLY	A	67	15.298	11.266	30.400	1.00	23.22	C	0.081
ATOM	1046	O	GLY	A	67	16.265	10.796	31.015	1.00	22.75	O	0.080
ATOM	1047	H	GLY	A	67	12.296	11.430	30.105	1.00	22.17	H	0.079
ATOM	1048	HA2	GLY	A	67	13.693	10.452	31.324	1.00	25.61	H	0.085
ATOM	1049	HA3	GLY	A	67	14.164	9.625	30.063	1.00	25.61	H	0.085
ATOM	1050	N	AARG	A	68	15.379	12.416	29.728	0.49	17.24	N	0.069
ATOM	1051	CA	AARG	A	68	16.651	13.121	29.642	0.49	19.65	C	0.074

```
ATOM   1052  C  AARG A  68      16.519  14.600  29.986  0.49 23.63           C  0.081
ATOM   1053  O  AARG A  68      17.405  15.393  29.640  0.49 21.95           O  0.078
ATOM   1054  CB AARG A  68      17.264  12.907  28.251  0.49 21.74           C  0.078
ATOM   1055  CG AARG A  68      16.557  13.623  27.118  0.49 20.90           C  0.076
ATOM   1056  CD AARG A  68      17.496  13.806  25.955  0.49 22.71           C  0.080
ATOM   1057  NE AARG A  68      16.913  14.572  24.866  0.49 18.63           N  0.072
ATOM   1058  CZ AARG A  68      17.425  14.621  23.647  0.49 21.69           C  0.078
ATOM   1059  NH1AARG A  68      18.472  13.880  23.311  0.49 19.21           N  0.073
ATOM   1060  NH2AARG A  68      16.861  15.411  22.740  0.49 18.80           N  0.073
ATOM   1061  H  AARG A  68      14.724  12.797  29.322  0.49 20.57           H  0.076
ATOM   1062  HA AARG A  68      17.269  12.759  30.296  0.49 23.46           H  0.081
ATOM   1063  HB2AARG A  68      18.180  13.224  28.269  0.49 25.96           H  0.085
ATOM   1064  HB3AARG A  68      17.245  11.958  28.051  0.49 25.96           H  0.085
ATOM   1065  HG2AARG A  68      15.797  13.098  26.823  0.49 24.96           H  0.084
ATOM   1066  HG3AARG A  68      16.261  14.497  27.418  0.49 24.96           H  0.084
ATOM   1067  HD2AARG A  68      18.288  14.277  26.261  0.49 27.13           H  0.087
ATOM   1068  HD3AARG A  68      17.742  12.934  25.609  0.49 27.13           H  0.087
ATOM   1069  HE AARG A  68      16.195  15.018  25.023  0.49 22.24           H  0.079
ATOM   1070 HH11AARG A  68      18.831  13.355  23.890  0.49 22.93           H  0.080
ATOM   1071 HH12AARG A  68      18.792  13.924  22.514  0.49 22.93           H  0.080
ATOM   1072 HH21AARG A  68      16.172  15.882  22.948  0.49 22.43           H  0.079
ATOM   1073 HH22AARG A  68      17.185  15.451  21.944  0.49 22.43           H  0.079
ATOM   1074  N  BARG A  68      15.376  12.416  29.727  0.51 17.24           N  0.069
ATOM   1075  CA BARG A  68      16.648  13.122  29.632  0.51 19.65           C  0.074
ATOM   1076  C  BARG A  68      16.514  14.601  29.976  0.51 23.64           C  0.081
ATOM   1077  O  BARG A  68      17.403  15.394  29.638  0.51 21.95           O  0.078
ATOM   1078  CB BARG A  68      17.244  12.911  28.232  0.51 21.73           C  0.078
ATOM   1079  CG BARG A  68      16.339  13.374  27.098  0.51 21.66           C  0.078
ATOM   1080  CD BARG A  68      17.110  13.765  25.850  0.51 20.67           C  0.076
ATOM   1081  NE BARG A  68      17.629  15.120  25.983  0.51 20.15           N  0.075
ATOM   1082  CZ BARG A  68      18.913  15.426  26.132  0.51 18.23           C  0.071
ATOM   1083  NH1BARG A  68      19.859  14.506  26.053  0.51 27.44           N  0.088
ATOM   1084  NH2BARG A  68      19.255  16.680  26.395  0.51 20.26           N  0.075
ATOM   1085  H  BARG A  68      14.718  12.798  29.326  0.51 20.56           H  0.076
ATOM   1086  HA BARG A  68      17.274  12.760  30.279  0.51 23.46           H  0.081
ATOM   1087  HB2BARG A  68      18.074  13.409  28.169  0.51 25.95           H  0.085
ATOM   1088  HB3BARG A  68      17.414  11.965  28.106  0.51 25.95           H  0.085
ATOM   1089  HG2BARG A  68      15.734  12.654  26.862  0.51 25.87           H  0.085
ATOM   1090  HG3BARG A  68      15.835  14.149  27.392  0.51 25.87           H  0.085
ATOM   1091  HD2BARG A  68      17.856  13.158  25.724  0.51 24.68           H  0.083
ATOM   1092  HD3BARG A  68      16.522  13.732  25.080  0.51 24.68           H  0.083
ATOM   1093  HE BARG A  68      17.064  15.767  25.964  0.51 24.06           H  0.082
ATOM   1094 HH11BARG A  68      19.650  13.686  25.902  0.51 32.81           H  0.096
ATOM   1095 HH12BARG A  68      20.684  14.728  26.153  0.51 32.81           H  0.096
ATOM   1096 HH21BARG A  68      18.647  17.285  26.467  0.51 24.19           H  0.082
ATOM   1097 HH22BARG A  68      20.083  16.889  26.493  0.51 24.19           H  0.082
ATOM   1098  N   THR A  69      15.446  14.991  30.683  1.00 13.58           N  0.062
ATOM   1099  CA  THR A  69      15.225  16.390  31.074  1.00 14.53           C  0.064
ATOM   1100  C   THR A  69      15.142  16.493  32.588  1.00 16.70           C  0.068
ATOM   1101  O   THR A  69      14.053  16.400  33.175  1.00 22.45           O  0.079
ATOM   1102  CB  THR A  69      13.951  16.930  30.429  1.00 14.00           C  0.063
ATOM   1103  OG1 THR A  69      14.024  16.651  29.028  1.00 14.50           O  0.064
ATOM   1104  CG2 THR A  69      13.809  18.418  30.636  1.00 19.66           C  0.074
ATOM   1105  HA  THR A  69      15.986  16.916  30.784  1.00 17.31           H  0.070
ATOM   1106  HB  THR A  69      13.173  16.514  30.831  1.00 16.68           H  0.068
ATOM   1107  HG1 THR A  69      14.030  15.821  28.900  1.00 17.28           H  0.070
ATOM   1108 HG21 THR A  69      12.954  18.720  30.291  1.00 23.47           H  0.081
ATOM   1109 HG22 THR A  69      13.859  18.628  31.581  1.00 23.47           H  0.081
ATOM   1110 HG23 THR A  69      14.519  18.888  30.171  1.00 23.47           H  0.081
ATOM   1111  N   PRO A  70      16.263  16.688  33.267  1.00 16.56           N  0.068
ATOM   1112  CA  PRO A  70      16.244  16.585  34.730  1.00 19.47           C  0.074
ATOM   1113  C   PRO A  70      15.311  17.580  35.383  1.00 23.64           C  0.081
ATOM   1114  O   PRO A  70      15.159  18.722  34.936  1.00 33.13           O  0.096
ATOM   1115  CB  PRO A  70      17.706  16.855  35.125  1.00 37.96           C  0.103
ATOM   1116  CG  PRO A  70      18.478  16.457  33.948  1.00 27.75           C  0.088
ATOM   1117  CD  PRO A  70      17.628  16.834  32.753  1.00 22.86           C  0.080
ATOM   1118  HA  PRO A  70      15.979  15.688  34.987  1.00 23.25           H  0.081
ATOM   1119  HB2 PRO A  70      17.829  17.797  35.322  1.00 45.43           H  0.113
ATOM   1120  HB3 PRO A  70      17.946  16.320  35.898  1.00 45.43           H  0.113
ATOM   1121  HG2 PRO A  70      19.323  16.934  33.933  1.00 33.18           H  0.096
ATOM   1122  HG3 PRO A  70      18.637  15.500  33.968  1.00 33.18           H  0.096
ATOM   1123  HD2 PRO A  70      17.800  17.749  32.479  1.00 27.30           H  0.087
ATOM   1124  HD3 PRO A  70      17.786  16.231  32.010  1.00 27.30           H  0.087
ATOM   1125  N   GLY A  71      14.688  17.128  36.470  1.00 27.67           N  0.088
ATOM   1126  CA  GLY A  71      13.848  17.987  37.270  1.00 37.61           C  0.103
ATOM   1127  C   GLY A  71      12.556  18.344  36.596  1.00 34.14           C  0.098
ATOM   1128  O   GLY A  71      11.914  19.329  36.972  1.00 37.28           O  0.102
ATOM   1129  H   GLY A  71      14.742  16.320  36.762  1.00 33.08           H  0.096
```

ATOM	1130	HA2	GLY	A	71	13.641	17.540	38.105	1.00	45.01	H	0.112
ATOM	1131	HA3	GLY	A	71	14.326	18.809	37.462	1.00	45.01	H	0.112
ATOM	1132	N	SER	A	72	12.158	17.567	35.605	1.00	29.98	N	0.092
ATOM	1133	CA	SER	A	72	11.029	17.930	34.773	1.00	42.32	C	0.109
ATOM	1134	C	SER	A	72	9.792	17.140	35.169	1.00	35.03	C	0.099
ATOM	1135	O	SER	A	72	9.845	16.087	35.810	1.00	35.26	O	0.099
ATOM	1136	CB	SER	A	72	11.356	17.710	33.289	1.00	37.40	C	0.102
ATOM	1137	OG	SER	A	72	11.549	16.335	33.003	1.00	30.64	O	0.093
ATOM	1138	H	SER	A	72	12.528	16.820	35.395	1.00	35.86	H	0.100
ATOM	1139	HA	SER	A	72	10.829	18.871	34.897	1.00	50.66	H	0.119
ATOM	1140	HB2	SER	A	72	10.618	18.042	32.753	1.00	44.75	H	0.112
ATOM	1141	HB3	SER	A	72	12.168	18.194	33.071	1.00	44.75	H	0.112
ATOM	1142	HG	SER	A	72	12.174	16.028	33.473	1.00	36.65	H	0.101
ATOM	1143	N	ARG	A	73	8.659	17.694	34.795	1.00	23.19	N	0.081
ATOM	1144	CA	ARG	A	73	7.384	17.058	35.031	1.00	27.16	C	0.087
ATOM	1145	C	ARG	A	73	6.934	16.424	33.723	1.00	27.92	C	0.088
ATOM	1146	O	ARG	A	73	7.547	16.619	32.654	1.00	21.34	O	0.077
ATOM	1147	CB	ARG	A	73	6.372	18.082	35.567	1.00	25.42	C	0.084
ATOM	1148	CG	ARG	A	73	6.932	18.894	36.737	1.00	27.91	C	0.088
ATOM	1149	CD	ARG	A	73	6.697	18.228	38.108	1.00	35.87	C	0.100
ATOM	1150	NE	ARG	A	73	5.434	18.674	38.699	1.00	51.81	N	0.120
ATOM	1151	CZ	ARG	A	73	4.404	17.888	38.988	1.00	64.83	C	0.135
ATOM	1152	NH1	ARG	A	73	4.458	16.574	38.801	1.00	52.18	N	0.121
ATOM	1153	NH2	ARG	A	73	3.293	18.434	39.481	1.00	48.31	N	0.116
ATOM	1154	H	ARG	A	73	8.602	18.454	34.396	1.00	27.71	H	0.088
ATOM	1155	HA	ARG	A	73	7.446	16.363	35.705	1.00	32.47	H	0.095
ATOM	1156	HB2	ARG	A	73	6.138	18.699	34.856	1.00	30.38	H	0.092
ATOM	1157	HB3	ARG	A	73	5.581	17.614	35.876	1.00	30.38	H	0.092
ATOM	1158	HG2	ARG	A	73	7.888	19.000	36.618	1.00	33.36	H	0.097
ATOM	1159	HG3	ARG	A	73	6.501	19.764	36.751	1.00	33.36	H	0.097
ATOM	1160	HD2	ARG	A	73	6.660	17.265	37.997	1.00	42.92	H	0.110
ATOM	1161	HD3	ARG	A	73	7.419	18.466	38.710	1.00	42.92	H	0.110
ATOM	1162	HE	ARG	A	73	5.353	19.513	38.874	1.00	62.05	H	0.132
ATOM	1163	HH11	ARG	A	73	5.172	16.213	38.486	1.00	62.49	H	0.132
ATOM	1164	HH12	ARG	A	73	3.778	16.084	38.995	1.00	62.49	H	0.132
ATOM	1165	HH21	ARG	A	73	3.251	19.283	39.608	1.00	57.85	H	0.127
ATOM	1166	HH22	ARG	A	73	2.618	17.936	39.672	1.00	57.85	H	0.127
ATOM	1167	N	ASN	A	74	5.896	15.616	33.852	1.00	19.12	N	0.073
ATOM	1168	CA	ASN	A	74	5.212	14.885	32.792	1.00	17.93	C	0.071
ATOM	1169	C	ASN	A	74	3.700	15.066	33.007	1.00	16.13	C	0.067
ATOM	1170	O	ASN	A	74	2.942	14.137	33.286	1.00	18.95	O	0.073
ATOM	1171	CB	ASN	A	74	5.632	13.431	32.862	1.00	17.65	C	0.070
ATOM	1172	CG	ASN	A	74	4.959	12.586	31.870	1.00	19.47	C	0.074
ATOM	1173	OD1	ASN	A	74	4.303	13.070	30.969	1.00	18.16	O	0.071
ATOM	1174	ND2	ASN	A	74	5.113	11.257	32.022	1.00	21.61	N	0.078
ATOM	1175	H	ASN	A	74	5.532	15.458	34.615	1.00	22.82	H	0.080
ATOM	1176	HA	ASN	A	74	5.426	15.230	31.911	1.00	21.40	H	0.077
ATOM	1177	HB2	ASN	A	74	6.588	13.372	32.703	1.00	21.06	H	0.077
ATOM	1178	HB3	ASN	A	74	5.420	13.084	33.742	1.00	21.06	H	0.077
ATOM	1179	HD21	ASN	A	74	4.739	10.715	31.468	1.00	25.82	H	0.085
ATOM	1180	HD22	ASN	A	74	5.585	10.950	32.672	1.00	25.82	H	0.085
ATOM	1181	N	LEU	A	75	3.285	16.321	32.949	1.00	17.66	N	0.070
ATOM	1182	CA	LEU	A	75	1.911	16.679	33.252	1.00	23.19	C	0.081
ATOM	1183	C	LEU	A	75	0.897	16.067	32.294	1.00	23.61	C	0.081
ATOM	1184	O	LEU	A	75	-0.270	15.915	32.680	1.00	21.10	O	0.077
ATOM	1185	CB	LEU	A	75	1.831	18.204	33.275	1.00	19.29	C	0.073
ATOM	1186	CG	LEU	A	75	2.553	18.835	34.477	1.00	25.33	C	0.084
ATOM	1187	CD1	LEU	A	75	2.501	20.353	34.445	1.00	30.73	C	0.093
ATOM	1188	CD2	LEU	A	75	1.974	18.356	35.800	1.00	29.62	C	0.091
ATOM	1189	H	LEU	A	75	3.782	16.989	32.735	1.00	21.06	H	0.077
ATOM	1190	HA	LEU	A	75	1.659	16.338	34.124	1.00	27.70	H	0.088
ATOM	1191	HB2	LEU	A	75	2.239	18.551	32.466	1.00	23.03	H	0.080
ATOM	1192	HB3	LEU	A	75	0.898	18.467	33.315	1.00	23.03	H	0.080
ATOM	1193	HG	LEU	A	75	3.479	18.553	34.415	1.00	30.27	H	0.092
ATOM	1194	HD11	LEU	A	75	3.396	20.702	34.577	1.00	36.75	H	0.101
ATOM	1195	HD12	LEU	A	75	2.157	20.639	33.585	1.00	36.75	H	0.101
ATOM	1196	HD13	LEU	A	75	1.917	20.664	35.155	1.00	36.75	H	0.101
ATOM	1197	HD21	LEU	A	75	2.351	18.886	36.520	1.00	35.42	H	0.100
ATOM	1198	HD22	LEU	A	75	1.010	18.462	35.780	1.00	35.42	H	0.100
ATOM	1199	HD23	LEU	A	75	2.201	17.421	35.924	1.00	35.42	H	0.100
ATOM	1200	N	CYS	A	76	1.279	15.688	31.070	1.00	15.84	N	0.067
ATOM	1201	CA	CYS	A	76	0.350	14.985	30.176	1.00	18.24	C	0.071
ATOM	1202	C	CYS	A	76	0.395	13.473	30.347	1.00	21.03	C	0.077
ATOM	1203	O	CYS	A	76	-0.389	12.748	29.712	1.00	22.81	O	0.080
ATOM	1204	CB	CYS	A	76	0.643	15.353	28.735	1.00	15.64	C	0.066
ATOM	1205	SG	CYS	A	76	0.152	17.115	28.377	1.00	17.63	S	0.070
ATOM	1206	H	CYS	A	76	2.061	15.825	30.738	1.00	18.88	H	0.073
ATOM	1207	HA	CYS	A	76	-0.554	15.272	30.375	1.00	21.77	H	0.078

ATOM	1208	HB2	CYS	A	76	1.593	15.259	28.565	1.00	18.64	H	0.072
ATOM	1209	HB3	CYS	A	76	0.141	14.769	28.145	1.00	18.64	H	0.072
ATOM	1210	N	ASN	A	77	1.282	12.998	31.203	1.00	16.65	N	0.068
ATOM	1211	CA	ASN	A	77	1.397	11.578	31.494	1.00	15.57	C	0.066
ATOM	1212	C	ASN	A	77	1.588	10.768	30.224	1.00	19.20	C	0.073
ATOM	1213	O	ASN	A	77	0.898	9.779	29.963	1.00	20.40	O	0.076
ATOM	1214	CB	ASN	A	77	0.167	11.152	32.279	1.00	23.17	C	0.081
ATOM	1215	CG	ASN	A	77	0.064	11.892	33.596	1.00	32.08	C	0.095
ATOM	1216	OD1	ASN	A	77	0.967	11.825	34.437	1.00	38.58	O	0.104
ATOM	1217	ND2	ASN	A	77	-0.975	12.702	33.728	1.00	42.83	N	0.109
ATOM	1218	H	ASN	A	77	1.841	13.487	31.637	1.00	19.85	H	0.075
ATOM	1219	HA	ASN	A	77	2.187	11.404	32.030	1.00	18.57	H	0.072
ATOM	1220	HB2	ASN	A	77	-0.628	11.344	31.758	1.00	27.68	H	0.088
ATOM	1221	HB3	ASN	A	77	0.220	10.202	32.467	1.00	27.68	H	0.088
ATOM	1222	HD21	ASN	A	77	-1.542	12.783	33.086	1.00	51.27	H	0.120
ATOM	1223	HD22	ASN	A	77	-1.083	13.148	34.456	1.00	51.27	H	0.120
ATOM	1224	N	ILE	A	78	2.593	11.163	29.439	1.00	16.88	N	0.069
ATOM	1225	CA	ILE	A	78	2.867	10.436	28.212	1.00	14.50	C	0.064
ATOM	1226	C	ILE	A	78	4.368	10.459	27.964	1.00	16.52	C	0.068
ATOM	1227	O	ILE	A	78	5.064	11.421	28.337	1.00	16.34	O	0.068
ATOM	1228	CB	ILE	A	78	2.150	11.025	26.985	1.00	21.07	C	0.077
ATOM	1229	CG1	ILE	A	78	2.400	12.538	26.903	1.00	21.67	C	0.078
ATOM	1230	CG2	ILE	A	78	0.677	10.648	26.972	1.00	36.84	C	0.102
ATOM	1231	CD1	ILE	A	78	2.372	13.046	25.497	1.00	27.35	C	0.087
ATOM	1232	H	ILE	A	78	3.112	11.830	29.597	1.00	20.14	H	0.075
ATOM	1233	HA	ILE	A	78	2.551	9.528	28.342	1.00	17.28	H	0.070
ATOM	1234	HB	ILE	A	78	2.526	10.635	26.181	1.00	25.16	H	0.084
ATOM	1235	HG12	ILE	A	78	1.710	13.001	27.404	1.00	25.89	H	0.085
ATOM	1236	HG13	ILE	A	78	3.272	12.736	27.278	1.00	25.89	H	0.085
ATOM	1237	HG21	ILE	A	78	0.260	11.030	26.184	1.00	44.09	H	0.111
ATOM	1238	HG22	ILE	A	78	0.598	9.681	26.952	1.00	44.09	H	0.111
ATOM	1239	HG23	ILE	A	78	0.254	10.997	27.772	1.00	44.09	H	0.111
ATOM	1240	HD11	ILE	A	78	2.501	14.008	25.507	1.00	32.70	H	0.096
ATOM	1241	HD12	ILE	A	78	3.084	12.622	24.994	1.00	32.70	H	0.096
ATOM	1242	HD13	ILE	A	78	1.513	12.832	25.101	1.00	32.70	H	0.096
ATOM	1243	N	PRO	A	79	4.900	9.423	27.352	1.00	22.89	N	0.080
ATOM	1244	CA	PRO	A	79	6.239	9.535	26.776	1.00	17.16	C	0.069
ATOM	1245	C	PRO	A	79	6.259	10.556	25.639	1.00	17.98	C	0.071
ATOM	1246	O	PRO	A	79	5.342	10.633	24.821	1.00	15.71	O	0.066
ATOM	1247	CB	PRO	A	79	6.530	8.113	26.289	1.00	16.79	C	0.069
ATOM	1248	CG	PRO	A	79	5.198	7.471	26.146	1.00	24.10	C	0.082
ATOM	1249	CD	PRO	A	79	4.317	8.073	27.177	1.00	20.53	C	0.076
ATOM	1250	HA	PRO	A	79	6.896	9.784	27.445	1.00	20.47	H	0.076
ATOM	1251	HB2	PRO	A	79	6.993	8.145	25.437	1.00	20.03	H	0.075
ATOM	1252	HB3	PRO	A	79	7.071	7.645	26.944	1.00	20.03	H	0.075
ATOM	1253	HG2	PRO	A	79	4.850	7.645	25.257	1.00	28.79	H	0.090
ATOM	1254	HG3	PRO	A	79	5.284	6.516	26.287	1.00	28.79	H	0.090
ATOM	1255	HD2	PRO	A	79	3.401	8.128	26.863	1.00	24.52	H	0.083
ATOM	1256	HD3	PRO	A	79	4.354	7.568	28.004	1.00	24.52	H	0.083
ATOM	1257	N	CYS	A	80	7.295	11.381	25.618	1.00	15.89	N	0.067
ATOM	1258	CA	CYS	A	80	7.389	12.367	24.547	1.00	8.97	C	0.050
ATOM	1259	C	CYS	A	80	7.322	11.695	23.177	1.00	12.38	C	0.059
ATOM	1260	O	CYS	A	80	6.791	12.261	22.226	1.00	14.04	O	0.063
ATOM	1261	CB	CYS	A	80	8.657	13.180	24.694	1.00	15.14	C	0.065
ATOM	1262	SG	CYS	A	80	8.783	14.111	26.198	1.00	15.13	S	0.065
ATOM	1263	H	CYS	A	80	7.937	11.393	26.190	1.00	18.94	H	0.073
ATOM	1264	HA	CYS	A	80	6.644	12.985	24.614	1.00	10.64	H	0.055
ATOM	1265	HB2	CYS	A	80	9.415	12.576	24.661	1.00	18.04	H	0.071
ATOM	1266	HB3	CYS	A	80	8.704	13.810	23.958	1.00	18.04	H	0.071
ATOM	1267	N	SER	A	81	7.801	10.453	23.047	1.00	17.96	N	0.071
ATOM	1268	CA	SER	A	81	7.718	9.795	21.736	1.00	12.16	C	0.058
ATOM	1269	C	SER	A	81	6.276	9.651	21.210	1.00	16.86	C	0.069
ATOM	1270	O	SER	A	81	6.058	9.610	19.979	1.00	17.27	O	0.070
ATOM	1271	CB	SER	A	81	8.375	8.404	21.824	1.00	22.36	C	0.079
ATOM	1272	OG	SER	A	81	7.682	7.569	22.748	1.00	21.53	O	0.078
ATOM	1273	H	SER	A	81	8.164	9.988	23.673	1.00	21.43	H	0.077
ATOM	1274	HA	SER	A	81	8.196	10.347	21.098	1.00	14.47	H	0.064
ATOM	1275	HB2	SER	A	81	8.355	7.990	20.947	1.00	26.70	H	0.086
ATOM	1276	HB3	SER	A	81	9.293	8.507	22.120	1.00	26.70	H	0.086
ATOM	1277	HG	SER	A	81	8.008	7.658	23.517	1.00	25.71	H	0.085
ATOM	1278	N	ALA	A	82	5.291	9.533	22.108	1.00	19.09	N	0.073
ATOM	1279	CA	ALA	A	82	3.884	9.458	21.711	1.00	18.66	C	0.072
ATOM	1280	C	ALA	A	82	3.456	10.660	20.887	1.00	17.71	C	0.070
ATOM	1281	O	ALA	A	82	2.513	10.579	20.080	1.00	19.03	O	0.073
ATOM	1282	CB	ALA	A	82	3.012	9.368	22.958	1.00	31.28	C	0.094
ATOM	1283	H	ALA	A	82	5.412	9.495	22.959	1.00	22.79	H	0.080
ATOM	1284	HA	ALA	A	82	3.759	8.662	21.171	1.00	22.27	H	0.079
ATOM	1285	HB1	ALA	A	82	2.083	9.289	22.688	1.00	37.41	H	0.102

ATOM	1286	HB2	ALA	A	82	3.274	8.588	23.471	1.00	37.41	H	0.102
ATOM	1287	HB3	ALA	A	82	3.135	10.171	23.488	1.00	37.41	H	0.102
ATOM	1288	N	LEU	A	83	4.099	11.792	21.102	1.00	16.84	N	0.069
ATOM	1289	CA	LEU	A	83	3.782	13.032	20.391	1.00	12.20	C	0.058
ATOM	1290	C	LEU	A	83	4.318	13.071	18.976	1.00	15.99	C	0.067
ATOM	1291	O	LEU	A	83	4.131	14.080	18.284	1.00	19.13	O	0.073
ATOM	1292	CB	LEU	A	83	4.365	14.186	21.184	1.00	10.63	C	0.055
ATOM	1293	CG	LEU	A	83	3.794	14.324	22.590	1.00	19.12	C	0.073
ATOM	1294	CD1	LEU	A	83	4.630	15.293	23.376	1.00	18.86	C	0.073
ATOM	1295	CD2	LEU	A	83	2.337	14.838	22.448	1.00	26.87	C	0.087
ATOM	1296	H	LEU	A	83	4.741	11.878	21.668	1.00	20.09	H	0.075
ATOM	1297	HA	LEU	A	83	2.820	13.131	20.327	1.00	14.51	H	0.064
ATOM	1298	HB2	LEU	A	83	5.323	14.053	21.266	1.00	12.63	H	0.059
ATOM	1299	HB3	LEU	A	83	4.184	15.011	20.709	1.00	12.63	H	0.059
ATOM	1300	HG	LEU	A	83	3.798	13.480	23.068	1.00	22.82	H	0.080
ATOM	1301	HD11	LEU	A	83	4.194	15.472	24.224	1.00	22.52	H	0.079
ATOM	1302	HD12	LEU	A	83	5.505	14.904	23.530	1.00	22.52	H	0.079
ATOM	1303	HD13	LEU	A	83	4.719	16.116	22.871	1.00	22.52	H	0.079
ATOM	1304	HD21	LEU	A	83	1.986	15.040	23.329	1.00	32.12	H	0.095
ATOM	1305	HD22	LEU	A	83	2.336	15.638	21.899	1.00	32.12	H	0.095
ATOM	1306	HD23	LEU	A	83	1.799	14.149	22.027	1.00	32.12	H	0.095
ATOM	1307	N	LEU	A	84	5.047	12.032	18.548	1.00	16.48	N	0.068
ATOM	1308	CA	LEU	A	84	5.635	11.973	17.212	1.00	14.35	C	0.063
ATOM	1309	C	LEU	A	84	4.835	11.113	16.255	1.00	19.82	C	0.074
ATOM	1310	O	LEU	A	84	5.188	11.020	15.072	1.00	18.68	O	0.072
ATOM	1311	CB	LEU	A	84	7.048	11.422	17.292	1.00	19.60	C	0.074
ATOM	1312	CG	LEU	A	84	8.027	12.251	18.109	1.00	17.50	C	0.070
ATOM	1313	CD1	LEU	A	84	9.440	11.690	17.903	1.00	19.40	C	0.074
ATOM	1314	CD2	LEU	A	84	7.993	13.719	17.780	1.00	12.92	C	0.060
ATOM	1315	H	LEU	A	84	5.215	11.337	19.026	1.00	19.65	H	0.074
ATOM	1316	HA	LEU	A	84	5.672	12.875	16.855	1.00	17.10	H	0.069
ATOM	1317	HB2	LEU	A	84	7.009	10.540	17.695	1.00	23.40	H	0.081
ATOM	1318	HB3	LEU	A	84	7.403	11.360	16.391	1.00	23.40	H	0.081
ATOM	1319	HG	LEU	A	84	7.767	12.190	19.042	1.00	20.88	H	0.076
ATOM	1320	HD11	LEU	A	84	10.033	12.077	18.567	1.00	23.15	H	0.080
ATOM	1321	HD12	LEU	A	84	9.414	10.726	18.004	1.00	23.15	H	0.080
ATOM	1322	HD13	LEU	A	84	9.745	11.922	17.012	1.00	23.15	H	0.080
ATOM	1323	HD21	LEU	A	84	8.767	14.149	18.176	1.00	15.38	H	0.066
ATOM	1324	HD22	LEU	A	84	8.012	13.827	16.816	1.00	15.38	H	0.066
ATOM	1325	HD23	LEU	A	84	7.179	14.105	18.139	1.00	15.38	H	0.066
ATOM	1326	N	SER	A	85	3.780	10.481	16.750	1.00	20.67	N	0.076
ATOM	1327	CA	SER	A	85	2.993	9.532	15.977	1.00	22.65	C	0.080
ATOM	1328	C	SER	A	85	2.270	10.184	14.808	1.00	25.85	C	0.085
ATOM	1329	O	SER	A	85	1.916	11.360	14.842	1.00	20.96	O	0.077
ATOM	1330	CB	SER	A	85	1.960	8.883	16.900	1.00	29.54	C	0.091
ATOM	1331	OG	SER	A	85	1.132	8.014	16.145	1.00	31.01	O	0.093
ATOM	1332	H	SER	A	85	3.490	10.587	17.552	1.00	24.69	H	0.083
ATOM	1333	HA	SER	A	85	3.591	8.864	15.604	1.00	27.06	H	0.087
ATOM	1334	HB2	SER	A	85	2.418	8.374	17.587	1.00	35.32	H	0.099
ATOM	1335	HB3	SER	A	85	1.414	9.574	17.307	1.00	35.32	H	0.099
ATOM	1336	HG	SER	A	85	0.668	7.539	16.661	1.00	37.09	H	0.102
ATOM	1337	N	SER	A	86	1.988	9.390	13.770	1.00	18.66	N	0.072
ATOM	1338	CA	SER	A	86	1.153	9.925	12.690	1.00	22.20	C	0.079
ATOM	1339	C	SER	A	86	-0.288	10.130	13.150	1.00	29.36	C	0.091
ATOM	1340	O	SER	A	86	-1.030	10.913	12.543	1.00	25.80	O	0.085
ATOM	1341	CB	SER	A	86	1.215	9.014	11.460	1.00	30.80	C	0.093
ATOM	1342	OG	SER	A	86	0.803	7.689	11.759	1.00	35.31	O	0.099
ATOM	1343	H	SER	A	86	2.255	8.578	13.672	1.00	22.27	H	0.079
ATOM	1344	HA	SER	A	86	1.500	10.786	12.410	1.00	26.52	H	0.086
ATOM	1345	HB2	SER	A	86	0.631	9.374	10.775	1.00	36.84	H	0.102
ATOM	1346	HB3	SER	A	86	2.129	8.989	11.137	1.00	36.84	H	0.102
ATOM	1347	HG	SER	A	86	1.242	7.392	12.411	1.00	42.24	H	0.109
ATOM	1348	N	ASP	A	87	-0.702	9.478	14.222	1.00	21.46	N	0.078
ATOM	1349	CA	ASP	A	87	-2.032	9.707	14.755	1.00	18.99	C	0.073
ATOM	1350	C	ASP	A	87	-1.921	10.811	15.794	1.00	21.75	C	0.078
ATOM	1351	O	ASP	A	87	-1.142	10.694	16.744	1.00	19.12	O	0.073
ATOM	1352	CB	ASP	A	87	-2.612	8.452	15.402	1.00	24.49	C	0.083
ATOM	1353	CG	ASP	A	87	-3.948	8.722	16.064	1.00	34.81	C	0.099
ATOM	1354	OD1	ASP	A	87	-4.951	8.716	15.328	1.00	44.39	O	0.111
ATOM	1355	OD2	ASP	A	87	-4.006	8.971	17.291	1.00	28.89	O	0.090
ATOM	1356	H	ASP	A	87	-0.233	8.902	14.657	1.00	25.63	H	0.085
ATOM	1357	HA	ASP	A	87	-2.635	9.962	14.039	1.00	22.66	H	0.080
ATOM	1358	HB2	ASP	A	87	-2.743	7.773	14.721	1.00	29.26	H	0.090
ATOM	1359	HB3	ASP	A	87	-1.997	8.129	16.079	1.00	29.26	H	0.090
ATOM	1360	N	ILE	A	88	-2.704	11.874	15.629	1.00	20.84	N	0.076
ATOM	1361	CA	ILE	A	88	-2.468	13.072	16.428	1.00	15.23	C	0.065
ATOM	1362	C	ILE	A	88	-3.123	13.033	17.800	1.00	15.59	C	0.066
ATOM	1363	O	ILE	A	88	-3.052	14.011	18.546	1.00	18.86	O	0.073

ATOM	1364	CB	ILE	A	88	-2.913	14.324	15.624	1.00	12.65	C	0.060
ATOM	1365	CG1	ILE	A	88	-4.422	14.327	15.363	1.00	31.72	C	0.094
ATOM	1366	CG2	ILE	A	88	-2.084	14.426	14.310	1.00	21.86	C	0.078
ATOM	1367	CD1	ILE	A	88	-4.946	15.731	14.907	1.00	23.11	C	0.080
ATOM	1368	H	ILE	A	88	-3.360	11.924	15.075	1.00	24.89	H	0.083
ATOM	1369	HA	ILE	A	88	-1.518	13.104	16.622	1.00	18.16	H	0.071
ATOM	1370	HB	ILE	A	88	-2.734	15.113	16.158	1.00	15.06	H	0.065
ATOM	1371	HG12	ILE	A	88	-4.624	13.687	14.663	1.00	37.94	H	0.103
ATOM	1372	HG13	ILE	A	88	-4.885	14.082	16.179	1.00	37.94	H	0.103
ATOM	1373	HG21	ILE	A	88	-2.320	15.248	13.852	1.00	26.11	H	0.085
ATOM	1374	HG22	ILE	A	88	-1.140	14.430	14.532	1.00	26.11	H	0.085
ATOM	1375	HG23	ILE	A	88	-2.289	13.663	13.747	1.00	26.11	H	0.085
ATOM	1376	HD11	ILE	A	88	-5.911	15.693	14.814	1.00	27.61	H	0.088
ATOM	1377	HD12	ILE	A	88	-4.704	16.391	15.575	1.00	27.61	H	0.088
ATOM	1378	HD13	ILE	A	88	-4.541	15.958	14.056	1.00	27.61	H	0.088
ATOM	1379	N	THR	A	89	-3.698	11.893	18.202	1.00	17.55	N	0.070
ATOM	1380	CA	THR	A	89	-4.403	11.849	19.484	1.00	14.85	C	0.064
ATOM	1381	C	THR	A	89	-3.565	12.365	20.655	1.00	19.23	C	0.073
ATOM	1382	O	THR	A	89	-4.020	13.179	21.457	1.00	19.26	O	0.073
ATOM	1383	CB	THR	A	89	-4.852	10.416	19.809	1.00	28.32	C	0.089
ATOM	1384	OG1	THR	A	89	-5.804	9.998	18.843	1.00	26.21	O	0.086
ATOM	1385	CG2	THR	A	89	-5.470	10.379	21.187	1.00	30.12	C	0.092
ATOM	1386	H	THR	A	89	-3.693	11.153	17.763	1.00	20.93	H	0.077
ATOM	1387	HA	THR	A	89	-5.176	12.425	19.381	1.00	17.70	H	0.070
ATOM	1388	HB	THR	A	89	-4.096	9.809	19.795	1.00	33.86	H	0.097
ATOM	1389	HG1	THR	A	89	-5.537	10.196	18.072	1.00	31.33	H	0.094
ATOM	1390	HG21	THR	A	89	-6.033	9.595	21.277	1.00	36.02	H	0.100
ATOM	1391	HG22	THR	A	89	-4.774	10.346	21.862	1.00	36.02	H	0.100
ATOM	1392	HG23	THR	A	89	-6.009	11.172	21.330	1.00	36.02	H	0.100
ATOM	1393	N	ALA	A	90	-2.360	11.837	20.830	1.00	15.34	N	0.066
ATOM	1394	CA	ALA	A	90	-1.656	12.220	22.049	1.00	15.51	C	0.066
ATOM	1395	C	ALA	A	90	-1.321	13.713	22.055	1.00	14.63	C	0.064
ATOM	1396	O	ALA	A	90	-1.401	14.374	23.108	1.00	16.66	O	0.068
ATOM	1397	CB	ALA	A	90	-0.389	11.374	22.209	1.00	24.65	C	0.083
ATOM	1398	H	ALA	A	90	-1.957	11.294	20.298	1.00	18.29	H	0.072
ATOM	1399	HA	ALA	A	90	-2.230	12.045	22.811	1.00	18.49	H	0.072
ATOM	1400	HB1	ALA	A	90	0.091	11.671	22.998	1.00	29.46	H	0.091
ATOM	1401	HB2	ALA	A	90	-0.642	10.443	22.307	1.00	29.46	H	0.091
ATOM	1402	HB3	ALA	A	90	0.167	11.486	21.422	1.00	29.46	H	0.091
ATOM	1403	N	SER	A	91	-0.945	14.254	20.896	1.00	19.91	N	0.075
ATOM	1404	CA	SER	A	91	-0.616	15.671	20.813	1.00	11.10	C	0.056
ATOM	1405	C	SER	A	91	-1.844	16.530	21.068	1.00	10.85	C	0.055
ATOM	1406	O	SER	A	91	-1.772	17.573	21.731	1.00	14.95	O	0.065
ATOM	1407	CB	SER	A	91	-0.060	16.000	19.436	1.00	16.38	C	0.068
ATOM	1408	OG	SER	A	91	1.314	15.708	19.354	1.00	16.06	O	0.067
ATOM	1409	H	SER	A	91	-0.874	13.827	20.154	1.00	23.77	H	0.082
ATOM	1410	HA	SER	A	91	0.059	15.869	21.481	1.00	13.20	H	0.061
ATOM	1411	HB2	SER	A	91	-0.533	15.473	18.773	1.00	19.53	H	0.074
ATOM	1412	HB3	SER	A	91	-0.190	16.946	19.263	1.00	19.53	H	0.074
ATOM	1413	HG	SER	A	91	1.465	14.946	19.674	1.00	19.15	H	0.073
ATOM	1414	N	VAL	A	92	-2.985	16.114	20.542	1.00	14.54	N	0.064
ATOM	1415	CA	VAL	A	92	-4.187	16.923	20.760	1.00	11.79	C	0.057
ATOM	1416	C	VAL	A	92	-4.555	16.925	22.219	1.00	14.35	C	0.063
ATOM	1417	O	VAL	A	92	-4.881	17.968	22.792	1.00	14.19	O	0.063
ATOM	1418	CB	VAL	A	92	-5.348	16.416	19.899	1.00	18.57	C	0.072
ATOM	1419	CG1	VAL	A	92	-6.645	17.088	20.378	1.00	20.80	C	0.076
ATOM	1420	CG2	VAL	A	92	-5.099	16.747	18.497	1.00	13.55	C	0.062
ATOM	1421	H	VAL	A	92	-3.093	15.401	20.075	1.00	17.32	H	0.070
ATOM	1422	HA	VAL	A	92	-3.992	17.834	20.491	1.00	14.03	H	0.063
ATOM	1423	HB	VAL	A	92	-5.435	15.453	19.979	1.00	22.16	H	0.079
ATOM	1424	HG11	VAL	A	92	-7.297	17.063	19.660	1.00	24.84	H	0.083
ATOM	1425	HG12	VAL	A	92	-6.984	16.606	21.149	1.00	24.84	H	0.083
ATOM	1426	HG13	VAL	A	92	-6.454	18.007	20.620	1.00	24.84	H	0.083
ATOM	1427	HG21	VAL	A	92	-5.813	16.378	17.954	1.00	16.14	H	0.067
ATOM	1428	HG22	VAL	A	92	-5.075	17.712	18.400	1.00	16.14	H	0.067
ATOM	1429	HG23	VAL	A	92	-4.249	16.365	18.229	1.00	16.14	H	0.067
ATOM	1430	N	ASN	A	93	-4.567	15.739	22.836	1.00	18.73	N	0.072
ATOM	1431	CA	ASN	A	93	-4.977	15.648	24.224	1.00	19.17	C	0.073
ATOM	1432	C	ASN	A	93	-4.055	16.448	25.122	1.00	15.04	C	0.065
ATOM	1433	O	ASN	A	93	-4.505	17.072	26.080	1.00	16.45	O	0.068
ATOM	1434	CB	ASN	A	93	-4.990	14.183	24.690	1.00	15.23	C	0.065
ATOM	1435	CG	ASN	A	93	-6.118	13.391	24.080	1.00	24.74	C	0.083
ATOM	1436	OD1	ASN	A	93	-7.036	13.943	23.492	1.00	32.12	O	0.095
ATOM	1437	ND2	ASN	A	93	-6.055	12.075	24.242	1.00	34.73	N	0.099
ATOM	1438	H	ASN	A	93	-4.346	14.991	22.474	1.00	22.36	H	0.079
ATOM	1439	HA	ASN	A	93	-5.877	16.004	24.293	1.00	22.88	H	0.080
ATOM	1440	HB2	ASN	A	93	-4.154	13.762	24.435	1.00	18.16	H	0.071
ATOM	1441	HB3	ASN	A	93	-5.092	14.159	25.655	1.00	18.16	H	0.071

ATOM	1442	HD21	ASN	A	93	-6.671	11.572	23.916	1.00	41.55	H	0.108
ATOM	1443	HD22	ASN	A	93	-5.399	11.726	24.674	1.00	41.55	H	0.108
ATOM	1444	N	CYS	A	94	-2.743	16.409	24.853	1.00	16.46	N	0.068
ATOM	1445	CA	CYS	A	94	-1.821	17.224	25.638	1.00	13.87	C	0.062
ATOM	1446	C	CYS	A	94	-2.047	18.716	25.381	1.00	11.34	C	0.056
ATOM	1447	O	CYS	A	94	-2.076	19.509	26.323	1.00	18.18	O	0.071
ATOM	1448	CB	CYS	A	94	-0.374	16.801	25.324	1.00	25.21	C	0.084
ATOM	1449	SG	CYS	A	94	0.873	17.483	26.478	1.00	18.26	S	0.071
ATOM	1450	H	CYS	A	94	-2.376	15.931	24.239	1.00	19.63	H	0.074
ATOM	1451	HA	CYS	A	94	-1.973	17.075	26.584	1.00	16.52	H	0.068
ATOM	1452	HB2	CYS	A	94	-0.318	15.833	25.368	1.00	30.14	H	0.092
ATOM	1453	HB3	CYS	A	94	-0.148	17.106	24.432	1.00	30.14	H	0.092
ATOM	1454	N	ALA	A	95	-2.225	19.106	24.119	1.00	15.17	N	0.065
ATOM	1455	CA	ALA	A	95	-2.483	20.507	23.787	1.00	8.96	C	0.050
ATOM	1456	C	ALA	A	95	-3.733	21.037	24.476	1.00	14.72	C	0.064
ATOM	1457	O	ALA	A	95	-3.791	22.209	24.842	1.00	13.67	O	0.062
ATOM	1458	CB	ALA	A	95	-2.637	20.647	22.281	1.00	13.25	C	0.061
ATOM	1459	H	ALA	A	95	-2.202	18.582	23.437	1.00	18.08	H	0.071
ATOM	1460	HA	ALA	A	95	-1.727	21.036	24.088	1.00	10.63	H	0.055
ATOM	1461	HB1	ALA	A	95	-2.707	21.588	22.057	1.00	15.78	H	0.066
ATOM	1462	HB2	ALA	A	95	-1.860	20.260	21.847	1.00	15.78	H	0.066
ATOM	1463	HB3	ALA	A	95	-3.439	20.179	22.001	1.00	15.78	H	0.066
ATOM	1464	N	LYS	A	96	-4.760	20.207	24.641	1.00	16.14	N	0.067
ATOM	1465	CA	LYS	A	96	-5.928	20.666	25.390	1.00	14.39	C	0.063
ATOM	1466	C	LYS	A	96	-5.583	20.987	26.840	1.00	21.69	C	0.078
ATOM	1467	O	LYS	A	96	-6.115	21.935	27.413	1.00	20.88	O	0.076
ATOM	1468	CB	LYS	A	96	-7.015	19.582	25.314	1.00	13.52	C	0.062
ATOM	1469	CG	LYS	A	96	-7.618	19.497	23.940	1.00	14.28	C	0.063
ATOM	1470	CD	LYS	A	96	-8.556	18.272	23.813	1.00	17.52	C	0.070
ATOM	1471	CE	LYS	A	96	-9.347	18.338	22.522	1.00	22.76	C	0.080
ATOM	1472	NZ	LYS	A	96	-10.284	17.145	22.482	1.00	24.81	N	0.083
ATOM	1473	H	LYS	A	96	-4.806	19.402	24.341	1.00	19.24	H	0.073
ATOM	1474	HA	LYS	A	96	-6.266	21.484	24.993	1.00	17.15	H	0.069
ATOM	1475	HB2	LYS	A	96	-6.623	18.721	25.529	1.00	16.11	H	0.067
ATOM	1476	HB3	LYS	A	96	-7.721	19.791	25.945	1.00	16.11	H	0.067
ATOM	1477	HG2	LYS	A	96	-8.136	20.298	23.765	1.00	17.02	H	0.069
ATOM	1478	HG3	LYS	A	96	-6.911	19.409	23.282	1.00	17.02	H	0.069
ATOM	1479	HD2	LYS	A	96	-8.029	17.458	23.811	1.00	20.91	H	0.077
ATOM	1480	HD3	LYS	A	96	-9.179	18.262	24.557	1.00	20.91	H	0.077
ATOM	1481	HE2	LYS	A	96	-9.869	19.155	22.491	1.00	27.19	H	0.087
ATOM	1482	HE3	LYS	A	96	-8.747	18.299	21.760	1.00	27.19	H	0.087
ATOM	1483	HZ1	LYS	A	96	-10.848	17.177	23.169	1.00	29.64	H	0.091
ATOM	1484	HZ2	LYS	A	96	-10.751	17.150	21.724	1.00	29.64	H	0.091
ATOM	1485	HZ3	LYS	A	96	-9.816	16.389	22.527	1.00	29.64	H	0.091
ATOM	1486	N	LYS	A	97	-4.719	20.195	27.467	1.00	17.74	N	0.070
ATOM	1487	CA	LYS	A	97	-4.309	20.536	28.823	1.00	13.72	C	0.062
ATOM	1488	C	LYS	A	97	-3.482	21.818	28.860	1.00	17.98	C	0.071
ATOM	1489	O	LYS	A	97	-3.674	22.668	29.743	1.00	18.18	O	0.071
ATOM	1490	CB	LYS	A	97	-3.511	19.388	29.430	1.00	12.60	C	0.059
ATOM	1491	CG	LYS	A	97	-3.488	19.437	30.930	1.00	30.80	C	0.093
ATOM	1492	CD	LYS	A	97	-2.650	18.280	31.498	1.00	36.22	C	0.101
ATOM	1493	CE	LYS	A	97	-3.490	17.027	31.699	1.00	45.55	C	0.113
ATOM	1494	NZ	LYS	A	97	-4.504	17.170	32.796	1.00	57.92	N	0.127
ATOM	1495	H	LYS	A	97	-4.369	19.480	27.142	1.00	21.16	H	0.077
ATOM	1496	HA	LYS	A	97	-5.109	20.678	29.352	1.00	16.34	H	0.068
ATOM	1497	HB2	LYS	A	97	-3.911	18.547	29.161	1.00	15.00	H	0.065
ATOM	1498	HB3	LYS	A	97	-2.596	19.436	29.113	1.00	15.00	H	0.065
ATOM	1499	HG2	LYS	A	97	-3.096	20.274	31.222	1.00	36.84	H	0.102
ATOM	1500	HG3	LYS	A	97	-4.393	19.358	31.271	1.00	36.84	H	0.102
ATOM	1501	HD2	LYS	A	97	-1.932	18.069	30.880	1.00	43.35	H	0.110
ATOM	1502	HD3	LYS	A	97	-2.282	18.541	32.357	1.00	43.35	H	0.110
ATOM	1503	HE2	LYS	A	97	-3.965	16.830	30.877	1.00	54.54	H	0.124
ATOM	1504	HE3	LYS	A	97	-2.905	16.288	31.929	1.00	54.54	H	0.124
ATOM	1505	HZ1	LYS	A	97	-4.942	16.403	32.908	1.00	69.38	H	0.139
ATOM	1506	HZ2	LYS	A	97	-4.099	17.386	33.558	1.00	69.38	H	0.139
ATOM	1507	HZ3	LYS	A	97	-5.088	17.809	32.588	1.00	69.38	H	0.139
ATOM	1508	N	ILE	A	98	-2.520	21.946	27.937	1.00	15.86	N	0.067
ATOM	1509	CA	ILE	A	98	-1.651	23.119	27.896	1.00	15.20	C	0.065
ATOM	1510	C	ILE	A	98	-2.474	24.381	27.738	1.00	12.51	C	0.059
ATOM	1511	O	ILE	A	98	-2.220	25.388	28.407	1.00	15.11	O	0.065
ATOM	1512	CB	ILE	A	98	-0.627	23.008	26.756	1.00	12.01	C	0.058
ATOM	1513	CG1	ILE	A	98	0.368	21.892	27.016	1.00	13.55	C	0.062
ATOM	1514	CG2	ILE	A	98	0.173	24.316	26.548	1.00	14.49	C	0.064
ATOM	1515	CD1	ILE	A	98	1.073	21.381	25.778	1.00	18.57	C	0.072
ATOM	1516	H	ILE	A	98	-2.352	21.365	27.325	1.00	18.91	H	0.073
ATOM	1517	HA	ILE	A	98	-1.172	23.158	28.738	1.00	18.12	H	0.071
ATOM	1518	HB	ILE	A	98	-1.148	22.820	25.959	1.00	14.28	H	0.063
ATOM	1519	HG12	ILE	A	98	1.047	22.219	27.627	1.00	16.13	H	0.067

```
ATOM   1520 HG13 ILE A  98      -0.104  21.143  27.415  1.00 16.13           H  0.067
ATOM   1521 HG21 ILE A  98       0.872  24.158  25.894  1.00 17.26           H  0.070
ATOM   1522 HG22 ILE A  98      -0.429  25.007  26.229  1.00 17.26           H  0.070
ATOM   1523 HG23 ILE A  98       0.565  24.584  27.393  1.00 17.26           H  0.070
ATOM   1524 HD11 ILE A  98       1.748  20.736  26.043  1.00 22.16           H  0.079
ATOM   1525 HD12 ILE A  98       0.423  20.959  25.195  1.00 22.16           H  0.079
ATOM   1526 HD13 ILE A  98       1.491  22.128  25.322  1.00 22.16           H  0.079
ATOM   1527  N   VAL A  99      -3.425  24.375  26.795  1.00 12.21           N  0.058
ATOM   1528  CA  VAL A  99      -4.131  25.622  26.482  1.00 12.43           C  0.059
ATOM   1529  C   VAL A  99      -5.132  25.970  27.556  1.00 24.10           C  0.082
ATOM   1530  O   VAL A  99      -5.700  27.075  27.538  1.00 18.75           O  0.072
ATOM   1531  CB  VAL A  99      -4.829  25.478  25.129  1.00  9.35           C  0.051
ATOM   1532  CG1 VAL A  99      -6.048  24.538  25.249  1.00 14.25           C  0.063
ATOM   1533  CG2 VAL A  99      -5.306  26.791  24.556  1.00 12.74           C  0.060
ATOM   1534  H   VAL A  99      -3.670  23.687  26.341  1.00 14.53           H  0.064
ATOM   1535  HA  VAL A  99      -3.499  26.355  26.419  1.00 14.79           H  0.064
ATOM   1536  HB  VAL A  99      -4.165  25.112  24.524  1.00 11.10           H  0.056
ATOM   1537 HG11 VAL A  99      -6.400  24.362  24.362  1.00 16.98           H  0.069
ATOM   1538 HG12 VAL A  99      -5.767  23.707  25.663  1.00 16.98           H  0.069
ATOM   1539 HG13 VAL A  99      -6.726  24.967  25.794  1.00 16.98           H  0.069
ATOM   1540 HG21 VAL A  99      -5.701  26.630  23.684  1.00 15.17           H  0.065
ATOM   1541 HG22 VAL A  99      -5.968  27.175  25.153  1.00 15.17           H  0.065
ATOM   1542 HG23 VAL A  99      -4.550  27.392  24.470  1.00 15.17           H  0.065
ATOM   1543  N   SER A 100      -5.390  25.037  28.459  1.00 16.69           N  0.068
ATOM   1544  CA  SER A 100      -6.309  25.274  29.553  1.00 18.57           C  0.072
ATOM   1545  C   SER A 100      -5.612  25.774  30.796  1.00 25.15           C  0.084
ATOM   1546  O   SER A 100      -6.287  26.013  31.798  1.00 19.91           O  0.075
ATOM   1547  CB  SER A 100      -7.070  23.988  29.890  1.00 18.70           C  0.072
ATOM   1548  OG  SER A 100      -7.755  23.481  28.777  1.00 19.53           O  0.074
ATOM   1549  H   SER A 100      -5.040  24.252  28.458  1.00 19.91           H  0.075
ATOM   1550  HA  SER A 100      -6.955  25.942  29.274  1.00 22.16           H  0.079
ATOM   1551  HB2 SER A 100      -6.435  23.321  30.196  1.00 22.32           H  0.079
ATOM   1552  HB3 SER A 100      -7.713  24.181  30.591  1.00 22.32           H  0.079
ATOM   1553  HG  SER A 100      -7.273  23.540  28.092  1.00 23.31           H  0.081
ATOM   1554  N   ASP A 101      -4.285  25.884  30.778  1.00 23.21           N  0.081
ATOM   1555  CA  ASP A 101      -3.526  26.140  32.032  1.00 25.32           C  0.084
ATOM   1556  C   ASP A 101      -3.392  27.612  32.423  1.00 32.08           C  0.095
ATOM   1557  O   ASP A 101      -2.729  27.843  33.445  1.00 25.65           O  0.085
ATOM   1558  CB  ASP A 101      -2.167  25.442  31.985  1.00 25.09           C  0.084
ATOM   1559  CG  ASP A 101      -1.787  24.871  33.344  1.00 43.13           C  0.110
ATOM   1560  OD1 ASP A 101      -2.548  24.043  33.866  1.00 53.85           O  0.123
ATOM   1561  OD2 ASP A 101      -0.774  25.292  33.889  1.00 36.62           O  0.101
ATOM   1562  H   ASP A 101      -3.707  25.798  29.922  1.00 27.73           H  0.088
ATOM   1563  HA  ASP A 101      -4.083  25.656  32.831  1.00 30.26           H  0.092
ATOM   1564  HB2 ASP A 101      -2.201  24.632  31.257  1.00 29.99           H  0.092
ATOM   1565  HB3 ASP A 101      -1.403  26.154  31.676  1.00 29.99           H  0.092
ATOM   1566  N   GLY A 102      -3.946  28.560  31.666  1.00 23.80           N  0.082
ATOM   1567  CA  GLY A 102      -4.011  29.937  32.147  1.00 20.53           C  0.076
ATOM   1568  C   GLY A 102      -3.720  30.997  31.100  1.00 21.02           C  0.077
ATOM   1569  O   GLY A 102      -4.347  32.067  31.081  1.00 18.14           O  0.071
ATOM   1570  H   GLY A 102      -4.278  28.436  30.883  1.00 28.43           H  0.089
ATOM   1571  HA2 GLY A 102      -4.901  30.103  32.495  1.00 24.51           H  0.083
ATOM   1572  HA3 GLY A 102      -3.367  30.046  32.864  1.00 24.51           H  0.083
ATOM   1573  N   ASN A 103      -2.788  30.694  30.206  1.00 19.16           N  0.073
ATOM   1574  CA  ASN A 103      -2.335  31.654  29.196  1.00 20.12           C  0.075
ATOM   1575  C   ASN A 103      -2.863  31.323  27.803  1.00 15.85           C  0.067
ATOM   1576  O   ASN A 103      -2.408  31.939  26.826  1.00 12.88           O  0.060
ATOM   1577  CB  ASN A 103      -0.805  31.699  29.194  1.00 24.41           C  0.083
ATOM   1578  CG  ASN A 103      -0.247  32.302  30.468  1.00 29.62           C  0.091
ATOM   1579  OD1 ASN A 103      -0.850  33.199  31.056  1.00 22.14           O  0.079
ATOM   1580  ND2 ASN A 103       0.871  31.766  30.940  1.00 32.97           N  0.096
ATOM   1581  H   ASN A 103      -2.395  29.931  30.160  1.00 22.87           H  0.080
ATOM   1582  HA  ASN A 103      -2.664  32.538  29.423  1.00 24.03           H  0.082
ATOM   1583  HB2 ASN A 103      -0.461  30.797  29.110  1.00 29.17           H  0.090
ATOM   1584  HB3 ASN A 103      -0.504  32.240  28.447  1.00 29.17           H  0.090
ATOM   1585 HD21 ASN A 103       1.224  32.075  31.661  1.00 39.44           H  0.105
ATOM   1586 HD22 ASN A 103       1.242  31.111  30.525  1.00 39.44           H  0.105
ATOM   1587  N   GLY A 104      -3.817  30.402  27.694  1.00 16.35           N  0.068
ATOM   1588  CA  GLY A 104      -4.360  30.091  26.375  1.00 13.64           C  0.062
ATOM   1589  C   GLY A 104      -3.272  29.681  25.403  1.00 14.39           C  0.063
ATOM   1590  O   GLY A 104      -2.317  28.987  25.767  1.00 13.21           O  0.061
ATOM   1591  H   GLY A 104      -4.156  29.957  28.347  1.00 19.50           H  0.074
ATOM   1592  HA2 GLY A 104      -4.996  29.364  26.450  1.00 16.24           H  0.067
ATOM   1593  HA3 GLY A 104      -4.813  30.872  26.019  1.00 16.24           H  0.067
ATOM   1594  N   MET A 105      -3.464  30.048  24.120  1.00 11.17           N  0.056
ATOM   1595  CA  MET A 105      -2.477  29.606  23.149  1.00 11.13           C  0.056
ATOM   1596  C   MET A 105      -1.209  30.411  23.177  1.00 12.69           C  0.060
ATOM   1597  O   MET A 105      -0.306  30.072  22.418  1.00 12.33           O  0.059
```

ATOM	1598	CB	MET	A	105	-3.018	29.612	21.715	1.00	11.17	C	0.056
ATOM	1599	CG	MET	A	105	-4.220	28.690	21.504	1.00	11.53	C	0.057
ATOM	1600	SD	MET	A	105	-4.538	28.335	19.747	1.00	12.96	S	0.060
ATOM	1601	CE	MET	A	105	-2.994	27.495	19.201	1.00	11.56	C	0.057
ATOM	1602	H	MET	A	105	-4.117	30.521	23.820	1.00	13.28	H	0.061
ATOM	1603	HA	MET	A	105	-2.286	28.682	23.373	1.00	13.23	H	0.061
ATOM	1604	HB2	MET	A	105	-3.293	30.514	21.490	1.00	13.29	H	0.061
ATOM	1605	HB3	MET	A	105	-2.313	29.320	21.116	1.00	13.29	H	0.061
ATOM	1606	HG2	MET	A	105	-4.056	27.848	21.955	1.00	13.72	H	0.062
ATOM	1607	HG3	MET	A	105	-5.012	29.115	21.871	1.00	13.72	H	0.062
ATOM	1608	HE1	MET	A	105	-3.088	27.234	18.271	1.00	13.75	H	0.062
ATOM	1609	HE2	MET	A	105	-2.249	28.108	19.298	1.00	13.75	H	0.062
ATOM	1610	HE3	MET	A	105	-2.850	26.709	19.752	1.00	13.75	H	0.062
ATOM	1611	N	ASN	A	106	-1.071	31.385	24.078	1.00	11.62	N	0.057
ATOM	1612	CA	ASN	A	106	0.228	32.045	24.188	1.00	13.39	C	0.061
ATOM	1613	C	ASN	A	106	1.309	31.087	24.660	1.00	16.30	C	0.068
ATOM	1614	O	ASN	A	106	2.500	31.414	24.589	1.00	15.28	O	0.065
ATOM	1615	CB	ASN	A	106	0.162	33.228	25.148	1.00	15.66	C	0.066
ATOM	1616	CG	ASN	A	106	-0.766	34.298	24.661	1.00	13.81	C	0.062
ATOM	1617	OD1	ASN	A	106	-0.457	34.990	23.685	1.00	13.35	O	0.061
ATOM	1618	ND2	ASN	A	106	-1.943	34.444	25.319	1.00	19.36	N	0.074
ATOM	1619	H	ASN	A	106	-1.683	31.669	24.611	1.00	13.82	H	0.062
ATOM	1620	HA	ASN	A	106	0.465	32.391	23.313	1.00	15.95	H	0.067
ATOM	1621	HB2	ASN	A	106	-0.156	32.921	26.011	1.00	18.67	H	0.072
ATOM	1622	HB3	ASN	A	106	1.048	33.614	25.239	1.00	18.67	H	0.072
ATOM	1623	HD21	ASN	A	106	-2.503	35.047	25.070	1.00	23.11	H	0.080
ATOM	1624	HD22	ASN	A	106	-2.129	33.933	25.986	1.00	23.11	H	0.080
ATOM	1625	N	ALA	A	107	0.923	29.930	25.144	1.00	9.47	N	0.051
ATOM	1626	CA	ALA	A	107	1.868	28.882	25.514	1.00	9.82	C	0.052
ATOM	1627	C	ALA	A	107	2.733	28.465	24.338	1.00	17.61	C	0.070
ATOM	1628	O	ALA	A	107	3.784	27.853	24.550	1.00	19.16	O	0.073
ATOM	1629	CB	ALA	A	107	1.111	27.665	26.066	1.00	15.30	C	0.065
ATOM	1630	H	ALA	A	107	0.102	29.711	25.275	1.00	11.24	H	0.056
ATOM	1631	HA	ALA	A	107	2.451	29.214	26.215	1.00	11.66	H	0.057
ATOM	1632	HB1	ALA	A	107	1.730	26.925	26.161	1.00	18.24	H	0.071
ATOM	1633	HB2	ALA	A	107	0.733	27.893	26.929	1.00	18.24	H	0.071
ATOM	1634	HB3	ALA	A	107	0.403	27.427	25.447	1.00	18.24	H	0.071
ATOM	1635	N	TRP	A	108	2.273	28.704	23.116	1.00	11.71	N	0.057
ATOM	1636	CA	TRP	A	108	3.009	28.390	21.902	1.00	10.39	C	0.054
ATOM	1637	C	TRP	A	108	3.560	29.710	21.396	1.00	14.49	C	0.064
ATOM	1638	O	TRP	A	108	2.817	30.551	20.880	1.00	14.81	O	0.064
ATOM	1639	CB	TRP	A	108	2.118	27.710	20.858	1.00	11.60	C	0.057
ATOM	1640	CG	TRP	A	108	1.828	26.281	21.198	1.00	14.21	C	0.063
ATOM	1641	CD1	TRP	A	108	2.632	25.214	20.959	1.00	12.14	C	0.058
ATOM	1642	CD2	TRP	A	108	0.667	25.772	21.863	1.00	10.29	C	0.054
ATOM	1643	NE1	TRP	A	108	2.083	24.094	21.481	1.00	15.81	N	0.067
ATOM	1644	CE2	TRP	A	108	0.857	24.397	22.020	1.00	12.95	C	0.060
ATOM	1645	CE3	TRP	A	108	-0.479	26.350	22.372	1.00	13.58	C	0.062
ATOM	1646	CZ2	TRP	A	108	-0.090	23.580	22.626	1.00	12.60	C	0.059
ATOM	1647	CZ3	TRP	A	108	-1.420	25.537	22.977	1.00	17.60	C	0.070
ATOM	1648	CH2	TRP	A	108	-1.200	24.176	23.131	1.00	12.08	C	0.058
ATOM	1649	H	TRP	A	108	1.506	29.061	22.960	1.00	13.93	H	0.062
ATOM	1650	HA	TRP	A	108	3.734	27.774	22.090	1.00	12.34	H	0.059
ATOM	1651	HB2	TRP	A	108	1.274	28.185	20.804	1.00	13.80	H	0.062
ATOM	1652	HB3	TRP	A	108	2.566	27.731	19.998	1.00	13.80	H	0.062
ATOM	1653	HD1	TRP	A	108	3.442	25.248	20.504	1.00	14.44	H	0.064
ATOM	1654	HE1	TRP	A	108	2.444	23.314	21.476	1.00	18.84	H	0.073
ATOM	1655	HE3	TRP	A	108	-0.615	27.267	22.309	1.00	16.18	H	0.067
ATOM	1656	HZ2	TRP	A	108	0.032	22.659	22.683	1.00	15.00	H	0.065
ATOM	1657	HZ3	TRP	A	108	-2.214	25.909	23.286	1.00	21.00	H	0.077
ATOM	1658	HH2	TRP	A	108	-1.825	23.662	23.589	1.00	14.37	H	0.063
ATOM	1659	N	VAL	A	109	4.862	29.906	21.554	1.00	18.60	N	0.072
ATOM	1660	CA	VAL	A	109	5.421	31.218	21.232	1.00	19.26	C	0.073
ATOM	1661	C	VAL	A	109	5.186	31.548	19.774	1.00	12.70	C	0.060
ATOM	1662	O	VAL	A	109	4.906	32.706	19.413	1.00	17.22	O	0.069
ATOM	1663	CB	VAL	A	109	6.922	31.233	21.590	1.00	20.80	C	0.076
ATOM	1664	CG1	VAL	A	109	7.585	32.450	21.003	1.00	31.68	C	0.094
ATOM	1665	CG2	VAL	A	109	7.071	31.214	23.093	1.00	21.53	C	0.078
ATOM	1666	H	VAL	A	109	5.426	29.321	21.835	1.00	22.20	H	0.079
ATOM	1667	HA	VAL	A	109	4.973	31.898	21.760	1.00	22.99	H	0.080
ATOM	1668	HB	VAL	A	109	7.359	30.450	21.220	1.00	24.84	H	0.083
ATOM	1669	HG11	VAL	A	109	8.455	32.566	21.418	1.00	37.90	H	0.103
ATOM	1670	HG12	VAL	A	109	7.689	32.323	20.047	1.00	37.90	H	0.103
ATOM	1671	HG13	VAL	A	109	7.030	33.226	21.176	1.00	37.90	H	0.103
ATOM	1672	HG21	VAL	A	109	8.015	31.221	23.317	1.00	25.72	H	0.085
ATOM	1673	HG22	VAL	A	109	6.638	31.999	23.463	1.00	25.72	H	0.085
ATOM	1674	HG23	VAL	A	109	6.654	30.411	23.442	1.00	25.72	H	0.085
ATOM	1675	N	ALA	A	110	5.263	30.538	18.916	1.00	11.73	N	0.057

ATOM	1676	CA	ALA	A	110	5.070	30.769	17.494	1.00	10.36	C	0.054
ATOM	1677	C	ALA	A	110	3.639	31.157	17.178	1.00	13.38	C	0.061
ATOM	1678	O	ALA	A	110	3.406	31.923	16.247	1.00	13.57	O	0.062
ATOM	1679	CB	ALA	A	110	5.428	29.547	16.685	1.00	18.94	C	0.073
ATOM	1680	H	ALA	A	110	5.423	29.720	19.130	1.00	13.95	H	0.062
ATOM	1681	HA	ALA	A	110	5.666	31.490	17.237	1.00	12.30	H	0.059
ATOM	1682	HB1	ALA	A	110	5.204	29.707	15.755	1.00	22.61	H	0.080
ATOM	1683	HB2	ALA	A	110	6.379	29.377	16.772	1.00	22.61	H	0.080
ATOM	1684	HB3	ALA	A	110	4.924	28.788	17.019	1.00	22.61	H	0.080
ATOM	1685	N	TRP	A	111	2.662	30.616	17.897	1.00	13.82	N	0.062
ATOM	1686	CA	TRP	A	111	1.312	31.142	17.712	1.00	11.16	C	0.056
ATOM	1687	C	TRP	A	111	1.256	32.612	18.097	1.00	17.64	C	0.070
ATOM	1688	O	TRP	A	111	0.658	33.423	17.400	1.00	13.76	O	0.062
ATOM	1689	CB	TRP	A	111	0.302	30.340	18.523	1.00	13.78	C	0.062
ATOM	1690	CG	TRP	A	111	-1.057	30.873	18.410	1.00	11.88	C	0.058
ATOM	1691	CD1	TRP	A	111	-1.974	30.567	17.437	1.00	8.76	C	0.050
ATOM	1692	CD2	TRP	A	111	-1.670	31.831	19.276	1.00	14.76	C	0.064
ATOM	1693	NE1	TRP	A	111	-3.150	31.277	17.666	1.00	10.63	N	0.055
ATOM	1694	CE2	TRP	A	111	-2.975	32.047	18.795	1.00	14.70	C	0.064
ATOM	1695	CE3	TRP	A	111	-1.257	32.498	20.442	1.00	15.18	C	0.065
ATOM	1696	CZ2	TRP	A	111	-3.858	32.910	19.415	1.00	10.53	C	0.054
ATOM	1697	CZ3	TRP	A	111	-2.131	33.346	21.056	1.00	13.24	C	0.061
ATOM	1698	CH2	TRP	A	111	-3.424	33.548	20.557	1.00	14.95	C	0.065
ATOM	1699	H	TRP	A	111	2.747	29.979	18.468	1.00	16.46	H	0.068
ATOM	1700	HA	TRP	A	111	1.071	31.052	16.777	1.00	13.27	H	0.061
ATOM	1701	HB2	TRP	A	111	0.294	29.425	18.203	1.00	16.41	H	0.068
ATOM	1702	HB3	TRP	A	111	0.557	30.363	19.458	1.00	16.41	H	0.068
ATOM	1703	HD1	TRP	A	111	-1.832	29.977	16.733	1.00	10.39	H	0.054
ATOM	1704	HE1	TRP	A	111	-3.863	31.241	17.186	1.00	12.64	H	0.059
ATOM	1705	HE3	TRP	A	111	-0.405	32.364	20.789	1.00	18.10	H	0.071
ATOM	1706	HZ2	TRP	A	111	-4.711	33.055	19.075	1.00	12.52	H	0.059
ATOM	1707	HZ3	TRP	A	111	-1.863	33.799	21.822	1.00	15.77	H	0.066
ATOM	1708	HH2	TRP	A	111	-4.002	34.123	21.005	1.00	17.82	H	0.071
ATOM	1709	N	ARG	A	112	1.844	32.983	19.231	1.00	11.93	N	0.058
ATOM	1710	CA	ARG	A	112	1.710	34.372	19.634	1.00	16.02	C	0.067
ATOM	1711	C	ARG	A	112	2.343	35.284	18.599	1.00	16.50	C	0.068
ATOM	1712	O	ARG	A	112	1.815	36.361	18.288	1.00	16.14	O	0.067
ATOM	1713	CB	ARG	A	112	2.333	34.626	21.006	1.00	14.84	C	0.064
ATOM	1714	CG	ARG	A	112	2.249	36.102	21.406	1.00	21.56	C	0.078
ATOM	1715	CD	ARG	A	112	2.672	36.331	22.866	1.00	32.67	C	0.096
ATOM	1716	NE	ARG	A	112	3.951	35.695	23.151	1.00	33.93	N	0.097
ATOM	1717	CZ	ARG	A	112	5.149	36.217	22.892	1.00	40.90	C	0.107
ATOM	1718	NH1	ARG	A	112	5.285	37.408	22.320	1.00	29.37	N	0.091
ATOM	1719	NH2	ARG	A	112	6.242	35.524	23.215	1.00	27.86	N	0.088
ATOM	1720	H	ARG	A	112	2.299	32.475	19.755	1.00	14.19	H	0.063
ATOM	1721	HA	ARG	A	112	0.765	34.573	19.711	1.00	19.10	H	0.073
ATOM	1722	HB2	ARG	A	112	1.861	34.103	21.672	1.00	17.69	H	0.070
ATOM	1723	HB3	ARG	A	112	3.268	34.370	20.984	1.00	17.69	H	0.070
ATOM	1724	HG2	ARG	A	112	2.838	36.621	20.836	1.00	25.75	H	0.085
ATOM	1725	HG3	ARG	A	112	1.335	36.408	21.305	1.00	25.75	H	0.085
ATOM	1726	HD2	ARG	A	112	2.760	37.282	23.030	1.00	39.09	H	0.105
ATOM	1727	HD3	ARG	A	112	2.003	35.952	23.458	1.00	39.09	H	0.105
ATOM	1728	HE	ARG	A	112	3.931	34.917	23.517	1.00	40.60	H	0.107
ATOM	1729	HH11	ARG	A	112	4.589	37.865	22.106	1.00	35.13	H	0.099
ATOM	1730	HH12	ARG	A	112	6.070	37.723	22.163	1.00	35.13	H	0.099
ATOM	1731	HH21	ARG	A	112	6.167	34.751	23.584	1.00	33.31	H	0.097
ATOM	1732	HH22	ARG	A	112	7.021	35.849	23.053	1.00	33.31	H	0.097
ATOM	1733	N	ASN	A	113	3.477	34.865	18.052	1.00	15.55	N	0.066
ATOM	1734	CA	ASN	A	113	4.235	35.771	17.219	1.00	11.90	C	0.058
ATOM	1735	C	ASN	A	113	3.857	35.693	15.760	1.00	15.82	C	0.067
ATOM	1736	O	ASN	A	113	4.140	36.646	15.028	1.00	13.58	O	0.062
ATOM	1737	CB	ASN	A	113	5.715	35.486	17.351	1.00	10.60	C	0.054
ATOM	1738	CG	ASN	A	113	6.269	35.887	18.685	1.00	15.15	C	0.065
ATOM	1739	OD1	ASN	A	113	5.841	36.880	19.284	1.00	19.43	O	0.074
ATOM	1740	ND2	ASN	A	113	7.267	35.139	19.138	1.00	19.07	N	0.073
ATOM	1741	H	ASN	A	113	3.816	34.081	18.148	1.00	18.54	H	0.072
ATOM	1742	HA	ASN	A	113	4.070	36.677	17.525	1.00	14.16	H	0.063
ATOM	1743	HB2	ASN	A	113	5.864	34.534	17.239	1.00	12.60	H	0.059
ATOM	1744	HB3	ASN	A	113	6.194	35.980	16.667	1.00	12.60	H	0.059
ATOM	1745	HD21	ASN	A	113	7.628	35.318	19.898	1.00	22.76	H	0.080
ATOM	1746	HD22	ASN	A	113	7.552	34.475	18.673	1.00	22.76	H	0.080
ATOM	1747	N	ARG	A	114	3.189	34.626	15.316	1.00	15.68	N	0.066
ATOM	1748	CA	ARG	A	114	2.922	34.485	13.891	1.00	12.84	C	0.060
ATOM	1749	C	ARG	A	114	1.462	34.223	13.528	1.00	14.66	C	0.064
ATOM	1750	O	ARG	A	114	1.111	34.327	12.345	1.00	13.47	O	0.061
ATOM	1751	CB	ARG	A	114	3.798	33.381	13.310	1.00	15.49	C	0.066
ATOM	1752	CG	ARG	A	114	5.314	33.613	13.633	1.00	13.25	C	0.061
ATOM	1753	CD	ARG	A	114	6.182	32.527	13.111	1.00	16.37	C	0.068

```
ATOM   1754  NE  ARG A 114       6.309  32.555  11.663  1.00 17.36           N  0.070
ATOM   1755  CZ  ARG A 114       7.109  31.729  11.017  1.00 14.34           C  0.063
ATOM   1756  NH1 ARG A 114       7.812  30.814  11.671  1.00 14.54           N  0.064
ATOM   1757  NH2 ARG A 114       7.198  31.814   9.696  1.00 14.49           N  0.064
ATOM   1758  H   ARG A 114       2.890  33.989  15.810  1.00 18.70           H  0.072
ATOM   1759  HA  ARG A 114       3.159  35.317  13.452  1.00 15.29           H  0.065
ATOM   1760  HB2 ARG A 114       3.532  32.529  13.691  1.00 18.47           H  0.072
ATOM   1761  HB3 ARG A 114       3.690  33.361  12.346  1.00 18.47           H  0.072
ATOM   1762  HG2 ARG A 114       5.600  34.447  13.228  1.00 15.78           H  0.066
ATOM   1763  HG3 ARG A 114       5.431  33.655  14.595  1.00 15.78           H  0.066
ATOM   1764  HD2 ARG A 114       7.070  32.622  13.492  1.00 19.52           H  0.074
ATOM   1765  HD3 ARG A 114       5.803  31.671  13.363  1.00 19.52           H  0.074
ATOM   1766  HE  ARG A 114       5.848  33.126  11.215  1.00 20.71           H  0.076
ATOM   1767 HH11 ARG A 114       7.749  30.754  12.527  1.00 17.32           H  0.070
ATOM   1768 HH12 ARG A 114       8.330  30.281  11.240  1.00 17.32           H  0.070
ATOM   1769 HH21 ARG A 114       6.737  32.403   9.271  1.00 17.27           H  0.070
ATOM   1770 HH22 ARG A 114       7.716  31.281   9.265  1.00 17.27           H  0.070
ATOM   1771  N   CYS A 115       0.604  33.931  14.507  1.00 11.14           N  0.056
ATOM   1772  CA  CYS A 115      -0.802  33.621  14.245  1.00 12.53           C  0.059
ATOM   1773  C   CYS A 115      -1.747  34.581  14.941  1.00 11.33           C  0.056
ATOM   1774  O   CYS A 115      -2.715  35.043  14.344  1.00 14.01           O  0.063
ATOM   1775  CB  CYS A 115      -1.106  32.200  14.740  1.00 15.87           C  0.067
ATOM   1776  SG  CYS A 115      -0.124  30.894  13.995  1.00 14.68           S  0.064
ATOM   1777  H   CYS A 115       0.815  33.906  15.340  1.00 13.24           H  0.061
ATOM   1778  HA  CYS A 115      -0.958  33.673  13.289  1.00 14.91           H  0.065
ATOM   1779  HB2 CYS A 115      -0.948  32.170  15.697  1.00 18.93           H  0.073
ATOM   1780  HB3 CYS A 115      -2.037  32.004  14.552  1.00 18.93           H  0.073
ATOM   1781  N   LYS A 116      -1.498  34.870  16.209  1.00 13.01           N  0.060
ATOM   1782  CA  LYS A 116      -2.341  35.777  16.980  1.00 16.18           C  0.067
ATOM   1783  C   LYS A 116      -2.625  37.088  16.253  1.00 15.90           C  0.067
ATOM   1784  O   LYS A 116      -1.723  37.798  15.817  1.00 15.00           O  0.065
ATOM   1785  CB  LYS A 116      -1.647  36.052  18.317  1.00 15.63           C  0.066
ATOM   1786  CG  LYS A 116      -2.422  36.864  19.321  1.00 12.99           C  0.060
ATOM   1787  CD  LYS A 116      -1.612  36.974  20.595  1.00 16.19           C  0.067
ATOM   1788  CE  LYS A 116      -2.415  37.633  21.751  1.00 17.00           C  0.069
ATOM   1789  NZ  LYS A 116      -1.598  37.776  23.012  1.00 12.73           N  0.060
ATOM   1790  H   LYS A 116      -0.836  34.551  16.656  1.00 15.49           H  0.066
ATOM   1791  HA  LYS A 116      -3.204  35.358  17.127  1.00 19.29           H  0.073
ATOM   1792  HB2 LYS A 116      -1.448  35.199  18.734  1.00 18.63           H  0.072
ATOM   1793  HB3 LYS A 116      -0.824  36.533  18.137  1.00 18.63           H  0.072
ATOM   1794  HG2 LYS A 116      -2.584  37.754  18.972  1.00 15.46           H  0.066
ATOM   1795  HG3 LYS A 116      -3.265  36.427  19.520  1.00 15.46           H  0.066
ATOM   1796  HD2 LYS A 116      -1.346  36.086  20.881  1.00 19.30           H  0.073
ATOM   1797  HD3 LYS A 116      -0.827  37.518  20.426  1.00 19.30           H  0.073
ATOM   1798  HE2 LYS A 116      -2.700  38.518  21.475  1.00 20.28           H  0.075
ATOM   1799  HE3 LYS A 116      -3.188  37.083  21.954  1.00 20.28           H  0.075
ATOM   1800  HZ1 LYS A 116      -1.199  37.005  23.206  1.00 15.15           H  0.065
ATOM   1801  HZ2 LYS A 116      -0.977  38.404  22.903  1.00 15.15           H  0.065
ATOM   1802  HZ3 LYS A 116      -2.125  38.007  23.691  1.00 15.15           H  0.065
ATOM   1803  N   GLY A 117      -3.912  37.400  16.125  1.00 15.66           N  0.066
ATOM   1804  CA  GLY A 117      -4.297  38.657  15.532  1.00 19.71           C  0.074
ATOM   1805  C   GLY A 117      -4.331  38.632  14.023  1.00 26.48           C  0.086
ATOM   1806  O   GLY A 117      -4.696  39.641  13.406  1.00 27.30           O  0.087
ATOM   1807  H   GLY A 117      -4.567  36.901  16.374  1.00 18.67           H  0.072
ATOM   1808  HA2 GLY A 117      -5.182  38.896  15.849  1.00 23.54           H  0.081
ATOM   1809  HA3 GLY A 117      -3.668  39.341  15.808  1.00 23.54           H  0.081
ATOM   1810  N   THR A 118      -3.951  37.529  13.402  1.00 18.32           N  0.072
ATOM   1811  CA  THR A 118      -3.951  37.441  11.949  1.00 17.39           C  0.070
ATOM   1812  C   THR A 118      -5.250  36.772  11.503  1.00 19.91           C  0.075
ATOM   1813  O   THR A 118      -6.069  36.339  12.303  1.00 15.68           O  0.066
ATOM   1814  CB  THR A 118      -2.723  36.670  11.443  1.00 17.66           C  0.070
ATOM   1815  OG1 THR A 118      -2.896  35.276  11.707  1.00 16.40           O  0.068
ATOM   1816  CG2 THR A 118      -1.437  37.203  12.082  1.00 17.88           C  0.071
ATOM   1817  H   THR A 118      -3.688  36.814  13.800  1.00 21.86           H  0.078
ATOM   1818  HA  THR A 118      -3.900  38.327  11.559  1.00 20.75           H  0.076
ATOM   1819  HB  THR A 118      -2.626  36.791  10.485  1.00 21.07           H  0.077
ATOM   1820  HG1 THR A 118      -3.453  35.168  12.326  1.00 19.55           H  0.074
ATOM   1821 HG21 THR A 118      -0.667  36.745  11.711  1.00 21.33           H  0.077
ATOM   1822 HG22 THR A 118      -1.351  38.154  11.909  1.00 21.33           H  0.077
ATOM   1823 HG23 THR A 118      -1.458  37.058  13.041  1.00 21.33           H  0.077
ATOM   1824  N   ASP A 119      -5.469  36.729  10.195  1.00 17.79           N  0.071
ATOM   1825  CA  ASP A 119      -6.672  36.078   9.634  1.00 20.78           C  0.076
ATOM   1826  C   ASP A 119      -6.420  34.578   9.636  1.00 16.81           C  0.069
ATOM   1827  O   ASP A 119      -5.970  34.066   8.636  1.00 22.78           O  0.080
ATOM   1828  CB  ASP A 119      -6.947  36.566   8.209  1.00 21.42           C  0.077
ATOM   1829  CG  ASP A 119      -8.203  36.029   7.557  1.00 41.47           C  0.108
ATOM   1830  OD1 ASP A 119      -8.750  35.021   8.026  1.00 29.65           O  0.091
ATOM   1831  OD2 ASP A 119      -8.603  36.640   6.576  1.00 43.50           O  0.110
```

ATOM	1832	H	ASP	A	119	-4.828	37.132	9.490	1.00	21.23	H	0.077
ATOM	1833	HA	ASP	A	119	-7.531	36.301	10.265	1.00	24.82	H	0.083
ATOM	1834	HB2	ASP	A	119	-7.022	37.652	8.230	1.00	25.58	H	0.085
ATOM	1835	HB3	ASP	A	119	-6.099	36.302	7.580	1.00	25.58	H	0.085
ATOM	1836	N	VAL	A	120	-6.681	33.920	10.747	1.00	16.94	N	0.069
ATOM	1837	CA	VAL	A	120	-6.385	32.486	10.799	1.00	14.69	C	0.064
ATOM	1838	C	VAL	A	120	-7.406	31.633	10.059	1.00	21.19	C	0.077
ATOM	1839	O	VAL	A	120	-7.137	30.443	9.809	1.00	18.16	O	0.071
ATOM	1840	CB	VAL	A	120	-6.265	32.036	12.268	1.00	16.99	C	0.069
ATOM	1841	CG1	VAL	A	120	-5.104	32.694	12.879	1.00	18.42	C	0.072
ATOM	1842	CG2	VAL	A	120	-7.501	32.403	13.074	1.00	20.70	C	0.076
ATOM	1843	H	VAL	A	120	-7.032	34.253	11.458	1.00	20.21	H	0.075
ATOM	1844	HA	VAL	A	120	-5.536	32.324	10.358	1.00	17.51	H	0.070
ATOM	1845	HB	VAL	A	120	-6.166	31.071	12.281	1.00	20.27	H	0.075
ATOM	1846	HG11	VAL	A	120	-5.015	32.390	13.796	1.00	21.98	H	0.078
ATOM	1847	HG12	VAL	A	120	-4.308	32.463	12.375	1.00	21.98	H	0.078
ATOM	1848	HG13	VAL	A	120	-5.239	33.655	12.861	1.00	21.98	H	0.078
ATOM	1849	HG21	VAL	A	120	-7.403	32.061	13.976	1.00	24.72	H	0.083
ATOM	1850	HG22	VAL	A	120	-7.589	33.369	13.094	1.00	24.72	H	0.083
ATOM	1851	HG23	VAL	A	120	-8.281	32.008	12.653	1.00	24.72	H	0.083
ATOM	1852	N	GLN	A	121	-8.571	32.203	9.696	1.00	21.72	N	0.078
ATOM	1853	CA	GLN	A	121	-9.550	31.465	8.883	1.00	17.13	C	0.069
ATOM	1854	C	GLN	A	121	-8.947	31.048	7.544	1.00	19.54	C	0.074
ATOM	1855	O	GLN	A	121	-9.372	30.069	6.933	1.00	22.42	O	0.079
ATOM	1856	CB	GLN	A	121	-10.791	32.332	8.631	1.00	24.97	C	0.084
ATOM	1857	CG	GLN	A	121	-11.690	32.638	9.836	1.00	48.74	C	0.117
ATOM	1858	CD	GLN	A	121	-10.940	32.986	11.127	1.00	67.31	C	0.137
ATOM	1859	OE1	GLN	A	121	-11.124	32.328	12.168	1.00	54.70	O	0.124
ATOM	1860	NE2	GLN	A	121	-10.138	34.068	11.088	1.00	50.63	N	0.119
ATOM	1861	H	GLN	A	121	-8.812	33.001	9.904	1.00	25.94	H	0.085
ATOM	1862	HA	GLN	A	121	-9.824	30.672	9.369	1.00	20.44	H	0.076
ATOM	1863	HB2	GLN	A	121	-10.492	33.185	8.280	1.00	29.84	H	0.091
ATOM	1864	HB3	GLN	A	121	-11.344	31.878	7.976	1.00	29.84	H	0.091
ATOM	1865	HG2	GLN	A	121	-12.255	33.395	9.614	1.00	58.37	H	0.128
ATOM	1866	HG3	GLN	A	121	-12.237	31.858	10.018	1.00	58.37	H	0.128
ATOM	1867	HE21	GLN	A	121	-10.066	34.525	10.363	1.00	60.63	H	0.130
ATOM	1868	HE22	GLN	A	121	-9.697	34.302	11.789	1.00	60.63	H	0.130
ATOM	1869	N	ALA	A	122	-7.965	31.806	7.072	1.00	15.02	N	0.065
ATOM	1870	CA	ALA	A	122	-7.286	31.483	5.825	1.00	15.94	C	0.067
ATOM	1871	C	ALA	A	122	-6.691	30.084	5.840	1.00	21.41	C	0.077
ATOM	1872	O	ALA	A	122	-6.606	29.440	4.791	1.00	18.11	O	0.071
ATOM	1873	CB	ALA	A	122	-6.175	32.513	5.585	1.00	19.70	C	0.074
ATOM	1874	H	ALA	A	122	-7.671	32.517	7.456	1.00	17.91	H	0.071
ATOM	1875	HA	ALA	A	122	-7.928	31.522	5.099	1.00	19.00	H	0.073
ATOM	1876	HB1	ALA	A	122	-5.717	32.296	4.758	1.00	23.51	H	0.081
ATOM	1877	HB2	ALA	A	122	-6.571	33.396	5.522	1.00	23.51	H	0.081
ATOM	1878	HB3	ALA	A	122	-5.550	32.482	6.326	1.00	23.51	H	0.081
ATOM	1879	N	TRP	A	123	-6.302	29.591	7.028	1.00	19.02	N	0.073
ATOM	1880	CA	TRP	A	123	-5.687	28.276	7.157	1.00	10.26	C	0.054
ATOM	1881	C	TRP	A	123	-6.647	27.123	6.895	1.00	15.02	C	0.065
ATOM	1882	O	TRP	A	123	-6.190	25.997	6.644	1.00	17.35	O	0.070
ATOM	1883	CB	TRP	A	123	-5.063	28.150	8.564	1.00	17.11	C	0.069
ATOM	1884	CG	TRP	A	123	-3.820	29.002	8.618	1.00	12.51	C	0.059
ATOM	1885	CD1	TRP	A	123	-3.700	30.210	9.203	1.00	21.27	C	0.077
ATOM	1886	CD2	TRP	A	123	-2.553	28.712	8.007	1.00	16.86	C	0.069
ATOM	1887	NE1	TRP	A	123	-2.441	30.700	9.005	1.00	15.17	N	0.065
ATOM	1888	CE2	TRP	A	123	-1.715	29.805	8.264	1.00	15.04	C	0.065
ATOM	1889	CE3	TRP	A	123	-2.053	27.631	7.257	1.00	16.54	C	0.068
ATOM	1890	CZ2	TRP	A	123	-0.388	29.847	7.841	1.00	19.51	C	0.074
ATOM	1891	CZ3	TRP	A	123	-0.740	27.683	6.816	1.00	27.91	C	0.088
ATOM	1892	CH2	TRP	A	123	0.076	28.789	7.102	1.00	25.51	C	0.084
ATOM	1893	H	TRP	A	123	-6.388	30.007	7.775	1.00	22.70	H	0.080
ATOM	1894	HA	TRP	A	123	-4.982	28.195	6.495	1.00	12.19	H	0.058
ATOM	1895	HB2	TRP	A	123	-5.691	28.462	9.234	1.00	20.41	H	0.076
ATOM	1896	HB3	TRP	A	123	-4.823	27.227	8.739	1.00	20.41	H	0.076
ATOM	1897	HD1	TRP	A	123	-4.375	30.645	9.672	1.00	25.40	H	0.084
ATOM	1898	HE1	TRP	A	123	-2.149	31.453	9.300	1.00	18.09	H	0.071
ATOM	1899	HE3	TRP	A	123	-2.591	26.899	7.061	1.00	19.73	H	0.074
ATOM	1900	HZ2	TRP	A	123	0.163	30.566	8.052	1.00	23.29	H	0.081
ATOM	1901	HZ3	TRP	A	123	-0.394	26.975	6.323	1.00	33.37	H	0.097
ATOM	1902	HH2	TRP	A	123	0.949	28.805	6.784	1.00	30.49	H	0.092
ATOM	1903	N	ILE	A	124	-7.951	27.356	7.001	1.00	18.44	N	0.072
ATOM	1904	CA	ILE	A	124	-8.942	26.325	6.731	1.00	18.31	C	0.072
ATOM	1905	C	ILE	A	124	-9.769	26.653	5.501	1.00	23.40	C	0.081
ATOM	1906	O	ILE	A	124	-10.687	25.897	5.168	1.00	21.54	O	0.078
ATOM	1907	CB	ILE	A	124	-9.861	26.084	7.944	1.00	22.60	C	0.080
ATOM	1908	CG1	ILE	A	124	-10.687	27.318	8.268	1.00	24.24	C	0.082
ATOM	1909	CG2	ILE	A	124	-9.021	25.653	9.160	1.00	20.22	C	0.075

ATOM	1910	CD1	ILE	A	124	-11.806	27.058	9.287	1.00	27.33	C	0.087
ATOM	1911	H	ILE	A	124	-8.288	28.113	7.231	1.00	22.00	H	0.078
ATOM	1912	HA	ILE	A	124	-8.474	25.493	6.561	1.00	21.85	H	0.078
ATOM	1913	HB	ILE	A	124	-10.481	25.372	7.718	1.00	26.99	H	0.087
ATOM	1914	HG12	ILE	A	124	-10.102	27.998	8.638	1.00	28.97	H	0.090
ATOM	1915	HG13	ILE	A	124	-11.098	27.643	7.452	1.00	28.97	H	0.090
ATOM	1916	HG21	ILE	A	124	-9.615	25.465	9.904	1.00	24.14	H	0.082
ATOM	1917	HG22	ILE	A	124	-8.518	24.856	8.930	1.00	24.14	H	0.082
ATOM	1918	HG23	ILE	A	124	-8.413	26.371	9.395	1.00	24.14	H	0.082
ATOM	1919	HD11	ILE	A	124	-12.383	27.837	9.328	1.00	32.67	H	0.096
ATOM	1920	HD12	ILE	A	124	-12.317	26.284	9.004	1.00	32.67	H	0.096
ATOM	1921	HD13	ILE	A	124	-11.410	26.893	10.157	1.00	32.67	H	0.096
ATOM	1922	N	ARG	A	125	-9.469	27.754	4.822	1.00	22.40	N	0.079
ATOM	1923	CA	ARG	A	125	-10.292	28.206	3.701	1.00	29.26	C	0.090
ATOM	1924	C	ARG	A	125	-10.223	27.175	2.583	1.00	25.07	C	0.084
ATOM	1925	O	ARG	A	125	-9.147	26.663	2.252	1.00	24.34	O	0.083
ATOM	1926	CB	ARG	A	125	-9.836	29.588	3.219	1.00	40.74	C	0.107
ATOM	1927	CG	ARG	A	125	-9.977	29.854	1.714	1.00	46.80	C	0.114
ATOM	1928	CD	ARG	A	125	-9.658	31.325	1.372	1.00	41.37	C	0.108
ATOM	1929	NE	ARG	A	125	-8.211	31.514	1.281	1.00	63.17	N	0.133
ATOM	1930	CZ	ARG	A	125	-7.550	32.576	1.728	1.00	44.98	C	0.112
ATOM	1931	NH1	ARG	A	125	-8.180	33.642	2.185	1.00	42.23	N	0.109
ATOM	1932	NH2	ARG	A	125	-6.220	32.563	1.718	1.00	48.43	N	0.116
ATOM	1933	H	ARG	A	125	-8.793	28.259	4.988	1.00	26.76	H	0.087
ATOM	1934	HA	ARG	A	125	-11.216	28.299	3.980	1.00	34.99	H	0.099
ATOM	1935	HB2	ARG	A	125	-10.364	30.259	3.679	1.00	48.77	H	0.117
ATOM	1936	HB3	ARG	A	125	-8.898	29.693	3.442	1.00	48.77	H	0.117
ATOM	1937	HG2	ARG	A	125	-9.360	29.286	1.228	1.00	56.04	H	0.125
ATOM	1938	HG3	ARG	A	125	-10.888	29.667	1.438	1.00	56.04	H	0.125
ATOM	1939	HD2	ARG	A	125	-10.056	31.556	0.518	1.00	49.53	H	0.118
ATOM	1940	HD3	ARG	A	125	-10.005	31.905	2.067	1.00	49.53	H	0.118
ATOM	1941	HE	ARG	A	125	-7.752	30.889	0.910	1.00	75.68	H	0.146
ATOM	1942	HH11	ARG	A	125	-9.040	33.660	2.198	1.00	50.56	H	0.119
ATOM	1943	HH12	ARG	A	125	-7.731	34.318	2.469	1.00	50.56	H	0.119
ATOM	1944	HH21	ARG	A	125	-5.799	31.872	1.424	1.00	57.99	H	0.127
ATOM	1945	HH22	ARG	A	125	-5.781	33.244	2.004	1.00	57.99	H	0.127
ATOM	1946	N	GLY	A	126	-11.399	26.829	2.037	1.00	29.34	N	0.091
ATOM	1947	CA	GLY	A	126	-11.487	25.864	0.972	1.00	33.50	C	0.097
ATOM	1948	C	GLY	A	126	-11.601	24.412	1.409	1.00	34.34	C	0.098
ATOM	1949	O	GLY	A	126	-12.087	23.595	0.623	1.00	25.97	O	0.085
ATOM	1950	H	GLY	A	126	-12.158	27.153	2.280	1.00	35.09	H	0.099
ATOM	1951	HA2	GLY	A	126	-12.267	26.069	0.435	1.00	40.08	H	0.106
ATOM	1952	HA3	GLY	A	126	-10.693	25.941	0.420	1.00	40.08	H	0.106
ATOM	1953	N	CYS	A	127	-11.185	24.080	2.638	1.00	25.34	N	0.084
ATOM	1954	CA	CYS	A	127	-11.093	22.688	3.061	1.00	26.43	C	0.086
ATOM	1955	C	CYS	A	127	-12.465	22.066	3.207	1.00	29.92	C	0.092
ATOM	1956	O	CYS	A	127	-13.395	22.709	3.690	1.00	23.30	O	0.081
ATOM	1957	CB	CYS	A	127	-10.397	22.565	4.404	1.00	18.04	C	0.071
ATOM	1958	SG	CYS	A	127	-8.761	23.320	4.415	1.00	21.59	S	0.078
ATOM	1959	H	CYS	A	127	-10.951	24.646	3.242	1.00	30.29	H	0.092
ATOM	1960	HA	CYS	A	127	-10.584	22.215	2.384	1.00	31.59	H	0.094
ATOM	1961	HB2	CYS	A	127	-10.936	23.007	5.079	1.00	21.53	H	0.078
ATOM	1962	HB3	CYS	A	127	-10.296	21.626	4.622	1.00	21.53	H	0.078
ATOM	1963	N	ARG	A	128	-12.562	20.777	2.850	1.00	23.80	N	0.082
ATOM	1964	CA	ARG	A	128	-13.755	19.981	3.123	1.00	31.34	C	0.094
ATOM	1965	C	ARG	A	128	-13.726	19.620	4.604	1.00	29.74	C	0.091
ATOM	1966	O	ARG	A	128	-12.824	18.904	5.057	1.00	37.86	O	0.103
ATOM	1967	CB	ARG	A	128	-13.786	18.732	2.241	1.00	35.19	C	0.099
ATOM	1968	CG	ARG	A	128	-15.037	17.886	2.417	1.00	54.15	C	0.123
ATOM	1969	CD	ARG	A	128	-15.377	17.077	1.159	1.00	65.26	C	0.135
ATOM	1970	NE	ARG	A	128	-16.586	16.280	1.350	1.00	61.01	N	0.131
ATOM	1971	CZ	ARG	A	128	-17.815	16.781	1.399	1.00	86.59	C	0.156
ATOM	1972	NH1	ARG	A	128	-18.042	18.080	1.259	1.00	72.14	N	0.142
ATOM	1973	NH2	ARG	A	128	-18.843	15.959	1.597	1.00	67.27	N	0.137
ATOM	1974	H	ARG	A	128	-11.941	20.342	2.446	1.00	28.44	H	0.089
ATOM	1975	HA	ARG	A	128	-14.556	20.491	2.928	1.00	37.49	H	0.102
ATOM	1976	HB2	ARG	A	128	-13.742	19.005	1.311	1.00	42.10	H	0.109
ATOM	1977	HB3	ARG	A	128	-13.021	18.177	2.460	1.00	42.10	H	0.109
ATOM	1978	HG2	ARG	A	128	-14.900	17.263	3.147	1.00	64.85	H	0.135
ATOM	1979	HG3	ARG	A	128	-15.788	18.467	2.613	1.00	64.85	H	0.135
ATOM	1980	HD2	ARG	A	128	-15.524	17.684	0.417	1.00	78.19	H	0.148
ATOM	1981	HD3	ARG	A	128	-14.643	16.476	0.956	1.00	78.19	H	0.148
ATOM	1982	HE	ARG	A	128	-16.495	15.430	1.437	1.00	73.09	H	0.143
ATOM	1983	HH11	ARG	A	128	-17.385	18.620	1.133	1.00	86.44	H	0.156
ATOM	1984	HH12	ARG	A	128	-18.846	18.382	1.294	1.00	86.44	H	0.156
ATOM	1985	HH21	ARG	A	128	-18.708	15.116	1.691	1.00	80.61	H	0.150
ATOM	1986	HH22	ARG	A	128	-19.644	16.273	1.630	1.00	80.61	H	0.150
ATOM	1987	N	LEU	A	129	-14.684	20.139	5.364	1.00	29.58	N	0.091

```
ATOM   1988  CA  LEU A 129     -14.693  19.925   6.814  1.00 33.47           C  0.097
ATOM   1989  C   LEU A 129     -16.104  19.588   7.278  1.00 50.16           C  0.118
ATOM   1990  O   LEU A 129     -16.828  18.869   6.586  1.00 57.41           O  0.127
ATOM   1991  CB  LEU A 129     -14.156  21.158   7.560  1.00 26.79           C  0.087
ATOM   1992  CG  LEU A 129     -12.686  21.457   7.345  1.00 30.03           C  0.092
ATOM   1993  CD1 LEU A 129     -12.354  22.889   7.724  1.00 35.81           C  0.100
ATOM   1994  CD2 LEU A 129     -11.880  20.485   8.191  1.00 32.98           C  0.096
ATOM   1995  OXT LEU A 129     -16.544  20.011   8.350  1.00 47.32           O  0.115
ATOM   1996  H   LEU A 129     -15.337  20.616   5.070  1.00 35.37           H  0.099
ATOM   1997  HA  LEU A 129     -14.106  19.185   7.036  1.00 40.04           H  0.106
ATOM   1998  HB2 LEU A 129     -14.656  21.935   7.264  1.00 32.02           H  0.095
ATOM   1999  HB3 LEU A 129     -14.288  21.021   8.511  1.00 32.02           H  0.095
ATOM   2000  HG  LEU A 129     -12.458  21.353   6.408  1.00 35.92           H  0.100
ATOM   2001 HD11 LEU A 129     -11.397  23.024   7.644  1.00 42.85           H  0.110
ATOM   2002 HD12 LEU A 129     -12.824  23.491   7.125  1.00 42.85           H  0.110
ATOM   2003 HD13 LEU A 129     -12.636  23.048   8.638  1.00 42.85           H  0.110
ATOM   2004 HD21 LEU A 129     -10.935  20.647   8.043  1.00 39.45           H  0.105
ATOM   2005 HD22 LEU A 129     -12.097  20.625   9.126  1.00 39.45           H  0.105
ATOM   2006 HD23 LEU A 129     -12.105  19.578   7.930  1.00 39.45           H  0.105
HETATM 2007 CL    CL A1131     -10.736  29.644  11.875  1.00 25.99          Cl  0.085
HETATM 2008 CL    CL A1135       7.486   7.539  29.688  1.00 37.26          Cl  0.102
HETATM 2009 CL    CL A1137      20.076  11.867  24.486  1.00 33.43          Cl  0.097
HETATM 2010 NA    NA A1138       9.664  15.826  31.415  1.00 23.68          Na  0.081
TER
HETATM 2011  C1  RII A1140     -12.045  35.735   4.387  0.57 37.59      A    C  0.103
HETATM 2012  C2  RII A1140      -9.488  36.215   3.874  0.57 30.64      A    C  0.093
HETATM 2013  C3  RII A1140     -10.220  34.145   5.444  0.57 19.03      A    C  0.073
HETATM 2014  C4  RII A1140     -11.317  36.273   8.506  0.57 34.01      A    C  0.098
HETATM 2015  C5  RII A1140     -13.338  35.523   8.721  0.57 28.61      A    C  0.089
HETATM 2016  C6  RII A1140     -12.978  35.423   7.456  0.57 32.67      A    C  0.096
HETATM 2017  C7  RII A1140     -10.375  38.981   6.543  0.57 31.44      A    C  0.094
HETATM 2018  C8  RII A1140     -11.484  40.221   5.180  0.57 31.07      A    C  0.093
HETATM 2019  C9  RII A1140     -11.529  38.943   4.752  0.57 30.48      A    C  0.092
HETATM 2020  N1  RII A1140     -11.688  35.898   7.301  0.57 25.02      A    N  0.084
HETATM 2021  N2  RII A1140     -12.279  36.061   9.391  0.57 37.26      A    N  0.102
HETATM 2022  N3  RII A1140     -10.819  38.161   5.631  0.57 19.94      A    N  0.075
HETATM 2023  N4  RII A1140     -10.768  40.231   6.285  0.57 25.59      A    N  0.085
HETATM 2024  O1  RII A1140     -12.985  35.572   3.728  0.57 29.91      A    O  0.091
HETATM 2025  O2  RII A1140      -8.876  36.378   2.936  0.57 35.15      A    O  0.099
HETATM 2026  O3  RII A1140     -10.045  33.038   5.412  0.57 33.08      A    O  0.096
HETATM 2027 RE1  RII A1140     -10.525  36.015   5.464  0.57 22.14      A   Re  0.079
HETATM 2028  H21 RII A1140     -12.242  36.232  10.231  0.57 44.59      A    H  0.112
HETATM 2029  H41 RII A1140     -10.492  36.641   8.716  0.57 40.69      A    H  0.107
HETATM 2030  H42 RII A1140     -10.588  40.922   6.764  0.57 30.58      A    H  0.093
HETATM 2031  H51 RII A1140     -14.150  35.266   9.086  0.57 34.22      A    H  0.098
HETATM 2032  H61 RII A1140     -13.509  35.085   6.772  0.57 39.08      A    H  0.105
HETATM 2033  H71 RII A1140      -9.857  38.731   7.274  0.57 37.60      A    H  0.103
HETATM 2034  H81 RII A1140     -11.883  40.955   4.774  0.57 37.16      A    H  0.102
HETATM 2035  H91 RII A1140     -11.964  38.638   3.990  0.57 36.46      A    H  0.101
HETATM 2036  C1  RI3 A1141      -7.745   9.168  13.190  0.84 48.96      A    C  0.117
HETATM 2037  C2  RI3 A1141      -8.292  11.720  13.122  0.84 34.19      A    C  0.098
HETATM 2038  C3  RI3 A1141      -6.002  10.902  13.911  0.84 19.74      A    C  0.074
HETATM 2039  C7  RI3 A1141     -10.491  11.041  15.967  0.84 26.47      A    C  0.086
HETATM 2040  C8  RI3 A1141     -11.832   9.469  15.387  0.84 42.61      A    C  0.109
HETATM 2041  C9  RI3 A1141     -10.649   9.283  14.780  0.84 35.64      A    C  0.100
HETATM 2042  N3  RI3 A1141      -9.794  10.291  15.160  0.84 36.74      A    N  0.101
HETATM 2043  N4  RI3 A1141     -11.720  10.556  16.116  0.84 43.44      A    N  0.110
HETATM 2044  O1  RI3 A1141      -7.714   8.333  12.384  0.84 36.42      A    O  0.101
HETATM 2045  O2  RI3 A1141      -8.609  12.438  12.320  0.84 29.66      A    O  0.091
HETATM 2046  O3  RI3 A1141      -4.982  11.152  13.523  0.84 33.16      A    O  0.096
HETATM 2047  O4  RI3 A1141      -7.265   9.134  16.106  0.84 27.56      A    O  0.088
HETATM 2048 RE1  RI3 A1141      -7.751  10.520  14.489  0.84 29.53      A   Re  0.091
HETATM 2049  H42 RI3 A1141     -12.337  10.900  16.605  0.84 52.01      A    H  0.121
HETATM 2050  H71 RI3 A1141     -10.181  11.811  16.382  0.84 31.65      A    H  0.094
HETATM 2051  H81 RI3 A1141     -12.584   8.932  15.308  0.84 51.00      A    H  0.119
HETATM 2052  H91 RI3 A1141     -10.439   8.587  14.202  0.84 42.64      A    H  0.109
HETATM 2053  C1  RII A1142       1.272  23.057  36.533  0.56 36.43      A    C  0.101
HETATM 2054  C2  RII A1142      -1.268  23.544  36.487  0.56 50.09      A    C  0.118
HETATM 2055  C3  RII A1142      -0.214  22.306  34.403  0.56 32.57      A    C  0.095
HETATM 2056  C4  RII A1142       1.999  24.714  32.764  0.56 40.82      A    C  0.107
HETATM 2057  C5  RII A1142       4.041  24.672  33.431  0.56 33.14      A    C  0.096
HETATM 2058  C6  RII A1142       3.259  24.354  34.446  0.56 36.69      A    C  0.101
HETATM 2059  C7  RII A1142      -0.220  26.956  36.004  0.56 37.61      A    C  0.103
HETATM 2060  C8  RII A1142       1.416  27.551  37.263  0.56 34.82      A    C  0.099
HETATM 2061  C9  RII A1142       1.565  26.253  36.939  0.56 43.53      A    C  0.110
HETATM 2062  N1  RII A1142       1.933  24.374  34.045  0.56 25.80      A    N  0.085
HETATM 2063  N2  RII A1142       3.243  24.904  32.361  0.56 30.85      A    N  0.093
HETATM 2064  N3  RII A1142       0.514  25.886  36.132  0.56 16.45      A    N  0.068
```

```
HETATM 2065  N4  RII A1142       0.314  27.971  36.680  0.56 30.02      A    N  0.092
HETATM 2066  O1  RII A1142       1.953  22.532  37.311  0.56 32.02      A    O  0.095
HETATM 2067  O2  RII A1142      -2.109  23.310  37.213  0.56 34.21      A    O  0.098
HETATM 2068  O3  RII A1142      -0.466  21.341  33.881  0.56 35.60      A    O  0.100
HETATM 2069 RE1  RII A1142       0.174  23.935  35.284  0.56 29.43      A   Re  0.091
HETATM 2070  H21 RII A1142       3.497  25.120  31.568  0.56 36.89      A    H  0.102
HETATM 2071  H41 RII A1142       1.273  24.804  32.194  0.56 48.86      A    H  0.117
HETATM 2072  H42 RII A1142      -0.010  28.765  36.726  0.56 35.90      A    H  0.100
HETATM 2073  H51 RII A1142       4.960  24.738  33.446  0.56 39.64      A    H  0.105
HETATM 2074  H61 RII A1142       3.556  24.157  35.304  0.56 43.91      A    H  0.111
HETATM 2075  H71 RII A1142      -1.005  27.004  35.508  0.56 45.01      A    H  0.112
HETATM 2076  H81 RII A1142       1.985  28.055  37.795  0.56 41.67      A    H  0.108
HETATM 2077  H91 RII A1142       2.258  25.698  37.209  0.56 52.11      A    H  0.121
HETATM 2078  O   HOH S   1       7.305  12.618   9.065  1.00 18.08           O  0.071
HETATM 2079  O   HOH S   2       4.779   9.013  10.492  1.00 27.56           O  0.088
HETATM 2080  O   HOH S   3       4.495  12.095   5.105  1.00 31.26           O  0.094
HETATM 2081  O   HOH S   4       2.042  19.929   4.247  1.00 26.24           O  0.086
HETATM 2082  O   HOH S   6      -4.269  21.223   1.780  1.00 26.15           O  0.086
HETATM 2083  O   HOH S   8      -4.545  18.229   2.615  1.00 23.86           O  0.082
HETATM 2084  O   HOH S  17     -13.507  24.275  25.274  1.00 23.69           O  0.081
HETATM 2085  O   HOH S  18     -10.301  32.073  23.817  1.00 21.02           O  0.077
HETATM 2086  O   HOH S  19      -5.887  34.536  16.655  1.00 23.21           O  0.081
HETATM 2087  O   HOH S  20      -8.953  33.826  15.858  1.00 27.13           O  0.087
HETATM 2088  O   HOH S  22      -0.619   7.367  22.390  1.00 34.19           O  0.098
HETATM 2089  O   HOH S  24       6.925  26.179   9.073  1.00 29.74           O  0.091
HETATM 2090  O   HOH S  25      -2.780  10.637  25.315  1.00 27.56           O  0.088
HETATM 2091  O   HOH S  26       8.514  27.853  15.502  1.00 18.34           O  0.072
HETATM 2092  O   HOH S  27       8.314  26.541  11.357  1.00 21.56           O  0.078
HETATM 2093  O   HOH S  29       8.552  25.926  19.755  1.00 28.13           O  0.089
HETATM 2094  O   HOH S  30       6.695  28.282  19.941  1.00 26.06           O  0.085
HETATM 2095  O   HOH S  31       9.673  20.208  10.677  1.00 26.16           O  0.086
HETATM 2096  O   HOH S  32       4.322  21.165   5.189  1.00 26.15           O  0.086
HETATM 2097  O   HOH S  33      -0.703  40.326  19.317  1.00 22.53           O  0.079
HETATM 2098  O   HOH S  35      -2.219  36.835   7.350  1.00 27.23           O  0.087
HETATM 2099  O   HOH S  36      12.892  14.956  14.492  1.00 15.05           O  0.065
HETATM 2100  O   HOH S  39      17.337  19.943  17.825  1.00 20.00           O  0.075
HETATM 2101  O   HOH S  41      14.652  21.861  31.496  1.00 31.89           O  0.094
HETATM 2102  O   HOH S  42      15.322  16.551  26.164  1.00 15.24           O  0.065
HETATM 2103  O   HOH S  44       2.958  17.947  19.443  1.00 11.32           O  0.056
HETATM 2104  O   HOH S  45      10.316  24.203  18.465  1.00 18.04           O  0.071
HETATM 2105  O   HOH S  46       5.348  23.002  25.219  1.00 21.32           O  0.077
HETATM 2106  O   HOH S  49      12.135   7.454  30.873  1.00 15.84           O  0.067
HETATM 2107  O   HOH S  50      11.360  14.486  30.397  1.00 15.20           O  0.065
HETATM 2108  O   HOH S  53       9.547  13.943  32.706  1.00 25.63           O  0.085
HETATM 2109  O   HOH S  54       4.395   8.404  30.529  1.00 26.68           O  0.086
HETATM 2110  O   HOH S  55       6.910  10.337  34.213  1.00 25.41           O  0.084
HETATM 2111  O   HOH S  56      -2.281  13.861  27.878  1.00 25.81           O  0.085
HETATM 2112  O   HOH S  57       5.408   7.654  18.109  1.00 25.96           O  0.085
HETATM 2113  O   HOH S  58       0.888   8.326  20.242  1.00 25.60           O  0.085
HETATM 2114  O   HOH S  59       2.572  15.126  16.649  1.00 24.29           O  0.082
HETATM 2115  O   HOH S  60       5.701   8.928  13.075  1.00 28.07           O  0.089
HETATM 2116  O   HOH S  61       0.925  13.491  16.110  1.00 16.73           O  0.068
HETATM 2117  O   HOH S  62       3.133   6.652  13.903  1.00 28.43           O  0.089
HETATM 2118  O   HOH S  63      -3.058  10.418  10.888  1.00 34.26           O  0.098
HETATM 2119  O   HOH S  64      -1.470   9.397  19.460  1.00 23.05           O  0.080
HETATM 2120  O   HOH S  65       0.467  12.429  18.797  1.00 22.31           O  0.079
HETATM 2121  O   HOH S  66      -1.460  12.952  25.390  1.00 18.96           O  0.073
HETATM 2122  O   HOH S  67      -6.594  16.425  27.669  1.00 29.08           O  0.090
HETATM 2123  O   HOH S  69      -7.702  13.427  20.600  1.00 25.44           O  0.084
HETATM 2124  O   HOH S  70      -9.386  14.726  23.732  1.00 25.21           O  0.084
HETATM 2125  O   HOH S  71      -4.966  22.189  32.070  1.00 35.12           O  0.099
HETATM 2126  O   HOH S  72      -5.653  29.124  29.495  1.00 16.66           O  0.068
HETATM 2127  O   HOH S  73      -1.660  28.221  28.377  1.00 21.50           O  0.078
HETATM 2128  O   HOH S  74       4.373  33.522  24.450  1.00 35.66           O  0.100
HETATM 2129  O   HOH S  75       6.458  27.657  22.596  1.00 23.34           O  0.081
HETATM 2130  O   HOH S  77       1.577  38.895  18.354  1.00 33.11           O  0.096
HETATM 2131  O   HOH S  78       4.812  39.371  19.039  1.00 28.84           O  0.090
HETATM 2132  O   HOH S  79       8.758  29.543   8.425  1.00 17.43           O  0.070
HETATM 2133  O   HOH S  80       2.065  34.892   9.900  1.00 20.33           O  0.075
HETATM 2134  O   HOH S  81       4.853  34.355   9.961  1.00 14.83           O  0.064
HETATM 2135  O   HOH S  82       1.191  37.310  15.505  1.00 16.41           O  0.068
HETATM 2136  O   HOH S  83      -1.229  40.190  14.621  1.00 25.30           O  0.084
HETATM 2137  O   HOH S  84       0.167  39.631  22.115  1.00 27.26           O  0.087
HETATM 2138  O   HOH S  85      -0.191  37.588  25.697  1.00 29.57           O  0.091
HETATM 2139  O   HOH S  86      -3.380  40.290  18.178  1.00 21.74           O  0.078
HETATM 2140  O   HOH S  88      -2.425  33.708   9.638  1.00 21.66           O  0.078
HETATM 2141  O   HOH S  89      -6.908  35.980  14.982  1.00 21.71           O  0.078
HETATM 2142  O   HOH S  90      -3.796  38.382   8.488  1.00 21.42           O  0.077
```

```
HETATM 2143  O   HOH S  91      -3.664  34.397   7.425  1.00 32.02           O  0.095
HETATM 2144  O   HOH S  92       0.500  33.437   8.289  1.00 25.93           O  0.085
HETATM 2145  O   HOH S  93     -20.233  13.634   1.804  1.00 22.27           O  0.079
HETATM 2146  O   HOH S  94     -10.118  18.689   4.843  1.00 32.91           O  0.096
HETATM 2147  O   HOH S  95     -10.256  19.586   1.328  1.00 34.40           O  0.098
HETATM 2148  O   HOH S  98       9.846  19.078   8.475  1.00 19.49           O  0.074
HETATM 2149  O   HOH S 100      19.206   8.115  32.762  1.00 36.42           O  0.101
HETATM 2150  O   HOH S 101       7.323  19.390   7.059  1.00 21.85           O  0.078
HETATM 2151  O   HOH S 104       2.835  26.853  29.620  1.00 22.57           O  0.079
HETATM 2152  O   HOH S 107      -9.038  15.653  26.570  1.00 35.40           O  0.100
HETATM 2153  O   HOH S 108      -2.641  30.065   4.973  1.00 33.76           O  0.097
HETATM 2154  O   HOH S 111       4.648  15.145  36.183  1.00 36.10           O  0.101
HETATM 2155  O   HOH S 113      -0.216  42.943  19.088  1.00 27.36           O  0.088
HETATM 2156  O   HOH S 114       2.662  29.783  28.981  1.00 27.15           O  0.087
HETATM 2157  O   HOH S 116      13.791   8.010  32.993  1.00 30.85           O  0.093
HETATM 2158  O   HOH S 117      20.003  20.003  18.600  1.00 25.89           O  0.085
HETATM 2159  O   HOH S 125      14.242  14.242  37.200  1.00 24.57           O  0.083
HETATM 2160  O   HOH S 131      16.279  17.412  17.410  1.00 22.47           O  0.079
HETATM 2161  O   HOH S 140      -0.869  28.853   3.029  1.00 43.40           O  0.110
HETATM 2162 RE    RE B   3     -15.173  25.407  12.205  0.18 34.74          Re  0.099
HETATM 2163 RE    RE B   4      15.282  21.767  33.664  0.14 38.65          Re  0.104
HETATM 2164 RE    RE B   5       8.279  32.796  16.962  0.31 25.05          Re  0.084
HETATM 2165 RE    RE B   6       0.437  25.207   3.842  0.22 40.93          Re  0.107
HETATM 2166 RE    RE B   7     -10.654  10.654   9.300  0.17 29.19          Re  0.090
HETATM 2167 RE    RE B   8     -16.132  16.183   8.075  0.34 48.63          Re  0.117
HETATM 2168 RE    RE B   9     -11.676   8.162  11.423  0.28 68.83          Re  0.139
HETATM 2169 BR    BR C   4      13.415  13.301  32.482  0.45 24.57          Br  0.083
END
```
