# Supplementary material for: Time-series analysis of rhenium(I) organometallic covalent binding to a model protein for drug development
Source: IUCrJ. 2024 Apr 19;11(Pt 3):359–73. doi: 10.1107/S2052252524002598 (PMC11067751; doi:10.1107/S2052252524002598)
Supplement: Supplementary file 9 [file m-11-00359-sup9.pdf]

IUCrJ

Volume 11 (2024)

Supporting information for article:

Time-series analysis of rhenium(I) organometallic covalent binding to a model protein for drug development

Francois J.F. Jacobs, John R. Helliwell and Alice Brink

With reference to Figure 5-7, our metal containing structure at 38 weeks was evaluated against three HEWL crystals, containing the same Na and Cl ions, in the $P4_32_12$ space group collected at a 100K, namely: 2w1y – red; 5uvj – green; 7ac2 – blue. In the images below, it is clear that insignificant movement is found in the three non-metal containing structures versus what is observed to our metal based protein.

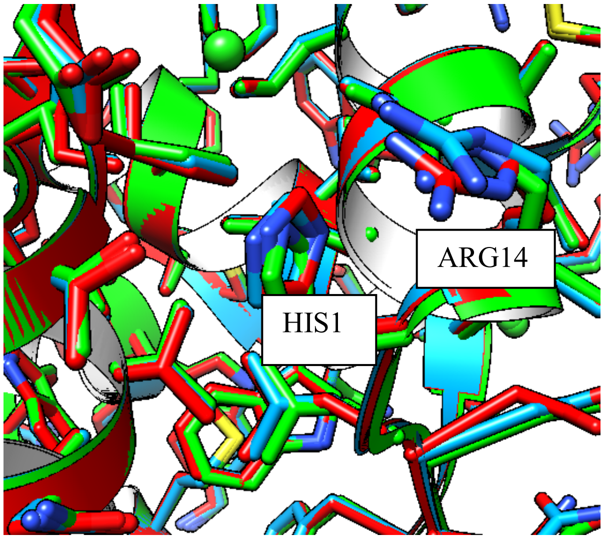

Figure S1a. For the comparison of 2w1y, 5uvj, 7ac2 structures, at the HIS15 site, the ARG14 does not move significantly, in comparison to our Figure 5.

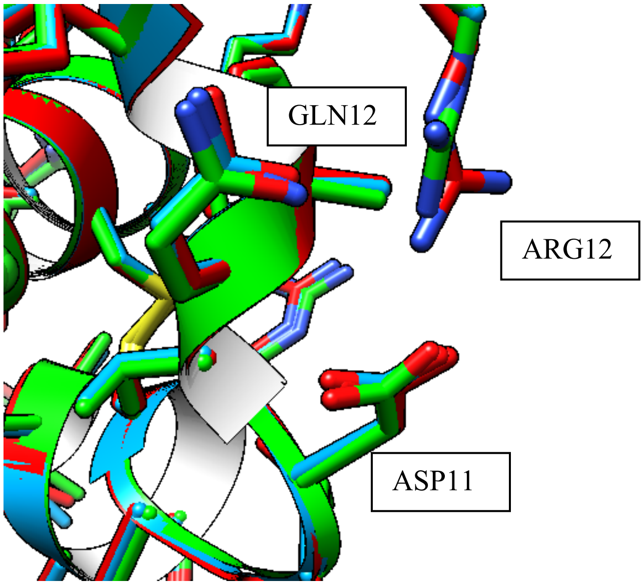

Figure S1b. Similarly for ARG125, GLN 121 and ASP 119, there are minimal changes in the side chain orientations for the 3 proteins containing no metal, versus our Figure 7 with bound metals.
